# Identifying mRNA, MicroRNA and Protein Profiles of Melanoma Exosomes

**DOI:** 10.1371/journal.pone.0046874

**Published:** 2012-10-09

**Authors:** Deyi Xiao, Joanna Ohlendorf, Yinlu Chen, Douglas D. Taylor, Shesh N. Rai, Sabine Waigel, Wolfgang Zacharias, Hongying Hao, Kelly M. McMasters

**Affiliations:** 1 Department of Surgery, University of Louisville School of Medicine, Louisville, Kentucky, United States of America; 2 Microarray facility, University of Louisville School of Medicine, Louisville, Kentucky, United States of America; 3 Department of Obstetrics, Gynecology, and Women’s Health, University of Louisville School of Medicine, Louisville, Kentucky, United States of America; 4 Department of Bioinformatics and Biostatistics, University of Louisville School of Medicine, Louisville, Kentucky, United States of America; 5 Department of Medicine and Department of Pharmacology and Toxicology, James Graham Brown Cancer Center, University of Louisville School of Medicine, Louisville, Kentucky, United States of America; Roswell Park Cancer Institute, United States of America

## Abstract

**Background:**

Exosomes are small membranous vesicles secreted into body fluids by multiple cell types, including tumor cells, and in various disease conditions. Tumor exosomes contain intact and functional mRNAs, small RNAs (including miRNAs), and proteins that can alter the cellular environment to favor tumor growth. Molecular profiling may increase our understanding of the role of exosomes in melanoma progression and may lead to discovery of useful biomarkers.

**Methodology/Principal Findings:**

In the present study, we used mRNA array profiling to identify thousands of exosomal mRNAs associated with melanoma progression and metastasis. Similarly, miRNA array profiling identified specific miRNAs, such as hsa-miR-31, -185, and -34b, involved in melanoma invasion. We also used proteomic analysis and discovered differentially expressed melanoma exosomal proteins, including HAPLN1, GRP78, syntenin-1, annexin A1, and annexin A2. Importantly, normal melanocytes acquired invasion ability through molecules transported in melanoma cell-derived exosomes.

**Conclusions/Significance:**

Our results indicate that melanoma-derived exosomes have unique gene expression signatures, miRNA and proteomics profiles compared to exosomes from normal melanocytes. To the best of our knowledge, this is the first in-depth screening of the whole transcriptome/miRNome/proteome expression in melanoma exosomes. These results provide a starting point for future more in-depth studies of tumor-derived melanoma exosomes, which will aid our understanding of melanoma biogenesis and new drug-targets that may be translated into clinical applications, or as non-invasive biomarkers for melanoma.

## Introduction

Exosomes are small endosome-derived vesicles ranging in size from 40–100 nm in diameter that are actively secreted from cells through exocytosis, a process normally used for receptor discharge and intercellular cross-talk [1]. Many types of cells have the capacity to release exosomes, including retinocytes, dendritic cells, B cells, T cells, mast cells, epithelial cells and tumor cells [2]–[7]. Secreted exosomes have been isolated and characterized *in vitro* from cultured cell lines, as well as *in vivo* in body fluids including blood, urine, saliva, amniotic fluid, and malignant pleural effusions [8]–[13]. Exosome levels in blood and other body fluids increases with advancing stage of cancer. This suggests an important role of tumor exosomes not only in cancer development and progression, but also potentially as biomarkers that can be identified through simple body fluid tests [8]–[12]. Thus, exosome technology may provide a powerful non-invasive and dynamic approach for detecting evolving genetic changes relative to tumor progression.

Exosomes have pleiotropic biological functions, including regulation of immune responses, antigen presentation, intercellular communication, tumor proliferation, and the transfer of RNA and proteins between cells. Tumor exosomes have intact and functional mRNAs, small RNAs, and proteins that can alter the cellular environment to favor tumor growth [14], [15]. Exosome mRNA can also produce protein in the presence of functional protein machinery. MicroRNAs (miRNAs) are short RNAs (21–23 nucleotides) that bind to the 3′ untranslated regions of target genes causing translational repression of the target gene, and stimulating rapid degradation of the target transcript. miRNAs represent a new species of genetic regulator, controlling the levels of potentially large numbers of proteins [16], [17]. The presence of specific oncogenic miRNA influences most fundamental biological processes by ultimately altering the expression levels of proteins either through interference with mRNA translation, or reduction in the stability of mRNA in the cytoplasm. There is increasing evidence that tumor exosome miRNA expression profiles may be indicative of disease risks and burdens. As such, exosome miRNAs are being assessed as possible biomarkers to aid the diagnosis and prediction of different stages of cancer, including melanoma [1], [9], [14], [18].

The network of miRNA-mRNA-protein influences most fundamental biological processes by ultimately altering protein expression level. Exploring the full spectrum of mRNA, miRNA, and protein expression signatures in melanoma cells and exosomes, and comparing these signatures with those of normal melanocytes, will provide the starting point for the generation of an mRNA, miRNA, and protein map of melanoma-derived exosomes that can then be used as useful diagnostic markers. However, few mRNA, miRNA or protein expression profiles have been generated from analysis of melanoma cell-derived versus normal melanocyte-derived exosomes. Similarly, little research has been completed that investigates expression profiles in cells versus those in exosomes. In this study, we profiled mRNA and miRNA expression in melanoma cells and exosomes, and compared this expression with that of normal melanocytes and exosomes. Our results indicate that distinct mRNA and miRNA signatures exist in melanoma exosomes. We also identified differentially-expressed proteins in melanoma versus normal melanocyte exosomes. Importantly, delivery of tumor exosomes to normal melanocytes conferred invasion ability to normal melanocytes. Since the molecular components of exosomes play important roles in intercellular signaling and tumor progression, the profiling of exosomal signatures will not only lead to further molecular mechanistic understanding of melanoma, but will also aid in the discovery of novel melanoma biomarkers.

## Materials and Methods

### Cell Lines and Culture Reagents

Two normal human epidermal melanocytes, HEMa-LP and NHEM-c cells, were purchased from Life Technologies (Carlsbad, CA) and PromoCell (Heidelberg, Germany), respectively. The human malignant melanoma cell lines A375 and SK-MEL-28 were purchased from American Type Culture Collection (Rockville, MD). A375 cells and SK-MEL-28 cells were maintained in Dulbecco’s Modified Eagle Medium (DMEM) and α minimal essential medium (α-MEM), respectively, supplemented with 10% exosome-depleted fetal bovine serum (FBS) and penicillin (100 U/mL)/streptomycin (100 µg/mL). FBS was depleted of exosomes by ultracentrifugation at 100,000×g for 16 h at 4°C. HEMa-LP cells and NHEM-c cells were cultured in Medium 254 supplemented with Human Melanocyte Growth Supplement-2 (HMGS-2) and Melanocyte Growth Medium M2 supplemented with Supplement Mix (PromoCell, Heidelberg, Germany), respectively, in a 5% CO_2_ incubator at 37°C. All other cell culture reagents were obtained from Life Technologies.

### Preparation and Isolation of Exosomes

Exosomes were purified from cell culture supernatants by a combination of ultrafiltration and ultracentrifugation. A three-step approach was used to isolate exosomes from culture media as described previously [19]. Initially, culture medium was collected and centrifuged at 400×g for 10 min to remove whole cells. The supernatant was then centrifuged at 15,000×g for 20 min to remove debris. The resulting cell-free medium was concentrated by ultrafiltration using Amicon stirred cell Model 8200 with a molecular weight cutoff membrane of 500,000 Daltons (Millipore, Billerica, MA). This concentrated material was then ultracentrifuged at 100,000×g for 90 min at 4°C to generate an exosome pellet. The pellet was resuspended and washed twice with PBS. Exosome quantity was determined using Nanodrop ND-1000 spectrophotometer at 420 nm (Thermo Fisher Scientific, Pittsburgh, PA).

Another exosome isolation method was performed using Exoquick-TC precipitation (System Biosciences, Mountain View, CA). Briefly, 10 ml of cell culture supernatant was centrifuged at 3000×g for 30 minutes to remove cells and cell debris. The supernatant was mixed with 2 ml of Exoquick-TC and refrigerated overnight. The Exoquick-TC/Cell supernatant mixture was then centrifuged at 10,000×g for 30 minutes at 4°C. The exosome pellet was washed twice with PBS and resuspended in serum-free media to be used in migration/invasion assay.

### Transmission Electron Microscopy (TEM)

Isolated exosomes were collected, washed in cacodylate buffer, and fixed in 4% glutaraldehyde (Polysciences, Warrington, PA) in cacodylate buffer overnight at 4°C, dehydrated with graded alcohol steps, and flat embedded in LX-112 epoxy resin (Ladd Industries, Burlington, VT). Sections were cut with an ultramicrotome. Mounted sections (70–80 nm) were collected on copper grids, stained with saturated solution of uranyl acetate, and submitted for imaging using a Philips CM12 Transmission Electron Microscope operating at 60 kV [20].

### RNA Isolation and Microarray Analysis

Total RNA from cells and exosomes were isolated using mirVana total RNA isolation kit (Life Technologies) according to the manufacturer’s guidelines. This protocol effectively recovers both mRNA and miRNA. RNA was quantified using Nanodrop ND-1000 (Thermo Fisher Scientific). The integrity of these total RNAs was assessed using Agilent 2100 Bioanalyzer (Agilent, Santa Clara, CA). Total high-quality RNA was converted to cDNA, transcribed and labeled, and then hybridized to human HG-U133 plus 2 arrays (Affymetrix, Santa Clara, CA) then scanned according to the standard protocol recommended by Affymetrix. The miRNA array profiling was performed by using the Affymetrix GeneChip miRNA Array 1.0. Two different RNA preparations from two cell lines and their exosomes were used, except that only one RNA preparation was used for HEMa-LP exosome miRNA array. Due to the limited number of passages (approximately 10), adequate exosomal RNA and proteins from HEMa-LP cells for multiple analyses was not available.

### mRNA and miRNA Expression Validation by Semi-quantitative Reverse Transcription–PCR

Briefly, total RNA (100 ng) from cell lines and exosomes were reverse transcribed with the SuperScript III First-Strand Synthesis System for RT-PCR (Life Technologies) for gene expression validation. mRNA primers were purchased from Life Technologies. For miRNA expression validation, total RNA (10 ng) was converted into cDNA using specific miRNA primers and miRNA reverse transcription kit (Life Technologies) and further amplified according to the manufacturer’s protocol. Quantitative RT-PCR reactions were completed on a 7500 Fast Real Time PCR system (Life Technologies). The relative quantity of the target mRNA or miRNA was normalized to an endogenous gene (GAPDH) or control miRNA and, relative to a calibrator; then fold changes were calculated with the 2^−ΔΔCt^ method. Samples were run in triplicate and at least 3 independent experiments were performed. Data are presented as mean ± SD. A *p* value of <0.05 was considered to be statistically significant.

### miRNA Target Gene Prediction

TargetScanHuman 6.0 (www.targetscan.org) was used for miRNA target gene predictions. Predicted target genes in combination with miRNA and whole-genome microarray data were used to visualize possible biological miRNA/mRNA interactions correlating to melanoma development and/or progression.

### Proteomic Analysis

The concentration of the exosome protein extracts were determined by protein assay and analyzed by 2-D DIGE (two-dimensional difference in gel electrophoresis). Equal amounts of protein extracts (25 µg) from A375 and HEMa-LP exosomes were labeled with Cy5 and Cy3, respectively. The two labeled exosome samples were simultaneous separated on a single 2-D gel, using isoelectric focusing (IEF) in the first dimension and SDS polyacrylamide gel electrophoresis (SDS-PAGE) in the second dimension. The gel was scanned using a Typhoon image scanner. ImageQuant software was used to generate the single and overlay images. Quantitative and comparative analysis of all spots was performed by using DeCyder “in-gel” analysis software to generate protein expression ratios between A375 and HEMa-LP exosomes. Protein spots of interest were picked from the 2-D gel and identified by mass spectrometry. Protein identification was based on peptide fingerprint mass mapping (using MS spectra) and peptide fragmentation mapping (using MS/MS spectra). Combined MS and MS/MS spectra were then submitted for database search using GPS Explore software to identify proteins from primary sequence database.

### Confocal Microscopy

HEMa-LP cells and isolated A375 exosomes were labeled using the green lipophilic fluorescent dye PKH67, and the red lipophilic fluorescent dye PKH26 (Sigma-Alrich, St Louis, MO), respectively, according to the manufacturer’s instructions. Briefly, HEMa-LP cells were trypsinized and resuspended. The quantities of exosomes were determined by optical density at 420 nm. The re-suspended cells and exosomes were incubated with the two different dyes respectively for 5 min at room temperature. The reaction was stopped by addition of 2 ml FBS. After washing with PBS, the green PKH67-labelled HEMa-LP cells were seeded on cover slips in 24-well plates and incubated with the red PKH26-labelled exosomes for 24 h. HEMa-LP cells were then washed with PBS and mounted with Mowiol (Calbiochem, La Jolla, CA). The scans were performed in a sequential mode to avoid channel crosstalk. Pictures were taken on an Olympus Fluoview 500 confocal microscope.

### MTT Assay

MTT (3-[4,5-Dimethylthiazol-2-Yl]-2,5-Diphenyltetrazolium Bromide) assay was conducted to assess cell survival and growth. HEMa-LP and NHEM-c cells were incubated with 20 µl of A375 or SK-MEL-28 melanoma exosomes (OD420 = 0.01), or cycloheximide (0.1 µg/ml) for 5 days of treatment. MTT assay was conducted as described previously [21].

### In vitro Migration/invasion Assay

BD BioCoat Matrigel control chambers and invasion chambers (BD Biosciences) were used according to the manufacturer’s protocol, and as previously described [22], [23]. Briefly, 28,000 HEMa-LP cells or NHEM-c cells were plated in each chamber in a 24-well plate. The next day, isolated A375 exosomes or SK-MEL-28 exosomes were resuspended in serum-free media. 100 ul of exosomes with an OD420 reading of 0.01 were added to each well. Serum-free media was used as a control. After 5 days of incubation, non-invading cells were removed from the upper surface of the membrane. Migrating cells in the control chamber and invading cells in the invasion chamber were fixed and stained with Diff-Quick (Siemens Healthcare Diagnostics, Deerfield, IL) and then counted in each insert. Protein synthesis inhibitor cycloheximide {3-[2-(3,5-dimethyl-2-oxocyclohexyl)-2-hydroxyethyl] glutarimide} was purchased from Sigma Chemical Co. (St. Louis, MO). Cycloheximide was prepared fresh in 0.9% saline, and used at a final concentration of 0.1 µg/ml where indicated. DNase, RNase A, and Protease K were purchased from Sigma Chemical Co. (St. Louis, MO), and used at final concentration of 100 µg/ml. Each invasion assay experiment was performed in triplicate and repeated three times. Results are presented as percent invasion, which was calculated by dividing the number of the cells that invaded through the invasion chamber by the number of the cells that migrated through the control chamber and multiplying by 100.

To check mRNA expression by RT-PCR analysis after invasion assay, HEMa-LP cells were plated in a 6-well plate. The next day, 500 µl of isolated A375 exosomes or SK-MEL-28 exosomes with an OD420 reading of 0.01 were added to each well. 500 µl of serum-free media was used as a control. After 5 days of incubation, culture media was removed. Cellular RNA was isolated as described in mRNA expression validation by semi-quantitative reverse transcription-PCR.

### Statistical Analysis

The mRNA and miRNA array data were analyzed using Partek Genomics Suite v6.5 (Partek Inc., St. Louis, Missouri). A False Discovery Rate (FDR) corrected *p*-value of <0.01 and a fold change of >2 were defined as upregulation, while a FDR corrected *p*-value of <0.01 and a fold change of <−2 were defined as downregulation unless otherwise stated. Ingenuity Pathway Analysis (IPA) software (Ingenuity Systems, Redwood City, CA) was used for gene network and pathway analysis. The statistical score of a pathway is defined as –log (*P* value) from Fisher’s exact test analysis.

For other experiments, data from three independent experiments were analyzed by Student *t*-test and are given as mean ± SD. A *p*-value of <0.05 was considered to be statistically significant.

### Accession Number

The mRNA array and miRNA array data have been deposited in NCBI’s Gene Expression Omnibus (GEO, http://www.ncbi.nlm.nih.gov/geo) and are accessible through GEO series accession number GSE 35389.

## Results

### Identification and Characterization of Exosomes

**Figure 1:**
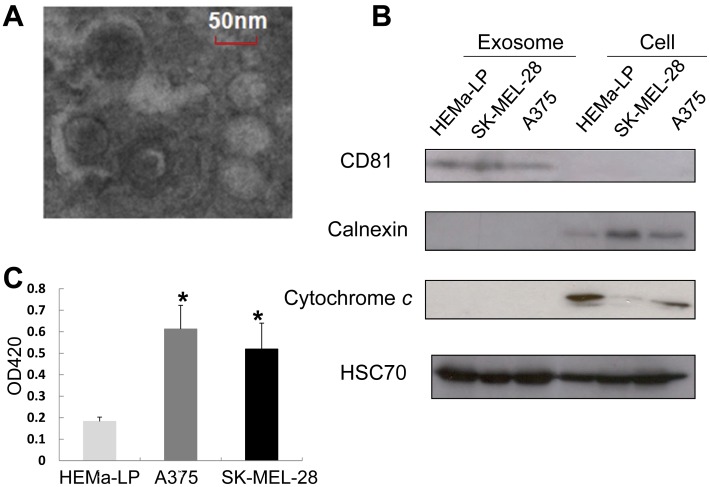
Identification and characterization of exosomes. Exosomes were isolated using a combination of ultrafiltration and ultracentrifugation. (**A**). Morphological characterization of exosomes derived from A375 cells by transmission electron microscopy. The image shows small vesicles ranging in size from 50 nm–100 nm in diameter. The scale bar indicates 50 nm. (**B**). Molecular characterization of exosomes derived from HEMa-LP, SK-MEL-28 and A375 cells by Western blotting. Protein extracts (50 µg) from cells or exosomes were assessed using antibodies against exosomal protein marker (CD81), endoplasmic reticulum marker (Calnexin), and mitochondrial protein marker (cytochrome *c*). HSC70, a protein expressed both in cells and exosomes, was used as a loading control. (**C**) Isolated exosomes from an average of 500 ml supernatant of HEMa-LP cells, SK-MEL-28 cells and A375 cells were resuspended in 100 µl of PBS and their quantities were determined using a Nanodrop ND-1000 spectrophotometer reading at OD420.

Exosomes are released into a variety of body fluids *in vivo,* and into the media of cultured cells *in vitro,* in order to execute important biological functions. Previously, the most common method for isolating exosomes from cultured cell-media was differential centrifugation, which is very time-consuming and labor-intensive. We used a technique combining ultrafiltration and ultracentrifugation, which allows for efficient exosome isolation from cultured media [19]. Purified vesicles from A375 cell culture supernatants were first examined by transmission electron microscopy (TEM), which showed that the isolated membrane-bound round-shaped vesicles ranged in size from about 50–100 nm in diameter (Fig. 1A). This size range is consistent with exosomes. Western blot revealed that the exosome-specific protein, CD81, was enriched in all exosome samples but not in cell lysates–confirming these vesicles as exosomes (Fig. 1B). Calnexin, an endoplasmic reticulum protein, was detectable in whole cell lysates but absent in the exosomes, indicating that the exosome preparations were not contaminated with other vesicles (Fig. 1B). A similar result was obtained for a mitochondrial protein, cytochrome *c* (Fig. 1B), demonstrating that there was no contamination with apoptotic vesicles. HSC70, which has been shown to be present in both cells and exosomes [24] was used as a loading control (Fig. 1B). These results confirmed the identification and characterization of isolated vesicles as exosomes. Exosome yields from A375 and SK-MEL-28 melanoma cells were much greater than those from HEMa-LP normal melanocytes as shown by OD420 values (Fig. 1C), confirming enhanced exosome secretion from tumor cells.

### Differential mRNA Expression Profiles of Exosomes Versus Cell Lines, and A375 Versus HEMa-LP Exosomes

**Figure 2:**
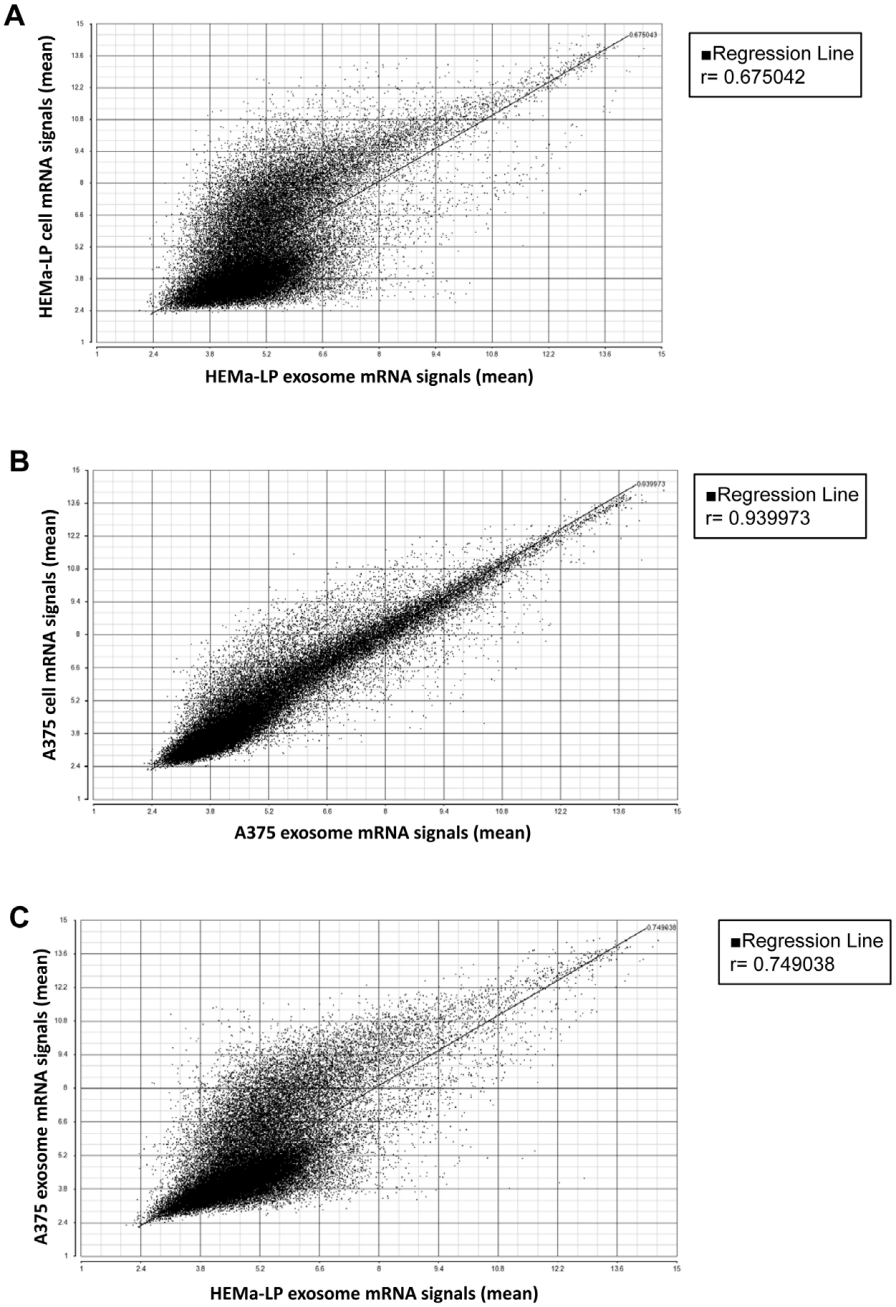
Correlation of mRNA signals between cells and exosomes. Affymetrix HU133 plus 2 arrays were used to analyze mRNA signals in HEMa-LP melanocytes and A375 melanoma cells as well as exosomes from the two cell lines. Two different arrays were performed from two different RNA preparations for each sample. Scatterplots of mRNA signals in HEMa-LP exosomes compared with their originating cells (**A**), A375 exosomes compared with their originating cells (**B**), and A375 exosomes compared with HEMa-LP exosomes (**C**). Regression analysis showed that mRNA signals in cells versus exosomes were correlated. mRNA signals in A375 exosomes were also correlated with those in HEMa-LP exosomes.

Whole-genome mRNA arrays were performed to identify genes differentially expressed in exosomes versus cell lines, and A375 exosomes versus HEMa-LP exosomes. Using Partek Genomics Suite for differential gene expression analysis, we identified 14,784 probe sets that were upregulated, and 13,671 probe sets that were downregulated in normal human melanocyte HEMa-LP exosomes versus HEMa-LP cells. In order to further analyze the genes that were differentially expressed in HEMa-LP exosomes versus HEMa-LP cells, we restricted the criteria of upregulation as a FDR corrected *p*-value of <0.01 and a fold change of >5, and downregulation as a FDR corrected *p*-value of <0.01 and a fold change of <−5. This resulted in 913 probe sets (813 genes) that were upregulated and 4921 probe sets (3642 genes) downregulated in HEMa-LP exosomes versus HEMa-LP cells (Table S1). Some of the genes in the array have multiple probe sets representing various splice forms that may have differential biological function. Ingenuity pathway analysis showed involvement of differentially expressed genes in post-translational modification (506 molecules), cellular growth and proliferation (975 molecules), cell death (778 molecules), gene expression (744 molecules), and cellular development (816 molecules) (Figure S1A). Those differentially expressed genes were involved in the protein ubiquitination (statistical score = 10.359), clathrin-mediated endocytosis signaling (statistical score = 9.472), and integrin signaling pathways (statistical score = 8.758) (Figure S1B). Regression analysis showed that mRNA signals between HEMa-LP cells and exosomes were correlated (r = 0.675) (Fig. 2A).

We also identified 842 probe sets (721genes) upregulated and 3,678 probe sets (2564 genes) downregulated in human melanoma cell line A375 exosomes versus A375 cells. The complete list of upregulated and downregulated genes (and their different splice-forms of probe sets) is given in Table S2. Ingenuity analysis showed involvement of those differentially expressed genes functioning in post-translational modification (344 molecules), cellular movement (402 molecules), molecular transport (396 molecules), cell death (556 molecules) and cellular growth and proliferation (698 molecules) (Figure S1C). Those differentially expressed genes were involved in N-glycan biosynthesis (statistical score = 11.505), sphingolipid metabolism (statistical score = 8.148), and antigen presentation pathways (statistical score = 6.1) (Figure S1D). Among these identified pathways, the N-glycan pathway has been shown to be involved in tyrosinase and melanin synthesis in melanoma cells as well as melanoma cell metastasis [25], [26]. Sphingolipid has been involved in a lipogenic pathway to boost Akt signaling [27]. A strong correlation of mRNA signals between A375 cells and exosomes was found (r = 0.93997) (Fig. 2B). These results show that normal cell-derived exosomes and melanoma cell-derived exosomes contain many mRNAs related to cellular growth and proliferation, cellular movement, and gene expression. Even though some of the mRNAs are differentially expressed between the cells and the cell-derived exosomes, the exosomal mRNAs have a strong correlation with the cellular mRNAs. This correlation was stronger between A375 melanoma cells and exosomes. These data suggest that exosome mRNA signatures may reveal information about gene signatures from within their parent cells.

After we compared the gene signatures between cells and exosomes, we were interested in exploring the difference between mRNA expression profiles of melanoma cell-derived exosomes and normal melanocyte-derived exosomes. For these analyses we once again defined upregulation as a FDR corrected *p*-value of <0.01 and a fold change of >5, and downregulation as a FDR corrected *p*-value of <0.01 and a fold change of <−5. We identified 3553 probe sets (2813 genes) upregulated, and 379 probe sets (333 genes) downregulated in A375 exosomes versus HEMa-LP exosomes (Table S3). Ingenuity analysis showed that 945 differentially expressed genes are associated with cancer, and 364 differentially expressed genes are associated with dermatological diseases and conditions (Figure S1E). Among the upregulated genes were TOP1 (DNA topoisomerase I), which is associated with advanced melanomas and poor prognosis [28]. Among the downregulated genes were TYRP1 (tyrosinase-related protein 1) and ABCB5 (ATP-binding cassette, sub-family B, member 5), both of which are related to melanoma progression and initiation [29]–[31]. Ingenuity analysis showed that those differentially expressed genes function in RNA post-transcriptional modification (198 molecules), cell cycle (481 molecules), gene expression (656 molecules), and cellular growth and proliferation (756 molecules) (Figure S1E). Those differentially expressed genes are involved in protein ubiquitination (statistical score = 17.066), estrogen receptor signaling (statistical score = 11.313), and aminoacyl-tRNA biosynthesis (statistical score = 9.84) pathways, all of which have been shown to be involved in melanoma growth and progression (Fig. S1F). Even though regression analysis showed that mRNA signals in A375 exosomes were somewhat correlated with those in HEMa-LP exosomes (r = 0.749038) (Fig. 2C), these results suggest that melanoma cell-derived exosomes have distinct mRNA profiles that differ from those of normal melanocyte-derived exosomes. Those differentially expressed mRNAs in melanoma exosomes may play important roles in tumor initiation, progression, and metastasis. This also implies that those exosomal mRNAs may serve as biomarkers to differentiate melanoma from normal melanocytes.

### Differential miRNA Expression Profiles of Exosomes Versus Cell Lines and A375 Versus HEMa-LP Exosomes

**Figure 3:**
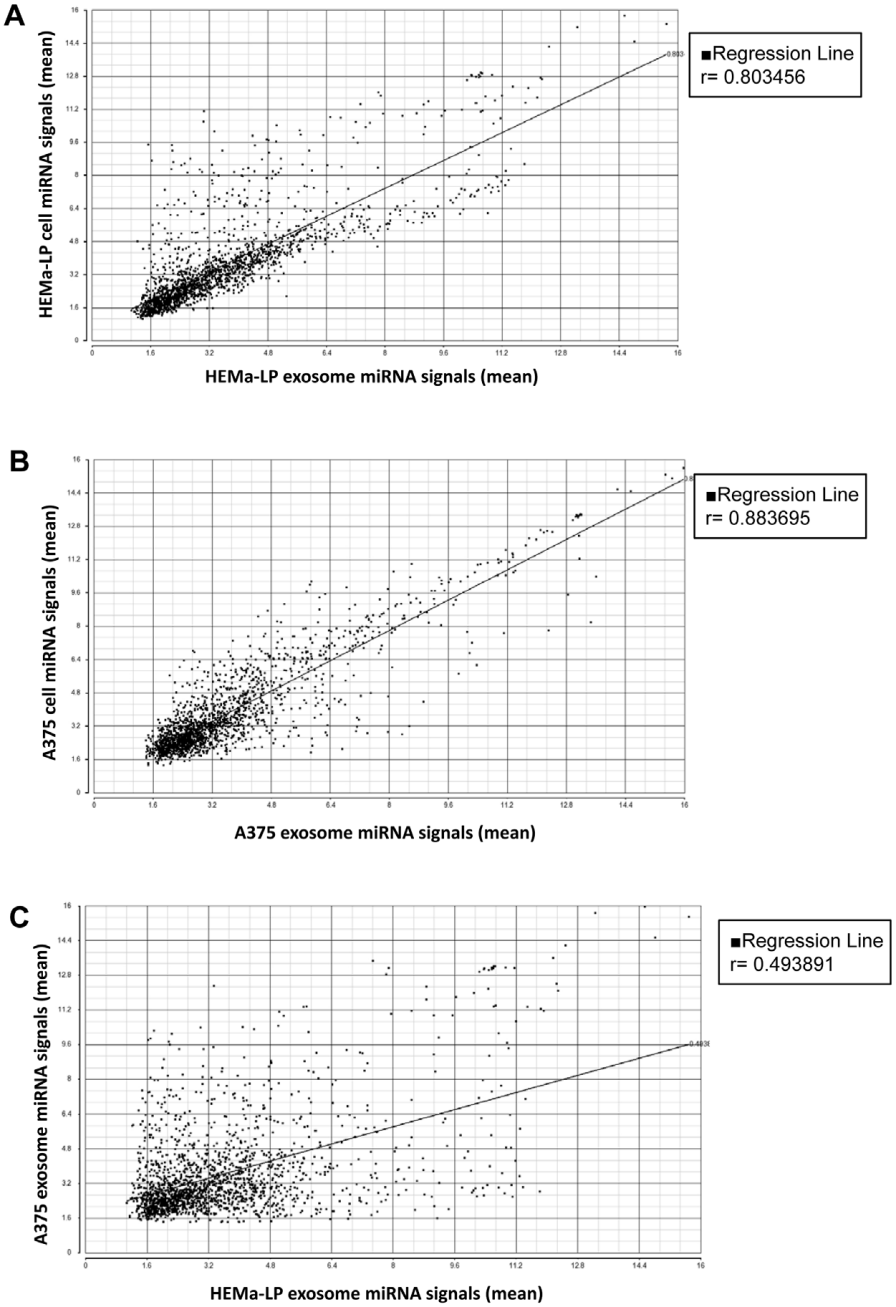
Correlation of miRNA signals between cells and exosomes. Affymetrix miRNA 1.0 arrays were used to analyze miRNA signals in HEMa-LP melanocytes and A375 melanoma cells, as well as exosomes from the two cell lines. Two different arrays were performed from two different RNA preparations for each sample, except for only one RNA preparation for HEMa-LP exosomal miRNA array. Scatterplots of miRNA signals in HEMa-LP exosomes compared with their originating cells (**A**), A375 exosomes compared with their originating cells (**B**), and A375 exosomes compared with HEMa-LP exosomes (**C**). Regression analysis showed that miRNA signals in HEMa-LP cells versus HEMa-LP exosomes, and A375 cells versus A375 exosomes were correlated, whereas miRNA signals in A375 exosomes versus HEMa-LP exosomes were not well correlated.

Emerging evidence shows that exosome miRNA have close relationships with tumorigenesis and metastasis [1], [9], [18]. In order to shed light on exosome miRNA profiles, miRNA arrays were performed to identify differentially expressed miRNAs in exosomes versus cell lines and A375 exosomes versus HEMa-LP exosomes. Using Partek Genomics Suite, we identified 14 miRNAs upregulated and 75 miRNAs downregulated in HEMa-LP exosomes versus HEMa-LP cells (Table S4). Ingenuity analysis showed the involvement of those differentially expressed miRNAs functioning in cell cycle (9 miRNAs), cellular development (12 miRNAs), cellular growth and proliferation (16 miRNAs), and cellular movement (11 miRNAs) (Figure S2A). A strong correlation of miRNA signals between HEMa-LP cells and exosomes was found (r = 0.803456) (Fig. 3A).

We also identified 28 miRNAs upregulated and 5 miRNAs downregulated in A375 exosomes versus A375 cells (Table S5). Ingenuity analysis showed many of these differentially expressed miRNA are associated with cancer (hsa-miR-1228, -125b-5p/−125a-5p/−125b, -195/−16-2, -339-5p/−3586-5p, -346, -494, -638). Other differentially expressed miRNAs also function in cellular growth and proliferation (hsa-miR-125 and hsa-miR-346), cellular development (hsa-miR-346), cellular movement (hsa-miR-125), and cell death (has-miR-193). A strong correlation of miRNA signals between A375 cells and exosomes was found (r = 0.883695) (Fig. 3B). The miRNA signatures of HEMa-LP exosomes versus HEMa-LP cells, and A375 exosomes versus A375 cells correlate well with those of their respective mRNA profiles. These results suggested that strong correlations of miRNA profiles exist between cells and cell-derived exosomes, suggesting that the exosomal miRNome largely represents miRNA signatures within their originating cells. Exosomes also contain many miRNAs that are linked with cellular growth and proliferation, cellular development and cellular movement.

To distinguish miRNA signatures between melanoma cell-derived exosomes and normal melanocyte-derived exosomes, we compared the miRNome in A375 and HEMa-LP exosomes. We identified 130 miRNAs upregulated and 98 miRNAs downregulated in A375 versus HEMa-LP exosomes (Table S6). Ingenuity analysis showed that many differentially expressed miRNAs were associated with cancer (70 miRNAs) (Figure S2B). These differentially expressed miRNAs also function in cellular growth and proliferation (22 miRNAs), cellular development (15 miRNAs), cellular movement (13 miRNAs), and cell cycle (9 miRNAs) (Figure S2B). Among the dysregulated miRNAs were hsa-miR-31 and -185, which are related to regulation of aggressive features of melanoma [32], and hsa-miR-34b, which has been shown to be involved in melanoma invasiveness [33]. We listed 15 dysregulated miRNAs that are known to be associated with melanoma metastasis after ingenuity analysis (Table 1). Regression analysis showed that miRNA signals were less correlated between A375 and HEMa-LP exosomes (r = 0.493891) (Fig. 3C). These results suggest that a substantial difference in miRNA expression profile exists between normal melanocyte-derived exosomes and melanoma cell-derived exosomes. Melanoma exosomes express a group of miRNAs that may play important roles in melanoma progression and metastasis.

**Table 1:** Differentially expressed miRNAs related to melanoma metastasis between A375 exosomes and HEMa-LP exosomes.

Probe ID	Genes in dataset	Fold Change
**hsa-let-7c_st**	let-7a/let-7f/let-7c (includes others)	61.026
**hsa-miR-138_st**	miR-138	51.192
**hsa-miR-125b_st**	miR-125b-5p/miR-125a-5p/miR-125b (includes others)	43.944
**hsa-miR-130a_st**	miR-130a/miR-130b/miR-301a (includes others)	39.317
**hsa-miR-34a_st**	miR-449a/miR-34a/miR-34c (includes others)	34.025
**hsa-miR-196a_st**	miR-196a/miR-196b	18.635
**hsa-miR-199b-3p_st**	miR-199a-3p	8.677
**hsa-miR-25_st**	miR-92a/miR-92b/miR-32 (includes others)	6.089
**hsa-miR-27a_st**	miR-27b/miR-27a	5.906
**hsa-miR-200b_st**	miR-429/miR-200b/miR-200c	**−**2.356
**hsa-miR-23b_st**	miR-23b/miR-23a/miR-23c (includes others)	**−**2.465
**hsa-miR-146a_st**	miR-146a/miR-146b/miR-146b-5p	**−**2.694
**hsa-miR-613_st**	miR-1/miR-206/miR-1a	**−**18.102
**hsa-miR-205_st**	miR-205	**−**18.922
**hsa-miR-149_st**	miR-149	**−**67.514

### Differential Protein Expression Signatures of A375 and HEMa-LP Exosomes

**Figure 4:**
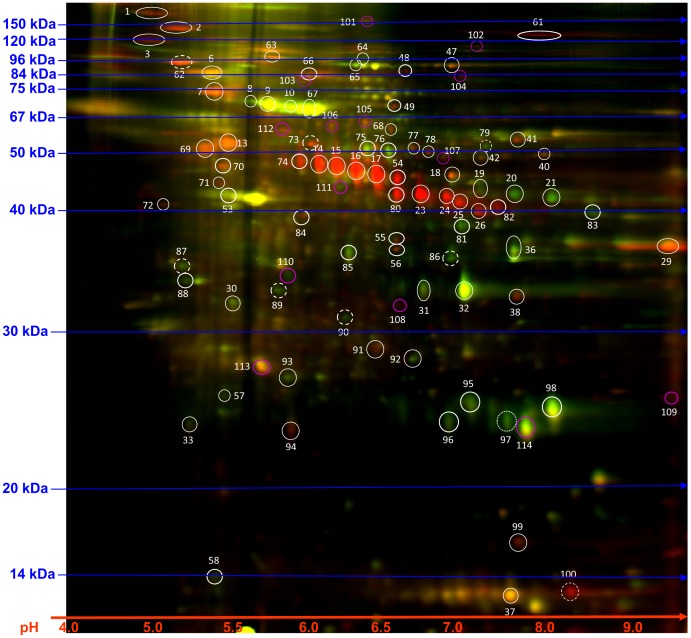
2-D DIGE analysis of A375 exosomes versus HEMa-LP exosomes. 25 µg of proteins from A375 exosomes (labeled with Cy5, red) and HEMa-LP exosomes (labeled with Cy3, green) were separated by isoelectric focusing (IEF) in the first dimension and SDS polyacrylamide gel electrophoresis (SDS-PAGE) in the second dimension. Overlay image was generated by ImageQuant software. Differentially expressed protein spots were circled and stored for further protein identification.

Functional mRNAs in exosomes can be translated and post-transcriptionally modified into protein to exert their function. miRNAs are upstream regulators that can simultaneously target large numbers of protein-coding genes and multiple cancer pathways. On the other hand, miRNAs are the direct functional product of the corresponding gene. Exosomal mRNAs, miRNAs, and proteins are woven together to form a large network of messengers and mediators for melanoma progression. Unveiling the protein profile in exosomes is the last necessary step toward the understanding of melanoma exosomes. To this end, we analyzed the protein profiles between the A375 and HEMa-LP exosomes. Figure 4 shows the 2-D overlapping image of A375 and HEMa-LP exosome protein expression. Selected proteins have been identified and are listed in Table 2. Among the identified proteins were annexin A1, annexin A2, syntenin-1, and hyaluronan and proteoglycan link protein 1 (HAPLN1), which all have functions related to angiogenesis, melanoma cell invasion, migration, and metastasis [23], [31], [34], [35]. Interestingly, annexin A1 was upregulated while annexin A2 was downregulated in A375 exosomes. These results show that tumor exosomes have some distinctive proteins that may have significant and specific activities during melanoma progression and metastasis.

**Table 2:** Identities of differentially expressed proteins in A375 exosomes versus HEMa-LP exosomes.

Assigned spot #	Ratio of A375 exosome/HEMa-LP exosome	Proteins identified	Protein accession number
**7**	6.75	78 KDa glucose-regulated protein precursor (GRP78)	gi|16507237
**13**	2.79	Tublin, alpha 1A (TUBA1B)	gi|18204869
**17 & 24**	36.13 & 15.37	Hyaluronan and proteoglycan link protein 1 (HAPLN1)	gi|4503053
**21**	−4.39	milk fat globule-EGF factor 8 protein (MFGE8)	gi|119622432
**32**	−2.82	Syntenin-1 isoform 1 (SDCBP, MDA-9)	gi|55749490
**36**	−2.5	Annexin A2 (ANXA2)	gi|50845388
**48**	5.93	brain glycogen phosphorylase(PYGB)	gi|62087740
**55& 56**	1.39 & 2.84	Annexin A1 (ANXA1)	gi|119582950, gi|4502101
**62**	2.78	Endoplasmin precursor (gp 96)	gi|4507677
**79**	−2.08	3-oxoacid CoA transferase (OXCT)	gi|48146215
**94**	2.96	ferritin, heavy polypeptide 1, isoform CRA_e	gi|119594401

### Correlation of Expression Levels of Selected miRNAs with their Predicted Targeted Genes

**Figure 5:**
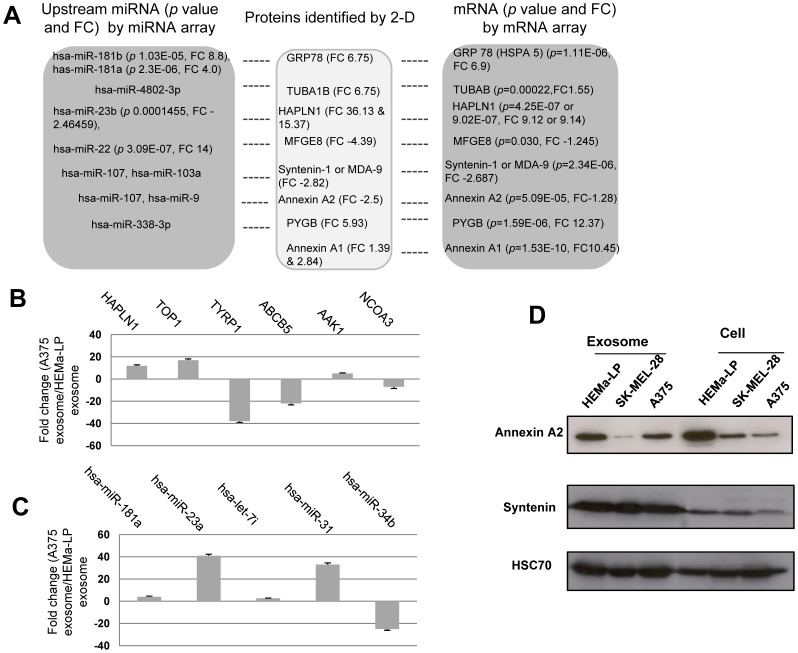
Interaction and validation of differentially expressed mRNAs, miRNAs, and proteins in A375 versus HEMa-LP exosomes. (**A**) Correlation of differentially expressed mRNAs, miRNAs, and proteins in A375 exosomes versus HEMa-LP exosomes (FC = fold change). Total exosomal RNA was reverse transcribed to cDNA for mRNA and miRNA validation. Expression levels of mRNA (**B**) or miRNA (**C**) were analyzed by RT-PCR. Samples were run in triplicate with at least 3 independent experiments. The bars represent normalized percentage (%) of fold change values with mean ± SD between A375 and HEMa-LP exosomes. (**D**) Total exosomal protein was extracted for Western blotting analysis using antibodies as designated. HSC70 was used as a loading control.

Having identified exosomal mRNA, miRNA, and protein signatures, the interactions among these differentially expressed mRNAs, miRNAs, and proteins were investigated. We focused on expression signatures between A375 exosomes and HEMa-LP exosomes. Figure 5A lists some of these mass spectrometry-identified proteins and correlates them with their differentially expressed genes in mRNA array, and their upstream miRNAs. Except for ferritin (heavy polypeptide 1), the alterations of the mRNA expression levels parallel their protein expression levels. We found no correlating mRNA probes for ferritin (heavy polypeptide 1) in mRNA array. However, another mRNA isoform of ferritin (light polypeptide) was found downregulated by 1.56 fold with a *p* value of 1.27E−06.

We also searched for the upstream miRNAs that may target identified differentially expressed proteins, using TargetScanHuman 6.0. Some of the upstream miRNAs were inversely expressed with their targeted proteins. These results implied that exosomal mRNAs, miRNAs, and proteins form an intricate network to execute signal transduction and melanoma progression.

We have confirmed some of the differentially expressed mRNA (Fig. 5B) and miRNA (Fig. 5C) by semi-quantitative real time RT-PCR. We also confirmed that 5 of the randomly-chosen non-differentially expressed mRNAs, or miRNAs, were not significantly expressed in this real time RT-PCR experiment (data not shown). Some of the differentially expressed protein levels were confirmed by Western blotting (Fig. 5D). These results validate our mRNA, miRNA array and 2-D proteomic results.

### Normal Melanocytes Acquire Invasiveness through Uptaking of Melanoma Cell-derived Exosomes

**Figure 6:**
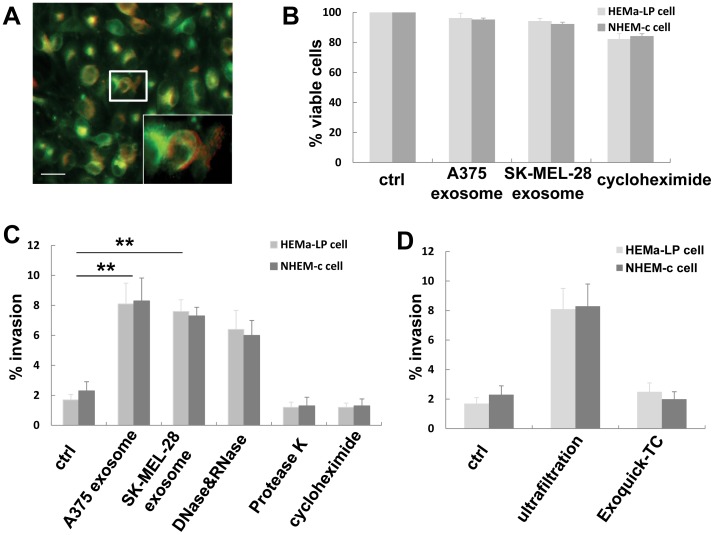
Melanoma exosomes are taken up by normal melanocytes and conferred invasion ability on normal melanocytes. (**A**) HEMa-LP cells and A375 exosomes were labeled using the green fluorescent dye PKH67 and the red fluorescent dye PKH26 respectively. After incubating the labeled A375 exosomes with HEMa-LP cells for 24 h, confocal microscopy images were taken (magnification, x600). The overlay image shows A375 exosomes (red) were internalized by HEMA-LP cells (green). The insert represents higher magnification of the boxed area (scale bar = 20 µm). (**B**) MTT assay of HEMa-LP and NHEM-c cells incubating with media only (control), A375 exosomes, SK-MEL-28 exosomes, and cycloheximide. (**C**) Percent invasion of HEMa-LP and NHEM-c cells after incubation of 5 days with A375 exosomes, SK-MEL-28 exosomes, pretreatment of DNase and RNase A with A375 exosomes, pretreatment of Protease K with A375 exosomes, and treatment of A375 exosomes with cycloheximide. (**D**) Comparison of percent invasion in HEMa-LP cells and NHEM-c cells using exosomes isolated by a combination of ultrafiltration and ultracentrifugation and by Exoquick-TC precipitation. Each invasion assay experiment was performed in triplicate, and data from three independent experiments are presented. (**p<0.01, significant in comparison with control).

In order to clarify how tumor-derived exosomes transport their active molecules into the cells and subsequently affect the function of the cells, we incubated A375 or SK-MEL-28 melanoma exosomes with normal melanocytes HEMa-LP and NHEM-c. We first checked whether cells can internalize exosomes *in vitro*. After incubation for 24 h, confocal microscopy demonstrated that red-fluorescent vesicles were internalized in green fluorescent-labeled cells, suggesting uptake of A375 exosomes by HEMa-LP cells (Fig. 6A).

We then examined whether melanoma exosomes affect the growth and proliferation of normal melanocytes. MTT analysis showed that both A375 and SK-MEL-28 exosomes have no obvious effects on HEMa-LP and NHEM-c cell growth and proliferation (Fig. 6B). Because the migration/invasion assay also controls for proliferation, we used this method to further support our MTT results, and to evaluate the invasion ability of normal melanocytes after uptake of melanoma exosomes. After incubation of A375 or SK-MEL-28 exosomes with HEMa-LP cells or NHEM-c cells for 5 days, results from the migration/invasion assay showed that the percent invasion of HEMa-LP cells or NHEM-c cells was significantly greater than those of the control cells (Fig. 6C). To ensure the exosome transfer mediates this invasion ability of normal melanocytes, we pretreated melanoma exosomes with DNase and RNase A or protease K, and then incubated with normal melanocytes. The results showed that pretreatment of melanoma exosomes with DNase and RNase A have no significant effects on the increased invasion ability of normal melanocytes. But protease K pretreatment did decrease the invasion ability of normal melanocytes (Fig. 6C). Interestingly, the addition of cycloheximide (at 0.1 µg/ml), a protein synthesis inhibitor, to HEMa-LP cell or NHEM-c cell media inhibited the invasion ability of HEMa-LP cells or NHEM-c cells promoted by A375 or SK-MEL-28 exosomes (Fig. 6C) without significantly inhibiting HEMa-LP or NHEM-c cell growth (more than 80% viable cells; Fig. 6B). These data suggest that melanoma exosomes can transport functionally active mRNA, miRNA and proteins into normal melanocytes and so provide normal cells with invasion ability. This process is dependent on new protein synthesis.

We compared the migration/invasion inducing capacity of exosomes isolated by another method, exosome precipitation by Exoquick-TC, with our method of combination of ultrafiltration and ultracentrifugation. The results showed that A375 or SK-MEL-28 exosomes precipitated from 10 ml of cell culture supernatant by Exoquick-TC have barely any effects on the invasion abilities of HEMa-LP or NHEM-c cells (Fig. 6D). This suggested that exosomes precipitated from 10 ml cell culture media may not be enough to induce the invasion ability of normal melanocytes. Sufficient melanoma exosomes might be needed to confer this normal cell invasion ability.

### Gene Expression Changes in Normal Melanocytes after Uptake of Melanoma Cell-derived Exosomes

**Figure 7:**
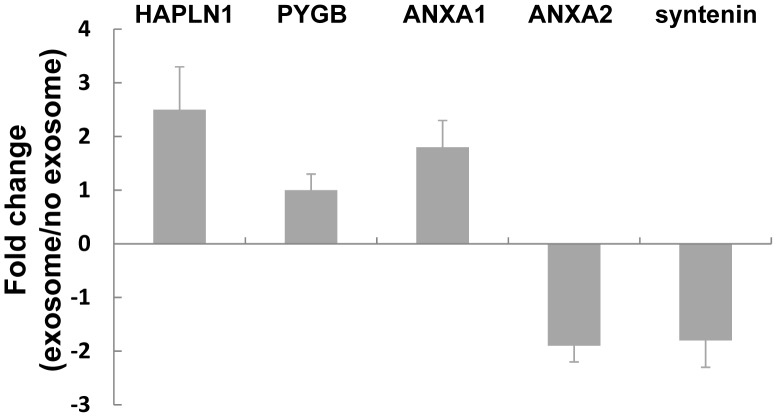
Gene expression changes of normal melanocyte after taking-up melanoma cell-derived exosomes. HEMa-LP cells were seeded in a 6-well plate. The next day, A375 exosomes were added into the plate. No exosome media was used as a control. After 5 days, total HEMa-LP cell RNA was isolated by mirVana kit and reverse transcribed to cDNA. Expression levels of mRNA were analyzed by RT-PCR. Samples were run in triplicate with at least 3 independent experiments. The bars represent normalized percentage (%) of fold change values with mean ± SD between HEMa-LP cells with exosomes and with no exosomes.

We further investigated if there were any gene expression changes in HEMa-LP cells after HEMa-LP cells acquired invasion ability through co-incubation with A375 melanoma exosomes. We focused on those genes that have distinguished differential expression in A375 exosomes: HAPLN1, PYGB, syntenin (MDA-9), ANXA1, and ANXA2. We expected that those highly expressed proteins in A375 melanoma exosomes might be detected in HEMa-LP cells after co-incubation. RT-PCR results showed that after incubating with A375 exosomes, ANXA2 and syntenin in HEMa-LP cells were modestly downregulated by 1.9 fold and 1.5 fold, respectively. HAPLN1, PYGB, and ANXA1 in HEMa-LP cells were upregulated by 2.5, 1, and 1.8 fold, respectively (Fig. 7). These changes suggest that after exosomes transfer their molecules into the cells, cell function is fine-tuned.

## Discussion

In the present study, we first investigated the mRNA signatures of melanoma cells and their exosomes and compared these signatures with those of normal melanocytes and their exosomes. We found large numbers of differentially expressed mRNAs in melanocytes compared with melanocyte-derived exosomes, and in melanoma cells compared with melanoma-derived exosomes. This is consistent with previous findings in glioblastoma microvesicles and their donor cells [14]. Exosomal mRNAs may transfer or shuttle signals between cells, and may contribute to important biological functions in normal cells, as well as malignant transformation in tumor cells [14], [15].

One interesting aspect of our findings was that when we examined the correlation of mRNA expression in cells compared with their exosomes, we noticed that melanoma cell-derived exosomes have a closer relationship with their originating melanoma cells than normal melanocyte-derived exosomes did with their originating non-cancer cells. This suggests that cancer-derived exosome mRNA profiles may more closely reflect mRNA profiles in cancer cells themselves, which would imply the potential of using exosomes as a biomarker for melanoma. By comparing mRNA profiles in melanoma exosomes with normal melanocyte-derived exosomes, we found that even though there are various biological processes and ontologies of those differentially expressed exosome mRNAs, many are linked to the advancement of melanoma. Indeed, several studies have shown that tumor exosomes have the ability to transport RNAs to promote tumor growth [14]. This finding also suggests the potential of using exosome profiles as biomarkers not only of the presence of disease, but also disease progression and response to therapy.

We then investigated miRNA signatures in melanoma cells and their exosomes and compared these signatures with those from normal melanocytes and their exosomes using miRNA arrays. We discovered that there are some differentially expressed miRNAs in melanocytes compared with melanocyte-derived exosomes, and also between melanoma cells compared with melanoma-derived exosomes. We also determined that many of these miRNA have important functions in cellular growth and proliferation, cellular development, cellular movement, and cell death. Our findings confirm earlier studies showing that miRNAs in exosomes have important biological functions [14]–[17]. An important finding from our study came from looking at the correlation of miRNA expression in cells compared with exosomes. Both melanoma cell-derived and normal melanocyte-derived exosomes miRNA profiles were strongly correlated with their originating cells. However, there was much weaker correlation between miRNA expression in melanoma exosomes compared with miRNA expression in normal melanocyte-derived exosomes. This strongly suggests that there are distinctive miRNA profiles between melanoma exosomes and normal melanocyte exosomes, which confirms the rationale behind many of the current studies investigating the usefulness of exosomal miRNA as tumor biomarkers in diseases, such as lung cancer and ovarian cancer [9], [18]. Our data show that differential expression of exosomal miRNA is more focused than mRNA expression. Furthermore, when we looked at the regression analysis of mRNA signals and miRNA signals between A375 and HEMa-LP exosomes, we showed that the difference of miRNA signals between A375 and HEMa-LP exosomes is much larger than that of mRNA signals. It is likely that this would be another advantage of using exosomal miRNA signatures in biomarker studies, instead of sophisticated and unbalanced mRNA data.

Through our proteomic approach, we identified exosomal proteins that are known to be associated with cell adhesion, migration, and invasion in melanoma. Some of these proteins have been identified by other researchers in similar studies. For example, Mears *et al.* compared the protein profiles of two melanoma cell lines, MeWo and SK-MEL-28 cells, with their exosomes [36]. They discovered several novel melanoma exosomal proteins, such as p120 catenin, radixin, and immunoglobulin superfamily member 8 (PGRL). Among the list of exosomal proteins they identified were syntenin 1 and annexin A2, which are also on our list of identified differentially expressed exosomal protein [36]. Although most reports have determined that syntenin-1 enhances melanoma cell migration, invasion and metastasis [37], [38], several discrepant findings have been observed about the role of syntenin-1 depending on the specific cellular environment investigated [39]. In our study, we observed that syntenin-1 protein expression was reduced in melanoma exosomes compared to normal melanocyte-derived exosomes. This is in line with findings in a B16 mouse melanoma model that show syntenin-1 has lower expression levels in melanoma secrotomes, but cells exhibit a greater capacity for cell invasion [23].

Annexin A1 has also been shown to amplify the ability of cells to become invasive and to enhance melanoma dissemination [40]. It is a key regulator of pathological angiogenesis and physiological angiogenic balance [34]. Similarly, annexin A2 is upregulated in various tumors and has been shown to play multiple roles in regulating cellular function, including angiogenesis, proliferation, apoptosis, cell migration, invasion and adhesion [31]. In our study, we found that protein expression levels of annexin A1 were upregulated, whereas annexin A2 levels were downregulated in A375 melanoma exosomes. Grewal and Enrich have summarized the differences in various isoforms of annexin protein expression patterns, subcellular localization and mode of action. They suggest that annexins are likely to differentially contribute and cooperate in fine-tuning of the activity of epidermal growth factor receptor (EGFR), thus regulate the growth of a variety of tumor cells [41].

Our study also revealed several novel proteins differentially expressed in exosomes that have not previously been identified in this context. The protein with the greatest differential expression in melanoma exosomes is hyaluronan and proteoglycan link protein 1 (HAPLN1). HAPLN1 is an extracellular matrix mucopolysaccharide that has been previously shown to promote metastasis in cancer cells, including B16F10 melanoma [42], [43]. HAPLN1 is also involved in melanoma development and extracellular matrix remodeling during the process of melanoma cell migration and melanoma progression [35], [44]. Further research investigating the role of HAPLN1 in exosomes may uncover novel mechanisms to explain potential roles for exosomes in melanoma progression. Additionally, HAPLN1 may also prove to be important clinically, as it is a specific exosomal protein that could be the focus of future melanoma biomarker studies.

Some differentially expressed exosomal proteins have already been explored as potential biomarkers in melanoma patients [1]. For example, Logozzi *et al.* designed an in-house sandwich ELISA (Exotest) and found that plasma exosomes expressing CD63 or caveolin-1 were significantly increased in melanoma patients compared to healthy donors. They further determined that the number of caveolin-1 positive plasma exosomes was significantly greater than the number of CD63 positive exosomes in melanoma patients [1]. Since then, CD63 has become commonly accepted as an exosome marker. Obviously, combining our findings with those of others, together with additional clinical validations will be the best way to provide us with a panel of proteins that will enable us to make more precise predictions of prognosis for melanoma patients. Similar exosomal proteomics techniques have been applied by many researchers to diseases other than those involving tumors [45], such as neurodegenerative disorders [46] and kidney disease [20], which further suggests that exosomal proteomics might provide a powerful diagnostic tool for many diseases.

We are aware that studies such as ours that examine exosomal mRNA, miRNA, and protein profiles produce large amounts of data. Indeed, our mRNA expression profile resulted in identification of thousands of disparate differentially expressed genes. However, we were able to focus this information better when combining miRNA and protein profiles. Proteins are the end-point molecules that execute biological functions after undergoing several sophisticated genetic processes, including transcription, translation and post-translational/post-transcriptional modifications. Our findings lead us to suggest that combining miRNA and protein profiles is a superior approach to identify future exosomal biomarker of disease. One example of how our data can be combined to provide potential new avenues of mechanistic melanoma research and biomarker studies is to look at interactions of highly differentially expressed proteins and miRNAs. In our study, HAPLN1, hsa-miR-23, and hsa-miR-21 were the three molecules at the top of our differentially expressed lists. TargetScan identified that HAPLN1 is targeted by hsa-miR-23, but HAPLN1 can also trigger upregulation of miR-21, which was previously shown to serve an essential role in the malignant progression of human gliomas [47]. Identifying how these three molecules interact in melanoma to contribute to metastasis and disease progression could potentially reveal new avenues of targeted therapy or biomarkers useful in diagnosis and prognosis.

Another novel finding of our study is that normal melanocytes can acquire invasiveness through the internalization of melanoma exosomes. Our data suggest that mRNA and miRNA within melanoma exosomes may be actively transported into normal melanocytes and induce normal melanocyte invasion ability. Pre-treatment of melanoma exosomes with DNase and RNase A didn’t affect the invasion ability of normal melanocytes rendered by melanoma exosome transfer. This excludes the possibility that DNA or RNA molecules in the cell supernatant might mediate this induced normal melanocyte invasion ability. It is exosome transportation that confers normal melanocytes’ invasion ability. Pre-treatment of Protease K abolished the normal melanocytes’ invasion ability rendered by the melanoma exosomes. The reason might be that Protease K disrupt the exosome membrane structure and affect the exosome transmission. A protein synthesis inhibitor was also able to inhibit normal melanocytes’ invasion ability acquired through uptake of melanoma exosomes. This further suggests that the whole process of exosome uptake, molecule transmission, and promotion of invasion requires new protein synthesis.

Although it seems unlikely that circulating exosomes impart a malignant phenotype to normal melanocytes *in vivo*, it demonstrates the principle that potent intercellular signaling via melanoma exosomes may alter disease progression and metastatic potential. Our findings are in accordance with results from other researchers showing that exosomes can transport RNA and proteins to other cells in order to promote tumor growth [14]. Exosomes released from melanoma cells can also prepare sentinel lymph nodes for tumor metastasis [48]. We attempted to use cytochalasin D to inhibit exosome uptake by HEMa-LP cells to further study the underlying mechanisms; however, cytochalasin D is toxic to the HEMa-LP cells (data not shown). This prevented us from obtaining useful data. Detailed mechanistic studies are needed to clarify how the uptake of exosomes contributes to melanoma progression.

We also compared our method of combination of ultrafiltration and ultracentrifugation with another exosome isolation method, Exoquick-TC precipitation, to assess whether there were differences in the ability of the exosomes to induce migration/invasion. Exosomes isolated by Exoquick-TC precipitation were not able to affect the normal melanocyte invasion ability. The reason for a discrepancy between the effects of exosomes isolated by different methods might be that Exoquick-TC precipitation couldn’t enrich enough exosomes. Indeed, we observed that sufficient exosomes are essential for the effective enhancement of normal melanocyte invasion ability (unpublished data).

We expected that after exosomes are taken up by normal melanocytes, those highly expressed genes in melanoma exosomes might also then be highly expressed in normal melanocytes. However, when assessing the gene expression changes of normal melanocyte after the uptake of melanoma cell-derived exosomes, we found minimal differential expression of those genes that were highly expressed in melanoma exosomes. We have considered two possible explanations for this finding. One is that, even though the invasive melanocytes significantly increased compared with control melanocytes, the portion of invasive melanocytes were a small part of the total melanocytes (about 10%, Fig. 6C). The other reason is that after exosomes transfer into melanocytes, there were multiple cellular processes and signal pathways that would need to act cooperatively in order to alter cellular gene expression to reflect the original highly expressed genes in melanoma exosomes. The best approach to identify the differentially expressed genes in normal melanocytes after the upake of exosomes would be through microarray screening. Microarray screening may also help to clarify the mechanisms of increased invasion ability of normal melanocyte after taking-up melanoma exosomes. This is one of our future research interests.

Since this work was based on two melanoma cell lines compared with two normal melanocyte cell lines, we understand that our differentially expressed mRNAs, miRNAs, and protein profiles cannot be universally applied to other melanoma cell lines. However, these data provide us with initial candidate molecules that may be important in melanoma tumorigenesis and tumor progression. Further exploration into the role of these molecules contained in melanoma exosomes is likely to unveil detailed molecular mechanisms of melanoma progression. Similarly, it is likely that samples from melanoma patients will also provide expression profiles that have some variation with cell lines. However, further investigation of exosomal profiles using clinical samples may lead to clinical translation using these profiles as disease biomarkers, or even through identification of new therapeutic targets.

In conclusion, to the best of our knowledge, this is the first comprehensive attempt to reveal the whole mRNA, miRNA and proteome of melanoma exosomes compared with normal melanocyte exosomes. Our study provides a starting point for future more in-depth explorations of tumor-derived exosomes. This exosome research will aid in understanding the molecular biology of melanoma, and may also define targets that can be translated into clinical applications as non-invasive biomarkers or as therapeutic targets for melanoma patients.

## Supporting Information

Figure S1 **Differentially expressed mRNAs in exosomes versus cell lines, and A375 versus HEMa-LP exosomes by Ingenuity Analysis.** Biological functions (A) and pathway analysis (B) of differentially expressed mRNAs in HEMa-LP exosomes versus HEMa-LP cells. Biological functions (C) and pathway analysis (D) of differentially expressed mRNAs in A375 exosomes versus A375 cells. Biological functions (E) and pathway analysis (F) of differentially expressed mRNAs in A375 exosomes versus HEMa-LP exosomes.
(PPT)

Figure S2 **Differentially expressed miRNAs in exosomes versus cell lines, and A375 versus HEMa-LP exosomes by Ingenuity Analysis.** Biological functions of differentially expressed miRNAs in HEMa-LP exosomes versus HEMa-LP cells (A) and in A375 exosomes versus HEMa-LP exosomes (B).
(PPT)

Table S1 **Differentially expressed mRNA probe sets in HEMa-LP exosomes versus HEMa-LP cells (FDR corrected** ***p*** **<0.01 and FC >5 or FC <−5).**
(DOCX)

Table S2 **Differentially expressed mRNA probe sets in A375 exosomes versus A375 cells (FDR corrected** ***p*** **<0.01 and FC >2 or FC <−2).**
(DOCX)

Table S3 **Differentially expressed mRNA probe sets in A375 exosomes versus HEMa-LP exosomes (FDR corrected p<0.01 and FC >5 or FC <−5).**
(DOCX)

Table S4 **Differentially expressed miRNAs in HEMa-LP exosomes versus HEMa-LP cells.**
(DOC)

Table S5 **Differentially expressed miRNAs in A375 exosomes versus A375 cells.**
(DOC)

Table S6 **Differentially expressed miRNAs in A375 exosomes versus HEMa-LP exosomes.**
(DOC)
