# Supplementary material for: Identifying mRNA, MicroRNA and Protein Profiles of Melanoma Exosomes
Source: PLoS One. 2012 Oct 9;7(10):e46874. doi: 10.1371/journal.pone.0046874 (PMC3467276; doi:10.1371/journal.pone.0046874)
Supplement: Figure S1 — Differentially expressed mRNAs in exosomes versus cell lines, and A375 versus HEMa-LP exosomes by Ingenuity Analysis. Biological functions (A) and pathway analysis (B) of differentially expressed mRNAs in HEMa-LP exosomes versus HEMa-LP cells. Biological functions (C) and pathway analysis (D) of differentially expressed mRNAs in A375 exosomes versus A375 cells. Biological functions (E) and pathway analysis (F) of differentially expressed mRNAs in A375 exosomes versus HEMa-LP exosomes. (PPT) [file pone.0046874.s001.ppt]

## Slide 1
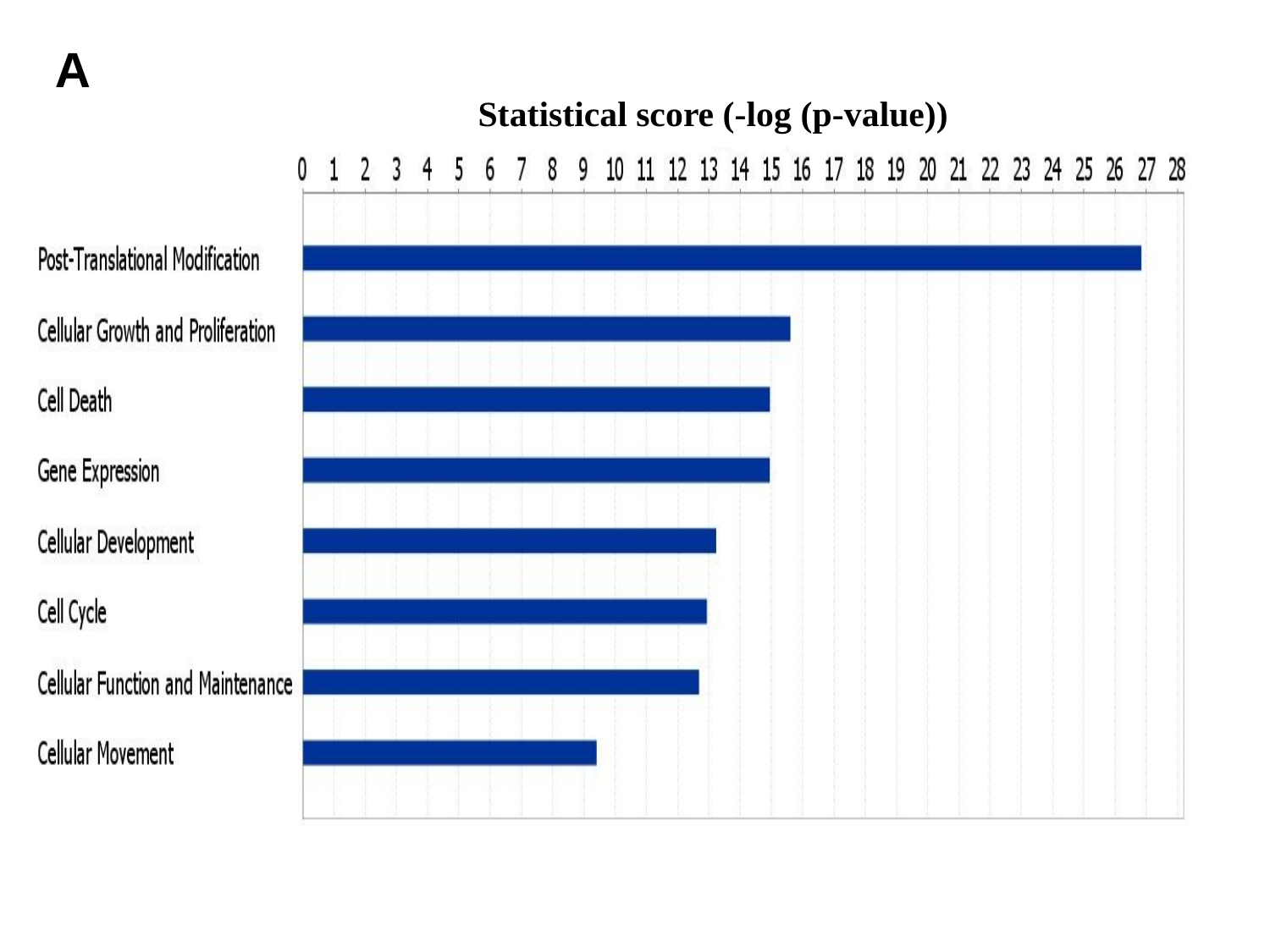

A
Statistical score (-log (p-value))

## Slide 2
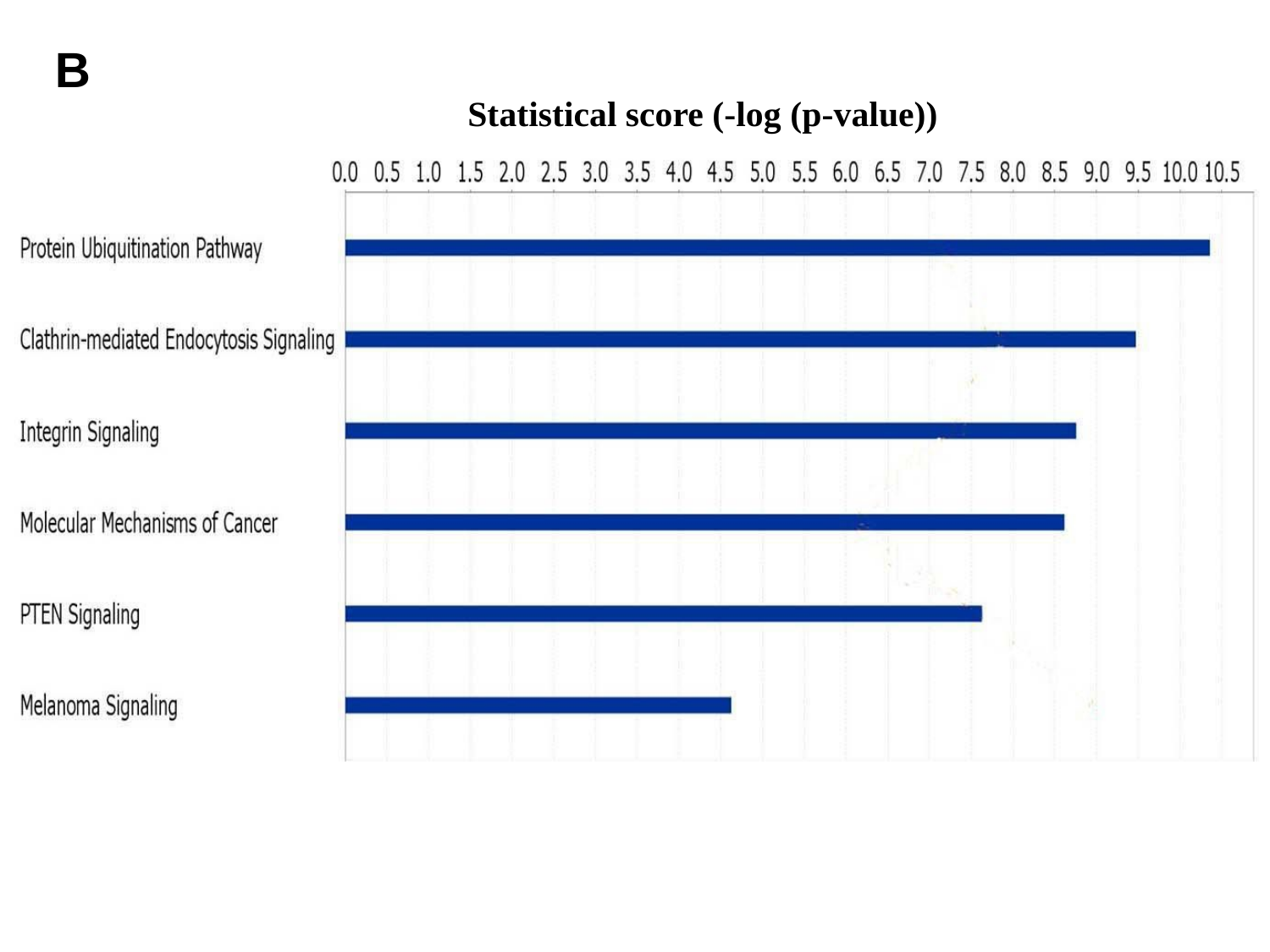

B
Statistical score (-log (p-value))

## Slide 3
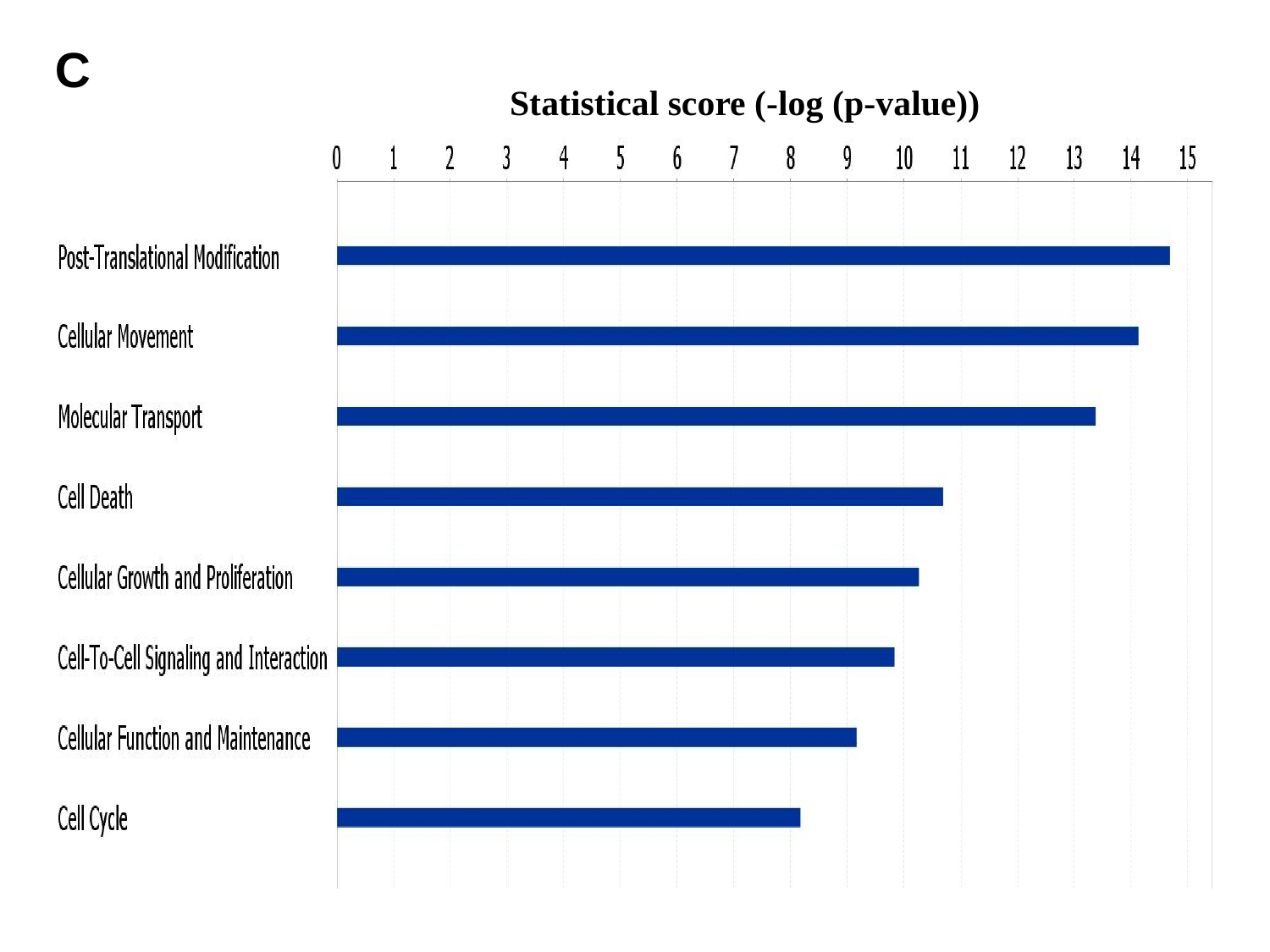

C
Statistical score (-log (p-value))

## Slide 4
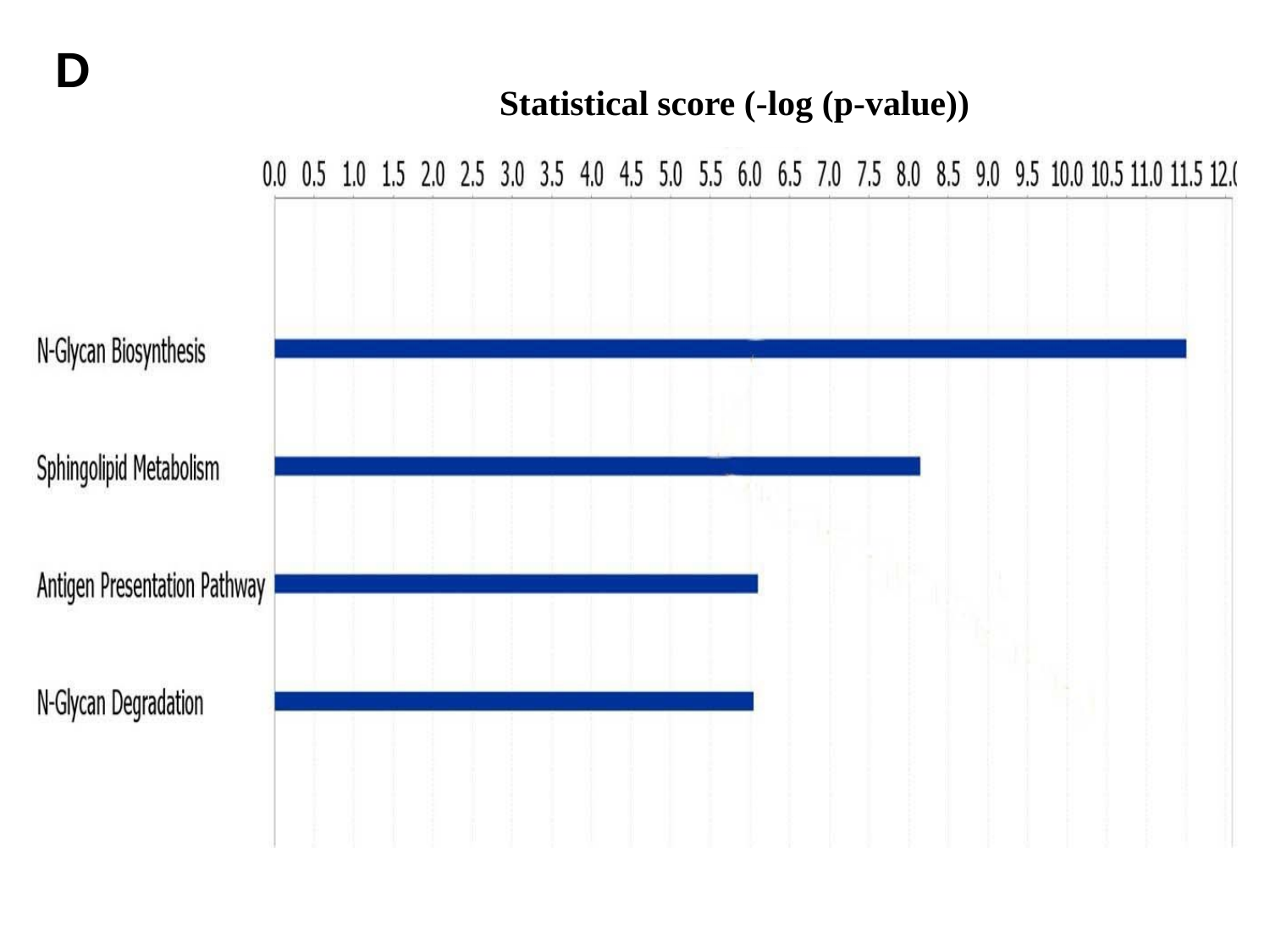

D
Statistical score (-log (p-value))

## Slide 5
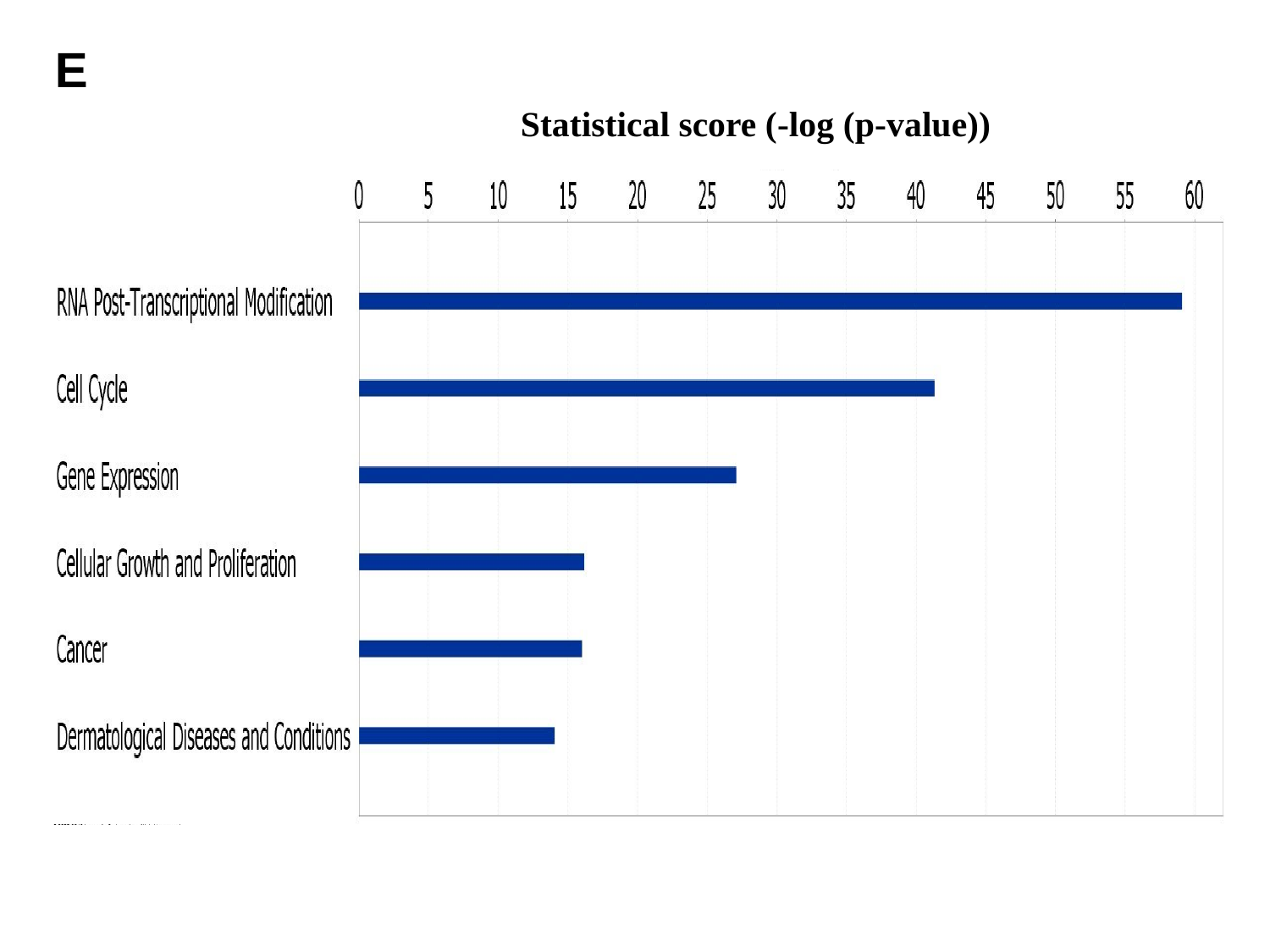

E
Statistical score (-log (p-value))

## Slide 6
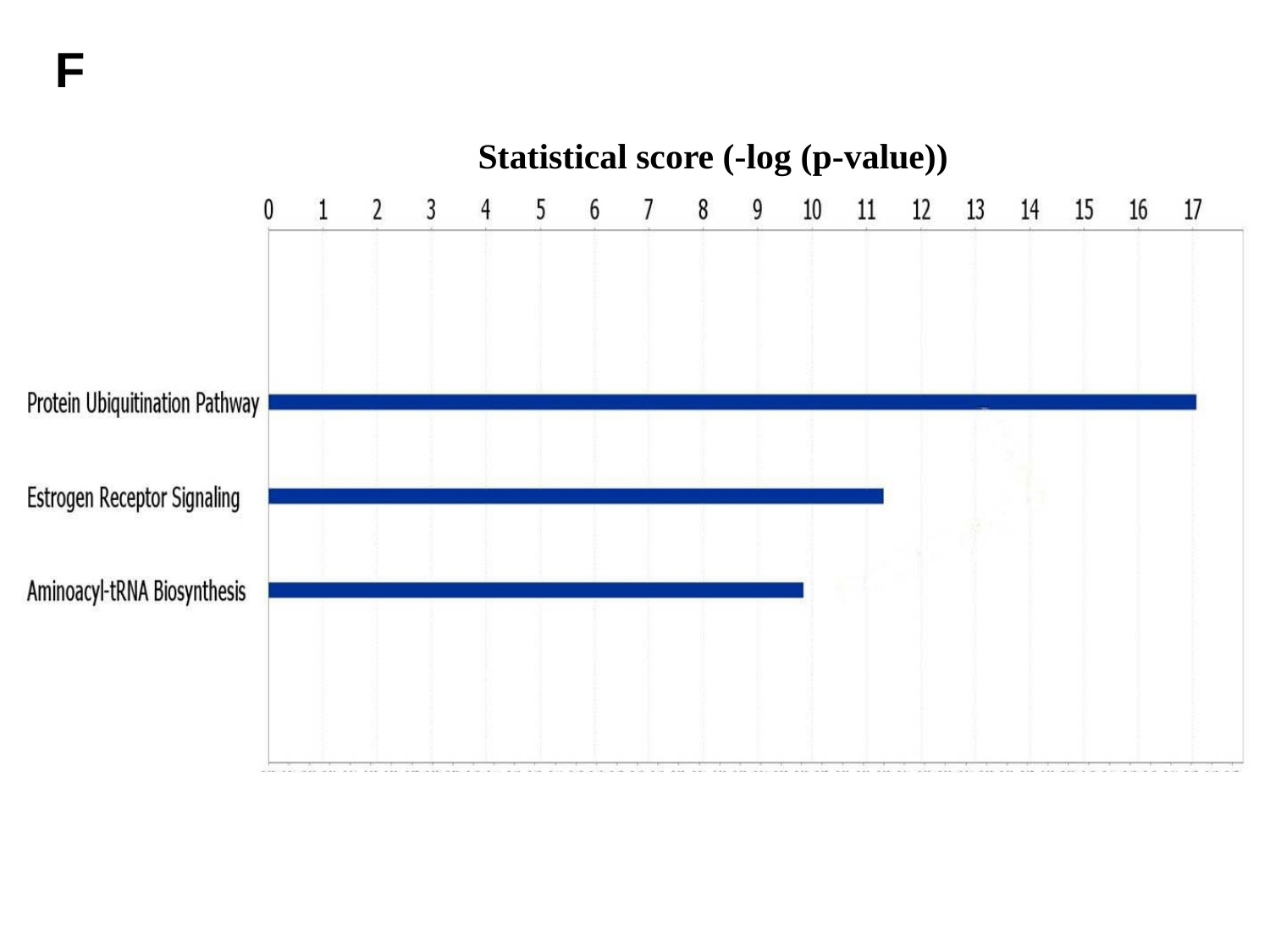

F
Statistical score (-log (p-value))
