# Supplementary figures and images for: Identifying mRNA, MicroRNA and Protein Profiles of Melanoma Exosomes
Source: PLoS One. 2012 Oct 9;7(10):e46874. doi: 10.1371/journal.pone.0046874 (PMC3467276; doi:10.1371/journal.pone.0046874)

## Slide 1
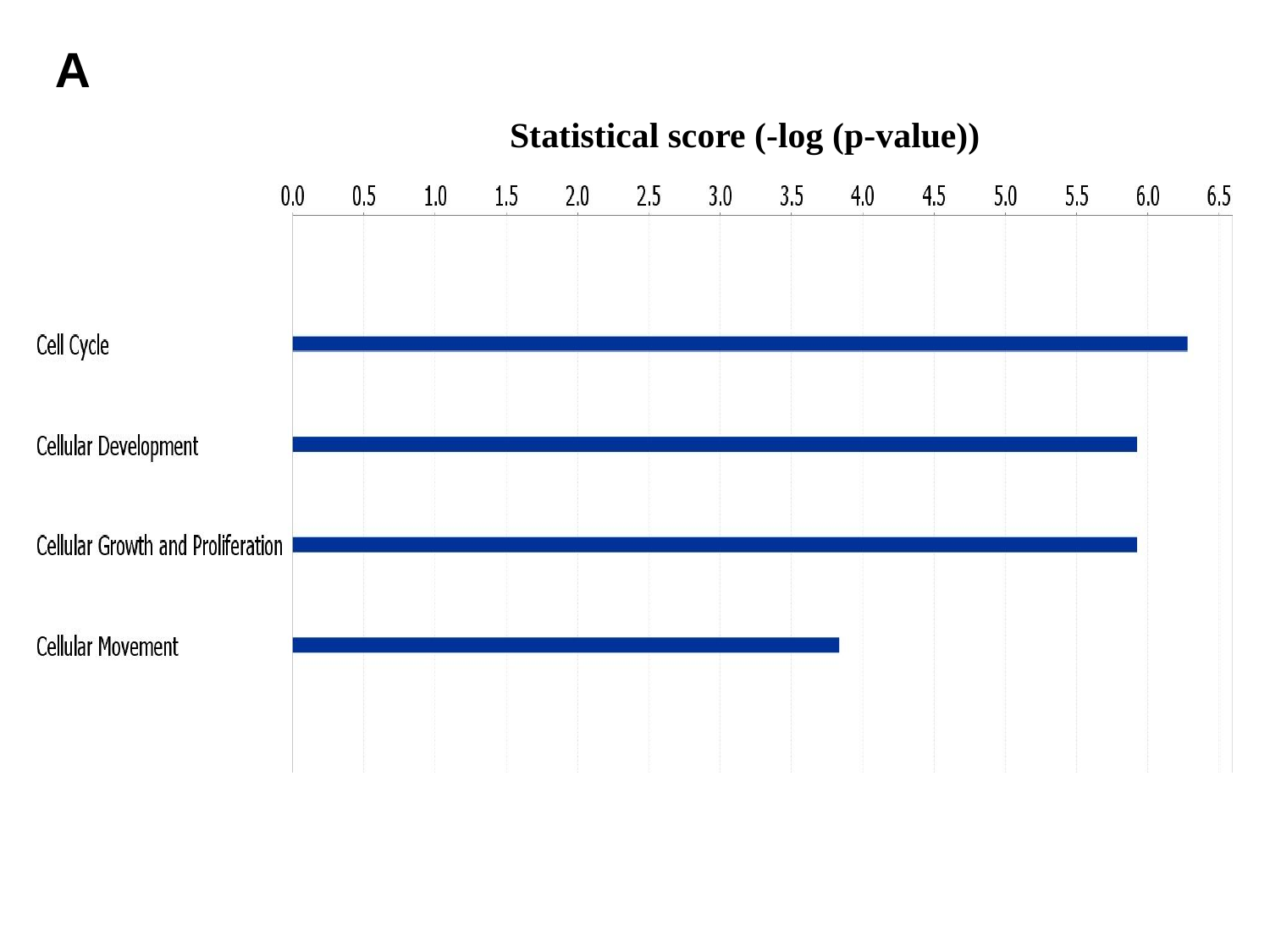

A
Statistical score (-log (p-value))

## Slide 2
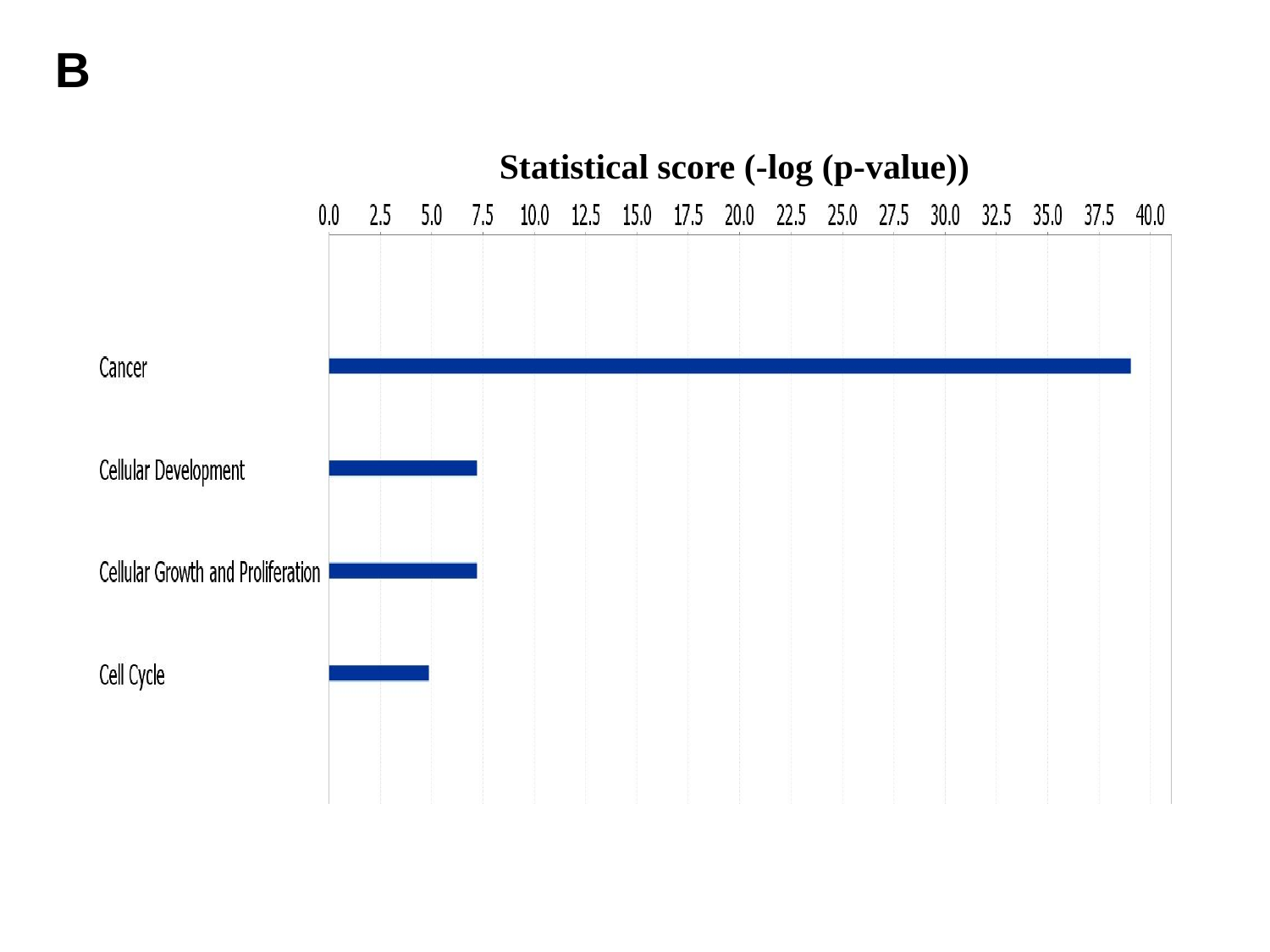

B
Statistical score (-log (p-value))

Supplement: Figure S2 — Differentially expressed miRNAs in exosomes versus cell lines, and A375 versus HEMa-LP exosomes by Ingenuity Analysis. Biological functions of differentially expressed miRNAs in HEMa-LP exosomes versus HEMa-LP cells (A) and in A375 exosomes versus HEMa-LP exosomes (B). (PPT) [file pone.0046874.s002.ppt]
