# Supplementary material for: Identifying mRNA, MicroRNA and Protein Profiles of Melanoma Exosomes
Source: PLoS One. 2012 Oct 9;7(10):e46874. doi: 10.1371/journal.pone.0046874 (PMC3467276; doi:10.1371/journal.pone.0046874)
Supplement: Table S1 — Differentially expressed mRNA probe sets in HEMa-LP exosomes versus HEMa-LP cells (FDR corrected p <0.01 and FC >5 or FC <−5). (DOCX) [file pone.0046874.s003.docx]

**Supplementary Table S1. Differentially expressed mRNA probe sets in HEMa-LP exosomes versus HEMa-LP cells (FDR corrected *p* < 0.01 and FC > 5 or FC < -5)**

| Probe Set ID | Gene Symbol | Gene Title | *p*-value | Fold Change |
| --- | --- | --- | --- | --- |
| 1558579_at | FLJ37786 | hypothetical LOC642691 | 3.37E-07 | 138.905 |
| 242344_at | GABRB2 | gamma-aminobutyric acid (GABA) A receptor, beta 2 | 3.30E-08 | 68.9541 |
| 232034_at | LOC203274 | Hypothetical protein LOC203274 | 3.80E-09 | 47.5815 |
| 243689_s_at | FRG1B | FSHD region gene 1 family, member B | 4.74E-07 | 46.7441 |
| 1553186_x_at | RASEF | RAS and EF-hand domain containing | 5.62E-09 | 43.9333 |
| 1553185_at | RASEF | RAS and EF-hand domain containing | 1.16E-08 | 43.4978 |
| 211565_at | SH3GL3 | SH3-domain GRB2-like 3 | 1.07E-07 | 42.523 |
| 222891_s_at | BCL11A | B-cell CLL/lymphoma 11A (zinc finger protein) | 2.98E-08 | 40.1701 |
| 232523_at | MEGF10 | multiple EGF-like-domains 10 | 5.28E-10 | 40.0672 |
| 244631_at | LOC389834 | ankyrin repeat domain 57 pseudogene | 2.64E-07 | 36.8863 |
| 228260_at | ELAVL2 | ELAV (embryonic lethal, abnormal vision, Drosophila)-like 2 (Hu antigen B) | 1.86E-08 | 36.609 |
| 1560431_at | PGM5P1 | phosphoglucomutase 5 pseudogene 1 | 2.96E-07 | 36.0777 |
| 220167_s_at | TP53TG3 /// TP53TG3B | TP53 target 3 /// TP53 target 3B | 6.13E-08 | 35.2851 |
| 1564856_s_at | LOC727924 | hypothetical LOC727924 | 2.86E-08 | 32.4877 |
| 242712_x_at | RANBP2 /// RGPD1 /// RGPD2 /// RGPD3 /// RGPD4 /// RGPD5 /// RGPD6 /// RGPD8 | RAN binding protein 2 /// RANBP2-like and GRIP domain containing 1 /// RANBP2-li | 3.92E-07 | 31.0186 |
| 210800_at | TIMM8A | translocase of inner mitochondrial membrane 8 homolog A (yeast) | 2.34E-06 | 30.4349 |
| 1558982_at | LOC375010 | ankyrin repeat domain 20 family, member A pseudogene | 2.26E-07 | 29.0335 |
| 224012_at | ANKRD20A1 /// ANKRD20A2 /// ANKRD20A3 /// ANKRD20A4 | ankyrin repeat domain 20 family, member A1 /// ankyrin repeat domain 20 family, | 4.69E-07 | 28.8746 |
| 233092_s_at | LOC100271840 | hypothetical LOC100271840 | 1.17E-07 | 27.1115 |
| 231882_at | FLJ39632 | hypothetical LOC642477 | 8.31E-07 | 27.075 |
| 224403_at | FCRL4 | Fc receptor-like 4 | 3.00E-07 | 26.5532 |
| 217137_x_at | LOC100289563 | hypothetical protein LOC100289563 | 8.17E-07 | 25.7191 |
| 224321_at | TMEFF2 | transmembrane protein with EGF-like and two follistatin-like domains 2 | 3.85E-10 | 25.609 |
| 224099_at | KCNH7 | potassium voltage-gated channel, subfamily H (eag-related), member 7 | 2.63E-07 | 24.8615 |
| 1558640_a_at | GUSBP1 | Glucuronidase, beta pseudogene 1 | 8.18E-08 | 24.5316 |
| 1570255_s_at | ANKRD20A1 /// ANKRD20A2 /// ANKRD20A3 /// ANKRD20A4 /// ANKRD20B /// LOC375010 | ankyrin repeat domain 20 family, member A1 /// ankyrin repeat domain 20 family, | 3.74E-06 | 24.2215 |
| 228321_s_at | NAA30 | N(alpha)-acetyltransferase 30, NatC catalytic subunit | 1.19E-07 | 22.4245 |
| 220771_at | LOC51152 | melanoma antigen | 1.19E-06 | 22.2945 |
| 1556876_s_at | TPTE2P2 | transmembrane phosphoinositide 3-phosphatase and tensin homolog 2 pseudogene 2 | 2.36E-06 | 21.5255 |
| 211494_s_at | SLC4A4 | solute carrier family 4, sodium bicarbonate cotransporter, member 4 | 5.65E-07 | 21.2251 |
| 1561271_at | CCDC144C /// LOC100134159 | coiled-coil domain containing 144C /// similar to Coiled-coil domain containing | 9.89E-07 | 21.1966 |
| 244508_at | 7-九月 | Septin 7 | 1.13E-06 | 21.1857 |
| 1568287_at | HMGA2 | high mobility group AT-hook 2 | 6.86E-06 | 21.048 |
| 1557283_a_at | ZNF519 | zinc finger protein 519 | 9.57E-08 | 20.5016 |
| 204712_at | WIF1 | WNT inhibitory factor 1 | 3.13E-06 | 20.4816 |
| 233815_at | NAALAD2 | N-acetylated alpha-linked acidic dipeptidase 2 | 3.72E-07 | 20.268 |
| 1560751_at | C18orf16 | chromosome 18 open reading frame 16 | 4.42E-07 | 20.1733 |
| 205782_at | FGF7 | fibroblast growth factor 7 (keratinocyte growth factor) | 3.32E-07 | 20.0793 |
| 1565483_at | EGFR | epidermal growth factor receptor (erythroblastic leukemia viral (v-erb-b) oncoge | 2.42E-06 | 19.9184 |
| 207135_at | HTR2A | 5-hydroxytryptamine (serotonin) receptor 2A | 9.22E-08 | 19.5119 |
| 216922_x_at | DAZ1 /// DAZ2 /// DAZ3 /// DAZ4 | deleted in azoospermia 1 /// deleted in azoospermia 2 /// deleted in azoospermia | 6.60E-07 | 18.7534 |
| 207598_x_at | XRCC2 | X-ray repair complementing defective repair in Chinese hamster cells 2 | 1.04E-06 | 18.5786 |
| 231124_x_at | LY9 | lymphocyte antigen 9 | 1.68E-08 | 18.363 |
| 211751_at | PDE4DIP | phosphodiesterase 4D interacting protein | 8.39E-07 | 18.3163 |
| 208281_x_at | DAZ1 /// DAZ2 /// DAZ3 /// DAZ4 | deleted in azoospermia 1 /// deleted in azoospermia 2 /// deleted in azoospermia | 1.97E-06 | 18.237 |
| 215599_at | GUSBP3 | glucuronidase, beta pseudogene 3 | 2.72E-07 | 18.1765 |
| 213382_at | MST1 /// MST1P2 /// MST1P9 | macrophage stimulating 1 (hepatocyte growth factor-like) /// macrophage stimulat | 6.84E-07 | 18.1638 |
| 1560207_at | C8orf81 | chromosome 8 open reading frame 81 | 5.99E-08 | 18.0491 |
| 222895_s_at | BCL11B | B-cell CLL/lymphoma 11B (zinc finger protein) | 1.77E-06 | 17.5268 |
| 232953_at | C20orf69 /// LOC100287060 /// LOC100287654 /// LOC100288169 /// LOC728323 /// PCMTD2 | chromosome 20 open reading frame 69 /// similar to hCG1984118 /// similar to Put | 3.12E-07 | 17.2513 |
| 224425_x_at | FKSG73 | ARP3 actin-related protein 3 homolog B pseudogene | 4.31E-07 | 17.2421 |
| 207480_s_at | MEIS2 | Meis homeobox 2 | 3.26E-09 | 17.0647 |
| 1557290_at | DPY19L2 /// DPY19L2P1 /// DPY19L2P2 /// DPY19L2P4 | dpy-19-like 2 (C. elegans) /// dpy-19-like 2 pseudogene 1 (C. elegans) /// dpy-1 | 4.27E-07 | 16.95 |
| 212386_at | TCF4 | transcription factor 4 | 1.79E-07 | 16.5269 |
| 231647_s_at | FCRL5 | Fc receptor-like 5 | 0.000363 | 16.4013 |
| 221698_s_at | CLEC7A | C-type lectin domain family 7, member A | 2.82E-06 | 16.2298 |
| 233096_at | KIAA1109 | KIAA1109 | 2.27E-05 | 16.098 |
| 242829_x_at | FBXL3 | F-box and leucine-rich repeat protein 3 | 2.19E-07 | 15.9876 |
| 220232_at | SCD5 | stearoyl-CoA desaturase 5 | 1.63E-07 | 15.9736 |
| 1559545_at | SNRPN | small nuclear ribonucleoprotein polypeptide N | 6.83E-08 | 15.9316 |
| 211682_x_at | UGT2B28 | UDP glucuronosyltransferase 2 family, polypeptide B28 | 1.27E-06 | 15.794 |
| 215228_at | NHLH2 | nescient helix loop helix 2 | 5.27E-07 | 15.4598 |
| 216229_x_at | HCG2P7 | HLA complex group 2 pseudogene 7 | 2.27E-06 | 15.4592 |
| 231024_at | LOC572558 | hypothetical locus LOC572558 | 3.57E-06 | 15.3549 |
| 232481_s_at | SLITRK6 | SLIT and NTRK-like family, member 6 | 9.70E-09 | 15.3461 |
| 1553633_s_at | NHEDC1 | Na+/H+ exchanger domain containing 1 | 1.11E-07 | 15.2614 |
| 224424_x_at | FKSG73 | ARP3 actin-related protein 3 homolog B pseudogene | 5.02E-06 | 15.2152 |
| 217487_x_at | FOLH1 | folate hydrolase (prostate-specific membrane antigen) 1 | 9.59E-08 | 15.1655 |
| 1552736_a_at | NETO1 | neuropilin (NRP) and tolloid (TLL)-like 1 | 4.77E-06 | 15.1257 |
| 206169_x_at | ZC3H7B | zinc finger CCCH-type containing 7B | 3.24E-05 | 15.09 |
| 205626_s_at | CALB1 | calbindin 1, 28kDa | 3.59E-08 | 15.068 |
| 220828_s_at | FLJ11292 | hypothetical protein FLJ11292 | 9.25E-07 | 14.978 |
| 219935_at | ADAMTS5 | ADAM metallopeptidase with thrombospondin type 1 motif, 5 | 8.55E-06 | 14.7879 |
| 1557080_s_at | ITGBL1 | integrin, beta-like 1 (with EGF-like repeat domains) | 5.50E-10 | 14.5994 |
| 1554741_s_at | FGF7 /// KGFLP1 /// KGFLP2 | fibroblast growth factor 7 (keratinocyte growth factor) /// keratinocyte growth | 8.44E-06 | 14.409 |
| 210718_s_at | ARL17A /// LOC100294341 | ADP-ribosylation factor-like 17A /// similar to ADP-ribosylation factor-like 17 | 2.37E-06 | 14.3763 |
| 1566145_s_at | LOC644450 | hypothetical protein LOC644450 | 1.95E-09 | 14.3436 |
| 206786_at | HTN3 | histatin 3 | 3.61E-07 | 14.3266 |
| 227646_at | EBF1 | early B-cell factor 1 | 4.14E-08 | 14.2196 |
| 223781_x_at | ADH4 | alcohol dehydrogenase 4 (class II), pi polypeptide | 4.13E-07 | 14.2157 |
| 224940_s_at | PAPPA | pregnancy-associated plasma protein A, pappalysin 1 | 1.72E-06 | 14.1057 |
| 234762_x_at | NLN | Neurolysin (metallopeptidase M3 family) | 6.78E-07 | 14.0921 |
| 216351_x_at | DAZ1 /// DAZ2 /// DAZ3 /// DAZ4 | deleted in azoospermia 1 /// deleted in azoospermia 2 /// deleted in azoospermia | 2.02E-05 | 14.0312 |
| 230040_at | ADAMTS18 | ADAM metallopeptidase with thrombospondin type 1 motif, 18 | 1.35E-06 | 13.958 |
| 235507_at | PCMTD1 | protein-L-isoaspartate (D-aspartate) O-methyltransferase domain containing 1 | 7.40E-07 | 13.9178 |
| 224095_at | LOC100128175 | similar to PRO2591 | 5.27E-08 | 13.8858 |
| 1552582_at | ABCC13 | ATP-binding cassette, sub-family C (CFTR/MRP), member 13, pseudogene | 6.73E-07 | 13.816 |
| 224402_s_at | FCRL4 | Fc receptor-like 4 | 6.62E-07 | 13.7876 |
| 215554_at | GPLD1 | glycosylphosphatidylinositol specific phospholipase D1 | 3.96E-06 | 13.7676 |
| 1564190_x_at | ZNF519 | zinc finger protein 519 | 3.46E-06 | 13.7395 |
| 236029_at | FAT3 | FAT tumor suppressor homolog 3 (Drosophila) | 6.55E-07 | 13.6953 |
| 216917_s_at | SYCP1 | synaptonemal complex protein 1 | 1.60E-07 | 13.6549 |
| 205529_s_at | RUNX1T1 | runt-related transcription factor 1; translocated to, 1 (cyclin D-related) | 5.03E-08 | 13.6502 |
| 1569940_at | SLC6A16 | Solute carrier family 6, member 16 | 5.90E-07 | 13.6482 |
| 1561477_at | CCDC144A | coiled-coil domain containing 144A | 1.02E-05 | 13.6357 |
| 215076_s_at | COL3A1 | collagen, type III, alpha 1 | 5.75E-06 | 13.6145 |
| 238605_at | NOL4 | nucleolar protein 4 | 6.70E-06 | 13.5797 |
| 229649_at | NRXN3 | neurexin 3 | 6.17E-07 | 13.5768 |
| 241624_at | LOC389834 | ankyrin repeat domain 57 pseudogene | 6.87E-08 | 13.5367 |
| 1555095_at | C6orf123 | chromosome 6 open reading frame 123 | 1.32E-06 | 13.4142 |
| 231886_at | LOC100134822 | similar to hCG1739109 | 2.03E-05 | 13.2675 |
| 219498_s_at | BCL11A | B-cell CLL/lymphoma 11A (zinc finger protein) | 1.62E-05 | 13.2558 |
| 232964_at | SPDYE1 | speedy homolog E1 (Xenopus laevis) | 7.18E-06 | 13.1297 |
| 235368_at | ADAMTS5 | ADAM metallopeptidase with thrombospondin type 1 motif, 5 | 1.19E-06 | 13.1121 |
| 222073_at | COL4A3 | collagen, type IV, alpha 3 (Goodpasture antigen) | 2.28E-07 | 13.0169 |
| 233604_at | FLJ22763 | hypothetical gene supported by AK026416 | 5.19E-06 | 12.9826 |
| 231213_at | PDE1A | phosphodiesterase 1A, calmodulin-dependent | 1.33E-05 | 12.8407 |
| 234331_s_at | FAM84A | family with sequence similarity 84, member A | 8.52E-07 | 12.8172 |
| 1556057_s_at | NEUROD1 | neurogenic differentiation 1 | 1.41E-06 | 12.7758 |
| 241977_s_at | RAB3C | RAB3C, member RAS oncogene family | 6.01E-06 | 12.7671 |
| 235957_at | GRIP1 | glutamate receptor interacting protein 1 | 1.66E-06 | 12.6361 |
| 1557261_at | WHAMML1 /// WHAMML2 | WAS protein homolog associated with actin, golgi membranes and microtubules-like | 5.71E-09 | 12.5771 |
| 206134_at | ADAMDEC1 | ADAM-like, decysin 1 | 3.05E-07 | 12.4275 |
| 244495_x_at | C18orf45 | chromosome 18 open reading frame 45 | 1.50E-06 | 12.4072 |
| 230645_at | FRMD3 | FERM domain containing 3 | 5.52E-06 | 12.3376 |
| 1555367_at | ZNF479 | zinc finger protein 479 | 3.74E-06 | 12.2383 |
| 215513_at | HYMAI | hydatidiform mole associated and imprinted (non-protein coding) | 2.18E-07 | 12.1618 |
| 219985_at | HS3ST3A1 | heparan sulfate (glucosamine) 3-O-sulfotransferase 3A1 | 1.64E-07 | 12.098 |
| 242766_at | ERLEC1P1 | endoplasmic reticulum lectin 1 pseudogene 1 | 1.29E-05 | 12.0617 |
| 214414_x_at | HBA1 /// HBA2 | hemoglobin, alpha 1 /// hemoglobin, alpha 2 | 1.10E-08 | 12.0233 |
| 224159_x_at | TRIM4 | tripartite motif-containing 4 | 2.81E-07 | 11.9999 |
| 1561197_at | LOC100294357 /// LOC442028 | hypothetical protein LOC100294357 /// hypothetical LOC442028 | 1.25E-06 | 11.988 |
| 234655_at | LOC100290132 | hypothetical protein LOC100290132 | 6.58E-06 | 11.9003 |
| 233463_at | RASSF6 | Ras association (RalGDS/AF-6) domain family member 6 | 1.28E-07 | 11.8262 |
| 244134_at | OXCT1 | 3-oxoacid CoA transferase 1 | 3.66E-09 | 11.7954 |
| 204679_at | KCNK1 | potassium channel, subfamily K, member 1 | 5.89E-07 | 11.79 |
| 220180_at | CCDC68 | coiled-coil domain containing 68 | 4.89E-06 | 11.7496 |
| 1562527_at | LOC283027 | hypothetical protein LOC283027 | 5.45E-06 | 11.7039 |
| 1555439_at | GTF3C3 | general transcription factor IIIC, polypeptide 3, 102kDa | 3.25E-06 | 11.7023 |
| 241399_at | FAM19A2 | family with sequence similarity 19 (chemokine (C-C motif)-like), member A2 | 1.42E-06 | 11.6547 |
| 240204_at | SNRPN | small nuclear ribonucleoprotein polypeptide N | 2.71E-06 | 11.6087 |
| 236266_at | RORA | RAR-related orphan receptor A | 1.69E-06 | 11.5429 |
| 206740_x_at | SYCP1 | synaptonemal complex protein 1 | 2.08E-05 | 11.4275 |
| 232547_at | SRCIN1 | SRC kinase signaling inhibitor 1 | 1.01E-05 | 11.4132 |
| 216837_at | EPHA5 | EPH receptor A5 | 7.91E-10 | 11.4004 |
| 224421_x_at | PMCHL1 | pro-melanin-concentrating hormone-like 1 | 3.81E-07 | 11.3846 |
| 207608_x_at | CYP1A2 | cytochrome P450, family 1, subfamily A, polypeptide 2 | 1.97E-07 | 11.3645 |
| 1565898_at | METT5D1 | Methyltransferase 5 domain containing 1 | 3.38E-05 | 11.3166 |
| 201893_x_at | DCN | decorin | 2.32E-06 | 11.2813 |
| 244313_at | CR1 | complement component (3b/4b) receptor 1 (Knops blood group) | 8.90E-06 | 11.236 |
| 201829_at | NET1 | neuroepithelial cell transforming 1 | 3.66E-06 | 11.2077 |
| 242770_at | LOC642236 | similar to FRG1 protein (FSHD region gene 1 protein) | 1.88E-06 | 11.2004 |
| 237737_at | LOC100289026 | similar to hCG1744891 | 2.45E-05 | 11.1388 |
| 224493_x_at | C18orf45 | chromosome 18 open reading frame 45 | 3.87E-06 | 11.1247 |
| 1566785_x_at | NSF | N-ethylmaleimide-sensitive factor | 2.00E-06 | 11.005 |
| 230258_at | GLIS3 | GLIS family zinc finger 3 | 2.11E-07 | 10.9499 |
| 224289_s_at | FKSG83 | FKSG83 | 1.32E-05 | 10.9238 |
| 210739_x_at | SLC4A4 | solute carrier family 4, sodium bicarbonate cotransporter, member 4 | 2.76E-06 | 10.9094 |
| 214594_x_at | ATP8B1 | ATPase, aminophospholipid transporter, class I, type 8B, member 1 | 0.000145 | 10.8839 |
| 1559716_at | INO80C | INO80 complex subunit C | 2.35E-07 | 10.8835 |
| 211585_at | NPAT | nuclear protein, ataxia-telangiectasia locus | 5.93E-07 | 10.8719 |
| 1566480_x_at | C17orf104 | Chromosome 17 open reading frame 104 | 1.06E-05 | 10.8625 |
| 229281_at | NPAS3 | neuronal PAS domain protein 3 | 8.11E-06 | 10.8085 |
| 215425_at | BTG3 | BTG family, member 3 | 3.30E-07 | 10.7598 |
| 209614_at | ADH1B | alcohol dehydrogenase 1B (class I), beta polypeptide | 1.23E-06 | 10.7518 |
| 1554642_at | RNF32 | ring finger protein 32 | 1.31E-07 | 10.5908 |
| 211454_x_at | FKSG49 | FKSG49 | 1.13E-08 | 10.5774 |
| 239481_at | FAM133A | family with sequence similarity 133, member A | 4.89E-06 | 10.5567 |
| 232073_at | PPFIA2 | protein tyrosine phosphatase, receptor type, f polypeptide (PTPRF), interacting | 2.10E-05 | 10.5174 |
| 208241_at | NRG1 | neuregulin 1 | 1.15E-06 | 10.4921 |
| 219497_s_at | BCL11A | B-cell CLL/lymphoma 11A (zinc finger protein) | 2.21E-07 | 10.4804 |
| 242912_at | P704P | prostate-specific P704P | 2.15E-05 | 10.4445 |
| 224418_x_at | PMCHL1 | pro-melanin-concentrating hormone-like 1 | 7.01E-06 | 10.4197 |
| 233549_at | PDE1A | phosphodiesterase 1A, calmodulin-dependent | 1.13E-08 | 10.4083 |
| 224519_at | LOC100132167 | similar to hCG1993567 | 1.60E-06 | 10.3684 |
| 231527_at | FLJ36840 | Hypothetical LOC645524 | 4.82E-06 | 10.3591 |
| 204913_s_at | SOX11 | SRY (sex determining region Y)-box 11 | 2.63E-07 | 10.3419 |
| 1563853_at | LOC283045 | hypothetical protein LOC283045 | 2.23E-07 | 10.2953 |
| 231031_at | KGFLP2 | keratinocyte growth factor-like protein 2 | 5.26E-07 | 10.2799 |
| 237475_x_at | CCDC152 | coiled-coil domain containing 152 | 5.01E-07 | 10.2686 |
| 211481_at | SLCO1A2 | solute carrier organic anion transporter family, member 1A2 | 6.62E-07 | 10.2636 |
| 1559224_at | LCE1E | late cornified envelope 1E | 7.32E-06 | 10.2287 |
| 206797_at | NAT2 | N-acetyltransferase 2 (arylamine N-acetyltransferase) | 1.36E-05 | 10.1816 |
| 1564160_at | DTHD1 | death domain containing 1 | 1.34E-06 | 10.1764 |
| 235638_at | RASSF6 | Ras association (RalGDS/AF-6) domain family member 6 | 1.60E-06 | 10.1763 |
| 224422_x_at | PMCHL2 | pro-melanin-concentrating hormone-like 2 | 2.92E-06 | 10.0815 |
| 1556183_at | FLJ40330 | hypothetical LOC645784 | 5.08E-06 | 10.0723 |
| 227662_at | SYNPO2 | synaptopodin 2 | 2.52E-07 | 10.0525 |
| 1555462_at | PPP1R1C | protein phosphatase 1, regulatory (inhibitor) subunit 1C | 2.79E-06 | 10.0456 |
| 209866_s_at | LPHN3 | latrophilin 3 | 1.17E-05 | 10.0363 |
| 1553422_s_at | A2BP1 | ataxin 2-binding protein 1 | 4.85E-06 | 10.0225 |
| 242578_x_at | SLC22A3 | Solute carrier family 22 (extraneuronal monoamine transporter), member 3 | 3.57E-07 | 9.92849 |
| 1555368_x_at | ZNF479 | zinc finger protein 479 | 1.43E-06 | 9.92015 |
| 1557618_at | LOC285768 | hypothetical LOC285768 | 9.35E-06 | 9.90256 |
| 236761_at | LHFPL3 | lipoma HMGIC fusion partner-like 3 | 1.09E-06 | 9.90219 |
| 244353_s_at | SLC2A12 | solute carrier family 2 (facilitated glucose transporter), member 12 | 2.62E-07 | 9.85407 |
| 242814_at | SERPINB9 | serpin peptidase inhibitor, clade B (ovalbumin), member 9 | 9.90E-06 | 9.84762 |
| 1570351_at | ADAMTS6 | ADAM metallopeptidase with thrombospondin type 1 motif, 6 | 1.78E-06 | 9.84457 |
| 232266_x_at | CDK13 | Cyclin-dependent kinase 13 | 2.07E-07 | 9.8321 |
| 206794_at | ERBB4 | v-erb-a erythroblastic leukemia viral oncogene homolog 4 (avian) | 2.66E-05 | 9.80353 |
| 209540_at | IGF1 | insulin-like growth factor 1 (somatomedin C) | 2.74E-07 | 9.78443 |
| 208120_x_at | FKSG49 | FKSG49 | 1.10E-09 | 9.78132 |
| 215856_at | SIGLEC15 | sialic acid binding Ig-like lectin 15 | 1.36E-06 | 9.69515 |
| 220115_s_at | CDH10 | cadherin 10, type 2 (T2-cadherin) | 1.30E-07 | 9.67574 |
| 238701_x_at | C11orf92 | chromosome 11 open reading frame 92 | 4.33E-08 | 9.60983 |
| 1562439_at | NCOA3 | Nuclear receptor coactivator 3 | 5.88E-06 | 9.60494 |
| 211616_s_at | HTR2A | 5-hydroxytryptamine (serotonin) receptor 2A | 3.93E-06 | 9.56393 |
| 1557664_at | LOC340239 | Hypothetical LOC340239 | 2.30E-05 | 9.52802 |
| 207936_x_at | RFPL3 | ret finger protein-like 3 | 3.15E-06 | 9.52377 |
| 1562440_at | MAP3K13 | Mitogen-activated protein kinase kinase kinase 13 | 5.87E-07 | 9.45398 |
| 1558195_at | LOC283404 | hypothetical LOC283404 | 1.82E-06 | 9.41915 |
| 1553153_at | ATP6V0D2 | ATPase, H+ transporting, lysosomal 38kDa, V0 subunit d2 | 1.46E-06 | 9.41336 |
| 1553666_at | CCDC34 | coiled-coil domain containing 34 | 1.03E-05 | 9.40152 |
| 214770_at | MSR1 | macrophage scavenger receptor 1 | 4.27E-05 | 9.39431 |
| 232099_at | PCDHB16 | protocadherin beta 16 | 1.49E-06 | 9.37857 |
| 1556288_at | C18orf62 | chromosome 18 open reading frame 62 | 2.11E-06 | 9.37762 |
| 1555141_a_at | NHEDC1 | Na+/H+ exchanger domain containing 1 | 1.74E-06 | 9.36849 |
| 242546_at | FLJ39632 | hypothetical LOC642477 | 1.57E-06 | 9.33852 |
| 220026_at | CLCA4 | chloride channel accessory 4 | 4.05E-05 | 9.32392 |
| 224419_x_at | PMCHL1 | pro-melanin-concentrating hormone-like 1 | 4.54E-06 | 9.3056 |
| 224370_s_at | CAPS2 | calcyphosine 2 | 3.57E-06 | 9.28225 |
| 206060_s_at | PTPN22 | protein tyrosine phosphatase, non-receptor type 22 (lymphoid) | 1.04E-05 | 9.26977 |
| 206826_at | PMP2 | peripheral myelin protein 2 | 4.36E-07 | 9.26633 |
| 214043_at | PTPRD | protein tyrosine phosphatase, receptor type, D | 5.12E-08 | 9.26534 |
| 216452_at | TRPM3 | transient receptor potential cation channel, subfamily M, member 3 | 4.24E-06 | 9.19368 |
| 241871_at | CAMK4 | calcium/calmodulin-dependent protein kinase IV | 1.49E-05 | 9.19344 |
| 233437_at | GABRA4 | gamma-aminobutyric acid (GABA) A receptor, alpha 4 | 5.61E-06 | 9.16523 |
| 1557122_s_at | GABRB2 | gamma-aminobutyric acid (GABA) A receptor, beta 2 | 8.38E-07 | 9.15095 |
| 219454_at | EGFL6 | EGF-like-domain, multiple 6 | 2.33E-08 | 9.15037 |
| 1552712_a_at | NMNAT2 | nicotinamide nucleotide adenylyltransferase 2 | 1.93E-07 | 9.12799 |
| 205609_at | ANGPT1 | angiopoietin 1 | 2.25E-06 | 9.11963 |
| 1554528_at | C3orf15 | chromosome 3 open reading frame 15 | 2.06E-06 | 9.11517 |
| 220205_at | TPTE | transmembrane phosphatase with tensin homology | 2.99E-07 | 9.09168 |
| 239748_x_at | OCIAD1 | OCIA domain containing 1 | 1.04E-06 | 9.08348 |
| 215311_at | NTRK3 | neurotrophic tyrosine kinase, receptor, type 3 | 3.48E-08 | 9.04653 |
| 1569960_at | BRD7P3 | bromodomain containing 7 pseudogene 3 | 2.69E-05 | 8.98622 |
| 1565484_x_at | EGFR | epidermal growth factor receptor (erythroblastic leukemia viral (v-erb-b) oncoge | 1.43E-06 | 8.98201 |
| 1560002_at | FAM27D1 | family with sequence similarity 27, member D1 | 7.53E-06 | 8.9783 |
| 214120_at | RFPL1S | RFPL1 antisense RNA (non-protein coding) | 1.94E-06 | 8.9498 |
| 222187_x_at | G3BP1 | GTPase activating protein (SH3 domain) binding protein 1 | 1.01E-06 | 8.94382 |
| 230008_at | THSD7A | thrombospondin, type I, domain containing 7A | 3.30E-06 | 8.91886 |
| 1566190_at | SUZ12 | Suppressor of zeste 12 homolog (Drosophila) | 7.44E-06 | 8.9022 |
| 1568646_x_at | ZNF208 | zinc finger protein 208 | 8.49E-07 | 8.86018 |
| 215563_s_at | MST1P9 | macrophage stimulating 1 (hepatocyte growth factor-like) pseudogene 9 | 5.65E-06 | 8.85656 |
| 209071_s_at | RGS5 | regulator of G-protein signaling 5 | 1.18E-05 | 8.8426 |
| 240863_at | CYP19A1 | cytochrome P450, family 19, subfamily A, polypeptide 1 | 3.35E-05 | 8.83988 |
| 220796_x_at | SLC35E1 | solute carrier family 35, member E1 | 8.38E-06 | 8.82519 |
| 1570222_at | NDST4 | N-deacetylase/N-sulfotransferase (heparan glucosaminyl) 4 | 5.47E-08 | 8.79614 |
| 220184_at | NANOG | Nanog homeobox | 1.82E-06 | 8.78619 |
| 215588_x_at | RIOK3 | RIO kinase 3 (yeast) | 5.06E-07 | 8.772 |
| 214162_at | LOC284244 | hypothetical protein LOC284244 | 1.21E-05 | 8.75935 |
| 1560662_s_at | WHAMML1 /// WHAMML2 | WAS protein homolog associated with actin, golgi membranes and microtubules-like | 9.35E-05 | 8.75865 |
| 1557770_at | IPO11 | importin 11 | 2.89E-06 | 8.70608 |
| 215208_x_at | RPL35A | Ribosomal protein L35a | 1.19E-06 | 8.68476 |
| 219908_at | DKK2 | dickkopf homolog 2 (Xenopus laevis) | 7.06E-06 | 8.68274 |
| 1568986_x_at | PIGT | phosphatidylinositol glycan anchor biosynthesis, class T | 6.13E-05 | 8.67617 |
| 234314_at | RALGAPA2 | Ral GTPase activating protein, alpha subunit 2 (catalytic) | 4.57E-07 | 8.6733 |
| 1562244_at | ZNF578 | Zinc finger protein 578 | 6.74E-06 | 8.65299 |
| 230895_at | HAPLN1 | hyaluronan and proteoglycan link protein 1 | 1.61E-07 | 8.6498 |
| 1555189_a_at | TAT | tyrosine aminotransferase | 5.03E-06 | 8.63154 |
| 213350_at | RPS11 | Ribosomal protein S11 | 1.60E-06 | 8.59439 |
| 1553471_at | AMAC1 | acyl-malonyl condensing enzyme 1 | 2.07E-07 | 8.59397 |
| 214920_at | THSD7A | thrombospondin, type I, domain containing 7A | 1.50E-05 | 8.59255 |
| 1553461_at | FAM9B | family with sequence similarity 9, member B | 1.12E-06 | 8.57764 |
| 236783_at | KCNIP4 | Kv channel interacting protein 4 | 4.68E-06 | 8.56494 |
| 224426_s_at | FKSG73 | ARP3 actin-related protein 3 homolog B pseudogene | 6.16E-06 | 8.55953 |
| 219743_at | HEY2 | hairy/enhancer-of-split related with YRPW motif 2 | 0.00014 | 8.5075 |
| 229349_at | LIN28B | lin-28 homolog B (C. elegans) | 6.11E-08 | 8.50273 |
| 206404_at | FGF9 | fibroblast growth factor 9 (glia-activating factor) | 4.42E-05 | 8.50226 |
| 210409_at | C6orf124 | chromosome 6 open reading frame 124 | 2.14E-06 | 8.47987 |
| 209840_s_at | LRRN3 | leucine rich repeat neuronal 3 | 3.65E-07 | 8.4774 |
| 232327_at | THSD7B | thrombospondin, type I, domain containing 7B | 8.88E-06 | 8.47615 |
| 1563906_at | SOBP | sine oculis binding protein homolog (Drosophila) | 1.28E-06 | 8.46639 |
| 243762_at | LOC100287445 | hypothetical protein LOC100287445 | 2.63E-06 | 8.45998 |
| 232644_x_at | OCIAD1 | OCIA domain containing 1 | 3.71E-09 | 8.42598 |
| 220374_at | KLHL28 | kelch-like 28 (Drosophila) | 3.68E-05 | 8.4225 |
| 206792_x_at | PDE4C | phosphodiesterase 4C, cAMP-specific (phosphodiesterase E1 dunce homolog, Drosoph | 1.68E-06 | 8.39323 |
| 206370_at | PIK3CG | phosphoinositide-3-kinase, catalytic, gamma polypeptide | 2.22E-07 | 8.39241 |
| 216595_at | FAM186A | family with sequence similarity 186, member A | 2.16E-05 | 8.39118 |
| 1560734_at | LOC727924 | hypothetical LOC727924 | 3.47E-06 | 8.38995 |
| 211568_at | BAI3 | brain-specific angiogenesis inhibitor 3 | 2.38E-06 | 8.34976 |
| 206960_at | LPAR4 | lysophosphatidic acid receptor 4 | 3.33E-06 | 8.32682 |
| 238303_at | STT3B | STT3, subunit of the oligosaccharyltransferase complex, homolog B (S. cerevisiae | 1.81E-06 | 8.30273 |
| 242601_at | HEPACAM2 | HEPACAM family member 2 | 5.58E-07 | 8.28178 |
| 232814_x_at | C14orf153 | Chromosome 14 open reading frame 153 | 9.13E-07 | 8.25054 |
| 206639_x_at | HTN1 | histatin 1 | 1.89E-05 | 8.24371 |
| 1562664_at | LOC286009 | hypothetical protein LOC286009 | 6.55E-06 | 8.23723 |
| 1564277_a_at | LOC100133920 /// LOC286297 | hypothetical protein LOC100133920 /// hypothetical protein LOC286297 | 2.36E-05 | 8.23109 |
| 205608_s_at | ANGPT1 | angiopoietin 1 | 1.52E-05 | 8.23079 |
| 229600_s_at | CPD | Carboxypeptidase D | 1.91E-06 | 8.21808 |
| 215323_at | LUZP2 | leucine zipper protein 2 | 1.55E-05 | 8.21623 |
| 230518_at | MPZL2 | myelin protein zero-like 2 | 2.64E-05 | 8.19836 |
| 241331_at | SKAP2 | Src kinase associated phosphoprotein 2 | 3.02E-06 | 8.17082 |
| 207874_s_at | CFHR4 | complement factor H-related 4 | 1.06E-05 | 8.1633 |
| 207575_at | GOLGA6A | golgin A6 family, member A | 3.10E-08 | 8.15294 |
| 222965_at | PRO2214 | hypothetical protein PRO2214 | 8.52E-06 | 8.14416 |
| 1561557_at | LIPA | Lipase A, lysosomal acid, cholesterol esterase | 1.33E-07 | 8.11809 |
| 207056_s_at | SLC4A8 | solute carrier family 4, sodium bicarbonate cotransporter, member 8 | 4.27E-05 | 8.10716 |
| 230061_at | TM4SF18 | Transmembrane 4 L six family member 18 | 4.87E-05 | 8.02608 |
| 234486_at | OR51B2 | olfactory receptor, family 51, subfamily B, member 2 | 4.35E-07 | 8.02419 |
| 1561254_at | LOC340340 | hypothetical LOC340340 | 8.30E-07 | 8.01977 |
| 221238_at | HMGN5 | high-mobility group nucleosome binding domain 5 | 1.80E-05 | 8.01507 |
| 225834_at | FAM72A /// FAM72B /// FAM72C /// FAM72D | family with sequence similarity 72, member A /// family with sequence similarity | 7.78E-07 | 8.00033 |
| 224288_x_at | FKSG49 | FKSG49 | 2.84E-06 | 7.98702 |
| 231441_at | C7orf62 | chromosome 7 open reading frame 62 | 2.82E-07 | 7.97848 |
| 206323_x_at | OPHN1 | oligophrenin 1 | 3.03E-09 | 7.97348 |
| 234949_at | FRG1B | FSHD region gene 1 family, member B | 2.94E-06 | 7.96154 |
| 220113_x_at | POLR1B | polymerase (RNA) I polypeptide B, 128kDa | 5.29E-07 | 7.95143 |
| 207706_at | USH2A | Usher syndrome 2A (autosomal recessive, mild) | 1.91E-05 | 7.93531 |
| 1562484_at | C17orf104 | chromosome 17 open reading frame 104 | 1.19E-05 | 7.92897 |
| 211821_x_at | GYPA | glycophorin A (MNS blood group) | 2.72E-06 | 7.92049 |
| 213222_at | PLCB1 | phospholipase C, beta 1 (phosphoinositide-specific) | 2.10E-06 | 7.92039 |
| 227235_at | GUCY1A3 | guanylate cyclase 1, soluble, alpha 3 | 2.02E-06 | 7.89219 |
| 205559_s_at | PCSK5 | proprotein convertase subtilisin/kexin type 5 | 1.83E-05 | 7.87988 |
| 220645_at | FAM55D | family with sequence similarity 55, member D | 1.58E-06 | 7.87644 |
| 207725_at | POU4F2 | POU class 4 homeobox 2 | 9.38E-08 | 7.87576 |
| 1552411_at | DEFB106A /// DEFB106B | defensin, beta 106A /// defensin, beta 106B | 1.80E-05 | 7.85518 |
| 243109_at | MCTP2 | multiple C2 domains, transmembrane 2 | 3.95E-05 | 7.85383 |
| 1563474_at | C12orf72 | chromosome 12 open reading frame 72 | 3.36E-06 | 7.84676 |
| 216440_at | ERC1 | ELKS/RAB6-interacting/CAST family member 1 | 2.47E-06 | 7.83976 |
| 214707_x_at | ALMS1 | Alstrom syndrome 1 | 2.84E-06 | 7.81186 |
| 220405_at | SNTG1 | syntrophin, gamma 1 | 3.11E-06 | 7.8101 |
| 233241_at | PLK1S1 | polo-like kinase 1 substrate 1 | 1.02E-06 | 7.80721 |
| 228128_x_at | PAPPA | pregnancy-associated plasma protein A, pappalysin 1 | 1.94E-05 | 7.79939 |
| 223977_s_at | C18orf2 | chromosome 18 open reading frame 2 | 1.42E-06 | 7.7898 |
| 222253_s_at | POM121L9P | POM121 membrane glycoprotein-like 9 (rat) pseudogene | 1.61E-06 | 7.78052 |
| 240268_at | LOC440117 | hypothetical gene supported by BC037858 | 2.67E-06 | 7.77907 |
| 234341_x_at | LOC91548 | hypothetical protein LOC91548 | 4.20E-09 | 7.77713 |
| 230496_at | FAM123A | family with sequence similarity 123A | 9.94E-07 | 7.77477 |
| 205656_at | PCDH17 | protocadherin 17 | 1.32E-06 | 7.77211 |
| 1557374_at | ABCC9 | ATP-binding cassette, sub-family C (CFTR/MRP), member 9 | 4.34E-06 | 7.74658 |
| 207010_at | GABRB1 | gamma-aminobutyric acid (GABA) A receptor, beta 1 | 1.15E-05 | 7.73632 |
| 230869_at | FAM155A | family with sequence similarity 155, member A | 3.13E-06 | 7.71837 |
| 217483_at | FOLH1 | folate hydrolase (prostate-specific membrane antigen) 1 | 5.29E-08 | 7.70712 |
| 1563674_at | FCRL2 | Fc receptor-like 2 | 3.27E-07 | 7.70085 |
| 214735_at | IPCEF1 | interaction protein for cytohesin exchange factors 1 | 3.74E-05 | 7.68237 |
| 206318_at | SPINLW1 | serine peptidase inhibitor-like, with Kunitz and WAP domains 1 (eppin) | 9.35E-07 | 7.67585 |
| 234712_at | C18orf2 | chromosome 18 open reading frame 2 | 1.37E-06 | 7.67556 |
| 215467_x_at | LOC647070 | hypothetical LOC647070 | 2.49E-08 | 7.66828 |
| 1554846_at | CCDC158 | coiled-coil domain containing 158 | 1.28E-07 | 7.64278 |
| 1553373_at | WDR64 | WD repeat domain 64 | 2.13E-08 | 7.63999 |
| 206048_at | OVOL2 | ovo-like 2 (Drosophila) | 4.63E-05 | 7.63718 |
| 228610_at | TM9SF3 | Transmembrane 9 superfamily member 3 | 2.31E-05 | 7.63022 |
| 213737_x_at | LOC728498 | Golgin subfamily A member 8-like protein 1 | 1.63E-06 | 7.61393 |
| 1563725_at | ZNF583 | zinc finger protein 583 | 2.33E-05 | 7.59004 |
| 1555071_at | TLL1 | tolloid-like 1 | 9.21E-06 | 7.58891 |
| 1554516_at | LOC100288109 /// LOC203274 | Hypothetical protein LOC100288109 /// Hypothetical protein LOC203274 | 4.81E-06 | 7.58863 |
| 206051_at | ELAVL4 | ELAV (embryonic lethal, abnormal vision, Drosophila)-like 4 (Hu antigen D) | 3.20E-06 | 7.58669 |
| 214222_at | DNAH7 | dynein, axonemal, heavy chain 7 | 1.57E-06 | 7.56382 |
| 216999_at | EPOR | erythropoietin receptor | 2.87E-06 | 7.54583 |
| 216206_x_at | MAP2K7 | mitogen-activated protein kinase kinase 7 | 0.000219 | 7.54419 |
| 228945_s_at | SLC39A8 | Solute carrier family 39 (zinc transporter), member 8 | 2.16E-06 | 7.52023 |
| 216786_at | NCRNA00230A | non-protein coding RNA 230A | 1.69E-05 | 7.49966 |
| 1564281_at | LOC285708 | hypothetical protein LOC285708 | 0.000861 | 7.49801 |
| 1561492_at | LOC647107 | hypothetical protein LOC647107 | 1.80E-05 | 7.47897 |
| 229994_at | NFIA | Nuclear factor I/A | 2.00E-06 | 7.47085 |
| 1554038_at | LARP1B | La ribonucleoprotein domain family, member 1B | 1.86E-07 | 7.46917 |
| 210729_at | NPY2R | neuropeptide Y receptor Y2 | 0.000124 | 7.46917 |
| 1553391_at | CXorf58 | chromosome X open reading frame 58 | 1.72E-06 | 7.46904 |
| 229084_at | CNTN4 | contactin 4 | 3.68E-05 | 7.46883 |
| 204406_at | FLT1 | fms-related tyrosine kinase 1 (vascular endothelial growth factor/vascular perme | 3.66E-07 | 7.44671 |
| 217282_at | MAN1A2 | mannosidase, alpha, class 1A, member 2 | 7.74E-07 | 7.43717 |
| 205381_at | LRRC17 | leucine rich repeat containing 17 | 3.35E-06 | 7.42622 |
| 208305_at | PGR | progesterone receptor | 2.86E-07 | 7.41921 |
| 1556770_a_at | FBXL13 | F-box and leucine-rich repeat protein 13 | 5.29E-08 | 7.4056 |
| 216877_at | DKFZp686O1327 | Hypothetical gene supported by BC043549; BX648102 | 2.73E-06 | 7.40458 |
| 225720_at | SYNPO2 | synaptopodin 2 | 3.20E-07 | 7.35825 |
| 1563505_at | DUSP16 | Dual specificity phosphatase 16 | 1.82E-06 | 7.33103 |
| 214354_x_at | SFTPB | surfactant protein B | 1.09E-06 | 7.30525 |
| 212387_at | TCF4 | transcription factor 4 | 4.99E-05 | 7.29405 |
| 1554141_s_at | WDR78 | WD repeat domain 78 | 2.84E-06 | 7.29102 |
| 232904_at | SLC7A14 | solute carrier family 7 (cationic amino acid transporter, y+ system), member 14 | 8.14E-06 | 7.29017 |
| 238073_at | ELAVL4 | ELAV (embryonic lethal, abnormal vision, Drosophila)-like 4 (Hu antigen D) | 1.22E-05 | 7.284 |
| 1558784_at | LOC100133089 | hypothetical protein LOC100133089 | 2.76E-08 | 7.26678 |
| 1561225_at | LOC338579 | hypothetical protein LOC338579 | 6.61E-07 | 7.26068 |
| 216153_x_at | RECK | reversion-inducing-cysteine-rich protein with kazal motifs | 1.80E-08 | 7.25781 |
| 239052_at | HNRNPD | Heterogeneous nuclear ribonucleoprotein D (AU-rich element RNA binding protein 1 | 8.19E-06 | 7.25047 |
| 233781_s_at | RIF1 | RAP1 interacting factor homolog (yeast) | 7.34E-09 | 7.22806 |
| 1562367_at | C15orf54 | chromosome 15 open reading frame 54 | 5.39E-06 | 7.21573 |
| 228553_at | ENAH | Enabled homolog (Drosophila) | 3.62E-06 | 7.2098 |
| 207409_at | LECT2 | leukocyte cell-derived chemotaxin 2 | 5.31E-06 | 7.19994 |
| 1558534_at | LOC641298 | SMG1 homolog, phosphatidylinositol 3-kinase-related kinase pseudogene | 8.32E-07 | 7.19334 |
| 210195_s_at | PSG1 | pregnancy specific beta-1-glycoprotein 1 | 3.98E-06 | 7.18802 |
| 210853_at | SCN11A | sodium channel, voltage-gated, type XI, alpha subunit | 8.82E-07 | 7.18539 |
| 214927_at | ITGBL1 | integrin, beta-like 1 (with EGF-like repeat domains) | 6.97E-08 | 7.15484 |
| 1558792_x_at | AP2A1 | Adaptor-related protein complex 2, alpha 1 subunit | 0.000351 | 7.13598 |
| 210742_at | CDC14A | CDC14 cell division cycle 14 homolog A (S. cerevisiae) | 1.40E-06 | 7.13164 |
| 220394_at | FGF20 | fibroblast growth factor 20 | 1.31E-06 | 7.13098 |
| 210498_at | CLTC | clathrin, heavy chain (Hc) | 2.95E-05 | 7.12424 |
| 223662_x_at | DDX59 | DEAD (Asp-Glu-Ala-Asp) box polypeptide 59 | 1.90E-07 | 7.12233 |
| 220725_x_at | DNAH3 | Dynein, axonemal, heavy chain 3 | 2.86E-10 | 7.10653 |
| 208443_x_at | SHOX2 | short stature homeobox 2 | 8.10E-06 | 7.10239 |
| 1563009_at | LOC284930 | Hypothetical protein LOC284930 | 1.04E-06 | 7.09894 |
| 221154_at | TRIM49 | tripartite motif-containing 49 | 1.24E-05 | 7.08302 |
| 215518_at | STXBP5L | syntaxin binding protein 5-like | 6.15E-06 | 7.07025 |
| 244395_at | FLJ41455 | hypothetical gene supported by AK123449; BX641014 | 0.000185 | 7.06736 |
| 1558601_at | LOC285194 | hypothetical LOC285194 | 2.25E-05 | 7.05173 |
| 243254_at | HIVEP2 | human immunodeficiency virus type I enhancer binding protein 2 | 3.11E-08 | 7.04822 |
| 1552745_at | SLCO6A1 | solute carrier organic anion transporter family, member 6A1 | 5.84E-06 | 7.0444 |
| 217016_x_at | TMEM212 | transmembrane protein 212 | 1.82E-05 | 7.04296 |
| 1562256_at | LOC728392 /// NLRP1 | Hypothetical protein LOC728392 /// NLR family, pyrin domain containing 1 | 4.69E-06 | 7.04152 |
| 1570183_at | FBXO34 | F-box protein 34 | 8.47E-06 | 7.0395 |
| 224667_x_at | ANAPC16 | anaphase promoting complex subunit 16 | 7.03E-09 | 7.02953 |
| 241065_x_at | CMAS | Cytidine monophosphate N-acetylneuraminic acid synthetase | 5.87E-06 | 7.02261 |
| 211617_at | ALDOAP2 | aldolase A, fructose-bisphosphate pseudogene 2 | 2.01E-06 | 7.01858 |
| 1557617_at | LOC100189589 | hypothetical LOC100189589 | 1.48E-06 | 7.01844 |
| 207149_at | CDH12 | cadherin 12, type 2 (N-cadherin 2) | 4.48E-06 | 7.01619 |
| 1561221_x_at | LOC728099 | hypothetical protein LOC728099 | 3.16E-06 | 7.00892 |
| 215590_x_at | LOC100128640 | hypothetical LOC100128640 | 1.18E-07 | 6.99765 |
| 1555253_at | COL25A1 | collagen, type XXV, alpha 1 | 5.11E-06 | 6.99735 |
| 207655_s_at | BLNK | B-cell linker | 3.79E-05 | 6.98301 |
| 208273_at | ZNF695 | zinc finger protein 695 | 1.68E-05 | 6.97619 |
| 1559226_x_at | LCE1E | late cornified envelope 1E | 2.10E-05 | 6.95638 |
| 1560712_at | TMPRSS11B | transmembrane protease, serine 11B | 5.00E-07 | 6.95462 |
| 233261_at | EBF1 | Early B-cell factor 1 | 4.29E-07 | 6.95251 |
| 1567247_at | OR5H1 | olfactory receptor, family 5, subfamily H, member 1 | 2.71E-05 | 6.94823 |
| 1560684_x_at | BCL8 | B-cell CLL/lymphoma 8 | 2.01E-06 | 6.94799 |
| 1562736_at | LHX9 | LIM homeobox 9 | 6.70E-05 | 6.94415 |
| 220335_x_at | CES3 | carboxylesterase 3 | 7.58E-07 | 6.94413 |
| 1555890_at | OR2A20P /// OR2A9P | olfactory receptor, family 2, subfamily A, member 20 pseudogene /// olfactory re | 3.47E-05 | 6.93986 |
| 213891_s_at | TCF4 | transcription factor 4 | 1.29E-08 | 6.9243 |
| 244455_at | KCNT2 | potassium channel, subfamily T, member 2 | 1.49E-05 | 6.9235 |
| 233631_x_at | KRTAP9-2 /// KRTAP9-8 | keratin associated protein 9-2 /// keratin associated protein 9-8 | 5.01E-06 | 6.91066 |
| 215179_x_at | PGF | Placental growth factor | 1.91E-07 | 6.90942 |
| 206551_x_at | KLHL24 | kelch-like 24 (Drosophila) | 6.26E-06 | 6.90616 |
| 240086_at | VPS36 | vacuolar protein sorting 36 homolog (S. cerevisiae) | 5.21E-06 | 6.90301 |
| 234103_at | KCNT2 | Potassium channel, subfamily T, member 2 | 1.32E-05 | 6.8938 |
| 233056_x_at | DLGAP4 | discs, large (Drosophila) homolog-associated protein 4 | 8.84E-07 | 6.89272 |
| 1554339_a_at | COG3 | component of oligomeric golgi complex 3 | 6.80E-06 | 6.88601 |
| 242377_x_at | THUMPD3 | THUMP domain containing 3 | 1.64E-07 | 6.877 |
| 232143_at | DNM1P41 | DNM1 pseudogene 41 | 7.05E-08 | 6.86739 |
| 239691_at | C12orf77 | chromosome 12 open reading frame 77 | 6.01E-06 | 6.86448 |
| 231898_x_at | SOX2OT | SOX2 overlapping transcript (non-protein coding) | 3.13E-05 | 6.86067 |
| 215582_x_at | MCM3AP | minichromosome maintenance complex component 3 associated protein | 3.66E-06 | 6.8602 |
| 1553335_x_at | LOC285696 | hypothetical LOC285696 | 1.44E-05 | 6.85878 |
| 244795_at | UQCC | ubiquinol-cytochrome c reductase complex chaperone | 1.77E-07 | 6.85557 |
| 1556126_s_at | GPATCH2 | G patch domain containing 2 | 3.17E-06 | 6.84172 |
| 1559950_at | FAM66C /// FAM66D | family with sequence similarity 66, member C /// family with sequence similarity | 7.00E-06 | 6.7803 |
| 1562257_x_at | LOC728392 /// NLRP1 | Hypothetical protein LOC728392 /// NLR family, pyrin domain containing 1 | 2.98E-06 | 6.76539 |
| 239104_at | MGC42157 | hypothetical locus MGC42157 | 1.87E-05 | 6.75973 |
| 1569557_at | ZNF248 | zinc finger protein 248 | 2.99E-07 | 6.74619 |
| 225987_at | STEAP4 | STEAP family member 4 | 9.75E-06 | 6.73975 |
| 210721_s_at | PAK7 | p21 protein (Cdc42/Rac)-activated kinase 7 | 3.62E-06 | 6.72887 |
| 214715_x_at | ZNF160 | zinc finger protein 160 | 1.41E-05 | 6.72218 |
| 238893_at | LOC338758 | hypothetical LOC338758 | 6.34E-07 | 6.72089 |
| 244427_at | KIF23 | Kinesin family member 23 | 1.57E-05 | 6.71075 |
| 1557682_a_at | LOC284688 | hypothetical LOC284688 | 0.000356 | 6.70908 |
| 1556704_s_at | LOC100133920 /// LOC286297 | hypothetical protein LOC100133920 /// hypothetical protein LOC286297 | 1.45E-05 | 6.70879 |
| 220351_at | CCRL1 | chemokine (C-C motif) receptor-like 1 | 7.18E-05 | 6.70704 |
| 215404_x_at | FGFR1 | fibroblast growth factor receptor 1 | 8.71E-06 | 6.69954 |
| 219290_x_at | DAPP1 | dual adaptor of phosphotyrosine and 3-phosphoinositides | 8.98E-07 | 6.69868 |
| 1565306_a_at | SLC8A1 | solute carrier family 8 (sodium/calcium exchanger), member 1 | 3.53E-06 | 6.69416 |
| 1562981_at | HBB | Hemoglobin, beta | 1.61E-05 | 6.69149 |
| 224148_at | FYB | FYN binding protein | 4.07E-06 | 6.67771 |
| 1552865_a_at | IFLTD1 | intermediate filament tail domain containing 1 | 5.07E-06 | 6.67677 |
| 234472_at | GALNT13 | UDP-N-acetyl-alpha-D-galactosamine:polypeptide N-acetylgalactosaminyltransferase | 1.24E-06 | 6.67198 |
| 213307_at | SHANK2 | SH3 and multiple ankyrin repeat domains 2 | 1.74E-05 | 6.65216 |
| 210832_x_at | PTGER3 | prostaglandin E receptor 3 (subtype EP3) | 3.09E-07 | 6.64414 |
| 212327_at | LIMCH1 | LIM and calponin homology domains 1 | 7.92E-06 | 6.6374 |
| 208448_x_at | IFNA16 | interferon, alpha 16 | 5.65E-07 | 6.63102 |
| 227265_at | FGL2 | fibrinogen-like 2 | 4.61E-05 | 6.63062 |
| 226344_at | ZMAT1 | zinc finger, matrin type 1 | 6.78E-05 | 6.62267 |
| 210254_at | MS4A3 | membrane-spanning 4-domains, subfamily A, member 3 (hematopoietic cell-specific) | 3.89E-08 | 6.6107 |
| 233123_at | SLC40A1 | Solute carrier family 40 (iron-regulated transporter), member 1 | 1.54E-05 | 6.61022 |
| 205370_x_at | DBT | dihydrolipoamide branched chain transacylase E2 | 3.47E-06 | 6.60599 |
| 1553155_x_at | ATP6V0D2 | ATPase, H+ transporting, lysosomal 38kDa, V0 subunit d2 | 1.45E-05 | 6.60483 |
| 1552674_at | DIRC1 | disrupted in renal carcinoma 1 | 2.30E-06 | 6.60062 |
| 215978_x_at | ZNF721 | zinc finger protein 721 | 4.67E-08 | 6.59208 |
| 206286_s_at | TDGF1 /// TDGF3 | teratocarcinoma-derived growth factor 1 /// teratocarcinoma-derived growth facto | 3.87E-06 | 6.56969 |
| 243631_at | MPHOSPH8 | M-phase phosphoprotein 8 | 0.000165 | 6.56497 |
| 238850_at | LOC645323 | hypothetical LOC645323 | 4.92E-06 | 6.55736 |
| 208262_x_at | MEFV | Mediterranean fever | 9.48E-06 | 6.55512 |
| 1564093_at | NEK1 | NIMA (never in mitosis gene a)-related kinase 1 | 9.14E-06 | 6.55489 |
| 236367_at | SMG7 | Smg-7 homolog, nonsense mediated mRNA decay factor (C. elegans) | 2.15E-08 | 6.5529 |
| 1554517_x_at | LOC100288109 /// LOC203274 | Hypothetical protein LOC100288109 /// Hypothetical protein LOC203274 | 1.74E-05 | 6.545 |
| 207770_x_at | CSH2 | chorionic somatomammotropin hormone 2 | 1.40E-05 | 6.54189 |
| 233164_x_at | RHBDD1 | rhomboid domain containing 1 | 5.29E-06 | 6.53371 |
| 220352_x_at | FLJ42627 | hypothetical LOC645644 | 6.46E-05 | 6.53106 |
| 1559640_at | ANKFN1 | Ankyrin-repeat and fibronectin type III domain containing 1 | 4.67E-05 | 6.53009 |
| 241998_at | C2orf80 | chromosome 2 open reading frame 80 | 6.07E-05 | 6.51913 |
| 1556929_at | PAPOLG | Poly(A) polymerase gamma | 5.84E-06 | 6.51885 |
| 216509_x_at | MLLT10 | myeloid/lymphoid or mixed-lineage leukemia (trithorax homolog, Drosophila); tran | 2.33E-05 | 6.51454 |
| 207870_at | AKAP9 | A kinase (PRKA) anchor protein (yotiao) 9 | 2.56E-05 | 6.49302 |
| 240735_at | CDC42BPA | CDC42 binding protein kinase alpha (DMPK-like) | 4.13E-06 | 6.49039 |
| 217003_s_at | ADAM5P | ADAM metallopeptidase domain 5, pseudogene | 2.68E-07 | 6.48938 |
| 1566889_at | THADA | Thyroid adenoma associated | 2.58E-05 | 6.48932 |
| 233504_at | C9orf84 | chromosome 9 open reading frame 84 | 6.80E-06 | 6.48085 |
| 1554252_a_at | LASS3 | LAG1 homolog, ceramide synthase 3 | 5.91E-06 | 6.48078 |
| 1557548_at | C10orf108 | chromosome 10 open reading frame 108 | 2.25E-06 | 6.47942 |
| 1563456_at | CCDC141 /// LOC285026 | coiled-coil domain containing 141 /// hypothetical protein LOC285026 | 1.58E-05 | 6.46966 |
| 242711_x_at | FANCM | Fanconi anemia, complementation group M | 1.92E-07 | 6.46867 |
| 230565_at | ATP6V1G3 | ATPase, H+ transporting, lysosomal 13kDa, V1 subunit G3 | 4.86E-06 | 6.46646 |
| 220660_at | C9orf27 | chromosome 9 open reading frame 27 | 2.01E-05 | 6.45065 |
| 215600_x_at | FBXW12 | F-box and WD repeat domain containing 12 | 1.28E-06 | 6.44763 |
| 1566108_at | MYNN | Myoneurin | 3.06E-05 | 6.44736 |
| 229376_at | PROX1 | prospero homeobox 1 | 1.05E-05 | 6.4408 |
| 220575_at | FAM106A | family with sequence similarity 106, member A | 1.66E-05 | 6.4393 |
| 215303_at | DCLK1 | doublecortin-like kinase 1 | 8.79E-06 | 6.42904 |
| 1570007_at | LRRC8C | leucine rich repeat containing 8 family, member C | 7.75E-07 | 6.4253 |
| 208261_x_at | IFNA10 | interferon, alpha 10 | 1.13E-06 | 6.4208 |
| 1568891_x_at | FANCD2 | Fanconi anemia, complementation group D2 | 1.07E-06 | 6.41854 |
| 221104_s_at | NIPSNAP3B | nipsnap homolog 3B (C. elegans) | 3.05E-05 | 6.41561 |
| 208119_s_at | ZNF93 | zinc finger protein 93 | 3.68E-06 | 6.40993 |
| 1562792_at | NIPAL1 | NIPA-like domain containing 1 | 3.15E-06 | 6.40695 |
| 1554996_at | LOC643955 /// ZNF479 | zinc finger protein 479 pseudogene /// zinc finger protein 479 | 1.98E-06 | 6.40186 |
| 1561894_at | LOC653739 | hypothetical protein LOC653739 | 5.24E-06 | 6.40044 |
| 1570238_at | ZNF527 | zinc finger protein 527 | 1.94E-05 | 6.39984 |
| 211349_at | SLC15A1 | solute carrier family 15 (oligopeptide transporter), member 1 | 2.17E-06 | 6.39729 |
| 221329_at | OR52A1 | olfactory receptor, family 52, subfamily A, member 1 | 4.35E-07 | 6.39615 |
| 217418_x_at | MS4A1 | membrane-spanning 4-domains, subfamily A, member 1 | 5.94E-06 | 6.3935 |
| 215321_at | RUNDC3B | RUN domain containing 3B | 0.000114 | 6.38215 |
| 239317_at | CEACAM21 | carcinoembryonic antigen-related cell adhesion molecule 21 | 3.31E-05 | 6.3765 |
| 222347_at | LOC644450 | hypothetical protein LOC644450 | 6.83E-06 | 6.37558 |
| 204580_at | MMP12 | matrix metallopeptidase 12 (macrophage elastase) | 1.50E-05 | 6.37308 |
| 243836_at | UNC80 | unc-80 homolog (C. elegans) | 7.37E-05 | 6.37152 |
| 211502_s_at | CDK14 | cyclin-dependent kinase 14 | 1.11E-05 | 6.35965 |
| 232577_at | LOC145945 | hypothetical protein LOC145945 | 1.34E-05 | 6.35729 |
| 205991_s_at | PRRX1 | paired related homeobox 1 | 1.41E-05 | 6.35495 |
| 206145_at | RHAG | Rh-associated glycoprotein | 1.48E-06 | 6.35261 |
| 210432_s_at | SCN3A | sodium channel, voltage-gated, type III, alpha subunit | 2.00E-05 | 6.35118 |
| 233868_x_at | ADAM33 | ADAM metallopeptidase domain 33 | 7.10E-09 | 6.34918 |
| 214607_at | PAK3 | p21 protein (Cdc42/Rac)-activated kinase 3 | 5.61E-06 | 6.33606 |
| 209242_at | PEG3 | paternally expressed 3 | 1.61E-06 | 6.33471 |
| 208137_x_at | ZNF611 | zinc finger protein 611 | 4.54E-06 | 6.32545 |
| 1554362_at | BTG4 | B-cell translocation gene 4 | 7.56E-06 | 6.32539 |
| 216045_at | CCDC144A | coiled-coil domain containing 144A | 1.11E-05 | 6.32269 |
| 1567023_at | OR5AK4P | olfactory receptor, family 5, subfamily AK, member 4 pseudogene | 6.10E-08 | 6.31575 |
| 1568286_at | HMGA2 | High mobility group AT-hook 2 | 8.02E-06 | 6.3044 |
| 1562939_at | LRRC16A | leucine rich repeat containing 16A | 1.75E-06 | 6.30196 |
| 243761_at | CDK14 | cyclin-dependent kinase 14 | 1.87E-06 | 6.29837 |
| 225299_at | MYO5B | myosin VB | 1.22E-05 | 6.28707 |
| 1559884_at | CDKN2BAS | CDKN2B antisense RNA (non-protein coding) | 1.41E-06 | 6.272 |
| 1559650_at | LOC100128081 | Hypothetical LOC100128081 | 2.66E-05 | 6.26833 |
| 221705_s_at | SIKE1 | suppressor of IKBKE 1 | 6.61E-05 | 6.26759 |
| 1559142_at | MYST3 | MYST histone acetyltransferase (monocytic leukemia) 3 | 4.43E-06 | 6.26544 |
| 230836_at | ST8SIA4 | ST8 alpha-N-acetyl-neuraminide alpha-2,8-sialyltransferase 4 | 2.36E-05 | 6.26292 |
| 211627_x_at | ESR1 | estrogen receptor 1 | 1.45E-05 | 6.24352 |
| 231303_at | NCRNA00158 | non-protein coding RNA 158 | 3.28E-06 | 6.23914 |
| 210135_s_at | SHOX2 | short stature homeobox 2 | 2.11E-06 | 6.23806 |
| 1559369_at | C5orf44 | chromosome 5 open reading frame 44 | 8.04E-07 | 6.23203 |
| 228462_at | IRX2 | iroquois homeobox 2 | 4.11E-06 | 6.22037 |
| 207227_x_at | RFPL2 | ret finger protein-like 2 | 5.62E-06 | 6.21933 |
| 244766_at | LOC100271836 /// LOC100288704 /// LOC440354 /// LOC595101 /// LOC641298 /// SMG1 | SMG1 homolog, phosphatidylinositol 3-kinase-related kinase pseudogene /// simila | 1.32E-05 | 6.21244 |
| 215573_at | CAT | Catalase | 2.15E-05 | 6.20781 |
| 1552497_a_at | SLAMF6 | SLAM family member 6 | 6.87E-08 | 6.20612 |
| 219651_at | DPPA4 | developmental pluripotency associated 4 | 7.33E-07 | 6.20593 |
| 1553397_at | CCDC13 | coiled-coil domain containing 13 | 2.27E-05 | 6.20527 |
| 222252_x_at | UBQLN4 | ubiquilin 4 | 1.10E-06 | 6.20233 |
| 239899_at | RNF145 | Ring finger protein 145 | 3.52E-06 | 6.19753 |
| 235956_at | KIAA1377 | KIAA1377 | 2.06E-06 | 6.18542 |
| 214407_x_at | GYPB | glycophorin B (MNS blood group) | 5.05E-06 | 6.16662 |
| 206765_at | KCNJ2 | potassium inwardly-rectifying channel, subfamily J, member 2 | 2.48E-05 | 6.16252 |
| 243161_x_at | ZFP42 | zinc finger protein 42 homolog (mouse) | 6.57E-06 | 6.16134 |
| 205825_at | PCSK1 | proprotein convertase subtilisin/kexin type 1 | 0.000145 | 6.15012 |
| 205929_at | GPA33 | glycoprotein A33 (transmembrane) | 4.70E-06 | 6.14718 |
| 206373_at | ZIC1 | Zic family member 1 (odd-paired homolog, Drosophila) | 7.95E-07 | 6.13811 |
| 211713_x_at | KIAA0101 | KIAA0101 | 1.06E-06 | 6.13729 |
| 1570009_at | LOC732096 | similar to hCG2040240 | 2.41E-05 | 6.12516 |
| 1562249_at | LOC285965 | hypothetical protein LOC285965 | 0.000529 | 6.12167 |
| 208123_at | KCNB2 | potassium voltage-gated channel, Shab-related subfamily, member 2 | 1.03E-06 | 6.11803 |
| 205889_s_at | JAKMIP2 | janus kinase and microtubule interacting protein 2 | 3.24E-05 | 6.11369 |
| 208182_x_at | IFNA14 | interferon, alpha 14 | 3.82E-07 | 6.11215 |
| 1568924_a_at | IQUB | IQ motif and ubiquitin domain containing | 0.000108 | 6.1106 |
| 1564200_at | LOC646324 | hypothetical LOC646324 | 0.000133 | 6.10595 |
| 219738_s_at | PCDH9 | protocadherin 9 | 2.60E-06 | 6.08157 |
| 220722_s_at | SLC5A7 | solute carrier family 5 (choline transporter), member 7 | 9.72E-05 | 6.07248 |
| 1568612_at | GABRG2 | gamma-aminobutyric acid (GABA) A receptor, gamma 2 | 7.67E-06 | 6.06722 |
| 1569583_at | EREG | epiregulin | 6.09E-06 | 6.06546 |
| 206089_at | NELL1 | NEL-like 1 (chicken) | 5.48E-06 | 6.06258 |
| 229530_at | GUCY1A3 | guanylate cyclase 1, soluble, alpha 3 | 1.57E-05 | 6.05034 |
| 237784_at | SUB1 | SUB1 homolog (S. cerevisiae) | 6.91E-05 | 6.04747 |
| 1554560_at | PGM5 | phosphoglucomutase 5 | 1.75E-06 | 6.04088 |
| 1569669_at | FOXR2 | forkhead box R2 | 1.24E-05 | 6.03452 |
| 1559167_x_at | MPV17L | MPV17 mitochondrial membrane protein-like | 1.69E-06 | 6.03069 |
| 215478_at | RIMS2 | regulating synaptic membrane exocytosis 2 | 0.000108 | 6.02543 |
| 1559952_x_at | FAM66C /// FAM66D | family with sequence similarity 66, member C /// family with sequence similarity | 8.00E-06 | 6.02368 |
| 201306_s_at | ANP32B | acidic (leucine-rich) nuclear phosphoprotein 32 family, member B | 1.63E-11 | 6.02151 |
| 227241_at | MUC15 | mucin 15, cell surface associated | 1.99E-06 | 6.0174 |
| 243998_at | KRT222 | keratin 222 | 7.20E-07 | 6.0131 |
| 206727_at | C9 | complement component 9 | 5.88E-05 | 6.01126 |
| 210356_x_at | MS4A1 | membrane-spanning 4-domains, subfamily A, member 1 | 1.16E-05 | 6.00538 |
| 219392_x_at | PRR11 | proline rich 11 | 5.09E-07 | 6.00018 |
| 210383_at | SCN1A | sodium channel, voltage-gated, type I, alpha subunit | 1.92E-05 | 5.99262 |
| 231770_x_at | C2orf86 | chromosome 2 open reading frame 86 | 1.73E-05 | 5.98992 |
| 226062_x_at | FAM63A | family with sequence similarity 63, member A | 2.41E-06 | 5.98886 |
| 232169_x_at | NDUFS8 | NADH dehydrogenase (ubiquinone) Fe-S protein 8, 23kDa (NADH-coenzyme Q reductase | 1.76E-05 | 5.97489 |
| 1568970_at | ADAM18 | ADAM metallopeptidase domain 18 | 2.96E-06 | 5.97459 |
| 219574_at | 1-三月 | membrane-associated ring finger (C3HC4) 1 | 2.24E-06 | 5.97175 |
| 241403_at | CLK4 | CDC-like kinase 4 | 4.47E-05 | 5.96779 |
| 220591_s_at | EFHC2 | EF-hand domain (C-terminal) containing 2 | 9.28E-07 | 5.96121 |
| 237719_x_at | RGS7BP | regulator of G-protein signaling 7 binding protein | 3.25E-05 | 5.9457 |
| 1566511_at | FBXO9 | F-box protein 9 | 2.57E-08 | 5.94485 |
| 214529_at | TSHB | thyroid stimulating hormone, beta | 9.78E-06 | 5.94335 |
| 228038_at | SOX2 | SRY (sex determining region Y)-box 2 | 3.90E-06 | 5.94116 |
| 207678_s_at | SOX30 | SRY (sex determining region Y)-box 30 | 1.41E-07 | 5.94006 |
| 228086_at | STK33 | serine/threonine kinase 33 | 1.43E-05 | 5.93731 |
| 210227_at | DLGAP2 | discs, large (Drosophila) homolog-associated protein 2 | 3.75E-06 | 5.93183 |
| 210311_at | FGF5 | fibroblast growth factor 5 | 1.22E-05 | 5.92952 |
| 1562223_at | LOC642426 | hypothetical LOC642426 | 0.001218 | 5.92855 |
| 240317_at | PCDHB4 | protocadherin beta 4 | 3.02E-06 | 5.91959 |
| 1558903_at | LOC284260 | hypothetical gene supported by BC011527; BC021928; BC011527; BC021928 | 1.28E-05 | 5.91582 |
| 219368_at | NAP1L2 | nucleosome assembly protein 1-like 2 | 7.07E-06 | 5.90928 |
| 1559355_at | NXPH2 | neurexophilin 2 | 1.71E-06 | 5.90552 |
| 242961_x_at | DDX58 | DEAD (Asp-Glu-Ala-Asp) box polypeptide 58 | 7.94E-06 | 5.90458 |
| 207437_at | NOVA1 | neuro-oncological ventral antigen 1 | 3.61E-05 | 5.90303 |
| 231671_at | FGA | Fibrinogen alpha chain | 3.70E-06 | 5.89366 |
| 224423_x_at | PMCHL2 | pro-melanin-concentrating hormone-like 2 | 5.96E-05 | 5.89101 |
| 1561222_at | LOC283432 | hypothetical protein LOC283432 | 1.92E-05 | 5.88207 |
| 223529_at | SYT4 | synaptotagmin IV | 2.51E-06 | 5.87751 |
| 214295_at | KIAA0485 | hypothetical LOC57235 | 6.34E-06 | 5.87527 |
| 231992_x_at | LOC493754 | RAB guanine nucleotide exchange factor (GEF) 1 pseudogene | 3.68E-07 | 5.86953 |
| 232645_at | LOC153684 | hypothetical LOC153684 | 8.27E-07 | 5.86024 |
| 1556351_at | HCN1 | hyperpolarization activated cyclic nucleotide-gated potassium channel 1 | 2.33E-05 | 5.85516 |
| 239911_at | ONECUT2 | one cut homeobox 2 | 1.97E-06 | 5.8483 |
| 231252_at | C2orf67 | chromosome 2 open reading frame 67 | 5.77E-05 | 5.84296 |
| 1553316_at | GPR82 | G protein-coupled receptor 82 | 5.30E-06 | 5.84177 |
| 209318_x_at | PLAGL1 | pleiomorphic adenoma gene-like 1 | 2.08E-05 | 5.83947 |
| 214598_at | CLDN8 | claudin 8 | 6.01E-06 | 5.83893 |
| 225283_at | ARRDC4 | arrestin domain containing 4 | 9.30E-08 | 5.83865 |
| 1564052_at | TREML4 | triggering receptor expressed on myeloid cells-like 4 | 3.70E-06 | 5.83709 |
| 230509_at | SNX22 | sorting nexin 22 | 3.88E-06 | 5.83635 |
| 222304_x_at | OR7E47P | olfactory receptor, family 7, subfamily E, member 47 pseudogene | 8.91E-06 | 5.83285 |
| 1569690_at | CCDC36 | coiled-coil domain containing 36 | 1.52E-06 | 5.83103 |
| 1558662_s_at | BANK1 | B-cell scaffold protein with ankyrin repeats 1 | 3.76E-06 | 5.8301 |
| 1557450_s_at | WHAMML2 | WAS protein homolog associated with actin, golgi membranes and microtubules-like | 7.06E-06 | 5.81398 |
| 240728_at | PLCB4 | Phospholipase C, beta 4 | 2.73E-07 | 5.80828 |
| 1568933_at | LOC646627 | phospholipase inhibitor | 1.66E-06 | 5.80654 |
| 233598_at | C20orf187 | chromosome 20 open reading frame 187 | 2.53E-06 | 5.80416 |
| 1559406_at | ANKRD18A | ankyrin repeat domain 18A | 1.67E-05 | 5.79832 |
| 204755_x_at | HLF | hepatic leukemia factor | 2.50E-07 | 5.79262 |
| 220720_x_at | FAM128B | family with sequence similarity 128, member B | 3.87E-07 | 5.78699 |
| 220518_at | ABI3BP | ABI family, member 3 (NESH) binding protein | 2.57E-05 | 5.78467 |
| 1568889_at | FANCD2 | Fanconi anemia, complementation group D2 | 9.60E-06 | 5.78308 |
| 1554797_at | SYT16 | synaptotagmin XVI | 2.10E-06 | 5.7822 |
| 1570207_at | FRRS1 | ferric-chelate reductase 1 | 2.47E-07 | 5.78179 |
| 1557666_s_at | C9orf98 | chromosome 9 open reading frame 98 | 3.22E-06 | 5.77908 |
| 207141_s_at | KCNJ3 | potassium inwardly-rectifying channel, subfamily J, member 3 | 9.51E-07 | 5.77472 |
| 209795_at | CD69 | CD69 molecule | 1.65E-05 | 5.77449 |
| 1554855_at | PARK2 | Parkinson disease (autosomal recessive, juvenile) 2, parkin | 5.55E-06 | 5.77262 |
| 1559276_at | LOC728606 | hypothetical LOC728606 | 6.85E-08 | 5.7695 |
| 1552895_a_at | C21orf99 | cancer-testis SP-1 | 5.17E-07 | 5.7637 |
| 238631_at | ZNF140 | Zinc finger protein 140 | 6.30E-05 | 5.76171 |
| 1562267_s_at | ZNF709 | zinc finger protein 709 | 1.21E-05 | 5.75828 |
| 214156_at | MYRIP | myosin VIIA and Rab interacting protein | 1.10E-05 | 5.75618 |
| 244460_at | TMEM225 | transmembrane protein 225 | 1.63E-05 | 5.75273 |
| 233536_at | ASXL3 | additional sex combs like 3 (Drosophila) | 1.55E-07 | 5.75239 |
| 231523_at | FGF14 | fibroblast growth factor 14 | 5.33E-05 | 5.7488 |
| 1556573_s_at | LOC286178 | hypothetical protein LOC286178 | 6.46E-06 | 5.74657 |
| 223697_x_at | C9orf64 | chromosome 9 open reading frame 64 | 3.50E-06 | 5.73336 |
| 211324_s_at | RGPD5 /// RGPD6 /// RGPD8 | RANBP2-like and GRIP domain containing 5 /// RANBP2-like and GRIP domain contain | 1.02E-05 | 5.73294 |
| 238222_at | GKN2 | gastrokine 2 | 0.000132 | 5.72726 |
| 207382_at | TP63 | tumor protein p63 | 3.06E-06 | 5.72141 |
| 241621_at | SMCHD1 | Structural maintenance of chromosomes flexible hinge domain containing 1 | 8.53E-06 | 5.7183 |
| 1558295_a_at | PPFIA2 | protein tyrosine phosphatase, receptor type, f polypeptide (PTPRF), interacting | 3.30E-06 | 5.7136 |
| 207981_s_at | ESRRG | estrogen-related receptor gamma | 5.13E-06 | 5.71357 |
| 244435_at | FAM196A | family with sequence similarity 196, member A | 0.000106 | 5.71308 |
| 221801_x_at | NEFL | neurofilament, light polypeptide | 4.29E-05 | 5.69915 |
| 1558791_at | LOC286467 | hypothetical LOC286467 | 4.52E-07 | 5.69488 |
| 232215_x_at | PRR11 | proline rich 11 | 1.71E-06 | 5.69209 |
| 216198_at | ATF7IP | activating transcription factor 7 interacting protein | 4.13E-05 | 5.68825 |
| 1560434_x_at | CLTA | clathrin, light chain A | 7.72E-08 | 5.67927 |
| 222639_s_at | PLBD1 | phospholipase B domain containing 1 | 2.78E-07 | 5.67845 |
| 1559471_s_at | D21S2088E | D21S2088E | 7.30E-06 | 5.67798 |
| 238526_at | RAB3IP | RAB3A interacting protein (rabin3) | 3.53E-05 | 5.66951 |
| 1557826_at | LOC338817 | hypothetical protein LOC338817 | 2.93E-06 | 5.6686 |
| 1555801_s_at | ZNF385B | zinc finger protein 385B | 8.59E-05 | 5.6685 |
| 220071_x_at | HAUS2 | HAUS augmin-like complex, subunit 2 | 8.53E-05 | 5.6676 |
| 1570259_at | LIMS1 | LIM and senescent cell antigen-like domains 1 | 2.93E-06 | 5.66517 |
| 213936_x_at | SFTPB | surfactant protein B | 7.01E-06 | 5.6626 |
| 206307_s_at | FOXD1 | forkhead box D1 | 1.74E-05 | 5.66209 |
| 233472_at | TCP11L1 | t-complex 11 (mouse)-like 1 | 2.42E-06 | 5.66011 |
| 1570048_at | DNAJC24 | DnaJ (Hsp40) homolog, subfamily C, member 24 | 1.61E-06 | 5.6579 |
| 241252_at | ESCO2 | establishment of cohesion 1 homolog 2 (S. cerevisiae) | 1.24E-05 | 5.65011 |
| 207392_x_at | UGT2B15 | UDP glucuronosyltransferase 2 family, polypeptide B15 | 1.13E-06 | 5.64568 |
| 233780_at | RIF1 | RAP1 interacting factor homolog (yeast) | 5.26E-06 | 5.6356 |
| 242136_x_at | MGC70870 | C-terminal binding protein 2 pseudogene | 8.23E-07 | 5.63143 |
| 203000_at | STMN2 | stathmin-like 2 | 5.01E-08 | 5.63085 |
| 215133_s_at | FAM153A /// FAM153B | family with sequence similarity 153, member A /// family with sequence similarit | 2.16E-05 | 5.62923 |
| 203698_s_at | FRZB | frizzled-related protein | 2.24E-06 | 5.62789 |
| 1554840_at | LOC280665 | anti-CNG alpha 1 cation channel translation product-like | 2.02E-05 | 5.62755 |
| 1553647_at | CDYL2 | chromodomain protein, Y-like 2 | 3.45E-06 | 5.62332 |
| 229796_at | SIX4 | SIX homeobox 4 | 1.84E-06 | 5.62149 |
| 233311_at | LOC145845 | hypothetical LOC145845 | 1.43E-05 | 5.61892 |
| 232848_at | LOC642345 | hCG1818123 | 1.10E-05 | 5.61552 |
| 239144_at | B3GAT2 | beta-1,3-glucuronyltransferase 2 (glucuronosyltransferase S) | 3.76E-07 | 5.6109 |
| 1558124_at | NUDCD2 | NudC domain containing 2 | 4.99E-05 | 5.60963 |
| 242077_x_at | C6orf150 | chromosome 6 open reading frame 150 | 2.51E-08 | 5.60482 |
| 212382_at | TCF4 | transcription factor 4 | 6.41E-05 | 5.59996 |
| 1558523_at | FAM184A | family with sequence similarity 184, member A | 1.08E-06 | 5.59926 |
| 232244_at | KIAA1161 | KIAA1161 | 2.74E-05 | 5.59837 |
| 232226_at | LRRC4C | leucine rich repeat containing 4C | 1.80E-06 | 5.59324 |
| 215527_at | KHDRBS2 | KH domain containing, RNA binding, signal transduction associated 2 | 2.75E-06 | 5.58922 |
| 229021_at | MCTP2 | multiple C2 domains, transmembrane 2 | 5.12E-05 | 5.58826 |
| 239178_at | FGF9 | fibroblast growth factor 9 (glia-activating factor) | 1.01E-05 | 5.5805 |
| 242608_x_at | FAM161B | Family with sequence similarity 161, member B | 9.85E-07 | 5.5758 |
| 233879_at | LOC374491 | TPTE and PTEN homologous inositol lipid phosphatase pseudogene | 6.96E-07 | 5.57553 |
| 235077_at | MEG3 | maternally expressed 3 (non-protein coding) | 1.61E-05 | 5.56797 |
| 212354_at | SULF1 | sulfatase 1 | 7.85E-05 | 5.5637 |
| 231399_at | RAB3IP | RAB3A interacting protein (rabin3) | 2.39E-05 | 5.56257 |
| 240963_x_at | PLXDC1 | Plexin domain containing 1 | 8.89E-07 | 5.5595 |
| 219059_s_at | LYVE1 | lymphatic vessel endothelial hyaluronan receptor 1 | 0.000943 | 5.55754 |
| 216632_at | NAV3 | Neuron navigator 3 | 7.95E-06 | 5.55594 |
| 231380_at | C8orf34 | chromosome 8 open reading frame 34 | 1.43E-06 | 5.5536 |
| 215383_x_at | SPG21 | spastic paraplegia 21 (autosomal recessive, Mast syndrome) | 5.52E-07 | 5.55042 |
| 215463_at | OR7E24 | olfactory receptor, family 7, subfamily E, member 24 | 1.25E-06 | 5.54305 |
| 206785_s_at | KLRC1 /// KLRC2 | killer cell lectin-like receptor subfamily C, member 1 /// killer cell lectin-li | 1.69E-05 | 5.54232 |
| 209244_s_at | KIF1C | kinesin family member 1C | 2.41E-05 | 5.54194 |
| 233227_at | KIAA1109 | KIAA1109 | 2.35E-06 | 5.53875 |
| 1555246_a_at | SCN1A | sodium channel, voltage-gated, type I, alpha subunit | 6.92E-07 | 5.53795 |
| 215301_at | SYCE1L | synaptonemal complex central element protein 1-like | 9.59E-05 | 5.53236 |
| 217494_s_at | LOC100291860 | hypothetical protein LOC100291860 | 9.69E-06 | 5.52598 |
| 238050_at | ANTXR2 | anthrax toxin receptor 2 | 1.81E-05 | 5.52094 |
| 1564473_at | ESCO2 | Establishment of cohesion 1 homolog 2 (S. cerevisiae) | 4.56E-07 | 5.51723 |
| 216850_at | SNRPN | small nuclear ribonucleoprotein polypeptide N | 1.91E-05 | 5.51698 |
| 1565027_at | OFCC1 | orofacial cleft 1 candidate 1 | 2.98E-07 | 5.51385 |
| 221271_at | IL21 | interleukin 21 | 9.01E-05 | 5.51168 |
| 1562558_at | LOC440704 | hypothetical gene supported by BC042042 | 1.88E-05 | 5.50917 |
| 239116_at | ANKRD10 | Ankyrin repeat domain 10 | 0.00013 | 5.50836 |
| 1554591_at | GDEP | Gene differentially expressed in prostate | 2.55E-05 | 5.50724 |
| 217477_at | PIP5K1B | phosphatidylinositol-4-phosphate 5-kinase, type I, beta | 5.58E-07 | 5.50303 |
| 220456_at | SPTLC3 | serine palmitoyltransferase, long chain base subunit 3 | 1.74E-07 | 5.49683 |
| 1559989_at | TPM4 | Tropomyosin 4 | 2.08E-07 | 5.49656 |
| 1555778_a_at | POSTN | periostin, osteoblast specific factor | 1.04E-05 | 5.49247 |
| 1569675_at | POU2AF1 | POU class 2 associating factor 1 | 1.09E-05 | 5.49096 |
| 1564504_at | ACCN5 | amiloride-sensitive cation channel 5, intestinal | 2.36E-05 | 5.49036 |
| 216965_x_at | SPG20 | spastic paraplegia 20 (Troyer syndrome) | 5.35E-06 | 5.48268 |
| 206104_at | ISL1 | ISL LIM homeobox 1 | 3.41E-05 | 5.48255 |
| 220869_at | UBA6 | ubiquitin-like modifier activating enzyme 6 | 0.000172 | 5.48153 |
| 221398_at | TAS2R8 | taste receptor, type 2, member 8 | 1.46E-07 | 5.47995 |
| 1560224_at | AHCTF1 | AT hook containing transcription factor 1 | 8.71E-06 | 5.47046 |
| 1553885_x_at | ZNF99 | zinc finger protein 99 | 1.21E-05 | 5.46864 |
| 235977_at | LONRF2 | LON peptidase N-terminal domain and ring finger 2 | 8.82E-06 | 5.46721 |
| 1559420_x_at | CACNB2 | calcium channel, voltage-dependent, beta 2 subunit | 1.29E-06 | 5.4641 |
| 212758_s_at | ZEB1 | zinc finger E-box binding homeobox 1 | 4.44E-06 | 5.46333 |
| 230303_at | SYNPR | synaptoporin | 4.08E-07 | 5.4445 |
| 241987_x_at | SNX31 | sorting nexin 31 | 3.57E-05 | 5.44417 |
| 1565620_at | AGAP4 | ArfGAP with GTPase domain, ankyrin repeat and PH domain 4 | 1.91E-06 | 5.44373 |
| 214611_at | GRIK1 | glutamate receptor, ionotropic, kainate 1 | 6.11E-06 | 5.43805 |
| 240979_at | LOC284100 | Hypothetical protein LOC284100 | 8.18E-06 | 5.43547 |
| 209604_s_at | GATA3 | GATA binding protein 3 | 3.48E-05 | 5.43154 |
| 219087_at | ASPN | asporin | 2.35E-05 | 5.431 |
| 1566272_at | RALGAPA1 | Ral GTPase activating protein, alpha subunit 1 (catalytic) | 1.50E-07 | 5.42833 |
| 241031_at | C2CD4A | C2 calcium-dependent domain containing 4A | 0.000128 | 5.42812 |
| 237866_at | PID1 | phosphotyrosine interaction domain containing 1 | 8.66E-06 | 5.42336 |
| 224817_at | SH3PXD2A | SH3 and PX domains 2A | 6.79E-09 | 5.4213 |
| 1568644_at | ZNF208 | zinc finger protein 208 | 3.01E-06 | 5.42092 |
| 1563632_at | LOC220980 | hypothetical protein LOC220980 | 2.78E-05 | 5.41299 |
| 1553472_at | FLJ32955 | hypothetical protein FLJ32955 | 7.31E-05 | 5.41204 |
| 214601_at | TPH1 | tryptophan hydroxylase 1 | 6.52E-05 | 5.41122 |
| 214984_at | LOC100271836 /// LOC440354 /// LOC595101 /// LOC641298 /// SMG1 | SMG1 homolog, phosphatidylinositol 3-kinase-related kinase pseudogene /// PI-3-k | 9.25E-06 | 5.41083 |
| 231001_at | FIBIN | fin bud initiation factor homolog (zebrafish) | 2.58E-05 | 5.39187 |
| 241305_at | KYNU | Kynureninase (L-kynurenine hydrolase) | 3.37E-05 | 5.39023 |
| 207062_at | IAPP | islet amyloid polypeptide | 0.000186 | 5.38383 |
| 1555118_at | ENTPD3 | ectonucleoside triphosphate diphosphohydrolase 3 | 3.10E-05 | 5.38235 |
| 204637_at | CGA | glycoprotein hormones, alpha polypeptide | 1.84E-05 | 5.38087 |
| 229357_at | ADAMTS5 | ADAM metallopeptidase with thrombospondin type 1 motif, 5 | 3.06E-07 | 5.37622 |
| 217292_at | MTMR7 | myotubularin related protein 7 | 1.65E-05 | 5.37296 |
| 1554583_a_at | C12orf72 | chromosome 12 open reading frame 72 | 6.67E-06 | 5.37 |
| 215063_x_at | LRRC40 | leucine rich repeat containing 40 | 6.03E-07 | 5.36157 |
| 1570486_at | SOX6 | SRY (sex determining region Y)-box 6 | 1.17E-07 | 5.36155 |
| 208427_s_at | ELAVL2 | ELAV (embryonic lethal, abnormal vision, Drosophila)-like 2 (Hu antigen B) | 8.38E-06 | 5.35898 |
| 1558820_a_at | C18orf34 | chromosome 18 open reading frame 34 | 2.68E-06 | 5.35757 |
| 228849_at | NTRK3 | neurotrophic tyrosine kinase, receptor, type 3 | 5.64E-06 | 5.35248 |
| 209914_s_at | NRXN1 | neurexin 1 | 4.35E-06 | 5.35162 |
| 1570119_at | PDS5B | PDS5, regulator of cohesion maintenance, homolog B (S. cerevisiae) | 7.04E-06 | 5.34949 |
| 206254_at | EGF | epidermal growth factor | 7.70E-06 | 5.34415 |
| 228915_at | DACH1 | dachshund homolog 1 (Drosophila) | 2.04E-05 | 5.34385 |
| 242093_at | SYTL5 | synaptotagmin-like 5 | 5.89E-08 | 5.34228 |
| 226420_at | MECOM | MDS1 and EVI1 complex locus | 4.71E-07 | 5.33806 |
| 221172_at | C7orf69 | chromosome 7 open reading frame 69 | 1.93E-05 | 5.33713 |
| 213697_at | HIPK3 | homeodomain interacting protein kinase 3 | 5.67E-06 | 5.3284 |
| 236715_x_at | UACA | uveal autoantigen with coiled-coil domains and ankyrin repeats | 1.09E-06 | 5.32682 |
| 205998_x_at | CYP3A4 | cytochrome P450, family 3, subfamily A, polypeptide 4 | 1.60E-06 | 5.3257 |
| 1552922_at | RIMS1 | regulating synaptic membrane exocytosis 1 | 8.15E-07 | 5.32386 |
| 207447_s_at | MGAT4C | mannosyl (alpha-1,3-)-glycoprotein beta-1,4-N-acetylglucosaminyltransferase, iso | 9.99E-06 | 5.32253 |
| 211164_at | EPHA3 | EPH receptor A3 | 3.08E-06 | 5.32115 |
| 1555365_x_at | C11orf58 | chromosome 11 open reading frame 58 | 0.000167 | 5.31939 |
| 210697_at | ZNF257 | zinc finger protein 257 | 9.70E-07 | 5.31314 |
| 1563160_at | F11 | coagulation factor XI | 1.71E-05 | 5.31235 |
| 232500_at | RALGAPA2 | Ral GTPase activating protein, alpha subunit 2 (catalytic) | 4.17E-09 | 5.3073 |
| 231178_at | SPATA4 | spermatogenesis associated 4 | 2.69E-06 | 5.30475 |
| 1553909_x_at | FAM178A | family with sequence similarity 178, member A | 1.66E-06 | 5.30364 |
| 243406_at | TMCO5B | transmembrane and coiled-coil domains 5B | 1.52E-05 | 5.30332 |
| 220872_at | PRO2964 | hypothetical protein PRO2964 | 1.07E-06 | 5.30234 |
| 217626_at | AKR1C1 | aldo-keto reductase family 1, member C1 (dihydrodiol dehydrogenase 1; 20-alpha ( | 3.42E-05 | 5.30227 |
| 220639_at | TM4SF20 | transmembrane 4 L six family member 20 | 0.00017 | 5.29908 |
| 1564591_a_at | TMC1 | transmembrane channel-like 1 | 2.85E-05 | 5.29887 |
| 203868_s_at | VCAM1 | vascular cell adhesion molecule 1 | 8.54E-07 | 5.29623 |
| 1554631_at | ATM | ataxia telangiectasia mutated | 2.40E-05 | 5.29583 |
| 228635_at | PCDH10 | protocadherin 10 | 2.55E-07 | 5.29366 |
| 224284_x_at | FKSG49 | FKSG49 | 5.15E-07 | 5.28794 |
| 242084_at | LOC339316 | hypothetical protein LOC339316 | 3.86E-05 | 5.28632 |
| 242889_x_at | LOC645431 | hypothetical LOC645431 | 2.79E-06 | 5.28178 |
| 242743_at | IL4R | Interleukin 4 receptor | 7.36E-07 | 5.27716 |
| 201884_at | CEACAM5 | carcinoembryonic antigen-related cell adhesion molecule 5 | 0.000891 | 5.27624 |
| 230271_at | ONECUT2 | one cut homeobox 2 | 5.78E-06 | 5.27343 |
| 216310_at | TAOK1 | TAO kinase 1 | 9.80E-07 | 5.27283 |
| 220866_at | ADAMTS6 | ADAM metallopeptidase with thrombospondin type 1 motif, 6 | 5.56E-07 | 5.27081 |
| 223629_at | PCDHB5 | protocadherin beta 5 | 7.72E-05 | 5.27034 |
| 205923_at | RELN | reelin | 3.29E-07 | 5.26857 |
| 1559623_at | C11orf54 | Chromosome 11 open reading frame 54 | 2.17E-05 | 5.26222 |
| 1564362_x_at | ZNF843 | zinc finger protein 843 | 2.31E-06 | 5.26213 |
| 235634_at | PURG | purine-rich element binding protein G | 9.23E-07 | 5.25688 |
| 241752_at | SLC8A1 | solute carrier family 8 (sodium/calcium exchanger), member 1 | 3.70E-06 | 5.25465 |
| 201305_x_at | ANP32B | acidic (leucine-rich) nuclear phosphoprotein 32 family, member B | 2.47E-07 | 5.25179 |
| 233338_at | LOC284861 | hypothetical LOC284861 | 5.47E-05 | 5.25057 |
| 220920_at | ATP10B | ATPase, class V, type 10B | 3.04E-07 | 5.24846 |
| 207166_at | GNGT1 | guanine nucleotide binding protein (G protein), gamma transducing activity polyp | 1.02E-05 | 5.24373 |
| 210393_at | LGR5 | leucine-rich repeat-containing G protein-coupled receptor 5 | 4.06E-06 | 5.24346 |
| 216314_at | CRISP1 | cysteine-rich secretory protein 1 | 4.06E-05 | 5.24248 |
| 1566093_at | ARHGEF12 | Rho guanine nucleotide exchange factor (GEF) 12 | 5.32E-06 | 5.23351 |
| 232734_at | TTC23 | tetratricopeptide repeat domain 23 | 8.22E-06 | 5.23193 |
| 215849_x_at | TTC18 | tetratricopeptide repeat domain 18 | 3.09E-05 | 5.23077 |
| 232170_at | S100A7A | S100 calcium binding protein A7A | 1.97E-05 | 5.23002 |
| 217121_at | TNKS | tankyrase, TRF1-interacting ankyrin-related ADP-ribose polymerase | 9.19E-06 | 5.22521 |
| 238520_at | TRERF1 | transcriptional regulating factor 1 | 1.07E-05 | 5.22504 |
| 1557206_at | C17orf104 | chromosome 17 open reading frame 104 | 2.03E-06 | 5.22351 |
| 208342_x_at | CSH2 | chorionic somatomammotropin hormone 2 | 3.79E-05 | 5.22313 |
| 1554737_at | FBN2 | fibrillin 2 | 7.43E-06 | 5.22251 |
| 202252_at | RAB13 | RAB13, member RAS oncogene family | 1.87E-12 | 5.21398 |
| 233621_s_at | ARHGEF12 | Rho guanine nucleotide exchange factor (GEF) 12 | 5.66E-05 | 5.21332 |
| 1566514_at | CWF19L2 | CWF19-like 2, cell cycle control (S. pombe) | 7.36E-05 | 5.21264 |
| 230190_at | NDFIP2 | Nedd4 family interacting protein 2 | 5.82E-07 | 5.2118 |
| 1557541_at | C9orf122 | chromosome 9 open reading frame 122 | 6.29E-06 | 5.20527 |
| 232196_at | LCA5L | Leber congenital amaurosis 5-like | 5.61E-06 | 5.20211 |
| 224941_at | PAPPA | pregnancy-associated plasma protein A, pappalysin 1 | 4.32E-07 | 5.20024 |
| 227236_at | TSPAN2 | tetraspanin 2 | 1.43E-05 | 5.19757 |
| 1568854_at | C6orf41 | chromosome 6 open reading frame 41 | 2.60E-05 | 5.19359 |
| 232621_at | USP48 | ubiquitin specific peptidase 48 | 4.84E-06 | 5.18683 |
| 222259_s_at | SPO11 | SPO11 meiotic protein covalently bound to DSB homolog (S. cerevisiae) | 1.07E-05 | 5.18228 |
| 239132_at | NOS1 | Nitric oxide synthase 1 (neuronal) | 1.92E-05 | 5.17977 |
| 231448_at | ADAD1 | adenosine deaminase domain containing 1 (testis-specific) | 3.48E-06 | 5.17941 |
| 210368_at | PCDHGA8 | protocadherin gamma subfamily A, 8 | 3.99E-05 | 5.17628 |
| 229609_at | LOC728190 | hypothetical LOC728190 | 8.51E-05 | 5.1759 |
| 220336_s_at | GP6 | glycoprotein VI (platelet) | 0.000576 | 5.17568 |
| 206895_at | PPP1R3A | protein phosphatase 1, regulatory (inhibitor) subunit 3A | 1.41E-05 | 5.17284 |
| 232271_at | HNF4G | hepatocyte nuclear factor 4, gamma | 9.48E-05 | 5.16917 |
| 244662_at | MBD5 | methyl-CpG binding domain protein 5 | 2.48E-05 | 5.1691 |
| 209638_x_at | RGS12 | regulator of G-protein signaling 12 | 1.08E-09 | 5.16411 |
| 243968_x_at | FCRL1 | Fc receptor-like 1 | 3.43E-05 | 5.16232 |
| 1554699_at | L3MBTL4 | l(3)mbt-like 4 (Drosophila) | 7.67E-06 | 5.16183 |
| 41553_at | OSGIN2 | Oxidative stress induced growth inhibitor family member 2 | 3.70E-08 | 5.16139 |
| 1553674_at | LRRIQ3 | leucine-rich repeats and IQ motif containing 3 | 3.80E-06 | 5.15787 |
| 1559078_at | BCL11A | B-cell CLL/lymphoma 11A (zinc finger protein) | 2.16E-05 | 5.15691 |
| 1553711_a_at | C4orf39 | chromosome 4 open reading frame 39 | 1.85E-05 | 5.15641 |
| 212328_at | LIMCH1 | LIM and calponin homology domains 1 | 0.000136 | 5.15581 |
| 1569811_at | LOC729307 | hypothetical protein LOC729307 | 8.32E-06 | 5.15102 |
| 205830_at | CLGN | calmegin | 7.03E-05 | 5.15063 |
| 221371_at | TNFSF18 | tumor necrosis factor (ligand) superfamily, member 18 | 8.79E-06 | 5.14993 |
| 1552863_a_at | CACNG6 | calcium channel, voltage-dependent, gamma subunit 6 | 1.65E-05 | 5.14793 |
| 219724_s_at | KIAA0748 | KIAA0748 | 3.32E-05 | 5.1476 |
| 1560683_at | BCL8 | B-cell CLL/lymphoma 8 | 0.000306 | 5.14692 |
| 204530_s_at | TOX | thymocyte selection-associated high mobility group box | 7.01E-06 | 5.13882 |
| 219954_s_at | GBA3 | glucosidase, beta, acid 3 (cytosolic) | 0.000112 | 5.13767 |
| 202485_s_at | MBD2 | methyl-CpG binding domain protein 2 | 6.19E-07 | 5.13663 |
| 207369_at | BRS3 | bombesin-like receptor 3 | 2.63E-05 | 5.13437 |
| 1559695_a_at | LOC100288745 | hypothetical protein LOC100288745 | 3.12E-06 | 5.12678 |
| 241984_at | FOXN3 | forkhead box N3 | 3.36E-05 | 5.12506 |
| 1559826_a_at | LOC401074 | hypothetical LOC401074 | 8.98E-05 | 5.12482 |
| 203961_at | NEBL | nebulette | 6.09E-06 | 5.12169 |
| 220429_at | NDST3 | N-deacetylase/N-sulfotransferase (heparan glucosaminyl) 3 | 5.15E-06 | 5.11897 |
| 243506_at | LOC642597 | hypothetical protein LOC642597 | 8.46E-06 | 5.11671 |
| 234474_x_at | IL6ST | interleukin 6 signal transducer (gp130, oncostatin M receptor) | 1.16E-06 | 5.11361 |
| 232586_x_at | OR7E126P | olfactory receptor, family 7, subfamily E, member 126 pseudogene | 6.54E-06 | 5.11227 |
| 235465_at | FAM123A | family with sequence similarity 123A | 7.47E-06 | 5.11016 |
| 234698_at | N6AMT1 | N-6 adenine-specific DNA methyltransferase 1 (putative) | 5.79E-05 | 5.09988 |
| 1560823_at | LOC340017 | hypothetical LOC340017 | 0.000698 | 5.09864 |
| 220792_at | PRDM5 | PR domain containing 5 | 2.87E-05 | 5.09787 |
| 220769_s_at | WDR78 | WD repeat domain 78 | 8.26E-06 | 5.09707 |
| 1556046_a_at | LOC157627 | hypothetical LOC157627 | 1.41E-05 | 5.09585 |
| 224213_at | C14orf91 | chromosome 14 open reading frame 91 | 0.000382 | 5.0958 |
| 202935_s_at | SOX9 | SRY (sex determining region Y)-box 9 | 0.000164 | 5.09556 |
| 238661_at | LOC100130155 | hypothetical protein LOC100130155 | 2.41E-06 | 5.0937 |
| 221584_s_at | KCNMA1 | potassium large conductance calcium-activated channel, subfamily M, alpha member | 4.32E-05 | 5.09334 |
| 210375_at | PTGER3 | prostaglandin E receptor 3 (subtype EP3) | 1.36E-05 | 5.09239 |
| 204914_s_at | SOX11 | SRY (sex determining region Y)-box 11 | 8.55E-07 | 5.08738 |
| 216970_at | RGS7 | regulator of G-protein signaling 7 | 2.05E-06 | 5.08007 |
| 209966_x_at | ESRRG | estrogen-related receptor gamma | 9.24E-07 | 5.07951 |
| 234457_at | NCRNA00171 | non-protein coding RNA 171 | 3.30E-05 | 5.07921 |
| 1570140_at | LOC653110 | hypothetical LOC653110 | 3.19E-07 | 5.07741 |
| 1556666_a_at | TTC6 | tetratricopeptide repeat domain 6 | 1.22E-05 | 5.07704 |
| 217552_x_at | CR1 | complement component (3b/4b) receptor 1 (Knops blood group) | 0.0002 | 5.0759 |
| 208356_x_at | CSH1 | chorionic somatomammotropin hormone 1 (placental lactogen) | 4.80E-07 | 5.07401 |
| 217342_x_at | FLJ11292 | hypothetical protein FLJ11292 | 1.23E-05 | 5.07297 |
| 210302_s_at | MAB21L2 | mab-21-like 2 (C. elegans) | 4.55E-06 | 5.07192 |
| 235382_at | AQPEP | laeverin | 0.000131 | 5.07093 |
| 1557493_x_at | LOC100240728 | hypothetical LOC100240728 | 9.20E-05 | 5.07058 |
| 211739_x_at | CSH1 | chorionic somatomammotropin hormone 1 (placental lactogen) | 8.16E-06 | 5.07038 |
| 1565544_at | RNF141 | ring finger protein 141 | 7.14E-07 | 5.07016 |
| 217049_x_at | PCDH11Y | protocadherin 11 Y-linked | 2.02E-06 | 5.06995 |
| 1559291_at | NCRNA00032 | Non-protein coding RNA 32 | 5.18E-05 | 5.06897 |
| 215657_at | SLC26A3 | Solute carrier family 26, member 3 | 3.84E-05 | 5.06857 |
| 207303_at | PDE1C | phosphodiesterase 1C, calmodulin-dependent 70kDa | 1.30E-05 | 5.06853 |
| 231592_at | TSIX | XIST antisense RNA (non-protein coding) | 8.94E-05 | 5.06603 |
| 206007_at | PRG4 | proteoglycan 4 | 6.60E-05 | 5.06457 |
| 208341_x_at | CSH2 | chorionic somatomammotropin hormone 2 | 1.03E-06 | 5.06424 |
| 238533_at | EPHA7 | EPH receptor A7 | 5.16E-05 | 5.06416 |
| 1556648_a_at | C10orf40 | chromosome 10 open reading frame 40 | 3.30E-06 | 5.06101 |
| 1569353_at | CP110 | CP110 protein | 3.71E-05 | 5.05931 |
| 224225_s_at | ETV7 | ets variant 7 | 1.50E-06 | 5.0572 |
| 221241_s_at | BCL2L14 | BCL2-like 14 (apoptosis facilitator) | 1.65E-05 | 5.0568 |
| 1569696_at | LOC402779 | hypothetical LOC402779 | 0.00017 | 5.05635 |
| 231325_at | UNC5D | unc-5 homolog D (C. elegans) | 2.22E-06 | 5.04799 |
| 206475_x_at | CSH1 | chorionic somatomammotropin hormone 1 (placental lactogen) | 1.09E-06 | 5.0473 |
| 1563450_at | DEFB107A /// DEFB107B | defensin, beta 107A /// defensin, beta 107B | 1.02E-05 | 5.04718 |
| 234163_at | UBE3A | Ubiquitin protein ligase E3A | 1.41E-06 | 5.04482 |
| 215508_at | BUB1 | budding uninhibited by benzimidazoles 1 homolog (yeast) | 0.000154 | 5.04367 |
| 1561514_at | LOC400655 | hypothetical gene supported by BC013370; BC034583 | 1.94E-05 | 5.04261 |
| 1554911_at | ABCC11 | ATP-binding cassette, sub-family C (CFTR/MRP), member 11 | 8.22E-06 | 5.04104 |
| 222357_at | ZBTB20 | zinc finger and BTB domain containing 20 | 5.74E-05 | 5.04001 |
| 1558501_at | DNM3 | dynamin 3 | 5.50E-05 | 5.03319 |
| 220790_s_at | MS4A5 | membrane-spanning 4-domains, subfamily A, member 5 | 1.07E-05 | 5.03242 |
| 235342_at | SPOCK3 | sparc/osteonectin, cwcv and kazal-like domains proteoglycan (testican) 3 | 4.89E-06 | 5.03047 |
| 237469_at | TOP2A | Topoisomerase (DNA) II alpha 170kDa | 8.21E-05 | 5.02755 |
| 206642_at | DSG1 | desmoglein 1 | 2.75E-06 | 5.0176 |
| 220061_at | ACSM5 | acyl-CoA synthetase medium-chain family member 5 | 4.73E-06 | 5.01695 |
| 209469_at | GPM6A | glycoprotein M6A | 1.52E-05 | 5.01646 |
| 223823_at | KCNMB2 | potassium large conductance calcium-activated channel, subfamily M, beta member | 0.00059 | 5.01605 |
| 207308_at | SLCO1A2 | solute carrier organic anion transporter family, member 1A2 | 0.000214 | 5.01421 |
| 229090_at | LOC220930 | hypothetical LOC220930 | 0.000361 | 5.01264 |
| 206135_at | ST18 | suppression of tumorigenicity 18 (breast carcinoma) (zinc finger protein) | 1.22E-06 | 5.00865 |
| 233204_at | LOC728153 | similar to FAM133B protein | 1.51E-05 | 5.00763 |
| 1552643_at | ZNF626 | zinc finger protein 626 | 6.80E-05 | 5.00235 |
| 207749_s_at | PPP2R3A | protein phosphatase 2 (formerly 2A), regulatory subunit B'', alpha | 1.53E-06 | -5.00015 |
| 1861_at | BAD | BCL2-associated agonist of cell death | 8.88E-06 | -5.0021 |
| 226676_at | ZNF521 | zinc finger protein 521 | 1.40E-07 | -5.00347 |
| 1564064_a_at | ATP11B | ATPase, class VI, type 11B | 9.17E-06 | -5.00574 |
| 226677_at | ZNF521 | zinc finger protein 521 | 5.81E-06 | -5.0061 |
| 218647_s_at | YRDC | yrdC domain containing (E. coli) | 6.21E-06 | -5.00725 |
| 222540_s_at | RSF1 | remodeling and spacing factor 1 | 0.000119 | -5.00758 |
| 202058_s_at | KPNA1 | karyopherin alpha 1 (importin alpha 5) | 4.01E-05 | -5.00761 |
| 201192_s_at | PITPNA | phosphatidylinositol transfer protein, alpha | 3.48E-08 | -5.00938 |
| 211379_x_at | B3GALNT1 | beta-1,3-N-acetylgalactosaminyltransferase 1 (globoside blood group) | 7.31E-05 | -5.01029 |
| 218422_s_at | RBM26 | RNA binding motif protein 26 | 1.84E-05 | -5.01069 |
| 231896_s_at | DENR | density-regulated protein | 9.67E-08 | -5.01141 |
| 225170_at | WDR5 | WD repeat domain 5 | 1.59E-07 | -5.0124 |
| 219099_at | C12orf5 | chromosome 12 open reading frame 5 | 4.95E-05 | -5.01331 |
| 225378_at | VPS37A | vacuolar protein sorting 37 homolog A (S. cerevisiae) | 6.27E-07 | -5.01392 |
| 225348_at | SFRS13A | splicing factor, arginine/serine-rich 13A | 9.90E-05 | -5.01398 |
| 218520_at | TBK1 | TANK-binding kinase 1 | 0.000174 | -5.01441 |
| 225675_at | C14orf101 | chromosome 14 open reading frame 101 | 2.07E-05 | -5.01619 |
| 227304_at | SMCR8 | Smith-Magenis syndrome chromosome region, candidate 8 | 0.000103 | -5.01648 |
| 203869_at | USP46 | ubiquitin specific peptidase 46 | 3.91E-08 | -5.01652 |
| 239014_at | CCAR1 | Cell division cycle and apoptosis regulator 1 | 4.64E-06 | -5.01801 |
| 223639_s_at | ZNRD1 | zinc ribbon domain containing 1 | 3.93E-06 | -5.01884 |
| 225262_at | FOSL2 | FOS-like antigen 2 | 2.65E-05 | -5.01967 |
| 226951_at | C2orf49 | chromosome 2 open reading frame 49 | 4.24E-08 | -5.01995 |
| 230179_at | LOC285812 | hypothetical protein LOC285812 | 1.82E-05 | -5.02022 |
| 218284_at | SMAD3 | SMAD family member 3 | 8.13E-06 | -5.02102 |
| 213286_at | ZFR | zinc finger RNA binding protein | 0.000617 | -5.02103 |
| 202416_at | DNAJC7 | DnaJ (Hsp40) homolog, subfamily C, member 7 | 1.50E-07 | -5.02236 |
| 212604_at | MRPS31 | mitochondrial ribosomal protein S31 | 3.32E-05 | -5.0225 |
| 212888_at | DICER1 | dicer 1, ribonuclease type III | 2.37E-06 | -5.02377 |
| 208503_s_at | GATAD1 | GATA zinc finger domain containing 1 | 1.11E-05 | -5.02382 |
| 218815_s_at | TMEM51 | transmembrane protein 51 | 1.05E-08 | -5.02568 |
| 223173_at | SPNS1 | spinster homolog 1 (Drosophila) | 1.64E-05 | -5.02591 |
| 208178_x_at | TRIO | triple functional domain (PTPRF interacting) | 4.33E-06 | -5.02594 |
| 204774_at | EVI2A | ecotropic viral integration site 2A | 1.56E-06 | -5.02604 |
| 224720_at | MIB1 | mindbomb homolog 1 (Drosophila) | 1.74E-07 | -5.02668 |
| 218496_at | RNASEH1 | ribonuclease H1 | 2.34E-09 | -5.02803 |
| 226326_at | PCGF5 | polycomb group ring finger 5 | 9.80E-07 | -5.03006 |
| 220770_s_at | C5orf54 | chromosome 5 open reading frame 54 | 6.85E-05 | -5.03051 |
| 221744_at | DCAF7 | DDB1 and CUL4 associated factor 7 | 1.46E-06 | -5.0311 |
| 212683_at | SLC25A44 | solute carrier family 25, member 44 | 7.22E-05 | -5.03139 |
| 223248_at | HSDL1 | hydroxysteroid dehydrogenase like 1 | 7.01E-07 | -5.03143 |
| 212050_at | WIPF2 | WAS/WASL interacting protein family, member 2 | 6.52E-05 | -5.03282 |
| 205880_at | PRKD1 | protein kinase D1 | 4.06E-06 | -5.03514 |
| 226406_at | C18orf25 | chromosome 18 open reading frame 25 | 3.85E-07 | -5.03557 |
| 221677_s_at | DONSON | downstream neighbor of SON | 1.23E-07 | -5.03664 |
| 209760_at | KIAA0922 | KIAA0922 | 9.71E-06 | -5.03689 |
| 204768_s_at | FEN1 | flap structure-specific endonuclease 1 | 5.79E-05 | -5.03702 |
| 203233_at | IL4R | interleukin 4 receptor | 4.15E-06 | -5.0377 |
| 201010_s_at | TXNIP | thioredoxin interacting protein | 4.78E-07 | -5.03868 |
| 223139_s_at | DHX36 | DEAH (Asp-Glu-Ala-His) box polypeptide 36 | 2.91E-06 | -5.0397 |
| 201695_s_at | PNP | purine nucleoside phosphorylase | 5.75E-05 | -5.0397 |
| 200777_s_at | BZW1 | basic leucine zipper and W2 domains 1 | 3.35E-07 | -5.04078 |
| 203097_s_at | RAPGEF2 | Rap guanine nucleotide exchange factor (GEF) 2 | 9.22E-06 | -5.04126 |
| 224665_at | ANAPC16 | anaphase promoting complex subunit 16 | 9.45E-06 | -5.04127 |
| 201223_s_at | RAD23B | RAD23 homolog B (S. cerevisiae) | 0.000113 | -5.04184 |
| 200965_s_at | ABLIM1 | actin binding LIM protein 1 | 4.17E-05 | -5.04312 |
| 212374_at | FEM1B | fem-1 homolog b (C. elegans) | 3.14E-06 | -5.04317 |
| 233970_s_at | TRMT6 | tRNA methyltransferase 6 homolog (S. cerevisiae) | 6.61E-06 | -5.04399 |
| 219151_s_at | RABL2A /// RABL2B | RAB, member of RAS oncogene family-like 2A /// RAB, member of RAS oncogene famil | 1.03E-06 | -5.0443 |
| 228908_s_at | LOC642852 | hypothetical LOC642852 | 5.87E-05 | -5.04661 |
| 223060_at | C14orf119 | chromosome 14 open reading frame 119 | 3.01E-08 | -5.04834 |
| 225707_at | ARL6IP6 | ADP-ribosylation-like factor 6 interacting protein 6 | 3.60E-05 | -5.05088 |
| 218593_at | RBM28 | RNA binding motif protein 28 | 8.34E-08 | -5.05285 |
| 218055_s_at | WDR41 | WD repeat domain 41 | 3.76E-05 | -5.05414 |
| 229346_at | NES | nestin | 0.000121 | -5.05424 |
| 209875_s_at | SPP1 | secreted phosphoprotein 1 | 7.40E-06 | -5.05488 |
| 213900_at | FAM189A2 | family with sequence similarity 189, member A2 | 6.82E-07 | -5.05492 |
| 1554556_a_at | ATP11B | ATPase, class VI, type 11B | 6.02E-07 | -5.05531 |
| 202440_s_at | ST5 | suppression of tumorigenicity 5 | 8.87E-06 | -5.0558 |
| 222528_s_at | SLC25A37 | solute carrier family 25, member 37 | 4.29E-06 | -5.05584 |
| 202049_s_at | ZMYM4 | zinc finger, MYM-type 4 | 8.52E-05 | -5.05666 |
| 213413_at | STON1 | stonin 1 | 6.72E-05 | -5.05686 |
| 212513_s_at | USP33 | ubiquitin specific peptidase 33 | 5.48E-07 | -5.05726 |
| 227204_at | PARD6G | par-6 partitioning defective 6 homolog gamma (C. elegans) | 1.35E-05 | -5.05744 |
| 225538_at | ZCCHC9 | zinc finger, CCHC domain containing 9 | 1.89E-06 | -5.05962 |
| 218534_s_at | AGGF1 | angiogenic factor with G patch and FHA domains 1 | 5.45E-05 | -5.06024 |
| 225404_at | C1orf212 | chromosome 1 open reading frame 212 | 1.09E-07 | -5.06097 |
| 215038_s_at | SETD2 | SET domain containing 2 | 3.03E-08 | -5.06158 |
| 209216_at | WDR45 | WD repeat domain 45 | 5.89E-08 | -5.06162 |
| 225991_at | TMEM41A | transmembrane protein 41A | 2.00E-05 | -5.06203 |
| 229235_at | ZCCHC11 | zinc finger, CCHC domain containing 11 | 1.39E-05 | -5.06269 |
| 218111_s_at | CMAS | cytidine monophosphate N-acetylneuraminic acid synthetase | 7.77E-06 | -5.06391 |
| 218099_at | TEX2 | testis expressed 2 | 5.40E-05 | -5.06423 |
| 225041_at | MPHOSPH8 | M-phase phosphoprotein 8 | 6.07E-07 | -5.06452 |
| 1552485_at | LACTB | lactamase, beta | 0.000386 | -5.06486 |
| 212753_at | PCGF3 | polycomb group ring finger 3 | 1.38E-05 | -5.0658 |
| 222443_s_at | RBM8A | RNA binding motif protein 8A | 1.05E-07 | -5.06624 |
| 212866_at | R3HCC1 | R3H domain and coiled-coil containing 1 | 6.74E-06 | -5.06722 |
| 205081_at | CRIP1 | cysteine-rich protein 1 (intestinal) | 2.46E-06 | -5.0684 |
| 235348_at | ABHD13 | abhydrolase domain containing 13 | 8.63E-05 | -5.06912 |
| 225865_x_at | TH1L | TH1-like (Drosophila) | 9.29E-08 | -5.06917 |
| 1553955_at | KLRAQ1 | KLRAQ motif containing 1 | 4.99E-06 | -5.07004 |
| 204977_at | DDX10 | DEAD (Asp-Glu-Ala-Asp) box polypeptide 10 | 1.39E-06 | -5.07023 |
| 212616_at | CHD9 | chromodomain helicase DNA binding protein 9 | 5.87E-05 | -5.07046 |
| 211954_s_at | IPO5 | importin 5 | 4.16E-08 | -5.07082 |
| 224810_s_at | ANKRD13A | ankyrin repeat domain 13A | 1.52E-05 | -5.07168 |
| 223347_at | MUM1 | melanoma associated antigen (mutated) 1 | 3.82E-05 | -5.07446 |
| 226370_at | KLHL15 | kelch-like 15 (Drosophila) | 1.76E-05 | -5.07484 |
| 201680_x_at | SRRT | serrate RNA effector molecule homolog (Arabidopsis) | 1.18E-05 | -5.07709 |
| 206584_at | LY96 | lymphocyte antigen 96 | 3.06E-07 | -5.07735 |
| 210269_s_at | SFRS17A | splicing factor, arginine/serine-rich 17A | 1.60E-05 | -5.07821 |
| 209055_s_at | CDC5L | CDC5 cell division cycle 5-like (S. pombe) | 5.76E-07 | -5.07965 |
| 203715_at | TBCE | tubulin folding cofactor E | 1.76E-05 | -5.08027 |
| 224577_at | ERGIC1 | endoplasmic reticulum-golgi intermediate compartment (ERGIC) 1 | 2.17E-06 | -5.08104 |
| 212891_s_at | GADD45GIP1 | growth arrest and DNA-damage-inducible, gamma interacting protein 1 | 4.67E-07 | -5.08109 |
| 224186_s_at | RNF123 | ring finger protein 123 | 1.60E-08 | -5.08192 |
| 205661_s_at | FLAD1 | FAD1 flavin adenine dinucleotide synthetase homolog (S. cerevisiae) | 1.45E-06 | -5.08219 |
| 200857_s_at | NCOR1 | nuclear receptor co-repressor 1 | 1.21E-05 | -5.08342 |
| 226251_at | ASXL2 | additional sex combs like 2 (Drosophila) | 6.04E-05 | -5.08343 |
| 222570_at | NCS1 | neuronal calcium sensor 1 | 9.07E-05 | -5.08383 |
| 1555882_at | SPIN3 | spindlin family, member 3 | 6.03E-06 | -5.08683 |
| 205442_at | MFAP3L | microfibrillar-associated protein 3-like | 0.000348 | -5.08703 |
| 223190_s_at | MLL5 | myeloid/lymphoid or mixed-lineage leukemia 5 (trithorax homolog, Drosophila) | 9.03E-06 | -5.08723 |
| 222601_at | UBA6 | ubiquitin-like modifier activating enzyme 6 | 8.83E-06 | -5.08758 |
| 212343_at | YIPF6 | Yip1 domain family, member 6 | 1.60E-05 | -5.08767 |
| 212696_s_at | RNF4 | ring finger protein 4 | 7.33E-07 | -5.08838 |
| 230270_at | PRPF38B | PRP38 pre-mRNA processing factor 38 (yeast) domain containing B | 4.54E-05 | -5.08859 |
| 204247_s_at | CDK5 | cyclin-dependent kinase 5 | 4.10E-07 | -5.08943 |
| 221732_at | CANT1 | calcium activated nucleotidase 1 | 7.81E-06 | -5.08966 |
| 226363_at | ABCC5 | ATP-binding cassette, sub-family C (CFTR/MRP), member 5 | 6.17E-05 | -5.09042 |
| 219130_at | CCDC76 | coiled-coil domain containing 76 | 0.000414 | -5.09145 |
| 222751_at | HERPUD2 | HERPUD family member 2 | 1.34E-05 | -5.09177 |
| 217902_s_at | HERC2 | hect domain and RLD 2 | 1.69E-06 | -5.09279 |
| 213608_s_at | SRRD | SRR1 domain containing | 2.93E-07 | -5.0964 |
| 226235_at | LOC339290 | hypothetical LOC339290 | 4.29E-06 | -5.09691 |
| 201900_s_at | AKR1A1 | aldo-keto reductase family 1, member A1 (aldehyde reductase) | 0.000114 | -5.09715 |
| 218477_at | TMEM14A | transmembrane protein 14A | 4.91E-05 | -5.09765 |
| 208815_x_at | HSPA4 | heat shock 70kDa protein 4 | 1.86E-07 | -5.09786 |
| 228492_at | USP9Y | ubiquitin specific peptidase 9, Y-linked | 2.14E-05 | -5.09937 |
| 225214_at | LOC100129034 | hypothetical protein LOC100129034 | 2.32E-07 | -5.09973 |
| 212318_at | TNPO3 | transportin 3 | 2.90E-08 | -5.10067 |
| 201708_s_at | NIPSNAP1 | nipsnap homolog 1 (C. elegans) | 2.81E-06 | -5.10087 |
| 225580_at | MRPL50 | mitochondrial ribosomal protein L50 | 6.49E-05 | -5.1011 |
| 218909_at | RPS6KC1 | ribosomal protein S6 kinase, 52kDa, polypeptide 1 | 6.70E-07 | -5.10168 |
| 225876_at | NIPAL3 | NIPA-like domain containing 3 | 8.81E-05 | -5.10303 |
| 205717_x_at | PCDHGA1 /// PCDHGA10 /// PCDHGA11 /// PCDHGA12 /// PCDHGA2 /// PCDHGA3 /// PCDHGA4 /// PCDHGA5 /// PCDHGA6 /// PCDHGA7 /// PCDHGA8 /// PCDHGA9 /// PCDHGB1 /// PCDHGB2 /// PCDHGB3 /// PCDHGB4 /// PCDHGB5 /// PCDHGB6 /// PCDHGB7 /// PCDHGC3 /// PCDHGC4 /// PCDHGC5 | protocadherin gamma subfamily A, 1 /// protocadherin gamma subfamily A, 10 /// p | 5.20E-06 | -5.10306 |
| 205131_x_at | CLEC11A | C-type lectin domain family 11, member A | 2.03E-06 | -5.10501 |
| 202406_s_at | TIAL1 | TIA1 cytotoxic granule-associated RNA binding protein-like 1 | 2.05E-08 | -5.1051 |
| 225642_at | KTI12 | KTI12 homolog, chromatin associated (S. cerevisiae) | 4.00E-07 | -5.10638 |
| 217745_s_at | NAA50 | N(alpha)-acetyltransferase 50, NatE catalytic subunit | 1.91E-05 | -5.10661 |
| 231927_at | ATF6 | activating transcription factor 6 | 2.07E-05 | -5.10774 |
| 222435_s_at | UBE2J1 | ubiquitin-conjugating enzyme E2, J1 (UBC6 homolog, yeast) | 4.17E-08 | -5.10776 |
| 224311_s_at | CAB39 | calcium binding protein 39 | 0.000485 | -5.10881 |
| 201172_x_at | ATP6V0E1 | ATPase, H+ transporting, lysosomal 9kDa, V0 subunit e1 | 1.41E-07 | -5.1099 |
| 203720_s_at | ERCC1 | excision repair cross-complementing rodent repair deficiency, complementation gr | 2.65E-05 | -5.11062 |
| 227856_at | C4orf32 | chromosome 4 open reading frame 32 | 0.000187 | -5.11071 |
| 1557053_s_at | UBE2G2 | ubiquitin-conjugating enzyme E2G 2 (UBC7 homolog, yeast) | 3.03E-05 | -5.11089 |
| 228217_s_at | PSMG4 | proteasome (prosome, macropain) assembly chaperone 4 | 4.86E-08 | -5.11217 |
| 212417_at | SCAMP1 | secretory carrier membrane protein 1 | 3.52E-05 | -5.11302 |
| 225104_at | ZNF598 | zinc finger protein 598 | 4.91E-06 | -5.11503 |
| 223234_at | MAD2L2 | MAD2 mitotic arrest deficient-like 2 (yeast) | 1.63E-05 | -5.11536 |
| 227280_s_at | CCNYL1 | cyclin Y-like 1 | 1.50E-05 | -5.11556 |
| 225728_at | SORBS2 | sorbin and SH3 domain containing 2 | 2.53E-06 | -5.11562 |
| 224919_at | MRPS6 | mitochondrial ribosomal protein S6 | 3.88E-08 | -5.11564 |
| 227699_at | C14orf149 | chromosome 14 open reading frame 149 | 6.64E-06 | -5.11582 |
| 222850_s_at | DNAJB14 | DnaJ (Hsp40) homolog, subfamily B, member 14 | 3.42E-05 | -5.11645 |
| 201417_at | SOX4 | SRY (sex determining region Y)-box 4 | 2.47E-07 | -5.11768 |
| 31874_at | GAS2L1 | growth arrest-specific 2 like 1 | 2.02E-05 | -5.11891 |
| 227477_at | ZMYND19 | zinc finger, MYND-type containing 19 | 6.57E-05 | -5.11964 |
| 225651_at | UBE2E2 | ubiquitin-conjugating enzyme E2E 2 (UBC4/5 homolog, yeast) | 0.000192 | -5.12305 |
| 218504_at | FAHD2A | fumarylacetoacetate hydrolase domain containing 2A | 1.21E-05 | -5.12316 |
| 215424_s_at | SNW1 | SNW domain containing 1 | 2.93E-08 | -5.12425 |
| 201207_at | TNFAIP1 | tumor necrosis factor, alpha-induced protein 1 (endothelial) | 1.21E-06 | -5.12478 |
| 223598_at | RAD23B | RAD23 homolog B (S. cerevisiae) | 1.90E-05 | -5.12532 |
| 232183_at | SERAC1 | serine active site containing 1 | 2.47E-05 | -5.12687 |
| 1555630_a_at | RAB34 | RAB34, member RAS oncogene family | 4.49E-06 | -5.1286 |
| 217043_s_at | MFN1 | mitofusin 1 | 1.11E-05 | -5.1289 |
| 211987_at | TOP2B | topoisomerase (DNA) II beta 180kDa | 2.82E-07 | -5.12935 |
| 202165_at | PPP1R2 | protein phosphatase 1, regulatory (inhibitor) subunit 2 | 9.45E-06 | -5.13049 |
| 228005_at | ZXDB | zinc finger, X-linked, duplicated B | 3.62E-05 | -5.13161 |
| 222262_s_at | ETNK1 | ethanolamine kinase 1 | 2.70E-06 | -5.13173 |
| 215749_s_at | GORASP1 | golgi reassembly stacking protein 1, 65kDa | 3.21E-06 | -5.13234 |
| 223434_at | GBP3 | guanylate binding protein 3 | 1.29E-05 | -5.13249 |
| 235158_at | TMEM209 | transmembrane protein 209 | 1.76E-05 | -5.13331 |
| 218340_s_at | UBA6 | ubiquitin-like modifier activating enzyme 6 | 2.04E-05 | -5.13418 |
| 233480_at | TMEM43 | Transmembrane protein 43 | 2.16E-05 | -5.13459 |
| 219341_at | CLN8 | ceroid-lipofuscinosis, neuronal 8 (epilepsy, progressive with mental retardation | 2.09E-05 | -5.13515 |
| 217751_at | GSTK1 | glutathione S-transferase kappa 1 | 2.39E-06 | -5.13884 |
| 218399_s_at | CDCA4 | cell division cycle associated 4 | 1.01E-05 | -5.13991 |
| 230634_x_at | ADAT3 | adenosine deaminase, tRNA-specific 3, TAD3 homolog (S. cerevisiae) | 1.70E-06 | -5.14027 |
| 225786_at | NCRNA00201 | non-protein coding RNA 201 | 3.50E-06 | -5.14186 |
| 224709_s_at | CDC42SE2 | CDC42 small effector 2 | 1.15E-06 | -5.142 |
| 220607_x_at | TH1L | TH1-like (Drosophila) | 6.87E-09 | -5.14206 |
| 212880_at | WDR7 | WD repeat domain 7 | 7.10E-06 | -5.14317 |
| 201157_s_at | NMT1 | N-myristoyltransferase 1 | 3.61E-09 | -5.14382 |
| 229113_s_at | C1orf86 | chromosome 1 open reading frame 86 | 5.40E-05 | -5.14384 |
| 233878_s_at | XRN2 | 5'-3' exoribonuclease 2 | 1.14E-06 | -5.14414 |
| 229577_at | AGPAT6 | 1-acylglycerol-3-phosphate O-acyltransferase 6 (lysophosphatidic acid acyltransf | 1.87E-06 | -5.14516 |
| 218869_at | MLYCD | malonyl-CoA decarboxylase | 1.93E-08 | -5.14558 |
| 202250_s_at | DCAF8 | DDB1 and CUL4 associated factor 8 | 7.07E-07 | -5.1457 |
| 202555_s_at | MYLK | myosin light chain kinase | 2.96E-06 | -5.1458 |
| 219401_at | XYLT2 | xylosyltransferase II | 2.90E-07 | -5.14608 |
| 204608_at | ASL | argininosuccinate lyase | 1.53E-06 | -5.14648 |
| 224871_at | TPRG1L | tumor protein p63 regulated 1-like | 3.25E-06 | -5.14714 |
| 229720_at | BAG1 | BCL2-associated athanogene | 1.89E-06 | -5.14734 |
| 235338_s_at | SETDB2 | SET domain, bifurcated 2 | 1.76E-06 | -5.14781 |
| 202189_x_at | PTBP1 | polypyrimidine tract binding protein 1 | 1.54E-08 | -5.14796 |
| 204274_at | EBAG9 | estrogen receptor binding site associated, antigen, 9 | 2.72E-05 | -5.14811 |
| 221500_s_at | STX16 | syntaxin 16 | 6.22E-09 | -5.1483 |
| 222204_s_at | RRN3 | RRN3 RNA polymerase I transcription factor homolog (S. cerevisiae) | 2.87E-06 | -5.14834 |
| 218854_at | DSE | dermatan sulfate epimerase | 6.03E-06 | -5.14843 |
| 225247_at | C19orf6 | chromosome 19 open reading frame 6 | 1.58E-05 | -5.14845 |
| 224703_at | DCAF5 | DDB1 and CUL4 associated factor 5 | 5.86E-06 | -5.14853 |
| 213318_s_at | BAT3 | HLA-B associated transcript 3 | 5.39E-05 | -5.14909 |
| 227567_at | LOC651250 | hypothetical LOC651250 | 1.48E-05 | -5.14968 |
| 209788_s_at | ERAP1 | endoplasmic reticulum aminopeptidase 1 | 2.33E-05 | -5.15004 |
| 221515_s_at | LCMT1 | leucine carboxyl methyltransferase 1 | 2.07E-06 | -5.15068 |
| 1555762_s_at | RBM15 | RNA binding motif protein 15 | 1.83E-05 | -5.15075 |
| 227064_at | ANKRD40 | ankyrin repeat domain 40 | 1.03E-09 | -5.15197 |
| 225969_at | ALKBH6 | alkB, alkylation repair homolog 6 (E. coli) | 5.42E-08 | -5.15287 |
| 201021_s_at | DSTN | destrin (actin depolymerizing factor) | 3.69E-06 | -5.15345 |
| 227932_at | ARIH2 | ariadne homolog 2 (Drosophila) | 2.56E-05 | -5.15613 |
| 229427_at | SEMA5A | sema domain, seven thrombospondin repeats (type 1 and type 1-like), transmembran | 2.65E-08 | -5.15632 |
| 228050_at | UTP15 | UTP15, U3 small nucleolar ribonucleoprotein, homolog (S. cerevisiae) | 5.04E-05 | -5.15747 |
| 203253_s_at | PPIP5K2 | diphosphoinositol pentakisphosphate kinase 2 | 1.76E-06 | -5.15907 |
| 213119_at | SLC36A1 | solute carrier family 36 (proton/amino acid symporter), member 1 | 2.09E-06 | -5.16005 |
| 224676_at | TMED4 | transmembrane emp24 protein transport domain containing 4 | 5.11E-07 | -5.1603 |
| 212078_s_at | MLL | myeloid/lymphoid or mixed-lineage leukemia (trithorax homolog, Drosophila) | 1.56E-06 | -5.16086 |
| 226435_at | PAPLN | papilin, proteoglycan-like sulfated glycoprotein | 2.76E-06 | -5.16156 |
| 212194_s_at | TM9SF4 | transmembrane 9 superfamily protein member 4 | 1.41E-05 | -5.163 |
| 202079_s_at | TRAK1 | trafficking protein, kinesin binding 1 | 5.23E-07 | -5.1633 |
| 224862_at | GNAQ | Guanine nucleotide binding protein (G protein), q polypeptide | 0.000145 | -5.16365 |
| 228931_at | COQ4 | coenzyme Q4 homolog (S. cerevisiae) | 2.28E-06 | -5.16397 |
| 217299_s_at | NBN | nibrin | 0.000931 | -5.16475 |
| 215495_s_at | SAMD4A | sterile alpha motif domain containing 4A | 1.21E-06 | -5.16519 |
| 203265_s_at | MAP2K4 | mitogen-activated protein kinase kinase 4 | 1.90E-05 | -5.16632 |
| 212783_at | RBBP6 | retinoblastoma binding protein 6 | 1.16E-06 | -5.1667 |
| 240386_at | TRPM1 | Transient receptor potential cation channel, subfamily M, member 1 | 5.47E-05 | -5.1667 |
| 222979_s_at | SURF4 | surfeit 4 | 0.000564 | -5.16764 |
| 209014_at | MAGED1 | melanoma antigen family D, 1 | 1.44E-08 | -5.1678 |
| 220663_at | IL1RAPL1 | interleukin 1 receptor accessory protein-like 1 | 3.06E-05 | -5.16857 |
| 201629_s_at | ACP1 | acid phosphatase 1, soluble | 7.97E-08 | -5.16883 |
| 218119_at | LOC10431 /// TIMM23 /// TIMM23B | translocase of inner mitochondrial membrane 23 homolog (yeast)-like /// transloc | 1.66E-05 | -5.16907 |
| 203043_at | ZBED1 | zinc finger, BED-type containing 1 | 1.91E-07 | -5.16914 |
| 216593_s_at | LOC100289666 /// PIGC | similar to phosphatidylinositol glycan, class C /// phosphatidylinositol glycan | 3.35E-06 | -5.16928 |
| 213988_s_at | SAT1 | spermidine/spermine N1-acetyltransferase 1 | 2.89E-06 | -5.17053 |
| 38241_at | BTN3A3 | butyrophilin, subfamily 3, member A3 | 4.64E-06 | -5.17129 |
| 202040_s_at | KDM5A | lysine (K)-specific demethylase 5A | 2.98E-06 | -5.17273 |
| 224627_at | GBA2 | glucosidase, beta (bile acid) 2 | 1.43E-06 | -5.17329 |
| 202891_at | NIT1 | nitrilase 1 | 9.09E-07 | -5.17333 |
| 226413_at | LOC400027 | hypothetical protein LOC400027 | 4.63E-06 | -5.17438 |
| 204516_at | ATXN7 | ataxin 7 | 2.14E-05 | -5.1746 |
| 227889_at | LPCAT2 | lysophosphatidylcholine acyltransferase 2 | 9.23E-07 | -5.17464 |
| 226012_at | ANKRD11 | ankyrin repeat domain 11 | 2.20E-05 | -5.17629 |
| 218861_at | RNF25 | ring finger protein 25 | 2.31E-06 | -5.17728 |
| 206569_at | IL24 | interleukin 24 | 2.73E-08 | -5.17776 |
| 213039_at | ARHGEF18 | Rho/Rac guanine nucleotide exchange factor (GEF) 18 | 3.80E-05 | -5.17859 |
| 226547_at | MYST3 | MYST histone acetyltransferase (monocytic leukemia) 3 | 4.74E-08 | -5.17898 |
| 228020_at | PTCD3 | Pentatricopeptide repeat domain 3 | 0.000989 | -5.18246 |
| 206860_s_at | MIOS | missing oocyte, meiosis regulator, homolog (Drosophila) | 1.45E-10 | -5.18314 |
| 223056_s_at | XPO5 | exportin 5 | 4.42E-06 | -5.18336 |
| 227476_at | LPGAT1 | lysophosphatidylglycerol acyltransferase 1 | 9.80E-07 | -5.1834 |
| 203079_s_at | CUL2 | cullin 2 | 1.21E-08 | -5.18347 |
| 226566_at | TRIM11 | tripartite motif-containing 11 | 9.24E-07 | -5.18445 |
| 214447_at | ETS1 | v-ets erythroblastosis virus E26 oncogene homolog 1 (avian) | 0.000131 | -5.18525 |
| 202983_at | HLTF | helicase-like transcription factor | 2.21E-06 | -5.18525 |
| 228652_at | ZNF776 | zinc finger protein 776 | 2.90E-05 | -5.18602 |
| 222728_s_at | TAF1D | TATA box binding protein (TBP)-associated factor, RNA polymerase I, D, 41kDa | 6.34E-08 | -5.1868 |
| 200849_s_at | AHCYL1 | adenosylhomocysteinase-like 1 | 4.39E-06 | -5.18681 |
| 212131_at | LSM14A | LSM14A, SCD6 homolog A (S. cerevisiae) | 2.42E-07 | -5.18717 |
| 217833_at | SYNCRIP | synaptotagmin binding, cytoplasmic RNA interacting protein | 2.58E-08 | -5.18937 |
| 219016_at | FASTKD5 | FAST kinase domains 5 | 1.79E-07 | -5.18955 |
| 225527_at | CEBPG | CCAAT/enhancer binding protein (C/EBP), gamma | 1.57E-05 | -5.19235 |
| 214155_s_at | LARP4 | La ribonucleoprotein domain family, member 4 | 6.17E-05 | -5.19242 |
| 228543_at | PET117 | cytochrome c oxidase assembly factor-like | 6.78E-06 | -5.19445 |
| 212799_at | STX6 | syntaxin 6 | 1.85E-06 | -5.195 |
| 222233_s_at | DCLRE1C | DNA cross-link repair 1C (PSO2 homolog, S. cerevisiae) | 0.000125 | -5.19656 |
| 222551_s_at | C8orf33 | chromosome 8 open reading frame 33 | 1.01E-07 | -5.19674 |
| 216985_s_at | STX3 | syntaxin 3 | 2.38E-05 | -5.1978 |
| 228771_at | ADRBK2 | adrenergic, beta, receptor kinase 2 | 9.03E-07 | -5.2017 |
| 222883_at | C1orf163 | chromosome 1 open reading frame 163 | 3.83E-06 | -5.20182 |
| 202184_s_at | NUP133 | nucleoporin 133kDa | 3.81E-06 | -5.20272 |
| 236562_at | ZNF439 | zinc finger protein 439 | 7.26E-06 | -5.20429 |
| 238480_at | TTC39C | tetratricopeptide repeat domain 39C | 2.10E-07 | -5.20621 |
| 210878_s_at | KDM3B | lysine (K)-specific demethylase 3B | 3.59E-06 | -5.20661 |
| 225944_at | NLN | neurolysin (metallopeptidase M3 family) | 4.57E-05 | -5.20728 |
| 219826_at | ZNF419 | zinc finger protein 419 | 1.72E-05 | -5.20774 |
| 211529_x_at | HLA-G | major histocompatibility complex, class I, G | 6.08E-06 | -5.20833 |
| 204132_s_at | FOXO3 /// FOXO3B | forkhead box O3 /// forkhead box O3B pseudogene | 3.58E-06 | -5.2096 |
| 223991_s_at | GALNT2 /// LOC100132910 | UDP-N-acetyl-alpha-D-galactosamine:polypeptide N-acetylgalactosaminyltransferase | 2.19E-07 | -5.21088 |
| 209608_s_at | ACAT2 | acetyl-CoA acetyltransferase 2 | 1.66E-07 | -5.21147 |
| 225521_at | ANAPC7 | anaphase promoting complex subunit 7 | 2.90E-05 | -5.21171 |
| 218051_s_at | NT5DC2 | 5'-nucleotidase domain containing 2 | 7.03E-07 | -5.21186 |
| 212540_at | CDC34 | cell division cycle 34 homolog (S. cerevisiae) | 1.96E-07 | -5.2122 |
| 224191_x_at | ROPN1 | ropporin, rhophilin associated protein 1 | 1.61E-06 | -5.21378 |
| 203166_at | CFDP1 | craniofacial development protein 1 | 6.77E-07 | -5.21425 |
| 222703_s_at | YRDC | yrdC domain containing (E. coli) | 1.47E-07 | -5.21725 |
| 223420_at | DNAJC14 | DnaJ (Hsp40) homolog, subfamily C, member 14 | 1.06E-06 | -5.2177 |
| 209607_x_at | SULT1A3 /// SULT1A4 | sulfotransferase family, cytosolic, 1A, phenol-preferring, member 3 /// sulfotra | 6.69E-06 | -5.21776 |
| 223242_s_at | MFSD11 | major facilitator superfamily domain containing 11 | 1.26E-05 | -5.22011 |
| 242455_at | POU3F2 | POU class 3 homeobox 2 | 1.21E-06 | -5.22305 |
| 204520_x_at | BRD1 | bromodomain containing 1 | 7.55E-05 | -5.22345 |
| 201545_s_at | PABPN1 | poly(A) binding protein, nuclear 1 | 4.17E-05 | -5.22361 |
| 209166_s_at | MAN2B1 | mannosidase, alpha, class 2B, member 1 | 4.06E-06 | -5.22578 |
| 225802_at | TOP1MT | topoisomerase (DNA) I, mitochondrial | 8.02E-05 | -5.22613 |
| 203195_s_at | NUP98 | nucleoporin 98kDa | 1.18E-07 | -5.22723 |
| 212218_s_at | FASN | fatty acid synthase | 2.05E-05 | -5.22724 |
| 200799_at | HSPA1A | heat shock 70kDa protein 1A | 8.35E-07 | -5.22874 |
| 36554_at | ASMTL | acetylserotonin O-methyltransferase-like | 9.34E-08 | -5.22949 |
| 201832_s_at | USO1 | USO1 vesicle docking protein homolog (yeast) | 2.52E-07 | -5.22961 |
| 213689_x_at | FAM69A | family with sequence similarity 69, member A | 0.000111 | -5.22985 |
| 217996_at | PHLDA1 | pleckstrin homology-like domain, family A, member 1 | 2.17E-07 | -5.23185 |
| 209056_s_at | CDC5L | CDC5 cell division cycle 5-like (S. pombe) | 2.52E-05 | -5.2319 |
| 1554627_a_at | ASCC1 | activating signal cointegrator 1 complex subunit 1 | 2.81E-06 | -5.23259 |
| 212492_s_at | KDM4B | lysine (K)-specific demethylase 4B | 7.79E-07 | -5.23306 |
| 212607_at | AKT3 | v-akt murine thymoma viral oncogene homolog 3 (protein kinase B, gamma) | 2.31E-07 | -5.23457 |
| 223081_at | PHF23 | PHD finger protein 23 | 1.32E-06 | -5.23502 |
| 218269_at | RNASEN | ribonuclease type III, nuclear | 8.98E-09 | -5.23529 |
| 218844_at | ACSF2 | acyl-CoA synthetase family member 2 | 5.17E-08 | -5.23537 |
| 219636_s_at | ARMC9 | armadillo repeat containing 9 | 1.29E-06 | -5.23615 |
| 217457_s_at | RAP1GDS1 | RAP1, GTP-GDP dissociation stimulator 1 | 5.71E-05 | -5.23845 |
| 205583_s_at | ALG13 | asparagine-linked glycosylation 13 homolog (S. cerevisiae) | 1.45E-05 | -5.23848 |
| 242828_at | FIGN | fidgetin | 2.80E-05 | -5.24055 |
| 219833_s_at | EFHC1 | EF-hand domain (C-terminal) containing 1 | 8.29E-07 | -5.24436 |
| 209281_s_at | ATP2B1 | ATPase, Ca++ transporting, plasma membrane 1 | 4.20E-05 | -5.24596 |
| 224318_s_at | KIAA1310 | KIAA1310 | 2.50E-07 | -5.24651 |
| 200945_s_at | SEC31A | SEC31 homolog A (S. cerevisiae) | 8.03E-07 | -5.24694 |
| 217821_s_at | WBP11 | WW domain binding protein 11 | 6.90E-07 | -5.24696 |
| 223332_x_at | RNF126 | ring finger protein 126 | 4.17E-06 | -5.24792 |
| 228256_s_at | EPB41L4A | erythrocyte membrane protein band 4.1 like 4A | 0.000114 | -5.24804 |
| 217973_at | DCXR | dicarbonyl/L-xylulose reductase | 3.46E-05 | -5.24882 |
| 211031_s_at | CLIP2 | CAP-GLY domain containing linker protein 2 | 5.79E-05 | -5.24974 |
| 223528_s_at | LOC731602 /// METT11D1 | similar to methyltransferase 11 domain containing 1 isoform 2 /// methyltransfer | 1.16E-05 | -5.24998 |
| 223879_s_at | OXR1 | oxidation resistance 1 | 6.85E-07 | -5.2514 |
| 212129_at | NIPA2 | non imprinted in Prader-Willi/Angelman syndrome 2 | 1.06E-09 | -5.25285 |
| 230884_s_at | SPG7 | spastic paraplegia 7 (pure and complicated autosomal recessive) | 4.30E-07 | -5.25553 |
| 213901_x_at | RBM9 | RNA binding motif protein 9 | 8.15E-06 | -5.25662 |
| 219565_at | CYP20A1 | cytochrome P450, family 20, subfamily A, polypeptide 1 | 6.33E-05 | -5.25782 |
| 226214_at | GDE1 | glycerophosphodiester phosphodiesterase 1 | 3.99E-09 | -5.25976 |
| 208974_x_at | KPNB1 | karyopherin (importin) beta 1 | 1.24E-07 | -5.26039 |
| 213083_at | SLC35D2 | solute carrier family 35, member D2 | 1.22E-06 | -5.2626 |
| 221190_s_at | C18orf8 | chromosome 18 open reading frame 8 | 6.47E-07 | -5.26466 |
| 204610_s_at | CCDC85B | coiled-coil domain containing 85B | 1.49E-06 | -5.26692 |
| 202866_at | DNAJB12 | DnaJ (Hsp40) homolog, subfamily B, member 12 | 0.000822 | -5.26702 |
| 212641_at | HIVEP2 | human immunodeficiency virus type I enhancer binding protein 2 | 0.000102 | -5.26716 |
| 235232_at | GMEB1 | glucocorticoid modulatory element binding protein 1 | 1.13E-07 | -5.26996 |
| 213793_s_at | HOMER1 | homer homolog 1 (Drosophila) | 4.95E-06 | -5.27015 |
| 225185_at | MRAS | muscle RAS oncogene homolog | 3.55E-07 | -5.27424 |
| 203919_at | TCEA2 | transcription elongation factor A (SII), 2 | 3.24E-07 | -5.27458 |
| 218899_s_at | BAALC | brain and acute leukemia, cytoplasmic | 3.84E-05 | -5.27569 |
| 205310_at | FBXO46 | F-box protein 46 | 1.82E-05 | -5.27749 |
| 226640_at | DAGLB | diacylglycerol lipase, beta | 2.12E-05 | -5.27862 |
| 1555781_at | PQLC2 | PQ loop repeat containing 2 | 1.13E-05 | -5.28065 |
| 214475_x_at | CAPN3 | calpain 3, (p94) | 9.44E-08 | -5.28074 |
| 226184_at | FMNL2 | formin-like 2 | 5.99E-07 | -5.28352 |
| 213460_x_at | NSUN5P2 | NOP2/Sun domain family, member 5 pseudogene 2 | 1.54E-07 | -5.28364 |
| 201230_s_at | ARIH2 | ariadne homolog 2 (Drosophila) | 1.39E-07 | -5.28404 |
| 218573_at | MAGEH1 | melanoma antigen family H, 1 | 1.44E-05 | -5.28454 |
| 202489_s_at | FXYD3 | FXYD domain containing ion transport regulator 3 | 1.56E-06 | -5.28513 |
| 213019_at | RANBP6 | RAN binding protein 6 | 1.64E-05 | -5.28524 |
| 39835_at | SBF1 | SET binding factor 1 | 7.27E-08 | -5.28547 |
| 212359_s_at | KIAA0913 | KIAA0913 | 6.42E-05 | -5.28573 |
| 218101_s_at | NDUFC2 | NADH dehydrogenase (ubiquinone) 1, subcomplex unknown, 2, 14.5kDa | 1.70E-10 | -5.28647 |
| 212184_s_at | TAB2 | TGF-beta activated kinase 1/MAP3K7 binding protein 2 | 5.79E-06 | -5.28744 |
| 235005_at | DIS3L | DIS3 mitotic control homolog (S. cerevisiae)-like | 5.34E-06 | -5.28794 |
| 202645_s_at | MEN1 | multiple endocrine neoplasia I | 9.95E-07 | -5.28843 |
| 233350_s_at | TEX264 | testis expressed 264 | 8.87E-06 | -5.28913 |
| 209308_s_at | BNIP2 | BCL2/adenovirus E1B 19kDa interacting protein 2 | 6.42E-05 | -5.28956 |
| 1553112_s_at | CDK8 | cyclin-dependent kinase 8 | 6.66E-07 | -5.28993 |
| 203944_x_at | BTN2A1 | butyrophilin, subfamily 2, member A1 | 1.01E-05 | -5.29012 |
| 209122_at | PLIN2 | perilipin 2 | 2.56E-06 | -5.29049 |
| 221647_s_at | RIC8A | resistance to inhibitors of cholinesterase 8 homolog A (C. elegans) | 1.18E-09 | -5.29254 |
| 227847_at | EPM2AIP1 | EPM2A (laforin) interacting protein 1 | 4.93E-07 | -5.29286 |
| 218129_s_at | NFYB | nuclear transcription factor Y, beta | 7.65E-05 | -5.29393 |
| 213741_s_at | KPNA1 | karyopherin alpha 1 (importin alpha 5) | 0.000574 | -5.29869 |
| 1555847_a_at | LOC284454 | hypothetical protein LOC284454 | 1.17E-06 | -5.29896 |
| 235689_at | MTFMT | mitochondrial methionyl-tRNA formyltransferase | 8.14E-06 | -5.29936 |
| 221830_at | RAP2A | RAP2A, member of RAS oncogene family | 5.25E-08 | -5.29954 |
| 221679_s_at | ABHD6 | abhydrolase domain containing 6 | 7.37E-06 | -5.29984 |
| 225509_at | SAP30L | SAP30-like | 1.70E-05 | -5.30002 |
| 227903_x_at | C19orf20 | chromosome 19 open reading frame 20 | 3.54E-06 | -5.30006 |
| 223325_at | TXNDC11 | thioredoxin domain containing 11 | 2.92E-06 | -5.30009 |
| 225738_at | RAPGEF1 | Rap guanine nucleotide exchange factor (GEF) 1 | 3.98E-05 | -5.30032 |
| 200864_s_at | RAB11A | RAB11A, member RAS oncogene family | 1.49E-05 | -5.3023 |
| 201132_at | HNRNPH2 | heterogeneous nuclear ribonucleoprotein H2 (H') | 6.89E-06 | -5.3026 |
| 203114_at | SSSCA1 | Sjogren syndrome/scleroderma autoantigen 1 | 4.30E-05 | -5.30423 |
| 203288_at | KIAA0355 | KIAA0355 | 2.08E-06 | -5.30556 |
| 212594_at | PDCD4 | programmed cell death 4 (neoplastic transformation inhibitor) | 1.26E-05 | -5.30579 |
| 222541_at | RSF1 | remodeling and spacing factor 1 | 9.27E-06 | -5.30588 |
| 217599_s_at | MDFIC | MyoD family inhibitor domain containing | 7.28E-05 | -5.30598 |
| 202486_at | AFG3L2 | AFG3 ATPase family gene 3-like 2 (yeast) | 1.02E-08 | -5.30649 |
| 202511_s_at | ATG5 | ATG5 autophagy related 5 homolog (S. cerevisiae) | 9.75E-05 | -5.30725 |
| 208264_s_at | EIF3J | eukaryotic translation initiation factor 3, subunit J | 9.17E-06 | -5.30904 |
| 213152_s_at | SFRS2B | splicing factor, arginine/serine-rich 2B | 1.54E-06 | -5.30967 |
| 202722_s_at | GFPT1 | glutamine--fructose-6-phosphate transaminase 1 | 1.21E-06 | -5.31038 |
| 212930_at | ATP2B1 | ATPase, Ca++ transporting, plasma membrane 1 | 9.20E-06 | -5.31441 |
| 218984_at | PUS7 | pseudouridylate synthase 7 homolog (S. cerevisiae) | 3.14E-07 | -5.31478 |
| 204308_s_at | TECPR2 | tectonin beta-propeller repeat containing 2 | 8.99E-07 | -5.31552 |
| 228002_at | IDI2 | isopentenyl-diphosphate delta isomerase 2 | 4.80E-07 | -5.31633 |
| 227475_at | FOXQ1 | forkhead box Q1 | 3.72E-05 | -5.31693 |
| 227027_at | GFPT1 | glutamine--fructose-6-phosphate transaminase 1 | 2.11E-05 | -5.31722 |
| 212896_at | SKIV2L2 | superkiller viralicidic activity 2-like 2 (S. cerevisiae) | 4.21E-09 | -5.31763 |
| 212514_x_at | DDX3X | DEAD (Asp-Glu-Ala-Asp) box polypeptide 3, X-linked | 1.35E-06 | -5.32145 |
| 228351_at | HEATR1 | HEAT repeat containing 1 | 2.69E-07 | -5.32158 |
| 209579_s_at | MBD4 | methyl-CpG binding domain protein 4 | 2.25E-05 | -5.32172 |
| 207347_at | ERCC6 | excision repair cross-complementing rodent repair deficiency, complementation gr | 6.45E-07 | -5.32271 |
| 229256_at | PGM2L1 | phosphoglucomutase 2-like 1 | 1.09E-07 | -5.32319 |
| 209293_x_at | ID4 | inhibitor of DNA binding 4, dominant negative helix-loop-helix protein | 4.80E-06 | -5.325 |
| 209183_s_at | C10orf10 | chromosome 10 open reading frame 10 | 6.23E-07 | -5.32511 |
| 202349_at | TOR1A | torsin family 1, member A (torsin A) | 1.27E-06 | -5.32594 |
| 204372_s_at | KHSRP | KH-type splicing regulatory protein | 5.95E-06 | -5.32636 |
| 216952_s_at | LMNB2 | lamin B2 | 4.57E-08 | -5.32817 |
| 212159_x_at | AP2A2 | adaptor-related protein complex 2, alpha 2 subunit | 3.48E-06 | -5.32835 |
| 218532_s_at | FAM134B | family with sequence similarity 134, member B | 9.63E-06 | -5.32915 |
| 235324_at | SFRS3 | splicing factor, arginine/serine-rich 3 | 7.87E-06 | -5.32954 |
| 201017_at | EIF1AX | eukaryotic translation initiation factor 1A, X-linked | 1.07E-05 | -5.33422 |
| 203334_at | DHX8 | DEAH (Asp-Glu-Ala-His) box polypeptide 8 | 2.53E-05 | -5.33503 |
| 219457_s_at | RIN3 | Ras and Rab interactor 3 | 5.60E-07 | -5.33784 |
| 1555789_s_at | PHF23 | PHD finger protein 23 | 1.17E-05 | -5.33885 |
| 233487_s_at | LRRC8A | leucine rich repeat containing 8 family, member A | 1.41E-05 | -5.34065 |
| 227962_at | ACOX1 | acyl-CoA oxidase 1, palmitoyl | 9.26E-07 | -5.3414 |
| 227606_s_at | STAMBPL1 | STAM binding protein-like 1 | 1.55E-07 | -5.34208 |
| 225216_at | FAM199X | Family with sequence similarity 199, X-linked | 8.21E-07 | -5.34251 |
| 218897_at | TMEM177 | transmembrane protein 177 | 8.32E-06 | -5.34287 |
| 218538_s_at | MRS2 | MRS2 magnesium homeostasis factor homolog (S. cerevisiae) | 1.25E-05 | -5.34307 |
| 202625_at | LYN | v-yes-1 Yamaguchi sarcoma viral related oncogene homolog | 3.15E-06 | -5.3434 |
| 202104_s_at | SPG7 | spastic paraplegia 7 (pure and complicated autosomal recessive) | 2.05E-06 | -5.34539 |
| 218263_s_at | ZBED5 | zinc finger, BED-type containing 5 | 2.65E-06 | -5.34539 |
| 229638_at | IRX3 | iroquois homeobox 3 | 1.87E-06 | -5.3456 |
| 202962_at | KIF13B | kinesin family member 13B | 1.25E-05 | -5.34815 |
| 225188_at | RAPH1 | Ras association (RalGDS/AF-6) and pleckstrin homology domains 1 | 1.56E-06 | -5.34986 |
| 208803_s_at | SRP72 | signal recognition particle 72kDa | 2.85E-06 | -5.35081 |
| 221499_s_at | STX16 | syntaxin 16 | 5.35E-07 | -5.352 |
| 203597_s_at | WBP4 | WW domain binding protein 4 (formin binding protein 21) | 2.16E-06 | -5.35251 |
| 204565_at | ACOT13 | acyl-CoA thioesterase 13 | 2.78E-06 | -5.35281 |
| 229174_at | C3orf38 | chromosome 3 open reading frame 38 | 7.26E-05 | -5.35429 |
| 213043_s_at | MED24 | mediator complex subunit 24 | 3.74E-05 | -5.35456 |
| 209581_at | PLA2G16 | phospholipase A2, group XVI | 1.83E-06 | -5.35509 |
| 226284_at | ZBTB2 | zinc finger and BTB domain containing 2 | 4.28E-05 | -5.35536 |
| 218460_at | HEATR2 | HEAT repeat containing 2 | 8.51E-09 | -5.35613 |
| 210104_at | MED6 | mediator complex subunit 6 | 2.17E-06 | -5.3563 |
| 201538_s_at | DUSP3 | dual specificity phosphatase 3 | 8.60E-05 | -5.35716 |
| 212396_s_at | KIAA0090 | KIAA0090 | 2.60E-07 | -5.3573 |
| 209161_at | PRPF4 | PRP4 pre-mRNA processing factor 4 homolog (yeast) | 1.44E-06 | -5.35858 |
| 228397_at | TUG1 | taurine upregulated 1 (non-protein coding) | 8.42E-06 | -5.35868 |
| 203356_at | CAPN7 | calpain 7 | 6.75E-06 | -5.35881 |
| 226106_at | RNF141 | ring finger protein 141 | 6.83E-06 | -5.35904 |
| 222785_x_at | C11orf1 | chromosome 11 open reading frame 1 | 4.49E-07 | -5.35945 |
| 223272_s_at | C1orf57 | chromosome 1 open reading frame 57 | 2.98E-06 | -5.35981 |
| 213016_at | BBX | bobby sox homolog (Drosophila) | 9.71E-07 | -5.36128 |
| 214268_s_at | MTMR4 | myotubularin related protein 4 | 1.20E-06 | -5.36232 |
| 219378_at | NAA16 | N(alpha)-acetyltransferase 16, NatA auxiliary subunit | 8.00E-06 | -5.36366 |
| 219477_s_at | THSD1 /// THSD1P1 | thrombospondin, type I, domain containing 1 /// thrombospondin, type I, domain c | 6.83E-05 | -5.36381 |
| 200787_s_at | PEA15 | phosphoprotein enriched in astrocytes 15 | 5.08E-07 | -5.36595 |
| 205851_at | NME6 | non-metastatic cells 6, protein expressed in (nucleoside-diphosphate kinase) | 9.18E-06 | -5.36646 |
| 228614_at | NCRNA00116 | non-protein coding RNA 116 | 3.93E-09 | -5.3666 |
| 214948_s_at | TMF1 | TATA element modulatory factor 1 | 3.18E-07 | -5.36714 |
| 225886_at | DDX5 | DEAD (Asp-Glu-Ala-Asp) box polypeptide 5 | 1.17E-06 | -5.36874 |
| 208985_s_at | EIF3J | eukaryotic translation initiation factor 3, subunit J | 1.04E-07 | -5.36884 |
| 202380_s_at | NKTR | natural killer-tumor recognition sequence | 1.58E-06 | -5.36921 |
| 212393_at | SBF1 | SET binding factor 1 | 2.36E-06 | -5.36927 |
| 203776_at | GPKOW | G patch domain and KOW motifs | 9.03E-07 | -5.36997 |
| 238716_at | FAM85A | family with sequence similarity 85, member A | 0.000149 | -5.37056 |
| 211991_s_at | HLA-DPA1 | major histocompatibility complex, class II, DP alpha 1 | 7.43E-07 | -5.37241 |
| 225870_s_at | TRAPPC5 | trafficking protein particle complex 5 | 5.58E-08 | -5.37362 |
| 201848_s_at | BNIP3 | BCL2/adenovirus E1B 19kDa interacting protein 3 | 8.74E-08 | -5.37504 |
| 217944_at | POMGNT1 | protein O-linked mannose beta1,2-N-acetylglucosaminyltransferase | 8.39E-06 | -5.3752 |
| 230078_at | RAPGEF6 | Rap guanine nucleotide exchange factor (GEF) 6 | 7.90E-06 | -5.37666 |
| 224610_at | SNHG1 | small nucleolar RNA host gene 1 (non-protein coding) | 3.68E-07 | -5.37718 |
| 40560_at | TBX2 | T-box 2 | 1.19E-07 | -5.37723 |
| 205139_s_at | UST | uronyl-2-sulfotransferase | 3.35E-05 | -5.37733 |
| 202246_s_at | CDK4 | cyclin-dependent kinase 4 | 1.39E-09 | -5.37742 |
| 226242_at | C1orf131 | chromosome 1 open reading frame 131 | 1.23E-07 | -5.3776 |
| 222018_at | NACA | nascent polypeptide-associated complex alpha subunit | 4.28E-05 | -5.37826 |
| 221069_s_at | TACO1 | translational activator of mitochondrially encoded cytochrome c oxidase I | 8.59E-06 | -5.3791 |
| 224959_at | SLC26A2 | solute carrier family 26 (sulfate transporter), member 2 | 2.18E-05 | -5.37939 |
| 227900_at | CBLB | Cas-Br-M (murine) ecotropic retroviral transforming sequence b | 0.000134 | -5.38025 |
| 203891_s_at | DAPK3 | death-associated protein kinase 3 | 1.99E-06 | -5.38093 |
| 239106_at | CA5BP | Carbonic anhydrase VB pseudogene | 6.74E-05 | -5.38111 |
| 218178_s_at | CHMP1B | chromatin modifying protein 1B | 1.32E-05 | -5.38197 |
| 214720_x_at | 10-九月 | septin 10 | 9.89E-05 | -5.38254 |
| 1554334_a_at | DNAJA4 | DnaJ (Hsp40) homolog, subfamily A, member 4 | 7.13E-06 | -5.38287 |
| 218152_at | HMG20A | high-mobility group 20A | 4.46E-06 | -5.38543 |
| 202318_s_at | SENP6 | SUMO1/sentrin specific peptidase 6 | 4.27E-05 | -5.38696 |
| 204820_s_at | BTN3A2 /// BTN3A3 | butyrophilin, subfamily 3, member A2 /// butyrophilin, subfamily 3, member A3 | 1.09E-06 | -5.388 |
| 228123_s_at | ABHD12 | abhydrolase domain containing 12 | 9.20E-07 | -5.38977 |
| 215416_s_at | STOML2 | stomatin (EPB72)-like 2 | 2.34E-07 | -5.39282 |
| 217813_s_at | SPIN1 | spindlin 1 | 5.21E-05 | -5.39303 |
| 205202_at | PCMT1 | protein-L-isoaspartate (D-aspartate) O-methyltransferase | 1.99E-07 | -5.3938 |
| 200710_at | ACADVL | acyl-CoA dehydrogenase, very long chain | 4.78E-05 | -5.398 |
| 202405_at | TIAL1 | TIA1 cytotoxic granule-associated RNA binding protein-like 1 | 1.64E-06 | -5.39945 |
| 226112_at | SGCB | sarcoglycan, beta (43kDa dystrophin-associated glycoprotein) | 7.74E-07 | -5.40012 |
| 212893_at | ZZZ3 | zinc finger, ZZ-type containing 3 | 2.46E-05 | -5.40105 |
| 1555736_a_at | AGTRAP | angiotensin II receptor-associated protein | 1.12E-06 | -5.40149 |
| 225440_at | AGPAT3 | 1-acylglycerol-3-phosphate O-acyltransferase 3 | 7.35E-06 | -5.40198 |
| 215446_s_at | LOX | lysyl oxidase | 6.77E-07 | -5.40296 |
| 209239_at | NFKB1 | nuclear factor of kappa light polypeptide gene enhancer in B-cells 1 | 1.65E-07 | -5.40502 |
| 201836_s_at | SUPT7L | suppressor of Ty 7 (S. cerevisiae)-like | 8.24E-06 | -5.40559 |
| 200593_s_at | HNRNPU | heterogeneous nuclear ribonucleoprotein U (scaffold attachment factor A) | 1.24E-05 | -5.40668 |
| 212122_at | RHOQ | ras homolog gene family, member Q | 1.51E-05 | -5.40697 |
| 219031_s_at | NIP7 | nuclear import 7 homolog (S. cerevisiae) | 1.34E-05 | -5.40795 |
| 224920_x_at | MYADM | myeloid-associated differentiation marker | 7.89E-06 | -5.40825 |
| 214285_at | FABP3 | fatty acid binding protein 3, muscle and heart (mammary-derived growth inhibitor | 1.44E-05 | -5.40947 |
| 244741_s_at | MGC9913 | hypothetical protein MGC9913 | 1.78E-05 | -5.41081 |
| 212908_at | DNAJC16 | DnaJ (Hsp40) homolog, subfamily C, member 16 | 2.57E-06 | -5.41308 |
| 232136_s_at | CTTNBP2 | cortactin binding protein 2 | 6.44E-06 | -5.41516 |
| 202855_s_at | SLC16A3 | solute carrier family 16, member 3 (monocarboxylic acid transporter 4) | 1.39E-06 | -5.41554 |
| 226021_at | RDH10 | retinol dehydrogenase 10 (all-trans) | 3.51E-05 | -5.41701 |
| 228201_at | ARL13B | ADP-ribosylation factor-like 13B | 1.04E-06 | -5.41729 |
| 218535_s_at | RIOK2 | RIO kinase 2 (yeast) | 4.24E-05 | -5.41732 |
| 209213_at | CBR1 | carbonyl reductase 1 | 1.34E-07 | -5.41735 |
| 211969_at | HSP90AA1 | heat shock protein 90kDa alpha (cytosolic), class A member 1 | 3.88E-06 | -5.4174 |
| 212476_at | ACAP2 | ArfGAP with coiled-coil, ankyrin repeat and PH domains 2 | 6.85E-06 | -5.41841 |
| 224984_at | NFAT5 | nuclear factor of activated T-cells 5, tonicity-responsive | 5.92E-08 | -5.41882 |
| 213656_s_at | KLC1 | kinesin light chain 1 | 5.19E-07 | -5.41944 |
| 203216_s_at | MYO6 | myosin VI | 1.41E-05 | -5.41962 |
| 202356_s_at | GTF2F1 | general transcription factor IIF, polypeptide 1, 74kDa | 2.01E-05 | -5.41967 |
| 222119_s_at | FBXO11 | F-box protein 11 | 2.64E-06 | -5.41995 |
| 209681_at | SLC19A2 | solute carrier family 19 (thiamine transporter), member 2 | 4.20E-05 | -5.4204 |
| 40562_at | GNA11 | guanine nucleotide binding protein (G protein), alpha 11 (Gq class) | 1.39E-05 | -5.42124 |
| 203047_at | STK10 | serine/threonine kinase 10 | 2.41E-08 | -5.42184 |
| 203006_at | INPP5A | inositol polyphosphate-5-phosphatase, 40kDa | 4.36E-09 | -5.42277 |
| 223187_s_at | ORMDL1 | ORM1-like 1 (S. cerevisiae) | 5.19E-08 | -5.42534 |
| 203978_at | NUBP1 | nucleotide binding protein 1 (MinD homolog, E. coli) | 3.46E-11 | -5.42584 |
| 227111_at | ZBTB34 | zinc finger and BTB domain containing 34 | 1.58E-05 | -5.42594 |
| 209786_at | HMGN4 | high mobility group nucleosomal binding domain 4 | 1.69E-05 | -5.42677 |
| 1552310_at | C15orf40 | chromosome 15 open reading frame 40 | 3.05E-10 | -5.42711 |
| 224605_at | C4orf3 | chromosome 4 open reading frame 3 | 3.16E-05 | -5.42755 |
| 202545_at | PRKCD | protein kinase C, delta | 8.97E-07 | -5.42756 |
| 234405_s_at | PHAX | phosphorylated adaptor for RNA export | 1.38E-05 | -5.42852 |
| 224392_s_at | OPN3 | opsin 3 | 1.69E-05 | -5.42858 |
| 201692_at | SIGMAR1 | sigma non-opioid intracellular receptor 1 | 1.65E-05 | -5.42875 |
| 202370_s_at | CBFB | core-binding factor, beta subunit | 1.35E-09 | -5.42898 |
| 226255_at | ZBTB33 | zinc finger and BTB domain containing 33 | 1.67E-05 | -5.42975 |
| 212196_at | IL6ST | interleukin 6 signal transducer (gp130, oncostatin M receptor) | 1.24E-06 | -5.43008 |
| 1552330_at | CENPBD1 | CENPB DNA-binding domains containing 1 | 4.59E-07 | -5.43024 |
| 201009_s_at | TXNIP | thioredoxin interacting protein | 0.000127 | -5.43034 |
| 208854_s_at | STK24 | serine/threonine kinase 24 (STE20 homolog, yeast) | 3.65E-08 | -5.43106 |
| 1566557_at | FLJ90757 | hypothetical LOC440465 | 0.000124 | -5.4316 |
| 224939_at | NUFIP2 | nuclear fragile X mental retardation protein interacting protein 2 | 5.08E-06 | -5.43212 |
| 201179_s_at | GNAI3 | guanine nucleotide binding protein (G protein), alpha inhibiting activity polype | 9.54E-08 | -5.43232 |
| 213325_at | PVRL3 | poliovirus receptor-related 3 | 8.20E-06 | -5.43299 |
| 219330_at | VANGL1 | vang-like 1 (van gogh, Drosophila) | 3.34E-05 | -5.43375 |
| 209620_s_at | ABCB7 | ATP-binding cassette, sub-family B (MDR/TAP), member 7 | 2.00E-07 | -5.43452 |
| 222454_s_at | PARVA | parvin, alpha | 9.81E-08 | -5.43458 |
| 224721_at | WDR75 | WD repeat domain 75 | 1.31E-05 | -5.43528 |
| 219489_s_at | NXN | nucleoredoxin | 7.63E-08 | -5.43536 |
| 224891_at | FOXO3 | forkhead box O3 | 3.79E-08 | -5.4355 |
| 216941_s_at | TAF1B | TATA box binding protein (TBP)-associated factor, RNA polymerase I, B, 63kDa | 2.36E-06 | -5.43608 |
| 219563_at | C14orf139 | chromosome 14 open reading frame 139 | 4.66E-08 | -5.43622 |
| 239742_at | TULP4 | Tubby like protein 4 | 2.51E-05 | -5.43717 |
| 209463_s_at | TAF12 | TAF12 RNA polymerase II, TATA box binding protein (TBP)-associated factor, 20kDa | 1.37E-06 | -5.43824 |
| 235096_at | LEO1 | Leo1, Paf1/RNA polymerase II complex component, homolog (S. cerevisiae) | 1.82E-05 | -5.439 |
| 1558279_a_at | KDSR | 3-ketodihydrosphingosine reductase | 7.61E-06 | -5.43985 |
| 209605_at | TST | thiosulfate sulfurtransferase (rhodanese) | 2.03E-07 | -5.44039 |
| 203420_at | FAM8A1 | family with sequence similarity 8, member A1 | 1.27E-05 | -5.44157 |
| 208911_s_at | PDHB | pyruvate dehydrogenase (lipoamide) beta | 4.37E-08 | -5.44164 |
| 202234_s_at | SLC16A1 | solute carrier family 16, member 1 (monocarboxylic acid transporter 1) | 9.22E-06 | -5.44205 |
| 208615_s_at | PTP4A2 | protein tyrosine phosphatase type IVA, member 2 | 2.21E-07 | -5.44259 |
| 201450_s_at | TIA1 | TIA1 cytotoxic granule-associated RNA binding protein | 1.59E-05 | -5.44272 |
| 216713_at | KRIT1 | KRIT1, ankyrin repeat containing | 1.15E-05 | -5.44523 |
| 221675_s_at | CHPT1 | choline phosphotransferase 1 | 5.75E-08 | -5.44641 |
| 219641_at | DET1 | de-etiolated homolog 1 (Arabidopsis) | 1.13E-05 | -5.44655 |
| 212629_s_at | PKN2 | protein kinase N2 | 5.03E-06 | -5.44718 |
| 219363_s_at | MTERFD1 | MTERF domain containing 1 | 6.26E-06 | -5.44797 |
| 217791_s_at | ALDH18A1 | aldehyde dehydrogenase 18 family, member A1 | 4.06E-07 | -5.44886 |
| 219022_at | C12orf43 | chromosome 12 open reading frame 43 | 1.21E-05 | -5.45035 |
| 221427_s_at | CCNL2 | cyclin L2 | 5.48E-06 | -5.45181 |
| 210754_s_at | LYN | v-yes-1 Yamaguchi sarcoma viral related oncogene homolog | 3.85E-05 | -5.45203 |
| 209226_s_at | TNPO1 | transportin 1 | 3.13E-09 | -5.45275 |
| 226038_at | LONRF1 | LON peptidase N-terminal domain and ring finger 1 | 1.11E-07 | -5.45385 |
| 201251_at | PKM2 | pyruvate kinase, muscle | 1.11E-06 | -5.45399 |
| 213341_at | FEM1C | fem-1 homolog c (C. elegans) | 6.27E-05 | -5.45436 |
| 212544_at | ZNHIT3 | zinc finger, HIT type 3 | 7.16E-08 | -5.45466 |
| 221261_x_at | MAGED4 /// MAGED4B | melanoma antigen family D, 4 /// melanoma antigen family D, 4B | 3.89E-06 | -5.45529 |
| 222101_s_at | DCHS1 | dachsous 1 (Drosophila) | 9.78E-06 | -5.45537 |
| 226685_at | SNTB2 | syntrophin, beta 2 (dystrophin-associated protein A1, 59kDa, basic component 2) | 1.11E-07 | -5.45684 |
| 220954_s_at | PILRB | paired immunoglobin-like type 2 receptor beta | 3.11E-06 | -5.45803 |
| 225327_at | KIAA1370 | KIAA1370 | 1.95E-06 | -5.4598 |
| 218187_s_at | C8orf33 | chromosome 8 open reading frame 33 | 8.72E-07 | -5.45988 |
| 210645_s_at | TTC3 | tetratricopeptide repeat domain 3 | 4.28E-07 | -5.45996 |
| 213548_s_at | CDV3 | CDV3 homolog (mouse) | 3.31E-06 | -5.4609 |
| 211528_x_at | HLA-G | major histocompatibility complex, class I, G | 1.82E-07 | -5.46127 |
| 205803_s_at | TRPC1 | transient receptor potential cation channel, subfamily C, member 1 | 4.41E-06 | -5.46147 |
| 226744_at | METT10D | methyltransferase 10 domain containing | 2.53E-07 | -5.46365 |
| 221645_s_at | ZNF83 | zinc finger protein 83 | 0.000115 | -5.46592 |
| 218820_at | C14orf132 | chromosome 14 open reading frame 132 | 3.25E-05 | -5.46685 |
| 201786_s_at | ADAR | adenosine deaminase, RNA-specific | 1.34E-07 | -5.46717 |
| 203778_at | MANBA | mannosidase, beta A, lysosomal | 2.42E-06 | -5.4694 |
| 201953_at | CIB1 | calcium and integrin binding 1 (calmyrin) | 1.80E-07 | -5.47142 |
| 210428_s_at | HGS | hepatocyte growth factor-regulated tyrosine kinase substrate | 5.15E-08 | -5.47144 |
| 226995_at | LOC642852 | hypothetical LOC642852 | 2.61E-06 | -5.47185 |
| 201083_s_at | BCLAF1 | BCL2-associated transcription factor 1 | 0.000178 | -5.47196 |
| 227489_at | SMURF2 | SMAD specific E3 ubiquitin protein ligase 2 | 2.35E-06 | -5.47262 |
| 213090_s_at | TAF4 | TAF4 RNA polymerase II, TATA box binding protein (TBP)-associated factor, 135kDa | 4.72E-06 | -5.47318 |
| 228662_at | SOCS7 | Suppressor of cytokine signaling 7 | 2.81E-06 | -5.4733 |
| 221269_s_at | SH3BGRL3 | SH3 domain binding glutamic acid-rich protein like 3 | 3.35E-07 | -5.47366 |
| 230903_s_at | C8orf42 | chromosome 8 open reading frame 42 | 1.07E-06 | -5.47507 |
| 218474_s_at | KCTD5 | potassium channel tetramerisation domain containing 5 | 2.60E-06 | -5.47546 |
| 218750_at | TAF1D | TATA box binding protein (TBP)-associated factor, RNA polymerase I, D, 41kDa | 6.94E-06 | -5.47598 |
| 225236_at | RBM18 | RNA binding motif protein 18 | 1.73E-06 | -5.47624 |
| 226493_at | KCTD18 | potassium channel tetramerisation domain containing 18 | 9.70E-06 | -5.47637 |
| 212871_at | MAPKAPK5 | mitogen-activated protein kinase-activated protein kinase 5 | 2.01E-05 | -5.4767 |
| 219816_s_at | RBM23 | RNA binding motif protein 23 | 3.32E-05 | -5.47892 |
| 223033_s_at | SCYL1 | SCY1-like 1 (S. cerevisiae) | 1.90E-06 | -5.47948 |
| 224680_at | TMED4 | transmembrane emp24 protein transport domain containing 4 | 2.56E-08 | -5.48253 |
| 223099_s_at | LONP2 | lon peptidase 2, peroxisomal | 9.61E-06 | -5.4828 |
| 215493_x_at | BTN2A1 | butyrophilin, subfamily 2, member A1 | 8.18E-06 | -5.4837 |
| 226104_at | RNF170 | ring finger protein 170 | 1.26E-06 | -5.48402 |
| 212268_at | SERPINB1 | serpin peptidase inhibitor, clade B (ovalbumin), member 1 | 5.07E-08 | -5.48424 |
| 209063_x_at | PAIP1 | poly(A) binding protein interacting protein 1 | 5.02E-05 | -5.48478 |
| 204042_at | WASF3 | WAS protein family, member 3 | 1.88E-08 | -5.48502 |
| 212119_at | RHOQ | ras homolog gene family, member Q | 3.66E-06 | -5.48667 |
| 230885_at | SPG7 | spastic paraplegia 7 (pure and complicated autosomal recessive) | 1.33E-07 | -5.48707 |
| 217995_at | SQRDL | sulfide quinone reductase-like (yeast) | 7.67E-08 | -5.4873 |
| 201697_s_at | DNMT1 | DNA (cytosine-5-)-methyltransferase 1 | 6.84E-08 | -5.48788 |
| 203630_s_at | COG5 | component of oligomeric golgi complex 5 | 9.43E-06 | -5.48895 |
| 225392_at | GFM2 | G elongation factor, mitochondrial 2 | 2.45E-05 | -5.48992 |
| 219408_at | PRMT7 | protein arginine methyltransferase 7 | 3.03E-05 | -5.4902 |
| 1564520_s_at | PRMT5 | protein arginine methyltransferase 5 | 9.68E-08 | -5.49386 |
| 222623_s_at | ZNF639 | zinc finger protein 639 | 1.13E-06 | -5.49434 |
| 218529_at | CD320 | CD320 molecule | 1.41E-08 | -5.49477 |
| 201988_s_at | CREBL2 | cAMP responsive element binding protein-like 2 | 1.55E-06 | -5.49482 |
| 225783_at | UBE2F | ubiquitin-conjugating enzyme E2F (putative) | 2.97E-07 | -5.49521 |
| 213189_at | MINA | MYC induced nuclear antigen | 2.82E-07 | -5.49528 |
| 213258_at | TFPI | tissue factor pathway inhibitor (lipoprotein-associated coagulation inhibitor) | 1.32E-05 | -5.4955 |
| 215884_s_at | UBQLN2 | ubiquilin 2 | 3.53E-06 | -5.4958 |
| 209626_s_at | OSBPL3 | oxysterol binding protein-like 3 | 4.21E-06 | -5.49725 |
| 217478_s_at | HLA-DMA | major histocompatibility complex, class II, DM alpha | 4.26E-06 | -5.49844 |
| 200974_at | ACTA2 | actin, alpha 2, smooth muscle, aorta | 1.89E-06 | -5.49922 |
| 215548_s_at | SCFD1 | sec1 family domain containing 1 | 4.81E-06 | -5.49934 |
| 228062_at | NAP1L5 | nucleosome assembly protein 1-like 5 | 6.58E-06 | -5.49958 |
| 201586_s_at | SFPQ | splicing factor proline/glutamine-rich | 6.62E-09 | -5.50017 |
| 564_at | GNA11 | guanine nucleotide binding protein (G protein), alpha 11 (Gq class) | 8.06E-06 | -5.50031 |
| 209268_at | VPS45 | vacuolar protein sorting 45 homolog (S. cerevisiae) | 4.83E-08 | -5.50103 |
| 213225_at | PPM1B | protein phosphatase, Mg2+/Mn2+ dependent, 1B | 0.000131 | -5.5013 |
| 232007_at | AGPAT5 | 1-acylglycerol-3-phosphate O-acyltransferase 5 (lysophosphatidic acid acyltransf | 1.10E-06 | -5.50294 |
| 204131_s_at | FOXO3 | forkhead box O3 | 5.77E-05 | -5.5033 |
| 1555790_a_at | TMEM192 /// ZNF320 | transmembrane protein 192 /// zinc finger protein 320 | 1.65E-06 | -5.50435 |
| 202176_at | ERCC3 | excision repair cross-complementing rodent repair deficiency, complementation gr | 5.89E-07 | -5.5049 |
| 202393_s_at | KLF10 | Kruppel-like factor 10 | 5.83E-06 | -5.50545 |
| 226354_at | LACTB | lactamase, beta | 1.19E-06 | -5.50583 |
| 204313_s_at | CREB1 | cAMP responsive element binding protein 1 | 8.33E-06 | -5.50612 |
| 213310_at | EIF2C2 | Eukaryotic translation initiation factor 2C, 2 | 5.24E-07 | -5.50617 |
| 226080_at | SSH2 | slingshot homolog 2 (Drosophila) | 1.54E-06 | -5.50791 |
| 219523_s_at | ODZ3 | odz, odd Oz/ten-m homolog 3 (Drosophila) | 1.03E-07 | -5.50937 |
| 226358_at | APH1B | anterior pharynx defective 1 homolog B (C. elegans) | 1.65E-05 | -5.5118 |
| 222990_at | UBQLN1 | ubiquilin 1 | 4.17E-06 | -5.51185 |
| 226423_at | PAQR8 | progestin and adipoQ receptor family member VIII | 2.01E-07 | -5.51289 |
| 209882_at | RIT1 | Ras-like without CAAX 1 | 1.26E-05 | -5.51492 |
| 217819_at | GOLGA7 | golgin A7 | 8.89E-07 | -5.51498 |
| 214460_at | LSAMP | limbic system-associated membrane protein | 3.12E-05 | -5.51505 |
| 222833_at | LPCAT2 | lysophosphatidylcholine acyltransferase 2 | 1.41E-05 | -5.51689 |
| 209928_s_at | MSC | musculin | 5.22E-06 | -5.517 |
| 210092_at | MAGOH | mago-nashi homolog, proliferation-associated (Drosophila) | 2.79E-07 | -5.5172 |
| 1566509_s_at | FBXO9 | F-box protein 9 | 5.33E-07 | -5.51742 |
| 204248_at | GNA11 | guanine nucleotide binding protein (G protein), alpha 11 (Gq class) | 3.39E-05 | -5.51748 |
| 223479_s_at | CHCHD5 | coiled-coil-helix-coiled-coil-helix domain containing 5 | 0.000354 | -5.51765 |
| 1555543_a_at | CLCC1 | chloride channel CLIC-like 1 | 1.73E-06 | -5.51785 |
| 224663_s_at | CFL2 | cofilin 2 (muscle) | 2.20E-06 | -5.51869 |
| 226293_at | MED19 | mediator complex subunit 19 | 8.65E-06 | -5.52034 |
| 202815_s_at | HEXIM1 | hexamethylene bis-acetamide inducible 1 | 7.60E-08 | -5.52077 |
| 217914_at | TPCN1 | two pore segment channel 1 | 3.99E-08 | -5.52108 |
| 225192_at | C10orf46 | chromosome 10 open reading frame 46 | 8.15E-06 | -5.52169 |
| 204868_at | ICT1 | immature colon carcinoma transcript 1 | 2.18E-06 | -5.52178 |
| 225989_at | HERC4 | hect domain and RLD 4 | 4.87E-07 | -5.52196 |
| 218567_x_at | DPP3 | dipeptidyl-peptidase 3 | 9.84E-07 | -5.52205 |
| 228488_at | TBC1D16 | TBC1 domain family, member 16 | 2.87E-06 | -5.52295 |
| 211432_s_at | TYRO3 | TYRO3 protein tyrosine kinase | 2.89E-05 | -5.52414 |
| 203552_at | MAP4K5 | mitogen-activated protein kinase kinase kinase kinase 5 | 3.51E-05 | -5.52485 |
| 227447_at | SKIV2L2 | superkiller viralicidic activity 2-like 2 (S. cerevisiae) | 1.21E-07 | -5.52551 |
| 223090_x_at | VEZT | vezatin, adherens junctions transmembrane protein | 0.000315 | -5.5265 |
| 228373_at | C16orf72 | chromosome 16 open reading frame 72 | 1.34E-06 | -5.52706 |
| 202595_s_at | LEPROTL1 | leptin receptor overlapping transcript-like 1 | 7.40E-08 | -5.52722 |
| 204022_at | WWP2 | WW domain containing E3 ubiquitin protein ligase 2 | 8.79E-08 | -5.52831 |
| 218737_at | SBNO1 | strawberry notch homolog 1 (Drosophila) | 3.27E-06 | -5.53091 |
| 214943_s_at | RBM34 | RNA binding motif protein 34 | 3.21E-06 | -5.53137 |
| 202521_at | CTCF | CCCTC-binding factor (zinc finger protein) | 1.69E-07 | -5.53333 |
| 203089_s_at | HTRA2 | HtrA serine peptidase 2 | 6.17E-06 | -5.53352 |
| 209865_at | SLC35A3 | solute carrier family 35 (UDP-N-acetylglucosamine (UDP-GlcNAc) transporter), mem | 4.06E-06 | -5.53377 |
| 228204_at | PSMB4 | Proteasome (prosome, macropain) subunit, beta type, 4 | 1.58E-07 | -5.53428 |
| 206046_at | ADAM23 | ADAM metallopeptidase domain 23 | 4.29E-07 | -5.53471 |
| 218076_s_at | ARHGAP17 | Rho GTPase activating protein 17 | 3.63E-09 | -5.53512 |
| 209356_x_at | EFEMP2 | EGF-containing fibulin-like extracellular matrix protein 2 | 6.04E-05 | -5.53538 |
| 223482_at | TMEM120A | transmembrane protein 120A | 1.06E-06 | -5.53587 |
| 238523_at | KLHL36 | kelch-like 36 (Drosophila) | 8.12E-06 | -5.53661 |
| 208652_at | PPP2CA | protein phosphatase 2, catalytic subunit, alpha isozyme | 1.74E-07 | -5.53669 |
| 202951_at | STK38 | serine/threonine kinase 38 | 1.78E-07 | -5.53764 |
| 201696_at | SFRS4 | splicing factor, arginine/serine-rich 4 | 2.84E-07 | -5.53835 |
| 213934_s_at | ZNF23 | zinc finger protein 23 (KOX 16) | 2.93E-05 | -5.53933 |
| 212342_at | YIPF6 | Yip1 domain family, member 6 | 1.52E-05 | -5.53939 |
| 201146_at | NFE2L2 | nuclear factor (erythroid-derived 2)-like 2 | 1.80E-06 | -5.53972 |
| 209310_s_at | CASP4 | caspase 4, apoptosis-related cysteine peptidase | 6.91E-06 | -5.54132 |
| 213407_at | PHLPP2 | PH domain and leucine rich repeat protein phosphatase 2 | 1.34E-06 | -5.54136 |
| 224445_s_at | ZFYVE21 | zinc finger, FYVE domain containing 21 | 6.43E-09 | -5.54501 |
| 222955_s_at | FAM45A /// FAM45B | family with sequence similarity 45, member A /// family with sequence similarity | 5.53E-06 | -5.54668 |
| 225277_at | SLC39A13 | solute carrier family 39 (zinc transporter), member 13 | 1.28E-06 | -5.54711 |
| 205684_s_at | DENND4C | DENN/MADD domain containing 4C | 2.97E-05 | -5.54803 |
| 225563_at | PAN3 | PAN3 poly(A) specific ribonuclease subunit homolog (S. cerevisiae) | 4.80E-06 | -5.54828 |
| 203194_s_at | NUP98 | nucleoporin 98kDa | 9.99E-07 | -5.54856 |
| 201520_s_at | GRSF1 | G-rich RNA sequence binding factor 1 | 9.79E-08 | -5.54899 |
| 218130_at | C17orf62 | chromosome 17 open reading frame 62 | 2.50E-05 | -5.54901 |
| 235056_at | ETV6 | ets variant 6 | 5.73E-05 | -5.54911 |
| 224453_s_at | ETNK1 | ethanolamine kinase 1 | 6.47E-06 | -5.54985 |
| 204305_at | MIPEP | mitochondrial intermediate peptidase | 6.14E-06 | -5.55131 |
| 202193_at | LIMK2 | LIM domain kinase 2 | 6.72E-06 | -5.55247 |
| 215089_s_at | RBM10 | RNA binding motif protein 10 | 1.59E-07 | -5.5527 |
| 226965_at | FAM116A | family with sequence similarity 116, member A | 3.26E-07 | -5.55311 |
| 218724_s_at | TGIF2 | TGFB-induced factor homeobox 2 | 2.35E-06 | -5.5536 |
| 226505_x_at | USP32 | ubiquitin specific peptidase 32 | 5.63E-07 | -5.55394 |
| 203269_at | NSMAF | neutral sphingomyelinase (N-SMase) activation associated factor | 2.90E-06 | -5.55467 |
| 219679_s_at | WAC | WW domain containing adaptor with coiled-coil | 4.14E-06 | -5.55492 |
| 209214_s_at | EWSR1 | Ewing sarcoma breakpoint region 1 | 4.28E-06 | -5.55614 |
| 225635_s_at | LOC401504 | Hypothetical gene supported by AK091718 | 3.33E-05 | -5.5579 |
| 212409_s_at | TOR1AIP1 | torsin A interacting protein 1 | 1.03E-06 | -5.55843 |
| 1557081_at | RBM25 | RNA binding motif protein 25 | 2.03E-07 | -5.56038 |
| 226783_at | AGXT2L2 | alanine-glyoxylate aminotransferase 2-like 2 | 2.04E-07 | -5.56198 |
| 200083_at | USP22 | ubiquitin specific peptidase 22 | 5.17E-09 | -5.56222 |
| 231530_s_at | C11orf1 | chromosome 11 open reading frame 1 | 2.79E-06 | -5.5632 |
| 204297_at | PIK3C3 | phosphoinositide-3-kinase, class 3 | 2.17E-06 | -5.56457 |
| 209385_s_at | PROSC | proline synthetase co-transcribed homolog (bacterial) | 1.02E-05 | -5.56477 |
| 230369_at | GPR161 | G protein-coupled receptor 161 | 8.45E-07 | -5.56604 |
| 200041_s_at | BAT1 | HLA-B associated transcript 1 | 2.68E-06 | -5.56805 |
| 201243_s_at | ATP1B1 | ATPase, Na+/K+ transporting, beta 1 polypeptide | 2.22E-08 | -5.56848 |
| 217866_at | CPSF7 | cleavage and polyadenylation specific factor 7, 59kDa | 2.40E-06 | -5.56849 |
| 207836_s_at | RBPMS | RNA binding protein with multiple splicing | 7.65E-08 | -5.56867 |
| 220355_s_at | PBRM1 | polybromo 1 | 2.88E-06 | -5.56987 |
| 237145_at | EIF2AK4 | eukaryotic translation initiation factor 2 alpha kinase 4 | 2.93E-05 | -5.57044 |
| 224377_s_at | RAB18 | RAB18, member RAS oncogene family | 1.22E-06 | -5.57047 |
| 204283_at | FARS2 | phenylalanyl-tRNA synthetase 2, mitochondrial | 7.97E-06 | -5.57096 |
| 217754_at | DDX56 | DEAD (Asp-Glu-Ala-Asp) box polypeptide 56 | 4.72E-07 | -5.57133 |
| 200727_s_at | ACTR2 | ARP2 actin-related protein 2 homolog (yeast) | 1.27E-05 | -5.57165 |
| 223306_at | EBPL | emopamil binding protein-like | 2.82E-07 | -5.57183 |
| 219053_s_at | VPS37C | vacuolar protein sorting 37 homolog C (S. cerevisiae) | 7.39E-06 | -5.57183 |
| 218056_at | BFAR | bifunctional apoptosis regulator | 2.93E-05 | -5.57208 |
| 206861_s_at | CGGBP1 | CGG triplet repeat binding protein 1 | 9.40E-08 | -5.57317 |
| 200878_at | EPAS1 | endothelial PAS domain protein 1 | 2.83E-06 | -5.57442 |
| 227433_at | KIAA2018 | KIAA2018 | 1.02E-05 | -5.5756 |
| 217936_at | ARHGAP5 | Rho GTPase activating protein 5 | 4.39E-05 | -5.57886 |
| 225008_at | ASPH | aspartate beta-hydroxylase | 4.81E-05 | -5.5802 |
| 223701_s_at | USP47 | ubiquitin specific peptidase 47 | 1.26E-05 | -5.58085 |
| 208992_s_at | STAT3 | signal transducer and activator of transcription 3 (acute-phase response factor) | 2.64E-06 | -5.58108 |
| 207144_s_at | CITED1 | Cbp/p300-interacting transactivator, with Glu/Asp-rich carboxy-terminal domain, | 2.20E-06 | -5.58136 |
| 226035_at | USP31 | ubiquitin specific peptidase 31 | 9.37E-05 | -5.58312 |
| 201993_x_at | HNRPDL | heterogeneous nuclear ribonucleoprotein D-like | 9.11E-08 | -5.58426 |
| 216267_s_at | TMEM115 | transmembrane protein 115 | 3.32E-06 | -5.58452 |
| 224998_at | CMTM4 | CKLF-like MARVEL transmembrane domain containing 4 | 2.75E-07 | -5.58505 |
| 207438_s_at | SNUPN | snurportin 1 | 3.90E-08 | -5.58706 |
| 201891_s_at | B2M | beta-2-microglobulin | 1.98E-06 | -5.58715 |
| 227653_at | TRMT5 | TRM5 tRNA methyltransferase 5 homolog (S. cerevisiae) | 4.34E-07 | -5.58803 |
| 201985_at | KIAA0196 | KIAA0196 | 6.96E-07 | -5.5884 |
| 226807_at | ZFP1 | zinc finger protein 1 homolog (mouse) | 3.89E-05 | -5.58855 |
| 214741_at | ZNF131 | zinc finger protein 131 | 1.40E-07 | -5.58903 |
| 1554260_a_at | FRYL | FRY-like | 7.01E-05 | -5.58986 |
| 223101_s_at | ARPC5L | actin related protein 2/3 complex, subunit 5-like | 4.31E-06 | -5.59086 |
| 212341_at | YIPF6 | Yip1 domain family, member 6 | 1.66E-07 | -5.59093 |
| 201494_at | PRCP | prolylcarboxypeptidase (angiotensinase C) | 0.000161 | -5.59175 |
| 224952_at | TANC2 | tetratricopeptide repeat, ankyrin repeat and coiled-coil containing 2 | 1.31E-06 | -5.59337 |
| 218378_s_at | PRKRIP1 | PRKR interacting protein 1 (IL11 inducible) | 2.70E-07 | -5.59428 |
| 217196_s_at | CAMSAP1L1 | calmodulin regulated spectrin-associated protein 1-like 1 | 3.15E-06 | -5.59551 |
| 223818_s_at | RSF1 | remodeling and spacing factor 1 | 1.58E-05 | -5.59795 |
| 200848_at | AHCYL1 | adenosylhomocysteinase-like 1 | 5.87E-06 | -5.59848 |
| 219197_s_at | SCUBE2 | signal peptide, CUB domain, EGF-like 2 | 3.36E-08 | -5.59935 |
| 201919_at | SLC25A36 | solute carrier family 25, member 36 | 2.85E-08 | -5.60137 |
| 227709_at | LOC100190939 | hypothetical LOC100190939 | 7.98E-06 | -5.60263 |
| 213505_s_at | SFRS14 | splicing factor, arginine/serine-rich 14 | 4.20E-06 | -5.60326 |
| 201778_s_at | KIAA0494 | KIAA0494 | 1.19E-09 | -5.60474 |
| 202355_s_at | GTF2F1 | general transcription factor IIF, polypeptide 1, 74kDa | 7.31E-07 | -5.60614 |
| 201817_at | UBE3C | ubiquitin protein ligase E3C | 3.49E-09 | -5.60686 |
| 1559517_a_at | SPIRE1 | spire homolog 1 (Drosophila) | 1.57E-05 | -5.60795 |
| 212673_at | METAP1 | methionyl aminopeptidase 1 | 6.40E-06 | -5.60965 |
| 1567014_s_at | NFE2L2 | nuclear factor (erythroid-derived 2)-like 2 | 4.48E-06 | -5.61013 |
| 225859_at | XIAP | X-linked inhibitor of apoptosis | 1.38E-06 | -5.61053 |
| 212305_s_at | MIA3 | melanoma inhibitory activity family, member 3 | 1.39E-07 | -5.61054 |
| 223478_at | TIMM8B | translocase of inner mitochondrial membrane 8 homolog B (yeast) | 2.79E-05 | -5.61138 |
| 239392_s_at | POGK | pogo transposable element with KRAB domain | 4.98E-06 | -5.61229 |
| 203164_at | SLC33A1 | solute carrier family 33 (acetyl-CoA transporter), member 1 | 1.54E-06 | -5.61333 |
| 200068_s_at | CANX | calnexin | 3.33E-11 | -5.61473 |
| 219303_at | RNF219 | ring finger protein 219 | 2.48E-06 | -5.6158 |
| 217741_s_at | ZFAND5 | zinc finger, AN1-type domain 5 | 3.13E-05 | -5.61605 |
| 204143_s_at | ENOSF1 | enolase superfamily member 1 | 1.33E-05 | -5.61781 |
| 220753_s_at | CRYL1 | crystallin, lambda 1 | 1.12E-08 | -5.61903 |
| 38069_at | CLCN7 | chloride channel 7 | 7.17E-08 | -5.62024 |
| 225508_at | KIAA1468 | KIAA1468 | 2.18E-06 | -5.62031 |
| 204078_at | SC65 | synaptonemal complex protein SC65 | 1.42E-05 | -5.62161 |
| 203192_at | ABCB6 | ATP-binding cassette, sub-family B (MDR/TAP), member 6 | 2.95E-05 | -5.62166 |
| 224228_s_at | PRDM7 | PR domain containing 7 | 0.000107 | -5.62432 |
| 225766_s_at | TNPO1 | transportin 1 | 3.23E-06 | -5.62725 |
| 202092_s_at | ARL2BP | ADP-ribosylation factor-like 2 binding protein | 3.86E-07 | -5.62756 |
| 228330_at | ZUFSP | zinc finger with UFM1-specific peptidase domain | 1.46E-05 | -5.6276 |
| 1554703_at | ARHGEF10 | Rho guanine nucleotide exchange factor (GEF) 10 | 4.85E-06 | -5.62856 |
| 233496_s_at | CFL2 | cofilin 2 (muscle) | 9.61E-06 | -5.62922 |
| 222775_s_at | MRPL35 | mitochondrial ribosomal protein L35 | 1.58E-09 | -5.63241 |
| 201722_s_at | GALNT1 | UDP-N-acetyl-alpha-D-galactosamine:polypeptide N-acetylgalactosaminyltransferase | 1.65E-06 | -5.63394 |
| 203297_s_at | JARID2 | jumonji, AT rich interactive domain 2 | 4.96E-08 | -5.63647 |
| 213018_at | GATAD1 | GATA zinc finger domain containing 1 | 2.27E-06 | -5.63717 |
| 200774_at | FAM120A | family with sequence similarity 120A | 1.32E-06 | -5.63783 |
| 210011_s_at | EWSR1 | Ewing sarcoma breakpoint region 1 | 1.99E-05 | -5.63881 |
| 210299_s_at | FHL1 | four and a half LIM domains 1 | 5.81E-05 | -5.64016 |
| 228031_at | TTPAL | tocopherol (alpha) transfer protein-like | 5.13E-07 | -5.64233 |
| 214743_at | CUX1 | cut-like homeobox 1 | 4.01E-09 | -5.64294 |
| 209944_at | ZNF410 | zinc finger protein 410 | 4.07E-06 | -5.64412 |
| 221493_at | TSPYL1 | TSPY-like 1 | 2.84E-06 | -5.6454 |
| 224823_at | MYLK | myosin light chain kinase | 4.54E-07 | -5.64564 |
| 217202_s_at | GLUL | glutamate-ammonia ligase | 2.60E-06 | -5.64876 |
| 202484_s_at | MBD2 | methyl-CpG binding domain protein 2 | 1.57E-07 | -5.64896 |
| 204790_at | SMAD7 | SMAD family member 7 | 2.58E-06 | -5.65047 |
| 220941_s_at | C21orf91 | chromosome 21 open reading frame 91 | 4.30E-06 | -5.65126 |
| 202130_at | RIOK3 | RIO kinase 3 (yeast) | 2.24E-08 | -5.654 |
| 208673_s_at | SFRS3 | splicing factor, arginine/serine-rich 3 | 1.18E-05 | -5.65416 |
| 232591_s_at | TMEM30A | transmembrane protein 30A | 6.27E-05 | -5.65474 |
| 211126_s_at | CSRP2 | cysteine and glycine-rich protein 2 | 1.99E-06 | -5.65613 |
| 202530_at | MAPK14 | mitogen-activated protein kinase 14 | 3.59E-07 | -5.65673 |
| 201783_s_at | RELA | v-rel reticuloendotheliosis viral oncogene homolog A (avian) | 2.61E-07 | -5.65741 |
| 211700_s_at | TRO | trophinin | 0.00011 | -5.65803 |
| 201029_s_at | CD99 | CD99 molecule | 5.40E-10 | -5.66265 |
| 226561_at | AGFG1 | ArfGAP with FG repeats 1 | 2.16E-06 | -5.66334 |
| 225915_at | CAB39L | calcium binding protein 39-like | 9.56E-06 | -5.6642 |
| 226607_at | C20orf194 | chromosome 20 open reading frame 194 | 1.39E-07 | -5.66448 |
| 208786_s_at | MAP1LC3B | microtubule-associated protein 1 light chain 3 beta | 7.19E-08 | -5.66542 |
| 228805_at | C5orf25 | chromosome 5 open reading frame 25 | 1.43E-06 | -5.66589 |
| 209259_s_at | SMC3 | structural maintenance of chromosomes 3 | 1.62E-05 | -5.66602 |
| 203437_at | TMEM11 | transmembrane protein 11 | 1.00E-06 | -5.66658 |
| 228124_at | ABHD12 | abhydrolase domain containing 12 | 4.06E-07 | -5.66688 |
| 225808_at | C17orf95 | chromosome 17 open reading frame 95 | 2.02E-07 | -5.66724 |
| 201191_at | PITPNA | phosphatidylinositol transfer protein, alpha | 4.33E-06 | -5.66744 |
| 223433_at | C7orf36 | chromosome 7 open reading frame 36 | 1.51E-06 | -5.66798 |
| 225368_at | HIPK2 | homeodomain interacting protein kinase 2 | 2.29E-06 | -5.66936 |
| 218855_at | TPRA1 | transmembrane protein, adipocyte asscociated 1 | 1.55E-06 | -5.66963 |
| 211038_s_at | CROCCL1 | ciliary rootlet coiled-coil, rootletin-like 1 | 3.70E-06 | -5.67073 |
| 229676_at | MTPAP | Mitochondrial poly(A) polymerase | 5.60E-07 | -5.67077 |
| 208649_s_at | VCP | valosin-containing protein | 1.70E-08 | -5.67136 |
| 208698_s_at | NONO | non-POU domain containing, octamer-binding | 6.90E-07 | -5.67191 |
| 203855_at | WDR47 | WD repeat domain 47 | 7.69E-05 | -5.67198 |
| 222469_s_at | TOLLIP | toll interacting protein | 7.89E-07 | -5.6722 |
| 225676_s_at | DCAF13 | DDB1 and CUL4 associated factor 13 | 5.18E-06 | -5.67231 |
| 202119_s_at | CPNE3 | copine III | 1.54E-06 | -5.67244 |
| 204045_at | TCEAL1 | transcription elongation factor A (SII)-like 1 | 3.32E-06 | -5.67274 |
| 202097_at | NUP153 | nucleoporin 153kDa | 1.30E-07 | -5.67497 |
| 1552627_a_at | ARHGAP5 | Rho GTPase activating protein 5 | 9.07E-06 | -5.67575 |
| 224711_at | YY1 | YY1 transcription factor | 2.39E-05 | -5.67604 |
| 209486_at | UTP3 | UTP3, small subunit (SSU) processome component, homolog (S. cerevisiae) | 1.30E-05 | -5.67665 |
| 213109_at | TNIK | TRAF2 and NCK interacting kinase | 1.22E-06 | -5.67707 |
| 205443_at | SNAPC1 | small nuclear RNA activating complex, polypeptide 1, 43kDa | 5.75E-06 | -5.6815 |
| 201245_s_at | OTUB1 | OTU domain, ubiquitin aldehyde binding 1 | 3.31E-08 | -5.68278 |
| 219895_at | FAM70A | family with sequence similarity 70, member A | 1.49E-05 | -5.68362 |
| 208794_s_at | SMARCA4 | SWI/SNF related, matrix associated, actin dependent regulator of chromatin, subf | 3.44E-07 | -5.684 |
| 208634_s_at | MACF1 | microtubule-actin crosslinking factor 1 | 1.31E-08 | -5.68555 |
| 222156_x_at | CCPG1 | cell cycle progression 1 | 8.35E-06 | -5.68565 |
| 222761_at | BIVM | basic, immunoglobulin-like variable motif containing | 1.04E-05 | -5.68681 |
| 224868_at | ZDHHC5 | zinc finger, DHHC-type containing 5 | 1.04E-05 | -5.69087 |
| 208963_x_at | FADS1 | fatty acid desaturase 1 | 2.49E-06 | -5.69297 |
| 225186_at | RAPH1 | Ras association (RalGDS/AF-6) and pleckstrin homology domains 1 | 1.85E-07 | -5.69516 |
| 202724_s_at | FOXO1 | forkhead box O1 | 5.15E-06 | -5.69524 |
| 226689_at | CISD2 | CDGSH iron sulfur domain 2 | 2.34E-06 | -5.70201 |
| 209578_s_at | POFUT2 | protein O-fucosyltransferase 2 | 2.91E-07 | -5.70241 |
| 225260_s_at | MRPL32 | mitochondrial ribosomal protein L32 | 2.40E-09 | -5.70611 |
| 228992_at | MED28 | Mediator complex subunit 28 | 1.59E-07 | -5.70679 |
| 204190_at | USPL1 | ubiquitin specific peptidase like 1 | 5.79E-06 | -5.70708 |
| 221514_at | UTP14A | UTP14, U3 small nucleolar ribonucleoprotein, homolog A (yeast) | 2.15E-06 | -5.70767 |
| 223225_s_at | SEH1L | SEH1-like (S. cerevisiae) | 0.000782 | -5.70775 |
| 201136_at | PLP2 | proteolipid protein 2 (colonic epithelium-enriched) | 1.08E-09 | -5.70798 |
| 217906_at | KLHDC2 | kelch domain containing 2 | 5.69E-07 | -5.70876 |
| 204409_s_at | EIF1AY | eukaryotic translation initiation factor 1A, Y-linked | 7.79E-05 | -5.71079 |
| 213001_at | ANGPTL2 | angiopoietin-like 2 | 2.58E-06 | -5.71087 |
| 219979_s_at | C11orf73 | chromosome 11 open reading frame 73 | 4.09E-05 | -5.71103 |
| 209033_s_at | DYRK1A | dual-specificity tyrosine-(Y)-phosphorylation regulated kinase 1A | 5.34E-10 | -5.7111 |
| 202813_at | TARBP1 | TAR (HIV-1) RNA binding protein 1 | 1.73E-06 | -5.71164 |
| 210852_s_at | AASS | aminoadipate-semialdehyde synthase | 5.52E-06 | -5.71256 |
| 213483_at | PPWD1 | peptidylprolyl isomerase domain and WD repeat containing 1 | 3.21E-06 | -5.71346 |
| 228318_s_at | CRIPAK | cysteine-rich PAK1 inhibitor | 5.69E-08 | -5.71453 |
| 36553_at | ASMTL | acetylserotonin O-methyltransferase-like | 2.48E-07 | -5.71493 |
| 220946_s_at | SETD2 | SET domain containing 2 | 0.000145 | -5.71695 |
| 202716_at | PTPN1 | protein tyrosine phosphatase, non-receptor type 1 | 3.84E-06 | -5.71901 |
| 227077_at | ZNF286A /// ZNF286B | zinc finger protein 286A /// zinc finger protein 286B | 1.91E-06 | -5.72015 |
| 217550_at | ATF6 | Activating transcription factor 6 | 7.32E-05 | -5.72081 |
| 223400_s_at | PBRM1 | polybromo 1 | 0.000117 | -5.72139 |
| 226541_at | FBXO30 | F-box protein 30 | 2.27E-06 | -5.72161 |
| 202062_s_at | SEL1L | sel-1 suppressor of lin-12-like (C. elegans) | 3.06E-05 | -5.72195 |
| 200874_s_at | NOP56 | NOP56 ribonucleoprotein homolog (yeast) | 8.06E-06 | -5.72573 |
| 212306_at | CLASP2 | cytoplasmic linker associated protein 2 | 5.21E-07 | -5.72842 |
| 202177_at | GAS6 | growth arrest-specific 6 | 3.87E-06 | -5.73052 |
| 204732_s_at | TRIM23 | tripartite motif-containing 23 | 9.09E-06 | -5.73181 |
| 222231_s_at | LRRC59 | leucine rich repeat containing 59 | 3.25E-08 | -5.73182 |
| 225466_at | PATL1 | protein associated with topoisomerase II homolog 1 (yeast) | 2.02E-06 | -5.73227 |
| 222529_at | SLC25A37 | solute carrier family 25, member 37 | 9.26E-07 | -5.73237 |
| 225890_at | C20orf72 | chromosome 20 open reading frame 72 | 5.12E-06 | -5.73309 |
| 225569_at | EIF2C2 | Eukaryotic translation initiation factor 2C, 2 | 5.43E-07 | -5.73358 |
| 208325_s_at | AKAP13 | A kinase (PRKA) anchor protein 13 | 2.27E-05 | -5.73468 |
| 230793_at | LRRC16A | leucine rich repeat containing 16A | 2.68E-05 | -5.73592 |
| 211686_s_at | MAK16 | MAK16 homolog (S. cerevisiae) | 1.37E-06 | -5.73611 |
| 218309_at | CAMK2N1 | calcium/calmodulin-dependent protein kinase II inhibitor 1 | 3.31E-07 | -5.73628 |
| 210970_s_at | IBTK | inhibitor of Bruton agammaglobulinemia tyrosine kinase | 6.62E-05 | -5.73638 |
| 216521_s_at | BRCC3 | BRCA1/BRCA2-containing complex, subunit 3 | 6.88E-05 | -5.73639 |
| 204653_at | TFAP2A | transcription factor AP-2 alpha (activating enhancer binding protein 2 alpha) | 1.03E-07 | -5.73738 |
| 201340_s_at | ENC1 | ectodermal-neural cortex 1 (with BTB-like domain) | 8.22E-05 | -5.73779 |
| 208097_s_at | TMX1 | thioredoxin-related transmembrane protein 1 | 1.56E-06 | -5.73782 |
| 218137_s_at | SMAP1 | small ArfGAP 1 | 1.76E-07 | -5.73807 |
| 223048_at | SDHAF2 | succinate dehydrogenase complex assembly factor 2 | 2.47E-05 | -5.73884 |
| 201927_s_at | PKP4 | plakophilin 4 | 3.83E-06 | -5.73987 |
| 227514_at | ITPRIPL2 | inositol 1,4,5-triphosphate receptor interacting protein-like 2 | 5.50E-05 | -5.74069 |
| 216060_s_at | DAAM1 | dishevelled associated activator of morphogenesis 1 | 2.97E-05 | -5.74261 |
| 221989_at | RPL10 | ribosomal protein L10 | 4.41E-08 | -5.74446 |
| 213511_s_at | MTMR1 | myotubularin related protein 1 | 4.86E-05 | -5.74618 |
| 201100_s_at | USP9X | ubiquitin specific peptidase 9, X-linked | 4.01E-08 | -5.74656 |
| 209566_at | INSIG2 | insulin induced gene 2 | 6.83E-07 | -5.74713 |
| 202654_x_at | 7-三月 | membrane-associated ring finger (C3HC4) 7 | 9.39E-06 | -5.74717 |
| 224849_at | TTC17 | tetratricopeptide repeat domain 17 | 2.81E-07 | -5.74981 |
| 211505_s_at | STAU1 | staufen, RNA binding protein, homolog 1 (Drosophila) | 1.16E-06 | -5.75005 |
| 228986_at | OSBPL8 | oxysterol binding protein-like 8 | 6.97E-05 | -5.7504 |
| 202156_s_at | CELF2 | CUGBP, Elav-like family member 2 | 1.22E-05 | -5.75124 |
| 226875_at | DOCK11 | dedicator of cytokinesis 11 | 8.21E-06 | -5.7514 |
| 226733_at | PFKFB2 | 6-phosphofructo-2-kinase/fructose-2,6-biphosphatase 2 | 3.71E-06 | -5.75145 |
| 229033_s_at | MUM1 | melanoma associated antigen (mutated) 1 | 2.12E-07 | -5.75161 |
| 201118_at | PGD | phosphogluconate dehydrogenase | 4.72E-07 | -5.75208 |
| 226202_at | ZNF398 | zinc finger protein 398 | 3.53E-06 | -5.75216 |
| 219029_at | C5orf28 | chromosome 5 open reading frame 28 | 5.88E-06 | -5.75422 |
| 219178_at | QTRTD1 | queuine tRNA-ribosyltransferase domain containing 1 | 8.68E-06 | -5.75501 |
| 209446_s_at | C7orf44 | chromosome 7 open reading frame 44 | 1.49E-07 | -5.75647 |
| 219332_at | MICALL2 | MICAL-like 2 | 3.69E-07 | -5.75666 |
| 201602_s_at | PPP1R12A | protein phosphatase 1, regulatory (inhibitor) subunit 12A | 6.30E-06 | -5.75749 |
| 222516_at | AP3M1 | adaptor-related protein complex 3, mu 1 subunit | 1.14E-07 | -5.75804 |
| 219600_s_at | TMEM50B | transmembrane protein 50B | 1.02E-05 | -5.76105 |
| 200652_at | SSR2 | signal sequence receptor, beta (translocon-associated protein beta) | 6.48E-08 | -5.76182 |
| 203207_s_at | MTFR1 | mitochondrial fission regulator 1 | 3.93E-07 | -5.7629 |
| 210616_s_at | SEC31A | SEC31 homolog A (S. cerevisiae) | 1.18E-05 | -5.76302 |
| 203300_x_at | AP1S2 | adaptor-related protein complex 1, sigma 2 subunit | 2.09E-06 | -5.76458 |
| 212380_at | FTSJD2 | FtsJ methyltransferase domain containing 2 | 1.32E-06 | -5.76481 |
| 221229_s_at | TRMT61B | tRNA methyltransferase 61 homolog B (S. cerevisiae) | 0.000292 | -5.76491 |
| 218539_at | FBXO34 | F-box protein 34 | 5.48E-07 | -5.76513 |
| 207855_s_at | CLCC1 | chloride channel CLIC-like 1 | 5.86E-07 | -5.76619 |
| 214739_at | LRCH3 | leucine-rich repeats and calponin homology (CH) domain containing 3 | 3.97E-06 | -5.7667 |
| 224714_at | MKI67IP | MKI67 (FHA domain) interacting nucleolar phosphoprotein | 8.06E-08 | -5.76684 |
| 224704_at | TNRC6A | trinucleotide repeat containing 6A | 1.80E-05 | -5.76778 |
| 218398_at | MRPS30 | mitochondrial ribosomal protein S30 | 8.63E-09 | -5.7682 |
| 202024_at | ASNA1 | arsA arsenite transporter, ATP-binding, homolog 1 (bacterial) | 3.76E-06 | -5.76835 |
| 226247_at | PLEKHA1 | pleckstrin homology domain containing, family A (phosphoinositide binding specif | 3.42E-05 | -5.76978 |
| 206976_s_at | HSPH1 | heat shock 105kDa/110kDa protein 1 | 2.41E-05 | -5.76995 |
| 223336_s_at | RAB18 | RAB18, member RAS oncogene family | 3.62E-07 | -5.77128 |
| 203252_at | CDK2AP2 | cyclin-dependent kinase 2 associated protein 2 | 1.82E-06 | -5.77141 |
| 1566603_s_at | RPUSD3 | RNA pseudouridylate synthase domain containing 3 | 7.03E-05 | -5.77313 |
| 201941_at | CPD | carboxypeptidase D | 1.48E-06 | -5.77387 |
| 203466_at | MPV17 | MpV17 mitochondrial inner membrane protein | 4.32E-05 | -5.77428 |
| 217790_s_at | SSR3 | signal sequence receptor, gamma (translocon-associated protein gamma) | 2.66E-06 | -5.77436 |
| 219751_at | SETD6 | SET domain containing 6 | 5.72E-06 | -5.77533 |
| 229741_at | MAVS | mitochondrial antiviral signaling protein | 3.36E-07 | -5.77713 |
| 228310_at | ENAH | enabled homolog (Drosophila) | 1.68E-07 | -5.77838 |
| 209667_at | CES2 | carboxylesterase 2 (intestine, liver) | 1.20E-07 | -5.77899 |
| 202680_at | GTF2E2 | general transcription factor IIE, polypeptide 2, beta 34kDa | 5.66E-07 | -5.78008 |
| 212375_at | EP400 | E1A binding protein p400 | 6.79E-07 | -5.78027 |
| 230739_at | C18orf19 | chromosome 18 open reading frame 19 | 6.70E-06 | -5.7809 |
| 223382_s_at | ZNRF1 | zinc and ring finger 1 | 4.87E-06 | -5.781 |
| 201937_s_at | DNPEP | aspartyl aminopeptidase | 0.00024 | -5.78101 |
| 220917_s_at | WDR19 | WD repeat domain 19 | 1.67E-05 | -5.78134 |
| 213882_at | TM2D1 | TM2 domain containing 1 | 8.10E-06 | -5.78162 |
| 213009_s_at | TRIM37 | tripartite motif-containing 37 | 2.97E-06 | -5.78209 |
| 214112_s_at | CXorf40A /// CXorf40B | chromosome X open reading frame 40A /// chromosome X open reading frame 40B | 5.40E-08 | -5.78342 |
| 218440_at | MCCC1 | methylcrotonoyl-CoA carboxylase 1 (alpha) | 1.29E-05 | -5.78475 |
| 205961_s_at | PSIP1 | PC4 and SFRS1 interacting protein 1 | 6.60E-06 | -5.78506 |
| 220610_s_at | LRRFIP2 | leucine rich repeat (in FLII) interacting protein 2 | 3.39E-08 | -5.78545 |
| 212674_s_at | DHX30 | DEAH (Asp-Glu-Ala-His) box polypeptide 30 | 0.000107 | -5.78721 |
| 208475_at | FRMD4A | FERM domain containing 4A | 9.94E-06 | -5.78838 |
| 219008_at | C2orf43 | chromosome 2 open reading frame 43 | 1.24E-06 | -5.78981 |
| 212902_at | SEC24A | SEC24 family, member A (S. cerevisiae) | 3.79E-05 | -5.79134 |
| 216604_s_at | SLC7A8 | solute carrier family 7 (amino acid transporter, L-type), member 8 | 1.27E-07 | -5.79191 |
| 218092_s_at | AGFG1 | ArfGAP with FG repeats 1 | 1.05E-07 | -5.79226 |
| 208907_s_at | MRPS18B | mitochondrial ribosomal protein S18B | 4.20E-08 | -5.79347 |
| 204791_at | NR2C1 | nuclear receptor subfamily 2, group C, member 1 | 4.97E-06 | -5.79423 |
| 202794_at | INPP1 | inositol polyphosphate-1-phosphatase | 2.95E-06 | -5.79567 |
| 211783_s_at | MTA1 | metastasis associated 1 | 2.63E-07 | -5.79573 |
| 219818_s_at | GPATCH1 | G patch domain containing 1 | 1.30E-07 | -5.79603 |
| 218397_at | FANCL | Fanconi anemia, complementation group L | 1.75E-05 | -5.79674 |
| 224163_s_at | DMAP1 | DNA methyltransferase 1 associated protein 1 | 5.40E-07 | -5.797 |
| 225364_at | STK4 | serine/threonine kinase 4 | 0.000111 | -5.79757 |
| 223268_at | C11orf54 | chromosome 11 open reading frame 54 | 1.02E-05 | -5.79936 |
| 225198_at | VAPA | VAMP (vesicle-associated membrane protein)-associated protein A, 33kDa | 8.72E-06 | -5.79946 |
| 1555841_at | C9orf30 | chromosome 9 open reading frame 30 | 3.29E-07 | -5.8011 |
| 223431_at | CNO | cappuccino homolog (mouse) | 2.03E-06 | -5.80342 |
| 212771_at | FAM171A1 | family with sequence similarity 171, member A1 | 5.21E-08 | -5.80342 |
| 1563445_x_at | LOC1518 | cathepsin L1 pseudogene | 6.02E-05 | -5.8036 |
| 203905_at | PARN | poly(A)-specific ribonuclease (deadenylation nuclease) | 6.64E-07 | -5.80443 |
| 207956_x_at | PDS5B | PDS5, regulator of cohesion maintenance, homolog B (S. cerevisiae) | 1.15E-07 | -5.80471 |
| 201777_s_at | KIAA0494 | KIAA0494 | 6.71E-06 | -5.80479 |
| 211763_s_at | UBE2B | ubiquitin-conjugating enzyme E2B (RAD6 homolog) | 9.61E-09 | -5.80537 |
| 222422_s_at | NDFIP1 | Nedd4 family interacting protein 1 | 1.71E-06 | -5.80568 |
| 225918_at | GLG1 | golgi glycoprotein 1 | 7.03E-06 | -5.80594 |
| 213073_at | ZFYVE26 | zinc finger, FYVE domain containing 26 | 5.11E-07 | -5.80751 |
| 218238_at | GTPBP4 | GTP binding protein 4 | 9.94E-08 | -5.80808 |
| 200825_s_at | HYOU1 | hypoxia up-regulated 1 | 5.83E-06 | -5.80891 |
| 218244_at | NOL8 | nucleolar protein 8 | 1.23E-06 | -5.80893 |
| 224730_at | DCAF7 | DDB1 and CUL4 associated factor 7 | 6.13E-06 | -5.80963 |
| 202898_at | SDC3 | syndecan 3 | 6.10E-06 | -5.80978 |
| 202723_s_at | FOXO1 | forkhead box O1 | 9.31E-06 | -5.81123 |
| 225291_at | PNPT1 | polyribonucleotide nucleotidyltransferase 1 | 5.92E-06 | -5.81163 |
| 222687_s_at | ACER3 | alkaline ceramidase 3 | 1.31E-07 | -5.81303 |
| 203968_s_at | CDC6 | cell division cycle 6 homolog (S. cerevisiae) | 2.08E-07 | -5.81317 |
| 227094_at | DHTKD1 | dehydrogenase E1 and transketolase domain containing 1 | 3.57E-06 | -5.81382 |
| 225132_at | FBXL3 | F-box and leucine-rich repeat protein 3 | 1.31E-07 | -5.81573 |
| 216199_s_at | MAP3K4 | mitogen-activated protein kinase kinase kinase 4 | 3.69E-05 | -5.81587 |
| 217861_s_at | PREB | prolactin regulatory element binding | 2.37E-06 | -5.81631 |
| 214686_at | ZNF266 | zinc finger protein 266 | 7.22E-06 | -5.81701 |
| 203848_at | AKAP8 | A kinase (PRKA) anchor protein 8 | 1.93E-08 | -5.81765 |
| 212329_at | SCAP | SREBF chaperone | 1.14E-08 | -5.81868 |
| 36994_at | ATP6V0C | ATPase, H+ transporting, lysosomal 16kDa, V0 subunit c | 4.26E-07 | -5.81918 |
| 201057_s_at | GOLGB1 | golgin B1 | 7.39E-07 | -5.82032 |
| 201711_x_at | RANBP2 | RAN binding protein 2 | 2.03E-05 | -5.82123 |
| 225265_at | RBMS1 | RNA binding motif, single stranded interacting protein 1 | 0.000159 | -5.82297 |
| 219633_at | TTPAL | tocopherol (alpha) transfer protein-like | 2.07E-06 | -5.82451 |
| 223227_at | BBS2 | Bardet-Biedl syndrome 2 | 1.74E-06 | -5.82717 |
| 201583_s_at | SEC23B | Sec23 homolog B (S. cerevisiae) | 1.17E-07 | -5.82767 |
| 226159_at | C5orf51 | chromosome 5 open reading frame 51 | 3.79E-07 | -5.82837 |
| 212894_at | SUPV3L1 | suppressor of var1, 3-like 1 (S. cerevisiae) | 2.02E-07 | -5.82906 |
| 202622_s_at | ATXN2 | ataxin 2 | 1.70E-06 | -5.82927 |
| 218940_at | C14orf138 | chromosome 14 open reading frame 138 | 1.12E-05 | -5.83047 |
| 209994_s_at | ABCB1 /// ABCB4 | ATP-binding cassette, sub-family B (MDR/TAP), member 1 /// ATP-binding cassette, | 6.84E-07 | -5.83154 |
| 225811_at | C11orf58 | chromosome 11 open reading frame 58 | 4.06E-08 | -5.83194 |
| 201474_s_at | ITGA3 | integrin, alpha 3 (antigen CD49C, alpha 3 subunit of VLA-3 receptor) | 3.49E-05 | -5.83403 |
| 203201_at | PMM2 | phosphomannomutase 2 | 3.03E-06 | -5.83538 |
| 203689_s_at | FMR1 | fragile X mental retardation 1 | 1.58E-05 | -5.83545 |
| 201279_s_at | DAB2 | disabled homolog 2, mitogen-responsive phosphoprotein (Drosophila) | 1.98E-06 | -5.83872 |
| 222406_s_at | PNRC2 | proline-rich nuclear receptor coactivator 2 | 4.45E-05 | -5.84024 |
| 210685_s_at | UBE4B | ubiquitination factor E4B (UFD2 homolog, yeast) | 1.65E-06 | -5.841 |
| 220143_x_at | LUC7L | LUC7-like (S. cerevisiae) | 2.61E-06 | -5.8412 |
| 227462_at | ERAP2 | endoplasmic reticulum aminopeptidase 2 | 4.36E-06 | -5.84133 |
| 227487_s_at | SERPINE2 | Serpin peptidase inhibitor, clade E (nexin, plasminogen activator inhibitor type | 2.43E-06 | -5.84268 |
| 210438_x_at | TROVE2 | TROVE domain family, member 2 | 2.88E-08 | -5.84275 |
| 226366_at | SHPRH | SNF2 histone linker PHD RING helicase | 3.11E-06 | -5.84415 |
| 213465_s_at | PPP1R7 | protein phosphatase 1, regulatory (inhibitor) subunit 7 | 2.75E-08 | -5.84686 |
| 207515_s_at | POLR1C | polymerase (RNA) I polypeptide C, 30kDa | 3.25E-08 | -5.8473 |
| 226259_at | EXOC6 | exocyst complex component 6 | 7.42E-07 | -5.84745 |
| 201128_s_at | ACLY | ATP citrate lyase | 1.17E-07 | -5.8484 |
| 218156_s_at | TSR1 | TSR1, 20S rRNA accumulation, homolog (S. cerevisiae) | 4.08E-08 | -5.84844 |
| 202208_s_at | ARL4C | ADP-ribosylation factor-like 4C | 1.60E-05 | -5.84967 |
| 228830_s_at | ATF7 | activating transcription factor 7 | 4.12E-06 | -5.85302 |
| 212137_at | LARP1 | La ribonucleoprotein domain family, member 1 | 1.54E-07 | -5.85419 |
| 1554365_a_at | PPP2R5C | protein phosphatase 2, regulatory subunit B', gamma | 8.00E-06 | -5.85443 |
| 217784_at | YKT6 | YKT6 v-SNARE homolog (S. cerevisiae) | 1.33E-06 | -5.85446 |
| 217427_s_at | HIRA | HIR histone cell cycle regulation defective homolog A (S. cerevisiae) | 5.30E-05 | -5.85548 |
| 225261_x_at | TH1L | TH1-like (Drosophila) | 9.45E-09 | -5.85552 |
| 212493_s_at | SETD2 | SET domain containing 2 | 1.50E-06 | -5.85737 |
| 201308_s_at | 11-九月 | septin 11 | 3.08E-05 | -5.85779 |
| 1557292_a_at | MCOLN3 | mucolipin 3 | 5.45E-07 | -5.86094 |
| 226853_at | BMP2K | BMP2 inducible kinase | 0.000162 | -5.86121 |
| 217930_s_at | TOLLIP | toll interacting protein | 2.10E-07 | -5.86141 |
| 206095_s_at | SFRS13A | splicing factor, arginine/serine-rich 13A | 1.08E-06 | -5.86164 |
| 223223_at | ARV1 | ARV1 homolog (S. cerevisiae) | 8.98E-08 | -5.86292 |
| 225963_at | KLHDC5 | kelch domain containing 5 | 6.01E-06 | -5.86345 |
| 212482_at | RMND5A | required for meiotic nuclear division 5 homolog A (S. cerevisiae) | 1.86E-08 | -5.8653 |
| 213579_s_at | EP300 | E1A binding protein p300 | 1.15E-05 | -5.86556 |
| 218179_s_at | C4orf41 | chromosome 4 open reading frame 41 | 3.17E-05 | -5.86859 |
| 52940_at | LOC100294402 /// SIGIRR | similar to single Ig IL-1R-related molecule /// single immunoglobulin and toll-i | 3.08E-07 | -5.86909 |
| 212712_at | CAMSAP1 | calmodulin regulated spectrin-associated protein 1 | 1.47E-06 | -5.86973 |
| 213931_at | ID2 /// ID2B | inhibitor of DNA binding 2, dominant negative helix-loop-helix protein /// inhib | 6.09E-07 | -5.87069 |
| 209387_s_at | TM4SF1 | transmembrane 4 L six family member 1 | 4.94E-07 | -5.87125 |
| 200701_at | NPC2 | Niemann-Pick disease, type C2 | 2.32E-10 | -5.87155 |
| 207108_s_at | NIPBL | Nipped-B homolog (Drosophila) | 2.21E-05 | -5.87229 |
| 226298_at | RUNDC1 | RUN domain containing 1 | 4.83E-06 | -5.87255 |
| 212796_s_at | TBC1D2B | TBC1 domain family, member 2B | 2.06E-07 | -5.87266 |
| 228953_at | WHAMM | WAS protein homolog associated with actin, golgi membranes and microtubules | 8.68E-07 | -5.8731 |
| 225231_at | CBL | Cas-Br-M (murine) ecotropic retroviral transforming sequence | 2.72E-06 | -5.87337 |
| 204488_at | DOLK | dolichol kinase | 5.99E-06 | -5.87351 |
| 221821_s_at | C12orf41 | chromosome 12 open reading frame 41 | 2.50E-06 | -5.87692 |
| 224963_at | SLC26A2 | solute carrier family 26 (sulfate transporter), member 2 | 1.60E-07 | -5.87887 |
| 227776_at | ACER3 | alkaline ceramidase 3 | 1.10E-07 | -5.87942 |
| 224904_at | PDPR | pyruvate dehydrogenase phosphatase regulatory subunit | 1.43E-05 | -5.88207 |
| 204266_s_at | CHKA | choline kinase alpha | 1.95E-07 | -5.88226 |
| 227093_at | USP36 | Ubiquitin specific peptidase 36 | 1.90E-07 | -5.88476 |
| 203718_at | PNPLA6 | patatin-like phospholipase domain containing 6 | 1.84E-05 | -5.88576 |
| 225646_at | CTSC | cathepsin C | 9.06E-06 | -5.88621 |
| 222715_s_at | SYNRG | synergin, gamma | 1.32E-06 | -5.88666 |
| 213469_at | PGAP1 | post-GPI attachment to proteins 1 | 8.03E-05 | -5.88743 |
| 219885_at | SLFN12 | schlafen family member 12 | 5.48E-06 | -5.88762 |
| 218927_s_at | CHST12 | carbohydrate (chondroitin 4) sulfotransferase 12 | 4.24E-08 | -5.88773 |
| 201963_at | ACSL1 | acyl-CoA synthetase long-chain family member 1 | 2.55E-05 | -5.89113 |
| 40420_at | STK10 | serine/threonine kinase 10 | 9.04E-07 | -5.89325 |
| 202204_s_at | AMFR | autocrine motility factor receptor | 3.11E-06 | -5.89429 |
| 219067_s_at | NSMCE4A | non-SMC element 4 homolog A (S. cerevisiae) | 1.05E-07 | -5.89675 |
| 218866_s_at | POLR3K | polymerase (RNA) III (DNA directed) polypeptide K, 12.3 kDa | 1.21E-07 | -5.89682 |
| 228328_at | KLHL28 | kelch-like 28 (Drosophila) | 1.79E-05 | -5.90104 |
| 224957_at | C18orf32 | chromosome 18 open reading frame 32 | 1.18E-06 | -5.90151 |
| 209304_x_at | GADD45B | growth arrest and DNA-damage-inducible, beta | 1.89E-06 | -5.90224 |
| 205681_at | BCL2A1 | BCL2-related protein A1 | 1.62E-05 | -5.90444 |
| 211185_s_at | SF3B1 | splicing factor 3b, subunit 1, 155kDa | 2.73E-07 | -5.90569 |
| 212150_at | EFR3A | EFR3 homolog A (S. cerevisiae) | 2.60E-05 | -5.90596 |
| 218846_at | MED23 | mediator complex subunit 23 | 3.15E-05 | -5.90708 |
| 225730_s_at | THUMPD3 | THUMP domain containing 3 | 5.23E-06 | -5.90777 |
| 226600_at | TMTC3 | transmembrane and tetratricopeptide repeat containing 3 | 5.82E-06 | -5.90974 |
| 214946_x_at | FAM21A /// FAM21B /// FAM21C /// FAM21D | family with sequence similarity 21, member A /// family with sequence similarity | 3.90E-07 | -5.91013 |
| 203879_at | PIK3CD | phosphoinositide-3-kinase, catalytic, delta polypeptide | 7.31E-07 | -5.91137 |
| 226151_x_at | CRYZL1 | crystallin, zeta (quinone reductase)-like 1 | 4.65E-09 | -5.91284 |
| 202295_s_at | CTSH | cathepsin H | 2.37E-07 | -5.91287 |
| 223061_at | CHID1 | chitinase domain containing 1 | 1.39E-07 | -5.9142 |
| 202080_s_at | TRAK1 | trafficking protein, kinesin binding 1 | 7.30E-07 | -5.91447 |
| 227861_at | TMEM161B | transmembrane protein 161B | 1.18E-05 | -5.91506 |
| 218970_s_at | CUTC | cutC copper transporter homolog (E. coli) | 1.50E-05 | -5.91547 |
| 212791_at | C1orf216 | chromosome 1 open reading frame 216 | 1.76E-07 | -5.91723 |
| 227534_at | C9orf21 | chromosome 9 open reading frame 21 | 2.88E-06 | -5.91752 |
| 201624_at | DARS | aspartyl-tRNA synthetase | 5.74E-06 | -5.9181 |
| 208842_s_at | GORASP2 | golgi reassembly stacking protein 2, 55kDa | 3.64E-06 | -5.91848 |
| 221079_s_at | METTL2A /// METTL2B | methyltransferase like 2A /// methyltransferase like 2B | 3.01E-05 | -5.91896 |
| 212943_at | KIAA0528 | KIAA0528 | 3.89E-06 | -5.91969 |
| 217959_s_at | TRAPPC4 | trafficking protein particle complex 4 | 4.55E-09 | -5.92086 |
| 212773_s_at | TOMM20 | translocase of outer mitochondrial membrane 20 homolog (yeast) | 2.24E-06 | -5.92178 |
| 202758_s_at | RFXANK | regulatory factor X-associated ankyrin-containing protein | 4.21E-06 | -5.92325 |
| 200671_s_at | SPTBN1 | spectrin, beta, non-erythrocytic 1 | 1.34E-05 | -5.92367 |
| 213017_at | ABHD3 | abhydrolase domain containing 3 | 7.61E-06 | -5.92401 |
| 58696_at | EXOSC4 | exosome component 4 | 1.40E-08 | -5.92409 |
| 222431_at | SPIN1 | spindlin 1 | 5.15E-05 | -5.92527 |
| 229285_at | RNASEL | ribonuclease L (2',5'-oligoisoadenylate synthetase-dependent) | 4.10E-07 | -5.92581 |
| 218131_s_at | GATAD2A | GATA zinc finger domain containing 2A | 2.52E-06 | -5.9274 |
| 203815_at | GSTT1 | glutathione S-transferase theta 1 | 1.16E-05 | -5.9285 |
| 204028_s_at | RABGAP1 | RAB GTPase activating protein 1 | 5.48E-08 | -5.92954 |
| 201659_s_at | ARL1 | ADP-ribosylation factor-like 1 | 3.43E-09 | -5.93068 |
| 218208_at | PQLC1 | PQ loop repeat containing 1 | 6.04E-06 | -5.93442 |
| 212253_x_at | DST | dystonin | 8.56E-05 | -5.93515 |
| 235907_at | TMEM33 | transmembrane protein 33 | 2.20E-06 | -5.93602 |
| 236420_s_at | ANO4 | anoctamin 4 | 1.94E-05 | -5.9365 |
| 1552978_a_at | SCAMP1 | secretory carrier membrane protein 1 | 2.67E-05 | -5.93801 |
| 223221_at | SCO1 | SCO cytochrome oxidase deficient homolog 1 (yeast) | 1.96E-06 | -5.94144 |
| 224735_at | CYBASC3 | cytochrome b, ascorbate dependent 3 | 9.45E-08 | -5.94245 |
| 226688_at | C3orf23 | chromosome 3 open reading frame 23 | 1.03E-05 | -5.94423 |
| 226711_at | FOXN2 | forkhead box N2 | 3.42E-06 | -5.94566 |
| 224806_at | TRIM25 | tripartite motif-containing 25 | 9.12E-09 | -5.9457 |
| 203098_at | CDYL | chromodomain protein, Y-like | 0.000127 | -5.94574 |
| 37028_at | PPP1R15A | protein phosphatase 1, regulatory (inhibitor) subunit 15A | 7.20E-07 | -5.94585 |
| 217518_at | MYOF | myoferlin | 5.72E-05 | -5.94625 |
| 202251_at | PRPF3 | PRP3 pre-mRNA processing factor 3 homolog (S. cerevisiae) | 1.75E-07 | -5.9519 |
| 215832_x_at | PICALM | phosphatidylinositol binding clathrin assembly protein | 1.06E-07 | -5.95205 |
| 204566_at | PPM1D | protein phosphatase, Mg2+/Mn2+ dependent, 1D | 7.14E-06 | -5.95208 |
| 203748_x_at | RBMS1 | RNA binding motif, single stranded interacting protein 1 | 2.75E-08 | -5.95219 |
| 224689_at | MANBAL | mannosidase, beta A, lysosomal-like | 4.21E-07 | -5.95259 |
| 240717_at | ABCB5 | ATP-binding cassette, sub-family B (MDR/TAP), member 5 | 4.64E-07 | -5.9529 |
| 218472_s_at | PELO | pelota homolog (Drosophila) | 3.33E-06 | -5.95326 |
| 202541_at | AIMP1 | aminoacyl tRNA synthetase complex-interacting multifunctional protein 1 | 7.33E-08 | -5.95521 |
| 209129_at | TRIP6 | thyroid hormone receptor interactor 6 | 5.31E-08 | -5.956 |
| 37577_at | ARHGAP19 | Rho GTPase activating protein 19 | 1.07E-07 | -5.95841 |
| 203782_s_at | POLRMT | polymerase (RNA) mitochondrial (DNA directed) | 7.20E-06 | -5.95845 |
| 218037_at | FAM134A | family with sequence similarity 134, member A | 2.99E-05 | -5.95887 |
| 201311_s_at | SH3BGRL | SH3 domain binding glutamic acid-rich protein like | 8.82E-07 | -5.95946 |
| 204517_at | PPIC | peptidylprolyl isomerase C (cyclophilin C) | 7.78E-06 | -5.9597 |
| 211953_s_at | IPO5 | importin 5 | 1.63E-05 | -5.96072 |
| 233748_x_at | PRKAG2 | protein kinase, AMP-activated, gamma 2 non-catalytic subunit | 2.36E-06 | -5.96183 |
| 212421_at | C22orf9 | chromosome 22 open reading frame 9 | 7.56E-09 | -5.96242 |
| 224446_at | LLPH | LLP homolog, long-term synaptic facilitation (Aplysia) | 3.03E-06 | -5.96283 |
| 225386_s_at | HNRPLL | heterogeneous nuclear ribonucleoprotein L-like | 5.00E-08 | -5.96597 |
| 218093_s_at | ANKRD10 | ankyrin repeat domain 10 | 2.24E-06 | -5.96806 |
| 242617_at | TMED8 | Transmembrane emp24 protein transport domain containing 8 | 0.000667 | -5.96811 |
| 225049_at | BLOC1S2 | biogenesis of lysosomal organelles complex-1, subunit 2 | 7.60E-08 | -5.96844 |
| 44654_at | G6PC3 | glucose 6 phosphatase, catalytic, 3 | 1.21E-07 | -5.97164 |
| 212760_at | UBR2 | ubiquitin protein ligase E3 component n-recognin 2 | 7.73E-06 | -5.97164 |
| 202076_at | BIRC2 | baculoviral IAP repeat-containing 2 | 1.21E-06 | -5.97304 |
| 1558014_s_at | FAR1 | fatty acyl CoA reductase 1 | 1.18E-05 | -5.97309 |
| 200854_at | NCOR1 | nuclear receptor co-repressor 1 | 7.50E-08 | -5.97317 |
| 201546_at | TRIP12 | thyroid hormone receptor interactor 12 | 2.75E-07 | -5.97325 |
| 208813_at | GOT1 | glutamic-oxaloacetic transaminase 1, soluble (aspartate aminotransferase 1) | 1.61E-05 | -5.97402 |
| 211799_x_at | HLA-C | major histocompatibility complex, class I, C | 1.25E-06 | -5.97518 |
| 202697_at | NUDT21 | nudix (nucleoside diphosphate linked moiety X)-type motif 21 | 7.86E-05 | -5.97518 |
| 208297_s_at | EVI5 | ecotropic viral integration site 5 | 2.69E-05 | -5.97523 |
| 227087_at | INPP4A | inositol polyphosphate-4-phosphatase, type I, 107kDa | 7.28E-06 | -5.97991 |
| 215030_at | GRSF1 | G-rich RNA sequence binding factor 1 | 7.49E-05 | -5.98062 |
| 205434_s_at | AAK1 | AP2 associated kinase 1 | 4.75E-08 | -5.98095 |
| 206593_s_at | MED22 | mediator complex subunit 22 | 9.24E-07 | -5.98185 |
| 209090_s_at | SH3GLB1 | SH3-domain GRB2-like endophilin B1 | 1.00E-05 | -5.98406 |
| 223297_at | AMMECR1L | AMME chromosomal region gene 1-like | 6.15E-08 | -5.98446 |
| 227239_at | FAM126A | family with sequence similarity 126, member A | 5.08E-07 | -5.98461 |
| 36830_at | MIPEP | mitochondrial intermediate peptidase | 1.14E-07 | -5.9852 |
| 200683_s_at | UBE2L3 | ubiquitin-conjugating enzyme E2L 3 | 6.72E-07 | -5.98676 |
| 218499_at | MST4 | serine/threonine protein kinase MST4 | 2.01E-05 | -5.98682 |
| 214173_x_at | C19orf2 | chromosome 19 open reading frame 2 | 3.06E-06 | -5.98847 |
| 222414_at | MLL3 | myeloid/lymphoid or mixed-lineage leukemia 3 | 1.60E-05 | -5.98914 |
| 201523_x_at | UBE2N | ubiquitin-conjugating enzyme E2N (UBC13 homolog, yeast) | 2.15E-05 | -5.98939 |
| 223243_s_at | EDEM3 | ER degradation enhancer, mannosidase alpha-like 3 | 6.36E-06 | -5.99042 |
| 218738_s_at | RNF138 | ring finger protein 138 | 5.82E-06 | -5.99089 |
| 214626_s_at | GANAB | glucosidase, alpha; neutral AB | 1.97E-07 | -5.99262 |
| 219335_at | ARMCX5 | armadillo repeat containing, X-linked 5 | 1.23E-06 | -5.99318 |
| 228446_at | KIAA2026 | KIAA2026 | 2.36E-05 | -5.9941 |
| 217976_s_at | DYNC1LI1 | dynein, cytoplasmic 1, light intermediate chain 1 | 8.04E-05 | -5.99528 |
| 225846_at | ESRP1 | epithelial splicing regulatory protein 1 | 2.07E-06 | -5.99575 |
| 201712_s_at | RANBP2 | RAN binding protein 2 | 3.89E-05 | -5.99654 |
| 212179_at | SFRS18 | splicing factor, arginine/serine-rich 18 | 2.05E-06 | -5.99742 |
| 227784_s_at | COG1 | component of oligomeric golgi complex 1 | 3.65E-05 | -5.99859 |
| 200744_s_at | GNB1 | guanine nucleotide binding protein (G protein), beta polypeptide 1 | 3.91E-07 | -6.00319 |
| 221843_s_at | KIAA1609 | KIAA1609 | 2.30E-05 | -6.00562 |
| 218826_at | SLC35F2 | solute carrier family 35, member F2 | 1.13E-05 | -6.00566 |
| 1554441_a_at | WAPAL | wings apart-like homolog (Drosophila) | 5.86E-07 | -6.00792 |
| 222488_s_at | DCTN4 | dynactin 4 (p62) | 3.58E-06 | -6.0088 |
| 205565_s_at | FXN | frataxin | 2.80E-05 | -6.00968 |
| 218961_s_at | PNKP | polynucleotide kinase 3'-phosphatase | 1.74E-07 | -6.01085 |
| 218318_s_at | NLK | nemo-like kinase | 6.12E-07 | -6.01178 |
| 216591_s_at | SDHC | succinate dehydrogenase complex, subunit C, integral membrane protein, 15kDa | 1.57E-06 | -6.0122 |
| 1554021_a_at | ZNF12 | zinc finger protein 12 | 3.60E-06 | -6.01223 |
| 209375_at | XPC | xeroderma pigmentosum, complementation group C | 4.47E-06 | -6.01239 |
| 230226_s_at | KDM5A | lysine (K)-specific demethylase 5A | 3.79E-06 | -6.01672 |
| 211382_s_at | TACC2 | transforming, acidic coiled-coil containing protein 2 | 7.68E-07 | -6.0168 |
| 224366_s_at | REPS1 | RALBP1 associated Eps domain containing 1 | 1.06E-05 | -6.01822 |
| 1555831_s_at | LRRC41 | leucine rich repeat containing 41 | 4.14E-07 | -6.01853 |
| 214433_s_at | SELENBP1 | selenium binding protein 1 | 5.62E-06 | -6.01985 |
| 216598_s_at | CCL2 | chemokine (C-C motif) ligand 2 | 4.03E-06 | -6.02099 |
| 223675_s_at | VEZT | vezatin, adherens junctions transmembrane protein | 2.07E-06 | -6.02164 |
| 210266_s_at | TRIM33 | tripartite motif-containing 33 | 3.31E-07 | -6.02233 |
| 221472_at | SERINC3 | serine incorporator 3 | 5.14E-07 | -6.02234 |
| 219217_at | NARS2 | asparaginyl-tRNA synthetase 2, mitochondrial (putative) | 1.27E-07 | -6.02252 |
| 202417_at | KEAP1 | kelch-like ECH-associated protein 1 | 3.91E-07 | -6.02434 |
| 1555270_a_at | WFS1 | Wolfram syndrome 1 (wolframin) | 3.42E-05 | -6.02472 |
| 232652_x_at | SCAND1 | SCAN domain containing 1 | 7.50E-05 | -6.02515 |
| 209286_at | CDC42EP3 | CDC42 effector protein (Rho GTPase binding) 3 | 1.39E-06 | -6.02617 |
| 204320_at | COL11A1 | collagen, type XI, alpha 1 | 6.18E-08 | -6.02659 |
| 221589_s_at | ALDH6A1 | aldehyde dehydrogenase 6 family, member A1 | 0.00043 | -6.02971 |
| 211162_x_at | SCD | stearoyl-CoA desaturase (delta-9-desaturase) | 5.55E-06 | -6.03035 |
| 226753_at | FAM76B | family with sequence similarity 76, member B | 1.31E-06 | -6.03222 |
| 219966_x_at | BANP | BTG3 associated nuclear protein | 2.40E-05 | -6.03405 |
| 209222_s_at | OSBPL2 | oxysterol binding protein-like 2 | 5.06E-07 | -6.03442 |
| 207922_s_at | MAEA | macrophage erythroblast attacher | 4.36E-06 | -6.0353 |
| 227522_at | CMBL | carboxymethylenebutenolidase homolog (Pseudomonas) | 3.60E-06 | -6.03618 |
| 213473_at | BRAP | BRCA1 associated protein | 4.00E-08 | -6.03714 |
| 225452_at | MED1 | mediator complex subunit 1 | 1.18E-05 | -6.03728 |
| 211098_x_at | TMCO1 | transmembrane and coiled-coil domains 1 | 1.56E-06 | -6.03735 |
| 207186_s_at | BPTF | bromodomain PHD finger transcription factor | 5.90E-09 | -6.03893 |
| 218139_s_at | MUDENG | MU-2/AP1M2 domain containing, death-inducing | 1.90E-06 | -6.04066 |
| 210283_x_at | LOC645139 /// PAIP1 | similar to poly(A) binding protein interacting protein 1 /// poly(A) binding pro | 0.000155 | -6.04299 |
| 223396_at | TMEM60 | transmembrane protein 60 | 1.55E-06 | -6.04329 |
| 218604_at | LEMD3 | LEM domain containing 3 | 2.70E-07 | -6.04469 |
| 204557_s_at | DZIP1 | DAZ interacting protein 1 | 1.01E-05 | -6.04474 |
| 218516_s_at | IMPAD1 | inositol monophosphatase domain containing 1 | 2.09E-07 | -6.04767 |
| 212627_s_at | EXOSC7 | exosome component 7 | 1.43E-06 | -6.05129 |
| 202316_x_at | UBE4B | ubiquitination factor E4B (UFD2 homolog, yeast) | 1.26E-06 | -6.05432 |
| 227741_at | PTPLB | protein tyrosine phosphatase-like (proline instead of catalytic arginine), membe | 0.000125 | -6.05551 |
| 1564198_a_at | C10orf90 | chromosome 10 open reading frame 90 | 3.79E-06 | -6.05702 |
| 221751_at | PANK3 | pantothenate kinase 3 | 1.54E-06 | -6.05805 |
| 203732_at | TRIP4 | thyroid hormone receptor interactor 4 | 8.15E-09 | -6.05911 |
| 223065_s_at | STARD3NL | STARD3 N-terminal like | 8.26E-08 | -6.05996 |
| 52164_at | C11orf24 | chromosome 11 open reading frame 24 | 1.15E-07 | -6.0603 |
| 211000_s_at | IL6ST | interleukin 6 signal transducer (gp130, oncostatin M receptor) | 2.31E-05 | -6.06036 |
| 224981_at | TMEM219 | transmembrane protein 219 | 2.93E-06 | -6.06402 |
| 207358_x_at | MACF1 | microtubule-actin crosslinking factor 1 | 9.78E-06 | -6.06527 |
| 223299_at | SEC11C | SEC11 homolog C (S. cerevisiae) | 1.24E-08 | -6.06527 |
| 228571_at | RBAK | RB-associated KRAB zinc finger | 3.81E-07 | -6.06583 |
| 201456_s_at | BUB3 | budding uninhibited by benzimidazoles 3 homolog (yeast) | 5.80E-06 | -6.06624 |
| 226195_at | C14orf179 | chromosome 14 open reading frame 179 | 5.45E-07 | -6.06665 |
| 213021_at | GOSR1 | golgi SNAP receptor complex member 1 | 8.28E-06 | -6.06744 |
| 202270_at | GBP1 | guanylate binding protein 1, interferon-inducible, 67kDa | 9.03E-06 | -6.06928 |
| 223184_s_at | AGPAT3 | 1-acylglycerol-3-phosphate O-acyltransferase 3 | 5.54E-05 | -6.0693 |
| 217860_at | LOC732160 /// NDUFA10 | similar to NADH dehydrogenase (ubiquinone) 1 alpha subcomplex, 10, 42kDa precurs | 1.17E-05 | -6.06956 |
| 214290_s_at | HIST2H2AA3 /// HIST2H2AA4 | histone cluster 2, H2aa3 /// histone cluster 2, H2aa4 | 2.30E-05 | -6.07109 |
| 212829_at | PIP4K2A | phosphatidylinositol-5-phosphate 4-kinase, type II, alpha | 3.98E-07 | -6.07117 |
| 212133_at | CYFIP1 | Cytoplasmic FMR1 interacting protein 1 | 3.38E-07 | -6.07176 |
| 201772_at | AZIN1 | antizyme inhibitor 1 | 5.32E-06 | -6.07188 |
| 212240_s_at | PIK3R1 | phosphoinositide-3-kinase, regulatory subunit 1 (alpha) | 1.03E-06 | -6.07298 |
| 227036_at | RASAL2 | RAS protein activator like 2 | 3.35E-06 | -6.07389 |
| 200964_at | UBA1 | ubiquitin-like modifier activating enzyme 1 | 2.37E-06 | -6.07396 |
| 221268_s_at | SGPP1 | sphingosine-1-phosphate phosphatase 1 | 5.29E-07 | -6.07413 |
| 1558015_s_at | ACTR2 | ARP2 actin-related protein 2 homolog (yeast) | 2.62E-07 | -6.07448 |
| 211992_at | WNK1 | WNK lysine deficient protein kinase 1 | 4.25E-08 | -6.07726 |
| 222906_at | FLVCR1 | feline leukemia virus subgroup C cellular receptor 1 | 5.61E-06 | -6.07826 |
| 221530_s_at | BHLHE41 | basic helix-loop-helix family, member e41 | 1.20E-06 | -6.07842 |
| 1554037_a_at | ZBTB24 | zinc finger and BTB domain containing 24 | 6.13E-06 | -6.08043 |
| 202765_s_at | FBN1 | fibrillin 1 | 2.83E-05 | -6.08234 |
| 202060_at | CTR9 | Ctr9, Paf1/RNA polymerase II complex component, homolog (S. cerevisiae) | 4.06E-05 | -6.08378 |
| 1564063_a_at | ATP11B | ATPase, class VI, type 11B | 4.23E-06 | -6.08446 |
| 203759_at | ST3GAL4 | ST3 beta-galactoside alpha-2,3-sialyltransferase 4 | 1.11E-08 | -6.08476 |
| 218848_at | THOC6 | THO complex 6 homolog (Drosophila) | 7.73E-10 | -6.08501 |
| 235798_at | TMEM170B | transmembrane protein 170B | 4.78E-05 | -6.08626 |
| 218786_at | NT5DC3 | 5'-nucleotidase domain containing 3 | 4.49E-06 | -6.08755 |
| 238010_at | C1orf174 | chromosome 1 open reading frame 174 | 6.90E-06 | -6.08802 |
| 224918_x_at | MGST1 | microsomal glutathione S-transferase 1 | 1.49E-08 | -6.0904 |
| 223218_s_at | NFKBIZ | nuclear factor of kappa light polypeptide gene enhancer in B-cells inhibitor, ze | 7.55E-06 | -6.09237 |
| 202607_at | NDST1 | N-deacetylase/N-sulfotransferase (heparan glucosaminyl) 1 | 1.39E-05 | -6.09258 |
| 221572_s_at | SLC26A6 | solute carrier family 26, member 6 | 1.74E-05 | -6.09337 |
| 218889_at | NOC3L | nucleolar complex associated 3 homolog (S. cerevisiae) | 9.18E-05 | -6.09891 |
| 218013_x_at | DCTN4 | dynactin 4 (p62) | 1.09E-06 | -6.10276 |
| 225128_at | KDELC2 | KDEL (Lys-Asp-Glu-Leu) containing 2 | 8.58E-06 | -6.10582 |
| 1568957_x_at | SRGAP2P1 | SLIT-ROBO Rho GTPase activating protein 2 pseudogene 1 | 5.05E-07 | -6.10637 |
| 213198_at | ACVR1B | activin A receptor, type IB | 1.55E-07 | -6.10704 |
| 226194_at | ZNF828 | zinc finger protein 828 | 5.67E-08 | -6.10774 |
| 226285_at | CAPRIN1 | cell cycle associated protein 1 | 5.79E-06 | -6.11017 |
| 226229_s_at | SSU72 | SSU72 RNA polymerase II CTD phosphatase homolog (S. cerevisiae) | 1.57E-05 | -6.11081 |
| 225084_at | EXOC5 | exocyst complex component 5 | 4.26E-07 | -6.11114 |
| 201932_at | LRRC41 | leucine rich repeat containing 41 | 1.87E-06 | -6.11167 |
| 200666_s_at | DNAJB1 | DnaJ (Hsp40) homolog, subfamily B, member 1 | 2.73E-07 | -6.11431 |
| 212117_at | RHOQ | ras homolog gene family, member Q | 2.45E-08 | -6.11503 |
| 219212_at | HSPA14 | heat shock 70kDa protein 14 | 1.51E-05 | -6.11549 |
| 216194_s_at | TBCB | tubulin folding cofactor B | 3.05E-06 | -6.11595 |
| 208460_at | GJC1 | gap junction protein, gamma 1, 45kDa | 2.88E-08 | -6.1175 |
| 217990_at | GMPR2 | guanosine monophosphate reductase 2 | 0.000253 | -6.11792 |
| 227099_s_at | AG2 | protein Ag2 homolog | 2.88E-05 | -6.11798 |
| 218084_x_at | FXYD5 | FXYD domain containing ion transport regulator 5 | 9.54E-07 | -6.12073 |
| 223547_at | JKAMP | JNK1/MAPK8-associated membrane protein | 1.55E-05 | -6.12424 |
| 223430_at | SIK2 | salt-inducible kinase 2 | 4.39E-06 | -6.13087 |
| 201685_s_at | TOX4 | TOX high mobility group box family member 4 | 1.17E-05 | -6.13088 |
| 217980_s_at | MRPL16 | mitochondrial ribosomal protein L16 | 1.81E-05 | -6.13224 |
| 212060_at | SR140 | U2-associated SR140 protein | 4.23E-06 | -6.13435 |
| 200806_s_at | HSPD1 | heat shock 60kDa protein 1 (chaperonin) | 2.40E-05 | -6.1373 |
| 218277_s_at | DHX40 | DEAH (Asp-Glu-Ala-His) box polypeptide 40 | 2.37E-07 | -6.13799 |
| 220046_s_at | CCNL1 | cyclin L1 | 2.86E-07 | -6.13919 |
| 203279_at | EDEM1 | ER degradation enhancer, mannosidase alpha-like 1 | 1.69E-07 | -6.13976 |
| 225337_at | ABHD2 | abhydrolase domain containing 2 | 2.69E-06 | -6.14086 |
| 222713_s_at | FANCF | Fanconi anemia, complementation group F | 2.18E-08 | -6.14104 |
| 202379_s_at | NKTR | natural killer-tumor recognition sequence | 2.86E-07 | -6.14117 |
| 226302_at | ATP8B1 | ATPase, aminophospholipid transporter, class I, type 8B, member 1 | 3.42E-05 | -6.14166 |
| 207621_s_at | PEMT | phosphatidylethanolamine N-methyltransferase | 1.08E-07 | -6.14347 |
| 225776_at | RBMS2 | RNA binding motif, single stranded interacting protein 2 | 9.16E-06 | -6.14366 |
| 201164_s_at | PUM1 | pumilio homolog 1 (Drosophila) | 3.74E-07 | -6.14428 |
| 201488_x_at | KHDRBS1 | KH domain containing, RNA binding, signal transduction associated 1 | 3.28E-07 | -6.14539 |
| 227846_at | GPR176 | G protein-coupled receptor 176 | 8.15E-08 | -6.1456 |
| 202558_s_at | HSPA13 | heat shock protein 70kDa family, member 13 | 1.10E-05 | -6.14608 |
| 203787_at | SSBP2 | single-stranded DNA binding protein 2 | 3.35E-05 | -6.14629 |
| 209354_at | TNFRSF14 | tumor necrosis factor receptor superfamily, member 14 (herpesvirus entry mediato | 7.14E-07 | -6.14804 |
| 36499_at | CELSR2 | cadherin, EGF LAG seven-pass G-type receptor 2 (flamingo homolog, Drosophila) | 2.03E-06 | -6.15124 |
| 225200_at | DPH3 | DPH3, KTI11 homolog (S. cerevisiae) | 4.73E-07 | -6.15228 |
| 224962_at | C9orf69 | chromosome 9 open reading frame 69 | 6.97E-07 | -6.15298 |
| 226938_at | DCAF4 | DDB1 and CUL4 associated factor 4 | 1.85E-06 | -6.15302 |
| 225416_at | RLIM | ring finger protein, LIM domain interacting | 8.05E-07 | -6.15322 |
| 229225_at | NRP2 | neuropilin 2 | 2.61E-06 | -6.15365 |
| 210987_x_at | TPM1 | tropomyosin 1 (alpha) | 1.79E-07 | -6.15461 |
| 201222_s_at | RAD23B | RAD23 homolog B (S. cerevisiae) | 3.38E-06 | -6.15583 |
| 216100_s_at | TOR1AIP1 | torsin A interacting protein 1 | 0.000124 | -6.15822 |
| 238034_at | CANX | calnexin | 3.46E-06 | -6.15937 |
| 222862_s_at | AK5 | adenylate kinase 5 | 1.35E-05 | -6.15962 |
| 224130_s_at | SRA1 | steroid receptor RNA activator 1 | 4.47E-06 | -6.16052 |
| 228497_at | SLC22A15 | solute carrier family 22, member 15 | 9.51E-09 | -6.16134 |
| 47550_at | LZTS1 | leucine zipper, putative tumor suppressor 1 | 3.16E-07 | -6.16257 |
| 208984_x_at | RBM10 | RNA binding motif protein 10 | 2.40E-06 | -6.16289 |
| 208804_s_at | SFRS6 | splicing factor, arginine/serine-rich 6 | 3.36E-12 | -6.16388 |
| 219553_at | NME7 | non-metastatic cells 7, protein expressed in (nucleoside-diphosphate kinase) | 1.29E-06 | -6.16576 |
| 204512_at | HIVEP1 | human immunodeficiency virus type I enhancer binding protein 1 | 1.32E-05 | -6.16869 |
| 207941_s_at | RBM39 | RNA binding motif protein 39 | 3.85E-07 | -6.16906 |
| 226781_at | C7orf55 | chromosome 7 open reading frame 55 | 3.62E-07 | -6.16944 |
| 211615_s_at | LRPPRC | leucine-rich PPR-motif containing | 6.45E-08 | -6.16994 |
| 224280_s_at | FAM54B | family with sequence similarity 54, member B | 6.31E-06 | -6.17072 |
| 201674_s_at | AKAP1 | A kinase (PRKA) anchor protein 1 | 6.98E-07 | -6.17179 |
| 209600_s_at | ACOX1 | acyl-CoA oxidase 1, palmitoyl | 4.13E-05 | -6.17279 |
| 225926_at | VTI1B | vesicle transport through interaction with t-SNAREs homolog 1B (yeast) | 6.46E-06 | -6.17327 |
| 218809_at | PANK2 | pantothenate kinase 2 | 3.55E-07 | -6.17427 |
| 224687_at | ANKIB1 | ankyrin repeat and IBR domain containing 1 | 9.58E-06 | -6.17498 |
| 203067_at | PDHX | pyruvate dehydrogenase complex, component X | 1.68E-08 | -6.17523 |
| 223024_at | AP1M1 | adaptor-related protein complex 1, mu 1 subunit | 1.81E-07 | -6.17593 |
| 221479_s_at | BNIP3L | BCL2/adenovirus E1B 19kDa interacting protein 3-like | 2.01E-08 | -6.17826 |
| 204671_s_at | ANKRD6 | ankyrin repeat domain 6 | 4.96E-07 | -6.18117 |
| 204601_at | N4BP1 | NEDD4 binding protein 1 | 1.74E-07 | -6.18145 |
| 212467_at | DNAJC13 | DnaJ (Hsp40) homolog, subfamily C, member 13 | 4.98E-05 | -6.18241 |
| 225219_at | SMAD5 | SMAD family member 5 | 1.18E-05 | -6.18476 |
| 224812_at | HIBADH | 3-hydroxyisobutyrate dehydrogenase | 1.62E-07 | -6.18638 |
| 221311_x_at | LYRM2 | LYR motif containing 2 | 2.85E-06 | -6.18682 |
| 222744_s_at | TMLHE | trimethyllysine hydroxylase, epsilon | 2.54E-06 | -6.18793 |
| 228391_at | CYP4V2 | cytochrome P450, family 4, subfamily V, polypeptide 2 | 2.99E-06 | -6.18903 |
| 209212_s_at | KLF5 | Kruppel-like factor 5 (intestinal) | 1.17E-07 | -6.18911 |
| 202916_s_at | FAM20B | family with sequence similarity 20, member B | 2.84E-05 | -6.1893 |
| 217932_at | MRPS7 | mitochondrial ribosomal protein S7 | 2.83E-07 | -6.19 |
| 213995_at | ATP5S | ATP synthase, H+ transporting, mitochondrial F0 complex, subunit s (factor B) | 1.00E-06 | -6.19082 |
| 212434_at | GRPEL1 | GrpE-like 1, mitochondrial (E. coli) | 1.38E-05 | -6.19165 |
| 217185_s_at | ZNF259 /// ZNF259P1 | zinc finger protein 259 /// zinc finger protein 259 pseudogene 1 | 2.03E-05 | -6.19242 |
| 223446_s_at | DTNBP1 | dystrobrevin binding protein 1 | 6.82E-05 | -6.19266 |
| 201127_s_at | ACLY | ATP citrate lyase | 8.20E-07 | -6.19569 |
| 225949_at | NRBP2 | nuclear receptor binding protein 2 | 3.12E-05 | -6.19626 |
| 223413_s_at | LYAR | Ly1 antibody reactive homolog (mouse) | 3.13E-06 | -6.19698 |
| 228956_at | UGT8 | UDP glycosyltransferase 8 | 2.52E-06 | -6.19883 |
| 232129_s_at | LZTS2 | leucine zipper, putative tumor suppressor 2 | 1.57E-06 | -6.19934 |
| 202420_s_at | DHX9 | DEAH (Asp-Glu-Ala-His) box polypeptide 9 | 6.42E-05 | -6.20117 |
| 202846_s_at | PIGC | phosphatidylinositol glycan anchor biosynthesis, class C | 1.07E-06 | -6.20438 |
| 218118_s_at | LOC10431 /// TIMM23 | translocase of inner mitochondrial membrane 23 homolog (yeast)-like /// transloc | 8.88E-08 | -6.20541 |
| 210962_s_at | AKAP9 | A kinase (PRKA) anchor protein (yotiao) 9 | 2.22E-08 | -6.20545 |
| 202611_s_at | MED14 | mediator complex subunit 14 | 1.57E-05 | -6.20611 |
| 202429_s_at | PPP3CA | protein phosphatase 3, catalytic subunit, alpha isozyme | 9.84E-07 | -6.20806 |
| 212846_at | RRP1B | ribosomal RNA processing 1 homolog B (S. cerevisiae) | 1.41E-06 | -6.20987 |
| 225821_s_at | BOD1L | biorientation of chromosomes in cell division 1-like | 1.78E-07 | -6.2104 |
| 206042_x_at | SNRPN /// SNURF | small nuclear ribonucleoprotein polypeptide N /// SNRPN upstream reading frame | 4.96E-07 | -6.21133 |
| 204538_x_at | NPIP | nuclear pore complex interacting protein | 1.69E-08 | -6.21266 |
| 203647_s_at | FDX1 | ferredoxin 1 | 2.42E-07 | -6.21271 |
| 226895_at | NFIC | Nuclear factor I/C (CCAAT-binding transcription factor) | 1.38E-07 | -6.21392 |
| 203627_at | IGF1R | insulin-like growth factor 1 receptor | 1.40E-06 | -6.21405 |
| 218276_s_at | SAV1 | salvador homolog 1 (Drosophila) | 5.17E-06 | -6.21405 |
| 228670_at | TEP1 | telomerase-associated protein 1 | 7.62E-06 | -6.2152 |
| 218733_at | MSL2 | male-specific lethal 2 homolog (Drosophila) | 3.86E-07 | -6.21572 |
| 203360_s_at | MYCBP | c-myc binding protein | 3.68E-06 | -6.21582 |
| 227413_at | UBLCP1 | ubiquitin-like domain containing CTD phosphatase 1 | 7.58E-08 | -6.21755 |
| 200778_s_at | 2-九月 | septin 2 | 4.73E-07 | -6.21964 |
| 214755_at | UAP1L1 | UDP-N-acteylglucosamine pyrophosphorylase 1-like 1 | 8.46E-06 | -6.22113 |
| 221984_s_at | FAM134A | family with sequence similarity 134, member A | 6.52E-06 | -6.22122 |
| 205003_at | DOCK4 | dedicator of cytokinesis 4 | 6.39E-05 | -6.2214 |
| 205596_s_at | SMURF2 | SMAD specific E3 ubiquitin protein ligase 2 | 3.58E-06 | -6.22163 |
| 225243_s_at | SLMAP | sarcolemma associated protein | 3.65E-07 | -6.22261 |
| 227224_at | RALGPS2 | Ral GEF with PH domain and SH3 binding motif 2 | 1.01E-05 | -6.2239 |
| 204630_s_at | GOSR1 | golgi SNAP receptor complex member 1 | 5.72E-09 | -6.22418 |
| 214356_s_at | KIAA0368 | KIAA0368 | 2.02E-05 | -6.22506 |
| 226176_s_at | USP42 | ubiquitin specific peptidase 42 | 4.64E-06 | -6.22542 |
| 1554433_a_at | ZNF146 | zinc finger protein 146 | 1.20E-06 | -6.22554 |
| 219143_s_at | RPP25 | ribonuclease P/MRP 25kDa subunit | 3.38E-06 | -6.22646 |
| 203537_at | PRPSAP2 | phosphoribosyl pyrophosphate synthetase-associated protein 2 | 3.62E-06 | -6.22674 |
| 1552619_a_at | ANLN | anillin, actin binding protein | 1.37E-06 | -6.23055 |
| 209625_at | PIGH | phosphatidylinositol glycan anchor biosynthesis, class H | 1.53E-05 | -6.23064 |
| 209427_at | SMTN | smoothelin | 7.65E-06 | -6.23152 |
| 222035_s_at | PAPOLA | poly(A) polymerase alpha | 1.59E-06 | -6.23173 |
| 1554574_a_at | CYB5R3 | cytochrome b5 reductase 3 | 4.75E-05 | -6.23325 |
| 225150_s_at | RTKN | rhotekin | 4.23E-06 | -6.23387 |
| 228149_at | C7orf60 | chromosome 7 open reading frame 60 | 2.29E-07 | -6.2344 |
| 222163_s_at | SPATA5L1 | spermatogenesis associated 5-like 1 | 1.42E-05 | -6.23487 |
| 218071_s_at | MKRN2 | makorin ring finger protein 2 | 2.66E-07 | -6.23584 |
| 225534_at | C8orf40 | chromosome 8 open reading frame 40 | 3.11E-08 | -6.23672 |
| 222427_s_at | LARS | leucyl-tRNA synthetase | 2.71E-06 | -6.237 |
| 202535_at | FADD | Fas (TNFRSF6)-associated via death domain | 6.13E-07 | -6.23759 |
| 228496_s_at | CRIM1 | Cysteine rich transmembrane BMP regulator 1 (chordin-like) | 1.89E-05 | -6.24322 |
| 209390_at | TSC1 | tuberous sclerosis 1 | 3.66E-06 | -6.24436 |
| 226007_at | ISCA2 | iron-sulfur cluster assembly 2 homolog (S. cerevisiae) | 4.99E-07 | -6.24731 |
| 202478_at | TRIB2 | tribbles homolog 2 (Drosophila) | 1.88E-07 | -6.24806 |
| 226888_at | CSNK1G1 | casein kinase 1, gamma 1 | 4.90E-07 | -6.24967 |
| 212397_at | RDX | radixin | 5.71E-07 | -6.24973 |
| 212798_s_at | ANKMY2 | ankyrin repeat and MYND domain containing 2 | 1.64E-06 | -6.25047 |
| 203075_at | SMAD2 | SMAD family member 2 | 0.000101 | -6.25196 |
| 227930_at | EIF2C4 | Eukaryotic translation initiation factor 2C, 4 | 1.44E-05 | -6.25287 |
| 1565951_s_at | CHML | choroideremia-like (Rab escort protein 2) | 7.00E-08 | -6.2533 |
| 201216_at | ERP29 | endoplasmic reticulum protein 29 | 3.26E-06 | -6.25481 |
| 225888_at | NAA25 | N(alpha)-acetyltransferase 25, NatB auxiliary subunit | 1.73E-05 | -6.25614 |
| 226402_at | CYP2U1 | cytochrome P450, family 2, subfamily U, polypeptide 1 | 5.73E-05 | -6.25691 |
| 226393_at | CYP2U1 | cytochrome P450, family 2, subfamily U, polypeptide 1 | 7.27E-06 | -6.2588 |
| 202934_at | HK2 | hexokinase 2 | 7.07E-08 | -6.25883 |
| 222759_at | SUV420H1 | suppressor of variegation 4-20 homolog 1 (Drosophila) | 1.36E-06 | -6.25907 |
| 202402_s_at | CARS | cysteinyl-tRNA synthetase | 5.25E-06 | -6.25908 |
| 202985_s_at | BAG5 | BCL2-associated athanogene 5 | 3.39E-09 | -6.26209 |
| 233655_s_at | HAUS6 | HAUS augmin-like complex, subunit 6 | 4.89E-07 | -6.26492 |
| 212687_at | LIMS1 | LIM and senescent cell antigen-like domains 1 | 1.11E-05 | -6.26546 |
| 203429_s_at | C1orf9 | chromosome 1 open reading frame 9 | 1.08E-05 | -6.2674 |
| 211501_s_at | EIF3B | eukaryotic translation initiation factor 3, subunit B | 5.74E-07 | -6.26966 |
| 225695_at | C2orf18 | chromosome 2 open reading frame 18 | 1.29E-07 | -6.27102 |
| 216685_s_at | MTAP | methylthioadenosine phosphorylase | 2.96E-08 | -6.27161 |
| 213082_s_at | SLC35D2 | solute carrier family 35, member D2 | 6.46E-07 | -6.27322 |
| 211297_s_at | CDK7 | cyclin-dependent kinase 7 | 2.63E-08 | -6.27443 |
| 212034_s_at | EXOC7 | exocyst complex component 7 | 3.47E-07 | -6.27526 |
| 227833_s_at | MBD6 | methyl-CpG binding domain protein 6 | 1.15E-05 | -6.27645 |
| 1554555_a_at | SETD6 | SET domain containing 6 | 1.06E-06 | -6.27692 |
| 209501_at | CDR2 | cerebellar degeneration-related protein 2, 62kDa | 4.48E-05 | -6.27704 |
| 208643_s_at | XRCC5 | X-ray repair complementing defective repair in Chinese hamster cells 5 (double-s | 1.05E-08 | -6.27714 |
| 218973_at | EFTUD1 | elongation factor Tu GTP binding domain containing 1 | 4.41E-06 | -6.27793 |
| 219260_s_at | C17orf81 | chromosome 17 open reading frame 81 | 9.08E-08 | -6.27931 |
| 225900_at | EXOC6B | exocyst complex component 6B | 3.34E-06 | -6.27966 |
| 228453_at | KIAA1632 | KIAA1632 | 3.51E-06 | -6.28126 |
| 214020_x_at | ITGB5 | Integrin, beta 5 | 8.84E-06 | -6.28242 |
| 212919_at | DCP2 | DCP2 decapping enzyme homolog (S. cerevisiae) | 3.85E-07 | -6.28257 |
| 213097_s_at | DNAJC2 | DnaJ (Hsp40) homolog, subfamily C, member 2 | 1.04E-07 | -6.28275 |
| 201369_s_at | ZFP36L2 | zinc finger protein 36, C3H type-like 2 | 1.10E-05 | -6.28282 |
| 216044_x_at | FAM69A | family with sequence similarity 69, member A | 2.79E-06 | -6.28289 |
| 202160_at | CREBBP | CREB binding protein | 2.23E-06 | -6.28556 |
| 200920_s_at | BTG1 | B-cell translocation gene 1, anti-proliferative | 2.33E-06 | -6.28659 |
| 201037_at | PFKP | phosphofructokinase, platelet | 1.29E-06 | -6.28739 |
| 235432_at | NPHP3 | nephronophthisis 3 (adolescent) | 4.19E-06 | -6.28804 |
| 222585_x_at | KRCC1 | lysine-rich coiled-coil 1 | 8.16E-07 | -6.2892 |
| 222024_s_at | AKAP13 | A kinase (PRKA) anchor protein 13 | 5.90E-06 | -6.29099 |
| 231577_s_at | GBP1 | guanylate binding protein 1, interferon-inducible, 67kDa | 1.06E-05 | -6.29172 |
| 222476_at | CNOT6 | CCR4-NOT transcription complex, subunit 6 | 6.35E-08 | -6.29203 |
| 201377_at | UBAP2L | ubiquitin associated protein 2-like | 2.75E-05 | -6.29299 |
| 228745_at | SGTB | small glutamine-rich tetratricopeptide repeat (TPR)-containing, beta | 1.33E-05 | -6.29352 |
| 225232_at | MTMR12 | myotubularin related protein 12 | 3.57E-07 | -6.29447 |
| 229501_s_at | USP8 | ubiquitin specific peptidase 8 | 7.96E-07 | -6.29484 |
| 1554390_s_at | ACTR2 | ARP2 actin-related protein 2 homolog (yeast) | 1.78E-06 | -6.29487 |
| 211779_x_at | AP2A2 | adaptor-related protein complex 2, alpha 2 subunit | 8.40E-07 | -6.29508 |
| 219537_x_at | DLL3 | delta-like 3 (Drosophila) | 2.01E-07 | -6.29562 |
| 200828_s_at | ZNF207 | zinc finger protein 207 | 1.22E-05 | -6.29592 |
| 223068_at | EML4 | echinoderm microtubule associated protein like 4 | 2.79E-06 | -6.29594 |
| 219286_s_at | RBM15 | RNA binding motif protein 15 | 4.38E-09 | -6.29674 |
| 1558621_at | CABLES1 | Cdk5 and Abl enzyme substrate 1 | 1.40E-08 | -6.29776 |
| 201166_s_at | PUM1 | pumilio homolog 1 (Drosophila) | 8.59E-07 | -6.29799 |
| 200624_s_at | MATR3 | matrin 3 | 1.11E-06 | -6.29809 |
| 212856_at | GRAMD4 | GRAM domain containing 4 | 2.33E-06 | -6.29858 |
| 222611_s_at | PSPC1 | paraspeckle component 1 | 8.65E-06 | -6.29858 |
| 211160_x_at | ACTN1 | actinin, alpha 1 | 1.93E-06 | -6.29995 |
| 201513_at | TSN | translin | 3.24E-06 | -6.30078 |
| 36566_at | CTNS | cystinosis, nephropathic | 1.76E-07 | -6.30083 |
| 225912_at | TP53INP1 | tumor protein p53 inducible nuclear protein 1 | 2.81E-06 | -6.30198 |
| 207338_s_at | ZNF200 | zinc finger protein 200 | 1.05E-06 | -6.30243 |
| 217858_s_at | ARMCX3 | armadillo repeat containing, X-linked 3 | 2.42E-07 | -6.30347 |
| 201911_s_at | FARP1 | FERM, RhoGEF (ARHGEF) and pleckstrin domain protein 1 (chondrocyte-derived) | 1.71E-06 | -6.30374 |
| 218214_at | C12orf44 | chromosome 12 open reading frame 44 | 2.34E-07 | -6.30518 |
| 226127_at | ALKBH3 | alkB, alkylation repair homolog 3 (E. coli) | 4.66E-07 | -6.30906 |
| 206103_at | RAC3 | ras-related C3 botulinum toxin substrate 3 (rho family, small GTP binding protei | 4.30E-06 | -6.3092 |
| 205088_at | MAMLD1 | mastermind-like domain containing 1 | 4.82E-06 | -6.31236 |
| 218478_s_at | ZCCHC8 | zinc finger, CCHC domain containing 8 | 4.48E-05 | -6.31491 |
| 218616_at | INTS12 | integrator complex subunit 12 | 3.25E-06 | -6.31721 |
| 216221_s_at | PUM2 | pumilio homolog 2 (Drosophila) | 4.36E-06 | -6.31972 |
| 202827_s_at | MMP14 | matrix metallopeptidase 14 (membrane-inserted) | 3.04E-05 | -6.32059 |
| 203159_at | GLS | glutaminase | 1.18E-06 | -6.32129 |
| 221510_s_at | GLS | glutaminase | 4.19E-05 | -6.3238 |
| 220134_x_at | FAM176B | family with sequence similarity 176, member B | 2.18E-05 | -6.32398 |
| 200615_s_at | AP2B1 | adaptor-related protein complex 2, beta 1 subunit | 2.53E-09 | -6.324 |
| 220199_s_at | AIDA | axin interactor, dorsalization associated | 1.52E-06 | -6.32547 |
| 229603_at | BBS12 | Bardet-Biedl syndrome 12 | 5.35E-07 | -6.32549 |
| 224898_at | WDR26 | WD repeat domain 26 | 6.43E-06 | -6.32604 |
| 223163_s_at | ZC3HC1 | zinc finger, C3HC-type containing 1 | 2.32E-09 | -6.32617 |
| 221765_at | UGCG | UDP-glucose ceramide glucosyltransferase | 1.48E-06 | -6.3276 |
| 223092_at | ANKH | ankylosis, progressive homolog (mouse) | 8.86E-08 | -6.3281 |
| 224368_s_at | NDRG3 | NDRG family member 3 | 6.59E-07 | -6.32995 |
| 213292_s_at | SNX13 | sorting nexin 13 | 9.85E-08 | -6.33016 |
| 227415_at | LOC283508 | hypothetical protein LOC283508 | 5.91E-05 | -6.33123 |
| 226137_at | ZFHX3 | zinc finger homeobox 3 | 1.06E-06 | -6.33123 |
| 223982_s_at | PNPLA8 | patatin-like phospholipase domain containing 8 | 2.24E-05 | -6.33135 |
| 225735_at | ANKRD50 | ankyrin repeat domain 50 | 0.000142 | -6.33153 |
| 201120_s_at | PGRMC1 | progesterone receptor membrane component 1 | 3.53E-07 | -6.33443 |
| 226914_at | ARPC5L | actin related protein 2/3 complex, subunit 5-like | 2.04E-08 | -6.33485 |
| 218947_s_at | MTPAP | mitochondrial poly(A) polymerase | 3.25E-06 | -6.33539 |
| 221904_at | FAM131A | family with sequence similarity 131, member A | 6.93E-06 | -6.33643 |
| 218100_s_at | IFT57 | intraflagellar transport 57 homolog (Chlamydomonas) | 7.61E-06 | -6.33767 |
| 205282_at | LRP8 | low density lipoprotein receptor-related protein 8, apolipoprotein e receptor | 3.58E-07 | -6.33943 |
| 213035_at | ANKRD28 | ankyrin repeat domain 28 | 1.54E-08 | -6.34033 |
| 214494_s_at | SPG7 | spastic paraplegia 7 (pure and complicated autosomal recessive) | 8.16E-06 | -6.34249 |
| 202599_s_at | NRIP1 | nuclear receptor interacting protein 1 | 4.48E-07 | -6.34274 |
| 202727_s_at | IFNGR1 | interferon gamma receptor 1 | 1.83E-06 | -6.34547 |
| 214693_x_at | NBPF10 | neuroblastoma breakpoint family, member 10 | 5.31E-06 | -6.34558 |
| 210514_x_at | HLA-G | major histocompatibility complex, class I, G | 1.85E-07 | -6.3498 |
| 203938_s_at | TAF1C | TATA box binding protein (TBP)-associated factor, RNA polymerase I, C, 110kDa | 1.20E-06 | -6.35018 |
| 209006_s_at | C1orf63 | chromosome 1 open reading frame 63 | 6.28E-05 | -6.35029 |
| 227748_at | RBMXL1 | RNA binding motif protein, X-linked-like 1 | 8.24E-05 | -6.35044 |
| 203215_s_at | MYO6 | myosin VI | 6.87E-08 | -6.35087 |
| 218175_at | CCDC92 | coiled-coil domain containing 92 | 4.30E-06 | -6.35108 |
| 223250_at | KLHL7 | kelch-like 7 (Drosophila) | 1.08E-05 | -6.3531 |
| 225352_at | SEC62 | SEC62 homolog (S. cerevisiae) | 3.43E-05 | -6.35325 |
| 218206_x_at | SCAND1 | SCAN domain containing 1 | 2.26E-06 | -6.35365 |
| 212648_at | DHX29 | DEAH (Asp-Glu-Ala-His) box polypeptide 29 | 9.60E-06 | -6.35641 |
| 241364_at | TMEM57 | transmembrane protein 57 | 5.66E-06 | -6.36036 |
| 205535_s_at | PCDH7 | protocadherin 7 | 8.97E-06 | -6.36099 |
| 202537_s_at | CHMP2B | chromatin modifying protein 2B | 2.19E-06 | -6.36325 |
| 202753_at | PSMD6 | proteasome (prosome, macropain) 26S subunit, non-ATPase, 6 | 8.05E-09 | -6.36631 |
| 212804_s_at | GAPVD1 | GTPase activating protein and VPS9 domains 1 | 5.02E-08 | -6.36814 |
| 201888_s_at | IL13RA1 | interleukin 13 receptor, alpha 1 | 4.60E-06 | -6.37147 |
| 218486_at | KLF11 | Kruppel-like factor 11 | 0.000126 | -6.37152 |
| 202449_s_at | RXRA | retinoid X receptor, alpha | 1.01E-07 | -6.37166 |
| 202738_s_at | PHKB | phosphorylase kinase, beta | 5.07E-06 | -6.37228 |
| 227728_at | PPM1A | protein phosphatase, Mg2+/Mn2+ dependent, 1A | 7.00E-06 | -6.37328 |
| 209217_s_at | WDR45 | WD repeat domain 45 | 6.04E-06 | -6.37546 |
| 214782_at | CTTN | cortactin | 2.03E-06 | -6.37629 |
| 226515_at | CCDC127 | coiled-coil domain containing 127 | 3.90E-05 | -6.37677 |
| 205315_s_at | SNTB2 | syntrophin, beta 2 (dystrophin-associated protein A1, 59kDa, basic component 2) | 1.06E-09 | -6.37911 |
| 225771_at | AP1G1 | adaptor-related protein complex 1, gamma 1 subunit | 6.67E-07 | -6.3799 |
| 203530_s_at | STX4 | syntaxin 4 | 3.01E-07 | -6.38204 |
| 204256_at | ELOVL6 | ELOVL family member 6, elongation of long chain fatty acids (FEN1/Elo2, SUR4/Elo | 6.86E-05 | -6.38248 |
| 213526_s_at | LIN37 | lin-37 homolog (C. elegans) | 8.22E-06 | -6.38353 |
| 229144_at | KAZ | kazrin | 2.76E-08 | -6.38413 |
| 214435_x_at | RALA | v-ral simian leukemia viral oncogene homolog A (ras related) | 6.01E-05 | -6.38487 |
| 213743_at | CCNT2 | cyclin T2 | 2.66E-06 | -6.38525 |
| 210235_s_at | PPFIA1 | protein tyrosine phosphatase, receptor type, f polypeptide (PTPRF), interacting | 2.53E-07 | -6.38652 |
| 202353_s_at | PSMD12 | proteasome (prosome, macropain) 26S subunit, non-ATPase, 12 | 6.70E-08 | -6.38683 |
| 207390_s_at | SMTN | smoothelin | 5.09E-07 | -6.38778 |
| 224840_at | FKBP5 | FK506 binding protein 5 | 7.37E-08 | -6.39014 |
| 212066_s_at | USP34 | ubiquitin specific peptidase 34 | 1.80E-07 | -6.39171 |
| 227766_at | LIG4 | ligase IV, DNA, ATP-dependent | 3.99E-05 | -6.39282 |
| 228272_at | DNLZ | DNL-type zinc finger | 3.01E-06 | -6.3932 |
| 212255_s_at | ATP2C1 | ATPase, Ca++ transporting, type 2C, member 1 | 1.55E-05 | -6.39385 |
| 207435_s_at | SRRM2 | serine/arginine repetitive matrix 2 | 1.79E-08 | -6.39504 |
| 203865_s_at | ADARB1 | adenosine deaminase, RNA-specific, B1 (RED1 homolog rat) | 6.02E-08 | -6.39589 |
| 214709_s_at | KTN1 | kinectin 1 (kinesin receptor) | 1.93E-05 | -6.39647 |
| 217892_s_at | LIMA1 | LIM domain and actin binding 1 | 8.53E-09 | -6.39753 |
| 202342_s_at | TRIM2 | tripartite motif-containing 2 | 5.14E-06 | -6.39768 |
| 213461_at | NUDT21 | nudix (nucleoside diphosphate linked moiety X)-type motif 21 | 1.53E-05 | -6.3989 |
| 205652_s_at | TTLL1 | tubulin tyrosine ligase-like family, member 1 | 2.74E-06 | -6.40054 |
| 204366_s_at | GTF3C2 | general transcription factor IIIC, polypeptide 2, beta 110kDa | 1.17E-09 | -6.4015 |
| 1552277_a_at | C9orf30 | chromosome 9 open reading frame 30 | 4.44E-08 | -6.40279 |
| 228089_x_at | TMEM179B | transmembrane protein 179B | 6.82E-08 | -6.40387 |
| 228817_at | ALG9 | asparagine-linked glycosylation 9, alpha-1,2-mannosyltransferase homolog (S. cer | 7.26E-06 | -6.40389 |
| 218793_s_at | SCML1 | sex comb on midleg-like 1 (Drosophila) | 2.95E-05 | -6.40559 |
| 202900_s_at | NUP88 | nucleoporin 88kDa | 5.26E-08 | -6.40834 |
| 202949_s_at | FHL2 | four and a half LIM domains 2 | 1.05E-10 | -6.40881 |
| 201124_at | ITGB5 | integrin, beta 5 | 2.95E-07 | -6.40918 |
| 203005_at | LTBR | lymphotoxin beta receptor (TNFR superfamily, member 3) | 3.81E-05 | -6.40967 |
| 204306_s_at | CD151 | CD151 molecule (Raph blood group) | 9.93E-07 | -6.4101 |
| 224702_at | TMEM167A | transmembrane protein 167A | 3.99E-07 | -6.41022 |
| 225325_at | MFSD6 | major facilitator superfamily domain containing 6 | 9.82E-07 | -6.41191 |
| 55093_at | CHPF2 | chondroitin polymerizing factor 2 | 3.59E-06 | -6.4131 |
| 206247_at | MICB | MHC class I polypeptide-related sequence B | 2.87E-05 | -6.41403 |
| 225077_at | CHD2 | chromodomain helicase DNA binding protein 2 | 6.42E-07 | -6.41994 |
| 215093_at | NSDHL | NAD(P) dependent steroid dehydrogenase-like | 1.11E-05 | -6.42031 |
| 213571_s_at | EIF4E2 | eukaryotic translation initiation factor 4E family member 2 | 1.54E-06 | -6.42331 |
| 218247_s_at | MEX3C | mex-3 homolog C (C. elegans) | 1.69E-05 | -6.42358 |
| 221931_s_at | SEH1L | SEH1-like (S. cerevisiae) | 1.31E-07 | -6.424 |
| 213233_s_at | KLHL9 | kelch-like 9 (Drosophila) | 1.41E-06 | -6.4244 |
| 218057_x_at | COX4NB | COX4 neighbor | 6.11E-06 | -6.42618 |
| 217886_at | EPS15 | epidermal growth factor receptor pathway substrate 15 | 7.74E-08 | -6.42673 |
| 222582_at | PRKAG2 | protein kinase, AMP-activated, gamma 2 non-catalytic subunit | 7.14E-06 | -6.42693 |
| 208814_at | HSPA4 | Heat shock 70kDa protein 4 | 6.65E-05 | -6.42703 |
| 1555278_a_at | CKAP5 | cytoskeleton associated protein 5 | 2.00E-06 | -6.43019 |
| 228737_at | TOX2 | TOX high mobility group box family member 2 | 5.37E-06 | -6.43134 |
| 228829_at | ATF7 | activating transcription factor 7 | 2.33E-07 | -6.43268 |
| 200702_s_at | DDX24 | DEAD (Asp-Glu-Ala-Asp) box polypeptide 24 | 3.81E-06 | -6.43311 |
| 202874_s_at | ATP6V1C1 | ATPase, H+ transporting, lysosomal 42kDa, V1 subunit C1 | 2.84E-06 | -6.43535 |
| 202195_s_at | TMED5 | transmembrane emp24 protein transport domain containing 5 | 6.01E-06 | -6.4385 |
| 32094_at | CHST3 | carbohydrate (chondroitin 6) sulfotransferase 3 | 1.22E-08 | -6.43888 |
| 222138_s_at | WDR13 | WD repeat domain 13 | 2.02E-08 | -6.44027 |
| 202767_at | ACP2 | acid phosphatase 2, lysosomal | 3.11E-07 | -6.44124 |
| 227521_at | FBXO33 | F-box protein 33 | 1.84E-07 | -6.44246 |
| 209363_s_at | MED21 | mediator complex subunit 21 | 8.29E-08 | -6.44499 |
| 213070_at | PIK3C2A | phosphoinositide-3-kinase, class 2, alpha polypeptide | 1.69E-05 | -6.44541 |
| 227046_at | SLC39A11 | solute carrier family 39 (metal ion transporter), member 11 | 3.56E-08 | -6.44689 |
| 209345_s_at | PI4K2A | phosphatidylinositol 4-kinase type 2 alpha | 1.53E-07 | -6.44764 |
| 226225_at | MCC | mutated in colorectal cancers | 1.94E-06 | -6.45029 |
| 202976_s_at | RHOBTB3 | Rho-related BTB domain containing 3 | 5.69E-06 | -6.45497 |
| 200892_s_at | TRA2B | transformer 2 beta homolog (Drosophila) | 3.65E-07 | -6.45681 |
| 210285_x_at | WTAP | Wilms tumor 1 associated protein | 1.73E-06 | -6.45756 |
| 202735_at | EBP | emopamil binding protein (sterol isomerase) | 1.24E-05 | -6.45772 |
| 209797_at | CNPY2 | canopy 2 homolog (zebrafish) | 1.69E-07 | -6.45785 |
| 225710_at | GNB4 | guanine nucleotide binding protein (G protein), beta polypeptide 4 | 3.90E-07 | -6.45815 |
| 209845_at | MKRN1 | makorin ring finger protein 1 | 0.000142 | -6.45926 |
| 203090_at | SDF2 | stromal cell-derived factor 2 | 3.50E-06 | -6.45998 |
| 227250_at | KREMEN1 | kringle containing transmembrane protein 1 | 1.11E-05 | -6.46137 |
| 223145_s_at | AKIRIN2 | akirin 2 | 4.66E-09 | -6.46238 |
| 211749_s_at | VAMP3 | vesicle-associated membrane protein 3 (cellubrevin) | 1.88E-05 | -6.46267 |
| 221484_at | B4GALT5 | UDP-Gal:betaGlcNAc beta 1,4- galactosyltransferase, polypeptide 5 | 3.57E-07 | -6.46462 |
| 208093_s_at | NDEL1 | nudE nuclear distribution gene E homolog (A. nidulans)-like 1 | 7.26E-07 | -6.46554 |
| 201156_s_at | RAB5C | RAB5C, member RAS oncogene family | 3.08E-08 | -6.46757 |
| 226513_at | ASB7 | ankyrin repeat and SOCS box-containing 7 | 2.21E-07 | -6.47188 |
| 201455_s_at | NPEPPS | aminopeptidase puromycin sensitive | 6.16E-07 | -6.47326 |
| 226380_at | PTPN21 | protein tyrosine phosphatase, non-receptor type 21 | 8.47E-07 | -6.47395 |
| 218049_s_at | MRPL13 | mitochondrial ribosomal protein L13 | 5.02E-07 | -6.47491 |
| 224831_at | CPEB4 | cytoplasmic polyadenylation element binding protein 4 | 2.37E-05 | -6.4751 |
| 219349_s_at | EXOC2 | exocyst complex component 2 | 1.66E-05 | -6.47617 |
| 206685_at | HCG4 | HLA complex group 4 | 1.25E-05 | -6.47681 |
| 227016_at | ERICH1 | glutamate-rich 1 | 5.80E-06 | -6.47749 |
| 224873_s_at | MRPS25 | mitochondrial ribosomal protein S25 | 4.36E-06 | -6.47768 |
| 217897_at | FXYD6 | FXYD domain containing ion transport regulator 6 | 1.26E-06 | -6.48004 |
| 223186_at | TMEM189 | transmembrane protein 189 | 4.03E-07 | -6.48302 |
| 1552610_a_at | JAK1 | Janus kinase 1 | 7.62E-06 | -6.48304 |
| 227109_at | CYP2R1 | cytochrome P450, family 2, subfamily R, polypeptide 1 | 5.24E-05 | -6.48433 |
| 212692_s_at | LRBA | LPS-responsive vesicle trafficking, beach and anchor containing | 9.97E-06 | -6.48456 |
| 210502_s_at | PPIE | peptidylprolyl isomerase E (cyclophilin E) | 3.19E-08 | -6.48465 |
| 230875_s_at | ATP11A | ATPase, class VI, type 11A | 2.65E-05 | -6.48467 |
| 221041_s_at | SLC17A5 | solute carrier family 17 (anion/sugar transporter), member 5 | 9.34E-06 | -6.48488 |
| 222642_s_at | TMEM33 | transmembrane protein 33 | 2.66E-05 | -6.48505 |
| 224574_at | C17orf49 | chromosome 17 open reading frame 49 | 2.37E-06 | -6.48658 |
| 219024_at | PLEKHA1 | pleckstrin homology domain containing, family A (phosphoinositide binding specif | 1.06E-06 | -6.48975 |
| 212909_at | LYPD1 | LY6/PLAUR domain containing 1 | 2.04E-06 | -6.49123 |
| 201687_s_at | API5 | apoptosis inhibitor 5 | 3.44E-06 | -6.49167 |
| 200813_s_at | PAFAH1B1 | platelet-activating factor acetylhydrolase 1b, regulatory subunit 1 (45kDa) | 2.04E-07 | -6.49246 |
| 235489_at | RHOJ | ras homolog gene family, member J | 9.37E-05 | -6.493 |
| 202659_at | PSMB10 | proteasome (prosome, macropain) subunit, beta type, 10 | 1.67E-06 | -6.49365 |
| 212095_s_at | MTUS1 | microtubule associated tumor suppressor 1 | 0.000112 | -6.49377 |
| 203203_s_at | KRR1 | KRR1, small subunit (SSU) processome component, homolog (yeast) | 1.39E-05 | -6.49543 |
| 219351_at | TRAPPC2 | trafficking protein particle complex 2 | 1.24E-05 | -6.49942 |
| 229521_at | FLJ36031 | hypothetical protein FLJ36031 | 8.60E-06 | -6.50018 |
| 214305_s_at | SF3B1 | splicing factor 3b, subunit 1, 155kDa | 5.30E-06 | -6.50096 |
| 226490_at | NHSL1 | NHS-like 1 | 1.16E-07 | -6.50241 |
| 219940_s_at | PCID2 | PCI domain containing 2 | 3.63E-07 | -6.50672 |
| 225226_at | FAM40A | family with sequence similarity 40, member A | 1.84E-07 | -6.50889 |
| 209472_at | CCBL2 | cysteine conjugate-beta lyase 2 | 3.33E-05 | -6.51074 |
| 203501_at | PGCP | plasma glutamate carboxypeptidase | 4.57E-05 | -6.51168 |
| 210802_s_at | DIMT1L | DIM1 dimethyladenosine transferase 1-like (S. cerevisiae) | 4.68E-07 | -6.51392 |
| 227031_at | SNX13 | sorting nexin 13 | 1.31E-05 | -6.51513 |
| 203266_s_at | MAP2K4 | mitogen-activated protein kinase kinase 4 | 9.91E-05 | -6.5165 |
| 218924_s_at | CTBS | chitobiase, di-N-acetyl- | 8.34E-06 | -6.51678 |
| 213302_at | PFAS | phosphoribosylformylglycinamidine synthase | 1.73E-06 | -6.51771 |
| 218307_at | RSAD1 | radical S-adenosyl methionine domain containing 1 | 3.88E-05 | -6.51923 |
| 1557049_at | BTBD19 | BTB (POZ) domain containing 19 | 1.25E-06 | -6.51957 |
| 224829_at | CPEB4 | cytoplasmic polyadenylation element binding protein 4 | 2.65E-06 | -6.52123 |
| 203644_s_at | MON1B | MON1 homolog B (yeast) | 9.84E-07 | -6.52194 |
| 201831_s_at | USO1 | USO1 vesicle docking protein homolog (yeast) | 3.75E-06 | -6.52262 |
| 224764_at | ARHGAP21 | Rho GTPase activating protein 21 | 3.29E-06 | -6.52797 |
| 212083_at | TEX261 | testis expressed 261 | 2.30E-09 | -6.53043 |
| 223592_s_at | RNF135 | ring finger protein 135 | 4.51E-06 | -6.53086 |
| 227530_at | AKAP12 | A kinase (PRKA) anchor protein 12 | 5.26E-07 | -6.53282 |
| 200604_s_at | PRKAR1A | protein kinase, cAMP-dependent, regulatory, type I, alpha (tissue specific extin | 6.31E-07 | -6.53307 |
| 201704_at | ENTPD6 | ectonucleoside triphosphate diphosphohydrolase 6 (putative function) | 1.32E-06 | -6.53515 |
| 209069_s_at | H3F3B | H3 histone, family 3B (H3.3B) | 1.32E-05 | -6.53559 |
| 219390_at | FKBP14 | FK506 binding protein 14, 22 kDa | 2.45E-05 | -6.53584 |
| 1552664_at | FLCN | folliculin | 0.000218 | -6.5392 |
| 213606_s_at | ARHGDIA | Rho GDP dissociation inhibitor (GDI) alpha | 0.000235 | -6.54422 |
| 218829_s_at | CHD7 | chromodomain helicase DNA binding protein 7 | 3.28E-08 | -6.54493 |
| 202677_at | RASA1 | RAS p21 protein activator (GTPase activating protein) 1 | 2.82E-06 | -6.54562 |
| 229050_s_at | SNHG7 | small nucleolar RNA host gene 7 (non-protein coding) | 1.30E-06 | -6.54752 |
| 222620_s_at | DNAJC1 | DnaJ (Hsp40) homolog, subfamily C, member 1 | 5.38E-08 | -6.54812 |
| 226976_at | KPNA6 | karyopherin alpha 6 (importin alpha 7) | 2.08E-06 | -6.54835 |
| 202055_at | KPNA1 | karyopherin alpha 1 (importin alpha 5) | 5.71E-08 | -6.5489 |
| 212355_at | KHNYN | KH and NYN domain containing | 8.64E-10 | -6.55029 |
| 221814_at | GPR124 | G protein-coupled receptor 124 | 0.000138 | -6.55042 |
| 225099_at | FBXO45 | F-box protein 45 | 1.19E-08 | -6.55105 |
| 35436_at | GOLGA2 | golgin A2 | 2.91E-07 | -6.55281 |
| 207405_s_at | RAD17 | RAD17 homolog (S. pombe) | 4.99E-06 | -6.55367 |
| 220675_s_at | PNPLA3 | patatin-like phospholipase domain containing 3 | 8.37E-05 | -6.55422 |
| 202315_s_at | BCR | breakpoint cluster region | 2.65E-05 | -6.55498 |
| 224903_at | CIRH1A | cirrhosis, autosomal recessive 1A (cirhin) | 2.44E-07 | -6.55601 |
| 221552_at | ABHD6 | abhydrolase domain containing 6 | 1.47E-07 | -6.55655 |
| 217840_at | DDX41 | DEAD (Asp-Glu-Ala-Asp) box polypeptide 41 | 3.88E-05 | -6.55844 |
| 215338_s_at | NKTR | natural killer-tumor recognition sequence | 0.000206 | -6.55978 |
| 219648_at | MREG | melanoregulin | 3.47E-07 | -6.56016 |
| 212935_at | MCF2L | MCF.2 cell line derived transforming sequence-like | 4.07E-08 | -6.56031 |
| 227796_at | ZFP62 | zinc finger protein 62 homolog (mouse) | 1.66E-05 | -6.56044 |
| 221501_x_at | LOC339047 | hypothetical protein LOC339047 | 3.57E-09 | -6.5617 |
| 203799_at | CD302 | CD302 molecule | 0.000116 | -6.56315 |
| 200732_s_at | PTP4A1 | protein tyrosine phosphatase type IVA, member 1 | 9.34E-10 | -6.56446 |
| 218360_at | RAB22A | RAB22A, member RAS oncogene family | 1.69E-07 | -6.56496 |
| 229145_at | ANAPC16 | anaphase promoting complex subunit 16 | 3.25E-06 | -6.56561 |
| 221430_s_at | RNF146 | ring finger protein 146 | 0.000162 | -6.56582 |
| 223351_at | C17orf80 | chromosome 17 open reading frame 80 | 8.86E-07 | -6.56633 |
| 213581_at | PDCD2 | programmed cell death 2 | 1.56E-07 | -6.56727 |
| 218972_at | TTC17 | tetratricopeptide repeat domain 17 | 8.69E-07 | -6.57507 |
| 226425_at | CLIP4 | CAP-GLY domain containing linker protein family, member 4 | 4.99E-07 | -6.5753 |
| 224884_at | AKAP13 | A kinase (PRKA) anchor protein 13 | 1.56E-05 | -6.57542 |
| 200685_at | SFRS11 | splicing factor, arginine/serine-rich 11 | 1.04E-05 | -6.5778 |
| 212503_s_at | DIP2C | DIP2 disco-interacting protein 2 homolog C (Drosophila) | 4.10E-08 | -6.57821 |
| 1552611_a_at | JAK1 | Janus kinase 1 | 7.86E-07 | -6.57903 |
| 222158_s_at | PPPDE1 | PPPDE peptidase domain containing 1 | 1.33E-07 | -6.57932 |
| 228766_at | CD36 | CD36 molecule (thrombospondin receptor) | 2.88E-08 | -6.57989 |
| 212756_s_at | UBR2 | ubiquitin protein ligase E3 component n-recognin 2 | 4.41E-07 | -6.58108 |
| 201152_s_at | MBNL1 | muscleblind-like (Drosophila) | 4.52E-09 | -6.58145 |
| 220768_s_at | CSNK1G3 | casein kinase 1, gamma 3 | 2.65E-07 | -6.58179 |
| 226625_at | TGFBR3 | transforming growth factor, beta receptor III | 1.81E-05 | -6.58254 |
| 212595_s_at | DAZAP2 | DAZ associated protein 2 | 1.71E-06 | -6.5836 |
| 212890_at | SLC38A10 | solute carrier family 38, member 10 | 1.15E-08 | -6.58371 |
| 231715_s_at | PYCR2 | pyrroline-5-carboxylate reductase family, member 2 | 2.75E-06 | -6.58424 |
| 201604_s_at | PPP1R12A | protein phosphatase 1, regulatory (inhibitor) subunit 12A | 6.11E-07 | -6.58603 |
| 214004_s_at | VGLL4 | vestigial like 4 (Drosophila) | 5.07E-06 | -6.58675 |
| 213027_at | TROVE2 | TROVE domain family, member 2 | 3.85E-07 | -6.58774 |
| 208653_s_at | CD164 | CD164 molecule, sialomucin | 7.70E-07 | -6.58797 |
| 229572_at | ATP6V0A2 | ATPase, H+ transporting, lysosomal V0 subunit a2 | 1.63E-09 | -6.58798 |
| 205884_at | ITGA4 | integrin, alpha 4 (antigen CD49D, alpha 4 subunit of VLA-4 receptor) | 2.28E-06 | -6.5897 |
| 222467_s_at | SAPS3 | SAPS domain family, member 3 | 2.00E-07 | -6.5924 |
| 200676_s_at | UBE2L3 | ubiquitin-conjugating enzyme E2L 3 | 1.29E-06 | -6.59293 |
| 220940_at | ANKRD36B | ankyrin repeat domain 36B | 1.20E-06 | -6.5951 |
| 55692_at | ELMO2 | engulfment and cell motility 2 | 8.72E-08 | -6.5953 |
| 233587_s_at | SIPA1L2 | signal-induced proliferation-associated 1 like 2 | 8.95E-09 | -6.59712 |
| 218840_s_at | NADSYN1 | NAD synthetase 1 | 1.87E-06 | -6.59804 |
| 224717_s_at | C19orf42 | chromosome 19 open reading frame 42 | 1.52E-07 | -6.6006 |
| 212535_at | MEF2A | myocyte enhancer factor 2A | 9.19E-06 | -6.60099 |
| 224352_s_at | CFL2 | cofilin 2 (muscle) | 3.89E-07 | -6.60187 |
| 207714_s_at | SERPINH1 | serpin peptidase inhibitor, clade H (heat shock protein 47), member 1, (collagen | 1.69E-06 | -6.60245 |
| 209082_s_at | COL18A1 | collagen, type XVIII, alpha 1 | 5.92E-07 | -6.60329 |
| 211947_s_at | BAT2L2 | HLA-B associated transcript 2-like 2 | 5.49E-05 | -6.60406 |
| 225161_at | GFM1 | G elongation factor, mitochondrial 1 | 1.78E-08 | -6.6041 |
| 212057_at | KIAA0182 | KIAA0182 | 3.00E-05 | -6.6044 |
| 232693_s_at | FBXO16 /// ZNF395 | F-box protein 16 /// zinc finger protein 395 | 1.21E-06 | -6.60513 |
| 225251_at | RAB24 | RAB24, member RAS oncogene family | 3.01E-06 | -6.6058 |
| 205809_s_at | WASL | Wiskott-Aldrich syndrome-like | 1.79E-05 | -6.60733 |
| 209228_x_at | TUSC3 | tumor suppressor candidate 3 | 8.51E-08 | -6.60802 |
| 1556283_s_at | FGFR1OP2 | FGFR1 oncogene partner 2 | 3.68E-06 | -6.60846 |
| 242794_at | MAML3 | mastermind-like 3 (Drosophila) | 4.38E-06 | -6.60907 |
| 1555411_a_at | CCNL1 | cyclin L1 | 1.65E-07 | -6.60957 |
| 202911_at | MSH6 | mutS homolog 6 (E. coli) | 7.47E-10 | -6.61089 |
| 205893_at | NLGN1 | neuroligin 1 | 3.25E-06 | -6.6118 |
| 226290_at | BDP1 | B double prime 1, subunit of RNA polymerase III transcription initiation factor | 5.91E-06 | -6.61264 |
| 201594_s_at | PPP4R1 | protein phosphatase 4, regulatory subunit 1 | 1.12E-06 | -6.61343 |
| 200607_s_at | RAD21 | RAD21 homolog (S. pombe) | 1.18E-07 | -6.61484 |
| 211367_s_at | CASP1 | caspase 1, apoptosis-related cysteine peptidase (interleukin 1, beta, convertase | 1.99E-05 | -6.61513 |
| 221559_s_at | MIS12 | MIS12, MIND kinetochore complex component, homolog (S. pombe) | 1.21E-05 | -6.61696 |
| 226390_at | STARD4 | StAR-related lipid transfer (START) domain containing 4 | 6.88E-07 | -6.61854 |
| 221536_s_at | LSG1 | large subunit GTPase 1 homolog (S. cerevisiae) | 1.72E-06 | -6.61898 |
| 205690_s_at | BUD31 | BUD31 homolog (S. cerevisiae) | 9.79E-10 | -6.6201 |
| 205210_at | TGFBRAP1 | transforming growth factor, beta receptor associated protein 1 | 3.68E-08 | -6.62116 |
| 221381_s_at | MORF4 /// MORF4L1 | mortality factor 4 /// mortality factor 4 like 1 | 2.18E-05 | -6.62214 |
| 214600_at | TEAD1 | TEA domain family member 1 (SV40 transcriptional enhancer factor) | 5.98E-05 | -6.62216 |
| 204806_x_at | HLA-F | major histocompatibility complex, class I, F | 2.48E-09 | -6.62244 |
| 218669_at | RAP2C | RAP2C, member of RAS oncogene family | 1.47E-08 | -6.62499 |
| 212589_at | RRAS2 | related RAS viral (r-ras) oncogene homolog 2 | 6.31E-08 | -6.62552 |
| 218626_at | EIF4ENIF1 | eukaryotic translation initiation factor 4E nuclear import factor 1 | 3.21E-06 | -6.62688 |
| 218109_s_at | MFSD1 | major facilitator superfamily domain containing 1 | 4.35E-08 | -6.63045 |
| 223084_s_at | CCNDBP1 | cyclin D-type binding-protein 1 | 8.18E-06 | -6.63076 |
| 227518_at | SLC35E1 | solute carrier family 35, member E1 | 1.86E-06 | -6.63458 |
| 201075_s_at | SMARCC1 | SWI/SNF related, matrix associated, actin dependent regulator of chromatin, subf | 1.25E-06 | -6.6399 |
| 208737_at | ATP6V1G1 | ATPase, H+ transporting, lysosomal 13kDa, V1 subunit G1 | 1.65E-07 | -6.64001 |
| 224622_at | TBC1D14 | TBC1 domain family, member 14 | 2.81E-06 | -6.64134 |
| 222821_s_at | GEMIN7 | gem (nuclear organelle) associated protein 7 | 2.49E-06 | -6.64231 |
| 226917_s_at | ANAPC4 | anaphase promoting complex subunit 4 | 2.16E-07 | -6.64253 |
| 221548_s_at | ILKAP | integrin-linked kinase-associated serine/threonine phosphatase 2C | 1.09E-06 | -6.64453 |
| 208952_s_at | LARP4B | La ribonucleoprotein domain family, member 4B | 1.67E-06 | -6.64587 |
| 214071_at | GNAL | guanine nucleotide binding protein (G protein), alpha activating activity polype | 3.25E-05 | -6.64596 |
| 204032_at | BCAR3 | breast cancer anti-estrogen resistance 3 | 2.64E-06 | -6.64627 |
| 204799_at | ZBED4 | zinc finger, BED-type containing 4 | 4.93E-05 | -6.64663 |
| 215009_s_at | SEC31A | SEC31 homolog A (S. cerevisiae) | 5.57E-05 | -6.65007 |
| 238458_at | EFHA2 | EF-hand domain family, member A2 | 1.29E-05 | -6.65023 |
| 221511_x_at | CCPG1 | cell cycle progression 1 | 8.49E-07 | -6.65041 |
| 202082_s_at | SEC14L1 | SEC14-like 1 (S. cerevisiae) | 1.40E-06 | -6.65435 |
| 225241_at | CCDC80 | coiled-coil domain containing 80 | 2.18E-06 | -6.65489 |
| 219221_at | ZBTB38 | zinc finger and BTB domain containing 38 | 4.01E-07 | -6.65518 |
| 214152_at | CCPG1 | cell cycle progression 1 | 1.94E-06 | -6.65766 |
| 219978_s_at | NUSAP1 | nucleolar and spindle associated protein 1 | 9.66E-09 | -6.65925 |
| 241342_at | TMEM65 | transmembrane protein 65 | 8.99E-07 | -6.6593 |
| 208839_s_at | CAND1 | cullin-associated and neddylation-dissociated 1 | 1.00E-08 | -6.6619 |
| 225460_at | SEC22C | SEC22 vesicle trafficking protein homolog C (S. cerevisiae) | 6.25E-07 | -6.66207 |
| 222963_s_at | IL1RAPL1 | interleukin 1 receptor accessory protein-like 1 | 2.81E-06 | -6.66323 |
| 227083_at | B3GALTL | beta 1,3-galactosyltransferase-like | 3.99E-06 | -6.66328 |
| 223284_at | NAT14 | N-acetyltransferase 14 (GCN5-related, putative) | 1.87E-06 | -6.66336 |
| 208908_s_at | CAST | calpastatin | 7.85E-06 | -6.6635 |
| 202926_at | NBAS | neuroblastoma amplified sequence | 3.29E-06 | -6.66457 |
| 225213_at | PPTC7 | PTC7 protein phosphatase homolog (S. cerevisiae) | 6.47E-07 | -6.66598 |
| 244050_at | PTPLAD2 | protein tyrosine phosphatase-like A domain containing 2 | 2.42E-05 | -6.66848 |
| 216996_s_at | FASTKD2 | FAST kinase domains 2 | 1.05E-07 | -6.66868 |
| 202307_s_at | TAP1 | transporter 1, ATP-binding cassette, sub-family B (MDR/TAP) | 3.43E-07 | -6.67093 |
| 213145_at | FBXL14 | F-box and leucine-rich repeat protein 14 | 2.84E-08 | -6.67166 |
| 201537_s_at | DUSP3 | dual specificity phosphatase 3 | 3.86E-07 | -6.67233 |
| 218149_s_at | ZNF395 | zinc finger protein 395 | 2.57E-09 | -6.67241 |
| 221547_at | PRPF18 | PRP18 pre-mRNA processing factor 18 homolog (S. cerevisiae) | 1.58E-08 | -6.67337 |
| 225892_at | IREB2 | iron-responsive element binding protein 2 | 9.06E-06 | -6.67357 |
| 209920_at | BMPR2 | bone morphogenetic protein receptor, type II (serine/threonine kinase) | 1.33E-05 | -6.67365 |
| 225805_at | HNRNPU | heterogeneous nuclear ribonucleoprotein U (scaffold attachment factor A) | 1.16E-06 | -6.67639 |
| 208988_at | KDM2A | lysine (K)-specific demethylase 2A | 0.000166 | -6.67909 |
| 222726_s_at | EXOC5 | exocyst complex component 5 | 1.09E-06 | -6.67918 |
| 201291_s_at | TOP2A | topoisomerase (DNA) II alpha 170kDa | 4.19E-05 | -6.67938 |
| 224995_at | SPIRE1 | spire homolog 1 (Drosophila) | 2.11E-07 | -6.68072 |
| 235315_at | TSC22D1 | TSC22 domain family, member 1 | 5.14E-06 | -6.68213 |
| 211612_s_at | IL13RA1 | interleukin 13 receptor, alpha 1 | 5.57E-05 | -6.68248 |
| 205458_at | MC1R | melanocortin 1 receptor (alpha melanocyte stimulating hormone receptor) | 3.23E-06 | -6.68367 |
| 65588_at | LOC388796 | hypothetical LOC388796 | 6.89E-09 | -6.6837 |
| 216088_s_at | PSMA7 | proteasome (prosome, macropain) subunit, alpha type, 7 | 4.41E-05 | -6.68409 |
| 222580_at | ZNF644 | zinc finger protein 644 | 3.52E-07 | -6.68441 |
| 204164_at | SIPA1 | signal-induced proliferation-associated 1 | 6.83E-06 | -6.68446 |
| 228365_at | CPNE8 | copine VIII | 3.17E-05 | -6.69061 |
| 204809_at | CLPX | ClpX caseinolytic peptidase X homolog (E. coli) | 1.39E-05 | -6.69321 |
| 210667_s_at | C21orf33 | chromosome 21 open reading frame 33 | 2.56E-07 | -6.69592 |
| 201445_at | CNN3 | calponin 3, acidic | 4.55E-07 | -6.6961 |
| 202867_s_at | DNAJB12 | DnaJ (Hsp40) homolog, subfamily B, member 12 | 6.03E-06 | -6.6966 |
| 209485_s_at | OSBPL1A | oxysterol binding protein-like 1A | 1.97E-07 | -6.69677 |
| 223198_x_at | COMMD5 | COMM domain containing 5 | 2.91E-07 | -6.69873 |
| 213948_x_at | CADM3 | cell adhesion molecule 3 | 6.17E-06 | -6.69905 |
| 202565_s_at | SVIL | supervillin | 8.79E-08 | -6.70051 |
| 219312_s_at | ZBTB10 | zinc finger and BTB domain containing 10 | 4.85E-06 | -6.7029 |
| 221257_x_at | FBXO38 | F-box protein 38 | 1.88E-06 | -6.70371 |
| 223318_s_at | ALKBH7 | alkB, alkylation repair homolog 7 (E. coli) | 9.58E-05 | -6.70424 |
| 203065_s_at | CAV1 | caveolin 1, caveolae protein, 22kDa | 1.27E-07 | -6.70499 |
| 226750_at | LARP1B | La ribonucleoprotein domain family, member 1B | 1.96E-08 | -6.70606 |
| 210845_s_at | PLAUR | plasminogen activator, urokinase receptor | 5.09E-06 | -6.70632 |
| 218401_s_at | ZNF281 | zinc finger protein 281 | 3.46E-06 | -6.70813 |
| 225954_s_at | MIDN | midnolin | 2.35E-06 | -6.70935 |
| 201351_s_at | YME1L1 | YME1-like 1 (S. cerevisiae) | 3.77E-06 | -6.71317 |
| 221875_x_at | HLA-F | major histocompatibility complex, class I, F | 2.67E-07 | -6.71472 |
| 227626_at | PAQR8 | progestin and adipoQ receptor family member VIII | 9.97E-07 | -6.7154 |
| 217831_s_at | NSFL1C | NSFL1 (p97) cofactor (p47) | 2.43E-06 | -6.71675 |
| 204562_at | IRF4 | interferon regulatory factor 4 | 2.84E-07 | -6.71875 |
| 35147_at | MCF2L | MCF.2 cell line derived transforming sequence-like | 3.80E-07 | -6.71939 |
| 1557067_s_at | LUC7L | LUC7-like (S. cerevisiae) | 2.56E-05 | -6.72076 |
| 1552703_s_at | CARD16 /// CASP1 | caspase recruitment domain family, member 16 /// caspase 1, apoptosis-related cy | 0.000267 | -6.72116 |
| 206865_at | HRK | harakiri, BCL2 interacting protein (contains only BH3 domain) | 1.03E-06 | -6.72178 |
| 206488_s_at | CD36 | CD36 molecule (thrombospondin receptor) | 2.66E-07 | -6.72228 |
| 218768_at | NUP107 | nucleoporin 107kDa | 2.36E-05 | -6.72434 |
| 212556_at | SCRIB | scribbled homolog (Drosophila) | 2.68E-07 | -6.72713 |
| 226886_at | GFPT1 | glutamine--fructose-6-phosphate transaminase 1 | 2.02E-05 | -6.72777 |
| 226319_s_at | THOC4 | THO complex 4 | 6.45E-06 | -6.72832 |
| 218219_s_at | LANCL2 | LanC lantibiotic synthetase component C-like 2 (bacterial) | 2.49E-07 | -6.72989 |
| 204350_s_at | MED7 | mediator complex subunit 7 | 3.75E-06 | -6.73039 |
| 223263_s_at | FGFR1OP2 | FGFR1 oncogene partner 2 | 6.76E-05 | -6.73233 |
| 221235_s_at | LOC100288263 /// TGFBRAP1 | hypothetical protein LOC100288263 /// transforming growth factor, beta receptor | 1.58E-07 | -6.73275 |
| 221988_at | C19orf42 | chromosome 19 open reading frame 42 | 3.07E-05 | -6.734 |
| 202606_s_at | TLK1 | tousled-like kinase 1 | 2.78E-06 | -6.74177 |
| 221503_s_at | KPNA3 | karyopherin alpha 3 (importin alpha 4) | 1.32E-07 | -6.74248 |
| 209095_at | DLD | dihydrolipoamide dehydrogenase | 3.20E-07 | -6.74365 |
| 225160_x_at | MDM2 | Mdm2 p53 binding protein homolog (mouse) | 5.50E-06 | -6.74392 |
| 228928_x_at | BANP | BTG3 associated nuclear protein | 4.66E-07 | -6.74403 |
| 201153_s_at | MBNL1 | muscleblind-like (Drosophila) | 3.68E-06 | -6.74477 |
| 238653_at | LRIG2 | Leucine-rich repeats and immunoglobulin-like domains 2 | 3.63E-06 | -6.74563 |
| 215134_at | PI4K2A | phosphatidylinositol 4-kinase type 2 alpha | 8.92E-07 | -6.7462 |
| 218788_s_at | SMYD3 | SET and MYND domain containing 3 | 3.00E-07 | -6.74711 |
| 202747_s_at | ITM2A | integral membrane protein 2A | 3.92E-06 | -6.74818 |
| 203912_s_at | DNASE1L1 | deoxyribonuclease I-like 1 | 1.78E-05 | -6.74869 |
| 223328_at | ARMC10 | armadillo repeat containing 10 | 8.66E-09 | -6.75086 |
| 219147_s_at | C9orf95 | chromosome 9 open reading frame 95 | 0.000242 | -6.75098 |
| 204198_s_at | RUNX3 | runt-related transcription factor 3 | 2.39E-07 | -6.75182 |
| 231822_at | CTTNBP2NL | CTTNBP2 N-terminal like | 3.15E-06 | -6.75211 |
| 227150_at | MTF1 | metal-regulatory transcription factor 1 | 1.88E-08 | -6.75382 |
| 226670_s_at | PABPC1L | poly(A) binding protein, cytoplasmic 1-like | 3.71E-07 | -6.75506 |
| 33132_at | CPSF1 | cleavage and polyadenylation specific factor 1, 160kDa | 7.17E-07 | -6.75607 |
| 230192_at | TRIM13 | tripartite motif-containing 13 | 1.74E-08 | -6.75701 |
| 242463_x_at | ZNF600 | zinc finger protein 600 | 8.57E-06 | -6.75827 |
| 232341_x_at | HABP4 | hyaluronan binding protein 4 | 1.10E-08 | -6.76069 |
| 1557411_s_at | SLC25A43 | solute carrier family 25, member 43 | 3.12E-05 | -6.76191 |
| 222811_at | FTSJD1 | FtsJ methyltransferase domain containing 1 | 6.13E-06 | -6.76782 |
| 218108_at | UBR7 | ubiquitin protein ligase E3 component n-recognin 7 (putative) | 3.25E-07 | -6.76828 |
| 225302_at | TMX3 | thioredoxin-related transmembrane protein 3 | 1.08E-05 | -6.77046 |
| 209306_s_at | SWAP70 | SWAP switching B-cell complex 70kDa subunit | 1.36E-06 | -6.77084 |
| 216988_s_at | PTP4A2 | protein tyrosine phosphatase type IVA, member 2 | 4.45E-07 | -6.77302 |
| 228408_s_at | SDAD1 | SDA1 domain containing 1 | 2.38E-09 | -6.7733 |
| 201071_x_at | SF3B1 | splicing factor 3b, subunit 1, 155kDa | 9.86E-09 | -6.77531 |
| 201724_s_at | GALNT1 | UDP-N-acetyl-alpha-D-galactosamine:polypeptide N-acetylgalactosaminyltransferase | 1.74E-07 | -6.77536 |
| 217785_s_at | YKT6 | YKT6 v-SNARE homolog (S. cerevisiae) | 3.71E-06 | -6.77748 |
| 209020_at | C20orf111 | chromosome 20 open reading frame 111 | 6.04E-09 | -6.78057 |
| 201240_s_at | LOC653566 /// SPCS2 | signal peptidase complex subunit 2 homolog pseudogene /// signal peptidase compl | 8.19E-09 | -6.78077 |
| 204593_s_at | SMCR7L | Smith-Magenis syndrome chromosome region, candidate 7-like | 5.78E-07 | -6.78264 |
| 229450_at | IFIT3 | interferon-induced protein with tetratricopeptide repeats 3 | 4.10E-06 | -6.78277 |
| 219219_at | TMEM160 | transmembrane protein 160 | 5.26E-07 | -6.784 |
| 225593_at | LSM10 | LSM10, U7 small nuclear RNA associated | 1.53E-06 | -6.78475 |
| 213320_at | PRMT3 | protein arginine methyltransferase 3 | 8.60E-07 | -6.78568 |
| 201655_s_at | HSPG2 | heparan sulfate proteoglycan 2 | 8.87E-06 | -6.78706 |
| 225229_at | AFF4 | AF4/FMR2 family, member 4 | 0.000104 | -6.78728 |
| 219628_at | ZMAT3 | zinc finger, matrin type 3 | 1.01E-05 | -6.7913 |
| 224615_x_at | HM13 | histocompatibility (minor) 13 | 1.09E-07 | -6.79188 |
| 218052_s_at | ATP13A1 | ATPase type 13A1 | 8.29E-07 | -6.79222 |
| 224679_at | MESDC2 | mesoderm development candidate 2 | 2.77E-06 | -6.79274 |
| 222505_at | LMBR1 | limb region 1 homolog (mouse) | 1.21E-05 | -6.79474 |
| 222607_s_at | DIS3 | DIS3 mitotic control homolog (S. cerevisiae) | 2.46E-05 | -6.79676 |
| 202623_at | EAPP | E2F-associated phosphoprotein | 2.16E-05 | -6.79728 |
| 201517_at | NCBP2 | nuclear cap binding protein subunit 2, 20kDa | 6.44E-07 | -6.79761 |
| 223452_s_at | ATL3 | atlastin GTPase 3 | 5.53E-06 | -6.79881 |
| 227718_at | PURB | purine-rich element binding protein B | 2.64E-06 | -6.80054 |
| 214550_s_at | TNPO3 | transportin 3 | 1.18E-06 | -6.80163 |
| 222395_s_at | UBE2Z | ubiquitin-conjugating enzyme E2Z | 1.58E-06 | -6.80172 |
| 209091_s_at | SH3GLB1 | SH3-domain GRB2-like endophilin B1 | 8.42E-09 | -6.80267 |
| 227249_at | NDE1 | NudE nuclear distribution gene E homolog 1 (A. nidulans) | 4.43E-08 | -6.80577 |
| 226179_at | SLC25A37 | solute carrier family 25, member 37 | 0.000118 | -6.80775 |
| 215966_x_at | GK3P | glycerol kinase 3 pseudogene | 9.31E-06 | -6.80877 |
| 1563111_a_at | PIGX | phosphatidylinositol glycan anchor biosynthesis, class X | 3.45E-05 | -6.80974 |
| 204384_at | GOLGA2 | golgin A2 | 2.87E-06 | -6.81013 |
| 225526_at | MKLN1 | muskelin 1, intracellular mediator containing kelch motifs | 3.26E-08 | -6.8136 |
| 231166_at | GPR155 | G protein-coupled receptor 155 | 3.44E-06 | -6.81469 |
| 208800_at | SRP72 | signal recognition particle 72kDa | 6.68E-06 | -6.8151 |
| 221935_s_at | C3orf64 | chromosome 3 open reading frame 64 | 3.88E-06 | -6.81593 |
| 226109_at | C21orf91 | chromosome 21 open reading frame 91 | 1.76E-07 | -6.81782 |
| 216266_s_at | ARFGEF1 | ADP-ribosylation factor guanine nucleotide-exchange factor 1(brefeldin A-inhibit | 1.16E-06 | -6.81792 |
| 203404_at | ARMCX2 | armadillo repeat containing, X-linked 2 | 6.48E-05 | -6.81855 |
| 218438_s_at | MED28 | mediator complex subunit 28 | 3.54E-06 | -6.81997 |
| 215016_x_at | DST | dystonin | 1.31E-07 | -6.82071 |
| 212601_at | ZZEF1 | zinc finger, ZZ-type with EF-hand domain 1 | 9.18E-06 | -6.82095 |
| 211363_s_at | MTAP | methylthioadenosine phosphorylase | 5.61E-06 | -6.82229 |
| 201883_s_at | B4GALT1 | UDP-Gal:betaGlcNAc beta 1,4- galactosyltransferase, polypeptide 1 | 7.25E-07 | -6.82435 |
| 222621_at | DNAJC1 | DnaJ (Hsp40) homolog, subfamily C, member 1 | 1.54E-08 | -6.82517 |
| 219124_at | C8orf41 | chromosome 8 open reading frame 41 | 5.67E-07 | -6.82564 |
| 202308_at | SREBF1 | sterol regulatory element binding transcription factor 1 | 6.97E-07 | -6.82637 |
| 229428_at | TIMM23 | translocase of inner mitochondrial membrane 23 homolog (yeast) | 1.86E-05 | -6.82732 |
| 217356_s_at | PGK1 | phosphoglycerate kinase 1 | 6.07E-07 | -6.82977 |
| 222629_at | REV1 | REV1 homolog (S. cerevisiae) | 7.18E-06 | -6.83027 |
| 218950_at | ARAP3 | ArfGAP with RhoGAP domain, ankyrin repeat and PH domain 3 | 2.14E-06 | -6.83196 |
| 224814_at | DPP7 | dipeptidyl-peptidase 7 | 7.78E-07 | -6.83208 |
| 201475_x_at | MARS | methionyl-tRNA synthetase | 4.48E-08 | -6.83258 |
| 234726_s_at | TMEM168 | transmembrane protein 168 | 6.74E-05 | -6.83263 |
| 212274_at | LPIN1 | lipin 1 | 3.28E-07 | -6.83517 |
| 208840_s_at | G3BP2 | GTPase activating protein (SH3 domain) binding protein 2 | 4.76E-07 | -6.83719 |
| 206652_at | ZMYM5 | zinc finger, MYM-type 5 | 0.000123 | -6.83883 |
| 207740_s_at | NUP62 | nucleoporin 62kDa | 1.86E-06 | -6.8424 |
| 238538_at | ANKRD11 | ankyrin repeat domain 11 | 4.31E-06 | -6.84305 |
| 217911_s_at | BAG3 | BCL2-associated athanogene 3 | 1.65E-06 | -6.84454 |
| 201133_s_at | PJA2 | praja ring finger 2 | 4.22E-06 | -6.84459 |
| 201647_s_at | SCARB2 | scavenger receptor class B, member 2 | 1.32E-06 | -6.84552 |
| 223085_at | RNF19A | ring finger protein 19A | 2.82E-07 | -6.84653 |
| 222394_at | PDCD6IP | programmed cell death 6 interacting protein | 1.03E-07 | -6.84676 |
| 202984_s_at | BAG5 | BCL2-associated athanogene 5 | 4.82E-06 | -6.84877 |
| 219204_s_at | SRR | serine racemase | 1.86E-05 | -6.84971 |
| 212322_at | SGPL1 | sphingosine-1-phosphate lyase 1 | 6.91E-07 | -6.84994 |
| 225925_s_at | USP48 | ubiquitin specific peptidase 48 | 0.000119 | -6.85074 |
| 227562_at | MAPKSP1 | MAPK scaffold protein 1 | 9.20E-08 | -6.85377 |
| 224872_at | DIP2B | DIP2 disco-interacting protein 2 homolog B (Drosophila) | 5.73E-06 | -6.85444 |
| 217221_x_at | RBM10 | RNA binding motif protein 10 | 2.06E-08 | -6.8549 |
| 212690_at | DDHD2 | DDHD domain containing 2 | 1.20E-06 | -6.85496 |
| 221803_s_at | NRBF2 | nuclear receptor binding factor 2 | 8.17E-06 | -6.85591 |
| 202167_s_at | MMS19 | MMS19 nucleotide excision repair homolog (S. cerevisiae) | 6.59E-07 | -6.85665 |
| 218164_at | SPATA20 | spermatogenesis associated 20 | 6.08E-06 | -6.85899 |
| 221918_at | CDK17 | cyclin-dependent kinase 17 | 2.53E-05 | -6.86262 |
| 226092_at | MPP5 | membrane protein, palmitoylated 5 (MAGUK p55 subfamily member 5) | 2.18E-09 | -6.86546 |
| 202566_s_at | SVIL | supervillin | 4.11E-08 | -6.866 |
| 202214_s_at | CUL4B | cullin 4B | 9.15E-07 | -6.86634 |
| 201339_s_at | SCP2 | sterol carrier protein 2 | 1.23E-07 | -6.86893 |
| 208407_s_at | CTNND1 | catenin (cadherin-associated protein), delta 1 | 8.11E-09 | -6.86897 |
| 208290_s_at | EIF5 | eukaryotic translation initiation factor 5 | 2.95E-06 | -6.86993 |
| 209708_at | MOXD1 | monooxygenase, DBH-like 1 | 7.26E-06 | -6.87055 |
| 218705_s_at | SNX24 | sorting nexin 24 | 2.42E-07 | -6.87111 |
| 202570_s_at | DLGAP4 | discs, large (Drosophila) homolog-associated protein 4 | 1.34E-06 | -6.87191 |
| 223313_s_at | MAGED4 /// MAGED4B | melanoma antigen family D, 4 /// melanoma antigen family D, 4B | 2.17E-06 | -6.873 |
| 203415_at | PDCD6 | programmed cell death 6 | 1.64E-06 | -6.87345 |
| 203285_s_at | HS2ST1 | heparan sulfate 2-O-sulfotransferase 1 | 8.17E-05 | -6.87524 |
| 222832_s_at | MFF | mitochondrial fission factor | 1.92E-06 | -6.87627 |
| 226697_at | FAM114A1 | family with sequence similarity 114, member A1 | 4.00E-06 | -6.8785 |
| 203737_s_at | PPRC1 | peroxisome proliferator-activated receptor gamma, coactivator-related 1 | 5.01E-06 | -6.87897 |
| 1554510_s_at | GHITM | growth hormone inducible transmembrane protein | 4.33E-07 | -6.88044 |
| 209627_s_at | OSBPL3 | oxysterol binding protein-like 3 | 5.98E-06 | -6.88177 |
| 216867_s_at | PDGFA | platelet-derived growth factor alpha polypeptide | 1.90E-05 | -6.88517 |
| 213358_at | KIAA0802 | KIAA0802 | 2.97E-06 | -6.88998 |
| 203165_s_at | SLC33A1 | solute carrier family 33 (acetyl-CoA transporter), member 1 | 1.50E-05 | -6.89882 |
| 213417_at | TBX2 | T-box 2 | 2.82E-06 | -6.89884 |
| 209042_s_at | UBE2G2 | ubiquitin-conjugating enzyme E2G 2 (UBC7 homolog, yeast) | 2.52E-07 | -6.90048 |
| 200956_s_at | SSRP1 | structure specific recognition protein 1 | 6.88E-06 | -6.90094 |
| 202219_at | SLC6A8 | solute carrier family 6 (neurotransmitter transporter, creatine), member 8 | 1.38E-07 | -6.90255 |
| 226447_at | ASH1L | ash1 (absent, small, or homeotic)-like (Drosophila) | 1.39E-06 | -6.90334 |
| 225218_at | ZFYVE27 | zinc finger, FYVE domain containing 27 | 4.44E-06 | -6.90555 |
| 209305_s_at | GADD45B | growth arrest and DNA-damage-inducible, beta | 5.79E-06 | -6.90642 |
| 235347_at | LRCH3 | leucine-rich repeats and calponin homology (CH) domain containing 3 | 5.39E-06 | -6.90645 |
| 210983_s_at | MCM7 | minichromosome maintenance complex component 7 | 3.29E-05 | -6.91237 |
| 212506_at | PICALM | phosphatidylinositol binding clathrin assembly protein | 1.97E-06 | -6.91388 |
| 201359_at | COPB1 | coatomer protein complex, subunit beta 1 | 1.00E-05 | -6.91403 |
| 225486_at | ARID2 | AT rich interactive domain 2 (ARID, RFX-like) | 5.38E-08 | -6.9161 |
| 212446_s_at | LASS6 | LAG1 homolog, ceramide synthase 6 | 3.72E-06 | -6.91965 |
| 213490_s_at | MAP2K2 | mitogen-activated protein kinase kinase 2 | 7.82E-07 | -6.92116 |
| 202333_s_at | UBE2B | ubiquitin-conjugating enzyme E2B (RAD6 homolog) | 9.65E-08 | -6.92258 |
| 219373_at | DPM3 | dolichyl-phosphate mannosyltransferase polypeptide 3 | 1.99E-07 | -6.92336 |
| 32541_at | PPP3CC | protein phosphatase 3, catalytic subunit, gamma isozyme | 1.37E-05 | -6.92362 |
| 201620_at | MBTPS1 | membrane-bound transcription factor peptidase, site 1 | 1.42E-08 | -6.92414 |
| 208906_at | BSCL2 | Berardinelli-Seip congenital lipodystrophy 2 (seipin) | 6.61E-08 | -6.92506 |
| 201529_s_at | RPA1 | replication protein A1, 70kDa | 5.53E-06 | -6.92544 |
| 204724_s_at | COL9A3 | collagen, type IX, alpha 3 | 4.52E-06 | -6.92587 |
| 205324_s_at | FTSJ1 | FtsJ homolog 1 (E. coli) | 5.14E-08 | -6.92596 |
| 212364_at | MYO1B | myosin IB | 1.61E-05 | -6.92702 |
| 212398_at | RDX | radixin | 5.15E-06 | -6.92911 |
| 202740_at | ACY1 | aminoacylase 1 | 3.34E-07 | -6.92984 |
| 212870_at | SOS2 | son of sevenless homolog 2 (Drosophila) | 2.77E-06 | -6.93227 |
| 207782_s_at | PSEN1 | presenilin 1 | 1.31E-06 | -6.93238 |
| 203234_at | UPP1 | uridine phosphorylase 1 | 0.000304 | -6.93327 |
| 224902_at | PDPR | pyruvate dehydrogenase phosphatase regulatory subunit | 1.46E-06 | -6.93406 |
| 212025_s_at | FLII | flightless I homolog (Drosophila) | 1.80E-06 | -6.93469 |
| 219575_s_at | COG8 /// PDF | component of oligomeric golgi complex 8 /// peptide deformylase (mitochondrial) | 8.93E-07 | -6.93485 |
| 218641_at | C11orf95 | chromosome 11 open reading frame 95 | 6.87E-07 | -6.93929 |
| 225551_at | CNST | consortin, connexin sorting protein | 3.34E-06 | -6.94213 |
| 205191_at | RP2 | retinitis pigmentosa 2 (X-linked recessive) | 4.17E-05 | -6.94386 |
| 220239_at | KLHL7 | kelch-like 7 (Drosophila) | 8.08E-08 | -6.94482 |
| 213617_s_at | C18orf10 | chromosome 18 open reading frame 10 | 4.67E-08 | -6.94689 |
| 210613_s_at | SYNGR1 | synaptogyrin 1 | 1.71E-06 | -6.94721 |
| 1556551_s_at | SLC39A6 | solute carrier family 39 (zinc transporter), member 6 | 1.13E-05 | -6.94767 |
| 218518_at | FAM13B | family with sequence similarity 13, member B | 8.81E-08 | -6.94809 |
| 218606_at | ZDHHC7 | zinc finger, DHHC-type containing 7 | 2.31E-07 | -6.94932 |
| 202647_s_at | NRAS | neuroblastoma RAS viral (v-ras) oncogene homolog | 3.25E-07 | -6.95018 |
| 204568_at | KIAA0831 | KIAA0831 | 4.92E-06 | -6.95378 |
| 212563_at | BOP1 /// LOC727967 | block of proliferation 1 /// similar to block of proliferation 1 | 2.01E-08 | -6.95435 |
| 225972_at | TMEM64 | transmembrane protein 64 | 3.12E-05 | -6.95967 |
| 219329_s_at | C2orf28 | chromosome 2 open reading frame 28 | 1.75E-08 | -6.96105 |
| 222212_s_at | LASS2 | LAG1 homolog, ceramide synthase 2 | 5.40E-07 | -6.96171 |
| 211675_s_at | MDFIC | MyoD family inhibitor domain containing | 2.84E-06 | -6.96172 |
| 202670_at | MAP2K1 | mitogen-activated protein kinase kinase 1 | 3.27E-07 | -6.96279 |
| 214934_at | ATP9B | ATPase, class II, type 9B | 5.11E-06 | -6.96464 |
| 204944_at | PTPRG | protein tyrosine phosphatase, receptor type, G | 4.86E-06 | -6.9656 |
| 243752_s_at | CYTH3 | cytohesin 3 | 1.89E-06 | -6.9679 |
| 208934_s_at | LGALS8 | lectin, galactoside-binding, soluble, 8 | 2.78E-07 | -6.96794 |
| 1558066_s_at | TBC1D16 | TBC1 domain family, member 16 | 0.000195 | -6.96798 |
| 201188_s_at | ITPR3 | inositol 1,4,5-triphosphate receptor, type 3 | 1.04E-05 | -6.96807 |
| 224961_at | SCYL2 | SCY1-like 2 (S. cerevisiae) | 1.64E-05 | -6.96836 |
| 223492_s_at | LRRFIP1 | leucine rich repeat (in FLII) interacting protein 1 | 4.10E-06 | -6.96851 |
| 222459_at | AKIRIN1 | akirin 1 | 0.000179 | -6.96959 |
| 211352_s_at | NCOA3 | nuclear receptor coactivator 3 | 5.83E-06 | -6.97173 |
| 1553654_at | SYT14 | synaptotagmin XIV | 1.15E-06 | -6.97296 |
| 203690_at | TUBGCP3 | tubulin, gamma complex associated protein 3 | 1.85E-06 | -6.97755 |
| 227558_at | CBX4 | chromobox homolog 4 (Pc class homolog, Drosophila) | 3.30E-07 | -6.9803 |
| 222995_s_at | RHBDD2 | rhomboid domain containing 2 | 7.51E-07 | -6.98436 |
| 212460_at | C14orf147 | chromosome 14 open reading frame 147 | 7.24E-06 | -6.98789 |
| 225313_at | C20orf177 | chromosome 20 open reading frame 177 | 7.42E-06 | -6.98794 |
| 212723_at | JMJD6 | jumonji domain containing 6 | 3.48E-06 | -6.99306 |
| 202181_at | KIAA0247 | KIAA0247 | 4.05E-09 | -6.99631 |
| 212447_at | KBTBD2 | kelch repeat and BTB (POZ) domain containing 2 | 4.96E-08 | -6.99728 |
| 201675_at | AKAP1 | A kinase (PRKA) anchor protein 1 | 3.58E-07 | -6.99765 |
| 211979_at | GPR107 | G protein-coupled receptor 107 | 0.000113 | -6.99867 |
| 204925_at | CTNS | cystinosis, nephropathic | 1.45E-06 | -6.99975 |
| 237094_at | FAM19A5 | family with sequence similarity 19 (chemokine (C-C motif)-like), member A5 | 3.42E-06 | -7.0012 |
| 214620_x_at | PAM | peptidylglycine alpha-amidating monooxygenase | 3.89E-06 | -7.00557 |
| 221449_s_at | ITFG1 | integrin alpha FG-GAP repeat containing 1 | 1.77E-05 | -7.00787 |
| 209158_s_at | CYTH2 | cytohesin 2 | 4.47E-06 | -7.00869 |
| 206030_at | ASPA | aspartoacylase (Canavan disease) | 6.71E-07 | -7.00946 |
| 201951_at | ALCAM | activated leukocyte cell adhesion molecule | 1.51E-05 | -7.00972 |
| 212651_at | RHOBTB1 | Rho-related BTB domain containing 1 | 3.11E-05 | -7.01059 |
| 222593_s_at | SPATS2 | spermatogenesis associated, serine-rich 2 | 9.88E-08 | -7.01182 |
| 202956_at | ARFGEF1 | ADP-ribosylation factor guanine nucleotide-exchange factor 1(brefeldin A-inhibit | 5.21E-06 | -7.01504 |
| 211488_s_at | ITGB8 | integrin, beta 8 | 5.52E-06 | -7.01628 |
| 219526_at | C14orf169 | chromosome 14 open reading frame 169 | 3.45E-08 | -7.01681 |
| 210627_s_at | MOGS | mannosyl-oligosaccharide glucosidase | 6.60E-08 | -7.01909 |
| 203833_s_at | TGOLN2 | trans-golgi network protein 2 | 3.07E-06 | -7.01928 |
| 203298_s_at | JARID2 | jumonji, AT rich interactive domain 2 | 7.18E-07 | -7.01934 |
| 224858_at | ZDHHC5 | zinc finger, DHHC-type containing 5 | 2.28E-07 | -7.02114 |
| 218446_s_at | FAM18B | family with sequence similarity 18, member B | 5.81E-05 | -7.02217 |
| 203222_s_at | TLE1 | transducin-like enhancer of split 1 (E(sp1) homolog, Drosophila) | 3.07E-08 | -7.02747 |
| 209040_s_at | PSMB8 | proteasome (prosome, macropain) subunit, beta type, 8 (large multifunctional pep | 2.51E-08 | -7.02751 |
| 223197_s_at | SMARCAD1 | SWI/SNF-related, matrix-associated actin-dependent regulator of chromatin, subfa | 5.88E-06 | -7.03042 |
| 206875_s_at | SLK | STE20-like kinase (yeast) | 4.64E-06 | -7.03204 |
| 207116_s_at | GAPDHS | glyceraldehyde-3-phosphate dehydrogenase, spermatogenic | 2.60E-05 | -7.03224 |
| 209073_s_at | NUMB | numb homolog (Drosophila) | 5.91E-06 | -7.03326 |
| 226778_at | C8orf42 | chromosome 8 open reading frame 42 | 2.07E-06 | -7.0358 |
| 200811_at | CIRBP | cold inducible RNA binding protein | 1.33E-07 | -7.03656 |
| 212694_s_at | PCCB | propionyl CoA carboxylase, beta polypeptide | 4.92E-06 | -7.04201 |
| 210154_at | ME2 | malic enzyme 2, NAD(+)-dependent, mitochondrial | 1.80E-05 | -7.04602 |
| 216064_s_at | AGA | aspartylglucosaminidase | 1.51E-07 | -7.0522 |
| 222630_at | RFX7 | regulatory factor X, 7 | 2.02E-06 | -7.05282 |
| 37950_at | PREP | prolyl endopeptidase | 1.59E-06 | -7.05296 |
| 212448_at | NEDD4L | neural precursor cell expressed, developmentally down-regulated 4-like | 1.18E-07 | -7.05455 |
| 1554132_a_at | FAM190B | family with sequence similarity 190, member B | 1.49E-05 | -7.05457 |
| 220553_s_at | PRPF39 | PRP39 pre-mRNA processing factor 39 homolog (S. cerevisiae) | 2.33E-05 | -7.06056 |
| 203522_at | CCS | copper chaperone for superoxide dismutase | 5.82E-05 | -7.06328 |
| 222872_x_at | OBFC2A | oligonucleotide/oligosaccharide-binding fold containing 2A | 4.10E-06 | -7.06529 |
| 219073_s_at | OSBPL10 | oxysterol binding protein-like 10 | 3.25E-08 | -7.06613 |
| 229354_at | AHRR | aryl-hydrocarbon receptor repressor | 1.05E-06 | -7.06655 |
| 222244_s_at | TUG1 | taurine upregulated 1 (non-protein coding) | 1.51E-06 | -7.0672 |
| 201376_s_at | HNRNPF | heterogeneous nuclear ribonucleoprotein F | 2.64E-08 | -7.06859 |
| 202164_s_at | CNOT8 | CCR4-NOT transcription complex, subunit 8 | 6.70E-07 | -7.06983 |
| 221597_s_at | TMEM208 | transmembrane protein 208 | 4.23E-05 | -7.06991 |
| 208979_at | NCOA6 | nuclear receptor coactivator 6 | 3.56E-08 | -7.07215 |
| 227607_at | STAMBPL1 | STAM binding protein-like 1 | 1.34E-06 | -7.07216 |
| 222532_at | SRPRB | signal recognition particle receptor, B subunit | 2.27E-05 | -7.07736 |
| 239377_at | EIF1AD | eukaryotic translation initiation factor 1A domain containing | 3.51E-07 | -7.07874 |
| 202816_s_at | SS18 | synovial sarcoma translocation, chromosome 18 | 2.62E-08 | -7.08104 |
| 218376_s_at | MICAL1 | microtubule associated monoxygenase, calponin and LIM domain containing 1 | 4.21E-07 | -7.08159 |
| 223591_at | RNF135 | ring finger protein 135 | 1.01E-06 | -7.0841 |
| 220248_x_at | NSFL1C | NSFL1 (p97) cofactor (p47) | 4.16E-07 | -7.08491 |
| 207616_s_at | TANK | TRAF family member-associated NFKB activator | 6.85E-06 | -7.08837 |
| 200911_s_at | TACC1 | transforming, acidic coiled-coil containing protein 1 | 4.53E-07 | -7.08839 |
| 202414_at | ERCC5 | excision repair cross-complementing rodent repair deficiency, complementation gr | 1.96E-06 | -7.08891 |
| 200898_s_at | MGEA5 | meningioma expressed antigen 5 (hyaluronidase) | 9.78E-08 | -7.08926 |
| 221566_s_at | NOL3 | nucleolar protein 3 (apoptosis repressor with CARD domain) | 2.63E-07 | -7.08973 |
| 220329_s_at | RMND1 | required for meiotic nuclear division 1 homolog (S. cerevisiae) | 2.18E-06 | -7.09179 |
| 217738_at | NAMPT | nicotinamide phosphoribosyltransferase | 8.31E-07 | -7.09247 |
| 211061_s_at | MGAT2 | mannosyl (alpha-1,6-)-glycoprotein beta-1,2-N-acetylglucosaminyltransferase | 1.99E-06 | -7.09346 |
| 221471_at | SERINC3 | serine incorporator 3 | 1.28E-07 | -7.09367 |
| 229194_at | PCGF5 | polycomb group ring finger 5 | 4.15E-06 | -7.09399 |
| 209157_at | DNAJA2 | DnaJ (Hsp40) homolog, subfamily A, member 2 | 1.17E-06 | -7.09604 |
| 219460_s_at | TMEM127 | transmembrane protein 127 | 2.91E-06 | -7.10136 |
| 224492_s_at | ZNF627 | zinc finger protein 627 | 2.13E-06 | -7.10146 |
| 207877_s_at | NVL | nuclear VCP-like | 1.23E-08 | -7.10283 |
| 225903_at | PIGU | phosphatidylinositol glycan anchor biosynthesis, class U | 1.18E-06 | -7.10874 |
| 214937_x_at | PCM1 | pericentriolar material 1 | 2.62E-07 | -7.10897 |
| 213902_at | ASAH1 | N-acylsphingosine amidohydrolase (acid ceramidase) 1 | 3.50E-08 | -7.1094 |
| 224945_at | BTBD7 | BTB (POZ) domain containing 7 | 4.79E-07 | -7.10992 |
| 218748_s_at | EXOC5 | exocyst complex component 5 | 7.97E-07 | -7.11145 |
| 1558080_s_at | DNAJC3 | DnaJ (Hsp40) homolog, subfamily C, member 3 | 1.87E-05 | -7.11206 |
| 225658_at | SPOPL | speckle-type POZ protein-like | 3.77E-07 | -7.11215 |
| 222574_s_at | DHX40 | DEAH (Asp-Glu-Ala-His) box polypeptide 40 | 9.84E-08 | -7.11307 |
| 203176_s_at | TFAM | transcription factor A, mitochondrial | 5.23E-07 | -7.11948 |
| 200087_s_at | TMED2 | transmembrane emp24 domain trafficking protein 2 | 2.05E-08 | -7.12038 |
| 1554079_at | GALNTL4 | UDP-N-acetyl-alpha-D-galactosamine:polypeptide N-acetylgalactosaminyltransferase | 3.86E-07 | -7.12045 |
| 224570_s_at | IRF2BP2 | interferon regulatory factor 2 binding protein 2 | 1.34E-07 | -7.12231 |
| 235051_at | CCDC50 | coiled-coil domain containing 50 | 3.21E-06 | -7.12271 |
| 202581_at | HSPA1A /// HSPA1B | heat shock 70kDa protein 1A /// heat shock 70kDa protein 1B | 2.10E-07 | -7.12351 |
| 200998_s_at | CKAP4 | cytoskeleton-associated protein 4 | 1.49E-05 | -7.12358 |
| 202775_s_at | SFRS8 | splicing factor, arginine/serine-rich 8 (suppressor-of-white-apricot homolog, Dr | 9.35E-07 | -7.12407 |
| 205659_at | HDAC9 | histone deacetylase 9 | 2.56E-07 | -7.12455 |
| 210463_x_at | TRMT1 | TRM1 tRNA methyltransferase 1 homolog (S. cerevisiae) | 1.25E-06 | -7.12538 |
| 202536_at | CHMP2B | chromatin modifying protein 2B | 7.06E-06 | -7.1284 |
| 201603_at | PPP1R12A | protein phosphatase 1, regulatory (inhibitor) subunit 12A | 9.59E-07 | -7.12888 |
| 218348_s_at | ZC3H7A | zinc finger CCCH-type containing 7A | 2.96E-07 | -7.13073 |
| 224986_s_at | PDPK1 | 3-phosphoinositide dependent protein kinase-1 | 1.28E-06 | -7.13194 |
| 227372_s_at | BAIAP2L1 | BAI1-associated protein 2-like 1 | 5.86E-07 | -7.13566 |
| 204257_at | FADS3 | fatty acid desaturase 3 | 1.49E-07 | -7.13655 |
| 230538_at | SHC4 | SHC (Src homology 2 domain containing) family, member 4 | 3.73E-05 | -7.14032 |
| 222014_x_at | MTO1 | mitochondrial translation optimization 1 homolog (S. cerevisiae) | 0.000104 | -7.14064 |
| 224252_s_at | FXYD5 | FXYD domain containing ion transport regulator 5 | 2.14E-07 | -7.14073 |
| 203243_s_at | PDLIM5 | PDZ and LIM domain 5 | 6.93E-06 | -7.14153 |
| 218695_at | EXOSC4 | exosome component 4 | 4.09E-05 | -7.14435 |
| 212264_s_at | WAPAL | wings apart-like homolog (Drosophila) | 1.80E-06 | -7.14854 |
| 215945_s_at | TRIM2 | tripartite motif-containing 2 | 4.40E-07 | -7.15201 |
| 1554167_a_at | GOLGA7 | golgin A7 | 3.16E-07 | -7.15217 |
| 225566_at | NRP2 | neuropilin 2 | 1.12E-06 | -7.15361 |
| 214501_s_at | H2AFY | H2A histone family, member Y | 1.47E-08 | -7.15501 |
| 241763_s_at | FBXO32 | F-box protein 32 | 4.87E-06 | -7.15521 |
| 229908_s_at | UNKL | unkempt homolog (Drosophila)-like | 8.46E-06 | -7.15568 |
| 213408_s_at | PI4KA /// PI4KAP1 /// PI4KAP2 | phosphatidylinositol 4-kinase, catalytic, alpha /// phosphatidylinositol 4-kinas | 2.05E-07 | -7.15585 |
| 201965_s_at | SETX | senataxin | 2.94E-08 | -7.15666 |
| 226464_at | C3orf58 | chromosome 3 open reading frame 58 | 6.05E-07 | -7.15754 |
| 227080_at | ZNF697 | zinc finger protein 697 | 3.16E-06 | -7.15947 |
| 220484_at | MCOLN3 | mucolipin 3 | 4.19E-06 | -7.16081 |
| 201089_at | ATP6V1B2 | ATPase, H+ transporting, lysosomal 56/58kDa, V1 subunit B2 | 6.62E-07 | -7.16182 |
| 208089_s_at | TDRD3 | tudor domain containing 3 | 1.55E-09 | -7.16409 |
| 38918_at | SOX13 | SRY (sex determining region Y)-box 13 | 5.32E-06 | -7.16414 |
| 207856_s_at | LOC150776 /// SMPD4 | sphingomyelin phosphodiesterase 4, neutral membrane pseudogene /// sphingomyelin | 8.21E-08 | -7.16612 |
| 228584_at | SGCB | sarcoglycan, beta (43kDa dystrophin-associated glycoprotein) | 4.15E-05 | -7.16616 |
| 235413_at | GGCX | gamma-glutamyl carboxylase | 1.40E-06 | -7.16679 |
| 209748_at | SPAST | spastin | 3.57E-05 | -7.16887 |
| 202780_at | OXCT1 | 3-oxoacid CoA transferase 1 | 2.43E-06 | -7.16948 |
| 226145_s_at | FRAS1 | Fraser syndrome 1 | 3.70E-09 | -7.1734 |
| 224469_s_at | INF2 | inverted formin, FH2 and WH2 domain containing | 1.86E-06 | -7.17439 |
| 218611_at | IER5 | immediate early response 5 | 9.12E-07 | -7.17694 |
| 203775_at | SLC25A13 | solute carrier family 25, member 13 (citrin) | 1.94E-05 | -7.17958 |
| 225649_s_at | STK35 | serine/threonine kinase 35 | 1.54E-06 | -7.17962 |
| 200664_s_at | DNAJB1 | DnaJ (Hsp40) homolog, subfamily B, member 1 | 6.80E-06 | -7.18092 |
| 212235_at | PLXND1 | plexin D1 | 1.51E-07 | -7.18346 |
| 204094_s_at | TSC22D2 | TSC22 domain family, member 2 | 7.11E-07 | -7.18479 |
| 229017_s_at | DSTYK | dual serine/threonine and tyrosine protein kinase | 5.64E-07 | -7.18702 |
| 220014_at | PRR16 | proline rich 16 | 0.000254 | -7.18854 |
| 207000_s_at | PPP3CC | protein phosphatase 3, catalytic subunit, gamma isozyme | 7.04E-06 | -7.18977 |
| 222787_s_at | TMEM106B | transmembrane protein 106B | 9.14E-06 | -7.19165 |
| 230029_x_at | UBR3 | ubiquitin protein ligase E3 component n-recognin 3 (putative) | 3.09E-06 | -7.19394 |
| 220092_s_at | ANTXR1 | anthrax toxin receptor 1 | 5.90E-06 | -7.19529 |
| 225760_at | MYSM1 | Myb-like, SWIRM and MPN domains 1 | 3.07E-05 | -7.198 |
| 225741_at | THUMPD3 | THUMP domain containing 3 | 4.99E-06 | -7.19896 |
| 203353_s_at | MBD1 | methyl-CpG binding domain protein 1 | 5.43E-09 | -7.19905 |
| 235022_at | C18orf19 | chromosome 18 open reading frame 19 | 8.48E-07 | -7.20623 |
| 204084_s_at | CLN5 | ceroid-lipofuscinosis, neuronal 5 | 4.21E-07 | -7.20692 |
| 222523_at | SENP2 | SUMO1/sentrin/SMT3 specific peptidase 2 | 2.01E-09 | -7.20816 |
| 218282_at | EDEM2 | ER degradation enhancer, mannosidase alpha-like 2 | 4.84E-06 | -7.20934 |
| 217890_s_at | PARVA | parvin, alpha | 3.89E-07 | -7.21217 |
| 212406_s_at | PCMTD2 | protein-L-isoaspartate (D-aspartate) O-methyltransferase domain containing 2 | 7.16E-07 | -7.21602 |
| 238756_at | GAS2L3 | Growth arrest-specific 2 like 3 | 2.67E-06 | -7.22332 |
| 1553107_s_at | C5orf24 | chromosome 5 open reading frame 24 | 0.000177 | -7.22367 |
| 202422_s_at | ACSL4 | acyl-CoA synthetase long-chain family member 4 | 3.33E-05 | -7.22655 |
| 1560060_s_at | VPS37C | vacuolar protein sorting 37 homolog C (S. cerevisiae) | 2.53E-08 | -7.22737 |
| 224768_at | IWS1 | IWS1 homolog (S. cerevisiae) | 1.38E-06 | -7.22783 |
| 224281_s_at | NGRN | neugrin, neurite outgrowth associated | 5.58E-09 | -7.22949 |
| 228834_at | TOB1 | transducer of ERBB2, 1 | 6.92E-06 | -7.2295 |
| 205340_at | ZBTB24 | zinc finger and BTB domain containing 24 | 1.26E-10 | -7.23168 |
| 225703_at | FBRSL1 | fibrosin-like 1 | 2.42E-06 | -7.234 |
| 220189_s_at | MGAT4B | mannosyl (alpha-1,3-)-glycoprotein beta-1,4-N-acetylglucosaminyltransferase, iso | 1.19E-07 | -7.23495 |
| 227247_at | PLEKHA8 | Pleckstrin homology domain containing, family A (phosphoinositide binding specif | 8.72E-08 | -7.23516 |
| 221531_at | WDR61 | WD repeat domain 61 | 5.80E-06 | -7.23585 |
| 217909_s_at | MLX | MAX-like protein X | 5.21E-05 | -7.23656 |
| 206562_s_at | CSNK1A1 | casein kinase 1, alpha 1 | 1.15E-07 | -7.24149 |
| 1555578_at | PTPRM | protein tyrosine phosphatase, receptor type, M | 3.13E-06 | -7.2431 |
| 1556059_s_at | SPEN | spen homolog, transcriptional regulator (Drosophila) | 6.56E-07 | -7.24369 |
| 204700_x_at | C1orf107 | chromosome 1 open reading frame 107 | 6.38E-07 | -7.24674 |
| 219013_at | GALNT11 | UDP-N-acetyl-alpha-D-galactosamine:polypeptide N-acetylgalactosaminyltransferase | 1.78E-07 | -7.24731 |
| 201313_at | ENO2 | enolase 2 (gamma, neuronal) | 6.26E-06 | -7.24754 |
| 209130_at | SNAP23 | synaptosomal-associated protein, 23kDa | 3.88E-06 | -7.25424 |
| 209292_at | ID4 | Inhibitor of DNA binding 4, dominant negative helix-loop-helix protein | 2.15E-05 | -7.25483 |
| 218375_at | NUDT9 | nudix (nucleoside diphosphate linked moiety X)-type motif 9 | 3.51E-06 | -7.25524 |
| 227133_at | FAM199X | family with sequence similarity 199, X-linked | 1.09E-05 | -7.25968 |
| 219546_at | BMP2K | BMP2 inducible kinase | 5.72E-06 | -7.26038 |
| 209717_at | EVI5 | ecotropic viral integration site 5 | 9.95E-06 | -7.26189 |
| 223077_at | TMOD3 | tropomodulin 3 (ubiquitous) | 2.43E-05 | -7.26215 |
| 201521_s_at | NCBP2 | nuclear cap binding protein subunit 2, 20kDa | 1.01E-06 | -7.26225 |
| 222874_s_at | CLN8 | ceroid-lipofuscinosis, neuronal 8 (epilepsy, progressive with mental retardation | 1.06E-07 | -7.26245 |
| 212230_at | PPAP2B | phosphatidic acid phosphatase type 2B | 1.41E-06 | -7.26383 |
| 225729_at | C6orf89 | chromosome 6 open reading frame 89 | 2.22E-05 | -7.26551 |
| 217997_at | PHLDA1 | pleckstrin homology-like domain, family A, member 1 | 3.17E-08 | -7.26641 |
| 201098_at | COPB2 | coatomer protein complex, subunit beta 2 (beta prime) | 1.29E-10 | -7.26903 |
| 209884_s_at | SLC4A7 | solute carrier family 4, sodium bicarbonate cotransporter, member 7 | 7.19E-07 | -7.27016 |
| 209656_s_at | TMEM47 | transmembrane protein 47 | 1.03E-06 | -7.27397 |
| 201952_at | ALCAM | activated leukocyte cell adhesion molecule | 3.96E-06 | -7.27431 |
| 224791_at | ASAP1 | ArfGAP with SH3 domain, ankyrin repeat and PH domain 1 | 2.41E-07 | -7.27459 |
| 220892_s_at | PSAT1 | phosphoserine aminotransferase 1 | 8.80E-06 | -7.27471 |
| 216602_s_at | FARSA | phenylalanyl-tRNA synthetase, alpha subunit | 5.51E-07 | -7.2754 |
| 223335_at | TMEM69 | transmembrane protein 69 | 3.58E-06 | -7.27734 |
| 209404_s_at | TMED7 | transmembrane emp24 protein transport domain containing 7 | 2.02E-05 | -7.27839 |
| 218292_s_at | PRKAG2 | protein kinase, AMP-activated, gamma 2 non-catalytic subunit | 5.79E-05 | -7.27903 |
| 218197_s_at | OXR1 | oxidation resistance 1 | 7.94E-06 | -7.28329 |
| 226650_at | ZFAND2A | zinc finger, AN1-type domain 2A | 5.14E-09 | -7.28353 |
| 209061_at | NCOA3 | nuclear receptor coactivator 3 | 7.64E-06 | -7.28444 |
| 213501_at | ACOX1 | acyl-CoA oxidase 1, palmitoyl | 5.40E-06 | -7.28522 |
| 214657_s_at | NEAT1 | nuclear paraspeckle assembly transcript 1 (non-protein coding) | 1.70E-05 | -7.28906 |
| 210541_s_at | TRIM27 | tripartite motif-containing 27 | 3.06E-07 | -7.2914 |
| 225522_at | AAK1 | AP2 associated kinase 1 | 4.92E-07 | -7.2925 |
| 206560_s_at | MIA | melanoma inhibitory activity | 1.38E-07 | -7.29275 |
| 208884_s_at | UBR5 | ubiquitin protein ligase E3 component n-recognin 5 | 7.54E-07 | -7.29435 |
| 226982_at | ELL2 | elongation factor, RNA polymerase II, 2 | 7.05E-05 | -7.29468 |
| 212199_at | MRFAP1L1 | Morf4 family associated protein 1-like 1 | 6.17E-07 | -7.3001 |
| 217826_s_at | UBE2J1 | ubiquitin-conjugating enzyme E2, J1 (UBC6 homolog, yeast) | 1.18E-07 | -7.3004 |
| 217965_s_at | SAP30BP | SAP30 binding protein | 1.00E-06 | -7.30098 |
| 224162_s_at | FBXO31 | F-box protein 31 | 9.98E-07 | -7.30303 |
| 205527_s_at | GEMIN4 | gem (nuclear organelle) associated protein 4 | 8.26E-05 | -7.3031 |
| 225857_s_at | LOC388796 | hypothetical LOC388796 | 1.12E-06 | -7.30761 |
| 221036_s_at | APH1B | anterior pharynx defective 1 homolog B (C. elegans) | 2.75E-06 | -7.31053 |
| 41037_at | TEAD4 | TEA domain family member 4 | 2.05E-08 | -7.31098 |
| 202551_s_at | CRIM1 | cysteine rich transmembrane BMP regulator 1 (chordin-like) | 7.98E-07 | -7.31107 |
| 222415_at | MLL3 | myeloid/lymphoid or mixed-lineage leukemia 3 | 2.42E-05 | -7.31178 |
| 201924_at | AFF1 | AF4/FMR2 family, member 1 | 8.23E-07 | -7.31228 |
| 212367_at | FEM1B | fem-1 homolog b (C. elegans) | 1.54E-06 | -7.3128 |
| 200767_s_at | FAM120A | family with sequence similarity 120A | 3.73E-06 | -7.31517 |
| 222392_x_at | PERP | PERP, TP53 apoptosis effector | 1.71E-07 | -7.31601 |
| 233929_x_at | WASH3P | WAS protein family homolog 3 pseudogene | 1.01E-07 | -7.31626 |
| 209388_at | PAPOLA | poly(A) polymerase alpha | 2.15E-05 | -7.31729 |
| 234294_x_at | GATAD2A | GATA zinc finger domain containing 2A | 4.56E-07 | -7.31925 |
| 218605_at | TFB2M | transcription factor B2, mitochondrial | 2.02E-05 | -7.32043 |
| 220773_s_at | GPHN | gephyrin | 1.23E-05 | -7.32048 |
| 227577_at | EXOC8 | exocyst complex component 8 | 4.00E-06 | -7.32068 |
| 235593_at | ZEB2 | zinc finger E-box binding homeobox 2 | 4.63E-06 | -7.32068 |
| 228135_at | C1orf52 | chromosome 1 open reading frame 52 | 2.60E-07 | -7.32308 |
| 212877_at | KLC1 | kinesin light chain 1 | 5.95E-06 | -7.32354 |
| 216032_s_at | ERGIC3 | ERGIC and golgi 3 | 2.55E-08 | -7.32467 |
| 212094_at | PEG10 | paternally expressed 10 | 3.54E-06 | -7.32488 |
| 225848_at | ZNF746 | zinc finger protein 746 | 3.07E-07 | -7.32633 |
| 225079_at | EMP2 | epithelial membrane protein 2 | 2.16E-06 | -7.33131 |
| 218827_s_at | CEP192 | centrosomal protein 192kDa | 5.88E-06 | -7.33142 |
| 226808_at | ZNF862 | zinc finger protein 862 | 5.14E-06 | -7.33285 |
| 223819_x_at | COMMD5 | COMM domain containing 5 | 5.49E-07 | -7.33705 |
| 217901_at | DSG2 | desmoglein 2 | 5.31E-07 | -7.33931 |
| 212180_at | CRKL | v-crk sarcoma virus CT10 oncogene homolog (avian)-like | 3.18E-07 | -7.3433 |
| 203657_s_at | CTSF | cathepsin F | 2.53E-08 | -7.34357 |
| 218113_at | TMEM2 | transmembrane protein 2 | 6.90E-06 | -7.34622 |
| 203499_at | EPHA2 | EPH receptor A2 | 2.16E-07 | -7.34961 |
| 213329_at | SRGAP2 | SLIT-ROBO Rho GTPase activating protein 2 | 1.42E-06 | -7.3504 |
| 215499_at | MAP2K3 | mitogen-activated protein kinase kinase 3 | 2.95E-07 | -7.35328 |
| 202752_x_at | SLC7A8 | solute carrier family 7 (amino acid transporter, L-type), member 8 | 9.26E-06 | -7.35531 |
| 224956_at | NUFIP2 | nuclear fragile X mental retardation protein interacting protein 2 | 1.20E-08 | -7.35567 |
| 224298_s_at | UBAC2 | UBA domain containing 2 | 1.59E-06 | -7.3569 |
| 1554036_at | ZBTB24 | zinc finger and BTB domain containing 24 | 1.29E-07 | -7.36002 |
| 202419_at | KDSR | 3-ketodihydrosphingosine reductase | 9.71E-07 | -7.36014 |
| 235339_at | SETDB2 | SET domain, bifurcated 2 | 3.00E-06 | -7.36402 |
| 212722_s_at | JMJD6 | jumonji domain containing 6 | 6.72E-06 | -7.36722 |
| 203049_s_at | TTC37 | tetratricopeptide repeat domain 37 | 0.000917 | -7.36728 |
| 202797_at | SACM1L | SAC1 suppressor of actin mutations 1-like (yeast) | 0.000126 | -7.36767 |
| 235775_at | TMTC2 | transmembrane and tetratricopeptide repeat containing 2 | 4.52E-05 | -7.37076 |
| 219249_s_at | FKBP10 | FK506 binding protein 10, 65 kDa | 2.08E-07 | -7.37414 |
| 208702_x_at | APLP2 | amyloid beta (A4) precursor-like protein 2 | 1.53E-06 | -7.37505 |
| 203544_s_at | STAM | signal transducing adaptor molecule (SH3 domain and ITAM motif) 1 | 8.00E-07 | -7.37959 |
| 209238_at | STX3 | syntaxin 3 | 1.03E-05 | -7.38052 |
| 225740_x_at | MDM4 | Mdm4 p53 binding protein homolog (mouse) | 3.75E-08 | -7.38133 |
| 224582_s_at | NUCKS1 | nuclear casein kinase and cyclin-dependent kinase substrate 1 | 3.79E-06 | -7.38274 |
| 208447_s_at | PRPS1 | phosphoribosyl pyrophosphate synthetase 1 | 1.19E-09 | -7.38295 |
| 208623_s_at | EZR | ezrin | 3.63E-08 | -7.38302 |
| 232008_s_at | BBX | bobby sox homolog (Drosophila) | 5.87E-06 | -7.38677 |
| 201171_at | ATP6V0E1 | ATPase, H+ transporting, lysosomal 9kDa, V0 subunit e1 | 6.99E-06 | -7.38739 |
| 225478_at | MFHAS1 | malignant fibrous histiocytoma amplified sequence 1 | 6.21E-08 | -7.388 |
| 226233_at | B3GALNT2 | beta-1,3-N-acetylgalactosaminyltransferase 2 | 9.92E-08 | -7.38886 |
| 1553587_a_at | POLE4 | polymerase (DNA-directed), epsilon 4 (p12 subunit) | 8.10E-09 | -7.38984 |
| 201880_at | ARIH1 | ariadne homolog, ubiquitin-conjugating enzyme E2 binding protein, 1 (Drosophila) | 4.79E-08 | -7.39199 |
| 32128_at | CCL18 | chemokine (C-C motif) ligand 18 (pulmonary and activation-regulated) | 1.73E-07 | -7.39527 |
| 238005_s_at | SIN3A | SIN3 homolog A, transcription regulator (yeast) | 1.83E-07 | -7.39558 |
| 204004_at | PAWR | PRKC, apoptosis, WT1, regulator | 1.05E-08 | -7.39786 |
| 208433_s_at | LRP8 | low density lipoprotein receptor-related protein 8, apolipoprotein e receptor | 5.53E-06 | -7.39816 |
| 212246_at | MCFD2 | multiple coagulation factor deficiency 2 | 2.95E-06 | -7.39966 |
| 208631_s_at | HADHA | hydroxyacyl-CoA dehydrogenase/3-ketoacyl-CoA thiolase/enoyl-CoA hydratase (trifu | 6.54E-08 | -7.40261 |
| 226087_at | LZIC | leucine zipper and CTNNBIP1 domain containing | 1.19E-06 | -7.40634 |
| 222217_s_at | SLC27A3 | solute carrier family 27 (fatty acid transporter), member 3 | 7.62E-08 | -7.40721 |
| 230588_s_at | LOC285074 /// LOC730268 | anaphase promoting complex subunit 1 pseudogene /// similar to anaphase promotin | 1.08E-07 | -7.40952 |
| 200946_x_at | GLUD1 | glutamate dehydrogenase 1 | 0.000107 | -7.41164 |
| 222481_at | FXC1 | fracture callus 1 homolog (rat) | 9.69E-07 | -7.41363 |
| 203521_s_at | ZNF318 | zinc finger protein 318 | 1.15E-07 | -7.41822 |
| 214359_s_at | HSP90AB1 | heat shock protein 90kDa alpha (cytosolic), class B member 1 | 2.68E-07 | -7.41921 |
| 211890_x_at | CAPN3 | calpain 3, (p94) | 1.42E-05 | -7.42005 |
| 214484_s_at | SIGMAR1 | sigma non-opioid intracellular receptor 1 | 7.57E-07 | -7.42175 |
| 91684_g_at | EXOSC4 | exosome component 4 | 2.36E-07 | -7.4223 |
| 202213_s_at | CUL4B | cullin 4B | 5.23E-06 | -7.4252 |
| 215913_s_at | GULP1 | GULP, engulfment adaptor PTB domain containing 1 | 4.40E-07 | -7.42921 |
| 202323_s_at | ACBD3 | acyl-CoA binding domain containing 3 | 4.19E-05 | -7.43755 |
| 212170_at | RBM12 | RNA binding motif protein 12 | 1.90E-07 | -7.43759 |
| 232946_s_at | NADSYN1 | NAD synthetase 1 | 2.45E-06 | -7.44015 |
| 223295_s_at | LUC7L | LUC7-like (S. cerevisiae) | 7.73E-07 | -7.4419 |
| 206770_s_at | SLC35A3 | solute carrier family 35 (UDP-N-acetylglucosamine (UDP-GlcNAc) transporter), mem | 5.15E-06 | -7.4431 |
| 227373_at | ATXN1L | ataxin 1-like | 1.12E-06 | -7.44339 |
| 210778_s_at | MXD4 | MAX dimerization protein 4 | 3.60E-06 | -7.44574 |
| 212206_s_at | H2AFV | H2A histone family, member V | 1.52E-06 | -7.45022 |
| 205748_s_at | RNF126 | ring finger protein 126 | 7.58E-08 | -7.45044 |
| 205547_s_at | TAGLN | transgelin | 3.60E-06 | -7.45058 |
| 224856_at | FKBP5 | FK506 binding protein 5 | 1.87E-05 | -7.45322 |
| 201054_at | HNRNPA0 | heterogeneous nuclear ribonucleoprotein A0 | 2.30E-07 | -7.45395 |
| 203039_s_at | NDUFS1 | NADH dehydrogenase (ubiquinone) Fe-S protein 1, 75kDa (NADH-coenzyme Q reductase | 9.88E-07 | -7.45479 |
| 208937_s_at | ID1 | inhibitor of DNA binding 1, dominant negative helix-loop-helix protein | 5.69E-07 | -7.45765 |
| 225921_at | NIN | ninein (GSK3B interacting protein) | 1.83E-07 | -7.45826 |
| 225269_s_at | RBMS1 | RNA binding motif, single stranded interacting protein 1 | 6.34E-07 | -7.45923 |
| 201996_s_at | SPEN | spen homolog, transcriptional regulator (Drosophila) | 1.61E-05 | -7.46077 |
| 202864_s_at | SP100 | SP100 nuclear antigen | 3.35E-06 | -7.46091 |
| 203706_s_at | FZD7 | frizzled homolog 7 (Drosophila) | 1.44E-08 | -7.46103 |
| 207760_s_at | NCOR2 | nuclear receptor co-repressor 2 | 5.60E-07 | -7.46139 |
| 212307_s_at | OGT | O-linked N-acetylglucosamine (GlcNAc) transferase (UDP-N-acetylglucosamine:polyp | 1.06E-06 | -7.46151 |
| 219972_s_at | C14orf135 | chromosome 14 open reading frame 135 | 9.88E-05 | -7.46279 |
| 223449_at | SEMA6A | sema domain, transmembrane domain (TM), and cytoplasmic domain, (semaphorin) 6A | 1.67E-06 | -7.46403 |
| 219121_s_at | ESRP1 | epithelial splicing regulatory protein 1 | 3.99E-09 | -7.46558 |
| 214092_x_at | SFRS14 | splicing factor, arginine/serine-rich 14 | 2.42E-05 | -7.46855 |
| 210749_x_at | DDR1 | discoidin domain receptor tyrosine kinase 1 | 4.52E-07 | -7.47253 |
| 216503_s_at | MLLT10 | myeloid/lymphoid or mixed-lineage leukemia (trithorax homolog, Drosophila); tran | 2.09E-05 | -7.47723 |
| 203785_s_at | DDX28 | DEAD (Asp-Glu-Ala-Asp) box polypeptide 28 | 7.30E-06 | -7.47727 |
| 223546_x_at | LUC7L | LUC7-like (S. cerevisiae) | 4.01E-05 | -7.47902 |
| 212724_at | RND3 | Rho family GTPase 3 | 4.96E-08 | -7.48216 |
| 214778_at | MEGF8 | multiple EGF-like-domains 8 | 5.76E-06 | -7.48344 |
| 235333_at | B4GALT6 | UDP-Gal:betaGlcNAc beta 1,4- galactosyltransferase, polypeptide 6 | 1.63E-07 | -7.48383 |
| 219913_s_at | CRNKL1 | crooked neck pre-mRNA splicing factor-like 1 (Drosophila) | 2.56E-06 | -7.48421 |
| 205286_at | TFAP2C | transcription factor AP-2 gamma (activating enhancer binding protein 2 gamma) | 1.22E-06 | -7.48469 |
| 218723_s_at | C13orf15 | chromosome 13 open reading frame 15 | 3.40E-08 | -7.48583 |
| 209934_s_at | ATP2C1 | ATPase, Ca++ transporting, type 2C, member 1 | 1.18E-06 | -7.48681 |
| 223041_at | CD99L2 | CD99 molecule-like 2 | 1.31E-08 | -7.4873 |
| 201433_s_at | PTDSS1 | phosphatidylserine synthase 1 | 2.82E-08 | -7.48939 |
| 203566_s_at | AGL | amylo-alpha-1, 6-glucosidase, 4-alpha-glucanotransferase | 5.43E-07 | -7.4897 |
| 225462_at | TMEM128 | transmembrane protein 128 | 5.01E-07 | -7.48981 |
| 242554_at | TPCN2 | Two pore segment channel 2 | 1.23E-05 | -7.49464 |
| 201639_s_at | CPSF1 | cleavage and polyadenylation specific factor 1, 160kDa | 4.21E-06 | -7.49654 |
| 223705_s_at | GPBP1 | GC-rich promoter binding protein 1 | 9.50E-06 | -7.49717 |
| 210276_s_at | NOL12 /// TRIOBP | nucleolar protein 12 /// TRIO and F-actin binding protein | 7.75E-06 | -7.49765 |
| 225477_s_at | NR2C2 | nuclear receptor subfamily 2, group C, member 2 | 2.74E-06 | -7.4978 |
| 203245_s_at | NCRNA00094 | non-protein coding RNA 94 | 1.30E-07 | -7.49992 |
| 203126_at | IMPA2 | inositol(myo)-1(or 4)-monophosphatase 2 | 5.79E-06 | -7.50079 |
| 208699_x_at | TKT | transketolase | 2.36E-05 | -7.50347 |
| 222423_at | NDFIP1 | Nedd4 family interacting protein 1 | 2.75E-07 | -7.50524 |
| 224718_at | YY1 | YY1 transcription factor | 4.76E-06 | -7.50582 |
| 203816_at | DGUOK | deoxyguanosine kinase | 1.68E-05 | -7.50638 |
| 217974_at | TM7SF3 | transmembrane 7 superfamily member 3 | 1.82E-07 | -7.50953 |
| 201862_s_at | LRRFIP1 | leucine rich repeat (in FLII) interacting protein 1 | 7.52E-08 | -7.51133 |
| 202569_s_at | MARK3 | MAP/microtubule affinity-regulating kinase 3 | 1.66E-05 | -7.51245 |
| 218090_s_at | WDR11 | WD repeat domain 11 | 2.37E-05 | -7.51605 |
| 218852_at | PPP2R3C | protein phosphatase 2 (formerly 2A), regulatory subunit B'', gamma | 6.30E-08 | -7.51895 |
| 233255_s_at | BIVM | basic, immunoglobulin-like variable motif containing | 6.91E-06 | -7.51955 |
| 201502_s_at | NFKBIA | nuclear factor of kappa light polypeptide gene enhancer in B-cells inhibitor, al | 2.21E-07 | -7.52189 |
| 209362_at | MED21 | mediator complex subunit 21 | 3.81E-05 | -7.52213 |
| 230251_at | C6orf176 | chromosome 6 open reading frame 176 | 5.30E-07 | -7.52425 |
| 218528_s_at | RNF38 | ring finger protein 38 | 1.98E-06 | -7.5315 |
| 209240_at | OGT | O-linked N-acetylglucosamine (GlcNAc) transferase (UDP-N-acetylglucosamine:polyp | 4.32E-08 | -7.53214 |
| 227277_at | MTDH | metadherin | 2.70E-06 | -7.53534 |
| 202071_at | SDC4 | syndecan 4 | 1.23E-08 | -7.53568 |
| 238519_at | DDI2 | DNA-damage inducible 1 homolog 2 (S. cerevisiae) | 2.90E-07 | -7.53677 |
| 226749_at | MRPS9 | mitochondrial ribosomal protein S9 | 1.40E-07 | -7.54017 |
| 214919_s_at | ANKHD1-EIF4EBP3 /// EIF4EBP3 | ANKHD1-EIF4EBP3 readthrough /// eukaryotic translation initiation factor 4E bind | 1.05E-07 | -7.5421 |
| 209498_at | CEACAM1 | carcinoembryonic antigen-related cell adhesion molecule 1 (biliary glycoprotein) | 7.03E-07 | -7.54536 |
| 221676_s_at | CORO1C | coronin, actin binding protein, 1C | 2.63E-07 | -7.55129 |
| 210762_s_at | DLC1 | deleted in liver cancer 1 | 3.75E-06 | -7.55318 |
| 219032_x_at | OPN3 | opsin 3 | 4.98E-06 | -7.55577 |
| 222870_s_at | B3GNT2 | UDP-GlcNAc:betaGal beta-1,3-N-acetylglucosaminyltransferase 2 | 1.87E-06 | -7.55596 |
| 229061_s_at | SLC25A13 | solute carrier family 25, member 13 (citrin) | 1.63E-06 | -7.55734 |
| 222654_at | IMPAD1 | inositol monophosphatase domain containing 1 | 5.70E-06 | -7.56637 |
| 218235_s_at | UTP11L | UTP11-like, U3 small nucleolar ribonucleoprotein, (yeast) | 2.43E-08 | -7.56734 |
| 32091_at | SLC25A44 | solute carrier family 25, member 44 | 2.81E-06 | -7.56922 |
| 201571_s_at | DCTD | dCMP deaminase | 1.14E-06 | -7.57235 |
| 226249_at | SNX30 | sorting nexin family member 30 | 2.24E-06 | -7.57267 |
| 233825_s_at | CD99L2 | CD99 molecule-like 2 | 2.69E-10 | -7.5731 |
| 203600_s_at | FAM193A | family with sequence similarity 193, member A | 5.15E-05 | -7.57446 |
| 212400_at | FAM102A | family with sequence similarity 102, member A | 4.99E-08 | -7.57491 |
| 243502_at | GJC1 | gap junction protein, gamma 1, 45kDa | 4.99E-06 | -7.5761 |
| 223059_s_at | FAM107B | family with sequence similarity 107, member B | 5.22E-07 | -7.57705 |
| 201387_s_at | UCHL1 | ubiquitin carboxyl-terminal esterase L1 (ubiquitin thiolesterase) | 5.90E-07 | -7.57707 |
| 209890_at | TSPAN5 | tetraspanin 5 | 1.08E-07 | -7.57718 |
| 225092_at | RABEP1 | rabaptin, RAB GTPase binding effector protein 1 | 1.71E-07 | -7.5777 |
| 212372_at | MYH10 | myosin, heavy chain 10, non-muscle | 1.02E-09 | -7.58064 |
| 211709_s_at | CLEC11A | C-type lectin domain family 11, member A | 8.83E-07 | -7.58183 |
| 212511_at | PICALM | phosphatidylinositol binding clathrin assembly protein | 4.85E-07 | -7.58186 |
| 214841_at | CNIH3 | cornichon homolog 3 (Drosophila) | 1.87E-06 | -7.58198 |
| 220261_s_at | ZDHHC4 | zinc finger, DHHC-type containing 4 | 3.36E-07 | -7.58224 |
| 201611_s_at | ICMT | isoprenylcysteine carboxyl methyltransferase | 1.92E-06 | -7.58435 |
| 219133_at | OXSM | 3-oxoacyl-ACP synthase, mitochondrial | 2.43E-06 | -7.59203 |
| 204298_s_at | LOX | lysyl oxidase | 1.83E-07 | -7.5923 |
| 217731_s_at | ITM2B | integral membrane protein 2B | 2.09E-06 | -7.59275 |
| 227001_at | NIPAL2 | NIPA-like domain containing 2 | 1.18E-06 | -7.59497 |
| 242550_at | EIF3B | eukaryotic translation initiation factor 3, subunit B | 1.42E-06 | -7.59511 |
| 202912_at | ADM | adrenomedullin | 1.84E-05 | -7.59566 |
| 200728_at | ACTR2 | ARP2 actin-related protein 2 homolog (yeast) | 2.69E-07 | -7.59685 |
| 219284_at | HSPBAP1 | HSPB (heat shock 27kDa) associated protein 1 | 1.67E-06 | -7.59772 |
| 224973_at | FAM46A | family with sequence similarity 46, member A | 2.49E-06 | -7.5984 |
| 201855_s_at | ATMIN | ATM interactor | 6.28E-06 | -7.60055 |
| 221428_s_at | TBL1XR1 | transducin (beta)-like 1 X-linked receptor 1 | 1.43E-05 | -7.60139 |
| 225090_at | SYVN1 | synovial apoptosis inhibitor 1, synoviolin | 1.14E-05 | -7.60996 |
| 210517_s_at | AKAP12 | A kinase (PRKA) anchor protein 12 | 2.59E-08 | -7.6103 |
| 202636_at | RNF103 | ring finger protein 103 | 4.35E-05 | -7.61063 |
| 233656_s_at | VPS54 | vacuolar protein sorting 54 homolog (S. cerevisiae) | 1.81E-07 | -7.61138 |
| 212779_at | KIAA1109 | KIAA1109 | 1.80E-06 | -7.61294 |
| 43544_at | MED16 | mediator complex subunit 16 | 2.51E-06 | -7.61513 |
| 1569069_s_at | TDRD3 | tudor domain containing 3 | 4.03E-07 | -7.61532 |
| 209725_at | UTP20 | UTP20, small subunit (SSU) processome component, homolog (yeast) | 4.11E-08 | -7.61831 |
| 228783_at | BVES | blood vessel epicardial substance | 1.30E-08 | -7.62038 |
| 213476_x_at | TUBB3 | tubulin, beta 3 | 1.64E-06 | -7.62117 |
| 235678_at | GM2A | GM2 ganglioside activator | 7.30E-07 | -7.62221 |
| 200628_s_at | WARS | tryptophanyl-tRNA synthetase | 2.70E-06 | -7.62246 |
| 204137_at | GPR137B | G protein-coupled receptor 137B | 1.47E-07 | -7.6229 |
| 204458_at | PLA2G15 | phospholipase A2, group XV | 1.26E-05 | -7.62665 |
| 223590_at | ZNF700 | zinc finger protein 700 | 2.39E-05 | -7.62977 |
| 220091_at | SLC2A6 | solute carrier family 2 (facilitated glucose transporter), member 6 | 5.39E-06 | -7.63047 |
| 1553105_s_at | DSG2 | desmoglein 2 | 1.46E-07 | -7.63164 |
| 222667_s_at | ASH1L | ash1 (absent, small, or homeotic)-like (Drosophila) | 1.70E-07 | -7.6369 |
| 209288_s_at | CDC42EP3 | CDC42 effector protein (Rho GTPase binding) 3 | 1.09E-06 | -7.64452 |
| 209902_at | ATR | ataxia telangiectasia and Rad3 related | 4.38E-06 | -7.64474 |
| 201771_at | SCAMP3 | secretory carrier membrane protein 3 | 1.32E-07 | -7.64649 |
| 208857_s_at | PCMT1 | protein-L-isoaspartate (D-aspartate) O-methyltransferase | 3.80E-06 | -7.64664 |
| 203094_at | MAD2L1BP | MAD2L1 binding protein | 1.11E-08 | -7.64706 |
| 212517_at | ATRN | attractin | 7.65E-06 | -7.64934 |
| 222473_s_at | ERBB2IP | erbb2 interacting protein | 6.03E-06 | -7.65283 |
| 208674_x_at | DDOST | dolichyl-diphosphooligosaccharide--protein glycosyltransferase | 3.37E-06 | -7.65604 |
| 203374_s_at | TPP2 | tripeptidyl peptidase II | 1.85E-06 | -7.65614 |
| 225273_at | WWC3 | WWC family member 3 | 1.12E-07 | -7.65697 |
| 205196_s_at | AP1S1 | adaptor-related protein complex 1, sigma 1 subunit | 9.11E-08 | -7.65973 |
| 209007_s_at | C1orf63 | chromosome 1 open reading frame 63 | 5.26E-06 | -7.66017 |
| 222432_s_at | CCDC47 | coiled-coil domain containing 47 | 7.90E-07 | -7.66017 |
| 228145_s_at | ZNF398 | zinc finger protein 398 | 6.13E-06 | -7.66035 |
| 209372_x_at | TUBB2A /// TUBB2B | tubulin, beta 2A /// tubulin, beta 2B | 2.93E-07 | -7.66189 |
| 212104_s_at | RBM9 | RNA binding motif protein 9 | 1.74E-06 | -7.66233 |
| 202616_s_at | MECP2 | methyl CpG binding protein 2 (Rett syndrome) | 2.94E-07 | -7.66529 |
| 213478_at | KAZ | kazrin | 6.89E-07 | -7.66618 |
| 213397_x_at | RNASE4 | ribonuclease, RNase A family, 4 | 1.00E-05 | -7.6662 |
| 215533_s_at | UBE4B | ubiquitination factor E4B (UFD2 homolog, yeast) | 2.11E-06 | -7.6675 |
| 224913_s_at | TIMM50 | translocase of inner mitochondrial membrane 50 homolog (S. cerevisiae) | 2.08E-05 | -7.66907 |
| 212621_at | TMEM194A | transmembrane protein 194A | 5.84E-06 | -7.66944 |
| 1552632_a_at | ARSG | arylsulfatase G | 9.10E-09 | -7.67109 |
| 212638_s_at | WWP1 | WW domain containing E3 ubiquitin protein ligase 1 | 9.07E-06 | -7.67152 |
| 227335_at | DIDO1 | death inducer-obliterator 1 | 4.13E-07 | -7.6726 |
| 204518_s_at | PPIC | peptidylprolyl isomerase C (cyclophilin C) | 1.20E-07 | -7.67397 |
| 218656_s_at | LHFP | lipoma HMGIC fusion partner | 1.60E-07 | -7.67442 |
| 225702_at | C8orf76 | chromosome 8 open reading frame 76 | 2.82E-07 | -7.67516 |
| 208127_s_at | SOCS5 | suppressor of cytokine signaling 5 | 2.85E-06 | -7.67557 |
| 201845_s_at | RYBP | RING1 and YY1 binding protein | 1.44E-07 | -7.67712 |
| 224977_at | C6orf89 | chromosome 6 open reading frame 89 | 1.87E-06 | -7.67823 |
| 1554482_a_at | SAR1B | SAR1 homolog B (S. cerevisiae) | 3.17E-06 | -7.68056 |
| 202667_s_at | SLC39A7 | solute carrier family 39 (zinc transporter), member 7 | 3.27E-06 | -7.68353 |
| 228302_x_at | CAMK2N1 | calcium/calmodulin-dependent protein kinase II inhibitor 1 | 1.03E-06 | -7.68591 |
| 226129_at | FAM83H | family with sequence similarity 83, member H | 1.13E-05 | -7.69166 |
| 214752_x_at | FLNA | filamin A, alpha | 5.28E-08 | -7.69746 |
| 201780_s_at | RNF13 | ring finger protein 13 | 1.37E-06 | -7.69947 |
| 1555154_a_at | QKI | quaking homolog, KH domain RNA binding (mouse) | 3.22E-06 | -7.69996 |
| 217746_s_at | PDCD6IP | programmed cell death 6 interacting protein | 1.86E-08 | -7.70142 |
| 209896_s_at | PTPN11 | protein tyrosine phosphatase, non-receptor type 11 | 4.66E-07 | -7.70186 |
| 215983_s_at | UBXN8 | UBX domain protein 8 | 1.76E-06 | -7.70313 |
| 214714_at | ZNF394 | zinc finger protein 394 | 1.29E-05 | -7.70338 |
| 209444_at | RAP1GDS1 | RAP1, GTP-GDP dissociation stimulator 1 | 1.31E-09 | -7.70508 |
| 204981_at | SLC22A18 | solute carrier family 22, member 18 | 1.07E-06 | -7.70757 |
| 202013_s_at | EXT2 | exostosin 2 | 1.25E-06 | -7.70785 |
| 206034_at | SERPINB8 | serpin peptidase inhibitor, clade B (ovalbumin), member 8 | 2.98E-07 | -7.71017 |
| 201751_at | JOSD1 | Josephin domain containing 1 | 8.85E-07 | -7.71206 |
| 201361_at | TMEM109 | transmembrane protein 109 | 9.44E-07 | -7.71304 |
| 212590_at | RRAS2 | related RAS viral (r-ras) oncogene homolog 2 | 6.38E-09 | -7.714 |
| 217933_s_at | LAP3 | leucine aminopeptidase 3 | 4.55E-05 | -7.71846 |
| 201284_s_at | APEH | N-acylaminoacyl-peptide hydrolase | 4.68E-09 | -7.71887 |
| 207945_s_at | CSNK1D | casein kinase 1, delta | 4.04E-06 | -7.71908 |
| 213720_s_at | SMARCA4 | SWI/SNF related, matrix associated, actin dependent regulator of chromatin, subf | 1.46E-08 | -7.71981 |
| 215111_s_at | TSC22D1 | TSC22 domain family, member 1 | 6.25E-07 | -7.72352 |
| 201176_s_at | ARCN1 | archain 1 | 7.19E-08 | -7.72583 |
| 222810_s_at | RASAL2 | RAS protein activator like 2 | 5.23E-07 | -7.7273 |
| 207628_s_at | WBSCR22 | Williams Beuren syndrome chromosome region 22 | 3.43E-06 | -7.73087 |
| 203319_s_at | ZNF148 | zinc finger protein 148 | 1.26E-07 | -7.73193 |
| 218670_at | PUS1 | pseudouridylate synthase 1 | 1.07E-05 | -7.74091 |
| 226713_at | CCDC50 | coiled-coil domain containing 50 | 4.02E-07 | -7.74484 |
| 227017_at | ERICH1 | glutamate-rich 1 | 5.03E-09 | -7.74489 |
| 228512_at | PTCD3 | Pentatricopeptide repeat domain 3 | 6.13E-05 | -7.74752 |
| 204223_at | PRELP | proline/arginine-rich end leucine-rich repeat protein | 1.48E-06 | -7.74836 |
| 213471_at | NPHP4 | nephronophthisis 4 | 2.00E-05 | -7.75143 |
| 225338_at | ZYG11B | zyg-11 homolog B (C. elegans) | 5.61E-06 | -7.75509 |
| 228990_at | SNHG12 | small nucleolar RNA host gene 12 (non-protein coding) | 2.29E-06 | -7.75845 |
| 207564_x_at | OGT | O-linked N-acetylglucosamine (GlcNAc) transferase (UDP-N-acetylglucosamine:polyp | 1.30E-06 | -7.76027 |
| 219239_s_at | ZNF654 | zinc finger protein 654 | 7.37E-05 | -7.76084 |
| 214198_s_at | DGCR2 | DiGeorge syndrome critical region gene 2 | 4.38E-07 | -7.76659 |
| 218668_s_at | RAP2C | RAP2C, member of RAS oncogene family | 1.25E-06 | -7.76802 |
| 227379_at | MBOAT1 | membrane bound O-acyltransferase domain containing 1 | 5.79E-07 | -7.77086 |
| 204840_s_at | EEA1 | early endosome antigen 1 | 2.58E-05 | -7.77173 |
| 203138_at | HAT1 | histone acetyltransferase 1 | 3.23E-07 | -7.7722 |
| 212192_at | KCTD12 | potassium channel tetramerisation domain containing 12 | 0.000432 | -7.77492 |
| 202271_at | FBXO28 | F-box protein 28 | 4.23E-05 | -7.77561 |
| 202651_at | LPGAT1 | lysophosphatidylglycerol acyltransferase 1 | 1.39E-07 | -7.77679 |
| 212591_at | RBM34 | RNA binding motif protein 34 | 4.82E-10 | -7.7793 |
| 225610_at | UHRF2 | ubiquitin-like with PHD and ring finger domains 2 | 5.05E-06 | -7.79383 |
| 212269_s_at | MCM3AP | minichromosome maintenance complex component 3 associated protein | 4.68E-07 | -7.79488 |
| 222203_s_at | RDH14 | retinol dehydrogenase 14 (all-trans/9-cis/11-cis) | 1.09E-06 | -7.79503 |
| 223625_at | FAM126A | family with sequence similarity 126, member A | 1.18E-09 | -7.79764 |
| 241762_at | FBXO32 | F-box protein 32 | 5.50E-06 | -7.7979 |
| 217893_s_at | AKIRIN1 | akirin 1 | 1.76E-08 | -7.8033 |
| 201730_s_at | TPR | translocated promoter region (to activated MET oncogene) | 0.000123 | -7.80605 |
| 205541_s_at | GSPT2 | G1 to S phase transition 2 | 3.80E-05 | -7.80683 |
| 203391_at | FKBP2 | FK506 binding protein 2, 13kDa | 3.02E-05 | -7.80843 |
| 212815_at | ASCC3 | activating signal cointegrator 1 complex subunit 3 | 1.04E-05 | -7.80999 |
| 213517_at | PCBP2 | poly(rC) binding protein 2 | 5.46E-06 | -7.81149 |
| 1553764_a_at | JUB | jub, ajuba homolog (Xenopus laevis) | 3.32E-07 | -7.81296 |
| 226894_at | SLC35A3 | solute carrier family 35 (UDP-N-acetylglucosamine (UDP-GlcNAc) transporter), mem | 5.14E-06 | -7.8135 |
| 203665_at | HMOX1 | heme oxygenase (decycling) 1 | 1.30E-05 | -7.81549 |
| 212805_at | PRUNE2 | prune homolog 2 (Drosophila) | 4.57E-07 | -7.81693 |
| 202453_s_at | GTF2H1 | general transcription factor IIH, polypeptide 1, 62kDa | 1.34E-06 | -7.81934 |
| 208938_at | PRCC | papillary renal cell carcinoma (translocation-associated) | 6.60E-07 | -7.82399 |
| 200730_s_at | PTP4A1 | protein tyrosine phosphatase type IVA, member 1 | 8.76E-08 | -7.82566 |
| 209576_at | GNAI1 | guanine nucleotide binding protein (G protein), alpha inhibiting activity polype | 8.00E-05 | -7.8268 |
| 201998_at | ST6GAL1 | ST6 beta-galactosamide alpha-2,6-sialyltranferase 1 | 1.50E-07 | -7.82788 |
| 215210_s_at | DLST | dihydrolipoamide S-succinyltransferase (E2 component of 2-oxo-glutarate complex) | 4.72E-05 | -7.83042 |
| 200842_s_at | EPRS | glutamyl-prolyl-tRNA synthetase | 1.12E-08 | -7.83674 |
| 212855_at | DCUN1D4 | DCN1, defective in cullin neddylation 1, domain containing 4 (S. cerevisiae) | 3.72E-06 | -7.83815 |
| 211375_s_at | ILF3 | interleukin enhancer binding factor 3, 90kDa | 1.13E-06 | -7.83906 |
| 227542_at | SOCS6 | suppressor of cytokine signaling 6 | 1.12E-05 | -7.83993 |
| 221571_at | TRAF3 | TNF receptor-associated factor 3 | 1.17E-07 | -7.84113 |
| 202418_at | YIF1A | Yip1 interacting factor homolog A (S. cerevisiae) | 1.36E-06 | -7.84132 |
| 222201_s_at | CASP8AP2 | caspase 8 associated protein 2 | 2.97E-06 | -7.84249 |
| 226082_s_at | SFRS15 | splicing factor, arginine/serine-rich 15 | 1.97E-06 | -7.84755 |
| 225070_at | NUS1 | nuclear undecaprenyl pyrophosphate synthase 1 homolog (S. cerevisiae) | 1.64E-07 | -7.84763 |
| 237069_s_at | TRPM1 | transient receptor potential cation channel, subfamily M, member 1 | 2.44E-07 | -7.84951 |
| 212247_at | NUP205 | nucleoporin 205kDa | 1.79E-07 | -7.85414 |
| 202763_at | CASP3 | caspase 3, apoptosis-related cysteine peptidase | 8.33E-08 | -7.85428 |
| 223156_at | MRPS23 | mitochondrial ribosomal protein S23 | 2.51E-06 | -7.85485 |
| 213123_at | MFAP3 | microfibrillar-associated protein 3 | 1.62E-06 | -7.85595 |
| 218120_s_at | HMOX2 | heme oxygenase (decycling) 2 | 6.18E-07 | -7.85874 |
| 222155_s_at | GPR172A | G protein-coupled receptor 172A | 1.85E-07 | -7.86347 |
| 222199_s_at | BIN3 | bridging integrator 3 | 4.36E-08 | -7.86446 |
| 214436_at | FBXL2 | F-box and leucine-rich repeat protein 2 | 5.84E-05 | -7.86604 |
| 200663_at | CD63 | CD63 molecule | 2.86E-09 | -7.86985 |
| 1554333_at | DNAJA4 | DnaJ (Hsp40) homolog, subfamily A, member 4 | 9.29E-05 | -7.87046 |
| 217992_s_at | EFHD2 | EF-hand domain family, member D2 | 7.53E-10 | -7.87072 |
| 221004_s_at | ITM2C | integral membrane protein 2C | 1.86E-08 | -7.87141 |
| 225901_at | PTPMT1 | protein tyrosine phosphatase, mitochondrial 1 | 9.21E-08 | -7.87489 |
| 205090_s_at | NAGPA | N-acetylglucosamine-1-phosphodiester alpha-N-acetylglucosaminidase | 3.54E-05 | -7.87737 |
| 222987_s_at | TMEM9 | transmembrane protein 9 | 2.35E-06 | -7.87973 |
| 212693_at | MDN1 | MDN1, midasin homolog (yeast) | 2.85E-07 | -7.87997 |
| 219717_at | DCAF16 | DDB1 and CUL4 associated factor 16 | 1.19E-05 | -7.883 |
| 214117_s_at | BTD | biotinidase | 5.33E-07 | -7.88577 |
| 200001_at | CAPNS1 | calpain, small subunit 1 | 2.15E-06 | -7.88782 |
| 207169_x_at | DDR1 | discoidin domain receptor tyrosine kinase 1 | 1.41E-06 | -7.89006 |
| 218716_x_at | MTO1 | mitochondrial translation optimization 1 homolog (S. cerevisiae) | 2.04E-06 | -7.89117 |
| 206059_at | ZNF91 | zinc finger protein 91 | 1.08E-06 | -7.89318 |
| 211935_at | ARL6IP1 | ADP-ribosylation factor-like 6 interacting protein 1 | 4.00E-07 | -7.89323 |
| 221736_at | RALGAPB | Ral GTPase activating protein, beta subunit (non-catalytic) | 3.06E-06 | -7.89438 |
| 224640_at | SPPL3 | signal peptide peptidase 3 | 4.80E-07 | -7.89518 |
| 224896_s_at | TTL | tubulin tyrosine ligase | 7.25E-08 | -7.8954 |
| 215075_s_at | GRB2 | growth factor receptor-bound protein 2 | 1.79E-07 | -7.89735 |
| 225375_at | TMEM199 | transmembrane protein 199 | 2.83E-06 | -7.89855 |
| 212633_at | KIAA0776 | KIAA0776 | 3.26E-05 | -7.90235 |
| 218008_at | C7orf42 | chromosome 7 open reading frame 42 | 6.15E-08 | -7.90319 |
| 202583_s_at | RANBP9 | RAN binding protein 9 | 6.42E-06 | -7.90511 |
| 223391_at | SGPP1 | sphingosine-1-phosphate phosphatase 1 | 3.77E-06 | -7.90882 |
| 1567458_s_at | RAC1 | ras-related C3 botulinum toxin substrate 1 (rho family, small GTP binding protei | 1.22E-05 | -7.91087 |
| 218025_s_at | PECI | peroxisomal D3,D2-enoyl-CoA isomerase | 1.68E-08 | -7.91215 |
| 203403_s_at | RNF6 | ring finger protein (C3H2C3 type) 6 | 3.66E-06 | -7.91479 |
| 229453_at | PDIA3 | protein disulfide isomerase family A, member 3 | 4.66E-09 | -7.91505 |
| 204489_s_at | CD44 | CD44 molecule (Indian blood group) | 2.02E-06 | -7.9169 |
| 213428_s_at | COL6A1 | collagen, type VI, alpha 1 | 9.81E-06 | -7.91868 |
| 222478_at | VPS36 | vacuolar protein sorting 36 homolog (S. cerevisiae) | 2.73E-07 | -7.9194 |
| 223002_s_at | XRN2 | 5'-3' exoribonuclease 2 | 8.02E-07 | -7.9231 |
| 201165_s_at | PUM1 | pumilio homolog 1 (Drosophila) | 4.33E-09 | -7.92415 |
| 227396_at | PTPRJ | protein tyrosine phosphatase, receptor type, J | 1.01E-06 | -7.92521 |
| 208867_s_at | CSNK1A1 | casein kinase 1, alpha 1 | 5.31E-06 | -7.9335 |
| 236356_at | NDUFS1 | NADH dehydrogenase (ubiquinone) Fe-S protein 1, 75kDa (NADH-coenzyme Q reductase | 1.82E-05 | -7.93602 |
| 208686_s_at | BRD2 | bromodomain containing 2 | 3.25E-07 | -7.93668 |
| 238418_at | SLC35B4 | solute carrier family 35, member B4 | 4.06E-05 | -7.93722 |
| 221437_s_at | MRPS15 | mitochondrial ribosomal protein S15 | 6.11E-06 | -7.94056 |
| 224866_at | FAR1 | fatty acyl CoA reductase 1 | 1.53E-05 | -7.94116 |
| 233558_s_at | C4orf41 | chromosome 4 open reading frame 41 | 8.41E-06 | -7.94152 |
| 214437_s_at | SHMT2 | serine hydroxymethyltransferase 2 (mitochondrial) | 1.03E-05 | -7.94259 |
| 213998_s_at | DDX17 | DEAD (Asp-Glu-Ala-Asp) box polypeptide 17 | 1.77E-06 | -7.94382 |
| 212281_s_at | TMEM97 | transmembrane protein 97 | 3.55E-10 | -7.94485 |
| 225493_at | LOC144438 | hypothetical LOC144438 | 4.93E-07 | -7.94514 |
| 201810_s_at | SH3BP5 | SH3-domain binding protein 5 (BTK-associated) | 2.00E-09 | -7.9505 |
| 1558700_s_at | ZNF260 | zinc finger protein 260 | 3.56E-05 | -7.95133 |
| 209648_x_at | SOCS5 | suppressor of cytokine signaling 5 | 4.04E-05 | -7.95224 |
| 217940_s_at | CARKD | carbohydrate kinase domain containing | 1.26E-07 | -7.95668 |
| 224756_s_at | BAT5 | HLA-B associated transcript 5 | 1.15E-08 | -7.96323 |
| 212229_s_at | FBXO21 | F-box protein 21 | 2.24E-06 | -7.96417 |
| 235059_at | RAB12 | RAB12, member RAS oncogene family | 5.82E-07 | -7.9643 |
| 204175_at | ZNF593 | zinc finger protein 593 | 3.18E-07 | -7.96909 |
| 201125_s_at | ITGB5 | integrin, beta 5 | 1.50E-08 | -7.97062 |
| 229119_s_at | ZSWIM7 | zinc finger, SWIM-type containing 7 | 2.25E-08 | -7.97306 |
| 212984_at | ATF2 | activating transcription factor 2 | 1.19E-05 | -7.97615 |
| 203435_s_at | MME | membrane metallo-endopeptidase | 5.24E-08 | -7.97683 |
| 225845_at | ZBTB44 | zinc finger and BTB domain containing 44 | 7.97E-07 | -7.97857 |
| 204862_s_at | NME3 | non-metastatic cells 3, protein expressed in | 1.52E-07 | -7.97917 |
| 218157_x_at | CDC42SE1 | CDC42 small effector 1 | 1.41E-08 | -7.98175 |
| 212644_s_at | MAPK1IP1L | mitogen-activated protein kinase 1 interacting protein 1-like | 5.67E-05 | -7.9831 |
| 213342_at | YAP1 | Yes-associated protein 1 | 1.86E-05 | -7.98323 |
| 210178_x_at | SFRS13A | splicing factor, arginine/serine-rich 13A | 1.00E-06 | -7.98351 |
| 226605_at | DGKQ | diacylglycerol kinase, theta 110kDa | 1.19E-07 | -7.98476 |
| 228077_at | MRI1 | methylthioribose-1-phosphate isomerase homolog (S. cerevisiae) | 1.25E-07 | -7.98619 |
| 211966_at | COL4A2 | collagen, type IV, alpha 2 | 1.24E-07 | -7.98853 |
| 212830_at | MEGF9 | multiple EGF-like-domains 9 | 4.48E-08 | -7.98994 |
| 225520_at | MTHFD1L | methylenetetrahydrofolate dehydrogenase (NADP+ dependent) 1-like | 1.86E-07 | -7.99025 |
| 236814_at | MDM4 | Mdm4 p53 binding protein homolog (mouse) | 8.33E-08 | -7.99255 |
| 202170_s_at | AASDHPPT | aminoadipate-semialdehyde dehydrogenase-phosphopantetheinyl transferase | 1.58E-07 | -7.99607 |
| 202006_at | PTPN12 | protein tyrosine phosphatase, non-receptor type 12 | 9.81E-07 | -7.99698 |
| 212635_at | TNPO1 | transportin 1 | 9.67E-07 | -7.99761 |
| 218681_s_at | SDF2L1 | stromal cell-derived factor 2-like 1 | 1.49E-08 | -7.99788 |
| 202774_s_at | SFRS8 | splicing factor, arginine/serine-rich 8 (suppressor-of-white-apricot homolog, Dr | 4.29E-06 | -7.9995 |
| 232473_at | PRPF18 | PRP18 pre-mRNA processing factor 18 homolog (S. cerevisiae) | 7.25E-09 | -8.00091 |
| 218224_at | PNMA1 | paraneoplastic antigen MA1 | 1.90E-07 | -8.00175 |
| 201323_at | EBNA1BP2 | EBNA1 binding protein 2 | 1.68E-08 | -8.00186 |
| 203604_at | ZNF516 | zinc finger protein 516 | 1.46E-05 | -8.00253 |
| 227199_at | DIP2A | DIP2 disco-interacting protein 2 homolog A (Drosophila) | 1.28E-05 | -8.0038 |
| 206414_s_at | ASAP2 | ArfGAP with SH3 domain, ankyrin repeat and PH domain 2 | 2.88E-08 | -8.00528 |
| 218853_s_at | MOSPD1 | motile sperm domain containing 1 | 2.05E-07 | -8.00541 |
| 226180_at | WDR36 | WD repeat domain 36 | 2.25E-08 | -8.00808 |
| 210186_s_at | FKBP1A | FK506 binding protein 1A, 12kDa | 1.33E-07 | -8.01026 |
| 223566_s_at | BCOR | BCL6 co-repressor | 7.89E-08 | -8.01355 |
| 218938_at | FBXL15 | F-box and leucine-rich repeat protein 15 | 1.37E-05 | -8.01495 |
| 209593_s_at | TOR1B | torsin family 1, member B (torsin B) | 4.54E-06 | -8.0157 |
| 212904_at | LRRC47 | leucine rich repeat containing 47 | 2.28E-08 | -8.01799 |
| 243463_s_at | RIT1 | Ras-like without CAAX 1 | 3.04E-05 | -8.02318 |
| 221718_s_at | AKAP13 | A kinase (PRKA) anchor protein 13 | 2.15E-07 | -8.02627 |
| 200753_x_at | SFRS2 | splicing factor, arginine/serine-rich 2 | 2.77E-06 | -8.02933 |
| 222569_at | UGGT1 | UDP-glucose glycoprotein glucosyltransferase 1 | 4.94E-05 | -8.02993 |
| 225681_at | CTHRC1 | collagen triple helix repeat containing 1 | 1.37E-09 | -8.03075 |
| 212911_at | DNAJC16 | DnaJ (Hsp40) homolog, subfamily C, member 16 | 0.000432 | -8.03259 |
| 218067_s_at | ARGLU1 | arginine and glutamate rich 1 | 1.11E-06 | -8.03267 |
| 220761_s_at | TAOK3 | TAO kinase 3 | 5.95E-07 | -8.033 |
| 218079_s_at | GGNBP2 | gametogenetin binding protein 2 | 2.70E-06 | -8.03357 |
| 46323_at | CANT1 | calcium activated nucleotidase 1 | 5.25E-07 | -8.04479 |
| 228057_at | DDIT4L | DNA-damage-inducible transcript 4-like | 2.87E-05 | -8.04704 |
| 207667_s_at | MAP2K3 | mitogen-activated protein kinase kinase 3 | 1.01E-07 | -8.05058 |
| 229114_at | GAB1 | GRB2-associated binding protein 1 | 2.44E-05 | -8.05725 |
| 212795_at | KIAA1033 | KIAA1033 | 6.54E-06 | -8.05818 |
| 212453_at | KIAA1279 | KIAA1279 | 5.93E-07 | -8.05925 |
| 227812_at | TNFRSF19 | tumor necrosis factor receptor superfamily, member 19 | 2.68E-07 | -8.05996 |
| 204710_s_at | WIPI2 | WD repeat domain, phosphoinositide interacting 2 | 3.77E-09 | -8.06103 |
| 224960_at | SCYL2 | SCY1-like 2 (S. cerevisiae) | 1.16E-08 | -8.06492 |
| 201109_s_at | THBS1 | thrombospondin 1 | 7.29E-08 | -8.06558 |
| 209751_s_at | TRAPPC2 /// TRAPPC2P1 | trafficking protein particle complex 2 /// trafficking protein particle complex | 7.91E-07 | -8.0665 |
| 32069_at | N4BP1 | NEDD4 binding protein 1 | 1.47E-06 | -8.06843 |
| 223267_at | RG9MTD1 | RNA (guanine-9-) methyltransferase domain containing 1 | 5.20E-06 | -8.06958 |
| 202469_s_at | CPSF6 | cleavage and polyadenylation specific factor 6, 68kDa | 7.06E-08 | -8.07073 |
| 201950_x_at | CAPZB | capping protein (actin filament) muscle Z-line, beta | 6.62E-06 | -8.07197 |
| 205097_at | SLC26A2 | solute carrier family 26 (sulfate transporter), member 2 | 8.55E-08 | -8.07255 |
| 225456_at | MED1 | mediator complex subunit 1 | 3.55E-08 | -8.07348 |
| 226120_at | TTC8 | tetratricopeptide repeat domain 8 | 3.66E-07 | -8.07357 |
| 219253_at | TMEM185B | transmembrane protein 185B (pseudogene) | 1.45E-05 | -8.07489 |
| 223211_at | HACL1 | 2-hydroxyacyl-CoA lyase 1 | 4.05E-09 | -8.08527 |
| 221781_s_at | DNAJC10 | DnaJ (Hsp40) homolog, subfamily C, member 10 | 3.01E-07 | -8.09457 |
| 212989_at | SGMS1 | sphingomyelin synthase 1 | 6.39E-05 | -8.09591 |
| 202350_s_at | MATN2 | matrilin 2 | 3.40E-07 | -8.09739 |
| 218771_at | PANK4 | pantothenate kinase 4 | 2.70E-07 | -8.0986 |
| 212239_at | PIK3R1 | phosphoinositide-3-kinase, regulatory subunit 1 (alpha) | 1.21E-07 | -8.10123 |
| 214992_s_at | DNASE2 | deoxyribonuclease II, lysosomal | 3.83E-08 | -8.10372 |
| 203669_s_at | DGAT1 | diacylglycerol O-acyltransferase homolog 1 (mouse) | 2.16E-07 | -8.10538 |
| 226789_at | EMB | embigin homolog (mouse) | 1.55E-08 | -8.10967 |
| 224518_s_at | ZNF559 | zinc finger protein 559 | 1.50E-07 | -8.11298 |
| 202263_at | CYB5R1 | cytochrome b5 reductase 1 | 6.25E-06 | -8.11331 |
| 214434_at | HSPA12A | heat shock 70kDa protein 12A | 2.82E-07 | -8.11837 |
| 223249_at | CLDN12 | claudin 12 | 1.65E-06 | -8.1201 |
| 218254_s_at | SAR1B | SAR1 homolog B (S. cerevisiae) | 1.42E-05 | -8.12524 |
| 220690_s_at | DHRS7B | dehydrogenase/reductase (SDR family) member 7B | 1.81E-06 | -8.12572 |
| 233936_s_at | GGNBP2 | gametogenetin binding protein 2 | 2.40E-06 | -8.1262 |
| 207826_s_at | ID3 | inhibitor of DNA binding 3, dominant negative helix-loop-helix protein | 1.54E-06 | -8.12822 |
| 202609_at | EPS8 | epidermal growth factor receptor pathway substrate 8 | 1.04E-08 | -8.13574 |
| 222514_at | RRAGC | Ras-related GTP binding C | 1.32E-07 | -8.13611 |
| 212096_s_at | MTUS1 | microtubule associated tumor suppressor 1 | 2.98E-07 | -8.13869 |
| 218082_s_at | UBP1 | upstream binding protein 1 (LBP-1a) | 1.88E-05 | -8.13978 |
| 219042_at | LZTS1 | leucine zipper, putative tumor suppressor 1 | 2.23E-07 | -8.14227 |
| 202762_at | ROCK2 | Rho-associated, coiled-coil containing protein kinase 2 | 1.68E-05 | -8.14479 |
| 208708_x_at | EIF5 | eukaryotic translation initiation factor 5 | 1.41E-05 | -8.14649 |
| 215177_s_at | ITGA6 | integrin, alpha 6 | 2.65E-05 | -8.1484 |
| 229265_at | SKI | v-ski sarcoma viral oncogene homolog (avian) | 7.45E-08 | -8.14921 |
| 223049_at | GRB2 | growth factor receptor-bound protein 2 | 6.39E-09 | -8.15197 |
| 202619_s_at | PLOD2 | procollagen-lysine, 2-oxoglutarate 5-dioxygenase 2 | 2.80E-08 | -8.15346 |
| 205452_at | PIGB | phosphatidylinositol glycan anchor biosynthesis, class B | 1.73E-06 | -8.15525 |
| 201196_s_at | AMD1 | adenosylmethionine decarboxylase 1 | 3.58E-08 | -8.15609 |
| 229173_at | KIAA1715 | KIAA1715 | 1.24E-05 | -8.15913 |
| 213279_at | DHRS1 | dehydrogenase/reductase (SDR family) member 1 | 2.84E-06 | -8.16237 |
| 231873_at | BMPR2 | bone morphogenetic protein receptor, type II (serine/threonine kinase) | 5.06E-05 | -8.16244 |
| 217966_s_at | FAM129A | family with sequence similarity 129, member A | 1.76E-08 | -8.16518 |
| 200670_at | XBP1 | X-box binding protein 1 | 0.000184 | -8.16794 |
| 212099_at | RHOB | ras homolog gene family, member B | 1.01E-06 | -8.17003 |
| 1555730_a_at | CFL1 | cofilin 1 (non-muscle) | 1.27E-05 | -8.17064 |
| 212711_at | CAMSAP1 | calmodulin regulated spectrin-associated protein 1 | 2.37E-07 | -8.17155 |
| 208922_s_at | NXF1 | nuclear RNA export factor 1 | 7.34E-09 | -8.17527 |
| 203502_at | BPGM | 2,3-bisphosphoglycerate mutase | 6.81E-06 | -8.17593 |
| 218268_at | TBC1D15 | TBC1 domain family, member 15 | 9.36E-08 | -8.1764 |
| 218064_s_at | AKAP8L | A kinase (PRKA) anchor protein 8-like | 4.00E-06 | -8.17735 |
| 208945_s_at | BECN1 | beclin 1, autophagy related | 7.61E-08 | -8.17926 |
| 200706_s_at | LITAF | lipopolysaccharide-induced TNF factor | 1.88E-05 | -8.17989 |
| 209430_at | BTAF1 | BTAF1 RNA polymerase II, B-TFIID transcription factor-associated, 170kDa (Mot1 h | 7.60E-07 | -8.17993 |
| 214659_x_at | YLPM1 | YLP motif containing 1 | 2.19E-06 | -8.18205 |
| 214736_s_at | ADD1 | adducin 1 (alpha) | 5.91E-06 | -8.18847 |
| 221020_s_at | SLC25A32 | solute carrier family 25, member 32 | 4.32E-06 | -8.19133 |
| 213093_at | PRKCA | protein kinase C, alpha | 4.52E-08 | -8.19334 |
| 213024_at | TMF1 | TATA element modulatory factor 1 | 3.43E-05 | -8.1956 |
| 218364_at | LRRFIP2 | leucine rich repeat (in FLII) interacting protein 2 | 1.92E-07 | -8.20139 |
| 214430_at | GLA | galactosidase, alpha | 1.03E-08 | -8.20208 |
| 203108_at | GPRC5A | G protein-coupled receptor, family C, group 5, member A | 1.03E-08 | -8.20558 |
| 31845_at | ELF4 | E74-like factor 4 (ets domain transcription factor) | 4.82E-09 | -8.20846 |
| 221806_s_at | SETD5 | SET domain containing 5 | 1.37E-06 | -8.20923 |
| 219709_x_at | FAM173A | family with sequence similarity 173, member A | 1.22E-07 | -8.20982 |
| 217971_at | MAPKSP1 | MAPK scaffold protein 1 | 1.80E-07 | -8.215 |
| 202014_at | PPP1R15A | protein phosphatase 1, regulatory (inhibitor) subunit 15A | 5.54E-07 | -8.22577 |
| 200901_s_at | M6PR | mannose-6-phosphate receptor (cation dependent) | 1.44E-06 | -8.22939 |
| 219571_s_at | ZNF12 | zinc finger protein 12 | 5.46E-05 | -8.23177 |
| 235410_at | NPHP3 | nephronophthisis 3 (adolescent) | 8.68E-07 | -8.23924 |
| 201244_s_at | RAF1 | v-raf-1 murine leukemia viral oncogene homolog 1 | 2.62E-07 | -8.24766 |
| 217943_s_at | MAP7D1 | MAP7 domain containing 1 | 1.04E-07 | -8.24781 |
| 215259_s_at | CADM4 | cell adhesion molecule 4 | 8.04E-08 | -8.2493 |
| 219355_at | CXorf57 | chromosome X open reading frame 57 | 2.29E-05 | -8.25036 |
| 208928_at | POR | P450 (cytochrome) oxidoreductase | 1.62E-05 | -8.25147 |
| 222028_at | ZNF45 | zinc finger protein 45 | 5.85E-05 | -8.25794 |
| 218505_at | WDR59 | WD repeat domain 59 | 6.69E-06 | -8.25912 |
| 212231_at | FBXO21 | F-box protein 21 | 2.44E-07 | -8.26104 |
| 221007_s_at | FIP1L1 | FIP1 like 1 (S. cerevisiae) | 5.18E-06 | -8.26192 |
| 211971_s_at | LRPPRC | leucine-rich PPR-motif containing | 1.49E-08 | -8.26279 |
| 225946_at | RASSF8 | Ras association (RalGDS/AF-6) domain family (N-terminal) member 8 | 2.61E-07 | -8.26295 |
| 228314_at | LRRC8C | leucine rich repeat containing 8 family, member C | 2.69E-06 | -8.26623 |
| 228046_at | ZNF827 | Zinc finger protein 827 | 8.91E-08 | -8.26696 |
| 225558_at | GIT2 | G protein-coupled receptor kinase interacting ArfGAP 2 | 4.11E-07 | -8.26751 |
| 212536_at | ATP11B | ATPase, class VI, type 11B | 2.02E-06 | -8.27236 |
| 46665_at | SEMA4C | sema domain, immunoglobulin domain (Ig), transmembrane domain (TM) and short cyt | 1.64E-09 | -8.27474 |
| 214830_at | SLC38A6 | solute carrier family 38, member 6 | 6.34E-06 | -8.27579 |
| 224885_s_at | KRTCAP2 | keratinocyte associated protein 2 | 6.61E-08 | -8.27798 |
| 226952_at | EAF1 | ELL associated factor 1 | 1.99E-07 | -8.27838 |
| 205618_at | PRRG1 | proline rich Gla (G-carboxyglutamic acid) 1 | 3.88E-07 | -8.27886 |
| 204969_s_at | RDX | radixin | 2.07E-06 | -8.27934 |
| 1567107_s_at | TPM4 | tropomyosin 4 | 2.78E-06 | -8.2822 |
| 217297_s_at | MYO9B | myosin IXB | 7.27E-06 | -8.28317 |
| 212370_x_at | FAM21A /// FAM21B /// FAM21C | family with sequence similarity 21, member A /// family with sequence similarity | 3.41E-07 | -8.28614 |
| 200758_s_at | NFE2L1 | nuclear factor (erythroid-derived 2)-like 1 | 2.34E-06 | -8.28632 |
| 74694_s_at | RABEP2 | rabaptin, RAB GTPase binding effector protein 2 | 1.38E-07 | -8.28758 |
| 200790_at | ODC1 | ornithine decarboxylase 1 | 9.80E-10 | -8.29055 |
| 201908_at | DVL3 | dishevelled, dsh homolog 3 (Drosophila) | 1.52E-07 | -8.29204 |
| 225708_at | MED29 | mediator complex subunit 29 | 1.70E-06 | -8.29624 |
| 224619_at | CASC4 | cancer susceptibility candidate 4 | 9.60E-06 | -8.29662 |
| 203489_at | SIVA1 | SIVA1, apoptosis-inducing factor | 5.84E-08 | -8.29669 |
| 201716_at | SNX1 | sorting nexin 1 | 5.71E-08 | -8.29858 |
| 202491_s_at | IKBKAP | inhibitor of kappa light polypeptide gene enhancer in B-cells, kinase complex-as | 1.03E-06 | -8.30004 |
| 225064_at | RABEP1 | rabaptin, RAB GTPase binding effector protein 1 | 1.71E-07 | -8.30378 |
| 201727_s_at | ELAVL1 | ELAV (embryonic lethal, abnormal vision, Drosophila)-like 1 (Hu antigen R) | 2.07E-07 | -8.30455 |
| 235882_at | VPS53 | vacuolar protein sorting 53 homolog (S. cerevisiae) | 3.70E-07 | -8.30591 |
| 207483_s_at | CAND1 | cullin-associated and neddylation-dissociated 1 | 9.64E-08 | -8.30924 |
| 200008_s_at | GDI2 | GDP dissociation inhibitor 2 | 1.84E-05 | -8.30936 |
| 217873_at | CAB39 | calcium binding protein 39 | 1.86E-08 | -8.31065 |
| 203739_at | ZNF217 | zinc finger protein 217 | 2.31E-06 | -8.31505 |
| 212378_at | GART | phosphoribosylglycinamide formyltransferase, phosphoribosylglycinamide synthetas | 5.04E-07 | -8.31807 |
| 222977_at | SURF4 | surfeit 4 | 1.90E-06 | -8.32246 |
| 1555948_s_at | FAM120A | family with sequence similarity 120A | 4.12E-05 | -8.32513 |
| 225461_at | EHMT1 | euchromatic histone-lysine N-methyltransferase 1 | 1.62E-07 | -8.32527 |
| 204937_s_at | ZNF274 | zinc finger protein 274 | 2.14E-08 | -8.32616 |
| 209412_at | TRAPPC10 | trafficking protein particle complex 10 | 8.60E-06 | -8.32839 |
| 223490_s_at | EXOSC3 | exosome component 3 | 3.99E-07 | -8.32951 |
| 222662_at | PPP1R3B | protein phosphatase 1, regulatory (inhibitor) subunit 3B | 1.11E-07 | -8.34501 |
| 214047_s_at | MBD4 | methyl-CpG binding domain protein 4 | 5.61E-06 | -8.34549 |
| 205158_at | RNASE4 | ribonuclease, RNase A family, 4 | 1.70E-06 | -8.34578 |
| 219952_s_at | MCOLN1 | mucolipin 1 | 1.22E-06 | -8.34948 |
| 204140_at | TPST1 | tyrosylprotein sulfotransferase 1 | 4.83E-07 | -8.35201 |
| 204088_at | P2RX4 | purinergic receptor P2X, ligand-gated ion channel, 4 | 8.69E-07 | -8.35345 |
| 224888_at | EPT1 | ethanolaminephosphotransferase 1 (CDP-ethanolamine-specific) | 2.63E-07 | -8.35413 |
| 201012_at | ANXA1 | annexin A1 | 2.78E-10 | -8.36032 |
| 202527_s_at | SMAD4 | SMAD family member 4 | 5.46E-06 | -8.36065 |
| 202126_at | PRPF4B | PRP4 pre-mRNA processing factor 4 homolog B (yeast) | 4.98E-06 | -8.36582 |
| 212697_at | FAM134C | family with sequence similarity 134, member C | 1.24E-06 | -8.36793 |
| 204658_at | TRA2A | transformer 2 alpha homolog (Drosophila) | 2.36E-07 | -8.36816 |
| 232914_s_at | SYTL2 | synaptotagmin-like 2 | 7.82E-06 | -8.37002 |
| 226094_at | PIK3C2A | phosphoinositide-3-kinase, class 2, alpha polypeptide | 1.90E-06 | -8.37088 |
| 205351_at | GGCX | gamma-glutamyl carboxylase | 2.90E-08 | -8.37564 |
| 205548_s_at | BTG3 | BTG family, member 3 | 2.33E-07 | -8.37741 |
| 213533_at | D4S234E /// FOXP1 | DNA segment on chromosome 4 (unique) 234 expressed sequence /// forkhead box P1 | 3.90E-08 | -8.37859 |
| 53071_s_at | C17orf101 | chromosome 17 open reading frame 101 | 2.76E-07 | -8.38441 |
| 209263_x_at | TSPAN4 | tetraspanin 4 | 8.59E-07 | -8.38857 |
| 201032_at | BLCAP | bladder cancer associated protein | 3.91E-05 | -8.39217 |
| 220305_at | MAVS | mitochondrial antiviral signaling protein | 1.88E-08 | -8.39461 |
| 202348_s_at | TOR1A | torsin family 1, member A (torsin A) | 3.06E-07 | -8.39577 |
| 224796_at | ASAP1 | ArfGAP with SH3 domain, ankyrin repeat and PH domain 1 | 5.77E-09 | -8.39721 |
| 212365_at | MYO1B | myosin IB | 7.50E-08 | -8.39914 |
| 210251_s_at | RUFY3 | RUN and FYVE domain containing 3 | 2.90E-06 | -8.39929 |
| 229120_s_at | CDC42SE1 | CDC42 small effector 1 | 7.98E-07 | -8.39997 |
| 204014_at | DUSP4 | dual specificity phosphatase 4 | 5.44E-07 | -8.40093 |
| 212775_at | OBSL1 | obscurin-like 1 | 8.93E-07 | -8.40846 |
| 202626_s_at | LYN | v-yes-1 Yamaguchi sarcoma viral related oncogene homolog | 3.84E-08 | -8.41029 |
| 221260_s_at | CSRNP2 | cysteine-serine-rich nuclear protein 2 | 5.28E-05 | -8.41454 |
| 223239_at | C14orf129 | chromosome 14 open reading frame 129 | 2.83E-07 | -8.41684 |
| 220202_s_at | RC3H2 | ring finger and CCCH-type zinc finger domains 2 | 1.87E-07 | -8.41873 |
| 209846_s_at | BTN3A2 | butyrophilin, subfamily 3, member A2 | 3.24E-06 | -8.42415 |
| 225868_at | TRIM47 | tripartite motif-containing 47 | 1.38E-07 | -8.42881 |
| 202143_s_at | COPS8 | COP9 constitutive photomorphogenic homolog subunit 8 (Arabidopsis) | 6.91E-07 | -8.43081 |
| 212108_at | FAF2 | Fas associated factor family member 2 | 1.63E-06 | -8.43409 |
| 202179_at | BLMH | bleomycin hydrolase | 9.17E-07 | -8.43427 |
| 210980_s_at | ASAH1 | N-acylsphingosine amidohydrolase (acid ceramidase) 1 | 2.74E-06 | -8.43487 |
| 224200_s_at | RAD18 | RAD18 homolog (S. cerevisiae) | 2.85E-05 | -8.44215 |
| 205105_at | MAN2A1 | mannosidase, alpha, class 2A, member 1 | 2.09E-07 | -8.44417 |
| 219278_at | MAP3K6 | mitogen-activated protein kinase kinase kinase 6 | 9.70E-06 | -8.44501 |
| 217849_s_at | CDC42BPB | CDC42 binding protein kinase beta (DMPK-like) | 2.21E-06 | -8.44513 |
| 229415_at | CYCS | cytochrome c, somatic | 1.80E-05 | -8.44565 |
| 228375_at | IGSF11 | immunoglobulin superfamily, member 11 | 1.28E-07 | -8.44784 |
| 222458_s_at | AKIRIN1 | akirin 1 | 8.98E-05 | -8.44809 |
| 222664_at | KCTD15 | potassium channel tetramerisation domain containing 15 | 2.02E-09 | -8.44924 |
| 222559_s_at | RPRD1A | regulation of nuclear pre-mRNA domain containing 1A | 2.10E-07 | -8.44952 |
| 212254_s_at | DST | dystonin | 7.60E-07 | -8.45103 |
| 221452_s_at | TMEM14B | transmembrane protein 14B | 9.15E-07 | -8.45455 |
| 226516_at | C19orf28 | chromosome 19 open reading frame 28 | 2.22E-06 | -8.45518 |
| 200605_s_at | PRKAR1A | protein kinase, cAMP-dependent, regulatory, type I, alpha (tissue specific extin | 2.60E-06 | -8.45908 |
| 229271_x_at | COL11A1 | collagen, type XI, alpha 1 | 0.00027 | -8.45994 |
| 213313_at | RABGAP1 | RAB GTPase activating protein 1 | 2.67E-06 | -8.46138 |
| 218469_at | GREM1 | gremlin 1, cysteine knot superfamily, homolog (Xenopus laevis) | 1.47E-05 | -8.46299 |
| 208820_at | PTK2 | PTK2 protein tyrosine kinase 2 | 8.90E-08 | -8.46677 |
| 224661_at | PIGY | phosphatidylinositol glycan anchor biosynthesis, class Y | 5.40E-06 | -8.4669 |
| 222983_s_at | PAIP2 | poly(A) binding protein interacting protein 2 | 9.75E-09 | -8.46927 |
| 201739_at | SGK1 | serum/glucocorticoid regulated kinase 1 | 3.90E-11 | -8.47304 |
| 223132_s_at | TRIM8 | tripartite motif-containing 8 | 3.84E-08 | -8.47925 |
| 235372_at | FCRLA | Fc receptor-like A | 1.20E-06 | -8.47971 |
| 208899_x_at | ATP6V1D | ATPase, H+ transporting, lysosomal 34kDa, V1 subunit D | 6.12E-07 | -8.48135 |
| 224643_at | PRRC1 | proline-rich coiled-coil 1 | 1.28E-07 | -8.48142 |
| 202211_at | ARFGAP3 | ADP-ribosylation factor GTPase activating protein 3 | 1.20E-05 | -8.4865 |
| 219479_at | KDELC1 | KDEL (Lys-Asp-Glu-Leu) containing 1 | 6.95E-08 | -8.48665 |
| 201819_at | SCARB1 | scavenger receptor class B, member 1 | 6.93E-08 | -8.48859 |
| 213005_s_at | KANK1 | KN motif and ankyrin repeat domains 1 | 1.09E-07 | -8.49048 |
| 200648_s_at | GLUL | glutamate-ammonia ligase | 8.02E-07 | -8.49555 |
| 203231_s_at | ATXN1 | ataxin 1 | 1.77E-06 | -8.49644 |
| 223138_s_at | DHX36 | DEAH (Asp-Glu-Ala-His) box polypeptide 36 | 2.78E-07 | -8.49645 |
| 216232_s_at | GCN1L1 | GCN1 general control of amino-acid synthesis 1-like 1 (yeast) | 7.00E-07 | -8.49707 |
| 213291_s_at | UBE3A | ubiquitin protein ligase E3A | 1.02E-06 | -8.49725 |
| 219464_at | CA14 | carbonic anhydrase XIV | 1.49E-07 | -8.49846 |
| 203946_s_at | ARG2 | arginase, type II | 8.40E-07 | -8.4992 |
| 208636_at | ACTN1 | actinin, alpha 1 | 7.34E-08 | -8.50187 |
| 1555225_at | C1orf43 | chromosome 1 open reading frame 43 | 1.84E-08 | -8.50364 |
| 226026_at | DIRC2 | disrupted in renal carcinoma 2 | 2.60E-06 | -8.51063 |
| 208892_s_at | DUSP6 | dual specificity phosphatase 6 | 1.32E-05 | -8.51235 |
| 214954_at | SUSD5 | sushi domain containing 5 | 1.03E-05 | -8.5146 |
| 229630_s_at | WTAP | Wilms tumor 1 associated protein | 1.16E-07 | -8.5152 |
| 244463_at | ADAM23 | ADAM metallopeptidase domain 23 | 1.26E-05 | -8.5177 |
| 212337_at | TUG1 | taurine upregulated 1 (non-protein coding) | 3.01E-06 | -8.52415 |
| 212481_s_at | TPM4 | tropomyosin 4 | 9.44E-08 | -8.52418 |
| 55081_at | MICALL1 | MICAL-like 1 | 3.37E-06 | -8.52458 |
| 238061_at | LGI3 | leucine-rich repeat LGI family, member 3 | 1.20E-06 | -8.53029 |
| 201742_x_at | SFRS1 | splicing factor, arginine/serine-rich 1 | 2.51E-05 | -8.53669 |
| 211776_s_at | EPB41L3 | erythrocyte membrane protein band 4.1-like 3 | 4.17E-05 | -8.53788 |
| 201580_s_at | TMX4 | thioredoxin-related transmembrane protein 4 | 2.12E-07 | -8.54188 |
| 216033_s_at | FYN | FYN oncogene related to SRC, FGR, YES | 2.99E-07 | -8.54463 |
| 202011_at | TJP1 | tight junction protein 1 (zona occludens 1) | 9.48E-08 | -8.55312 |
| 205329_s_at | SNX4 | sorting nexin 4 | 2.55E-07 | -8.55404 |
| 202808_at | C10orf26 | chromosome 10 open reading frame 26 | 1.42E-09 | -8.55693 |
| 200927_s_at | RAB14 | RAB14, member RAS oncogene family | 9.16E-07 | -8.55737 |
| 224247_s_at | MRPS10 | mitochondrial ribosomal protein S10 | 3.42E-07 | -8.5643 |
| 209825_s_at | UCK2 | uridine-cytidine kinase 2 | 1.47E-08 | -8.56762 |
| 225884_s_at | GZF1 | GDNF-inducible zinc finger protein 1 | 2.78E-06 | -8.57 |
| 202303_x_at | SMARCA5 | SWI/SNF related, matrix associated, actin dependent regulator of chromatin, subf | 2.61E-07 | -8.57233 |
| 214545_s_at | PROSC | proline synthetase co-transcribed homolog (bacterial) | 3.29E-05 | -8.57517 |
| 217989_at | HSD17B11 | hydroxysteroid (17-beta) dehydrogenase 11 | 1.00E-05 | -8.57594 |
| 226684_at | ATG2B | ATG2 autophagy related 2 homolog B (S. cerevisiae) | 5.64E-07 | -8.57949 |
| 203324_s_at | CAV2 | caveolin 2 | 9.82E-06 | -8.58219 |
| 220370_s_at | USP36 | ubiquitin specific peptidase 36 | 2.44E-05 | -8.58459 |
| 204215_at | C7orf23 | chromosome 7 open reading frame 23 | 6.78E-08 | -8.58486 |
| 231736_x_at | MGST1 | microsomal glutathione S-transferase 1 | 7.76E-07 | -8.5885 |
| 212282_at | TMEM97 | transmembrane protein 97 | 1.30E-08 | -8.59004 |
| 201798_s_at | MYOF | myoferlin | 8.38E-09 | -8.59145 |
| 223241_at | SNX8 | sorting nexin 8 | 7.97E-07 | -8.5939 |
| 223531_x_at | GPR89A /// GPR89B /// GPR89C | G protein-coupled receptor 89A /// G protein-coupled receptor 89B /// G protein- | 8.35E-08 | -8.61062 |
| 209120_at | NR2F2 | nuclear receptor subfamily 2, group F, member 2 | 9.52E-06 | -8.61231 |
| 203580_s_at | SLC7A6 | solute carrier family 7 (cationic amino acid transporter, y+ system), member 6 | 2.19E-07 | -8.61359 |
| 226785_at | ATP11C | ATPase, class VI, type 11C | 7.11E-06 | -8.61363 |
| 223200_s_at | LSG1 | large subunit GTPase 1 homolog (S. cerevisiae) | 2.45E-08 | -8.61392 |
| 228433_at | NFYA | nuclear transcription factor Y, alpha | 6.32E-07 | -8.61521 |
| 215084_s_at | LRRC42 | leucine rich repeat containing 42 | 1.06E-07 | -8.62068 |
| 224744_at | IMPAD1 | inositol monophosphatase domain containing 1 | 1.42E-05 | -8.62213 |
| 214553_s_at | ARPP19 | cAMP-regulated phosphoprotein, 19kDa | 2.24E-06 | -8.62568 |
| 224002_s_at | FKBP7 | FK506 binding protein 7 | 5.15E-06 | -8.62816 |
| 212885_at | MPHOSPH10 | M-phase phosphoprotein 10 (U3 small nucleolar ribonucleoprotein) | 2.98E-06 | -8.62931 |
| 222127_s_at | EXOC1 | exocyst complex component 1 | 9.49E-08 | -8.63096 |
| 200033_at | DDX5 | DEAD (Asp-Glu-Ala-Asp) box polypeptide 5 | 5.84E-07 | -8.63552 |
| 224859_at | CD276 | CD276 molecule | 2.56E-05 | -8.63569 |
| 217317_s_at | HERC2P2 /// HERC2P3 /// LOC440248 | hect domain and RLD 2 pseudogene 2 /// hect domain and RLD 2 pseudogene 3 /// he | 1.60E-07 | -8.6377 |
| 235747_at | SLC25A16 | Solute carrier family 25 (mitochondrial carrier; Graves disease autoantigen), me | 1.29E-06 | -8.64677 |
| 227105_at | CSPP1 | centrosome and spindle pole associated protein 1 | 2.72E-07 | -8.64767 |
| 211404_s_at | APLP2 | amyloid beta (A4) precursor-like protein 2 | 3.64E-06 | -8.64912 |
| 226732_at | RBM33 | RNA binding motif protein 33 | 6.55E-08 | -8.64958 |
| 218370_s_at | S100PBP | S100P binding protein | 9.29E-07 | -8.65341 |
| 200827_at | PLOD1 | procollagen-lysine 1, 2-oxoglutarate 5-dioxygenase 1 | 2.80E-06 | -8.65357 |
| 202464_s_at | PFKFB3 | 6-phosphofructo-2-kinase/fructose-2,6-biphosphatase 3 | 2.64E-06 | -8.65416 |
| 225435_at | SSR1 | signal sequence receptor, alpha | 7.01E-07 | -8.65739 |
| 241396_at | NEDD4L | neural precursor cell expressed, developmentally down-regulated 4-like | 6.84E-07 | -8.65829 |
| 219432_at | EVC | Ellis van Creveld syndrome | 1.15E-06 | -8.65927 |
| 204105_s_at | NRCAM | neuronal cell adhesion molecule | 2.41E-06 | -8.66211 |
| 202731_at | PDCD4 | programmed cell death 4 (neoplastic transformation inhibitor) | 2.50E-06 | -8.66315 |
| 226321_at | LYSMD3 | LysM, putative peptidoglycan-binding, domain containing 3 | 1.14E-06 | -8.66344 |
| 214149_s_at | ATP6V0E1 | ATPase, H+ transporting, lysosomal 9kDa, V0 subunit e1 | 1.63E-05 | -8.66504 |
| 212073_at | CSNK2A1 /// CSNK2A1P | casein kinase 2, alpha 1 polypeptide /// casein kinase 2, alpha 1 polypeptide ps | 2.18E-06 | -8.66834 |
| 211084_x_at | PRKD3 | protein kinase D3 | 3.55E-07 | -8.67249 |
| 229650_s_at | C19orf42 | chromosome 19 open reading frame 42 | 2.04E-07 | -8.67403 |
| 227263_at | C8orf58 | chromosome 8 open reading frame 58 | 2.50E-06 | -8.67464 |
| 225402_at | TP53RK | TP53 regulating kinase | 1.98E-06 | -8.67482 |
| 203367_at | DUSP14 | dual specificity phosphatase 14 | 1.38E-07 | -8.67812 |
| 201167_x_at | ARHGDIA | Rho GDP dissociation inhibitor (GDI) alpha | 3.28E-06 | -8.67917 |
| 209572_s_at | EED | embryonic ectoderm development | 6.07E-07 | -8.68097 |
| 235401_s_at | FCRLA | Fc receptor-like A | 5.14E-06 | -8.68367 |
| 59644_at | BMP2K | BMP2 inducible kinase | 2.52E-05 | -8.68683 |
| 207785_s_at | RBPJ | recombination signal binding protein for immunoglobulin kappa J region | 1.41E-06 | -8.6922 |
| 201995_at | EXT1 | exostosin 1 | 1.27E-10 | -8.69368 |
| 230656_s_at | CIRH1A | cirrhosis, autosomal recessive 1A (cirhin) | 4.54E-08 | -8.6937 |
| 224535_s_at | MRP63 | mitochondrial ribosomal protein 63 | 4.70E-06 | -8.69713 |
| 227692_at | GNAI1 | guanine nucleotide binding protein (G protein), alpha inhibiting activity polype | 1.38E-06 | -8.69788 |
| 203717_at | DPP4 | dipeptidyl-peptidase 4 | 2.48E-07 | -8.69956 |
| 208765_s_at | HNRNPR | heterogeneous nuclear ribonucleoprotein R | 7.27E-09 | -8.69995 |
| 205079_s_at | MPDZ | multiple PDZ domain protein | 1.88E-08 | -8.70422 |
| 224994_at | CAMK2D | calcium/calmodulin-dependent protein kinase II delta | 6.72E-06 | -8.71353 |
| 206052_s_at | SLBP | stem-loop binding protein | 3.29E-06 | -8.71874 |
| 202746_at | ITM2A | integral membrane protein 2A | 1.84E-05 | -8.71943 |
| 201975_at | CLIP1 | CAP-GLY domain containing linker protein 1 | 3.39E-06 | -8.72434 |
| 223474_at | C14orf4 | chromosome 14 open reading frame 4 | 9.70E-08 | -8.72535 |
| 201668_x_at | MARCKS | myristoylated alanine-rich protein kinase C substrate | 1.19E-05 | -8.72604 |
| 201898_s_at | UBE2A | ubiquitin-conjugating enzyme E2A (RAD6 homolog) | 3.56E-07 | -8.72614 |
| 212271_at | MAPK1 | mitogen-activated protein kinase 1 | 2.36E-06 | -8.72627 |
| 200712_s_at | MAPRE1 | microtubule-associated protein, RP/EB family, member 1 | 1.57E-06 | -8.72912 |
| 202073_at | OPTN | optineurin | 1.99E-05 | -8.73014 |
| 226635_at | LOC401504 | Hypothetical gene supported by AK091718 | 9.39E-07 | -8.73068 |
| 218184_at | TULP4 | tubby like protein 4 | 2.19E-05 | -8.7329 |
| 203511_s_at | TRAPPC3 | trafficking protein particle complex 3 | 9.60E-09 | -8.73461 |
| 201448_at | TIA1 | TIA1 cytotoxic granule-associated RNA binding protein | 5.77E-06 | -8.73799 |
| 211996_s_at | LOC100132247 /// LOC348162 /// LOC613037 /// LOC728888 /// NPIPL3 | similar to Uncharacterized protein KIAA0220 /// hypothetical protein 348162 /// | 1.98E-08 | -8.74115 |
| 221688_s_at | IMP3 | IMP3, U3 small nucleolar ribonucleoprotein, homolog (yeast) | 1.62E-10 | -8.74588 |
| 202131_s_at | RIOK3 | RIO kinase 3 (yeast) | 3.24E-05 | -8.74659 |
| 200891_s_at | SSR1 | signal sequence receptor, alpha | 5.44E-06 | -8.75044 |
| 200699_at | KDELR2 | KDEL (Lys-Asp-Glu-Leu) endoplasmic reticulum protein retention receptor 2 | 1.26E-07 | -8.75315 |
| 203087_s_at | KIF2A | kinesin heavy chain member 2A | 6.52E-06 | -8.75411 |
| 211068_x_at | FAM21C /// FAM21D | family with sequence similarity 21, member C /// family with sequence similarity | 8.91E-08 | -8.75437 |
| 215034_s_at | TM4SF1 | transmembrane 4 L six family member 1 | 1.14E-06 | -8.76085 |
| 232902_s_at | RARS2 | arginyl-tRNA synthetase 2, mitochondrial | 3.38E-05 | -8.763 |
| 225492_at | TMEM33 | transmembrane protein 33 | 4.51E-09 | -8.76429 |
| 201486_at | RCN2 | reticulocalbin 2, EF-hand calcium binding domain | 2.41E-07 | -8.76464 |
| 203675_at | NUCB2 | nucleobindin 2 | 8.04E-07 | -8.76602 |
| 209184_s_at | IRS2 | insulin receptor substrate 2 | 5.39E-07 | -8.76722 |
| 233186_s_at | BANP | BTG3 associated nuclear protein | 2.33E-07 | -8.77164 |
| 214499_s_at | BCLAF1 | BCL2-associated transcription factor 1 | 2.41E-06 | -8.7734 |
| 238346_s_at | TGS1 | trimethylguanosine synthase homolog (S. cerevisiae) | 1.31E-07 | -8.77483 |
| 213457_at | MFHAS1 | malignant fibrous histiocytoma amplified sequence 1 | 4.17E-05 | -8.78179 |
| 203659_s_at | TRIM13 | tripartite motif-containing 13 | 4.58E-07 | -8.78383 |
| 221983_at | FAM134A | family with sequence similarity 134, member A | 4.81E-07 | -8.78432 |
| 213117_at | KLHL9 | kelch-like 9 (Drosophila) | 2.54E-05 | -8.78921 |
| 222646_s_at | ERO1L | ERO1-like (S. cerevisiae) | 1.67E-05 | -8.78934 |
| 212006_at | UBXN4 | UBX domain protein 4 | 1.86E-06 | -8.79095 |
| 225407_at | MBP | myelin basic protein | 2.76E-07 | -8.79124 |
| 204699_s_at | C1orf107 | chromosome 1 open reading frame 107 | 2.11E-05 | -8.79156 |
| 228293_at | DEPDC7 | DEP domain containing 7 | 5.47E-05 | -8.7917 |
| 213698_at | ZMYM6 | zinc finger, MYM-type 6 | 1.71E-07 | -8.79498 |
| 242837_at | SFRS4 | Splicing factor, arginine/serine-rich 4 | 7.37E-08 | -8.79593 |
| 213702_x_at | ASAH1 | N-acylsphingosine amidohydrolase (acid ceramidase) 1 | 2.98E-08 | -8.79621 |
| 203196_at | ABCC4 | ATP-binding cassette, sub-family C (CFTR/MRP), member 4 | 5.58E-09 | -8.80276 |
| 200096_s_at | ATP6V0E1 | ATPase, H+ transporting, lysosomal 9kDa, V0 subunit e1 | 3.12E-07 | -8.80555 |
| 215794_x_at | GLUD2 | glutamate dehydrogenase 2 | 6.74E-08 | -8.80816 |
| 201087_at | PXN | paxillin | 1.10E-07 | -8.8118 |
| 203392_s_at | CTBP1 | C-terminal binding protein 1 | 1.31E-07 | -8.81601 |
| 209086_x_at | MCAM | melanoma cell adhesion molecule | 2.64E-05 | -8.81761 |
| 1555403_a_at | CDH19 | cadherin 19, type 2 | 5.66E-06 | -8.82023 |
| 212416_at | SCAMP1 | secretory carrier membrane protein 1 | 2.46E-06 | -8.82215 |
| 218001_at | MRPS2 | mitochondrial ribosomal protein S2 | 3.12E-06 | -8.82241 |
| 225294_s_at | TRAPPC1 | trafficking protein particle complex 1 | 6.20E-06 | -8.82245 |
| 202413_s_at | USP1 | ubiquitin specific peptidase 1 | 4.13E-07 | -8.82633 |
| 233030_at | PNPLA3 | patatin-like phospholipase domain containing 3 | 5.03E-08 | -8.82832 |
| 229889_at | C17orf76 | chromosome 17 open reading frame 76 | 3.37E-07 | -8.8294 |
| 204684_at | NPTX1 | neuronal pentraxin I | 3.74E-06 | -8.83171 |
| 211945_s_at | ITGB1 | integrin, beta 1 (fibronectin receptor, beta polypeptide, antigen CD29 includes | 9.11E-07 | -8.83189 |
| 224787_s_at | RAB18 | RAB18, member RAS oncogene family | 1.46E-07 | -8.83208 |
| 225761_at | PAPD4 | PAP associated domain containing 4 | 2.51E-06 | -8.83326 |
| 1554149_at | CLDND1 | claudin domain containing 1 | 2.38E-05 | -8.83673 |
| 203068_at | KLHL21 | kelch-like 21 (Drosophila) | 7.08E-07 | -8.83748 |
| 201499_s_at | USP7 | ubiquitin specific peptidase 7 (herpes virus-associated) | 3.92E-07 | -8.84066 |
| 204369_at | PIK3CA | phosphoinositide-3-kinase, catalytic, alpha polypeptide | 4.50E-06 | -8.84124 |
| 218740_s_at | CDK5RAP3 | CDK5 regulatory subunit associated protein 3 | 1.42E-07 | -8.84486 |
| 200805_at | LMAN2 | lectin, mannose-binding 2 | 9.79E-07 | -8.84764 |
| 209015_s_at | DNAJB6 | DnaJ (Hsp40) homolog, subfamily B, member 6 | 7.27E-08 | -8.84926 |
| 212290_at | SLC7A1 | solute carrier family 7 (cationic amino acid transporter, y+ system), member 1 | 4.31E-08 | -8.84944 |
| 211535_s_at | FGFR1 | fibroblast growth factor receptor 1 | 1.03E-06 | -8.84978 |
| 218203_at | ALG5 | asparagine-linked glycosylation 5, dolichyl-phosphate beta-glucosyltransferase h | 1.25E-06 | -8.85397 |
| 227639_at | PIGK | phosphatidylinositol glycan anchor biosynthesis, class K | 1.05E-07 | -8.85669 |
| 203884_s_at | RAB11FIP2 | RAB11 family interacting protein 2 (class I) | 2.46E-06 | -8.85701 |
| 202153_s_at | NUP62 | nucleoporin 62kDa | 6.50E-08 | -8.86021 |
| 205227_at | IL1RAP | interleukin 1 receptor accessory protein | 6.49E-06 | -8.86592 |
| 200905_x_at | HLA-E | major histocompatibility complex, class I, E | 8.27E-09 | -8.86787 |
| 211985_s_at | CALM1 | calmodulin 1 (phosphorylase kinase, delta) | 3.25E-08 | -8.87378 |
| 208620_at | PCBP1 | poly(rC) binding protein 1 | 5.17E-08 | -8.881 |
| 226503_at | RIF1 | RAP1 interacting factor homolog (yeast) | 1.20E-05 | -8.88632 |
| 225588_s_at | TMEM129 | transmembrane protein 129 | 5.17E-06 | -8.88634 |
| 203311_s_at | ARF6 | ADP-ribosylation factor 6 | 2.44E-06 | -8.88832 |
| 202230_s_at | CHERP | calcium homeostasis endoplasmic reticulum protein | 2.04E-06 | -8.89336 |
| 214730_s_at | GLG1 | golgi glycoprotein 1 | 1.03E-08 | -8.89547 |
| 205029_s_at | FABP7 | fatty acid binding protein 7, brain | 2.44E-07 | -8.90306 |
| 225125_at | MMGT1 | membrane magnesium transporter 1 | 4.69E-08 | -8.9038 |
| 203583_at | UNC50 | unc-50 homolog (C. elegans) | 2.09E-07 | -8.91011 |
| 200798_x_at | MCL1 | myeloid cell leukemia sequence 1 (BCL2-related) | 5.40E-06 | -8.91015 |
| 216092_s_at | SLC7A8 | solute carrier family 7 (amino acid transporter, L-type), member 8 | 1.39E-05 | -8.91782 |
| 217523_at | CD44 | CD44 molecule (Indian blood group) | 1.98E-05 | -8.91839 |
| 225032_at | FNDC3B | fibronectin type III domain containing 3B | 1.74E-06 | -8.91982 |
| 233665_x_at | MTO1 | mitochondrial translation optimization 1 homolog (S. cerevisiae) | 6.23E-07 | -8.92165 |
| 225180_at | TTC14 | tetratricopeptide repeat domain 14 | 1.40E-05 | -8.92352 |
| 203127_s_at | SPTLC2 | serine palmitoyltransferase, long chain base subunit 2 | 9.78E-07 | -8.92839 |
| 225634_at | ZC3HAV1 | zinc finger CCCH-type, antiviral 1 | 3.93E-05 | -8.92877 |
| 1553954_at | ALG14 | asparagine-linked glycosylation 14 homolog (S. cerevisiae) | 3.83E-06 | -8.93029 |
| 201182_s_at | CHD4 | chromodomain helicase DNA binding protein 4 | 6.27E-08 | -8.93328 |
| 200700_s_at | KDELR2 | KDEL (Lys-Asp-Glu-Leu) endoplasmic reticulum protein retention receptor 2 | 1.36E-07 | -8.93606 |
| 202615_at | GNAQ | Guanine nucleotide binding protein (G protein), q polypeptide | 8.73E-08 | -8.93979 |
| 225424_at | GPAM | glycerol-3-phosphate acyltransferase, mitochondrial | 8.15E-06 | -8.94112 |
| 201198_s_at | PSMD1 | proteasome (prosome, macropain) 26S subunit, non-ATPase, 1 | 1.26E-07 | -8.94149 |
| 222065_s_at | FLII | flightless I homolog (Drosophila) | 2.54E-06 | -8.94308 |
| 205633_s_at | ALAS1 | aminolevulinate, delta-, synthase 1 | 1.21E-07 | -8.94828 |
| 218249_at | ZDHHC6 | zinc finger, DHHC-type containing 6 | 1.34E-06 | -8.9507 |
| 203100_s_at | CDYL | chromodomain protein, Y-like | 5.54E-05 | -8.95231 |
| 235060_at | LOC100190986 | hypothetical LOC100190986 | 8.61E-06 | -8.95517 |
| 204355_at | DHX30 | DEAH (Asp-Glu-Ala-His) box polypeptide 30 | 4.49E-08 | -8.95684 |
| 224917_at | MIR21 | microRNA 21 | 2.89E-05 | -8.9572 |
| 1552790_a_at | SEC62 | SEC62 homolog (S. cerevisiae) | 6.69E-06 | -8.95874 |
| 200766_at | CTSD | cathepsin D | 1.88E-06 | -8.96381 |
| 226350_at | CHML | choroideremia-like (Rab escort protein 2) | 2.18E-07 | -8.97743 |
| 218921_at | SIGIRR | single immunoglobulin and toll-interleukin 1 receptor (TIR) domain | 3.81E-07 | -8.97769 |
| 214933_at | CACNA1A | calcium channel, voltage-dependent, P/Q type, alpha 1A subunit | 9.99E-07 | -8.97934 |
| 218319_at | PELI1 | pellino homolog 1 (Drosophila) | 8.56E-07 | -8.98107 |
| 209520_s_at | NCBP1 | nuclear cap binding protein subunit 1, 80kDa | 5.68E-08 | -8.98916 |
| 223206_s_at | NMRAL1 | NmrA-like family domain containing 1 | 7.12E-08 | -8.99273 |
| 214459_x_at | HLA-C | major histocompatibility complex, class I, C | 2.15E-07 | -8.99422 |
| 225381_at | LOC399959 | hypothetical LOC399959 | 4.19E-08 | -8.99698 |
| 200757_s_at | CALU | calumenin | 5.38E-06 | -9.00221 |
| 226372_at | CHST11 | carbohydrate (chondroitin 4) sulfotransferase 11 | 3.01E-07 | -9.00301 |
| 214196_s_at | TPP1 | tripeptidyl peptidase I | 1.32E-06 | -9.0033 |
| 218428_s_at | REV1 | REV1 homolog (S. cerevisiae) | 5.04E-05 | -9.00778 |
| 201548_s_at | KDM5B | lysine (K)-specific demethylase 5B | 4.14E-07 | -9.00855 |
| 216526_x_at | HLA-C | major histocompatibility complex, class I, C | 9.82E-08 | -9.01009 |
| 212761_at | TCF7L2 | transcription factor 7-like 2 (T-cell specific, HMG-box) | 1.74E-06 | -9.01056 |
| 210338_s_at | HSPA8 | heat shock 70kDa protein 8 | 4.18E-09 | -9.01111 |
| 201478_s_at | DKC1 | dyskeratosis congenita 1, dyskerin | 7.12E-08 | -9.0124 |
| 58780_s_at | FLJ10357 | protein SOLO | 5.04E-07 | -9.01376 |
| 209199_s_at | MEF2C | myocyte enhancer factor 2C | 5.85E-06 | -9.01423 |
| 211804_s_at | CDK2 | cyclin-dependent kinase 2 | 1.06E-06 | -9.0184 |
| 223098_s_at | LONP2 | lon peptidase 2, peroxisomal | 2.91E-07 | -9.02697 |
| 200883_at | UQCRC2 | ubiquinol-cytochrome c reductase core protein II | 9.35E-07 | -9.0291 |
| 212321_at | SGPL1 | sphingosine-1-phosphate lyase 1 | 3.18E-06 | -9.03273 |
| 210145_at | PLA2G4A | phospholipase A2, group IVA (cytosolic, calcium-dependent) | 3.01E-07 | -9.03274 |
| 208710_s_at | AP3D1 | adaptor-related protein complex 3, delta 1 subunit | 3.22E-07 | -9.03299 |
| 204616_at | UCHL3 | ubiquitin carboxyl-terminal esterase L3 (ubiquitin thiolesterase) | 3.94E-09 | -9.03521 |
| 201894_s_at | SSR1 | signal sequence receptor, alpha | 3.60E-08 | -9.03584 |
| 200769_s_at | MAT2A | methionine adenosyltransferase II, alpha | 1.97E-07 | -9.03629 |
| 229812_at | USP48 | ubiquitin specific peptidase 48 | 5.55E-07 | -9.03785 |
| 224790_at | ASAP1 | ArfGAP with SH3 domain, ankyrin repeat and PH domain 1 | 3.65E-08 | -9.03919 |
| 208964_s_at | FADS1 | fatty acid desaturase 1 | 2.41E-05 | -9.04214 |
| 221899_at | N4BP2L2 | NEDD4 binding protein 2-like 2 | 1.41E-05 | -9.06552 |
| 202269_x_at | GBP1 | guanylate binding protein 1, interferon-inducible, 67kDa | 1.26E-07 | -9.06856 |
| 212802_s_at | GAPVD1 | GTPase activating protein and VPS9 domains 1 | 1.16E-07 | -9.07035 |
| 202354_s_at | GTF2F1 | general transcription factor IIF, polypeptide 1, 74kDa | 3.70E-06 | -9.07161 |
| 203323_at | CAV2 | caveolin 2 | 1.05E-06 | -9.07406 |
| 217832_at | SYNCRIP | synaptotagmin binding, cytoplasmic RNA interacting protein | 1.39E-08 | -9.08116 |
| 207543_s_at | P4HA1 | prolyl 4-hydroxylase, alpha polypeptide I | 3.74E-05 | -9.08618 |
| 202548_s_at | ARHGEF7 | Rho guanine nucleotide exchange factor (GEF) 7 | 1.47E-07 | -9.08743 |
| 213729_at | PRPF40A | PRP40 pre-mRNA processing factor 40 homolog A (S. cerevisiae) | 6.67E-07 | -9.08786 |
| 204524_at | PDPK1 | 3-phosphoinositide dependent protein kinase-1 | 9.94E-09 | -9.09043 |
| 217787_s_at | GALNT2 | UDP-N-acetyl-alpha-D-galactosamine:polypeptide N-acetylgalactosaminyltransferase | 1.05E-06 | -9.093 |
| 218386_x_at | USP16 | ubiquitin specific peptidase 16 | 7.70E-07 | -9.0942 |
| 201266_at | TXNRD1 | thioredoxin reductase 1 | 2.39E-10 | -9.09425 |
| 1554807_a_at | SPIRE1 | spire homolog 1 (Drosophila) | 4.73E-07 | -9.10733 |
| 234982_at | UBR3 | ubiquitin protein ligase E3 component n-recognin 3 (putative) | 1.09E-06 | -9.11072 |
| 209834_at | CHST3 | carbohydrate (chondroitin 6) sulfotransferase 3 | 8.52E-08 | -9.11379 |
| 217957_at | C16orf80 | chromosome 16 open reading frame 80 | 1.40E-06 | -9.11734 |
| 229394_s_at | GRLF1 | glucocorticoid receptor DNA binding factor 1 | 4.61E-05 | -9.12138 |
| 241966_at | MYO5A | myosin VA (heavy chain 12, myoxin) | 2.64E-06 | -9.12188 |
| 201086_x_at | SON | SON DNA binding protein | 5.87E-07 | -9.12342 |
| 222912_at | ARRB1 | arrestin, beta 1 | 2.25E-07 | -9.1237 |
| 233461_x_at | ZNF226 | zinc finger protein 226 | 6.40E-07 | -9.12394 |
| 1554821_a_at | ZBED1 | zinc finger, BED-type containing 1 | 8.80E-07 | -9.1266 |
| 218028_at | ELOVL1 | elongation of very long chain fatty acids (FEN1/Elo2, SUR4/Elo3, yeast)-like 1 | 3.96E-07 | -9.12812 |
| 208706_s_at | EIF5 | eukaryotic translation initiation factor 5 | 3.56E-08 | -9.12913 |
| 203491_s_at | CEP57 | centrosomal protein 57kDa | 8.66E-06 | -9.13013 |
| 204373_s_at | CEP350 | centrosomal protein 350kDa | 2.26E-06 | -9.13255 |
| 226161_at | SLC30A6 | solute carrier family 30 (zinc transporter), member 6 | 1.01E-05 | -9.14173 |
| 218331_s_at | C10orf18 | chromosome 10 open reading frame 18 | 1.31E-07 | -9.14338 |
| 210839_s_at | ENPP2 | ectonucleotide pyrophosphatase/phosphodiesterase 2 | 4.64E-07 | -9.14492 |
| 201069_at | MMP2 | matrix metallopeptidase 2 (gelatinase A, 72kDa gelatinase, 72kDa type IV collage | 4.69E-07 | -9.1457 |
| 225512_at | ZBTB38 | zinc finger and BTB domain containing 38 | 4.86E-08 | -9.14579 |
| 204083_s_at | TPM2 | tropomyosin 2 (beta) | 2.88E-06 | -9.14636 |
| 208760_at | UBE2I | Ubiquitin-conjugating enzyme E2I (UBC9 homolog, yeast) | 4.15E-08 | -9.15251 |
| 219433_at | BCOR | BCL6 co-repressor | 4.99E-05 | -9.15254 |
| 209398_at | HIST1H1C | histone cluster 1, H1c | 8.01E-06 | -9.1566 |
| 217869_at | HSD17B12 | hydroxysteroid (17-beta) dehydrogenase 12 | 1.11E-08 | -9.17461 |
| 203217_s_at | ST3GAL5 | ST3 beta-galactoside alpha-2,3-sialyltransferase 5 | 5.84E-08 | -9.17706 |
| 218732_at | PTRH2 | peptidyl-tRNA hydrolase 2 | 1.39E-08 | -9.17934 |
| 209336_at | PWP2 | PWP2 periodic tryptophan protein homolog (yeast) | 3.17E-07 | -9.18282 |
| 217208_s_at | DLG1 | discs, large homolog 1 (Drosophila) | 2.85E-06 | -9.18642 |
| 224559_at | MALAT1 | metastasis associated lung adenocarcinoma transcript 1 (non-protein coding) | 0.003086 | -9.19217 |
| 204975_at | EMP2 | epithelial membrane protein 2 | 1.60E-08 | -9.19243 |
| 222439_s_at | THRAP3 | thyroid hormone receptor associated protein 3 | 2.32E-06 | -9.19373 |
| 203120_at | TP53BP2 | tumor protein p53 binding protein, 2 | 1.53E-07 | -9.19443 |
| 204824_at | ENDOG | endonuclease G | 5.99E-07 | -9.21237 |
| 225688_s_at | PHLDB2 | pleckstrin homology-like domain, family B, member 2 | 2.04E-05 | -9.2158 |
| 229410_at | SLC35E1 | solute carrier family 35, member E1 | 2.47E-07 | -9.22057 |
| 214895_s_at | ADAM10 | ADAM metallopeptidase domain 10 | 7.40E-06 | -9.22371 |
| 227401_at | IL17D | interleukin 17D | 1.10E-07 | -9.22779 |
| 215716_s_at | ATP2B1 | ATPase, Ca++ transporting, plasma membrane 1 | 1.78E-06 | -9.22848 |
| 224900_at | ANKFY1 | ankyrin repeat and FYVE domain containing 1 | 1.08E-07 | -9.22926 |
| 203857_s_at | PDIA5 | protein disulfide isomerase family A, member 5 | 1.90E-06 | -9.23582 |
| 224624_at | LRRC8A | leucine rich repeat containing 8 family, member A | 2.74E-07 | -9.2378 |
| 204897_at | PTGER4 | prostaglandin E receptor 4 (subtype EP4) | 5.32E-06 | -9.23792 |
| 200052_s_at | ILF2 | interleukin enhancer binding factor 2, 45kDa | 1.39E-09 | -9.23816 |
| 213470_s_at | HNRNPH1 | heterogeneous nuclear ribonucleoprotein H1 (H) | 3.05E-06 | -9.23875 |
| 214882_s_at | SFRS2 | splicing factor, arginine/serine-rich 2 | 1.85E-08 | -9.24517 |
| 202656_s_at | SERTAD2 | SERTA domain containing 2 | 3.32E-08 | -9.24532 |
| 226460_at | FNIP2 | folliculin interacting protein 2 | 2.21E-08 | -9.24734 |
| 203301_s_at | DMTF1 | cyclin D binding myb-like transcription factor 1 | 3.01E-05 | -9.25393 |
| 209868_s_at | RBMS1 | RNA binding motif, single stranded interacting protein 1 | 2.97E-11 | -9.25496 |
| 212158_at | SDC2 | syndecan 2 | 1.43E-06 | -9.25852 |
| 215501_s_at | DUSP10 | dual specificity phosphatase 10 | 7.86E-07 | -9.2612 |
| 200867_at | RNF114 | ring finger protein 114 | 2.64E-07 | -9.26758 |
| 219649_at | ALG6 | asparagine-linked glycosylation 6, alpha-1,3-glucosyltransferase homolog (S. cer | 8.13E-07 | -9.26824 |
| 200617_at | MLEC | malectin | 8.82E-07 | -9.26882 |
| 225043_at | SLC15A4 | solute carrier family 15, member 4 | 2.45E-07 | -9.26882 |
| 200641_s_at | YWHAZ | tyrosine 3-monooxygenase/tryptophan 5-monooxygenase activation protein, zeta pol | 1.52E-05 | -9.27128 |
| 203636_at | MID1 | midline 1 (Opitz/BBB syndrome) | 2.38E-10 | -9.27134 |
| 221970_s_at | NOL11 | nucleolar protein 11 | 2.32E-05 | -9.27281 |
| 202359_s_at | SNX19 | sorting nexin 19 | 1.25E-05 | -9.2769 |
| 202123_s_at | ABL1 | c-abl oncogene 1, receptor tyrosine kinase | 2.55E-06 | -9.27848 |
| 222442_s_at | ARL8B | ADP-ribosylation factor-like 8B | 5.70E-08 | -9.28074 |
| 201561_s_at | CLSTN1 | calsyntenin 1 | 1.68E-07 | -9.28484 |
| 212747_at | ANKS1A | ankyrin repeat and sterile alpha motif domain containing 1A | 4.38E-09 | -9.29359 |
| 209953_s_at | CDC37 | cell division cycle 37 homolog (S. cerevisiae) | 5.44E-14 | -9.29388 |
| 222587_s_at | GALNT7 | UDP-N-acetyl-alpha-D-galactosamine:polypeptide N-acetylgalactosaminyltransferase | 5.96E-07 | -9.29405 |
| 223216_x_at | ZNF395 | zinc finger protein 395 | 3.37E-07 | -9.29492 |
| 225376_at | C20orf11 | chromosome 20 open reading frame 11 | 8.47E-09 | -9.30162 |
| 222480_at | UBE2Q1 | ubiquitin-conjugating enzyme E2Q family member 1 | 1.59E-08 | -9.30437 |
| 221782_at | DNAJC10 | DnaJ (Hsp40) homolog, subfamily C, member 10 | 6.34E-05 | -9.3093 |
| 209088_s_at | UBN1 | ubinuclein 1 | 9.91E-07 | -9.31572 |
| 234660_s_at | DIS3 | DIS3 mitotic control homolog (S. cerevisiae) | 2.61E-07 | -9.31629 |
| 226554_at | ZBTB7A | zinc finger and BTB domain containing 7A | 7.54E-08 | -9.31656 |
| 228920_at | ZNF260 | zinc finger protein 260 | 3.50E-08 | -9.31881 |
| 228280_at | ZC3HAV1L | zinc finger CCCH-type, antiviral 1-like | 5.44E-06 | -9.32862 |
| 204805_s_at | H1FX | H1 histone family, member X | 3.90E-06 | -9.33189 |
| 208729_x_at | HLA-B | major histocompatibility complex, class I, B | 1.86E-07 | -9.33339 |
| 224922_at | CSNK2A2 | casein kinase 2, alpha prime polypeptide | 2.34E-08 | -9.34251 |
| 1556285_s_at | PPA2 | pyrophosphatase (inorganic) 2 | 1.96E-07 | -9.34263 |
| 202407_s_at | PRPF31 | PRP31 pre-mRNA processing factor 31 homolog (S. cerevisiae) | 2.95E-06 | -9.34331 |
| 208633_s_at | MACF1 | microtubule-actin crosslinking factor 1 | 4.38E-06 | -9.3466 |
| 224628_at | ERLEC1 | endoplasmic reticulum lectin 1 | 4.97E-08 | -9.34686 |
| 201566_x_at | ID2 | inhibitor of DNA binding 2, dominant negative helix-loop-helix protein | 7.94E-07 | -9.34879 |
| 209682_at | CBLB | Cas-Br-M (murine) ecotropic retroviral transforming sequence b | 5.59E-07 | -9.34946 |
| 237651_x_at | C6orf218 | chromosome 6 open reading frame 218 | 3.84E-06 | -9.3503 |
| 219375_at | CEPT1 | choline/ethanolamine phosphotransferase 1 | 2.14E-08 | -9.351 |
| 200940_s_at | RERE | arginine-glutamic acid dipeptide (RE) repeats | 2.81E-07 | -9.35156 |
| 233085_s_at | OBFC2A | oligonucleotide/oligosaccharide-binding fold containing 2A | 5.00E-06 | -9.35237 |
| 212570_at | ENDOD1 | endonuclease domain containing 1 | 4.40E-07 | -9.35324 |
| 1557953_at | ZKSCAN1 | zinc finger with KRAB and SCAN domains 1 | 1.01E-08 | -9.36052 |
| 1555058_a_at | LPGAT1 | lysophosphatidylglycerol acyltransferase 1 | 7.55E-06 | -9.36886 |
| 202857_at | CNPY2 | canopy 2 homolog (zebrafish) | 4.71E-07 | -9.36943 |
| 204262_s_at | PSEN2 | presenilin 2 (Alzheimer disease 4) | 5.79E-07 | -9.36978 |
| 203083_at | THBS2 | thrombospondin 2 | 5.21E-06 | -9.37206 |
| 221702_s_at | TM2D3 | TM2 domain containing 3 | 6.29E-09 | -9.37495 |
| 206544_x_at | SMARCA2 | SWI/SNF related, matrix associated, actin dependent regulator of chromatin, subf | 6.29E-07 | -9.37712 |
| 203553_s_at | MAP4K5 | mitogen-activated protein kinase kinase kinase kinase 5 | 1.60E-06 | -9.37733 |
| 235142_at | ZBTB8A | zinc finger and BTB domain containing 8A | 2.22E-06 | -9.37839 |
| 218803_at | CHFR | checkpoint with forkhead and ring finger domains | 2.96E-07 | -9.38474 |
| 206710_s_at | EPB41L3 | erythrocyte membrane protein band 4.1-like 3 | 6.13E-05 | -9.38502 |
| 222456_s_at | LIMA1 | LIM domain and actin binding 1 | 5.50E-05 | -9.39315 |
| 205005_s_at | NMT2 | N-myristoyltransferase 2 | 1.91E-05 | -9.39451 |
| 201593_s_at | ZC3H15 | zinc finger CCCH-type containing 15 | 5.55E-06 | -9.39572 |
| 228181_at | SLC30A1 | solute carrier family 30 (zinc transporter), member 1 | 5.58E-08 | -9.39942 |
| 221669_s_at | ACAD8 | acyl-CoA dehydrogenase family, member 8 | 9.86E-06 | -9.40603 |
| 219026_s_at | RASAL2 | RAS protein activator like 2 | 4.31E-07 | -9.40656 |
| 229900_at | CD109 | CD109 molecule | 5.62E-07 | -9.40709 |
| 202499_s_at | SLC2A3 | solute carrier family 2 (facilitated glucose transporter), member 3 | 8.65E-09 | -9.40738 |
| 203981_s_at | ABCD4 | ATP-binding cassette, sub-family D (ALD), member 4 | 5.68E-09 | -9.4076 |
| 1568618_a_at | GALNT1 | UDP-N-acetyl-alpha-D-galactosamine:polypeptide N-acetylgalactosaminyltransferase | 1.93E-08 | -9.41417 |
| 205715_at | BST1 | bone marrow stromal cell antigen 1 | 3.83E-08 | -9.4159 |
| 217836_s_at | YY1AP1 | YY1 associated protein 1 | 3.16E-06 | -9.41713 |
| 212201_at | ANKLE2 | ankyrin repeat and LEM domain containing 2 | 5.55E-06 | -9.42119 |
| 203574_at | NFIL3 | nuclear factor, interleukin 3 regulated | 4.29E-07 | -9.42451 |
| 204203_at | CEBPG | CCAAT/enhancer binding protein (C/EBP), gamma | 4.57E-05 | -9.42745 |
| 219681_s_at | RAB11FIP1 | RAB11 family interacting protein 1 (class I) | 1.96E-07 | -9.42908 |
| 210974_s_at | AP3D1 | adaptor-related protein complex 3, delta 1 subunit | 3.23E-06 | -9.43037 |
| 201549_x_at | KDM5B | lysine (K)-specific demethylase 5B | 6.95E-08 | -9.43107 |
| 218402_s_at | HPS4 | Hermansky-Pudlak syndrome 4 | 9.23E-07 | -9.43496 |
| 214703_s_at | MAN2B2 | mannosidase, alpha, class 2B, member 2 | 6.15E-07 | -9.44218 |
| 210589_s_at | GBAP1 | glucosidase, beta, acid pseudogene 1 | 6.28E-08 | -9.44225 |
| 225288_at | COL27A1 | collagen, type XXVII, alpha 1 | 2.16E-06 | -9.44603 |
| 221123_x_at | ZNF395 | zinc finger protein 395 | 2.74E-08 | -9.44741 |
| 211257_x_at | ZNF638 | zinc finger protein 638 | 1.88E-07 | -9.44866 |
| 222445_at | SLC39A9 | solute carrier family 39 (zinc transporter), member 9 | 1.64E-09 | -9.45863 |
| 214527_s_at | PQBP1 | polyglutamine binding protein 1 | 1.38E-05 | -9.46318 |
| 213262_at | SACS | spastic ataxia of Charlevoix-Saguenay (sacsin) | 9.04E-06 | -9.46605 |
| 212665_at | TIPARP | TCDD-inducible poly(ADP-ribose) polymerase | 2.07E-07 | -9.46716 |
| 202396_at | TCERG1 | transcription elongation regulator 1 | 2.96E-07 | -9.46772 |
| 209162_s_at | PRPF4 | PRP4 pre-mRNA processing factor 4 homolog (yeast) | 4.56E-06 | -9.47994 |
| 207614_s_at | CUL1 | cullin 1 | 3.16E-07 | -9.48021 |
| 233167_at | SELO | selenoprotein O | 2.32E-07 | -9.48135 |
| 212418_at | ELF1 | E74-like factor 1 (ets domain transcription factor) | 2.32E-07 | -9.489 |
| 225922_at | FNIP2 | folliculin interacting protein 2 | 5.29E-07 | -9.48976 |
| 222457_s_at | LIMA1 | LIM domain and actin binding 1 | 2.19E-08 | -9.49017 |
| 203827_at | WIPI1 | WD repeat domain, phosphoinositide interacting 1 | 1.51E-06 | -9.49046 |
| 207983_s_at | STAG2 | stromal antigen 2 | 2.94E-07 | -9.49183 |
| 1555945_s_at | FAM120A | family with sequence similarity 120A | 9.70E-07 | -9.49328 |
| 208454_s_at | PGCP | plasma glutamate carboxypeptidase | 1.22E-06 | -9.50305 |
| 212508_at | MOAP1 | modulator of apoptosis 1 | 4.41E-06 | -9.50763 |
| 206854_s_at | MAP3K7 | mitogen-activated protein kinase kinase kinase 7 | 1.05E-07 | -9.5099 |
| 201346_at | ADIPOR2 | adiponectin receptor 2 | 1.64E-06 | -9.51561 |
| 219129_s_at | SAP30L | SAP30-like | 8.82E-08 | -9.52202 |
| 224799_at | NDFIP2 | Nedd4 family interacting protein 2 | 3.54E-06 | -9.52346 |
| 218515_at | GCFC1 | GC-rich sequence DNA-binding factor 1 | 2.65E-07 | -9.53225 |
| 225448_at | NAPG | N-ethylmaleimide-sensitive factor attachment protein, gamma | 7.74E-05 | -9.5323 |
| 226850_at | SUMF1 | sulfatase modifying factor 1 | 1.82E-07 | -9.53423 |
| 208811_s_at | DNAJB6 /// TMEM135 | DnaJ (Hsp40) homolog, subfamily B, member 6 /// transmembrane protein 135 | 7.42E-07 | -9.53509 |
| 209234_at | KIF1B | kinesin family member 1B | 9.92E-07 | -9.54187 |
| 1559957_a_at | LOC642852 | hypothetical LOC642852 | 6.99E-08 | -9.55498 |
| 233642_s_at | HEATR5B | HEAT repeat containing 5B | 3.13E-07 | -9.5552 |
| 213305_s_at | PPP2R5C | protein phosphatase 2, regulatory subunit B', gamma | 4.53E-06 | -9.56108 |
| 221823_at | C5orf30 | chromosome 5 open reading frame 30 | 7.23E-08 | -9.57001 |
| 228674_s_at | EML4 | echinoderm microtubule associated protein like 4 | 3.90E-06 | -9.57052 |
| 227408_s_at | SNX25 | sorting nexin 25 | 1.11E-06 | -9.57131 |
| 201039_s_at | RAD23A | RAD23 homolog A (S. cerevisiae) | 1.20E-06 | -9.57223 |
| 213668_s_at | SOX4 | SRY (sex determining region Y)-box 4 | 2.09E-05 | -9.58016 |
| 205498_at | GHR | growth hormone receptor | 2.39E-07 | -9.58131 |
| 224725_at | MIB1 | mindbomb homolog 1 (Drosophila) | 6.85E-06 | -9.58565 |
| 225810_at | MTMR10 | myotubularin related protein 10 | 1.76E-06 | -9.58783 |
| 203313_s_at | TGIF1 | TGFB-induced factor homeobox 1 | 9.36E-07 | -9.59204 |
| 214449_s_at | RHOQ | ras homolog gene family, member Q | 6.00E-05 | -9.59381 |
| 208991_at | STAT3 | signal transducer and activator of transcription 3 (acute-phase response factor) | 2.98E-06 | -9.59403 |
| 218223_s_at | PLEKHO1 | pleckstrin homology domain containing, family O member 1 | 1.42E-07 | -9.59506 |
| 208625_s_at | EIF4G1 | eukaryotic translation initiation factor 4 gamma, 1 | 1.04E-07 | -9.59709 |
| 217805_at | ILF3 | interleukin enhancer binding factor 3, 90kDa | 1.48E-07 | -9.59871 |
| 209080_x_at | GLRX3 | glutaredoxin 3 | 5.68E-09 | -9.60028 |
| 213746_s_at | FLNA | filamin A, alpha | 2.37E-06 | -9.60319 |
| 201371_s_at | CUL3 | cullin 3 | 5.35E-08 | -9.60369 |
| 200902_at | 15-九月 | 15 kDa selenoprotein | 2.19E-05 | -9.60523 |
| 210026_s_at | CARD10 | caspase recruitment domain family, member 10 | 2.97E-07 | -9.61095 |
| 208685_x_at | BRD2 | bromodomain containing 2 | 1.16E-06 | -9.61225 |
| 218999_at | TMEM140 | transmembrane protein 140 | 1.44E-06 | -9.61362 |
| 201170_s_at | BHLHE40 | basic helix-loop-helix family, member e40 | 1.81E-07 | -9.61549 |
| 205512_s_at | AIFM1 | apoptosis-inducing factor, mitochondrion-associated, 1 | 2.37E-10 | -9.61887 |
| 233093_s_at | BIRC6 | baculoviral IAP repeat-containing 6 | 2.09E-06 | -9.62645 |
| 220688_s_at | MRTO4 | mRNA turnover 4 homolog (S. cerevisiae) | 8.38E-05 | -9.62991 |
| 224571_at | IRF2BP2 | interferon regulatory factor 2 binding protein 2 | 5.19E-08 | -9.63029 |
| 228822_s_at | USP16 | ubiquitin specific peptidase 16 | 6.83E-07 | -9.63148 |
| 208962_s_at | FADS1 | fatty acid desaturase 1 | 1.05E-07 | -9.63296 |
| 224895_at | YAP1 | Yes-associated protein 1 | 1.27E-09 | -9.63804 |
| 201023_at | TAF7 | TAF7 RNA polymerase II, TATA box binding protein (TBP)-associated factor, 55kDa | 1.31E-07 | -9.63837 |
| 208788_at | ELOVL5 | ELOVL family member 5, elongation of long chain fatty acids (FEN1/Elo2, SUR4/Elo | 1.51E-08 | -9.64121 |
| 222805_at | MANEA | mannosidase, endo-alpha | 1.48E-06 | -9.65119 |
| 225051_at | EPB41 | erythrocyte membrane protein band 4.1 (elliptocytosis 1, RH-linked) | 8.52E-07 | -9.66401 |
| 215696_s_at | SEC16A | SEC16 homolog A (S. cerevisiae) | 2.08E-08 | -9.66501 |
| 225296_at | ZNF317 | zinc finger protein 317 | 2.38E-07 | -9.66631 |
| 200046_at | DAD1 | defender against cell death 1 | 1.36E-09 | -9.67303 |
| 212776_s_at | OBSL1 | obscurin-like 1 | 1.11E-06 | -9.67923 |
| 229958_at | CLN8 | ceroid-lipofuscinosis, neuronal 8 (epilepsy, progressive with mental retardation | 2.25E-06 | -9.67952 |
| 213131_at | OLFM1 | olfactomedin 1 | 2.38E-06 | -9.68381 |
| 225881_at | SLC35B4 | solute carrier family 35, member B4 | 2.07E-06 | -9.68589 |
| 205273_s_at | PITRM1 | pitrilysin metallopeptidase 1 | 1.33E-08 | -9.6885 |
| 224938_at | NUFIP2 | nuclear fragile X mental retardation protein interacting protein 2 | 1.35E-06 | -9.69031 |
| 209092_s_at | GLOD4 | glyoxalase domain containing 4 | 5.08E-08 | -9.69995 |
| 209190_s_at | DIAPH1 | diaphanous homolog 1 (Drosophila) | 8.54E-08 | -9.70226 |
| 218146_at | GLT8D1 | glycosyltransferase 8 domain containing 1 | 4.25E-07 | -9.70331 |
| 228080_at | LAYN | layilin | 4.33E-07 | -9.70741 |
| 212666_at | SMURF1 | SMAD specific E3 ubiquitin protein ligase 1 | 2.60E-06 | -9.71274 |
| 201971_s_at | ATP6V1A | ATPase, H+ transporting, lysosomal 70kDa, V1 subunit A | 7.63E-07 | -9.71288 |
| 204847_at | ZBTB11 | zinc finger and BTB domain containing 11 | 2.12E-06 | -9.71696 |
| 217770_at | PIGT | phosphatidylinositol glycan anchor biosynthesis, class T | 1.18E-05 | -9.71908 |
| 208629_s_at | HADHA | hydroxyacyl-CoA dehydrogenase/3-ketoacyl-CoA thiolase/enoyl-CoA hydratase (trifu | 4.24E-08 | -9.72052 |
| 1558097_at | C22orf30 | chromosome 22 open reading frame 30 | 6.38E-06 | -9.72236 |
| 226524_at | C3orf38 | chromosome 3 open reading frame 38 | 7.74E-07 | -9.72664 |
| 209471_s_at | FNTA | farnesyltransferase, CAAX box, alpha | 6.71E-07 | -9.72674 |
| 205089_at | ZNF7 | zinc finger protein 7 | 5.00E-08 | -9.72849 |
| 1557950_at | SDCCAG1 | serologically defined colon cancer antigen 1 | 1.07E-06 | -9.73098 |
| 221768_at | SFPQ | Splicing factor proline/glutamine-rich (polypyrimidine tract binding protein ass | 1.95E-05 | -9.7333 |
| 212742_at | RNF115 | ring finger protein 115 | 8.65E-07 | -9.73949 |
| 219192_at | UBAP2 | ubiquitin associated protein 2 | 1.86E-08 | -9.74203 |
| 209109_s_at | TSPAN6 | tetraspanin 6 | 2.31E-07 | -9.74207 |
| 223808_s_at | PTPMT1 | protein tyrosine phosphatase, mitochondrial 1 | 1.06E-06 | -9.75354 |
| 226663_at | ANKRD10 | ankyrin repeat domain 10 | 2.37E-06 | -9.75423 |
| 214252_s_at | CLN5 | ceroid-lipofuscinosis, neuronal 5 | 1.98E-06 | -9.75441 |
| 213532_at | ADAM17 | ADAM metallopeptidase domain 17 | 2.95E-08 | -9.75574 |
| 211911_x_at | HLA-B | major histocompatibility complex, class I, B | 1.36E-07 | -9.75872 |
| 223017_at | TXNDC12 | thioredoxin domain containing 12 (endoplasmic reticulum) | 1.82E-10 | -9.76186 |
| 202352_s_at | PSMD12 | proteasome (prosome, macropain) 26S subunit, non-ATPase, 12 | 6.25E-10 | -9.76212 |
| 211950_at | UBR4 | ubiquitin protein ligase E3 component n-recognin 4 | 2.19E-09 | -9.76297 |
| 207871_s_at | ST7 | suppression of tumorigenicity 7 | 1.48E-07 | -9.76973 |
| 204352_at | TRAF5 | TNF receptor-associated factor 5 | 7.05E-08 | -9.77066 |
| 212862_at | CDS2 | CDP-diacylglycerol synthase (phosphatidate cytidylyltransferase) 2 | 1.07E-08 | -9.7715 |
| 207541_s_at | EXOSC10 | exosome component 10 | 3.41E-05 | -9.77326 |
| 206015_s_at | FOXJ3 | forkhead box J3 | 1.44E-06 | -9.78199 |
| 212103_at | KPNA6 | karyopherin alpha 6 (importin alpha 7) | 3.16E-07 | -9.78577 |
| 212098_at | LOC151162 /// MGAT5 | hypothetical LOC151162 /// mannosyl (alpha-1,6-)-glycoprotein beta-1,6-N-acetyl- | 2.46E-08 | -9.78986 |
| 226421_at | AMMECR1 | Alport syndrome, mental retardation, midface hypoplasia and elliptocytosis chrom | 3.05E-06 | -9.7946 |
| 208664_s_at | TTC3 | tetratricopeptide repeat domain 3 | 5.20E-06 | -9.80104 |
| 202671_s_at | PDXK | pyridoxal (pyridoxine, vitamin B6) kinase | 9.12E-07 | -9.80336 |
| 213360_s_at | POM121 /// POM121C | POM121 membrane glycoprotein (rat) /// POM121 membrane glycoprotein C | 1.85E-07 | -9.80575 |
| 225414_at | RNF149 | ring finger protein 149 | 1.39E-07 | -9.81152 |
| 222391_at | TMEM30A | transmembrane protein 30A | 5.61E-07 | -9.81516 |
| 217855_x_at | SDF4 | stromal cell derived factor 4 | 1.98E-07 | -9.81822 |
| 214728_x_at | SMARCA4 | SWI/SNF related, matrix associated, actin dependent regulator of chromatin, subf | 1.13E-06 | -9.81932 |
| 1555950_a_at | CD55 | CD55 molecule, decay accelerating factor for complement (Cromer blood group) | 2.18E-07 | -9.83895 |
| 227305_s_at | SMCR8 | Smith-Magenis syndrome chromosome region, candidate 8 | 1.03E-07 | -9.84436 |
| 212267_at | WAPAL | wings apart-like homolog (Drosophila) | 2.43E-08 | -9.84457 |
| 218326_s_at | LGR4 | leucine-rich repeat-containing G protein-coupled receptor 4 | 1.75E-05 | -9.85305 |
| 221787_at | C6orf120 | chromosome 6 open reading frame 120 | 6.89E-07 | -9.86529 |
| 228456_s_at | LOC149832 | hypothetical protein LOC149832 | 3.85E-06 | -9.86689 |
| 204627_s_at | ITGB3 | integrin, beta 3 (platelet glycoprotein IIIa, antigen CD61) | 1.55E-08 | -9.86899 |
| 211015_s_at | HSPA4 | heat shock 70kDa protein 4 | 3.24E-07 | -9.86901 |
| 227451_s_at | CCDC90A | Coiled-coil domain containing 90A | 1.04E-06 | -9.8752 |
| 221504_s_at | ATP6V1H | ATPase, H+ transporting, lysosomal 50/57kDa, V1 subunit H | 1.17E-07 | -9.88114 |
| 213295_at | CYLD | cylindromatosis (turban tumor syndrome) | 6.01E-08 | -9.88335 |
| 226312_at | RICTOR | RPTOR independent companion of MTOR, complex 2 | 9.75E-07 | -9.88624 |
| 229310_at | KLHL29 | kelch-like 29 (Drosophila) | 2.78E-08 | -9.89074 |
| 208817_at | COMT | catechol-O-methyltransferase | 4.91E-07 | -9.89468 |
| 219118_at | FKBP11 | FK506 binding protein 11, 19 kDa | 1.41E-08 | -9.8966 |
| 224802_at | NDFIP2 | Nedd4 family interacting protein 2 | 3.77E-05 | -9.89798 |
| 205603_s_at | DIAPH2 | diaphanous homolog 2 (Drosophila) | 6.37E-08 | -9.90152 |
| 243492_at | THEM4 | thioesterase superfamily member 4 | 1.74E-07 | -9.90435 |
| 232067_at | C6orf168 | chromosome 6 open reading frame 168 | 2.53E-07 | -9.90472 |
| 228574_at | TMTC2 | Transmembrane and tetratricopeptide repeat containing 2 | 4.68E-06 | -9.90703 |
| 209900_s_at | SLC16A1 | solute carrier family 16, member 1 (monocarboxylic acid transporter 1) | 1.19E-05 | -9.90977 |
| 203624_at | SFRS17A | splicing factor, arginine/serine-rich 17A | 2.31E-06 | -9.91147 |
| 200847_s_at | TMEM66 | transmembrane protein 66 | 8.05E-07 | -9.91151 |
| 217763_s_at | RAB31 | RAB31, member RAS oncogene family | 1.01E-05 | -9.91481 |
| 200768_s_at | MAT2A | methionine adenosyltransferase II, alpha | 1.25E-07 | -9.91592 |
| 218917_s_at | ARID1A | AT rich interactive domain 1A (SWI-like) | 8.76E-09 | -9.91632 |
| 202083_s_at | SEC14L1 | SEC14-like 1 (S. cerevisiae) | 1.85E-05 | -9.92199 |
| 225278_at | PRKAB2 | protein kinase, AMP-activated, beta 2 non-catalytic subunit | 1.33E-06 | -9.9256 |
| 212500_at | ADO | 2-aminoethanethiol (cysteamine) dioxygenase | 8.07E-06 | -9.9334 |
| 209585_s_at | MINPP1 | multiple inositol-polyphosphate phosphatase 1 | 2.42E-06 | -9.93826 |
| 212702_s_at | BICD2 | bicaudal D homolog 2 (Drosophila) | 4.15E-08 | -9.93965 |
| 209815_at | PTCH1 | patched homolog 1 (Drosophila) | 9.21E-07 | -9.9403 |
| 209447_at | SYNE1 | spectrin repeat containing, nuclear envelope 1 | 4.24E-05 | -9.94174 |
| 218533_s_at | UCKL1 | uridine-cytidine kinase 1-like 1 | 2.16E-06 | -9.94225 |
| 222538_s_at | APPL1 | adaptor protein, phosphotyrosine interaction, PH domain and leucine zipper conta | 1.24E-07 | -9.94639 |
| 227454_at | TAOK1 | TAO kinase 1 | 1.35E-05 | -9.96007 |
| 213032_at | NFIB | nuclear factor I/B | 1.30E-07 | -9.96009 |
| 209101_at | CTGF | connective tissue growth factor | 1.44E-06 | -9.96143 |
| 41512_at | BRAP | BRCA1 associated protein | 5.83E-08 | -9.96453 |
| 202369_s_at | TRAM2 | translocation associated membrane protein 2 | 2.36E-06 | -9.96872 |
| 227685_at | TMF1 | TATA element modulatory factor 1 | 3.44E-07 | -9.97048 |
| 202127_at | PRPF4B | PRP4 pre-mRNA processing factor 4 homolog B (yeast) | 5.10E-06 | -9.97164 |
| 205195_at | AP1S1 | adaptor-related protein complex 1, sigma 1 subunit | 4.42E-06 | -9.97401 |
| 222616_s_at | USP16 | ubiquitin specific peptidase 16 | 3.16E-07 | -9.98004 |
| 213883_s_at | TM2D1 | TM2 domain containing 1 | 7.80E-07 | -9.98295 |
| 202990_at | PYGL | phosphorylase, glycogen, liver | 1.70E-07 | -9.98724 |
| 230364_at | CHPT1 | choline phosphotransferase 1 | 5.36E-07 | -9.99585 |
| 212300_at | TXLNA | taxilin alpha | 6.69E-09 | -9.99837 |
| 226353_at | SPPL2A | signal peptide peptidase-like 2A | 4.24E-06 | -10.0027 |
| 218594_at | HEATR1 | HEAT repeat containing 1 | 0.000128 | -10.004 |
| 212183_at | NUDT4 | nudix (nucleoside diphosphate linked moiety X)-type motif 4 | 1.34E-07 | -10.004 |
| 200954_at | ATP6V0C | ATPase, H+ transporting, lysosomal 16kDa, V0 subunit c | 2.52E-08 | -10.0048 |
| 205078_at | PIGF | phosphatidylinositol glycan anchor biosynthesis, class F | 1.97E-05 | -10.006 |
| 209030_s_at | CADM1 | cell adhesion molecule 1 | 9.16E-10 | -10.008 |
| 227256_at | USP31 | ubiquitin specific peptidase 31 | 5.86E-06 | -10.013 |
| 226837_at | SPRED1 | sprouty-related, EVH1 domain containing 1 | 1.50E-05 | -10.0141 |
| 205084_at | BCAP29 | B-cell receptor-associated protein 29 | 2.18E-06 | -10.0149 |
| 202759_s_at | AKAP2 /// PALM2-AKAP2 | A kinase (PRKA) anchor protein 2 /// PALM2-AKAP2 readthrough | 5.70E-08 | -10.0158 |
| 225189_s_at | RAPH1 | Ras association (RalGDS/AF-6) and pleckstrin homology domains 1 | 4.81E-07 | -10.0177 |
| 212496_s_at | KDM4B | lysine (K)-specific demethylase 4B | 2.14E-07 | -10.0188 |
| 202915_s_at | FAM20B | family with sequence similarity 20, member B | 9.39E-08 | -10.0194 |
| 201779_s_at | RNF13 | ring finger protein 13 | 2.44E-06 | -10.0214 |
| 218456_at | CAPRIN2 | caprin family member 2 | 8.91E-06 | -10.0314 |
| 202301_s_at | RSRC2 | arginine/serine-rich coiled-coil 2 | 1.13E-07 | -10.0323 |
| 212097_at | CAV1 | caveolin 1, caveolae protein, 22kDa | 4.70E-07 | -10.0434 |
| 205022_s_at | FOXN3 | forkhead box N3 | 2.30E-07 | -10.0435 |
| 223457_at | COPG2 | coatomer protein complex, subunit gamma 2 | 4.83E-06 | -10.0458 |
| 200729_s_at | ACTR2 | ARP2 actin-related protein 2 homolog (yeast) | 3.06E-06 | -10.0494 |
| 224472_x_at | SDF4 | stromal cell derived factor 4 | 3.41E-06 | -10.0505 |
| 222421_at | UBE2H | ubiquitin-conjugating enzyme E2H (UBC8 homolog, yeast) | 8.45E-08 | -10.0599 |
| 203105_s_at | DNM1L | dynamin 1-like | 1.12E-07 | -10.0608 |
| 218098_at | ARFGEF2 | ADP-ribosylation factor guanine nucleotide-exchange factor 2 (brefeldin A-inhibi | 1.64E-06 | -10.0621 |
| 204167_at | BTD | biotinidase | 2.87E-05 | -10.0753 |
| 221423_s_at | YIPF5 | Yip1 domain family, member 5 | 8.22E-06 | -10.0872 |
| 201837_s_at | SUPT7L | suppressor of Ty 7 (S. cerevisiae)-like | 3.56E-07 | -10.0891 |
| 203320_at | SH2B3 | SH2B adaptor protein 3 | 1.01E-07 | -10.0937 |
| 220642_x_at | GPR89A /// GPR89B /// GPR89C | G protein-coupled receptor 89A /// G protein-coupled receptor 89B /// G protein- | 2.41E-06 | -10.0968 |
| 218027_at | MRPL15 | mitochondrial ribosomal protein L15 | 1.59E-06 | -10.0989 |
| 231968_at | UGGT1 | UDP-glucose glycoprotein glucosyltransferase 1 | 7.00E-08 | -10.1002 |
| 202289_s_at | TACC2 | transforming, acidic coiled-coil containing protein 2 | 1.65E-05 | -10.1058 |
| 206600_s_at | SLC16A5 | solute carrier family 16, member 5 (monocarboxylic acid transporter 6) | 1.44E-05 | -10.1093 |
| 201020_at | YWHAH | tyrosine 3-monooxygenase/tryptophan 5-monooxygenase activation protein, eta poly | 1.46E-09 | -10.1142 |
| 223594_at | TMEM117 | transmembrane protein 117 | 2.51E-07 | -10.116 |
| 221741_s_at | YTHDF1 | YTH domain family, member 1 | 1.03E-08 | -10.1169 |
| 213194_at | ROBO1 | roundabout, axon guidance receptor, homolog 1 (Drosophila) | 1.27E-06 | -10.1196 |
| 206100_at | CPM | carboxypeptidase M | 4.48E-05 | -10.1217 |
| 202982_s_at | ACOT1 /// ACOT2 | acyl-CoA thioesterase 1 /// acyl-CoA thioesterase 2 | 1.70E-06 | -10.1255 |
| 201151_s_at | MBNL1 | muscleblind-like (Drosophila) | 4.42E-06 | -10.1258 |
| 201648_at | JAK1 | Janus kinase 1 | 4.25E-08 | -10.1269 |
| 218017_s_at | HGSNAT | heparan-alpha-glucosaminide N-acetyltransferase | 7.30E-06 | -10.1297 |
| 213135_at | TIAM1 | T-cell lymphoma invasion and metastasis 1 | 5.10E-06 | -10.1344 |
| 221522_at | ANKRD27 | ankyrin repeat domain 27 (VPS9 domain) | 2.51E-05 | -10.138 |
| 209420_s_at | SMPD1 | sphingomyelin phosphodiesterase 1, acid lysosomal | 5.79E-06 | -10.1492 |
| 226739_at | RNF169 | ring finger protein 169 | 6.51E-08 | -10.1624 |
| 201551_s_at | LAMP1 | lysosomal-associated membrane protein 1 | 7.79E-07 | -10.164 |
| 227978_s_at | ZADH2 | zinc binding alcohol dehydrogenase domain containing 2 | 7.01E-06 | -10.1665 |
| 203890_s_at | DAPK3 | death-associated protein kinase 3 | 1.24E-06 | -10.1733 |
| 219496_at | ANKRD57 | ankyrin repeat domain 57 | 7.90E-07 | -10.175 |
| 225202_at | RHOBTB3 | Rho-related BTB domain containing 3 | 1.08E-07 | -10.1756 |
| 216268_s_at | JAG1 | jagged 1 (Alagille syndrome) | 5.60E-07 | -10.1826 |
| 211708_s_at | SCD | stearoyl-CoA desaturase (delta-9-desaturase) | 2.94E-08 | -10.1865 |
| 201072_s_at | SMARCC1 | SWI/SNF related, matrix associated, actin dependent regulator of chromatin, subf | 8.91E-07 | -10.1923 |
| 211563_s_at | C19orf2 | chromosome 19 open reading frame 2 | 2.15E-06 | -10.2045 |
| 225157_at | MLXIP | MLX interacting protein | 5.89E-07 | -10.2057 |
| 225101_s_at | SNX14 | sorting nexin 14 | 6.28E-06 | -10.2081 |
| 201485_s_at | RCN2 | reticulocalbin 2, EF-hand calcium binding domain | 3.41E-08 | -10.2141 |
| 91816_f_at | MEX3D | mex-3 homolog D (C. elegans) | 2.37E-06 | -10.2221 |
| 202998_s_at | LOXL2 | lysyl oxidase-like 2 | 2.01E-06 | -10.2237 |
| 224842_at | SMG1 | SMG1 homolog, phosphatidylinositol 3-kinase-related kinase (C. elegans) | 9.04E-06 | -10.2328 |
| 228106_at | DCAF16 | DDB1 and CUL4 associated factor 16 | 6.92E-07 | -10.236 |
| 208694_at | PRKDC | protein kinase, DNA-activated, catalytic polypeptide | 2.69E-06 | -10.2465 |
| 225052_at | TMEM203 | transmembrane protein 203 | 8.56E-08 | -10.2489 |
| 231257_at | TCERG1L | transcription elongation regulator 1-like | 4.20E-06 | -10.2555 |
| 228418_at | EXOC5 | exocyst complex component 5 | 1.19E-05 | -10.2672 |
| 225149_at | PCID2 | PCI domain containing 2 | 1.63E-07 | -10.2681 |
| 227990_at | SLU7 | SLU7 splicing factor homolog (S. cerevisiae) | 2.22E-06 | -10.2729 |
| 225623_at | KIAA1737 | KIAA1737 | 4.49E-07 | -10.2756 |
| 211383_s_at | WDR37 | WD repeat domain 37 | 4.88E-07 | -10.2782 |
| 212458_at | SPRED2 | sprouty-related, EVH1 domain containing 2 | 5.47E-10 | -10.282 |
| 204497_at | ADCY9 | adenylate cyclase 9 | 5.88E-06 | -10.2842 |
| 212766_s_at | ISG20L2 | interferon stimulated exonuclease gene 20kDa-like 2 | 3.80E-10 | -10.2851 |
| 225450_at | AMOTL1 | angiomotin like 1 | 5.36E-09 | -10.2991 |
| 203708_at | PDE4B | phosphodiesterase 4B, cAMP-specific (phosphodiesterase E4 dunce homolog, Drosoph | 4.47E-08 | -10.2998 |
| 200816_s_at | PAFAH1B1 | platelet-activating factor acetylhydrolase 1b, regulatory subunit 1 (45kDa) | 5.51E-06 | -10.3 |
| 226825_s_at | TMEM165 | transmembrane protein 165 | 8.27E-07 | -10.3006 |
| 224883_at | PLDN | pallidin homolog (mouse) | 1.50E-09 | -10.3034 |
| 224631_at | ZFP91 | zinc finger protein 91 homolog (mouse) | 3.65E-08 | -10.3062 |
| 208478_s_at | BAX | BCL2-associated X protein | 3.95E-06 | -10.3183 |
| 212628_at | PKN2 | protein kinase N2 | 9.87E-08 | -10.3222 |
| 236620_at | RIF1 | RAP1 interacting factor homolog (yeast) | 2.48E-06 | -10.3317 |
| 212806_at | PRUNE2 | prune homolog 2 (Drosophila) | 6.11E-06 | -10.3405 |
| 222837_s_at | NAA15 | N(alpha)-acetyltransferase 15, NatA auxiliary subunit | 3.86E-06 | -10.3423 |
| 211928_at | DYNC1H1 | dynein, cytoplasmic 1, heavy chain 1 | 1.82E-09 | -10.3428 |
| 211081_s_at | MAP4K5 | mitogen-activated protein kinase kinase kinase kinase 5 | 5.46E-05 | -10.3492 |
| 226050_at | TMCO3 | transmembrane and coiled-coil domains 3 | 1.12E-08 | -10.3545 |
| 201790_s_at | DHCR7 | 7-dehydrocholesterol reductase | 1.19E-05 | -10.3588 |
| 225002_s_at | SUMF2 | sulfatase modifying factor 2 | 2.56E-08 | -10.3633 |
| 1555797_a_at | ARPC5 | actin related protein 2/3 complex, subunit 5, 16kDa | 5.30E-07 | -10.3711 |
| 222988_s_at | TMEM9 | transmembrane protein 9 | 1.83E-08 | -10.3727 |
| 223743_s_at | MRPL4 | mitochondrial ribosomal protein L4 | 2.36E-07 | -10.376 |
| 201797_s_at | VARS | valyl-tRNA synthetase | 6.77E-09 | -10.3767 |
| 235334_at | ST6GALNAC3 | ST6 (alpha-N-acetyl-neuraminyl-2,3-beta-galactosyl-1,3)-N-acetylgalactosaminide | 9.78E-08 | -10.3784 |
| 205000_at | DDX3Y | DEAD (Asp-Glu-Ala-Asp) box polypeptide 3, Y-linked | 1.06E-06 | -10.3854 |
| 209630_s_at | FBXW2 | F-box and WD repeat domain containing 2 | 5.45E-06 | -10.3875 |
| 229232_at | LRRC57 | leucine rich repeat containing 57 | 1.02E-05 | -10.3876 |
| 201917_s_at | SLC25A36 | solute carrier family 25, member 36 | 6.41E-08 | -10.3887 |
| 203987_at | FZD6 | frizzled homolog 6 (Drosophila) | 1.95E-06 | -10.3924 |
| 225539_at | ZNF295 | zinc finger protein 295 | 1.31E-07 | -10.4021 |
| 226005_at | UBE2G1 | ubiquitin-conjugating enzyme E2G 1 (UBC7 homolog, yeast) | 1.88E-08 | -10.4079 |
| 202993_at | ILVBL | ilvB (bacterial acetolactate synthase)-like | 3.24E-08 | -10.4129 |
| 208705_s_at | EIF5 | eukaryotic translation initiation factor 5 | 2.17E-07 | -10.4132 |
| 209208_at | MPDU1 | mannose-P-dolichol utilization defect 1 | 5.07E-08 | -10.4195 |
| 203635_at | DSCR3 | Down syndrome critical region gene 3 | 9.56E-09 | -10.4219 |
| 209674_at | CRY1 | cryptochrome 1 (photolyase-like) | 1.94E-06 | -10.4247 |
| 213169_at | SEMA5A | sema domain, seven thrombospondin repeats (type 1 and type 1-like), transmembran | 1.82E-07 | -10.4302 |
| 1558093_s_at | MATR3 | matrin 3 | 7.32E-06 | -10.4316 |
| 1555193_a_at | ZNF277 | zinc finger protein 277 | 2.05E-05 | -10.4338 |
| 212429_s_at | GTF3C2 | general transcription factor IIIC, polypeptide 2, beta 110kDa | 1.41E-06 | -10.4399 |
| 212474_at | AVL9 | AVL9 homolog (S. cerevisiase) | 4.80E-07 | -10.4422 |
| 1569594_a_at | SDCCAG1 | serologically defined colon cancer antigen 1 | 5.13E-07 | -10.4476 |
| 212009_s_at | STIP1 | stress-induced-phosphoprotein 1 | 4.42E-07 | -10.4477 |
| 209175_at | SEC23IP | SEC23 interacting protein | 6.62E-08 | -10.4556 |
| 212875_s_at | C2CD2 | C2 calcium-dependent domain containing 2 | 4.29E-07 | -10.4577 |
| 212708_at | MSL1 | male-specific lethal 1 homolog (Drosophila) | 7.08E-09 | -10.4589 |
| 209707_at | PIGK | phosphatidylinositol glycan anchor biosynthesis, class K | 2.62E-06 | -10.4688 |
| 227420_at | TNFAIP8L1 | tumor necrosis factor, alpha-induced protein 8-like 1 | 1.57E-09 | -10.4699 |
| 203633_at | CPT1A | carnitine palmitoyltransferase 1A (liver) | 1.49E-06 | -10.4782 |
| 201099_at | USP9X | ubiquitin specific peptidase 9, X-linked | 1.48E-07 | -10.4822 |
| 225153_at | GFM1 | G elongation factor, mitochondrial 1 | 5.84E-06 | -10.4935 |
| 201060_x_at | STOM | stomatin | 3.63E-07 | -10.4976 |
| 212163_at | KIDINS220 | kinase D-interacting substrate, 220kDa | 7.23E-07 | -10.5003 |
| 209509_s_at | DPAGT1 | dolichyl-phosphate (UDP-N-acetylglucosamine) N-acetylglucosaminephosphotransfera | 4.81E-08 | -10.509 |
| 210935_s_at | WDR1 | WD repeat domain 1 | 4.61E-07 | -10.5099 |
| 221853_s_at | NOMO1 /// NOMO2 /// NOMO3 | NODAL modulator 1 /// NODAL modulator 2 /// NODAL modulator 3 | 3.80E-05 | -10.511 |
| 202653_s_at | 7-三月 | membrane-associated ring finger (C3HC4) 7 | 8.77E-07 | -10.519 |
| 226479_at | KBTBD6 | kelch repeat and BTB (POZ) domain containing 6 | 2.61E-06 | -10.5255 |
| 223046_at | EGLN1 | egl nine homolog 1 (C. elegans) | 4.35E-08 | -10.5307 |
| 226143_at | RAI1 | retinoic acid induced 1 | 2.82E-07 | -10.5363 |
| 209570_s_at | D4S234E /// FOXP1 | DNA segment on chromosome 4 (unique) 234 expressed sequence /// forkhead box P1 | 2.14E-07 | -10.5429 |
| 205904_at | MICA | MHC class I polypeptide-related sequence A | 2.22E-05 | -10.5478 |
| 218686_s_at | RHBDF1 | rhomboid 5 homolog 1 (Drosophila) | 8.70E-10 | -10.5536 |
| 225056_at | SIPA1L2 | signal-induced proliferation-associated 1 like 2 | 6.99E-07 | -10.5581 |
| 212689_s_at | KDM3A | lysine (K)-specific demethylase 3A | 2.97E-10 | -10.5601 |
| 225657_at | LOC152217 | hypothetical LOC152217 | 3.29E-07 | -10.5739 |
| 201861_s_at | LRRFIP1 | leucine rich repeat (in FLII) interacting protein 1 | 3.08E-06 | -10.5759 |
| 204654_s_at | TFAP2A | transcription factor AP-2 alpha (activating enhancer binding protein 2 alpha) | 1.42E-06 | -10.5778 |
| 214894_x_at | MACF1 | microtubule-actin crosslinking factor 1 | 1.96E-05 | -10.5806 |
| 202766_s_at | FBN1 | fibrillin 1 | 1.19E-08 | -10.5903 |
| 238462_at | UBASH3B | ubiquitin associated and SH3 domain containing B | 3.46E-07 | -10.5941 |
| 218273_s_at | PDP1 | pyruvate dehyrogenase phosphatase catalytic subunit 1 | 1.22E-06 | -10.5994 |
| 202838_at | FUCA1 | fucosidase, alpha-L- 1, tissue | 9.23E-10 | -10.6091 |
| 208843_s_at | GORASP2 | golgi reassembly stacking protein 2, 55kDa | 3.49E-07 | -10.6115 |
| 225147_at | CYTH3 | cytohesin 3 | 1.25E-06 | -10.6182 |
| 218501_at | ARHGEF3 | Rho guanine nucleotide exchange factor (GEF) 3 | 9.61E-07 | -10.6222 |
| 225785_at | REEP3 | receptor accessory protein 3 | 1.05E-05 | -10.6227 |
| 201791_s_at | DHCR7 | 7-dehydrocholesterol reductase | 4.62E-08 | -10.6245 |
| 239572_at | GJA3 | gap junction protein, alpha 3, 46kDa | 8.84E-07 | -10.6255 |
| 208810_at | DNAJB6 /// TMEM135 | DnaJ (Hsp40) homolog, subfamily B, member 6 /// transmembrane protein 135 | 2.14E-06 | -10.6282 |
| 202968_s_at | DYRK2 | dual-specificity tyrosine-(Y)-phosphorylation regulated kinase 2 | 4.28E-06 | -10.6527 |
| 217225_x_at | NOMO1 /// NOMO2 /// NOMO3 | NODAL modulator 1 /// NODAL modulator 2 /// NODAL modulator 3 | 2.23E-08 | -10.6564 |
| 201914_s_at | SEC63 | SEC63 homolog (S. cerevisiae) | 2.01E-05 | -10.6782 |
| 219522_at | FJX1 | four jointed box 1 (Drosophila) | 2.51E-06 | -10.6804 |
| 212018_s_at | RSL1D1 | ribosomal L1 domain containing 1 | 1.47E-07 | -10.6819 |
| 203973_s_at | CEBPD | CCAAT/enhancer binding protein (C/EBP), delta | 1.68E-07 | -10.6902 |
| 212441_at | KIAA0232 | KIAA0232 | 4.84E-08 | -10.6914 |
| 201901_s_at | YY1 | YY1 transcription factor | 4.34E-10 | -10.6934 |
| 219158_s_at | NAA15 | N(alpha)-acetyltransferase 15, NatA auxiliary subunit | 4.04E-07 | -10.6942 |
| 212135_s_at | ATP2B4 | ATPase, Ca++ transporting, plasma membrane 4 | 1.01E-07 | -10.6973 |
| 210041_s_at | PGM3 | phosphoglucomutase 3 | 2.77E-08 | -10.698 |
| 203284_s_at | HS2ST1 | heparan sulfate 2-O-sulfotransferase 1 | 2.01E-07 | -10.7028 |
| 222975_s_at | CSDE1 | cold shock domain containing E1, RNA-binding | 8.89E-08 | -10.7072 |
| 212288_at | FNBP1 | formin binding protein 1 | 3.10E-06 | -10.7119 |
| 202749_at | WRB | tryptophan rich basic protein | 2.85E-08 | -10.7183 |
| 220925_at | NAA35 | N(alpha)-acetyltransferase 35, NatC auxiliary subunit | 2.57E-07 | -10.7195 |
| 225035_x_at | BTNL8 /// LOC100288778 /// WASH1 /// WASH2P /// WASH3P /// WASH7P | butyrophilin-like 8 /// similar to WAS protein family homolog 1 /// WAS protein | 3.19E-06 | -10.7217 |
| 201967_at | RBM6 | RNA binding motif protein 6 | 1.55E-07 | -10.7232 |
| 230083_at | USP53 | ubiquitin specific peptidase 53 | 5.78E-07 | -10.7235 |
| 210840_s_at | IQGAP1 | IQ motif containing GTPase activating protein 1 | 1.99E-08 | -10.7298 |
| 225173_at | ARHGAP18 | Rho GTPase activating protein 18 | 7.42E-08 | -10.7316 |
| 223650_s_at | NRBF2 | nuclear receptor binding factor 2 | 6.40E-08 | -10.7373 |
| 226478_at | TM7SF3 | transmembrane 7 superfamily member 3 | 1.96E-06 | -10.7495 |
| 224576_at | ERGIC1 | endoplasmic reticulum-golgi intermediate compartment (ERGIC) 1 | 8.30E-10 | -10.7497 |
| 63825_at | ABHD2 | abhydrolase domain containing 2 | 5.46E-08 | -10.7505 |
| 213140_s_at | SS18L1 | synovial sarcoma translocation gene on chromosome 18-like 1 | 2.47E-07 | -10.7545 |
| 202412_s_at | USP1 | ubiquitin specific peptidase 1 | 7.57E-06 | -10.7588 |
| 209185_s_at | IRS2 | insulin receptor substrate 2 | 7.58E-10 | -10.7596 |
| 219549_s_at | RTN3 | reticulon 3 | 1.00E-09 | -10.7598 |
| 35820_at | GM2A | GM2 ganglioside activator | 2.18E-08 | -10.7609 |
| 225932_s_at | HNRNPA2B1 | heterogeneous nuclear ribonucleoprotein A2/B1 | 4.49E-08 | -10.7649 |
| 212792_at | DPY19L1 | dpy-19-like 1 (C. elegans) | 2.34E-06 | -10.7675 |
| 212345_s_at | CREB3L2 | cAMP responsive element binding protein 3-like 2 | 4.13E-08 | -10.769 |
| 202760_s_at | AKAP2 /// PALM2-AKAP2 | A kinase (PRKA) anchor protein 2 /// PALM2-AKAP2 readthrough | 1.72E-05 | -10.7692 |
| 225647_s_at | CTSC | cathepsin C | 1.05E-09 | -10.7749 |
| 217865_at | RNF130 | ring finger protein 130 | 4.18E-09 | -10.7801 |
| 208879_x_at | PRPF6 | PRP6 pre-mRNA processing factor 6 homolog (S. cerevisiae) | 2.86E-06 | -10.7839 |
| 212461_at | AZIN1 | antizyme inhibitor 1 | 1.37E-08 | -10.7889 |
| 231817_at | USP53 | ubiquitin specific peptidase 53 | 5.61E-08 | -10.7924 |
| 242931_at | LONRF3 | LON peptidase N-terminal domain and ring finger 3 | 1.83E-07 | -10.8001 |
| 204194_at | BACH1 | BTB and CNC homology 1, basic leucine zipper transcription factor 1 | 4.41E-07 | -10.8008 |
| 203771_s_at | BLVRA | biliverdin reductase A | 5.57E-07 | -10.8043 |
| 229797_at | MCOLN3 | mucolipin 3 | 1.83E-05 | -10.8101 |
| 203497_at | MED1 | mediator complex subunit 1 | 9.24E-08 | -10.8344 |
| 212606_at | WDFY3 | WD repeat and FYVE domain containing 3 | 2.66E-09 | -10.8366 |
| 218096_at | AGPAT5 | 1-acylglycerol-3-phosphate O-acyltransferase 5 (lysophosphatidic acid acyltransf | 2.30E-07 | -10.8503 |
| 201189_s_at | ITPR3 | inositol 1,4,5-triphosphate receptor, type 3 | 2.66E-06 | -10.8519 |
| 213734_at | WSB2 | WD repeat and SOCS box-containing 2 | 1.72E-07 | -10.8529 |
| 217122_s_at | SLC35E2 | similar to solute carrier family 35, member E2 | 7.12E-10 | -10.8532 |
| 208442_s_at | ATM | ataxia telangiectasia mutated | 2.75E-08 | -10.8573 |
| 221473_x_at | SERINC3 | serine incorporator 3 | 2.07E-06 | -10.858 |
| 210793_s_at | NUP98 | nucleoporin 98kDa | 5.26E-07 | -10.8705 |
| 1553106_at | C5orf24 | chromosome 5 open reading frame 24 | 3.76E-05 | -10.8709 |
| 202084_s_at | SEC14L1 | SEC14-like 1 (S. cerevisiae) | 9.84E-08 | -10.8715 |
| 228961_at | MIER3 | mesoderm induction early response 1, family member 3 | 4.08E-05 | -10.8717 |
| 224818_at | SORT1 | sortilin 1 | 8.97E-07 | -10.8792 |
| 228748_at | CD59 | CD59 molecule, complement regulatory protein | 1.01E-05 | -10.886 |
| 226432_at | ETNK1 | ethanolamine kinase 1 | 8.28E-06 | -10.8893 |
| 202655_at | MANF | mesencephalic astrocyte-derived neurotrophic factor | 1.75E-08 | -10.8905 |
| 208838_at | CAND1 | cullin-associated and neddylation-dissociated 1 | 7.71E-07 | -10.8953 |
| 223183_at | AGPAT3 | 1-acylglycerol-3-phosphate O-acyltransferase 3 | 4.86E-07 | -10.8987 |
| 201200_at | CREG1 | cellular repressor of E1A-stimulated genes 1 | 1.22E-05 | -10.9062 |
| 209093_s_at | GBA /// GBAP1 | glucosidase, beta, acid /// glucosidase, beta, acid pseudogene 1 | 8.06E-07 | -10.9158 |
| 225852_at | ANKRD17 | ankyrin repeat domain 17 | 3.50E-08 | -10.9242 |
| 230788_at | GCNT2 | glucosaminyl (N-acetyl) transferase 2, I-branching enzyme (I blood group) | 1.03E-05 | -10.9267 |
| 1553118_at | THEM4 | thioesterase superfamily member 4 | 3.11E-07 | -10.9268 |
| 211048_s_at | PDIA4 | protein disulfide isomerase family A, member 4 | 4.32E-07 | -10.9296 |
| 221245_s_at | FZD5 | frizzled homolog 5 (Drosophila) | 1.04E-07 | -10.9305 |
| 206864_s_at | HRK | harakiri, BCL2 interacting protein (contains only BH3 domain) | 9.11E-10 | -10.9366 |
| 227341_at | BEND7 | BEN domain containing 7 | 3.98E-06 | -10.9369 |
| 218107_at | WDR26 | WD repeat domain 26 | 2.68E-06 | -10.9465 |
| 201408_at | PPP1CB | protein phosphatase 1, catalytic subunit, beta isozyme | 5.67E-06 | -10.9498 |
| 213552_at | GLCE | glucuronic acid epimerase | 4.35E-06 | -10.9533 |
| 224949_at | YIPF5 | Yip1 domain family, member 5 | 5.60E-06 | -10.9547 |
| 227964_at | FRMD8 | FERM domain containing 8 | 1.99E-07 | -10.9554 |
| 216321_s_at | NR3C1 | nuclear receptor subfamily 3, group C, member 1 (glucocorticoid receptor) | 2.78E-07 | -10.9663 |
| 1556200_a_at | C10orf90 | chromosome 10 open reading frame 90 | 1.95E-06 | -10.9694 |
| 209580_s_at | MBD4 | methyl-CpG binding domain protein 4 | 2.98E-07 | -10.9698 |
| 224881_at | VKORC1L1 | vitamin K epoxide reductase complex, subunit 1-like 1 | 1.71E-09 | -10.9719 |
| 222416_at | ALDH18A1 | aldehyde dehydrogenase 18 family, member A1 | 4.44E-07 | -10.9858 |
| 203971_at | SLC31A1 | solute carrier family 31 (copper transporters), member 1 | 6.79E-07 | -10.9901 |
| 203758_at | CTSO | cathepsin O | 2.25E-06 | -10.9926 |
| 204957_at | ORC5L | origin recognition complex, subunit 5-like (yeast) | 8.76E-08 | -10.9968 |
| 202842_s_at | DNAJB9 | DnaJ (Hsp40) homolog, subfamily B, member 9 | 3.73E-06 | -10.9975 |
| 212178_s_at | POM121 /// POM121C | POM121 membrane glycoprotein (rat) /// POM121 membrane glycoprotein C | 2.47E-06 | -10.9982 |
| 236248_x_at | TADA2B | transcriptional adaptor 2B | 1.07E-05 | -11.0065 |
| 202067_s_at | LDLR | low density lipoprotein receptor | 5.86E-08 | -11.0072 |
| 202075_s_at | PLTP | phospholipid transfer protein | 8.70E-08 | -11.0077 |
| 210868_s_at | ELOVL6 | ELOVL family member 6, elongation of long chain fatty acids (FEN1/Elo2, SUR4/Elo | 6.39E-07 | -11.0131 |
| 222668_at | KCTD15 | potassium channel tetramerisation domain containing 15 | 8.10E-09 | -11.0137 |
| 209127_s_at | SART3 | squamous cell carcinoma antigen recognized by T cells 3 | 4.79E-06 | -11.0209 |
| 213154_s_at | BICD2 | bicaudal D homolog 2 (Drosophila) | 4.44E-07 | -11.0257 |
| 225078_at | EMP2 | epithelial membrane protein 2 | 4.80E-07 | -11.0297 |
| 209321_s_at | ADCY3 | adenylate cyclase 3 | 1.65E-06 | -11.0333 |
| 201363_s_at | IVNS1ABP | influenza virus NS1A binding protein | 1.27E-07 | -11.0343 |
| 212232_at | FNBP4 | formin binding protein 4 | 2.51E-08 | -11.0414 |
| 218330_s_at | NAV2 | neuron navigator 2 | 1.82E-06 | -11.0463 |
| 207700_s_at | NCOA3 | nuclear receptor coactivator 3 | 4.44E-08 | -11.0518 |
| 224716_at | SLC35B2 | solute carrier family 35, member B2 | 4.06E-08 | -11.0693 |
| 226538_at | MAN2A1 | mannosidase, alpha, class 2A, member 1 | 9.21E-07 | -11.0733 |
| 217894_at | KCTD3 | potassium channel tetramerisation domain containing 3 | 4.73E-06 | -11.0737 |
| 204544_at | HPS5 | Hermansky-Pudlak syndrome 5 | 6.63E-06 | -11.0817 |
| 204526_s_at | TBC1D8 | TBC1 domain family, member 8 (with GRAM domain) | 1.00E-08 | -11.0839 |
| 208722_s_at | ANAPC5 | anaphase promoting complex subunit 5 | 4.87E-07 | -11.0889 |
| 223289_s_at | USP38 | ubiquitin specific peptidase 38 | 2.72E-05 | -11.0896 |
| 212468_at | SPAG9 | sperm associated antigen 9 | 4.53E-07 | -11.0922 |
| 213338_at | TMEM158 | transmembrane protein 158 (gene/pseudogene) | 1.40E-06 | -11.116 |
| 219374_s_at | ALG9 | asparagine-linked glycosylation 9, alpha-1,2-mannosyltransferase homolog (S. cer | 5.93E-09 | -11.1217 |
| 201462_at | SCRN1 | secernin 1 | 8.62E-10 | -11.1239 |
| 224250_s_at | SECISBP2 | SECIS binding protein 2 | 3.38E-10 | -11.1264 |
| 224893_at | ATL3 | atlastin GTPase 3 | 1.24E-05 | -11.1326 |
| 224743_at | IMPAD1 | inositol monophosphatase domain containing 1 | 3.62E-09 | -11.1434 |
| 242100_at | CHSY3 | chondroitin sulfate synthase 3 | 2.36E-06 | -11.1518 |
| 224481_s_at | HECTD1 | HECT domain containing 1 | 1.25E-08 | -11.152 |
| 201737_s_at | 6-三月 | membrane-associated ring finger (C3HC4) 6 | 1.54E-05 | -11.1553 |
| 209211_at | KLF5 | Kruppel-like factor 5 (intestinal) | 4.13E-05 | -11.1578 |
| 222691_at | SLC35B3 | solute carrier family 35, member B3 | 4.97E-07 | -11.161 |
| 34031_i_at | KRIT1 | KRIT1, ankyrin repeat containing | 9.02E-07 | -11.1704 |
| 226764_at | ZNF827 | zinc finger protein 827 | 1.10E-07 | -11.1706 |
| 227698_s_at | RAB40C | RAB40C, member RAS oncogene family | 8.10E-06 | -11.1773 |
| 213423_x_at | TUSC3 | tumor suppressor candidate 3 | 7.12E-07 | -11.1818 |
| 223113_at | TMEM138 | transmembrane protein 138 | 3.79E-09 | -11.1826 |
| 213165_at | CEP350 | centrosomal protein 350kDa | 2.61E-07 | -11.1847 |
| 227176_at | SLC2A13 | solute carrier family 2 (facilitated glucose transporter), member 13 | 8.79E-08 | -11.1916 |
| 218962_s_at | TMEM168 | transmembrane protein 168 | 5.06E-07 | -11.1931 |
| 224887_at | GNPTG | N-acetylglucosamine-1-phosphate transferase, gamma subunit | 1.79E-07 | -11.1971 |
| 202404_s_at | COL1A2 | collagen, type I, alpha 2 | 3.56E-07 | -11.201 |
| 225230_at | DRAM2 | DNA-damage regulated autophagy modulator 2 | 6.76E-08 | -11.2052 |
| 223723_at | MFI2 | antigen p97 (melanoma associated) identified by monoclonal antibodies 133.2 and | 8.85E-08 | -11.2076 |
| 225519_at | PPP4R2 | protein phosphatase 4, regulatory subunit 2 | 1.94E-06 | -11.2087 |
| 202613_at | CTPS | CTP synthase | 1.13E-07 | -11.2089 |
| 226291_at | ALS2 | amyotrophic lateral sclerosis 2 (juvenile) | 2.69E-06 | -11.212 |
| 1558199_at | FN1 | fibronectin 1 | 1.62E-06 | -11.2154 |
| 211926_s_at | MYH9 | myosin, heavy chain 9, non-muscle | 2.23E-09 | -11.2214 |
| 45288_at | ABHD6 | abhydrolase domain containing 6 | 1.77E-07 | -11.2249 |
| 212622_at | TMEM41B | transmembrane protein 41B | 3.45E-07 | -11.2269 |
| 235791_x_at | CHD1 | chromodomain helicase DNA binding protein 1 | 3.95E-07 | -11.2358 |
| 220234_at | CA8 | carbonic anhydrase VIII | 1.55E-07 | -11.2468 |
| 204857_at | MAD1L1 | MAD1 mitotic arrest deficient-like 1 (yeast) | 3.54E-06 | -11.249 |
| 219015_s_at | ALG13 | asparagine-linked glycosylation 13 homolog (S. cerevisiae) | 1.16E-06 | -11.2498 |
| 204360_s_at | NAGLU | N-acetylglucosaminidase, alpha- | 2.91E-06 | -11.251 |
| 222526_at | GATAD2A | GATA zinc finger domain containing 2A | 2.66E-07 | -11.2523 |
| 213004_at | ANGPTL2 | angiopoietin-like 2 | 9.18E-06 | -11.2534 |
| 201823_s_at | RNF14 | ring finger protein 14 | 2.00E-07 | -11.2568 |
| 224937_at | PTGFRN | prostaglandin F2 receptor negative regulator | 6.21E-07 | -11.2614 |
| 223331_s_at | DDX20 | DEAD (Asp-Glu-Ala-Asp) box polypeptide 20 | 1.56E-06 | -11.264 |
| 202386_s_at | KIAA0430 | KIAA0430 | 6.62E-09 | -11.2643 |
| 1552977_a_at | CNPY3 | canopy 3 homolog (zebrafish) | 1.07E-06 | -11.2669 |
| 235443_at | LOC100131067 | hypothetical protein LOC100131067 | 1.30E-06 | -11.2687 |
| 223120_at | FUCA2 | fucosidase, alpha-L- 2, plasma | 2.61E-06 | -11.2698 |
| 241612_at | FOXD3 | forkhead box D3 | 5.58E-06 | -11.2824 |
| 227188_at | C21orf63 | chromosome 21 open reading frame 63 | 1.31E-07 | -11.2827 |
| 214151_s_at | CCPG1 | cell cycle progression 1 | 2.84E-08 | -11.2831 |
| 209421_at | MSH2 | mutS homolog 2, colon cancer, nonpolyposis type 1 (E. coli) | 5.75E-07 | -11.2904 |
| 207305_s_at | KIAA1012 | KIAA1012 | 1.90E-08 | -11.291 |
| 203628_at | IGF1R | insulin-like growth factor 1 receptor | 9.67E-06 | -11.296 |
| 201773_at | ADNP | activity-dependent neuroprotector homeobox | 5.57E-07 | -11.297 |
| 218019_s_at | PDXK | pyridoxal (pyridoxine, vitamin B6) kinase | 1.36E-06 | -11.3007 |
| 210156_s_at | PCMT1 | protein-L-isoaspartate (D-aspartate) O-methyltransferase | 1.59E-07 | -11.3009 |
| 1553959_a_at | B3GALT6 | UDP-Gal:betaGal beta 1,3-galactosyltransferase polypeptide 6 | 4.25E-08 | -11.315 |
| 203128_at | SPTLC2 | serine palmitoyltransferase, long chain base subunit 2 | 3.25E-05 | -11.3294 |
| 225913_at | SGK269 | NKF3 kinase family member | 1.98E-08 | -11.3302 |
| 1558511_s_at | ESYT2 | extended synaptotagmin-like protein 2 | 1.43E-05 | -11.333 |
| 213249_at | FBXL7 | F-box and leucine-rich repeat protein 7 | 2.07E-07 | -11.3363 |
| 226409_at | TBC1D20 | TBC1 domain family, member 20 | 6.62E-07 | -11.3385 |
| 208943_s_at | SEC62 | SEC62 homolog (S. cerevisiae) | 5.16E-10 | -11.3466 |
| 228824_s_at | PTGR1 | prostaglandin reductase 1 | 6.56E-08 | -11.3509 |
| 200672_x_at | SPTBN1 | spectrin, beta, non-erythrocytic 1 | 4.95E-07 | -11.3545 |
| 201368_at | ZFP36L2 | zinc finger protein 36, C3H type-like 2 | 2.36E-08 | -11.3622 |
| 244546_at | CYCS | cytochrome c, somatic | 5.15E-07 | -11.3715 |
| 201490_s_at | PPIF | peptidylprolyl isomerase F | 1.64E-07 | -11.3725 |
| 208773_s_at | ANKHD1 /// ANKHD1-EIF4EBP3 | ankyrin repeat and KH domain containing 1 /// ANKHD1-EIF4EBP3 readthrough | 6.30E-07 | -11.3737 |
| 212245_at | MCFD2 | multiple coagulation factor deficiency 2 | 6.60E-07 | -11.3744 |
| 228563_at | GJC1 | gap junction protein, gamma 1, 45kDa | 8.24E-07 | -11.3971 |
| 209210_s_at | FERMT2 | fermitin family homolog 2 (Drosophila) | 2.11E-07 | -11.399 |
| 222552_at | GOLT1B | golgi transport 1 homolog B (S. cerevisiae) | 2.83E-07 | -11.4038 |
| 212063_at | CD44 | CD44 molecule (Indian blood group) | 1.49E-07 | -11.4073 |
| 218302_at | PSENEN | presenilin enhancer 2 homolog (C. elegans) | 7.15E-06 | -11.4226 |
| 224951_at | LASS5 | LAG1 homolog, ceramide synthase 5 | 1.76E-06 | -11.4515 |
| 201656_at | ITGA6 | integrin, alpha 6 | 1.39E-06 | -11.4532 |
| 208453_s_at | XPNPEP1 | X-prolyl aminopeptidase (aminopeptidase P) 1, soluble | 2.28E-06 | -11.4621 |
| 225677_at | BCAP29 | B-cell receptor-associated protein 29 | 4.75E-07 | -11.4646 |
| 201504_s_at | TSN | translin | 2.39E-07 | -11.4663 |
| 208022_s_at | CDC14B | CDC14 cell division cycle 14 homolog B (S. cerevisiae) | 2.35E-08 | -11.4746 |
| 212113_at | ATXN7L3B | ataxin 7-like 3B | 3.75E-06 | -11.4749 |
| 209341_s_at | IKBKB | inhibitor of kappa light polypeptide gene enhancer in B-cells, kinase beta | 2.95E-08 | -11.484 |
| 225365_at | ZDHHC20 | zinc finger, DHHC-type containing 20 | 7.91E-06 | -11.489 |
| 223136_at | AIG1 | androgen-induced 1 | 6.96E-08 | -11.4961 |
| 225752_at | NIPA1 | non imprinted in Prader-Willi/Angelman syndrome 1 | 1.78E-06 | -11.4985 |
| 226025_at | ANKRD28 | ankyrin repeat domain 28 | 2.12E-09 | -11.51 |
| 213775_x_at | ZNF638 | zinc finger protein 638 | 4.10E-08 | -11.5169 |
| 209142_s_at | UBE2G1 | ubiquitin-conjugating enzyme E2G 1 (UBC7 homolog, yeast) | 3.69E-07 | -11.5274 |
| 225121_at | TBC1D23 | TBC1 domain family, member 23 | 9.56E-06 | -11.5358 |
| 1552256_a_at | SCARB1 | scavenger receptor class B, member 1 | 1.37E-07 | -11.5518 |
| 226386_at | C7orf30 | chromosome 7 open reading frame 30 | 1.03E-08 | -11.5605 |
| 1558254_s_at | SRPK2 | SFRS protein kinase 2 | 4.57E-06 | -11.57 |
| 200918_s_at | SRPR | signal recognition particle receptor (docking protein) | 1.69E-08 | -11.5785 |
| 212169_at | FKBP9 | FK506 binding protein 9, 63 kDa | 5.29E-08 | -11.5799 |
| 212978_at | LRRC8B | leucine rich repeat containing 8 family, member B | 1.23E-05 | -11.5804 |
| 212276_at | LPIN1 | lipin 1 | 2.96E-09 | -11.5826 |
| 37892_at | COL11A1 | collagen, type XI, alpha 1 | 3.19E-08 | -11.5834 |
| 218556_at | ORMDL2 | ORM1-like 2 (S. cerevisiae) | 1.52E-06 | -11.5961 |
| 217678_at | SLC7A11 | solute carrier family 7, (cationic amino acid transporter, y+ system) member 11 | 5.31E-06 | -11.5977 |
| 218306_s_at | HERC1 | hect (homologous to the E6-AP (UBE3A) carboxyl terminus) domain and RCC1 (CHC1)- | 3.27E-07 | -11.6076 |
| 212612_at | RCOR1 | REST corepressor 1 | 3.44E-07 | -11.6133 |
| 208893_s_at | DUSP6 | dual specificity phosphatase 6 | 1.44E-06 | -11.6219 |
| 226510_at | HEATR5A | HEAT repeat containing 5A | 8.07E-06 | -11.6234 |
| 226974_at | NEDD4L | neural precursor cell expressed, developmentally down-regulated 4-like | 6.33E-08 | -11.6268 |
| 223170_at | TMEM98 | transmembrane protein 98 | 5.80E-08 | -11.6375 |
| 228851_s_at | ENSA | endosulfine alpha | 9.92E-07 | -11.6401 |
| 213111_at | PIKFYVE | phosphoinositide kinase, FYVE finger containing | 6.13E-07 | -11.6417 |
| 226015_at | ZNF12 | zinc finger protein 12 | 6.58E-09 | -11.6503 |
| 224776_at | AGPAT6 | 1-acylglycerol-3-phosphate O-acyltransferase 6 (lysophosphatidic acid acyltransf | 4.43E-07 | -11.6524 |
| 219929_s_at | ZFYVE21 | zinc finger, FYVE domain containing 21 | 7.62E-07 | -11.6569 |
| 217949_s_at | VKORC1 | vitamin K epoxide reductase complex, subunit 1 | 7.70E-10 | -11.6655 |
| 212599_at | AUTS2 | autism susceptibility candidate 2 | 3.58E-06 | -11.6662 |
| 202568_s_at | MARK3 | MAP/microtubule affinity-regulating kinase 3 | 3.10E-08 | -11.6694 |
| 201398_s_at | TRAM1 | translocation associated membrane protein 1 | 2.28E-09 | -11.6768 |
| 1552486_s_at | LACTB | lactamase, beta | 2.15E-06 | -11.6809 |
| 203579_s_at | SLC7A6 | solute carrier family 7 (cationic amino acid transporter, y+ system), member 6 | 1.84E-05 | -11.6907 |
| 209365_s_at | ECM1 | extracellular matrix protein 1 | 2.19E-06 | -11.6981 |
| 203256_at | CDH3 | cadherin 3, type 1, P-cadherin (placental) | 9.54E-08 | -11.7029 |
| 207606_s_at | ARHGAP12 | Rho GTPase activating protein 12 | 3.28E-06 | -11.7071 |
| 215092_s_at | NFAT5 | nuclear factor of activated T-cells 5, tonicity-responsive | 7.98E-06 | -11.7169 |
| 234942_s_at | DNTTIP1 | deoxynucleotidyltransferase, terminal, interacting protein 1 | 7.13E-09 | -11.7382 |
| 225182_at | TMEM50B | transmembrane protein 50B | 3.77E-07 | -11.7388 |
| 214055_x_at | BAT2L2 | HLA-B associated transcript 2-like 2 | 3.28E-05 | -11.7536 |
| 230256_at | C1orf104 | Chromosome 1 open reading frame 104 | 3.07E-06 | -11.7562 |
| 201631_s_at | IER3 | immediate early response 3 | 1.85E-07 | -11.7571 |
| 225780_at | RSC1A1 | regulatory solute carrier protein, family 1, member 1 | 1.21E-06 | -11.7683 |
| 212399_s_at | VGLL4 | vestigial like 4 (Drosophila) | 2.49E-07 | -11.7739 |
| 202946_s_at | BTBD3 | BTB (POZ) domain containing 3 | 9.17E-08 | -11.7871 |
| 229459_at | FAM19A5 | family with sequence similarity 19 (chemokine (C-C motif)-like), member A5 | 6.05E-07 | -11.7871 |
| 228450_at | PLEKHA7 | pleckstrin homology domain containing, family A member 7 | 5.22E-07 | -11.7933 |
| 212412_at | PDLIM5 | PDZ and LIM domain 5 | 9.64E-08 | -11.7947 |
| 212388_at | USP24 | ubiquitin specific peptidase 24 | 1.25E-05 | -11.7965 |
| 210612_s_at | SYNJ2 | synaptojanin 2 | 2.65E-06 | -11.7977 |
| 218396_at | VPS13C | vacuolar protein sorting 13 homolog C (S. cerevisiae) | 1.06E-05 | -11.7989 |
| 225158_at | GFM1 | G elongation factor, mitochondrial 1 | 3.33E-06 | -11.7991 |
| 216620_s_at | ARHGEF10 | Rho guanine nucleotide exchange factor (GEF) 10 | 6.89E-07 | -11.8031 |
| 222430_s_at | YTHDF2 | YTH domain family, member 2 | 5.09E-07 | -11.806 |
| 209307_at | SWAP70 | SWAP switching B-cell complex 70kDa subunit | 2.58E-07 | -11.8077 |
[truncated: 102,639 more chars]
