# Supplementary material for: Identifying mRNA, MicroRNA and Protein Profiles of Melanoma Exosomes
Source: PLoS One. 2012 Oct 9;7(10):e46874. doi: 10.1371/journal.pone.0046874 (PMC3467276; doi:10.1371/journal.pone.0046874)
Supplement: Table S2 — Differentially expressed mRNA probe sets in A375 exosomes versus A375 cells (FDR corrected p <0.01 and FC >2 or FC <−2). (DOCX) [file pone.0046874.s004.docx]

**Supplementary table S2. Differentially expressed mRNA probe sets in A375 exosomes versus A375 cells (FDR corrected *p* < 0.01 and FC > 2 or FC < -2)**

| Probeset ID | Gene Symbol | Gene Title | *p*-value | Fold Change |
| --- | --- | --- | --- | --- |
| 226018_at | C7orf41 | chromosome 7 open reading frame 41 | 1.55E-07 | 35.256 |
| 202125_s_at | TRAK2 | trafficking protein, kinesin binding 2 | 2.52E-11 | 23.9552 |
| 235054_at | NUDT16 | nudix (nucleoside diphosphate linked moiety X)-type motif 16 | 5.57E-10 | 23.4812 |
| 202437_s_at | CYP1B1 | cytochrome P450, family 1, subfamily B, polypeptide 1 | 6.97E-07 | 23.0524 |
| 219309_at | CTA-216E10.6 | hypothetical FLJ23584 | 1.77E-07 | 22.5127 |
| 225283_at | ARRDC4 | arrestin domain containing 4 | 3.17E-09 | 22.3204 |
| 219011_at | PLEKHA4 | pleckstrin homology domain containing, family A | 1.68E-08 | 20.9104 |
| 202436_s_at | CYP1B1 | cytochrome P450, family 1, subfamily B, polypeptide 1 | 4.77E-08 | 20.6014 |
| 202124_s_at | TRAK2 | trafficking protein, kinesin binding 2 | 1.63E-08 | 18.9632 |
| 228937_at | C13orf31 | chromosome 13 open reading frame 31 | 2.50E-06 | 17.4971 |
| 213252_at | SH3PXD2A | SH3 and PX domains 2A | 1.89E-06 | 14.5224 |
| 212845_at | SAMD4A | sterile alpha motif domain containing 4A | 4.76E-11 | 14.145 |
| 226234_at | GDF11 | growth differentiation factor 11 | 7.70E-09 | 14.0506 |
| 226977_at | C5orf53 | chromosome 5 open reading frame 53 | 5.18E-08 | 13.6913 |
| 201929_s_at | PKP4 | plakophilin 4 | 6.60E-08 | 13.2348 |
| 223469_at | PGPEP1 | pyroglutamyl-peptidase I | 1.43E-06 | 12.307 |
| 228857_at | GNL1 /// LOC285831 | guanine nucleotide binding protein-like 1 /// hypothetical protein LOC285831 | 1.51E-07 | 11.8758 |
| 236429_at | ZNF83 | zinc finger protein 83 | 1.09E-05 | 11.6237 |
| 201829_at | NET1 | neuroepithelial cell transforming 1 | 3.63E-06 | 11.244 |
| 228231_at | LOC100287081 | similar to hCG1999172 | 1.49E-07 | 11.1814 |
| 208740_at | SAP18 | Sin3A-associated protein, 18kDa | 2.88E-08 | 11.1473 |
| 202435_s_at | CYP1B1 | cytochrome P450, family 1, subfamily B, polypeptide 1 | 2.52E-07 | 10.3664 |
| 214414_x_at | HBA1 /// HBA2 | hemoglobin, alpha 1 /// hemoglobin, alpha 2 | 1.78E-08 | 9.909 |
| 212764_at | ZEB1 | zinc finger E-box binding homeobox 1 | 4.27E-05 | 9.12679 |
| 226656_at | CRTAP | cartilage associated protein | 8.06E-10 | 8.8118 |
| 224817_at | SH3PXD2A | SH3 and PX domains 2A | 1.64E-09 | 8.52468 |
| 208741_at | SAP18 | Sin3A-associated protein, 18kDa | 2.44E-06 | 8.47444 |
| 209311_at | BCL2L2 | BCL2-like 2 | 1.26E-07 | 8.25949 |
| 227274_at | SYNJ2BP | synaptojanin 2 binding protein | 7.50E-11 | 7.97198 |
| 1568597_at | LOC646762 | hypothetical LOC646762 | 8.63E-08 | 7.87492 |
| 202252_at | RAB13 | RAB13, member RAS oncogene family | 5.02E-13 | 7.81211 |
| 201928_at | PKP4 | plakophilin 4 | 3.30E-09 | 7.75047 |
| 223279_s_at | UACA | uveal autoantigen with coiled-coil domains and ankyrin repeats | 1.48E-08 | 7.58257 |
| 212801_at | CIT | citron (rho-interacting, serine/threonine kinase 21) | 1.32E-07 | 7.29697 |
| 203940_s_at | VASH1 | vasohibin 1 | 2.47E-07 | 7.25455 |
| 226232_at | GDF11 | growth differentiation factor 11 | 2.58E-05 | 6.98338 |
| 209244_s_at | KIF1C | kinesin family member 1C | 1.20E-05 | 6.89241 |
| 1553186_x_at | RASEF | RAS and EF-hand domain containing | 3.13E-07 | 6.88666 |
| 224763_at | RPL37 | Ribosomal protein L37 | 5.63E-07 | 6.83994 |
| 201927_s_at | PKP4 | plakophilin 4 | 2.41E-06 | 6.62404 |
| 235722_at | SYNJ2BP | synaptojanin 2 binding protein | 6.71E-06 | 6.59636 |
| 1553185_at | RASEF | RAS and EF-hand domain containing | 7.34E-07 | 6.56308 |
| 239952_at | ZEB1 | zinc finger E-box binding homeobox 1 | 5.73E-05 | 6.49761 |
| 201830_s_at | NET1 | neuroepithelial cell transforming 1 | 1.45E-06 | 6.40214 |
| 203853_s_at | GAB2 | GRB2-associated binding protein 2 | 2.45E-08 | 6.35743 |
| 1558075_at | LOC339047 | Hypothetical protein LOC339047 | 6.48E-07 | 6.25354 |
| 201010_s_at | TXNIP | thioredoxin interacting protein | 2.40E-07 | 6.14468 |
| 208120_x_at | FKSG49 /// tcag7.1056 | FKSG49 /// hypothetical LOC402469 | 4.36E-09 | 6.12653 |
| 234762_x_at | NLN | Neurolysin (metallopeptidase M3 family) | 6.58E-06 | 6.04818 |
| 214041_x_at | RPL37A | Ribosomal protein L37a | 0.0001722 | 5.99716 |
| 232266_x_at | CDC2L5 | Cell division cycle 2-like 5 (cholinesterase-related cell division controller) | 9.04E-07 | 5.94736 |
| 238853_at | RAB3IP | RAB3A interacting protein (rabin3) | 8.23E-06 | 5.92521 |
| 224836_at | TP53INP2 | tumor protein p53 inducible nuclear protein 2 | 1.78E-08 | 5.81758 |
| 231024_at | LOC572558 | hypothetical locus LOC572558 | 4.80E-05 | 5.73401 |
| 200897_s_at | PALLD | palladin, cytoskeletal associated protein | 4.08E-09 | 5.70875 |
| 237475_x_at | CCDC152 | coiled-coil domain containing 152 | 2.79E-06 | 5.70831 |
| 214993_at | ASPHD1 | aspartate beta-hydroxylase domain containing 1 | 4.01E-05 | 5.65905 |
| 226969_at | MTR | 5-methyltetrahydrofolate-homocysteine methyltransferase | 1.74E-06 | 5.60611 |
| 203590_at | DYNC1LI2 | dynein, cytoplasmic 1, light intermediate chain 2 | 1.39E-07 | 5.50814 |
| 228686_at | FLJ33630 | hypothetical LOC644873 | 2.89E-07 | 5.45656 |
| 200906_s_at | PALLD | palladin, cytoskeletal associated protein | 8.57E-06 | 5.44298 |
| 227164_at | SFRS1 | splicing factor, arginine/serine-rich 1 | 6.39E-06 | 5.32882 |
| 214313_s_at | EIF5B | Eukaryotic translation initiation factor 5B | 4.51E-06 | 5.31262 |
| 227138_at | CRTAP | cartilage associated protein | 3.15E-07 | 5.29419 |
| 1553997_a_at | ASPHD1 | aspartate beta-hydroxylase domain containing 1 | 3.67E-06 | 5.22599 |
| 224159_x_at | TRIM4 | tripartite motif-containing 4 | 3.20E-06 | 5.19446 |
| 224767_at | RPL37 | Ribosomal protein L37 | 4.89E-06 | 5.18643 |
| 224766_at | RPL37 | Ribosomal protein L37 | 1.32E-06 | 5.16001 |
| 226206_at | MAFK | v-maf musculoaponeurotic fibrosarcoma oncogene homolog K (avian) | 1.67E-06 | 5.15432 |
| 228318_s_at | CRIPAK | cysteine-rich PAK1 inhibitor | 8.66E-08 | 5.07608 |
| 202748_at | GBP2 | guanylate binding protein 2, interferon-inducible | 5.66E-06 | 5.07252 |
| 1566145_s_at | LOC644450 | hypothetical protein LOC644450 | 3.82E-08 | 5.05127 |
| 226591_at | SNRPN | small nuclear ribonucleoprotein polypeptide N | 2.20E-05 | 5.03792 |
| 224616_at | DYNC1LI2 | dynein, cytoplasmic 1, light intermediate chain 2 | 9.75E-09 | 5.01401 |
| 214722_at | NOTCH2NL | Notch homolog 2 (Drosophila) N-terminal like | 8.71E-07 | 5.01353 |
| 201009_s_at | TXNIP | thioredoxin interacting protein | 0.0001667 | 5.01283 |
| 200907_s_at | PALLD | palladin, cytoskeletal associated protein | 9.90E-05 | 5.01053 |
| 225146_at | C9orf25 | chromosome 9 open reading frame 25 | 3.54E-06 | 5.00245 |
| 231399_at | RAB3IP | RAB3A interacting protein (rabin3) | 3.54E-05 | 4.96982 |
| 205168_at | DDR2 | discoidin domain receptor tyrosine kinase 2 | 6.79E-06 | 4.9566 |
| 229893_at | FRMD3 | FERM domain containing 3 | 6.82E-06 | 4.94373 |
| 226084_at | MAP1B | microtubule-associated protein 1B | 1.65E-06 | 4.93386 |
| 212758_s_at | ZEB1 | zinc finger E-box binding homeobox 1 | 6.39E-06 | 4.93171 |
| 225442_at | DDR2 | discoidin domain receptor tyrosine kinase 2 | 9.26E-07 | 4.92289 |
| 203181_x_at | SRPK2 | SFRS protein kinase 2 | 6.97E-09 | 4.90363 |
| 224321_at | TMEFF2 | transmembrane protein with EGF-like and two follistatin-like domains 2 | 2.76E-08 | 4.8964 |
| 211454_x_at | FKSG49 | FKSG49 | 1.20E-07 | 4.88777 |
| 230645_at | FRMD3 | FERM domain containing 3 | 8.35E-05 | 4.80777 |
| 237202_at | PGPEP1 | pyroglutamyl-peptidase I | 1.85E-06 | 4.7324 |
| 206169_x_at | ZC3H7B | zinc finger CCCH-type containing 7B | 0.0007459 | 4.68864 |
| 214594_x_at | ATP8B1 | ATPase, class I, type 8B, member 1 | 0.0015865 | 4.6232 |
| 227561_at | DDR2 | discoidin domain receptor tyrosine kinase 2 | 2.90E-06 | 4.61482 |
| 216860_s_at | GDF11 | growth differentiation factor 11 | 1.06E-05 | 4.59877 |
| 212437_at | CENPB | centromere protein B, 80kDa | 5.00E-06 | 4.58848 |
| 223781_x_at | ADH4 | alcohol dehydrogenase 4 (class II), pi polypeptide | 1.13E-05 | 4.54198 |
| 1562062_at | KIAA1245 /// NBPF1 /// NBPF10 /// NBPF11 /// NBPF12 /// NBPF20 /// NBPF3 /// NBPF8 /// RP11-94I2.2 | KIAA1245 /// neuroblastoma breakpoint family, member 1 /// neuroblastoma breakpo | 2.55E-06 | 4.50777 |
| 224614_at | DYNC1LI2 | dynein, cytoplasmic 1, light intermediate chain 2 | 2.76E-05 | 4.49424 |
| 203170_at | RRP8 | ribosomal RNA processing 8, methyltransferase, homolog (yeast) | 8.62E-06 | 4.47173 |
| 239017_at | DVWA | dual von Willebrand factor A domains | 6.08E-06 | 4.40991 |
| 215208_x_at | RPL35A | Ribosomal protein L35a | 1.10E-05 | 4.39771 |
| 234562_x_at | LOC728678 | Similar to p40 | 0.0002235 | 4.3753 |
| 212233_at | MAP1B | microtubule-associated protein 1B | 2.99E-07 | 4.35884 |
| 221606_s_at | NSBP1 | nucleosomal binding protein 1 | 5.61E-07 | 4.35708 |
| 206792_x_at | PDE4C | phosphodiesterase 4C, cAMP-specific (phosphodiesterase E1 dunce homolog, Drosoph | 1.46E-05 | 4.35354 |
| 226587_at | SNRPN | small nuclear ribonucleoprotein polypeptide N | 0.0001134 | 4.31368 |
| 230076_at | PITPNM3 | PITPNM family member 3 | 1.58E-05 | 4.30589 |
| 210875_s_at | ZEB1 | zinc finger E-box binding homeobox 1 | 0.0005206 | 4.29676 |
| 238866_at | C19orf68 | chromosome 19 open reading frame 68 | 8.76E-05 | 4.28634 |
| 1555485_s_at | FAM153B | family with sequence similarity 153, member B | 1.40E-08 | 4.27261 |
| 211600_at | PTPRO | protein tyrosine phosphatase, receptor type, O | 2.73E-07 | 4.16957 |
| 226991_at | NFATC2 | Nuclear factor of activated T-cells, cytoplasmic, calcineurin-dependent 2 | 4.34E-07 | 4.16358 |
| 215588_x_at | RIOK3 | RIO kinase 3 (yeast) | 6.05E-06 | 4.16296 |
| 242578_x_at | SLC22A3 | Solute carrier family 22 (extraneuronal monoamine transporter), member 3 | 6.01E-06 | 4.15054 |
| 220232_at | SCD5 | stearoyl-CoA desaturase 5 | 8.62E-06 | 4.11926 |
| 220796_x_at | SLC35E1 | solute carrier family 35, member E1 | 0.0001015 | 4.10367 |
| 1566480_x_at | FLJ35848 | Hypothetical protein FLJ35848 | 0.0002133 | 4.09812 |
| 225207_at | PDK4 | pyruvate dehydrogenase kinase, isozyme 4 | 4.60E-07 | 4.09527 |
| 1563529_at | HYDIN2 | hydrocephalus inducing homolog 2 (mouse) | 4.86E-05 | 4.07195 |
| 1557261_at | WHAMML1 /// WHAMML2 | WAS protein homolog associated with actin, golgi membranes and microtubules-like | 1.94E-07 | 4.06735 |
| 201694_s_at | EGR1 | early growth response 1 | 7.15E-05 | 4.03717 |
| 1561171_a_at | LOC100132025 /// LOC283767 /// LOC727832 | transmembrane domain-containing protein ENSP00000320207-like /// golgi autoantig | 4.75E-06 | 4.03381 |
| 202840_at | TAF15 | TAF15 RNA polymerase II, TATA box binding protein (TBP)-associated factor, 68kDa | 0.0001097 | 4.02658 |
| 224667_x_at | C10orf104 | chromosome 10 open reading frame 104 | 5.34E-08 | 4.01199 |
| 218546_at | C1orf115 | chromosome 1 open reading frame 115 | 6.41E-09 | 4.00235 |
| 224493_x_at | C18orf45 | chromosome 18 open reading frame 45 | 9.48E-05 | 3.99765 |
| 221519_at | FBXW4 | F-box and WD repeat domain containing 4 | 1.53E-05 | 3.98021 |
| 214715_x_at | ZNF160 | zinc finger protein 160 | 8.93E-05 | 3.9792 |
| 1560119_at | LOC389634 | hypothetical LOC389634 | 2.15E-05 | 3.97869 |
| 244495_x_at | C18orf45 | chromosome 18 open reading frame 45 | 5.06E-05 | 3.96546 |
| 1558404_at | LOC644242 | Hypothetical protein LOC644242 | 1.70E-06 | 3.96345 |
| 1553909_x_at | FAM178A | family with sequence similarity 178, member A | 5.15E-06 | 3.96315 |
| 205113_at | NEFM | neurofilament, medium polypeptide | 4.14E-05 | 3.96274 |
| 215978_x_at | LOC152719 | hypothetical protein LOC152719 | 3.07E-07 | 3.95418 |
| 219156_at | SYNJ2BP | synaptojanin 2 binding protein | 8.48E-07 | 3.94057 |
| 231886_at | DKFZP434B2016 | similar to hypothetical protein LOC284701 | 0.0007157 | 3.92779 |
| 214102_at | ARAP2 | ArfGAP with RhoGAP domain, ankyrin repeat and PH domain 2 | 0.000191 | 3.90855 |
| 238701_x_at | C11orf92 | chromosome 11 open reading frame 92 | 8.89E-07 | 3.90442 |
| 207076_s_at | ASS1 | argininosuccinate synthetase 1 | 7.89E-06 | 3.89607 |
| 215599_at | LOC653188 | glucuronidase, beta pseudogene | 2.39E-05 | 3.87753 |
| 235132_at | LOC254128 | hypothetical protein LOC254128 | 0.000137 | 3.85866 |
| 212402_at | ZC3H13 | zinc finger CCCH-type containing 13 | 6.71E-10 | 3.85044 |
| 213382_at | MST1 /// MSTP2 /// MSTP9 | macrophage stimulating 1 (hepatocyte growth factor-like) /// macrophage stimulat | 6.01E-05 | 3.84917 |
| 242961_x_at | DDX58 | DEAD (Asp-Glu-Ala-Asp) box polypeptide 58 | 3.98E-05 | 3.83702 |
| 1553252_a_at | BRWD3 | bromodomain and WD repeat domain containing 3 | 3.46E-05 | 3.81308 |
| 1562063_x_at | KIAA1245 /// NBPF1 /// NBPF10 /// NBPF11 /// NBPF12 /// NBPF20 /// NBPF3 /// NBPF8 /// RP11-94I2.2 | KIAA1245 /// neuroblastoma breakpoint family, member 1 /// neuroblastoma breakpo | 1.08E-06 | 3.81099 |
| 244738_at | BRWD3 | bromodomain and WD repeat domain containing 3 | 6.16E-05 | 3.80479 |
| 227850_x_at | CDC42EP5 | CDC42 effector protein (Rho GTPase binding) 5 | 1.02E-05 | 3.79085 |
| 222771_s_at | MYEF2 | myelin expression factor 2 | 5.77E-06 | 3.77195 |
| 222576_s_at | EIF2C1 | eukaryotic translation initiation factor 2C, 1 | 8.82E-05 | 3.76198 |
| 232814_x_at | KLC1 | Kinesin light chain 1 | 1.42E-05 | 3.75179 |
| 216153_x_at | RECK | reversion-inducing-cysteine-rich protein with kazal motifs | 2.04E-07 | 3.74013 |
| 206323_x_at | OPHN1 | oligophrenin 1 | 4.61E-08 | 3.73414 |
| 236715_x_at | UACA | uveal autoantigen with coiled-coil domains and ankyrin repeats | 4.51E-06 | 3.72359 |
| 1566785_x_at | LOC728806 | Similar to Vesicle-fusing ATPase (Vesicular-fusion protein NSF) (N-ethylmaleimid | 6.67E-05 | 3.71639 |
| 215179_x_at | PGF | Placental growth factor | 1.93E-06 | 3.70402 |
| 213413_at | STON1 | stonin 1 | 0.0002268 | 3.68788 |
| 215404_x_at | FGFR1 | fibroblast growth factor receptor 1 | 7.68E-05 | 3.68407 |
| 234341_x_at | LOC91548 | hypothetical protein LOC91548 | 6.55E-08 | 3.65358 |
| 218859_s_at | ESF1 | ESF1, nucleolar pre-rRNA processing protein, homolog (S. cerevisiae) | 0.0002548 | 3.65056 |
| 220753_s_at | CRYL1 | crystallin, lambda 1 | 6.30E-08 | 3.64169 |
| 1568864_at | LOC100131691 | hypothetical protein LOC100131691 | 6.24E-05 | 3.6261 |
| 226868_at | GLT8D3 | glycosyltransferase 8 domain containing 3 | 2.95E-07 | 3.61716 |
| 228388_at | NFKBIB | nuclear factor of kappa light polypeptide gene enhancer in B-cells inhibitor, be | 5.22E-06 | 3.61157 |
| 210474_s_at | CDC2L1 /// CDC2L2 | cell division cycle 2-like 1 (PITSLRE proteins) /// cell division cycle 2-like 2 | 4.38E-06 | 3.60567 |
| 231018_at | PALM3 | Paralemmin-3 | 0.0001047 | 3.60094 |
| 212912_at | RPS6KA2 | ribosomal protein S6 kinase, 90kDa, polypeptide 2 | 2.00E-05 | 3.56914 |
| 223662_x_at | DDX59 | DEAD (Asp-Glu-Ala-Asp) box polypeptide 59 | 2.50E-06 | 3.56601 |
| 220828_s_at | FLJ11292 | hypothetical protein FLJ11292 | 7.61E-05 | 3.56074 |
| 223236_at | CCDC55 | coiled-coil domain containing 55 | 1.87E-06 | 3.54141 |
| 201008_s_at | TXNIP | thioredoxin interacting protein | 1.02E-05 | 3.53454 |
| 209959_at | NR4A3 | nuclear receptor subfamily 4, group A, member 3 | 1.13E-06 | 3.53069 |
| 1558579_at | FLJ37786 | hypothetical LOC642691 | 0.0008481 | 3.52524 |
| 201104_x_at | NBPF10 /// NBPF11 /// NBPF12 /// NBPF14 /// NBPF15 /// NBPF16 /// NBPF20 /// NBPF8 /// RP11-94I2.2 | neuroblastoma breakpoint family, member 10 /// neuroblastoma breakpoint family, | 1.48E-08 | 3.50407 |
| 201082_s_at | DCTN1 | dynactin 1 (p150, glued homolog, Drosophila) | 3.45E-06 | 3.49204 |
| 227536_at | ZC3H13 | zinc finger CCCH-type containing 13 | 1.69E-05 | 3.48849 |
| 233056_x_at | DLGAP4 | discs, large (Drosophila) homolog-associated protein 4 | 1.18E-05 | 3.46661 |
| 222409_at | CORO1C | coronin, actin binding protein, 1C | 1.97E-10 | 3.46633 |
| 232676_x_at | MYEF2 | myelin expression factor 2 | 1.30E-05 | 3.46498 |
| 202768_at | FOSB | FBJ murine osteosarcoma viral oncogene homolog B | 0.000114 | 3.45798 |
| 223707_at | RPL27A | ribosomal protein L27a | 0.0005978 | 3.45507 |
| 231124_x_at | LY9 | lymphocyte antigen 9 | 2.72E-06 | 3.44637 |
| 1560006_a_at | LOC646762 | hypothetical LOC646762 | 3.82E-06 | 3.44623 |
| 218642_s_at | CHCHD7 | coiled-coil-helix-coiled-coil-helix domain containing 7 | 6.94E-06 | 3.44406 |
| 219290_x_at | DAPP1 | dual adaptor of phosphotyrosine and 3-phosphoinositides | 1.14E-05 | 3.43798 |
| 221943_x_at | RPL38 | Ribosomal protein L38 | 4.66E-06 | 3.43242 |
| 1553142_at | C13orf31 | chromosome 13 open reading frame 31 | 2.35E-05 | 3.43109 |
| 215856_at | SIGLEC15 | sialic acid binding Ig-like lectin 15 | 4.83E-05 | 3.4291 |
| 214707_x_at | ALMS1 | Alstrom syndrome 1 | 5.68E-05 | 3.412 |
| 242889_x_at | LOC645431 | hypothetical LOC645431 | 1.67E-05 | 3.40797 |
| 239748_x_at | OCIAD1 | OCIA domain containing 1 | 3.27E-05 | 3.40335 |
| 216229_x_at | HCG2P7 | HLA complex group 2 pseudogene 7 | 0.0002344 | 3.39957 |
| 222891_s_at | BCL11A | B-cell CLL/lymphoma 11A (zinc finger protein) | 2.05E-05 | 3.39592 |
| 215600_x_at | FBXW12 | F-box and WD repeat domain containing 12 | 1.53E-05 | 3.3926 |
| 226062_x_at | FAM63A | family with sequence similarity 63, member A | 2.28E-05 | 3.38432 |
| 225817_at | CGNL1 | cingulin-like 1 | 1.27E-06 | 3.37365 |
| 204403_x_at | FAM115A | family with sequence similarity 115, member A | 4.65E-06 | 3.3724 |
| 232169_x_at | NDUFS8 | NADH dehydrogenase (ubiquinone) Fe-S protein 8, 23kDa (NADH-coenzyme Q reductase | 0.0001599 | 3.36921 |
| 1557633_at | POM121L8P | POM121 membrane glycoprotein-like 8 (rat) pseudogene | 0.0001939 | 3.36618 |
| 205730_s_at | ABLIM3 | actin binding LIM protein family, member 3 | 3.88E-09 | 3.36544 |
| 209726_at | CA11 | carbonic anhydrase XI | 2.71E-07 | 3.35983 |
| 224288_x_at | FKSG49 | FKSG49 | 6.61E-05 | 3.34756 |
| 233893_s_at | KIAA1530 | KIAA1530 | 2.80E-08 | 3.33974 |
| 1555140_a_at | BCL2L2 | BCL2-like 2 | 0.0001079 | 3.33445 |
| 225996_at | LONRF2 | LON peptidase N-terminal domain and ring finger 2 | 9.19E-06 | 3.32235 |
| 231770_x_at | C2orf86 | chromosome 2 open reading frame 86 | 0.0001698 | 3.31891 |
| 1567247_at | OR5H1 | olfactory receptor, family 5, subfamily H, member 1 | 0.0004084 | 3.30844 |
| 223697_x_at | C9orf64 | chromosome 9 open reading frame 64 | 3.20E-05 | 3.30156 |
| 1553335_x_at | LOC285696 | hypothetical LOC285696 | 0.0002192 | 3.3007 |
| 218966_at | MYO5C | myosin VC | 0.0002962 | 3.29902 |
| 235977_at | LONRF2 | LON peptidase N-terminal domain and ring finger 2 | 6.94E-05 | 3.28138 |
| 226740_x_at | NBPF1 /// NBPF10 /// NBPF11 /// NBPF14 /// NBPF15 /// NBPF16 /// NBPF20 /// NBPF8 /// RP11-94I2.2 | neuroblastoma breakpoint family, member 1 /// neuroblastoma breakpoint family, m | 2.41E-07 | 3.26901 |
| 220720_x_at | FAM128B | family with sequence similarity 128, member B | 4.20E-06 | 3.23307 |
| 220184_at | NANOG | Nanog homeobox | 6.64E-05 | 3.22629 |
| 215063_x_at | LRRC40 | leucine rich repeat containing 40 | 5.09E-06 | 3.22283 |
| 207428_x_at | CDC2L1 /// CDC2L2 | cell division cycle 2-like 1 (PITSLRE proteins) /// cell division cycle 2-like 2 | 2.11E-06 | 3.21565 |
| 208914_at | GGA2 | golgi associated, gamma adaptin ear containing, ARF binding protein 2 | 4.54E-06 | 3.21434 |
| 232215_x_at | PRR11 | proline rich 11 | 1.77E-05 | 3.21249 |
| 222252_x_at | UBQLN4 | ubiquilin 4 | 1.52E-05 | 3.20919 |
| 225205_at | KIF3B | kinesin family member 3B | 2.35E-08 | 3.2039 |
| 214693_x_at | NBPF10 | neuroblastoma breakpoint family, member 10 | 7.77E-05 | 3.1959 |
| 206081_at | SLC24A1 | solute carrier family 24 (sodium/potassium/calcium exchanger), member 1 | 1.64E-05 | 3.18677 |
| 243483_at | TRPM8 | transient receptor potential cation channel, subfamily M, member 8 | 7.53E-05 | 3.17706 |
| 204906_at | RPS6KA2 | ribosomal protein S6 kinase, 90kDa, polypeptide 2 | 0.0002106 | 3.17134 |
| 1555065_x_at | USP6 | ubiquitin specific peptidase 6 (Tre-2 oncogene) | 1.24E-05 | 3.16712 |
| 222207_x_at | LOC441258 | Williams Beuren syndrome chromosome region 19 pseudogene | 0.0003614 | 3.16409 |
| 226055_at | ARRDC2 | arrestin domain containing 2 | 0.0003462 | 3.15752 |
| 200696_s_at | GSN | gelsolin (amyloidosis, Finnish type) | 5.29E-07 | 3.15266 |
| 230109_at | PDE7B | phosphodiesterase 7B | 4.24E-05 | 3.14925 |
| 210686_x_at | SLC25A16 | solute carrier family 25 (mitochondrial carrier; Graves disease autoantigen), me | 2.75E-07 | 3.14822 |
| 220071_x_at | HAUS2 | HAUS augmin-like complex, subunit 2 | 0.0008438 | 3.13578 |
| 205599_at | TRAF1 | TNF receptor-associated factor 1 | 2.90E-05 | 3.12969 |
| 226905_at | FAM101B | family with sequence similarity 101, member B | 9.88E-08 | 3.11243 |
| 232731_x_at | LOC100190938 | hypothetical LOC100190938 | 0.001542 | 3.1114 |
| 242377_x_at | THUMPD3 | THUMP domain containing 3 | 3.82E-06 | 3.10567 |
| 231992_x_at | LOC493754 | RAB guanine nucleotide exchange factor (GEF) 1 pseudogene | 5.22E-06 | 3.09529 |
| 242418_at | C2orf27A | chromosome 2 open reading frame 27A | 0.0002278 | 3.09328 |
| 221238_at | NSBP1 | nucleosomal binding protein 1 | 0.0005606 | 3.09292 |
| 205334_at | S100A1 | S100 calcium binding protein A1 | 0.0003025 | 3.09028 |
| 222444_at | ARMCX3 | armadillo repeat containing, X-linked 3 | 0.0006997 | 3.08702 |
| 215311_at | NTRK3 | neurotrophic tyrosine kinase, receptor, type 3 | 1.95E-06 | 3.06216 |
| 227992_s_at | NCRNA00085 | non-protein coding RNA 85 | 0.0003287 | 3.05885 |
| 233868_x_at | ADAM33 | ADAM metallopeptidase domain 33 | 1.44E-07 | 3.05538 |
| 233831_at | LOC100289465 /// LOC100291666 | similar to serologically defined breast cancer antigen NY-BR-40 /// similar to s | 0.0006133 | 3.05127 |
| 224837_at | FOXP1 | forkhead box P1 | 9.06E-07 | 3.0452 |
| 215067_x_at | PRDX2 | peroxiredoxin 2 | 0.0001417 | 3.02722 |
| 226141_at | CCDC149 | coiled-coil domain containing 149 | 8.58E-05 | 3.0217 |
| 207608_x_at | CYP1A2 | cytochrome P450, family 1, subfamily A, polypeptide 2 | 2.05E-05 | 3.01837 |
| 222253_s_at | POM121L9P | POM121 membrane glycoprotein-like 9 (rat) pseudogene | 5.94E-05 | 3.017 |
| 1570566_at | LOC100292875 | hypothetical protein LOC100292875 | 6.89E-05 | 3.01324 |
| 210718_s_at | ARL17P1 /// LOC100294341 | ADP-ribosylation factor-like 17 pseudogene 1 /// similar to ADP-ribosylation fac | 0.000375 | 3.0094 |
| 205370_x_at | DBT | dihydrolipoamide branched chain transacylase E2 | 7.92E-05 | 3.00549 |
| 1553471_at | AMAC1 | acyl-malonyl condensing enzyme 1 | 1.13E-05 | 2.98339 |
| 209864_at | FRAT2 | frequently rearranged in advanced T-cell lymphomas 2 | 0.0003959 | 2.98094 |
| 206571_s_at | MAP4K4 | mitogen-activated protein kinase kinase kinase kinase 4 | 1.14E-07 | 2.98022 |
| 1558982_at | LOC375010 | ankyrin repeat domain 20 family, member A pseudogene | 0.0001636 | 2.96835 |
| 229264_at | LOC100292701 | hypothetical LOC100292701 | 2.06E-06 | 2.96638 |
| 234665_x_at | HHLA3 | HERV-H LTR-associating 3 | 8.05E-05 | 2.96173 |
| 209019_s_at | PINK1 | PTEN induced putative kinase 1 | 5.82E-05 | 2.95729 |
| 1570210_x_at | SAPS2 | SAPS domain family, member 2 | 0.0001118 | 2.95577 |
| 242344_at | GABRB2 | gamma-aminobutyric acid (GABA) A receptor, beta 2 | 0.0001017 | 2.94009 |
| 217137_x_at | LOC100291812 | hypothetical protein LOC100291812 | 0.0004638 | 2.93762 |
| 201306_s_at | ANP32B | acidic (leucine-rich) nuclear phosphoprotein 32 family, member B | 3.50E-10 | 2.93472 |
| 227717_at | FLJ41603 | FLJ41603 protein | 2.72E-05 | 2.92916 |
| 227917_at | FAM85A | family with sequence similarity 85, member A | 0.0003577 | 2.9197 |
| 227792_at | ITPRIPL2 | inositol 1,4,5-triphosphate receptor interacting protein-like 2 | 2.07E-05 | 2.9183 |
| 242829_x_at | FBXL3 | F-box and leucine-rich repeat protein 3 | 5.84E-05 | 2.91338 |
| 227279_at | TCEAL3 | transcription elongation factor A (SII)-like 3 | 7.37E-06 | 2.9121 |
| 215529_x_at | DIP2A | DIP2 disco-interacting protein 2 homolog A (Drosophila) | 0.0002282 | 2.90993 |
| 213205_s_at | RAD54L2 | RAD54-like 2 (S. cerevisiae) | 4.65E-06 | 2.90609 |
| 1553672_at | ENAH | enabled homolog (Drosophila) | 6.23E-06 | 2.89787 |
| 229447_x_at | NBPF11 /// NBPF20 /// NBPF8 /// RP11-94I2.2 | neuroblastoma breakpoint family, member 11 /// neuroblastoma breakpoint family, | 8.07E-05 | 2.89683 |
| 1560080_at | DIAPH1 | Diaphanous homolog 1 (Drosophila) | 3.54E-06 | 2.89652 |
| 235153_at | RNF183 | ring finger protein 183 | 8.12E-05 | 2.89461 |
| 215002_at | LOC100132247 /// LOC100288332 /// LOC348162 /// LOC728734 /// LOC728888 /// LOC729602 /// LOC729978 /// LOC730153 /// NPIPL2 /// NPIPL3 | similar to Uncharacterized protein KIAA0220 /// similar to acyl-CoA synthetase m | 5.90E-05 | 2.89291 |
| 1561477_at | CCDC144A | coiled-coil domain containing 144A | 0.0015472 | 2.88213 |
| 1556704_s_at | LOC100133920 /// LOC286297 | hypothetical protein LOC100133920 /// hypothetical protein LOC286297 | 0.0004027 | 2.87845 |
| 1555141_a_at | NHEDC1 | Na+/H+ exchanger domain containing 1 | 0.0001346 | 2.87448 |
| 224425_x_at | FKSG73 | ARP3 actin-related protein 3 homolog B pseudogene | 0.0001398 | 2.87085 |
| 208137_x_at | ZNF611 | zinc finger protein 611 | 0.0001149 | 2.86827 |
| 202760_s_at | PALM2-AKAP2 | PALM2-AKAP2 readthrough transcript | 0.001548 | 2.86783 |
| 220113_x_at | POLR1B | polymerase (RNA) I polypeptide B, 128kDa | 2.82E-05 | 2.86618 |
| 1556929_at | PAPOLG | Poly(A) polymerase gamma | 0.0001612 | 2.86415 |
| 233338_at | LOC284861 | hypothetical LOC284861 | 0.0006811 | 2.86393 |
| 227404_s_at | EGR1 | Early growth response 1 | 0.0009981 | 2.86133 |
| 232702_at | RABGAP1L | RAB GTPase activating protein 1-like | 6.00E-07 | 2.85856 |
| 201693_s_at | EGR1 | early growth response 1 | 0.0005631 | 2.85602 |
| 231424_at | SLC5A12 | solute carrier family 5 (sodium/glucose cotransporter), member 12 | 0.0011102 | 2.85107 |
| 227461_at | STON2 | stonin 2 | 8.39E-06 | 2.84458 |
| 218155_x_at | TSR1 | TSR1, 20S rRNA accumulation, homolog (S. cerevisiae) | 3.52E-06 | 2.84446 |
| 205954_at | RXRG | retinoid X receptor, gamma | 0.0011204 | 2.84209 |
| 36711_at | MAFF | v-maf musculoaponeurotic fibrosarcoma oncogene homolog F (avian) | 1.20E-05 | 2.83523 |
| 220725_x_at | DNAH3 | Dynein, axonemal, heavy chain 3 | 1.27E-08 | 2.83038 |
| 201027_s_at | EIF5B | eukaryotic translation initiation factor 5B | 6.18E-06 | 2.82901 |
| 201103_x_at | NBPF10 /// NBPF11 /// NBPF15 /// NBPF16 /// NBPF20 /// NBPF8 /// RP11-94I2.2 | neuroblastoma breakpoint family, member 10 /// neuroblastoma breakpoint family, | 1.41E-07 | 2.82581 |
| 1566514_at | CWF19L2 | CWF19-like 2, cell cycle control (S. pombe) | 0.0009498 | 2.81866 |
| 206088_at | LRRC37A /// LRRC37A2 /// LRRC37A3 | leucine rich repeat containing 37A /// leucine rich repeat containing 37, member | 0.0002789 | 2.81583 |
| 242114_at | BOLA2 | BolA homolog 2 (E. coli) | 0.0001582 | 2.8059 |
| 1559315_s_at | LOC144481 | hypothetical protein LOC144481 | 0.0005833 | 2.79482 |
| 215383_x_at | SPG21 | spastic paraplegia 21 (autosomal recessive, Mast syndrome) | 1.12E-05 | 2.79357 |
| 204041_at | MAOB | monoamine oxidase B | 2.36E-05 | 2.7932 |
| 220335_x_at | CES3 | carboxylesterase 3 | 3.20E-05 | 2.78179 |
| 203943_at | KIF3B | kinesin family member 3B | 1.22E-05 | 2.7816 |
| 208861_s_at | ATRX | alpha thalassemia/mental retardation syndrome X-linked (RAD54 homolog, S. cerevi | 2.81E-07 | 2.78059 |
| 1565620_at | AGAP4 | ArfGAP with GTPase domain, ankyrin repeat and PH domain 4 | 3.71E-05 | 2.76999 |
| 241975_at | LOC399959 | Hypothetical gene supported by BX647608 | 6.00E-05 | 2.76654 |
| 1563853_at | LOC283045 | hypothetical protein LOC283045 | 2.93E-05 | 2.76492 |
| 239629_at | CFLAR | CASP8 and FADD-like apoptosis regulator | 2.24E-05 | 2.76298 |
| 228160_at | LOC339290 | hypothetical LOC339290 | 4.39E-06 | 2.76128 |
| 1554642_at | RNF32 | ring finger protein 32 | 1.91E-05 | 2.75758 |
| 1563674_at | FCRL2 | Fc receptor-like 2 | 2.02E-05 | 2.75421 |
| 215582_x_at | MCM3AP | minichromosome maintenance complex component 3 associated protein | 0.0001512 | 2.74478 |
| 1553674_at | LRRIQ3 | leucine-rich repeats and IQ motif containing 3 | 6.34E-05 | 2.74467 |
| 1562428_at | LOC654780 | SFPQ | 5.51E-05 | 2.73757 |
| 1555264_a_at | LOC646982 | twelve-thirteen translocation leukemia gene | 3.45E-05 | 2.73446 |
| 201305_x_at | ANP32B | acidic (leucine-rich) nuclear phosphoprotein 32 family, member B | 4.80E-06 | 2.73103 |
| 1559810_at | LOC642313 | hypothetical LOC642313 | 5.61E-06 | 2.73086 |
| 213612_x_at | NBPF10 /// NBPF15 /// NBPF16 /// NBPF20 /// NBPF8 | neuroblastoma breakpoint family, member 10 /// neuroblastoma breakpoint family, | 2.06E-09 | 2.72763 |
| 202180_s_at | MVP | major vault protein | 1.62E-06 | 2.72702 |
| 228697_at | HINT3 | histidine triad nucleotide binding protein 3 | 4.70E-05 | 2.72635 |
| 1569557_at | ZNF248 | zinc finger protein 248 | 1.33E-05 | 2.72539 |
| 1562792_at | NIPAL1 | NIPA-like domain containing 1 | 0.0001113 | 2.72492 |
| 1565483_at | EGFR | epidermal growth factor receptor (erythroblastic leukemia viral (v-erb-b) oncoge | 0.0011743 | 2.72201 |
| 241874_at | C5orf53 | chromosome 5 open reading frame 53 | 8.22E-06 | 2.72152 |
| 232168_x_at | MACF1 | microtubule-actin crosslinking factor 1 | 4.45E-05 | 2.72021 |
| 225111_s_at | NAPB | N-ethylmaleimide-sensitive factor attachment protein, beta | 2.47E-06 | 2.71442 |
| 226795_at | LRCH1 | leucine-rich repeats and calponin homology (CH) domain containing 1 | 0.0001398 | 2.71437 |
| 201481_s_at | PYGB | phosphorylase, glycogen; brain | 0.0003233 | 2.71359 |
| 237909_at | ADAM6 | ADAM metallopeptidase domain 6 (pseudogene) | 3.13E-06 | 2.71272 |
| 1560199_x_at | LOC728153 | similar to FAM133B protein | 0.0001513 | 2.70737 |
| 201024_x_at | EIF5B | eukaryotic translation initiation factor 5B | 1.16E-06 | 2.7065 |
| 1562974_at | LOC100133899 | hypothetical protein LOC100133899 | 1.18E-05 | 2.70599 |
| 1554332_a_at | hCG_2018279 | hypothetical protein LOC100127888 | 4.02E-05 | 2.70251 |
| 203525_s_at | APC | adenomatous polyposis coli | 3.61E-05 | 2.69372 |
| 220920_at | ATP10B | ATPase, class V, type 10B | 6.38E-06 | 2.69366 |
| 207133_x_at | ALPK1 | alpha-kinase 1 | 6.67E-06 | 2.68833 |
| 222187_x_at | G3BP1 | GTPase activating protein (SH3 domain) binding protein 1 | 0.0001029 | 2.68767 |
| 204290_s_at | ALDH6A1 | aldehyde dehydrogenase 6 family, member A1 | 0.0004846 | 2.68702 |
| 206228_at | PAX2 | paired box 2 | 7.07E-05 | 2.68303 |
| 209018_s_at | PINK1 | PTEN induced putative kinase 1 | 1.80E-05 | 2.67349 |
| 237786_at | MAGI2 | Membrane associated guanylate kinase, WW and PDZ domain containing 2 | 0.0003409 | 2.67156 |
| 220411_x_at | PODNL1 | podocan-like 1 | 2.91E-05 | 2.67034 |
| 242077_x_at | C6orf150 | chromosome 6 open reading frame 150 | 7.19E-07 | 2.66873 |
| 1568780_at | LOC649305 | hypothetical LOC649305 | 0.0010181 | 2.66847 |
| 216045_at | CCDC144A | coiled-coil domain containing 144A | 0.0003958 | 2.66708 |
| 218181_s_at | MAP4K4 | mitogen-activated protein kinase kinase kinase kinase 4 | 2.42E-06 | 2.66702 |
| 203527_s_at | APC | adenomatous polyposis coli | 0.0011439 | 2.66153 |
| 212401_s_at | CDC2L2 | cell division cycle 2-like 2 (PITSLRE proteins) | 2.66E-08 | 2.66111 |
| 215228_at | NHLH2 | nescient helix loop helix 2 | 0.0002095 | 2.65293 |
| 212028_at | RBM25 | RNA binding motif protein 25 | 3.09E-06 | 2.65105 |
| 211289_x_at | CDC2L1 /// CDC2L2 | cell division cycle 2-like 1 (PITSLRE proteins) /// cell division cycle 2-like 2 | 4.82E-05 | 2.65077 |
| 212791_at | C1orf216 | chromosome 1 open reading frame 216 | 6.21E-06 | 2.64711 |
| 226178_at | SOCS4 | suppressor of cytokine signaling 4 | 0.0001095 | 2.64559 |
| 226694_at | AKAP2 /// PALM2-AKAP2 | A kinase (PRKA) anchor protein 2 /// PALM2-AKAP2 readthrough transcript | 0.0008995 | 2.6397 |
| 1557347_at | MCPH1 | microcephalin 1 | 0.0001802 | 2.63825 |
| 1554810_at | PLA2G4C | phospholipase A2, group IVC (cytosolic, calcium-independent) | 1.60E-05 | 2.63716 |
| 206286_s_at | TDGF1 /// TDGF3 | teratocarcinoma-derived growth factor 1 /// teratocarcinoma-derived growth facto | 0.0001765 | 2.63566 |
| 219823_at | LIN28 | lin-28 homolog (C. elegans) | 9.15E-05 | 2.63218 |
| 201026_at | EIF5B | eukaryotic translation initiation factor 5B | 0.0002062 | 2.6286 |
| 207010_at | GABRB1 | gamma-aminobutyric acid (GABA) A receptor, beta 1 | 0.0007759 | 2.62825 |
| 239318_at | FAM118B | family with sequence similarity 118, member B | 0.0016853 | 2.62485 |
| 239481_at | FAM133A | family with sequence similarity 133, member A | 0.0007657 | 2.62308 |
| 208831_x_at | SUPT6H | suppressor of Ty 6 homolog (S. cerevisiae) | 7.14E-06 | 2.62186 |
| 225803_at | FBXO32 | F-box protein 32 | 4.56E-05 | 2.62171 |
| 242600_at | FRMD3 | FERM domain containing 3 | 3.88E-05 | 2.62124 |
| 218697_at | NCKIPSD | NCK interacting protein with SH3 domain | 3.39E-05 | 2.62094 |
| 229849_at | WIPF3 | WAS/WASL interacting protein family, member 3 | 2.39E-05 | 2.6152 |
| 1561937_x_at | IGHA1 /// IGHG1 /// IGHM /// IGHV4-31 /// LOC100287372 /// LOC100291056 /// LOC100293211 | immunoglobulin heavy constant alpha 1 /// immunoglobulin heavy constant gamma 1 | 0.0002273 | 2.61497 |
| 236130_at | SNORA37 | small nucleolar RNA, H/ACA box 37 | 0.0002904 | 2.61165 |
| 207227_x_at | RFPL2 | ret finger protein-like 2 | 0.0002256 | 2.60981 |
| 207135_at | HTR2A | 5-hydroxytryptamine (serotonin) receptor 2A | 7.06E-05 | 2.6083 |
| 203305_at | F13A1 | coagulation factor XIII, A1 polypeptide | 6.52E-05 | 2.60823 |
| 216310_at | TAOK1 | TAO kinase 1 | 2.47E-05 | 2.60818 |
| 208411_x_at | PPEF2 | protein phosphatase, EF-hand calcium binding domain 2 | 8.54E-06 | 2.60802 |
| 237395_at | CYP4Z1 | cytochrome P450, family 4, subfamily Z, polypeptide 1 | 0.0005061 | 2.60483 |
| 240111_at | RHOBTB3 | Rho-related BTB domain containing 3 | 2.19E-05 | 2.60395 |
| 235677_at | SRR | Serine racemase | 4.67E-07 | 2.59638 |
| 1556606_at | NAV2 | neuron navigator 2 | 1.88E-05 | 2.59626 |
| 1554786_at | CASS4 | Cas scaffolding protein family member 4 | 0.0001174 | 2.58659 |
| 1563863_x_at | TCEANC | transcription elongation factor A (SII) N-terminal and central domain containing | 0.0001808 | 2.58515 |
| 242712_x_at | RANBP2 /// RGPD1 /// RGPD2 /// RGPD3 /// RGPD4 /// RGPD5 /// RGPD6 /// RGPD7 /// RGPD8 | RAN binding protein 2 /// RANBP2-like and GRIP domain containing 1 /// RANBP2-li | 0.0006362 | 2.58514 |
| 216459_x_at | TIGD1L | tigger transposable element derived 1-like | 1.07E-05 | 2.58509 |
| 242608_x_at | FAM161B | Family with sequence similarity 161, member B | 3.20E-05 | 2.58128 |
| 228370_at | SNRPN | small nuclear ribonucleoprotein polypeptide N | 0.0004096 | 2.58083 |
| 212610_at | PTPN11 | protein tyrosine phosphatase, non-receptor type 11 | 2.25E-05 | 2.57932 |
| 231876_at | TRIM56 | tripartite motif-containing 56 | 9.06E-05 | 2.57761 |
| 207365_x_at | USP34 | ubiquitin specific peptidase 34 | 1.43E-05 | 2.56739 |
| 214605_x_at | GPR1 | G protein-coupled receptor 1 | 0.001187 | 2.56373 |
| 200595_s_at | EIF3A | eukaryotic translation initiation factor 3, subunit A | 0.0002309 | 2.55807 |
| 214662_at | WDR43 | WD repeat domain 43 | 3.23E-05 | 2.55789 |
| 238430_x_at | SLFN5 | schlafen family member 5 | 0.0001832 | 2.55733 |
| 227514_at | ITPRIPL2 | inositol 1,4,5-triphosphate receptor interacting protein-like 2 | 0.0016353 | 2.55605 |
| 1560201_at | ZNF713 | zinc finger protein 713 | 8.62E-05 | 2.55319 |
| 232244_at | KIAA1161 | KIAA1161 | 0.0008119 | 2.55208 |
| 1553153_at | ATP6V0D2 | ATPase, H+ transporting, lysosomal 38kDa, V0 subunit d2 | 0.0002243 | 2.55155 |
| 201025_at | EIF5B | eukaryotic translation initiation factor 5B | 7.29E-05 | 2.55043 |
| 216660_at | MYO7B | myosin VIIB | 7.11E-05 | 2.54687 |
| 1556643_at | LOC100128718 | Hypothetical protein LOC100128718 | 0.0004426 | 2.54627 |
| 209896_s_at | PTPN11 | protein tyrosine phosphatase, non-receptor type 11 | 4.53E-05 | 2.54581 |
| 222665_at | FAM82B | family with sequence similarity 82, member B | 1.92E-05 | 2.54318 |
| 202052_s_at | RAI14 | retinoic acid induced 14 | 8.37E-07 | 2.5425 |
| 227000_at | C7orf41 | chromosome 7 open reading frame 41 | 0.0005098 | 2.54191 |
| 231825_x_at | ATF7IP | activating transcription factor 7 interacting protein | 5.19E-06 | 2.53881 |
| 239390_at | GTDC1 | glycosyltransferase-like domain containing 1 | 0.0008433 | 2.53798 |
| 1554516_at | LOC203274 | Hypothetical protein LOC203274 | 0.0004084 | 2.53346 |
| 1553878_at | GOT1L1 | glutamic-oxaloacetic transaminase 1-like 1 | 0.0002297 | 2.5327 |
| 209291_at | ID4 | inhibitor of DNA binding 4, dominant negative helix-loop-helix protein | 6.44E-05 | 2.53103 |
| 202759_s_at | AKAP2 /// PALM2-AKAP2 | A kinase (PRKA) anchor protein 2 /// PALM2-AKAP2 readthrough transcript | 1.24E-05 | 2.53039 |
| 213936_x_at | SFTPB | surfactant protein B | 0.0002511 | 2.52687 |
| 224632_at | GPATCH4 | G patch domain containing 4 | 0.0001116 | 2.52673 |
| 1568619_s_at | ITPRIPL2 | inositol 1,4,5-triphosphate receptor interacting protein-like 2 | 5.63E-05 | 2.52618 |
| 1562267_s_at | ZNF709 | zinc finger protein 709 | 0.0004433 | 2.52567 |
| 226100_at | MLL5 | myeloid/lymphoid or mixed-lineage leukemia 5 (trithorax homolog, Drosophila) | 0.0004838 | 2.52331 |
| 206551_x_at | KLHL24 | kelch-like 24 (Drosophila) | 0.0004127 | 2.52305 |
| 1560445_x_at | ARHGEF1 | Rho guanine nucleotide exchange factor (GEF) 1 | 1.30E-05 | 2.5212 |
| 211481_at | SLCO1A2 | solute carrier organic anion transporter family, member 1A2 | 0.0001422 | 2.5197 |
| 233536_at | ASXL3 | additional sex combs like 3 (Drosophila) | 6.78E-06 | 2.5194 |
| 241133_at | TRBV27 | T cell receptor beta variable 27 | 2.18E-06 | 2.51864 |
| 210368_at | PCDHGA8 | protocadherin gamma subfamily A, 8 | 0.0009856 | 2.5102 |
| 211452_x_at | LRRFIP1 | leucine rich repeat (in FLII) interacting protein 1 | 5.16E-05 | 2.5085 |
| 203244_at | PEX5 | peroxisomal biogenesis factor 5 | 5.61E-07 | 2.50737 |
| 1559616_x_at | ZNF626 | zinc finger protein 626 | 0.0001181 | 2.50659 |
| 209895_at | PTPN11 | protein tyrosine phosphatase, non-receptor type 11 | 0.0001735 | 2.50641 |
| 219451_at | MSRB2 | methionine sulfoxide reductase B2 | 0.0001318 | 2.50323 |
| 200597_at | EIF3A | eukaryotic translation initiation factor 3, subunit A | 6.57E-05 | 2.50199 |
| 232533_at | METTL8 | methyltransferase like 8 | 7.37E-05 | 2.50124 |
| 242662_at | PCSK6 | Proprotein convertase subtilisin/kexin type 6 | 0.0005677 | 2.49906 |
| 223924_at | TTC25 | tetratricopeptide repeat domain 25 | 0.0003661 | 2.4968 |
| 242531_at | RRAGC | Ras-related GTP binding C | 0.0011911 | 2.49602 |
| 213876_x_at | ZRSR2 | zinc finger (CCCH type), RNA-binding motif and serine/arginine rich 2 | 0.0005758 | 2.49487 |
| 226484_at | ZBTB47 | zinc finger and BTB domain containing 47 | 5.73E-06 | 2.4904 |
| 202553_s_at | SYF2 | SYF2 homolog, RNA splicing factor (S. cerevisiae) | 0.0003286 | 2.4901 |
| 215563_s_at | MSTP9 | macrophage stimulating, pseudogene 9 | 0.000779 | 2.48888 |
| 229110_at | LOC100128844 | Hypothetical protein LOC100128844 | 0.0013594 | 2.48365 |
| 200596_s_at | EIF3A | eukaryotic translation initiation factor 3, subunit A | 1.75E-05 | 2.48305 |
| 232196_at | LCA5L | Leber congenital amaurosis 5-like | 0.0001721 | 2.48068 |
| 1557104_at | ZNF397OS | Zinc finger protein 397 opposite strand | 6.73E-05 | 2.48065 |
| 222921_s_at | HEY2 | hairy/enhancer-of-split related with YRPW motif 2 | 0.0003886 | 2.47368 |
| 234312_s_at | ACSS2 | acyl-CoA synthetase short-chain family member 2 | 0.0001036 | 2.47194 |
| 200606_at | DSP | desmoplakin | 8.72E-05 | 2.47102 |
| 225514_at | C14orf21 | chromosome 14 open reading frame 21 | 2.56E-06 | 2.4705 |
| 220252_x_at | CXorf21 | chromosome X open reading frame 21 | 0.0004202 | 2.46666 |
| 206746_at | BFSP1 | beaded filament structural protein 1, filensin | 3.99E-06 | 2.46655 |
| 208420_x_at | SUPT6H | suppressor of Ty 6 homolog (S. cerevisiae) | 1.39E-05 | 2.46488 |
| 1570351_at | ADAMTS6 | ADAM metallopeptidase with thrombospondin type 1 motif, 6 | 0.0003717 | 2.46431 |
| 225116_at | HIPK2 | homeodomain interacting protein kinase 2 | 9.43E-06 | 2.46286 |
| 65585_at | FAM86B1 | family with sequence similarity 86, member B1 | 2.56E-05 | 2.46257 |
| 232644_x_at | OCIAD1 | OCIA domain containing 1 | 6.34E-07 | 2.46119 |
| 1558784_at | LOC100133089 | hypothetical protein LOC100133089 | 3.01E-06 | 2.46093 |
| 217016_x_at | TMEM212 | transmembrane protein 212 | 0.0013267 | 2.46073 |
| 232420_x_at | hCG_2022304 | similar to hCG2022304 | 6.24E-05 | 2.45849 |
| 211616_s_at | HTR2A | 5-hydroxytryptamine (serotonin) receptor 2A | 0.0007259 | 2.45774 |
| 208174_x_at | ZRSR2 | zinc finger (CCCH type), RNA-binding motif and serine/arginine rich 2 | 5.10E-07 | 2.4572 |
| 1564559_at | LOC728073 | hypothetical protein LOC728073 | 0.000408 | 2.45465 |
| 233720_at | SORBS2 | Sorbin and SH3 domain containing 2 | 1.34E-06 | 2.44893 |
| 213197_at | ASTN1 | astrotactin 1 | 5.29E-05 | 2.44635 |
| 1561170_at | LOC100132025 | transmembrane domain-containing protein ENSP00000320207-like | 0.0008367 | 2.44593 |
| 206056_x_at | SPN | sialophorin | 2.19E-05 | 2.43752 |
| 228396_at | PRKG1 | protein kinase, cGMP-dependent, type I | 0.000416 | 2.43701 |
| 216415_at | DNAH3 | Dynein, axonemal, heavy chain 3 | 0.0014164 | 2.43684 |
| 213089_at | LOC100272216 | hypothetical LOC100272216 | 4.49E-06 | 2.43635 |
| 208807_s_at | CHD3 | chromodomain helicase DNA binding protein 3 | 2.53E-05 | 2.43557 |
| 212566_at | MAP4 | microtubule-associated protein 4 | 1.66E-05 | 2.43398 |
| 211994_at | WNK1 | WNK lysine deficient protein kinase 1 | 4.58E-07 | 2.43348 |
| 213933_at | PTGER3 | prostaglandin E receptor 3 (subtype EP3) | 0.0018384 | 2.43168 |
| 1569583_at | EREG | epiregulin | 0.0003455 | 2.42996 |
| 219028_at | HIPK2 | homeodomain interacting protein kinase 2 | 0.0007322 | 2.42985 |
| 234798_x_at | LOC100287547 | hypothetical protein LOC100287547 | 6.49E-06 | 2.4241 |
| 218487_at | ALAD | aminolevulinate, delta-, dehydratase | 0.000118 | 2.42405 |
| 1558725_at | FLJ37078 | Hypothetical protein FLJ37078 | 0.0005294 | 2.424 |
| 1556528_at | LOC285326 | hypothetical protein LOC285326 | 0.0001397 | 2.42244 |
| 225986_x_at | CPSF2 | cleavage and polyadenylation specific factor 2, 100kDa | 2.36E-06 | 2.42119 |
| 1569886_a_at | GLB1L3 | galactosidase, beta 1-like 3 | 0.000389 | 2.41998 |
| 1558640_a_at | LOC728411 | Glucuronidase, beta pseudogene | 0.0001517 | 2.41941 |
| 239688_at | SMC1A | structural maintenance of chromosomes 1A | 0.0002706 | 2.41771 |
| 235361_at | STAMBP | STAM binding protein | 2.26E-05 | 2.41561 |
| 207835_at | FBLN1 | fibulin 1 | 0.0007448 | 2.41262 |
| 217760_at | TRIM44 | tripartite motif-containing 44 | 4.23E-06 | 2.41167 |
| 1555756_a_at | CLEC7A | C-type lectin domain family 7, member A | 1.06E-05 | 2.41003 |
| 1564362_x_at | ZNF843 | zinc finger protein 843 | 9.23E-05 | 2.40809 |
| 211932_at | HNRNPA3 | heterogeneous nuclear ribonucleoprotein A3 | 0.0008262 | 2.40803 |
| 216188_at | MYCNOS | v-myc myelocytomatosis viral related oncogene, neuroblastoma derived (avian) opp | 0.0001769 | 2.40454 |
| 216189_at | LOC100289109 | hypothetical protein LOC100289109 | 0.0002666 | 2.40239 |
| 211585_at | NPAT | nuclear protein, ataxia-telangiectasia locus | 0.000198 | 2.40138 |
| 213644_at | CCDC46 | coiled-coil domain containing 46 | 9.90E-06 | 2.40064 |
| 233399_x_at | ZNF252 | Zinc finger protein 252 | 0.0001897 | 2.4001 |
| 1566511_at | FBXO9 | F-box protein 9 | 1.77E-06 | 2.39842 |
| 240058_at | LOC100129597 | Hypothetical protein LOC100129597 | 0.0004526 | 2.3984 |
| 1560128_x_at | LOC441108 | Hypothetical gene supported by AK128882 | 0.0010211 | 2.39786 |
| 239593_at | TMEM213 | transmembrane protein 213 | 0.0010654 | 2.39781 |
| 208247_at | C3orf51 | chromosome 3 open reading frame 51 | 0.0002596 | 2.39722 |
| 1558423_at | LOC349114 | hypothetical LOC349114 | 0.000905 | 2.39496 |
| 212027_at | RBM25 | RNA binding motif protein 25 | 0.0009437 | 2.3938 |
| 207734_at | LAX1 | lymphocyte transmembrane adaptor 1 | 0.0005493 | 2.39336 |
| 230435_at | LOC375190 | hypothetical protein LOC375190 | 0.0004173 | 2.39196 |
| 234974_at | GALM | galactose mutarotase (aldose 1-epimerase) | 0.0002177 | 2.38963 |
| 213307_at | SHANK2 | SH3 and multiple ankyrin repeat domains 2 | 0.0013058 | 2.38817 |
| 207317_s_at | CASQ2 | calsequestrin 2 (cardiac muscle) | 9.03E-05 | 2.38705 |
| 1564485_at | LOC100131551 | hypothetical LOC100131551 | 5.63E-06 | 2.38516 |
| 233321_x_at | LOC90834 | hypothetical protein BC001742 | 1.56E-05 | 2.3828 |
| 214354_x_at | SFTPB | surfactant protein B | 0.000135 | 2.37895 |
| 220164_s_at | FBXO40 | F-box protein 40 | 0.0004923 | 2.37826 |
| 213553_x_at | APOC1 | apolipoprotein C-I | 0.0004793 | 2.3777 |
| 204337_at | RGS4 | regulator of G-protein signaling 4 | 0.001468 | 2.37574 |
| 214088_s_at | FUT3 | fucosyltransferase 3 (galactoside 3(4)-L-fucosyltransferase, Lewis blood group) | 7.49E-05 | 2.3751 |
| 222765_x_at | ESF1 | ESF1, nucleolar pre-rRNA processing protein, homolog (S. cerevisiae) | 0.0018322 | 2.37453 |
| 219305_x_at | FBXO2 | F-box protein 2 | 1.76E-07 | 2.37291 |
| 1553461_at | FAM9B | family with sequence similarity 9, member B | 0.0002181 | 2.36954 |
| 1558698_at | ZNF264 | zinc finger protein 264 | 0.0005331 | 2.36707 |
| 233813_at | PPP1R16B | protein phosphatase 1, regulatory (inhibitor) subunit 16B | 0.00013 | 2.36701 |
| 233693_at | C1orf201 | chromosome 1 open reading frame 201 | 0.0004725 | 2.36637 |
| 224012_at | ANKRD20A1 /// ANKRD20A2 /// ANKRD20A3 /// ANKRD20A4 | ankyrin repeat domain 20 family, member A1 /// ankyrin repeat domain 20 family, | 0.0011192 | 2.36531 |
| 209847_at | CDH17 | cadherin 17, LI cadherin (liver-intestine) | 4.38E-05 | 2.36426 |
| 238447_at | RBMS3 | RNA binding motif, single stranded interacting protein | 0.0005975 | 2.36403 |
| 225987_at | STEAP4 | STEAP family member 4 | 0.0008577 | 2.36284 |
| 204621_s_at | NR4A2 | nuclear receptor subfamily 4, group A, member 2 | 0.0001338 | 2.36213 |
| 242036_x_at | ATP2B3 | ATPase, Ca++ transporting, plasma membrane 3 | 0.00143 | 2.36209 |
| 210595_at | ZNF235 | zinc finger protein 235 | 0.0003141 | 2.3618 |
| 1559471_s_at | D21S2088E | D21S2088E | 0.0003986 | 2.36135 |
| 1563862_at | TCEANC | transcription elongation factor A (SII) N-terminal and central domain containing | 0.0007536 | 2.35998 |
| 1552770_s_at | ZNF563 | zinc finger protein 563 | 0.000118 | 2.35987 |
| 1563906_at | SOBP | sine oculis binding protein homolog (Drosophila) | 0.0002464 | 2.35916 |
| 224284_x_at | FKSG49 | FKSG49 | 2.53E-05 | 2.35864 |
| 204492_at | ARHGAP11A | Rho GTPase activating protein 11A | 6.53E-05 | 2.35788 |
| 240363_at | ANK1 | ankyrin 1, erythrocytic | 7.44E-05 | 2.35764 |
| 206318_at | SPINLW1 | serine peptidase inhibitor-like, with Kunitz and WAP domains 1 (eppin) | 0.0001426 | 2.35612 |
| 202331_at | BCKDHA | branched chain keto acid dehydrogenase E1, alpha polypeptide | 8.72E-06 | 2.35575 |
| 224372_at | DCAF6 /// UNC5B | DDB1 and CUL4 associated factor 6 /// unc-5 homolog B (C. elegans) | 1.03E-06 | 2.35548 |
| 1565484_x_at | EGFR | epidermal growth factor receptor (erythroblastic leukemia viral (v-erb-b) oncoge | 0.0003205 | 2.35482 |
| 210195_s_at | PSG1 | pregnancy specific beta-1-glycoprotein 1 | 0.0004652 | 2.35191 |
| 211690_at | RPS6 | ribosomal protein S6 | 0.0001001 | 2.3507 |
| 204836_at | GLDC | glycine dehydrogenase (decarboxylating) | 0.0003668 | 2.34996 |
| 205998_x_at | CYP3A4 | cytochrome P450, family 3, subfamily A, polypeptide 4 | 8.02E-05 | 2.34766 |
| 238602_at | DIS3L2 | DIS3 mitotic control homolog (S. cerevisiae)-like 2 | 3.63E-05 | 2.34267 |
| 207936_x_at | RFPL3 | ret finger protein-like 3 | 0.000792 | 2.34011 |
| 220575_at | FAM106A | family with sequence similarity 106, member A | 0.0012925 | 2.33993 |
| 229069_at | SARNP | SAP domain containing ribonucleoprotein | 0.0002415 | 2.33887 |
| 1552497_a_at | SLAMF6 | SLAM family member 6 | 6.39E-06 | 2.33839 |
| 207598_x_at | XRCC2 | X-ray repair complementing defective repair in Chinese hamster cells 2 | 0.001164 | 2.33832 |
| 207575_at | GOLGA6 | golgi autoantigen, golgin subfamily a, 6 | 6.65E-06 | 2.33724 |
| 203398_s_at | GALNT3 | UDP-N-acetyl-alpha-D-galactosamine:polypeptide N-acetylgalactosaminyltransferase | 0.0002437 | 2.33524 |
| 220456_at | SPTLC3 | serine palmitoyltransferase, long chain base subunit 3 | 1.08E-05 | 2.3327 |
| 233866_at | KLHL5 | kelch-like 5 (Drosophila) | 5.39E-05 | 2.33128 |
| 214329_x_at | TNFSF10 | tumor necrosis factor (ligand) superfamily, member 10 | 0.0005646 | 2.33029 |
| 244565_at | HMX2 | H6 family homeobox 2 | 0.0001478 | 2.32933 |
| 212032_s_at | PTOV1 | prostate tumor overexpressed 1 | 9.04E-05 | 2.32519 |
| 208212_s_at | ALK | anaplastic lymphoma receptor tyrosine kinase | 5.84E-05 | 2.32383 |
| 1556062_at | RPP30 | ribonuclease P/MRP 30kDa subunit | 0.0013812 | 2.32212 |
| 232034_at | LOC203274 | Hypothetical protein LOC203274 | 3.17E-05 | 2.31807 |
| 207436_x_at | KIAA0894 | KIAA0894 protein | 0.0001533 | 2.31685 |
| 202157_s_at | CUGBP2 | CUG triplet repeat, RNA binding protein 2 | 0.0001962 | 2.31616 |
| 221588_x_at | ALDH6A1 | aldehyde dehydrogenase 6 family, member A1 | 0.0001523 | 2.31507 |
| 203561_at | FCGR2A | Fc fragment of IgG, low affinity IIa, receptor (CD32) | 1.62E-05 | 2.31426 |
| 213951_s_at | PSMC3IP | PSMC3 interacting protein | 0.0006747 | 2.3135 |
| 201072_s_at | SMARCC1 | SWI/SNF related, matrix associated, actin dependent regulator of chromatin, subf | 0.0003173 | 2.31203 |
| 224099_at | KCNH7 | potassium voltage-gated channel, subfamily H (eag-related), member 7 | 0.0006104 | 2.31037 |
| 1555786_s_at | C14orf34 | chromosome 14 open reading frame 34 | 4.87E-06 | 2.30735 |
| 205011_at | VWA5A | von Willebrand factor A domain containing 5A | 0.0004851 | 2.30658 |
| 218678_at | NES | nestin | 0.0001265 | 2.30192 |
| 218931_at | RAB17 | RAB17, member RAS oncogene family | 5.20E-05 | 2.30153 |
| 1559790_at | LOC646241 | hypothetical protein LOC646241 | 0.0016243 | 2.30033 |
| 232455_x_at | LOC340085 | hypothetical protein LOC340085 | 7.83E-05 | 2.29629 |
| 1555653_at | HNRNPA3 | heterogeneous nuclear ribonucleoprotein A3 | 0.0003753 | 2.29546 |
| 207577_at | HTR4 | 5-hydroxytryptamine (serotonin) receptor 4 | 4.58E-05 | 2.29321 |
| 219392_x_at | PRR11 | proline rich 11 | 4.63E-05 | 2.29234 |
| 232689_at | LOC284561 | hypothetical protein LOC284561 | 5.75E-05 | 2.29147 |
| 233193_x_at | INTS4 | integrator complex subunit 4 | 2.06E-05 | 2.28996 |
| 202028_s_at | RPL38 | ribosomal protein L38 | 1.12E-05 | 2.28948 |
| 239132_at | NOS1 | Nitric oxide synthase 1 (neuronal) | 0.0008865 | 2.2885 |
| 205500_at | C5 | complement component 5 | 2.57E-05 | 2.28688 |
| 231031_at | KGFLP2 | keratinocyte growth factor-like protein 2 | 0.0002136 | 2.28664 |
| 214235_at | CYP3A5 | cytochrome P450, family 3, subfamily A, polypeptide 5 | 0.0009379 | 2.28263 |
| 210421_s_at | SLC24A1 | solute carrier family 24 (sodium/potassium/calcium exchanger), member 1 | 0.0009506 | 2.28244 |
| 206344_at | PON1 | paraoxonase 1 | 0.0014839 | 2.27933 |
| 212036_s_at | PNN | pinin, desmosome associated protein | 8.95E-05 | 2.27809 |
| 230699_at | PGLS | 6-phosphogluconolactonase | 0.0007326 | 2.27594 |
| 218145_at | TRIB3 | tribbles homolog 3 (Drosophila) | 0.0006966 | 2.26678 |
| 241596_at | NUDT10 | nudix (nucleoside diphosphate linked moiety X)-type motif 10 | 0.0004868 | 2.26342 |
| 211494_s_at | SLC4A4 | solute carrier family 4, sodium bicarbonate cotransporter, member 4 | 0.0010534 | 2.26271 |
| 220007_at | METTL8 | methyltransferase like 8 | 0.000885 | 2.26249 |
| 238868_at | UACA | uveal autoantigen with coiled-coil domains and ankyrin repeats | 2.20E-05 | 2.26229 |
| 1559716_at | INO80C | INO80 complex subunit C | 0.0001244 | 2.26015 |
| 212419_at | ZCCHC24 | zinc finger, CCHC domain containing 24 | 2.08E-06 | 2.25982 |
| 1554631_at | ATM | ataxia telangiectasia mutated | 0.0012713 | 2.25656 |
| 1553706_at | HTRA4 | HtrA serine peptidase 4 | 0.0001595 | 2.25631 |
| 213836_s_at | WIPI1 | WD repeat domain, phosphoinositide interacting 1 | 0.0002643 | 2.25316 |
| 1554517_x_at | LOC203274 | Hypothetical protein LOC203274 | 0.0017861 | 2.25131 |
| 1555181_a_at | ST3GAL3 | ST3 beta-galactoside alpha-2,3-sialyltransferase 3 | 0.0005959 | 2.25053 |
| 1557474_at | LOC284578 | hypothetical LOC284578 | 0.0016276 | 2.24855 |
| 222639_s_at | PLBD1 | phospholipase B domain containing 1 | 2.47E-05 | 2.2483 |
| 216965_x_at | SPG20 | spastic paraplegia 20 (Troyer syndrome) | 0.0003693 | 2.24715 |
| 232991_at | ARL17 | ADP-ribosylation factor-like 17 | 0.0008619 | 2.246 |
| 211775_x_at | MGC13053 | hypothetical MGC13053 | 0.0012075 | 2.24549 |
| 236981_at | C17orf99 | chromosome 17 open reading frame 99 | 0.0010999 | 2.24521 |
| 216428_x_at | KIR3DX1 | killer cell immunoglobulin-like receptor, three domains, X1 | 0.001505 | 2.24464 |
| 1565759_at | RPL13 | Ribosomal protein L13 | 0.0015277 | 2.24313 |
| 225821_s_at | BOD1L | biorientation of chromosomes in cell division 1-like | 2.18E-05 | 2.24287 |
| 232021_at | GLT8D3 | glycosyltransferase 8 domain containing 3 | 0.0014156 | 2.23575 |
| 1568999_at | PSG4 | pregnancy specific beta-1-glycoprotein 4 | 0.0009921 | 2.23526 |
| 215761_at | DMXL2 | Dmx-like 2 | 0.0001174 | 2.23496 |
| 243792_x_at | PTPN13 | Protein tyrosine phosphatase, non-receptor type 13 (APO-1/CD95 (Fas)-associated | 0.0009763 | 2.23381 |
| 215271_at | TNN | tenascin N | 0.0001804 | 2.23358 |
| 235650_at | FLJ23834 | hypothetical protein FLJ23834 | 2.95E-05 | 2.23286 |
| 1553155_x_at | ATP6V0D2 | ATPase, H+ transporting, lysosomal 38kDa, V0 subunit d2 | 0.0016488 | 2.23188 |
| 238008_at | PRR18 | proline rich 18 | 0.0005497 | 2.22999 |
| 215657_at | SLC26A3 | Solute carrier family 26, member 3 | 0.0018291 | 2.22682 |
| 225398_at | RPUSD4 | RNA pseudouridylate synthase domain containing 4 | 4.52E-05 | 2.22616 |
| 224079_at | IL17C | interleukin 17C | 5.62E-05 | 2.22538 |
| 228310_at | ENAH | enabled homolog (Drosophila) | 1.73E-05 | 2.22445 |
| 1567023_at | OR5AK4P | olfactory receptor, family 5, subfamily AK, member 4 pseudogene | 8.68E-06 | 2.22115 |
| 224373_s_at | DCAF6 /// HNRNPM /// UNC5B | DDB1 and CUL4 associated factor 6 /// heterogeneous nuclear ribonucleoprotein M | 8.46E-05 | 2.22107 |
| 222073_at | COL4A3 | collagen, type IV, alpha 3 (Goodpasture antigen) | 0.0002041 | 2.22046 |
| 228285_at | TDRD9 | tudor domain containing 9 | 0.0001964 | 2.21891 |
| 212031_at | RBM25 | RNA binding motif protein 25 | 1.24E-05 | 2.21866 |
| 1552863_a_at | CACNG6 | calcium channel, voltage-dependent, gamma subunit 6 | 0.0009237 | 2.21864 |
| 1570111_at | C14orf48 | chromosome 14 open reading frame 48 | 4.87E-05 | 2.21833 |
| 1565765_x_at | C13orf33 | Chromosome 13 open reading frame 33 | 0.0014704 | 2.21815 |
| 1553919_at | C9orf62 | chromosome 9 open reading frame 62 | 5.36E-05 | 2.21765 |
| 244716_x_at | TMIGD2 | transmembrane and immunoglobulin domain containing 2 | 2.05E-05 | 2.21623 |
| 218757_s_at | UPF3B | UPF3 regulator of nonsense transcripts homolog B (yeast) | 0.0002923 | 2.21515 |
| 1554985_at | ZNF396 | zinc finger protein 396 | 0.000686 | 2.21479 |
| 1569934_at | PREX2 | Phosphatidylinositol-3,4,5-trisphosphate-dependent Rac exchange factor 2 | 0.0012839 | 2.21376 |
| 204072_s_at | FRY | furry homolog (Drosophila) | 0.0003518 | 2.21103 |
| 203976_s_at | CHAF1A | chromatin assembly factor 1, subunit A (p150) | 0.0010674 | 2.2107 |
| 222683_at | RNF20 | ring finger protein 20 | 8.29E-06 | 2.21015 |
| 210739_x_at | SLC4A4 | solute carrier family 4, sodium bicarbonate cotransporter, member 4 | 0.0013792 | 2.20983 |
| 209747_at | TGFB3 | transforming growth factor, beta 3 | 9.60E-05 | 2.20661 |
| 224419_x_at | PMCHL1 | pro-melanin-concentrating hormone-like 1 | 0.0015154 | 2.20531 |
| 214389_at | SLC5A12 | solute carrier family 5 (sodium/glucose cotransporter), member 12 | 0.0001391 | 2.20513 |
| 1570025_at | TACC2 | transforming, acidic coiled-coil containing protein 2 | 0.0010608 | 2.20153 |
| 1557450_s_at | WHAMML2 | WAS protein homolog associated with actin, golgi membranes and microtubules-like | 0.0006678 | 2.19657 |
| 240607_at | MIAT | Myocardial infarction associated transcript (non-protein coding) | 0.0002045 | 2.19639 |
| 1563505_at | DUSP16 | Dual specificity phosphatase 16 | 0.0003758 | 2.19633 |
| 242800_at | NHS | Nance-Horan syndrome (congenital cataracts and dental anomalies) | 0.0006627 | 2.19428 |
| 1557345_at | LOC283516 | hypothetical protein LOC283516 | 0.0003817 | 2.19197 |
| 222796_at | PTCD1 | pentatricopeptide repeat domain 1 | 0.0008494 | 2.19189 |
| 1555305_at | FOXJ2 | forkhead box J2 | 0.0006344 | 2.1856 |
| 229218_at | COL1A2 | collagen, type I, alpha 2 | 0.0005432 | 2.18235 |
| 1560001_at | LOC100131581 | hypothetical LOC100131581 | 3.35E-05 | 2.17904 |
| 1559687_at | TMEM221 | transmembrane protein 221 | 0.0015975 | 2.17786 |
| 222194_at | FAM66D | family with sequence similarity 66, member D | 0.0012553 | 2.17684 |
| 211260_at | BMP7 | bone morphogenetic protein 7 | 0.0002878 | 2.17488 |
| 202022_at | ALDOC | aldolase C, fructose-bisphosphate | 6.91E-05 | 2.17487 |
| 201073_s_at | SMARCC1 | SWI/SNF related, matrix associated, actin dependent regulator of chromatin, subf | 0.0012633 | 2.17421 |
| 206819_at | POM121L9P | POM121 membrane glycoprotein-like 9 (rat) pseudogene | 4.85E-05 | 2.17408 |
| 223751_x_at | TLR10 | toll-like receptor 10 | 7.66E-05 | 2.17171 |
| 1568891_x_at | FANCD2 | Fanconi anemia, complementation group D2 | 0.0001698 | 2.17164 |
| 208990_s_at | HNRNPH3 | heterogeneous nuclear ribonucleoprotein H3 (2H9) | 0.000308 | 2.17109 |
| 222139_at | KIAA1466 | KIAA1466 gene | 0.000576 | 2.17096 |
| 1553016_at | GPR113 | G protein-coupled receptor 113 | 4.16E-05 | 2.17071 |
| 235154_at | TAF3 | TAF3 RNA polymerase II, TATA box binding protein (TBP)-associated factor, 140kDa | 0.0004664 | 2.17011 |
| 241399_at | FAM19A2 | family with sequence similarity 19 (chemokine (C-C motif)-like), member A2 | 0.0009998 | 2.16931 |
| 1553387_at | ATM | ataxia telangiectasia mutated | 0.000115 | 2.16759 |
| 219157_at | KLHL2 | kelch-like 2, Mayven (Drosophila) | 0.0006129 | 2.1669 |
| 223619_x_at | PECR | peroxisomal trans-2-enoyl-CoA reductase | 0.0001297 | 2.16669 |
| 232968_at | FANK1 | fibronectin type III and ankyrin repeat domains 1 | 0.0002174 | 2.16553 |
| 212037_at | PNN | pinin, desmosome associated protein | 4.03E-05 | 2.16331 |
| 203455_s_at | SAT1 | spermidine/spermine N1-acetyltransferase 1 | 0.0008764 | 2.16293 |
| 1552809_at | RFX4 | regulatory factor X, 4 (influences HLA class II expression) | 0.0003218 | 2.16288 |
| 1562650_at | FRYL | FRY-like | 0.0003129 | 2.16139 |
| 1558791_at | LOC286467 | hypothetical LOC286467 | 5.33E-05 | 2.16005 |
| 1560558_at | C9orf80 | chromosome 9 open reading frame 80 | 6.65E-05 | 2.16 |
| 202565_s_at | SVIL | supervillin | 1.84E-05 | 2.15944 |
| 37028_at | PPP1R15A | protein phosphatase 1, regulatory (inhibitor) subunit 15A | 9.59E-05 | 2.15917 |
| 237335_at | ZP1 | zona pellucida glycoprotein 1 (sperm receptor) | 0.0007369 | 2.15807 |
| 215463_at | OR7E24 | olfactory receptor, family 7, subfamily E, member 24 | 0.0001295 | 2.15759 |
| 213394_at | MAPKBP1 | mitogen-activated protein kinase binding protein 1 | 9.40E-05 | 2.15604 |
| 1553394_a_at | TFAP2B | transcription factor AP-2 beta (activating enhancer binding protein 2 beta) | 2.22E-05 | 2.15428 |
| 1569615_at | LOC100293264 | hypothetical protein LOC100293264 | 0.0007178 | 2.15417 |
| 242064_at | SDK2 | sidekick homolog 2 (chicken) | 0.0001211 | 2.15211 |
| 202074_s_at | OPTN | optineurin | 0.0006025 | 2.15186 |
| 226544_x_at | MUTED | muted homolog (mouse) | 4.49E-06 | 2.15153 |
| 1553020_at | SMCR5 | Smith-Magenis syndrome chromosome region, candidate 5 (non-protein coding) | 8.50E-06 | 2.15117 |
| 1569956_at | MYLK | myosin light chain kinase | 0.0007417 | 2.15062 |
| 1562697_at | LOC339988 | hypothetical protein LOC339988 | 0.0009473 | 2.15053 |
| 33850_at | MAP4 | microtubule-associated protein 4 | 0.0003243 | 2.15004 |
| 201746_at | TP53 | tumor protein p53 | 0.00017 | 2.14949 |
| 204020_at | PURA | purine-rich element binding protein A | 2.54E-05 | 2.14863 |
| 209851_at | ZC3H13 | zinc finger CCCH-type containing 13 | 0.0005943 | 2.14749 |
| 1556060_a_at | ZNF451 | zinc finger protein 451 | 0.0004544 | 2.14623 |
| 211780_x_at | DCTN1 | dynactin 1 (p150, glued homolog, Drosophila) | 0.0001098 | 2.14546 |
| 1564209_at | LOC282980 | hypothetical protein LOC282980 | 0.0001991 | 2.14513 |
| 232298_at | hCG_1806964 | hypothetical LOC401093 | 0.000495 | 2.1448 |
| 203973_s_at | CEBPD | CCAAT/enhancer binding protein (C/EBP), delta | 0.0001259 | 2.14429 |
| 200695_at | PPP2R1A | protein phosphatase 2 (formerly 2A), regulatory subunit A, alpha isoform | 0.0014598 | 2.14097 |
| 221152_at | COL8A1 | collagen, type VIII, alpha 1 | 0.0006008 | 2.13952 |
| 222521_x_at | NDUFC2 | NADH dehydrogenase (ubiquinone) 1, subcomplex unknown, 2, 14.5kDa | 3.64E-05 | 2.13819 |
| 1568857_a_at | NBR1 | Neighbor of BRCA1 gene 1 | 0.000152 | 2.13749 |
| 238504_at | C6orf57 | chromosome 6 open reading frame 57 | 4.44E-05 | 2.13685 |
| 211316_x_at | CFLAR | CASP8 and FADD-like apoptosis regulator | 0.0002673 | 2.13653 |
| 215513_at | HYMAI | hydatidiform mole associated and imprinted (non-protein coding) | 0.0002227 | 2.13615 |
| 1553373_at | WDR64 | WD repeat domain 64 | 7.39E-06 | 2.13614 |
| 1560204_at | NT5DC4 | 5'-nucleotidase domain containing 4 | 0.0013002 | 2.13468 |
| 217759_at | TRIM44 | tripartite motif-containing 44 | 5.59E-05 | 2.13433 |
| 237088_at | C7orf4 | chromosome 7 open reading frame 4 | 0.0009125 | 2.13363 |
| 239173_at | INADL | InaD-like (Drosophila) | 0.0001212 | 2.13228 |
| 231704_at | ZNF498 | zinc finger protein 498 | 9.14E-05 | 2.13167 |
| 212423_at | ZCCHC24 | zinc finger, CCHC domain containing 24 | 7.73E-06 | 2.13015 |
| 1553797_a_at | LOC150622 | hypothetical LOC150622 | 0.00081 | 2.12957 |
| 1568889_at | FANCD2 | Fanconi anemia, complementation group D2 | 0.0010732 | 2.12917 |
| 208824_x_at | PCTK1 | PCTAIRE protein kinase 1 | 0.0006936 | 2.12727 |
| 228411_at | PARD3B | par-3 partitioning defective 3 homolog B (C. elegans) | 2.34E-06 | 2.12577 |
| 241642_x_at | TLK1 | tousled-like kinase 1 | 3.93E-05 | 2.1255 |
| 214372_x_at | ERN2 | endoplasmic reticulum to nucleus signaling 2 | 3.50E-05 | 2.12517 |
| 1568604_a_at | CADPS | Ca++-dependent secretion activator | 0.0010983 | 2.12453 |
| 205193_at | MAFF | v-maf musculoaponeurotic fibrosarcoma oncogene homolog F (avian) | 1.97E-05 | 2.12407 |
| 223110_at | KIAA1429 | KIAA1429 | 0.0001946 | 2.12339 |
| 233164_x_at | RHBDD1 | rhomboid domain containing 1 | 0.0009238 | 2.11975 |
| 209383_at | DDIT3 /// NR1H3 | DNA-damage-inducible transcript 3 /// nuclear receptor subfamily 1, group H, mem | 0.0002763 | 2.11913 |
| 1561093_at | SLC22A25 | solute carrier family 22, member 25 | 8.91E-06 | 2.11889 |
| 228617_at | XAF1 | XIAP associated factor 1 | 0.0010658 | 2.11501 |
| 236863_at | C17orf67 | chromosome 17 open reading frame 67 | 2.22E-06 | 2.1142 |
| 1555726_at | GAFA3 | FGF-2 activity-associated protein 3 | 0.0001204 | 2.11215 |
| 1562142_at | SKIV2L2 | Superkiller viralicidic activity 2-like 2 (S. cerevisiae) | 1.27E-05 | 2.10929 |
| 1562321_at | PDK4 | pyruvate dehydrogenase kinase, isozyme 4 | 0.0018027 | 2.10489 |
| 1569940_at | SLC6A16 | Solute carrier family 6, member 16 | 0.0007845 | 2.10468 |
| 207221_at | F2RL3 | coagulation factor II (thrombin) receptor-like 3 | 8.24E-05 | 2.1043 |
| 222290_at | OR2A20P /// OR2A5 /// OR2A9P | olfactory receptor, family 2, subfamily A, member 20 pseudogene /// olfactory re | 0.0001731 | 2.10273 |
| 220577_at | GVIN1 | GTPase, very large interferon inducible 1 | 0.0010051 | 2.10174 |
| 230539_at | FAM182A | family with sequence similarity 182, member A | 0.0002025 | 2.10004 |
| 234085_at | C13orf38 | chromosome 13 open reading frame 38 | 1.76E-05 | 2.09979 |
| 206808_at | HNRNPA3P1 | heterogeneous nuclear ribonucleoprotein A3 pseudogene 1 | 0.0006301 | 2.09912 |
| 232643_at | POLR2F | Polymerase (RNA) II (DNA directed) polypeptide F | 0.0001008 | 2.09828 |
| 213543_at | SGCD | sarcoglycan, delta (35kDa dystrophin-associated glycoprotein) | 0.0002057 | 2.09792 |
| 203827_at | WIPI1 | WD repeat domain, phosphoinositide interacting 1 | 0.0008456 | 2.0964 |
| 206754_s_at | CYP2B6 /// CYP2B7P1 | cytochrome P450, family 2, subfamily B, polypeptide 6 /// cytochrome P450, famil | 0.0002787 | 2.0962 |
| 231514_at | C1orf94 | chromosome 1 open reading frame 94 | 0.0006664 | 2.09505 |
| 218040_at | PRPF38B | PRP38 pre-mRNA processing factor 38 (yeast) domain containing B | 7.26E-06 | 2.09457 |
| 210254_at | MS4A3 | membrane-spanning 4-domains, subfamily A, member 3 (hematopoietic cell-specific) | 1.01E-05 | 2.09298 |
| 221176_x_at | WBSCR23 | Williams-Beuren syndrome chromosome region 23 | 0.0004534 | 2.09224 |
| 235546_at | SPINT1 | Serine peptidase inhibitor, Kunitz type 1 | 0.0005393 | 2.0911 |
| 210592_s_at | SAT1 | spermidine/spermine N1-acetyltransferase 1 | 1.68E-05 | 2.09058 |
| 232720_at | LINGO2 | leucine rich repeat and Ig domain containing 2 | 8.32E-06 | 2.09027 |
| 1557374_at | ABCC9 | ATP-binding cassette, sub-family C (CFTR/MRP), member 9 | 0.0013649 | 2.08537 |
| 231567_s_at | CCDC62 | coiled-coil domain containing 62 | 0.0006515 | 2.08339 |
| 207523_at | C6orf10 | chromosome 6 open reading frame 10 | 0.0004119 | 2.08284 |
| 1559144_x_at | LOC100130581 | hypothetical LOC100130581 | 5.33E-05 | 2.08065 |
| 217342_x_at | FLJ11292 | hypothetical protein FLJ11292 | 0.0010564 | 2.0801 |
| 239398_at | KLHL31 | kelch-like 31 (Drosophila) | 0.0002075 | 2.07989 |
| 208404_x_at | KCNJ5 | potassium inwardly-rectifying channel, subfamily J, member 5 | 0.0002414 | 2.07906 |
| 220102_at | FOXL2 | forkhead box L2 | 0.0002707 | 2.07784 |
| 201310_s_at | C5orf13 | chromosome 5 open reading frame 13 | 7.29E-05 | 2.07621 |
| 1557891_s_at | LOC729178 | hypothetical protein LOC729178 | 0.0014592 | 2.07475 |
| 213350_at | RPS11 | Ribosomal protein S11 | 0.0007581 | 2.07386 |
| 202371_at | TCEAL4 | transcription elongation factor A (SII)-like 4 | 0.0017423 | 2.07336 |
| 202489_s_at | FXYD3 | FXYD domain containing ion transport regulator 3 | 0.0001844 | 2.07302 |
| 1560631_at | CALCOCO2 | calcium binding and coiled-coil domain 2 | 6.85E-05 | 2.07225 |
| 1554318_at | LOC541473 | FK506 binding protein 6, 36kDa pseudogene | 0.0012469 | 2.07152 |
| 208383_s_at | PCK1 | phosphoenolpyruvate carboxykinase 1 (soluble) | 0.0001173 | 2.07107 |
| 216763_at | KANK1 | KN motif and ankyrin repeat domains 1 | 0.0001525 | 2.071 |
| 202132_at | WWTR1 | WW domain containing transcription regulator 1 | 2.32E-05 | 2.07053 |
| 205988_at | CD84 | CD84 molecule | 0.0018765 | 2.06979 |
| 232722_at | RNASET2 | ribonuclease T2 | 2.20E-05 | 2.06973 |
| 222421_at | UBE2H | ubiquitin-conjugating enzyme E2H (UBC8 homolog, yeast) | 7.48E-05 | 2.06818 |
| 1570200_at | HELB | helicase (DNA) B | 0.0003029 | 2.06707 |
| 244296_at | hCG_1776047 | HCG1776047 | 0.0013862 | 2.06557 |
| 228509_at | SPHKAP | SPHK1 interactor, AKAP domain containing | 0.0002185 | 2.06556 |
| 1554744_at | CARD16 | caspase recruitment domain family, member 16 | 0.0015394 | 2.06423 |
| 220791_x_at | SCN11A | sodium channel, voltage-gated, type XI, alpha subunit | 1.92E-06 | 2.06411 |
| 223824_at | RNLS | renalase, FAD-dependent amine oxidase | 0.0001576 | 2.06349 |
| 215787_at | ACTA2 | Actin, alpha 2, smooth muscle, aorta | 4.44E-05 | 2.06326 |
| 210538_s_at | BIRC3 | baculoviral IAP repeat-containing 3 | 0.0008281 | 2.06289 |
| 202566_s_at | SVIL | supervillin | 1.35E-05 | 2.0628 |
| 202364_at | MXI1 | MAX interactor 1 | 7.84E-07 | 2.06252 |
| 201416_at | SOX4 | SRY (sex determining region Y)-box 4 | 7.04E-05 | 2.06197 |
| 228884_at | LRRC27 | leucine rich repeat containing 27 | 0.0008673 | 2.05978 |
| 217484_at | CR1 | complement component (3b/4b) receptor 1 (Knops blood group) | 0.000187 | 2.05949 |
| 222215_at | SLC38A7 | solute carrier family 38, member 7 | 0.0001677 | 2.05934 |
| 1554367_at | CALHM1 | calcium homeostasis modulator 1 | 2.99E-05 | 2.05763 |
| 1557080_s_at | ITGBL1 | integrin, beta-like 1 (with EGF-like repeat domains) | 1.39E-06 | 2.05737 |
| 207623_at | ABCF2 | ATP-binding cassette, sub-family F (GCN20), member 2 | 0.0001537 | 2.0564 |
| 214650_x_at | MOG | myelin oligodendrocyte glycoprotein | 0.001089 | 2.0549 |
| 1557827_at | C10orf103 | chromosome 10 open reading frame 103 | 0.0006533 | 2.05483 |
| 228462_at | IRX2 | iroquois homeobox 2 | 0.0008061 | 2.05302 |
| 208386_x_at | DMC1 | DMC1 dosage suppressor of mck1 homolog, meiosis-specific homologous recombinatio | 0.0002544 | 2.05157 |
| 220866_at | ADAMTS6 | ADAM metallopeptidase with thrombospondin type 1 motif, 6 | 7.51E-05 | 2.04914 |
| 1559989_at | TPM4 | Tropomyosin 4 | 3.39E-05 | 2.047 |
| 212776_s_at | OBSL1 | obscurin-like 1 | 0.0008014 | 2.04592 |
| 1561846_s_at | hCG_2011852 | hypothetical protein LOC643677 | 0.0001357 | 2.04502 |
| 215717_s_at | FBN2 | fibrillin 2 | 0.0011515 | 2.04484 |
| 215590_x_at | LOC100128640 | Hypothetical protein LOC100128640 | 4.26E-05 | 2.04287 |
| 1561557_at | LIPA | Lipase A, lysosomal acid, cholesterol esterase | 7.32E-05 | 2.04207 |
| 207119_at | PRKG1 | protein kinase, cGMP-dependent, type I | 0.0015288 | 2.04175 |
| 208723_at | USP11 | ubiquitin specific peptidase 11 | 5.20E-05 | 2.04167 |
| 236900_x_at | LOC126661 | Hypothetical protein LOC126661 | 0.0017153 | 2.04142 |
| 232485_at | RUNDC2A | RUN domain containing 2A | 3.03E-06 | 2.04135 |
| 1552895_a_at | C21orf99 | cancer-testis SP-1 | 9.75E-05 | 2.04022 |
| 209638_x_at | RGS12 | regulator of G-protein signaling 12 | 1.58E-07 | 2.03995 |
| 235781_at | CACNA1B | calcium channel, voltage-dependent, N type, alpha 1B subunit | 0.0004282 | 2.03838 |
| 227909_at | NCRNA00086 /// NCRNA00087 | non-protein coding RNA 86 /// non-protein coding RNA 87 | 0.0006917 | 2.03828 |
| 218625_at | NRN1 | neuritin 1 | 0.0008677 | 2.03782 |
| 227808_at | DNAJC15 | DnaJ (Hsp40) homolog, subfamily C, member 15 | 0.0004195 | 2.03718 |
| 215297_at | hCG_2009921 | hypothetical locus LOC441204 | 1.71E-05 | 2.03547 |
| 1553602_at | MUCL1 | mucin-like 1 | 0.0001662 | 2.03449 |
| 215368_at | NEB | nebulin | 0.0003794 | 2.03411 |
| 232502_at | FLJ34077 /// LOC728558 | weakly similar to zinc finger protein 195 /// hypothetical LOC728558 | 9.55E-05 | 2.03407 |
| 1557605_a_at | LOC401312 | hypothetical LOC401312 | 0.0001143 | 2.03406 |
| 233815_at | NAALAD2 | N-acetylated alpha-linked acidic dipeptidase 2 | 0.0013961 | 2.03373 |
| 1560390_s_at | LOC284440 | hypothetical LOC284440 | 4.07E-05 | 2.03263 |
| 219270_at | CHAC1 | ChaC, cation transport regulator homolog 1 (E. coli) | 0.0008862 | 2.03149 |
| 242119_at | PROX1 | Prospero homeobox 1 | 0.0005852 | 2.03044 |
| 1552414_at | WFDC9 | WAP four-disulfide core domain 9 | 5.76E-05 | 2.03007 |
| 216755_at | OSBPL10 | oxysterol binding protein-like 10 | 0.0003369 | 2.02927 |
| 212854_x_at | NBPF10 | neuroblastoma breakpoint family, member 10 | 2.20E-06 | 2.0281 |
| 234486_at | OR51B2 | olfactory receptor, family 51, subfamily B, member 2 | 0.0002296 | 2.02711 |
| 1568843_at | TTLL13 | tubulin tyrosine ligase-like family, member 13 | 0.0007871 | 2.02677 |
| 227084_at | DTNA | dystrobrevin, alpha | 0.0002041 | 2.0255 |
| 217414_x_at | HBA1 /// HBA2 | hemoglobin, alpha 1 /// hemoglobin, alpha 2 | 6.87E-05 | 2.02272 |
| 224322_at | ARID4B | AT rich interactive domain 4B (RBP1-like) | 0.0016393 | 2.0222 |
| 1558738_at | NOL3 | Nucleolar protein 3 (apoptosis repressor with CARD domain) | 0.0006588 | 2.02162 |
| 1554769_at | ZNF785 | zinc finger protein 785 | 0.0009666 | 2.0196 |
| 1566129_at | LIMS1 | LIM and senescent cell antigen-like domains 1 | 0.0013509 | 2.01758 |
| 1566665_at | hCG_2011852 | hypothetical protein LOC643677 | 0.0002693 | 2.01729 |
| 230102_at | ETV5 | Ets variant 5 | 0.0001263 | 2.01715 |
| 211821_x_at | GYPA | glycophorin A (MNS blood group) | 0.0012223 | 2.01622 |
| 221859_at | SYT13 | synaptotagmin XIII | 0.0006187 | 2.01569 |
| 1563513_at | SYTL4 | synaptotagmin-like 4 | 0.001131 | 2.0147 |
| 1562919_at | FAM45A | Family with sequence similarity 45, member A | 0.000446 | 2.01439 |
| 216440_at | ERC1 | ELKS/RAB6-interacting/CAST family member 1 | 0.0011037 | 2.01357 |
| 1570414_x_at | FLJ13197 | hypothetical FLJ13197 | 1.37E-05 | 2.01213 |
| 1556505_at | LOC100131366 | Hypothetical protein LOC100131366 | 0.0001702 | 2.01208 |
| 1556046_a_at | LOC157627 | hypothetical LOC157627 | 0.0015367 | 2.01174 |
| 212077_at | CALD1 | caldesmon 1 | 1.86E-05 | 2.01168 |
| 1563793_at | LOC100130278 | hypothetical protein LOC100130278 | 0.0001734 | 2.01038 |
| 218858_at | DEPDC6 | DEP domain containing 6 | 3.07E-05 | 2.01035 |
| 1561169_at | LOC727818 | similar to PRP38 pre-mRNA processing factor 38 (yeast) domain containing B | 0.0005358 | 2.01004 |
| 211874_s_at | MYST4 | MYST histone acetyltransferase (monocytic leukemia) 4 | 0.0015048 | 2.00934 |
| 244377_at | SLC1A4 | Solute carrier family 1 (glutamate/neutral amino acid transporter), member 4 | 0.0005706 | 2.00889 |
| 241851_x_at | LOC100130429 | hypothetical LOC100130429 | 0.0003028 | 2.00875 |
| 216748_at | PYHIN1 | pyrin and HIN domain family, member 1 | 0.0008257 | 2.00812 |
| 214945_at | FAM153A /// FAM153B /// FAM153C | family with sequence similarity 153, member A /// family with sequence similarit | 0.0011505 | 2.00807 |
| 203256_at | CDH3 | cadherin 3, type 1, P-cadherin (placental) | 0.0001515 | 2.00796 |
| 229125_at | KANK4 | KN motif and ankyrin repeat domains 4 | 9.17E-05 | 2.00681 |
| 229737_at | LOC100134123 | hypothetical protein LOC100134123 | 0.0002536 | 2.00663 |
| 242272_at | ZNF785 | zinc finger protein 785 | 0.0011808 | 2.00601 |
| 241331_at | SKAP2 | Src kinase associated phosphoprotein 2 | 0.0015032 | 2.00542 |
| 242195_x_at | NUMBL | numb homolog (Drosophila)-like | 4.75E-05 | 2.00401 |
| 226445_s_at | TRIM41 | tripartite motif-containing 41 | 0.0005896 | 2.00316 |
| 231359_at | APOH | Apolipoprotein H (beta-2-glycoprotein I) | 0.0002247 | 2.00269 |
| 241624_at | LOC389834 | ankyrin repeat domain 57 pseudogene | 0.0001572 | 2.00241 |
| 1566860_at | LOC145663 | hypothetical protein LOC145663 | 0.0013715 | 2.00228 |
| 227230_s_at | KIAA1211 | KIAA1211 | 0.0001205 | 2.00153 |
| 208190_s_at | LSR | lipolysis stimulated lipoprotein receptor | 0.0002525 | 2.00144 |
| 230122_at | MLLT10 | myeloid/lymphoid or mixed-lineage leukemia (trithorax homolog, Drosophila); tran | 0.0016885 | -2.00007 |
| 224717_s_at | C19orf42 | chromosome 19 open reading frame 42 | 5.43E-05 | -2.00011 |
| 218111_s_at | CMAS | cytidine monophosphate N-acetylneuraminic acid synthetase | 0.0009227 | -2.00051 |
| 211530_x_at | HLA-G | major histocompatibility complex, class I, G | 0.0002353 | -2.00081 |
| 220295_x_at | DEPDC1 | DEP domain containing 1 | 0.0012279 | -2.0015 |
| 225260_s_at | MRPL32 | mitochondrial ribosomal protein L32 | 5.84E-07 | -2.00155 |
| 222715_s_at | SYNRG | synergin, gamma | 0.0002939 | -2.00164 |
| 230185_at | THAP9 | THAP domain containing 9 | 0.0016563 | -2.0018 |
| 234472_at | GALNT13 | UDP-N-acetyl-alpha-D-galactosamine:polypeptide N-acetylgalactosaminyltransferase | 0.0004011 | -2.0026 |
| 227658_s_at | PLEKHA3 | pleckstrin homology domain containing, family A (phosphoinositide binding specif | 8.12E-06 | -2.00275 |
| 226705_at | FGFR1 | fibroblast growth factor receptor 1 | 0.0003089 | -2.00281 |
| 239208_s_at | C21orf57 | Chromosome 21 open reading frame 57 | 0.0015822 | -2.00299 |
| 231954_at | DKFZP434I0714 | hypothetical protein DKFZP434I0714 | 8.75E-05 | -2.00313 |
| 230285_at | SVIP | small VCP/p97-interacting protein | 0.0002469 | -2.00318 |
| 218871_x_at | CSGALNACT2 | chondroitin sulfate N-acetylgalactosaminyltransferase 2 | 0.0016512 | -2.00322 |
| 233078_at | API5 | apoptosis inhibitor 5 | 0.0001515 | -2.00388 |
| 206373_at | ZIC1 | Zic family member 1 (odd-paired homolog, Drosophila) | 0.0002055 | -2.00432 |
| 205528_s_at | RUNX1T1 | runt-related transcription factor 1; translocated to, 1 (cyclin D-related) | 0.0005377 | -2.00488 |
| 224518_s_at | ZNF559 | zinc finger protein 559 | 9.49E-05 | -2.00492 |
| 218436_at | SIL1 | SIL1 homolog, endoplasmic reticulum chaperone (S. cerevisiae) | 0.0001208 | -2.00555 |
| 211996_s_at | LOC100132247 /// LOC348162 /// LOC613037 /// LOC728888 /// NPIPL3 | similar to Uncharacterized protein KIAA0220 /// hypothetical protein 348162 /// | 1.64E-05 | -2.00686 |
| 244519_at | ASXL1 | additional sex combs like 1 (Drosophila) | 0.0013646 | -2.00732 |
| 211009_s_at | ZNF271 | zinc finger protein 271 | 0.0002822 | -2.00762 |
| 231300_at | C16orf93 | chromosome 16 open reading frame 93 | 0.0007019 | -2.00961 |
| 228482_at | CDRT4 /// FAM18B2 | CMT1A duplicated region transcript 4 /// family with sequence similarity 18, mem | 0.0004138 | -2.0097 |
| 213718_at | RBM4 | RNA binding motif protein 4 | 0.0010034 | -2.01002 |
| 57703_at | SENP5 | SUMO1/sentrin specific peptidase 5 | 0.0002895 | -2.01032 |
| 1555790_a_at | TMEM192 /// ZNF320 | transmembrane protein 192 /// zinc finger protein 320 | 0.0002814 | -2.01039 |
| 239014_at | CCAR1 | Cell division cycle and apoptosis regulator 1 | 0.0005397 | -2.01087 |
| 215710_at | ST3GAL4 | ST3 beta-galactoside alpha-2,3-sialyltransferase 4 | 0.0010638 | -2.01131 |
| 228746_s_at | CDV3 | CDV3 homolog (mouse) | 0.0001099 | -2.01193 |
| 40020_at | CELSR3 | cadherin, EGF LAG seven-pass G-type receptor 3 (flamingo homolog, Drosophila) | 0.0002578 | -2.01234 |
| 1554867_a_at | PRR16 | proline rich 16 | 0.0005834 | -2.01318 |
| 200892_s_at | TRA2B | transformer 2 beta homolog (Drosophila) | 0.0001112 | -2.01321 |
| 221002_s_at | TSPAN14 | tetraspanin 14 | 7.66E-05 | -2.01334 |
| 202898_at | SDC3 | syndecan 3 | 0.0010834 | -2.0136 |
| 212783_at | RBBP6 | retinoblastoma binding protein 6 | 0.0001626 | -2.01372 |
| 205875_s_at | TREX1 | three prime repair exonuclease 1 | 3.13E-05 | -2.01386 |
| 226019_at | OMA1 | OMA1 homolog, zinc metallopeptidase (S. cerevisiae) | 0.000134 | -2.01501 |
| 212715_s_at | MICAL3 | microtubule associated monoxygenase, calponin and LIM domain containing 3 | 0.0004941 | -2.01524 |
| 215855_s_at | TMF1 | TATA element modulatory factor 1 | 0.000122 | -2.0156 |
| 219556_at | C16orf59 | chromosome 16 open reading frame 59 | 0.0008844 | -2.01611 |
| 207621_s_at | PEMT | phosphatidylethanolamine N-methyltransferase | 2.92E-05 | -2.0169 |
| 218013_x_at | DCTN4 | dynactin 4 (p62) | 0.0002575 | -2.01705 |
| 229785_at | KRIT1 | KRIT1, ankyrin repeat containing | 0.0001418 | -2.01753 |
| 228038_at | SOX2 | SRY (sex determining region Y)-box 2 | 0.0007638 | -2.01771 |
| 226856_at | MUSTN1 | musculoskeletal, embryonic nuclear protein 1 | 0.0001593 | -2.01786 |
| 202262_x_at | DDAH2 | dimethylarginine dimethylaminohydrolase 2 | 0.0002387 | -2.01791 |
| 226720_at | PWWP2A | PWWP domain containing 2A | 0.0002275 | -2.01871 |
| 221951_at | TMEM80 | transmembrane protein 80 | 0.0006538 | -2.01879 |
| 234994_at | TMEM200A | transmembrane protein 200A | 1.68E-05 | -2.01928 |
| 213851_at | TMEM110 | transmembrane protein 110 | 6.92E-05 | -2.01947 |
| 209790_s_at | CASP6 | caspase 6, apoptosis-related cysteine peptidase | 0.0010328 | -2.02021 |
| 203497_at | MED1 | mediator complex subunit 1 | 0.0001171 | -2.02035 |
| 227244_s_at | SSU72 | SSU72 RNA polymerase II CTD phosphatase homolog (S. cerevisiae) | 0.0011929 | -2.02037 |
| 215509_s_at | BUB1 | budding uninhibited by benzimidazoles 1 homolog (yeast) | 0.0002136 | -2.02043 |
| 212281_s_at | TMEM97 | transmembrane protein 97 | 2.26E-07 | -2.02137 |
| 220097_s_at | TMEM104 | transmembrane protein 104 | 9.60E-05 | -2.02229 |
| 241912_at | ZNF814 | zinc finger protein 814 | 0.0006714 | -2.02341 |
| 224931_at | SLC41A3 | solute carrier family 41, member 3 | 0.0018754 | -2.0235 |
| 220955_x_at | RAB23 | RAB23, member RAS oncogene family | 0.001876 | -2.02392 |
| 203739_at | ZNF217 | zinc finger protein 217 | 0.0011567 | -2.0242 |
| 204243_at | RLF | rearranged L-myc fusion | 0.0004997 | -2.02486 |
| 214045_at | LIAS | lipoic acid synthetase | 4.95E-06 | -2.0249 |
| 212602_at | WDFY3 | WD repeat and FYVE domain containing 3 | 6.58E-05 | -2.02528 |
| 219182_at | FLJ22167 | hypothetical protein FLJ22167 | 0.0018108 | -2.02557 |
| 235359_at | LRRC33 | leucine rich repeat containing 33 | 0.0010948 | -2.02574 |
| 230556_at | IMMP1L | IMP1 inner mitochondrial membrane peptidase-like (S. cerevisiae) | 0.0008427 | -2.02647 |
| 213788_s_at | NCRNA00094 | non-protein coding RNA 94 | 0.0005285 | -2.02702 |
| 211085_s_at | STK4 | serine/threonine kinase 4 | 0.0016113 | -2.02747 |
| 210706_s_at | RNF24 | ring finger protein 24 | 3.61E-05 | -2.02813 |
| 219494_at | RAD54B | RAD54 homolog B (S. cerevisiae) | 0.0002612 | -2.02889 |
| 226631_at | METTL10 | methyltransferase like 10 | 0.0001212 | -2.0289 |
| 203061_s_at | MDC1 | mediator of DNA-damage checkpoint 1 | 0.0001001 | -2.02933 |
| 229016_s_at | TRERF1 | transcriptional regulating factor 1 | 6.49E-05 | -2.02964 |
| 205511_at | FLJ10038 | hypothetical protein FLJ10038 | 0.0011272 | -2.03095 |
| 226997_at | ADAMTS12 | ADAM metallopeptidase with thrombospondin type 1 motif, 12 | 0.0002642 | -2.03125 |
| 204224_s_at | GCH1 | GTP cyclohydrolase 1 | 0.0017833 | -2.03156 |
| 209699_x_at | AKR1C2 | aldo-keto reductase family 1, member C2 (dihydrodiol dehydrogenase 2; bile acid | 0.0001338 | -2.03321 |
| 204962_s_at | CENPA | centromere protein A | 5.68E-09 | -2.03398 |
| 208707_at | EIF5 | eukaryotic translation initiation factor 5 | 7.20E-05 | -2.03409 |
| 227165_at | SKA3 | spindle and kinetochore associated complex subunit 3 | 0.0012412 | -2.03458 |
| 336_at | TBXA2R | thromboxane A2 receptor | 0.0006215 | -2.03542 |
| 220230_s_at | CYB5R2 | cytochrome b5 reductase 2 | 0.0001547 | -2.03543 |
| 212008_at | UBXN4 | UBX domain protein 4 | 0.0008822 | -2.03584 |
| 221036_s_at | APH1B | anterior pharynx defective 1 homolog B (C. elegans) | 0.0009324 | -2.03604 |
| 204377_s_at | VPRBP | Vpr (HIV-1) binding protein | 0.0013193 | -2.03656 |
| 218067_s_at | ARGLU1 | arginine and glutamate rich 1 | 0.000528 | -2.03661 |
| 227593_at | hCG_2008140 | hypothetical LOC729614 | 0.000977 | -2.03749 |
| 227873_at | TXNDC15 | thioredoxin domain containing 15 | 0.0009984 | -2.03775 |
| 1554053_at | SPTLC1 | serine palmitoyltransferase, long chain base subunit 1 | 0.0011462 | -2.03789 |
| 1552430_at | WDR17 | WD repeat domain 17 | 0.0010252 | -2.03824 |
| 219848_s_at | ZNF432 | zinc finger protein 432 | 0.001685 | -2.0391 |
| 212337_at | TUG1 | taurine upregulated 1 (non-protein coding) | 0.0014681 | -2.03934 |
| 226995_at | LOC642852 | hypothetical LOC642852 | 0.0003781 | -2.03945 |
| 227352_at | C19orf39 | chromosome 19 open reading frame 39 | 0.0014882 | -2.03983 |
| 223078_s_at | TMOD3 | tropomodulin 3 (ubiquitous) | 0.0017725 | -2.03986 |
| 218854_at | DSE | dermatan sulfate epimerase | 0.0006682 | -2.04029 |
| 200793_s_at | ACO2 | aconitase 2, mitochondrial | 0.00045 | -2.04032 |
| 224947_at | RNF26 | ring finger protein 26 | 0.0007824 | -2.04061 |
| 204887_s_at | PLK4 | polo-like kinase 4 (Drosophila) | 1.19E-06 | -2.04085 |
| 226529_at | TMEM106B | transmembrane protein 106B | 2.18E-05 | -2.04116 |
| 1559957_a_at | LOC642852 | hypothetical LOC642852 | 6.09E-05 | -2.04121 |
| 209307_at | SWAP70 | SWAP switching B-cell complex 70kDa subunit | 0.0003415 | -2.04135 |
| 207439_s_at | SLC35A2 | solute carrier family 35 (UDP-galactose transporter), member A2 | 0.0001446 | -2.04165 |
| 225486_at | ARID2 | AT rich interactive domain 2 (ARID, RFX-like) | 1.94E-05 | -2.04202 |
| 232398_at | CCDC150 | coiled-coil domain containing 150 | 1.00E-05 | -2.04204 |
| 226767_s_at | FAHD1 | fumarylacetoacetate hydrolase domain containing 1 | 0.0008441 | -2.04214 |
| 214958_s_at | TMC6 | transmembrane channel-like 6 | 0.0002352 | -2.04225 |
| 1557385_at | FAM161A | family with sequence similarity 161, member A | 0.001465 | -2.04225 |
| 222583_s_at | NUP50 | nucleoporin 50kDa | 0.0003152 | -2.04245 |
| 238529_at | LOC730631 | Hypothetical LOC730631 | 0.0008486 | -2.04251 |
| 235089_at | FBXL20 | F-box and leucine-rich repeat protein 20 | 0.0001112 | -2.04272 |
| 218136_s_at | SLC25A37 | solute carrier family 25, member 37 | 0.000944 | -2.04306 |
| 202800_at | SLC1A3 | solute carrier family 1 (glial high affinity glutamate transporter), member 3 | 0.0014136 | -2.04355 |
| 203347_s_at | MTF2 | metal response element binding transcription factor 2 | 0.0018117 | -2.04395 |
| 207390_s_at | SMTN | smoothelin | 0.0001303 | -2.04496 |
| 215346_at | CD40 | CD40 molecule, TNF receptor superfamily member 5 | 0.0002088 | -2.04517 |
| 228122_at | CCDC66 | coiled-coil domain containing 66 | 1.61E-05 | -2.04562 |
| 226145_s_at | FRAS1 | Fraser syndrome 1 | 1.55E-06 | -2.04608 |
| 227606_s_at | STAMBPL1 | STAM binding protein-like 1 | 2.32E-05 | -2.04637 |
| 209195_s_at | ADCY6 | adenylate cyclase 6 | 2.38E-05 | -2.04655 |
| 211801_x_at | MFN1 | mitofusin 1 | 0.0005251 | -2.04683 |
| 202813_at | TARBP1 | TAR (HIV-1) RNA binding protein 1 | 0.0002874 | -2.04789 |
| 243815_at | PGBD4 | piggyBac transposable element derived 4 | 0.0015851 | -2.04804 |
| 222626_at | RBM26 | RNA binding motif protein 26 | 0.0002672 | -2.04844 |
| 204773_at | IL11RA | interleukin 11 receptor, alpha | 0.0006922 | -2.04863 |
| 228141_at | GPX8 | glutathione peroxidase 8 (putative) | 0.0009615 | -2.04865 |
| 219769_at | INCENP | inner centromere protein antigens 135/155kDa | 0.0008028 | -2.04947 |
| 214513_s_at | CREB1 | cAMP responsive element binding protein 1 | 0.0008796 | -2.04948 |
| 1558014_s_at | FAR1 | fatty acyl CoA reductase 1 | 0.0018448 | -2.04963 |
| 202770_s_at | CCNG2 | cyclin G2 | 0.0007567 | -2.0501 |
| 203291_at | CNOT4 | CCR4-NOT transcription complex, subunit 4 | 0.0005462 | -2.05094 |
| 202190_at | CSTF1 | cleavage stimulation factor, 3' pre-RNA, subunit 1, 50kDa | 0.0002014 | -2.05095 |
| 224980_at | LEMD2 | LEM domain containing 2 | 0.0001562 | -2.0511 |
| 202017_at | EPHX1 | epoxide hydrolase 1, microsomal (xenobiotic) | 0.0011857 | -2.05171 |
| 214211_at | FTH1 | ferritin, heavy polypeptide 1 | 5.05E-06 | -2.05176 |
| 205816_at | ITGB8 | integrin, beta 8 | 9.55E-05 | -2.05218 |
| 218248_at | FAM111A | family with sequence similarity 111, member A | 8.47E-05 | -2.0525 |
| 222673_x_at | FAM122B | family with sequence similarity 122B | 0.0018767 | -2.05283 |
| 1565149_at | DYNC2H1 | dynein, cytoplasmic 2, heavy chain 1 | 6.37E-05 | -2.05295 |
| 215789_s_at | AJAP1 | adherens junctions associated protein 1 | 0.000814 | -2.05323 |
| 217538_at | SGSM2 | small G protein signaling modulator 2 | 0.0012216 | -2.05435 |
| 64900_at | FLJ22167 | hypothetical protein FLJ22167 | 0.000453 | -2.05483 |
| 204827_s_at | CCNF | cyclin F | 2.32E-05 | -2.05513 |
| 214778_at | MEGF8 | multiple EGF-like-domains 8 | 0.0017655 | -2.05662 |
| 207183_at | GPR19 | G protein-coupled receptor 19 | 0.0007209 | -2.05683 |
| 204293_at | SGSH | N-sulfoglucosamine sulfohydrolase | 0.0010812 | -2.05702 |
| 212606_at | WDFY3 | WD repeat and FYVE domain containing 3 | 3.26E-06 | -2.05831 |
| 241820_at | RIF1 | RAP1 interacting factor homolog (yeast) | 0.0006041 | -2.05861 |
| 1554167_a_at | GOLGA7 | golgi autoantigen, golgin subfamily a, 7 | 0.0001099 | -2.05862 |
| 1555797_a_at | ARPC5 | actin related protein 2/3 complex, subunit 5, 16kDa | 0.0004615 | -2.0592 |
| 201792_at | AEBP1 | AE binding protein 1 | 0.0009462 | -2.05975 |
| 214020_x_at | ITGB5 | Integrin, beta 5 | 0.0015973 | -2.05984 |
| 236953_s_at | NHLRC3 | NHL repeat containing 3 | 0.0005665 | -2.06008 |
| 224929_at | TMEM173 | transmembrane protein 173 | 0.0002545 | -2.06104 |
| 203149_at | PVRL2 | poliovirus receptor-related 2 (herpesvirus entry mediator B) | 1.73E-05 | -2.06174 |
| 218370_s_at | S100PBP | S100P binding protein | 0.000493 | -2.06174 |
| 217547_x_at | ZNF675 | zinc finger protein 675 | 4.82E-05 | -2.06196 |
| 210172_at | SF1 | splicing factor 1 | 0.000585 | -2.06246 |
| 204523_at | ZNF140 | zinc finger protein 140 | 0.0003394 | -2.06259 |
| 230746_s_at | LOC100288985 | hypothetical protein LOC100288985 | 0.000949 | -2.06358 |
| 218724_s_at | TGIF2 | TGFB-induced factor homeobox 2 | 0.0003292 | -2.06376 |
| 238001_at | KCTD6 | potassium channel tetramerisation domain containing 6 | 0.0005321 | -2.06384 |
| 209689_at | CCDC93 | coiled-coil domain containing 93 | 0.0009797 | -2.06425 |
| 47550_at | LZTS1 | leucine zipper, putative tumor suppressor 1 | 6.83E-05 | -2.06585 |
| 203810_at | DNAJB4 | DnaJ (Hsp40) homolog, subfamily B, member 4 | 3.99E-05 | -2.06657 |
| 227014_at | ASPHD2 | aspartate beta-hydroxylase domain containing 2 | 9.25E-06 | -2.06747 |
| 223284_at | NAT14 | N-acetyltransferase 14 (GCN5-related, putative) | 0.000455 | -2.0676 |
| 225002_s_at | SUMF2 | sulfatase modifying factor 2 | 2.56E-05 | -2.06783 |
| 235593_at | ZEB2 | zinc finger E-box binding homeobox 2 | 0.0013276 | -2.06816 |
| 210108_at | CACNA1D | calcium channel, voltage-dependent, L type, alpha 1D subunit | 0.0003497 | -2.06872 |
| 200841_s_at | EPRS | glutamyl-prolyl-tRNA synthetase | 0.0001248 | -2.06902 |
| 230910_s_at | LOC100292183 | hypothetical protein LOC100292183 | 0.0001153 | -2.06926 |
| 203890_s_at | DAPK3 | death-associated protein kinase 3 | 0.0009161 | -2.0695 |
| 227626_at | PAQR8 | progestin and adipoQ receptor family member VIII | 0.0002585 | -2.06957 |
| 1555860_x_at | LOC440944 | hypothetical LOC440944 | 0.0013489 | -2.06965 |
| 227737_at | SRPRB | Signal recognition particle receptor, B subunit | 7.13E-05 | -2.06969 |
| 212810_s_at | SLC1A4 | solute carrier family 1 (glutamate/neutral amino acid transporter), member 4 | 0.001583 | -2.0697 |
| 204670_x_at | HLA-DRB1 /// HLA-DRB4 | major histocompatibility complex, class II, DR beta 1 /// major histocompatibili | 8.16E-05 | -2.06994 |
| 203265_s_at | MAP2K4 | mitogen-activated protein kinase kinase 4 | 0.0016985 | -2.07022 |
| 38241_at | BTN3A3 | butyrophilin, subfamily 3, member A3 | 0.0004781 | -2.07031 |
| 1555465_at | MCOLN2 | mucolipin 2 | 0.0001353 | -2.07039 |
| 237968_at | ATL2 | atlastin GTPase 2 | 9.04E-05 | -2.07114 |
| 205094_at | PEX12 | peroxisomal biogenesis factor 12 | 0.0006495 | -2.07208 |
| 231730_at | SEPSECS | Sep (O-phosphoserine) tRNA:Sec (selenocysteine) tRNA synthase | 0.0004061 | -2.07266 |
| 229175_at | SMYD4 | SET and MYND domain containing 4 | 0.0001968 | -2.07273 |
| 226927_at | C12orf73 | chromosome 12 open reading frame 73 | 7.81E-05 | -2.07274 |
| 213510_x_at | LOC220594 | TL132 protein | 0.0006182 | -2.07296 |
| 210124_x_at | SEMA4F | sema domain, immunoglobulin domain (Ig), transmembrane domain (TM) and short cyt | 0.0002089 | -2.07337 |
| 205449_at | SAC3D1 | SAC3 domain containing 1 | 8.85E-05 | -2.07354 |
| 202991_at | STARD3 | StAR-related lipid transfer (START) domain containing 3 | 7.84E-05 | -2.0739 |
| 1557165_s_at | KLHL18 | kelch-like 18 (Drosophila) | 0.0006092 | -2.07509 |
| 1553830_s_at | MAGEA2 /// MAGEA2B | melanoma antigen family A, 2 /// melanoma antigen family A, 2B | 4.51E-07 | -2.07537 |
| 228416_at | ACVR2A | activin A receptor, type IIA | 0.0015963 | -2.07696 |
| 229450_at | IFIT3 | interferon-induced protein with tetratricopeptide repeats 3 | 0.0009422 | -2.077 |
| 222529_at | SLC25A37 | solute carrier family 25, member 37 | 0.0001447 | -2.07753 |
| 1555500_s_at | SLC2A4RG | SLC2A4 regulator | 0.0001044 | -2.07774 |
| 221675_s_at | CHPT1 | choline phosphotransferase 1 | 8.34E-06 | -2.07837 |
| 201838_s_at | SUPT7L | suppressor of Ty 7 (S. cerevisiae)-like | 0.000421 | -2.0786 |
| 201777_s_at | KIAA0494 | KIAA0494 | 0.000933 | -2.07878 |
| 202111_at | SLC4A2 | solute carrier family 4, anion exchanger, member 2 (erythrocyte membrane protein | 0.0008588 | -2.07914 |
| 227326_at | MXRA7 | matrix-remodelling associated 7 | 0.0003065 | -2.07934 |
| 222555_s_at | MRPL44 | mitochondrial ribosomal protein L44 | 4.99E-06 | -2.07936 |
| 205523_at | HAPLN1 | hyaluronan and proteoglycan link protein 1 | 0.0002569 | -2.07974 |
| 201411_s_at | PLEKHB2 | pleckstrin homology domain containing, family B (evectins) member 2 | 0.001524 | -2.07992 |
| 204283_at | FARS2 | phenylalanyl-tRNA synthetase 2, mitochondrial | 0.0009517 | -2.08162 |
| 225291_at | PNPT1 | polyribonucleotide nucleotidyltransferase 1 | 0.0008278 | -2.08168 |
| 1568815_a_at | DDX50 | DEAD (Asp-Glu-Ala-Asp) box polypeptide 50 | 0.0002322 | -2.08179 |
| 219029_at | C5orf28 | chromosome 5 open reading frame 28 | 0.0007969 | -2.08213 |
| 234347_s_at | DENR | density-regulated protein | 0.000429 | -2.08292 |
| 223847_s_at | ERGIC1 | endoplasmic reticulum-golgi intermediate compartment (ERGIC) 1 | 0.0002301 | -2.08301 |
| 201482_at | QSOX1 | quiescin Q6 sulfhydryl oxidase 1 | 0.0004879 | -2.08495 |
| 225561_at | SELT | selenoprotein T | 2.21E-05 | -2.08559 |
| 222551_s_at | C8orf33 | chromosome 8 open reading frame 33 | 1.20E-05 | -2.08603 |
| 230078_at | RAPGEF6 | Rap guanine nucleotide exchange factor (GEF) 6 | 0.0008325 | -2.08612 |
| 241374_at | TMEM39A | transmembrane protein 39A | 0.0004795 | -2.0862 |
| 203952_at | ATF6 | activating transcription factor 6 | 3.99E-05 | -2.08643 |
| 206316_s_at | KNTC1 | kinetochore associated 1 | 1.71E-05 | -2.08688 |
| 223452_s_at | ATL3 | atlastin GTPase 3 | 0.0011968 | -2.08697 |
| 244038_at | WDR89 | WD repeat domain 89 | 0.0002132 | -2.08718 |
| 215489_x_at | HOMER3 | homer homolog 3 (Drosophila) | 0.0011645 | -2.08804 |
| 225253_s_at | METTL2A /// METTL2B | methyltransferase like 2A /// methyltransferase like 2B | 5.16E-05 | -2.08844 |
| 213742_at | SFRS11 | splicing factor, arginine/serine-rich 11 | 0.0006875 | -2.08869 |
| 227127_at | TMEM110 | Transmembrane protein 110 | 0.0002084 | -2.08917 |
| 214169_at | C7orf20 /// UNC84A | chromosome 7 open reading frame 20 /// unc-84 homolog A (C. elegans) | 0.001696 | -2.08923 |
| 227840_at | C2orf76 | chromosome 2 open reading frame 76 | 0.0009082 | -2.09019 |
| 212315_s_at | NUP210 | nucleoporin 210kDa | 0.0014129 | -2.09074 |
| 213499_at | CLCN2 | chloride channel 2 | 0.0014534 | -2.09092 |
| 1565951_s_at | CHML | choroideremia-like (Rab escort protein 2) | 1.52E-05 | -2.09176 |
| 213727_x_at | MPPE1 | metallophosphoesterase 1 | 0.0004238 | -2.09243 |
| 212575_at | C19orf6 | chromosome 19 open reading frame 6 | 0.0010142 | -2.09263 |
| 218692_at | GOLSYN | Golgi-localized protein | 5.49E-05 | -2.09361 |
| 200799_at | HSPA1A | heat shock 70kDa protein 1A | 9.11E-05 | -2.09395 |
| 221208_s_at | C11orf61 | chromosome 11 open reading frame 61 | 0.000108 | -2.0943 |
| 203865_s_at | ADARB1 | adenosine deaminase, RNA-specific, B1 (RED1 homolog rat) | 1.39E-05 | -2.09507 |
| 220486_x_at | TMEM164 | transmembrane protein 164 | 0.0007851 | -2.09515 |
| 1007_s_at | DDR1 | discoidin domain receptor tyrosine kinase 1 | 1.92E-06 | -2.09524 |
| 208229_at | FGFR2 | fibroblast growth factor receptor 2 | 0.0003167 | -2.09534 |
| 209975_at | CYP2E1 | cytochrome P450, family 2, subfamily E, polypeptide 1 | 0.000181 | -2.09595 |
| 201666_at | TIMP1 | TIMP metallopeptidase inhibitor 1 | 0.0002691 | -2.09634 |
| 204790_at | SMAD7 | SMAD family member 7 | 0.0003361 | -2.09673 |
| 214963_at | NUP160 | nucleoporin 160kDa | 3.96E-05 | -2.09711 |
| 203430_at | HEBP2 | heme binding protein 2 | 7.63E-06 | -2.09773 |
| 224887_at | GNPTG | N-acetylglucosamine-1-phosphate transferase, gamma subunit | 0.0001757 | -2.0978 |
| 225612_s_at | B3GNT5 | UDP-GlcNAc:betaGal beta-1,3-N-acetylglucosaminyltransferase 5 | 2.59E-05 | -2.09786 |
| 201202_at | PCNA | proliferating cell nuclear antigen | 0.0003952 | -2.09791 |
| 227490_at | WDFY2 | WD repeat and FYVE domain containing 2 | 0.0002667 | -2.09802 |
| 236700_at | EIF3C | eukaryotic translation initiation factor 3, subunit C | 0.0016452 | -2.09856 |
| 206487_at | UNC84A | unc-84 homolog A (C. elegans) | 0.0007307 | -2.09861 |
| 46947_at | GNL3L | guanine nucleotide binding protein-like 3 (nucleolar)-like | 0.0007009 | -2.09931 |
| 203400_s_at | TF | transferrin | 0.0005765 | -2.09968 |
| 201905_s_at | CTDSPL | CTD (carboxy-terminal domain, RNA polymerase II, polypeptide A) small phosphatas | 1.31E-05 | -2.10017 |
| 205345_at | BARD1 | BRCA1 associated RING domain 1 | 0.0002304 | -2.10045 |
| 222839_s_at | PAPOLG | poly(A) polymerase gamma | 0.0003421 | -2.1005 |
| 202915_s_at | FAM20B | family with sequence similarity 20, member B | 7.26E-05 | -2.1007 |
| 1554499_s_at | PPA2 | pyrophosphatase (inorganic) 2 | 0.0001471 | -2.10077 |
| 1554602_at | RBM8A | RNA binding motif protein 8A | 0.000336 | -2.1011 |
| 213594_x_at | FUSIP1 | FUS interacting protein (serine/arginine-rich) 1 | 1.95E-06 | -2.10121 |
| 1555952_at | SLC19A1 | solute carrier family 19 (folate transporter), member 1 | 0.0015434 | -2.10144 |
| 206170_at | ADRB2 | adrenergic, beta-2-, receptor, surface | 0.0001149 | -2.10251 |
| 204061_at | PRKX | protein kinase, X-linked | 0.0001467 | -2.10272 |
| 210995_s_at | TRIM23 | tripartite motif-containing 23 | 0.0010573 | -2.10275 |
| 212154_at | SDC2 | syndecan 2 | 0.0018242 | -2.10292 |
| 213479_at | NPTX2 | neuronal pentraxin II | 0.0016643 | -2.1037 |
| 220436_at | RP11-138L21.1 | similar to cell recognition molecule CASPR3 | 0.0016777 | -2.10399 |
| 226064_s_at | DGAT2 | diacylglycerol O-acyltransferase homolog 2 (mouse) | 0.0011884 | -2.10497 |
| 202418_at | YIF1A | Yip1 interacting factor homolog A (S. cerevisiae) | 0.0004651 | -2.10509 |
| 223701_s_at | USP47 | ubiquitin specific peptidase 47 | 0.0013308 | -2.10511 |
| 201727_s_at | ELAVL1 | ELAV (embryonic lethal, abnormal vision, Drosophila)-like 1 (Hu antigen R) | 9.33E-05 | -2.10556 |
| 204806_x_at | HLA-F | major histocompatibility complex, class I, F | 6.46E-07 | -2.10567 |
| 212340_at | YIPF6 | Yip1 domain family, member 6 | 6.27E-05 | -2.10586 |
| 208902_s_at | RPS28 | ribosomal protein S28 | 0.0001921 | -2.10591 |
| 207980_s_at | CITED2 | Cbp/p300-interacting transactivator, with Glu/Asp-rich carboxy-terminal domain, | 0.0007106 | -2.10672 |
| 223212_at | ZDHHC16 | zinc finger, DHHC-type containing 16 | 4.28E-06 | -2.10706 |
| 222843_at | FIGNL1 | fidgetin-like 1 | 2.05E-05 | -2.10747 |
| 204126_s_at | CDC45L | CDC45 cell division cycle 45-like (S. cerevisiae) | 1.04E-05 | -2.1081 |
| 219450_at | C4orf19 | chromosome 4 open reading frame 19 | 5.25E-05 | -2.10894 |
| 1554478_a_at | HEATR3 | HEAT repeat containing 3 | 0.0004698 | -2.10896 |
| 230364_at | CHPT1 | choline phosphotransferase 1 | 0.0003584 | -2.109 |
| 217944_at | POMGNT1 | protein O-linked mannose beta1,2-N-acetylglucosaminyltransferase | 0.0008121 | -2.10921 |
| 1569302_at | KIAA1731 | KIAA1731 | 0.0004062 | -2.10949 |
| 204886_at | PLK4 | polo-like kinase 4 (Drosophila) | 0.0001856 | -2.10958 |
| 219303_at | RNF219 | ring finger protein 219 | 0.0003041 | -2.10958 |
| 224818_at | SORT1 | sortilin 1 | 0.0006929 | -2.1098 |
| 220060_s_at | C12orf48 | chromosome 12 open reading frame 48 | 0.0002944 | -2.11007 |
| 221520_s_at | CDCA8 | cell division cycle associated 8 | 0.0017046 | -2.11104 |
| 209954_x_at | SS18 | synovial sarcoma translocation, chromosome 18 | 0.0001031 | -2.11119 |
| 219904_at | ZSCAN5A | zinc finger and SCAN domain containing 5A | 0.0003629 | -2.11161 |
| 226370_at | KLHL15 | kelch-like 15 (Drosophila) | 0.0013047 | -2.11166 |
| 203148_s_at | TRIM14 | tripartite motif-containing 14 | 2.56E-05 | -2.11174 |
| 219294_at | CENPQ | centromere protein Q | 0.0001548 | -2.11264 |
| 227693_at | WDR20 | WD repeat domain 20 | 0.0007307 | -2.11314 |
| 225161_at | GFM1 | G elongation factor, mitochondrial 1 | 4.35E-06 | -2.11315 |
| 213360_s_at | POM121 /// POM121C | POM121 membrane glycoprotein (rat) /// POM121 membrane glycoprotein C | 0.0001253 | -2.11329 |
| 206175_x_at | ZNF222 | zinc finger protein 222 | 0.000102 | -2.1143 |
| 227234_at | LOC100132815 | Hypothetical protein LOC100132815 | 0.001091 | -2.1146 |
| 203934_at | KDR | kinase insert domain receptor (a type III receptor tyrosine kinase) | 0.0004683 | -2.11483 |
| 229344_x_at | RIMKLB | ribosomal modification protein rimK-like family member B | 0.0001594 | -2.11567 |
| 224460_s_at | L2HGDH | L-2-hydroxyglutarate dehydrogenase | 0.0009386 | -2.11613 |
| 232994_s_at | RGNEF | Rho-guanine nucleotide exchange factor | 0.0010569 | -2.11615 |
| 225009_at | CMTM4 | CKLF-like MARVEL transmembrane domain containing 4 | 0.0004401 | -2.11628 |
| 202466_at | POLS | polymerase (DNA directed) sigma | 2.00E-05 | -2.1169 |
| 210416_s_at | CHEK2 | CHK2 checkpoint homolog (S. pombe) | 0.0005682 | -2.11705 |
| 201172_x_at | ATP6V0E1 | ATPase, H+ transporting, lysosomal 9kDa, V0 subunit e1 | 1.39E-05 | -2.1172 |
| 207798_s_at | ATXN2L | ataxin 2-like | 1.86E-06 | -2.11737 |
| 218929_at | CDKN2AIP | CDKN2A interacting protein | 0.0018359 | -2.11746 |
| 242455_at | POU3F2 | POU class 3 homeobox 2 | 0.0001187 | -2.11766 |
| 232138_at | MBNL2 | Muscleblind-like 2 (Drosophila) | 0.0004276 | -2.11783 |
| 207856_s_at | LOC150776 /// SMPD4 | sphingomyelin phosphodiesterase 4, neutral membrane pseudogene /// sphingomyelin | 2.43E-05 | -2.1186 |
| 210537_s_at | TADA2L | transcriptional adaptor 2 (ADA2 homolog, yeast)-like | 7.10E-05 | -2.11932 |
| 213902_at | ASAH1 | N-acylsphingosine amidohydrolase (acid ceramidase) 1 | 1.03E-05 | -2.11942 |
| 242538_at | TFDP1 | Transcription factor Dp-1 | 0.0011262 | -2.11979 |
| 231720_s_at | JAM3 | junctional adhesion molecule 3 | 0.0004397 | -2.12035 |
| 212830_at | MEGF9 | multiple EGF-like-domains 9 | 1.83E-05 | -2.12063 |
| 230492_s_at | RP5-1022P6.2 | hypothetical protein KIAA1434 | 0.0004683 | -2.12092 |
| 202027_at | TMEM184B | transmembrane protein 184B | 0.0008713 | -2.12145 |
| 214785_at | VPS13A | vacuolar protein sorting 13 homolog A (S. cerevisiae) | 0.0011336 | -2.12171 |
| 227632_at | TBC1D24 | TBC1 domain family, member 24 | 0.0006619 | -2.12177 |
| 204245_s_at | RPP14 | ribonuclease P/MRP 14kDa subunit | 3.18E-05 | -2.12189 |
| 225686_at | SKA2 | spindle and kinetochore associated complex subunit 2 | 0.000111 | -2.12223 |
| 209054_s_at | WHSC1 | Wolf-Hirschhorn syndrome candidate 1 | 4.17E-07 | -2.12257 |
| 212546_s_at | FRYL | FRY-like | 0.000941 | -2.12265 |
| 202226_s_at | CRK | v-crk sarcoma virus CT10 oncogene homolog (avian) | 7.36E-05 | -2.12301 |
| 242635_s_at | NAPEPLD | N-acyl phosphatidylethanolamine phospholipase D | 0.0003639 | -2.12344 |
| 205658_s_at | SNAPC4 | small nuclear RNA activating complex, polypeptide 4, 190kDa | 2.38E-05 | -2.12357 |
| 223292_s_at | MRPS15 | mitochondrial ribosomal protein S15 | 0.0001335 | -2.12382 |
| 235467_s_at | KCNC4 | potassium voltage-gated channel, Shaw-related subfamily, member 4 | 4.77E-05 | -2.12415 |
| 208296_x_at | TNFAIP8 | tumor necrosis factor, alpha-induced protein 8 | 0.0006589 | -2.12421 |
| 38158_at | ESPL1 | extra spindle pole bodies homolog 1 (S. cerevisiae) | 6.65E-07 | -2.12486 |
| 239913_at | SLC10A4 | solute carrier family 10 (sodium/bile acid cotransporter family), member 4 | 1.78E-05 | -2.12505 |
| 41660_at | CELSR1 | cadherin, EGF LAG seven-pass G-type receptor 1 (flamingo homolog, Drosophila) | 6.10E-05 | -2.12541 |
| 208504_x_at | PCDHB11 | protocadherin beta 11 | 0.0001606 | -2.12544 |
| 229665_at | CSTF3 | cleavage stimulation factor, 3' pre-RNA, subunit 3, 77kDa | 0.0015012 | -2.12714 |
| 227213_at | ADAT2 | adenosine deaminase, tRNA-specific 2, TAD2 homolog (S. cerevisiae) | 0.0002017 | -2.12748 |
| 218732_at | PTRH2 | peptidyl-tRNA hydrolase 2 | 8.32E-06 | -2.12757 |
| 209626_s_at | OSBPL3 | oxysterol binding protein-like 3 | 0.0004364 | -2.12772 |
| 202363_at | SPOCK1 | sparc/osteonectin, cwcv and kazal-like domains proteoglycan (testican) 1 | 0.0005236 | -2.1283 |
| 207013_s_at | MMP16 | matrix metallopeptidase 16 (membrane-inserted) | 8.83E-06 | -2.12915 |
| 214036_at | EFNA5 | ephrin-A5 | 4.66E-05 | -2.13032 |
| 235400_at | FCRLA | Fc receptor-like A | 0.0001225 | -2.13045 |
| 212020_s_at | MKI67 | antigen identified by monoclonal antibody Ki-67 | 1.34E-05 | -2.13052 |
| 235333_at | B4GALT6 | UDP-Gal:betaGlcNAc beta 1,4- galactosyltransferase, polypeptide 6 | 5.11E-05 | -2.13103 |
| 203126_at | IMPA2 | inositol(myo)-1(or 4)-monophosphatase 2 | 0.0013921 | -2.13197 |
| 222992_s_at | NDUFB9 | NADH dehydrogenase (ubiquinone) 1 beta subcomplex, 9, 22kDa | 2.31E-06 | -2.13303 |
| 218918_at | MAN1C1 | mannosidase, alpha, class 1C, member 1 | 0.0003775 | -2.1339 |
| 219378_at | NARG1L | NMDA receptor regulated 1-like | 0.0007107 | -2.13453 |
| 204689_at | HHEX | hematopoietically expressed homeobox | 0.0013423 | -2.13457 |
| 201823_s_at | RNF14 | ring finger protein 14 | 0.0001734 | -2.13473 |
| 201062_at | STOM | stomatin | 0.0003453 | -2.13519 |
| 210962_s_at | AKAP9 | A kinase (PRKA) anchor protein (yotiao) 9 | 4.08E-06 | -2.13538 |
| 201508_at | IGFBP4 | insulin-like growth factor binding protein 4 | 0.0003744 | -2.13554 |
| 220399_at | NCRNA00115 | non-protein coding RNA 115 | 7.82E-06 | -2.13556 |
| 223201_s_at | TMEM164 | transmembrane protein 164 | 0.000518 | -2.13581 |
| 209608_s_at | ACAT2 | acetyl-Coenzyme A acetyltransferase 2 | 1.62E-05 | -2.13718 |
| 231846_at | FOXRED2 | FAD-dependent oxidoreductase domain containing 2 | 0.0004223 | -2.13998 |
| 212556_at | SCRIB | scribbled homolog (Drosophila) | 5.82E-05 | -2.14009 |
| 217867_x_at | BACE2 | beta-site APP-cleaving enzyme 2 | 0.0001684 | -2.14018 |
| 56821_at | SLC38A7 | solute carrier family 38, member 7 | 0.0001427 | -2.14107 |
| 1552378_s_at | RDH10 | retinol dehydrogenase 10 (all-trans) | 1.17E-05 | -2.14169 |
| 234299_s_at | NIN | ninein (GSK3B interacting protein) | 5.60E-05 | -2.14209 |
| 226356_at | FAM73B | family with sequence similarity 73, member B | 8.47E-05 | -2.14231 |
| 216526_x_at | HLA-C | major histocompatibility complex, class I, C | 4.99E-05 | -2.14256 |
| 218897_at | TMEM177 | transmembrane protein 177 | 0.0007078 | -2.1426 |
| 244811_at | PHIP | pleckstrin homology domain interacting protein | 9.22E-05 | -2.14371 |
| 226555_at | INO80D | INO80 complex subunit D | 0.0001778 | -2.14408 |
| 218485_s_at | SLC35C1 | solute carrier family 35, member C1 | 7.91E-06 | -2.14551 |
| 219785_s_at | FBXO31 /// LOC100293820 | F-box protein 31 /// hypothetical protein LOC100293820 | 8.05E-05 | -2.14649 |
| 201504_s_at | TSN | translin | 0.0002045 | -2.14749 |
| 219423_x_at | TNFRSF25 | tumor necrosis factor receptor superfamily, member 25 | 0.0001477 | -2.14756 |
| 207084_at | POU3F2 | POU class 3 homeobox 2 | 0.0006871 | -2.14777 |
| 226668_at | WDSUB1 | WD repeat, sterile alpha motif and U-box domain containing 1 | 0.0005405 | -2.14815 |
| 219449_s_at | TMEM70 | transmembrane protein 70 | 5.48E-07 | -2.14875 |
| 203226_s_at | TSPAN31 | tetraspanin 31 | 0.000157 | -2.15068 |
| 238493_at | ZNF506 | zinc finger protein 506 | 0.0001665 | -2.15133 |
| 205443_at | SNAPC1 | small nuclear RNA activating complex, polypeptide 1, 43kDa | 0.0005951 | -2.15148 |
| 226946_at | C5orf33 | chromosome 5 open reading frame 33 | 5.68E-06 | -2.1522 |
| 203725_at | GADD45A | growth arrest and DNA-damage-inducible, alpha | 0.000446 | -2.15221 |
| 210959_s_at | SRD5A1 | steroid-5-alpha-reductase, alpha polypeptide 1 (3-oxo-5 alpha-steroid delta 4-de | 0.0018234 | -2.15315 |
| 1554894_a_at | PCBD2 | pterin-4 alpha-carbinolamine dehydratase/dimerization cofactor of hepatocyte nuc | 7.08E-06 | -2.15329 |
| 233186_s_at | BANP | BTG3 associated nuclear protein | 0.0001019 | -2.15364 |
| 212282_at | TMEM97 | transmembrane protein 97 | 5.93E-06 | -2.15372 |
| 208070_s_at | REV3L | REV3-like, catalytic subunit of DNA polymerase zeta (yeast) | 0.0011012 | -2.15384 |
| 223513_at | CENPJ | centromere protein J | 5.00E-05 | -2.15471 |
| 203056_s_at | PRDM2 | PR domain containing 2, with ZNF domain | 0.0015238 | -2.15487 |
| 210656_at | EED | embryonic ectoderm development | 0.0012499 | -2.15582 |
| 235204_at | ENTPD7 | ectonucleoside triphosphate diphosphohydrolase 7 | 0.0001484 | -2.15609 |
| 223666_at | SNX5 | sorting nexin 5 | 0.0009046 | -2.15648 |
| 213650_at | GOLGA8A /// GOLGA8B | golgi autoantigen, golgin subfamily a, 8A /// golgi autoantigen, golgin subfamil | 0.000484 | -2.15675 |
| 207891_s_at | HAUS7 /// TREX2 | HAUS augmin-like complex, subunit 7 /// three prime repair exonuclease 2 | 0.0007694 | -2.15723 |
| 223857_x_at | TMEM85 | transmembrane protein 85 | 0.0001664 | -2.15847 |
| 224164_at | TPM3 | tropomyosin 3 | 0.0011367 | -2.1589 |
| 239761_at | GCNT1 | glucosaminyl (N-acetyl) transferase 1, core 2 (beta-1,6-N-acetylglucosaminyltran | 0.0012224 | -2.15897 |
| 205287_s_at | TFAP2C | transcription factor AP-2 gamma (activating enhancer binding protein 2 gamma) | 0.0001404 | -2.15942 |
| 213386_at | C9orf125 | chromosome 9 open reading frame 125 | 9.44E-06 | -2.15984 |
| 218032_at | SNN | stannin | 3.07E-05 | -2.16018 |
| 227072_at | RTTN | rotatin | 0.0001494 | -2.16147 |
| 236192_at | HOOK3 | hook homolog 3 (Drosophila) | 0.0007982 | -2.16229 |
| 200729_s_at | ACTR2 | ARP2 actin-related protein 2 homolog (yeast) | 0.0014524 | -2.16235 |
| 1557350_at | G3BP1 | GTPase activating protein (SH3 domain) binding protein 1 | 0.0001118 | -2.16288 |
| 226473_at | CBX2 | chromobox homolog 2 (Pc class homolog, Drosophila) | 2.52E-05 | -2.16329 |
| 203505_at | ABCA1 | ATP-binding cassette, sub-family A (ABC1), member 1 | 9.26E-06 | -2.16406 |
| 221695_s_at | MAP3K2 | mitogen-activated protein kinase kinase kinase 2 | 0.0013232 | -2.1642 |
| 226934_at | CPSF6 | cleavage and polyadenylation specific factor 6, 68kDa | 0.0018567 | -2.16447 |
| 228184_at | DISP1 | dispatched homolog 1 (Drosophila) | 0.001022 | -2.1649 |
| 220825_s_at | KIRREL | kin of IRRE like (Drosophila) | 5.74E-05 | -2.1658 |
| 211017_s_at | NF2 | neurofibromin 2 (merlin) | 0.0009414 | -2.16654 |
| 226020_s_at | DAB1 /// OMA1 | disabled homolog 1 (Drosophila) /// OMA1 homolog, zinc metallopeptidase (S. cere | 0.0003453 | -2.16656 |
| 35671_at | GTF3C1 | general transcription factor IIIC, polypeptide 1, alpha 220kDa | 2.86E-05 | -2.16659 |
| 210514_x_at | HLA-G | major histocompatibility complex, class I, G | 3.11E-05 | -2.16662 |
| 221436_s_at | CDCA3 | cell division cycle associated 3 | 0.0001332 | -2.16675 |
| 224873_s_at | MRPS25 | mitochondrial ribosomal protein S25 | 0.0006495 | -2.16708 |
| 223786_at | CHST6 | carbohydrate (N-acetylglucosamine 6-O) sulfotransferase 6 | 0.0009104 | -2.16768 |
| 222958_s_at | DEPDC1 | DEP domain containing 1 | 4.82E-06 | -2.16769 |
| 1565436_s_at | MLL | myeloid/lymphoid or mixed-lineage leukemia (trithorax homolog, Drosophila) | 0.0012402 | -2.16783 |
| 200748_s_at | FTH1 | ferritin, heavy polypeptide 1 | 9.48E-06 | -2.16798 |
| 225744_at | ZDHHC8 | zinc finger, DHHC-type containing 8 | 0.0001732 | -2.1682 |
| 214537_at | HIST1H1D | histone cluster 1, H1d | 0.0012213 | -2.16842 |
| 232007_at | AGPAT5 | 1-acylglycerol-3-phosphate O-acyltransferase 5 (lysophosphatidic acid acyltransf | 0.0001081 | -2.16843 |
| 203771_s_at | BLVRA | biliverdin reductase A | 0.0003638 | -2.16908 |
| 205928_at | ZNF443 | zinc finger protein 443 | 0.0002485 | -2.16913 |
| 212958_x_at | PAM | peptidylglycine alpha-amidating monooxygenase | 0.0001943 | -2.17072 |
| 243011_at | FAM55C | family with sequence similarity 55, member C | 0.0002901 | -2.17198 |
| 234488_s_at | GMCL1 /// GMCL1L | germ cell-less homolog 1 (Drosophila) /// germ cell-less homolog 1 (Drosophila)- | 0.0002862 | -2.17212 |
| 210338_s_at | HSPA8 | heat shock 70kDa protein 8 | 2.07E-06 | -2.17254 |
| 213355_at | ST3GAL6 | ST3 beta-galactoside alpha-2,3-sialyltransferase 6 | 0.0014738 | -2.17289 |
| 1554408_a_at | TK1 | thymidine kinase 1, soluble | 1.02E-05 | -2.17325 |
| 232563_at | ZNF684 | zinc finger protein 684 | 0.0018764 | -2.17367 |
| 205519_at | WDR76 | WD repeat domain 76 | 0.0002897 | -2.17431 |
| 208475_at | FRMD4A | FERM domain containing 4A | 0.000962 | -2.17459 |
| 225432_s_at | CSRP2BP | CSRP2 binding protein | 0.0003323 | -2.17529 |
| 227241_at | MUC15 | mucin 15, cell surface associated | 0.0002466 | -2.17564 |
| 213932_x_at | HLA-A | major histocompatibility complex, class I, A | 2.79E-05 | -2.17589 |
| 219411_at | ELMO3 | engulfment and cell motility 3 | 0.0003135 | -2.17607 |
| 243610_at | C9orf135 | chromosome 9 open reading frame 135 | 0.0002539 | -2.17669 |
| 215114_at | SENP3 | SUMO1/sentrin/SMT3 specific peptidase 3 | 0.0003967 | -2.17688 |
| 222747_s_at | SCML1 | sex comb on midleg-like 1 (Drosophila) | 0.0007407 | -2.17711 |
| 219002_at | FASTKD1 | FAST kinase domains 1 | 0.0002663 | -2.1783 |
| 203972_s_at | PEX3 | peroxisomal biogenesis factor 3 | 3.06E-06 | -2.18011 |
| 223898_at | ZNF670 | zinc finger protein 670 | 0.0010831 | -2.18068 |
| 226014_at | EIF3F | Eukaryotic translation initiation factor 3, subunit F | 0.0004022 | -2.18078 |
| 228638_at | FAM76A | Family with sequence similarity 76, member A | 0.0014742 | -2.18113 |
| 228378_at | C12orf29 | chromosome 12 open reading frame 29 | 0.0001847 | -2.18138 |
| 37170_at | BMP2K | BMP2 inducible kinase | 0.0002605 | -2.18139 |
| 1559881_s_at | ZNF12 | zinc finger protein 12 | 0.0005336 | -2.183 |
| 227925_at | FLJ39051 | Hypothetical gene supported by AK096370 | 0.0003637 | -2.18338 |
| 232228_at | ZNF530 | zinc finger protein 530 | 0.0018497 | -2.18361 |
| 202880_s_at | CYTH1 | cytohesin 1 | 0.0008377 | -2.18367 |
| 1555202_a_at | RPRD1A | regulation of nuclear pre-mRNA domain containing 1A | 0.001165 | -2.18377 |
| 226840_at | H2AFY | H2A histone family, member Y | 0.0001081 | -2.18436 |
| 201971_s_at | ATP6V1A | ATPase, H+ transporting, lysosomal 70kDa, V1 subunit A | 0.0003596 | -2.18464 |
| 203702_s_at | TTLL4 | tubulin tyrosine ligase-like family, member 4 | 0.0018446 | -2.18484 |
| 202153_s_at | NUP62 | nucleoporin 62kDa | 2.77E-05 | -2.18549 |
| 222963_s_at | IL1RAPL1 | interleukin 1 receptor accessory protein-like 1 | 0.0004436 | -2.1859 |
| 212129_at | NIPA2 | non imprinted in Prader-Willi/Angelman syndrome 2 | 9.49E-08 | -2.18609 |
| 211572_s_at | SLC23A2 | solute carrier family 23 (nucleobase transporters), member 2 | 0.001301 | -2.18634 |
| 1552928_s_at | MAP3K7IP3 | mitogen-activated protein kinase kinase kinase 7 interacting protein 3 | 0.0005465 | -2.18783 |
| 206473_at | MBTPS2 | membrane-bound transcription factor peptidase, site 2 | 4.90E-06 | -2.18882 |
| 226080_at | SSH2 | slingshot homolog 2 (Drosophila) | 0.0001397 | -2.18898 |
| 209468_at | LRP5 | low density lipoprotein receptor-related protein 5 | 0.0001222 | -2.18941 |
| 222713_s_at | FANCF | Fanconi anemia, complementation group F | 3.21E-06 | -2.18955 |
| 203270_at | DTYMK | deoxythymidylate kinase (thymidylate kinase) | 0.000565 | -2.18958 |
| 1556389_at | CNPY3 | canopy 3 homolog (zebrafish) | 2.46E-05 | -2.19035 |
| 224566_at | NEAT1 | nuclear paraspeckle assembly transcript 1 (non-protein coding) | 0.0001679 | -2.19056 |
| 230508_at | DKK3 | dickkopf homolog 3 (Xenopus laevis) | 0.001246 | -2.19195 |
| 212062_at | ATP9A | ATPase, class II, type 9A | 0.0005072 | -2.19198 |
| 38671_at | PLXND1 | plexin D1 | 0.0012017 | -2.192 |
| 218940_at | C14orf138 | chromosome 14 open reading frame 138 | 0.0010359 | -2.1926 |
| 203710_at | ITPR1 | inositol 1,4,5-triphosphate receptor, type 1 | 0.0001008 | -2.19262 |
| 238470_at | SYS1 | SYS1 Golgi-localized integral membrane protein homolog (S. cerevisiae) | 0.0006605 | -2.19285 |
| 207264_at | KDELR3 | KDEL (Lys-Asp-Glu-Leu) endoplasmic reticulum protein retention receptor 3 | 0.0011368 | -2.19384 |
| 228847_at | EXOC3 | exocyst complex component 3 | 0.0005345 | -2.19411 |
| 236128_at | ZNF91 | zinc finger protein 91 | 0.0007623 | -2.19437 |
| 202969_at | DYRK2 | dual-specificity tyrosine-(Y)-phosphorylation regulated kinase 2 | 0.0003098 | -2.19522 |
| 1556186_s_at | KIAA0090 | KIAA0090 | 0.0001433 | -2.19584 |
| 229671_s_at | C21orf45 | Chromosome 21 open reading frame 45 | 3.05E-05 | -2.1959 |
| 212856_at | GRAMD4 | GRAM domain containing 4 | 0.0003047 | -2.19738 |
| 244470_at | RLIM | Ring finger protein, LIM domain interacting | 0.0005441 | -2.19767 |
| 218733_at | MSL2 | male-specific lethal 2 homolog (Drosophila) | 5.34E-05 | -2.198 |
| 243000_at | CDK6 | cyclin-dependent kinase 6 | 0.0001956 | -2.19861 |
| 238974_at | C2orf69 | chromosome 2 open reading frame 69 | 3.91E-05 | -2.19877 |
| 230050_at | NACC2 | NACC family member 2, BEN and BTB (POZ) domain containing | 0.0002695 | -2.19929 |
| 215023_s_at | PEX1 | peroxisomal biogenesis factor 1 | 0.0008543 | -2.19993 |
| 1554885_a_at | PRIM2 | primase, DNA, polypeptide 2 (58kDa) | 0.0002816 | -2.2007 |
| 225489_at | TMEM18 | transmembrane protein 18 | 0.0018618 | -2.20199 |
| 236471_at | NFE2L3 | nuclear factor (erythroid-derived 2)-like 3 | 4.43E-05 | -2.20259 |
| 210233_at | IL1RAP | interleukin 1 receptor accessory protein | 0.0011168 | -2.20382 |
| 210334_x_at | BIRC5 | baculoviral IAP repeat-containing 5 | 0.0005934 | -2.2052 |
| 200068_s_at | CANX | calnexin | 3.57E-09 | -2.20522 |
| 213916_at | ZNF20 /// ZNF625 | zinc finger protein 20 /// zinc finger protein 625 | 0.0003558 | -2.20523 |
| 208459_s_at | XPO7 | exportin 7 | 8.35E-05 | -2.20596 |
| 204828_at | RAD9A | RAD9 homolog A (S. pombe) | 2.22E-05 | -2.20686 |
| 1554878_a_at | ABCD3 | ATP-binding cassette, sub-family D (ALD), member 3 | 6.49E-06 | -2.20695 |
| 205066_s_at | ENPP1 | ectonucleotide pyrophosphatase/phosphodiesterase 1 | 0.0008373 | -2.20698 |
| 212141_at | MCM4 | minichromosome maintenance complex component 4 | 0.0009486 | -2.20737 |
| 212416_at | SCAMP1 | secretory carrier membrane protein 1 | 0.0007693 | -2.20787 |
| 226200_at | VARS2 | valyl-tRNA synthetase 2, mitochondrial (putative) | 0.0001399 | -2.20791 |
| 225733_at | B3GALT6 | UDP-Gal:betaGal beta 1,3-galactosyltransferase polypeptide 6 | 9.15E-06 | -2.20929 |
| 203729_at | EMP3 | epithelial membrane protein 3 | 4.55E-05 | -2.20954 |
| 209209_s_at | FERMT2 | fermitin family homolog 2 (Drosophila) | 0.000821 | -2.20985 |
| 203785_s_at | DDX28 | DEAD (Asp-Glu-Ala-Asp) box polypeptide 28 | 0.0013317 | -2.21036 |
| 228345_at | CHIC1 | cysteine-rich hydrophobic domain 1 | 4.55E-05 | -2.2116 |
| 213926_s_at | AGFG1 | ArfGAP with FG repeats 1 | 0.0006015 | -2.212 |
| 222688_at | ACER3 | alkaline ceramidase 3 | 0.0010605 | -2.21203 |
| 234979_at | BCDIN3D | BCDIN3 domain containing | 0.0003466 | -2.2121 |
| 201450_s_at | TIA1 | TIA1 cytotoxic granule-associated RNA binding protein | 0.0010815 | -2.21272 |
| 237215_s_at | TFRC | transferrin receptor (p90, CD71) | 8.96E-06 | -2.21278 |
| 1555351_s_at | PPHLN1 | periphilin 1 | 7.24E-06 | -2.21329 |
| 203921_at | CHST2 | carbohydrate (N-acetylglucosamine-6-O) sulfotransferase 2 | 0.0002965 | -2.21428 |
| 214620_x_at | PAM | peptidylglycine alpha-amidating monooxygenase | 0.0006292 | -2.21459 |
| 213845_at | GRIK2 | glutamate receptor, ionotropic, kainate 2 | 0.0003081 | -2.21462 |
| 218101_s_at | NDUFC2 | NADH dehydrogenase (ubiquinone) 1, subcomplex unknown, 2, 14.5kDa | 1.42E-08 | -2.21471 |
| 243772_at | SDCCAG8 | serologically defined colon cancer antigen 8 | 0.0007811 | -2.21474 |
| 217791_s_at | ALDH18A1 | aldehyde dehydrogenase 18 family, member A1 | 3.45E-05 | -2.2148 |
| 227166_at | DNAJC18 | DnaJ (Hsp40) homolog, subfamily C, member 18 | 0.0001403 | -2.21579 |
| 214795_at | ZMYND8 | zinc finger, MYND-type containing 8 | 0.0002288 | -2.21588 |
| 205733_at | BLM | Bloom syndrome, RecQ helicase-like | 3.66E-05 | -2.2159 |
| 206095_s_at | FUSIP1 | FUS interacting protein (serine/arginine-rich) 1 | 0.0001116 | -2.21604 |
| 223043_at | TMEM85 | transmembrane protein 85 | 6.39E-06 | -2.21638 |
| 215220_s_at | TPR | translocated promoter region (to activated MET oncogene) | 0.0017163 | -2.21695 |
| 206116_s_at | TPM1 | tropomyosin 1 (alpha) | 9.25E-05 | -2.21761 |
| 202607_at | NDST1 | N-deacetylase/N-sulfotransferase (heparan glucosaminyl) 1 | 0.0013211 | -2.21768 |
| 232480_at | LOC400931 | hypothetical LOC400931 | 0.0004386 | -2.21832 |
| 223248_at | HSDL1 | hydroxysteroid dehydrogenase like 1 | 4.39E-05 | -2.21853 |
| 218261_at | AP1M2 | adaptor-related protein complex 1, mu 2 subunit | 0.0008339 | -2.2188 |
| 204652_s_at | NRF1 | nuclear respiratory factor 1 | 0.0001731 | -2.21898 |
| 208442_s_at | ATM | ataxia telangiectasia mutated | 1.80E-05 | -2.2191 |
| 236976_at | FANCA | Fanconi anemia, complementation group A | 0.0010793 | -2.21934 |
| 220892_s_at | PSAT1 | phosphoserine aminotransferase 1 | 0.0014235 | -2.21967 |
| 217362_x_at | HLA-DRB6 | major histocompatibility complex, class II, DR beta 6 (pseudogene) | 0.0001417 | -2.21991 |
| 211504_x_at | ROCK2 | Rho-associated, coiled-coil containing protein kinase 2 | 1.43E-05 | -2.22017 |
| 226104_at | RNF170 | ring finger protein 170 | 0.0001029 | -2.22051 |
| 219340_s_at | CLN8 | ceroid-lipofuscinosis, neuronal 8 (epilepsy, progressive with mental retardation | 6.02E-05 | -2.22096 |
| 1553694_a_at | PIK3C2A | phosphoinositide-3-kinase, class 2, alpha polypeptide | 0.0002155 | -2.2211 |
| 225718_at | KIAA1715 | KIAA1715 | 4.18E-05 | -2.22134 |
| 220123_at | SLC35F5 | solute carrier family 35, member F5 | 0.0010678 | -2.22149 |
| 240449_at | ZNF341 | zinc finger protein 341 | 0.0005703 | -2.22273 |
| 243550_at | ZDHHC21 | zinc finger, DHHC-type containing 21 | 0.000193 | -2.22306 |
| 208127_s_at | SOCS5 | suppressor of cytokine signaling 5 | 0.0005886 | -2.22419 |
| 219262_at | SUV39H2 | suppressor of variegation 3-9 homolog 2 (Drosophila) | 0.0001422 | -2.22464 |
| 238609_at | C7orf38 | chromosome 7 open reading frame 38 | 0.0013116 | -2.22465 |
| 1569868_s_at | EME2 | essential meiotic endonuclease 1 homolog 2 (S. pombe) | 4.87E-05 | -2.22508 |
| 217419_x_at | AGRN | agrin | 2.56E-05 | -2.2254 |
| 229579_s_at | DISP2 | dispatched homolog 2 (Drosophila) | 0.000165 | -2.22568 |
| 221875_x_at | HLA-F | major histocompatibility complex, class I, F | 4.32E-05 | -2.22721 |
| 204143_s_at | ENOSF1 | enolase superfamily member 1 | 0.0009675 | -2.22853 |
| 201869_s_at | TBL1X | transducin (beta)-like 1X-linked | 0.0008654 | -2.22863 |
| 1554415_at | TAF5L | TAF5-like RNA polymerase II, p300/CBP-associated factor (PCAF)-associated factor | 7.72E-06 | -2.22876 |
| 1554606_at | CEP120 | centrosomal protein 120kDa | 0.0005944 | -2.22935 |
| 204808_s_at | TMEM5 | transmembrane protein 5 | 0.0001413 | -2.23011 |
| 1553244_at | FANCB | Fanconi anemia, complementation group B | 2.80E-05 | -2.23029 |
| 212021_s_at | MKI67 | antigen identified by monoclonal antibody Ki-67 | 0.0001188 | -2.2304 |
| 201215_at | PLS3 | plastin 3 (T isoform) | 2.55E-05 | -2.23043 |
| 201185_at | HTRA1 | HtrA serine peptidase 1 | 2.69E-05 | -2.2309 |
| 227968_at | PDDC1 | Parkinson disease 7 domain containing 1 | 2.49E-05 | -2.23103 |
| 1555783_x_at | PQLC2 | PQ loop repeat containing 2 | 0.0011432 | -2.23123 |
| 219074_at | TMEM184C | transmembrane protein 184C | 0.0008523 | -2.23149 |
| 232053_x_at | RHBDD2 | rhomboid domain containing 2 | 0.0004877 | -2.23169 |
| 202145_at | LY6E | lymphocyte antigen 6 complex, locus E | 2.90E-05 | -2.23301 |
| 215533_s_at | UBE4B | ubiquitination factor E4B (UFD2 homolog, yeast) | 0.0004332 | -2.23405 |
| 212811_x_at | SLC1A4 | solute carrier family 1 (glutamate/neutral amino acid transporter), member 4 | 7.13E-07 | -2.23448 |
| 210285_x_at | WTAP | Wilms tumor 1 associated protein | 0.0002212 | -2.23496 |
| 213638_at | PHACTR1 | phosphatase and actin regulator 1 | 7.81E-05 | -2.23525 |
| 228468_at | MASTL | microtubule associated serine/threonine kinase-like | 0.0002334 | -2.23527 |
| 223130_s_at | MYLIP | myosin regulatory light chain interacting protein | 3.10E-06 | -2.23568 |
| 1555679_a_at | RTN4IP1 | reticulon 4 interacting protein 1 | 0.0013064 | -2.23653 |
| 222846_at | RAB8B | RAB8B, member RAS oncogene family | 0.0010112 | -2.23857 |
| 211804_s_at | CDK2 | cyclin-dependent kinase 2 | 0.000344 | -2.23907 |
| 208812_x_at | HLA-C | major histocompatibility complex, class I, C | 9.34E-06 | -2.23919 |
| 219198_at | GTF3C4 | general transcription factor IIIC, polypeptide 4, 90kDa | 0.0001937 | -2.2392 |
| 223041_at | CD99L2 | CD99 molecule-like 2 | 3.03E-06 | -2.23988 |
| 217899_at | TMEM214 | transmembrane protein 214 | 0.0003934 | -2.24068 |
| 218726_at | HJURP | Holliday junction recognition protein | 0.0002214 | -2.24101 |
| 223176_at | KCTD20 | potassium channel tetramerisation domain containing 20 | 0.0003988 | -2.24154 |
| 218086_at | NPDC1 | neural proliferation, differentiation and control, 1 | 8.67E-05 | -2.24285 |
| 203804_s_at | LUC7L3 | LUC7-like 3 (S. cerevisiae) | 0.0010966 | -2.24432 |
| 238850_at | LOC645323 | hypothetical LOC645323 | 0.0005922 | -2.24439 |
| 206675_s_at | SKIL | SKI-like oncogene | 0.0005245 | -2.24512 |
| 236217_at | SLC31A1 | solute carrier family 31 (copper transporters), member 1 | 0.0003183 | -2.24524 |
| 210385_s_at | ERAP1 | endoplasmic reticulum aminopeptidase 1 | 6.28E-05 | -2.24557 |
| 223808_s_at | PTPMT1 | protein tyrosine phosphatase, mitochondrial 1 | 0.0004062 | -2.24563 |
| 221815_at | ABHD2 | abhydrolase domain containing 2 | 3.30E-05 | -2.2459 |
| 223577_x_at | MALAT1 | metastasis associated lung adenocarcinoma transcript 1 (non-protein coding) | 0.0002328 | -2.2482 |
| 239757_at | ZFAND6 | Zinc finger, AN1-type domain 6 | 0.0001088 | -2.24824 |
| 213528_at | C1orf156 | chromosome 1 open reading frame 156 | 3.59E-05 | -2.24831 |
| 218782_s_at | ATAD2 | ATPase family, AAA domain containing 2 | 0.0001779 | -2.24837 |
| 218703_at | SEC22A | SEC22 vesicle trafficking protein homolog A (S. cerevisiae) | 0.0003583 | -2.24844 |
| 1564175_at | LOC401074 | Hypothetical LOC401074 | 0.000314 | -2.24888 |
| 215258_at | CADM4 | cell adhesion molecule 4 | 5.21E-06 | -2.2489 |
| 210635_s_at | KLHL20 | kelch-like 20 (Drosophila) | 4.13E-05 | -2.24911 |
| 206704_at | CLCN5 | chloride channel 5 | 0.0001773 | -2.24913 |
| 229958_at | CLN8 | ceroid-lipofuscinosis, neuronal 8 (epilepsy, progressive with mental retardation | 0.0007806 | -2.25035 |
| 226726_at | MBOAT2 | membrane bound O-acyltransferase domain containing 2 | 0.0003865 | -2.25037 |
| 201029_s_at | CD99 | CD99 molecule | 5.10E-08 | -2.25041 |
| 203918_at | PCDH1 | protocadherin 1 | 0.0003785 | -2.25043 |
| 212067_s_at | C1R | complement component 1, r subcomponent | 4.06E-06 | -2.25124 |
| 223798_at | SLC41A2 | solute carrier family 41, member 2 | 0.0001169 | -2.25139 |
| 1555041_a_at | NAGA | N-acetylgalactosaminidase, alpha- | 0.0004092 | -2.25178 |
| 209490_s_at | PPT2 | palmitoyl-protein thioesterase 2 | 2.17E-05 | -2.25213 |
| 212509_s_at | MXRA7 | matrix-remodelling associated 7 | 1.98E-05 | -2.25215 |
| 230150_at | BCAP29 | B-cell receptor-associated protein 29 | 0.0010694 | -2.25228 |
| 211864_s_at | MYOF | myoferlin | 0.0004606 | -2.25278 |
| 211653_x_at | AKR1C2 | aldo-keto reductase family 1, member C2 (dihydrodiol dehydrogenase 2; bile acid | 7.82E-05 | -2.25293 |
| 215991_s_at | KIAA0090 | KIAA0090 | 0.0001036 | -2.25329 |
| 219687_at | HHAT | hedgehog acyltransferase | 0.0002378 | -2.25334 |
| 226541_at | FBXO30 | F-box protein 30 | 0.0001864 | -2.25346 |
| 207564_x_at | OGT | O-linked N-acetylglucosamine (GlcNAc) transferase (UDP-N-acetylglucosamine:polyp | 0.0002699 | -2.25346 |
| 202445_s_at | NOTCH2 | Notch homolog 2 (Drosophila) | 3.48E-06 | -2.25395 |
| 222468_at | KIAA0319L | KIAA0319-like | 0.0010512 | -2.25444 |
| 222147_s_at | ACTR5 | ARP5 actin-related protein 5 homolog (yeast) | 4.04E-05 | -2.25461 |
| 212322_at | SGPL1 | sphingosine-1-phosphate lyase 1 | 0.0001041 | -2.25521 |
| 228433_at | NFYA | nuclear transcription factor Y, alpha | 0.0001797 | -2.25547 |
| 204180_s_at | ZBTB43 | zinc finger and BTB domain containing 43 | 8.11E-06 | -2.25562 |
| 202295_s_at | CTSH | cathepsin H | 2.35E-05 | -2.2562 |
| 200954_at | ATP6V0C | ATPase, H+ transporting, lysosomal 16kDa, V0 subunit c | 1.20E-05 | -2.25655 |
| 209906_at | C3AR1 | complement component 3a receptor 1 | 0.0005645 | -2.25698 |
| 223187_s_at | ORMDL1 | ORM1-like 1 (S. cerevisiae) | 3.98E-06 | -2.25719 |
| 209305_s_at | GADD45B | growth arrest and DNA-damage-inducible, beta | 0.0007632 | -2.25796 |
| 205077_s_at | PIGF | phosphatidylinositol glycan anchor biosynthesis, class F | 1.02E-06 | -2.25801 |
| 220651_s_at | MCM10 | minichromosome maintenance complex component 10 | 0.0008442 | -2.25835 |
| 204215_at | C7orf23 | chromosome 7 open reading frame 23 | 2.09E-05 | -2.25864 |
| 1556053_at | DNAJC7 | DnaJ (Hsp40) homolog, subfamily C, member 7 | 0.0001122 | -2.2593 |
| 212394_at | KIAA0090 | KIAA0090 | 1.03E-05 | -2.2596 |
| 225140_at | KLF3 | Kruppel-like factor 3 (basic) | 0.0007804 | -2.26018 |
| 201631_s_at | IER3 | immediate early response 3 | 0.0001188 | -2.26021 |
| 205594_at | ZNF652 | zinc finger protein 652 | 0.0013325 | -2.26146 |
| 218683_at | PTBP2 | polypyrimidine tract binding protein 2 | 0.0001632 | -2.26238 |
| 235040_at | RUNDC1 | RUN domain containing 1 | 0.0001913 | -2.26317 |
| 1568126_at | ANXA2 | annexin A2 | 0.0003632 | -2.26348 |
| 209309_at | AZGP1 | alpha-2-glycoprotein 1, zinc-binding | 0.0005545 | -2.26353 |
| 213346_at | C13orf27 | chromosome 13 open reading frame 27 | 9.35E-05 | -2.2637 |
| 222741_s_at | C6orf64 | chromosome 6 open reading frame 64 | 0.0017659 | -2.26385 |
| 226308_at | HAUS8 | HAUS augmin-like complex, subunit 8 | 0.0003496 | -2.26398 |
| 243529_at | MARS2 | methionyl-tRNA synthetase 2, mitochondrial | 4.65E-05 | -2.26406 |
| 222645_s_at | KCTD5 | potassium channel tetramerisation domain containing 5 | 3.96E-05 | -2.26447 |
| 234725_s_at | SEMA4B | sema domain, immunoglobulin domain (Ig), transmembrane domain (TM) and short cyt | 1.33E-06 | -2.26477 |
| 202908_at | WFS1 | Wolfram syndrome 1 (wolframin) | 0.0001372 | -2.26568 |
| 202944_at | NAGA | N-acetylgalactosaminidase, alpha- | 0.0014687 | -2.26578 |
| 204937_s_at | ZNF274 | zinc finger protein 274 | 6.08E-06 | -2.26671 |
| 233564_s_at | CDADC1 | cytidine and dCMP deaminase domain containing 1 | 0.0003477 | -2.26672 |
| 235618_at | ZNF507 | zinc finger protein 507 | 0.00042 | -2.26697 |
| 204365_s_at | REEP1 | receptor accessory protein 1 | 5.14E-06 | -2.2672 |
| 235299_at | SLC41A2 | solute carrier family 41, member 2 | 0.0018211 | -2.26804 |
| 222786_at | CHST12 | carbohydrate (chondroitin 4) sulfotransferase 12 | 0.000668 | -2.26852 |
| 1552729_at | SNHG7 | small nucleolar RNA host gene 7 (non-protein coding) | 0.0005553 | -2.26861 |
| 231944_at | ERO1LB | ERO1-like beta (S. cerevisiae) | 7.32E-05 | -2.26879 |
| 201711_x_at | RANBP2 | RAN binding protein 2 | 0.0014016 | -2.26908 |
| 201647_s_at | SCARB2 | scavenger receptor class B, member 2 | 0.000183 | -2.26941 |
| 209604_s_at | GATA3 | GATA binding protein 3 | 0.0018377 | -2.26988 |
| 207149_at | CDH12 | cadherin 12, type 2 (N-cadherin 2) | 0.0006095 | -2.27013 |
| 235849_at | SCARA5 | scavenger receptor class A, member 5 (putative) | 0.000496 | -2.27104 |
| 225447_at | GPD2 | glycerol-3-phosphate dehydrogenase 2 (mitochondrial) | 0.0001321 | -2.27106 |
| 223459_s_at | C1orf56 | chromosome 1 open reading frame 56 | 0.000774 | -2.27136 |
| 205437_at | ZNF211 | zinc finger protein 211 | 4.67E-05 | -2.27213 |
| 219626_at | MAP7D3 | MAP7 domain containing 3 | 0.0017286 | -2.27213 |
| 1554930_a_at | FUT8 | fucosyltransferase 8 (alpha (1,6) fucosyltransferase) | 0.0013583 | -2.27229 |
| 242762_s_at | FAM171B | family with sequence similarity 171, member B | 0.0016213 | -2.27287 |
| 232149_s_at | NSMAF | neutral sphingomyelinase (N-SMase) activation associated factor | 0.0005411 | -2.27361 |
| 223258_s_at | G2E3 | G2/M-phase specific E3 ubiquitin ligase | 0.0004128 | -2.27368 |
| 213140_s_at | SS18L1 | synovial sarcoma translocation gene on chromosome 18-like 1 | 0.0001211 | -2.27498 |
| 228597_at | C21orf45 | chromosome 21 open reading frame 45 | 0.0013998 | -2.27539 |
| 219751_at | SETD6 | SET domain containing 6 | 0.000425 | -2.27617 |
| 223908_at | HDAC8 | histone deacetylase 8 | 2.88E-05 | -2.27652 |
| 1552682_a_at | CASC5 | cancer susceptibility candidate 5 | 0.0004689 | -2.27731 |
| 213971_s_at | LOC100292841 /// SUZ12 /// SUZ12P | similar to KIAA0160 /// suppressor of zeste 12 homolog (Drosophila) /// suppress | 0.0007117 | -2.27747 |
| 231403_at | TRIO | Triple functional domain (PTPRF interacting) | 0.001054 | -2.27798 |
| 226975_at | RNPC3 | RNA-binding region (RNP1, RRM) containing 3 | 0.0001649 | -2.27909 |
| 1558027_s_at | PRKAB2 | protein kinase, AMP-activated, beta 2 non-catalytic subunit | 0.0002944 | -2.27912 |
| 218151_x_at | GPR172A | G protein-coupled receptor 172A | 0.000517 | -2.27946 |
| 218340_s_at | UBA6 | ubiquitin-like modifier activating enzyme 6 | 0.0009332 | -2.27946 |
| 225071_at | NUS1 | nuclear undecaprenyl pyrophosphate synthase 1 homolog (S. cerevisiae) | 2.74E-05 | -2.28064 |
| 219615_s_at | KCNK5 | potassium channel, subfamily K, member 5 | 0.0011959 | -2.28076 |
| 231192_at | LPAR3 | lysophosphatidic acid receptor 3 | 0.0011223 | -2.28203 |
| 213274_s_at | CTSB | cathepsin B | 0.0001554 | -2.28228 |
| 226384_at | PPAPDC1B | phosphatidic acid phosphatase type 2 domain containing 1B | 7.46E-06 | -2.28314 |
| 1553749_at | FAM76B | family with sequence similarity 76, member B | 0.0007651 | -2.28389 |
| 217478_s_at | HLA-DMA /// HLA-DMB | major histocompatibility complex, class II, DM alpha /// major histocompatibilit | 0.0002702 | -2.284 |
| 218349_s_at | ZWILCH | Zwilch, kinetochore associated, homolog (Drosophila) | 0.000782 | -2.28406 |
| 222995_s_at | RHBDD2 | rhomboid domain containing 2 | 0.0001091 | -2.28432 |
| 222621_at | DNAJC1 | DnaJ (Hsp40) homolog, subfamily C, member 1 | 2.33E-06 | -2.28464 |
| 238756_at | GAS2L3 | Growth arrest-specific 2 like 3 | 0.0003926 | -2.28519 |
| 204877_s_at | TAOK2 | TAO kinase 2 | 0.0016088 | -2.28548 |
| 212311_at | KIAA0746 | KIAA0746 protein | 0.0004715 | -2.28582 |
| 1552531_a_at | NLRP11 | NLR family, pyrin domain containing 11 | 3.31E-05 | -2.28585 |
| 219663_s_at | TMEM121 | transmembrane protein 121 | 0.0014678 | -2.28632 |
| 213178_s_at | MAPK8IP3 | mitogen-activated protein kinase 8 interacting protein 3 | 0.000182 | -2.28738 |
| 203384_s_at | GOLGA1 | golgi autoantigen, golgin subfamily a, 1 | 1.25E-05 | -2.28869 |
| 239413_at | CEP152 | centrosomal protein 152kDa | 9.89E-05 | -2.2891 |
| 1554397_s_at | UEVLD | UEV and lactate/malate dehyrogenase domains | 0.0002985 | -2.28916 |
| 215873_x_at | ABCC10 | ATP-binding cassette, sub-family C (CFTR/MRP), member 10 | 0.000367 | -2.28916 |
| 218988_at | SLC35E3 | solute carrier family 35, member E3 | 8.42E-05 | -2.28976 |
| 206572_x_at | ZNF85 | zinc finger protein 85 | 0.0002932 | -2.2899 |
| 207826_s_at | ID3 | inhibitor of DNA binding 3, dominant negative helix-loop-helix protein | 0.0003196 | -2.29046 |
| 232101_s_at | PIGN | phosphatidylinositol glycan anchor biosynthesis, class N | 2.49E-05 | -2.29056 |
| 224628_at | ERLEC1 | endoplasmic reticulum lectin 1 | 1.75E-05 | -2.29074 |
| 223490_s_at | EXOSC3 | exosome component 3 | 9.58E-05 | -2.29075 |
| 229863_s_at | C3orf75 | chromosome 3 open reading frame 75 | 0.00034 | -2.29089 |
| 205909_at | POLE2 | polymerase (DNA directed), epsilon 2 (p59 subunit) | 0.0018735 | -2.29108 |
| 213244_at | SCAMP4 | secretory carrier membrane protein 4 | 0.0012316 | -2.2918 |
| 1555384_a_at | LARP4 | La ribonucleoprotein domain family, member 4 | 7.13E-05 | -2.29265 |
| 203227_s_at | TSPAN31 | tetraspanin 31 | 0.0002987 | -2.29374 |
| 232881_at | GNASAS | GNAS antisense RNA (non-protein coding) | 0.0002317 | -2.29411 |
| 203679_at | TMED1 | transmembrane emp24 protein transport domain containing 1 | 9.12E-06 | -2.29448 |
| 223489_x_at | EXOSC3 | exosome component 3 | 0.0003343 | -2.29449 |
| 202586_at | POLR2L | polymerase (RNA) II (DNA directed) polypeptide L, 7.6kDa | 0.0002267 | -2.29451 |
| 226524_at | C3orf38 | chromosome 3 open reading frame 38 | 0.0002614 | -2.29531 |
| 201389_at | ITGA5 | integrin, alpha 5 (fibronectin receptor, alpha polypeptide) | 3.82E-06 | -2.2954 |
| 216962_at | RPAIN | RPA interacting protein | 7.38E-05 | -2.2956 |
| 212671_s_at | HLA-DQA1 /// HLA-DQA2 /// LOC100294224 /// LOC100294317 | major histocompatibility complex, class II, DQ alpha 1 /// major histocompatibil | 0.0003617 | -2.29611 |
| 224435_at | C10orf58 | chromosome 10 open reading frame 58 | 6.67E-06 | -2.29624 |
| 213296_at | RER1 | RER1 retention in endoplasmic reticulum 1 homolog (S. cerevisiae) | 1.63E-05 | -2.29628 |
| 202316_x_at | UBE4B | ubiquitination factor E4B (UFD2 homolog, yeast) | 0.0001117 | -2.29827 |
| 205729_at | OSMR | oncostatin M receptor | 2.22E-05 | -2.29838 |
| 217900_at | IARS2 | isoleucyl-tRNA synthetase 2, mitochondrial | 1.74E-05 | -2.2986 |
| 219133_at | OXSM | 3-oxoacyl-ACP synthase, mitochondrial | 0.0003971 | -2.29875 |
| 1569190_at | SCLT1 | sodium channel and clathrin linker 1 | 7.60E-06 | -2.2992 |
| 218947_s_at | MTPAP | mitochondrial poly(A) polymerase | 0.0003095 | -2.30138 |
| 200796_s_at | MCL1 | myeloid cell leukemia sequence 1 (BCL2-related) | 0.0009467 | -2.30157 |
| 219254_at | C17orf101 | chromosome 17 open reading frame 101 | 0.0003364 | -2.30173 |
| 202562_s_at | C14orf1 | chromosome 14 open reading frame 1 | 1.82E-06 | -2.30271 |
| 53071_s_at | C17orf101 | chromosome 17 open reading frame 101 | 6.61E-05 | -2.30297 |
| 207338_s_at | ZNF200 | zinc finger protein 200 | 0.0001044 | -2.3034 |
| 226859_at | DNAJC25 | DnaJ (Hsp40) homolog, subfamily C , member 25 | 5.68E-05 | -2.30384 |
| 232921_at | KIAA1549 | KIAA1549 | 8.16E-05 | -2.30509 |
| 236657_at | LOC100288911 | hypothetical protein LOC100288911 | 0.0001091 | -2.30665 |
| 228904_at | HOXB3 | homeobox B3 | 0.0009087 | -2.30846 |
| 1554321_a_at | CPNE1 /// NFS1 | copine I /// NFS1 nitrogen fixation 1 homolog (S. cerevisiae) | 7.91E-05 | -2.30866 |
| 205702_at | PHTF1 | putative homeodomain transcription factor 1 | 0.0008222 | -2.30894 |
| 220012_at | ERO1LB | ERO1-like beta (S. cerevisiae) | 0.000814 | -2.31048 |
| 205358_at | GRIA2 | glutamate receptor, ionotropic, AMPA 2 | 7.54E-05 | -2.31062 |
| 1552295_a_at | SLC39A13 | solute carrier family 39 (zinc transporter), member 13 | 0.00012 | -2.31093 |
| 1554105_at | TMEM185A | transmembrane protein 185A | 8.61E-05 | -2.31236 |
| 204125_at | NDUFAF1 | NADH dehydrogenase (ubiquinone) 1 alpha subcomplex, assembly factor 1 | 1.51E-05 | -2.31269 |
| 216915_s_at | PTPN12 | protein tyrosine phosphatase, non-receptor type 12 | 9.45E-06 | -2.31309 |
| 223079_s_at | GLS | glutaminase | 0.0003087 | -2.31329 |
| 214500_at | H2AFY | H2A histone family, member Y | 0.0008614 | -2.31356 |
| 213046_at | PABPN1 | poly(A) binding protein, nuclear 1 | 3.67E-05 | -2.31401 |
| 204105_s_at | NRCAM | neuronal cell adhesion molecule | 0.0005312 | -2.3143 |
| 215942_s_at | GTSE1 | G-2 and S-phase expressed 1 | 0.0001035 | -2.31449 |
| 217596_at | UPF3A | UPF3 regulator of nonsense transcripts homolog A (yeast) | 0.0002183 | -2.31455 |
| 222074_at | UROD | uroporphyrinogen decarboxylase | 0.0009314 | -2.31457 |
| 207610_s_at | EMR2 | egf-like module containing, mucin-like, hormone receptor-like 2 | 0.0003807 | -2.3146 |
| 1554277_s_at | FANCM | Fanconi anemia, complementation group M | 0.0013667 | -2.31485 |
| 219895_at | FAM70A | family with sequence similarity 70, member A | 0.0008701 | -2.31514 |
| 211799_x_at | HLA-C | major histocompatibility complex, class I, C | 0.0001005 | -2.31576 |
| 216855_s_at | HNRNPU | heterogeneous nuclear ribonucleoprotein U (scaffold attachment factor A) | 0.0011435 | -2.31576 |
| 227367_at | SLCO3A1 | solute carrier organic anion transporter family, member 3A1 | 0.0007246 | -2.31587 |
| 202118_s_at | CPNE3 | copine III | 0.0015618 | -2.31593 |
| 208963_x_at | FADS1 | fatty acid desaturase 1 | 0.0001663 | -2.31642 |
| 205543_at | HSPA4L | heat shock 70kDa protein 4-like | 9.33E-05 | -2.31708 |
| 237116_at | LOC646903 | hypothetical LOC646903 | 0.0005458 | -2.31763 |
| 36545_s_at | SFI1 | Sfi1 homolog, spindle assembly associated (yeast) | 6.27E-05 | -2.31864 |
| 207724_s_at | SPAST | spastin | 3.30E-05 | -2.31905 |
| 217540_at | FAM55C | family with sequence similarity 55, member C | 0.0001963 | -2.31982 |
| 222037_at | MCM4 | minichromosome maintenance complex component 4 | 0.0003719 | -2.321 |
| 227455_at | C6orf136 | chromosome 6 open reading frame 136 | 0.0006772 | -2.32246 |
| 1557384_at | ZNF131 | Zinc finger protein 131 | 0.0015525 | -2.32275 |
| 1557137_at | TMEM17 | transmembrane protein 17 | 0.000286 | -2.32556 |
| 241360_at | CCDC15 | coiled-coil domain containing 15 | 0.0002985 | -2.32667 |
| 226469_s_at | GGT7 | gamma-glutamyltransferase 7 | 1.51E-05 | -2.32723 |
| 213269_at | ZNF248 | zinc finger protein 248 | 0.000295 | -2.32752 |
| 223909_s_at | HDAC8 | histone deacetylase 8 | 3.17E-06 | -2.32757 |
| 202783_at | NNT | nicotinamide nucleotide transhydrogenase | 8.60E-05 | -2.32813 |
| 212341_at | YIPF6 | Yip1 domain family, member 6 | 1.11E-05 | -2.32834 |
| 213075_at | OLFML2A | olfactomedin-like 2A | 6.47E-05 | -2.32868 |
| 241666_at | C3orf23 | chromosome 3 open reading frame 23 | 1.99E-05 | -2.32875 |
| 209743_s_at | ITCH | itchy E3 ubiquitin protein ligase homolog (mouse) | 9.86E-05 | -2.32949 |
| 219474_at | C3orf52 | chromosome 3 open reading frame 52 | 0.0003555 | -2.32956 |
| 218608_at | ATP13A2 | ATPase type 13A2 | 0.0001098 | -2.32966 |
| 1552634_a_at | ZNF101 | zinc finger protein 101 | 0.0003677 | -2.33072 |
| 202204_s_at | AMFR | autocrine motility factor receptor | 0.0002191 | -2.33158 |
| 218162_at | OLFML3 | olfactomedin-like 3 | 0.001607 | -2.33239 |
| 201490_s_at | PPIF | peptidylprolyl isomerase F | 7.90E-05 | -2.33289 |
| 222201_s_at | CASP8AP2 | caspase 8 associated protein 2 | 0.0004724 | -2.33317 |
| 211488_s_at | ITGB8 | integrin, beta 8 | 0.0006173 | -2.33355 |
| 206748_s_at | SPAG9 | sperm associated antigen 9 | 1.35E-05 | -2.33384 |
| 204373_s_at | CEP350 | centrosomal protein 350kDa | 0.0005396 | -2.33389 |
| 229033_s_at | MUM1 | melanoma associated antigen (mutated) 1 | 1.52E-05 | -2.33441 |
| 227083_at | B3GALTL | beta 1,3-galactosyltransferase-like | 0.0003946 | -2.33531 |
| 204262_s_at | PSEN2 | presenilin 2 (Alzheimer disease 4) | 0.0001618 | -2.33632 |
| 222833_at | LPCAT2 | lysophosphatidylcholine acyltransferase 2 | 0.0007154 | -2.33643 |
| 230021_at | C15orf42 | chromosome 15 open reading frame 42 | 0.0012136 | -2.33644 |
| 201723_s_at | GALNT1 | UDP-N-acetyl-alpha-D-galactosamine:polypeptide N-acetylgalactosaminyltransferase | 5.23E-05 | -2.33673 |
| 225125_at | MMGT1 | membrane magnesium transporter 1 | 1.26E-05 | -2.33802 |
| 218727_at | SLC38A7 | solute carrier family 38, member 7 | 9.29E-05 | -2.33835 |
| 231822_at | CTTNBP2NL | CTTNBP2 N-terminal like | 0.0003259 | -2.33869 |
| 209016_s_at | KRT7 | keratin 7 | 0.0013423 | -2.3387 |
| 210688_s_at | CPT1A | carnitine palmitoyltransferase 1A (liver) | 0.0008637 | -2.33878 |
| 208793_x_at | SMARCA4 | SWI/SNF related, matrix associated, actin dependent regulator of chromatin, subf | 0.0004688 | -2.33882 |
| 205209_at | ACVR1B | activin A receptor, type IB | 0.0004458 | -2.33895 |
| 204603_at | EXO1 | exonuclease 1 | 0.0002582 | -2.33902 |
| 207740_s_at | NUP62 | nucleoporin 62kDa | 0.0002066 | -2.33912 |
| 210859_x_at | CLN3 | ceroid-lipofuscinosis, neuronal 3 | 0.0004308 | -2.34018 |
| 225992_at | MLLT10 | myeloid/lymphoid or mixed-lineage leukemia (trithorax homolog, Drosophila); tran | 0.0003609 | -2.34036 |
| 226130_at | RPS16 | ribosomal protein S16 | 1.47E-05 | -2.34286 |
| 212822_at | HEG1 | HEG homolog 1 (zebrafish) | 0.0010223 | -2.34351 |
| 209610_s_at | SLC1A4 | solute carrier family 1 (glutamate/neutral amino acid transporter), member 4 | 0.00018 | -2.34405 |
| 212815_at | ASCC3 | activating signal cointegrator 1 complex subunit 3 | 0.0014068 | -2.34451 |
| 213584_s_at | CREBZF | CREB/ATF bZIP transcription factor | 5.45E-05 | -2.34552 |
| 227610_at | TSPAN11 | tetraspanin 11 | 2.15E-05 | -2.34601 |
| 1555923_a_at | C10orf114 | chromosome 10 open reading frame 114 | 4.67E-05 | -2.34664 |
| 212807_s_at | SORT1 | sortilin 1 | 3.73E-05 | -2.34689 |
| 204739_at | CENPC1 | centromere protein C 1 | 0.0008447 | -2.34726 |
| 225127_at | TMEM181 | transmembrane protein 181 | 1.66E-05 | -2.34765 |
| 1552678_a_at | USP28 | ubiquitin specific peptidase 28 | 0.0001999 | -2.34834 |
| 228574_at | TMTC2 | Transmembrane and tetratricopeptide repeat containing 2 | 0.0012065 | -2.34915 |
| 232154_at | LOC199800 | hypothetical protein LOC199800 | 0.0005433 | -2.35005 |
| 242996_at | MTRF1 | mitochondrial translational release factor 1 | 0.0006935 | -2.35039 |
| 201747_s_at | SAFB | scaffold attachment factor B | 2.13E-05 | -2.35089 |
| 224218_s_at | TRPS1 | trichorhinophalangeal syndrome I | 1.04E-05 | -2.3512 |
| 1560402_at | GAS5 | growth arrest-specific 5 (non-protein coding) | 0.0008409 | -2.35159 |
| 212966_at | HIC2 | hypermethylated in cancer 2 | 0.0011581 | -2.35354 |
| 219311_at | CEP76 | centrosomal protein 76kDa | 0.0012919 | -2.35405 |
| 203932_at | HLA-DMB | major histocompatibility complex, class II, DM beta | 2.07E-05 | -2.35416 |
| 214959_s_at | API5 | apoptosis inhibitor 5 | 6.32E-05 | -2.35434 |
| 218855_at | TPRA1 | transmembrane protein, adipocyte asscociated 1 | 9.35E-05 | -2.35481 |
| 218532_s_at | FAM134B | family with sequence similarity 134, member B | 0.0004277 | -2.35574 |
| 212538_at | DOCK9 | dedicator of cytokinesis 9 | 0.0008409 | -2.35582 |
| 214866_at | PLAUR | plasminogen activator, urokinase receptor | 1.82E-05 | -2.35595 |
| 231861_at | LRP10 | low density lipoprotein receptor-related protein 10 | 0.0002952 | -2.35606 |
| 223642_at | ZIC2 | Zic family member 2 (odd-paired homolog, Drosophila) | 0.0001049 | -2.35625 |
| 214116_at | BTD | biotinidase | 0.0001278 | -2.35638 |
| 221450_x_at | PCDHB13 | protocadherin beta 13 | 0.0002126 | -2.35677 |
| 234982_at | UBR3 | ubiquitin protein ligase E3 component n-recognin 3 (putative) | 0.0002564 | -2.35795 |
| 203637_s_at | MID1 | midline 1 (Opitz/BBB syndrome) | 0.0006825 | -2.35816 |
| 226666_at | DAAM1 | dishevelled associated activator of morphogenesis 1 | 0.0003741 | -2.35838 |
| 239433_at | LRRC8E | leucine rich repeat containing 8 family, member E | 3.91E-05 | -2.3589 |
| 211256_x_at | BTN2A1 | butyrophilin, subfamily 2, member A1 | 8.57E-05 | -2.35906 |
| 228930_at | SCARNA15 | Small Cajal body-specific RNA 15 | 0.0006 | -2.35918 |
| 229091_s_at | CCNJ | cyclin J | 0.000886 | -2.36009 |
| 204453_at | ZNF84 | zinc finger protein 84 | 0.0011401 | -2.3605 |
| 212327_at | LIMCH1 | LIM and calponin homology domains 1 | 0.0006829 | -2.3616 |
| 1552344_s_at | CNOT7 | CCR4-NOT transcription complex, subunit 7 | 4.09E-05 | -2.36219 |
| 218718_at | PDGFC | platelet derived growth factor C | 0.0001029 | -2.36282 |
| 210448_s_at | P2RX5 | purinergic receptor P2X, ligand-gated ion channel, 5 | 0.0005347 | -2.36344 |
| 210561_s_at | WSB1 | WD repeat and SOCS box-containing 1 | 6.39E-05 | -2.36509 |
| 37408_at | MRC2 | mannose receptor, C type 2 | 1.08E-06 | -2.36548 |
| 218640_s_at | PLEKHF2 | pleckstrin homology domain containing, family F (with FYVE domain) member 2 | 0.0010467 | -2.36581 |
| 204981_at | SLC22A18 | solute carrier family 22, member 18 | 0.0001588 | -2.36589 |
| 203015_s_at | SSX2IP | synovial sarcoma, X breakpoint 2 interacting protein | 0.000908 | -2.36607 |
| 209797_at | CNPY2 | canopy 2 homolog (zebrafish) | 1.61E-05 | -2.36639 |
| 217859_s_at | SLC39A9 | solute carrier family 39 (zinc transporter), member 9 | 0.0002896 | -2.36761 |
| 219885_at | SLFN12 | schlafen family member 12 | 0.0003355 | -2.36817 |
| 226743_at | SLFN11 | schlafen family member 11 | 0.0001713 | -2.36872 |
| 229399_at | C10orf118 | chromosome 10 open reading frame 118 | 0.0014582 | -2.36968 |
| 238960_s_at | LARP4 | La ribonucleoprotein domain family, member 4 | 0.0010964 | -2.37009 |
| 217785_s_at | YKT6 | YKT6 v-SNARE homolog (S. cerevisiae) | 0.0003515 | -2.37119 |
| AFFX-HUMISGF3A/M97935_MB_at | STAT1 | signal transducer and activator of transcription 1, 91kDa | 0.0010156 | -2.37119 |
| 219531_at | CEP72 | centrosomal protein 72kDa | 3.74E-05 | -2.37173 |
| 227896_at | BCCIP | BRCA2 and CDKN1A interacting protein | 0.0001459 | -2.37173 |
| 222816_s_at | ZCCHC2 | zinc finger, CCHC domain containing 2 | 0.0001648 | -2.37258 |
| 210240_s_at | CDKN2D | cyclin-dependent kinase inhibitor 2D (p19, inhibits CDK4) | 0.0004086 | -2.37286 |
| 206288_at | PGGT1B | protein geranylgeranyltransferase type I, beta subunit | 0.0005172 | -2.37328 |
| 223306_at | EBPL | emopamil binding protein-like | 1.61E-05 | -2.37392 |
| 226404_at | LOC643167 /// RBM39 | similar to RNA binding motif protein 39 /// RNA binding motif protein 39 | 6.90E-05 | -2.37398 |
| 1559993_at | SFXN3 | Sideroflexin 3 | 0.000674 | -2.37435 |
| 235030_at | FAM55C | family with sequence similarity 55, member C | 0.0001166 | -2.37464 |
| 1554029_a_at | TTC37 | tetratricopeptide repeat domain 37 | 0.0016216 | -2.37484 |
| 228820_at | XPNPEP3 | X-prolyl aminopeptidase (aminopeptidase P) 3, putative | 0.0007765 | -2.3757 |
| 228654_at | SPIN4 | spindlin family, member 4 | 0.0009782 | -2.3758 |
| 204579_at | FGFR4 | fibroblast growth factor receptor 4 | 0.0003031 | -2.37605 |
| 218514_at | C17orf71 | chromosome 17 open reading frame 71 | 0.0002722 | -2.37609 |
| 233208_x_at | CPSF2 | cleavage and polyadenylation specific factor 2, 100kDa | 0.0006615 | -2.37622 |
| 1554249_a_at | ZNF638 | zinc finger protein 638 | 0.0002931 | -2.37725 |
| 218684_at | LRRC8D | leucine rich repeat containing 8 family, member D | 0.0008958 | -2.37768 |
| 226981_at | MLL | Myeloid/lymphoid or mixed-lineage leukemia (trithorax homolog, Drosophila) | 0.0001908 | -2.3777 |
| 224716_at | SLC35B2 | solute carrier family 35, member B2 | 1.70E-05 | -2.37784 |
| 218344_s_at | RCOR3 | REST corepressor 3 | 0.0001367 | -2.37831 |
| 210861_s_at | WISP3 | WNT1 inducible signaling pathway protein 3 | 7.47E-05 | -2.37867 |
| 229715_at | DKFZp686O24166 | Hypothetical protein DKFZp686O24166 | 0.0001784 | -2.379 |
| 235142_at | ZBTB8A | zinc finger and BTB domain containing 8A | 0.0005009 | -2.37928 |
| 201688_s_at | TPD52 | tumor protein D52 | 3.47E-05 | -2.38007 |
| 223494_at | MGEA5 | meningioma expressed antigen 5 (hyaluronidase) | 0.0002093 | -2.38167 |
| 221432_s_at | SLC25A28 | solute carrier family 25, member 28 | 0.0004614 | -2.38342 |
| 235314_at | RPL32P3 | ribosomal protein L32 pseudogene 3 | 0.0003604 | -2.38441 |
| 201939_at | PLK2 | polo-like kinase 2 (Drosophila) | 4.50E-05 | -2.38448 |
| 200800_s_at | HSPA1A /// HSPA1B | heat shock 70kDa protein 1A /// heat shock 70kDa protein 1B | 4.95E-07 | -2.38456 |
| 214875_x_at | APLP2 | amyloid beta (A4) precursor-like protein 2 | 0.0010023 | -2.38498 |
| 203229_s_at | CLK2 | CDC-like kinase 2 | 0.0015777 | -2.38588 |
| 222212_s_at | LASS2 | LAG1 homolog, ceramide synthase 2 | 5.89E-05 | -2.38611 |
| 226112_at | SGCB | sarcoglycan, beta (43kDa dystrophin-associated glycoprotein) | 3.73E-05 | -2.38664 |
| 227444_at | ARMCX4 | Armadillo repeat containing, X-linked 4 | 0.000182 | -2.38687 |
| 221640_s_at | LRDD | leucine-rich repeats and death domain containing | 5.14E-05 | -2.38717 |
| 205498_at | GHR | growth hormone receptor | 6.42E-05 | -2.38749 |
| 212384_at | BAT1 | HLA-B associated transcript 1 | 0.0004795 | -2.38942 |
| 209709_s_at | HMMR | hyaluronan-mediated motility receptor (RHAMM) | 3.85E-05 | -2.38976 |
| 232774_x_at | ZIK1 | zinc finger protein interacting with K protein 1 homolog (mouse) | 1.93E-05 | -2.38996 |
| 204732_s_at | TRIM23 | tripartite motif-containing 23 | 0.0004664 | -2.39018 |
| 203650_at | PROCR | protein C receptor, endothelial (EPCR) | 2.37E-05 | -2.39032 |
| 204222_s_at | GLIPR1 | GLI pathogenesis-related 1 | 0.0003403 | -2.39066 |
| 232816_s_at | DDX11 | DEAD/H (Asp-Glu-Ala-Asp/His) box polypeptide 11 (CHL1-like helicase homolog, S. | 0.000916 | -2.39145 |
| 235085_at | PRAGMIN | homolog of rat pragma of Rnd2 | 0.0013718 | -2.39314 |
| 216969_s_at | KIF22 | kinesin family member 22 | 0.0003297 | -2.39497 |
| 221473_x_at | SERINC3 | serine incorporator 3 | 0.0006358 | -2.39522 |
| 216468_s_at | ZNF682 | zinc finger protein 682 | 0.0007214 | -2.3958 |
| 219470_x_at | CCNJ | cyclin J | 0.0002827 | -2.39593 |
| 206613_s_at | TAF1A | TATA box binding protein (TBP)-associated factor, RNA polymerase I, A, 48kDa | 6.88E-05 | -2.39674 |
| 231927_at | ATF6 | Activating transcription factor 6 | 0.0006776 | -2.39698 |
| 213712_at | ELOVL2 | elongation of very long chain fatty acids (FEN1/Elo2, SUR4/Elo3, yeast)-like 2 | 0.0001134 | -2.3973 |
| 238959_at | LARP4 | La ribonucleoprotein domain family, member 4 | 0.0005277 | -2.39732 |
| 244743_x_at | ZNF138 | zinc finger protein 138 | 0.0016537 | -2.39762 |
| 224669_at | SYS1 | SYS1 Golgi-localized integral membrane protein homolog (S. cerevisiae) | 0.000299 | -2.39808 |
| 213670_x_at | NSUN5B | NOL1/NOP2/Sun domain family, member 5B | 0.001206 | -2.39827 |
| 220477_s_at | C20orf30 | chromosome 20 open reading frame 30 | 0.000173 | -2.39849 |
| 238333_s_at | MTG1 | Mitochondrial GTPase 1 homolog (S. cerevisiae) | 1.14E-05 | -2.40124 |
| 226314_at | CHST14 | carbohydrate (N-acetylgalactosamine 4-0) sulfotransferase 14 | 6.29E-05 | -2.40127 |
| 215537_x_at | DDAH2 | dimethylarginine dimethylaminohydrolase 2 | 0.0012459 | -2.40209 |
| 219901_at | FGD6 | FYVE, RhoGEF and PH domain containing 6 | 0.0003572 | -2.40257 |
| 222781_s_at | C9orf40 | chromosome 9 open reading frame 40 | 1.52E-05 | -2.40287 |
| 223352_s_at | C17orf80 | chromosome 17 open reading frame 80 | 0.000268 | -2.40428 |
| 224314_s_at | EGLN1 | egl nine homolog 1 (C. elegans) | 1.58E-06 | -2.40478 |
| 211432_s_at | TYRO3 | TYRO3 protein tyrosine kinase | 0.0011554 | -2.405 |
| 232529_at | SP3 | Sp3 transcription factor | 0.000167 | -2.40514 |
| 227199_at | DIP2A | DIP2 disco-interacting protein 2 homolog A (Drosophila) | 0.0015348 | -2.40607 |
| 228251_at | UBXN6 | UBX domain protein 6 | 0.0003155 | -2.40621 |
| 212279_at | TMEM97 | transmembrane protein 97 | 0.0001496 | -2.40667 |
| 1553691_at | B3GALNT2 | beta-1,3-N-acetylgalactosaminyltransferase 2 | 1.38E-05 | -2.4069 |
| 202023_at | EFNA1 | ephrin-A1 | 2.31E-05 | -2.40815 |
| 217790_s_at | SSR3 | signal sequence receptor, gamma (translocon-associated protein gamma) | 0.0001437 | -2.4087 |
| 227650_at | HSPA14 | heat shock 70kDa protein 14 | 0.0001696 | -2.40876 |
| 1555745_a_at | LYZ | lysozyme (renal amyloidosis) | 0.0011382 | -2.40903 |
| 211911_x_at | HLA-B | major histocompatibility complex, class I, B | 3.67E-05 | -2.40965 |
| 202032_s_at | MAN2A2 | mannosidase, alpha, class 2A, member 2 | 0.0001653 | -2.4097 |
| 203685_at | BCL2 | B-cell CLL/lymphoma 2 | 3.43E-05 | -2.41024 |
| 203636_at | MID1 | midline 1 (Opitz/BBB syndrome) | 6.15E-08 | -2.41156 |
| 222267_at | TMEM209 | transmembrane protein 209 | 0.0010775 | -2.4122 |
| 228696_at | SLC45A3 | solute carrier family 45, member 3 | 0.0001644 | -2.41243 |
| 204312_x_at | CREB1 | cAMP responsive element binding protein 1 | 4.86E-05 | -2.4125 |
| 226679_at | SLC26A11 | solute carrier family 26, member 11 | 0.0012615 | -2.41255 |
| 236649_at | DTWD1 | DTW domain containing 1 | 0.0003687 | -2.41262 |
| 1555789_s_at | PHF23 | PHD finger protein 23 | 0.000444 | -2.41397 |
| 222811_at | FTSJD1 | FtsJ methyltransferase domain containing 1 | 0.0004982 | -2.41432 |
| 206163_at | MAB21L1 | mab-21-like 1 (C. elegans) | 0.0008136 | -2.41511 |
| 216870_x_at | DLEU2 | deleted in lymphocytic leukemia 2 (non-protein coding) | 0.0001131 | -2.41538 |
| 210425_x_at | GOLGA8B | golgi autoantigen, golgin subfamily a, 8B | 0.0008185 | -2.4156 |
| 218614_at | C12orf35 | chromosome 12 open reading frame 35 | 0.0001989 | -2.41595 |
| 227019_at | C1orf226 | chromosome 1 open reading frame 226 | 0.0009252 | -2.416 |
| 218368_s_at | TNFRSF12A | tumor necrosis factor receptor superfamily, member 12A | 6.04E-05 | -2.41616 |
| 228523_at | NANOS1 | nanos homolog 1 (Drosophila) | 5.84E-05 | -2.41709 |
| 211810_s_at | GALC | galactosylceramidase | 3.09E-06 | -2.41727 |
| 212178_s_at | POM121 /// POM121C | POM121 membrane glycoprotein (rat) /// POM121 membrane glycoprotein C | 0.000723 | -2.41825 |
| 204899_s_at | SAP30 | Sin3A-associated protein, 30kDa | 5.64E-06 | -2.41899 |
| 214719_at | SLC46A3 | solute carrier family 46, member 3 | 0.0001814 | -2.41914 |
| 1553167_a_at | SEPSECS | Sep (O-phosphoserine) tRNA:Sec (selenocysteine) tRNA synthase | 0.0005565 | -2.42017 |
| 209773_s_at | RRM2 | ribonucleotide reductase M2 | 3.31E-06 | -2.42046 |
| 204490_s_at | CD44 | CD44 molecule (Indian blood group) | 1.20E-06 | -2.42066 |
| 214247_s_at | DKK3 | dickkopf homolog 3 (Xenopus laevis) | 0.0001959 | -2.42077 |
| 219247_s_at | ZDHHC14 | zinc finger, DHHC-type containing 14 | 0.0018279 | -2.42077 |
| 228089_x_at | TMEM179B | transmembrane protein 179B | 5.54E-06 | -2.42192 |
| 205089_at | ZNF7 | zinc finger protein 7 | 1.34E-05 | -2.42218 |
| 233030_at | PNPLA3 | patatin-like phospholipase domain containing 3 | 1.04E-05 | -2.42334 |
| 224953_at | YIPF5 | Yip1 domain family, member 5 | 8.41E-05 | -2.4241 |
| 244804_at | SQSTM1 | sequestosome 1 | 0.0008158 | -2.42553 |
| 227150_at | MTF1 | metal-regulatory transcription factor 1 | 1.82E-06 | -2.42562 |
| 208704_x_at | APLP2 | amyloid beta (A4) precursor-like protein 2 | 1.56E-05 | -2.42572 |
| 225711_at | ARL6IP6 | ADP-ribosylation-like factor 6 interacting protein 6 | 0.0001341 | -2.42599 |
| 206071_s_at | EPHA3 | EPH receptor A3 | 1.58E-05 | -2.42643 |
| 210214_s_at | BMPR2 | bone morphogenetic protein receptor, type II (serine/threonine kinase) | 0.000349 | -2.42675 |
| 1554132_a_at | FAM190B | family with sequence similarity 190, member B | 0.0012125 | -2.42681 |
| 233814_at | EFNA5 | ephrin-A5 | 0.0014868 | -2.42682 |
| 224002_s_at | FKBP7 | FK506 binding protein 7 | 0.0007793 | -2.4271 |
| 218548_x_at | TEX264 | testis expressed 264 | 5.61E-06 | -2.42864 |
| 1554417_s_at | APH1A | anterior pharynx defective 1 homolog A (C. elegans) | 0.0004476 | -2.42893 |
| 218159_at | DDRGK1 | DDRGK domain containing 1 | 0.0003108 | -2.42947 |
| 230464_at | S1PR5 | sphingosine-1-phosphate receptor 5 | 8.87E-05 | -2.42992 |
| 213326_at | VAMP1 | vesicle-associated membrane protein 1 (synaptobrevin 1) | 0.0007085 | -2.43108 |
| 242028_at | ZNF709 | zinc finger protein 709 | 0.0004426 | -2.43115 |
| 1553292_s_at | FLJ25006 | uncharacterized serine/threonine-protein kinase SgK494 | 3.50E-05 | -2.43119 |
| 229533_x_at | ZNF680 | zinc finger protein 680 | 9.43E-05 | -2.43148 |
| 228859_at | C4orf21 | chromosome 4 open reading frame 21 | 0.0011538 | -2.43188 |
| 1556613_s_at | LOC203107 | hypothetical protein LOC203107 | 8.95E-06 | -2.43208 |
| 213517_at | PCBP2 | poly(rC) binding protein 2 | 0.0006315 | -2.43233 |
| 209112_at | CDKN1B | cyclin-dependent kinase inhibitor 1B (p27, Kip1) | 9.36E-05 | -2.43247 |
| 225731_at | ANKRD50 | ankyrin repeat domain 50 | 0.0006673 | -2.43371 |
| 201447_at | TIA1 | TIA1 cytotoxic granule-associated RNA binding protein | 0.0009614 | -2.434 |
| 218656_s_at | LHFP | lipoma HMGIC fusion partner | 2.11E-05 | -2.43461 |
| 227338_at | LOC440983 | hypothetical gene supported by BC066916 | 0.0001735 | -2.43463 |
| 204774_at | EVI2A | ecotropic viral integration site 2A | 5.03E-05 | -2.43467 |
| 203085_s_at | TGFB1 | transforming growth factor, beta 1 | 7.27E-05 | -2.43509 |
| 218979_at | RMI1 | RMI1, RecQ mediated genome instability 1, homolog (S. cerevisiae) | 0.0002328 | -2.43517 |
| 1564651_at | LOC221710 | hypothetical protein LOC221710 | 5.83E-05 | -2.43524 |
| 205226_at | PDGFRL | platelet-derived growth factor receptor-like | 0.0002698 | -2.43728 |
| 224624_at | LRRC8A | leucine rich repeat containing 8 family, member A | 5.83E-05 | -2.43729 |
| 219660_s_at | ATP8A2 | ATPase, aminophospholipid transporter-like, class I, type 8A, member 2 | 0.0012773 | -2.43734 |
| 214060_at | SSBP1 | single-stranded DNA binding protein 1 | 4.68E-05 | -2.43887 |
| 211165_x_at | EPHB2 | EPH receptor B2 | 5.77E-06 | -2.43894 |
| 214209_s_at | ABCB9 | ATP-binding cassette, sub-family B (MDR/TAP), member 9 | 0.000171 | -2.44064 |
| 230029_x_at | UBR3 | ubiquitin protein ligase E3 component n-recognin 3 (putative) | 0.0002923 | -2.44098 |
| 212098_at | LOC151162 /// MGAT5 | hypothetical LOC151162 /// mannosyl (alpha-1,6-)-glycoprotein beta-1,6-N-acetyl- | 6.45E-06 | -2.44114 |
| 223688_s_at | LY6K | lymphocyte antigen 6 complex, locus K | 0.0010395 | -2.44167 |
| 218430_s_at | RFX7 | regulatory factor X, 7 | 0.0005928 | -2.44189 |
| 217403_s_at | ZNF227 | zinc finger protein 227 | 0.0002053 | -2.44429 |
| 201211_s_at | DDX3X | DEAD (Asp-Glu-Ala-Asp) box polypeptide 3, X-linked | 0.0001218 | -2.44434 |
| 211220_s_at | HSF2 | heat shock transcription factor 2 | 0.0011603 | -2.44435 |
| 221079_s_at | METTL2A /// METTL2B | methyltransferase like 2A /// methyltransferase like 2B | 0.0013379 | -2.44499 |
| 231766_s_at | COL12A1 | collagen, type XII, alpha 1 | 2.63E-05 | -2.4453 |
| 238614_x_at | ZNF430 | zinc finger protein 430 | 0.0006744 | -2.44553 |
| 242706_s_at | MED23 | mediator complex subunit 23 | 0.0009009 | -2.44573 |
| 227804_at | TLCD1 | TLC domain containing 1 | 0.0004592 | -2.44599 |
| 219499_at | SEC61A2 | Sec61 alpha 2 subunit (S. cerevisiae) | 0.0004657 | -2.44622 |
| 228490_at | ABHD2 | abhydrolase domain containing 2 | 5.67E-07 | -2.44655 |
| 204807_at | TMEM5 | transmembrane protein 5 | 0.0005979 | -2.44721 |
| 203213_at | CDC2 | cell division cycle 2, G1 to S and G2 to M | 0.0007319 | -2.44819 |
| 227620_at | SLC44A1 | solute carrier family 44, member 1 | 0.0005525 | -2.44895 |
| 1553954_at | ALG14 | asparagine-linked glycosylation 14 homolog (S. cerevisiae) | 0.0006135 | -2.44962 |
| 241946_at | ZDHHC21 | zinc finger, DHHC-type containing 21 | 0.0002518 | -2.44992 |
| 205174_s_at | QPCT | glutaminyl-peptide cyclotransferase | 0.0010145 | -2.45014 |
| 227776_at | ACER3 | alkaline ceramidase 3 | 6.22E-06 | -2.45038 |
| 209834_at | CHST3 | carbohydrate (chondroitin 6) sulfotransferase 3 | 1.76E-05 | -2.45061 |
| 236507_at | ZDHHC3 | zinc finger, DHHC-type containing 3 | 0.0005912 | -2.4507 |
| 220264_s_at | GPR107 | G protein-coupled receptor 107 | 0.0001837 | -2.45184 |
| 208614_s_at | FLNB | filamin B, beta | 4.02E-05 | -2.45199 |
| 1552625_a_at | TRNT1 | tRNA nucleotidyl transferase, CCA-adding, 1 | 0.0001929 | -2.45391 |
| 218501_at | ARHGEF3 | Rho guanine nucleotide exchange factor (GEF) 3 | 0.0002571 | -2.45399 |
| 34697_at | LRP6 | low density lipoprotein receptor-related protein 6 | 0.0003411 | -2.45533 |
| 200758_s_at | NFE2L1 | nuclear factor (erythroid-derived 2)-like 1 | 0.0003187 | -2.45557 |
| 243606_at | FAM55C | family with sequence similarity 55, member C | 0.0016389 | -2.45677 |
| 227162_at | ZBTB26 | zinc finger and BTB domain containing 26 | 0.0007161 | -2.45694 |
| 228908_s_at | LOC642852 | hypothetical LOC642852 | 0.0014508 | -2.45947 |
| 212076_at | MLL | myeloid/lymphoid or mixed-lineage leukemia (trithorax homolog, Drosophila) | 8.06E-05 | -2.46049 |
| 210982_s_at | HLA-DRA | major histocompatibility complex, class II, DR alpha | 9.88E-06 | -2.46097 |
| 201883_s_at | B4GALT1 | UDP-Gal:betaGlcNAc beta 1,4- galactosyltransferase, polypeptide 1 | 6.01E-05 | -2.46339 |
| 225675_at | C14orf101 | chromosome 14 open reading frame 101 | 0.0005421 | -2.46343 |
| 236356_at | NDUFS1 | NADH dehydrogenase (ubiquinone) Fe-S protein 1, 75kDa (NADH-coenzyme Q reductase | 0.0017865 | -2.46394 |
| 219627_at | ZNF767 | zinc finger family member 767 | 0.0001387 | -2.4644 |
| 1554493_s_at | THADA | thyroid adenoma associated | 0.0003213 | -2.4655 |
| 208510_s_at | PPARG | peroxisome proliferator-activated receptor gamma | 3.93E-05 | -2.46694 |
| 207686_s_at | CASP8 | caspase 8, apoptosis-related cysteine peptidase | 1.44E-05 | -2.46715 |
| 209140_x_at | HLA-B | major histocompatibility complex, class I, B | 3.54E-06 | -2.46805 |
| 204315_s_at | GTSE1 | G-2 and S-phase expressed 1 | 4.05E-05 | -2.46874 |
| 1556009_at | PEX13 | peroxisomal biogenesis factor 13 | 0.0012676 | -2.46902 |
| 201655_s_at | HSPG2 | heparan sulfate proteoglycan 2 | 0.0006147 | -2.46956 |
| 221677_s_at | ATP5O /// DONSON | ATP synthase, H+ transporting, mitochondrial F1 complex, O subunit /// downstrea | 3.84E-06 | -2.47052 |
| 238917_s_at | DENND5B | DENN/MADD domain containing 5B | 0.000214 | -2.47079 |
| 227628_at | GPX8 | glutathione peroxidase 8 (putative) | 0.0002253 | -2.47108 |
| 243552_at | MBTD1 | mbt domain containing 1 | 0.0003539 | -2.47118 |
| 213883_s_at | TM2D1 | TM2 domain containing 1 | 0.0001749 | -2.4716 |
| 203432_at | TMPO | thymopoietin | 1.65E-05 | -2.47233 |
| 216996_s_at | FASTKD2 | FAST kinase domains 2 | 8.36E-06 | -2.47259 |
| 218900_at | CNNM4 | cyclin M4 | 0.0004072 | -2.4728 |
| 230192_at | TRIM13 | tripartite motif-containing 13 | 1.48E-06 | -2.47325 |
| 219711_at | ZNF586 | zinc finger protein 586 | 0.0003215 | -2.47538 |
| 209774_x_at | CXCL2 | chemokine (C-X-C motif) ligand 2 | 0.0002452 | -2.47558 |
| 230686_s_at | SLC13A3 | solute carrier family 13 (sodium-dependent dicarboxylate transporter), member 3 | 7.31E-05 | -2.47568 |
| 215790_at | AJAP1 | adherens junctions associated protein 1 | 0.0001557 | -2.47571 |
| 238549_at | CBFA2T2 | core-binding factor, runt domain, alpha subunit 2; translocated to, 2 | 8.80E-06 | -2.47618 |
| 229240_at | ZDHHC21 | Zinc finger, DHHC-type containing 21 | 0.000715 | -2.47652 |
| 209320_at | ADCY3 | adenylate cyclase 3 | 0.0003228 | -2.4771 |
| 202867_s_at | DNAJB12 | DnaJ (Hsp40) homolog, subfamily B, member 12 | 0.0004069 | -2.47718 |
| 228331_at | C11orf31 | chromosome 11 open reading frame 31 | 0.0001803 | -2.47829 |
| 213234_at | KIAA1467 | KIAA1467 | 8.60E-05 | -2.47939 |
| 228762_at | LFNG | LFNG O-fucosylpeptide 3-beta-N-acetylglucosaminyltransferase | 9.89E-05 | -2.47962 |
| 239193_at | FUBP3 | far upstream element (FUSE) binding protein 3 | 0.0008051 | -2.48035 |
| 219502_at | NEIL3 | nei endonuclease VIII-like 3 (E. coli) | 4.80E-05 | -2.48102 |
| 213628_at | CLCC1 | chloride channel CLIC-like 1 | 9.97E-05 | -2.48137 |
| 233496_s_at | CFL2 | cofilin 2 (muscle) | 0.0003677 | -2.48319 |
| 230521_at | C9orf100 | chromosome 9 open reading frame 100 | 5.21E-07 | -2.48369 |
| 219906_at | FLJ10213 | hypothetical protein FLJ10213 | 0.0001836 | -2.484 |
| 225230_at | DRAM2 | DNA-damage regulated autophagy modulator 2 | 2.16E-05 | -2.48452 |
| 1552628_a_at | HERPUD2 | HERPUD family member 2 | 4.41E-05 | -2.48558 |
| 226899_at | UNC5B | unc-5 homolog B (C. elegans) | 0.0001632 | -2.4859 |
| 203938_s_at | TAF1C | TATA box binding protein (TBP)-associated factor, RNA polymerase I, C, 110kDa | 7.38E-05 | -2.48663 |
| 229732_at | ZNF823 | zinc finger protein 823 | 0.0008058 | -2.48718 |
| 210156_s_at | PCMT1 | protein-L-isoaspartate (D-aspartate) O-methyltransferase | 5.00E-05 | -2.48726 |
| 226041_at | NAPEPLD | N-acyl phosphatidylethanolamine phospholipase D | 1.98E-05 | -2.48856 |
| 217904_s_at | BACE1 | beta-site APP-cleaving enzyme 1 | 2.27E-05 | -2.48927 |
| 215493_x_at | BTN2A1 | butyrophilin, subfamily 2, member A1 | 0.0002854 | -2.49087 |
| 216488_s_at | ATP11A | ATPase, class VI, type 11A | 0.0002552 | -2.49133 |
| 222445_at | SLC39A9 | solute carrier family 39 (zinc transporter), member 9 | 3.57E-07 | -2.49155 |
| 220386_s_at | EML4 | echinoderm microtubule associated protein like 4 | 0.0002267 | -2.49185 |
| 219929_s_at | ZFYVE21 | zinc finger, FYVE domain containing 21 | 0.0002336 | -2.49203 |
| 224152_s_at | PBRM1 | polybromo 1 | 9.26E-05 | -2.49206 |
| 214962_s_at | NUP160 | nucleoporin 160kDa | 5.44E-05 | -2.49256 |
| 202843_at | DNAJB9 | DnaJ (Hsp40) homolog, subfamily B, member 9 | 0.0003815 | -2.4943 |
| 241606_s_at | TRUB1 | TruB pseudouridine (psi) synthase homolog 1 (E. coli) | 0.000343 | -2.49444 |
| 232674_at | UCN2 | urocortin 2 | 0.0003929 | -2.49469 |
| 1569366_a_at | ZNF569 | zinc finger protein 569 | 4.42E-05 | -2.49544 |
| 242138_at | DLX1 | distal-less homeobox 1 | 9.74E-05 | -2.49819 |
| 218302_at | PSENEN | presenilin enhancer 2 homolog (C. elegans) | 0.001669 | -2.49833 |
| 212201_at | ANKLE2 | ankyrin repeat and LEM domain containing 2 | 0.0008696 | -2.49835 |
| 218342_s_at | ERMP1 | endoplasmic reticulum metallopeptidase 1 | 0.0010592 | -2.49919 |
| 218589_at | LPAR6 | lysophosphatidic acid receptor 6 | 0.0005518 | -2.49987 |
| 214085_x_at | GLIPR1 | GLI pathogenesis-related 1 | 0.0004175 | -2.49991 |
| 212169_at | FKBP9 | FK506 binding protein 9, 63 kDa | 1.76E-05 | -2.50047 |
| 212853_at | DCUN1D4 | DCN1, defective in cullin neddylation 1, domain containing 4 (S. cerevisiae) | 0.0002473 | -2.50078 |
| 202516_s_at | DLG1 | discs, large homolog 1 (Drosophila) | 0.0002212 | -2.50308 |
| 233825_s_at | CD99L2 | CD99 molecule-like 2 | 3.08E-08 | -2.5032 |
| 207304_at | ZNF45 | zinc finger protein 45 | 6.70E-05 | -2.50351 |
| 235374_at | MDH1 | Malate dehydrogenase 1, NAD (soluble) | 2.76E-05 | -2.50447 |
| 213895_at | EMP1 | epithelial membrane protein 1 | 0.0001562 | -2.50514 |
| 224640_at | UNQ1887 | signal peptide peptidase 3 | 5.52E-05 | -2.50536 |
| 222217_s_at | SLC27A3 | solute carrier family 27 (fatty acid transporter), member 3 | 7.68E-06 | -2.50634 |
| 208916_at | SLC1A5 | solute carrier family 1 (neutral amino acid transporter), member 5 | 0.0005279 | -2.50636 |
| 1556821_x_at | DLEU2 | deleted in lymphocytic leukemia 2 (non-protein coding) | 0.0013581 | -2.50725 |
| 209450_at | OSGEP | O-sialoglycoprotein endopeptidase | 0.0003381 | -2.50861 |
| 225059_at | AGTRAP | angiotensin II receptor-associated protein | 2.79E-06 | -2.50971 |
| 219540_at | ZNF267 | zinc finger protein 267 | 0.0003574 | -2.51066 |
| 229251_s_at | TPCN2 | two pore segment channel 2 | 0.0001387 | -2.51117 |
| 1554628_at | ZNF57 | zinc finger protein 57 | 8.08E-05 | -2.51168 |
| 1552481_s_at | MAN1A2 | mannosidase, alpha, class 1A, member 2 | 0.0005443 | -2.51172 |
| 227031_at | SNX13 | sorting nexin 13 | 0.0007056 | -2.51298 |
| 235707_at | LOC221710 | hypothetical protein LOC221710 | 6.92E-05 | -2.51303 |
| 200654_at | P4HB | prolyl 4-hydroxylase, beta polypeptide | 2.60E-05 | -2.51309 |
| 205791_x_at | ZNF230 | zinc finger protein 230 | 0.0008295 | -2.51331 |
| 223089_at | VEZT | vezatin, adherens junctions transmembrane protein | 0.0006457 | -2.51336 |
| 223298_s_at | NT5C3 | 5'-nucleotidase, cytosolic III | 0.0001869 | -2.51398 |
| 227339_at | RGMB | RGM domain family, member B | 0.0011137 | -2.515 |
| 204668_at | RNF24 | ring finger protein 24 | 0.0003657 | -2.51531 |
| 243786_at | ZDHHC20 | zinc finger, DHHC-type containing 20 | 0.0005356 | -2.51634 |
| 204489_s_at | CD44 | CD44 molecule (Indian blood group) | 0.0002111 | -2.51776 |
| 203970_s_at | PEX3 | peroxisomal biogenesis factor 3 | 0.0001538 | -2.51887 |
| 207643_s_at | TNFRSF1A | tumor necrosis factor receptor superfamily, member 1A | 0.0004907 | -2.51898 |
| 204190_at | USPL1 | ubiquitin specific peptidase like 1 | 0.0002189 | -2.51915 |
| 202853_s_at | RYK | RYK receptor-like tyrosine kinase | 0.0004512 | -2.51969 |
| 218375_at | NUDT9 | nudix (nucleoside diphosphate linked moiety X)-type motif 9 | 0.000278 | -2.52026 |
| 229467_at | PCBP2 | Poly(rC) binding protein 2 | 0.0004585 | -2.52109 |
| 222787_s_at | TMEM106B | transmembrane protein 106B | 0.0006533 | -2.52321 |
| 214588_s_at | MFAP3 | microfibrillar-associated protein 3 | 2.74E-06 | -2.5233 |
| 238511_at | LOC440288 | similar to FLJ16518 protein | 0.0004786 | -2.52405 |
| 232931_at | SNRNP200 | small nuclear ribonucleoprotein 200kDa (U5) | 7.37E-05 | -2.52429 |
| 219329_s_at | C2orf28 | chromosome 2 open reading frame 28 | 1.44E-06 | -2.52443 |
| 210630_s_at | RAD52 | RAD52 homolog (S. cerevisiae) | 5.47E-05 | -2.52511 |
| 226577_at | PSEN1 | presenilin 1 | 0.0010121 | -2.52533 |
| 205490_x_at | GJB3 | gap junction protein, beta 3, 31kDa | 0.0001092 | -2.52541 |
| 204364_s_at | REEP1 | receptor accessory protein 1 | 0.0009399 | -2.52551 |
| 1555882_at | SPIN3 | spindlin family, member 3 | 0.0001529 | -2.52636 |
| 211506_s_at | IL8 | interleukin 8 | 0.0010242 | -2.52642 |
| 212162_at | KIDINS220 | kinase D-interacting substrate, 220kDa | 2.77E-05 | -2.52668 |
| 203836_s_at | MAP3K5 | mitogen-activated protein kinase kinase kinase 5 | 5.78E-05 | -2.52683 |
| 208622_s_at | EZR | ezrin | 1.28E-05 | -2.52774 |
| 218500_at | C8orf55 | chromosome 8 open reading frame 55 | 0.0016274 | -2.53013 |
| 210892_s_at | GTF2I | general transcription factor IIi | 8.69E-05 | -2.53062 |
| 221732_at | CANT1 | calcium activated nucleotidase 1 | 0.0001938 | -2.53127 |
| 219588_s_at | NCAPG2 | non-SMC condensin II complex, subunit G2 | 2.30E-05 | -2.5314 |
| 224630_at | ERLEC1 | endoplasmic reticulum lectin 1 | 3.62E-05 | -2.53169 |
| 213380_x_at | MSTP9 | macrophage stimulating, pseudogene 9 | 0.0001251 | -2.53201 |
| 202128_at | KIAA0317 | KIAA0317 | 4.67E-06 | -2.53333 |
| 244777_at | DCP2 | DCP2 decapping enzyme homolog (S. cerevisiae) | 0.0010606 | -2.53353 |
| 204350_s_at | MED7 | mediator complex subunit 7 | 0.0002314 | -2.53472 |
| 215092_s_at | NFAT5 | nuclear factor of activated T-cells 5, tonicity-responsive | 0.0017864 | -2.53503 |
| 218311_at | MAP4K3 | mitogen-activated protein kinase kinase kinase kinase 3 | 0.0003458 | -2.5351 |
| 215093_at | NSDHL | NAD(P) dependent steroid dehydrogenase-like | 0.0005523 | -2.53535 |
| 222689_at | ACER3 | alkaline ceramidase 3 | 0.001559 | -2.53557 |
| 228953_at | WHAMM | WAS protein homolog associated with actin, golgi membranes and microtubules | 3.75E-05 | -2.53565 |
| 224828_at | CPEB4 | cytoplasmic polyadenylation element binding protein 4 | 0.0007447 | -2.53566 |
| 234992_x_at | ECT2 | epithelial cell transforming sequence 2 oncogene | 2.29E-05 | -2.53593 |
| 226202_at | ZNF398 | zinc finger protein 398 | 0.0001348 | -2.53649 |
| 202533_s_at | DHFR | dihydrofolate reductase | 4.08E-05 | -2.53679 |
| 224564_s_at | RTN3 | reticulon 3 | 0.0002277 | -2.53827 |
| 239432_at | FLJ31306 | hypothetical LOC379025 | 0.0003212 | -2.5386 |
| 210269_s_at | SFRS17A | splicing factor, arginine/serine-rich 17A | 0.0003721 | -2.53912 |
| 213599_at | OIP5 | Opa interacting protein 5 | 7.75E-05 | -2.53919 |
| 206343_s_at | NRG1 | neuregulin 1 | 7.06E-05 | -2.53939 |
| 209865_at | SLC35A3 | solute carrier family 35 (UDP-N-acetylglucosamine (UDP-GlcNAc) transporter), mem | 0.0001348 | -2.53978 |
| 218665_at | FZD4 | frizzled homolog 4 (Drosophila) | 7.79E-05 | -2.53983 |
| 228902_at | NUP214 | nucleoporin 214kDa | 2.54E-06 | -2.54021 |
| 203118_at | PCSK7 | proprotein convertase subtilisin/kexin type 7 | 0.0001198 | -2.54045 |
| 226783_at | AGXT2L2 | alanine-glyoxylate aminotransferase 2-like 2 | 7.49E-06 | -2.54182 |
| 221258_s_at | KIF18A | kinesin family member 18A | 8.37E-06 | -2.54217 |
| 243531_at | ORAOV1 | oral cancer overexpressed 1 | 0.0016893 | -2.54223 |
| 1559382_at | C19orf42 | chromosome 19 open reading frame 42 | 9.15E-06 | -2.54313 |
| 212470_at | SPAG9 | sperm associated antigen 9 | 6.00E-05 | -2.54476 |
| 210650_s_at | PCLO | piccolo (presynaptic cytomatrix protein) | 0.0001904 | -2.54534 |
| 223759_s_at | GSG2 | germ cell associated 2 (haspin) | 4.83E-05 | -2.54543 |
| 210054_at | HAUS3 | HAUS augmin-like complex, subunit 3 | 0.0001163 | -2.54621 |
| 200791_s_at | IQGAP1 | IQ motif containing GTPase activating protein 1 | 4.93E-07 | -2.54734 |
| 223748_at | SLC4A11 | solute carrier family 4, sodium borate transporter, member 11 | 3.39E-06 | -2.54762 |
| 213167_s_at | SLC5A3 | solute carrier family 5 (sodium/myo-inositol cotransporter), member 3 | 8.99E-05 | -2.54851 |
| 235648_at | ZNF567 | zinc finger protein 567 | 0.0007681 | -2.54962 |
| 225681_at | CTHRC1 | collagen triple helix repeat containing 1 | 1.64E-07 | -2.55061 |
| 240983_s_at | CARS | cysteinyl-tRNA synthetase | 0.0003583 | -2.55086 |
| 200769_s_at | MAT2A | methionine adenosyltransferase II, alpha | 3.00E-05 | -2.5513 |
| 225807_at | JUB | jub, ajuba homolog (Xenopus laevis) | 0.0001938 | -2.55238 |
| 225666_at | TMTC4 | transmembrane and tetratricopeptide repeat containing 4 | 0.0004579 | -2.55441 |
| 205283_at | FKTN | fukutin | 0.0001033 | -2.5545 |
| 202749_at | WRB | tryptophan rich basic protein | 7.00E-06 | -2.55491 |
| 225339_at | SPAG9 | sperm associated antigen 9 | 6.52E-05 | -2.55501 |
| 205139_s_at | UST | uronyl-2-sulfotransferase | 0.0008548 | -2.55598 |
| 216125_s_at | RANBP9 | RAN binding protein 9 | 0.0014683 | -2.55725 |
| 32094_at | CHST3 | carbohydrate (chondroitin 6) sulfotransferase 3 | 7.21E-07 | -2.55786 |
| 205745_x_at | ADAM17 | ADAM metallopeptidase domain 17 | 0.0001779 | -2.5581 |
| 223614_at | MMP16 | matrix metallopeptidase 16 (membrane-inserted) | 5.06E-06 | -2.55932 |
| 209465_x_at | PTN | pleiotrophin | 0.0018109 | -2.55987 |
| 206290_s_at | RGS7 | regulator of G-protein signaling 7 | 7.98E-05 | -2.55988 |
| 205148_s_at | CLCN4 | chloride channel 4 | 0.0001182 | -2.56091 |
| 205112_at | PLCE1 | phospholipase C, epsilon 1 | 8.97E-06 | -2.56131 |
| 235653_s_at | THAP6 | THAP domain containing 6 | 0.0008552 | -2.56169 |
| 230793_at | LRRC16A | leucine rich repeat containing 16A | 0.000839 | -2.56205 |
| 201438_at | COL6A3 | collagen, type VI, alpha 3 | 0.0001912 | -2.56345 |
| 214696_at | C17orf91 | chromosome 17 open reading frame 91 | 8.32E-06 | -2.56588 |
| 235177_at | FAM119A | family with sequence similarity 119, member A | 2.33E-05 | -2.56624 |
| 227197_at | SGEF | Src homology 3 domain-containing guanine nucleotide exchange factor | 2.02E-06 | -2.56697 |
| 222519_s_at | IFT57 | intraflagellar transport 57 homolog (Chlamydomonas) | 3.45E-06 | -2.56719 |
| 208944_at | TGFBR2 | transforming growth factor, beta receptor II (70/80kDa) | 6.05E-05 | -2.56746 |
| 233230_s_at | SLAIN2 | SLAIN motif family, member 2 | 0.0001992 | -2.56795 |
| 206038_s_at | NR2C2 | nuclear receptor subfamily 2, group C, member 2 | 0.0014765 | -2.57012 |
| 227751_at | PDCD5 | programmed cell death 5 | 3.80E-05 | -2.57048 |
| 202620_s_at | PLOD2 | procollagen-lysine, 2-oxoglutarate 5-dioxygenase 2 | 0.0003992 | -2.57085 |
| 220985_s_at | RNF170 | ring finger protein 170 | 0.0011404 | -2.57165 |
| 232060_at | ROR1 | receptor tyrosine kinase-like orphan receptor 1 | 0.0006297 | -2.57266 |
| 1555953_at | SLC19A1 | solute carrier family 19 (folate transporter), member 1 | 0.0001509 | -2.5727 |
| 207528_s_at | SLC7A11 | solute carrier family 7, (cationic amino acid transporter, y+ system) member 11 | 0.0003075 | -2.5731 |
| 229492_at | VANGL1 | vang-like 1 (van gogh, Drosophila) | 0.0001585 | -2.5732 |
| 235496_at | HRCT1 | histidine rich carboxyl terminus 1 | 6.21E-05 | -2.57457 |
| 242919_at | ZNF253 | zinc finger protein 253 | 1.23E-05 | -2.57476 |
| 229831_at | CNTN3 | contactin 3 (plasmacytoma associated) | 0.0002507 | -2.57576 |
| 212959_s_at | GNPTAB | N-acetylglucosamine-1-phosphate transferase, alpha and beta subunits | 0.0007773 | -2.5761 |
| 225752_at | NIPA1 | non imprinted in Prader-Willi/Angelman syndrome 1 | 0.0004092 | -2.57619 |
| 241366_at | RBAK | RB-associated KRAB zinc finger | 0.0002043 | -2.57692 |
| 232475_at | C15orf42 | chromosome 15 open reading frame 42 | 8.52E-05 | -2.57717 |
| 214917_at | PRKAA1 | protein kinase, AMP-activated, alpha 1 catalytic subunit | 0.0012811 | -2.57764 |
| 211404_s_at | APLP2 | amyloid beta (A4) precursor-like protein 2 | 0.0004005 | -2.5781 |
| 207791_s_at | RAB1A | RAB1A, member RAS oncogene family | 6.90E-05 | -2.57856 |
| 205016_at | TGFA | transforming growth factor, alpha | 0.0002819 | -2.57916 |
| 231775_at | TNFRSF10A | tumor necrosis factor receptor superfamily, member 10a | 0.0004875 | -2.57923 |
| 223675_s_at | VEZT | vezatin, adherens junctions transmembrane protein | 8.45E-05 | -2.57981 |
| 226935_s_at | CLPTM1L | CLPTM1-like | 4.37E-07 | -2.58005 |
| 220918_at | C21orf96 | chromosome 21 open reading frame 96 | 1.70E-05 | -2.58038 |
| 213702_x_at | ASAH1 | N-acylsphingosine amidohydrolase (acid ceramidase) 1 | 4.12E-06 | -2.5804 |
| 218276_s_at | SAV1 | salvador homolog 1 (Drosophila) | 0.0002223 | -2.58042 |
| 213341_at | FEM1C | fem-1 homolog c (C. elegans) | 0.0014988 | -2.58065 |
| 232483_at | MED17 | mediator complex subunit 17 | 0.000935 | -2.58105 |
| 228801_at | ORMDL1 | ORM1-like 1 (S. cerevisiae) | 0.0006074 | -2.58184 |
| 203065_s_at | CAV1 | caveolin 1, caveolae protein, 22kDa | 7.79E-06 | -2.5819 |
| 228573_at | ANTXR2 | anthrax toxin receptor 2 | 0.000432 | -2.58247 |
| 202514_at | DLG1 | discs, large homolog 1 (Drosophila) | 0.0001297 | -2.58303 |
| 205926_at | IL27RA | interleukin 27 receptor, alpha | 1.57E-05 | -2.5839 |
| 1554345_a_at | GIN1 | gypsy retrotransposon integrase 1 | 0.0008871 | -2.5854 |
| 201079_at | SYNGR2 | synaptogyrin 2 | 6.38E-06 | -2.58594 |
| 232392_at | SFRS3 | Splicing factor, arginine/serine-rich 3 | 0.0001049 | -2.58665 |
| 236029_at | FAT3 | FAT tumor suppressor homolog 3 (Drosophila) | 0.0002307 | -2.58744 |
| 239002_at | ASPM | asp (abnormal spindle) homolog, microcephaly associated (Drosophila) | 6.64E-05 | -2.58766 |
| 229694_at | BRWD2 | bromodomain and WD repeat domain containing 2 | 0.0005335 | -2.58775 |
| 210667_s_at | C21orf33 /// PWP2 | chromosome 21 open reading frame 33 /// PWP2 periodic tryptophan protein homolog | 1.52E-05 | -2.58931 |
| 204032_at | BCAR3 | breast cancer anti-estrogen resistance 3 | 0.0001409 | -2.58994 |
| 1568954_s_at | C16orf72 | chromosome 16 open reading frame 72 | 0.0004751 | -2.59016 |
| 202766_s_at | FBN1 | fibrillin 1 | 2.64E-06 | -2.59094 |
| 218777_at | REEP4 | receptor accessory protein 4 | 1.53E-07 | -2.59286 |
| 221484_at | B4GALT5 | UDP-Gal:betaGlcNAc beta 1,4- galactosyltransferase, polypeptide 5 | 1.87E-05 | -2.5929 |
| 236656_s_at | LOC100288911 | hypothetical protein LOC100288911 | 4.56E-06 | -2.59323 |
| 238562_at | UTP23 | UTP23, small subunit (SSU) processome component, homolog (yeast) | 0.0002607 | -2.59364 |
| 222446_s_at | BACE2 | beta-site APP-cleaving enzyme 2 | 8.51E-07 | -2.59536 |
| 211537_x_at | MAP3K7 | mitogen-activated protein kinase kinase kinase 7 | 0.0005112 | -2.59541 |
| 210980_s_at | ASAH1 | N-acylsphingosine amidohydrolase (acid ceramidase) 1 | 0.0002773 | -2.59658 |
| 206788_s_at | CBFB | core-binding factor, beta subunit | 2.52E-05 | -2.59835 |
| 229335_at | CADM4 | cell adhesion molecule 4 | 0.0001867 | -2.59929 |
| 229134_at | VANGL1 | vang-like 1 (van gogh, Drosophila) | 0.0006417 | -2.59938 |
| 222806_s_at | C11orf30 | chromosome 11 open reading frame 30 | 2.72E-05 | -2.60076 |
| 233924_s_at | EXOC6 | exocyst complex component 6 | 0.0002735 | -2.60169 |
| 212001_at | SFRS14 | splicing factor, arginine/serine-rich 14 | 4.35E-06 | -2.60271 |
| 1565717_s_at | FUS /// NR1H3 | fusion (involved in t(12;16) in malignant liposarcoma) /// nuclear receptor subf | 1.64E-05 | -2.6048 |
| 230895_at | HAPLN1 | hyaluronan and proteoglycan link protein 1 | 1.94E-05 | -2.60498 |
| 209055_s_at | CDC5L | CDC5 cell division cycle 5-like (S. pombe) | 1.30E-05 | -2.6054 |
| 1553048_a_at | PIP4K2B | phosphatidylinositol-5-phosphate 4-kinase, type II, beta | 0.0001353 | -2.6061 |
| 212133_at | CYFIP1 | Cytoplasmic FMR1 interacting protein 1 | 1.41E-05 | -2.60633 |
| 211811_s_at | PCDHA6 | protocadherin alpha 6 | 0.0001271 | -2.60661 |
| 1555830_s_at | ESYT2 | extended synaptotagmin-like protein 2 | 0.0003503 | -2.60683 |
| 235006_at | CDKN2AIPNL | CDKN2A interacting protein N-terminal like | 7.47E-05 | -2.60804 |
| 223358_s_at | PDE7A | phosphodiesterase 7A | 0.0003225 | -2.60984 |
| 64408_s_at | CALML4 | calmodulin-like 4 | 0.0002971 | -2.61039 |
| 205524_s_at | HAPLN1 | hyaluronan and proteoglycan link protein 1 | 8.78E-07 | -2.61051 |
| 204955_at | SRPX | sushi-repeat-containing protein, X-linked | 6.11E-05 | -2.61095 |
| 202392_s_at | C22orf30 /// PISD | chromosome 22 open reading frame 30 /// phosphatidylserine decarboxylase | 3.43E-05 | -2.61146 |
| 203660_s_at | PCNT | pericentrin | 6.11E-05 | -2.61248 |
| 228671_at | TMEM201 | transmembrane protein 201 | 6.89E-06 | -2.6131 |
| 212881_at | PIAS4 | protein inhibitor of activated STAT, 4 | 0.0005412 | -2.61311 |
| 212953_x_at | CALR | calreticulin | 1.02E-06 | -2.61339 |
| 203372_s_at | SOCS2 | suppressor of cytokine signaling 2 | 0.0002579 | -2.61395 |
| 212345_s_at | CREB3L2 | cAMP responsive element binding protein 3-like 2 | 8.83E-06 | -2.61419 |
| 216005_at | TNC | Tenascin C | 0.0009183 | -2.61441 |
| 225548_at | SHROOM3 | shroom family member 3 | 0.0003369 | -2.61731 |
| 210074_at | CTSL2 | cathepsin L2 | 0.0001325 | -2.61749 |
| 217234_s_at | EZR | ezrin | 5.07E-05 | -2.61761 |
| 231904_at | U2AF1 | U2 small nuclear RNA auxiliary factor 1 | 0.0001849 | -2.61809 |
| 227408_s_at | SNX25 | sorting nexin 25 | 0.0001559 | -2.61831 |
| 1554474_a_at | MOXD1 | monooxygenase, DBH-like 1 | 1.65E-05 | -2.61842 |
| 220843_s_at | DCAF13 | DDB1 and CUL4 associated factor 13 | 0.0002111 | -2.61842 |
| 203665_at | HMOX1 | heme oxygenase (decycling) 1 | 0.0009089 | -2.6191 |
| 211450_s_at | MSH6 | mutS homolog 6 (E. coli) | 0.0002037 | -2.61972 |
| 236268_at | SEC22C | SEC22 vesicle trafficking protein homolog C (S. cerevisiae) | 0.0003645 | -2.61984 |
| 202857_at | CNPY2 | canopy 2 homolog (zebrafish) | 6.50E-05 | -2.62057 |
| 234991_at | ZXDC | ZXD family zinc finger C | 7.07E-05 | -2.6208 |
| 200766_at | CTSD | cathepsin D | 0.0002146 | -2.62236 |
| 238273_at | PL-5283 /// SLC13A4 | PL-5283 protein /// solute carrier family 13 (sodium/sulfate symporters), member | 0.0003957 | -2.62249 |
| 204244_s_at | DBF4 | DBF4 homolog (S. cerevisiae) | 1.96E-05 | -2.62295 |
| 216836_s_at | ERBB2 | v-erb-b2 erythroblastic leukemia viral oncogene homolog 2, neuro/glioblastoma de | 4.69E-05 | -2.62296 |
| 232287_at | PGBD3 | piggyBac transposable element derived 3 | 2.67E-07 | -2.62315 |
| 214723_x_at | ANKRD36 | ankyrin repeat domain 36 | 0.0013887 | -2.62316 |
| 223602_at | USP30 | ubiquitin specific peptidase 30 | 0.0003918 | -2.62367 |
| 223735_at | ARL6 | ADP-ribosylation factor-like 6 | 0.0002611 | -2.62431 |
| 224934_at | YIPF5 | Yip1 domain family, member 5 | 0.000753 | -2.62535 |
| 206066_s_at | RAD51C | RAD51 homolog C (S. cerevisiae) | 0.0014729 | -2.62572 |
| 1561961_at | DKFZp686A1627 | PHD finger protein 2 pseudogene-like | 0.0001791 | -2.62613 |
| 220046_s_at | CCNL1 | cyclin L1 | 1.18E-05 | -2.62771 |
| 212960_at | TBC1D9 | TBC1 domain family, member 9 (with GRAM domain) | 0.0002679 | -2.6308 |
| 204274_at | EBAG9 | estrogen receptor binding site associated, antigen, 9 | 0.0005218 | -2.63137 |
| 229666_s_at | CSTF3 | cleavage stimulation factor, 3' pre-RNA, subunit 3, 77kDa | 5.69E-05 | -2.63245 |
| 220019_s_at | ZNF224 | zinc finger protein 224 | 1.49E-05 | -2.63405 |
| 203293_s_at | LMAN1 | lectin, mannose-binding, 1 | 0.0017935 | -2.63461 |
| 209160_at | AKR1C3 | aldo-keto reductase family 1, member C3 (3-alpha hydroxysteroid dehydrogenase, t | 2.08E-05 | -2.63493 |
| 203944_x_at | BTN2A1 | butyrophilin, subfamily 2, member A1 | 0.0002218 | -2.63528 |
| 204863_s_at | IL6ST | interleukin 6 signal transducer (gp130, oncostatin M receptor) | 0.0004727 | -2.63618 |
| 222939_s_at | SLC16A10 | solute carrier family 16, member 10 (aromatic amino acid transporter) | 0.0003025 | -2.63626 |
| 213875_x_at | C6orf62 | chromosome 6 open reading frame 62 | 0.0009448 | -2.6363 |
| 210006_at | ABHD14A | abhydrolase domain containing 14A | 0.0001758 | -2.63634 |
| 201875_s_at | MPZL1 | myelin protein zero-like 1 | 0.0001488 | -2.63789 |
| 224801_at | NDFIP2 | Nedd4 family interacting protein 2 | 4.07E-06 | -2.6389 |
| 226498_at | FLT1 | fms-related tyrosine kinase 1 (vascular endothelial growth factor/vascular perme | 0.0007286 | -2.64107 |
| 226099_at | ELL2 | elongation factor, RNA polymerase II, 2 | 0.000517 | -2.64128 |
| 221319_at | PCDHB8 | protocadherin beta 8 | 0.0001034 | -2.64164 |
| 211343_s_at | COL13A1 | collagen, type XIII, alpha 1 | 7.41E-05 | -2.64262 |
| 201216_at | ERP29 | endoplasmic reticulum protein 29 | 0.0001275 | -2.6427 |
| 225033_at | ST3GAL1 | ST3 beta-galactoside alpha-2,3-sialyltransferase 1 | 1.38E-06 | -2.64331 |
| 223061_at | CHID1 | chitinase domain containing 1 | 4.95E-06 | -2.64342 |
| 200652_at | SSR2 | signal sequence receptor, beta (translocon-associated protein beta) | 2.14E-06 | -2.64345 |
| 236898_at | LOC100290882 | similar to hCG1994130 | 6.79E-05 | -2.64444 |
| 238633_at | EPC1 | Enhancer of polycomb homolog 1 (Drosophila) | 0.0002113 | -2.64485 |
| 218498_s_at | ERO1L | ERO1-like (S. cerevisiae) | 0.0003132 | -2.64517 |
| 218419_s_at | TMUB2 | transmembrane and ubiquitin-like domain containing 2 | 5.52E-05 | -2.6452 |
| 224987_at | C6orf89 | chromosome 6 open reading frame 89 | 0.0004699 | -2.64561 |
| 214765_s_at | NAAA | N-acylethanolamine acid amidase | 0.0005654 | -2.6462 |
| 203992_s_at | KDM6A | lysine (K)-specific demethylase 6A | 0.0008171 | -2.64762 |
| 203153_at | IFIT1 | interferon-induced protein with tetratricopeptide repeats 1 | 4.47E-05 | -2.64846 |
| 201609_x_at | ICMT | isoprenylcysteine carboxyl methyltransferase | 3.01E-05 | -2.64919 |
| 226721_at | DPY19L4 | dpy-19-like 4 (C. elegans) | 0.0005885 | -2.64974 |
| 230574_at | LOC100130938 | hypothetical LOC100130938 | 0.0009546 | -2.64974 |
| 215011_at | SNHG3 | small nucleolar RNA host gene 3 (non-protein coding) | 0.0014801 | -2.6498 |
| 222586_s_at | OSBPL11 | oxysterol binding protein-like 11 | 0.0001188 | -2.65 |
| 225228_at | DRAM2 | DNA-damage regulated autophagy modulator 2 | 0.0003729 | -2.6504 |
| 212097_at | CAV1 | caveolin 1, caveolae protein, 22kDa | 7.21E-05 | -2.65067 |
| 1560017_at | TMTC3 | transmembrane and tetratricopeptide repeat containing 3 | 2.20E-05 | -2.65133 |
| 234724_x_at | PCDHB18 | protocadherin beta 18 pseudogene | 0.0004091 | -2.65183 |
| 1554780_a_at | PHTF2 | putative homeodomain transcription factor 2 | 3.94E-06 | -2.65233 |
| 206693_at | IL7 | interleukin 7 | 8.14E-06 | -2.65361 |
| 217466_x_at | RPS2 | ribosomal protein S2 | 2.88E-05 | -2.65486 |
| 237817_at | SSR3 | Signal sequence receptor, gamma (translocon-associated protein gamma) | 0.0012976 | -2.65561 |
| 226050_at | TMCO3 | transmembrane and coiled-coil domains 3 | 2.03E-06 | -2.65576 |
| 204919_at | PRR4 | proline rich 4 (lacrimal) | 3.03E-05 | -2.6565 |
| 212463_at | CD59 | CD59 molecule, complement regulatory protein | 5.66E-06 | -2.6574 |
| 1552329_at | RBBP6 | retinoblastoma binding protein 6 | 4.78E-05 | -2.65944 |
| 212947_at | SLC9A8 | solute carrier family 9 (sodium/hydrogen exchanger), member 8 | 1.58E-06 | -2.65997 |
| 64371_at | SFRS14 | splicing factor, arginine/serine-rich 14 | 6.02E-07 | -2.66049 |
| 1553528_a_at | TAF5 | TAF5 RNA polymerase II, TATA box binding protein (TBP)-associated factor, 100kDa | 2.39E-05 | -2.66122 |
| 226891_at | C3orf21 | chromosome 3 open reading frame 21 | 6.88E-05 | -2.66133 |
| 219650_at | ERCC6L | excision repair cross-complementing rodent repair deficiency, complementation gr | 0.0011167 | -2.66154 |
| 225186_at | RAPH1 | Ras association (RalGDS/AF-6) and pleckstrin homology domains 1 | 5.55E-06 | -2.66354 |
| 201692_at | SIGMAR1 | sigma non-opioid intracellular receptor 1 | 0.000362 | -2.66418 |
| 224706_at | KIAA2013 | KIAA2013 | 0.0003872 | -2.66421 |
| 1553959_a_at | B3GALT6 | UDP-Gal:betaGal beta 1,3-galactosyltransferase polypeptide 6 | 9.13E-06 | -2.66491 |
| 213947_s_at | NUP210 | nucleoporin 210kDa | 4.58E-05 | -2.6654 |
| 220162_s_at | CARD9 | caspase recruitment domain family, member 9 | 1.77E-05 | -2.66608 |
| 223524_s_at | TMEM108 | transmembrane protein 108 | 3.59E-06 | -2.66689 |
| 1555476_at | IREB2 | iron-responsive element binding protein 2 | 1.07E-05 | -2.66719 |
| 223894_s_at | AKTIP | AKT interacting protein | 0.0004004 | -2.66739 |
| 211406_at | IER3IP1 | immediate early response 3 interacting protein 1 | 0.0002028 | -2.66759 |
| 216574_s_at | hCG_2024410 /// RPE | rcRPE /// ribulose-5-phosphate-3-epimerase | 0.0002779 | -2.66847 |
| 229250_at | TPCN2 | two pore segment channel 2 | 1.01E-05 | -2.6707 |
| 209578_s_at | POFUT2 | protein O-fucosyltransferase 2 | 8.53E-06 | -2.67092 |
| 226322_at | TMTC1 | transmembrane and tetratricopeptide repeat containing 1 | 9.17E-05 | -2.67141 |
| 218884_s_at | GUF1 | GUF1 GTPase homolog (S. cerevisiae) | 0.0002237 | -2.67235 |
| 1552717_s_at | CEP170 /// CEP170L | centrosomal protein 170kDa /// centrosomal protein 170kDa-like | 0.0006057 | -2.67292 |
| 201946_s_at | CCT2 | chaperonin containing TCP1, subunit 2 (beta) | 3.59E-05 | -2.67397 |
| 224341_x_at | TLR4 | toll-like receptor 4 | 3.79E-06 | -2.67409 |
| 1553148_a_at | SNX13 | sorting nexin 13 | 0.0006366 | -2.67489 |
| 202459_s_at | LPIN2 | lipin 2 | 0.0006716 | -2.67538 |
| 202651_at | LPGAT1 | lysophosphatidylglycerol acyltransferase 1 | 1.06E-05 | -2.67612 |
| 222309_at | C6orf62 | Chromosome 6 open reading frame 62 | 0.0018656 | -2.67805 |
| 218263_s_at | ZBED5 | zinc finger, BED-type containing 5 | 5.82E-05 | -2.6785 |
| 226689_at | CISD2 | CDGSH iron sulfur domain 2 | 6.40E-05 | -2.67859 |
| 201653_at | CNIH | cornichon homolog (Drosophila) | 5.69E-05 | -2.6786 |
| 222262_s_at | ETNK1 | ethanolamine kinase 1 | 5.14E-05 | -2.67895 |
| 205873_at | PIGL | phosphatidylinositol glycan anchor biosynthesis, class L | 8.20E-05 | -2.67976 |
| 211538_s_at | HSPA2 | heat shock 70kDa protein 2 | 6.92E-06 | -2.68048 |
| 224959_at | SLC26A2 | solute carrier family 26 (sulfate transporter), member 2 | 0.0004398 | -2.6821 |
| 210935_s_at | WDR1 | WD repeat domain 1 | 7.39E-05 | -2.68238 |
| 238561_s_at | UTP23 | UTP23, small subunit (SSU) processome component, homolog (yeast) | 0.000248 | -2.68299 |
| 242760_x_at | PIGB | phosphatidylinositol glycan anchor biosynthesis, class B | 0.0002922 | -2.68325 |
| 230134_s_at | RC3H2 | ring finger and CCCH-type zinc finger domains 2 | 0.0017248 | -2.68327 |
| 209943_at | FBXL4 | F-box and leucine-rich repeat protein 4 | 0.0004723 | -2.6834 |
| 214048_at | MBD4 | methyl-CpG binding domain protein 4 | 0.0008512 | -2.68369 |
| 238435_at | CA5BP | carbonic anhydrase VB pseudogene | 0.0001254 | -2.68403 |
| 201063_at | RCN1 | reticulocalbin 1, EF-hand calcium binding domain | 9.86E-10 | -2.68453 |
| 202846_s_at | PIGC | phosphatidylinositol glycan anchor biosynthesis, class C | 3.89E-05 | -2.68454 |
| 212998_x_at | HLA-DQB1 /// LOC100294318 | major histocompatibility complex, class II, DQ beta 1 /// similar to major histo | 0.0001252 | -2.68454 |
| 204005_s_at | PAWR | PRKC, apoptosis, WT1, regulator | 0.0007382 | -2.68496 |
| 219048_at | PIGN | phosphatidylinositol glycan anchor biosynthesis, class N | 2.43E-05 | -2.68541 |
| 203988_s_at | FUT8 | fucosyltransferase 8 (alpha (1,6) fucosyltransferase) | 0.0005022 | -2.68607 |
| 235315_at | TSC22D1 | TSC22 domain family, member 1 | 0.0002177 | -2.68698 |
| 210942_s_at | ST3GAL6 | ST3 beta-galactoside alpha-2,3-sialyltransferase 6 | 0.00017 | -2.68738 |
| 232013_at | C9orf102 | chromosome 9 open reading frame 102 | 0.0006973 | -2.68845 |
| 202662_s_at | ITPR2 | inositol 1,4,5-triphosphate receptor, type 2 | 0.000826 | -2.69018 |
| 219495_s_at | ZNF180 | zinc finger protein 180 | 0.0007431 | -2.69036 |
| 205499_at | SRPX2 | sushi-repeat-containing protein, X-linked 2 | 2.47E-05 | -2.69099 |
| 218846_at | MED23 | mediator complex subunit 23 | 0.0008121 | -2.6912 |
| 212255_s_at | ATP2C1 | ATPase, Ca++ transporting, type 2C, member 1 | 0.0005337 | -2.69133 |
| 210594_x_at | MPZL1 | myelin protein zero-like 1 | 0.0001079 | -2.69204 |
| 230448_at | SLC38A10 | solute carrier family 38, member 10 | 0.0003785 | -2.69221 |
| 1555058_a_at | LPGAT1 | lysophosphatidylglycerol acyltransferase 1 | 0.0007484 | -2.69228 |
| 225521_at | ANAPC7 | anaphase promoting complex subunit 7 | 0.0005071 | -2.69319 |
| 213647_at | DNA2 | DNA replication helicase 2 homolog (yeast) | 0.0008198 | -2.69546 |
| 209823_x_at | HLA-DQB1 | major histocompatibility complex, class II, DQ beta 1 | 0.0002852 | -2.69579 |
| 211991_s_at | HLA-DPA1 | major histocompatibility complex, class II, DP alpha 1 | 1.65E-05 | -2.69627 |
| 209755_at | NMNAT2 | nicotinamide nucleotide adenylyltransferase 2 | 0.0001259 | -2.69711 |
| 204306_s_at | CD151 | CD151 molecule (Raph blood group) | 3.89E-05 | -2.69716 |
| 210733_at | TRAM1 | translocation associated membrane protein 1 | 0.0001992 | -2.69837 |
| 213078_x_at | LPCAT4 | lysophosphatidylcholine acyltransferase 4 | 2.35E-05 | -2.69862 |
| 225687_at | FAM83D | family with sequence similarity 83, member D | 1.96E-06 | -2.69941 |
| 212079_s_at | MLL | myeloid/lymphoid or mixed-lineage leukemia (trithorax homolog, Drosophila) | 0.0002481 | -2.7006 |
| 217947_at | CMTM6 | CKLF-like MARVEL transmembrane domain containing 6 | 0.0005365 | -2.70246 |
| 223274_at | TCF19 | transcription factor 19 | 2.06E-05 | -2.70275 |
| 212964_at | HIC2 | hypermethylated in cancer 2 | 0.000118 | -2.70342 |
| 211162_x_at | SCD | stearoyl-CoA desaturase (delta-9-desaturase) | 0.000166 | -2.70357 |
| 1555004_a_at | RBL1 | retinoblastoma-like 1 (p107) | 0.0005643 | -2.70399 |
| 219544_at | C13orf34 | chromosome 13 open reading frame 34 | 6.10E-06 | -2.70433 |
| 209406_at | BAG2 | BCL2-associated athanogene 2 | 4.97E-06 | -2.70503 |
| 225779_at | SLC27A4 | solute carrier family 27 (fatty acid transporter), member 4 | 4.38E-07 | -2.70591 |
| 209240_at | OGT | O-linked N-acetylglucosamine (GlcNAc) transferase (UDP-N-acetylglucosamine:polyp | 2.89E-06 | -2.70599 |
| 206141_at | MOCS3 | molybdenum cofactor synthesis 3 | 0.0002167 | -2.7068 |
| 207357_s_at | GALNT10 | UDP-N-acetyl-alpha-D-galactosamine:polypeptide N-acetylgalactosaminyltransferase | 0.000632 | -2.70687 |
| 244881_at | LMLN | leishmanolysin-like (metallopeptidase M8 family) | 1.47E-05 | -2.70822 |
| 216804_s_at | PDLIM5 | PDZ and LIM domain 5 | 4.27E-05 | -2.70848 |
| 226771_at | ATP8B2 | ATPase, class I, type 8B, member 2 | 0.000542 | -2.7093 |
| 226028_at | ROBO4 | roundabout homolog 4, magic roundabout (Drosophila) | 3.49E-05 | -2.70981 |
| 221194_s_at | RNFT1 | ring finger protein, transmembrane 1 | 0.000765 | -2.71016 |
| 235812_at | TMEM188 | transmembrane protein 188 | 0.0007024 | -2.71044 |
| 234978_at | SLC36A4 | solute carrier family 36 (proton/amino acid symporter), member 4 | 2.89E-05 | -2.71197 |
| 205239_at | AREG | amphiregulin | 4.64E-05 | -2.7125 |
| 225346_at | MTERFD3 | MTERF domain containing 3 | 8.15E-06 | -2.71269 |
| 212408_at | TOR1AIP1 | torsin A interacting protein 1 | 8.95E-06 | -2.71404 |
| 201398_s_at | TRAM1 | translocation associated membrane protein 1 | 4.90E-07 | -2.71816 |
| 218161_s_at | CLN6 | ceroid-lipofuscinosis, neuronal 6, late infantile, variant | 3.37E-06 | -2.71872 |
| 1558028_x_at | LOC647979 | hypothetical LOC647979 | 0.0005879 | -2.71982 |
| 201729_s_at | KIAA0100 | KIAA0100 | 0.0010639 | -2.7199 |
| 210994_x_at | TRIM23 | tripartite motif-containing 23 | 0.0001192 | -2.72061 |
| 227936_at | TMEM68 | transmembrane protein 68 | 1.00E-06 | -2.7209 |
| 206029_at | ANKRD1 | ankyrin repeat domain 1 (cardiac muscle) | 0.0005123 | -2.72141 |
| 204257_at | FADS3 | fatty acid desaturase 3 | 8.06E-06 | -2.72193 |
| 220770_s_at | C5orf54 | chromosome 5 open reading frame 54 | 0.0009476 | -2.72269 |
| 1554450_s_at | MIER3 | mesoderm induction early response 1, family member 3 | 7.45E-05 | -2.7228 |
| 209052_s_at | WHSC1 | Wolf-Hirschhorn syndrome candidate 1 | 0.0002024 | -2.72302 |
| 1556064_at | LOC284926 | hypothetical protein LOC284926 | 5.18E-05 | -2.72307 |
| 212177_at | SFRS18 | splicing factor, arginine/serine-rich 18 | 0.0001723 | -2.72316 |
| 208779_x_at | DDR1 | discoidin domain receptor tyrosine kinase 1 | 1.90E-06 | -2.7239 |
| 207388_s_at | PTGES | prostaglandin E synthase | 0.0001789 | -2.72528 |
| 218456_at | CAPRIN2 | caprin family member 2 | 0.0009521 | -2.72665 |
| 213246_at | C14orf109 | chromosome 14 open reading frame 109 | 5.04E-05 | -2.72666 |
| 214016_s_at | SFPQ | splicing factor proline/glutamine-rich (polypyrimidine tract binding protein ass | 4.70E-06 | -2.72685 |
| 209312_x_at | HLA-DRB1 /// HLA-DRB4 /// HLA-DRB5 | major histocompatibility complex, class II, DR beta 1 /// major histocompatibili | 6.20E-05 | -2.72696 |
| 208729_x_at | HLA-B | major histocompatibility complex, class I, B | 2.08E-05 | -2.72796 |
| 209994_s_at | ABCB1 /// ABCB4 | ATP-binding cassette, sub-family B (MDR/TAP), member 1 /// ATP-binding cassette, | 1.87E-05 | -2.72919 |
| 228913_at | LOC100190939 | hypothetical LOC100190939 | 0.000512 | -2.72985 |
| 201448_at | TIA1 | TIA1 cytotoxic granule-associated RNA binding protein | 0.0004576 | -2.73068 |
| 207746_at | POLQ | polymerase (DNA directed), theta | 9.18E-05 | -2.73266 |
| 225660_at | SEMA6A | sema domain, transmembrane domain (TM), and cytoplasmic domain, (semaphorin) 6A | 9.57E-05 | -2.73463 |
| 228456_s_at | LOC149832 | hypothetical protein LOC149832 | 0.0004199 | -2.73505 |
| 214172_x_at | RYK | RYK receptor-like tyrosine kinase | 8.89E-05 | -2.7352 |
| 210749_x_at | DDR1 | discoidin domain receptor tyrosine kinase 1 | 2.64E-05 | -2.73526 |
| 35626_at | SGSH | N-sulfoglucosamine sulfohydrolase | 5.80E-05 | -2.73662 |
| 234023_s_at | CENPJ | centromere protein J | 4.05E-06 | -2.73838 |
| 203798_s_at | VSNL1 | visinin-like 1 | 0.000348 | -2.73856 |
| 202038_at | UBE4A | ubiquitination factor E4A (UFD2 homolog, yeast) | 9.03E-06 | -2.7387 |
| 204291_at | ZNF518A | zinc finger protein 518A | 0.0002014 | -2.73916 |
| 209421_at | MSH2 | mutS homolog 2, colon cancer, nonpolyposis type 1 (E. coli) | 9.58E-05 | -2.73996 |
| 208248_x_at | APLP2 | amyloid beta (A4) precursor-like protein 2 | 0.000264 | -2.74099 |
| 209891_at | SPC25 | SPC25, NDC80 kinetochore complex component, homolog (S. cerevisiae) | 1.36E-05 | -2.74132 |
| 212190_at | SERPINE2 | serpin peptidase inhibitor, clade E (nexin, plasminogen activator inhibitor type | 1.99E-07 | -2.7418 |
| 223183_at | AGPAT3 | 1-acylglycerol-3-phosphate O-acyltransferase 3 | 7.48E-05 | -2.74188 |
| 212198_s_at | TM9SF4 | transmembrane 9 superfamily protein member 4 | 6.50E-07 | -2.74282 |
| 226796_at | ABHD15 | abhydrolase domain containing 15 | 0.0002263 | -2.74388 |
| 206232_s_at | B4GALT6 | UDP-Gal:betaGlcNAc beta 1,4- galactosyltransferase, polypeptide 6 | 3.22E-05 | -2.74424 |
| 219154_at | TMEM120B | transmembrane protein 120B | 0.0013089 | -2.74428 |
| 205198_s_at | ATP7A | ATPase, Cu++ transporting, alpha polypeptide | 4.08E-05 | -2.74558 |
| 200699_at | KDELR2 | KDEL (Lys-Asp-Glu-Leu) endoplasmic reticulum protein retention receptor 2 | 1.16E-05 | -2.74567 |
| 227467_at | RDH10 | retinol dehydrogenase 10 (all-trans) | 6.17E-06 | -2.74608 |
| 204079_at | TPST2 | tyrosylprotein sulfotransferase 2 | 6.78E-05 | -2.74673 |
| 223170_at | TMEM98 | transmembrane protein 98 | 1.11E-05 | -2.74685 |
| 224615_x_at | HM13 | histocompatibility (minor) 13 | 4.82E-06 | -2.74801 |
| 216684_s_at | SS18 | synovial sarcoma translocation, chromosome 18 | 5.79E-05 | -2.74892 |
| 236692_at | LOC729839 | similar to DTW domain containing 2 | 0.0001882 | -2.7512 |
| 208613_s_at | FLNB | filamin B, beta | 7.35E-05 | -2.75208 |
| 208962_s_at | FADS1 | fatty acid desaturase 1 | 1.23E-05 | -2.75236 |
| 224881_at | VKORC1L1 | vitamin K epoxide reductase complex, subunit 1-like 1 | 2.94E-07 | -2.75289 |
| 218875_s_at | FBXO5 | F-box protein 5 | 0.0007581 | -2.75307 |
| 205078_at | PIGF | phosphatidylinositol glycan anchor biosynthesis, class F | 0.0018245 | -2.75312 |
| 212023_s_at | MKI67 | antigen identified by monoclonal antibody Ki-67 | 0.0008311 | -2.75322 |
| 226470_at | GGT7 | gamma-glutamyltransferase 7 | 2.00E-06 | -2.75355 |
| 200998_s_at | CKAP4 | cytoskeleton-associated protein 4 | 0.0006141 | -2.75468 |
| 1554271_a_at | CENPL | centromere protein L | 2.40E-06 | -2.75701 |
| 213901_x_at | RBM9 | RNA binding motif protein 9 | 0.0001376 | -2.75791 |
| 214976_at | RPL13 | ribosomal protein L13 | 9.83E-06 | -2.75808 |
| 218424_s_at | STEAP3 | STEAP family member 3 | 0.0006885 | -2.76041 |
| 216048_s_at | RHOBTB3 | Rho-related BTB domain containing 3 | 9.18E-05 | -2.76156 |
| 219634_at | CHST11 | carbohydrate (chondroitin 4) sulfotransferase 11 | 2.28E-06 | -2.7627 |
| 219014_at | PLAC8 | placenta-specific 8 | 0.0008335 | -2.76357 |
| 226432_at | ETNK1 | ethanolamine kinase 1 | 0.0009982 | -2.76392 |
| 233884_at | HIVEP3 | human immunodeficiency virus type I enhancer binding protein 3 | 1.82E-06 | -2.76459 |
| 218910_at | ANO10 | anoctamin 10 | 0.0017834 | -2.76509 |
| 1559954_s_at | DDX42 | DEAD (Asp-Glu-Ala-Asp) box polypeptide 42 | 1.89E-05 | -2.76559 |
| 224596_at | SLC44A1 | solute carrier family 44, member 1 | 0.0001308 | -2.76571 |
| 242037_at | ASPH | Aspartate beta-hydroxylase | 0.0006481 | -2.76599 |
| 223001_at | OSTC | oligosaccharyltransferase complex subunit | 7.59E-06 | -2.76643 |
| 225833_at | DAGLB | diacylglycerol lipase, beta | 0.0002711 | -2.76657 |
| 205433_at | BCHE | butyrylcholinesterase | 7.16E-05 | -2.7669 |
| 34764_at | LARS2 | leucyl-tRNA synthetase 2, mitochondrial | 0.000133 | -2.76735 |
| 228253_at | LOXL3 | lysyl oxidase-like 3 | 1.19E-06 | -2.76909 |
| 205579_at | HRH1 | histamine receptor H1 | 3.38E-06 | -2.76914 |
| 212325_at | LIMCH1 | LIM and calponin homology domains 1 | 4.13E-05 | -2.77064 |
| 232067_at | C6orf168 | chromosome 6 open reading frame 168 | 2.98E-05 | -2.77128 |
| 210954_s_at | TSC22D2 | TSC22 domain family, member 2 | 1.79E-05 | -2.77315 |
| 238783_at | hCG_25653 /// TMEM161B | hCG25653 /// transmembrane protein 161B | 0.0009154 | -2.77338 |
| 205559_s_at | PCSK5 | proprotein convertase subtilisin/kexin type 5 | 0.0009345 | -2.77397 |
| 1554456_a_at | LINS1 | lines homolog 1 (Drosophila) | 7.40E-07 | -2.77421 |
| 203481_at | FAM178A | family with sequence similarity 178, member A | 0.0002601 | -2.77568 |
| 222678_s_at | DCUN1D1 | DCN1, defective in cullin neddylation 1, domain containing 1 (S. cerevisiae) | 0.0002736 | -2.77612 |
| 240221_at | CSNK1A1 | Casein kinase 1, alpha 1 | 3.52E-06 | -2.77613 |
| 238122_at | RBM12B | RNA binding motif protein 12B | 0.0001772 | -2.77723 |
| 214995_s_at | APOBEC3F /// APOBEC3G | apolipoprotein B mRNA editing enzyme, catalytic polypeptide-like 3F /// apolipop | 2.81E-07 | -2.77756 |
| 218636_s_at | MAN1B1 | mannosidase, alpha, class 1B, member 1 | 5.86E-05 | -2.77815 |
| 203279_at | EDEM1 | ER degradation enhancer, mannosidase alpha-like 1 | 5.06E-06 | -2.7782 |
| 244659_at | LOC100131015 | hypothetical LOC100131015 | 0.0005088 | -2.78002 |
| 214991_s_at | PIGO | phosphatidylinositol glycan anchor biosynthesis, class O | 4.10E-05 | -2.78197 |
| 204328_at | TMC6 | transmembrane channel-like 6 | 0.0001594 | -2.78306 |
| 36499_at | CELSR2 | cadherin, EGF LAG seven-pass G-type receptor 2 (flamingo homolog, Drosophila) | 5.73E-05 | -2.78314 |
| 227859_at | DNAJC27 | DnaJ (Hsp40) homolog, subfamily C, member 27 | 6.34E-05 | -2.78499 |
| 1556049_at | RTN4 | reticulon 4 | 9.73E-05 | -2.78578 |
| 200096_s_at | ATP6V0E1 | ATPase, H+ transporting, lysosomal 9kDa, V0 subunit e1 | 2.61E-05 | -2.78611 |
| 1552610_a_at | JAK1 | Janus kinase 1 | 0.0002362 | -2.7873 |
| 236022_at | MYO19 | myosin XIX | 6.77E-06 | -2.78773 |
| 205085_at | ORC1L | origin recognition complex, subunit 1-like (yeast) | 1.48E-06 | -2.78873 |
| 211990_at | HLA-DPA1 | major histocompatibility complex, class II, DP alpha 1 | 1.55E-05 | -2.78944 |
| 208894_at | HLA-DRA | major histocompatibility complex, class II, DR alpha | 2.09E-05 | -2.78993 |
| 240452_at | GSPT1 | G1 to S phase transition 1 | 0.000166 | -2.79048 |
| 223223_at | ARV1 | ARV1 homolog (S. cerevisiae) | 2.28E-06 | -2.79062 |
| 238885_at | KIAA1549 | KIAA1549 | 0.0011447 | -2.79108 |
| 1555781_at | PQLC2 | PQ loop repeat containing 2 | 0.0001789 | -2.79123 |
| 226422_at | ERGIC2 | ERGIC and golgi 2 | 1.40E-06 | -2.79197 |
| 231108_at | FUS | fusion (involved in t(12;16) in malignant liposarcoma) | 1.88E-05 | -2.79604 |
| 238736_at | REV3L | REV3-like, catalytic subunit of DNA polymerase zeta (yeast) | 0.0001918 | -2.79605 |
| 212720_at | PAPOLA | poly(A) polymerase alpha | 0.000575 | -2.79677 |
| 1557218_s_at | FANCB | Fanconi anemia, complementation group B | 0.0001262 | -2.79678 |
| 221920_s_at | SLC25A37 | solute carrier family 25, member 37 | 0.001027 | -2.79923 |
| 212235_at | PLXND1 | plexin D1 | 7.01E-06 | -2.80294 |
| 212940_at | COL6A1 | collagen, type VI, alpha 1 | 0.000785 | -2.80317 |
| 1553099_at | TIGD1 | tigger transposable element derived 1 | 7.12E-06 | -2.80325 |
| 1567224_at | HMGA2 | high mobility group AT-hook 2 | 6.67E-05 | -2.80496 |
| 210042_s_at | CTSZ | cathepsin Z | 4.43E-05 | -2.80613 |
| 225975_at | PCDH18 | protocadherin 18 | 0.0001047 | -2.80616 |
| 212245_at | MCFD2 | multiple coagulation factor deficiency 2 | 9.72E-05 | -2.80681 |
| 211373_s_at | PSEN2 | presenilin 2 (Alzheimer disease 4) | 2.61E-05 | -2.80706 |
| 235239_at | QSOX2 | quiescin Q6 sulfhydryl oxidase 2 | 0.0001463 | -2.80801 |
| 210260_s_at | TNFAIP8 | tumor necrosis factor, alpha-induced protein 8 | 5.32E-06 | -2.81041 |
| 207329_at | MMP8 | matrix metallopeptidase 8 (neutrophil collagenase) | 1.40E-05 | -2.81088 |
| 223325_at | TXNDC11 | thioredoxin domain containing 11 | 4.70E-05 | -2.814 |
| 225839_at | RBM33 | RNA binding motif protein 33 | 6.45E-05 | -2.815 |
| 234944_s_at | FAM54A | family with sequence similarity 54, member A | 1.03E-05 | -2.81584 |
| 200087_s_at | TMED2 | transmembrane emp24 domain trafficking protein 2 | 9.25E-07 | -2.81634 |
| 208702_x_at | APLP2 | amyloid beta (A4) precursor-like protein 2 | 7.03E-05 | -2.81651 |
| 224858_at | ZDHHC5 | zinc finger, DHHC-type containing 5 | 9.55E-06 | -2.81662 |
| 235206_at | SCAND1 | SCAN domain containing 1 | 8.38E-05 | -2.81766 |
| 1553313_s_at | SLC5A3 | solute carrier family 5 (sodium/myo-inositol cotransporter), member 3 | 0.0013775 | -2.82004 |
| 229650_s_at | C19orf42 | chromosome 19 open reading frame 42 | 1.54E-05 | -2.82163 |
| 232098_at | DST | dystonin | 0.0001576 | -2.82175 |
| 232661_s_at | C7orf64 | chromosome 7 open reading frame 64 | 0.0007396 | -2.82199 |
| 209728_at | HLA-DRB4 | major histocompatibility complex, class II, DR beta 4 | 0.0001223 | -2.82201 |
| 221760_at | MAN1A1 | Mannosidase, alpha, class 1A, member 1 | 0.0003575 | -2.82428 |
| 227687_at | HYLS1 | hydrolethalus syndrome 1 | 5.14E-06 | -2.82458 |
| 221817_at | DOLPP1 | dolichyl pyrophosphate phosphatase 1 | 0.000186 | -2.83063 |
| 37079_at | YDD19 | nuclear undecaprenyl pyrophosphate synthase 1 pseudogene | 0.0004055 | -2.83297 |
| 206079_at | CHML | choroideremia-like (Rab escort protein 2) | 0.0011734 | -2.83313 |
| 231871_at | GPR180 | G protein-coupled receptor 180 | 1.16E-05 | -2.83339 |
| 204709_s_at | KIF23 | kinesin family member 23 | 9.54E-05 | -2.83571 |
| 209748_at | SPAST | spastin | 0.0011939 | -2.83623 |
| 208844_at | VDAC3 | voltage-dependent anion channel 3 | 0.0009828 | -2.83753 |
| 202075_s_at | PLTP | phospholipid transfer protein | 1.20E-05 | -2.8383 |
| 226008_at | NDNL2 | necdin-like 2 | 4.26E-05 | -2.83835 |
| 226164_x_at | RIMKLB | Ribosomal modification protein rimK-like family member B | 0.0004201 | -2.83853 |
| 1555594_a_at | MBNL1 | muscleblind-like (Drosophila) | 0.0001729 | -2.83856 |
| 223765_s_at | KBTBD4 | kelch repeat and BTB (POZ) domain containing 4 | 2.24E-06 | -2.83878 |
| 1555201_a_at | RMND1 | required for meiotic nuclear division 1 homolog (S. cerevisiae) | 0.0004107 | -2.83958 |
| 226615_at | XPR1 | xenotropic and polytropic retrovirus receptor | 0.0001308 | -2.84021 |
| 238856_s_at | PANK2 | Pantothenate kinase 2 | 0.0003272 | -2.8425 |
| 235341_at | DNAJC3 | DnaJ (Hsp40) homolog, subfamily C, member 3 | 3.32E-05 | -2.8428 |
| 217599_s_at | MDFIC | MyoD family inhibitor domain containing | 0.0009523 | -2.84298 |
| 206383_s_at | G3BP2 | GTPase activating protein (SH3 domain) binding protein 2 | 0.0001143 | -2.8437 |
| 225707_at | ARL6IP6 | ADP-ribosylation-like factor 6 interacting protein 6 | 0.0004187 | -2.84493 |
| 202856_s_at | SLC16A3 | solute carrier family 16, member 3 (monocarboxylic acid transporter 4) | 3.03E-06 | -2.8472 |
| 212697_at | FAM134C | family with sequence similarity 134, member C | 7.65E-05 | -2.84753 |
| 225337_at | ABHD2 | abhydrolase domain containing 2 | 6.59E-05 | -2.84763 |
| 221983_at | FAM134A | family with sequence similarity 134, member A | 3.49E-05 | -2.84796 |
| 216252_x_at | FAS | Fas (TNF receptor superfamily, member 6) | 1.71E-05 | -2.84814 |
| 1552627_a_at | ARHGAP5 | Rho GTPase activating protein 5 | 0.0001641 | -2.85007 |
| 213957_s_at | CEP350 | centrosomal protein 350kDa | 2.02E-05 | -2.85013 |
| 224904_at | PDPR | pyruvate dehydrogenase phosphatase regulatory subunit | 0.000282 | -2.85184 |
| 208610_s_at | SRRM2 | serine/arginine repetitive matrix 2 | 1.88E-05 | -2.85322 |
| 217974_at | TM7SF3 | transmembrane 7 superfamily member 3 | 8.65E-06 | -2.85383 |
| 213927_at | MAP3K9 | mitogen-activated protein kinase kinase kinase 9 | 4.54E-05 | -2.85429 |
| 227247_at | PLEKHA8 | Pleckstrin homology domain containing, family A (phosphoinositide binding specif | 3.76E-06 | -2.85505 |
| 222581_at | XPR1 | xenotropic and polytropic retrovirus receptor | 1.17E-06 | -2.85507 |
| 214626_s_at | GANAB | glucosidase, alpha; neutral AB | 4.65E-06 | -2.85726 |
| 1558173_a_at | LUZP1 | leucine zipper protein 1 | 8.15E-05 | -2.85857 |
| 222699_s_at | PLEKHF2 | pleckstrin homology domain containing, family F (with FYVE domain) member 2 | 0.0002045 | -2.85862 |
| 206046_at | ADAM23 | ADAM metallopeptidase domain 23 | 7.61E-06 | -2.86057 |
| 204781_s_at | FAS | Fas (TNF receptor superfamily, member 6) | 1.10E-06 | -2.8618 |
| 243835_at | ZDHHC21 | Zinc finger, DHHC-type containing 21 | 4.33E-05 | -2.86237 |
| 201412_at | LRP10 | low density lipoprotein receptor-related protein 10 | 0.0003704 | -2.86445 |
| 226363_at | ABCC5 | ATP-binding cassette, sub-family C (CFTR/MRP), member 5 | 0.000688 | -2.86469 |
| 207069_s_at | SMAD6 | SMAD family member 6 | 3.42E-06 | -2.86519 |
| 210572_at | PCDHA2 | protocadherin alpha 2 | 0.0005134 | -2.86668 |
| 204969_s_at | RDX | radixin | 0.0001173 | -2.86669 |
| 210841_s_at | NRP2 | neuropilin 2 | 7.48E-06 | -2.86809 |
| 224977_at | C6orf89 | chromosome 6 open reading frame 89 | 8.63E-05 | -2.86867 |
| 209237_s_at | SLC23A2 | solute carrier family 23 (nucleobase transporters), member 2 | 1.09E-05 | -2.86906 |
| 202669_s_at | EFNB2 | ephrin-B2 | 0.0004548 | -2.87055 |
| 201340_s_at | ENC1 | ectodermal-neural cortex (with BTB-like domain) | 0.0012829 | -2.87338 |
| 223020_at | CLPTM1L | CLPTM1-like | 0.0001529 | -2.87456 |
| 1555514_a_at | PIAS2 | protein inhibitor of activated STAT, 2 | 2.32E-05 | -2.87531 |
| 242157_at | CHD9 | Chromodomain helicase DNA binding protein 9 | 1.52E-05 | -2.87691 |
| 229272_at | FNBP4 | formin binding protein 4 | 0.0001004 | -2.87723 |
| 224595_at | SLC44A1 | solute carrier family 44, member 1 | 4.08E-07 | -2.87747 |
| 235773_at | ZIK1 | zinc finger protein interacting with K protein 1 homolog (mouse) | 9.89E-07 | -2.8801 |
| 226630_at | C14orf106 | chromosome 14 open reading frame 106 | 5.77E-06 | -2.8811 |
| 218748_s_at | EXOC5 | exocyst complex component 5 | 2.96E-05 | -2.88189 |
| 1552931_a_at | PDE8A | phosphodiesterase 8A | 0.0016204 | -2.88192 |
| 223993_s_at | CNIH4 | cornichon homolog 4 (Drosophila) | 8.19E-08 | -2.88404 |
| 202358_s_at | SNX19 | sorting nexin 19 | 5.45E-05 | -2.88686 |
| 234980_at | TMEM56 | transmembrane protein 56 | 0.0001186 | -2.88772 |
| 223590_at | ZNF700 | zinc finger protein 700 | 0.0008931 | -2.8885 |
| 1560031_at | FRMD4A | FERM domain containing 4A | 0.0008119 | -2.88869 |
| 221739_at | C19orf10 | chromosome 19 open reading frame 10 | 0.0004948 | -2.88871 |
| 204854_at | GPR162 /// LEPREL2 | G protein-coupled receptor 162 /// leprecan-like 2 | 0.0001144 | -2.89136 |
| 222392_x_at | PERP | PERP, TP53 apoptosis effector | 7.03E-06 | -2.89235 |
| 1554008_at | OSMR | oncostatin M receptor | 3.95E-07 | -2.89253 |
| 203950_s_at | CLCN6 | chloride channel 6 | 2.23E-05 | -2.89263 |
| 1569003_at | TMEM49 | transmembrane protein 49 | 3.79E-06 | -2.89346 |
| 202756_s_at | GPC1 | glypican 1 | 1.27E-05 | -2.89608 |
| 1554036_at | ZBTB24 | zinc finger and BTB domain containing 24 | 5.36E-06 | -2.89636 |
| 1554696_s_at | TYMS | thymidylate synthetase | 0.000602 | -2.89677 |
| 219304_s_at | PDGFD | platelet derived growth factor D | 0.000327 | -2.89687 |
| 227295_at | IKIP | IKK interacting protein | 0.0010023 | -2.8977 |
| 219679_s_at | WAC | WW domain containing adaptor with coiled-coil | 6.58E-05 | -2.89828 |
| 227955_s_at | EFNA5 | ephrin-A5 | 3.32E-06 | -2.89861 |
| 228194_s_at | SORCS1 | sortilin-related VPS10 domain containing receptor 1 | 0.0001007 | -2.89918 |
| 214315_x_at | CALR | calreticulin | 1.20E-05 | -2.90031 |
| 63825_at | ABHD2 | abhydrolase domain containing 2 | 6.34E-06 | -2.9008 |
| 201116_s_at | CPE | carboxypeptidase E | 2.29E-05 | -2.90295 |
| 204458_at | PLA2G15 | phospholipase A2, group XV | 0.0004806 | -2.90295 |
| 1557905_s_at | CD44 | CD44 molecule (Indian blood group) | 1.58E-05 | -2.90448 |
| 229236_s_at | SFXN4 | sideroflexin 4 | 3.53E-05 | -2.90509 |
| 228061_at | CCDC126 | coiled-coil domain containing 126 | 6.17E-05 | -2.90533 |
| 219439_at | C1GALT1 | core 1 synthase, glycoprotein-N-acetylgalactosamine 3-beta-galactosyltransferase | 0.0013459 | -2.90682 |
| 216450_x_at | HSP90B1 | heat shock protein 90kDa beta (Grp94), member 1 | 0.0003698 | -2.90714 |
| 200918_s_at | SRPR | signal recognition particle receptor (docking protein) | 2.35E-06 | -2.90877 |
| 238794_at | C10orf78 | chromosome 10 open reading frame 78 | 0.0009367 | -2.90894 |
| 211136_s_at | CLPTM1 | cleft lip and palate associated transmembrane protein 1 | 2.66E-05 | -2.90926 |
| 210178_x_at | FUSIP1 | FUS interacting protein (serine/arginine-rich) 1 | 4.87E-05 | -2.91038 |
| 1555106_a_at | CTDSPL2 | CTD (carboxy-terminal domain, RNA polymerase II, polypeptide A) small phosphatas | 8.85E-05 | -2.9113 |
| 226094_at | PIK3C2A | phosphoinositide-3-kinase, class 2, alpha polypeptide | 0.0001023 | -2.91227 |
| 212321_at | SGPL1 | sphingosine-1-phosphate lyase 1 | 0.0002017 | -2.91516 |
| 235453_at | TOR1AIP2 | torsin A interacting protein 2 | 1.22E-05 | -2.91574 |
| 232235_at | DSEL | dermatan sulfate epimerase-like | 0.0004171 | -2.91677 |
| 203669_s_at | DGAT1 | diacylglycerol O-acyltransferase homolog 1 (mouse) | 1.13E-05 | -2.91686 |
| 1555446_s_at | TRAPPC10 | trafficking protein particle complex 10 | 0.0002936 | -2.9174 |
| 201476_s_at | RRM1 | ribonucleotide reductase M1 | 0.0001032 | -2.91785 |
| 221908_at | RNFT2 | ring finger protein, transmembrane 2 | 4.87E-06 | -2.91857 |
| 225695_at | C2orf18 | chromosome 2 open reading frame 18 | 3.15E-06 | -2.91866 |
| 244334_at | TRAM1L1 | translocation associated membrane protein 1-like 1 | 5.03E-05 | -2.91878 |
| 205571_at | LIPT1 | lipoyltransferase 1 | 0.0003122 | -2.91966 |
| 212314_at | KIAA0746 | KIAA0746 protein | 0.0001834 | -2.92041 |
| 222263_at | SLC35E1 | solute carrier family 35, member E1 | 2.60E-05 | -2.92089 |
| 204317_at | GTSE1 | G-2 and S-phase expressed 1 | 5.38E-06 | -2.92285 |
| 217543_s_at | MBTPS1 | membrane-bound transcription factor peptidase, site 1 | 6.09E-07 | -2.92502 |
| 228923_at | S100A6 | S100 calcium binding protein A6 | 9.35E-05 | -2.92544 |
| 213165_at | CEP350 | centrosomal protein 350kDa | 3.06E-05 | -2.92593 |
| 238541_at | C21orf58 | chromosome 21 open reading frame 58 | 2.57E-05 | -2.92863 |
| 1558015_s_at | ACTR2 | ARP2 actin-related protein 2 homolog (yeast) | 5.59E-06 | -2.93117 |
| 210530_s_at | NR2C1 | nuclear receptor subfamily 2, group C, member 1 | 5.88E-06 | -2.93171 |
| 236622_at | PIGM | phosphatidylinositol glycan anchor biosynthesis, class M | 0.000729 | -2.93185 |
| 222129_at | FAM134A | family with sequence similarity 134, member A | 0.000746 | -2.93438 |
| 229173_at | KIAA1715 | KIAA1715 | 0.000536 | -2.93459 |
| 227919_at | UCA1 | urothelial cancer associated 1 (non-protein coding) | 3.79E-06 | -2.93616 |
| 212144_at | UNC84B | unc-84 homolog B (C. elegans) | 1.98E-06 | -2.93734 |
| 205246_at | PEX13 | peroxisomal biogenesis factor 13 | 3.60E-05 | -2.93778 |
| 216944_s_at | ITPR1 | inositol 1,4,5-triphosphate receptor, type 1 | 0.0002654 | -2.94025 |
| 222691_at | SLC35B3 | solute carrier family 35, member B3 | 5.52E-05 | -2.94127 |
| 224470_at | SEC22C | SEC22 vesicle trafficking protein homolog C (S. cerevisiae) | 3.73E-06 | -2.94206 |
| 210191_s_at | PHTF1 | putative homeodomain transcription factor 1 | 5.32E-05 | -2.94515 |
| 201506_at | TGFBI | transforming growth factor, beta-induced, 68kDa | 3.87E-05 | -2.94529 |
| 244462_at | ZNF224 | zinc finger protein 224 | 0.0002099 | -2.94634 |
| 205074_at | SLC22A5 | solute carrier family 22 (organic cation/carnitine transporter), member 5 | 0.0004959 | -2.95024 |
| 239106_at | CA5BP | Carbonic anhydrase VB pseudogene | 0.0007703 | -2.95137 |
| 1569057_s_at | MIA3 | melanoma inhibitory activity family, member 3 | 1.54E-05 | -2.95153 |
| 231869_at | KIAA1586 | KIAA1586 | 0.0002497 | -2.95285 |
| 219238_at | PIGV | phosphatidylinositol glycan anchor biosynthesis, class V | 4.08E-05 | -2.95358 |
| 232296_s_at | GFM1 | G elongation factor, mitochondrial 1 | 0.0001545 | -2.9555 |
| 204359_at | FLRT2 | fibronectin leucine rich transmembrane protein 2 | 1.74E-05 | -2.95555 |
| 228566_at | RPRD1A | Regulation of nuclear pre-mRNA domain containing 1A | 6.11E-05 | -2.95568 |
| 212536_at | ATP11B | ATPase, class VI, type 11B | 9.72E-05 | -2.95581 |
| 1555858_at | LOC440944 | hypothetical LOC440944 | 0.0001634 | -2.95612 |
| 202765_s_at | FBN1 | fibrillin 1 | 0.0004947 | -2.95636 |
| 226185_at | CDS1 | CDP-diacylglycerol synthase (phosphatidate cytidylyltransferase) 1 | 0.0002351 | -2.95693 |
| 217921_at | MAN1A2 | mannosidase, alpha, class 1A, member 2 | 0.0003908 | -2.95737 |
| 213792_s_at | INSR | insulin receptor | 9.40E-06 | -2.95779 |
| 201771_at | SCAMP3 | secretory carrier membrane protein 3 | 5.45E-06 | -2.95909 |
| 204605_at | CGRRF1 | cell growth regulator with ring finger domain 1 | 0.0014753 | -2.95933 |
| 244427_at | KIF23 | Kinesin family member 23 | 0.0003754 | -2.96096 |
| 221984_s_at | FAM134A | family with sequence similarity 134, member A | 0.0001299 | -2.96483 |
| 212123_at | TCTN3 | tectonic family member 3 | 4.14E-05 | -2.96508 |
| 241343_at | RNASEH1 | Ribonuclease H1 | 7.97E-06 | -2.96578 |
| 218728_s_at | CNIH4 | cornichon homolog 4 (Drosophila) | 1.33E-05 | -2.96643 |
| 205606_at | LRP6 | low density lipoprotein receptor-related protein 6 | 5.26E-05 | -2.96663 |
| 207782_s_at | PSEN1 | presenilin 1 | 3.80E-05 | -2.96838 |
| 204457_s_at | GAS1 | growth arrest-specific 1 | 0.0003635 | -2.96856 |
| 204444_at | KIF11 | kinesin family member 11 | 1.10E-05 | -2.969 |
| 238004_at | PGBD2 | piggyBac transposable element derived 2 | 2.01E-05 | -2.96922 |
| 218930_s_at | TMEM106B | transmembrane protein 106B | 7.39E-05 | -2.96957 |
| 1555167_s_at | NAMPT | nicotinamide phosphoribosyltransferase | 0.0004966 | -2.97041 |
| 210639_s_at | ATG5 | ATG5 autophagy related 5 homolog (S. cerevisiae) | 0.0001112 | -2.97074 |
| 219973_at | ARSJ | arylsulfatase family, member J | 0.0002514 | -2.97296 |
| 202793_at | LPCAT3 | lysophosphatidylcholine acyltransferase 3 | 0.0006911 | -2.97398 |
| 231229_at | HILS1 | histone linker H1 domain, spermatid-specific 1 | 6.36E-05 | -2.97599 |
| 218507_at | C7orf68 | chromosome 7 open reading frame 68 | 8.42E-05 | -2.97784 |
| 203981_s_at | ABCD4 | ATP-binding cassette, sub-family D (ALD), member 4 | 4.17E-07 | -2.97828 |
| 201200_at | CREG1 | cellular repressor of E1A-stimulated genes 1 | 0.0009788 | -2.97901 |
| 203418_at | CCNA2 | cyclin A2 | 0.0001086 | -2.98244 |
| 233595_at | USP34 | ubiquitin specific peptidase 34 | 0.0001618 | -2.98245 |
| 214838_at | SFT2D2 | SFT2 domain containing 2 | 0.0001169 | -2.98257 |
| 228793_at | JMJD1C | jumonji domain containing 1C | 0.0004382 | -2.98276 |
| 219282_s_at | TRPV2 | transient receptor potential cation channel, subfamily V, member 2 | 8.35E-06 | -2.98284 |
| 204601_at | N4BP1 | NEDD4 binding protein 1 | 3.59E-06 | -2.98463 |
| 1555039_a_at | ABCC4 | ATP-binding cassette, sub-family C (CFTR/MRP), member 4 | 0.0001014 | -2.98468 |
| 227970_at | GPR157 | G protein-coupled receptor 157 | 3.05E-05 | -2.98584 |
| 219565_at | CYP20A1 | cytochrome P450, family 20, subfamily A, polypeptide 1 | 0.0006356 | -2.98738 |
| 209537_at | EXTL2 | exostoses (multiple)-like 2 | 2.43E-06 | -2.98789 |
| 1554890_a_at | TIA1 | TIA1 cytotoxic granule-associated RNA binding protein | 3.53E-05 | -2.99057 |
| 215313_x_at | HLA-A | major histocompatibility complex, class I, A | 1.56E-05 | -2.99233 |
| 1554522_at | CNNM2 | cyclin M2 | 0.0005074 | -2.99263 |
| 221618_s_at | TAF9B | TAF9B RNA polymerase II, TATA box binding protein (TBP)-associated factor, 31kDa | 0.0004246 | -2.99615 |
| 223533_at | LRRC8C | leucine rich repeat containing 8 family, member C | 0.0002752 | -2.99651 |
| 200847_s_at | TMEM66 | transmembrane protein 66 | 5.95E-05 | -2.99813 |
| 223917_s_at | SLC39A3 | solute carrier family 39 (zinc transporter), member 3 | 2.69E-06 | -3.00021 |
| 242438_at | ASXL1 | additional sex combs like 1 (Drosophila) | 3.92E-06 | -3.0016 |
| 234140_s_at | STIM2 | stromal interaction molecule 2 | 7.77E-05 | -3.00429 |
| 220525_s_at | AUP1 | ancient ubiquitous protein 1 | 3.12E-06 | -3.00479 |
| 213213_at | DIDO1 | death inducer-obliterator 1 | 2.21E-05 | -3.00511 |
| 208788_at | ELOVL5 | ELOVL family member 5, elongation of long chain fatty acids (FEN1/Elo2, SUR4/Elo | 1.12E-06 | -3.00597 |
| 212328_at | LIMCH1 | LIM and calponin homology domains 1 | 0.0011814 | -3.00725 |
| 223180_s_at | C18orf55 | chromosome 18 open reading frame 55 | 2.80E-05 | -3.00871 |
| 225646_at | CTSC | cathepsin C | 0.0001389 | -3.00877 |
| 230110_at | MCOLN2 | mucolipin 2 | 0.0006577 | -3.00958 |
| 201998_at | ST6GAL1 | ST6 beta-galactosamide alpha-2,6-sialyltranferase 1 | 6.04E-06 | -3.00993 |
| 209665_at | CYB561D2 | cytochrome b-561 domain containing 2 | 5.11E-05 | -3.01007 |
| 33736_at | STOML1 | stomatin (EPB72)-like 1 | 0.0001458 | -3.01013 |
| 213372_at | PAQR3 | progestin and adipoQ receptor family member III | 0.0003196 | -3.01113 |
| 227335_at | DIDO1 | death inducer-obliterator 1 | 1.54E-05 | -3.01139 |
| 213789_at | LOC100292959 | similar to hCG2042049 | 5.76E-07 | -3.01256 |
| 212000_at | SFRS14 | splicing factor, arginine/serine-rich 14 | 6.39E-05 | -3.01353 |
| 231989_s_at | LOC641298 | SMG1 homolog, phosphatidylinositol 3-kinase-related kinase pseudogene | 0.0002318 | -3.01385 |
| 202378_s_at | LEPROT | leptin receptor overlapping transcript | 2.08E-07 | -3.01406 |
| 223217_s_at | NFKBIZ | nuclear factor of kappa light polypeptide gene enhancer in B-cells inhibitor, ze | 0.0002995 | -3.01619 |
| 218130_at | C17orf62 | chromosome 17 open reading frame 62 | 0.0002997 | -3.01638 |
| 202465_at | PCOLCE | procollagen C-endopeptidase enhancer | 1.70E-06 | -3.01643 |
| 200046_at | DAD1 | defender against cell death 1 | 1.01E-07 | -3.01649 |
| 201474_s_at | ITGA3 | integrin, alpha 3 (antigen CD49C, alpha 3 subunit of VLA-3 receptor) | 0.000479 | -3.01774 |
| 235463_s_at | LASS6 | LAG1 homolog, ceramide synthase 6 | 0.0001291 | -3.01808 |
| 238551_at | FUT11 | fucosyltransferase 11 (alpha (1,3) fucosyltransferase) | 6.12E-06 | -3.01854 |
| 229287_at | PCNX | pecanex homolog (Drosophila) | 9.47E-05 | -3.02039 |
| 201742_x_at | SFRS1 | splicing factor, arginine/serine-rich 1 | 0.0009953 | -3.02111 |
| 210946_at | PPAP2A | phosphatidic acid phosphatase type 2A | 0.0001207 | -3.02173 |
| 234863_x_at | FBXO5 | F-box protein 5 | 1.02E-05 | -3.02233 |
| 221799_at | CHPF2 | chondroitin polymerizing factor 2 | 9.89E-07 | -3.02294 |
| 202595_s_at | LEPROTL1 | leptin receptor overlapping transcript-like 1 | 9.84E-07 | -3.0245 |
| 204686_at | IRS1 | insulin receptor substrate 1 | 1.50E-05 | -3.02453 |
| 213577_at | SQLE | squalene epoxidase | 5.82E-06 | -3.02561 |
| 224298_s_at | UBAC2 | UBA domain containing 2 | 4.92E-05 | -3.02642 |
| 211016_x_at | HSPA4 | heat shock 70kDa protein 4 | 6.01E-06 | -3.02686 |
| 221679_s_at | ABHD6 | abhydrolase domain containing 6 | 7.82E-05 | -3.02725 |
| 206985_at | HSD17B3 | hydroxysteroid (17-beta) dehydrogenase 3 | 0.0001814 | -3.02757 |
| 227379_at | MBOAT1 | membrane bound O-acyltransferase domain containing 1 | 2.15E-05 | -3.02877 |
| 201125_s_at | ITGB5 | integrin, beta 5 | 6.31E-07 | -3.02897 |
| 203044_at | CHSY1 | chondroitin sulfate synthase 1 | 1.75E-05 | -3.03009 |
| 1557984_s_at | RPAP3 | RNA polymerase II associated protein 3 | 0.0003033 | -3.03127 |
| 225412_at | TMEM87B | transmembrane protein 87B | 7.58E-05 | -3.03167 |
| 221759_at | G6PC3 | glucose 6 phosphatase, catalytic, 3 | 9.05E-06 | -3.03175 |
| 224901_at | SCD5 | stearoyl-CoA desaturase 5 | 1.17E-05 | -3.0318 |
| 213485_s_at | ABCC10 | ATP-binding cassette, sub-family C (CFTR/MRP), member 10 | 0.0010788 | -3.03391 |
| 222045_s_at | PCIF1 | PDX1 C-terminal inhibiting factor 1 | 1.70E-05 | -3.03507 |
| 212342_at | YIPF6 | Yip1 domain family, member 6 | 0.0001794 | -3.03526 |
| 206172_at | IL13RA2 | interleukin 13 receptor, alpha 2 | 0.0002723 | -3.0369 |
| 201891_s_at | B2M | beta-2-microglobulin | 2.56E-05 | -3.03703 |
| 218573_at | MAGEH1 | melanoma antigen family H, 1 | 0.0001447 | -3.03823 |
| 229105_at | GPR39 | G protein-coupled receptor 39 | 3.92E-05 | -3.03829 |
| 220240_s_at | TMCO3 | transmembrane and coiled-coil domains 3 | 0.0006101 | -3.03844 |
| 1552283_s_at | ZDHHC11 | zinc finger, DHHC-type containing 11 | 4.99E-06 | -3.03889 |
| 210916_s_at | CD44 | CD44 molecule (Indian blood group) | 6.64E-07 | -3.04085 |
| 212063_at | CD44 | CD44 molecule (Indian blood group) | 1.51E-05 | -3.04292 |
| 223314_at | TSPAN14 | tetraspanin 14 | 0.000232 | -3.04329 |
| 219869_s_at | SLC39A8 | solute carrier family 39 (zinc transporter), member 8 | 5.43E-05 | -3.04507 |
| 217914_at | TPCN1 | two pore segment channel 1 | 5.11E-07 | -3.0451 |
| 225516_at | SLC7A2 | solute carrier family 7 (cationic amino acid transporter, y+ system), member 2 | 6.03E-06 | -3.04577 |
| 208782_at | FSTL1 | follistatin-like 1 | 0.0002315 | -3.04733 |
| 222203_s_at | RDH14 | retinol dehydrogenase 14 (all-trans/9-cis/11-cis) | 3.86E-05 | -3.04943 |
| 203633_at | CPT1A | carnitine palmitoyltransferase 1A (liver) | 0.0001127 | -3.04974 |
| 212078_s_at | MLL | myeloid/lymphoid or mixed-lineage leukemia (trithorax homolog, Drosophila) | 1.50E-05 | -3.05071 |
| 208818_s_at | COMT | catechol-O-methyltransferase | 7.05E-07 | -3.05302 |
| 225717_at | KIAA1715 | KIAA1715 | 3.72E-05 | -3.05635 |
| 211703_s_at | TM2D1 | TM2 domain containing 1 | 2.62E-05 | -3.0567 |
| 202498_s_at | SLC2A3 | solute carrier family 2 (facilitated glucose transporter), member 3 | 8.48E-05 | -3.057 |
| 242722_at | LMO7 | LIM domain 7 | 0.0013144 | -3.05914 |
| 235675_at | DHFRL1 | dihydrofolate reductase-like 1 | 7.67E-05 | -3.05953 |
| 215708_s_at | PRIM2 | primase, DNA, polypeptide 2 (58kDa) | 0.0004518 | -3.06019 |
| 1554102_a_at | TMTC4 | transmembrane and tetratricopeptide repeat containing 4 | 6.36E-07 | -3.06124 |
| 228997_at | TRNAU1AP | tRNA selenocysteine 1 associated protein 1 | 0.0001028 | -3.06181 |
| 244165_at | C10orf18 | chromosome 10 open reading frame 18 | 0.0003618 | -3.06197 |
| 231849_at | KRT80 | keratin 80 | 3.01E-05 | -3.06468 |
| 204628_s_at | ITGB3 | integrin, beta 3 (platelet glycoprotein IIIa, antigen CD61) | 0.0004434 | -3.06678 |
| 226437_at | YIF1B | Yip1 interacting factor homolog B (S. cerevisiae) | 0.0001673 | -3.06819 |
| 1560265_at | GRIK2 | glutamate receptor, ionotropic, kainate 2 | 6.56E-07 | -3.06855 |
| 201915_at | SEC63 | SEC63 homolog (S. cerevisiae) | 0.0003697 | -3.06932 |
| 232263_at | SLC6A15 | solute carrier family 6 (neutral amino acid transporter), member 15 | 0.0002871 | -3.07367 |
| 233167_at | RP3-402G11.5 | selenoprotein O | 1.40E-05 | -3.074 |
| 224680_at | TMED4 | transmembrane emp24 protein transport domain containing 4 | 3.05E-07 | -3.07495 |
| 225352_at | SEC62 | SEC62 homolog (S. cerevisiae) | 0.0005557 | -3.07522 |
| 202296_s_at | RER1 | RER1 retention in endoplasmic reticulum 1 homolog (S. cerevisiae) | 3.20E-08 | -3.07567 |
| 241464_s_at | LOC400931 | hypothetical LOC400931 | 1.01E-05 | -3.07623 |
| 225689_at | C3orf39 | chromosome 3 open reading frame 39 | 3.72E-05 | -3.07774 |
| 226712_at | SSR1 | signal sequence receptor, alpha | 3.74E-06 | -3.07873 |
| 230788_at | GCNT2 | glucosaminyl (N-acetyl) transferase 2, I-branching enzyme (I blood group) | 0.0007173 | -3.07938 |
| 239512_at | SFRS4 | splicing factor, arginine/serine-rich 4 | 0.0004544 | -3.08033 |
| 1554465_s_at | ZNF673 /// ZNF674 | zinc finger family member 673 /// zinc finger family member 674 | 3.65E-05 | -3.082 |
| 242439_s_at | ASXL1 | additional sex combs like 1 (Drosophila) | 1.87E-06 | -3.08228 |
| 223594_at | TMEM117 | transmembrane protein 117 | 1.75E-05 | -3.08356 |
| 202037_s_at | SFRP1 | secreted frizzled-related protein 1 | 8.98E-06 | -3.08599 |
| 217920_at | MAN1A2 | mannosidase, alpha, class 1A, member 2 | 0.0002582 | -3.08835 |
| 209754_s_at | TMPO | thymopoietin | 1.11E-05 | -3.0895 |
| 216449_x_at | HSP90B1 | heat shock protein 90kDa beta (Grp94), member 1 | 1.72E-05 | -3.08967 |
| 1552304_at | ALG10 | asparagine-linked glycosylation 10, alpha-1,2-glucosyltransferase homolog (S. po | 0.0001823 | -3.0897 |
| 224619_at | CASC4 | cancer susceptibility candidate 4 | 0.000341 | -3.0924 |
| 238034_at | CANX | calnexin | 5.49E-05 | -3.09426 |
| 225003_at | TMEM205 | transmembrane protein 205 | 4.64E-05 | -3.09527 |
| 229969_at | SEC63 | SEC63 homolog (S. cerevisiae) | 4.78E-05 | -3.09555 |
| 202842_s_at | DNAJB9 | DnaJ (Hsp40) homolog, subfamily B, member 9 | 0.0002782 | -3.09629 |
| 201779_s_at | RNF13 | ring finger protein 13 | 0.0001495 | -3.09727 |
| 214629_x_at | RTN4 | reticulon 4 | 2.56E-06 | -3.09744 |
| 223396_at | TMEM60 | transmembrane protein 60 | 2.36E-05 | -3.09772 |
| 209735_at | ABCG2 | ATP-binding cassette, sub-family G (WHITE), member 2 | 1.70E-05 | -3.10075 |
| 218146_at | GLT8D1 | glycosyltransferase 8 domain containing 1 | 2.56E-05 | -3.1008 |
| 223242_s_at | MFSD11 | major facilitator superfamily domain containing 11 | 0.0001103 | -3.10087 |
| 219131_at | UBIAD1 | UbiA prenyltransferase domain containing 1 | 5.43E-05 | -3.10104 |
| 213379_at | COQ2 | coenzyme Q2 homolog, prenyltransferase (yeast) | 2.41E-06 | -3.10314 |
| 232441_at | KRR1 | KRR1, small subunit (SSU) processome component, homolog (yeast) | 0.0004331 | -3.10419 |
| 225575_at | LIFR | leukemia inhibitory factor receptor alpha | 0.0003903 | -3.10672 |
| 236019_at | RAB12 | RAB12, member RAS oncogene family | 0.0002889 | -3.107 |
| 1556151_at | ITFG1 | Integrin alpha FG-GAP repeat containing 1 | 2.62E-05 | -3.10899 |
| 223541_at | HAS3 | hyaluronan synthase 3 | 3.54E-06 | -3.11021 |
| 209835_x_at | CD44 | CD44 molecule (Indian blood group) | 8.85E-05 | -3.11021 |
| 222453_at | CYBRD1 | cytochrome b reductase 1 | 2.39E-05 | -3.11047 |
| 202433_at | SLC35B1 | solute carrier family 35, member B1 | 1.48E-08 | -3.1113 |
| 222528_s_at | SLC25A37 | solute carrier family 25, member 37 | 3.40E-05 | -3.11185 |
| 236227_at | TMEM161B | transmembrane protein 161B | 0.0003667 | -3.11299 |
| 230885_at | SPG7 | spastic paraplegia 7 (pure and complicated autosomal recessive) | 1.47E-06 | -3.11311 |
| 209099_x_at | JAG1 | jagged 1 (Alagille syndrome) | 6.80E-07 | -3.11319 |
| 222700_at | ATL2 | atlastin GTPase 2 | 0.0005283 | -3.11336 |
| 215239_x_at | ZNF273 | zinc finger protein 273 | 0.0001579 | -3.11573 |
| 228097_at | MYLIP | myosin regulatory light chain interacting protein | 0.0004495 | -3.11595 |
| 213292_s_at | SNX13 | sorting nexin 13 | 1.75E-06 | -3.1166 |
| 213391_at | DPY19L4 | dpy-19-like 4 (C. elegans) | 6.92E-06 | -3.11666 |
| 212737_at | GM2A | GM2 ganglioside activator | 1.84E-06 | -3.11668 |
| 218686_s_at | RHBDF1 | rhomboid 5 homolog 1 (Drosophila) | 6.82E-08 | -3.11698 |
| 215285_s_at | PHTF1 | putative homeodomain transcription factor 1 | 0.0005069 | -3.11998 |
| 204065_at | CHST10 | carbohydrate sulfotransferase 10 | 3.35E-05 | -3.12286 |
| 235170_at | ZNF92 | zinc finger protein 92 | 0.0001275 | -3.12335 |
| 46665_at | SEMA4C | sema domain, immunoglobulin domain (Ig), transmembrane domain (TM) and short cyt | 6.59E-08 | -3.12463 |
| 224902_at | PDPR | pyruvate dehydrogenase phosphatase regulatory subunit | 3.24E-05 | -3.12521 |
| 210807_s_at | SLC16A7 | solute carrier family 16, member 7 (monocarboxylic acid transporter 2) | 0.001103 | -3.12591 |
| 224868_at | ZDHHC5 | zinc finger, DHHC-type containing 5 | 0.0001168 | -3.12756 |
| 224708_at | KIAA2013 | KIAA2013 | 0.0002838 | -3.12848 |
| 212083_at | TEX261 | testis expressed 261 | 4.52E-08 | -3.12925 |
| 202008_s_at | NID1 | nidogen 1 | 0.0001715 | -3.12959 |
| 200700_s_at | KDELR2 | KDEL (Lys-Asp-Glu-Leu) endoplasmic reticulum protein retention receptor 2 | 6.46E-06 | -3.13056 |
| 226625_at | TGFBR3 | transforming growth factor, beta receptor III | 0.0003091 | -3.13059 |
| 208336_s_at | TECR | trans-2,3-enoyl-CoA reductase | 3.89E-06 | -3.13169 |
| 1554553_s_at | YIF1B | Yip1 interacting factor homolog B (S. cerevisiae) | 1.19E-05 | -3.13224 |
| 218008_at | C7orf42 | chromosome 7 open reading frame 42 | 2.09E-06 | -3.13291 |
| 227858_at | PCNXL3 | pecanex-like 3 (Drosophila) | 0.0001305 | -3.13338 |
| 211559_s_at | CCNG2 | cyclin G2 | 0.0014818 | -3.13387 |
| 214727_at | BRCA2 | breast cancer 2, early onset | 1.99E-05 | -3.13512 |
| 210282_at | ZMYM2 | zinc finger, MYM-type 2 | 4.89E-05 | -3.13864 |
| 223104_at | JAGN1 | jagunal homolog 1 (Drosophila) | 1.91E-06 | -3.13994 |
| 214703_s_at | MAN2B2 | mannosidase, alpha, class 2B, member 2 | 3.20E-05 | -3.14023 |
| 1563111_a_at | PIGX | phosphatidylinositol glycan anchor biosynthesis, class X | 0.0006143 | -3.14257 |
| 1552575_a_at | C6orf141 | chromosome 6 open reading frame 141 | 0.0001295 | -3.14296 |
| 223568_s_at | PPAPDC1B | phosphatidic acid phosphatase type 2 domain containing 1B | 5.71E-06 | -3.14528 |
| 208960_s_at | KLF6 | Kruppel-like factor 6 | 1.07E-05 | -3.14677 |
| 223772_s_at | TMEM87A | transmembrane protein 87A | 3.87E-06 | -3.14695 |
| 1554424_at | FIP1L1 | FIP1 like 1 (S. cerevisiae) | 0.0006649 | -3.14883 |
| 212913_at | C6orf26 /// MSH5 | chromosome 6 open reading frame 26 /// mutS homolog 5 (E. coli) | 0.000422 | -3.1502 |
| 219576_at | MAP7D3 | MAP7 domain containing 3 | 0.0001181 | -3.15049 |
| 228817_at | ALG9 | asparagine-linked glycosylation 9, alpha-1,2-mannosyltransferase homolog (S. cer | 0.0001152 | -3.15375 |
| 210559_s_at | CDC2 | cell division cycle 2, G1 to S and G2 to M | 2.26E-06 | -3.15403 |
| 219922_s_at | LTBP3 | latent transforming growth factor beta binding protein 3 | 4.01E-06 | -3.16233 |
| 206382_s_at | BDNF | brain-derived neurotrophic factor | 0.0003813 | -3.16258 |
| 211596_s_at | LRIG1 | leucine-rich repeats and immunoglobulin-like domains 1 | 0.0002526 | -3.16359 |
| 236420_s_at | ANO4 | anoctamin 4 | 0.0002308 | -3.16364 |
| 213758_at | COX4I1 | cytochrome c oxidase subunit IV isoform 1 | 2.47E-05 | -3.165 |
| 202407_s_at | PRPF31 | PRP31 pre-mRNA processing factor 31 homolog (S. cerevisiae) | 0.0001353 | -3.16503 |
| 214829_at | AASS | aminoadipate-semialdehyde synthase | 0.0003438 | -3.16531 |
| 209566_at | INSIG2 | insulin induced gene 2 | 7.94E-06 | -3.16863 |
| 210317_s_at | YWHAE | tyrosine 3-monooxygenase/tryptophan 5-monooxygenase activation protein, epsilon | 3.61E-05 | -3.16939 |
| 209732_at | CLEC2B | C-type lectin domain family 2, member B | 6.23E-07 | -3.1744 |
| 215207_x_at | NUS1 /// YDD19 | nuclear undecaprenyl pyrophosphate synthase 1 homolog (S. cerevisiae) /// nuclea | 0.0002988 | -3.17558 |
| 220441_at | DNAJC22 | DnaJ (Hsp40) homolog, subfamily C, member 22 | 2.09E-05 | -3.17586 |
| 212225_at | EIF1 | eukaryotic translation initiation factor 1 | 0.0001403 | -3.17601 |
| 1553956_at | ALS2CR4 | amyotrophic lateral sclerosis 2 (juvenile) chromosome region, candidate 4 | 6.33E-05 | -3.1761 |
| 202704_at | TOB1 | transducer of ERBB2, 1 | 6.49E-05 | -3.17741 |
| 209902_at | ATR | ataxia telangiectasia and Rad3 related | 0.0001141 | -3.17785 |
| 214494_s_at | SPG7 | spastic paraplegia 7 (pure and complicated autosomal recessive) | 0.0001206 | -3.17793 |
| 208837_at | TMED3 | transmembrane emp24 protein transport domain containing 3 | 6.81E-06 | -3.17914 |
| 1570253_a_at | RHEBL1 | Ras homolog enriched in brain like 1 | 9.02E-05 | -3.17942 |
| 217173_s_at | LDLR | low density lipoprotein receptor | 1.72E-05 | -3.1797 |
| 223010_s_at | OCIAD1 | OCIA domain containing 1 | 0.000113 | -3.18071 |
| 202380_s_at | NKTR | natural killer-tumor recognition sequence | 1.41E-05 | -3.18113 |
| 221317_x_at | PCDHB6 | protocadherin beta 6 | 6.03E-06 | -3.18475 |
| 44654_at | G6PC3 | glucose 6 phosphatase, catalytic, 3 | 1.58E-06 | -3.18958 |
| 233851_s_at | TOR3A | torsin family 3, member A | 0.0005844 | -3.18961 |
| 204360_s_at | NAGLU | N-acetylglucosaminidase, alpha- | 0.0002007 | -3.18965 |
| 204724_s_at | COL9A3 | collagen, type IX, alpha 3 | 8.70E-05 | -3.19112 |
| 223854_at | PCDHB10 | protocadherin beta 10 | 0.0002078 | -3.1938 |
| 225830_at | PDZD8 | PDZ domain containing 8 | 0.0004578 | -3.19612 |
| 222687_s_at | ACER3 | alkaline ceramidase 3 | 1.54E-06 | -3.19617 |
| 216521_s_at | BRCC3 | BRCA1/BRCA2-containing complex, subunit 3 | 0.0006554 | -3.19662 |
| 240419_at | SLC6A15 | solute carrier family 6 (neutral amino acid transporter), member 15 | 7.78E-05 | -3.19792 |
| 217544_at | LOC729806 | similar to hCG1725380 | 0.0016219 | -3.19826 |
| 213998_s_at | DDX17 | DEAD (Asp-Glu-Ala-Asp) box polypeptide 17 | 5.12E-05 | -3.20005 |
| 203675_at | NUCB2 | nucleobindin 2 | 3.11E-05 | -3.20109 |
| 214545_s_at | PROSC | proline synthetase co-transcribed homolog (bacterial) | 0.0009833 | -3.20166 |
| 209510_at | RNF139 | ring finger protein 139 | 4.33E-05 | -3.20501 |
| 227578_at | LOC100128191 | hypothetical protein LOC100128191 | 0.0001522 | -3.20532 |
| 218681_s_at | SDF2L1 | stromal cell-derived factor 2-like 1 | 4.72E-07 | -3.20588 |
| 217984_at | RNASET2 | ribonuclease T2 | 3.09E-05 | -3.21053 |
| 226132_s_at | MANEAL | mannosidase, endo-alpha-like | 1.28E-06 | -3.21437 |
| 208322_s_at | ST3GAL1 | ST3 beta-galactoside alpha-2,3-sialyltransferase 1 | 5.41E-05 | -3.21521 |
| 205107_s_at | EFNA4 | ephrin-A4 | 0.0003988 | -3.21578 |
| 222874_s_at | CLN8 | ceroid-lipofuscinosis, neuronal 8 (epilepsy, progressive with mental retardation | 2.45E-06 | -3.2159 |
| 209263_x_at | TSPAN4 | tetraspanin 4 | 2.86E-05 | -3.22002 |
| 203718_at | PNPLA6 | patatin-like phospholipase domain containing 6 | 0.0001958 | -3.22132 |
| 205452_at | PIGB | phosphatidylinositol glycan anchor biosynthesis, class B | 5.22E-05 | -3.22248 |
| 220285_at | FAM108B1 | family with sequence similarity 108, member B1 | 0.0006269 | -3.223 |
| 211708_s_at | SCD | stearoyl-CoA desaturase (delta-9-desaturase) | 1.73E-06 | -3.22424 |
| 204128_s_at | RFC3 | replication factor C (activator 1) 3, 38kDa | 3.06E-05 | -3.22536 |
| 218446_s_at | FAM18B | family with sequence similarity 18, member B | 0.000956 | -3.22848 |
| 205339_at | STIL | SCL/TAL1 interrupting locus | 3.81E-05 | -3.22866 |
| 1554260_a_at | FRYL | FRY-like | 0.0005875 | -3.22992 |
| 213548_s_at | CDV3 | CDV3 homolog (mouse) | 2.87E-05 | -3.23007 |
| 206460_at | AJAP1 | adherens junctions associated protein 1 | 2.21E-06 | -3.23267 |
| 229846_s_at | MAPKAP1 | mitogen-activated protein kinase associated protein 1 | 1.17E-06 | -3.23403 |
| 224799_at | NDFIP2 | Nedd4 family interacting protein 2 | 0.0001521 | -3.23446 |
| 201337_s_at | VAMP3 | vesicle-associated membrane protein 3 (cellubrevin) | 0.0006347 | -3.24015 |
| 212014_x_at | CD44 | CD44 molecule (Indian blood group) | 7.07E-05 | -3.24065 |
| 231867_at | ODZ2 | odz, odd Oz/ten-m homolog 2 (Drosophila) | 2.05E-05 | -3.24291 |
| 213198_at | ACVR1B | activin A receptor, type IB | 1.99E-06 | -3.24358 |
| 243683_at | MORF4L2 | Mortality factor 4 like 2 | 0.0001626 | -3.24589 |
| 219342_at | CASD1 | CAS1 domain containing 1 | 0.0007941 | -3.24593 |
| 213119_at | SLC36A1 | solute carrier family 36 (proton/amino acid symporter), member 1 | 1.46E-05 | -3.24938 |
| 207079_s_at | MED6 | mediator complex subunit 6 | 1.98E-05 | -3.24949 |
| 220085_at | HELLS | helicase, lymphoid-specific | 0.0001903 | -3.25383 |
| 202978_s_at | CREBZF | CREB/ATF bZIP transcription factor | 4.21E-05 | -3.25421 |
| 204928_s_at | SLC10A3 | solute carrier family 10 (sodium/bile acid cotransporter family), member 3 | 0.000698 | -3.2583 |
| 211080_s_at | NEK2 | NIMA (never in mitosis gene a)-related kinase 2 | 0.0003488 | -3.26155 |
| 221410_x_at | PCDHB3 | protocadherin beta 3 | 2.89E-07 | -3.26236 |
| 210540_s_at | B4GALT4 | UDP-Gal:betaGlcNAc beta 1,4- galactosyltransferase, polypeptide 4 | 4.54E-06 | -3.26416 |
| 1552287_s_at | AFG3L1 | AFG3 ATPase family gene 3-like 1 (S. cerevisiae) | 1.08E-05 | -3.26574 |
| 35820_at | GM2A | GM2 ganglioside activator | 1.38E-06 | -3.26755 |
| 208817_at | COMT | catechol-O-methyltransferase | 2.38E-05 | -3.26819 |
| 203799_at | CD302 | CD302 molecule | 0.00142 | -3.27006 |
| 200755_s_at | CALU | calumenin | 5.10E-06 | -3.27124 |
| 223341_s_at | SCOC | short coiled-coil protein | 0.0003243 | -3.27152 |
| 216503_s_at | MLLT10 | myeloid/lymphoid or mixed-lineage leukemia (trithorax homolog, Drosophila); tran | 0.0004111 | -3.27259 |
| 224949_at | YIPF5 | Yip1 domain family, member 5 | 0.0003092 | -3.27339 |
| 202718_at | IGFBP2 | insulin-like growth factor binding protein 2, 36kDa | 3.90E-05 | -3.2737 |
| 229908_s_at | UNKL | unkempt homolog (Drosophila)-like | 0.0001542 | -3.27449 |
| 214117_s_at | BTD | biotinidase | 1.39E-05 | -3.2755 |
| 220940_at | ANKRD36B | ankyrin repeat domain 36B | 1.82E-05 | -3.2759 |
| 1552621_at | POLR2J2 | polymerase (RNA) II (DNA directed) polypeptide J2 | 1.48E-05 | -3.27887 |
| 1555225_at | C1orf43 | chromosome 1 open reading frame 43 | 6.15E-07 | -3.28002 |
| 219403_s_at | HPSE | heparanase | 3.54E-06 | -3.28299 |
| 227037_at | PLD6 | phospholipase D family, member 6 | 0.0010407 | -3.28372 |
| 229672_at | UQCC | ubiquinol-cytochrome c reductase complex chaperone | 0.0006954 | -3.2843 |
| 201611_s_at | ICMT | isoprenylcysteine carboxyl methyltransferase | 4.28E-05 | -3.28495 |
| 1552417_a_at | NEDD1 | neural precursor cell expressed, developmentally down-regulated 1 | 7.89E-05 | -3.28695 |
| 222034_at | GNB2L1 | Guanine nucleotide binding protein (G protein), beta polypeptide 2-like 1 | 6.64E-06 | -3.28727 |
| 238070_at | CHD1L | Chromodomain helicase DNA binding protein 1-like | 0.0001706 | -3.28738 |
| 243502_at | GJC1 | gap junction protein, gamma 1, 45kDa | 0.0001072 | -3.28803 |
| 1555240_s_at | GNG12 | guanine nucleotide binding protein (G protein), gamma 12 | 0.0001003 | -3.29138 |
| 208715_at | TMCO1 | transmembrane and coiled-coil domains 1 | 5.50E-05 | -3.29439 |
| 220954_s_at | PILRB | paired immunoglobin-like type 2 receptor beta | 2.44E-05 | -3.29561 |
| 209796_s_at | CNPY2 | canopy 2 homolog (zebrafish) | 1.03E-06 | -3.29679 |
| 1552256_a_at | SCARB1 | scavenger receptor class B, member 1 | 9.52E-06 | -3.30095 |
| 217800_s_at | NDFIP1 | Nedd4 family interacting protein 1 | 2.22E-06 | -3.3018 |
| 228506_at | NSMCE4A | non-SMC element 4 homolog A (S. cerevisiae) | 0.0006653 | -3.30212 |
| 202450_s_at | CTSK | cathepsin K | 2.62E-08 | -3.30244 |
| 225622_at | PAG1 | phosphoprotein associated with glycosphingolipid microdomains 1 | 6.21E-06 | -3.30268 |
| 206770_s_at | SLC35A3 | solute carrier family 35 (UDP-N-acetylglucosamine (UDP-GlcNAc) transporter), mem | 0.0001028 | -3.3034 |
| 203834_s_at | TGOLN2 | trans-golgi network protein 2 | 2.97E-05 | -3.30711 |
| 229882_at | RPS15A | ribosomal protein S15a | 1.16E-08 | -3.30799 |
| 233461_x_at | ZNF226 | zinc finger protein 226 | 2.36E-05 | -3.30813 |
| 205715_at | BST1 | bone marrow stromal cell antigen 1 | 1.61E-06 | -3.30899 |
| 200756_x_at | CALU | calumenin | 2.51E-07 | -3.30954 |
| 225566_at | NRP2 | neuropilin 2 | 2.07E-05 | -3.30983 |
| 234993_at | ABHD13 | abhydrolase domain containing 13 | 3.78E-06 | -3.31055 |
| 228624_at | TMEM144 | transmembrane protein 144 | 3.56E-06 | -3.31056 |
| 238012_at | DPP7 | Dipeptidyl-peptidase 7 | 0.0006397 | -3.31056 |
| 201995_at | EXT1 | exostoses (multiple) 1 | 4.38E-09 | -3.3129 |
| 222644_s_at | GLT25D1 | glycosyltransferase 25 domain containing 1 | 2.53E-05 | -3.31315 |
| 217208_s_at | DLG1 | discs, large homolog 1 (Drosophila) | 0.0001006 | -3.31471 |
| 230147_at | F2RL2 | coagulation factor II (thrombin) receptor-like 2 | 2.21E-06 | -3.31577 |
| 202388_at | RGS2 | regulator of G-protein signaling 2, 24kDa | 2.58E-06 | -3.31727 |
| 228069_at | FAM54A | family with sequence similarity 54, member A | 0.000428 | -3.31757 |
| 219271_at | GALNT14 | UDP-N-acetyl-alpha-D-galactosamine:polypeptide N-acetylgalactosaminyltransferase | 0.0001126 | -3.31956 |
| 1568618_a_at | GALNT1 | UDP-N-acetyl-alpha-D-galactosamine:polypeptide N-acetylgalactosaminyltransferase | 7.99E-07 | -3.32107 |
| 1554768_a_at | MAD2L1 | MAD2 mitotic arrest deficient-like 1 (yeast) | 4.66E-06 | -3.32325 |
| 1559258_a_at | CXorf61 | chromosome X open reading frame 61 | 0.0003622 | -3.32334 |
| 218282_at | EDEM2 | ER degradation enhancer, mannosidase alpha-like 2 | 8.57E-05 | -3.32547 |
| 44120_at | ADCK2 | aarF domain containing kinase 2 | 2.83E-06 | -3.32839 |
| 212204_at | TMEM87A | transmembrane protein 87A | 2.48E-07 | -3.33064 |
| 225365_at | ZDHHC20 | zinc finger, DHHC-type containing 20 | 0.000438 | -3.33258 |
| 204645_at | CCNT2 | cyclin T2 | 0.0010808 | -3.33426 |
| 218017_s_at | HGSNAT | heparan-alpha-glucosaminide N-acetyltransferase | 0.0003035 | -3.33444 |
| 212658_at | LHFPL2 | lipoma HMGIC fusion partner-like 2 | 0.0001459 | -3.33591 |
| 220206_at | ZMYM1 | zinc finger, MYM-type 1 | 0.0001358 | -3.33883 |
| 209778_at | TRIP11 | thyroid hormone receptor interactor 11 | 0.0001714 | -3.33944 |
| 1553994_at | NT5E | 5'-nucleotidase, ecto (CD73) | 3.04E-05 | -3.34267 |
| 233350_s_at | TEX264 | testis expressed 264 | 5.71E-05 | -3.343 |
| 215236_s_at | PICALM | phosphatidylinositol binding clathrin assembly protein | 4.40E-05 | -3.34325 |
| 235413_at | GGCX | gamma-glutamyl carboxylase | 2.46E-05 | -3.3454 |
| 231918_s_at | GFM2 | G elongation factor, mitochondrial 2 | 8.69E-05 | -3.34631 |
| 1554462_a_at | DNAJB9 | DnaJ (Hsp40) homolog, subfamily B, member 9 | 5.78E-05 | -3.34784 |
| 204451_at | FZD1 | frizzled homolog 1 (Drosophila) | 2.32E-05 | -3.34873 |
| 204925_at | CTNS | cystinosis, nephropathic | 2.35E-05 | -3.3492 |
| 55093_at | CHPF2 | chondroitin polymerizing factor 2 | 4.35E-05 | -3.35414 |
| 1554556_a_at | ATP11B | ATPase, class VI, type 11B | 3.38E-06 | -3.35519 |
| 211979_at | GPR107 | G protein-coupled receptor 107 | 0.0014706 | -3.35604 |
| 225882_at | SLC35B4 | solute carrier family 35, member B4 | 7.93E-06 | -3.35737 |
| 1552312_a_at | MFAP3 | microfibrillar-associated protein 3 | 0.0002615 | -3.35748 |
| 227617_at | TMEM201 | transmembrane protein 201 | 0.0001444 | -3.3578 |
| 207904_s_at | LNPEP | leucyl/cystinyl aminopeptidase | 8.99E-05 | -3.35873 |
| 219918_s_at | ASPM | asp (abnormal spindle) homolog, microcephaly associated (Drosophila) | 1.97E-08 | -3.36022 |
| 209135_at | ASPH | aspartate beta-hydroxylase | 9.54E-05 | -3.36081 |
| 212285_s_at | AGRN | agrin | 2.57E-06 | -3.36115 |
| 213578_at | BMPR1A | bone morphogenetic protein receptor, type IA | 2.71E-05 | -3.36247 |
| 200707_at | PRKCSH | protein kinase C substrate 80K-H | 0.0001777 | -3.36466 |
| 218135_at | ERGIC2 | ERGIC and golgi 2 | 9.80E-08 | -3.36551 |
| 225849_s_at | SFT2D1 | SFT2 domain containing 1 | 2.77E-07 | -3.36603 |
| 202071_at | SDC4 | syndecan 4 | 2.55E-07 | -3.37117 |
| 219990_at | E2F8 | E2F transcription factor 8 | 8.91E-06 | -3.37437 |
| 226612_at | FLJ25076 | probable ubiquitin-conjugating enzyme E2 FLJ25076 | 0.0003167 | -3.37654 |
| 228087_at | CCDC126 | coiled-coil domain containing 126 | 0.0007708 | -3.37804 |
| 212398_at | RDX | radixin | 7.52E-05 | -3.37888 |
| 244786_at | SNHG10 | small nucleolar RNA host gene 10 (non-protein coding) | 0.0002576 | -3.38095 |
| 235348_at | ABHD13 | abhydrolase domain containing 13 | 0.0004219 | -3.38618 |
| 227535_at | C15orf24 | Chromosome 15 open reading frame 24 | 6.60E-05 | -3.38691 |
| 1553112_s_at | CDK8 | cyclin-dependent kinase 8 | 4.19E-06 | -3.38722 |
| 203214_x_at | CDC2 | cell division cycle 2, G1 to S and G2 to M | 6.62E-05 | -3.38767 |
| 235334_at | ST6GALNAC3 | ST6 (alpha-N-acetyl-neuraminyl-2,3-beta-galactosyl-1,3)-N-acetylgalactosaminide | 4.63E-06 | -3.38925 |
| 223324_s_at | TRPM7 | transient receptor potential cation channel, subfamily M, member 7 | 0.0001786 | -3.39052 |
| 200911_s_at | TACC1 | transforming, acidic coiled-coil containing protein 1 | 7.33E-06 | -3.39316 |
| 217811_at | SELT | selenoprotein T | 9.46E-07 | -3.39453 |
| 204204_at | SLC31A2 | solute carrier family 31 (copper transporters), member 2 | 8.41E-05 | -3.39473 |
| 1555736_a_at | AGTRAP | angiotensin II receptor-associated protein | 7.45E-06 | -3.39498 |
| 221156_x_at | CCPG1 | cell cycle progression 1 | 0.0001048 | -3.39554 |
| 209631_s_at | GPR37 | G protein-coupled receptor 37 (endothelin receptor type B-like) | 5.46E-05 | -3.39715 |
| 202638_s_at | ICAM1 | intercellular adhesion molecule 1 | 2.14E-05 | -3.39908 |
| 202593_s_at | GDE1 | glycerophosphodiester phosphodiesterase 1 | 0.000112 | -3.40011 |
| 220368_s_at | SMEK1 | SMEK homolog 1, suppressor of mek1 (Dictyostelium) | 8.31E-05 | -3.40124 |
| 1555960_at | HINT1 | histidine triad nucleotide binding protein 1 | 0.0002439 | -3.40318 |
| 219326_s_at | B3GNT2 | UDP-GlcNAc:betaGal beta-1,3-N-acetylglucosaminyltransferase 2 | 0.0001672 | -3.40443 |
| 201819_at | SCARB1 | scavenger receptor class B, member 1 | 1.90E-06 | -3.40527 |
| 228047_at | SNORA72 | small nucleolar RNA, H/ACA box 72 | 9.41E-05 | -3.40537 |
| 222774_s_at | NETO2 | neuropilin (NRP) and tolloid (TLL)-like 2 | 0.0002091 | -3.40709 |
| 225070_at | NUS1 | nuclear undecaprenyl pyrophosphate synthase 1 homolog (S. cerevisiae) | 3.55E-06 | -3.40732 |
| 222988_s_at | TMEM9 | transmembrane protein 9 | 8.61E-07 | -3.40745 |
| 226912_at | ZDHHC23 | zinc finger, DHHC-type containing 23 | 5.43E-05 | -3.40903 |
| 212850_s_at | LRP4 | low density lipoprotein receptor-related protein 4 | 2.79E-05 | -3.40956 |
| 209625_at | PIGH | phosphatidylinositol glycan anchor biosynthesis, class H | 0.0001496 | -3.41066 |
| 202104_s_at | SPG7 | spastic paraplegia 7 (pure and complicated autosomal recessive) | 1.27E-05 | -3.41389 |
| 202654_x_at | 7-三月 | membrane-associated ring finger (C3HC4) 7 | 7.21E-05 | -3.41404 |
| 232278_s_at | DEPDC1 | DEP domain containing 1 | 0.0001176 | -3.41579 |
| 229997_at | VANGL1 | vang-like 1 (van gogh, Drosophila) | 8.68E-05 | -3.41631 |
| 205090_s_at | NAGPA | N-acetylglucosamine-1-phosphodiester alpha-N-acetylglucosaminidase | 0.0006364 | -3.41771 |
| 223569_at | PPAPDC1B | phosphatidic acid phosphatase type 2 domain containing 1B | 2.12E-05 | -3.41792 |
| 209365_s_at | ECM1 | extracellular matrix protein 1 | 0.0001216 | -3.41813 |
| 212908_at | DNAJC16 | DnaJ (Hsp40) homolog, subfamily C, member 16 | 1.65E-05 | -3.41841 |
| 217766_s_at | TMEM50A | transmembrane protein 50A | 0.0007643 | -3.41914 |
| 1554149_at | CLDND1 | claudin domain containing 1 | 0.0005866 | -3.41921 |
| 201874_at | MPZL1 | myelin protein zero-like 1 | 2.17E-05 | -3.41952 |
| 1558924_s_at | CLIP1 | CAP-GLY domain containing linker protein 1 | 0.0003539 | -3.41998 |
| 202830_s_at | SLC37A4 | solute carrier family 37 (glucose-6-phosphate transporter), member 4 | 0.0003736 | -3.42145 |
| 226333_at | IL6R | interleukin 6 receptor | 0.0001609 | -3.42463 |
| 225341_at | MTERFD3 | MTERF domain containing 3 | 5.40E-06 | -3.42496 |
| 208759_at | NCSTN | nicastrin | 2.16E-06 | -3.42522 |
| 225656_at | EFHC1 | EF-hand domain (C-terminal) containing 1 | 1.30E-06 | -3.42546 |
| 200599_s_at | HSP90B1 | heat shock protein 90kDa beta (Grp94), member 1 | 9.03E-06 | -3.42831 |
| 213582_at | ATP11A | ATPase, class VI, type 11A | 4.00E-05 | -3.42951 |
| 202263_at | CYB5R1 | cytochrome b5 reductase 1 | 0.0001314 | -3.43111 |
| 218562_s_at | TMEM57 | transmembrane protein 57 | 0.0002834 | -3.43221 |
| 204597_x_at | STC1 | stanniocalcin 1 | 8.76E-07 | -3.43316 |
| 210510_s_at | NRP1 | neuropilin 1 | 1.61E-06 | -3.43359 |
| 214753_at | N4BP2L2 | NEDD4 binding protein 2-like 2 | 5.84E-05 | -3.4337 |
| 233555_s_at | SULF2 | sulfatase 2 | 2.72E-05 | -3.43543 |
| 235275_at | BMP8B | Bone morphogenetic protein 8b | 1.13E-05 | -3.43923 |
| 223182_s_at | AGPAT3 | 1-acylglycerol-3-phosphate O-acyltransferase 3 | 3.61E-06 | -3.44164 |
| 212779_at | KIAA1109 | KIAA1109 | 3.26E-05 | -3.4458 |
| 212202_s_at | TMEM87A | transmembrane protein 87A | 0.0003083 | -3.44657 |
| 208829_at | TAPBP | TAP binding protein (tapasin) | 4.85E-05 | -3.4466 |
| 213743_at | CCNT2 | cyclin T2 | 2.82E-05 | -3.4469 |
| 214683_s_at | CLK1 /// PPIL3 | CDC-like kinase 1 /// peptidylprolyl isomerase (cyclophilin)-like 3 | 0.0015023 | -3.44788 |
| 213672_at | MARS | methionyl-tRNA synthetase | 7.85E-06 | -3.45025 |
| 209822_s_at | VLDLR | very low density lipoprotein receptor | 0.0001827 | -3.45128 |
| 209920_at | BMPR2 | bone morphogenetic protein receptor, type II (serine/threonine kinase) | 0.0001526 | -3.45162 |
| 46323_at | CANT1 | calcium activated nucleotidase 1 | 1.13E-05 | -3.45272 |
| 200665_s_at | SPARC | secreted protein, acidic, cysteine-rich (osteonectin) | 1.02E-05 | -3.45302 |
| 209279_s_at | NSDHL | NAD(P) dependent steroid dehydrogenase-like | 0.0001194 | -3.45779 |
| 214769_at | CLCN4 | chloride channel 4 | 0.0008058 | -3.45862 |
| 211721_s_at | ZNF551 | zinc finger protein 551 | 3.93E-06 | -3.45868 |
| 212702_s_at | BICD2 | bicaudal D homolog 2 (Drosophila) | 1.61E-06 | -3.45906 |
| 203971_at | SLC31A1 | solute carrier family 31 (copper transporters), member 1 | 3.21E-05 | -3.46174 |
| 1553764_a_at | JUB | jub, ajuba homolog (Xenopus laevis) | 6.51E-06 | -3.46358 |
| 227828_s_at | FAM176A | family with sequence similarity 176, member A | 4.60E-05 | -3.46422 |
| 226877_at | RPL32P3 | ribosomal protein L32 pseudogene 3 | 7.88E-07 | -3.46555 |
| 229870_at | LOC644656 | Hypothetical protein LOC644656 | 7.53E-05 | -3.46686 |
| 213154_s_at | BICD2 | bicaudal D homolog 2 (Drosophila) | 2.12E-05 | -3.46741 |
| 224776_at | AGPAT6 | 1-acylglycerol-3-phosphate O-acyltransferase 6 (lysophosphatidic acid acyltransf | 2.41E-05 | -3.46854 |
| 216236_s_at | SLC2A14 /// SLC2A3 | solute carrier family 2 (facilitated glucose transporter), member 14 /// solute | 1.30E-06 | -3.46943 |
| 244463_at | ADAM23 | ADAM metallopeptidase domain 23 | 0.0002778 | -3.46951 |
| 206271_at | TLR3 | toll-like receptor 3 | 1.77E-05 | -3.47086 |
| 213728_at | LAMP1 | lysosomal-associated membrane protein 1 | 8.99E-05 | -3.47343 |
| 51228_at | RBM12B | RNA binding motif protein 12B | 5.77E-05 | -3.47544 |
| 202336_s_at | PAM | peptidylglycine alpha-amidating monooxygenase | 2.68E-06 | -3.47562 |
| 227882_at | FKRP | fukutin related protein | 5.35E-06 | -3.47669 |
| 230256_at | C1orf104 | Chromosome 1 open reading frame 104 | 0.0001569 | -3.47681 |
| 205018_s_at | MBNL2 | muscleblind-like 2 (Drosophila) | 2.38E-05 | -3.47735 |
| 208872_s_at | REEP5 | receptor accessory protein 5 | 2.76E-08 | -3.47772 |
| 216267_s_at | TMEM115 | transmembrane protein 115 | 2.18E-05 | -3.47798 |
| 220980_s_at | ADPGK | ADP-dependent glucokinase | 1.14E-05 | -3.48082 |
| 235035_at | SLC35E1 | solute carrier family 35, member E1 | 7.82E-05 | -3.48253 |
| 224849_at | TTC17 | tetratricopeptide repeat domain 17 | 2.08E-06 | -3.48294 |
| 1557360_at | LRPPRC | leucine-rich PPR-motif containing | 5.95E-05 | -3.48344 |
| 230676_s_at | TMEM19 | transmembrane protein 19 | 9.87E-05 | -3.48375 |
| 207169_x_at | DDR1 | discoidin domain receptor tyrosine kinase 1 | 2.69E-05 | -3.48405 |
| 1557100_s_at | HECTD1 | HECT domain containing 1 | 3.32E-05 | -3.4862 |
| 242263_at | TMED5 | Transmembrane emp24 protein transport domain containing 5 | 2.83E-05 | -3.48896 |
| 217294_s_at | ENO1 | enolase 1, (alpha) | 0.0001965 | -3.489 |
| 205165_at | CELSR3 | cadherin, EGF LAG seven-pass G-type receptor 3 (flamingo homolog, Drosophila) | 0.0001616 | -3.48919 |
| 230058_at | LOC646891 /// SDCCAG3 | similar to Serologically defined colon cancer antigen 3 /// serologically define | 0.0006089 | -3.49021 |
| 230748_at | SLC16A6 | solute carrier family 16, member 6 (monocarboxylic acid transporter 7) | 1.10E-05 | -3.49057 |
| 203210_s_at | RFC5 | replication factor C (activator 1) 5, 36.5kDa | 6.10E-06 | -3.4985 |
| 201433_s_at | PTDSS1 | phosphatidylserine synthase 1 | 4.78E-07 | -3.49891 |
| 218717_s_at | LEPREL1 | leprecan-like 1 | 0.0004874 | -3.49997 |
| 1557217_a_at | FANCB | Fanconi anemia, complementation group B | 0.000108 | -3.50093 |
| 226882_x_at | WDR4 | WD repeat domain 4 | 1.08E-06 | -3.50198 |
| 223892_s_at | TMBIM4 | transmembrane BAX inhibitor motif containing 4 | 0.0003329 | -3.50233 |
| 222556_at | ALG5 | asparagine-linked glycosylation 5, dolichyl-phosphate beta-glucosyltransferase h | 8.12E-08 | -3.50487 |
| 213470_s_at | HNRNPH1 | heterogeneous nuclear ribonucleoprotein H1 (H) | 8.41E-05 | -3.50555 |
| 222477_s_at | TM7SF3 | transmembrane 7 superfamily member 3 | 6.36E-08 | -3.50685 |
| 214660_at | PELO | Pelota homolog (Drosophila) | 0.0003444 | -3.51198 |
| 235060_at | LOC100190986 | hypothetical LOC100190986 | 0.0002078 | -3.51205 |
| 219175_s_at | SLC41A3 | solute carrier family 41, member 3 | 4.54E-06 | -3.51375 |
| 238681_at | GDPD1 | glycerophosphodiester phosphodiesterase domain containing 1 | 0.0001064 | -3.51581 |
| 209826_at | EGFL8 /// PPT2 | EGF-like-domain, multiple 8 /// palmitoyl-protein thioesterase 2 | 3.40E-06 | -3.51782 |
| 223181_at | C18orf55 | chromosome 18 open reading frame 55 | 1.08E-05 | -3.51892 |
| 221893_s_at | ADCK2 | aarF domain containing kinase 2 | 5.50E-05 | -3.51906 |
| 235590_at | FAM178A | family with sequence similarity 178, member A | 0.0001784 | -3.51968 |
| 235103_at | MAN2A1 | mannosidase, alpha, class 2A, member 1 | 0.0002631 | -3.51992 |
| 225008_at | ASPH | aspartate beta-hydroxylase | 0.0002779 | -3.52008 |
| 212256_at | GALNT10 | UDP-N-acetyl-alpha-D-galactosamine:polypeptide N-acetylgalactosaminyltransferase | 1.04E-05 | -3.52105 |
| 225677_at | BCAP29 | B-cell receptor-associated protein 29 | 2.31E-05 | -3.52269 |
| 209934_s_at | ATP2C1 | ATPase, Ca++ transporting, type 2C, member 1 | 1.84E-05 | -3.52598 |
| 226789_at | EMB | embigin homolog (mouse) | 3.19E-07 | -3.52679 |
| 210069_at | CHKB-CPT1B /// CPT1B | choline kinase-like, carnitine palmitoyltransferase 1B (muscle) transcription un | 3.07E-05 | -3.52805 |
| 203910_at | ARHGAP29 | Rho GTPase activating protein 29 | 1.77E-05 | -3.52838 |
| 229966_at | EWSR1 | Ewing sarcoma breakpoint region 1 | 4.69E-05 | -3.52854 |
| 202348_s_at | TOR1A | torsin family 1, member A (torsin A) | 6.72E-06 | -3.52974 |
| 204415_at | IFI6 | interferon, alpha-inducible protein 6 | 0.0002558 | -3.53199 |
| 209656_s_at | TMEM47 | transmembrane protein 47 | 1.48E-05 | -3.53266 |
| 235311_at | FKBP14 | FK506 binding protein 14, 22 kDa | 2.93E-05 | -3.53352 |
| 235202_x_at | IKIP | IKK interacting protein | 0.0002599 | -3.53429 |
| 217778_at | SLC39A1 | solute carrier family 39 (zinc transporter), member 1 | 1.37E-05 | -3.5347 |
| 232068_s_at | TLR4 | toll-like receptor 4 | 0.0001088 | -3.53518 |
| 224458_at | C9orf125 | chromosome 9 open reading frame 125 | 1.95E-05 | -3.53521 |
| 218473_s_at | GLT25D1 | glycosyltransferase 25 domain containing 1 | 5.64E-05 | -3.53701 |
| 234733_s_at | FANCM | Fanconi anemia, complementation group M | 7.71E-05 | -3.53749 |
| 210346_s_at | CLK4 | CDC-like kinase 4 | 0.0002063 | -3.53862 |
| 1563445_x_at | CTSLL3 | cathepsin L-like 3 | 0.0003808 | -3.5387 |
| 213558_at | PCLO | piccolo (presynaptic cytomatrix protein) | 3.14E-05 | -3.54035 |
| 1554433_a_at | ZNF146 | zinc finger protein 146 | 1.05E-05 | -3.54121 |
| 229349_at | LIN28B | lin-28 homolog B (C. elegans) | 1.39E-06 | -3.54603 |
| 213624_at | SMPDL3A | sphingomyelin phosphodiesterase, acid-like 3A | 4.75E-06 | -3.54779 |
| 65884_at | MAN1B1 | mannosidase, alpha, class 1B, member 1 | 1.09E-05 | -3.54893 |
| 210852_s_at | AASS | aminoadipate-semialdehyde synthase | 3.51E-05 | -3.55111 |
| 214954_at | SUSD5 | sushi domain containing 5 | 0.0002066 | -3.5527 |
| 224694_at | ANTXR1 | anthrax toxin receptor 1 | 1.64E-05 | -3.55286 |
| 210286_s_at | SLC4A7 | solute carrier family 4, sodium bicarbonate cotransporter, member 7 | 6.60E-07 | -3.55407 |
| 209884_s_at | SLC4A7 | solute carrier family 4, sodium bicarbonate cotransporter, member 7 | 1.00E-05 | -3.55641 |
| 203627_at | IGF1R | insulin-like growth factor 1 receptor | 1.19E-05 | -3.55785 |
| 217989_at | HSD17B11 | hydroxysteroid (17-beta) dehydrogenase 11 | 0.0002026 | -3.55896 |
| 203476_at | TPBG | trophoblast glycoprotein | 6.27E-05 | -3.55902 |
| 221471_at | SERINC3 | serine incorporator 3 | 1.68E-06 | -3.56071 |
| 200967_at | PPIB | peptidylprolyl isomerase B (cyclophilin B) | 8.08E-07 | -3.56318 |
| 222506_at | LMBR1 | limb region 1 homolog (mouse) | 4.87E-06 | -3.56544 |
| 204273_at | EDNRB | endothelin receptor type B | 0.0002993 | -3.56748 |
| 221786_at | C6orf120 | chromosome 6 open reading frame 120 | 0.0002125 | -3.56818 |
| 225806_at | JUB | jub, ajuba homolog (Xenopus laevis) | 5.13E-06 | -3.57099 |
| 1554689_a_at | NLGN4X | neuroligin 4, X-linked | 5.28E-06 | -3.57177 |
| 212989_at | SGMS1 | sphingomyelin synthase 1 | 0.0009775 | -3.57279 |
| 201809_s_at | ENG | endoglin | 3.24E-06 | -3.57357 |
| 1558693_s_at | C1orf85 | chromosome 1 open reading frame 85 | 9.10E-05 | -3.57478 |
| 222423_at | NDFIP1 | Nedd4 family interacting protein 1 | 4.15E-06 | -3.57573 |
| 212196_at | IL6ST | interleukin 6 signal transducer (gp130, oncostatin M receptor) | 6.55E-06 | -3.57814 |
| 225387_at | TSPAN5 | tetraspanin 5 | 0.0001349 | -3.57913 |
| 209163_at | CYB561 | cytochrome b-561 | 2.98E-05 | -3.57955 |
| 204140_at | TPST1 | tyrosylprotein sulfotransferase 1 | 9.72E-06 | -3.58122 |
| 1566472_s_at | RETSAT | retinol saturase (all-trans-retinol 13,14-reductase) | 5.00E-05 | -3.58491 |
| 229274_at | GNAS | GNAS complex locus | 0.0001048 | -3.58604 |
| 201486_at | RCN2 | reticulocalbin 2, EF-hand calcium binding domain | 5.55E-06 | -3.58804 |
| 235168_at | PIGM | phosphatidylinositol glycan anchor biosynthesis, class M | 1.62E-05 | -3.59394 |
| 1557067_s_at | LUC7L | LUC7-like (S. cerevisiae) | 0.0002435 | -3.59551 |
| 1552766_at | HS6ST2 | heparan sulfate 6-O-sulfotransferase 2 | 1.56E-05 | -3.59793 |
| 205120_s_at | SGCB | sarcoglycan, beta (43kDa dystrophin-associated glycoprotein) | 5.13E-07 | -3.59828 |
| 211945_s_at | ITGB1 | integrin, beta 1 (fibronectin receptor, beta polypeptide, antigen CD29 includes | 2.05E-05 | -3.60181 |
| 225872_at | SLC35F5 | solute carrier family 35, member F5 | 0.0001747 | -3.60476 |
| 223299_at | SEC11C | SEC11 homolog C (S. cerevisiae) | 9.47E-08 | -3.60657 |
| 200922_at | KDELR1 | KDEL (Lys-Asp-Glu-Leu) endoplasmic reticulum protein retention receptor 1 | 2.14E-05 | -3.60796 |
| 206668_s_at | SCAMP1 | secretory carrier membrane protein 1 | 3.92E-05 | -3.60814 |
| 222690_s_at | TMEM39A | transmembrane protein 39A | 0.0004286 | -3.60931 |
| 218721_s_at | C1orf27 | chromosome 1 open reading frame 27 | 0.0001961 | -3.61157 |
| 225128_at | KDELC2 | KDEL (Lys-Asp-Glu-Leu) containing 2 | 6.22E-05 | -3.61184 |
| 232473_at | PRPF18 | PRP18 pre-mRNA processing factor 18 homolog (S. cerevisiae) | 1.29E-07 | -3.61249 |
| 209041_s_at | UBE2G2 | ubiquitin-conjugating enzyme E2G 2 (UBC7 homolog, yeast) | 1.10E-05 | -3.61314 |
| 212195_at | IL6ST | interleukin 6 signal transducer (gp130, oncostatin M receptor) | 1.65E-05 | -3.61429 |
| 202784_s_at | NNT | nicotinamide nucleotide transhydrogenase | 0.0006023 | -3.61663 |
| 234985_at | LDLRAD3 | low density lipoprotein receptor class A domain containing 3 | 0.0001849 | -3.61686 |
| 235927_at | XPO1 | exportin 1 (CRM1 homolog, yeast) | 4.25E-05 | -3.61753 |
| 204158_s_at | TCIRG1 | T-cell, immune regulator 1, ATPase, H+ transporting, lysosomal V0 subunit A3 | 4.81E-05 | -3.61814 |
| 226150_at | PPAPDC1B | phosphatidic acid phosphatase type 2 domain containing 1B | 1.69E-05 | -3.61859 |
| 215719_x_at | FAS | Fas (TNF receptor superfamily, member 6) | 0.0001421 | -3.61973 |
| 217788_s_at | GALNT2 | UDP-N-acetyl-alpha-D-galactosamine:polypeptide N-acetylgalactosaminyltransferase | 1.92E-05 | -3.62029 |
| 201422_at | IFI30 | interferon, gamma-inducible protein 30 | 0.0001477 | -3.62139 |
| 212022_s_at | MKI67 | antigen identified by monoclonal antibody Ki-67 | 1.09E-06 | -3.62205 |
| 205351_at | GGCX | gamma-glutamyl carboxylase | 5.75E-07 | -3.62244 |
| 238831_at | TMEM33 | transmembrane protein 33 | 0.0002973 | -3.6228 |
| 208853_s_at | CANX | calnexin | 6.22E-06 | -3.62333 |
| 222845_x_at | TMBIM4 | transmembrane BAX inhibitor motif containing 4 | 2.79E-05 | -3.62572 |
| 213017_at | ABHD3 | abhydrolase domain containing 3 | 4.92E-05 | -3.62808 |
| 202637_s_at | ICAM1 | intercellular adhesion molecule 1 | 3.32E-05 | -3.62857 |
| 219737_s_at | PCDH9 | protocadherin 9 | 6.60E-06 | -3.63047 |
| 210567_s_at | SKP2 | S-phase kinase-associated protein 2 (p45) | 6.66E-05 | -3.63077 |
| 225401_at | C1orf85 | chromosome 1 open reading frame 85 | 2.10E-06 | -3.6315 |
| 219229_at | SLCO3A1 | solute carrier organic anion transporter family, member 3A1 | 3.55E-05 | -3.63267 |
| 203583_at | UNC50 | unc-50 homolog (C. elegans) | 4.76E-06 | -3.63348 |
| 208852_s_at | CANX | calnexin | 2.25E-06 | -3.6338 |
| 224916_at | TMEM173 | transmembrane protein 173 | 0.0001517 | -3.63488 |
| 227354_at | PAG1 | phosphoprotein associated with glycosphingolipid microdomains 1 | 0.0001125 | -3.63491 |
| 208476_s_at | FRMD4A | FERM domain containing 4A | 1.35E-05 | -3.63558 |
| 210840_s_at | IQGAP1 | IQ motif containing GTPase activating protein 1 | 7.44E-07 | -3.63973 |
| 225627_s_at | CACHD1 | cache domain containing 1 | 4.68E-06 | -3.6402 |
| 201060_x_at | STOM | stomatin | 1.23E-05 | -3.64252 |
| 238475_at | ALG10B | asparagine-linked glycosylation 10, alpha-1,2-glucosyltransferase homolog B (yea | 1.40E-05 | -3.64532 |
| 205945_at | IL6R | interleukin 6 receptor | 5.70E-05 | -3.64559 |
| 222162_s_at | ADAMTS1 | ADAM metallopeptidase with thrombospondin type 1 motif, 1 | 3.06E-06 | -3.64586 |
| 227889_at | LPCAT2 | lysophosphatidylcholine acyltransferase 2 | 3.79E-06 | -3.64606 |
| 200917_s_at | SRPR | signal recognition particle receptor (docking protein) | 0.0002382 | -3.64678 |
| 204497_at | ADCY9 | adenylate cyclase 9 | 0.0001722 | -3.64782 |
| 212329_at | SCAP | SREBF chaperone | 7.17E-08 | -3.64784 |
| 1553603_s_at | ATL2 | atlastin GTPase 2 | 6.55E-05 | -3.64865 |
| 211769_x_at | SERINC3 | serine incorporator 3 | 1.80E-05 | -3.65064 |
| 225060_at | LRP11 | low density lipoprotein receptor-related protein 11 | 8.37E-06 | -3.65142 |
| 231697_s_at | TMEM49 | Transmembrane protein 49 | 4.87E-07 | -3.65384 |
| 214484_s_at | SIGMAR1 | sigma non-opioid intracellular receptor 1 | 9.87E-06 | -3.65454 |
| 201028_s_at | CD99 | CD99 molecule | 1.56E-07 | -3.65526 |
| 205097_at | SLC26A2 | solute carrier family 26 (sulfate transporter), member 2 | 1.45E-06 | -3.65756 |
| 225786_at | NCRNA00201 | non-protein coding RNA 201 | 1.37E-05 | -3.65849 |
| 206855_s_at | HYAL2 | hyaluronoglucosaminidase 2 | 3.66E-06 | -3.65866 |
| 200742_s_at | TPP1 | tripeptidyl peptidase I | 2.15E-05 | -3.65921 |
| 218249_at | ZDHHC6 | zinc finger, DHHC-type containing 6 | 2.89E-05 | -3.65941 |
| 205327_s_at | ACVR2A | activin A receptor, type IIA | 2.04E-05 | -3.65962 |
| 204271_s_at | EDNRB | endothelin receptor type B | 0.0001004 | -3.66065 |
| 1562442_a_at | SSBP1 | single-stranded DNA binding protein 1 | 6.78E-06 | -3.66201 |
| 201765_s_at | HEXA | hexosaminidase A (alpha polypeptide) | 5.47E-06 | -3.66464 |
| 201171_at | ATP6V0E1 | ATPase, H+ transporting, lysosomal 9kDa, V0 subunit e1 | 8.42E-05 | -3.66691 |
| 201561_s_at | CLSTN1 | calsyntenin 1 | 4.08E-06 | -3.66825 |
| 202062_s_at | SEL1L | sel-1 suppressor of lin-12-like (C. elegans) | 0.0001623 | -3.66878 |
| 210842_at | NRP2 | neuropilin 2 | 0.0006595 | -3.66899 |
| 200743_s_at | TPP1 | tripeptidyl peptidase I | 4.99E-07 | -3.67288 |
| 227152_at | C12orf35 | chromosome 12 open reading frame 35 | 0.0005851 | -3.67334 |
| 238419_at | PHLDB2 | pleckstrin homology-like domain, family B, member 2 | 1.84E-05 | -3.67527 |
| 233827_s_at | SUPT16H | suppressor of Ty 16 homolog (S. cerevisiae) | 0.0011768 | -3.67593 |
| 200698_at | KDELR2 | KDEL (Lys-Asp-Glu-Leu) endoplasmic reticulum protein retention receptor 2 | 1.59E-06 | -3.6797 |
| 235068_at | ZDHHC21 | zinc finger, DHHC-type containing 21 | 1.93E-05 | -3.68102 |
| 1552921_a_at | FIGNL1 | fidgetin-like 1 | 1.18E-05 | -3.68138 |
| 232636_at | SLITRK4 | SLIT and NTRK-like family, member 4 | 4.29E-06 | -3.68177 |
| 225325_at | MFSD6 | major facilitator superfamily domain containing 6 | 7.92E-06 | -3.68229 |
| 212847_at | FUBP1 | Far upstream element (FUSE) binding protein 1 | 6.50E-05 | -3.6831 |
| 203071_at | SEMA3B | sema domain, immunoglobulin domain (Ig), short basic domain, secreted, (semaphor | 3.18E-05 | -3.68597 |
| 238474_at | NUP43 | nucleoporin 43kDa | 0.0002702 | -3.68824 |
| 223714_at | ZNF256 | zinc finger protein 256 | 9.51E-05 | -3.69038 |
| 236957_at | CDCA2 | cell division cycle associated 2 | 0.0001531 | -3.69165 |
| 1564064_a_at | ATP11B | ATPase, class VI, type 11B | 3.09E-05 | -3.69202 |
| 241866_at | SLC16A7 | solute carrier family 16, member 7 (monocarboxylic acid transporter 2) | 5.79E-06 | -3.69259 |
| 227055_at | METTL7B | methyltransferase like 7B | 2.05E-05 | -3.69351 |
| 215739_s_at | TUBGCP3 | tubulin, gamma complex associated protein 3 | 3.24E-05 | -3.69783 |
| 228123_s_at | ABHD12 | abhydrolase domain containing 12 | 4.10E-06 | -3.6979 |
| 223773_s_at | SNHG12 | small nucleolar RNA host gene 12 (non-protein coding) | 3.59E-06 | -3.69812 |
| 243166_at | SLC30A5 | solute carrier family 30 (zinc transporter), member 5 | 7.96E-07 | -3.69834 |
| 210968_s_at | RTN4 | reticulon 4 | 7.17E-06 | -3.70208 |
| 208928_at | POR | P450 (cytochrome) oxidoreductase | 0.0002441 | -3.70397 |
| 226161_at | SLC30A6 | solute carrier family 30 (zinc transporter), member 6 | 0.0002028 | -3.70551 |
| 201151_s_at | MBNL1 | muscleblind-like (Drosophila) | 0.0001182 | -3.7056 |
| 210180_s_at | TRA2B | transformer 2 beta homolog (Drosophila) | 6.96E-05 | -3.7068 |
| 229453_at | PDIA3 | Protein disulfide isomerase family A, member 3 | 7.12E-08 | -3.71112 |
| 211212_s_at | ORC5L | origin recognition complex, subunit 5-like (yeast) | 0.0002123 | -3.71161 |
| 225991_at | TMEM41A | transmembrane protein 41A | 6.73E-05 | -3.71799 |
| 221646_s_at | ZDHHC11 | zinc finger, DHHC-type containing 11 | 1.74E-05 | -3.719 |
| 211284_s_at | GRN | granulin | 0.0002498 | -3.71921 |
| 201920_at | SLC20A1 | solute carrier family 20 (phosphate transporter), member 1 | 2.58E-07 | -3.72093 |
| 204333_s_at | AGA | aspartylglucosaminidase | 0.0001986 | -3.72147 |
| 235037_at | TMEM41A | transmembrane protein 41A | 7.39E-07 | -3.72215 |
| 221572_s_at | SLC26A6 | solute carrier family 26, member 6 | 0.0001078 | -3.72453 |
| 224325_at | FZD8 | frizzled homolog 8 (Drosophila) | 6.59E-06 | -3.72536 |
| 228530_at | C13orf37 | Chromosome 13 open reading frame 37 | 0.0002344 | -3.72567 |
| 239219_at | AURKB | aurora kinase B | 4.44E-05 | -3.72707 |
| 216338_s_at | YIPF3 | Yip1 domain family, member 3 | 8.83E-06 | -3.72752 |
| 200663_at | CD63 | CD63 molecule | 4.22E-08 | -3.72761 |
| 226502_at | ELMOD2 | ELMO/CED-12 domain containing 2 | 5.27E-05 | -3.72812 |
| 218587_s_at | KTELC1 | KTEL (Lys-Tyr-Glu-Leu) containing 1 | 9.24E-06 | -3.72821 |
| 212142_at | MCM4 | minichromosome maintenance complex component 4 | 0.0001651 | -3.73294 |
| 209006_s_at | C1orf63 | chromosome 1 open reading frame 63 | 0.0004146 | -3.73413 |
| 222505_at | LMBR1 | limb region 1 homolog (mouse) | 0.0001041 | -3.73476 |
| 206667_s_at | SCAMP1 | secretory carrier membrane protein 1 | 9.43E-05 | -3.7354 |
| 219237_s_at | DNAJB14 | DnaJ (Hsp40) homolog, subfamily B, member 14 | 7.47E-05 | -3.73625 |
| 1558152_at | LOC100131262 | hypothetical LOC100131262 | 0.0001049 | -3.74143 |
| 211576_s_at | SLC19A1 | solute carrier family 19 (folate transporter), member 1 | 0.0001134 | -3.74187 |
| 227370_at | FAM171B | family with sequence similarity 171, member B | 1.72E-06 | -3.74433 |
| 218237_s_at | SLC38A1 | solute carrier family 38, member 1 | 1.01E-06 | -3.74536 |
| 201780_s_at | RNF13 | ring finger protein 13 | 1.76E-05 | -3.74598 |
| 239352_at | SLC6A15 | solute carrier family 6 (neutral amino acid transporter), member 15 | 0.0009088 | -3.74681 |
| 205618_at | PRRG1 | proline rich Gla (G-carboxyglutamic acid) 1 | 6.24E-06 | -3.74691 |
| 232504_at | LOC285628 | hypothetical protein LOC285628 | 2.73E-05 | -3.74828 |
| 205535_s_at | PCDH7 | protocadherin 7 | 6.26E-05 | -3.74948 |
| 226752_at | FAM174A | family with sequence similarity 174, member A | 0.0001515 | -3.75212 |
| 218770_s_at | TMEM39B | transmembrane protein 39B | 6.47E-05 | -3.75255 |
| 212771_at | FAM171A1 | family with sequence similarity 171, member A1 | 2.84E-07 | -3.75326 |
| 222412_s_at | SSR3 | signal sequence receptor, gamma (translocon-associated protein gamma) | 0.0001115 | -3.75836 |
| 225626_at | PAG1 | phosphoprotein associated with glycosphingolipid microdomains 1 | 5.23E-05 | -3.75926 |
| 1555847_a_at | LOC284454 | hypothetical protein LOC284454 | 4.54E-06 | -3.76068 |
| 209277_at | TFPI2 | tissue factor pathway inhibitor 2 | 3.74E-06 | -3.76075 |
| 1554726_at | ZNF655 | zinc finger protein 655 | 0.0001021 | -3.76327 |
| 226216_at | INSR | insulin receptor | 9.65E-05 | -3.76577 |
| 221060_s_at | TLR4 | toll-like receptor 4 | 7.85E-05 | -3.76757 |
| 227059_at | GPC6 | glypican 6 | 0.0007739 | -3.77325 |
| 221853_s_at | NOMO1 /// NOMO2 /// NOMO3 | NODAL modulator 1 /// NODAL modulator 2 /// NODAL modulator 3 | 0.0009018 | -3.77422 |
| 228505_s_at | TMEM170A | transmembrane protein 170A | 0.0001508 | -3.77539 |
| 209420_s_at | SMPD1 | sphingomyelin phosphodiesterase 1, acid lysosomal | 0.0001419 | -3.77672 |
| 226213_at | ERBB3 | v-erb-b2 erythroblastic leukemia viral oncogene homolog 3 (avian) | 6.48E-06 | -3.77869 |
| 223566_s_at | BCOR | BCL6 co-repressor | 1.13E-06 | -3.77964 |
| 219134_at | ELTD1 | EGF, latrophilin and seven transmembrane domain containing 1 | 2.61E-05 | -3.78012 |
| 235775_at | TMTC2 | transmembrane and tetratricopeptide repeat containing 2 | 0.000439 | -3.78043 |
| 225330_at | IGF1R | insulin-like growth factor 1 receptor | 2.95E-05 | -3.7806 |
| 200931_s_at | VCL | vinculin | 1.28E-05 | -3.78138 |
| 1555325_s_at | LOC100287515 /// ZNF26 | similar to zinc finger protein 26 (KOX 20) /// zinc finger protein 26 | 4.68E-05 | -3.78299 |
| 204554_at | PPP1R3D | protein phosphatase 1, regulatory (inhibitor) subunit 3D | 0.0001699 | -3.78392 |
| 203628_at | IGF1R | insulin-like growth factor 1 receptor | 0.0002938 | -3.78471 |
| 219274_at | TSPAN12 | tetraspanin 12 | 0.0001044 | -3.78619 |
| 204087_s_at | SLC5A6 | solute carrier family 5 (sodium-dependent vitamin transporter), member 6 | 1.06E-05 | -3.78635 |
| 201259_s_at | SYPL1 | synaptophysin-like 1 | 4.03E-07 | -3.78682 |
| 244439_at | SPRED1 | sprouty-related, EVH1 domain containing 1 | 6.32E-05 | -3.78895 |
| 228834_at | TOB1 | transducer of ERBB2, 1 | 6.80E-05 | -3.78992 |
| 220549_at | RAD54B | RAD54 homolog B (S. cerevisiae) | 3.12E-07 | -3.79098 |
| 205704_s_at | ATP6V0A2 | ATPase, H+ transporting, lysosomal V0 subunit a2 | 0.0001781 | -3.79332 |
| 217317_s_at | HERC2P2 /// HERC2P3 /// LOC440248 | hect domain and RLD 2 pseudogene 2 /// hect domain and RLD 2 pseudogene 3 /// he | 2.77E-06 | -3.79456 |
| 226360_at | ZNRF3 | zinc and ring finger 3 | 2.24E-05 | -3.79456 |
| 209179_s_at | MBOAT7 | membrane bound O-acyltransferase domain containing 7 | 8.54E-05 | -3.79547 |
| 209935_at | ATP2C1 | ATPase, Ca++ transporting, type 2C, member 1 | 0.0008848 | -3.79675 |
| 206500_s_at | C14orf106 | chromosome 14 open reading frame 106 | 0.0001882 | -3.7973 |
| 222235_s_at | CSGALNACT2 /// RP1-19N1.1 | chondroitin sulfate N-acetylgalactosaminyltransferase 2 /// novel protein simila | 4.87E-05 | -3.80273 |
| 1557081_at | RBM25 | RNA binding motif protein 25 | 8.98E-07 | -3.80285 |
| 242429_at | ZNF567 | zinc finger protein 567 | 0.0002895 | -3.80297 |
| 219296_at | ZDHHC13 | zinc finger, DHHC-type containing 13 | 1.08E-05 | -3.80387 |
| 203069_at | SV2A | synaptic vesicle glycoprotein 2A | 5.39E-07 | -3.80607 |
| 212890_at | SLC38A10 | solute carrier family 38, member 10 | 8.96E-08 | -3.80664 |
| 202805_s_at | ABCC1 | ATP-binding cassette, sub-family C (CFTR/MRP), member 1 | 8.76E-06 | -3.80755 |
| 210387_at | HIST1H2BG | histone cluster 1, H2bg | 1.17E-05 | -3.80872 |
| 211934_x_at | GANAB | glucosidase, alpha; neutral AB | 1.76E-06 | -3.80932 |
| 201724_s_at | GALNT1 | UDP-N-acetyl-alpha-D-galactosamine:polypeptide N-acetylgalactosaminyltransferase | 1.45E-06 | -3.81517 |
| 223723_at | MFI2 | antigen p97 (melanoma associated) identified by monoclonal antibodies 133.2 and | 2.92E-06 | -3.82091 |
| 218804_at | ANO1 | anoctamin 1, calcium activated chloride channel | 1.48E-05 | -3.82205 |
| 210697_at | ZNF257 | zinc finger protein 257 | 3.55E-06 | -3.82266 |
| 1554557_at | ATP11B | ATPase, class VI, type 11B | 2.27E-05 | -3.82341 |
| 205858_at | NGFR | nerve growth factor receptor (TNFR superfamily, member 16) | 1.21E-06 | -3.82405 |
| 1569142_at | TRIM13 | tripartite motif-containing 13 | 0.0016882 | -3.82784 |
| 203028_s_at | CYBA | cytochrome b-245, alpha polypeptide | 1.11E-05 | -3.82828 |
| 207012_at | MMP16 | matrix metallopeptidase 16 (membrane-inserted) | 2.45E-05 | -3.83614 |
| 209655_s_at | TMEM47 | transmembrane protein 47 | 2.44E-05 | -3.83616 |
| 240172_at | ERGIC2 | ERGIC and golgi 2 | 0.0001631 | -3.83685 |
| 223017_at | TXNDC12 | thioredoxin domain containing 12 (endoplasmic reticulum) | 4.29E-09 | -3.8381 |
| 203090_at | SDF2 | stromal cell-derived factor 2 | 2.36E-05 | -3.83917 |
| 223173_at | SPNS1 | spinster homolog 1 (Drosophila) | 4.69E-05 | -3.83989 |
| 206695_x_at | ZNF43 | zinc finger protein 43 | 0.0001002 | -3.84475 |
| 205891_at | ADORA2B | adenosine A2b receptor | 4.15E-07 | -3.8458 |
| 36566_at | CTNS | cystinosis, nephropathic | 1.13E-06 | -3.84734 |
| 212667_at | SPARC | secreted protein, acidic, cysteine-rich (osteonectin) | 3.13E-06 | -3.84864 |
| 231534_at | CDC2 | Cell division cycle 2, G1 to S and G2 to M | 2.53E-05 | -3.84906 |
| 242146_at | SNRPA1 | Small nuclear ribonucleoprotein polypeptide A' | 0.0003126 | -3.8531 |
| 1564063_a_at | ATP11B | ATPase, class VI, type 11B | 2.31E-05 | -3.85383 |
| 221274_s_at | LMAN2L | lectin, mannose-binding 2-like | 5.50E-08 | -3.85745 |
| 202064_s_at | SEL1L | sel-1 suppressor of lin-12-like (C. elegans) | 9.22E-05 | -3.85811 |
| 203854_at | CFI | complement factor I | 4.59E-06 | -3.86525 |
| 209294_x_at | TNFRSF10B | tumor necrosis factor receptor superfamily, member 10b | 1.59E-05 | -3.86561 |
| 220091_at | SLC2A6 | solute carrier family 2 (facilitated glucose transporter), member 6 | 5.71E-05 | -3.86652 |
| 221844_x_at | SPCS3 | signal peptidase complex subunit 3 homolog (S. cerevisiae) | 3.64E-05 | -3.87157 |
| 228635_at | PCDH10 | protocadherin 10 | 8.76E-07 | -3.87199 |
| 211967_at | TMEM123 | transmembrane protein 123 | 6.08E-06 | -3.87248 |
| 1554452_a_at | C7orf68 | chromosome 7 open reading frame 68 | 2.49E-05 | -3.87346 |
| 201887_at | IL13RA1 | interleukin 13 receptor, alpha 1 | 1.74E-05 | -3.87438 |
| 222088_s_at | SLC2A14 /// SLC2A3 | solute carrier family 2 (facilitated glucose transporter), member 14 /// solute | 2.37E-05 | -3.87493 |
| 213436_at | CNR1 | cannabinoid receptor 1 (brain) | 0.000107 | -3.8752 |
| 213689_x_at | FAM69A | family with sequence similarity 69, member A | 0.0003367 | -3.8753 |
| 203741_s_at | ADCY7 | adenylate cyclase 7 | 6.58E-05 | -3.8772 |
| 219460_s_at | TMEM127 | transmembrane protein 127 | 2.51E-05 | -3.87938 |
| 218902_at | NOTCH1 | Notch homolog 1, translocation-associated (Drosophila) | 4.16E-05 | -3.87978 |
| 215193_x_at | HLA-DRB1 /// HLA-DRB3 /// HLA-DRB4 | major histocompatibility complex, class II, DR beta 1 /// major histocompatibili | 4.38E-06 | -3.88653 |
| 217855_x_at | SDF4 | stromal cell derived factor 4 | 4.29E-06 | -3.89138 |
| 238797_at | TRIM11 | tripartite motif-containing 11 | 4.99E-06 | -3.894 |
| 207265_s_at | KDELR3 | KDEL (Lys-Asp-Glu-Leu) endoplasmic reticulum protein retention receptor 3 | 6.72E-08 | -3.89521 |
| 228584_at | SGCB | sarcoglycan, beta (43kDa dystrophin-associated glycoprotein) | 0.0003324 | -3.89536 |
| 1553928_at | ELMOD2 | ELMO/CED-12 domain containing 2 | 6.62E-06 | -3.8967 |
| 209236_at | SLC23A2 | solute carrier family 23 (nucleobase transporters), member 2 | 1.10E-05 | -3.89829 |
| 212226_s_at | PPAP2B | phosphatidic acid phosphatase type 2B | 3.06E-06 | -3.89975 |
| 224610_at | SNHG1 | small nucleolar RNA host gene 1 (non-protein coding) | 1.29E-06 | -3.9 |
| 224639_at | UNQ1887 | signal peptide peptidase 3 | 2.90E-05 | -3.90149 |
| 229574_at | TRA2A | transformer 2 alpha homolog (Drosophila) | 6.55E-06 | -3.90223 |
| 230964_at | FREM2 | FRAS1 related extracellular matrix protein 2 | 2.15E-05 | -3.90451 |
| 223073_at | HIATL1 | hippocampus abundant transcript-like 1 | 3.47E-05 | -3.90744 |
| 228455_at | RBM15 | RNA binding motif protein 15 | 9.33E-06 | -3.90748 |
| 51146_at | PIGV | phosphatidylinositol glycan anchor biosynthesis, class V | 3.39E-05 | -3.90803 |
| 226747_at | TXNDC16 | thioredoxin domain containing 16 | 2.52E-06 | -3.91331 |
| 201625_s_at | INSIG1 | insulin induced gene 1 | 0.0002159 | -3.91419 |
| 225288_at | COL27A1 | collagen, type XXVII, alpha 1 | 3.94E-05 | -3.9142 |
| 218124_at | RETSAT | retinol saturase (all-trans-retinol 13,14-reductase) | 2.30E-06 | -3.91441 |
| 203306_s_at | SLC35A1 | solute carrier family 35 (CMP-sialic acid transporter), member A1 | 0.0002619 | -3.91501 |
| 209086_x_at | MCAM | melanoma cell adhesion molecule | 0.0003653 | -3.91864 |
| 223355_at | ALG1 | asparagine-linked glycosylation 1, beta-1,4-mannosyltransferase homolog (S. cere | 9.87E-06 | -3.91871 |
| 211977_at | GPR107 | G protein-coupled receptor 107 | 2.16E-07 | -3.9204 |
| 224906_at | ANO6 | anoctamin 6 | 1.59E-05 | -3.92421 |
| 220265_at | GPR107 | G protein-coupled receptor 107 | 3.90E-05 | -3.925 |
| 220178_at | C19orf28 | chromosome 19 open reading frame 28 | 3.20E-05 | -3.92935 |
| 212349_at | POFUT1 | protein O-fucosyltransferase 1 | 7.49E-06 | -3.92939 |
| 207111_at | EMR1 | egf-like module containing, mucin-like, hormone receptor-like 1 | 4.94E-05 | -3.93127 |
| 223391_at | SGPP1 | sphingosine-1-phosphate phosphatase 1 | 4.13E-05 | -3.93732 |
| 200838_at | CTSB | cathepsin B | 5.22E-06 | -3.93774 |
| 224453_s_at | ETNK1 | ethanolamine kinase 1 | 2.36E-05 | -3.94236 |
| 223925_s_at | MTPN | myotrophin | 7.62E-05 | -3.94306 |
| 217225_x_at | NOMO1 /// NOMO2 /// NOMO3 | NODAL modulator 1 /// NODAL modulator 2 /// NODAL modulator 3 | 5.73E-07 | -3.94415 |
| 204469_at | PTPRZ1 | protein tyrosine phosphatase, receptor-type, Z polypeptide 1 | 6.79E-05 | -3.94687 |
| 224724_at | SULF2 | sulfatase 2 | 0.0003391 | -3.95341 |
| 212136_at | ATP2B4 | ATPase, Ca++ transporting, plasma membrane 4 | 1.51E-05 | -3.95529 |
| 200602_at | APP | amyloid beta (A4) precursor protein | 0.0002843 | -3.9581 |
| 212106_at | FAF2 | Fas associated factor family member 2 | 0.0003407 | -3.95961 |
| 201722_s_at | GALNT1 | UDP-N-acetyl-alpha-D-galactosamine:polypeptide N-acetylgalactosaminyltransferase | 6.31E-06 | -3.96068 |
| 203659_s_at | TRIM13 | tripartite motif-containing 13 | 6.79E-06 | -3.96134 |
| 227861_at | TMEM161B | transmembrane protein 161B | 5.15E-05 | -3.96214 |
| 217188_s_at | C14orf1 | chromosome 14 open reading frame 1 | 2.79E-06 | -3.96317 |
| 209897_s_at | SLIT2 | slit homolog 2 (Drosophila) | 5.03E-06 | -3.96431 |
| 202419_at | KDSR | 3-ketodihydrosphingosine reductase | 8.60E-06 | -3.96689 |
| 227223_at | LOC643167 /// RBM39 | similar to RNA binding motif protein 39 /// RNA binding motif protein 39 | 6.63E-05 | -3.96776 |
| 218203_at | ALG5 | asparagine-linked glycosylation 5, dolichyl-phosphate beta-glucosyltransferase h | 1.84E-05 | -3.96882 |
| 200968_s_at | PPIB | peptidylprolyl isomerase B (cyclophilin B) | 4.34E-07 | -3.96957 |
| 214291_at | LOC729046 /// RPL17 | similar to ribosomal protein L17 /// ribosomal protein L17 | 5.16E-05 | -3.97141 |
| 208621_s_at | EZR | ezrin | 0.0002751 | -3.97171 |
| 203688_at | PKD2 | polycystic kidney disease 2 (autosomal dominant) | 4.36E-05 | -3.97599 |
| 1554037_a_at | ZBTB24 | zinc finger and BTB domain containing 24 | 2.92E-05 | -3.97635 |
| 227100_at | B3GALTL | beta 1,3-galactosyltransferase-like | 2.74E-05 | -3.97641 |
| 210674_s_at | PCDHA1 /// PCDHA10 /// PCDHA11 /// PCDHA12 /// PCDHA13 /// PCDHA2 /// PCDHA3 /// PCDHA4 /// PCDHA5 /// PCDHA6 /// PCDHA7 /// PCDHA8 /// PCDHA9 /// PCDHAC1 /// PCDHAC2 | protocadherin alpha 1 /// protocadherin alpha 10 /// protocadherin alpha 11 /// | 4.23E-05 | -3.97683 |
| 212135_s_at | ATP2B4 | ATPase, Ca++ transporting, plasma membrane 4 | 2.49E-06 | -3.97772 |
| 212662_at | PVR | poliovirus receptor | 4.05E-06 | -3.98108 |
| 210756_s_at | NOTCH2 | Notch homolog 2 (Drosophila) | 6.01E-07 | -3.98243 |
| 201446_s_at | TIA1 | TIA1 cytotoxic granule-associated RNA binding protein | 0.0001707 | -3.98265 |
| 236249_at | IKIP | IKK interacting protein | 0.0002012 | -3.98278 |
| 201916_s_at | SEC63 | SEC63 homolog (S. cerevisiae) | 3.40E-05 | -3.98627 |
| 208890_s_at | PLXNB2 | plexin B2 | 4.48E-07 | -3.989 |
| 1554451_s_at | DNAJC14 | DnaJ (Hsp40) homolog, subfamily C, member 14 | 5.46E-05 | -3.99468 |
| 213227_at | PGRMC2 | progesterone receptor membrane component 2 | 5.71E-08 | -3.9947 |
| 239596_at | SLC30A7 | solute carrier family 30 (zinc transporter), member 7 | 0.0002272 | -3.99568 |
| 217983_s_at | RNASET2 | ribonuclease T2 | 1.39E-05 | -3.99971 |
| 235197_s_at | OSTM1 | osteopetrosis associated transmembrane protein 1 | 0.0001846 | -4.00011 |
| 203510_at | MET | met proto-oncogene (hepatocyte growth factor receptor) | 8.04E-06 | -4.00037 |
| 225582_at | ITPRIP | inositol 1,4,5-triphosphate receptor interacting protein | 7.24E-05 | -4.00142 |
| 200999_s_at | CKAP4 | cytoskeleton-associated protein 4 | 0.0002235 | -4.0025 |
| 200805_at | LMAN2 | lectin, mannose-binding 2 | 1.40E-05 | -4.00256 |
| 212045_at | GLG1 | golgi apparatus protein 1 | 2.66E-06 | -4.00327 |
| 224582_s_at | NUCKS1 | nuclear casein kinase and cyclin-dependent kinase substrate 1 | 3.19E-05 | -4.0033 |
| 208703_s_at | APLP2 | amyloid beta (A4) precursor-like protein 2 | 3.78E-06 | -4.00439 |
| 204975_at | EMP2 | epithelial membrane protein 2 | 2.63E-07 | -4.00473 |
| 209788_s_at | ERAP1 | endoplasmic reticulum aminopeptidase 1 | 5.99E-05 | -4.01584 |
| 212468_at | SPAG9 | sperm associated antigen 9 | 1.15E-05 | -4.01604 |
| 217787_s_at | GALNT2 | UDP-N-acetyl-alpha-D-galactosamine:polypeptide N-acetylgalactosaminyltransferase | 1.58E-05 | -4.01825 |
| 211844_s_at | NRP2 | neuropilin 2 | 1.21E-05 | -4.02298 |
| 229839_at | SCARA5 | Scavenger receptor class A, member 5 (putative) | 0.0001399 | -4.02552 |
| 226609_at | DCBLD1 | discoidin, CUB and LCCL domain containing 1 | 6.54E-05 | -4.0318 |
| 226908_at | LRIG3 | leucine-rich repeats and immunoglobulin-like domains 3 | 8.25E-05 | -4.03413 |
| 229900_at | CD109 | CD109 molecule | 9.20E-06 | -4.03549 |
| 213123_at | MFAP3 | microfibrillar-associated protein 3 | 1.59E-05 | -4.03617 |
| 230272_at | LOC645323 | hypothetical LOC645323 | 1.81E-06 | -4.03934 |
| 202349_at | TOR1A | torsin family 1, member A (torsin A) | 3.67E-06 | -4.04299 |
| 212200_at | ANKLE2 | ankyrin repeat and LEM domain containing 2 | 6.96E-06 | -4.04436 |
| 1557918_s_at | SLC16A1 | solute carrier family 16, member 1 (monocarboxylic acid transporter 1) | 0.0001197 | -4.04487 |
| 203528_at | SEMA4D | sema domain, immunoglobulin domain (Ig), transmembrane domain (TM) and short cyt | 2.08E-05 | -4.04779 |
| 214198_s_at | DGCR2 | DiGeorge syndrome critical region gene 2 | 4.16E-06 | -4.05711 |
| 232032_x_at | SDF4 | stromal cell derived factor 4 | 3.55E-06 | -4.05787 |
| 201399_s_at | TRAM1 | translocation associated membrane protein 1 | 2.42E-05 | -4.05962 |
| 204379_s_at | FGFR3 | fibroblast growth factor receptor 3 | 4.57E-06 | -4.0609 |
| 209380_s_at | ABCC5 | ATP-binding cassette, sub-family C (CFTR/MRP), member 5 | 2.03E-05 | -4.06128 |
| 225101_s_at | SNX14 | sorting nexin 14 | 0.0001153 | -4.06247 |
| 223047_at | CMTM6 | CKLF-like MARVEL transmembrane domain containing 6 | 4.99E-06 | -4.0655 |
| 208757_at | TMED9 | transmembrane emp24 protein transport domain containing 9 | 1.10E-06 | -4.06819 |
| 1558093_s_at | MATR3 | matrin 3 | 0.0001397 | -4.06932 |
| 1554482_a_at | SAR1B | SAR1 homolog B (S. cerevisiae) | 2.80E-05 | -4.07049 |
| 204160_s_at | ENPP4 | ectonucleotide pyrophosphatase/phosphodiesterase 4 (putative function) | 0.0003294 | -4.07294 |
| 226274_at | CLCN5 | chloride channel 5 | 5.84E-05 | -4.07552 |
| 200866_s_at | PSAP | prosaposin | 0.000718 | -4.07827 |
| 223774_at | SNHG12 | small nucleolar RNA host gene 12 (non-protein coding) | 2.47E-06 | -4.08135 |
| 205953_at | LRIG2 | leucine-rich repeats and immunoglobulin-like domains 2 | 3.89E-06 | -4.08345 |
| 209007_s_at | C1orf63 | chromosome 1 open reading frame 63 | 4.48E-05 | -4.08671 |
| 217731_s_at | ITM2B | integral membrane protein 2B | 1.77E-05 | -4.08723 |
| 219654_at | PTPLA | protein tyrosine phosphatase-like (proline instead of catalytic arginine), membe | 5.62E-06 | -4.08762 |
| 241425_at | NUPL1 | Nucleoporin like 1 | 4.10E-07 | -4.08887 |
| 205798_at | IL7R | interleukin 7 receptor | 0.0001121 | -4.09892 |
| 225850_at | SFT2D1 | SFT2 domain containing 1 | 5.59E-06 | -4.09955 |
| 205015_s_at | TGFA | transforming growth factor, alpha | 4.40E-05 | -4.10087 |
| 225316_at | MFSD2 | major facilitator superfamily domain containing 2 | 4.21E-06 | -4.10156 |
| 217437_s_at | TACC1 | transforming, acidic coiled-coil containing protein 1 | 0.0001843 | -4.10496 |
| 222752_s_at | TMEM206 | transmembrane protein 206 | 1.78E-07 | -4.10643 |
| 208873_s_at | REEP5 | receptor accessory protein 5 | 2.61E-05 | -4.10998 |
| 211090_s_at | PRPF4B | PRP4 pre-mRNA processing factor 4 homolog B (yeast) | 0.0010906 | -4.1138 |
| 201620_at | MBTPS1 | membrane-bound transcription factor peptidase, site 1 | 9.20E-08 | -4.11603 |
| 210007_s_at | GPD2 | glycerol-3-phosphate dehydrogenase 2 (mitochondrial) | 7.11E-05 | -4.11652 |
| 226686_at | CISD2 | CDGSH iron sulfur domain 2 | 0.0018005 | -4.11895 |
| 222568_at | UGGT1 | UDP-glucose glycoprotein glucosyltransferase 1 | 3.42E-05 | -4.1194 |
| 221542_s_at | ERLIN2 | ER lipid raft associated 2 | 7.03E-09 | -4.12224 |
| 224850_at | ATAD1 | ATPase family, AAA domain containing 1 | 2.12E-05 | -4.13208 |
| 218313_s_at | GALNT7 | UDP-N-acetyl-alpha-D-galactosamine:polypeptide N-acetylgalactosaminyltransferase | 1.26E-05 | -4.1336 |
| 201576_s_at | GLB1 | galactosidase, beta 1 | 3.79E-06 | -4.1339 |
| 1552977_a_at | CNPY3 | canopy 3 homolog (zebrafish) | 2.45E-05 | -4.13495 |
| 205876_at | LIFR | leukemia inhibitory factor receptor alpha | 1.39E-05 | -4.13617 |
| 1552978_a_at | SCAMP1 | secretory carrier membrane protein 1 | 9.77E-05 | -4.13626 |
| 220948_s_at | ATP1A1 | ATPase, Na+/K+ transporting, alpha 1 polypeptide | 3.54E-07 | -4.13735 |
| 1555247_a_at | RAPGEF6 | Rap guanine nucleotide exchange factor (GEF) 6 | 0.0003302 | -4.13938 |
| 202297_s_at | RER1 | RER1 retention in endoplasmic reticulum 1 homolog (S. cerevisiae) | 1.24E-06 | -4.13948 |
| 225881_at | SLC35B4 | solute carrier family 35, member B4 | 3.18E-05 | -4.14584 |
| 209712_at | SLC35D1 | solute carrier family 35 (UDP-glucuronic acid/UDP-N-acetylgalactosamine dual tra | 0.0002552 | -4.15352 |
| 221909_at | RNFT2 | ring finger protein, transmembrane 2 | 1.70E-08 | -4.15537 |
| 201888_s_at | IL13RA1 | interleukin 13 receptor, alpha 1 | 2.12E-05 | -4.15925 |
| 229126_at | TMEM19 | transmembrane protein 19 | 3.63E-06 | -4.15934 |
| 204017_at | KDELR3 | KDEL (Lys-Asp-Glu-Leu) endoplasmic reticulum protein retention receptor 3 | 2.08E-05 | -4.16 |
| 202636_at | RNF103 | ring finger protein 103 | 0.0003162 | -4.16012 |
| 212685_s_at | TBL2 | transducin (beta)-like 2 | 0.0001364 | -4.16665 |
| 204780_s_at | FAS | Fas (TNF receptor superfamily, member 6) | 1.72E-07 | -4.16668 |
| 215190_at | EIF3M | eukaryotic translation initiation factor 3, subunit M | 1.37E-05 | -4.16682 |
| 1556006_s_at | CSNK1A1 | Casein kinase 1, alpha 1 | 3.36E-05 | -4.16684 |
| 219600_s_at | TMEM50B | transmembrane protein 50B | 3.32E-05 | -4.17261 |
| 226334_s_at | AHSA2 | AHA1, activator of heat shock 90kDa protein ATPase homolog 2 (yeast) | 1.57E-06 | -4.17283 |
| 222552_at | GOLT1B | golgi transport 1 homolog B (S. cerevisiae) | 6.59E-06 | -4.17316 |
| 209607_x_at | SULT1A3 /// SULT1A4 | sulfotransferase family, cytosolic, 1A, phenol-preferring, member 3 /// sulfotra | 1.56E-05 | -4.17368 |
| 226318_at | TBRG1 | transforming growth factor beta regulator 1 | 0.0001793 | -4.1752 |
| 1552680_a_at | CASC5 | cancer susceptibility candidate 5 | 8.12E-05 | -4.17555 |
| 212108_at | FAF2 | Fas associated factor family member 2 | 1.70E-05 | -4.17646 |
| 201790_s_at | DHCR7 | 7-dehydrocholesterol reductase | 0.000197 | -4.17666 |
| 201889_at | FAM3C | family with sequence similarity 3, member C | 0.0001609 | -4.17917 |
| 210904_s_at | IL13RA1 | interleukin 13 receptor, alpha 1 | 1.95E-06 | -4.18449 |
| 1555938_x_at | VIM | vimentin | 0.0001662 | -4.18595 |
| 201859_at | SRGN | serglycin | 2.08E-05 | -4.18643 |
| 205896_at | SLC22A4 | solute carrier family 22 (organic cation/ergothioneine transporter), member 4 | 1.10E-05 | -4.18865 |
| 206756_at | CHST7 | carbohydrate (N-acetylglucosamine 6-O) sulfotransferase 7 | 1.20E-05 | -4.19473 |
| 207808_s_at | PROS1 | protein S (alpha) | 1.38E-06 | -4.19618 |
| 217678_at | SLC7A11 | solute carrier family 7, (cationic amino acid transporter, y+ system) member 11 | 0.0001164 | -4.19677 |
| 222422_s_at | NDFIP1 | Nedd4 family interacting protein 1 | 5.66E-06 | -4.19839 |
| 219117_s_at | FKBP11 | FK506 binding protein 11, 19 kDa | 5.54E-07 | -4.19877 |
| 229221_at | CD44 | CD44 molecule (Indian blood group) | 1.99E-05 | -4.20089 |
| 202454_s_at | ERBB3 | v-erb-b2 erythroblastic leukemia viral oncogene homolog 3 (avian) | 0.0012 | -4.20162 |
| 225420_at | GPAM | glycerol-3-phosphate acyltransferase, mitochondrial | 0.0001759 | -4.2033 |
| 225180_at | TTC14 | tetratricopeptide repeat domain 14 | 0.0001552 | -4.2038 |
| 39248_at | AQP3 | aquaporin 3 (Gill blood group) | 2.39E-05 | -4.20889 |
| 212982_at | ZDHHC17 | zinc finger, DHHC-type containing 17 | 3.83E-06 | -4.20931 |
| 228314_at | LRRC8C | leucine rich repeat containing 8 family, member C | 2.54E-05 | -4.21036 |
| 223470_at | PIGM | phosphatidylinositol glycan anchor biosynthesis, class M | 0.0004127 | -4.21926 |
| 209575_at | IL10RB | interleukin 10 receptor, beta | 0.0001336 | -4.22134 |
| 238010_at | C1orf174 | chromosome 1 open reading frame 174 | 2.58E-05 | -4.22269 |
| 209708_at | MOXD1 | monooxygenase, DBH-like 1 | 3.93E-05 | -4.2227 |
| 219558_at | ATP13A3 | ATPase type 13A3 | 8.89E-06 | -4.22392 |
| 229509_at | MFSD8 | major facilitator superfamily domain containing 8 | 0.0001295 | -4.22461 |
| 218191_s_at | LMBRD1 | LMBR1 domain containing 1 | 0.0001135 | -4.22468 |
| 225218_at | ZFYVE27 | zinc finger, FYVE domain containing 27 | 2.45E-05 | -4.22799 |
| 223991_s_at | GALNT2 /// LOC100132910 | UDP-N-acetyl-alpha-D-galactosamine:polypeptide N-acetylgalactosaminyltransferase | 4.91E-07 | -4.22803 |
| 216593_s_at | LOC100289848 /// PIGC | similar to phosphatidylinositol glycan, class C /// phosphatidylinositol glycan | 7.20E-06 | -4.22921 |
| 203851_at | IGFBP6 | insulin-like growth factor binding protein 6 | 1.64E-05 | -4.23165 |
| 214913_at | ADAMTS3 | ADAM metallopeptidase with thrombospondin type 1 motif, 3 | 3.59E-05 | -4.23242 |
| 202185_at | PLOD3 | procollagen-lysine, 2-oxoglutarate 5-dioxygenase 3 | 3.34E-06 | -4.23636 |
| 223218_s_at | NFKBIZ | nuclear factor of kappa light polypeptide gene enhancer in B-cells inhibitor, ze | 2.78E-05 | -4.2382 |
| 222750_s_at | SRD5A3 | steroid 5 alpha-reductase 3 | 2.75E-06 | -4.23849 |
| 216231_s_at | B2M | beta-2-microglobulin | 4.85E-08 | -4.24355 |
| 219952_s_at | MCOLN1 | mucolipin 1 | 1.16E-05 | -4.24413 |
| 218326_s_at | LGR4 | leucine-rich repeat-containing G protein-coupled receptor 4 | 0.0002376 | -4.24543 |
| 206533_at | CHRNA5 | cholinergic receptor, nicotinic, alpha 5 | 3.12E-05 | -4.24563 |
| 212813_at | JAM3 | junctional adhesion molecule 3 | 6.21E-06 | -4.24652 |
| 200670_at | XBP1 | X-box binding protein 1 | 0.0013667 | -4.24969 |
| 224698_at | ESYT2 | extended synaptotagmin-like protein 2 | 1.21E-05 | -4.25689 |
| 212105_s_at | DHX9 | DEAH (Asp-Glu-Ala-His) box polypeptide 9 | 0.0002117 | -4.25864 |
| 204332_s_at | AGA | aspartylglucosaminidase | 2.23E-05 | -4.2594 |
| 221552_at | ABHD6 | abhydrolase domain containing 6 | 6.87E-07 | -4.26626 |
| 202655_at | MANF | mesencephalic astrocyte-derived neurotrophic factor | 3.40E-07 | -4.27359 |
| 219924_s_at | ZMYM6 | zinc finger, MYM-type 6 | 1.94E-05 | -4.27607 |
| 227449_at | EPHA4 | EPH receptor A4 | 1.35E-06 | -4.27673 |
| 225621_at | ALG2 | asparagine-linked glycosylation 2, alpha-1,3-mannosyltransferase homolog (S. cer | 6.29E-05 | -4.27974 |
| 224589_at | XIST | X (inactive)-specific transcript (non-protein coding) | 1.26E-05 | -4.28711 |
| 200839_s_at | CTSB | cathepsin B | 5.00E-06 | -4.2877 |
| 201505_at | LAMB1 | laminin, beta 1 | 8.78E-05 | -4.28787 |
| 222979_s_at | SURF4 | surfeit 4 | 0.001068 | -4.28832 |
| 215983_s_at | UBXN8 | UBX domain protein 8 | 1.28E-05 | -4.29067 |
| 222206_s_at | NCLN | nicalin homolog (zebrafish) | 8.70E-06 | -4.29308 |
| 210405_x_at | TNFRSF10B | tumor necrosis factor receptor superfamily, member 10b | 0.0005964 | -4.29436 |
| 212290_at | SLC7A1 | solute carrier family 7 (cationic amino acid transporter, y+ system), member 1 | 4.75E-07 | -4.29553 |
| 227277_at | MTDH | metadherin | 1.80E-05 | -4.30494 |
| 226483_at | TMEM68 | transmembrane protein 68 | 3.88E-05 | -4.30527 |
| 223254_s_at | G2E3 | G2/M-phase specific E3 ubiquitin ligase | 2.93E-05 | -4.30595 |
| 212070_at | GPR56 | G protein-coupled receptor 56 | 4.84E-08 | -4.30604 |
| 222735_at | TMEM38B | transmembrane protein 38B | 5.08E-07 | -4.30687 |
| 203024_s_at | C5orf15 | chromosome 5 open reading frame 15 | 5.27E-06 | -4.31177 |
| 224410_s_at | LMBR1 | limb region 1 homolog (mouse) | 0.0001054 | -4.31648 |
| 201162_at | IGFBP7 | insulin-like growth factor binding protein 7 | 9.08E-08 | -4.31706 |
| 219401_at | XYLT2 | xylosyltransferase II | 5.66E-07 | -4.32147 |
| 203440_at | CDH2 | cadherin 2, type 1, N-cadherin (neuronal) | 8.22E-06 | -4.3217 |
| 200827_at | PLOD1 | procollagen-lysine 1, 2-oxoglutarate 5-dioxygenase 1 | 2.69E-05 | -4.32581 |
| 229225_at | NRP2 | neuropilin 2 | 9.24E-06 | -4.32622 |
| 200678_x_at | GRN | granulin | 4.75E-05 | -4.32974 |
| 208721_s_at | ANAPC5 | anaphase promoting complex subunit 5 | 2.97E-05 | -4.33077 |
| 244623_at | KCNQ5 | potassium voltage-gated channel, KQT-like subfamily, member 5 | 4.86E-05 | -4.3318 |
| 212628_at | PKN2 | protein kinase N2 | 1.56E-06 | -4.33274 |
| 1552790_a_at | SEC62 | SEC62 homolog (S. cerevisiae) | 6.84E-05 | -4.33412 |
| 227961_at | CTSB | cathepsin B | 1.84E-06 | -4.33455 |
| 238653_at | LRIG2 | Leucine-rich repeats and immunoglobulin-like domains 2 | 1.69E-05 | -4.33671 |
| 222573_s_at | SAV1 | salvador homolog 1 (Drosophila) | 0.0002647 | -4.33717 |
| 225174_at | DNAJC10 | DnaJ (Hsp40) homolog, subfamily C, member 10 | 0.0001241 | -4.3379 |
| 202979_s_at | CREBZF | CREB/ATF bZIP transcription factor | 0.0003059 | -4.34056 |
| 205739_x_at | ZNF107 | zinc finger protein 107 | 1.61E-05 | -4.34209 |
| 226640_at | DAGLB | diacylglycerol lipase, beta | 4.34E-05 | -4.3424 |
| 208937_s_at | ID1 | inhibitor of DNA binding 1, dominant negative helix-loop-helix protein | 3.62E-06 | -4.34568 |
| 221268_s_at | SGPP1 | sphingosine-1-phosphate phosphatase 1 | 1.78E-06 | -4.3463 |
| 200831_s_at | SCD | stearoyl-CoA desaturase (delta-9-desaturase) | 5.06E-06 | -4.34882 |
| 214039_s_at | LAPTM4B | lysosomal protein transmembrane 4 beta | 5.01E-05 | -4.34923 |
| 202368_s_at | TRAM2 | translocation associated membrane protein 2 | 4.69E-05 | -4.35594 |
| 229465_s_at | PTPRS | Protein tyrosine phosphatase, receptor type, S | 1.98E-06 | -4.35719 |
| 230884_s_at | SPG7 | spastic paraplegia 7 (pure and complicated autosomal recessive) | 8.73E-07 | -4.36173 |
| 216044_x_at | FAM69A | family with sequence similarity 69, member A | 1.02E-05 | -4.36335 |
| 219972_s_at | C14orf135 | chromosome 14 open reading frame 135 | 0.0005476 | -4.36692 |
| 1555388_s_at | SNX25 | sorting nexin 25 | 4.29E-06 | -4.37022 |
| 209815_at | PTCH1 | patched homolog 1 (Drosophila) | 1.24E-05 | -4.371 |
| 201734_at | CLCN3 | Chloride channel 3 | 6.25E-07 | -4.37213 |
| 200617_at | MLEC | malectin | 9.94E-06 | -4.37328 |
| 1554690_a_at | TACC1 | transforming, acidic coiled-coil containing protein 1 | 1.52E-05 | -4.37554 |
| 202203_s_at | AMFR | autocrine motility factor receptor | 1.16E-05 | -4.37726 |
| 201732_s_at | CLCN3 | chloride channel 3 | 0.0001258 | -4.37764 |
| 200078_s_at | ATP6V0B | ATPase, H+ transporting, lysosomal 21kDa, V0 subunit b | 2.17E-07 | -4.37896 |
| 218556_at | ORMDL2 | ORM1-like 2 (S. cerevisiae) | 2.93E-05 | -4.37919 |
| 224918_x_at | MGST1 | microsomal glutathione S-transferase 1 | 4.97E-08 | -4.37969 |
| 223315_at | NTN4 | netrin 4 | 2.10E-06 | -4.37994 |
| 205832_at | CPA4 | carboxypeptidase A4 | 1.20E-05 | -4.38158 |
| 218927_s_at | CHST12 | carbohydrate (chondroitin 4) sulfotransferase 12 | 1.26E-07 | -4.38352 |
| 216483_s_at | C19orf10 | chromosome 19 open reading frame 10 | 3.85E-06 | -4.38464 |
| 202202_s_at | LAMA4 | laminin, alpha 4 | 4.29E-05 | -4.38495 |
| 233487_s_at | LRRC8A | leucine rich repeat containing 8 family, member A | 2.91E-05 | -4.38527 |
| 202594_at | LEPROTL1 | leptin receptor overlapping transcript-like 1 | 9.60E-05 | -4.38769 |
| 235125_x_at | FAM73A | family with sequence similarity 73, member A | 1.91E-05 | -4.38782 |
| 225647_s_at | CTSC | cathepsin C | 1.80E-08 | -4.38903 |
| 224983_at | SCARB2 | scavenger receptor class B, member 2 | 2.14E-06 | -4.39185 |
| 202307_s_at | TAP1 | transporter 1, ATP-binding cassette, sub-family B (MDR/TAP) | 1.50E-06 | -4.39266 |
| 211651_s_at | LAMB1 | laminin, beta 1 | 3.28E-05 | -4.39434 |
| 222736_s_at | TMEM38B | transmembrane protein 38B | 2.12E-05 | -4.39793 |
| 200825_s_at | HYOU1 | hypoxia up-regulated 1 | 1.58E-05 | -4.4034 |
| 209147_s_at | PPAP2A | phosphatidic acid phosphatase type 2A | 1.05E-05 | -4.40341 |
| 203127_s_at | SPTLC2 | serine palmitoyltransferase, long chain base subunit 2 | 9.68E-06 | -4.40556 |
| 210624_s_at | ILVBL | ilvB (bacterial acetolactate synthase)-like | 3.15E-05 | -4.40963 |
| 201212_at | LGMN | legumain | 1.23E-05 | -4.41018 |
| 201240_s_at | LOC653566 /// SPCS2 | signal peptidase complex subunit 2 homolog pseudogene /// signal peptidase compl | 3.75E-08 | -4.41054 |
| 224580_at | SLC38A1 | solute carrier family 38, member 1 | 3.31E-06 | -4.41284 |
| 218972_at | TTC17 | tetratricopeptide repeat domain 17 | 3.54E-06 | -4.41358 |
| 200985_s_at | CD59 | CD59 molecule, complement regulatory protein | 2.96E-07 | -4.4148 |
| 212446_s_at | LASS6 | LAG1 homolog, ceramide synthase 6 | 1.74E-05 | -4.41517 |
| 238909_at | S100A10 | S100 calcium binding protein A10 | 1.35E-06 | -4.41701 |
| 221787_at | C6orf120 | chromosome 6 open reading frame 120 | 8.78E-06 | -4.41963 |
| 239579_at | EPHX4 | epoxide hydrolase 4 | 7.57E-06 | -4.42709 |
| 220885_s_at | CENPJ | centromere protein J | 5.94E-05 | -4.42779 |
| 205893_at | NLGN1 | neuroligin 1 | 1.31E-05 | -4.42917 |
| 1558511_s_at | ESYT2 | extended synaptotagmin-like protein 2 | 0.0002302 | -4.43708 |
| 224950_at | PTGFRN | prostaglandin F2 receptor negative regulator | 5.18E-06 | -4.44074 |
| 213532_at | ADAM17 | ADAM metallopeptidase domain 17 | 3.69E-07 | -4.44099 |
| 203441_s_at | CDH2 | cadherin 2, type 1, N-cadherin (neuronal) | 1.12E-05 | -4.44986 |
| 200901_s_at | M6PR | mannose-6-phosphate receptor (cation dependent) | 1.09E-05 | -4.45232 |
| 1554242_a_at | COCH | coagulation factor C homolog, cochlin (Limulus polyphemus) | 2.37E-06 | -4.45325 |
| 202727_s_at | IFNGR1 | interferon gamma receptor 1 | 6.39E-06 | -4.45613 |
| 228124_at | ABHD12 | abhydrolase domain containing 12 | 9.74E-07 | -4.46766 |
| 212009_s_at | STIP1 | stress-induced-phosphoprotein 1 | 6.26E-06 | -4.4708 |
| 209031_at | CADM1 | cell adhesion molecule 1 | 2.12E-07 | -4.47107 |
| 218953_s_at | PCYOX1L | prenylcysteine oxidase 1 like | 9.93E-08 | -4.47187 |
| 212251_at | MTDH | metadherin | 1.84E-05 | -4.47344 |
| 218273_s_at | PDP1 | pyruvate dehyrogenase phosphatase catalytic subunit 1 | 1.75E-05 | -4.47351 |
| 225247_at | C19orf6 | chromosome 19 open reading frame 6 | 2.65E-05 | -4.47379 |
| 200972_at | TSPAN3 | tetraspanin 3 | 1.36E-06 | -4.47721 |
| 214657_s_at | NEAT1 | nuclear paraspeckle assembly transcript 1 (non-protein coding) | 8.53E-05 | -4.48012 |
| 227526_at | CDON | Cdon homolog (mouse) | 7.85E-05 | -4.48254 |
| 230836_at | ST8SIA4 | ST8 alpha-N-acetyl-neuraminide alpha-2,8-sialyltransferase 4 | 7.48E-05 | -4.48296 |
| 212862_at | CDS2 | CDP-diacylglycerol synthase (phosphatidate cytidylyltransferase) 2 | 1.30E-07 | -4.48557 |
| 211924_s_at | PLAUR | plasminogen activator, urokinase receptor | 6.58E-06 | -4.48659 |
| 218888_s_at | NETO2 | neuropilin (NRP) and tolloid (TLL)-like 2 | 6.39E-07 | -4.48724 |
| 239572_at | GJA3 | gap junction protein, alpha 3, 46kDa | 1.27E-05 | -4.49193 |
| 226077_at | RNF145 | ring finger protein 145 | 1.16E-05 | -4.49438 |
| 224756_s_at | BAT5 | HLA-B associated transcript 5 | 7.92E-08 | -4.49593 |
| 218096_at | AGPAT5 | 1-acylglycerol-3-phosphate O-acyltransferase 5 (lysophosphatidic acid acyltransf | 3.53E-06 | -4.49697 |
| 206805_at | SEMA3A | sema domain, immunoglobulin domain (Ig), short basic domain, secreted, (semaphor | 6.23E-07 | -4.4973 |
| 210625_s_at | AKAP1 | A kinase (PRKA) anchor protein 1 | 3.32E-05 | -4.4989 |
| 218898_at | FAM57A | family with sequence similarity 57, member A | 6.39E-07 | -4.49991 |
| 1552648_a_at | TNFRSF10A | tumor necrosis factor receptor superfamily, member 10a | 3.10E-05 | -4.50136 |
| 229410_at | SLC35E1 | solute carrier family 35, member E1 | 2.48E-06 | -4.50218 |
| 225182_at | TMEM50B | transmembrane protein 50B | 6.88E-06 | -4.50831 |
| 219390_at | FKBP14 | FK506 binding protein 14, 22 kDa | 8.65E-05 | -4.50868 |
| 200761_s_at | ARL6IP5 | ADP-ribosylation-like factor 6 interacting protein 5 | 0.0003663 | -4.51034 |
| 215933_s_at | HHEX | hematopoietically expressed homeobox | 8.80E-06 | -4.51252 |
| 209030_s_at | CADM1 | cell adhesion molecule 1 | 1.16E-08 | -4.51329 |
| 211000_s_at | IL6ST | interleukin 6 signal transducer (gp130, oncostatin M receptor) | 6.42E-05 | -4.51388 |
| 210692_s_at | SLC43A3 | solute carrier family 43, member 3 | 2.23E-05 | -4.51452 |
| 208858_s_at | ESYT1 | extended synaptotagmin-like protein 1 | 5.61E-08 | -4.51665 |
| 221253_s_at | TXNDC5 | thioredoxin domain containing 5 (endoplasmic reticulum) | 2.33E-08 | -4.51668 |
| 201675_at | AKAP1 | A kinase (PRKA) anchor protein 1 | 1.62E-06 | -4.51677 |
| 202194_at | TMED5 | transmembrane emp24 protein transport domain containing 5 | 2.70E-07 | -4.51742 |
| 218028_at | ELOVL1 | elongation of very long chain fatty acids (FEN1/Elo2, SUR4/Elo3, yeast)-like 1 | 3.80E-06 | -4.52076 |
| 222401_s_at | TMEM50A | transmembrane protein 50A | 1.63E-07 | -4.52791 |
| 201925_s_at | CD55 | CD55 molecule, decay accelerating factor for complement (Cromer blood group) | 7.87E-07 | -4.53147 |
| 217770_at | PIGT | phosphatidylinositol glycan anchor biosynthesis, class T | 0.0001231 | -4.53409 |
| 204769_s_at | TAP2 | transporter 2, ATP-binding cassette, sub-family B (MDR/TAP) | 1.92E-06 | -4.5347 |
| 221427_s_at | CCNL2 | cyclin L2 | 1.07E-05 | -4.53779 |
| 201580_s_at | TMX4 | thioredoxin-related transmembrane protein 4 | 1.68E-06 | -4.5404 |
| 202667_s_at | SLC39A7 | solute carrier family 39 (zinc transporter), member 7 | 1.87E-05 | -4.54265 |
| 218519_at | SLC35A5 | solute carrier family 35, member A5 | 4.37E-06 | -4.54317 |
| 224699_s_at | ESYT2 | extended synaptotagmin-like protein 2 | 1.45E-06 | -4.54362 |
| 203939_at | NT5E | 5'-nucleotidase, ecto (CD73) | 0.0001959 | -4.544 |
| 209921_at | SLC7A11 | solute carrier family 7, (cationic amino acid transporter, y+ system) member 11 | 1.26E-05 | -4.54484 |
| 219523_s_at | ODZ3 | odz, odd Oz/ten-m homolog 3 (Drosophila) | 2.10E-07 | -4.54725 |
| 226665_at | AHSA2 | AHA1, activator of heat shock 90kDa protein ATPase homolog 2 (yeast) | 9.13E-05 | -4.55395 |
| 204167_at | BTD | biotinidase | 0.000308 | -4.55504 |
| 228879_at | SNORD104 | small nucleolar RNA, C/D box 104 | 8.33E-06 | -4.5609 |
| 212629_s_at | PKN2 | protein kinase N2 | 9.59E-06 | -4.5637 |
| 202497_x_at | SLC2A3 | solute carrier family 2 (facilitated glucose transporter), member 3 | 3.02E-05 | -4.56511 |
| 219118_at | FKBP11 | FK506 binding protein 11, 19 kDa | 1.64E-07 | -4.56678 |
| 222987_s_at | TMEM9 | transmembrane protein 9 | 1.42E-05 | -4.56749 |
| 213653_at | METTL3 | methyltransferase like 3 | 7.84E-05 | -4.56863 |
| 203283_s_at | HS2ST1 | heparan sulfate 2-O-sulfotransferase 1 | 6.22E-05 | -4.57271 |
| 209404_s_at | TMED7 | transmembrane emp24 protein transport domain containing 7 | 9.29E-05 | -4.57305 |
| 214830_at | SLC38A6 | solute carrier family 38, member 6 | 4.28E-05 | -4.57334 |
| 204401_at | KCNN4 | potassium intermediate/small conductance calcium-activated channel, subfamily N, | 1.12E-07 | -4.57521 |
| 242705_x_at | LOC100289219 | similar to hCG1981348 | 1.07E-05 | -4.5792 |
| 212246_at | MCFD2 | multiple coagulation factor deficiency 2 | 1.46E-05 | -4.5806 |
| 211852_s_at | ATRN | attractin | 1.01E-05 | -4.58217 |
| 204948_s_at | FST | follistatin | 1.32E-05 | -4.58654 |
| 203128_at | SPTLC2 | serine palmitoyltransferase, long chain base subunit 2 | 0.0004406 | -4.59379 |
| 225246_at | STIM2 | stromal interaction molecule 2 | 9.59E-08 | -4.59593 |
| 209760_at | KIAA0922 | KIAA0922 | 1.36E-05 | -4.59634 |
| 209890_at | TSPAN5 | tetraspanin 5 | 5.86E-07 | -4.59661 |
| 223531_x_at | GPR89A /// GPR89B /// GPR89C | G protein-coupled receptor 89A /// G protein-coupled receptor 89B /// G protein- | 6.47E-07 | -4.60091 |
| 201121_s_at | PGRMC1 | progesterone receptor membrane component 1 | 4.32E-08 | -4.60107 |
| 205229_s_at | COCH | coagulation factor C homolog, cochlin (Limulus polyphemus) | 1.05E-05 | -4.60157 |
| 40472_at | LPCAT4 | lysophosphatidylcholine acyltransferase 4 | 1.94E-07 | -4.6062 |
| 204841_s_at | EEA1 | early endosome antigen 1 | 4.81E-06 | -4.60663 |
| 218257_s_at | UGGT1 | UDP-glucose glycoprotein glucosyltransferase 1 | 2.82E-05 | -4.60769 |
| 219201_s_at | TWSG1 | twisted gastrulation homolog 1 (Drosophila) | 1.48E-05 | -4.60835 |
| 207057_at | SLC16A7 | solute carrier family 16, member 7 (monocarboxylic acid transporter 2) | 5.24E-05 | -4.60844 |
| 221245_s_at | FZD5 | frizzled homolog 5 (Drosophila) | 1.49E-06 | -4.61149 |
| 225829_at | PDZD8 | PDZ domain containing 8 | 1.13E-05 | -4.6127 |
| 202359_s_at | SNX19 | sorting nexin 19 | 0.000108 | -4.61568 |
| 201175_at | TMX2 | thioredoxin-related transmembrane protein 2 | 1.55E-06 | -4.62094 |
| 214093_s_at | FUBP1 | far upstream element (FUSE) binding protein 1 | 0.0002662 | -4.62257 |
| 202499_s_at | SLC2A3 | solute carrier family 2 (facilitated glucose transporter), member 3 | 8.38E-08 | -4.63181 |
| 229572_at | ATP6V0A2 | ATPase, H+ transporting, lysosomal V0 subunit a2 | 5.62E-09 | -4.6319 |
| 218772_x_at | TMEM38B | transmembrane protein 38B | 7.77E-05 | -4.63196 |
| 220092_s_at | ANTXR1 | anthrax toxin receptor 1 | 2.56E-05 | -4.6335 |
| 204076_at | ENTPD4 | ectonucleoside triphosphate diphosphohydrolase 4 | 4.21E-07 | -4.63452 |
| 212297_at | ATP13A3 | ATPase type 13A3 | 0.0001134 | -4.63611 |
| 213469_at | PGAP1 | post-GPI attachment to proteins 1 | 0.0001797 | -4.64727 |
| 222418_s_at | TMEM43 | transmembrane protein 43 | 6.75E-08 | -4.65019 |
| 200871_s_at | PSAP | prosaposin | 1.30E-08 | -4.65048 |
| 227556_at | NME7 | non-metastatic cells 7, protein expressed in (nucleoside-diphosphate kinase) | 0.0002222 | -4.65212 |
| 219532_at | ELOVL4 | elongation of very long chain fatty acids (FEN1/Elo2, SUR4/Elo3, yeast)-like 4 | 1.04E-05 | -4.65392 |
| 219937_at | TRHDE | thyrotropin-releasing hormone degrading enzyme | 0.0005329 | -4.6569 |
| 226534_at | KITLG | KIT ligand | 9.74E-05 | -4.65807 |
| 226894_at | SLC35A3 | Solute carrier family 35 (UDP-N-acetylglucosamine (UDP-GlcNAc) transporter), mem | 2.77E-05 | -4.66031 |
| 212417_at | SCAMP1 | secretory carrier membrane protein 1 | 4.92E-05 | -4.66159 |
| 208836_at | ATP1B3 | ATPase, Na+/K+ transporting, beta 3 polypeptide | 1.07E-05 | -4.66199 |
| 206848_at | FAM36A /// HOXA7 | family with sequence similarity 36, member A /// homeobox A7 | 1.45E-05 | -4.66385 |
| 233011_at | ANXA1 | Annexin A1 | 3.04E-05 | -4.66386 |
| 216041_x_at | GRN | granulin | 1.55E-07 | -4.66547 |
| 229981_at | SNX5 | sorting nexin 5 | 9.04E-09 | -4.66615 |
| 208925_at | CLDND1 | claudin domain containing 1 | 0.0001332 | -4.67096 |
| 219675_s_at | UXS1 | UDP-glucuronate decarboxylase 1 | 6.81E-06 | -4.67196 |
| 201660_at | ACSL3 | Acyl-CoA synthetase long-chain family member 3 | 2.35E-06 | -4.67991 |
| 238542_at | ULBP2 | UL16 binding protein 2 | 1.31E-05 | -4.68173 |
| 217553_at | MGC87042 | STEAP family protein MGC87042 | 7.03E-06 | -4.68188 |
| 201894_s_at | SSR1 | signal sequence receptor, alpha | 2.96E-07 | -4.69345 |
| 202181_at | KIAA0247 | KIAA0247 | 1.60E-08 | -4.69476 |
| 204234_s_at | ZNF195 | zinc finger protein 195 | 1.39E-05 | -4.69843 |
| 242121_at | NCRNA00182 | non-protein coding RNA 182 | 6.70E-05 | -4.70058 |
| 202619_s_at | PLOD2 | procollagen-lysine, 2-oxoglutarate 5-dioxygenase 2 | 1.72E-07 | -4.70305 |
| 208029_s_at | LAPTM4B | lysosomal protein transmembrane 4 beta | 5.21E-07 | -4.70348 |
| 209208_at | MPDU1 | mannose-P-dolichol utilization defect 1 | 5.98E-07 | -4.7043 |
| 203580_s_at | SLC7A6 | solute carrier family 7 (cationic amino acid transporter, y+ system), member 6 | 1.55E-06 | -4.70633 |
| 227268_at | RNFT1 | ring finger protein, transmembrane 1 | 0.000191 | -4.71364 |
| 203578_s_at | SLC7A6 | solute carrier family 7 (cationic amino acid transporter, y+ system), member 6 | 6.17E-06 | -4.71715 |
| 223320_s_at | ABCB10 | ATP-binding cassette, sub-family B (MDR/TAP), member 10 | 1.51E-05 | -4.72155 |
| 222977_at | SURF4 | surfeit 4 | 1.17E-05 | -4.72487 |
| 208674_x_at | DDOST | dolichyl-diphosphooligosaccharide-protein glycosyltransferase | 1.64E-05 | -4.72719 |
| 223741_s_at | TTYH2 | tweety homolog 2 (Drosophila) | 0.0001176 | -4.72734 |
| 209197_at | SYT11 | synaptotagmin XI | 0.0002775 | -4.74018 |
| 211340_s_at | MCAM | melanoma cell adhesion molecule | 3.54E-05 | -4.7414 |
| 218193_s_at | GOLT1B | golgi transport 1 homolog B (S. cerevisiae) | 7.06E-06 | -4.74233 |
| 207809_s_at | ATP6AP1 | ATPase, H+ transporting, lysosomal accessory protein 1 | 8.84E-07 | -4.74438 |
| 213857_s_at | CD47 | CD47 molecule | 1.24E-05 | -4.74492 |
| 226571_s_at | PTPRS | protein tyrosine phosphatase, receptor type, S | 9.95E-06 | -4.7455 |
| 226850_at | SUMF1 | sulfatase modifying factor 1 | 1.64E-06 | -4.74909 |
| 216607_s_at | CYP51A1 | cytochrome P450, family 51, subfamily A, polypeptide 1 | 0.0002438 | -4.75013 |
| 204470_at | CXCL1 | chemokine (C-X-C motif) ligand 1 (melanoma growth stimulating activity, alpha) | 2.01E-07 | -4.75027 |
| 226021_at | RDH10 | retinol dehydrogenase 10 (all-trans) | 5.56E-05 | -4.75499 |
| 225090_at | SYVN1 | synovial apoptosis inhibitor 1, synoviolin | 5.23E-05 | -4.75796 |
| 212454_x_at | HNRPDL | heterogeneous nuclear ribonucleoprotein D-like | 4.64E-07 | -4.76142 |
| 218358_at | CRELD2 | cysteine-rich with EGF-like domains 2 | 1.24E-06 | -4.76269 |
| 235749_at | UGGT2 | UDP-glucose glycoprotein glucosyltransferase 2 | 3.76E-05 | -4.76295 |
| 211061_s_at | MGAT2 | mannosyl (alpha-1,6-)-glycoprotein beta-1,2-N-acetylglucosaminyltransferase | 7.54E-06 | -4.76512 |
| 208798_x_at | GOLGA8A | golgi autoantigen, golgin subfamily a, 8A | 1.46E-07 | -4.76853 |
| 210788_s_at | DHRS7 | dehydrogenase/reductase (SDR family) member 7 | 8.07E-07 | -4.7823 |
| 225576_at | C6orf72 | chromosome 6 open reading frame 72 | 3.99E-07 | -4.78394 |
| 213537_at | HLA-DPA1 | major histocompatibility complex, class II, DP alpha 1 | 3.02E-05 | -4.78424 |
| 202235_at | SLC16A1 | solute carrier family 16, member 1 (monocarboxylic acid transporter 1) | 4.94E-05 | -4.78577 |
| 212310_at | MIA3 | melanoma inhibitory activity family, member 3 | 1.30E-05 | -4.78737 |
| 242837_at | SFRS4 | Splicing factor, arginine/serine-rich 4 | 5.20E-07 | -4.78783 |
| 217889_s_at | CYBRD1 | cytochrome b reductase 1 | 7.80E-07 | -4.79256 |
| 203285_s_at | HS2ST1 | heparan sulfate 2-O-sulfotransferase 1 | 0.0002596 | -4.79627 |
| 219374_s_at | ALG9 | asparagine-linked glycosylation 9, alpha-1,2-mannosyltransferase homolog (S. cer | 7.73E-08 | -4.79711 |
| 229352_at | SPESP1 | sperm equatorial segment protein 1 | 4.60E-06 | -4.79856 |
| 202036_s_at | SFRP1 | secreted frizzled-related protein 1 | 2.79E-05 | -4.79902 |
| 226731_at | PELO | Pelota homolog (Drosophila) | 9.17E-06 | -4.79914 |
| 235198_at | OSTM1 | osteopetrosis associated transmembrane protein 1 | 4.37E-06 | -4.80035 |
| 225079_at | EMP2 | epithelial membrane protein 2 | 8.78E-06 | -4.80196 |
| 206701_x_at | EDNRB | endothelin receptor type B | 7.65E-06 | -4.80261 |
| 234672_s_at | TMEM48 | transmembrane protein 48 | 9.82E-07 | -4.80372 |
| 203579_s_at | SLC7A6 | solute carrier family 7 (cationic amino acid transporter, y+ system), member 6 | 0.000235 | -4.80389 |
| 225673_at | MYADM | myeloid-associated differentiation marker | 0.0005155 | -4.80479 |
| 225043_at | SLC15A4 | solute carrier family 15, member 4 | 1.95E-06 | -4.80604 |
| 219549_s_at | RTN3 | reticulon 3 | 1.20E-08 | -4.80845 |
| 231810_at | BRI3BP | BRI3 binding protein | 4.60E-05 | -4.81059 |
| 1558279_a_at | KDSR | 3-ketodihydrosphingosine reductase | 1.18E-05 | -4.81328 |
| 232099_at | PCDHB16 | protocadherin beta 16 | 1.19E-05 | -4.81418 |
| 229676_at | MTPAP | Mitochondrial poly(A) polymerase | 1.01E-06 | -4.81536 |
| 210087_s_at | MPZL1 | myelin protein zero-like 1 | 3.99E-06 | -4.82933 |
| 224472_x_at | SDF4 | stromal cell derived factor 4 | 3.16E-05 | -4.83152 |
| 218333_at | DERL2 | Der1-like domain family, member 2 | 1.03E-06 | -4.84437 |
| 201627_s_at | INSIG1 | insulin induced gene 1 | 1.21E-05 | -4.84446 |
| 1559822_s_at | LOC644215 /// MTDH | hypothetical LOC644215 /// metadherin | 1.31E-06 | -4.84462 |
| 45288_at | ABHD6 | abhydrolase domain containing 6 | 2.22E-06 | -4.84852 |
| 204736_s_at | CSPG4 | chondroitin sulfate proteoglycan 4 | 3.38E-05 | -4.85515 |
| 201791_s_at | DHCR7 | 7-dehydrocholesterol reductase | 5.06E-07 | -4.86053 |
| 220495_s_at | TXNDC15 | thioredoxin domain containing 15 | 1.87E-05 | -4.86096 |
| 202278_s_at | SPTLC1 | serine palmitoyltransferase, long chain base subunit 1 | 1.33E-06 | -4.86231 |
| 217869_at | HSD17B12 | hydroxysteroid (17-beta) dehydrogenase 12 | 8.30E-08 | -4.86287 |
| 208677_s_at | BSG | basigin (Ok blood group) | 6.14E-06 | -4.86312 |
| 204715_at | PANX1 | pannexin 1 | 2.55E-06 | -4.8645 |
| 243042_at | FAM73A | family with sequence similarity 73, member A | 1.74E-06 | -4.86886 |
| 209032_s_at | CADM1 | cell adhesion molecule 1 | 0.0004025 | -4.87047 |
| 221989_at | RPL10 | ribosomal protein L10 | 7.97E-08 | -4.8719 |
| 214321_at | NOV | nephroblastoma overexpressed gene | 0.0001023 | -4.87256 |
| 214430_at | GLA | galactosidase, alpha | 5.65E-08 | -4.87417 |
| 218347_at | TYW1 | tRNA-yW synthesizing protein 1 homolog (S. cerevisiae) | 5.35E-06 | -4.87829 |
| 212121_at | TCTN3 | tectonic family member 3 | 1.38E-06 | -4.88144 |
| 225595_at | CREBZF | CREB/ATF bZIP transcription factor | 6.87E-06 | -4.88419 |
| 219481_at | TTC13 | tetratricopeptide repeat domain 13 | 1.79E-07 | -4.88736 |
| 212395_s_at | KIAA0090 | KIAA0090 | 1.15E-06 | -4.88856 |
| 209355_s_at | PPAP2B | phosphatidic acid phosphatase type 2B | 1.05E-05 | -4.88903 |
| 219569_s_at | TMEM22 | transmembrane protein 22 | 6.70E-08 | -4.89115 |
| 201286_at | SDC1 | syndecan 1 | 7.49E-06 | -4.89146 |
| 209228_x_at | TUSC3 | tumor suppressor candidate 3 | 2.39E-07 | -4.89202 |
| 230261_at | ST8SIA4 | ST8 alpha-N-acetyl-neuraminide alpha-2,8-sialyltransferase 4 | 5.54E-06 | -4.89676 |
| 225321_s_at | PILRB | paired immunoglobin-like type 2 receptor beta | 1.96E-07 | -4.8999 |
| 227771_at | LIFR | leukemia inhibitory factor receptor alpha | 6.68E-07 | -4.90019 |
| 225674_at | BCAP29 | B-cell receptor-associated protein 29 | 8.34E-07 | -4.90382 |
| 226301_at | C6orf192 | chromosome 6 open reading frame 192 | 9.75E-05 | -4.90663 |
| 214930_at | SLITRK5 | SLIT and NTRK-like family, member 5 | 3.34E-05 | -4.91344 |
| 216976_s_at | RYK | RYK receptor-like tyrosine kinase | 2.37E-05 | -4.91685 |
| 202369_s_at | TRAM2 | translocation associated membrane protein 2 | 2.02E-05 | -4.92086 |
| 204404_at | SLC12A2 | solute carrier family 12 (sodium/potassium/chloride transporters), member 2 | 1.35E-06 | -4.9216 |
| 217882_at | TMEM111 | transmembrane protein 111 | 1.73E-07 | -4.92348 |
| 242669_at | UFM1 | Ubiquitin-fold modifier 1 | 6.35E-05 | -4.92554 |
| 203304_at | BAMBI | BMP and activin membrane-bound inhibitor homolog (Xenopus laevis) | 7.12E-07 | -4.92567 |
| 215224_at | SNORA21 | small nucleolar RNA, H/ACA box 21 | 1.05E-05 | -4.92898 |
| 203167_at | TIMP2 | TIMP metallopeptidase inhibitor 2 | 1.93E-05 | -4.9313 |
| 218801_at | UGGT2 | UDP-glucose glycoprotein glucosyltransferase 2 | 1.92E-06 | -4.93658 |
| 211814_s_at | CCNE2 | cyclin E2 | 2.13E-05 | -4.94052 |
| 214581_x_at | TNFRSF21 | tumor necrosis factor receptor superfamily, member 21 | 3.17E-09 | -4.96051 |
| 228941_at | ALG10B | asparagine-linked glycosylation 10, alpha-1,2-glucosyltransferase homolog B (yea | 3.23E-05 | -4.9633 |
| 224629_at | LMAN1 | lectin, mannose-binding, 1 | 3.11E-08 | -4.96478 |
| 220690_s_at | DHRS7B | dehydrogenase/reductase (SDR family) member 7B | 8.71E-06 | -4.96796 |
| 212442_s_at | LASS6 | LAG1 homolog, ceramide synthase 6 | 7.87E-07 | -4.96813 |
| 200935_at | CALR | calreticulin | 1.47E-06 | -4.97042 |
| 206233_at | B4GALT6 | UDP-Gal:betaGlcNAc beta 1,4- galactosyltransferase, polypeptide 6 | 7.13E-05 | -4.97096 |
| 208658_at | PDIA4 | protein disulfide isomerase family A, member 4 | 1.57E-06 | -4.97103 |
| 1554018_at | GPNMB | glycoprotein (transmembrane) nmb | 0.0001094 | -4.97783 |
| 201300_s_at | PRNP | prion protein | 5.46E-06 | -4.98443 |
| 210139_s_at | PMP22 | peripheral myelin protein 22 | 1.46E-07 | -4.98594 |
| 217771_at | GOLM1 | golgi membrane protein 1 | 2.15E-05 | -4.98666 |
| 218856_at | TNFRSF21 | tumor necrosis factor receptor superfamily, member 21 | 0.0001018 | -4.98787 |
| 204832_s_at | BMPR1A | bone morphogenetic protein receptor, type IA | 0.000101 | -4.98887 |
| 212305_s_at | MIA3 | melanoma inhibitory activity family, member 3 | 2.12E-07 | -4.992 |
| 1554918_a_at | ABCC4 | ATP-binding cassette, sub-family C (CFTR/MRP), member 4 | 1.14E-06 | -4.99646 |
| 201287_s_at | SDC1 | syndecan 1 | 4.59E-05 | -4.99793 |
| 212570_at | ENDOD1 | endonuclease domain containing 1 | 3.08E-06 | -5.00051 |
| 222155_s_at | GPR172A | G protein-coupled receptor 172A | 8.05E-07 | -5.00237 |
| 219522_at | FJX1 | four jointed box 1 (Drosophila) | 2.39E-05 | -5.00919 |
| 209351_at | KRT14 | keratin 14 | 1.12E-05 | -5.00958 |
| 217716_s_at | SEC61A1 | Sec61 alpha 1 subunit (S. cerevisiae) | 4.27E-07 | -5.00988 |
| 201858_s_at | SRGN | serglycin | 1.49E-06 | -5.01175 |
| 204163_at | EMILIN1 | elastin microfibril interfacer 1 | 4.51E-05 | -5.01277 |
| 203123_s_at | SLC11A2 | solute carrier family 11 (proton-coupled divalent metal ion transporters), membe | 6.55E-07 | -5.01533 |
| 223113_at | TMEM138 | transmembrane protein 138 | 4.23E-08 | -5.01773 |
| 223547_at | JKAMP | JNK1/MAPK8-associated membrane protein | 3.04E-05 | -5.02218 |
| 212194_s_at | TM9SF4 | transmembrane 9 superfamily protein member 4 | 1.55E-05 | -5.02604 |
| 225178_at | TTC14 | tetratricopeptide repeat domain 14 | 2.02E-05 | -5.03118 |
| 204078_at | SC65 | synaptonemal complex protein SC65 | 2.08E-05 | -5.03273 |
| 227146_at | QSOX2 | quiescin Q6 sulfhydryl oxidase 2 | 1.14E-05 | -5.0406 |
| 232481_s_at | SLITRK6 | SLIT and NTRK-like family, member 6 | 2.21E-07 | -5.04169 |
| 208433_s_at | LRP8 | low density lipoprotein receptor-related protein 8, apolipoprotein e receptor | 1.91E-05 | -5.04181 |
| 201914_s_at | SEC63 | SEC63 homolog (S. cerevisiae) | 0.0001756 | -5.04274 |
| 209093_s_at | GBA /// GBAP | glucosidase, beta; acid (includes glucosylceramidase) /// glucosidase, beta; aci | 8.03E-06 | -5.04349 |
| 221485_at | B4GALT5 | UDP-Gal:betaGlcNAc beta 1,4- galactosyltransferase, polypeptide 5 | 2.34E-05 | -5.044 |
| 225669_at | IFNAR1 | interferon (alpha, beta and omega) receptor 1 | 7.62E-07 | -5.0457 |
| 222978_at | SURF4 | surfeit 4 | 5.88E-06 | -5.04812 |
| 212352_s_at | TMED10 | transmembrane emp24-like trafficking protein 10 (yeast) | 7.03E-07 | -5.04946 |
| 209326_at | SLC35A2 | solute carrier family 35 (UDP-galactose transporter), member A2 | 7.49E-06 | -5.05187 |
| 214992_s_at | DNASE2 | deoxyribonuclease II, lysosomal | 1.76E-07 | -5.05222 |
| 227405_s_at | FZD8 | frizzled homolog 8 (Drosophila) | 6.19E-05 | -5.053 |
| 238418_at | SLC35B4 | solute carrier family 35, member B4 | 0.0001626 | -5.06128 |
| 222385_x_at | SEC61A1 | Sec61 alpha 1 subunit (S. cerevisiae) | 2.22E-05 | -5.06282 |
| 203504_s_at | ABCA1 | ATP-binding cassette, sub-family A (ABC1), member 1 | 5.72E-06 | -5.06497 |
| 218476_at | POMT1 | protein-O-mannosyltransferase 1 | 7.76E-07 | -5.0718 |
| 203935_at | ACVR1 | activin A receptor, type I | 8.35E-05 | -5.07647 |
| 203460_s_at | PSEN1 | presenilin 1 | 9.71E-07 | -5.08176 |
| 209227_at | TUSC3 | tumor suppressor candidate 3 | 7.23E-07 | -5.08436 |
| 200902_at | 15-九月 | 15 kDa selenoprotein | 0.0001427 | -5.09352 |
| 225492_at | TMEM33 | transmembrane protein 33 | 2.50E-08 | -5.1058 |
| 218615_s_at | TMEM39A | transmembrane protein 39A | 1.84E-05 | -5.10921 |
| 228485_s_at | SLC44A1 | solute carrier family 44, member 1 | 6.62E-07 | -5.11123 |
| 207549_x_at | CD46 | CD46 molecule, complement regulatory protein | 3.74E-05 | -5.11281 |
| 215470_at | GTF2H2B | general transcription factor IIH, polypeptide 2B | 8.53E-06 | -5.11691 |
| 219003_s_at | MANEA | mannosidase, endo-alpha | 5.17E-07 | -5.12365 |
| 232195_at | GPR158 | G protein-coupled receptor 158 | 1.89E-07 | -5.12586 |
| 202993_at | ILVBL | ilvB (bacterial acetolactate synthase)-like | 2.77E-07 | -5.12861 |
| 225571_at | LIFR | leukemia inhibitory factor receptor alpha | 3.25E-05 | -5.13839 |
| 241364_at | TMEM57 | transmembrane protein 57 | 1.15E-05 | -5.14351 |
| 222603_at | ERMP1 | endoplasmic reticulum metallopeptidase 1 | 1.43E-05 | -5.14555 |
| 215259_s_at | CADM4 | cell adhesion molecule 4 | 3.62E-07 | -5.15077 |
| 212622_at | TMEM41B | transmembrane protein 41B | 3.43E-06 | -5.15817 |
| 223008_s_at | C9orf5 | chromosome 9 open reading frame 5 | 6.46E-06 | -5.15966 |
| 222587_s_at | GALNT7 | UDP-N-acetyl-alpha-D-galactosamine:polypeptide N-acetylgalactosaminyltransferase | 3.64E-06 | -5.16268 |
| 210768_x_at | TMCO1 | transmembrane and coiled-coil domains 1 | 2.25E-06 | -5.16897 |
| 207871_s_at | ST7 | suppression of tumorigenicity 7 | 1.04E-06 | -5.16904 |
| 202441_at | ERLIN1 | ER lipid raft associated 1 | 1.44E-08 | -5.17564 |
| 227309_at | YOD1 | YOD1 OTU deubiquinating enzyme 1 homolog (S. cerevisiae) | 1.56E-05 | -5.18155 |
| 231738_at | PCDHB7 | protocadherin beta 7 | 1.88E-05 | -5.18617 |
| 226760_at | MBTPS2 | membrane-bound transcription factor peptidase, site 2 | 3.07E-06 | -5.18756 |
| 222982_x_at | SLC38A2 | solute carrier family 38, member 2 | 3.28E-07 | -5.18902 |
| 225661_at | IFNAR1 | interferon (alpha, beta and omega) receptor 1 | 0.0009906 | -5.20155 |
| 209529_at | PPAP2C | phosphatidic acid phosphatase type 2C | 4.21E-06 | -5.20266 |
| 221543_s_at | ERLIN2 | ER lipid raft associated 2 | 4.07E-05 | -5.20347 |
| 243857_at | MORF4L2 | Mortality factor 4 like 2 | 6.08E-06 | -5.21347 |
| 209386_at | TM4SF1 | transmembrane 4 L six family member 1 | 1.49E-05 | -5.21648 |
| 218529_at | CD320 | CD320 molecule | 1.70E-08 | -5.21877 |
| 224676_at | TMED4 | transmembrane emp24 protein transport domain containing 4 | 4.91E-07 | -5.21925 |
| 209466_x_at | PTN | pleiotrophin | 4.32E-05 | -5.22223 |
| 225583_at | UXS1 | UDP-glucuronate decarboxylase 1 | 0.0002652 | -5.2253 |
| 214632_at | NRP2 | neuropilin 2 | 7.68E-06 | -5.23295 |
| 221935_s_at | C3orf64 | chromosome 3 open reading frame 64 | 9.14E-06 | -5.24888 |
| 202888_s_at | ANPEP | alanyl (membrane) aminopeptidase | 2.07E-05 | -5.24943 |
| 223575_at | KIAA1549 | KIAA1549 | 4.77E-09 | -5.24969 |
| 218604_at | LEMD3 | LEM domain containing 3 | 4.38E-07 | -5.2529 |
| 224793_s_at | TGFBR1 | transforming growth factor, beta receptor 1 | 1.20E-05 | -5.25531 |
| 212633_at | KIAA0776 | KIAA0776 | 0.0001137 | -5.25747 |
| 225524_at | ANTXR2 | anthrax toxin receptor 2 | 1.55E-05 | -5.26215 |
| 208499_s_at | DNAJC3 | DnaJ (Hsp40) homolog, subfamily C, member 3 | 2.82E-05 | -5.27132 |
| 224679_at | MESDC2 | mesoderm development candidate 2 | 6.37E-06 | -5.27408 |
| 201926_s_at | CD55 | CD55 molecule, decay accelerating factor for complement (Cromer blood group) | 4.76E-08 | -5.27422 |
| 239598_s_at | LPCAT2 | lysophosphatidylcholine acyltransferase 2 | 3.14E-05 | -5.2762 |
| 200661_at | CTSA | cathepsin A | 1.14E-05 | -5.27709 |
| 200923_at | LGALS3BP | lectin, galactoside-binding, soluble, 3 binding protein | 1.71E-07 | -5.27772 |
| 201876_at | PON2 | paraoxonase 2 | 3.18E-06 | -5.28141 |
| 1566403_at | SNORA68 | small nucleolar RNA, H/ACA box 68 | 1.34E-05 | -5.28479 |
| 211137_s_at | ATP2C1 | ATPase, Ca++ transporting, type 2C, member 1 | 3.91E-06 | -5.28582 |
| 1555564_a_at | CFI | complement factor I | 2.33E-07 | -5.28623 |
| 223006_s_at | C9orf5 | chromosome 9 open reading frame 5 | 5.97E-07 | -5.28663 |
| 207855_s_at | CLCC1 | chloride channel CLIC-like 1 | 7.91E-07 | -5.28832 |
| 209087_x_at | MCAM | melanoma cell adhesion molecule | 1.26E-06 | -5.29121 |
| 224743_at | IMPAD1 | inositol monophosphatase domain containing 1 | 3.30E-08 | -5.29236 |
| 203102_s_at | MGAT2 | mannosyl (alpha-1,6-)-glycoprotein beta-1,2-N-acetylglucosaminyltransferase | 0.000151 | -5.2926 |
| 227880_s_at | TMEM185A | transmembrane protein 185A | 3.88E-06 | -5.2994 |
| 219295_s_at | PCOLCE2 | procollagen C-endopeptidase enhancer 2 | 6.45E-05 | -5.30203 |
| 226478_at | TM7SF3 | transmembrane 7 superfamily member 3 | 1.55E-05 | -5.30585 |
| 224937_at | PTGFRN | prostaglandin F2 receptor negative regulator | 5.56E-06 | -5.31293 |
| 200771_at | LAMC1 | laminin, gamma 1 (formerly LAMB2) | 4.21E-07 | -5.31483 |
| 203705_s_at | FZD7 | frizzled homolog 7 (Drosophila) | 3.85E-06 | -5.31548 |
| 213425_at | WNT5A | wingless-type MMTV integration site family, member 5A | 7.98E-05 | -5.31777 |
| 242550_at | EIF3B | eukaryotic translation initiation factor 3, subunit B | 4.43E-06 | -5.32262 |
| 201581_at | TMX4 | thioredoxin-related transmembrane protein 4 | 1.53E-05 | -5.3238 |
| 218113_at | TMEM2 | transmembrane protein 2 | 1.92E-05 | -5.32906 |
| 209295_at | TNFRSF10B | tumor necrosis factor receptor superfamily, member 10b | 1.55E-06 | -5.3309 |
| 225918_at | GLG1 | golgi apparatus protein 1 | 9.37E-06 | -5.33584 |
| 200675_at | CD81 | CD81 molecule | 1.15E-06 | -5.33639 |
| 1554101_a_at | TMTC4 | transmembrane and tetratricopeptide repeat containing 4 | 1.85E-06 | -5.33747 |
| 202686_s_at | AXL | AXL receptor tyrosine kinase | 3.64E-06 | -5.3415 |
| 216080_s_at | FADS3 | fatty acid desaturase 3 | 0.0001773 | -5.34788 |
| 201942_s_at | CPD | carboxypeptidase D | 2.63E-06 | -5.3484 |
| 225057_at | SLC15A4 | solute carrier family 15, member 4 | 9.51E-08 | -5.35626 |
| 200770_s_at | LAMC1 | laminin, gamma 1 (formerly LAMB2) | 1.21E-07 | -5.35814 |
| 202998_s_at | LOXL2 | lysyl oxidase-like 2 | 1.36E-05 | -5.36092 |
| 208358_s_at | UGT8 | UDP glycosyltransferase 8 | 2.82E-06 | -5.36246 |
| 223441_at | SLC17A5 | solute carrier family 17 (anion/sugar transporter), member 5 | 1.04E-05 | -5.3631 |
| 210845_s_at | PLAUR | plasminogen activator, urokinase receptor | 1.06E-05 | -5.36316 |
| 219649_at | ALG6 | asparagine-linked glycosylation 6, alpha-1,3-glucosyltransferase homolog (S. cer | 4.29E-06 | -5.36597 |
| 219330_at | VANGL1 | vang-like 1 (van gogh, Drosophila) | 3.49E-05 | -5.36727 |
| 208926_at | NEU1 | sialidase 1 (lysosomal sialidase) | 1.69E-06 | -5.3733 |
| 218109_s_at | MFSD1 | major facilitator superfamily domain containing 1 | 8.77E-08 | -5.37497 |
| 209593_s_at | TOR1B | torsin family 1, member B (torsin B) | 1.58E-05 | -5.37778 |
| 1555411_a_at | CCNL1 | cyclin L1 | 3.29E-07 | -5.37906 |
| 219263_at | RNF128 | ring finger protein 128 | 1.82E-06 | -5.38259 |
| 207543_s_at | P4HA1 | prolyl 4-hydroxylase, alpha polypeptide I | 0.0001734 | -5.38412 |
| 225424_at | GPAM | glycerol-3-phosphate acyltransferase, mitochondrial | 3.75E-05 | -5.38457 |
| 202314_at | CYP51A1 | cytochrome P450, family 51, subfamily A, polypeptide 1 | 0.0006369 | -5.40326 |
| 226671_at | LAMP2 | lysosomal-associated membrane protein 2 | 1.47E-05 | -5.4092 |
| 211574_s_at | CD46 | CD46 molecule, complement regulatory protein | 8.76E-07 | -5.41502 |
| 222870_s_at | B3GNT2 | UDP-GlcNAc:betaGal beta-1,3-N-acetylglucosaminyltransferase 2 | 5.36E-06 | -5.42818 |
| 213423_x_at | TUSC3 | tumor suppressor candidate 3 | 5.79E-06 | -5.43382 |
| 200803_s_at | TMBIM6 | transmembrane BAX inhibitor motif containing 6 | 6.80E-09 | -5.43884 |
| 225633_at | DPY19L3 | dpy-19-like 3 (C. elegans) | 2.37E-06 | -5.4393 |
| 201674_s_at | AKAP1 | A kinase (PRKA) anchor protein 1 | 1.07E-06 | -5.43941 |
| 213094_at | GPR126 | G protein-coupled receptor 126 | 8.25E-06 | -5.44951 |
| 204518_s_at | PPIC | peptidylprolyl isomerase C (cyclophilin C) | 3.57E-07 | -5.45262 |
| 225594_at | CREBZF | CREB/ATF bZIP transcription factor | 6.74E-06 | -5.45561 |
| 207761_s_at | METTL7A | methyltransferase like 7A | 3.49E-07 | -5.45696 |
| 227396_at | PTPRJ | protein tyrosine phosphatase, receptor type, J | 3.26E-06 | -5.46043 |
| 201148_s_at | TIMP3 | TIMP metallopeptidase inhibitor 3 | 3.54E-08 | -5.46547 |
| 203789_s_at | SEMA3C | sema domain, immunoglobulin domain (Ig), short basic domain, secreted, (semaphor | 6.26E-05 | -5.46762 |
| 225406_at | TWSG1 | twisted gastrulation homolog 1 (Drosophila) | 1.99E-05 | -5.47006 |
| 212074_at | C7orf20 /// UNC84A | chromosome 7 open reading frame 20 /// unc-84 homolog A (C. elegans) | 5.84E-07 | -5.47536 |
| 213872_at | C6orf62 | Chromosome 6 open reading frame 62 | 3.05E-07 | -5.48653 |
| 204488_at | DOLK | dolichol kinase | 7.53E-06 | -5.48991 |
| 201944_at | HEXB | hexosaminidase B (beta polypeptide) | 1.60E-08 | -5.49805 |
| 205904_at | MICA | MHC class I polypeptide-related sequence A | 0.0001384 | -5.5205 |
| 201701_s_at | PGRMC2 | progesterone receptor membrane component 2 | 0.0001771 | -5.5419 |
| 202605_at | GUSB | glucuronidase, beta | 3.22E-08 | -5.54399 |
| 230425_at | EPHB1 | EPH receptor B1 | 1.16E-05 | -5.54433 |
| 225847_at | NCEH1 | neutral cholesterol ester hydrolase 1 | 4.92E-07 | -5.55885 |
| 224963_at | SLC26A2 | solute carrier family 26 (sulfate transporter), member 2 | 1.93E-07 | -5.55935 |
| 212295_s_at | SLC7A1 | solute carrier family 7 (cationic amino acid transporter, y+ system), member 1 | 9.10E-07 | -5.56589 |
| 206953_s_at | LPHN2 | latrophilin 2 | 5.08E-06 | -5.56681 |
| 209387_s_at | TM4SF1 | transmembrane 4 L six family member 1 | 5.92E-07 | -5.56877 |
| 216264_s_at | LAMB2 | laminin, beta 2 (laminin S) | 3.48E-06 | -5.57395 |
| 225302_at | TMX3 | thioredoxin-related transmembrane protein 3 | 2.01E-05 | -5.57945 |
| 204517_at | PPIC | peptidylprolyl isomerase C (cyclophilin C) | 9.69E-06 | -5.58013 |
| 231725_at | PCDHB2 | protocadherin beta 2 | 7.31E-06 | -5.58197 |
| 224700_at | STT3B | STT3, subunit of the oligosaccharyltransferase complex, homolog B (S. cerevisiae | 3.82E-06 | -5.5929 |
| 226381_at | PS1TP4 | HBV preS1-transactivated protein 4 | 1.44E-05 | -5.59415 |
| 228157_at | ZNF207 | zinc finger protein 207 | 8.27E-06 | -5.59481 |
| 1558678_s_at | MALAT1 | metastasis associated lung adenocarcinoma transcript 1 (non-protein coding) | 2.40E-05 | -5.59577 |
| 211612_s_at | IL13RA1 | interleukin 13 receptor, alpha 1 | 9.71E-05 | -5.59629 |
| 200983_x_at | CD59 | CD59 molecule, complement regulatory protein | 5.68E-06 | -5.60136 |
| 201242_s_at | ATP1B1 | ATPase, Na+/K+ transporting, beta 1 polypeptide | 6.29E-06 | -5.61031 |
| 225144_at | BMPR2 | bone morphogenetic protein receptor, type II (serine/threonine kinase) | 9.15E-05 | -5.6186 |
| 236007_at | AKAP10 | A kinase (PRKA) anchor protein 10 | 2.92E-06 | -5.6191 |
| 200635_s_at | PTPRF | protein tyrosine phosphatase, receptor type, F | 2.20E-06 | -5.62692 |
| 234726_s_at | TMEM168 | transmembrane protein 168 | 0.0001229 | -5.62935 |
| 223120_at | FUCA2 | fucosidase, alpha-L- 2, plasma | 1.88E-05 | -5.6365 |
| 203706_s_at | FZD7 | frizzled homolog 7 (Drosophila) | 3.53E-08 | -5.63863 |
| 205885_s_at | ITGA4 | integrin, alpha 4 (antigen CD49D, alpha 4 subunit of VLA-4 receptor) | 1.68E-06 | -5.64539 |
| 225107_at | HNRNPA2B1 | heterogeneous nuclear ribonucleoprotein A2/B1 | 2.08E-06 | -5.64913 |
| 225903_at | PIGU | phosphatidylinositol glycan anchor biosynthesis, class U | 2.47E-06 | -5.65056 |
| 202539_s_at | HMGCR | 3-hydroxy-3-methylglutaryl-Coenzyme A reductase | 1.25E-07 | -5.65201 |
| 202035_s_at | SFRP1 | secreted frizzled-related protein 1 | 7.48E-07 | -5.65713 |
| 222753_s_at | SPCS3 | signal peptidase complex subunit 3 homolog (S. cerevisiae) | 4.54E-08 | -5.66748 |
| 202195_s_at | TMED5 | transmembrane emp24 protein transport domain containing 5 | 9.05E-06 | -5.67706 |
| 211966_at | COL4A2 | collagen, type IV, alpha 2 | 3.60E-07 | -5.67746 |
| 204627_s_at | ITGB3 | integrin, beta 3 (platelet glycoprotein IIIa, antigen CD61) | 8.07E-08 | -5.67855 |
| 217732_s_at | ITM2B | integral membrane protein 2B | 2.07E-06 | -5.68228 |
| 221899_at | N4BP2L2 | NEDD4 binding protein 2-like 2 | 5.57E-05 | -5.68515 |
| 212930_at | ATP2B1 | ATPase, Ca++ transporting, plasma membrane 1 | 7.30E-06 | -5.68696 |
| 242900_at | ALG10B | asparagine-linked glycosylation 10, alpha-1,2-glucosyltransferase homolog B (yea | 0.0002298 | -5.68771 |
| 211098_x_at | TMCO1 | transmembrane and coiled-coil domains 1 | 1.91E-06 | -5.68902 |
| 208675_s_at | DDOST | dolichyl-diphosphooligosaccharide-protein glycosyltransferase | 0.0001683 | -5.69454 |
| 224692_at | PPP1R15B | protein phosphatase 1, regulatory (inhibitor) subunit 15B | 1.23E-05 | -5.7005 |
| 201011_at | RPN1 | ribophorin I | 9.43E-07 | -5.71552 |
| 207714_s_at | SERPINH1 | serpin peptidase inhibitor, clade H (heat shock protein 47), member 1, (collagen | 2.68E-06 | -5.72752 |
| 218147_s_at | GLT8D1 | glycosyltransferase 8 domain containing 1 | 5.21E-06 | -5.73066 |
| 224413_s_at | TM2D2 | TM2 domain containing 2 | 5.68E-07 | -5.74146 |
| 209446_s_at | C7orf44 | chromosome 7 open reading frame 44 | 1.50E-07 | -5.74539 |
| 224579_at | SLC38A1 | solute carrier family 38, member 1 | 9.05E-06 | -5.74735 |
| 57163_at | ELOVL1 | elongation of very long chain fatty acids (FEN1/Elo2, SUR4/Elo3, yeast)-like 1 | 1.34E-06 | -5.74947 |
| 219525_at | SLC47A1 | solute carrier family 47, member 1 | 4.63E-07 | -5.75516 |
| 1560916_a_at | DPY19L1 | dpy-19-like 1 (C. elegans) | 1.34E-05 | -5.76552 |
| 202067_s_at | LDLR | low density lipoprotein receptor | 3.80E-07 | -5.77011 |
| 226785_at | ATP11C | ATPase, class VI, type 11C | 2.34E-05 | -5.77724 |
| 202127_at | PRPF4B | PRP4 pre-mRNA processing factor 4 homolog B (yeast) | 2.47E-05 | -5.77777 |
| 222850_s_at | DNAJB14 | DnaJ (Hsp40) homolog, subfamily B, member 14 | 2.26E-05 | -5.78226 |
| 204688_at | SGCE | sarcoglycan, epsilon | 5.64E-07 | -5.78248 |
| 231735_s_at | MALAT1 | metastasis associated lung adenocarcinoma transcript 1 (non-protein coding) | 6.74E-05 | -5.78254 |
| 208716_s_at | TMCO1 | transmembrane and coiled-coil domains 1 | 1.06E-07 | -5.78327 |
| 205282_at | LRP8 | low density lipoprotein receptor-related protein 8, apolipoprotein e receptor | 4.83E-07 | -5.78931 |
| 212634_at | KIAA0776 | KIAA0776 | 4.02E-05 | -5.79012 |
| 231736_x_at | MGST1 | microsomal glutathione S-transferase 1 | 2.55E-06 | -5.80112 |
| 210367_s_at | PTGES | prostaglandin E synthase | 1.60E-06 | -5.80153 |
| 227385_at | PPAPDC2 | phosphatidic acid phosphatase type 2 domain containing 2 | 5.36E-06 | -5.80937 |
| 202767_at | ACP2 | acid phosphatase 2, lysosomal | 4.35E-07 | -5.81685 |
| 200975_at | PPT1 | palmitoyl-protein thioesterase 1 | 6.89E-05 | -5.81793 |
| 229787_s_at | OGT | O-linked N-acetylglucosamine (GlcNAc) transferase (UDP-N-acetylglucosamine:polyp | 1.19E-05 | -5.82919 |
| 203833_s_at | TGOLN2 | trans-golgi network protein 2 | 5.53E-06 | -5.82923 |
| 200677_at | PTTG1IP | pituitary tumor-transforming 1 interacting protein | 1.81E-09 | -5.82989 |
| 200620_at | TMEM59 | transmembrane protein 59 | 7.06E-08 | -5.83125 |
| 212128_s_at | DAG1 | dystroglycan 1 (dystrophin-associated glycoprotein 1) | 2.26E-05 | -5.83168 |
| 1555419_a_at | ASAH1 | N-acylsphingosine amidohydrolase (acid ceramidase) 1 | 6.93E-05 | -5.83709 |
| 217744_s_at | PERP | PERP, TP53 apoptosis effector | 3.85E-05 | -5.83735 |
| 1554148_a_at | SLC33A1 | solute carrier family 33 (acetyl-CoA transporter), member 1 | 5.08E-05 | -5.83894 |
| 225435_at | SSR1 | signal sequence receptor, alpha | 2.30E-06 | -5.84199 |
| 231968_at | UGGT1 | UDP-glucose glycoprotein glucosyltransferase 1 | 3.49E-07 | -5.84726 |
| 223000_s_at | F11R | F11 receptor | 3.04E-06 | -5.84754 |
| 205746_s_at | ADAM17 | ADAM metallopeptidase domain 17 | 1.01E-05 | -5.84992 |
| 230097_at | GART | Phosphoribosylglycinamide formyltransferase, phosphoribosylglycinamide synthetas | 4.34E-07 | -5.86491 |
| 225095_at | SPTLC2 | Serine palmitoyltransferase, long chain base subunit 2 | 1.89E-06 | -5.86659 |
| 222681_at | KTELC1 | KTEL (Lys-Tyr-Glu-Leu) containing 1 | 3.91E-07 | -5.86788 |
| 204256_at | ELOVL6 | ELOVL family member 6, elongation of long chain fatty acids (FEN1/Elo2, SUR4/Elo | 8.92E-05 | -5.86861 |
| 202308_at | SREBF1 | sterol regulatory element binding transcription factor 1 | 1.13E-06 | -5.87567 |
| 204791_at | NR2C1 | nuclear receptor subfamily 2, group C, member 1 | 4.74E-06 | -5.87759 |
| 201239_s_at | LOC653566 /// SPCS2 | signal peptidase complex subunit 2 homolog pseudogene /// signal peptidase compl | 3.72E-07 | -5.88135 |
| 202735_at | EBP | emopamil binding protein (sterol isomerase) | 1.67E-05 | -5.8905 |
| 224455_s_at | ADPGK | ADP-dependent glucokinase | 9.87E-05 | -5.90942 |
| 203549_s_at | LPL | lipoprotein lipase | 9.98E-06 | -5.91748 |
| 213428_s_at | COL6A1 | collagen, type VI, alpha 1 | 2.36E-05 | -5.92395 |
| 200636_s_at | PTPRF | protein tyrosine phosphatase, receptor type, F | 2.52E-07 | -5.92689 |
| 201005_at | CD9 | CD9 molecule | 5.16E-06 | -5.93751 |
| 224688_at | C7orf42 | chromosome 7 open reading frame 42 | 4.27E-05 | -5.93957 |
| 210589_s_at | GBAP | glucosidase, beta; acid, pseudogene | 2.48E-07 | -5.94655 |
| 1555326_a_at | ADAM9 | ADAM metallopeptidase domain 9 (meltrin gamma) | 7.22E-06 | -5.94672 |
| 228181_at | SLC30A1 | solute carrier family 30 (zinc transporter), member 1 | 2.17E-07 | -5.95691 |
| 209585_s_at | MINPP1 | multiple inositol polyphosphate histidine phosphatase, 1 | 1.06E-05 | -5.95745 |
| 201735_s_at | CLCN3 | chloride channel 3 | 3.70E-06 | -5.95937 |
| 212409_s_at | TOR1AIP1 | torsin A interacting protein 1 | 8.11E-07 | -5.96308 |
| 224888_at | SELI | selenoprotein I | 7.32E-07 | -5.97201 |
| 1555403_a_at | CDH19 | cadherin 19, type 2 | 1.79E-05 | -5.97465 |
| 200598_s_at | HSP90B1 | heat shock protein 90kDa beta (Grp94), member 1 | 2.76E-08 | -5.97956 |
| 223629_at | PCDHB5 | protocadherin beta 5 | 5.07E-05 | -5.9857 |
| 226464_at | C3orf58 | chromosome 3 open reading frame 58 | 1.06E-06 | -5.98609 |
| 1555950_a_at | CD55 | CD55 molecule, decay accelerating factor for complement (Cromer blood group) | 9.28E-07 | -5.99511 |
| 201860_s_at | PLAT | plasminogen activator, tissue | 2.95E-05 | -5.99988 |
| 217775_s_at | RDH11 | retinol dehydrogenase 11 (all-trans/9-cis/11-cis) | 8.77E-06 | -6.00417 |
| 220926_s_at | EDEM3 | ER degradation enhancer, mannosidase alpha-like 3 | 0.0002286 | -6.01222 |
| 200837_at | BCAP31 | B-cell receptor-associated protein 31 | 2.06E-10 | -6.01382 |
| 239719_at | CD109 | CD109 molecule | 2.50E-07 | -6.01659 |
| 212248_at | MTDH | metadherin | 1.56E-07 | -6.01861 |
| 208639_x_at | PDIA6 | protein disulfide isomerase family A, member 6 | 7.47E-08 | -6.03258 |
| 210627_s_at | MOGS | mannosyl-oligosaccharide glucosidase | 1.06E-07 | -6.04116 |
| 212043_at | TGOLN2 | trans-golgi network protein 2 | 1.64E-06 | -6.04153 |
| 235976_at | SLITRK6 | SLIT and NTRK-like family, member 6 | 8.00E-05 | -6.04411 |
| 201203_s_at | RRBP1 | ribosome binding protein 1 homolog 180kDa (dog) | 1.24E-05 | -6.04413 |
| 227062_at | NEAT1 | nuclear paraspeckle assembly transcript 1 (non-protein coding) | 8.92E-05 | -6.05493 |
| 201243_s_at | ATP1B1 | ATPase, Na+/K+ transporting, beta 1 polypeptide | 1.66E-08 | -6.05812 |
| 200929_at | TMED10 | transmembrane emp24-like trafficking protein 10 (yeast) | 3.09E-09 | -6.07073 |
| 226860_at | TMEM19 | transmembrane protein 19 | 1.31E-05 | -6.08159 |
| 205084_at | BCAP29 | B-cell receptor-associated protein 29 | 9.13E-06 | -6.08477 |
| 228949_at | GPR177 | G protein-coupled receptor 177 | 0.0001788 | -6.10003 |
| 213113_s_at | SLC43A3 | solute carrier family 43, member 3 | 1.82E-07 | -6.10821 |
| 202228_s_at | NPTN | neuroplastin | 1.70E-05 | -6.1185 |
| 223249_at | CLDN12 | claudin 12 | 3.89E-06 | -6.12144 |
| 205812_s_at | TMED9 | transmembrane emp24 protein transport domain containing 9 | 1.62E-05 | -6.12655 |
| 203284_s_at | HS2ST1 | heparan sulfate 2-O-sulfotransferase 1 | 9.78E-07 | -6.14386 |
| 209109_s_at | TSPAN6 | tetraspanin 6 | 8.87E-07 | -6.14475 |
| 203857_s_at | PDIA5 | protein disulfide isomerase family A, member 5 | 6.23E-06 | -6.14902 |
| 228563_at | GJC1 | gap junction protein, gamma 1, 45kDa | 4.62E-06 | -6.15439 |
| 205542_at | STEAP1 | six transmembrane epithelial antigen of the prostate 1 | 1.11E-06 | -6.16032 |
| 206907_at | TNFSF9 | tumor necrosis factor (ligand) superfamily, member 9 | 2.75E-06 | -6.16422 |
| 223007_s_at | C9orf5 | chromosome 9 open reading frame 5 | 1.14E-09 | -6.16455 |
| 224996_at | ASPH | aspartate beta-hydroxylase | 9.07E-07 | -6.16588 |
| 201645_at | TNC | tenascin C | 4.58E-05 | -6.16686 |
| 204084_s_at | CLN5 | ceroid-lipofuscinosis, neuronal 5 | 6.80E-07 | -6.1839 |
| 201553_s_at | LAMP1 | lysosomal-associated membrane protein 1 | 1.84E-06 | -6.18454 |
| 235241_at | SLC38A9 | solute carrier family 38, member 9 | 5.93E-08 | -6.18711 |
| 223243_s_at | EDEM3 | ER degradation enhancer, mannosidase alpha-like 3 | 5.71E-06 | -6.19199 |
| 207668_x_at | PDIA6 | protein disulfide isomerase family A, member 6 | 4.67E-06 | -6.19328 |
| 223209_s_at | SELS | selenoprotein S | 3.39E-07 | -6.19517 |
| 213664_at | SLC1A1 | solute carrier family 1 (neuronal/epithelial high affinity glutamate transporter | 1.59E-05 | -6.1965 |
| 200760_s_at | ARL6IP5 | ADP-ribosylation-like factor 6 interacting protein 5 | 1.07E-05 | -6.19719 |
| 206023_at | NMU | neuromedin U | 1.29E-06 | -6.20507 |
| 211676_s_at | IFNGR1 | interferon gamma receptor 1 | 1.49E-05 | -6.20727 |
| 213698_at | ZMYM6 | zinc finger, MYM-type 6 | 4.81E-07 | -6.21566 |
| 224755_at | TM9SF3 | transmembrane 9 superfamily member 3 | 1.59E-05 | -6.21719 |
| 227518_at | SLC35E1 | solute carrier family 35, member E1 | 2.28E-06 | -6.22241 |
| 229850_at | KDSR | 3-ketodihydrosphingosine reductase | 1.62E-05 | -6.23309 |
| 1558080_s_at | DNAJC3 | DnaJ (Hsp40) homolog, subfamily C, member 3 | 2.80E-05 | -6.23461 |
| 205990_s_at | WNT5A | wingless-type MMTV integration site family, member 5A | 7.00E-05 | -6.23518 |
| 209509_s_at | DPAGT1 | dolichyl-phosphate (UDP-N-acetylglucosamine) N-acetylglucosaminephosphotransfera | 2.14E-07 | -6.23984 |
| 226279_at | PRSS23 | protease, serine, 23 | 1.56E-06 | -6.23984 |
| 212230_at | PPAP2B | phosphatidic acid phosphatase type 2B | 2.24E-06 | -6.2512 |
| 213238_at | ATP10D | ATPase, class V, type 10D | 2.80E-05 | -6.27136 |
| 226670_s_at | PABPC1L | poly(A) binding protein, cytoplasmic 1-like | 4.69E-07 | -6.27524 |
| 200616_s_at | MLEC | malectin | 7.16E-09 | -6.29121 |
| 202458_at | PRSS23 | protease, serine, 23 | 5.90E-07 | -6.29547 |
| 200924_s_at | SLC3A2 | solute carrier family 3 (activators of dibasic and neutral amino acid transport) | 8.94E-09 | -6.2958 |
| 208653_s_at | CD164 | CD164 molecule, sialomucin | 8.74E-07 | -6.32962 |
| 204840_s_at | EEA1 | early endosome antigen 1 | 4.71E-05 | -6.33628 |
| 222062_at | IL27RA | interleukin 27 receptor, alpha | 3.96E-07 | -6.33878 |
| 213069_at | HEG1 | HEG homolog 1 (zebrafish) | 4.74E-08 | -6.34624 |
| 217758_s_at | TM9SF3 | transmembrane 9 superfamily member 3 | 1.24E-05 | -6.34779 |
| 201443_s_at | ATP6AP2 | ATPase, H+ transporting, lysosomal accessory protein 2 | 1.42E-05 | -6.34979 |
| 218826_at | SLC35F2 | solute carrier family 35, member F2 | 9.39E-06 | -6.36267 |
| 205513_at | TCN1 | transcobalamin I (vitamin B12 binding protein, R binder family) | 3.82E-08 | -6.38045 |
| 224209_s_at | GDA | guanine deaminase | 1.71E-06 | -6.39363 |
| 220990_s_at | MIR21 /// TMEM49 | microRNA 21 /// transmembrane protein 49 | 1.42E-07 | -6.39657 |
| 227812_at | TNFRSF19 | tumor necrosis factor receptor superfamily, member 19 | 5.36E-07 | -6.40424 |
| 225411_at | TMEM87B | transmembrane protein 87B | 2.83E-06 | -6.41612 |
| 203196_at | ABCC4 | ATP-binding cassette, sub-family C (CFTR/MRP), member 4 | 1.43E-08 | -6.41914 |
| 1552306_at | ALG10 | asparagine-linked glycosylation 10, alpha-1,2-glucosyltransferase homolog (S. po | 2.00E-05 | -6.42398 |
| 218052_s_at | ATP13A1 | ATPase type 13A1 | 9.84E-07 | -6.43036 |
| 203188_at | B3GNT1 | UDP-GlcNAc:betaGal beta-1,3-N-acetylglucosaminyltransferase 1 | 5.86E-06 | -6.44135 |
| 219402_s_at | DERL1 | Der1-like domain family, member 1 | 3.94E-07 | -6.44155 |
| 214730_s_at | GLG1 | golgi apparatus protein 1 | 2.68E-08 | -6.4453 |
| 1552426_a_at | TM2D3 | TM2 domain containing 3 | 3.70E-06 | -6.46678 |
| 208460_at | GJC1 | gap junction protein, gamma 1, 45kDa | 2.39E-08 | -6.48047 |
| 223687_s_at | LY6K | lymphocyte antigen 6 complex, locus K | 9.81E-06 | -6.48474 |
| 236241_at | MED31 | mediator complex subunit 31 | 6.29E-05 | -6.48576 |
| 226545_at | CD109 | CD109 molecule | 2.82E-07 | -6.50158 |
| 220750_s_at | LEPRE1 | leucine proline-enriched proteoglycan (leprecan) 1 | 1.54E-05 | -6.51158 |
| 221702_s_at | TM2D3 | TM2 domain containing 3 | 1.82E-08 | -6.51405 |
| 221765_at | UGCG | UDP-glucose ceramide glucosyltransferase | 1.34E-06 | -6.52416 |
| 219375_at | CEPT1 | choline/ethanolamine phosphotransferase 1 | 6.09E-08 | -6.52772 |
| 201951_at | ALCAM | activated leukocyte cell adhesion molecule | 1.87E-05 | -6.53167 |
| 213325_at | PVRL3 | poliovirus receptor-related 3 | 4.47E-06 | -6.5375 |
| 200804_at | TMBIM6 | transmembrane BAX inhibitor motif containing 6 | 7.54E-07 | -6.53929 |
| 202668_at | EFNB2 | ephrin-B2 | 2.33E-05 | -6.54216 |
| 208767_s_at | LAPTM4B | lysosomal protein transmembrane 4 beta | 1.52E-06 | -6.54288 |
| 201186_at | LRPAP1 | low density lipoprotein receptor-related protein associated protein 1 | 1.32E-07 | -6.54373 |
| 200984_s_at | CD59 | CD59 molecule, complement regulatory protein | 1.08E-08 | -6.54417 |
| 1555543_a_at | CLCC1 | chloride channel CLIC-like 1 | 9.80E-07 | -6.5535 |
| 220342_x_at | EDEM3 | ER degradation enhancer, mannosidase alpha-like 3 | 5.19E-06 | -6.55454 |
| 1553995_a_at | NT5E | 5'-nucleotidase, ecto (CD73) | 6.83E-06 | -6.55659 |
| 222391_at | TMEM30A | transmembrane protein 30A | 1.77E-06 | -6.55703 |
| 201195_s_at | SLC7A5 | solute carrier family 7 (cationic amino acid transporter, y+ system), member 5 | 2.08E-06 | -6.562 |
| 221449_s_at | ITFG1 | integrin alpha FG-GAP repeat containing 1 | 2.16E-05 | -6.56337 |
| 227741_at | PTPLB | protein tyrosine phosphatase-like (proline instead of catalytic arginine), membe | 9.74E-05 | -6.56858 |
| 220642_x_at | GPR89A /// GPR89B /// GPR89C | G protein-coupled receptor 89A /// G protein-coupled receptor 89B /// G protein- | 8.02E-06 | -6.58217 |
| 209681_at | SLC19A2 | solute carrier family 19 (thiamine transporter), member 2 | 2.23E-05 | -6.59347 |
| 216268_s_at | JAG1 | jagged 1 (Alagille syndrome) | 1.91E-06 | -6.59775 |
| 205789_at | CD1D | CD1d molecule | 9.68E-08 | -6.60974 |
| 221423_s_at | YIPF5 | Yip1 domain family, member 5 | 2.65E-05 | -6.61249 |
| 225664_at | COL12A1 | collagen, type XII, alpha 1 | 6.57E-07 | -6.61527 |
| 225463_x_at | GPR89A | G protein-coupled receptor 89A | 1.03E-05 | -6.62467 |
| 212861_at | MFSD5 | major facilitator superfamily domain containing 5 | 1.33E-06 | -6.6302 |
| 202912_at | ADM | adrenomedullin | 2.74E-05 | -6.63372 |
| 205884_at | ITGA4 | integrin, alpha 4 (antigen CD49D, alpha 4 subunit of VLA-4 receptor) | 2.23E-06 | -6.64183 |
| 227033_at | PDIA3 | protein disulfide isomerase family A, member 3 | 1.47E-05 | -6.65382 |
| 226538_at | MAN2A1 | mannosidase, alpha, class 2A, member 1 | 3.75E-06 | -6.65452 |
| 201361_at | TMEM109 | transmembrane protein 109 | 1.46E-06 | -6.6683 |
| 210868_s_at | ELOVL6 | ELOVL family member 6, elongation of long chain fatty acids (FEN1/Elo2, SUR4/Elo | 2.56E-06 | -6.66842 |
| 212864_at | CDS2 | CDP-diacylglycerol synthase (phosphatidate cytidylyltransferase) 2 | 4.87E-07 | -6.66982 |
| 201149_s_at | TIMP3 | TIMP metallopeptidase inhibitor 3 | 1.20E-07 | -6.67717 |
| 230270_at | PRPF38B | PRP38 pre-mRNA processing factor 38 (yeast) domain containing B | 1.86E-05 | -6.68169 |
| 217898_at | C15orf24 | chromosome 15 open reading frame 24 | 8.00E-06 | -6.6842 |
| 212640_at | PTPLB | protein tyrosine phosphatase-like (proline instead of catalytic arginine), membe | 1.17E-09 | -6.68692 |
| 1555920_at | CBX3 | Chromobox homolog 3 (HP1 gamma homolog, Drosophila) | 1.59E-06 | -6.68785 |
| 223304_at | SLC37A3 | solute carrier family 37 (glycerol-3-phosphate transporter), member 3 | 3.84E-06 | -6.6928 |
| 213865_at | DCBLD2 | discoidin, CUB and LCCL domain containing 2 | 1.60E-07 | -6.69324 |
| 201646_at | SCARB2 | scavenger receptor class B, member 2 | 1.43E-06 | -6.70667 |
| 225835_at | SLC12A2 | solute carrier family 12 (sodium/potassium/chloride transporters), member 2 | 4.17E-05 | -6.71058 |
| 213399_x_at | RPN2 | ribophorin II | 2.00E-08 | -6.71429 |
| 217776_at | RDH11 | retinol dehydrogenase 11 (all-trans/9-cis/11-cis) | 3.70E-06 | -6.71447 |
| 224588_at | XIST | X (inactive)-specific transcript (non-protein coding) | 6.17E-06 | -6.71553 |
| 223054_at | DNAJB11 | DnaJ (Hsp40) homolog, subfamily B, member 11 | 1.14E-05 | -6.73161 |
| 211964_at | COL4A2 | collagen, type IV, alpha 2 | 7.22E-07 | -6.73447 |
| 209321_s_at | ADCY3 | adenylate cyclase 3 | 6.37E-06 | -6.74598 |
| 202088_at | SLC39A6 | solute carrier family 39 (zinc transporter), member 6 | 1.08E-07 | -6.76491 |
| 227639_at | PIGK | phosphatidylinositol glycan anchor biosynthesis, class K | 2.29E-07 | -6.77476 |
| 218459_at | TOR3A | torsin family 3, member A | 6.28E-06 | -6.77572 |
| 222507_s_at | TMEM9B | TMEM9 domain family, member B | 4.85E-08 | -6.78872 |
| 221782_at | DNAJC10 | DnaJ (Hsp40) homolog, subfamily C, member 10 | 0.0001492 | -6.80149 |
| 215034_s_at | TM4SF1 | transmembrane 4 L six family member 1 | 2.36E-06 | -6.80246 |
| 202007_at | NID1 | nidogen 1 | 3.15E-05 | -6.80917 |
| 1555996_s_at | EIF4A2 | eukaryotic translation initiation factor 4A, isoform 2 | 4.33E-06 | -6.83053 |
| 209146_at | SC4MOL | sterol-C4-methyl oxidase-like | 3.95E-05 | -6.83444 |
| 200832_s_at | SCD | stearoyl-CoA desaturase (delta-9-desaturase) | 8.11E-07 | -6.84648 |
| 201744_s_at | LUM | lumican | 6.20E-06 | -6.85382 |
| 226601_at | SLC30A7 | solute carrier family 30 (zinc transporter), member 7 | 1.15E-06 | -6.85477 |
| 200637_s_at | PTPRF | protein tyrosine phosphatase, receptor type, F | 5.19E-06 | -6.85709 |
| 226847_at | FST | follistatin | 3.31E-06 | -6.86387 |
| 218041_x_at | SLC38A2 | solute carrier family 38, member 2 | 3.32E-06 | -6.86731 |
| 215716_s_at | ATP2B1 | ATPase, Ca++ transporting, plasma membrane 1 | 4.13E-06 | -6.87247 |
| 201061_s_at | STOM | stomatin | 1.35E-07 | -6.87727 |
| 204702_s_at | NFE2L3 | nuclear factor (erythroid-derived 2)-like 3 | 2.10E-06 | -6.88463 |
| 201204_s_at | RRBP1 | ribosome binding protein 1 homolog 180kDa (dog) | 1.40E-06 | -6.89093 |
| 209108_at | TSPAN6 | tetraspanin 6 | 1.59E-06 | -6.89246 |
| 218073_s_at | TMEM48 | transmembrane protein 48 | 0.0001865 | -6.91329 |
| 210869_s_at | MCAM | melanoma cell adhesion molecule | 3.27E-07 | -6.91387 |
| 201260_s_at | SYPL1 | synaptophysin-like 1 | 1.30E-05 | -6.92625 |
| 223435_s_at | PCDHA1 /// PCDHA10 /// PCDHA11 /// PCDHA12 /// PCDHA13 /// PCDHA2 /// PCDHA3 /// PCDHA4 /// PCDHA5 /// PCDHA6 /// PCDHA7 /// PCDHA8 /// PCDHA9 /// PCDHAC1 /// PCDHAC2 | protocadherin alpha 1 /// protocadherin alpha 10 /// protocadherin alpha 11 /// | 1.01E-05 | -6.93689 |
| 210495_x_at | FN1 | fibronectin 1 | 6.13E-08 | -6.96153 |
| 204475_at | MMP1 | matrix metallopeptidase 1 (interstitial collagenase) | 1.02E-06 | -6.97962 |
| 209711_at | SLC35D1 | solute carrier family 35 (UDP-glucuronic acid/UDP-N-acetylgalactosamine dual tra | 1.02E-05 | -6.9862 |
| 212361_s_at | ATP2A2 | ATPase, Ca++ transporting, cardiac muscle, slow twitch 2 | 8.18E-09 | -6.99234 |
| 209149_s_at | TM9SF1 | transmembrane 9 superfamily member 1 | 1.04E-06 | -6.99395 |
| 208405_s_at | CD164 | CD164 molecule, sialomucin | 2.90E-06 | -7.00126 |
| 216100_s_at | TOR1AIP1 | torsin A interacting protein 1 | 8.41E-05 | -7.00531 |
| 242918_at | NASP | Nuclear autoantigenic sperm protein (histone-binding) | 1.58E-05 | -7.00539 |
| 221768_at | SFPQ | Splicing factor proline/glutamine-rich (polypyrimidine tract binding protein ass | 4.77E-05 | -7.00811 |
| 232176_at | SLITRK6 | SLIT and NTRK-like family, member 6 | 1.44E-05 | -7.01449 |
| 214252_s_at | CLN5 | ceroid-lipofuscinosis, neuronal 5 | 4.95E-06 | -7.01784 |
| 208959_s_at | ERP44 | endoplasmic reticulum protein 44 | 9.44E-06 | -7.02289 |
| 1556551_s_at | SLC39A6 | solute carrier family 39 (zinc transporter), member 6 | 1.09E-05 | -7.02432 |
| 210473_s_at | GPR125 | G protein-coupled receptor 125 | 7.30E-07 | -7.02806 |
| 211075_s_at | CD47 | CD47 molecule | 1.92E-06 | -7.03017 |
| 200821_at | LAMP2 | lysosomal-associated membrane protein 2 | 7.01E-06 | -7.03573 |
| 235907_at | TMEM33 | transmembrane protein 33 | 1.28E-06 | -7.03804 |
| 200891_s_at | SSR1 | signal sequence receptor, alpha | 1.01E-05 | -7.03923 |
| 219253_at | TMEM185B | transmembrane protein 185B (pseudogene) | 2.13E-05 | -7.05933 |
| 225222_at | HIAT1 | hippocampus abundant transcript 1 | 7.81E-05 | -7.0659 |
| 203038_at | PTPRK | protein tyrosine phosphatase, receptor type, K | 1.86E-06 | -7.07974 |
| 232323_s_at | TTC17 | tetratricopeptide repeat domain 17 | 3.58E-06 | -7.08359 |
| 220924_s_at | SLC38A2 | solute carrier family 38, member 2 | 1.31E-06 | -7.08409 |
| 211423_s_at | SC5DL | sterol-C5-desaturase (ERG3 delta-5-desaturase homolog, S. cerevisiae)-like | 4.21E-05 | -7.08628 |
| 230875_s_at | ATP11A | ATPase, class VI, type 11A | 2.02E-05 | -7.08653 |
| 209264_s_at | TSPAN4 | tetraspanin 4 | 1.42E-06 | -7.09084 |
| 200673_at | LAPTM4A | lysosomal protein transmembrane 4 alpha | 6.51E-05 | -7.09785 |
| 222018_at | NACA /// NACAP1 | nascent polypeptide-associated complex alpha subunit /// nascent-polypeptide-ass | 1.77E-05 | -7.10413 |
| 202351_at | ITGAV | integrin, alpha V (vitronectin receptor, alpha polypeptide, antigen CD51) | 1.00E-05 | -7.10538 |
| 201551_s_at | LAMP1 | lysosomal-associated membrane protein 1 | 2.09E-06 | -7.11876 |
| 206155_at | ABCC2 | ATP-binding cassette, sub-family C (CFTR/MRP), member 2 | 7.32E-07 | -7.11883 |
| 221728_x_at | XIST | X (inactive)-specific transcript (non-protein coding) | 2.40E-05 | -7.12124 |
| 226996_at | LCLAT1 | lysocardiolipin acyltransferase 1 | 5.43E-07 | -7.12178 |
| 209186_at | ATP2A2 | ATPase, Ca++ transporting, cardiac muscle, slow twitch 2 | 9.64E-08 | -7.12656 |
| 202910_s_at | CD97 | CD97 molecule | 1.16E-06 | -7.12973 |
| 204085_s_at | CLN5 | ceroid-lipofuscinosis, neuronal 5 | 1.50E-05 | -7.13844 |
| 209780_at | PHTF2 | putative homeodomain transcription factor 2 | 2.83E-07 | -7.16732 |
| 1565162_s_at | MGST1 | microsomal glutathione S-transferase 1 | 5.20E-07 | -7.17709 |
| 209198_s_at | SYT11 | synaptotagmin XI | 3.15E-08 | -7.17917 |
| 220253_s_at | LRP12 | low density lipoprotein-related protein 12 | 5.01E-05 | -7.18439 |
| 224911_s_at | DCBLD2 | discoidin, CUB and LCCL domain containing 2 | 1.65E-07 | -7.19036 |
| 228238_at | GAS5 | growth arrest-specific 5 (non-protein coding) | 1.78E-06 | -7.19829 |
| 202444_s_at | ERLIN1 | ER lipid raft associated 1 | 2.28E-06 | -7.20992 |
| 213416_at | ITGA4 | integrin, alpha 4 (antigen CD49D, alpha 4 subunit of VLA-4 receptor) | 1.03E-05 | -7.245 |
| 201485_s_at | RCN2 | reticulocalbin 2, EF-hand calcium binding domain | 8.85E-08 | -7.24977 |
| 202733_at | P4HA2 | prolyl 4-hydroxylase, alpha polypeptide II | 9.30E-09 | -7.25658 |
| 201923_at | PRDX4 | peroxiredoxin 4 | 1.98E-07 | -7.27256 |
| 212792_at | DPY19L1 | dpy-19-like 1 (C. elegans) | 6.74E-06 | -7.27619 |
| 209203_s_at | BICD2 | bicaudal D homolog 2 (Drosophila) | 9.84E-07 | -7.31619 |
| 242943_at | ST8SIA4 | ST8 alpha-N-acetyl-neuraminide alpha-2,8-sialyltransferase 4 | 2.34E-06 | -7.31895 |
| 234989_at | NEAT1 | nuclear paraspeckle assembly transcript 1 (non-protein coding) | 0.0001311 | -7.33113 |
| 232238_at | ASPM | asp (abnormal spindle) homolog, microcephaly associated (Drosophila) | 5.01E-06 | -7.33534 |
| 202087_s_at | CTSL1 | cathepsin L1 | 4.11E-08 | -7.33555 |
| 200656_s_at | P4HB | prolyl 4-hydroxylase, beta polypeptide | 1.16E-06 | -7.35723 |
| 203032_s_at | FH | fumarate hydratase | 7.57E-06 | -7.37559 |
| 218172_s_at | DERL1 | Der1-like domain family, member 1 | 2.22E-05 | -7.37638 |
| 208638_at | PDIA6 | protein disulfide isomerase family A, member 6 | 1.23E-06 | -7.37824 |
| 212517_at | ATRN | attractin | 8.49E-06 | -7.37997 |
| 209250_at | DEGS1 | degenerative spermatocyte homolog 1, lipid desaturase (Drosophila) | 3.19E-06 | -7.38437 |
| 202061_s_at | SEL1L | sel-1 suppressor of lin-12-like (C. elegans) | 4.02E-08 | -7.4002 |
| 201120_s_at | PGRMC1 | progesterone receptor membrane component 1 | 2.18E-07 | -7.40159 |
| 201289_at | CYR61 | cysteine-rich, angiogenic inducer, 61 | 8.44E-07 | -7.40161 |
| 205417_s_at | DAG1 | dystroglycan 1 (dystrophin-associated glycoprotein 1) | 4.38E-07 | -7.40509 |
| 208654_s_at | CD164 | CD164 molecule, sialomucin | 1.90E-06 | -7.40921 |
| 212507_at | TMEM131 | transmembrane protein 131 | 1.60E-06 | -7.4132 |
| 225078_at | EMP2 | epithelial membrane protein 2 | 1.40E-06 | -7.41769 |
| 227176_at | SLC2A13 | solute carrier family 2 (facilitated glucose transporter), member 13 | 2.65E-07 | -7.43922 |
| 222108_at | AMIGO2 | adhesion molecule with Ig-like domain 2 | 7.61E-06 | -7.45664 |
| 209707_at | PIGK | phosphatidylinositol glycan anchor biosynthesis, class K | 6.54E-06 | -7.45909 |
| 203429_s_at | C1orf9 | chromosome 1 open reading frame 9 | 6.32E-06 | -7.4657 |
| 201952_at | ALCAM | activated leukocyte cell adhesion molecule | 3.66E-06 | -7.4703 |
| 203989_x_at | F2R | coagulation factor II (thrombin) receptor | 7.06E-05 | -7.47213 |
| 222999_s_at | CCNL2 | cyclin L2 | 1.46E-06 | -7.48019 |
| 200890_s_at | SSR1 | signal sequence receptor, alpha | 3.08E-06 | -7.4813 |
| 210220_at | FZD2 | frizzled homolog 2 (Drosophila) | 2.68E-06 | -7.48952 |
| 226217_at | SLC30A7 | solute carrier family 30 (zinc transporter), member 7 | 3.05E-06 | -7.50772 |
| 218196_at | OSTM1 | osteopetrosis associated transmembrane protein 1 | 3.35E-06 | -7.52815 |
| 223253_at | EPDR1 | ependymin related protein 1 (zebrafish) | 4.60E-08 | -7.53545 |
| 222805_at | MANEA | mannosidase, endo-alpha | 2.93E-06 | -7.53981 |
| 225745_at | LRP6 | low density lipoprotein receptor-related protein 6 | 8.26E-07 | -7.54955 |
| 209998_at | PIGO | phosphatidylinositol glycan anchor biosynthesis, class O | 2.64E-10 | -7.55463 |
| 225284_at | DNAJC3 | DnaJ (Hsp40) homolog, subfamily C, member 3 | 1.72E-06 | -7.56481 |
| 213491_x_at | RPN2 | ribophorin II | 4.76E-07 | -7.58578 |
| 212396_s_at | KIAA0090 | KIAA0090 | 8.46E-08 | -7.59106 |
| 211737_x_at | PTN | pleiotrophin | 9.89E-07 | -7.59837 |
| 211048_s_at | PDIA4 | protein disulfide isomerase family A, member 4 | 1.15E-06 | -7.59872 |
| 201147_s_at | TIMP3 | TIMP metallopeptidase inhibitor 3 | 3.89E-09 | -7.61158 |
| 222405_at | PTPLAD1 | protein tyrosine phosphatase-like A domain containing 1 | 1.04E-05 | -7.61723 |
| 222611_s_at | PSPC1 | paraspeckle component 1 | 4.84E-06 | -7.62967 |
| 202771_at | FAM38A | family with sequence similarity 38, member A | 2.77E-06 | -7.63833 |
| 209476_at | TMX1 | thioredoxin-related transmembrane protein 1 | 1.34E-05 | -7.63888 |
| 218962_s_at | TMEM168 | transmembrane protein 168 | 1.39E-06 | -7.65666 |
| 201150_s_at | TIMP3 | TIMP metallopeptidase inhibitor 3 | 3.13E-06 | -7.65683 |
| 222646_s_at | ERO1L | ERO1-like (S. cerevisiae) | 2.44E-05 | -7.66429 |
| 212573_at | ENDOD1 | endonuclease domain containing 1 | 7.41E-07 | -7.67443 |
| 1556035_s_at | ZNF207 | zinc finger protein 207 | 3.09E-06 | -7.67529 |
| 201206_s_at | RRBP1 | ribosome binding protein 1 homolog 180kDa (dog) | 6.56E-07 | -7.71008 |
| 202381_at | ADAM9 | ADAM metallopeptidase domain 9 (meltrin gamma) | 4.67E-06 | -7.71699 |
| 228776_at | GJC1 | gap junction protein, gamma 1, 45kDa | 2.31E-07 | -7.71747 |
| 204324_s_at | GOLIM4 | golgi integral membrane protein 4 | 3.88E-06 | -7.71995 |
| 244669_at | SNHG5 /// SNORD50A /// SNORD50B | small nucleolar RNA host gene 5 (non-protein coding) /// small nucleolar RNA, C/ | 5.75E-06 | -7.73803 |
| 218696_at | EIF2AK3 | eukaryotic translation initiation factor 2-alpha kinase 3 | 3.58E-06 | -7.74115 |
| 209267_s_at | SLC39A8 | solute carrier family 39 (zinc transporter), member 8 | 6.06E-06 | -7.77442 |
| 226016_at | CD47 | CD47 molecule | 1.57E-06 | -7.7894 |
| 202540_s_at | HMGCR | 3-hydroxy-3-methylglutaryl-Coenzyme A reductase | 1.40E-08 | -7.79301 |
| 203124_s_at | SLC11A2 | solute carrier family 11 (proton-coupled divalent metal ion transporters), membe | 3.63E-06 | -7.81097 |
| 203548_s_at | LPL | lipoprotein lipase | 1.60E-07 | -7.81473 |
| 213446_s_at | IQGAP1 | IQ motif containing GTPase activating protein 1 | 1.18E-05 | -7.83264 |
| 213787_s_at | EBP | emopamil binding protein (sterol isomerase) | 1.73E-06 | -7.84221 |
| 213359_at | HNRNPD | Heterogeneous nuclear ribonucleoprotein D (AU-rich element RNA binding protein 1 | 1.63E-06 | -7.85261 |
| 203499_at | EPHA2 | EPH receptor A2 | 1.77E-07 | -7.8693 |
| 219631_at | LRP12 | low density lipoprotein-related protein 12 | 5.84E-05 | -7.87871 |
| 231866_at | LNPEP | leucyl/cystinyl aminopeptidase | 1.86E-07 | -7.87928 |
| 213552_at | GLCE | glucuronic acid epimerase | 1.03E-05 | -7.88622 |
| 208146_s_at | CPVL | carboxypeptidase, vitellogenic-like | 1.02E-06 | -7.89116 |
| 203165_s_at | SLC33A1 | solute carrier family 33 (acetyl-CoA transporter), member 1 | 1.01E-05 | -7.90596 |
| 230621_at | IAH1 | isoamyl acetate-hydrolyzing esterase 1 homolog (S. cerevisiae) | 9.76E-06 | -7.90653 |
| 209946_at | VEGFC | vascular endothelial growth factor C | 9.18E-08 | -7.90843 |
| 226825_s_at | TMEM165 | transmembrane protein 165 | 1.68E-06 | -7.91091 |
| 213459_at | RPL37A | ribosomal protein L37a | 2.54E-08 | -7.94329 |
| 217795_s_at | TMEM43 | transmembrane protein 43 | 4.83E-06 | -7.94362 |
| 213194_at | ROBO1 | roundabout, axon guidance receptor, homolog 1 (Drosophila) | 2.43E-06 | -7.95828 |
| 222543_at | DERL1 | Der1-like domain family, member 1 | 1.76E-07 | -7.96154 |
| 218095_s_at | TMEM165 | transmembrane protein 165 | 1.50E-06 | -7.9676 |
| 226026_at | DIRC2 | disrupted in renal carcinoma 2 | 3.12E-06 | -7.97512 |
| 212623_at | TMEM41B | transmembrane protein 41B | 1.01E-06 | -7.98259 |
| 221972_s_at | SDF4 | stromal cell derived factor 4 | 3.87E-08 | -7.99266 |
| 212942_s_at | KIAA1199 | KIAA1199 | 1.26E-06 | -8.00582 |
| 203545_at | ALG8 | asparagine-linked glycosylation 8, alpha-1,3-glucosyltransferase homolog (S. cer | 1.86E-06 | -8.01772 |
| 215707_s_at | PRNP | prion protein | 1.95E-05 | -8.01896 |
| 201818_at | LPCAT1 | lysophosphatidylcholine acyltransferase 1 | 4.24E-06 | -8.02728 |
| 202558_s_at | HSPA13 | heat shock protein 70kDa family, member 13 | 4.85E-06 | -8.06307 |
| 212110_at | SLC39A14 | solute carrier family 39 (zinc transporter), member 14 | 1.15E-08 | -8.07179 |
| 212250_at | MTDH | metadherin | 4.52E-07 | -8.07331 |
| 205105_at | MAN2A1 | mannosidase, alpha, class 2A, member 1 | 2.37E-07 | -8.07608 |
| 214297_at | CSPG4 | chondroitin sulfate proteoglycan 4 | 3.20E-08 | -8.09552 |
| 201795_at | LBR | lamin B receptor | 5.16E-07 | -8.09747 |
| 222569_at | UGGT1 | UDP-glucose glycoprotein glucosyltransferase 1 | 4.82E-05 | -8.10467 |
| 204638_at | ACP5 | acid phosphatase 5, tartrate resistant | 3.94E-08 | -8.12136 |
| 218516_s_at | IMPAD1 | inositol monophosphatase domain containing 1 | 8.43E-08 | -8.13171 |
| 224852_at | TTC17 | tetratricopeptide repeat domain 17 | 8.16E-06 | -8.13528 |
| 225414_at | RNF149 | ring finger protein 149 | 2.32E-07 | -8.13595 |
| 235158_at | TMEM209 | transmembrane protein 209 | 4.12E-06 | -8.14919 |
| 221958_s_at | GPR177 | G protein-coupled receptor 177 | 1.31E-06 | -8.16772 |
| 212621_at | TMEM194A | transmembrane protein 194A | 4.83E-06 | -8.20373 |
| 209281_s_at | ATP2B1 | ATPase, Ca++ transporting, plasma membrane 1 | 1.05E-05 | -8.20859 |
| 206070_s_at | EPHA3 | EPH receptor A3 | 4.95E-06 | -8.21078 |
| 216442_x_at | FN1 | fibronectin 1 | 1.87E-07 | -8.21338 |
| 222399_s_at | TM9SF3 | transmembrane 9 superfamily member 3 | 6.20E-07 | -8.23083 |
| 216064_s_at | AGA | aspartylglucosaminidase | 9.57E-08 | -8.24171 |
| 212040_at | TGOLN2 | trans-golgi network protein 2 | 9.13E-07 | -8.24617 |
| 204944_at | PTPRG | protein tyrosine phosphatase, receptor type, G | 2.92E-06 | -8.30277 |
| 201656_at | ITGA6 | integrin, alpha 6 | 3.19E-06 | -8.30634 |
| 201579_at | FAT1 | FAT tumor suppressor homolog 1 (Drosophila) | 7.43E-06 | -8.31258 |
| 201736_s_at | 6-三月 | membrane-associated ring finger (C3HC4) 6 | 4.00E-07 | -8.32029 |
| 226604_at | TMTC3 | transmembrane and tetratricopeptide repeat containing 3 | 1.12E-06 | -8.33349 |
| 208689_s_at | RPN2 | ribophorin II | 2.96E-08 | -8.34982 |
| 227671_at | XIST | X (inactive)-specific transcript (non-protein coding) | 5.79E-06 | -8.35687 |
| 213506_at | F2RL1 | coagulation factor II (thrombin) receptor-like 1 | 3.22E-06 | -8.36839 |
| 224590_at | XIST | X (inactive)-specific transcript (non-protein coding) | 4.05E-07 | -8.37223 |
| 226663_at | ANKRD10 | ankyrin repeat domain 10 | 3.56E-06 | -8.37659 |
| 214845_s_at | CALU | calumenin | 1.85E-05 | -8.38327 |
| 212163_at | KIDINS220 | kinase D-interacting substrate, 220kDa | 1.31E-06 | -8.39281 |
| 228754_at | SLC6A6 | solute carrier family 6 (neurotransmitter transporter, taurine), member 6 | 2.90E-06 | -8.40089 |
| 214658_at | TMED7 | transmembrane emp24 protein transport domain containing 7 | 1.89E-05 | -8.40568 |
| 205173_x_at | CD58 | CD58 molecule | 1.29E-07 | -8.41989 |
| 207966_s_at | GLG1 | golgi apparatus protein 1 | 1.17E-06 | -8.42442 |
| 224675_at | MESDC2 | mesoderm development candidate 2 | 1.92E-10 | -8.43405 |
| 225885_at | EEA1 | early endosome antigen 1 | 1.05E-06 | -8.43891 |
| 202277_at | SPTLC1 | serine palmitoyltransferase, long chain base subunit 1 | 6.70E-07 | -8.44362 |
| 203192_at | ABCB6 | ATP-binding cassette, sub-family B (MDR/TAP), member 6 | 8.64E-06 | -8.44533 |
| 201941_at | CPD | carboxypeptidase D | 4.58E-07 | -8.46807 |
| 202443_x_at | NOTCH2 | Notch homolog 2 (Drosophila) | 8.08E-05 | -8.47846 |
| 230031_at | HSPA5 | heat shock 70kDa protein 5 (glucose-regulated protein, 78kDa) | 3.28E-06 | -8.50198 |
| 226060_at | RFT1 | RFT1 homolog (S. cerevisiae) | 2.03E-07 | -8.53754 |
| 228956_at | UGT8 | UDP glycosyltransferase 8 | 9.71E-07 | -8.53928 |
| 232489_at | CCDC76 | coiled-coil domain containing 76 | 8.38E-07 | -8.55853 |
| 224899_s_at | MAGT1 | magnesium transporter 1 | 2.81E-06 | -8.56004 |
| 202838_at | FUCA1 | fucosidase, alpha-L- 1, tissue | 1.61E-09 | -8.61033 |
| 206157_at | PTX3 | pentraxin-related gene, rapidly induced by IL-1 beta | 2.49E-05 | -8.65683 |
| 1553530_a_at | ITGB1 | integrin, beta 1 (fibronectin receptor, beta polypeptide, antigen CD29 includes | 3.66E-06 | -8.67228 |
| 206656_s_at | C20orf3 | chromosome 20 open reading frame 3 | 1.75E-08 | -8.67241 |
| 218217_at | SCPEP1 | serine carboxypeptidase 1 | 2.22E-05 | -8.67465 |
| 224480_s_at | AGPAT9 | 1-acylglycerol-3-phosphate O-acyltransferase 9 | 2.84E-07 | -8.68013 |
| 222140_s_at | GPR89A /// GPR89B /// GPR89C | G protein-coupled receptor 89A /// G protein-coupled receptor 89B /// G protein- | 5.73E-09 | -8.70342 |
| 226353_at | SPPL2A | signal peptide peptidase-like 2A | 6.06E-06 | -8.73077 |
| 204881_s_at | UGCG | UDP-glucose ceramide glucosyltransferase | 6.77E-06 | -8.73294 |
| 221860_at | HNRNPL | heterogeneous nuclear ribonucleoprotein L | 3.37E-06 | -8.73576 |
| 210830_s_at | PON2 | paraoxonase 2 | 3.85E-06 | -8.74825 |
| 221781_s_at | DNAJC10 | DnaJ (Hsp40) homolog, subfamily C, member 10 | 2.41E-07 | -8.75789 |
| 218140_x_at | SRPRB | signal recognition particle receptor, B subunit | 2.84E-09 | -8.76404 |
| 208612_at | PDIA3 | protein disulfide isomerase family A, member 3 | 5.30E-07 | -8.78686 |
| 1555274_a_at | SELI | selenoprotein I | 1.06E-07 | -8.79214 |
| 201141_at | GPNMB | glycoprotein (transmembrane) nmb | 3.14E-08 | -8.80829 |
| 231579_s_at | TIMP2 | TIMP metallopeptidase inhibitor 2 | 6.04E-08 | -8.8227 |
| 225274_at | PCYOX1 | prenylcysteine oxidase 1 | 1.57E-05 | -8.82451 |
| 211935_at | ARL6IP1 | ADP-ribosylation factor-like 6 interacting protein 1 | 2.92E-07 | -8.82529 |
| 223005_s_at | C9orf5 | chromosome 9 open reading frame 5 | 7.53E-08 | -8.83808 |
| 201552_at | LAMP1 | lysosomal-associated membrane protein 1 | 4.63E-06 | -8.83978 |
| 206376_at | SLC6A15 | solute carrier family 6 (neutral amino acid transporter), member 15 | 1.87E-07 | -8.85517 |
| 201578_at | PODXL | podocalyxin-like | 3.11E-07 | -8.85738 |
| 201078_at | TM9SF2 | transmembrane 9 superfamily member 2 | 2.63E-07 | -8.86647 |
| 227314_at | ITGA2 | integrin, alpha 2 (CD49B, alpha 2 subunit of VLA-2 receptor) | 1.86E-05 | -8.88616 |
| 211980_at | COL4A1 | collagen, type IV, alpha 1 | 6.07E-07 | -8.90238 |
| 221553_at | MAGT1 | magnesium transporter 1 | 4.28E-05 | -8.90388 |
| 212740_at | PIK3R4 | phosphoinositide-3-kinase, regulatory subunit 4 | 7.43E-07 | -8.9365 |
| 202013_s_at | EXT2 | exostoses (multiple) 2 | 8.23E-07 | -8.94574 |
| 1555561_a_at | UGGT2 | UDP-glucose glycoprotein glucosyltransferase 2 | 1.39E-05 | -8.94662 |
| 201661_s_at | ACSL3 | acyl-CoA synthetase long-chain family member 3 | 3.53E-08 | -8.96184 |
| 206761_at | CD96 | CD96 molecule | 5.28E-05 | -8.96531 |
| 221729_at | COL5A2 | collagen, type V, alpha 2 | 1.32E-07 | -8.98493 |
| 211744_s_at | CD58 | CD58 molecule | 3.67E-06 | -9.00989 |
| 1553678_a_at | ITGB1 | integrin, beta 1 (fibronectin receptor, beta polypeptide, antigen CD29 includes | 1.02E-06 | -9.01635 |
| 202804_at | ABCC1 | ATP-binding cassette, sub-family C (CFTR/MRP), member 1 | 1.62E-08 | -9.02008 |
| 230494_at | SLC20A1 | Solute carrier family 20 (phosphate transporter), member 1 | 4.11E-07 | -9.0366 |
| 203164_at | SLC33A1 | solute carrier family 33 (acetyl-CoA transporter), member 1 | 3.63E-07 | -9.03688 |
| 201847_at | LIPA | lipase A, lysosomal acid, cholesterol esterase | 5.16E-10 | -9.04967 |
| 212944_at | SLC5A3 | solute carrier family 5 (sodium/myo-inositol cotransporter), member 3 | 8.53E-07 | -9.06904 |
| 212907_at | SLC30A1 | Solute carrier family 30 (zinc transporter), member 1 | 1.62E-07 | -9.08893 |
| 201325_s_at | EMP1 | epithelial membrane protein 1 | 2.85E-06 | -9.11763 |
| 216640_s_at | PDIA6 | protein disulfide isomerase family A, member 6 | 3.29E-09 | -9.13244 |
| 212377_s_at | NOTCH2 | Notch homolog 2 (Drosophila) | 2.65E-06 | -9.15722 |
| 200889_s_at | SSR1 | signal sequence receptor, alpha | 5.79E-08 | -9.20374 |
| 222906_at | FLVCR1 | feline leukemia virus subgroup C cellular receptor 1 | 1.66E-06 | -9.20718 |
| 201662_s_at | ACSL3 | acyl-CoA synthetase long-chain family member 3 | 3.68E-06 | -9.20728 |
| 222642_s_at | TMEM33 | transmembrane protein 33 | 9.78E-06 | -9.21771 |
| 200862_at | DHCR24 | 24-dehydrocholesterol reductase | 1.52E-07 | -9.22208 |
| 211936_at | HSPA5 | heat shock 70kDa protein 5 (glucose-regulated protein, 78kDa) | 4.80E-07 | -9.24314 |
| 203042_at | LAMP2 | lysosomal-associated membrane protein 2 | 1.97E-06 | -9.24824 |
| 201250_s_at | SLC2A1 | solute carrier family 2 (facilitated glucose transporter), member 1 | 8.75E-06 | -9.31109 |
| 209875_s_at | SPP1 | secreted phosphoprotein 1 | 1.12E-06 | -9.33404 |
| 221577_x_at | GDF15 /// LOC100292463 | growth differentiation factor 15 /// similar to growth differentiation factor 15 | 1.47E-07 | -9.34645 |
| 1552767_a_at | HS6ST2 | heparan sulfate 6-O-sulfotransferase 2 | 1.57E-07 | -9.37954 |
| 212464_s_at | FN1 | fibronectin 1 | 1.88E-09 | -9.38527 |
| 214805_at | EIF4A1 | Eukaryotic translation initiation factor 4A, isoform 1 | 1.88E-06 | -9.41431 |
| 224744_at | IMPAD1 | inositol monophosphatase domain containing 1 | 1.12E-05 | -9.41916 |
| 200973_s_at | TSPAN3 | tetraspanin 3 | 1.79E-06 | -9.43727 |
| 213164_at | SLC5A3 | solute carrier family 5 (sodium/myo-inositol cotransporter), member 3 | 1.71E-06 | -9.45234 |
| 221561_at | SOAT1 | sterol O-acyltransferase 1 | 2.55E-05 | -9.47457 |
| 217523_at | CD44 | CD44 molecule (Indian blood group) | 1.68E-05 | -9.48352 |
| 219283_at | C1GALT1C1 | C1GALT1-specific chaperone 1 | 2.58E-06 | -9.49836 |
| 204417_at | GALC | galactosylceramidase | 6.55E-07 | -9.53492 |
| 201737_s_at | 6-三月 | membrane-associated ring finger (C3HC4) 6 | 2.27E-05 | -9.53579 |
| 218989_x_at | SLC30A5 | solute carrier family 30 (zinc transporter), member 5 | 1.01E-06 | -9.55472 |
| 202679_at | NPC1 | Niemann-Pick disease, type C1 | 2.02E-07 | -9.60969 |
| 211981_at | COL4A1 | collagen, type IV, alpha 1 | 1.46E-07 | -9.61538 |
| 222432_s_at | CCDC47 | coiled-coil domain containing 47 | 4.21E-07 | -9.62107 |
| 232432_s_at | SLC30A5 | solute carrier family 30 (zinc transporter), member 5 | 2.86E-08 | -9.62807 |
| 218465_at | TMEM33 | transmembrane protein 33 | 5.76E-07 | -9.66358 |
| 1553105_s_at | DSG2 | desmoglein 2 | 7.38E-08 | -9.76754 |
| 205534_at | PCDH7 | protocadherin 7 | 4.20E-07 | -9.76904 |
| 239443_at | PCDHB6 | protocadherin beta 6 | 5.78E-07 | -9.77568 |
| 202552_s_at | CRIM1 | cysteine rich transmembrane BMP regulator 1 (chordin-like) | 6.20E-07 | -9.7792 |
| 206898_at | CDH19 | cadherin 19, type 2 | 3.84E-05 | -9.78916 |
| 224967_at | UGCG | UDP-glucose ceramide glucosyltransferase | 2.54E-07 | -9.79311 |
| 224560_at | TIMP2 | TIMP metallopeptidase inhibitor 2 | 1.52E-06 | -9.82538 |
| 225973_at | TAP2 | transporter 2, ATP-binding cassette, sub-family B (MDR/TAP) | 2.57E-06 | -9.83963 |
| 223475_at | CRISPLD1 | cysteine-rich secretory protein LCCL domain containing 1 | 6.01E-07 | -9.84153 |
| 217901_at | DSG2 | desmoglein 2 | 2.35E-07 | -9.84703 |
| 223839_s_at | SCD | stearoyl-CoA desaturase (delta-9-desaturase) | 1.75E-06 | -9.87128 |
| 222532_at | SRPRB | signal recognition particle receptor, B subunit | 9.09E-06 | -9.88542 |
| 228990_at | SNHG12 | small nucleolar RNA host gene 12 (non-protein coding) | 1.18E-06 | -9.90087 |
| 208691_at | TFRC | transferrin receptor (p90, CD71) | 1.46E-05 | -9.95832 |
| 203108_at | GPRC5A | G protein-coupled receptor, family C, group 5, member A | 6.06E-09 | -9.9631 |
| 221538_s_at | PLXNA1 | plexin A1 | 7.00E-07 | -9.98442 |
| 228950_s_at | GPR177 | G protein-coupled receptor 177 | 2.69E-08 | -9.98991 |
| 210896_s_at | ASPH | aspartate beta-hydroxylase | 9.83E-06 | -9.99029 |
| 212298_at | NRP1 | neuropilin 1 | 4.57E-07 | -10.0073 |
| 201393_s_at | IGF2R | insulin-like growth factor 2 receptor | 1.39E-06 | -10.0509 |
| 212334_at | GNS | glucosamine (N-acetyl)-6-sulfatase | 4.20E-06 | -10.0621 |
| 225303_at | KIRREL | kin of IRRE like (Drosophila) | 3.22E-07 | -10.0679 |
| 216202_s_at | SPTLC2 | serine palmitoyltransferase, long chain base subunit 2 | 5.02E-07 | -10.105 |
| 218750_at | TAF1D | TATA box binding protein (TBP)-associated factor, RNA polymerase I, D, 41kDa | 1.13E-06 | -10.1117 |
| 232591_s_at | TMEM30A | transmembrane protein 30A | 1.18E-05 | -10.1185 |
| 1564494_s_at | P4HB | prolyl 4-hydroxylase, beta polypeptide | 9.57E-08 | -10.1537 |
| 218817_at | SPCS3 | signal peptidase complex subunit 3 homolog (S. cerevisiae) | 3.84E-06 | -10.1644 |
| 201392_s_at | IGF2R | insulin-like growth factor 2 receptor | 1.62E-06 | -10.2258 |
| 225295_at | SLC39A10 | solute carrier family 39 (zinc transporter), member 10 | 1.64E-07 | -10.2375 |
| 202939_at | ZMPSTE24 | zinc metallopeptidase (STE24 homolog, S. cerevisiae) | 1.39E-08 | -10.4005 |
| 227260_at | ANKRD10 | Ankyrin repeat domain 10 | 2.54E-07 | -10.4061 |
| 226218_at | IL7R | interleukin 7 receptor | 4.43E-06 | -10.4147 |
| 202604_x_at | ADAM10 | ADAM metallopeptidase domain 10 | 5.80E-06 | -10.4221 |
| 201943_s_at | CPD | carboxypeptidase D | 4.56E-06 | -10.4795 |
| 201842_s_at | EFEMP1 | EGF-containing fibulin-like extracellular matrix protein 1 | 2.17E-07 | -10.5013 |
| 203987_at | FZD6 | frizzled homolog 6 (Drosophila) | 1.88E-06 | -10.5419 |
| 202068_s_at | LDLR | low density lipoprotein receptor | 2.35E-08 | -10.5587 |
| 203909_at | SLC9A6 | solute carrier family 9 (sodium/hydrogen exchanger), member 6 | 3.28E-07 | -10.6153 |
| 209900_s_at | SLC16A1 | solute carrier family 16, member 1 (monocarboxylic acid transporter 1) | 9.96E-06 | -10.6439 |
| 202557_at | HSPA13 | heat shock protein 70kDa family, member 13 | 2.01E-06 | -10.652 |
| 204584_at | L1CAM | L1 cell adhesion molecule | 1.29E-06 | -10.6657 |
| 211719_x_at | FN1 | fibronectin 1 | 2.44E-07 | -10.682 |
| 209101_at | CTGF | connective tissue growth factor | 1.19E-06 | -10.722 |
| 1555334_s_at | SLC30A5 | solute carrier family 30 (zinc transporter), member 5 | 3.15E-06 | -10.7586 |
| 222654_at | IMPAD1 | inositol monophosphatase domain containing 1 | 2.22E-06 | -10.7603 |
| 214218_s_at | XIST | X (inactive)-specific transcript (non-protein coding) | 7.51E-07 | -10.7952 |
| 223215_s_at | JKAMP | JNK1/MAPK8-associated membrane protein | 4.79E-07 | -10.8373 |
| 201487_at | CTSC | cathepsin C | 6.26E-09 | -10.8978 |
| 227487_s_at | SERPINE2 | Serpin peptidase inhibitor, clade E (nexin, plasminogen activator inhibitor type | 4.02E-07 | -10.9399 |
| 228173_at | GNAS | GNAS complex locus | 1.76E-05 | -10.9556 |
| 232291_at | MIR17HG | MIR17 host gene (non-protein coding) | 1.45E-06 | -10.9638 |
| 221919_at | HNRNPA1 | heterogeneous nuclear ribonucleoprotein A1 | 3.09E-05 | -10.9886 |
| 217814_at | CCDC47 | coiled-coil domain containing 47 | 5.17E-05 | -11.0566 |
| 224568_x_at | MALAT1 | metastasis associated lung adenocarcinoma transcript 1 (non-protein coding) | 0.0001415 | -11.1419 |
| 209150_s_at | TM9SF1 | transmembrane 9 superfamily member 1 | 4.53E-09 | -11.2158 |
| 212335_at | GNS | glucosamine (N-acetyl)-6-sulfatase | 9.88E-07 | -11.257 |
| 203560_at | GGH | gamma-glutamyl hydrolase (conjugase, folylpolygammaglutamyl hydrolase) | 2.13E-07 | -11.306 |
| 203041_s_at | LAMP2 | lysosomal-associated membrane protein 2 | 9.21E-08 | -11.3129 |
| 220254_at | LRP12 | low density lipoprotein-related protein 12 | 7.73E-06 | -11.3476 |
| 209218_at | SQLE | squalene epoxidase | 2.19E-07 | -11.4861 |
| 202234_s_at | SLC16A1 | solute carrier family 16, member 1 (monocarboxylic acid transporter 1) | 1.07E-06 | -11.4946 |
| 230030_at | HS6ST2 | heparan sulfate 6-O-sulfotransferase 2 | 7.55E-07 | -11.6618 |
| 226600_at | TMTC3 | transmembrane and tetratricopeptide repeat containing 3 | 8.22E-07 | -11.8895 |
| 223940_x_at | MALAT1 | metastasis associated lung adenocarcinoma transcript 1 (non-protein coding) | 3.20E-06 | -11.9601 |
| 219250_s_at | FLRT3 | fibronectin leucine rich transmembrane protein 3 | 7.93E-07 | -11.9617 |
| 201163_s_at | IGFBP7 | insulin-like growth factor binding protein 7 | 0.0001169 | -11.9965 |
| 204426_at | TMED2 | transmembrane emp24 domain trafficking protein 2 | 2.28E-05 | -12.1006 |
| 202012_s_at | EXT2 | exostoses (multiple) 2 | 7.70E-08 | -12.1215 |
| 207332_s_at | TFRC | transferrin receptor (p90, CD71) | 6.95E-09 | -12.2876 |
| 230265_at | SEL1L | Sel-1 suppressor of lin-12-like (C. elegans) | 9.24E-06 | -12.3784 |
| 219479_at | KDELC1 | KDEL (Lys-Asp-Glu-Leu) containing 1 | 2.53E-08 | -12.588 |
| 201324_at | EMP1 | epithelial membrane protein 1 | 1.67E-06 | -12.7525 |
| 208783_s_at | CD46 | CD46 molecule, complement regulatory protein | 5.74E-06 | -12.9425 |
| 209278_s_at | TFPI2 | tissue factor pathway inhibitor 2 | 1.92E-08 | -12.9541 |
| 216942_s_at | CD58 | CD58 molecule | 5.79E-08 | -13.0048 |
| 201940_at | CPD | carboxypeptidase D | 1.27E-06 | -13.1105 |
| 229899_s_at | C20orf199 | chromosome 20 open reading frame 199 | 3.59E-06 | -13.2474 |
| 217777_s_at | PTPLAD1 | protein tyrosine phosphatase-like A domain containing 1 | 7.98E-07 | -13.2558 |
| 1555120_at | CD96 | CD96 molecule | 1.81E-06 | -13.284 |
| 202236_s_at | SLC16A1 | solute carrier family 16, member 1 (monocarboxylic acid transporter 1) | 4.99E-08 | -13.3239 |
| 222404_x_at | PTPLAD1 | protein tyrosine phosphatase-like A domain containing 1 | 1.61E-08 | -13.3564 |
| 210764_s_at | CYR61 | cysteine-rich, angiogenic inducer, 61 | 5.09E-08 | -13.7628 |
| 207345_at | FST | follistatin | 5.23E-06 | -13.9206 |
| 218652_s_at | PIGG | phosphatidylinositol glycan anchor biosynthesis, class G | 2.29E-07 | -14.2021 |
| 202223_at | STT3A | STT3, subunit of the oligosaccharyltransferase complex, homolog A (S. cerevisiae | 5.41E-07 | -14.3758 |
| 1555460_a_at | SLC39A6 | solute carrier family 39 (zinc transporter), member 6 | 2.61E-06 | -14.9944 |
| 213649_at | SFRS7 | splicing factor, arginine/serine-rich 7, 35kDa | 4.44E-07 | -14.9967 |
| 204427_s_at | TMED2 | transmembrane emp24 domain trafficking protein 2 | 5.31E-06 | -15.0798 |
| 217743_s_at | TMEM30A | transmembrane protein 30A | 7.32E-06 | -15.3259 |
| 215177_s_at | ITGA6 | integrin, alpha 6 | 5.62E-06 | -15.4727 |
| 234000_s_at | PTPLAD1 | protein tyrosine phosphatase-like A domain containing 1 | 3.88E-08 | -15.709 |
| 203803_at | PCYOX1 | prenylcysteine oxidase 1 | 2.32E-06 | -15.9148 |
| 221730_at | COL5A2 | collagen, type V, alpha 2 | 3.95E-08 | -16.0241 |
| 202089_s_at | SLC39A6 | solute carrier family 39 (zinc transporter), member 6 | 6.70E-08 | -16.0417 |
| 200900_s_at | M6PR | mannose-6-phosphate receptor (cation dependent) | 2.14E-06 | -16.0601 |
| 222853_at | FLRT3 | fibronectin leucine rich transmembrane protein 3 | 1.43E-05 | -16.3719 |
| 207431_s_at | DEGS1 | degenerative spermatocyte homolog 1, lipid desaturase (Drosophila) | 5.37E-06 | -16.5093 |
| 227517_s_at | GAS5 | growth arrest-specific 5 (non-protein coding) | 5.07E-09 | -16.6185 |
| 214895_s_at | ADAM10 | ADAM metallopeptidase domain 10 | 1.86E-06 | -16.619 |
| 224917_at | MIR21 | microRNA 21 | 6.74E-06 | -16.7312 |
| 1555878_at | RPS24 | Ribosomal protein S24 | 2.25E-07 | -16.9962 |
| 1554679_a_at | LAPTM4B | lysosomal protein transmembrane 4 beta | 1.16E-07 | -17.4328 |
| 201108_s_at | THBS1 | thrombospondin 1 | 9.04E-06 | -17.546 |
| 201444_s_at | ATP6AP2 | ATPase, H+ transporting, lysosomal accessory protein 2 | 7.52E-07 | -18.1079 |
| 201110_s_at | THBS1 | thrombospondin 1 | 5.46E-06 | -18.6687 |
| 235086_at | THBS1 | Thrombospondin 1 | 4.16E-07 | -19.59 |
| 228496_s_at | CRIM1 | Cysteine rich transmembrane BMP regulator 1 (chordin-like) | 9.19E-07 | -21.4683 |
| 208097_s_at | TMX1 | thioredoxin-related transmembrane protein 1 | 5.46E-08 | -21.5641 |
| 222040_at | HNRNPA1 | heterogeneous nuclear ribonucleoprotein A1 | 2.60E-10 | -22.3148 |
| 202551_s_at | CRIM1 | cysteine rich transmembrane BMP regulator 1 (chordin-like) | 5.20E-08 | -23.2588 |
| 201109_s_at | THBS1 | thrombospondin 1 | 4.47E-09 | -27.9195 |
| 204602_at | DKK1 | dickkopf homolog 1 (Xenopus laevis) | 3.36E-09 | -49.5911 |

FC= Fold change
