# Supplementary material for: Identifying mRNA, MicroRNA and Protein Profiles of Melanoma Exosomes
Source: PLoS One. 2012 Oct 9;7(10):e46874. doi: 10.1371/journal.pone.0046874 (PMC3467276; doi:10.1371/journal.pone.0046874)
Supplement: Table S3 — Differentially expressed mRNA probe sets in A375 exosomes versus HEMa-LP exosomes (FDR corrected p<0.01 and FC >5 or FC <−5). (DOCX) [file pone.0046874.s005.docx]

**Supplementary Table S3. Differentially expressed mRNA probe sets in A375 exosomes versus HEMa-LP exosomes (FDR corrected p < 0.01 and FC > 5 or FC < -5)**

| Probeset ID | Gene Symbol | Gene Title | *p*-value | Fold Change |
| --- | --- | --- | --- | --- |
| 206172_at | IL13RA2 | interleukin 13 receptor, alpha 2 | 2.29E-08 | 252.587 |
| 214254_at | MAGEA4 | melanoma antigen family A, 4 | 2.25E-08 | 151.952 |
| 214451_at | TFAP2B | transcription factor AP-2 beta (activating enhancer binding protein 2 beta) | 2.96E-11 | 147.491 |
| 1557910_at | HSP90AB1 | heat shock protein 90kDa alpha (cytosolic), class B member 1 | 1.46E-07 | 129.662 |
| 205047_s_at | ASNS | asparagine synthetase (glutamine-hydrolyzing) | 2.22E-08 | 127.13 |
| 1567912_s_at | CT45A1 /// CT45A2 /// CT45A3 /// CT45A4 /// CT45A5 /// CT45A6 /// LOC100133581 | cancer/testis antigen family 45, member A1 /// cancer/testis antigen family 45, | 2.82E-08 | 110.883 |
| 212764_at | ZEB1 | zinc finger E-box binding homeobox 1 | 5.98E-07 | 97.902 |
| 210467_x_at | MAGEA12 | melanoma antigen family A, 12 | 3.63E-10 | 96.4183 |
| 204698_at | ISG20 | interferon stimulated exonuclease gene 20kDa | 6.48E-10 | 92.4454 |
| 209258_s_at | SMC3 | structural maintenance of chromosomes 3 | 9.39E-08 | 91.0301 |
| 235700_at | CT45A5 | cancer/testis antigen family 45, member A5 | 1.04E-07 | 85.8678 |
| 202237_at | NNMT | nicotinamide N-methyltransferase | 3.78E-11 | 84.6153 |
| 209942_x_at | MAGEA3 | melanoma antigen family A, 3 | 1.60E-08 | 82.9397 |
| 211674_x_at | CTAG1A /// CTAG1B | cancer/testis antigen 1A /// cancer/testis antigen 1B | 7.06E-09 | 82.7485 |
| 206201_s_at | MEOX2 | mesenchyme homeobox 2 | 3.15E-07 | 79.6159 |
| 203186_s_at | S100A4 | S100 calcium binding protein A4 | 4.72E-10 | 76.4952 |
| 1553394_a_at | TFAP2B | transcription factor AP-2 beta (activating enhancer binding protein 2 beta) | 7.67E-10 | 75.9662 |
| 208835_s_at | LUC7L3 | LUC7-like 3 (S. cerevisiae) | 3.14E-10 | 70.2199 |
| 208900_s_at | TOP1 | topoisomerase (DNA) I | 3.02E-07 | 69.4003 |
| 219959_at | MOCOS | molybdenum cofactor sulfurase | 2.85E-08 | 68.3857 |
| 214612_x_at | MAGEA6 | melanoma antigen family A, 6 | 3.45E-09 | 66.8191 |
| 227711_at | GTSF1 | gametocyte specific factor 1 | 3.49E-10 | 66.6747 |
| 213671_s_at | MARS | methionyl-tRNA synthetase | 1.15E-08 | 62.2072 |
| 210546_x_at | CTAG1A /// CTAG1B | cancer/testis antigen 1A /// cancer/testis antigen 1B | 8.83E-10 | 58.0741 |
| 211564_s_at | PDLIM4 | PDZ and LIM domain 4 | 1.89E-08 | 57.4946 |
| 202376_at | SERPINA3 | serpin peptidase inhibitor, clade A (alpha-1 antiproteinase, antitrypsin), membe | 5.46E-07 | 53.0624 |
| 211990_at | HLA-DPA1 | major histocompatibility complex, class II, DP alpha 1 | 5.30E-09 | 51.3508 |
| 201123_s_at | EIF5A | eukaryotic translation initiation factor 5A | 4.12E-06 | 50.0624 |
| 224657_at | ERRFI1 | ERBB receptor feedback inhibitor 1 | 3.03E-07 | 49.878 |
| 214603_at | MAGEA2 /// MAGEA2B | melanoma antigen family A, 2 /// melanoma antigen family A, 2B | 2.30E-10 | 49.8474 |
| 228318_s_at | CRIPAK | cysteine-rich PAK1 inhibitor | 4.55E-10 | 49.6958 |
| 1552619_a_at | ANLN | anillin, actin binding protein | 1.51E-08 | 49.4173 |
| 226695_at | PRRX1 | paired related homeobox 1 | 9.96E-08 | 48.785 |
| 204083_s_at | TPM2 | tropomyosin 2 (beta) | 1.02E-07 | 48.7679 |
| 216602_s_at | FARSA | phenylalanyl-tRNA synthetase, alpha subunit | 1.01E-08 | 48.5312 |
| 223413_s_at | LYAR | Ly1 antibody reactive homolog (mouse) | 3.61E-08 | 47.7111 |
| 208937_s_at | ID1 | inhibitor of DNA binding 1, dominant negative helix-loop-helix protein | 1.16E-08 | 47.3541 |
| 1553830_s_at | MAGEA2 /// MAGEA2B | melanoma antigen family A, 2 /// melanoma antigen family A, 2B | 2.15E-11 | 47.1619 |
| 224567_x_at | MALAT1 | metastasis associated lung adenocarcinoma transcript 1 (non-protein coding) | 3.17E-06 | 47.0486 |
| 1568609_s_at | FAM91A2 /// FLJ39739 /// LOC100132057 /// LOC100286793 /// LOC728855 /// LOC728875 | family with sequence similarity 91, member A2 /// hypothetical FLJ39739 /// simi | 2.52E-09 | 44.8687 |
| 222122_s_at | THOC2 | THO complex 2 | 8.62E-10 | 42.0229 |
| 208894_at | HLA-DRA | major histocompatibility complex, class II, DR alpha | 1.01E-08 | 41.4656 |
| 220445_s_at | CSAG2 /// CSAG3 | CSAG family, member 2 /// CSAG family, member 3 | 4.55E-09 | 41.1851 |
| 225857_s_at | LOC388796 | hypothetical LOC388796 | 2.72E-08 | 40.9264 |
| 33304_at | ISG20 | interferon stimulated exonuclease gene 20kDa | 3.66E-09 | 40.8358 |
| 210982_s_at | HLA-DRA | major histocompatibility complex, class II, DR alpha | 2.27E-09 | 39.9276 |
| 201291_s_at | TOP2A | topoisomerase (DNA) II alpha 170kDa | 9.16E-07 | 38.5311 |
| 201927_s_at | PKP4 | plakophilin 4 | 4.95E-08 | 37.8768 |
| 201946_s_at | CCT2 | chaperonin containing TCP1, subunit 2 (beta) | 1.60E-08 | 37.7676 |
| 215714_s_at | SMARCA4 | SWI/SNF related, matrix associated, actin dependent regulator of chromatin, subf | 1.27E-08 | 37.6995 |
| 208930_s_at | ILF3 | interleukin enhancer binding factor 3, 90kDa | 3.33E-07 | 37.4083 |
| 210983_s_at | MCM7 | minichromosome maintenance complex component 7 | 8.72E-07 | 36.3694 |
| 65588_at | LOC388796 | hypothetical LOC388796 | 1.55E-10 | 35.8525 |
| 220925_at | NAA35 | N(alpha)-acetyltransferase 35, NatC auxiliary subunit | 2.23E-08 | 35.6205 |
| 221805_at | NEFL | neurofilament, light polypeptide | 4.50E-08 | 35.5326 |
| 225285_at | BCAT1 | branched chain amino-acid transaminase 1, cytosolic | 3.70E-08 | 34.9128 |
| 212885_at | MPHOSPH10 | M-phase phosphoprotein 10 (U3 small nucleolar ribonucleoprotein) | 1.54E-07 | 34.8446 |
| 1561691_at | LOC285735 | hypothetical LOC285735 | 3.62E-11 | 34.7062 |
| 212027_at | RBM25 | RNA binding motif protein 25 | 3.07E-07 | 34.6178 |
| 206569_at | IL24 | interleukin 24 | 2.85E-10 | 33.9374 |
| 219437_s_at | ANKRD11 | ankyrin repeat domain 11 | 1.68E-09 | 33.8878 |
| 218156_s_at | TSR1 | TSR1, 20S rRNA accumulation, homolog (S. cerevisiae) | 6.56E-10 | 33.8184 |
| 217979_at | TSPAN13 | tetraspanin 13 | 1.54E-09 | 33.439 |
| 201948_at | GNL2 | guanine nucleotide binding protein-like 2 (nucleolar) | 1.33E-07 | 33.3738 |
| 224632_at | GPATCH4 | G patch domain containing 4 | 4.54E-08 | 33.3483 |
| 223403_s_at | POLR1B | polymerase (RNA) I polypeptide B, 128kDa | 8.33E-08 | 33.338 |
| 204331_s_at | MRPS12 | mitochondrial ribosomal protein S12 | 1.59E-05 | 33.2655 |
| 209049_s_at | ZMYND8 | zinc finger, MYND-type containing 8 | 1.30E-09 | 33.0408 |
| 201072_s_at | SMARCC1 | SWI/SNF related, matrix associated, actin dependent regulator of chromatin, subf | 7.84E-08 | 32.8744 |
| 225821_s_at | BOD1L | biorientation of chromosomes in cell division 1-like | 3.75E-09 | 32.5724 |
| 224736_at | CCAR1 | cell division cycle and apoptosis regulator 1 | 8.55E-07 | 32.3945 |
| 200956_s_at | SSRP1 | structure specific recognition protein 1 | 2.13E-07 | 32.3086 |
| 203109_at | UBE2M | ubiquitin-conjugating enzyme E2M (UBC12 homolog, yeast) | 9.17E-11 | 32.2646 |
| 218016_s_at | POLR3E | polymerase (RNA) III (DNA directed) polypeptide E (80kD) | 8.04E-09 | 31.8269 |
| 208025_s_at | HMGA2 | high mobility group AT-hook 2 | 8.34E-09 | 31.7939 |
| 201797_s_at | VARS | valyl-tRNA synthetase | 6.51E-10 | 31.7658 |
| 217294_s_at | ENO1 | enolase 1, (alpha) | 5.39E-07 | 31.6225 |
| 208859_s_at | ATRX | alpha thalassemia/mental retardation syndrome X-linked (RAD54 homolog, S. cerevi | 4.01E-07 | 31.5718 |
| 203843_at | RPS6KA3 | ribosomal protein S6 kinase, 90kDa, polypeptide 3 | 3.19E-09 | 31.4801 |
| 212428_at | KIAA0368 | KIAA0368 | 1.62E-09 | 31.3637 |
| 220062_s_at | MAGEC2 | melanoma antigen family C, 2 | 8.02E-08 | 31.2867 |
| 218594_at | HEATR1 | HEAT repeat containing 1 | 1.29E-05 | 31.0451 |
| 203543_s_at | KLF9 | Kruppel-like factor 9 | 2.23E-07 | 30.7399 |
| 226969_at | MTR | 5-methyltetrahydrofolate-homocysteine methyltransferase | 3.03E-08 | 30.1514 |
| 202132_at | WWTR1 | WW domain containing transcription regulator 1 | 2.47E-09 | 30.0341 |
| 203023_at | NOP16 | NOP16 nucleolar protein homolog (yeast) | 5.65E-09 | 29.913 |
| 212520_s_at | SMARCA4 | SWI/SNF related, matrix associated, actin dependent regulator of chromatin, subf | 1.51E-08 | 29.7378 |
| 218824_at | PNMAL1 | PNMA-like 1 | 7.47E-08 | 29.2995 |
| 214693_x_at | NBPF10 | neuroblastoma breakpoint family, member 10 | 1.50E-07 | 29.2173 |
| 211991_s_at | HLA-DPA1 | major histocompatibility complex, class II, DP alpha 1 | 1.17E-08 | 29.1947 |
| 209257_s_at | SMC3 | structural maintenance of chromosomes 3 | 2.68E-08 | 28.8393 |
| 202102_s_at | BRD4 | bromodomain containing 4 | 3.39E-07 | 28.7865 |
| 203180_at | ALDH1A3 | aldehyde dehydrogenase 1 family, member A3 | 8.35E-09 | 28.2486 |
| 200000_s_at | PRPF8 | PRP8 pre-mRNA processing factor 8 homolog (S. cerevisiae) | 5.61E-07 | 28.2163 |
| 201039_s_at | RAD23A | RAD23 homolog A (S. cerevisiae) | 1.20E-07 | 27.7956 |
| 227852_at | RP9 | retinitis pigmentosa 9 (autosomal dominant) | 2.46E-07 | 27.7552 |
| 221916_at | NEFL | neurofilament, light polypeptide | 1.17E-08 | 27.6344 |
| 225381_at | LOC399959 | hypothetical LOC399959 | 3.61E-09 | 27.3679 |
| 200598_s_at | HSP90B1 | heat shock protein 90kDa beta (Grp94), member 1 | 6.96E-10 | 27.3254 |
| 212766_s_at | ISG20L2 | interferon stimulated exonuclease gene 20kDa-like 2 | 4.72E-11 | 27.1073 |
| 210503_at | MAGEA11 | melanoma antigen family A, 11 | 6.69E-07 | 26.6724 |
| 219978_s_at | NUSAP1 | nucleolar and spindle associated protein 1 | 3.61E-10 | 26.6316 |
| 204086_at | PRAME | preferentially expressed antigen in melanoma | 2.36E-08 | 26.2517 |
| 217388_s_at | KYNU | kynureninase (L-kynurenine hydrolase) | 1.27E-08 | 26.2196 |
| 208777_s_at | PSMD11 | proteasome (prosome, macropain) 26S subunit, non-ATPase, 11 | 2.88E-07 | 26.178 |
| 222420_s_at | UBE2H | ubiquitin-conjugating enzyme E2H (UBC8 homolog, yeast) | 1.39E-06 | 25.9992 |
| 212018_s_at | RSL1D1 | ribosomal L1 domain containing 1 | 2.20E-08 | 25.9341 |
| 209048_s_at | ZMYND8 | zinc finger, MYND-type containing 8 | 4.08E-09 | 25.8075 |
| 201596_x_at | KRT18 | keratin 18 | 2.29E-08 | 25.7551 |
| 201475_x_at | MARS | methionyl-tRNA synthetase | 1.98E-09 | 25.4669 |
| 213097_s_at | DNAJC2 | DnaJ (Hsp40) homolog, subfamily C, member 2 | 3.57E-09 | 25.3117 |
| 209162_s_at | PRPF4 | PRP4 pre-mRNA processing factor 4 homolog (yeast) | 5.37E-07 | 25.2952 |
| 209619_at | CD74 | CD74 molecule, major histocompatibility complex, class II invariant chain | 9.75E-09 | 25.2322 |
| 223490_s_at | EXOSC3 | exosome component 3 | 3.26E-08 | 25.1857 |
| 214531_s_at | SNX1 | sorting nexin 1 | 1.84E-06 | 25.0847 |
| 209896_s_at | PTPN11 | protein tyrosine phosphatase, non-receptor type 11 | 3.09E-08 | 24.9784 |
| 227990_at | SLU7 | SLU7 splicing factor homolog (S. cerevisiae) | 3.28E-07 | 24.9674 |
| 227529_s_at | AKAP12 | A kinase (PRKA) anchor protein 12 | 4.36E-06 | 24.9495 |
| 230656_s_at | CIRH1A | cirrhosis, autosomal recessive 1A (cirhin) | 4.26E-09 | 24.8475 |
| 201505_at | LAMB1 | laminin, beta 1 | 9.09E-07 | 24.3173 |
| 212190_at | SERPINE2 | serpin peptidase inhibitor, clade E (nexin, plasminogen activator inhibitor type | 2.03E-10 | 24.3021 |
| 202238_s_at | NNMT | nicotinamide N-methyltransferase | 4.36E-07 | 24.1613 |
| 208931_s_at | ILF3 | interleukin enhancer binding factor 3, 90kDa | 2.26E-09 | 24.113 |
| 201323_at | EBNA1BP2 | EBNA1 binding protein 2 | 1.32E-09 | 24.066 |
| 202740_at | ACY1 | aminoacylase 1 | 1.73E-08 | 24.0632 |
| 201912_s_at | GSPT1 | G1 to S phase transition 1 | 3.47E-07 | 24.0417 |
| 213067_at | MYH10 | myosin, heavy chain 10, non-muscle | 1.35E-09 | 24.0405 |
| 214911_s_at | BRD2 | bromodomain containing 2 | 3.17E-08 | 23.9573 |
| 212761_at | TCF7L2 | transcription factor 7-like 2 (T-cell specific, HMG-box) | 1.98E-07 | 23.866 |
| 221514_at | UTP14A | UTP14, U3 small nucleolar ribonucleoprotein, homolog A (yeast) | 6.13E-08 | 23.8269 |
| 233080_s_at | PRPF40A | PRP40 pre-mRNA processing factor 40 homolog A (S. cerevisiae) | 3.45E-08 | 23.7176 |
| 201478_s_at | DKC1 | dyskeratosis congenita 1, dyskerin | 8.06E-09 | 23.7073 |
| 204915_s_at | SOX11 | SRY (sex determining region Y)-box 11 | 9.57E-09 | 23.5366 |
| 212219_at | PSME4 | proteasome (prosome, macropain) activator subunit 4 | 1.23E-06 | 23.5039 |
| 214437_s_at | SHMT2 | serine hydroxymethyltransferase 2 (mitochondrial) | 8.62E-07 | 23.4883 |
| 218605_at | TFB2M | transcription factor B2, mitochondrial | 1.36E-06 | 23.4618 |
| 208965_s_at | IFI16 | interferon, gamma-inducible protein 16 | 6.40E-08 | 23.434 |
| 200692_s_at | HSPA9 | heat shock 70kDa protein 9 (mortalin) | 9.14E-08 | 23.3907 |
| 211968_s_at | HSP90AA1 | heat shock protein 90kDa alpha (cytosolic), class A member 1 | 6.37E-08 | 23.2187 |
| 212501_at | CEBPB | CCAAT/enhancer binding protein (C/EBP), beta | 3.45E-08 | 23.039 |
| 220057_at | XAGE1A /// XAGE1B /// XAGE1C /// XAGE1D /// XAGE1E | X antigen family, member 1A /// X antigen family, member 1B /// X antigen family | 1.51E-08 | 22.8058 |
| 224558_s_at | MALAT1 | metastasis associated lung adenocarcinoma transcript 1 (non-protein coding) | 1.15E-08 | 22.7605 |
| 206363_at | MAF | v-maf musculoaponeurotic fibrosarcoma oncogene homolog (avian) | 6.00E-08 | 22.676 |
| 206640_x_at | GAGE12C /// GAGE12D /// GAGE12E /// GAGE12F /// GAGE12G /// GAGE12H /// GAGE12I /// GAGE2A /// GAGE2C /// GAGE4 /// GAGE5 /// GAGE6 /// GAGE7 | G antigen 12C /// G antigen 12D /// G antigen 12E /// G antigen 12F /// G antige | 4.12E-08 | 22.6541 |
| 1555225_at | C1orf43 | chromosome 1 open reading frame 43 | 1.94E-09 | 22.5694 |
| 209161_at | PRPF4 | PRP4 pre-mRNA processing factor 4 homolog (yeast) | 3.63E-08 | 22.5483 |
| 224851_at | CDK6 | cyclin-dependent kinase 6 | 7.54E-07 | 22.3712 |
| 201183_s_at | CHD4 | chromodomain helicase DNA binding protein 4 | 1.12E-06 | 22.2209 |
| 226242_at | C1orf131 | chromosome 1 open reading frame 131 | 3.21E-09 | 22.1361 |
| 204011_at | SPRY2 | sprouty homolog 2 (Drosophila) | 7.60E-07 | 22.1298 |
| 234942_s_at | DNTTIP1 | deoxynucleotidyltransferase, terminal, interacting protein 1 | 1.81E-09 | 22.1233 |
| 218795_at | ACP6 | acid phosphatase 6, lysophosphatidic | 8.62E-08 | 22.0522 |
| 218872_at | TESC | tescalcin | 6.87E-07 | 21.9395 |
| 204724_s_at | COL9A3 | collagen, type IX, alpha 3 | 2.85E-07 | 21.9299 |
| 203816_at | DGUOK | deoxyguanosine kinase | 1.39E-06 | 21.8316 |
| 212591_at | RBM34 | RNA binding motif protein 34 | 4.22E-11 | 21.7289 |
| 200842_s_at | EPRS | glutamyl-prolyl-tRNA synthetase | 1.02E-09 | 21.5888 |
| 204805_s_at | H1FX | H1 histone family, member X | 5.97E-07 | 21.5017 |
| 222464_s_at | C10orf119 | chromosome 10 open reading frame 119 | 2.69E-09 | 21.4932 |
| 1554089_s_at | SBDS /// SBDSP1 | Shwachman-Bodian-Diamond syndrome /// Shwachman-Bodian-Diamond syndrome pseudoge | 5.07E-07 | 21.4698 |
| 201730_s_at | TPR | translocated promoter region (to activated MET oncogene) | 1.25E-05 | 21.3592 |
| 202402_s_at | CARS | cysteinyl-tRNA synthetase | 2.55E-07 | 21.279 |
| 217805_at | ILF3 | interleukin enhancer binding factor 3, 90kDa | 2.46E-08 | 21.26 |
| 236565_s_at | LARP6 | La ribonucleoprotein domain family, member 6 | 3.33E-09 | 21.2566 |
| 202489_s_at | FXYD3 | FXYD domain containing ion transport regulator 3 | 4.23E-08 | 21.1989 |
| 201774_s_at | NCAPD2 | non-SMC condensin I complex, subunit D2 | 2.42E-06 | 21.1815 |
| 219067_s_at | NSMCE4A | non-SMC element 4 homolog A (S. cerevisiae) | 4.10E-09 | 21.1265 |
| 221755_at | EHBP1L1 | EH domain binding protein 1-like 1 | 5.74E-07 | 21.074 |
| 205462_s_at | HPCAL1 | hippocalcin-like 1 | 8.06E-06 | 20.9754 |
| 221970_s_at | NOL11 | nucleolar protein 11 | 3.80E-06 | 20.8426 |
| 209127_s_at | SART3 | squamous cell carcinoma antigen recognized by T cells 3 | 1.20E-06 | 20.7605 |
| 201075_s_at | SMARCC1 | SWI/SNF related, matrix associated, actin dependent regulator of chromatin, subf | 7.62E-08 | 20.7462 |
| 208694_at | PRKDC | protein kinase, DNA-activated, catalytic polypeptide | 5.67E-07 | 20.6503 |
| 202946_s_at | BTBD3 | BTB (POZ) domain containing 3 | 2.73E-08 | 20.5377 |
| 225436_at | FAM108C1 | family with sequence similarity 108, member C1 | 7.48E-08 | 20.4764 |
| 209520_s_at | NCBP1 | nuclear cap binding protein subunit 1, 80kDa | 8.56E-09 | 20.3734 |
| 222837_s_at | NAA15 | N(alpha)-acetyltransferase 15, NatA auxiliary subunit | 8.65E-07 | 20.2854 |
| 208095_s_at | SRP72 | signal recognition particle 72kDa | 1.01E-06 | 20.2636 |
| 212563_at | BOP1 /// LOC727967 | block of proliferation 1 /// similar to block of proliferation 1 | 1.45E-09 | 20.2224 |
| 204355_at | DHX30 | DEAH (Asp-Glu-Ala-His) box polypeptide 30 | 6.80E-09 | 20.2149 |
| 209953_s_at | CDC37 | cell division cycle 37 homolog (S. cerevisiae) | 9.08E-15 | 20.1898 |
| 209122_at | PLIN2 | perilipin 2 | 7.73E-08 | 20.1834 |
| 221987_s_at | TSR1 | TSR1, 20S rRNA accumulation, homolog (S. cerevisiae) | 1.26E-06 | 20.1816 |
| 218768_at | NUP107 | nucleoporin 107kDa | 1.65E-06 | 20.1234 |
| 200798_x_at | MCL1 | myeloid cell leukemia sequence 1 (BCL2-related) | 8.43E-07 | 20.0117 |
| 211944_at | BAT2L2 | HLA-B associated transcript 2-like 2 | 3.49E-08 | 19.9689 |
| 201970_s_at | NASP | nuclear autoantigenic sperm protein (histone-binding) | 5.94E-09 | 19.8993 |
| 234701_at | ANKRD11 | ankyrin repeat domain 11 | 9.77E-08 | 19.8311 |
| 226335_at | RPS6KA3 | ribosomal protein S6 kinase, 90kDa, polypeptide 3 | 2.46E-09 | 19.7974 |
| 226925_at | ACPL2 | acid phosphatase-like 2 | 5.50E-10 | 19.7884 |
| 226213_at | ERBB3 | v-erb-b2 erythroblastic leukemia viral oncogene homolog 3 (avian) | 5.37E-08 | 19.769 |
| 218472_s_at | PELO | pelota homolog (Drosophila) | 1.59E-07 | 19.6812 |
| 223092_at | ANKH | ankylosis, progressive homolog (mouse) | 5.05E-09 | 19.6786 |
| 201377_at | UBAP2L | ubiquitin associated protein 2-like | 1.65E-06 | 19.6218 |
| 223381_at | NUF2 | NUF2, NDC80 kinetochore complex component, homolog (S. cerevisiae) | 8.27E-08 | 19.5186 |
| 225864_at | FAM84B | family with sequence similarity 84, member B | 5.23E-06 | 19.4885 |
| 227856_at | C4orf32 | chromosome 4 open reading frame 32 | 6.02E-06 | 19.4713 |
| 211948_x_at | BAT2L2 | HLA-B associated transcript 2-like 2 | 6.47E-09 | 19.4401 |
| 220651_s_at | MCM10 | minichromosome maintenance complex component 10 | 5.17E-07 | 19.4191 |
| 202134_s_at | WWTR1 | WW domain containing transcription regulator 1 | 1.17E-07 | 19.4119 |
| 203189_s_at | NDUFS8 | NADH dehydrogenase (ubiquinone) Fe-S protein 8, 23kDa (NADH-coenzyme Q reductase | 3.12E-09 | 19.3649 |
| 201697_s_at | DNMT1 | DNA (cytosine-5-)-methyltransferase 1 | 2.50E-09 | 19.3202 |
| 1554466_a_at | C16orf13 | chromosome 16 open reading frame 13 | 1.49E-07 | 19.2878 |
| 201716_at | SNX1 | sorting nexin 1 | 7.79E-09 | 19.171 |
| 205016_at | TGFA | transforming growth factor, alpha | 3.89E-07 | 19.1556 |
| 213410_at | C10orf137 | chromosome 10 open reading frame 137 | 5.74E-08 | 19.138 |
| 228106_at | DCAF16 | DDB1 and CUL4 associated factor 16 | 1.68E-07 | 19.1266 |
| 202697_at | NUDT21 | nudix (nucleoside diphosphate linked moiety X)-type motif 21 | 4.39E-06 | 18.9619 |
| 200964_at | UBA1 | ubiquitin-like modifier activating enzyme 1 | 1.30E-07 | 18.9501 |
| 214055_x_at | BAT2L2 | HLA-B associated transcript 2-like 2 | 1.18E-05 | 18.9441 |
| 223743_s_at | MRPL4 | mitochondrial ribosomal protein L4 | 6.09E-08 | 18.837 |
| 212543_at | AIM1 | absent in melanoma 1 | 3.91E-08 | 18.8255 |
| 223156_at | MRPS23 | mitochondrial ribosomal protein S23 | 3.09E-07 | 18.8169 |
| 227243_s_at | EBF3 | early B-cell factor 3 | 1.04E-08 | 18.8057 |
| 208235_x_at | GAGE12F /// GAGE12G /// GAGE12I /// GAGE5 /// GAGE7 | G antigen 12F /// G antigen 12G /// G antigen 12I /// G antigen 5 /// G antigen | 1.04E-08 | 18.8013 |
| 205324_s_at | FTSJ1 | FtsJ homolog 1 (E. coli) | 4.34E-09 | 18.6628 |
| 205042_at | GNE | glucosamine (UDP-N-acetyl)-2-epimerase/N-acetylmannosamine kinase | 4.29E-07 | 18.5951 |
| 226675_s_at | MALAT1 | metastasis associated lung adenocarcinoma transcript 1 (non-protein coding) | 0.0002112 | 18.5677 |
| 1554678_s_at | HNRPDL | heterogeneous nuclear ribonucleoprotein D-like | 7.52E-06 | 18.5644 |
| 210474_s_at | CDK11A /// CDK11B | cyclin-dependent kinase 11A /// cyclin-dependent kinase 11B | 3.31E-08 | 18.5464 |
| 203852_s_at | SMN1 /// SMN2 | survival of motor neuron 1, telomeric /// survival of motor neuron 2, centromeri | 9.06E-06 | 18.4447 |
| 210543_s_at | PRKDC | protein kinase, DNA-activated, catalytic polypeptide | 1.97E-07 | 18.4326 |
| 212776_s_at | OBSL1 | obscurin-like 1 | 2.51E-07 | 18.4223 |
| 224847_at | CDK6 | cyclin-dependent kinase 6 | 6.74E-09 | 18.4204 |
| 207325_x_at | MAGEA1 | melanoma antigen family A, 1 (directs expression of antigen MZ2-E) | 2.22E-08 | 18.3671 |
| 200710_at | ACADVL | acyl-CoA dehydrogenase, very long chain | 1.99E-06 | 18.3454 |
| 224579_at | SLC38A1 | solute carrier family 38, member 1 | 4.50E-07 | 18.3279 |
| 233655_s_at | HAUS6 | HAUS augmin-like complex, subunit 6 | 3.15E-08 | 18.3058 |
| 202230_s_at | CHERP | calcium homeostasis endoplasmic reticulum protein | 3.75E-07 | 18.2974 |
| 224903_at | CIRH1A | cirrhosis, autosomal recessive 1A (cirhin) | 1.82E-08 | 18.2837 |
| 203105_s_at | DNM1L | dynamin 1-like | 2.85E-08 | 18.2723 |
| 214728_x_at | SMARCA4 | SWI/SNF related, matrix associated, actin dependent regulator of chromatin, subf | 2.72E-07 | 18.2321 |
| 213400_s_at | TBL1X | transducin (beta)-like 1X-linked | 1.05E-09 | 18.2181 |
| 229893_at | FRMD3 | FERM domain containing 3 | 2.01E-07 | 18.1843 |
| 200920_s_at | BTG1 | B-cell translocation gene 1, anti-proliferative | 1.56E-07 | 18.1704 |
| 224635_s_at | BIRC6 | baculoviral IAP repeat-containing 6 | 6.25E-08 | 18.1454 |
| 211651_s_at | LAMB1 | laminin, beta 1 | 6.42E-07 | 18.1112 |
| 201229_s_at | ARIH2 | ariadne homolog 2 (Drosophila) | 3.52E-07 | 18.0537 |
| 206560_s_at | MIA | melanoma inhibitory activity | 1.46E-08 | 18.0429 |
| 207826_s_at | ID3 | inhibitor of DNA binding 3, dominant negative helix-loop-helix protein | 2.28E-07 | 18.0229 |
| 231715_s_at | PYCR2 | pyrroline-5-carboxylate reductase family, member 2 | 2.20E-07 | 17.9431 |
| 204203_at | CEBPG | CCAAT/enhancer binding protein (C/EBP), gamma | 1.07E-05 | 17.915 |
| 241359_at | TLCD2 | TLC domain containing 2 | 3.83E-08 | 17.8576 |
| 218695_at | EXOSC4 | exosome component 4 | 4.45E-06 | 17.8132 |
| 226749_at | MRPS9 | mitochondrial ribosomal protein S9 | 1.68E-08 | 17.8122 |
| 226465_s_at | SON | SON DNA binding protein | 4.34E-07 | 17.8069 |
| 1555471_a_at | FMN2 | formin 2 | 4.94E-09 | 17.8062 |
| 231265_at | COX7B2 | cytochrome c oxidase subunit VIIb2 | 3.68E-08 | 17.7773 |
| 200913_at | PPM1G | protein phosphatase, Mg2+/Mn2+ dependent, 1G | 7.36E-06 | 17.7754 |
| 212060_at | SR140 | U2-associated SR140 protein | 2.76E-07 | 17.7518 |
| 225946_at | RASSF8 | Ras association (RalGDS/AF-6) domain family (N-terminal) member 8 | 4.17E-08 | 17.6837 |
| 202352_s_at | PSMD12 | proteasome (prosome, macropain) 26S subunit, non-ATPase, 12 | 1.56E-10 | 17.6557 |
| 239425_at | DCUN1D5 | DCN1, defective in cullin neddylation 1, domain containing 5 (S. cerevisiae) | 1.36E-06 | 17.6522 |
| 201920_at | SLC20A1 | solute carrier family 20 (phosphate transporter), member 1 | 2.42E-09 | 17.6401 |
| 212902_at | SEC24A | SEC24 family, member A (S. cerevisiae) | 2.16E-06 | 17.6348 |
| 201176_s_at | ARCN1 | archain 1 | 9.49E-09 | 17.6259 |
| 210372_s_at | TPD52L1 | tumor protein D52-like 1 | 4.77E-08 | 17.6044 |
| 204175_at | ZNF593 | zinc finger protein 593 | 4.63E-08 | 17.595 |
| 208938_at | PRCC | papillary renal cell carcinoma (translocation-associated) | 9.23E-08 | 17.5197 |
| 1553613_s_at | FOXC1 | forkhead box C1 | 3.05E-07 | 17.5084 |
| 218438_s_at | MED28 | mediator complex subunit 28 | 3.33E-07 | 17.5077 |
| 233970_s_at | TRMT6 | tRNA methyltransferase 6 homolog (S. cerevisiae) | 2.27E-07 | 17.5046 |
| 214095_at | SHMT2 | serine hydroxymethyltransferase 2 (mitochondrial) | 9.32E-08 | 17.5019 |
| 1554168_a_at | SH3KBP1 | SH3-domain kinase binding protein 1 | 3.17E-08 | 17.4506 |
| 200052_s_at | ILF2 | interleukin enhancer binding factor 2, 45kDa | 3.07E-10 | 17.4437 |
| 230493_at | SHISA2 | shisa homolog 2 (Xenopus laevis) | 3.85E-06 | 17.4427 |
| 1555427_s_at | SYNCRIP | synaptotagmin binding, cytoplasmic RNA interacting protein | 5.17E-08 | 17.4021 |
| 236381_s_at | WDR8 | WD repeat domain 8 | 1.03E-07 | 17.3893 |
| 205961_s_at | PSIP1 | PC4 and SFRS1 interacting protein 1 | 3.72E-07 | 17.3801 |
| 201631_s_at | IER3 | immediate early response 3 | 7.78E-08 | 17.3017 |
| 210102_at | VWA5A | von Willebrand factor A domain containing 5A | 2.71E-07 | 17.292 |
| 202289_s_at | TACC2 | transforming, acidic coiled-coil containing protein 2 | 4.88E-06 | 17.2888 |
| 223024_at | AP1M1 | adaptor-related protein complex 1, mu 1 subunit | 1.25E-08 | 17.2578 |
| 204521_at | C12orf24 | chromosome 12 open reading frame 24 | 1.14E-07 | 17.1951 |
| 211503_s_at | RAB14 | RAB14, member RAS oncogene family | 1.66E-07 | 17.1576 |
| 242093_at | SYTL5 | synaptotagmin-like 5 | 2.50E-09 | 17.151 |
| 208934_s_at | LGALS8 | lectin, galactoside-binding, soluble, 8 | 2.86E-08 | 17.1429 |
| 208310_s_at | C7orf28A /// C7orf28B | chromosome 7 open reading frame 28A /// chromosome 7 open reading frame 28B | 6.43E-07 | 17.1356 |
| 215193_x_at | HLA-DRB1 /// HLA-DRB3 /// HLA-DRB4 | major histocompatibility complex, class II, DR beta 1 /// major histocompatibili | 5.48E-08 | 17.1233 |
| 225512_at | ZBTB38 | zinc finger and BTB domain containing 38 | 1.10E-08 | 17.0891 |
| 204768_s_at | FEN1 | flap structure-specific endonuclease 1 | 2.21E-06 | 17.0352 |
| 201292_at | TOP2A | topoisomerase (DNA) II alpha 170kDa | 5.07E-06 | 17.0344 |
| 207606_s_at | ARHGAP12 | Rho GTPase activating protein 12 | 1.43E-06 | 16.9609 |
| 231718_at | SLU7 | SLU7 splicing factor homolog (S. cerevisiae) | 3.92E-08 | 16.9481 |
| 214313_s_at | EIF5B | Eukaryotic translation initiation factor 5B | 1.99E-07 | 16.9452 |
| 203360_s_at | MYCBP | c-myc binding protein | 2.76E-07 | 16.9298 |
| 224574_at | C17orf49 | chromosome 17 open reading frame 49 | 2.03E-07 | 16.9216 |
| 208803_s_at | SRP72 | signal recognition particle 72kDa | 1.28E-07 | 16.9205 |
| 214290_s_at | HIST2H2AA3 /// HIST2H2AA4 | histone cluster 2, H2aa3 /// histone cluster 2, H2aa4 | 1.66E-06 | 16.8822 |
| 218336_at | PFDN2 | prefoldin subunit 2 | 1.52E-09 | 16.8792 |
| 210052_s_at | TPX2 | TPX2, microtubule-associated, homolog (Xenopus laevis) | 1.40E-09 | 16.8776 |
| 214527_s_at | PQBP1 | polyglutamine binding protein 1 | 3.60E-06 | 16.8669 |
| 208879_x_at | PRPF6 | PRP6 pre-mRNA processing factor 6 homolog (S. cerevisiae) | 1.04E-06 | 16.8343 |
| 236814_at | MDM4 | Mdm4 p53 binding protein homolog (mouse) | 1.34E-08 | 16.7845 |
| 203087_s_at | KIF2A | kinesin heavy chain member 2A | 1.40E-06 | 16.7484 |
| 205364_at | ACOX2 | acyl-CoA oxidase 2, branched chain | 3.97E-09 | 16.7136 |
| 207281_x_at | VCX2 | variable charge, X-linked 2 | 1.61E-10 | 16.704 |
| 212088_at | PMPCA | peptidase (mitochondrial processing) alpha | 5.95E-07 | 16.6967 |
| 224596_at | SLC44A1 | solute carrier family 44, member 1 | 3.48E-07 | 16.6747 |
| 216685_s_at | MTAP | methylthioadenosine phosphorylase | 2.30E-09 | 16.6682 |
| 208744_x_at | HSPH1 | heat shock 105kDa/110kDa protein 1 | 2.48E-06 | 16.6644 |
| 218866_s_at | POLR3K | polymerase (RNA) III (DNA directed) polypeptide K, 12.3 kDa | 7.72E-09 | 16.6443 |
| 227249_at | NDE1 | NudE nuclear distribution gene E homolog 1 (A. nidulans) | 4.50E-09 | 16.6312 |
| 203946_s_at | ARG2 | arginase, type II | 1.66E-07 | 16.6119 |
| 211450_s_at | MSH6 | mutS homolog 6 (E. coli) | 4.06E-07 | 16.6035 |
| 208723_at | USP11 | ubiquitin specific peptidase 11 | 1.61E-08 | 16.5939 |
| 201252_at | PSMC4 | proteasome (prosome, macropain) 26S subunit, ATPase, 4 | 5.33E-08 | 16.5681 |
| 201714_at | TUBG1 | tubulin, gamma 1 | 1.58E-11 | 16.507 |
| 226319_s_at | THOC4 | THO complex 4 | 6.64E-07 | 16.4871 |
| 226897_s_at | ZC3H7A | zinc finger CCCH-type containing 7A | 9.53E-08 | 16.4867 |
| 201023_at | TAF7 | TAF7 RNA polymerase II, TATA box binding protein (TBP)-associated factor, 55kDa | 3.68E-08 | 16.4674 |
| 201423_s_at | CUL4A | cullin 4A | 1.37E-07 | 16.4009 |
| 209080_x_at | GLRX3 | glutaredoxin 3 | 1.59E-09 | 16.3895 |
| 231984_at | MTAP | methylthioadenosine phosphorylase | 1.89E-07 | 16.3702 |
| 211686_s_at | MAK16 | MAK16 homolog (S. cerevisiae) | 8.43E-08 | 16.3363 |
| 209421_at | MSH2 | mutS homolog 2, colon cancer, nonpolyposis type 1 (E. coli) | 2.48E-07 | 16.3262 |
| 210502_s_at | PPIE | peptidylprolyl isomerase E (cyclophilin E) | 2.91E-09 | 16.2695 |
| 222608_s_at | ANLN | anillin, actin binding protein | 8.21E-06 | 16.2657 |
| 228077_at | MRI1 | methylthioribose-1-phosphate isomerase homolog (S. cerevisiae) | 2.18E-08 | 16.2163 |
| 211375_s_at | ILF3 | interleukin enhancer binding factor 3, 90kDa | 1.89E-07 | 16.2043 |
| 227520_at | TXLNG | taxilin gamma | 2.68E-09 | 16.1994 |
| 201593_s_at | ZC3H15 | zinc finger CCCH-type containing 15 | 1.55E-06 | 16.1357 |
| 225519_at | PPP4R2 | protein phosphatase 4, regulatory subunit 2 | 8.44E-07 | 16.1329 |
| 202413_s_at | USP1 | ubiquitin specific peptidase 1 | 9.65E-08 | 16.1172 |
| 210092_at | MAGOH | mago-nashi homolog, proliferation-associated (Drosophila) | 1.53E-08 | 16.1135 |
| 212515_s_at | DDX3X | DEAD (Asp-Glu-Ala-Asp) box polypeptide 3, X-linked | 3.34E-06 | 16.1123 |
| 202613_at | CTPS | CTP synthase | 4.90E-08 | 16.0976 |
| 218014_at | NUP85 | nucleoporin 85kDa | 4.29E-09 | 16.0777 |
| 213750_at | RSL1D1 | ribosomal L1 domain containing 1 | 7.72E-08 | 16.0458 |
| 201564_s_at | FSCN1 | fascin homolog 1, actin-bundling protein (Strongylocentrotus purpuratus) | 1.42E-06 | 16.0303 |
| 218859_s_at | ESF1 | ESF1, nucleolar pre-rRNA processing protein, homolog (S. cerevisiae) | 3.22E-06 | 15.9873 |
| 205321_at | EIF2S3 | eukaryotic translation initiation factor 2, subunit 3 gamma, 52kDa | 9.05E-08 | 15.9462 |
| 202412_s_at | USP1 | ubiquitin specific peptidase 1 | 3.10E-06 | 15.8924 |
| 218663_at | NCAPG | non-SMC condensin I complex, subunit G | 3.25E-05 | 15.8555 |
| 203103_s_at | PRPF19 | PRP19/PSO4 pre-mRNA processing factor 19 homolog (S. cerevisiae) | 2.07E-08 | 15.8525 |
| 201251_at | PKM2 | pyruvate kinase, muscle | 6.12E-08 | 15.8315 |
| 221517_s_at | MED17 | mediator complex subunit 17 | 1.44E-07 | 15.8016 |
| 209580_s_at | MBD4 | methyl-CpG binding domain protein 4 | 1.29E-07 | 15.7576 |
| 210511_s_at | INHBA | inhibin, beta A | 4.74E-09 | 15.6734 |
| 201182_s_at | CHD4 | chromodomain helicase DNA binding protein 4 | 1.60E-08 | 15.6708 |
| 225564_at | SPATA13 | spermatogenesis associated 13 | 1.37E-06 | 15.6289 |
| 221007_s_at | FIP1L1 | FIP1 like 1 (S. cerevisiae) | 1.10E-06 | 15.5897 |
| 220688_s_at | MRTO4 | mRNA turnover 4 homolog (S. cerevisiae) | 2.78E-05 | 15.5868 |
| 227145_at | LOXL4 | lysyl oxidase-like 4 | 2.98E-08 | 15.5611 |
| 211015_s_at | HSPA4 | heat shock 70kDa protein 4 | 1.10E-07 | 15.5588 |
| 209825_s_at | UCK2 | uridine-cytidine kinase 2 | 3.39E-09 | 15.5549 |
| 212604_at | MRPS31 | mitochondrial ribosomal protein S31 | 1.50E-06 | 15.5223 |
| 201101_s_at | BCLAF1 | BCL2-associated transcription factor 1 | 8.34E-07 | 15.51 |
| 1555278_a_at | CKAP5 | cytoskeleton associated protein 5 | 2.01E-07 | 15.5073 |
| 235096_at | LEO1 | Leo1, Paf1/RNA polymerase II complex component, homolog (S. cerevisiae) | 1.09E-06 | 15.4643 |
| 236429_at | ZNF83 | zinc finger protein 83 | 5.71E-06 | 15.4625 |
| 212168_at | RBM12 | RNA binding motif protein 12 | 9.48E-08 | 15.447 |
| 205315_s_at | SNTB2 | syntrophin, beta 2 (dystrophin-associated protein A1, 59kDa, basic component 2) | 1.03E-10 | 15.4281 |
| 212709_at | NUP160 | nucleoporin 160kDa | 6.41E-06 | 15.4217 |
| 202900_s_at | NUP88 | nucleoporin 88kDa | 5.21E-09 | 15.3978 |
| 226184_at | FMNL2 | formin-like 2 | 3.12E-08 | 15.3853 |
| 225647_s_at | CTSC | cathepsin C | 4.58E-10 | 15.3423 |
| 219553_at | NME7 | non-metastatic cells 7, protein expressed in (nucleoside-diphosphate kinase) | 1.16E-07 | 15.3288 |
| 238346_s_at | TGS1 | trimethylguanosine synthase homolog (S. cerevisiae) | 3.36E-08 | 15.3266 |
| 211071_s_at | MLLT11 | myeloid/lymphoid or mixed-lineage leukemia (trithorax homolog, Drosophila); tran | 4.35E-12 | 15.3227 |
| 206059_at | ZNF91 | zinc finger protein 91 | 2.07E-07 | 15.315 |
| 204238_s_at | C6orf108 | chromosome 6 open reading frame 108 | 6.20E-07 | 15.3113 |
| 223666_at | SNX5 | sorting nexin 5 | 6.49E-07 | 15.31 |
| 213262_at | SACS | spastic ataxia of Charlevoix-Saguenay (sacsin) | 2.91E-06 | 15.2919 |
| 222443_s_at | RBM8A | RNA binding motif protein 8A | 4.72E-09 | 15.2603 |
| 209572_s_at | EED | embryonic ectoderm development | 1.53E-07 | 15.2401 |
| 212378_at | GART | phosphoribosylglycinamide formyltransferase, phosphoribosylglycinamide synthetas | 1.13E-07 | 15.2214 |
| 228310_at | ENAH | enabled homolog (Drosophila) | 1.22E-08 | 15.211 |
| 212693_at | MDN1 | MDN1, midasin homolog (yeast) | 5.58E-08 | 15.0767 |
| 218889_at | NOC3L | nucleolar complex associated 3 homolog (S. cerevisiae) | 8.88E-06 | 15.0678 |
| 229669_at | LOC440416 | hypothetical gene supported by BC072410 | 1.66E-06 | 15.0637 |
| 209142_s_at | UBE2G1 | ubiquitin-conjugating enzyme E2G 1 (UBC7 homolog, yeast) | 2.00E-07 | 15.0262 |
| 218398_at | MRPS30 | mitochondrial ribosomal protein S30 | 6.35E-10 | 15.019 |
| 204127_at | RFC3 | replication factor C (activator 1) 3, 38kDa | 2.46E-07 | 15.0162 |
| 222417_s_at | SNX5 | sorting nexin 5 | 2.55E-07 | 14.9768 |
| 221269_s_at | SH3BGRL3 | SH3 domain binding glutamic acid-rich protein like 3 | 2.09E-08 | 14.9763 |
| 217900_at | IARS2 | isoleucyl-tRNA synthetase 2, mitochondrial | 1.62E-08 | 14.9648 |
| 208800_at | SRP72 | signal recognition particle 72kDa | 8.84E-07 | 14.9466 |
| 235722_at | SYNJ2BP | synaptojanin 2 binding protein | 8.05E-07 | 14.9231 |
| 214464_at | CDC42BPA | CDC42 binding protein kinase alpha (DMPK-like) | 3.56E-07 | 14.9206 |
| 225253_s_at | METTL2A /// METTL2B | methyltransferase like 2A /// methyltransferase like 2B | 2.42E-08 | 14.9043 |
| 225740_x_at | MDM4 | Mdm4 p53 binding protein homolog (mouse) | 6.20E-09 | 14.8894 |
| 223251_s_at | ANKRD10 | ankyrin repeat domain 10 | 1.13E-10 | 14.8585 |
| 217221_x_at | RBM10 | RNA binding motif protein 10 | 2.74E-09 | 14.8353 |
| 206042_x_at | SNRPN /// SNURF | small nuclear ribonucleoprotein polypeptide N /// SNRPN upstream reading frame | 4.87E-08 | 14.8231 |
| 203176_s_at | TFAM | transcription factor A, mitochondrial | 7.93E-08 | 14.7984 |
| 213315_x_at | CXorf40A | chromosome X open reading frame 40A | 3.44E-07 | 14.7854 |
| 230407_at | SBNO1 | strawberry notch homolog 1 (Drosophila) | 6.33E-09 | 14.7818 |
| 216212_s_at | DKC1 | dyskeratosis congenita 1, dyskerin | 4.00E-07 | 14.7683 |
| 203341_at | CEBPZ | CCAAT/enhancer binding protein (C/EBP), zeta | 1.21E-09 | 14.7587 |
| 201666_at | TIMP1 | TIMP metallopeptidase inhibitor 1 | 1.48E-07 | 14.7416 |
| 242138_at | DLX1 | distal-less homeobox 1 | 1.78E-07 | 14.7413 |
| 217836_s_at | YY1AP1 | YY1 associated protein 1 | 1.08E-06 | 14.7369 |
| 221780_s_at | DDX27 | DEAD (Asp-Glu-Ala-Asp) box polypeptide 27 | 1.17E-08 | 14.7317 |
| 202106_at | GOLGA3 | golgin A3 | 1.67E-08 | 14.7114 |
| 213720_s_at | SMARCA4 | SWI/SNF related, matrix associated, actin dependent regulator of chromatin, subf | 2.83E-09 | 14.6761 |
| 203973_s_at | CEBPD | CCAAT/enhancer binding protein (C/EBP), delta | 7.95E-08 | 14.6574 |
| 202469_s_at | CPSF6 | cleavage and polyadenylation specific factor 6, 68kDa | 1.57E-08 | 14.6526 |
| 200990_at | TRIM28 | tripartite motif-containing 28 | 4.18E-07 | 14.6405 |
| 232008_s_at | BBX | bobby sox homolog (Drosophila) | 1.04E-06 | 14.6142 |
| 224631_at | ZFP91 | zinc finger protein 91 homolog (mouse) | 1.59E-08 | 14.6103 |
| 211289_x_at | CDK11A /// CDK11B | cyclin-dependent kinase 11A /// cyclin-dependent kinase 11B | 1.27E-07 | 14.5542 |
| 202356_s_at | GTF2F1 | general transcription factor IIF, polypeptide 1, 74kDa | 1.35E-06 | 14.5438 |
| 217957_at | C16orf80 | chromosome 16 open reading frame 80 | 4.50E-07 | 14.5382 |
| 209265_s_at | METTL3 | methyltransferase like 3 | 6.06E-08 | 14.5262 |
| 200628_s_at | WARS | tryptophanyl-tRNA synthetase | 5.30E-07 | 14.5052 |
| 218491_s_at | THYN1 | thymocyte nuclear protein 1 | 6.79E-06 | 14.5014 |
| 1555226_s_at | C1orf43 | chromosome 1 open reading frame 43 | 2.02E-05 | 14.4928 |
| 228523_at | NANOS1 | nanos homolog 1 (Drosophila) | 8.71E-08 | 14.4597 |
| 222991_s_at | UBQLN1 | ubiquilin 1 | 3.50E-09 | 14.4429 |
| 212898_at | KIAA0406 | KIAA0406 | 3.78E-08 | 14.4347 |
| 204957_at | ORC5L | origin recognition complex, subunit 5-like (yeast) | 4.65E-08 | 14.3824 |
| 225827_at | EIF2C2 | eukaryotic translation initiation factor 2C, 2 | 1.58E-07 | 14.3771 |
| 224634_at | GPATCH4 | G patch domain containing 4 | 6.48E-06 | 14.3575 |
| 225391_at | LOC93622 | hypothetical LOC93622 | 8.26E-06 | 14.3535 |
| 223241_at | SNX8 | sorting nexin 8 | 2.24E-07 | 14.3373 |
| 203818_s_at | SF3A3 | splicing factor 3a, subunit 3, 60kDa | 1.58E-09 | 14.3339 |
| 204480_s_at | C9orf16 | chromosome 9 open reading frame 16 | 1.32E-07 | 14.3153 |
| 204967_at | SHROOM2 | shroom family member 2 | 6.33E-09 | 14.3008 |
| 200843_s_at | EPRS | glutamyl-prolyl-tRNA synthetase | 7.78E-07 | 14.2886 |
| 225996_at | LONRF2 | LON peptidase N-terminal domain and ring finger 2 | 8.30E-08 | 14.288 |
| 201388_at | PSMD3 | proteasome (prosome, macropain) 26S subunit, non-ATPase, 3 | 7.04E-07 | 14.2798 |
| 223267_at | RG9MTD1 | RNA (guanine-9-) methyltransferase domain containing 1 | 1.25E-06 | 14.273 |
| 211363_s_at | MTAP | methylthioadenosine phosphorylase | 8.24E-07 | 14.2685 |
| 1553976_a_at | DPCD | deleted in primary ciliary dyskinesia homolog (mouse) | 1.28E-07 | 14.2673 |
| 208772_at | ANKHD1 /// ANKHD1-EIF4EBP3 | ankyrin repeat and KH domain containing 1 /// ANKHD1-EIF4EBP3 readthrough | 1.86E-07 | 14.2622 |
| 223020_at | CLPTM1L | CLPTM1-like | 7.22E-07 | 14.2337 |
| 202986_at | ARNT2 | aryl-hydrocarbon receptor nuclear translocator 2 | 3.32E-09 | 14.2226 |
| 225904_at | C1orf96 | chromosome 1 open reading frame 96 | 2.61E-08 | 14.22 |
| 201935_s_at | EIF4G3 | eukaryotic translation initiation factor 4 gamma, 3 | 1.17E-09 | 14.2072 |
| 208984_x_at | RBM10 | RNA binding motif protein 10 | 2.56E-07 | 14.2039 |
| 203203_s_at | KRR1 | KRR1, small subunit (SSU) processome component, homolog (yeast) | 1.79E-06 | 14.2025 |
| 227864_s_at | FAM125A | family with sequence similarity 125, member A | 8.92E-07 | 14.1997 |
| 222421_at | UBE2H | ubiquitin-conjugating enzyme E2H (UBC8 homolog, yeast) | 3.70E-08 | 14.1711 |
| 209796_s_at | CNPY2 | canopy 2 homolog (zebrafish) | 8.86E-09 | 14.1524 |
| 224516_s_at | CXXC5 | CXXC finger 5 | 4.86E-08 | 14.1089 |
| 204444_at | KIF11 | kinesin family member 11 | 5.72E-08 | 14.0814 |
| 202722_s_at | GFPT1 | glutamine--fructose-6-phosphate transaminase 1 | 7.83E-08 | 14.076 |
| 219037_at | RRP15 | ribosomal RNA processing 15 homolog (S. cerevisiae) | 1.21E-06 | 14.0543 |
| 209903_s_at | ATR | ataxia telangiectasia and Rad3 related | 3.21E-07 | 14.0478 |
| 226430_at | RELL1 | RELT-like 1 | 2.88E-07 | 14.0314 |
| 217851_s_at | SLMO2 | slowmo homolog 2 (Drosophila) | 1.07E-05 | 14.0175 |
| 201490_s_at | PPIF | peptidylprolyl isomerase F | 1.00E-07 | 14.0075 |
| 212036_s_at | PNN | pinin, desmosome associated protein | 9.67E-08 | 14.0058 |
| 201614_s_at | RUVBL1 | RuvB-like 1 (E. coli) | 4.87E-06 | 13.9849 |
| 225450_at | AMOTL1 | angiomotin like 1 | 2.56E-09 | 13.9815 |
| 213454_at | APITD1 | apoptosis-inducing, TAF9-like domain 1 | 4.21E-09 | 13.9697 |
| 219155_at | PITPNC1 | phosphatidylinositol transfer protein, cytoplasmic 1 | 1.65E-06 | 13.9685 |
| 208670_s_at | EID1 | EP300 interacting inhibitor of differentiation 1 | 4.25E-09 | 13.9553 |
| 201583_s_at | SEC23B | Sec23 homolog B (S. cerevisiae) | 1.06E-08 | 13.94 |
| 212231_at | FBXO21 | F-box protein 21 | 6.52E-08 | 13.9386 |
| 218107_at | WDR26 | WD repeat domain 26 | 1.52E-06 | 13.9171 |
| 221933_at | NLGN4X | neuroligin 4, X-linked | 1.82E-08 | 13.8969 |
| 213844_at | HOXA5 | homeobox A5 | 6.31E-08 | 13.8784 |
| 203786_s_at | TPD52L1 | tumor protein D52-like 1 | 1.38E-08 | 13.8736 |
| 225730_s_at | THUMPD3 | THUMP domain containing 3 | 5.15E-07 | 13.8703 |
| 202037_s_at | SFRP1 | secreted frizzled-related protein 1 | 5.94E-08 | 13.8663 |
| 207785_s_at | RBPJ | recombination signal binding protein for immunoglobulin kappa J region | 4.44E-07 | 13.8536 |
| 218595_s_at | HEATR1 | HEAT repeat containing 1 | 2.43E-08 | 13.8425 |
| 202170_s_at | AASDHPPT | aminoadipate-semialdehyde dehydrogenase-phosphopantetheinyl transferase | 3.90E-08 | 13.8361 |
| 208705_s_at | EIF5 | eukaryotic translation initiation factor 5 | 1.10E-07 | 13.8235 |
| 209654_at | KIAA0947 | KIAA0947 | 5.41E-07 | 13.8184 |
| 211558_s_at | DHPS | deoxyhypusine synthase | 2.40E-06 | 13.814 |
| 207336_at | SOX5 | SRY (sex determining region Y)-box 5 | 2.42E-07 | 13.8073 |
| 202296_s_at | RER1 | RER1 retention in endoplasmic reticulum 1 homolog (S. cerevisiae) | 1.99E-10 | 13.8028 |
| 208991_at | STAT3 | signal transducer and activator of transcription 3 (acute-phase response factor) | 1.24E-06 | 13.7942 |
| 225852_at | ANKRD17 | ankyrin repeat domain 17 | 2.01E-08 | 13.7914 |
| 227916_x_at | EXOSC3 | exosome component 3 | 9.12E-09 | 13.7804 |
| 235022_at | C18orf19 | chromosome 18 open reading frame 19 | 1.57E-07 | 13.7801 |
| 217959_s_at | TRAPPC4 | trafficking protein particle complex 4 | 4.44E-10 | 13.7769 |
| 222028_at | ZNF45 | zinc finger protein 45 | 1.68E-05 | 13.7523 |
| 223275_at | PRMT6 | protein arginine methyltransferase 6 | 5.10E-06 | 13.7429 |
| 219158_s_at | NAA15 | N(alpha)-acetyltransferase 15, NatA auxiliary subunit | 2.22E-07 | 13.7361 |
| 226290_at | BDP1 | B double prime 1, subunit of RNA polymerase III transcription initiation factor | 8.59E-07 | 13.7331 |
| 200815_s_at | PAFAH1B1 | platelet-activating factor acetylhydrolase 1b, regulatory subunit 1 (45kDa) | 5.49E-08 | 13.7273 |
| 235009_at | BOD1L | biorientation of chromosomes in cell division 1-like | 1.73E-06 | 13.719 |
| 203334_at | DHX8 | DEAH (Asp-Glu-Ala-His) box polypeptide 8 | 1.85E-06 | 13.719 |
| 203491_s_at | CEP57 | centrosomal protein 57kDa | 3.21E-06 | 13.717 |
| 222834_s_at | GNG12 | guanine nucleotide binding protein (G protein), gamma 12 | 8.85E-08 | 13.71 |
| 230876_at | ZNF883 | zinc finger protein 883 | 6.36E-06 | 13.7079 |
| 223230_at | PRPF38A | PRP38 pre-mRNA processing factor 38 (yeast) domain containing A | 5.60E-07 | 13.7 |
| 201548_s_at | KDM5B | lysine (K)-specific demethylase 5B | 1.47E-07 | 13.6994 |
| 218220_at | C12orf10 | chromosome 12 open reading frame 10 | 4.78E-06 | 13.6977 |
| 238949_at | RNF145 | ring finger protein 145 | 1.25E-05 | 13.6976 |
| 219015_s_at | ALG13 | asparagine-linked glycosylation 13 homolog (S. cerevisiae) | 7.34E-07 | 13.6831 |
| 213399_x_at | RPN2 | ribophorin II | 2.99E-09 | 13.6811 |
| 207739_s_at | GAGE1 /// GAGE12F /// GAGE12G /// GAGE12I /// GAGE12J /// GAGE2A /// GAGE2B /// GAGE2C /// GAGE2D /// GAGE2E /// GAGE3 /// GAGE4 /// GAGE5 /// GAGE6 /// GAGE7 /// GAGE8 | G antigen 1 /// G antigen 12F /// G antigen 12G /// G antigen 12I /// G antigen | 1.98E-06 | 13.6636 |
| 206385_s_at | ANK3 | ankyrin 3, node of Ranvier (ankyrin G) | 2.68E-08 | 13.6519 |
| 207831_x_at | DHPS | deoxyhypusine synthase | 1.38E-06 | 13.6305 |
| 91684_g_at | EXOSC4 | exosome component 4 | 4.87E-08 | 13.6256 |
| 202113_s_at | SNX2 | sorting nexin 2 | 4.41E-06 | 13.6142 |
| 219309_at | C22orf46 | chromosome 22 open reading frame 46 | 5.04E-07 | 13.6109 |
| 228095_at | PHF14 | PHD finger protein 14 | 1.54E-07 | 13.6108 |
| 230027_s_at | MRPL43 | mitochondrial ribosomal protein L43 | 6.30E-08 | 13.5781 |
| 201694_s_at | EGR1 | early growth response 1 | 1.89E-06 | 13.5504 |
| 225368_at | HIPK2 | homeodomain interacting protein kinase 2 | 2.05E-07 | 13.5442 |
| 205097_at | SLC26A2 | solute carrier family 26 (sulfate transporter), member 2 | 2.29E-08 | 13.523 |
| 1552310_at | C15orf40 | chromosome 15 open reading frame 40 | 2.30E-11 | 13.504 |
| 213581_at | PDCD2 | programmed cell death 2 | 2.26E-08 | 13.4865 |
| 218533_s_at | UCKL1 | uridine-cytidine kinase 1-like 1 | 1.03E-06 | 13.4787 |
| 214642_x_at | MAGEA5 | melanoma antigen family A, 5 | 3.17E-06 | 13.4535 |
| 201386_s_at | DHX15 | DEAH (Asp-Glu-Ala-His) box polypeptide 15 | 3.77E-09 | 13.4519 |
| 226952_at | EAF1 | ELL associated factor 1 | 5.79E-08 | 13.4484 |
| 203245_s_at | NCRNA00094 | non-protein coding RNA 94 | 2.86E-08 | 13.4382 |
| 209175_at | SEC23IP | SEC23 interacting protein | 3.61E-08 | 13.4358 |
| 202115_s_at | NOC2L | nucleolar complex associated 2 homolog (S. cerevisiae) | 6.76E-08 | 13.3821 |
| 215416_s_at | STOML2 | stomatin (EPB72)-like 2 | 1.78E-08 | 13.3739 |
| 224692_at | PPP1R15B | protein phosphatase 1, regulatory (inhibitor) subunit 15B | 1.18E-06 | 13.3733 |
| 205690_s_at | BUD31 | BUD31 homolog (S. cerevisiae) | 1.47E-10 | 13.3625 |
| 201014_s_at | PAICS | phosphoribosylaminoimidazole carboxylase, phosphoribosylaminoimidazole succinoca | 5.00E-08 | 13.3499 |
| 201645_at | TNC | tenascin C | 5.91E-06 | 13.3246 |
| 218235_s_at | UTP11L | UTP11-like, U3 small nucleolar ribonucleoprotein, (yeast) | 5.57E-09 | 13.3163 |
| 228341_at | NUDT16 | nudix (nucleoside diphosphate linked moiety X)-type motif 16 | 1.38E-08 | 13.2937 |
| 226424_at | CAPS | calcyphosine | 6.82E-08 | 13.2811 |
| 224177_s_at | CXorf26 | chromosome X open reading frame 26 | 4.22E-09 | 13.2785 |
| 222574_s_at | DHX40 | DEAH (Asp-Glu-Ala-His) box polypeptide 40 | 1.89E-08 | 13.2785 |
| 225676_s_at | DCAF13 | DDB1 and CUL4 associated factor 13 | 4.92E-07 | 13.2766 |
| 203060_s_at | PAPSS2 | 3'-phosphoadenosine 5'-phosphosulfate synthase 2 | 2.83E-09 | 13.2649 |
| 204630_s_at | GOSR1 | golgi SNAP receptor complex member 1 | 7.20E-10 | 13.2588 |
| 209408_at | KIF2C | kinesin family member 2C | 3.84E-07 | 13.2577 |
| 217999_s_at | PHLDA1 | pleckstrin homology-like domain, family A, member 1 | 4.25E-06 | 13.2569 |
| 225310_at | RBMX | RNA binding motif protein, X-linked | 9.42E-08 | 13.2426 |
| 208813_at | GOT1 | glutamic-oxaloacetic transaminase 1, soluble (aspartate aminotransferase 1) | 1.86E-06 | 13.2385 |
| 223229_at | UBE2T | ubiquitin-conjugating enzyme E2T (putative) | 4.70E-07 | 13.2148 |
| 208698_s_at | NONO | non-POU domain containing, octamer-binding | 6.50E-08 | 13.209 |
| 216860_s_at | GDF11 | growth differentiation factor 11 | 4.78E-07 | 13.2088 |
| 215089_s_at | RBM10 | RNA binding motif protein 10 | 1.39E-08 | 13.2084 |
| 234660_s_at | DIS3 | DIS3 mitotic control homolog (S. cerevisiae) | 1.10E-07 | 13.1956 |
| 212300_at | TXLNA | taxilin alpha | 3.39E-09 | 13.19 |
| 214501_s_at | H2AFY | H2A histone family, member Y | 2.91E-09 | 13.185 |
| 221513_s_at | UTP14A /// UTP14C | UTP14, U3 small nucleolar ribonucleoprotein, homolog A (yeast) /// UTP14, U3 sma | 9.33E-08 | 13.1738 |
| 220145_at | MAP9 | microtubule-associated protein 9 | 2.38E-06 | 13.1404 |
| 213729_at | PRPF40A | PRP40 pre-mRNA processing factor 40 homolog A (S. cerevisiae) | 2.67E-07 | 13.1379 |
| 205110_s_at | FGF13 | fibroblast growth factor 13 | 5.72E-07 | 13.1346 |
| 217828_at | SLTM | SAFB-like, transcription modulator | 5.99E-09 | 13.1152 |
| 215222_x_at | MACF1 | microtubule-actin crosslinking factor 1 | 9.29E-07 | 13.1138 |
| 218481_at | EXOSC5 | exosome component 5 | 1.57E-07 | 13.1051 |
| 223002_s_at | XRN2 | 5'-3' exoribonuclease 2 | 2.20E-07 | 13.1041 |
| 216088_s_at | PSMA7 | proteasome (prosome, macropain) subunit, alpha type, 7 | 7.61E-06 | 13.0972 |
| 205286_at | TFAP2C | transcription factor AP-2 gamma (activating enhancer binding protein 2 gamma) | 2.85E-07 | 13.0957 |
| 203068_at | KLHL21 | kelch-like 21 (Drosophila) | 2.64E-07 | 13.0929 |
| 212028_at | RBM25 | RNA binding motif protein 25 | 9.63E-09 | 13.0799 |
| 219717_at | DCAF16 | DDB1 and CUL4 associated factor 16 | 3.29E-06 | 13.0772 |
| 227067_x_at | NOTCH2NL | Notch homolog 2 (Drosophila) N-terminal like | 3.98E-09 | 13.0756 |
| 1558404_at | LOC644242 | Hypothetical protein LOC644242 | 4.16E-08 | 13.073 |
| 208663_s_at | TTC3 | tetratricopeptide repeat domain 3 | 4.24E-07 | 13.0617 |
| 219221_at | ZBTB38 | zinc finger and BTB domain containing 38 | 6.55E-08 | 13.0615 |
| 218214_at | C12orf44 | chromosome 12 open reading frame 44 | 3.22E-08 | 13.0491 |
| 1558678_s_at | MALAT1 | metastasis associated lung adenocarcinoma transcript 1 (non-protein coding) | 2.33E-06 | 13.0236 |
| 212429_s_at | GTF3C2 | general transcription factor IIIC, polypeptide 2, beta 110kDa | 8.33E-07 | 12.9999 |
| 218145_at | TRIB3 | tribbles homolog 3 (Drosophila) | 1.02E-06 | 12.9932 |
| 226426_at | ADNP | activity-dependent neuroprotector homeobox | 3.25E-07 | 12.9871 |
| 207086_x_at | GAGE1 /// GAGE12C /// GAGE12D /// GAGE12E /// GAGE12F /// GAGE12G /// GAGE12H /// GAGE12I /// GAGE12J /// GAGE2A /// GAGE2C /// GAGE2D /// GAGE2E /// GAGE4 /// GAGE5 /// GAGE6 /// GAGE7 /// GAGE8 | G antigen 1 /// G antigen 12C /// G antigen 12D /// G antigen 12E /// G antigen | 6.12E-08 | 12.977 |
| 212904_at | LRRC47 | leucine rich repeat containing 47 | 6.57E-09 | 12.9739 |
| 204809_at | CLPX | ClpX caseinolytic peptidase X homolog (E. coli) | 2.43E-06 | 12.9326 |
| 203048_s_at | TTC37 | tetratricopeptide repeat domain 37 | 4.22E-06 | 12.9218 |
| 242939_at | TFDP1 | transcription factor Dp-1 | 7.84E-06 | 12.8985 |
| 219348_at | USE1 | unconventional SNARE in the ER 1 homolog (S. cerevisiae) | 2.10E-07 | 12.8958 |
| 203358_s_at | EZH2 | enhancer of zeste homolog 2 (Drosophila) | 1.58E-06 | 12.8831 |
| 209024_s_at | SYNCRIP | synaptotagmin binding, cytoplasmic RNA interacting protein | 6.64E-07 | 12.8719 |
| 226528_at | MTX3 | metaxin 3 | 5.70E-11 | 12.8683 |
| 209945_s_at | GSK3B | glycogen synthase kinase 3 beta | 1.04E-07 | 12.8559 |
| 223457_at | COPG2 | coatomer protein complex, subunit gamma 2 | 2.66E-06 | 12.8523 |
| 213338_at | TMEM158 | transmembrane protein 158 (gene/pseudogene) | 9.91E-07 | 12.828 |
| 226517_at | BCAT1 | branched chain amino-acid transaminase 1, cytosolic | 4.60E-07 | 12.8218 |
| 227174_at | WDR72 | WD repeat domain 72 | 3.91E-07 | 12.8053 |
| 223110_at | KIAA1429 | KIAA1429 | 1.60E-07 | 12.7962 |
| 201689_s_at | TPD52 | tumor protein D52 | 1.61E-06 | 12.7856 |
| 218738_s_at | RNF138 | ring finger protein 138 | 7.25E-07 | 12.7833 |
| 208722_s_at | ANAPC5 | anaphase promoting complex subunit 5 | 3.46E-07 | 12.7812 |
| 201498_at | USP7 | ubiquitin specific peptidase 7 (herpes virus-associated) | 7.24E-08 | 12.7674 |
| 222975_s_at | CSDE1 | cold shock domain containing E1, RNA-binding | 5.80E-08 | 12.7628 |
| 218936_s_at | CCDC59 | coiled-coil domain containing 59 | 8.89E-07 | 12.7548 |
| 202642_s_at | TRRAP | transformation/transcription domain-associated protein | 1.58E-07 | 12.7476 |
| 222439_s_at | THRAP3 | thyroid hormone receptor associated protein 3 | 1.03E-06 | 12.7429 |
| 221706_s_at | USE1 | unconventional SNARE in the ER 1 homolog (S. cerevisiae) | 6.59E-07 | 12.7427 |
| 222209_s_at | TMEM135 | transmembrane protein 135 | 6.37E-07 | 12.7418 |
| 227404_s_at | EGR1 | Early growth response 1 | 6.95E-06 | 12.7351 |
| 223206_s_at | NMRAL1 | NmrA-like family domain containing 1 | 2.96E-08 | 12.7318 |
| 224848_at | CDK6 | cyclin-dependent kinase 6 | 6.21E-08 | 12.7171 |
| 212758_s_at | ZEB1 | zinc finger E-box binding homeobox 1 | 4.08E-07 | 12.71 |
| 201761_at | MTHFD2 | methylenetetrahydrofolate dehydrogenase (NADP+ dependent) 2, methenyltetrahydrof | 2.02E-09 | 12.699 |
| 225688_s_at | PHLDB2 | pleckstrin homology-like domain, family B, member 2 | 9.32E-06 | 12.6868 |
| 217755_at | HN1 | hematological and neurological expressed 1 | 5.09E-05 | 12.6838 |
| 226847_at | FST | follistatin | 6.47E-07 | 12.6644 |
| 231729_s_at | CAPS | calcyphosine | 5.12E-07 | 12.6634 |
| 217185_s_at | ZNF259 /// ZNF259P1 | zinc finger protein 259 /// zinc finger protein 259 pseudogene 1 | 2.93E-06 | 12.6604 |
| 209336_at | PWP2 | PWP2 periodic tryptophan protein homolog (yeast) | 1.42E-07 | 12.6603 |
| 219575_s_at | COG8 /// PDF | component of oligomeric golgi complex 8 /// peptide deformylase (mitochondrial) | 1.80E-07 | 12.6289 |
| 211509_s_at | RTN4 | reticulon 4 | 2.54E-05 | 12.6247 |
| 241937_s_at | WDR4 | WD repeat domain 4 | 2.55E-08 | 12.5834 |
| 212268_at | SERPINB1 | serpin peptidase inhibitor, clade B (ovalbumin), member 1 | 4.71E-09 | 12.5796 |
| 209340_at | UAP1 | UDP-N-acteylglucosamine pyrophosphorylase 1 | 8.83E-09 | 12.562 |
| 226591_at | SNRPN | small nuclear ribonucleoprotein polypeptide N | 1.61E-06 | 12.5381 |
| 211992_at | WNK1 | WNK lysine deficient protein kinase 1 | 5.67E-09 | 12.5168 |
| 214011_s_at | NOP16 | NOP16 nucleolar protein homolog (yeast) | 5.92E-09 | 12.5079 |
| 212689_s_at | KDM3A | lysine (K)-specific demethylase 3A | 1.97E-10 | 12.4943 |
| 212264_s_at | WAPAL | wings apart-like homolog (Drosophila) | 4.10E-07 | 12.4939 |
| 209233_at | EMG1 | EMG1 nucleolar protein homolog (S. cerevisiae) | 1.05E-06 | 12.4875 |
| 221806_s_at | SETD5 | SET domain containing 5 | 4.69E-07 | 12.4676 |
| 212896_at | SKIV2L2 | superkiller viralicidic activity 2-like 2 (S. cerevisiae) | 3.57E-10 | 12.4649 |
| 218001_at | MRPS2 | mitochondrial ribosomal protein S2 | 1.31E-06 | 12.4521 |
| 212229_s_at | FBXO21 | F-box protein 21 | 7.09E-07 | 12.4413 |
| 207165_at | HMMR | hyaluronan-mediated motility receptor (RHAMM) | 8.53E-08 | 12.4298 |
| 205748_s_at | RNF126 | ring finger protein 126 | 1.95E-08 | 12.4253 |
| 225196_s_at | MRPS26 | mitochondrial ribosomal protein S26 | 1.46E-07 | 12.4083 |
| 218049_s_at | MRPL13 | mitochondrial ribosomal protein L13 | 8.49E-08 | 12.4033 |
| 203525_s_at | APC | adenomatous polyposis coli | 1.50E-07 | 12.3958 |
| 212722_s_at | JMJD6 | jumonji domain containing 6 | 1.72E-06 | 12.3886 |
| 204022_at | WWP2 | WW domain containing E3 ubiquitin protein ligase 2 | 8.75E-09 | 12.373 |
| 201481_s_at | PYGB | phosphorylase, glycogen; brain | 1.59E-06 | 12.3726 |
| 203114_at | SSSCA1 | Sjogren syndrome/scleroderma autoantigen 1 | 3.95E-06 | 12.3694 |
| 204977_at | DDX10 | DEAD (Asp-Glu-Ala-Asp) box polypeptide 10 | 1.03E-07 | 12.3548 |
| 1555730_a_at | CFL1 | cofilin 1 (non-muscle) | 4.44E-06 | 12.353 |
| 201549_x_at | KDM5B | lysine (K)-specific demethylase 5B | 3.53E-08 | 12.3476 |
| 202179_at | BLMH | bleomycin hydrolase | 3.46E-07 | 12.3415 |
| 220924_s_at | SLC38A2 | solute carrier family 38, member 2 | 2.99E-07 | 12.3318 |
| 202753_at | PSMD6 | proteasome (prosome, macropain) 26S subunit, non-ATPase, 6 | 1.30E-09 | 12.3003 |
| 205046_at | CENPE | centromere protein E, 312kDa | 4.54E-09 | 12.2864 |
| 226914_at | ARPC5L | actin related protein 2/3 complex, subunit 5-like | 3.26E-09 | 12.2854 |
| 1553118_at | THEM4 | thioesterase superfamily member 4 | 2.37E-07 | 12.2242 |
| 212775_at | OBSL1 | obscurin-like 1 | 3.42E-07 | 12.2226 |
| 208689_s_at | RPN2 | ribophorin II | 1.10E-08 | 12.2216 |
| 203067_at | PDHX | pyruvate dehydrogenase complex, component X | 2.50E-09 | 12.2191 |
| 208155_x_at | GAGE1 /// GAGE12F /// GAGE12G /// GAGE12I /// GAGE12J /// GAGE4 /// GAGE5 /// GAGE6 /// GAGE7 | G antigen 1 /// G antigen 12F /// G antigen 12G /// G antigen 12I /// G antigen | 3.70E-07 | 12.2152 |
| 201479_at | DKC1 | dyskeratosis congenita 1, dyskerin | 6.37E-10 | 12.2063 |
| 226732_at | RBM33 | RNA binding motif protein 33 | 2.72E-08 | 12.1708 |
| 218100_s_at | IFT57 | intraflagellar transport 57 homolog (Chlamydomonas) | 1.29E-06 | 12.1512 |
| 226233_at | B3GALNT2 | beta-1,3-N-acetylgalactosaminyltransferase 2 | 2.65E-08 | 12.1231 |
| 225681_at | CTHRC1 | collagen triple helix repeat containing 1 | 4.67E-10 | 12.1087 |
| 201147_s_at | TIMP3 | TIMP metallopeptidase inhibitor 3 | 1.13E-09 | 12.1022 |
| 207624_s_at | RPGR | retinitis pigmentosa GTPase regulator | 1.27E-06 | 12.0997 |
| 226685_at | SNTB2 | syntrophin, beta 2 (dystrophin-associated protein A1, 59kDa, basic component 2) | 1.12E-08 | 12.0986 |
| 218782_s_at | ATAD2 | ATPase family, AAA domain containing 2 | 2.58E-07 | 12.0644 |
| 208821_at | SNRPB | small nuclear ribonucleoprotein polypeptides B and B1 | 4.01E-09 | 12.0592 |
| 224523_s_at | C3orf26 | chromosome 3 open reading frame 26 | 7.36E-07 | 12.0509 |
| 202481_at | DHRS3 | dehydrogenase/reductase (SDR family) member 3 | 4.20E-09 | 12.0491 |
| 224450_s_at | RIOK1 | RIO kinase 1 (yeast) | 4.52E-06 | 12.0486 |
| 233893_s_at | KIAA1530 | KIAA1530 | 3.67E-10 | 12.0222 |
| 202251_at | PRPF3 | PRP3 pre-mRNA processing factor 3 homolog (S. cerevisiae) | 2.42E-08 | 12.009 |
| 217850_at | GNL3 | guanine nucleotide binding protein-like 3 (nucleolar) | 2.72E-08 | 12.0082 |
| 217832_at | SYNCRIP | synaptotagmin binding, cytoplasmic RNA interacting protein | 6.84E-09 | 11.9994 |
| 211505_s_at | STAU1 | staufen, RNA binding protein, homolog 1 (Drosophila) | 1.46E-07 | 11.9629 |
| 214374_s_at | PPFIBP1 | PTPRF interacting protein, binding protein 1 (liprin beta 1) | 2.25E-05 | 11.9568 |
| 204396_s_at | GRK5 | G protein-coupled receptor kinase 5 | 5.75E-08 | 11.9436 |
| 213291_s_at | UBE3A | ubiquitin protein ligase E3A | 4.25E-07 | 11.9409 |
| 218277_s_at | DHX40 | DEAH (Asp-Glu-Ala-His) box polypeptide 40 | 3.68E-08 | 11.9326 |
| 228851_s_at | ENSA | endosulfine alpha | 9.35E-07 | 11.9306 |
| 210811_s_at | DDX49 | DEAD (Asp-Glu-Ala-Asp) box polypeptide 49 | 2.83E-07 | 11.9261 |
| 222450_at | PMEPA1 | prostate transmembrane protein, androgen induced 1 | 8.38E-08 | 11.9163 |
| 203905_at | PARN | poly(A)-specific ribonuclease (deadenylation nuclease) | 8.65E-08 | 11.9007 |
| 218883_s_at | MLF1IP | MLF1 interacting protein | 1.06E-08 | 11.9001 |
| 209417_s_at | IFI35 | interferon-induced protein 35 | 4.15E-07 | 11.8975 |
| 225646_at | CTSC | cathepsin C | 1.27E-06 | 11.8843 |
| 212247_at | NUP205 | nucleoporin 205kDa | 6.02E-08 | 11.8774 |
| 212075_s_at | CSNK2A1 | casein kinase 2, alpha 1 polypeptide | 2.14E-07 | 11.8733 |
| 218307_at | RSAD1 | radical S-adenosyl methionine domain containing 1 | 7.76E-06 | 11.8723 |
| 201459_at | RUVBL2 | RuvB-like 2 (E. coli) | 9.56E-07 | 11.859 |
| 203044_at | CHSY1 | chondroitin sulfate synthase 1 | 1.54E-07 | 11.8534 |
| 228857_at | GNL1 | guanine nucleotide binding protein-like 1 | 1.51E-07 | 11.8473 |
| 228293_at | DEPDC7 | DEP domain containing 7 | 2.62E-05 | 11.8304 |
| 219960_s_at | UCHL5 | ubiquitin carboxyl-terminal hydrolase L5 | 9.35E-10 | 11.8261 |
| 226005_at | UBE2G1 | ubiquitin-conjugating enzyme E2G 1 (UBC7 homolog, yeast) | 1.37E-08 | 11.8215 |
| 208610_s_at | SRRM2 | serine/arginine repetitive matrix 2 | 1.20E-07 | 11.8214 |
| 225035_x_at | BTNL8 /// LOC100288778 /// WASH1 /// WASH2P /// WASH3P /// WASH7P | butyrophilin-like 8 /// similar to WAS protein family homolog 1 /// WAS protein | 2.51E-06 | 11.8205 |
| 218757_s_at | UPF3B | UPF3 regulator of nonsense transcripts homolog B (yeast) | 4.14E-07 | 11.8147 |
| 218105_s_at | MRPL4 | mitochondrial ribosomal protein L4 | 9.60E-08 | 11.7956 |
| 213571_s_at | EIF4E2 | eukaryotic translation initiation factor 4E family member 2 | 2.88E-07 | 11.7894 |
| 208750_s_at | ARF1 | ADP-ribosylation factor 1 | 7.74E-07 | 11.7847 |
| 218080_x_at | FAF1 | Fas (TNFRSF6) associated factor 1 | 1.02E-06 | 11.7755 |
| 204914_s_at | SOX11 | SRY (sex determining region Y)-box 11 | 7.20E-08 | 11.7709 |
| 218590_at | C10orf2 | chromosome 10 open reading frame 2 | 1.27E-07 | 11.76 |
| 205541_s_at | GSPT2 | G1 to S phase transition 2 | 1.34E-05 | 11.7165 |
| 58696_at | EXOSC4 | exosome component 4 | 2.01E-09 | 11.7134 |
| 204868_at | ICT1 | immature colon carcinoma transcript 1 | 2.51E-07 | 11.7075 |
| 219212_at | HSPA14 | heat shock 70kDa protein 14 | 2.52E-06 | 11.7023 |
| 203392_s_at | CTBP1 | C-terminal binding protein 1 | 6.32E-08 | 11.7022 |
| 217786_at | PRMT5 | protein arginine methyltransferase 5 | 1.57E-06 | 11.696 |
| 202911_at | MSH6 | mutS homolog 6 (E. coli) | 1.54E-10 | 11.6946 |
| 234311_s_at | GTPBP10 | GTP-binding protein 10 (putative) | 3.16E-08 | 11.6901 |
| 204033_at | TRIP13 | thyroid hormone receptor interactor 13 | 3.07E-09 | 11.6899 |
| 211318_s_at | RAE1 | RAE1 RNA export 1 homolog (S. pombe) | 6.15E-09 | 11.6605 |
| 201264_at | COPE | coatomer protein complex, subunit epsilon | 4.41E-07 | 11.6524 |
| 209259_s_at | SMC3 | structural maintenance of chromosomes 3 | 2.12E-06 | 11.642 |
| 218206_x_at | SCAND1 | SCAN domain containing 1 | 4.23E-07 | 11.6397 |
| 43544_at | MED16 | mediator complex subunit 16 | 8.19E-07 | 11.6304 |
| 210337_s_at | ACLY | ATP citrate lyase | 5.14E-08 | 11.6248 |
| 205919_at | HBE1 | hemoglobin, epsilon 1 | 3.77E-07 | 11.6239 |
| 209312_x_at | HLA-DRB1 /// HLA-DRB4 /// HLA-DRB5 | major histocompatibility complex, class II, DR beta 1 /// major histocompatibili | 3.30E-07 | 11.6213 |
| 217885_at | IPO9 | importin 9 | 1.34E-07 | 11.6176 |
| 228204_at | PSMB4 | Proteasome (prosome, macropain) subunit, beta type, 4 | 1.84E-08 | 11.6125 |
| 232652_x_at | SCAND1 | SCAN domain containing 1 | 1.25E-05 | 11.6122 |
| 208114_s_at | ISG20L2 | interferon stimulated exonuclease gene 20kDa-like 2 | 8.30E-07 | 11.6067 |
| 203690_at | TUBGCP3 | tubulin, gamma complex associated protein 3 | 4.68E-07 | 11.6001 |
| 203968_s_at | CDC6 | cell division cycle 6 homolog (S. cerevisiae) | 2.88E-08 | 11.5979 |
| 210766_s_at | CSE1L | CSE1 chromosome segregation 1-like (yeast) | 2.06E-08 | 11.5832 |
| 223138_s_at | DHX36 | DEAH (Asp-Glu-Ala-His) box polypeptide 36 | 1.24E-07 | 11.583 |
| 222616_s_at | USP16 | ubiquitin specific peptidase 16 | 2.18E-07 | 11.5618 |
| 215733_x_at | CTAG2 | cancer/testis antigen 2 | 1.61E-06 | 11.5606 |
| 223219_s_at | CNOT10 | CCR4-NOT transcription complex, subunit 10 | 1.13E-07 | 11.5512 |
| 203542_s_at | KLF9 | Kruppel-like factor 9 | 1.82E-08 | 11.5452 |
| 220890_s_at | DDX47 | DEAD (Asp-Glu-Ala-Asp) box polypeptide 47 | 4.74E-08 | 11.5321 |
| 208734_x_at | RAB2A | RAB2A, member RAS oncogene family | 2.12E-06 | 11.5258 |
| 202758_s_at | RFXANK | regulatory factor X-associated ankyrin-containing protein | 6.44E-07 | 11.5254 |
| 238768_at | C2orf68 | chromosome 2 open reading frame 68 | 9.50E-07 | 11.5083 |
| 213775_x_at | ZNF638 | zinc finger protein 638 | 4.11E-08 | 11.5067 |
| 205003_at | DOCK4 | dedicator of cytokinesis 4 | 1.20E-05 | 11.5064 |
| 223038_s_at | FAM60A /// LOC728115 | family with sequence similarity 60, member A /// similar to family with sequence | 1.33E-07 | 11.5033 |
| 211501_s_at | EIF3B | eukaryotic translation initiation factor 3, subunit B | 1.05E-07 | 11.4805 |
| 207541_s_at | EXOSC10 | exosome component 10 | 2.30E-05 | 11.4797 |
| 217754_at | DDX56 | DEAD (Asp-Glu-Ala-Asp) box polypeptide 56 | 5.84E-08 | 11.4637 |
| 1554600_s_at | LMNA | lamin A/C | 1.22E-07 | 11.4578 |
| 226635_at | LOC401504 | Hypothetical gene supported by AK091718 | 4.66E-07 | 11.4545 |
| 211950_at | UBR4 | ubiquitin protein ligase E3 component n-recognin 4 | 1.46E-09 | 11.442 |
| 225702_at | C8orf76 | chromosome 8 open reading frame 76 | 9.72E-08 | 11.4376 |
| 209190_s_at | DIAPH1 | diaphanous homolog 1 (Drosophila) | 5.63E-08 | 11.4375 |
| 222667_s_at | ASH1L | ash1 (absent, small, or homeotic)-like (Drosophila) | 5.79E-08 | 11.4208 |
| 201281_at | ADRM1 | adhesion regulating molecule 1 | 1.04E-07 | 11.4163 |
| 228231_at | LOC100287081 | similar to hCG1999172 | 1.42E-07 | 11.4123 |
| 222740_at | ATAD2 | ATPase family, AAA domain containing 2 | 3.53E-06 | 11.4111 |
| 218678_at | NES | nestin | 2.43E-07 | 11.4077 |
| 206108_s_at | SFRS6 | splicing factor, arginine/serine-rich 6 | 6.69E-06 | 11.4009 |
| 205195_at | AP1S1 | adaptor-related protein complex 1, sigma 1 subunit | 3.17E-06 | 11.3901 |
| 227413_at | UBLCP1 | ubiquitin-like domain containing CTD phosphatase 1 | 1.38E-08 | 11.3604 |
| 1553709_a_at | PRPF38A | PRP38 pre-mRNA processing factor 38 (yeast) domain containing A | 2.34E-07 | 11.3558 |
| 214314_s_at | EIF5B | eukaryotic translation initiation factor 5B | 9.32E-06 | 11.3408 |
| 238462_at | UBASH3B | ubiquitin associated and SH3 domain containing B | 2.93E-07 | 11.3274 |
| 203976_s_at | CHAF1A | chromatin assembly factor 1, subunit A (p150) | 1.89E-06 | 11.3102 |
| 223711_s_at | THYN1 | thymocyte nuclear protein 1 | 5.06E-09 | 11.3092 |
| 213032_at | NFIB | nuclear factor I/B | 9.41E-08 | 11.3071 |
| 208948_s_at | STAU1 | staufen, RNA binding protein, homolog 1 (Drosophila) | 3.70E-11 | 11.3043 |
| 211971_s_at | LRPPRC | leucine-rich PPR-motif containing | 6.51E-09 | 11.2934 |
| 212434_at | GRPEL1 | GrpE-like 1, mitochondrial (E. coli) | 2.61E-06 | 11.2824 |
| 208685_x_at | BRD2 | bromodomain containing 2 | 7.71E-07 | 11.282 |
| 205443_at | SNAPC1 | small nuclear RNA activating complex, polypeptide 1, 43kDa | 8.09E-07 | 11.2734 |
| 209435_s_at | ARHGEF2 | Rho/Rac guanine nucleotide exchange factor (GEF) 2 | 5.77E-09 | 11.2615 |
| 225056_at | SIPA1L2 | signal-induced proliferation-associated 1 like 2 | 5.96E-07 | 11.255 |
| 211403_x_at | VCX2 | variable charge, X-linked 2 | 1.24E-07 | 11.2432 |
| 221020_s_at | SLC25A32 | solute carrier family 25, member 32 | 1.89E-06 | 11.2414 |
| 213302_at | PFAS | phosphoribosylformylglycinamidine synthase | 3.83E-07 | 11.2145 |
| 201664_at | SMC4 | structural maintenance of chromosomes 4 | 8.00E-08 | 11.2095 |
| 202301_s_at | RSRC2 | arginine/serine-rich coiled-coil 2 | 8.57E-08 | 11.2093 |
| 201606_s_at | PWP1 | PWP1 homolog (S. cerevisiae) | 6.83E-09 | 11.1903 |
| 202396_at | TCERG1 | transcription elongation regulator 1 | 1.93E-07 | 11.1895 |
| 214173_x_at | C19orf2 | chromosome 19 open reading frame 2 | 5.22E-07 | 11.1792 |
| 218268_at | TBC1D15 | TBC1 domain family, member 15 | 4.09E-08 | 11.1779 |
| 212058_at | SR140 | U2-associated SR140 protein | 8.82E-09 | 11.1625 |
| 204247_s_at | CDK5 | cyclin-dependent kinase 5 | 3.92E-08 | 11.1596 |
| 212458_at | SPRED2 | sprouty-related, EVH1 domain containing 2 | 4.46E-10 | 11.1532 |
| 220607_x_at | TH1L | TH1-like (Drosophila) | 6.78E-10 | 11.1398 |
| 209418_s_at | THOC5 | THO complex 5 | 1.03E-08 | 11.1379 |
| 203719_at | ERCC1 | excision repair cross-complementing rodent repair deficiency, complementation gr | 9.80E-08 | 11.1319 |
| 221741_s_at | YTHDF1 | YTH domain family, member 1 | 8.10E-09 | 11.1296 |
| 209094_at | DDAH1 | dimethylarginine dimethylaminohydrolase 1 | 3.36E-09 | 11.1164 |
| 206550_s_at | NUP155 | nucleoporin 155kDa | 9.06E-09 | 11.111 |
| 218027_at | MRPL15 | mitochondrial ribosomal protein L15 | 1.25E-06 | 11.1099 |
| 225120_at | PURB | purine-rich element binding protein B | 4.53E-08 | 11.093 |
| 206513_at | AIM2 | absent in melanoma 2 | 2.71E-06 | 11.0921 |
| 223043_at | TMEM85 | transmembrane protein 85 | 8.95E-09 | 11.0813 |
| 209864_at | FRAT2 | frequently rearranged in advanced T-cell lymphomas 2 | 4.39E-06 | 11.0754 |
| 208852_s_at | CANX | calnexin | 5.58E-08 | 11.0711 |
| 218308_at | TACC3 | transforming, acidic coiled-coil containing protein 3 | 7.47E-07 | 11.068 |
| 201454_s_at | NPEPPS | aminopeptidase puromycin sensitive | 1.13E-09 | 11.0666 |
| 213696_s_at | MED8 | mediator complex subunit 8 | 5.64E-08 | 11.0611 |
| 204640_s_at | SPOP | speckle-type POZ protein | 3.32E-10 | 11.049 |
| 225845_at | ZBTB44 | zinc finger and BTB domain containing 44 | 3.36E-07 | 11.0386 |
| 217964_at | TTC19 | tetratricopeptide repeat domain 19 | 8.15E-08 | 11.0347 |
| 202038_at | UBE4A | ubiquitination factor E4A (UFD2 homolog, yeast) | 5.28E-08 | 11.0256 |
| 225207_at | PDK4 | pyruvate dehydrogenase kinase, isozyme 4 | 1.93E-08 | 11.0239 |
| 225377_at | C9orf86 | chromosome 9 open reading frame 86 | 2.74E-06 | 11.0138 |
| 206015_s_at | FOXJ3 | forkhead box J3 | 1.07E-06 | 10.9962 |
| 208249_s_at | TGDS | TDP-glucose 4,6-dehydratase | 4.30E-08 | 10.9952 |
| 212742_at | RNF115 | ring finger protein 115 | 6.37E-07 | 10.9829 |
| 201391_at | TRAP1 | TNF receptor-associated protein 1 | 3.79E-09 | 10.9813 |
| 208895_s_at | DDX18 | DEAD (Asp-Glu-Ala-Asp) box polypeptide 18 | 1.00E-09 | 10.9793 |
| 204720_s_at | DNAJC6 | DnaJ (Hsp40) homolog, subfamily C, member 6 | 7.43E-07 | 10.978 |
| 229667_s_at | HOXB8 | homeobox B8 | 1.49E-06 | 10.9774 |
| 207828_s_at | CENPF | centromere protein F, 350/400ka (mitosin) | 1.59E-09 | 10.9585 |
| 202035_s_at | SFRP1 | secreted frizzled-related protein 1 | 1.09E-07 | 10.9567 |
| 200948_at | MLF2 | myeloid leukemia factor 2 | 7.27E-06 | 10.934 |
| 219258_at | TIPIN | TIMELESS interacting protein | 8.59E-09 | 10.9271 |
| 212158_at | SDC2 | syndecan 2 | 9.39E-07 | 10.9216 |
| 217927_at | SPCS1 | signal peptidase complex subunit 1 homolog (S. cerevisiae) | 6.78E-06 | 10.9203 |
| 202887_s_at | DDIT4 | DNA-damage-inducible transcript 4 | 2.80E-06 | 10.9173 |
| 202174_s_at | PCM1 | pericentriolar material 1 | 1.19E-06 | 10.9135 |
| 201087_at | PXN | paxillin | 6.29E-08 | 10.9022 |
| 225052_at | TMEM203 | transmembrane protein 203 | 7.35E-08 | 10.8841 |
| 209217_s_at | WDR45 | WD repeat domain 45 | 1.36E-06 | 10.8834 |
| 201901_s_at | YY1 | YY1 transcription factor | 4.15E-10 | 10.882 |
| 217788_s_at | GALNT2 | UDP-N-acetyl-alpha-D-galactosamine:polypeptide N-acetylgalactosaminyltransferase | 5.10E-07 | 10.8636 |
| 202693_s_at | STK17A | serine/threonine kinase 17a | 1.81E-06 | 10.8633 |
| 228423_at | MAP9 | microtubule-associated protein 9 | 6.69E-06 | 10.8524 |
| 202354_s_at | GTF2F1 | general transcription factor IIF, polypeptide 1, 74kDa | 2.34E-06 | 10.8498 |
| 225119_at | CHMP4B | chromatin modifying protein 4B | 4.65E-08 | 10.8485 |
| 218984_at | PUS7 | pseudouridylate synthase 7 homolog (S. cerevisiae) | 3.77E-08 | 10.8472 |
| 223439_at | NKAP | NFKB activating protein | 1.93E-07 | 10.8368 |
| 222559_s_at | RPRD1A | regulation of nuclear pre-mRNA domain containing 1A | 1.09E-07 | 10.8329 |
| 227522_at | CMBL | carboxymethylenebutenolidase homolog (Pseudomonas) | 6.82E-07 | 10.8321 |
| 219322_s_at | WDR8 | WD repeat domain 8 | 2.52E-08 | 10.8271 |
| 209398_at | HIST1H1C | histone cluster 1, H1c | 5.23E-06 | 10.8265 |
| 221712_s_at | WDR74 | WD repeat domain 74 | 2.76E-08 | 10.8258 |
| 223140_s_at | DHX36 | DEAH (Asp-Glu-Ala-His) box polypeptide 36 | 1.36E-07 | 10.8177 |
| 212653_s_at | EHBP1 | EH domain binding protein 1 | 1.14E-05 | 10.8168 |
| 218917_s_at | ARID1A | AT rich interactive domain 1A (SWI-like) | 7.03E-09 | 10.8087 |
| 219260_s_at | C17orf81 | chromosome 17 open reading frame 81 | 1.93E-08 | 10.8066 |
| 212603_at | MRPS31 | mitochondrial ribosomal protein S31 | 1.33E-05 | 10.7979 |
| 217478_s_at | HLA-DMA | major histocompatibility complex, class II, DM alpha | 5.94E-07 | 10.7953 |
| 204824_at | ENDOG | endonuclease G | 3.98E-07 | 10.7875 |
| 216471_x_at | SSX2 /// SSX2B | synovial sarcoma, X breakpoint 2 /// synovial sarcoma, X breakpoint 2B | 7.74E-07 | 10.7868 |
| 203320_at | SH2B3 | SH2B adaptor protein 3 | 8.56E-08 | 10.7858 |
| 221489_s_at | SPRY4 | sprouty homolog 4 (Drosophila) | 1.87E-08 | 10.7835 |
| 204476_s_at | PC | pyruvate carboxylase | 6.73E-08 | 10.7818 |
| 211672_s_at | ARPC4 | actin related protein 2/3 complex, subunit 4, 20kDa | 2.54E-05 | 10.7713 |
| 214149_s_at | ATP6V0E1 | ATPase, H+ transporting, lysosomal 9kDa, V0 subunit e1 | 9.32E-06 | 10.7643 |
| 218761_at | RNF111 | ring finger protein 111 | 2.15E-06 | 10.7577 |
| 219030_at | TPRKB | TP53RK binding protein | 8.44E-08 | 10.7492 |
| 225736_at | FBXO22 | F-box protein 22 | 4.19E-06 | 10.739 |
| 201587_s_at | IRAK1 | interleukin-1 receptor-associated kinase 1 | 4.53E-09 | 10.7388 |
| 214894_x_at | MACF1 | microtubule-actin crosslinking factor 1 | 1.89E-05 | 10.7361 |
| 202870_s_at | CDC20 | cell division cycle 20 homolog (S. cerevisiae) | 1.17E-06 | 10.7314 |
| 203058_s_at | PAPSS2 | 3'-phosphoadenosine 5'-phosphosulfate synthase 2 | 3.70E-07 | 10.7242 |
| 201830_s_at | NET1 | neuroepithelial cell transforming 1 | 3.40E-07 | 10.7226 |
| 211376_s_at | NSMCE4A | non-SMC element 4 homolog A (S. cerevisiae) | 2.01E-08 | 10.7206 |
| 207147_at | DLX2 | distal-less homeobox 2 | 1.62E-06 | 10.7146 |
| 201198_s_at | PSMD1 | proteasome (prosome, macropain) 26S subunit, non-ATPase, 1 | 7.86E-08 | 10.7085 |
| 205895_s_at | NOLC1 | nucleolar and coiled-body phosphoprotein 1 | 1.59E-06 | 10.7074 |
| 208815_x_at | HSPA4 | heat shock 70kDa protein 4 | 1.99E-08 | 10.6995 |
| 224806_at | TRIM25 | tripartite motif-containing 25 | 1.67E-09 | 10.6679 |
| 212073_at | CSNK2A1 /// CSNK2A1P | casein kinase 2, alpha 1 polypeptide /// casein kinase 2, alpha 1 polypeptide ps | 1.27E-06 | 10.665 |
| 224671_at | MRPL10 | mitochondrial ribosomal protein L10 | 1.49E-08 | 10.6591 |
| 224130_s_at | SRA1 | steroid receptor RNA activator 1 | 9.44E-07 | 10.6562 |
| 226320_at | THOC4 | THO complex 4 | 1.38E-06 | 10.6424 |
| 201997_s_at | SPEN | spen homolog, transcriptional regulator (Drosophila) | 1.10E-05 | 10.6416 |
| 200884_at | CKB | creatine kinase, brain | 1.41E-07 | 10.6303 |
| 212674_s_at | DHX30 | DEAH (Asp-Glu-Ala-His) box polypeptide 30 | 1.95E-05 | 10.6297 |
| 204948_s_at | FST | follistatin | 9.97E-07 | 10.6266 |
| 201867_s_at | TBL1X | transducin (beta)-like 1X-linked | 1.08E-05 | 10.6211 |
| 220239_at | KLHL7 | kelch-like 7 (Drosophila) | 2.48E-08 | 10.607 |
| 211257_x_at | ZNF638 | zinc finger protein 638 | 1.40E-07 | 10.6067 |
| 218755_at | KIF20A | kinesin family member 20A | 7.72E-08 | 10.6024 |
| 202491_s_at | IKBKAP | inhibitor of kappa light polypeptide gene enhancer in B-cells, kinase complex-as | 5.39E-07 | 10.602 |
| 217884_at | NAT10 | N-acetyltransferase 10 (GCN5-related) | 2.20E-08 | 10.597 |
| 209382_at | POLR3C | polymerase (RNA) III (DNA directed) polypeptide C (62kD) | 1.20E-06 | 10.5879 |
| 201017_at | EIF1AX | eukaryotic translation initiation factor 1A, X-linked | 1.43E-06 | 10.5861 |
| 225116_at | HIPK2 | homeodomain interacting protein kinase 2 | 3.15E-08 | 10.5858 |
| 209157_at | DNAJA2 | DnaJ (Hsp40) homolog, subfamily A, member 2 | 3.89E-07 | 10.5795 |
| 204557_s_at | DZIP1 | DAZ interacting protein 1 | 2.07E-06 | 10.5765 |
| 227105_at | CSPP1 | centrosome and spindle pole associated protein 1 | 1.61E-07 | 10.5593 |
| 212007_at | UBXN4 | UBX domain protein 4 | 7.49E-10 | 10.5564 |
| 212103_at | KPNA6 | karyopherin alpha 6 (importin alpha 7) | 2.61E-07 | 10.5504 |
| 201085_s_at | SON | SON DNA binding protein | 9.77E-05 | 10.5499 |
| 224566_at | NEAT1 | nuclear paraspeckle assembly transcript 1 (non-protein coding) | 2.77E-07 | 10.5492 |
| 202107_s_at | MCM2 | minichromosome maintenance complex component 2 | 9.52E-09 | 10.5482 |
| 208790_s_at | PTRF | polymerase I and transcript release factor | 2.17E-06 | 10.546 |
| 225741_at | THUMPD3 | THUMP domain containing 3 | 1.76E-06 | 10.5457 |
| 228662_at | SOCS7 | Suppressor of cytokine signaling 7 | 4.08E-07 | 10.5438 |
| 208708_x_at | EIF5 | eukaryotic translation initiation factor 5 | 7.17E-06 | 10.5411 |
| 225082_at | CPSF3 | cleavage and polyadenylation specific factor 3, 73kDa | 4.36E-07 | 10.5409 |
| 206854_s_at | MAP3K7 | mitogen-activated protein kinase kinase kinase 7 | 8.06E-08 | 10.54 |
| 218041_x_at | SLC38A2 | solute carrier family 38, member 2 | 1.02E-06 | 10.5333 |
| 207428_x_at | CDK11A /// CDK11B | cyclin-dependent kinase 11A /// cyclin-dependent kinase 11B | 3.27E-08 | 10.5184 |
| 223211_at | HACL1 | 2-hydroxyacyl-CoA lyase 1 | 1.99E-09 | 10.5152 |
| 201663_s_at | SMC4 | structural maintenance of chromosomes 4 | 3.50E-10 | 10.5148 |
| 209239_at | NFKB1 | nuclear factor of kappa light polypeptide gene enhancer in B-cells 1 | 2.27E-08 | 10.5097 |
| 219100_at | OBFC1 | oligonucleotide/oligosaccharide-binding fold containing 1 | 2.14E-08 | 10.5004 |
| 218397_at | FANCL | Fanconi anemia, complementation group L | 3.18E-06 | 10.4983 |
| 216232_s_at | GCN1L1 | GCN1 general control of amino-acid synthesis 1-like 1 (yeast) | 4.01E-07 | 10.4901 |
| 201065_s_at | GTF2I /// GTF2IP1 /// LOC100093631 | general transcription factor IIi /// general transcription factor IIi, pseudogen | 1.11E-06 | 10.4843 |
| 213852_at | RBM8A | RNA binding motif protein 8A | 4.63E-09 | 10.4739 |
| 208765_s_at | HNRNPR | heterogeneous nuclear ribonucleoprotein R | 4.46E-09 | 10.4529 |
| 201012_at | ANXA1 | annexin A1 | 1.53E-10 | 10.4487 |
| 202333_s_at | UBE2B | ubiquitin-conjugating enzyme E2B (RAD6 homolog) | 3.05E-08 | 10.4445 |
| 218602_s_at | HAUS6 | HAUS augmin-like complex, subunit 6 | 1.56E-06 | 10.4425 |
| 222430_s_at | YTHDF2 | YTH domain family, member 2 | 6.89E-07 | 10.4415 |
| 225932_s_at | HNRNPA2B1 | heterogeneous nuclear ribonucleoprotein A2/B1 | 4.85E-08 | 10.4336 |
| 218219_s_at | LANCL2 | LanC lantibiotic synthetase component C-like 2 (bacterial) | 7.26E-08 | 10.4282 |
| 202246_s_at | CDK4 | cyclin-dependent kinase 4 | 1.90E-10 | 10.4281 |
| 225766_s_at | TNPO1 | transportin 1 | 5.32E-07 | 10.4253 |
| 218448_at | C20orf11 | chromosome 20 open reading frame 11 | 4.29E-07 | 10.4166 |
| 203095_at | MTIF2 | mitochondrial translational initiation factor 2 | 1.81E-07 | 10.4142 |
| 212802_s_at | GAPVD1 | GTPase activating protein and VPS9 domains 1 | 8.09E-08 | 10.414 |
| 214435_x_at | RALA | v-ral simian leukemia viral oncogene homolog A (ras related) | 1.56E-05 | 10.4007 |
| 214499_s_at | BCLAF1 | BCL2-associated transcription factor 1 | 1.54E-06 | 10.3995 |
| 222668_at | KCTD15 | potassium channel tetramerisation domain containing 15 | 9.38E-09 | 10.3905 |
| 212801_at | CIT | citron (rho-interacting, serine/threonine kinase 21) | 4.96E-08 | 10.3899 |
| 201425_at | ALDH2 | aldehyde dehydrogenase 2 family (mitochondrial) | 1.41E-07 | 10.386 |
| 229346_at | NES | nestin | 1.47E-05 | 10.3852 |
| 211297_s_at | CDK7 | cyclin-dependent kinase 7 | 6.18E-09 | 10.3837 |
| 201512_s_at | TOMM70A | translocase of outer mitochondrial membrane 70 homolog A (S. cerevisiae) | 1.42E-06 | 10.3836 |
| 211954_s_at | IPO5 | importin 5 | 4.67E-09 | 10.3823 |
| 214744_s_at | RPL23 | ribosomal protein L23 | 6.25E-08 | 10.3797 |
| 226606_s_at | GTPBP5 | GTP binding protein 5 (putative) | 6.41E-08 | 10.3617 |
| 205996_s_at | AK2 | adenylate kinase 2 | 9.42E-07 | 10.3547 |
| 212532_s_at | LSM12 | LSM12 homolog (S. cerevisiae) | 7.70E-08 | 10.3483 |
| 225447_at | GPD2 | glycerol-3-phosphate dehydrogenase 2 (mitochondrial) | 2.95E-07 | 10.3474 |
| 218670_at | PUS1 | pseudouridylate synthase 1 | 4.94E-06 | 10.3403 |
| 202416_at | DNAJC7 | DnaJ (Hsp40) homolog, subfamily C, member 7 | 1.66E-08 | 10.3244 |
| 224250_s_at | SECISBP2 | SECIS binding protein 2 | 4.09E-10 | 10.3229 |
| 218331_s_at | C10orf18 | chromosome 10 open reading frame 18 | 9.55E-08 | 10.3202 |
| 201300_s_at | PRNP | prion protein | 6.02E-07 | 10.3194 |
| 217952_x_at | PHF3 | PHD finger protein 3 | 1.70E-06 | 10.3165 |
| 207956_x_at | PDS5B | PDS5, regulator of cohesion maintenance, homolog B (S. cerevisiae) | 2.12E-08 | 10.3105 |
| 216199_s_at | MAP3K4 | mitogen-activated protein kinase kinase kinase 4 | 7.21E-06 | 10.3084 |
| 210312_s_at | IFT20 | intraflagellar transport 20 homolog (Chlamydomonas) | 1.07E-06 | 10.3066 |
| 201499_s_at | USP7 | ubiquitin specific peptidase 7 (herpes virus-associated) | 2.62E-07 | 10.3066 |
| 200058_s_at | SNRNP200 | small nuclear ribonucleoprotein 200kDa (U5) | 6.68E-09 | 10.3058 |
| 202657_s_at | SERTAD2 | SERTA domain containing 2 | 1.32E-09 | 10.3047 |
| 205633_s_at | ALAS1 | aminolevulinate, delta-, synthase 1 | 8.37E-08 | 10.3008 |
| 221742_at | CELF1 | CUGBP, Elav-like family member 1 | 1.16E-08 | 10.289 |
| 212307_s_at | OGT | O-linked N-acetylglucosamine (GlcNAc) transferase (UDP-N-acetylglucosamine:polyp | 4.40E-07 | 10.2844 |
| 221773_at | ELK3 | ELK3, ETS-domain protein (SRF accessory protein 2) | 8.27E-06 | 10.2784 |
| 208625_s_at | EIF4G1 | eukaryotic translation initiation factor 4 gamma, 1 | 8.71E-08 | 10.2695 |
| 227787_s_at | MED30 | mediator complex subunit 30 | 1.15E-08 | 10.2664 |
| 201104_x_at | LOC200030 /// NBPF10 /// NBPF11 /// NBPF14 /// NBPF15 /// NBPF16 /// NBPF8 | neuroblastoma breakpoint family, member 11-like /// neuroblastoma breakpoint fam | 3.63E-10 | 10.2659 |
| 221522_at | ANKRD27 | ankyrin repeat domain 27 (VPS9 domain) | 2.44E-05 | 10.2635 |
| 212891_s_at | GADD45GIP1 | growth arrest and DNA-damage-inducible, gamma interacting protein 1 | 5.50E-08 | 10.2607 |
| 201096_s_at | ARF4 | ADP-ribosylation factor 4 | 9.42E-10 | 10.2598 |
| 212267_at | WAPAL | wings apart-like homolog (Drosophila) | 2.19E-08 | 10.2569 |
| 205168_at | DDR2 | discoidin domain receptor tyrosine kinase 2 | 7.48E-07 | 10.2488 |
| 1565717_s_at | FUS | fused in sarcoma | 8.63E-08 | 10.2394 |
| 202159_at | FARSA | phenylalanyl-tRNA synthetase, alpha subunit | 1.45E-08 | 10.2368 |
| 242648_at | KLHL8 | kelch-like 8 (Drosophila) | 7.01E-07 | 10.2262 |
| 228931_at | COQ4 | coenzyme Q4 homolog (S. cerevisiae) | 2.90E-07 | 10.2235 |
| 201615_x_at | CALD1 | caldesmon 1 | 8.23E-08 | 10.222 |
| 218103_at | FTSJ3 | FtsJ homolog 3 (E. coli) | 2.86E-06 | 10.2161 |
| 202671_s_at | PDXK | pyridoxal (pyridoxine, vitamin B6) kinase | 8.22E-07 | 10.2105 |
| 214359_s_at | HSP90AB1 | heat shock protein 90kDa alpha (cytosolic), class B member 1 | 1.11E-07 | 10.21 |
| 1554464_a_at | CRTAP | cartilage associated protein | 2.79E-06 | 10.2031 |
| 212648_at | DHX29 | DEAH (Asp-Glu-Ala-His) box polypeptide 29 | 2.53E-06 | 10.1989 |
| 220248_x_at | NSFL1C | NSFL1 (p97) cofactor (p47) | 1.51E-07 | 10.198 |
| 200069_at | SART3 | squamous cell carcinoma antigen recognized by T cells 3 | 2.91E-08 | 10.1917 |
| 224583_at | COTL1 | coactosin-like 1 (Dictyostelium) | 2.03E-08 | 10.1891 |
| 203607_at | INPP5F | inositol polyphosphate-5-phosphatase F | 9.22E-07 | 10.1878 |
| 212971_at | CARS | cysteinyl-tRNA synthetase | 9.28E-09 | 10.1828 |
| 34031_i_at | KRIT1 | KRIT1, ankyrin repeat containing | 1.14E-06 | 10.1798 |
| 201695_s_at | PNP | purine nucleoside phosphorylase | 7.13E-06 | 10.176 |
| 211997_x_at | H3F3B | H3 histone, family 3B (H3.3B) | 1.40E-10 | 10.1712 |
| 222607_s_at | DIS3 | DIS3 mitotic control homolog (S. cerevisiae) | 8.10E-06 | 10.168 |
| 210463_x_at | TRMT1 | TRM1 tRNA methyltransferase 1 homolog (S. cerevisiae) | 4.66E-07 | 10.159 |
| 218319_at | PELI1 | pellino homolog 1 (Drosophila) | 6.19E-07 | 10.1562 |
| 217821_s_at | WBP11 | WW domain binding protein 11 | 9.39E-08 | 10.1537 |
| 227766_at | LIG4 | ligase IV, DNA, ATP-dependent | 1.10E-05 | 10.1509 |
| 208794_s_at | SMARCA4 | SWI/SNF related, matrix associated, actin dependent regulator of chromatin, subf | 6.19E-08 | 10.148 |
| 214661_s_at | NOP14 | NOP14 nucleolar protein homolog (yeast) | 1.07E-06 | 10.1405 |
| 221974_at | IPW | imprinted in Prader-Willi syndrome (non-protein coding) | 3.80E-06 | 10.1215 |
| 222467_s_at | SAPS3 | SAPS domain family, member 3 | 5.90E-08 | 10.1208 |
| 202384_s_at | TCOF1 | Treacher Collins-Franceschetti syndrome 1 | 2.38E-07 | 10.1117 |
| 225493_at | LOC144438 | hypothetical LOC144438 | 2.56E-07 | 10.1083 |
| 213483_at | PPWD1 | peptidylprolyl isomerase domain and WD repeat containing 1 | 6.04E-07 | 10.0962 |
| 214047_s_at | MBD4 | methyl-CpG binding domain protein 4 | 3.39E-06 | 10.0953 |
| 213305_s_at | PPP2R5C | protein phosphatase 2, regulatory subunit B', gamma | 3.94E-06 | 10.0939 |
| 227447_at | SKIV2L2 | superkiller viralicidic activity 2-like 2 (S. cerevisiae) | 2.00E-08 | 10.0842 |
| 212725_s_at | TUG1 | taurine upregulated 1 (non-protein coding) | 7.61E-07 | 10.0714 |
| 210470_x_at | NONO | non-POU domain containing, octamer-binding | 1.02E-08 | 10.0658 |
| 201284_s_at | APEH | N-acylaminoacyl-peptide hydrolase | 2.26E-09 | 10.0584 |
| 202454_s_at | ERBB3 | v-erb-b2 erythroblastic leukemia viral oncogene homolog 3 (avian) | 9.02E-05 | 10.0505 |
| 216952_s_at | LMNB2 | lamin B2 | 6.69E-09 | 10.0456 |
| 202271_at | FBXO28 | F-box protein 28 | 2.15E-05 | 10.0423 |
| 224576_at | ERGIC1 | endoplasmic reticulum-golgi intermediate compartment (ERGIC) 1 | 9.88E-10 | 10.0416 |
| 217940_s_at | CARKD | carbohydrate kinase domain containing | 6.72E-08 | 10.0281 |
| 224217_s_at | FAF1 | Fas (TNFRSF6) associated factor 1 | 2.50E-08 | 10.0215 |
| 204513_s_at | ELMO1 | engulfment and cell motility 1 | 6.34E-07 | 10.0213 |
| 204772_s_at | TTF1 | transcription termination factor, RNA polymerase I | 7.38E-08 | 10.0194 |
| 208624_s_at | EIF4G1 | eukaryotic translation initiation factor 4 gamma, 1 | 6.79E-06 | 10.0093 |
| 1564520_s_at | PRMT5 | protein arginine methyltransferase 5 | 1.60E-08 | 10.0021 |
| 209533_s_at | PLAA | phospholipase A2-activating protein | 1.34E-06 | 10 |
| 226180_at | WDR36 | WD repeat domain 36 | 1.23E-08 | 9.99958 |
| 207079_s_at | MED6 | mediator complex subunit 6 | 3.87E-07 | 9.98719 |
| 203776_at | GPKOW | G patch domain and KOW motifs | 1.40E-07 | 9.98281 |
| 213252_at | SH3PXD2A | SH3 and PX domains 2A | 4.60E-06 | 9.97609 |
| 214427_at | NOP2 | NOP2 nucleolar protein homolog (yeast) | 8.15E-06 | 9.97279 |
| 223705_s_at | GPBP1 | GC-rich promoter binding protein 1 | 4.38E-06 | 9.97017 |
| 213135_at | TIAM1 | T-cell lymphoma invasion and metastasis 1 | 5.33E-06 | 9.96593 |
| 226364_at | HIP1 | Huntingtin interacting protein 1 | 2.74E-06 | 9.96577 |
| 218515_at | GCFC1 | GC-rich sequence DNA-binding factor 1 | 2.37E-07 | 9.96171 |
| 201529_s_at | RPA1 | replication protein A1, 70kDa | 2.01E-06 | 9.96164 |
| 213016_at | BBX | bobby sox homolog (Drosophila) | 1.50E-07 | 9.96072 |
| 225402_at | TP53RK | TP53 regulating kinase | 1.38E-06 | 9.94532 |
| 200828_s_at | ZNF207 | zinc finger protein 207 | 3.33E-06 | 9.93801 |
| 223414_s_at | LYAR | Ly1 antibody reactive homolog (mouse) | 2.88E-07 | 9.9259 |
| 222702_x_at | CRIPT | cysteine-rich PDZ-binding protein | 3.47E-09 | 9.92537 |
| 209196_at | WDR46 | WD repeat domain 46 | 6.86E-06 | 9.92375 |
| 218662_s_at | NCAPG | non-SMC condensin I complex, subunit G | 6.25E-06 | 9.92237 |
| 209017_s_at | LONP1 | lon peptidase 1, mitochondrial | 5.43E-10 | 9.9206 |
| 203819_s_at | IGF2BP3 | insulin-like growth factor 2 mRNA binding protein 3 | 8.16E-08 | 9.9197 |
| 200041_s_at | BAT1 | HLA-B associated transcript 1 | 4.83E-07 | 9.91075 |
| 212723_at | JMJD6 | jumonji domain containing 6 | 1.31E-06 | 9.91003 |
| 217980_s_at | MRPL16 | mitochondrial ribosomal protein L16 | 4.59E-06 | 9.90678 |
| 203209_at | RFC5 | replication factor C (activator 1) 5, 36.5kDa | 1.23E-07 | 9.90401 |
| 1555240_s_at | GNG12 | guanine nucleotide binding protein (G protein), gamma 12 | 2.26E-06 | 9.89297 |
| 227369_at | SERBP1 | SERPINE1 mRNA binding protein 1 | 9.74E-08 | 9.89287 |
| 207483_s_at | CAND1 | cullin-associated and neddylation-dissociated 1 | 6.02E-08 | 9.88949 |
| 226771_at | ATP8B2 | ATPase, class I, type 8B, member 2 | 4.79E-06 | 9.88268 |
| 213491_x_at | RPN2 | ribophorin II | 2.29E-07 | 9.88224 |
| 202146_at | IFRD1 | interferon-related developmental regulator 1 | 2.11E-06 | 9.88107 |
| 225163_at | FRMD4A | FERM domain containing 4A | 1.30E-05 | 9.87484 |
| 214102_at | ARAP2 | ArfGAP with RhoGAP domain, ankyrin repeat and PH domain 2 | 9.85E-06 | 9.87194 |
| 209980_s_at | SHMT1 | serine hydroxymethyltransferase 1 (soluble) | 1.53E-06 | 9.86751 |
| 225261_x_at | TH1L | TH1-like (Drosophila) | 2.01E-09 | 9.86727 |
| 209539_at | ARHGEF6 | Rac/Cdc42 guanine nucleotide exchange factor (GEF) 6 | 1.15E-09 | 9.86331 |
| 217339_x_at | CTAG1A /// CTAG1B | cancer/testis antigen 1A /// cancer/testis antigen 1B | 5.22E-08 | 9.86217 |
| 203422_at | POLD1 | polymerase (DNA directed), delta 1, catalytic subunit 125kDa | 6.92E-05 | 9.86075 |
| 208675_s_at | DDOST | dolichyl-diphosphooligosaccharide--protein glycosyltransferase | 3.58E-05 | 9.82901 |
| 203201_at | PMM2 | phosphomannomutase 2 | 6.56E-07 | 9.82755 |
| 201898_s_at | UBE2A | ubiquitin-conjugating enzyme E2A (RAD6 homolog) | 2.60E-07 | 9.81694 |
| 227378_x_at | C16orf13 | chromosome 16 open reading frame 13 | 1.38E-07 | 9.81393 |
| 202802_at | DHPS | deoxyhypusine synthase | 6.19E-09 | 9.80636 |
| 227305_s_at | SMCR8 | Smith-Magenis syndrome chromosome region, candidate 8 | 1.04E-07 | 9.80603 |
| 212919_at | DCP2 | DCP2 decapping enzyme homolog (S. cerevisiae) | 1.06E-07 | 9.8047 |
| 218788_s_at | SMYD3 | SET and MYND domain containing 3 | 1.03E-07 | 9.80346 |
| 227998_at | S100A16 | S100 calcium binding protein A16 | 1.18E-07 | 9.7998 |
| 201629_s_at | ACP1 | acid phosphatase 1, soluble | 1.12E-08 | 9.79946 |
| 228075_x_at | TFB1M | transcription factor B1, mitochondrial | 2.31E-10 | 9.79488 |
| 212721_at | SFRS12 | splicing factor, arginine/serine-rich 12 | 1.28E-06 | 9.78195 |
| 202545_at | PRKCD | protein kinase C, delta | 1.52E-07 | 9.77998 |
| 215498_s_at | MAP2K3 | mitogen-activated protein kinase kinase 3 | 1.51E-06 | 9.77969 |
| 225842_at | PHLDA1 | pleckstrin homology-like domain, family A, member 1 | 3.00E-07 | 9.77893 |
| 225038_s_at | SURF6 | surfeit 6 | 5.67E-05 | 9.77831 |
| 218935_at | EHD3 | EH-domain containing 3 | 5.46E-06 | 9.77769 |
| 1554555_a_at | SETD6 | SET domain containing 6 | 2.96E-07 | 9.76056 |
| 212422_at | PDCD11 | programmed cell death 11 | 3.10E-09 | 9.75754 |
| 208810_at | DNAJB6 /// TMEM135 | DnaJ (Hsp40) homolog, subfamily B, member 6 /// transmembrane protein 135 | 2.66E-06 | 9.75466 |
| 200918_s_at | SRPR | signal recognition particle receptor (docking protein) | 2.60E-08 | 9.75043 |
| 201770_at | SNRPA | small nuclear ribonucleoprotein polypeptide A | 5.47E-07 | 9.74757 |
| 212546_s_at | FRYL | FRY-like | 1.75E-06 | 9.73846 |
| 202323_s_at | ACBD3 | acyl-CoA binding domain containing 3 | 2.03E-05 | 9.73459 |
| 202706_s_at | UMPS | uridine monophosphate synthetase | 3.25E-05 | 9.73371 |
| 201589_at | SMC1A | structural maintenance of chromosomes 1A | 1.20E-07 | 9.733 |
| 206468_s_at | METTL13 | methyltransferase like 13 | 6.17E-07 | 9.72897 |
| 223011_s_at | OCIAD1 | OCIA domain containing 1 | 1.54E-05 | 9.72749 |
| 226800_at | EFCAB7 | EF-hand calcium binding domain 7 | 1.06E-06 | 9.72568 |
| 222572_at | PDP1 | pyruvate dehyrogenase phosphatase catalytic subunit 1 | 5.80E-06 | 9.70916 |
| 214882_s_at | SFRS2 | splicing factor, arginine/serine-rich 2 | 1.62E-08 | 9.70753 |
| 205361_s_at | PFDN4 | prefoldin subunit 4 | 1.16E-05 | 9.69699 |
| 201164_s_at | PUM1 | pumilio homolog 1 (Drosophila) | 9.84E-08 | 9.69405 |
| 222983_s_at | PAIP2 | poly(A) binding protein interacting protein 2 | 6.77E-09 | 9.68993 |
| 208828_at | POLE3 | polymerase (DNA directed), epsilon 3 (p17 subunit) | 1.18E-08 | 9.68824 |
| 218697_at | NCKIPSD | NCK interacting protein with SH3 domain | 2.20E-07 | 9.68476 |
| 200955_at | IMMT | inner membrane protein, mitochondrial | 2.41E-06 | 9.68071 |
| 205527_s_at | GEMIN4 | gem (nuclear organelle) associated protein 4 | 3.88E-05 | 9.67959 |
| 222792_s_at | CCDC59 | coiled-coil domain containing 59 | 1.97E-08 | 9.67117 |
| 227312_at | SNTB2 | syntrophin, beta 2 (dystrophin-associated protein A1, 59kDa, basic component 2) | 1.90E-07 | 9.66905 |
| 1558508_a_at | C1orf53 | chromosome 1 open reading frame 53 | 5.75E-07 | 9.6687 |
| 212048_s_at | YARS | tyrosyl-tRNA synthetase | 4.80E-08 | 9.65249 |
| 224721_at | WDR75 | WD repeat domain 75 | 2.36E-06 | 9.64783 |
| 200687_s_at | SF3B3 | splicing factor 3b, subunit 3, 130kDa | 1.27E-06 | 9.6402 |
| 228242_at | N4BP2 | NEDD4 binding protein 2 | 6.62E-05 | 9.63676 |
| 1559399_s_at | ZCCHC10 | zinc finger, CCHC domain containing 10 | 1.61E-06 | 9.62753 |
| 208910_s_at | C1QBP | complement component 1, q subcomponent binding protein | 1.15E-10 | 9.62503 |
| 223773_s_at | SNHG12 | small nucleolar RNA host gene 12 (non-protein coding) | 1.39E-07 | 9.61521 |
| 207877_s_at | NVL | nuclear VCP-like | 5.19E-09 | 9.61491 |
| 225104_at | ZNF598 | zinc finger protein 598 | 7.13E-07 | 9.61443 |
| 201603_at | PPP1R12A | protein phosphatase 1, regulatory (inhibitor) subunit 12A | 4.14E-07 | 9.61278 |
| 213794_s_at | NGDN | neuroguidin, EIF4E binding protein | 1.20E-07 | 9.60181 |
| 202505_at | SNRPB2 | small nuclear ribonucleoprotein polypeptide B'' | 1.63E-07 | 9.60137 |
| 201794_s_at | SMG7 | Smg-7 homolog, nonsense mediated mRNA decay factor (C. elegans) | 9.12E-08 | 9.59659 |
| 225866_at | RPF2 | ribosome production factor 2 homolog (S. cerevisiae) | 3.97E-10 | 9.5792 |
| 218244_at | NOL8 | nucleolar protein 8 | 2.78E-07 | 9.57425 |
| 215735_s_at | TSC2 | tuberous sclerosis 2 | 4.43E-06 | 9.57366 |
| 200868_s_at | RNF114 | ring finger protein 114 | 2.64E-06 | 9.57189 |
| 212961_x_at | CXorf40B | chromosome X open reading frame 40B | 1.46E-05 | 9.57149 |
| 224922_at | CSNK2A2 | casein kinase 2, alpha prime polypeptide | 2.19E-08 | 9.56654 |
| 202568_s_at | MARK3 | MAP/microtubule affinity-regulating kinase 3 | 5.13E-08 | 9.56603 |
| 202190_at | CSTF1 | cleavage stimulation factor, 3' pre-RNA, subunit 1, 50kDa | 2.58E-07 | 9.5514 |
| 200892_s_at | TRA2B | transformer 2 beta homolog (Drosophila) | 1.18E-07 | 9.5437 |
| 203446_s_at | OCRL | oculocerebrorenal syndrome of Lowe | 2.34E-06 | 9.53898 |
| 217911_s_at | BAG3 | BCL2-associated athanogene 3 | 6.44E-07 | 9.53409 |
| 210758_at | PSIP1 | PC4 and SFRS1 interacting protein 1 | 1.37E-07 | 9.5312 |
| 217909_s_at | MLX | MAX-like protein X | 2.47E-05 | 9.53086 |
| 211922_s_at | CAT | catalase | 8.20E-05 | 9.53024 |
| 201098_at | COPB2 | coatomer protein complex, subunit beta 2 (beta prime) | 5.99E-11 | 9.52564 |
| 208029_s_at | LAPTM4B | lysosomal protein transmembrane 4 beta | 5.57E-08 | 9.52192 |
| 208290_s_at | EIF5 | eukaryotic translation initiation factor 5 | 1.17E-06 | 9.51785 |
| 218098_at | ARFGEF2 | ADP-ribosylation factor guanine nucleotide-exchange factor 2 (brefeldin A-inhibi | 1.89E-06 | 9.51219 |
| 204610_s_at | CCDC85B | coiled-coil domain containing 85B | 2.45E-07 | 9.50084 |
| 203696_s_at | RFC2 | replication factor C (activator 1) 2, 40kDa | 6.41E-06 | 9.49959 |
| 201082_s_at | DCTN1 | dynactin 1 (p150, glued homolog, Drosophila) | 1.06E-07 | 9.49644 |
| 228645_at | SNHG9 | small nucleolar RNA host gene 9 (non-protein coding) | 9.98E-08 | 9.49389 |
| 1555495_a_at | CWC27 | CWC27 spliceosome-associated protein homolog (S. cerevisiae) | 9.77E-06 | 9.48447 |
| 218108_at | UBR7 | ubiquitin protein ligase E3 component n-recognin 7 (putative) | 1.23E-07 | 9.48425 |
| 208838_at | CAND1 | cullin-associated and neddylation-dissociated 1 | 1.10E-06 | 9.48138 |
| 212160_at | XPOT | exportin, tRNA (nuclear export receptor for tRNAs) | 4.66E-05 | 9.46972 |
| 206052_s_at | SLBP | stem-loop binding protein | 2.64E-06 | 9.46578 |
| 200790_at | ODC1 | ornithine decarboxylase 1 | 6.81E-10 | 9.46572 |
| 209861_s_at | METAP2 | methionyl aminopeptidase 2 | 6.62E-07 | 9.46211 |
| 215294_s_at | SMARCA1 | SWI/SNF related, matrix associated, actin dependent regulator of chromatin, subf | 3.82E-06 | 9.45997 |
| 219220_x_at | MRPS22 | mitochondrial ribosomal protein S22 | 1.84E-06 | 9.44738 |
| 210534_s_at | B9D1 | B9 protein domain 1 | 3.94E-06 | 9.44724 |
| 202420_s_at | DHX9 | DEAH (Asp-Glu-Ala-His) box polypeptide 9 | 1.95E-05 | 9.44672 |
| 217894_at | KCTD3 | potassium channel tetramerisation domain containing 3 | 7.06E-06 | 9.44277 |
| 216913_s_at | RRP12 | ribosomal RNA processing 12 homolog (S. cerevisiae) | 1.21E-05 | 9.44204 |
| 219108_x_at | DDX27 /// SS18 | DEAD (Asp-Glu-Ala-Asp) box polypeptide 27 /// synovial sarcoma translocation, ch | 2.42E-07 | 9.42901 |
| 208992_s_at | STAT3 | signal transducer and activator of transcription 3 (acute-phase response factor) | 5.50E-07 | 9.40825 |
| 219918_s_at | ASPM | asp (abnormal spindle) homolog, microcephaly associated (Drosophila) | 4.95E-10 | 9.40648 |
| 201652_at | COPS5 | COP9 constitutive photomorphogenic homolog subunit 5 (Arabidopsis) | 6.95E-09 | 9.4001 |
| 218082_s_at | UBP1 | upstream binding protein 1 (LBP-1a) | 1.28E-05 | 9.39655 |
| 227587_at | KRI1 | KRI1 homolog (S. cerevisiae) | 2.23E-07 | 9.3965 |
| 202963_at | RFX5 | regulatory factor X, 5 (influences HLA class II expression) | 2.25E-07 | 9.39174 |
| 222714_s_at | LACTB2 | lactamase, beta 2 | 3.02E-06 | 9.38941 |
| 225708_at | MED29 | mediator complex subunit 29 | 1.22E-06 | 9.38117 |
| 203764_at | DLGAP5 | discs, large (Drosophila) homolog-associated protein 5 | 1.40E-06 | 9.36877 |
| 201773_at | ADNP | activity-dependent neuroprotector homeobox | 8.97E-07 | 9.36574 |
| 202040_s_at | KDM5A | lysine (K)-specific demethylase 5A | 4.81E-07 | 9.36339 |
| 1558953_s_at | CEP164 | centrosomal protein 164kDa | 2.19E-08 | 9.36276 |
| 202748_at | GBP2 | guanylate binding protein 2, interferon-inducible | 8.57E-07 | 9.35924 |
| 235199_at | RNF125 | ring finger protein 125 | 1.12E-05 | 9.35795 |
| 227932_at | ARIH2 | ariadne homolog 2 (Drosophila) | 4.21E-06 | 9.34833 |
| 201639_s_at | CPSF1 | cleavage and polyadenylation specific factor 1, 160kDa | 2.28E-06 | 9.34683 |
| 200899_s_at | MGEA5 | meningioma expressed antigen 5 (hyaluronidase) | 3.92E-05 | 9.33724 |
| 207769_s_at | PQBP1 | polyglutamine binding protein 1 | 1.71E-06 | 9.33407 |
| 208637_x_at | ACTN1 | actinin, alpha 1 | 2.23E-07 | 9.33127 |
| 218961_s_at | PNKP | polynucleotide kinase 3'-phosphatase | 4.69E-08 | 9.3308 |
| 209511_at | POLR2F | polymerase (RNA) II (DNA directed) polypeptide F | 4.73E-08 | 9.32966 |
| 231307_at | LOC100130725 | similar to PAGE-5 protein | 2.63E-06 | 9.32795 |
| 224959_at | SLC26A2 | solute carrier family 26 (sulfate transporter), member 2 | 4.18E-06 | 9.32701 |
| 212816_s_at | CBS | cystathionine-beta-synthase | 1.68E-07 | 9.31831 |
| 208308_s_at | GPI | glucose-6-phosphate isomerase | 1.61E-07 | 9.30846 |
| 207628_s_at | WBSCR22 | Williams Beuren syndrome chromosome region 22 | 2.06E-06 | 9.30826 |
| 203720_s_at | ERCC1 | excision repair cross-complementing rodent repair deficiency, complementation gr | 4.28E-06 | 9.29988 |
| 208103_s_at | ANP32E | acidic (leucine-rich) nuclear phosphoprotein 32 family, member E | 3.72E-08 | 9.29593 |
| 201602_s_at | PPP1R12A | protein phosphatase 1, regulatory (inhibitor) subunit 12A | 1.52E-06 | 9.29269 |
| 203715_at | TBCE | tubulin folding cofactor E | 2.78E-06 | 9.29221 |
| 208684_at | COPA | coatomer protein complex, subunit alpha | 7.21E-09 | 9.28728 |
| 213007_at | FANCI | Fanconi anemia, complementation group I | 1.74E-09 | 9.28593 |
| 218123_at | C21orf59 | chromosome 21 open reading frame 59 | 3.98E-09 | 9.28152 |
| 202814_s_at | HEXIM1 | hexamethylene bis-acetamide inducible 1 | 1.02E-07 | 9.27857 |
| 209642_at | BUB1 | budding uninhibited by benzimidazoles 1 homolog (yeast) | 6.41E-08 | 9.27757 |
| 215009_s_at | SEC31A | SEC31 homolog A (S. cerevisiae) | 2.20E-05 | 9.27451 |
| 210213_s_at | EIF6 | eukaryotic translation initiation factor 6 | 6.87E-09 | 9.27337 |
| 200867_at | RNF114 | ring finger protein 114 | 2.64E-07 | 9.26868 |
| 201111_at | CSE1L | CSE1 chromosome segregation 1-like (yeast) | 5.54E-10 | 9.26666 |
| 223633_s_at | BCAN | brevican | 4.24E-06 | 9.26049 |
| 218064_s_at | AKAP8L | A kinase (PRKA) anchor protein 8-like | 2.85E-06 | 9.25769 |
| 226127_at | ALKBH3 | alkB, alkylation repair homolog 3 (E. coli) | 1.51E-07 | 9.25749 |
| 211382_s_at | TACC2 | transforming, acidic coiled-coil containing protein 2 | 2.14E-07 | 9.25292 |
| 203002_at | AMOTL2 | angiomotin like 2 | 1.03E-06 | 9.24925 |
| 225005_at | PHF13 | PHD finger protein 13 | 1.98E-06 | 9.24432 |
| 224705_s_at | TNRC6A | trinucleotide repeat containing 6A | 7.37E-06 | 9.23911 |
| 215696_s_at | SEC16A | SEC16 homolog A (S. cerevisiae) | 2.36E-08 | 9.22933 |
| 208985_s_at | EIF3J | eukaryotic translation initiation factor 3, subunit J | 1.96E-08 | 9.22752 |
| 208892_s_at | DUSP6 | dual specificity phosphatase 6 | 1.06E-05 | 9.22751 |
| 233955_x_at | CXXC5 | CXXC finger 5 | 9.14E-08 | 9.22642 |
| 202541_at | AIMP1 | aminoacyl tRNA synthetase complex-interacting multifunctional protein 1 | 1.98E-08 | 9.22107 |
| 225315_at | MRPL21 | mitochondrial ribosomal protein L21 | 1.73E-08 | 9.21802 |
| 208633_s_at | MACF1 | microtubule-actin crosslinking factor 1 | 4.55E-06 | 9.21598 |
| 218269_at | RNASEN | ribonuclease type III, nuclear | 1.55E-09 | 9.21385 |
| 203513_at | SPG11 | spastic paraplegia 11 (autosomal recessive) | 8.71E-08 | 9.21349 |
| 203597_s_at | WBP4 | WW domain binding protein 4 (formin binding protein 21) | 4.11E-07 | 9.21008 |
| 225041_at | MPHOSPH8 | M-phase phosphoprotein 8 | 9.39E-08 | 9.20629 |
| 208711_s_at | CCND1 | cyclin D1 | 5.44E-06 | 9.2026 |
| 225749_at | C16orf91 | chromosome 16 open reading frame 91 | 7.02E-09 | 9.20211 |
| 202194_at | TMED5 | transmembrane emp24 protein transport domain containing 5 | 2.70E-08 | 9.19346 |
| 212269_s_at | MCM3AP | minichromosome maintenance complex component 3 associated protein | 2.96E-07 | 9.18901 |
| 1567214_a_at | PNN | pinin, desmosome associated protein | 1.17E-05 | 9.17542 |
| 212461_at | AZIN1 | antizyme inhibitor 1 | 2.08E-08 | 9.17443 |
| 224825_at | DNTTIP1 | deoxynucleotidyltransferase, terminal, interacting protein 1 | 1.00E-06 | 9.16562 |
| 233558_s_at | C4orf41 | chromosome 4 open reading frame 41 | 5.70E-06 | 9.16062 |
| 227094_at | DHTKD1 | dehydrogenase E1 and transketolase domain containing 1 | 9.18E-07 | 9.16014 |
| 218009_s_at | PRC1 | protein regulator of cytokinesis 1 | 1.54E-07 | 9.15959 |
| 233849_s_at | ARHGAP5 | Rho GTPase activating protein 5 | 0.0001571 | 9.15773 |
| 227993_at | METAP2 | methionyl aminopeptidase 2 | 1.87E-06 | 9.15726 |
| 217831_s_at | NSFL1C | NSFL1 (p97) cofactor (p47) | 9.97E-07 | 9.15581 |
| 218618_s_at | FNDC3B | fibronectin type III domain containing 3B | 7.29E-09 | 9.15297 |
| 230204_at | HAPLN1 | hyaluronan and proteoglycan link protein 1 | 9.02E-07 | 9.14908 |
| 230083_at | USP53 | ubiquitin specific peptidase 53 | 8.73E-07 | 9.14777 |
| 225099_at | FBXO45 | F-box protein 45 | 4.48E-09 | 9.14341 |
| 244103_at | C1orf55 | chromosome 1 open reading frame 55 | 3.30E-08 | 9.14283 |
| 242794_at | MAML3 | mastermind-like 3 (Drosophila) | 1.72E-06 | 9.13872 |
| 221452_s_at | TMEM14B | transmembrane protein 14B | 7.40E-07 | 9.13799 |
| 212773_s_at | TOMM20 | translocase of outer mitochondrial membrane 20 homolog (yeast) | 6.15E-07 | 9.13686 |
| 218882_s_at | WDR3 | WD repeat domain 3 | 8.38E-09 | 9.1336 |
| 218238_at | GTPBP4 | GTP binding protein 4 | 2.54E-08 | 9.13035 |
| 223049_at | GRB2 | growth factor receptor-bound protein 2 | 4.66E-09 | 9.13019 |
| 209623_at | MCCC2 | methylcrotonoyl-CoA carboxylase 2 (beta) | 1.72E-08 | 9.12892 |
| 205523_at | HAPLN1 | hyaluronan and proteoglycan link protein 1 | 4.25E-07 | 9.12807 |
| 213318_s_at | BAT3 | HLA-B associated transcript 3 | 9.55E-06 | 9.12609 |
| 202184_s_at | NUP133 | nucleoporin 133kDa | 6.75E-07 | 9.11814 |
| 218460_at | HEATR2 | HEAT repeat containing 2 | 1.64E-09 | 9.11809 |
| 202060_at | CTR9 | Ctr9, Paf1/RNA polymerase II complex component, homolog (S. cerevisiae) | 1.26E-05 | 9.11533 |
| 206174_s_at | PPP6C | protein phosphatase 6, catalytic subunit | 6.83E-07 | 9.1052 |
| 211594_s_at | MRPL9 | mitochondrial ribosomal protein L9 | 1.28E-06 | 9.0953 |
| 223513_at | CENPJ | centromere protein J | 1.00E-07 | 9.09466 |
| 218443_s_at | DAZAP1 | DAZ associated protein 1 | 8.41E-08 | 9.08546 |
| 37577_at | ARHGAP19 | Rho GTPase activating protein 19 | 3.03E-08 | 9.07624 |
| 218728_s_at | CNIH4 | cornichon homolog 4 (Drosophila) | 2.04E-07 | 9.07296 |
| 206746_at | BFSP1 | beaded filament structural protein 1, filensin | 1.98E-08 | 9.07015 |
| 223271_s_at | CTDSPL2 | CTD (carboxy-terminal domain, RNA polymerase II, polypeptide A) small phosphatas | 3.35E-08 | 9.06939 |
| 235610_at | ALKBH8 | alkB, alkylation repair homolog 8 (E. coli) | 2.63E-05 | 9.06622 |
| 203367_at | DUSP14 | dual specificity phosphatase 14 | 1.22E-07 | 9.06596 |
| 201157_s_at | NMT1 | N-myristoyltransferase 1 | 6.09E-10 | 9.06443 |
| 202670_at | MAP2K1 | mitogen-activated protein kinase kinase 1 | 1.53E-07 | 9.05682 |
| 203022_at | RNASEH2A | ribonuclease H2, subunit A | 1.56E-08 | 9.05648 |
| 228408_s_at | SDAD1 | SDA1 domain containing 1 | 1.02E-09 | 9.05536 |
| 217973_at | DCXR | dicarbonyl/L-xylulose reductase | 6.64E-06 | 9.05509 |
| 233588_x_at | PFDN6 | prefoldin subunit 6 | 8.06E-06 | 9.05331 |
| 216237_s_at | MCM5 | minichromosome maintenance complex component 5 | 2.60E-09 | 9.05007 |
| 220083_x_at | UCHL5 | ubiquitin carboxyl-terminal hydrolase L5 | 1.60E-06 | 9.04443 |
| 219017_at | ETNK1 | ethanolamine kinase 1 | 1.82E-08 | 9.04228 |
| 214381_at | LOC441601 | septin 7 pseudogene | 6.47E-07 | 9.0394 |
| 222037_at | MCM4 | minichromosome maintenance complex component 4 | 1.49E-06 | 9.039 |
| 209216_at | WDR45 | WD repeat domain 45 | 9.48E-09 | 9.03789 |
| 202466_at | PAPD7 | PAP associated domain containing 7 | 3.43E-08 | 9.03725 |
| 218647_s_at | YRDC | yrdC domain containing (E. coli) | 9.86E-07 | 9.03449 |
| 210691_s_at | CACYBP | calcyclin binding protein | 1.91E-05 | 9.03052 |
| 203830_at | C17orf75 | chromosome 17 open reading frame 75 | 9.83E-06 | 9.02744 |
| 226293_at | MED19 | mediator complex subunit 19 | 1.96E-06 | 9.0232 |
| 207643_s_at | TNFRSF1A | tumor necrosis factor receptor superfamily, member 1A | 3.50E-06 | 9.02049 |
| 225194_at | PLRG1 | pleiotropic regulator 1 (PRL1 homolog, Arabidopsis) | 4.15E-07 | 9.01442 |
| 228050_at | UTP15 | UTP15, U3 small nucleolar ribonucleoprotein, homolog (S. cerevisiae) | 9.27E-06 | 9.01182 |
| 223294_at | CXorf26 | chromosome X open reading frame 26 | 3.98E-09 | 9.01018 |
| 91703_at | EHBP1L1 | EH domain binding protein 1-like 1 | 4.10E-07 | 9.008 |
| 223592_s_at | RNF135 | ring finger protein 135 | 1.78E-06 | 9.00663 |
| 209140_x_at | HLA-B | major histocompatibility complex, class I, B | 1.79E-08 | 9.0043 |
| 204709_s_at | KIF23 | kinesin family member 23 | 1.25E-06 | 9.00367 |
| 234464_s_at | EME1 | essential meiotic endonuclease 1 homolog 1 (S. pombe) | 4.26E-08 | 8.99673 |
| 213342_at | YAP1 | Yes-associated protein 1 | 1.35E-05 | 8.98465 |
| 219028_at | HIPK2 | homeodomain interacting protein kinase 2 | 4.34E-06 | 8.98399 |
| 213320_at | PRMT3 | protein arginine methyltransferase 3 | 3.82E-07 | 8.9806 |
| 201616_s_at | CALD1 | caldesmon 1 | 1.06E-06 | 8.98049 |
| 209507_at | RPA3 | replication protein A3, 14kDa | 1.57E-07 | 8.97816 |
| 201165_s_at | PUM1 | pumilio homolog 1 (Drosophila) | 3.05E-09 | 8.97723 |
| 208891_at | DUSP6 | dual specificity phosphatase 6 | 2.36E-05 | 8.96854 |
| 217784_at | YKT6 | YKT6 v-SNARE homolog (S. cerevisiae) | 3.68E-07 | 8.96281 |
| 224563_at | WASF2 | WAS protein family, member 2 | 3.40E-08 | 8.95822 |
| 201632_at | EIF2B1 | eukaryotic translation initiation factor 2B, subunit 1 alpha, 26kDa | 4.10E-08 | 8.95223 |
| 228183_s_at | RPAIN | RPA interacting protein | 2.85E-09 | 8.9478 |
| 226447_at | ASH1L | ash1 (absent, small, or homeotic)-like (Drosophila) | 6.62E-07 | 8.94647 |
| 238877_at | EYA4 | eyes absent homolog 4 (Drosophila) | 6.37E-08 | 8.94573 |
| 225481_at | FRMD6 | FERM domain containing 6 | 1.76E-06 | 8.93935 |
| 200753_x_at | SFRS2 | splicing factor, arginine/serine-rich 2 | 2.06E-06 | 8.93703 |
| 218317_x_at | GIYD1 /// GIYD2 | GIY-YIG domain containing 1 /// GIY-YIG domain containing 2 | 1.00E-06 | 8.93217 |
| 209581_at | PLA2G16 | phospholipase A2, group XVI | 3.78E-07 | 8.93202 |
| 1555874_x_at | MGC21881 | hypothetical locus MGC21881 | 4.22E-07 | 8.93128 |
| 202011_at | TJP1 | tight junction protein 1 (zona occludens 1) | 8.42E-08 | 8.92925 |
| 236641_at | KIF14 | kinesin family member 14 | 1.33E-06 | 8.92445 |
| 208927_at | SPOP | speckle-type POZ protein | 9.35E-08 | 8.92166 |
| 200634_at | PFN1 | profilin 1 | 1.50E-06 | 8.92036 |
| 202774_s_at | SFRS8 | splicing factor, arginine/serine-rich 8 (suppressor-of-white-apricot homolog, Dr | 3.18E-06 | 8.92019 |
| 214157_at | GNAS | GNAS complex locus | 1.02E-07 | 8.91769 |
| 214086_s_at | PARP2 | poly (ADP-ribose) polymerase 2 | 1.74E-06 | 8.91614 |
| 1552349_a_at | PRSS33 | protease, serine, 33 | 1.64E-06 | 8.91595 |
| 209728_at | HLA-DRB4 | major histocompatibility complex, class II, DR beta 4 | 1.62E-06 | 8.91578 |
| 209895_at | PTPN11 | protein tyrosine phosphatase, non-receptor type 11 | 1.14E-06 | 8.91426 |
| 202081_at | IER2 | immediate early response 2 | 3.85E-07 | 8.90679 |
| 223221_at | SCO1 | SCO cytochrome oxidase deficient homolog 1 (yeast) | 5.85E-07 | 8.9016 |
| 201408_at | PPP1CB | protein phosphatase 1, catalytic subunit, beta isozyme | 9.63E-06 | 8.90102 |
| 223335_at | TMEM69 | transmembrane protein 69 | 2.02E-06 | 8.90017 |
| 203374_s_at | TPP2 | tripeptidyl peptidase II | 1.22E-06 | 8.89725 |
| 212174_at | AK2 | adenylate kinase 2 | 1.05E-08 | 8.89709 |
| 228053_s_at | TOMM5 | translocase of outer mitochondrial membrane 5 homolog (yeast) | 1.32E-06 | 8.88949 |
| 225527_at | CEBPG | CCAAT/enhancer binding protein (C/EBP), gamma | 3.01E-06 | 8.88847 |
| 208932_at | PPP4C | protein phosphatase 4, catalytic subunit | 4.89E-07 | 8.87972 |
| 209020_at | C20orf111 | chromosome 20 open reading frame 111 | 2.74E-09 | 8.87743 |
| 209715_at | CBX5 | chromobox homolog 5 (HP1 alpha homolog, Drosophila) | 4.06E-06 | 8.8768 |
| 231807_at | KIAA1217 | KIAA1217 | 8.66E-08 | 8.87665 |
| 204646_at | DPYD | dihydropyrimidine dehydrogenase | 1.61E-08 | 8.87388 |
| 202006_at | PTPN12 | protein tyrosine phosphatase, non-receptor type 12 | 7.35E-07 | 8.87162 |
| 226837_at | SPRED1 | sprouty-related, EVH1 domain containing 1 | 2.05E-05 | 8.86859 |
| 201221_s_at | SNRNP70 | small nuclear ribonucleoprotein 70kDa (U1) | 2.82E-06 | 8.86743 |
| 208893_s_at | DUSP6 | dual specificity phosphatase 6 | 2.87E-06 | 8.86461 |
| 208264_s_at | EIF3J | eukaryotic translation initiation factor 3, subunit J | 1.90E-06 | 8.86105 |
| 1558930_at | LOC728192 | hypothetical protein LOC728192 | 2.04E-06 | 8.8553 |
| 229826_at | LOC440957 | similar to CG32736-PA | 4.28E-05 | 8.85427 |
| 208598_s_at | HUWE1 | HECT, UBA and WWE domain containing 1 | 2.58E-08 | 8.85422 |
| 222703_s_at | YRDC | yrdC domain containing (E. coli) | 2.81E-08 | 8.84995 |
| 226194_at | ZNF828 | zinc finger protein 828 | 1.86E-08 | 8.84936 |
| 203782_s_at | POLRMT | polymerase (RNA) mitochondrial (DNA directed) | 2.22E-06 | 8.84623 |
| 223407_at | C16orf48 | chromosome 16 open reading frame 48 | 4.97E-08 | 8.84436 |
| 216977_x_at | SNRPA1 | small nuclear ribonucleoprotein polypeptide A' | 3.65E-08 | 8.84349 |
| 223082_at | SH3KBP1 | SH3-domain kinase binding protein 1 | 7.15E-10 | 8.8416 |
| 203714_s_at | TBCE | tubulin folding cofactor E | 2.12E-08 | 8.83716 |
| 219031_s_at | NIP7 | nuclear import 7 homolog (S. cerevisiae) | 3.02E-06 | 8.83667 |
| 231817_at | USP53 | ubiquitin specific peptidase 53 | 9.48E-08 | 8.83429 |
| 200776_s_at | BZW1 /// BZW1L1 | basic leucine zipper and W2 domains 1 /// basic leucine zipper and W2 domains 1 | 1.05E-06 | 8.83363 |
| 231955_s_at | HIBADH | 3-hydroxyisobutyrate dehydrogenase | 4.31E-06 | 8.83282 |
| 201690_s_at | TPD52 | tumor protein D52 | 7.28E-07 | 8.83156 |
| 201310_s_at | C5orf13 | chromosome 5 open reading frame 13 | 1.20E-07 | 8.82677 |
| 201074_at | SMARCC1 | SWI/SNF related, matrix associated, actin dependent regulator of chromatin, subf | 1.99E-06 | 8.8247 |
| 223016_x_at | ZRANB2 | zinc finger, RAN-binding domain containing 2 | 4.06E-07 | 8.82352 |
| 1554510_s_at | GHITM | growth hormone inducible transmembrane protein | 2.11E-07 | 8.81682 |
| 224186_s_at | RNF123 | ring finger protein 123 | 2.78E-09 | 8.81651 |
| 218135_at | ERGIC2 | ERGIC and golgi 2 | 2.99E-09 | 8.8088 |
| 205603_s_at | DIAPH2 | diaphanous homolog 2 (Drosophila) | 8.71E-08 | 8.80514 |
| 203258_at | DRAP1 | DR1-associated protein 1 (negative cofactor 2 alpha) | 4.55E-07 | 8.80323 |
| 212789_at | NCAPD3 | non-SMC condensin II complex, subunit D3 | 5.67E-09 | 8.79555 |
| 218576_s_at | DUSP12 | dual specificity phosphatase 12 | 3.71E-07 | 8.79261 |
| 202760_s_at | AKAP2 /// PALM2-AKAP2 | A kinase (PRKA) anchor protein 2 /// PALM2-AKAP2 readthrough | 2.87E-05 | 8.7923 |
| 208691_at | TFRC | transferrin receptor (p90, CD71) | 2.01E-05 | 8.78987 |
| 204148_s_at | POMZP3 /// ZP3 | POM (POM121 homolog, rat) and ZP3 fusion /// zona pellucida glycoprotein 3 (sper | 2.13E-07 | 8.78466 |
| 212175_s_at | AK2 | adenylate kinase 2 | 6.87E-07 | 8.78406 |
| 222416_at | ALDH18A1 | aldehyde dehydrogenase 18 family, member A1 | 7.95E-07 | 8.77835 |
| 204872_at | TLE4 | transducin-like enhancer of split 4 (E(sp1) homolog, Drosophila) | 1.58E-08 | 8.77833 |
| 210053_at | TAF5 | TAF5 RNA polymerase II, TATA box binding protein (TBP)-associated factor, 100kDa | 1.63E-07 | 8.77191 |
| 203790_s_at | HRSP12 | heat-responsive protein 12 | 1.02E-07 | 8.76988 |
| 1555945_s_at | FAM120A | family with sequence similarity 120A | 1.20E-06 | 8.76633 |
| AFFX-HSAC07/X00351_5_at | ACTB | actin, beta | 0.0001717 | 8.76373 |
| 226350_at | CHML | choroideremia-like (Rab escort protein 2) | 2.33E-07 | 8.76205 |
| 209725_at | UTP20 | UTP20, small subunit (SSU) processome component, homolog (yeast) | 2.76E-08 | 8.76189 |
| 218653_at | SLC25A15 | solute carrier family 25 (mitochondrial carrier; ornithine transporter) member 1 | 2.47E-08 | 8.75951 |
| 225376_at | C20orf11 | chromosome 20 open reading frame 11 | 9.98E-09 | 8.75758 |
| 203967_at | CDC6 | cell division cycle 6 homolog (S. cerevisiae) | 1.66E-07 | 8.75314 |
| 228619_x_at | TIPRL | TIP41, TOR signaling pathway regulator-like (S. cerevisiae) | 3.02E-08 | 8.74175 |
| 206562_s_at | CSNK1A1 | casein kinase 1, alpha 1 | 6.72E-08 | 8.73846 |
| 213239_at | PIBF1 | progesterone immunomodulatory binding factor 1 | 6.55E-07 | 8.73775 |
| 208907_s_at | MRPS18B | mitochondrial ribosomal protein S18B | 1.20E-08 | 8.73484 |
| 225291_at | PNPT1 | polyribonucleotide nucleotidyltransferase 1 | 1.74E-06 | 8.73025 |
| 203526_s_at | APC | adenomatous polyposis coli | 1.55E-05 | 8.7114 |
| 218622_at | NUP37 | nucleoporin 37kDa | 4.50E-09 | 8.7114 |
| 223397_s_at | NIP7 | nuclear import 7 homolog (S. cerevisiae) | 2.51E-07 | 8.70844 |
| 218543_s_at | PARP12 | poly (ADP-ribose) polymerase family, member 12 | 1.69E-05 | 8.70814 |
| 225802_at | TOP1MT | topoisomerase (DNA) I, mitochondrial | 1.71E-05 | 8.70752 |
| 208774_at | CSNK1D | casein kinase 1, delta | 2.76E-06 | 8.70496 |
| 201967_at | RBM6 | RNA binding motif protein 6 | 2.68E-07 | 8.70481 |
| 219276_x_at | C9orf82 | chromosome 9 open reading frame 82 | 1.19E-05 | 8.70453 |
| 225149_at | PCID2 | PCI domain containing 2 | 2.53E-07 | 8.70423 |
| 221539_at | EIF4EBP1 | eukaryotic translation initiation factor 4E binding protein 1 | 1.06E-05 | 8.70292 |
| 209088_s_at | UBN1 | ubinuclein 1 | 1.19E-06 | 8.69961 |
| 205053_at | PRIM1 | primase, DNA, polypeptide 1 (49kDa) | 5.63E-10 | 8.68867 |
| 220261_s_at | ZDHHC4 | zinc finger, DHHC-type containing 4 | 2.28E-07 | 8.68564 |
| 225456_at | MED1 | mediator complex subunit 1 | 2.89E-08 | 8.68401 |
| 205512_s_at | AIFM1 | apoptosis-inducing factor, mitochondrion-associated, 1 | 3.13E-10 | 8.67967 |
| 205194_at | PSPH | phosphoserine phosphatase | 1.19E-06 | 8.67893 |
| 203497_at | MED1 | mediator complex subunit 1 | 1.66E-07 | 8.67005 |
| 201368_at | ZFP36L2 | zinc finger protein 36, C3H type-like 2 | 4.78E-08 | 8.6623 |
| 214662_at | WDR43 | WD repeat domain 43 | 2.42E-07 | 8.66227 |
| 200737_at | PGK1 | phosphoglycerate kinase 1 | 7.73E-08 | 8.64982 |
| 223716_s_at | ZRANB2 | zinc finger, RAN-binding domain containing 2 | 3.16E-06 | 8.64879 |
| 200593_s_at | HNRNPU | heterogeneous nuclear ribonucleoprotein U (scaffold attachment factor A) | 2.94E-06 | 8.64679 |
| 231530_s_at | C11orf1 | chromosome 11 open reading frame 1 | 7.21E-07 | 8.64493 |
| 210732_s_at | LGALS8 | lectin, galactoside-binding, soluble, 8 | 8.38E-07 | 8.63702 |
| 201523_x_at | UBE2N | ubiquitin-conjugating enzyme E2N (UBC13 homolog, yeast) | 7.28E-06 | 8.63687 |
| 222163_s_at | SPATA5L1 | spermatogenesis associated 5-like 1 | 5.44E-06 | 8.63476 |
| 229947_at | PI15 | peptidase inhibitor 15 | 5.62E-06 | 8.62333 |
| 217356_s_at | PGK1 | phosphoglycerate kinase 1 | 3.08E-07 | 8.6169 |
| 209463_s_at | TAF12 | TAF12 RNA polymerase II, TATA box binding protein (TBP)-associated factor, 20kDa | 3.29E-07 | 8.61628 |
| 221752_at | SSH1 | slingshot homolog 1 (Drosophila) | 2.87E-09 | 8.61271 |
| 221743_at | CELF1 | CUGBP, Elav-like family member 1 | 5.67E-06 | 8.60902 |
| 217898_at | C15orf24 | chromosome 15 open reading frame 24 | 3.84E-06 | 8.60888 |
| 220129_at | SOHLH2 | spermatogenesis and oogenesis specific basic helix-loop-helix 2 | 2.47E-07 | 8.60766 |
| 212420_at | ELF1 | E74-like factor 1 (ets domain transcription factor) | 5.00E-06 | 8.60715 |
| 218187_s_at | C8orf33 | chromosome 8 open reading frame 33 | 2.13E-07 | 8.60667 |
| 203737_s_at | PPRC1 | peroxisome proliferator-activated receptor gamma, coactivator-related 1 | 2.63E-06 | 8.60457 |
| 226017_at | CMTM7 | CKLF-like MARVEL transmembrane domain containing 7 | 1.81E-07 | 8.60386 |
| 213329_at | SRGAP2 | SLIT-ROBO Rho GTPase activating protein 2 | 9.06E-07 | 8.60363 |
| 209751_s_at | TRAPPC2 /// TRAPPC2P1 | trafficking protein particle complex 2 /// trafficking protein particle complex | 6.61E-07 | 8.59976 |
| 210266_s_at | TRIM33 | tripartite motif-containing 33 | 1.13E-07 | 8.59276 |
| 202548_s_at | ARHGEF7 | Rho guanine nucleotide exchange factor (GEF) 7 | 1.71E-07 | 8.58991 |
| 203712_at | KIAA0020 | KIAA0020 | 2.50E-06 | 8.58708 |
| 202261_at | VPS72 | vacuolar protein sorting 72 homolog (S. cerevisiae) | 5.07E-09 | 8.58677 |
| 225887_at | C13orf23 | chromosome 13 open reading frame 23 | 5.06E-06 | 8.58515 |
| 1553107_s_at | C5orf24 | chromosome 5 open reading frame 24 | 0.0001108 | 8.58431 |
| 218395_at | ACTR6 | ARP6 actin-related protein 6 homolog (yeast) | 6.31E-07 | 8.58417 |
| 217299_s_at | NBN | nibrin | 0.0002168 | 8.58348 |
| 214659_x_at | YLPM1 | YLP motif containing 1 | 1.92E-06 | 8.58269 |
| 203848_at | AKAP8 | A kinase (PRKA) anchor protein 8 | 5.86E-09 | 8.58018 |
| 218461_at | GPN3 | GPN-loop GTPase 3 | 4.67E-08 | 8.57528 |
| 222982_x_at | SLC38A2 | solute carrier family 38, member 2 | 6.71E-08 | 8.57382 |
| 208812_x_at | HLA-C | major histocompatibility complex, class I, C | 2.80E-08 | 8.57054 |
| 213021_at | GOSR1 | golgi SNAP receptor complex member 1 | 2.96E-06 | 8.57043 |
| 202776_at | DNTTIP2 | deoxynucleotidyltransferase, terminal, interacting protein 2 | 2.31E-07 | 8.56478 |
| 201488_x_at | KHDRBS1 | KH domain containing, RNA binding, signal transduction associated 1 | 1.21E-07 | 8.55953 |
| 217893_s_at | AKIRIN1 | akirin 1 | 1.35E-08 | 8.55917 |
| 210042_s_at | CTSZ | cathepsin Z | 6.07E-07 | 8.55787 |
| 202819_s_at | TCEB3 | transcription elongation factor B (SIII), polypeptide 3 (110kDa, elongin A) | 4.05E-06 | 8.54919 |
| 221797_at | C17orf90 | chromosome 17 open reading frame 90 | 1.17E-06 | 8.54697 |
| 228026_at | SIKE1 | suppressor of IKBKE 1 | 2.04E-06 | 8.54675 |
| 214943_s_at | RBM34 | RNA binding motif protein 34 | 8.42E-07 | 8.54292 |
| 224614_at | DYNC1LI2 | dynein, cytoplasmic 1, light intermediate chain 2 | 3.47E-06 | 8.53978 |
| 201378_s_at | UBAP2L | ubiquitin associated protein 2-like | 3.22E-05 | 8.53822 |
| 205726_at | DIAPH2 | diaphanous homolog 2 (Drosophila) | 1.85E-06 | 8.53671 |
| 202395_at | NSF | N-ethylmaleimide-sensitive factor | 1.82E-05 | 8.53441 |
| 208773_s_at | ANKHD1 /// ANKHD1-EIF4EBP3 | ankyrin repeat and KH domain containing 1 /// ANKHD1-EIF4EBP3 readthrough | 1.33E-06 | 8.53076 |
| 200604_s_at | PRKAR1A | protein kinase, cAMP-dependent, regulatory, type I, alpha (tissue specific extin | 2.87E-07 | 8.52948 |
| 207663_x_at | GAGE3 | G antigen 3 | 9.06E-08 | 8.51707 |
| 1553972_a_at | CBS | cystathionine-beta-synthase | 6.35E-06 | 8.51628 |
| 211858_x_at | GNAS | GNAS complex locus | 1.22E-10 | 8.51516 |
| 224578_at | RCC2 | regulator of chromosome condensation 2 | 1.54E-08 | 8.5123 |
| 203775_at | SLC25A13 | solute carrier family 25, member 13 (citrin) | 1.20E-05 | 8.51145 |
| 217933_s_at | LAP3 | leucine aminopeptidase 3 | 3.48E-05 | 8.51127 |
| 233803_s_at | MYBBP1A | MYB binding protein (P160) 1a | 9.94E-07 | 8.51061 |
| 214670_at | ZKSCAN1 | zinc finger with KRAB and SCAN domains 1 | 7.34E-07 | 8.50935 |
| 217866_at | CPSF7 | cleavage and polyadenylation specific factor 7, 59kDa | 6.52E-07 | 8.50786 |
| 200883_at | UQCRC2 | ubiquinol-cytochrome c reductase core protein II | 1.10E-06 | 8.49643 |
| 201786_s_at | ADAR | adenosine deaminase, RNA-specific | 3.39E-08 | 8.49372 |
| 225367_at | PGM2 | phosphoglucomutase 2 | 1.66E-06 | 8.48686 |
| 238538_at | ANKRD11 | ankyrin repeat domain 11 | 2.31E-06 | 8.48605 |
| 229050_s_at | SNHG7 | small nucleolar RNA host gene 7 (non-protein coding) | 6.05E-07 | 8.48537 |
| 225364_at | STK4 | serine/threonine kinase 4 | 3.65E-05 | 8.48427 |
| 32091_at | SLC25A44 | solute carrier family 25, member 44 | 2.03E-06 | 8.48389 |
| 210517_s_at | AKAP12 | A kinase (PRKA) anchor protein 12 | 1.90E-08 | 8.48266 |
| 218478_s_at | ZCCHC8 | zinc finger, CCHC domain containing 8 | 1.90E-05 | 8.48265 |
| 201200_at | CREG1 | cellular repressor of E1A-stimulated genes 1 | 2.33E-05 | 8.48213 |
| 219148_at | PBK | PDZ binding kinase | 3.41E-07 | 8.47586 |
| 204593_s_at | SMCR7L | Smith-Magenis syndrome chromosome region, candidate 7-like | 3.01E-07 | 8.4747 |
| 228386_s_at | DDX59 | DEAD (Asp-Glu-Ala-Asp) box polypeptide 59 | 3.38E-07 | 8.47242 |
| 202133_at | WWTR1 | WW domain containing transcription regulator 1 | 1.76E-06 | 8.46892 |
| 211953_s_at | IPO5 | importin 5 | 5.72E-06 | 8.46459 |
| 218932_at | ZNHIT6 | zinc finger, HIT type 6 | 1.96E-08 | 8.46445 |
| 212815_at | ASCC3 | activating signal cointegrator 1 complex subunit 3 | 8.34E-06 | 8.46129 |
| 225014_at | C4orf52 | chromosome 4 open reading frame 52 | 7.80E-10 | 8.45654 |
| 212124_at | ZMIZ1 | zinc finger, MIZ-type containing 1 | 3.30E-08 | 8.45553 |
| 201057_s_at | GOLGB1 | golgin B1 | 2.36E-07 | 8.45542 |
| 227942_s_at | CRIPT | cysteine-rich PDZ-binding protein | 3.39E-05 | 8.45446 |
| 235521_at | HOXA3 | homeobox A3 | 4.29E-07 | 8.45384 |
| 236620_at | RIF1 | RAP1 interacting factor homolog (yeast) | 4.21E-06 | 8.45172 |
| 204956_at | MTAP | methylthioadenosine phosphorylase | 2.39E-05 | 8.4496 |
| 209288_s_at | CDC42EP3 | CDC42 effector protein (Rho GTPase binding) 3 | 8.21E-07 | 8.44874 |
| 235677_at | SRR | Serine racemase | 3.83E-09 | 8.4463 |
| 202844_s_at | RALBP1 | ralA binding protein 1 | 4.85E-06 | 8.44574 |
| 221688_s_at | IMP3 | IMP3, U3 small nucleolar ribonucleoprotein, homolog (yeast) | 1.79E-10 | 8.44182 |
| 228737_at | TOX2 | TOX high mobility group box family member 2 | 2.42E-06 | 8.42924 |
| 228272_at | DNLZ | DNL-type zinc finger | 1.33E-06 | 8.4237 |
| 212671_s_at | HLA-DQA1 /// HLA-DQA2 /// LOC100294224 /// LOC100294317 | major histocompatibility complex, class II, DQ alpha 1 /// major histocompatibil | 1.62E-06 | 8.42354 |
| 224982_at | AKT1S1 | AKT1 substrate 1 (proline-rich) | 3.76E-07 | 8.42102 |
| 203762_s_at | DYNC2LI1 | dynein, cytoplasmic 2, light intermediate chain 1 | 7.32E-07 | 8.42094 |
| 202645_s_at | MEN1 | multiple endocrine neoplasia I | 2.31E-07 | 8.41978 |
| 219913_s_at | CRNKL1 | crooked neck pre-mRNA splicing factor-like 1 (Drosophila) | 1.83E-06 | 8.4124 |
| 214356_s_at | KIAA0368 | KIAA0368 | 8.32E-06 | 8.41199 |
| 201731_s_at | TPR | translocated promoter region (to activated MET oncogene) | 4.23E-09 | 8.4104 |
| 218057_x_at | COX4NB | COX4 neighbor | 2.76E-06 | 8.40971 |
| 202109_at | ARFIP2 | ADP-ribosylation factor interacting protein 2 | 1.04E-09 | 8.40845 |
| 202715_at | CAD | carbamoyl-phosphate synthetase 2, aspartate transcarbamylase, and dihydroorotase | 7.68E-08 | 8.39797 |
| 225006_x_at | TH1L | TH1-like (Drosophila) | 1.83E-06 | 8.39464 |
| 222427_s_at | LARS | leucyl-tRNA synthetase | 1.12E-06 | 8.3883 |
| 212926_at | SMC5 | structural maintenance of chromosomes 5 | 1.58E-06 | 8.38778 |
| 212141_at | MCM4 | minichromosome maintenance complex component 4 | 3.58E-06 | 8.37533 |
| 200802_at | SARS | seryl-tRNA synthetase | 2.37E-06 | 8.37068 |
| 217950_at | NOSIP | nitric oxide synthase interacting protein | 9.17E-08 | 8.36454 |
| 218237_s_at | SLC38A1 | solute carrier family 38, member 1 | 6.00E-08 | 8.36117 |
| 241803_s_at | LOC401522 | hypothetical LOC401522 | 1.01E-06 | 8.35733 |
| 229742_at | C15orf61 | chromosome 15 open reading frame 61 | 1.90E-06 | 8.35522 |
| 235205_at | LOC346887 | similar to solute carrier family 16 (monocarboxylic acid transporters), member 1 | 2.56E-07 | 8.3477 |
| 222609_s_at | EXOSC1 | exosome component 1 | 1.12E-06 | 8.34605 |
| 213008_at | FANCI | Fanconi anemia, complementation group I | 7.42E-06 | 8.34605 |
| 215111_s_at | TSC22D1 | TSC22 domain family, member 1 | 5.02E-07 | 8.34516 |
| 208804_s_at | SFRS6 | splicing factor, arginine/serine-rich 6 | 1.33E-12 | 8.34372 |
| 201558_at | RAE1 | RAE1 RNA export 1 homolog (S. pombe) | 9.01E-08 | 8.34363 |
| 210445_at | FABP6 | fatty acid binding protein 6, ileal | 2.29E-07 | 8.33391 |
| 211038_s_at | CROCCL1 | ciliary rootlet coiled-coil, rootletin-like 1 | 1.13E-06 | 8.33378 |
| 201624_at | DARS | aspartyl-tRNA synthetase | 2.04E-06 | 8.32862 |
| 223139_s_at | DHX36 | DEAH (Asp-Glu-Ala-His) box polypeptide 36 | 5.87E-07 | 8.32795 |
| 223109_at | TRUB2 | TruB pseudouridine (psi) synthase homolog 2 (E. coli) | 9.49E-09 | 8.32681 |
| 228351_at | HEATR1 | HEAT repeat containing 1 | 6.55E-08 | 8.32081 |
| 201371_s_at | CUL3 | cullin 3 | 7.91E-08 | 8.32028 |
| 220643_s_at | FAIM | Fas apoptotic inhibitory molecule | 7.05E-06 | 8.31559 |
| 1559827_at | LOC401074 | hypothetical LOC401074 | 1.51E-07 | 8.31224 |
| 204531_s_at | BRCA1 | breast cancer 1, early onset | 7.52E-08 | 8.31208 |
| 208693_s_at | GARS | glycyl-tRNA synthetase | 3.06E-09 | 8.30911 |
| 238005_s_at | SIN3A | SIN3 homolog A, transcription regulator (yeast) | 1.31E-07 | 8.30172 |
| 227530_at | AKAP12 | A kinase (PRKA) anchor protein 12 | 2.58E-07 | 8.30064 |
| 223101_s_at | ARPC5L | actin related protein 2/3 complex, subunit 5-like | 1.27E-06 | 8.29519 |
| 210415_s_at | ODF2 | outer dense fiber of sperm tails 2 | 1.38E-07 | 8.29438 |
| 221776_s_at | BRD7 | bromodomain containing 7 | 1.67E-06 | 8.29416 |
| 204710_s_at | WIPI2 | WD repeat domain, phosphoinositide interacting 2 | 3.48E-09 | 8.28935 |
| 222127_s_at | EXOC1 | exocyst complex component 1 | 1.06E-07 | 8.28757 |
| 209444_at | RAP1GDS1 | RAP1, GTP-GDP dissociation stimulator 1 | 1.07E-09 | 8.28671 |
| 226661_at | CDCA2 | cell division cycle associated 2 | 5.97E-05 | 8.2792 |
| 202891_at | NIT1 | nitrilase 1 | 2.04E-07 | 8.27578 |
| 223018_at | NOB1 | NIN1/RPN12 binding protein 1 homolog (S. cerevisiae) | 6.94E-07 | 8.27547 |
| 202290_at | PDAP1 | PDGFA associated protein 1 | 1.17E-07 | 8.27358 |
| 224913_s_at | TIMM50 | translocase of inner mitochondrial membrane 50 homolog (S. cerevisiae) | 1.69E-05 | 8.27025 |
| 204836_at | GLDC | glycine dehydrogenase (decarboxylating) | 2.04E-06 | 8.26993 |
| 206445_s_at | PRMT1 | protein arginine methyltransferase 1 | 8.67E-06 | 8.26961 |
| 211955_at | IPO5 | importin 5 | 2.05E-09 | 8.26871 |
| 218306_s_at | HERC1 | hect (homologous to the E6-AP (UBE3A) carboxyl terminus) domain and RCC1 (CHC1)- | 7.92E-07 | 8.26794 |
| 202069_s_at | IDH3A | isocitrate dehydrogenase 3 (NAD+) alpha | 4.16E-07 | 8.26565 |
| 203263_s_at | ARHGEF9 | Cdc42 guanine nucleotide exchange factor (GEF) 9 | 1.37E-06 | 8.26318 |
| 214175_x_at | PDLIM4 | PDZ and LIM domain 4 | 7.08E-07 | 8.26014 |
| 201103_x_at | LOC200030 /// NBPF10 /// NBPF11 /// NBPF15 /// NBPF16 /// NBPF8 /// NBPF9 | neuroblastoma breakpoint family, member 11-like /// neuroblastoma breakpoint fam | 2.03E-09 | 8.25984 |
| 203978_at | NUBP1 | nucleotide binding protein 1 (MinD homolog, E. coli) | 9.13E-12 | 8.25981 |
| 214141_x_at | SFRS7 | splicing factor, arginine/serine-rich 7, 35kDa | 5.88E-09 | 8.25894 |
| 211864_s_at | MYOF | myoferlin | 1.94E-06 | 8.25793 |
| 201369_s_at | ZFP36L2 | zinc finger protein 36, C3H type-like 2 | 4.90E-06 | 8.25227 |
| 201487_at | CTSC | cathepsin C | 1.32E-08 | 8.24645 |
| 203428_s_at | ASF1A | ASF1 anti-silencing function 1 homolog A (S. cerevisiae) | 1.54E-05 | 8.24319 |
| 223234_at | MAD2L2 | MAD2 mitotic arrest deficient-like 2 (yeast) | 3.65E-06 | 8.23154 |
| 205425_at | HIP1 | huntingtin interacting protein 1 | 7.41E-08 | 8.23011 |
| 223163_s_at | ZC3HC1 | zinc finger, C3HC-type containing 1 | 1.05E-09 | 8.22627 |
| 200999_s_at | CKAP4 | cytoskeleton-associated protein 4 | 2.08E-05 | 8.22546 |
| 206074_s_at | HMGA1 | high mobility group AT-hook 1 | 8.10E-07 | 8.22365 |
| 207493_x_at | SSX2 /// SSX2B | synovial sarcoma, X breakpoint 2 /// synovial sarcoma, X breakpoint 2B | 6.44E-07 | 8.22158 |
| 221654_s_at | USP3 | ubiquitin specific peptidase 3 | 4.91E-08 | 8.22072 |
| 222441_x_at | SLMO2 | slowmo homolog 2 (Drosophila) | 6.54E-07 | 8.21966 |
| 235692_at | SH3KBP1 | SH3-domain kinase binding protein 1 | 6.03E-08 | 8.21571 |
| 209040_s_at | PSMB8 | proteasome (prosome, macropain) subunit, beta type, 8 (large multifunctional pep | 1.58E-08 | 8.21485 |
| 231784_s_at | DCAF13 | DDB1 and CUL4 associated factor 13 | 3.77E-06 | 8.21307 |
| 212871_at | MAPKAPK5 | mitogen-activated protein kinase-activated protein kinase 5 | 5.78E-06 | 8.20808 |
| 203417_at | MFAP2 | microfibrillar-associated protein 2 | 6.02E-06 | 8.20782 |
| 204847_at | ZBTB11 | zinc finger and BTB domain containing 11 | 3.34E-06 | 8.20781 |
| 212878_s_at | KLC1 | kinesin light chain 1 | 3.64E-07 | 8.2049 |
| 210026_s_at | CARD10 | caspase recruitment domain family, member 10 | 4.57E-07 | 8.20465 |
| 204146_at | RAD51AP1 | RAD51 associated protein 1 | 3.63E-06 | 8.20072 |
| 214016_s_at | SFPQ | splicing factor proline/glutamine-rich | 5.81E-08 | 8.19955 |
| 228135_at | C1orf52 | chromosome 1 open reading frame 52 | 1.87E-07 | 8.19833 |
| 231728_at | CAPS | calcyphosine | 1.02E-06 | 8.19693 |
| 209247_s_at | ABCF2 | ATP-binding cassette, sub-family F (GCN20), member 2 | 1.29E-07 | 8.19625 |
| 201245_s_at | OTUB1 | OTU domain, ubiquitin aldehyde binding 1 | 1.06E-08 | 8.1944 |
| 217861_s_at | PREB | prolactin regulatory element binding | 8.27E-07 | 8.19231 |
| 223591_at | RNF135 | ring finger protein 135 | 6.64E-07 | 8.18779 |
| 225158_at | GFM1 | G elongation factor, mitochondrial 1 | 8.52E-06 | 8.18403 |
| 225580_at | MRPL50 | mitochondrial ribosomal protein L50 | 1.50E-05 | 8.17495 |
| 226452_at | PDK1 | pyruvate dehydrogenase kinase, isozyme 1 | 7.30E-08 | 8.17019 |
| 202537_s_at | CHMP2B | chromatin modifying protein 2B | 1.04E-06 | 8.16844 |
| 203138_at | HAT1 | histone acetyltransferase 1 | 2.80E-07 | 8.16841 |
| 213360_s_at | POM121 /// POM121C | POM121 membrane glycoprotein (rat) /// POM121 membrane glycoprotein C | 3.05E-07 | 8.16612 |
| 204366_s_at | GTF3C2 | general transcription factor IIIC, polypeptide 2, beta 110kDa | 5.58E-10 | 8.16414 |
| 208453_s_at | XPNPEP1 | X-prolyl aminopeptidase (aminopeptidase P) 1, soluble | 5.53E-06 | 8.15229 |
| 209445_x_at | C7orf44 | chromosome 7 open reading frame 44 | 1.27E-08 | 8.15142 |
| 208884_s_at | UBR5 | ubiquitin protein ligase E3 component n-recognin 5 | 5.47E-07 | 8.14677 |
| 225933_at | CCDC137 | coiled-coil domain containing 137 | 6.15E-07 | 8.14625 |
| 213746_s_at | FLNA | filamin A, alpha | 3.69E-06 | 8.14448 |
| 218593_at | RBM28 | RNA binding motif protein 28 | 1.78E-08 | 8.14182 |
| 217140_s_at | VDAC1 | voltage-dependent anion channel 1 | 4.54E-07 | 8.14137 |
| 222466_s_at | MRPL42 | mitochondrial ribosomal protein L42 | 2.63E-07 | 8.1398 |
| 213511_s_at | MTMR1 | myotubularin related protein 1 | 1.71E-05 | 8.13031 |
| 229447_x_at | LOC200030 /// NBPF11 /// NBPF8 | neuroblastoma breakpoint family, member 11-like /// neuroblastoma breakpoint fam | 1.56E-06 | 8.13029 |
| 222474_s_at | TOMM22 | translocase of outer mitochondrial membrane 22 homolog (yeast) | 1.11E-07 | 8.12996 |
| 213301_x_at | TRIM24 | tripartite motif-containing 24 | 8.59E-06 | 8.12981 |
| 212199_at | MRFAP1L1 | Morf4 family associated protein 1-like 1 | 4.52E-07 | 8.12921 |
| 200745_s_at | GNB1 | guanine nucleotide binding protein (G protein), beta polypeptide 1 | 1.70E-07 | 8.12798 |
| 1558254_s_at | SRPK2 | SFRS protein kinase 2 | 1.14E-05 | 8.12168 |
| 202072_at | HNRNPL | heterogeneous nuclear ribonucleoprotein L | 1.05E-07 | 8.11802 |
| 208880_s_at | PRPF6 | PRP6 pre-mRNA processing factor 6 homolog (S. cerevisiae) | 8.46E-08 | 8.10234 |
| 226181_at | TUBE1 | tubulin, epsilon 1 | 0.0003341 | 8.09448 |
| 213447_at | IPW | imprinted in Prader-Willi syndrome (non-protein coding) | 3.26E-07 | 8.09383 |
| 210104_at | MED6 | mediator complex subunit 6 | 5.90E-07 | 8.09224 |
| 207983_s_at | STAG2 | stromal antigen 2 | 4.55E-07 | 8.09151 |
| 212512_s_at | CARM1 | coactivator-associated arginine methyltransferase 1 | 5.92E-07 | 8.08681 |
| 212584_at | AQR | aquarius homolog (mouse) | 3.40E-08 | 8.08389 |
| 219083_at | SHQ1 | SHQ1 homolog (S. cerevisiae) | 4.85E-07 | 8.07931 |
| 224821_at | ABHD14B | abhydrolase domain containing 14B | 3.04E-08 | 8.07855 |
| 228980_at | RFFL | ring finger and FYVE-like domain containing 1 | 2.00E-06 | 8.07853 |
| 212612_at | RCOR1 | REST corepressor 1 | 8.95E-07 | 8.06549 |
| 213313_at | RABGAP1 | RAB GTPase activating protein 1 | 3.06E-06 | 8.06234 |
| 212625_at | STX10 | syntaxin 10 | 4.08E-08 | 8.05927 |
| 208634_s_at | MACF1 | microtubule-actin crosslinking factor 1 | 4.40E-09 | 8.05344 |
| 225133_at | KLF3 | Kruppel-like factor 3 (basic) | 2.31E-05 | 8.05044 |
| 208152_s_at | DDX21 | DEAD (Asp-Glu-Ala-Asp) box polypeptide 21 | 9.14E-09 | 8.04798 |
| 200619_at | SF3B2 | splicing factor 3b, subunit 2, 145kDa | 8.99E-09 | 8.04531 |
| 218982_s_at | MRPS17 /// ZNF713 | mitochondrial ribosomal protein S17 /// zinc finger protein 713 | 5.55E-08 | 8.04522 |
| 225086_at | FAM98B | Family with sequence similarity 98, member B | 1.13E-07 | 8.04459 |
| 202779_s_at | UBE2S | ubiquitin-conjugating enzyme E2S | 3.46E-05 | 8.04459 |
| 213175_s_at | SNRPB | small nuclear ribonucleoprotein polypeptides B and B1 | 1.33E-09 | 8.04053 |
| 244519_at | ASXL1 | additional sex combs like 1 (Drosophila) | 2.85E-06 | 8.03747 |
| 203975_s_at | CHAF1A | chromatin assembly factor 1, subunit A (p150) | 1.63E-08 | 8.03071 |
| 219940_s_at | PCID2 | PCI domain containing 2 | 1.93E-07 | 8.02762 |
| 201913_s_at | COASY | CoA synthase | 8.72E-08 | 8.02567 |
| 212510_at | GPD1L | glycerol-3-phosphate dehydrogenase 1-like | 1.68E-06 | 8.01445 |
| 204744_s_at | IARS | isoleucyl-tRNA synthetase | 1.77E-10 | 8.01275 |
| 228752_at | EFCAB4B | EF-hand calcium binding domain 4B | 2.02E-06 | 8.01166 |
| 224766_at | RPL37 | Ribosomal protein L37 | 3.23E-07 | 8.0093 |
| 225313_at | C20orf177 | chromosome 20 open reading frame 177 | 4.99E-06 | 8.00589 |
| 219787_s_at | ECT2 | epithelial cell transforming sequence 2 oncogene | 2.42E-05 | 8.00588 |
| 201223_s_at | RAD23B | RAD23 homolog B (S. cerevisiae) | 2.70E-05 | 8.00565 |
| 236152_at | PAGE5 | P antigen family, member 5 (prostate associated) | 1.75E-06 | 8.00261 |
| 208706_s_at | EIF5 | eukaryotic translation initiation factor 5 | 5.14E-08 | 8.0023 |
| 223200_s_at | LSG1 | large subunit GTPase 1 homolog (S. cerevisiae) | 3.02E-08 | 8.00145 |
| 221637_s_at | C11orf48 | chromosome 11 open reading frame 48 | 1.81E-07 | 8.00071 |
| 209853_s_at | PSME3 | proteasome (prosome, macropain) activator subunit 3 (PA28 gamma; Ki) | 1.13E-06 | 7.99987 |
| 225865_x_at | TH1L | TH1-like (Drosophila) | 2.12E-08 | 7.99902 |
| 215905_s_at | SNRNP40 | small nuclear ribonucleoprotein 40kDa (U5) | 6.15E-06 | 7.99189 |
| 225657_at | LOC152217 | hypothetical LOC152217 | 6.98E-07 | 7.99084 |
| 210802_s_at | DIMT1L | DIM1 dimethyladenosine transferase 1-like (S. cerevisiae) | 2.53E-07 | 7.98947 |
| 223286_at | C17orf81 | chromosome 17 open reading frame 81 | 1.38E-07 | 7.98808 |
| 208445_s_at | BAZ1B | bromodomain adjacent to zinc finger domain, 1B | 5.96E-07 | 7.98789 |
| 227068_at | PGK1 | phosphoglycerate kinase 1 | 7.48E-09 | 7.98488 |
| 200051_at | SART1 | squamous cell carcinoma antigen recognized by T cells | 1.15E-06 | 7.97912 |
| 226003_at | KIF21A | kinesin family member 21A | 2.39E-06 | 7.97313 |
| 219922_s_at | LTBP3 | latent transforming growth factor beta binding protein 3 | 1.22E-07 | 7.97114 |
| 203582_s_at | RAB4A | RAB4A, member RAS oncogene family | 4.79E-07 | 7.97114 |
| 221735_at | WDR48 | WD repeat domain 48 | 8.89E-06 | 7.96717 |
| 223469_at | PGPEP1 | pyroglutamyl-peptidase I | 4.41E-06 | 7.96327 |
| 226626_at | THOC2 | THO complex 2 | 1.70E-12 | 7.95983 |
| 202975_s_at | RHOBTB3 | Rho-related BTB domain containing 3 | 6.01E-07 | 7.95957 |
| 225892_at | IREB2 | iron-responsive element binding protein 2 | 5.39E-06 | 7.95956 |
| 200927_s_at | RAB14 | RAB14, member RAS oncogene family | 1.12E-06 | 7.95645 |
| 211370_s_at | MAP2K5 | mitogen-activated protein kinase kinase 5 | 9.58E-07 | 7.95584 |
| 221503_s_at | KPNA3 | karyopherin alpha 3 (importin alpha 4) | 8.08E-08 | 7.94526 |
| 202686_s_at | AXL | AXL receptor tyrosine kinase | 1.04E-06 | 7.94433 |
| 206613_s_at | TAF1A | TATA box binding protein (TBP)-associated factor, RNA polymerase I, A, 48kDa | 4.43E-07 | 7.94273 |
| 203415_at | PDCD6 | programmed cell death 6 | 1.07E-06 | 7.93429 |
| 225625_at | ALKBH2 | alkB, alkylation repair homolog 2 (E. coli) | 2.12E-06 | 7.93394 |
| 32402_s_at | SYMPK | symplekin | 1.05E-06 | 7.93366 |
| 222768_s_at | TRMT6 | tRNA methyltransferase 6 homolog (S. cerevisiae) | 7.24E-05 | 7.9331 |
| 218586_at | C20orf20 | chromosome 20 open reading frame 20 | 8.90E-07 | 7.92913 |
| 224247_s_at | MRPS10 | mitochondrial ribosomal protein S10 | 4.25E-07 | 7.92856 |
| 225459_at | AMOTL1 | angiomotin like 1 | 9.77E-10 | 7.92622 |
| 224952_at | TANC2 | tetratricopeptide repeat, ankyrin repeat and coiled-coil containing 2 | 4.41E-07 | 7.92505 |
| 223506_at | ZC3H8 | zinc finger CCCH-type containing 8 | 4.96E-07 | 7.91957 |
| 212072_s_at | CSNK2A1 | casein kinase 2, alpha 1 polypeptide | 2.03E-07 | 7.91916 |
| 204021_s_at | PURA | purine-rich element binding protein A | 1.03E-06 | 7.91536 |
| 208911_s_at | PDHB | pyruvate dehydrogenase (lipoamide) beta | 1.32E-08 | 7.91527 |
| 202465_at | PCOLCE | procollagen C-endopeptidase enhancer | 4.07E-08 | 7.91464 |
| 204023_at | RFC4 | replication factor C (activator 1) 4, 37kDa | 1.70E-08 | 7.91287 |
| 225443_at | DCP1A | DCP1 decapping enzyme homolog A (S. cerevisiae) | 1.02E-07 | 7.90167 |
| 202303_x_at | SMARCA5 | SWI/SNF related, matrix associated, actin dependent regulator of chromatin, subf | 3.29E-07 | 7.89513 |
| 218348_s_at | ZC3H7A | zinc finger CCCH-type containing 7A | 2.19E-07 | 7.89353 |
| 218919_at | ZFAND1 | zinc finger, AN1-type domain 1 | 3.77E-06 | 7.87899 |
| 200732_s_at | PTP4A1 | protein tyrosine phosphatase type IVA, member 1 | 5.37E-10 | 7.87414 |
| 244764_at | HIVEP3 | Human immunodeficiency virus type I enhancer binding protein 3 | 1.25E-06 | 7.87205 |
| 202506_at | SSFA2 | sperm specific antigen 2 | 1.47E-05 | 7.86914 |
| 228674_s_at | EML4 | echinoderm microtubule associated protein like 4 | 6.66E-06 | 7.8624 |
| 201680_x_at | SRRT | serrate RNA effector molecule homolog (Arabidopsis) | 2.92E-06 | 7.86195 |
| 221845_s_at | CLPB | ClpB caseinolytic peptidase B homolog (E. coli) | 2.35E-08 | 7.8605 |
| 217841_s_at | PPME1 | protein phosphatase methylesterase 1 | 6.77E-06 | 7.85882 |
| 208795_s_at | MCM7 | minichromosome maintenance complex component 7 | 6.58E-08 | 7.85209 |
| 213012_at | NEDD4 | neural precursor cell expressed, developmentally down-regulated 4 | 1.11E-06 | 7.84867 |
| 202241_at | TRIB1 | tribbles homolog 1 (Drosophila) | 3.41E-07 | 7.84695 |
| 200679_x_at | HMGB1 | high-mobility group box 1 | 6.49E-07 | 7.8463 |
| 209357_at | CITED2 | Cbp/p300-interacting transactivator, with Glu/Asp-rich carboxy-terminal domain, | 6.14E-06 | 7.84606 |
| 208686_s_at | BRD2 | bromodomain containing 2 | 3.36E-07 | 7.84466 |
| 213892_s_at | APRT | adenine phosphoribosyltransferase | 7.15E-08 | 7.84144 |
| 204268_at | S100A2 | S100 calcium binding protein A2 | 2.90E-05 | 7.83153 |
| 219911_s_at | SLCO4A1 | solute carrier organic anion transporter family, member 4A1 | 0.0001793 | 7.82732 |
| 201962_s_at | RNF41 | ring finger protein 41 | 6.18E-07 | 7.82699 |
| 205100_at | GFPT2 | glutamine-fructose-6-phosphate transaminase 2 | 5.71E-08 | 7.82265 |
| 225154_at | SYAP1 | synapse associated protein 1, SAP47 homolog (Drosophila) | 1.26E-09 | 7.82064 |
| 202599_s_at | NRIP1 | nuclear receptor interacting protein 1 | 2.36E-07 | 7.82048 |
| 224654_at | DDX21 | DEAD (Asp-Glu-Ala-Asp) box polypeptide 21 | 5.09E-09 | 7.82024 |
| 210178_x_at | SFRS13A | splicing factor, arginine/serine-rich 13A | 1.06E-06 | 7.81958 |
| 217949_s_at | VKORC1 | vitamin K epoxide reductase complex, subunit 1 | 2.24E-09 | 7.81854 |
| 207088_s_at | SLC25A11 | solute carrier family 25 (mitochondrial carrier; oxoglutarate carrier), member 1 | 3.60E-06 | 7.81695 |
| 202167_s_at | MMS19 | MMS19 nucleotide excision repair homolog (S. cerevisiae) | 4.46E-07 | 7.81629 |
| 225528_at | IPO8 | importin 8 | 6.14E-08 | 7.81154 |
| 203755_at | BUB1B | budding uninhibited by benzimidazoles 1 homolog beta (yeast) | 1.33E-08 | 7.80407 |
| 218598_at | RINT1 | RAD50 interactor 1 | 5.78E-05 | 7.8032 |
| 220663_at | IL1RAPL1 | interleukin 1 receptor accessory protein-like 1 | 8.37E-06 | 7.79809 |
| 209882_at | RIT1 | Ras-like without CAAX 1 | 4.27E-06 | 7.79741 |
| 219709_x_at | FAM173A | family with sequence similarity 173, member A | 1.42E-07 | 7.79726 |
| 217798_at | CNOT2 | CCR4-NOT transcription complex, subunit 2 | 1.08E-05 | 7.7926 |
| 212804_s_at | GAPVD1 | GTPase activating protein and VPS9 domains 1 | 2.70E-08 | 7.79211 |
| 224968_at | CCDC104 | coiled-coil domain containing 104 | 3.71E-07 | 7.79128 |
| 202154_x_at | TUBB3 | tubulin, beta 3 | 9.29E-08 | 7.77789 |
| 202847_at | PCK2 | phosphoenolpyruvate carboxykinase 2 (mitochondrial) | 3.59E-09 | 7.77558 |
| 201241_at | DDX1 | DEAD (Asp-Glu-Ala-Asp) box polypeptide 1 | 1.32E-08 | 7.77268 |
| 219073_s_at | OSBPL10 | oxysterol binding protein-like 10 | 2.45E-08 | 7.76989 |
| 218530_at | FHOD1 | formin homology 2 domain containing 1 | 8.46E-06 | 7.76956 |
| 224565_at | NEAT1 | nuclear paraspeckle assembly transcript 1 (non-protein coding) | 5.03E-06 | 7.76849 |
| 226740_x_at | LOC200030 /// NBPF1 /// NBPF10 /// NBPF11 /// NBPF14 /// NBPF15 /// NBPF16 /// NBPF8 /// NBPF9 | neuroblastoma breakpoint family, member 11-like /// neuroblastoma breakpoint fam | 9.12E-09 | 7.76596 |
| 1555867_at | GNG4 | guanine nucleotide binding protein (G protein), gamma 4 | 9.46E-07 | 7.76413 |
| 219275_at | PDCD5 | programmed cell death 5 | 5.00E-07 | 7.76356 |
| 218284_at | SMAD3 | SMAD family member 3 | 1.99E-06 | 7.76313 |
| 219169_s_at | TFB1M | transcription factor B1, mitochondrial | 3.51E-06 | 7.76306 |
| 207614_s_at | CUL1 | cullin 1 | 5.50E-07 | 7.76057 |
| 203832_at | SNRPF | small nuclear ribonucleoprotein polypeptide F | 5.46E-08 | 7.75859 |
| 218848_at | THOC6 | THO complex 6 homolog (Drosophila) | 3.63E-10 | 7.7585 |
| 203964_at | NMI | N-myc (and STAT) interactor | 5.26E-07 | 7.7565 |
| 207023_x_at | KRT10 | keratin 10 | 4.36E-06 | 7.74461 |
| 218229_s_at | POGK | pogo transposable element with KRAB domain | 1.82E-08 | 7.74315 |
| 207358_x_at | MACF1 | microtubule-actin crosslinking factor 1 | 4.65E-06 | 7.74269 |
| 223331_s_at | DDX20 | DEAD (Asp-Glu-Ala-Asp) box polypeptide 20 | 4.20E-06 | 7.7426 |
| 201153_s_at | MBNL1 | muscleblind-like (Drosophila) | 2.45E-06 | 7.73829 |
| 227322_s_at | BCCIP | BRCA2 and CDKN1A interacting protein | 2.13E-09 | 7.73439 |
| 209015_s_at | DNAJB6 | DnaJ (Hsp40) homolog, subfamily B, member 6 | 1.06E-07 | 7.73429 |
| 225442_at | DDR2 | discoidin domain receptor tyrosine kinase 2 | 2.10E-07 | 7.73345 |
| 200652_at | SSR2 | signal sequence receptor, beta (translocon-associated protein beta) | 2.56E-08 | 7.73285 |
| 204771_s_at | TTF1 | transcription termination factor, RNA polymerase I | 4.25E-06 | 7.72789 |
| 222540_s_at | RSF1 | remodeling and spacing factor 1 | 3.07E-05 | 7.72559 |
| 226917_s_at | ANAPC4 | anaphase promoting complex subunit 4 | 1.37E-07 | 7.7242 |
| 200607_s_at | RAD21 | RAD21 homolog (S. pombe) | 7.37E-08 | 7.72153 |
| 221437_s_at | MRPS15 | mitochondrial ribosomal protein S15 | 6.62E-06 | 7.71924 |
| 209425_at | AMACR /// C1QTNF3 | alpha-methylacyl-CoA racemase /// C1q and tumor necrosis factor related protein | 1.98E-06 | 7.71721 |
| 223474_at | C14orf4 | chromosome 14 open reading frame 4 | 1.38E-07 | 7.7142 |
| 214548_x_at | GNAS | GNAS complex locus | 9.28E-09 | 7.71296 |
| 210438_x_at | TROVE2 | TROVE domain family, member 2 | 1.20E-08 | 7.71209 |
| 212178_s_at | POM121 /// POM121C | POM121 membrane glycoprotein (rat) /// POM121 membrane glycoprotein C | 6.33E-06 | 7.71181 |
| 233936_s_at | GGNBP2 | gametogenetin binding protein 2 | 2.79E-06 | 7.70542 |
| 215004_s_at | SF4 | splicing factor 4 | 9.39E-07 | 7.7016 |
| 208811_s_at | DNAJB6 /// TMEM135 | DnaJ (Hsp40) homolog, subfamily B, member 6 /// transmembrane protein 135 | 1.34E-06 | 7.70157 |
| 227514_at | ITPRIPL2 | inositol 1,4,5-triphosphate receptor interacting protein-like 2 | 2.25E-05 | 7.69961 |
| 226781_at | C7orf55 | chromosome 7 open reading frame 55 | 1.83E-07 | 7.69886 |
| 201462_at | SCRN1 | secernin 1 | 2.33E-09 | 7.69597 |
| 225106_s_at | OGFOD1 | 2-oxoglutarate and iron-dependent oxygenase domain containing 1 | 1.18E-07 | 7.69367 |
| 203575_at | CSNK2A2 | casein kinase 2, alpha prime polypeptide | 8.46E-09 | 7.69277 |
| 225167_at | FRMD4A | FERM domain containing 4A | 1.51E-06 | 7.69101 |
| 218715_at | UTP6 | UTP6, small subunit (SSU) processome component, homolog (yeast) | 8.06E-08 | 7.69066 |
| 210497_x_at | SSX2 /// SSX2B | synovial sarcoma, X breakpoint 2 /// synovial sarcoma, X breakpoint 2B | 7.63E-07 | 7.68716 |
| 215075_s_at | GRB2 | growth factor receptor-bound protein 2 | 1.94E-07 | 7.68694 |
| 208336_s_at | TECR | trans-2,3-enoyl-CoA reductase | 1.25E-07 | 7.6868 |
| 208831_x_at | SUPT6H | suppressor of Ty 6 homolog (S. cerevisiae) | 8.44E-08 | 7.68376 |
| 227992_s_at | NCRNA00085 | non-protein coding RNA 85 | 1.08E-05 | 7.68371 |
| 227558_at | CBX4 | chromobox homolog 4 (Pc class homolog, Drosophila) | 2.48E-07 | 7.67929 |
| 225743_at | RPUSD3 | RNA pseudouridylate synthase domain containing 3 | 4.52E-09 | 7.67624 |
| 210460_s_at | PSMD4 | proteasome (prosome, macropain) 26S subunit, non-ATPase, 4 | 4.57E-08 | 7.67423 |
| 213476_x_at | TUBB3 | tubulin, beta 3 | 1.61E-06 | 7.67397 |
| 217782_s_at | GPS1 | G protein pathway suppressor 1 | 7.20E-08 | 7.6705 |
| 208951_at | ALDH7A1 | aldehyde dehydrogenase 7 family, member A1 | 5.83E-06 | 7.66839 |
| 201673_s_at | GYS1 | glycogen synthase 1 (muscle) | 2.03E-06 | 7.66731 |
| 223306_at | EBPL | emopamil binding protein-like | 1.02E-07 | 7.66429 |
| 223135_s_at | BBX | bobby sox homolog (Drosophila) | 8.68E-08 | 7.6594 |
| 212493_s_at | SETD2 | SET domain containing 2 | 6.50E-07 | 7.65398 |
| 203401_at | PRPS2 | phosphoribosyl pyrophosphate synthetase 2 | 1.97E-05 | 7.6537 |
| 203155_at | SETDB1 | SET domain, bifurcated 1 | 3.00E-06 | 7.65304 |
| 225168_at | FRMD4A | FERM domain containing 4A | 2.97E-05 | 7.65237 |
| 215038_s_at | SETD2 | SET domain containing 2 | 7.80E-09 | 7.64934 |
| 209426_s_at | AMACR /// C1QTNF3 | alpha-methylacyl-CoA racemase /// C1q and tumor necrosis factor related protein | 2.63E-06 | 7.64714 |
| 218474_s_at | KCTD5 | potassium channel tetramerisation domain containing 5 | 9.03E-07 | 7.64066 |
| 202085_at | TJP2 | tight junction protein 2 (zona occludens 2) | 3.15E-06 | 7.64028 |
| 223328_at | ARMC10 | armadillo repeat containing 10 | 5.95E-09 | 7.63834 |
| 205429_s_at | MPP6 | membrane protein, palmitoylated 6 (MAGUK p55 subfamily member 6) | 2.75E-06 | 7.63015 |
| 209674_at | CRY1 | cryptochrome 1 (photolyase-like) | 4.49E-06 | 7.62954 |
| 211202_s_at | KDM5B | lysine (K)-specific demethylase 5B | 1.89E-05 | 7.62268 |
| 201848_s_at | BNIP3 | BCL2/adenovirus E1B 19kDa interacting protein 3 | 2.83E-08 | 7.62223 |
| 223132_s_at | TRIM8 | tripartite motif-containing 8 | 5.23E-08 | 7.61824 |
| 212022_s_at | MKI67 | antigen identified by monoclonal antibody Ki-67 | 7.25E-08 | 7.61742 |
| 214500_at | H2AFY | H2A histone family, member Y | 5.89E-06 | 7.6156 |
| 222867_s_at | MED31 | mediator complex subunit 31 | 7.32E-09 | 7.61499 |
| 204372_s_at | KHSRP | KH-type splicing regulatory protein | 1.90E-06 | 7.61444 |
| 208974_x_at | KPNB1 | karyopherin (importin) beta 1 | 3.75E-08 | 7.60908 |
| 225526_at | MKLN1 | muskelin 1, intracellular mediator containing kelch motifs | 2.33E-08 | 7.60783 |
| 210681_s_at | USP15 | ubiquitin specific peptidase 15 | 3.15E-06 | 7.60524 |
| 213364_s_at | SNX1 | sorting nexin 1 | 5.24E-07 | 7.60452 |
| 219539_at | GEMIN6 | gem (nuclear organelle) associated protein 6 | 5.19E-06 | 7.5974 |
| 222996_s_at | CXXC5 | CXXC finger 5 | 1.14E-06 | 7.59593 |
| 201473_at | JUNB | jun B proto-oncogene | 1.32E-05 | 7.58627 |
| 225687_at | FAM83D | family with sequence similarity 83, member D | 2.81E-08 | 7.58474 |
| 224919_at | MRPS6 | mitochondrial ribosomal protein S6 | 1.07E-08 | 7.58234 |
| 1555780_a_at | RHEB | Ras homolog enriched in brain | 2.19E-05 | 7.58171 |
| 65585_at | FAM86B1 | family with sequence similarity 86, member B1 | 2.18E-07 | 7.5783 |
| 1556283_s_at | FGFR1OP2 | FGFR1 oncogene partner 2 | 2.44E-06 | 7.57611 |
| 206102_at | GINS1 | GINS complex subunit 1 (Psf1 homolog) | 9.57E-08 | 7.57306 |
| 212348_s_at | KDM1A | lysine (K)-specific demethylase 1A | 1.58E-06 | 7.5723 |
| 209211_at | KLF5 | Kruppel-like factor 5 (intestinal) | 0.0001117 | 7.57223 |
| 226087_at | LZIC | leucine zipper and CTNNBIP1 domain containing | 1.11E-06 | 7.57082 |
| 204097_s_at | RBMX2 | RNA binding motif protein, X-linked 2 | 6.83E-06 | 7.57021 |
| 224842_at | SMG1 | SMG1 homolog, phosphatidylinositol 3-kinase-related kinase (C. elegans) | 2.03E-05 | 7.56789 |
| 222785_x_at | C11orf1 | chromosome 11 open reading frame 1 | 1.48E-07 | 7.56497 |
| 214039_s_at | LAPTM4B | lysosomal protein transmembrane 4 beta | 7.89E-06 | 7.56168 |
| 212030_at | RBM25 | RNA binding motif protein 25 | 1.13E-06 | 7.56145 |
| 233540_s_at | CDK5RAP2 | CDK5 regulatory subunit associated protein 2 | 6.36E-07 | 7.55783 |
| 223639_s_at | ZNRD1 | zinc ribbon domain containing 1 | 1.04E-06 | 7.55426 |
| 218535_s_at | RIOK2 | RIO kinase 2 (yeast) | 1.50E-05 | 7.55406 |
| 218302_at | PSENEN | presenilin enhancer 2 homolog (C. elegans) | 2.11E-05 | 7.55348 |
| 217932_at | MRPS7 | mitochondrial ribosomal protein S7 | 1.53E-07 | 7.54951 |
| 222250_s_at | INTS7 | integrator complex subunit 7 | 1.76E-06 | 7.54949 |
| 213018_at | GATAD1 | GATA zinc finger domain containing 1 | 9.02E-07 | 7.54545 |
| 203432_at | TMPO | thymopoietin | 1.44E-07 | 7.54513 |
| 206055_s_at | SNRPA1 | small nuclear ribonucleoprotein polypeptide A' | 5.17E-06 | 7.54499 |
| 203680_at | PRKAR2B | protein kinase, cAMP-dependent, regulatory, type II, beta | 7.70E-06 | 7.53559 |
| 239896_at | LOC100289098 | Hypothetical protein LOC100289098 | 4.58E-07 | 7.53545 |
| 217833_at | SYNCRIP | synaptotagmin binding, cytoplasmic RNA interacting protein | 7.61E-09 | 7.53544 |
| 228302_x_at | CAMK2N1 | calcium/calmodulin-dependent protein kinase II inhibitor 1 | 1.10E-06 | 7.52591 |
| 201232_s_at | PSMD13 | proteasome (prosome, macropain) 26S subunit, non-ATPase, 13 | 4.84E-09 | 7.52478 |
| 200940_s_at | RERE | arginine-glutamic acid dipeptide (RE) repeats | 5.17E-07 | 7.5218 |
| 202406_s_at | TIAL1 | TIA1 cytotoxic granule-associated RNA binding protein-like 1 | 5.73E-09 | 7.51743 |
| 209053_s_at | WHSC1 | Wolf-Hirschhorn syndrome candidate 1 | 2.31E-07 | 7.51536 |
| 208863_s_at | SFRS1 | splicing factor, arginine/serine-rich 1 | 6.21E-06 | 7.5123 |
| 203396_at | PSMA4 | proteasome (prosome, macropain) subunit, alpha type, 4 | 1.35E-07 | 7.51099 |
| 227003_at | RAB28 | RAB28, member RAS oncogene family | 1.69E-07 | 7.50655 |
| 214766_s_at | AHCTF1 | AT hook containing transcription factor 1 | 0.0002598 | 7.50501 |
| 209185_s_at | IRS2 | insulin receptor substrate 2 | 2.03E-09 | 7.50276 |
| 225698_at | NCRNA00219 | non-protein coding RNA 219 | 7.71E-06 | 7.50193 |
| 203035_s_at | PIAS3 | protein inhibitor of activated STAT, 3 | 1.64E-07 | 7.50114 |
| 205310_at | FBXO46 | F-box protein 46 | 5.95E-06 | 7.49793 |
| 203462_x_at | EIF3B | eukaryotic translation initiation factor 3, subunit B | 2.12E-06 | 7.49641 |
| 201939_at | PLK2 | polo-like kinase 2 (Drosophila) | 3.25E-07 | 7.494 |
| 1554365_a_at | PPP2R5C | protein phosphatase 2, regulatory subunit B', gamma | 3.72E-06 | 7.49213 |
| 222728_s_at | TAF1D | TATA box binding protein (TBP)-associated factor, RNA polymerase I, D, 41kDa | 1.90E-08 | 7.49157 |
| 231713_s_at | ELP2 | elongation protein 2 homolog (S. cerevisiae) | 9.89E-05 | 7.49032 |
| 211951_at | NOLC1 | nucleolar and coiled-body phosphoprotein 1 | 8.83E-06 | 7.48926 |
| 203418_at | CCNA2 | cyclin A2 | 3.17E-06 | 7.48696 |
| 209383_at | DDIT3 | DNA-damage-inducible transcript 3 | 9.32E-07 | 7.48669 |
| 221563_at | DUSP10 | dual specificity phosphatase 10 | 1.99E-09 | 7.48566 |
| 222890_at | CCDC113 | coiled-coil domain containing 113 | 3.14E-06 | 7.48456 |
| 201244_s_at | RAF1 | v-raf-1 murine leukemia viral oncogene homolog 1 | 3.47E-07 | 7.48316 |
| 222199_s_at | BIN3 | bridging integrator 3 | 5.05E-08 | 7.48277 |
| 212411_at | IMP4 | IMP4, U3 small nucleolar ribonucleoprotein, homolog (yeast) | 9.06E-09 | 7.47762 |
| 204205_at | APOBEC3G | apolipoprotein B mRNA editing enzyme, catalytic polypeptide-like 3G | 1.38E-07 | 7.47215 |
| 203701_s_at | TRMT1 | TRM1 tRNA methyltransferase 1 homolog (S. cerevisiae) | 2.73E-07 | 7.47032 |
| 222398_s_at | EFTUD2 | elongation factor Tu GTP binding domain containing 2 | 4.20E-08 | 7.46975 |
| 224628_at | ERLEC1 | endoplasmic reticulum lectin 1 | 9.33E-08 | 7.46862 |
| 238515_at | NUDT16 | nudix (nucleoside diphosphate linked moiety X)-type motif 16 | 4.52E-06 | 7.46691 |
| 216306_x_at | PTBP1 | polypyrimidine tract binding protein 1 | 5.38E-10 | 7.45987 |
| 208954_s_at | LARP4B | La ribonucleoprotein domain family, member 4B | 1.00E-06 | 7.45648 |
| 208541_x_at | TFAM | transcription factor A, mitochondrial | 1.19E-07 | 7.45248 |
| 224911_s_at | DCBLD2 | discoidin, CUB and LCCL domain containing 2 | 1.49E-07 | 7.444 |
| 1558173_a_at | LUZP1 | leucine zipper protein 1 | 1.89E-06 | 7.44143 |
| 218842_at | RPAP3 | RNA polymerase II associated protein 3 | 1.49E-06 | 7.4399 |
| 33132_at | CPSF1 | cleavage and polyadenylation specific factor 1, 160kDa | 5.36E-07 | 7.43829 |
| 208478_s_at | BAX | BCL2-associated X protein | 9.59E-06 | 7.43443 |
| 227110_at | HNRNPC | heterogeneous nuclear ribonucleoprotein C (C1/C2) | 3.40E-08 | 7.43405 |
| 209268_at | VPS45 | vacuolar protein sorting 45 homolog (S. cerevisiae) | 1.83E-08 | 7.43305 |
| 1557953_at | ZKSCAN1 | zinc finger with KRAB and SCAN domains 1 | 1.95E-08 | 7.43117 |
| 201457_x_at | BUB3 | budding uninhibited by benzimidazoles 3 homolog (yeast) | 4.18E-08 | 7.43114 |
| 200849_s_at | AHCYL1 | adenosylhomocysteinase-like 1 | 1.37E-06 | 7.43069 |
| 200987_x_at | PSME3 | proteasome (prosome, macropain) activator subunit 3 (PA28 gamma; Ki) | 1.03E-06 | 7.42773 |
| 231182_at | WIPF1 | WAS/WASL interacting protein family, member 1 | 8.51E-08 | 7.42544 |
| 205453_at | HOXB2 | homeobox B2 | 1.21E-08 | 7.42218 |
| 227077_at | ZNF286A /// ZNF286B | zinc finger protein 286A /// zinc finger protein 286B | 8.38E-07 | 7.42024 |
| 239392_s_at | POGK | pogo transposable element with KRAB domain | 2.06E-06 | 7.42012 |
| 203246_s_at | NPRL2 | nitrogen permease regulator-like 2 (S. cerevisiae) | 8.43E-06 | 7.41987 |
| 224799_at | NDFIP2 | Nedd4 family interacting protein 2 | 7.05E-06 | 7.41905 |
| 209424_s_at | AMACR /// C1QTNF3 | alpha-methylacyl-CoA racemase /// C1q and tumor necrosis factor related protein | 1.19E-06 | 7.41665 |
| 225213_at | PPTC7 | PTC7 protein phosphatase homolog (S. cerevisiae) | 4.68E-07 | 7.41489 |
| 202293_at | STAG1 | stromal antigen 1 | 1.24E-06 | 7.409 |
| 212607_at | AKT3 | v-akt murine thymoma viral oncogene homolog 3 (protein kinase B, gamma) | 7.43E-08 | 7.40732 |
| 212694_s_at | PCCB | propionyl CoA carboxylase, beta polypeptide | 4.24E-06 | 7.40692 |
| 218744_s_at | PACSIN3 | protein kinase C and casein kinase substrate in neurons 3 | 2.23E-07 | 7.40527 |
| 218497_s_at | RNASEH1 | ribonuclease H1 | 1.94E-07 | 7.40289 |
| 226722_at | FAM20C | family with sequence similarity 20, member C | 1.37E-05 | 7.40199 |
| 222527_s_at | RBM22 | RNA binding motif protein 22 | 8.55E-05 | 7.40014 |
| 41037_at | TEAD4 | TEA domain family member 4 | 1.98E-08 | 7.39998 |
| 201267_s_at | PSMC3 | proteasome (prosome, macropain) 26S subunit, ATPase, 3 | 2.24E-07 | 7.39872 |
| 50374_at | C17orf90 | chromosome 17 open reading frame 90 | 2.03E-06 | 7.39741 |
| 222666_s_at | RCL1 | RNA terminal phosphate cyclase-like 1 | 4.30E-07 | 7.38749 |
| 201586_s_at | SFPQ | splicing factor proline/glutamine-rich | 2.55E-09 | 7.38611 |
| 208420_x_at | SUPT6H | suppressor of Ty 6 homolog (S. cerevisiae) | 1.26E-07 | 7.38413 |
| 225551_at | CNST | consortin, connexin sorting protein | 2.78E-06 | 7.38327 |
| 201708_s_at | NIPSNAP1 | nipsnap homolog 1 (C. elegans) | 8.40E-07 | 7.38236 |
| 223363_at | PSMG3 | proteasome (prosome, macropain) assembly chaperone 3 | 3.36E-09 | 7.37384 |
| 201687_s_at | API5 | apoptosis inhibitor 5 | 2.34E-06 | 7.37266 |
| 202417_at | KEAP1 | kelch-like ECH-associated protein 1 | 2.07E-07 | 7.37001 |
| 200875_s_at | NOP56 | NOP56 ribonucleoprotein homolog (yeast) | 1.28E-09 | 7.36476 |
| 226421_at | AMMECR1 | Alport syndrome, mental retardation, midface hypoplasia and elliptocytosis chrom | 6.67E-06 | 7.36471 |
| 202578_s_at | DDX19A | DEAD (Asp-Glu-Ala-As) box polypeptide 19A | 6.34E-08 | 7.36246 |
| 204157_s_at | SIK3 | SIK family kinase 3 | 1.28E-07 | 7.35865 |
| 224478_s_at | C7orf50 | chromosome 7 open reading frame 50 | 5.67E-06 | 7.35753 |
| 222774_s_at | NETO2 | neuropilin (NRP) and tolloid (TLL)-like 2 | 1.30E-05 | 7.35748 |
| 201379_s_at | TPD52L2 | tumor protein D52-like 2 | 1.71E-06 | 7.35671 |
| 203664_s_at | POLR2D | polymerase (RNA) II (DNA directed) polypeptide D | 3.76E-06 | 7.35228 |
| 202297_s_at | RER1 | RER1 retention in endoplasmic reticulum 1 homolog (S. cerevisiae) | 1.65E-07 | 7.35096 |
| 208079_s_at | AURKA | aurora kinase A | 5.32E-09 | 7.35063 |
| 212388_at | USP24 | ubiquitin specific peptidase 24 | 4.27E-05 | 7.34875 |
| 204658_at | TRA2A | transformer 2 alpha homolog (Drosophila) | 3.44E-07 | 7.34573 |
| 218976_at | DNAJC12 | DnaJ (Hsp40) homolog, subfamily C, member 12 | 1.28E-05 | 7.34399 |
| 226479_at | KBTBD6 | kelch repeat and BTB (POZ) domain containing 6 | 6.92E-06 | 7.34398 |
| 235266_at | ATAD2 | ATPase family, AAA domain containing 2 | 1.43E-05 | 7.34392 |
| 203856_at | VRK1 | vaccinia related kinase 1 | 1.11E-07 | 7.3433 |
| 201024_x_at | EIF5B | eukaryotic translation initiation factor 5B | 1.85E-08 | 7.34263 |
| 200968_s_at | PPIB | peptidylprolyl isomerase B (cyclophilin B) | 4.82E-08 | 7.33789 |
| 212232_at | FNBP4 | formin binding protein 4 | 7.66E-08 | 7.33489 |
| 223332_x_at | RNF126 | ring finger protein 126 | 1.41E-06 | 7.33316 |
| 210418_s_at | IDH3B | isocitrate dehydrogenase 3 (NAD+) beta | 1.64E-06 | 7.32831 |
| 35436_at | GOLGA2 | golgin A2 | 2.07E-07 | 7.3234 |
| 228031_at | TTPAL | tocopherol (alpha) transfer protein-like | 2.23E-07 | 7.32287 |
| 224956_at | NUFIP2 | nuclear fragile X mental retardation protein interacting protein 2 | 1.22E-08 | 7.32209 |
| 201489_at | PPIF | peptidylprolyl isomerase F | 1.69E-07 | 7.32163 |
| 203267_s_at | DRG2 | developmentally regulated GTP binding protein 2 | 8.01E-08 | 7.32088 |
| 204244_s_at | DBF4 | DBF4 homolog (S. cerevisiae) | 2.75E-07 | 7.31712 |
| 206656_s_at | C20orf3 | chromosome 20 open reading frame 3 | 2.87E-08 | 7.31034 |
| 218260_at | DDA1 | DET1 and DDB1 associated 1 | 2.36E-07 | 7.30343 |
| 206958_s_at | UPF3A | UPF3 regulator of nonsense transcripts homolog A (yeast) | 8.88E-07 | 7.30252 |
| 203511_s_at | TRAPPC3 | trafficking protein particle complex 3 | 1.61E-08 | 7.3015 |
| 225439_at | NUDCD1 | NudC domain containing 1 | 1.36E-05 | 7.30002 |
| 208393_s_at | RAD50 | RAD50 homolog (S. cerevisiae) | 2.31E-06 | 7.29971 |
| 203919_at | TCEA2 | transcription elongation factor A (SII), 2 | 1.12E-07 | 7.29791 |
| 223010_s_at | OCIAD1 | OCIA domain containing 1 | 4.99E-06 | 7.29633 |
| 225209_s_at | UBE2J2 | ubiquitin-conjugating enzyme E2, J2 (UBC6 homolog, yeast) | 3.35E-09 | 7.29566 |
| 1552348_at | PRSS33 | protease, serine, 33 | 1.36E-07 | 7.29564 |
| 225823_at | C19orf70 | chromosome 19 open reading frame 70 | 1.18E-05 | 7.28892 |
| 222029_x_at | PFDN6 | prefoldin subunit 6 | 1.03E-05 | 7.28765 |
| 225945_at | ZNF655 | zinc finger protein 655 | 7.78E-06 | 7.28515 |
| 222700_at | ATL2 | atlastin GTPase 2 | 2.30E-05 | 7.28243 |
| 224610_at | SNHG1 | small nucleolar RNA host gene 1 (non-protein coding) | 1.37E-07 | 7.27916 |
| 209285_s_at | C3orf63 | chromosome 3 open reading frame 63 | 8.68E-07 | 7.27765 |
| 210817_s_at | CALCOCO2 | calcium binding and coiled-coil domain 2 | 4.29E-08 | 7.27692 |
| 220892_s_at | PSAT1 | phosphoserine aminotransferase 1 | 8.80E-06 | 7.27409 |
| 204384_at | GOLGA2 | golgin A2 | 2.36E-06 | 7.26762 |
| 201837_s_at | SUPT7L | suppressor of Ty 7 (S. cerevisiae)-like | 8.83E-07 | 7.26623 |
| 231202_at | ALDH1L2 | aldehyde dehydrogenase 1 family, member L2 | 3.86E-05 | 7.26444 |
| 200977_s_at | TAX1BP1 | Tax1 (human T-cell leukemia virus type I) binding protein 1 | 2.83E-08 | 7.26444 |
| 221277_s_at | PUS3 | pseudouridylate synthase 3 | 5.46E-08 | 7.26401 |
| 206016_at | CCDC22 | coiled-coil domain containing 22 | 3.86E-05 | 7.26398 |
| 225617_at | ODF2 | outer dense fiber of sperm tails 2 | 7.83E-07 | 7.26375 |
| 226276_at | TMEM167A | transmembrane protein 167A | 8.03E-07 | 7.2636 |
| 202024_at | ASNA1 | arsA arsenite transporter, ATP-binding, homolog 1 (bacterial) | 1.82E-06 | 7.26348 |
| 201725_at | CDC123 | cell division cycle 123 homolog (S. cerevisiae) | 1.21E-06 | 7.26293 |
| 204215_at | C7orf23 | chromosome 7 open reading frame 23 | 1.10E-07 | 7.26192 |
| 219563_at | C14orf139 | chromosome 14 open reading frame 139 | 1.81E-08 | 7.2618 |
| 202164_s_at | CNOT8 | CCR4-NOT transcription complex, subunit 8 | 6.19E-07 | 7.25828 |
| 201827_at | SMARCD2 | SWI/SNF related, matrix associated, actin dependent regulator of chromatin, subf | 1.61E-08 | 7.25732 |
| 221559_s_at | MIS12 | MIS12, MIND kinetochore complex component, homolog (S. pombe) | 9.13E-06 | 7.256 |
| 1556062_at | RPP30 | ribonuclease P/MRP 30kDa subunit | 1.18E-05 | 7.25303 |
| 224608_s_at | VPS25 | vacuolar protein sorting 25 homolog (S. cerevisiae) | 8.50E-10 | 7.2492 |
| 205329_s_at | SNX4 | sorting nexin 4 | 4.12E-07 | 7.2482 |
| 225243_s_at | SLMAP | sarcolemma associated protein | 2.27E-07 | 7.24662 |
| 203489_at | SIVA1 | SIVA1, apoptosis-inducing factor | 8.68E-08 | 7.24265 |
| 219117_s_at | FKBP11 | FK506 binding protein 11, 19 kDa | 8.15E-08 | 7.2416 |
| 229394_s_at | GRLF1 | glucocorticoid receptor DNA binding factor 1 | 8.65E-05 | 7.24083 |
| 208922_s_at | NXF1 | nuclear RNA export factor 1 | 1.05E-08 | 7.24016 |
| 203774_at | MTR | 5-methyltetrahydrofolate-homocysteine methyltransferase | 1.23E-07 | 7.23968 |
| 229630_s_at | WTAP | Wilms tumor 1 associated protein | 1.86E-07 | 7.23743 |
| 225143_at | SFXN4 | sideroflexin 4 | 6.88E-05 | 7.23622 |
| 218691_s_at | PDLIM4 | PDZ and LIM domain 4 | 9.58E-07 | 7.23571 |
| 209100_at | IFRD2 | interferon-related developmental regulator 2 | 4.21E-08 | 7.2349 |
| 223231_at | TATDN1 | TatD DNase domain containing 1 | 3.68E-06 | 7.23477 |
| 226386_at | C7orf30 | chromosome 7 open reading frame 30 | 3.68E-08 | 7.23462 |
| 218799_at | GPN2 | GPN-loop GTPase 2 | 1.10E-05 | 7.2303 |
| 203073_at | COG2 | component of oligomeric golgi complex 2 | 4.65E-07 | 7.22495 |
| 202623_at | EAPP | E2F-associated phosphoprotein | 1.80E-05 | 7.22471 |
| 213655_at | YWHAE | Tyrosine 3-monooxygenase/tryptophan 5-monooxygenase activation protein, epsilon | 1.51E-06 | 7.22053 |
| 211653_x_at | AKR1C2 | aldo-keto reductase family 1, member C2 (dihydrodiol dehydrogenase 2; bile acid | 4.33E-07 | 7.21967 |
| 209459_s_at | ABAT | 4-aminobutyrate aminotransferase | 8.20E-05 | 7.21855 |
| 235911_at | MFI2 | antigen p97 (melanoma associated) identified by monoclonal antibodies 133.2 and | 3.31E-06 | 7.2182 |
| 225219_at | SMAD5 | SMAD family member 5 | 7.37E-06 | 7.21749 |
| 201932_at | LRRC41 | leucine rich repeat containing 41 | 1.11E-06 | 7.21687 |
| 224446_at | LLPH | LLP homolog, long-term synaptic facilitation (Aplysia) | 1.67E-06 | 7.21654 |
| 226881_at | GRPEL2 | GrpE-like 2, mitochondrial (E. coli) | 3.27E-07 | 7.21233 |
| 202407_s_at | PRPF31 | PRP31 pre-mRNA processing factor 31 homolog (S. cerevisiae) | 6.08E-06 | 7.21165 |
| 204835_at | POLA1 | polymerase (DNA directed), alpha 1, catalytic subunit | 1.32E-07 | 7.2098 |
| 217168_s_at | HERPUD1 | homocysteine-inducible, endoplasmic reticulum stress-inducible, ubiquitin-like d | 1.05E-05 | 7.20857 |
| 201522_x_at | SNRPN /// SNURF | small nuclear ribonucleoprotein polypeptide N /// SNRPN upstream reading frame | 9.25E-09 | 7.20695 |
| 212783_at | RBBP6 | retinoblastoma binding protein 6 | 3.90E-07 | 7.20448 |
| 204186_s_at | PPID | peptidylprolyl isomerase D | 4.52E-06 | 7.20385 |
| 201063_at | RCN1 | reticulocalbin 1, EF-hand calcium binding domain | 1.55E-11 | 7.20335 |
| 201595_s_at | ZC3H15 | zinc finger CCCH-type containing 15 | 2.57E-06 | 7.2014 |
| 230363_s_at | INPP5F | inositol polyphosphate-5-phosphatase F | 1.31E-06 | 7.19767 |
| 200666_s_at | DNAJB1 | DnaJ (Hsp40) homolog, subfamily B, member 1 | 1.63E-07 | 7.19649 |
| 224444_s_at | C1orf97 | chromosome 1 open reading frame 97 | 3.47E-07 | 7.19432 |
| 226051_at | SELM | selenoprotein M | 4.78E-07 | 7.1927 |
| 216035_x_at | TCF7L2 | transcription factor 7-like 2 (T-cell specific, HMG-box) | 5.23E-06 | 7.19058 |
| 212927_at | SMC5 | structural maintenance of chromosomes 5 | 1.26E-06 | 7.18972 |
| 218668_s_at | RAP2C | RAP2C, member of RAS oncogene family | 1.57E-06 | 7.18876 |
| 201170_s_at | BHLHE40 | basic helix-loop-helix family, member e40 | 4.11E-07 | 7.18774 |
| 210428_s_at | HGS | hepatocyte growth factor-regulated tyrosine kinase substrate | 2.12E-08 | 7.18769 |
| 202189_x_at | PTBP1 | polypyrimidine tract binding protein 1 | 5.08E-09 | 7.18633 |
| 233929_x_at | WASH3P | WAS protein family homolog 3 pseudogene | 1.06E-07 | 7.18488 |
| 201800_s_at | OSBP | oxysterol binding protein | 1.88E-05 | 7.18258 |
| 238996_x_at | ALDOA | aldolase A, fructose-bisphosphate | 1.02E-05 | 7.18147 |
| 217958_at | TRAPPC4 | trafficking protein particle complex 4 | 1.21E-08 | 7.18044 |
| 217785_s_at | YKT6 | YKT6 v-SNARE homolog (S. cerevisiae) | 3.12E-06 | 7.17935 |
| 202775_s_at | SFRS8 | splicing factor, arginine/serine-rich 8 (suppressor-of-white-apricot homolog, Dr | 9.15E-07 | 7.1758 |
| 210283_x_at | LOC645139 /// PAIP1 | similar to poly(A) binding protein interacting protein 1 /// poly(A) binding pro | 9.31E-05 | 7.17477 |
| 219363_s_at | MTERFD1 | MTERF domain containing 1 | 2.59E-06 | 7.17393 |
| 219286_s_at | RBM15 | RNA binding motif protein 15 | 2.92E-09 | 7.16324 |
| 203576_at | BCAT2 | branched chain amino-acid transaminase 2, mitochondrial | 2.72E-06 | 7.16177 |
| 226176_s_at | USP42 | ubiquitin specific peptidase 42 | 3.01E-06 | 7.16121 |
| 207320_x_at | STAU1 | staufen, RNA binding protein, homolog 1 (Drosophila) | 8.54E-11 | 7.15644 |
| 214993_at | ASPHD1 | aspartate beta-hydroxylase domain containing 1 | 1.93E-05 | 7.15442 |
| 203039_s_at | NDUFS1 | NADH dehydrogenase (ubiquinone) Fe-S protein 1, 75kDa (NADH-coenzyme Q reductase | 1.12E-06 | 7.1539 |
| 210968_s_at | RTN4 | reticulon 4 | 6.48E-07 | 7.15185 |
| 202355_s_at | GTF2F1 | general transcription factor IIF, polypeptide 1, 74kDa | 3.34E-07 | 7.15179 |
| 209095_at | DLD | dihydrolipoamide dehydrogenase | 2.67E-07 | 7.15169 |
| 212893_at | ZZZ3 | zinc finger, ZZ-type containing 3 | 1.01E-05 | 7.14832 |
| 209732_at | CLEC2B | C-type lectin domain family 2, member B | 2.62E-08 | 7.14658 |
| 1569669_at | FOXR2 | forkhead box R2 | 7.34E-06 | 7.14323 |
| 202690_s_at | SNRPD1 | small nuclear ribonucleoprotein D1 polypeptide 16kDa | 1.97E-07 | 7.14266 |
| 201397_at | PHGDH | phosphoglycerate dehydrogenase | 1.63E-05 | 7.14253 |
| 225461_at | EHMT1 | euchromatic histone-lysine N-methyltransferase 1 | 2.54E-07 | 7.13948 |
| 201233_at | PSMD13 | proteasome (prosome, macropain) 26S subunit, non-ATPase, 13 | 4.90E-06 | 7.1382 |
| 222680_s_at | DTL | denticleless homolog (Drosophila) | 1.61E-06 | 7.1378 |
| 224879_at | C9orf123 | chromosome 9 open reading frame 123 | 5.32E-08 | 7.13699 |
| 202183_s_at | KIF22 | kinesin family member 22 | 3.27E-06 | 7.13679 |
| 200906_s_at | PALLD | palladin, cytoskeletal associated protein | 3.59E-06 | 7.13576 |
| 202078_at | COPS3 | COP9 constitutive photomorphogenic homolog subunit 3 (Arabidopsis) | 1.02E-05 | 7.13446 |
| 220746_s_at | UIMC1 | ubiquitin interaction motif containing 1 | 1.86E-07 | 7.13428 |
| 207791_s_at | RAB1A | RAB1A, member RAS oncogene family | 9.81E-07 | 7.13347 |
| 208979_at | NCOA6 | nuclear receptor coactivator 6 | 3.47E-08 | 7.13311 |
| 223528_s_at | LOC731602 /// METT11D1 | similar to methyltransferase 11 domain containing 1 isoform 2 /// methyltransfer | 4.28E-06 | 7.13254 |
| 226867_at | DENND4C | DENN/MADD domain containing 4C | 2.06E-07 | 7.13182 |
| 202042_at | HARS | histidyl-tRNA synthetase | 1.52E-06 | 7.13178 |
| 206075_s_at | CSNK2A1 | casein kinase 2, alpha 1 polypeptide | 1.35E-06 | 7.13083 |
| 223096_at | NOP58 | NOP58 ribonucleoprotein homolog (yeast) | 2.71E-06 | 7.13023 |
| 204258_at | CHD1 | chromodomain helicase DNA binding protein 1 | 7.56E-07 | 7.12884 |
| 1560060_s_at | VPS37C | vacuolar protein sorting 37 homolog C (S. cerevisiae) | 2.64E-08 | 7.12756 |
| 225146_at | C9orf25 | chromosome 9 open reading frame 25 | 1.10E-06 | 7.1236 |
| 201196_s_at | AMD1 | adenosylmethionine decarboxylase 1 | 5.33E-08 | 7.12216 |
| 222393_s_at | NAA50 | N(alpha)-acetyltransferase 50, NatE catalytic subunit | 8.56E-06 | 7.12024 |
| 203072_at | MYO1E | myosin IE | 3.19E-05 | 7.11775 |
| 212474_at | AVL9 | AVL9 homolog (S. cerevisiase) | 1.38E-06 | 7.11625 |
| 210125_s_at | BANF1 | barrier to autointegration factor 1 | 1.04E-06 | 7.11461 |
| 226312_at | RICTOR | RPTOR independent companion of MTOR, complex 2 | 2.44E-06 | 7.11037 |
| 227517_s_at | GAS5 | growth arrest-specific 5 (non-protein coding) | 4.36E-08 | 7.10719 |
| 223618_at | FMN2 | formin 2 | 4.99E-08 | 7.10277 |
| 202414_at | ERCC5 | excision repair cross-complementing rodent repair deficiency, complementation gr | 1.95E-06 | 7.10137 |
| 204370_at | CLP1 | CLP1, cleavage and polyadenylation factor I subunit, homolog (S. cerevisiae) | 5.88E-06 | 7.1002 |
| 225135_at | SIN3A | SIN3 homolog A, transcription regulator (yeast) | 1.68E-08 | 7.09427 |
| 210949_s_at | EIF3C /// EIF3CL | eukaryotic translation initiation factor 3, subunit C /// eukaryotic translation | 1.39E-07 | 7.09244 |
| 201950_x_at | CAPZB | capping protein (actin filament) muscle Z-line, beta | 9.63E-06 | 7.09064 |
| 227164_at | SFRS1 | splicing factor, arginine/serine-rich 1 | 2.53E-06 | 7.09003 |
| 212762_s_at | TCF7L2 | transcription factor 7-like 2 (T-cell specific, HMG-box) | 5.09E-06 | 7.08863 |
| 226868_at | GXYLT1 | glucoside xylosyltransferase 1 | 2.40E-08 | 7.08762 |
| 200033_at | DDX5 | DEAD (Asp-Glu-Ala-Asp) box polypeptide 5 | 1.03E-06 | 7.08507 |
| 226963_at | BTF3L4 | basic transcription factor 3-like 4 | 2.49E-08 | 7.08412 |
| 213190_at | COG7 | component of oligomeric golgi complex 7 | 8.09E-06 | 7.08332 |
| 218386_x_at | USP16 | ubiquitin specific peptidase 16 | 1.57E-06 | 7.08325 |
| 229461_x_at | NEGR1 | neuronal growth regulator 1 | 1.74E-06 | 7.08273 |
| 227330_x_at | LOC100132288 /// LOC100233156 /// MAFIP | hypothetical protein LOC100132288 /// hypothetical protein LOC100233156 /// MAFF | 5.50E-09 | 7.07728 |
| 212544_at | ZNHIT3 | zinc finger, HIT type 3 | 3.06E-08 | 7.07359 |
| 204767_s_at | FEN1 | flap structure-specific endonuclease 1 | 8.34E-09 | 7.07329 |
| 217872_at | PIH1D1 | PIH1 domain containing 1 | 1.53E-05 | 7.07185 |
| 211833_s_at | BAX | BCL2-associated X protein | 1.71E-06 | 7.07041 |
| 226566_at | TRIM11 | tripartite motif-containing 11 | 3.31E-07 | 7.06989 |
| 225635_s_at | LOC401504 | Hypothetical gene supported by AK091718 | 1.56E-05 | 7.06911 |
| 200806_s_at | HSPD1 | heat shock 60kDa protein 1 (chaperonin) | 1.55E-05 | 7.06656 |
| 224725_at | MIB1 | mindbomb homolog 1 (Drosophila) | 1.60E-05 | 7.06291 |
| 220990_s_at | MIR21 /// TMEM49 | microRNA 21 /// transmembrane protein 49 | 1.04E-07 | 7.06208 |
| 235394_at | PLAA | phospholipase A2-activating protein | 2.07E-05 | 7.05973 |
| 208860_s_at | ATRX | alpha thalassemia/mental retardation syndrome X-linked (RAD54 homolog, S. cerevi | 5.47E-05 | 7.05849 |
| 34764_at | LARS2 | leucyl-tRNA synthetase 2, mitochondrial | 3.08E-06 | 7.05679 |
| 200769_s_at | MAT2A | methionine adenosyltransferase II, alpha | 4.00E-07 | 7.05432 |
| 203686_at | MPG | N-methylpurine-DNA glycosylase | 1.66E-07 | 7.05313 |
| 202429_s_at | PPP3CA | protein phosphatase 3, catalytic subunit, alpha isozyme | 6.60E-07 | 7.05157 |
| 200957_s_at | SSRP1 | structure specific recognition protein 1 | 7.24E-07 | 7.04984 |
| 203082_at | BMS1 | BMS1 homolog, ribosome assembly protein (yeast) | 3.61E-07 | 7.04414 |
| 201506_at | TGFBI | transforming growth factor, beta-induced, 68kDa | 1.22E-06 | 7.03729 |
| 41512_at | BRAP | BRCA1 associated protein | 1.55E-07 | 7.03712 |
| 211998_at | H3F3B | H3 histone, family 3B (H3.3B) | 6.79E-09 | 7.03691 |
| 219933_at | GLRX2 | glutaredoxin 2 | 9.04E-09 | 7.03255 |
| 218253_s_at | LGTN | ligatin | 4.63E-06 | 7.03218 |
| 208652_at | PPP2CA | protein phosphatase 2, catalytic subunit, alpha isozyme | 7.97E-08 | 7.03191 |
| 218796_at | FERMT1 | fermitin family homolog 1 (Drosophila) | 2.31E-07 | 7.03158 |
| 224511_s_at | TXNDC17 | thioredoxin domain containing 17 | 8.21E-10 | 7.03132 |
| 218564_at | RFWD3 | ring finger and WD repeat domain 3 | 6.00E-06 | 7.02987 |
| 211964_at | COL4A2 | collagen, type IV, alpha 2 | 6.33E-07 | 7.02963 |
| 214988_s_at | SON | SON DNA binding protein | 4.27E-06 | 7.02911 |
| 209464_at | AURKB | aurora kinase B | 1.29E-07 | 7.027 |
| 218911_at | YEATS4 | YEATS domain containing 4 | 1.73E-05 | 7.02624 |
| 215084_s_at | LRRC42 | leucine rich repeat containing 42 | 1.92E-07 | 7.02622 |
| 202302_s_at | RSRC2 | arginine/serine-rich coiled-coil 2 | 1.28E-06 | 7.026 |
| 226895_at | NFIC | Nuclear factor I/C (CCAAT-binding transcription factor) | 9.35E-08 | 7.02522 |
| 204286_s_at | PMAIP1 | phorbol-12-myristate-13-acetate-induced protein 1 | 9.26E-08 | 7.02022 |
| 243492_at | THEM4 | thioesterase superfamily member 4 | 4.58E-07 | 7.01697 |
| 200946_x_at | GLUD1 | glutamate dehydrogenase 1 | 0.0001252 | 7.01604 |
| 224714_at | MKI67IP | MKI67 (FHA domain) interacting nucleolar phosphoprotein | 4.28E-08 | 7.01594 |
| 214794_at | PA2G4 | proliferation-associated 2G4, 38kDa | 4.86E-07 | 7.0157 |
| 204162_at | NDC80 | NDC80 homolog, kinetochore complex component (S. cerevisiae) | 5.39E-06 | 7.01407 |
| 212139_at | GCN1L1 | GCN1 general control of amino-acid synthesis 1-like 1 (yeast) | 1.30E-05 | 7.01261 |
| 227027_at | GFPT1 | glutamine--fructose-6-phosphate transaminase 1 | 8.64E-06 | 7.01138 |
| 217127_at | CTH | cystathionase (cystathionine gamma-lyase) | 2.37E-06 | 7.01041 |
| 202633_at | TOPBP1 | topoisomerase (DNA) II binding protein 1 | 8.06E-07 | 7.00614 |
| 205196_s_at | AP1S1 | adaptor-related protein complex 1, sigma 1 subunit | 1.19E-07 | 7.00226 |
| 212708_at | MSL1 | male-specific lethal 1 homolog (Drosophila) | 2.17E-08 | 7.00158 |
| 213027_at | TROVE2 | TROVE domain family, member 2 | 3.18E-07 | 7.00137 |
| 224252_s_at | FXYD5 | FXYD domain containing ion transport regulator 5 | 2.27E-07 | 6.99946 |
| 226886_at | GFPT1 | glutamine--fructose-6-phosphate transaminase 1 | 1.80E-05 | 6.99742 |
| 201177_s_at | UBA2 | ubiquitin-like modifier activating enzyme 2 | 2.12E-05 | 6.99668 |
| 222204_s_at | RRN3 | RRN3 RNA polymerase I transcription factor homolog (S. cerevisiae) | 1.04E-06 | 6.99601 |
| 201713_s_at | RANBP2 | RAN binding protein 2 | 6.36E-06 | 6.99234 |
| 212650_at | EHBP1 | EH domain binding protein 1 | 6.19E-06 | 6.98491 |
| 225969_at | ALKBH6 | alkB, alkylation repair homolog 6 (E. coli) | 1.96E-08 | 6.9812 |
| 208986_at | TCF12 | transcription factor 12 | 1.66E-08 | 6.97962 |
| 223687_s_at | LY6K | lymphocyte antigen 6 complex, locus K | 7.86E-06 | 6.97267 |
| 200730_s_at | PTP4A1 | protein tyrosine phosphatase type IVA, member 1 | 1.24E-07 | 6.97042 |
| 209624_s_at | MCCC2 | methylcrotonoyl-CoA carboxylase 2 (beta) | 1.77E-07 | 6.96789 |
| 212113_at | ATXN7L3B | ataxin 7-like 3B | 1.43E-05 | 6.96504 |
| 225081_s_at | CDCA7L | cell division cycle associated 7-like | 7.45E-09 | 6.96435 |
| 215931_s_at | ARFGEF2 | ADP-ribosylation factor guanine nucleotide-exchange factor 2 (brefeldin A-inhibi | 7.70E-07 | 6.96426 |
| 222832_s_at | MFF | mitochondrial fission factor | 1.84E-06 | 6.96409 |
| 201066_at | CYC1 | cytochrome c-1 | 1.13E-06 | 6.95751 |
| 201742_x_at | SFRS1 | splicing factor, arginine/serine-rich 1 | 4.47E-05 | 6.95617 |
| 219818_s_at | GPATCH1 | G patch domain containing 1 | 7.24E-08 | 6.9545 |
| 205761_s_at | DUS4L | dihydrouridine synthase 4-like (S. cerevisiae) | 2.88E-05 | 6.95354 |
| 211285_s_at | UBE3A | ubiquitin protein ligase E3A | 3.13E-07 | 6.95292 |
| 216321_s_at | NR3C1 | nuclear receptor subfamily 3, group C, member 1 (glucocorticoid receptor) | 9.73E-07 | 6.95124 |
| 200606_at | DSP | desmoplakin | 1.03E-06 | 6.95054 |
| 219798_s_at | MEPCE | methylphosphate capping enzyme | 4.73E-06 | 6.94655 |
| 228355_s_at | NDUFAF2 | NADH dehydrogenase (ubiquinone) 1 alpha subcomplex, assembly factor 2 | 7.86E-09 | 6.94317 |
| 203694_s_at | DHX16 | DEAH (Asp-Glu-Ala-His) box polypeptide 16 | 2.48E-07 | 6.94316 |
| 212432_at | GRPEL1 | GrpE-like 1, mitochondrial (E. coli) | 1.00E-08 | 6.93949 |
| 233665_x_at | MTO1 | mitochondrial translation optimization 1 homolog (S. cerevisiae) | 1.28E-06 | 6.93895 |
| 210386_s_at | MTX1 | metaxin 1 | 1.04E-07 | 6.93754 |
| 218401_s_at | ZNF281 | zinc finger protein 281 | 3.12E-06 | 6.93443 |
| 201013_s_at | PAICS | phosphoribosylaminoimidazole carboxylase, phosphoribosylaminoimidazole succinoca | 6.72E-10 | 6.93428 |
| 217746_s_at | PDCD6IP | programmed cell death 6 interacting protein | 2.55E-08 | 6.93122 |
| 200848_at | AHCYL1 | adenosylhomocysteinase-like 1 | 2.96E-06 | 6.92812 |
| 212445_s_at | NEDD4L | neural precursor cell expressed, developmentally down-regulated 4-like | 6.79E-10 | 6.92812 |
| 216194_s_at | TBCB | tubulin folding cofactor B | 2.06E-06 | 6.92762 |
| 208936_x_at | LGALS8 | lectin, galactoside-binding, soluble, 8 | 2.48E-07 | 6.91842 |
| 209513_s_at | HSDL2 | hydroxysteroid dehydrogenase like 2 | 2.59E-06 | 6.91773 |
| 202521_at | CTCF | CCCTC-binding factor (zinc finger protein) | 8.15E-08 | 6.91665 |
| 223272_s_at | C1orf57 | chromosome 1 open reading frame 57 | 1.29E-06 | 6.91635 |
| 224467_s_at | PDCD2L | programmed cell death 2-like | 2.18E-06 | 6.90804 |
| 222632_s_at | LZTFL1 | leucine zipper transcription factor-like 1 | 9.52E-07 | 6.90673 |
| 220768_s_at | CSNK1G3 | casein kinase 1, gamma 3 | 2.28E-07 | 6.90529 |
| 217815_at | SUPT16H | suppressor of Ty 16 homolog (S. cerevisiae) | 1.23E-07 | 6.90362 |
| 1053_at | RFC2 | replication factor C (activator 1) 2, 40kDa | 4.14E-07 | 6.90284 |
| 211936_at | HSPA5 | heat shock 70kDa protein 5 (glucose-regulated protein, 78kDa) | 1.11E-06 | 6.90189 |
| 226980_at | DEPDC1B | DEP domain containing 1B | 1.17E-05 | 6.90145 |
| 215780_s_at | LOC642869 /// SET | SET translocation (myeloid leukemia-associated) pseudogene /// SET nuclear oncog | 2.26E-06 | 6.89992 |
| 203319_s_at | ZNF148 | zinc finger protein 148 | 1.78E-07 | 6.89918 |
| 201555_at | MCM3 | minichromosome maintenance complex component 3 | 3.31E-07 | 6.89653 |
| 202542_s_at | AIMP1 | aminoacyl tRNA synthetase complex-interacting multifunctional protein 1 | 2.33E-05 | 6.89497 |
| 223436_s_at | TRPT1 | tRNA phosphotransferase 1 | 3.75E-06 | 6.89333 |
| 209845_at | MKRN1 | makorin ring finger protein 1 | 0.0001172 | 6.89322 |
| 222036_s_at | MCM4 | minichromosome maintenance complex component 4 | 4.66E-08 | 6.89266 |
| 222734_at | WARS2 | tryptophanyl tRNA synthetase 2, mitochondrial | 2.02E-08 | 6.89246 |
| 223076_s_at | NSUN2 | NOP2/Sun domain family, member 2 | 1.58E-06 | 6.89147 |
| 211725_s_at | BID | BH3 interacting domain death agonist | 5.94E-08 | 6.88922 |
| 220789_s_at | TBRG4 | transforming growth factor beta regulator 4 | 9.47E-09 | 6.88768 |
| 219151_s_at | RABL2A /// RABL2B | RAB, member of RAS oncogene family-like 2A /// RAB, member of RAS oncogene famil | 3.62E-07 | 6.88744 |
| 202560_s_at | C1orf77 | chromosome 1 open reading frame 77 | 3.21E-07 | 6.88672 |
| 225649_s_at | STK35 | serine/threonine kinase 35 | 1.75E-06 | 6.88667 |
| 212260_at | GIGYF2 | GRB10 interacting GYF protein 2 | 4.77E-09 | 6.88563 |
| 223227_at | BBS2 | Bardet-Biedl syndrome 2 | 1.02E-06 | 6.88372 |
| 201929_s_at | PKP4 | plakophilin 4 | 3.76E-07 | 6.8814 |
| 229748_x_at | LOC100132288 | hypothetical protein LOC100132288 | 1.78E-08 | 6.88034 |
| 217985_s_at | BAZ1A | bromodomain adjacent to zinc finger domain, 1A | 6.89E-06 | 6.87994 |
| 203746_s_at | HCCS | holocytochrome c synthase | 8.49E-08 | 6.87772 |
| 218305_at | IPO4 | importin 4 | 8.33E-05 | 6.87335 |
| 210933_s_at | FSCN1 | fascin homolog 1, actin-bundling protein (Strongylocentrotus purpuratus) | 5.82E-06 | 6.86959 |
| 200045_at | ABCF1 | ATP-binding cassette, sub-family F (GCN20), member 1 | 6.44E-09 | 6.86646 |
| 202386_s_at | KIAA0430 | KIAA0430 | 2.60E-08 | 6.86546 |
| 213126_at | MED8 | mediator complex subunit 8 | 3.46E-06 | 6.86501 |
| 227698_s_at | RAB40C | RAB40C, member RAS oncogene family | 3.00E-05 | 6.86489 |
| 212596_s_at | HMGXB4 | HMG box domain containing 4 | 3.71E-06 | 6.86348 |
| 212730_at | SYNM | synemin, intermediate filament protein | 2.43E-10 | 6.86242 |
| 203820_s_at | IGF2BP3 | insulin-like growth factor 2 mRNA binding protein 3 | 5.96E-06 | 6.8618 |
| 217873_at | CAB39 | calcium binding protein 39 | 3.29E-08 | 6.86134 |
| 201113_at | TUFM | Tu translation elongation factor, mitochondrial | 1.06E-08 | 6.86103 |
| 222433_at | ENAH | enabled homolog (Drosophila) | 1.59E-07 | 6.85766 |
| 202915_s_at | FAM20B | family with sequence similarity 20, member B | 2.74E-07 | 6.85678 |
| 226027_at | C9orf119 | chromosome 9 open reading frame 119 | 5.56E-07 | 6.84692 |
| 205129_at | NPM3 | nucleophosmin/nucleoplasmin 3 | 9.87E-07 | 6.8451 |
| 215273_s_at | TADA3 | transcriptional adaptor 3 | 5.10E-07 | 6.84384 |
| 201456_s_at | BUB3 | budding uninhibited by benzimidazoles 3 homolog (yeast) | 3.96E-06 | 6.84373 |
| 212514_x_at | DDX3X | DEAD (Asp-Glu-Ala-Asp) box polypeptide 3, X-linked | 5.91E-07 | 6.84329 |
| 211969_at | HSP90AA1 | heat shock protein 90kDa alpha (cytosolic), class A member 1 | 1.81E-06 | 6.84164 |
| 218399_s_at | CDCA4 | cell division cycle associated 4 | 3.93E-06 | 6.83782 |
| 210573_s_at | POLR3C | polymerase (RNA) III (DNA directed) polypeptide C (62kD) | 1.62E-06 | 6.83648 |
| 215136_s_at | EXOSC8 | exosome component 8 | 2.81E-07 | 6.8346 |
| 215438_x_at | GSPT1 | G1 to S phase transition 1 | 1.32E-06 | 6.83144 |
| 218256_s_at | NUP54 | nucleoporin 54kDa | 1.62E-05 | 6.83092 |
| 202385_s_at | TCOF1 | Treacher Collins-Franceschetti syndrome 1 | 2.33E-07 | 6.83005 |
| 219244_s_at | MRPL46 | mitochondrial ribosomal protein L46 | 2.06E-08 | 6.82841 |
| 203252_at | CDK2AP2 | cyclin-dependent kinase 2 associated protein 2 | 1.06E-06 | 6.8275 |
| 229120_s_at | CDC42SE1 | CDC42 small effector 1 | 1.47E-06 | 6.82587 |
| 203565_s_at | MNAT1 | menage a trois homolog 1, cyclin H assembly factor (Xenopus laevis) | 7.95E-06 | 6.82575 |
| 225153_at | GFM1 | G elongation factor, mitochondrial 1 | 1.90E-05 | 6.82294 |
| 208820_at | PTK2 | PTK2 protein tyrosine kinase 2 | 1.68E-07 | 6.81954 |
| 200898_s_at | MGEA5 | meningioma expressed antigen 5 (hyaluronidase) | 1.10E-07 | 6.81928 |
| 203897_at | LYRM1 | LYR motif containing 1 | 1.45E-06 | 6.81915 |
| 223993_s_at | CNIH4 | cornichon homolog 4 (Drosophila) | 2.34E-09 | 6.81878 |
| 216457_s_at | SF3A1 | splicing factor 3a, subunit 1, 120kDa | 3.02E-07 | 6.81269 |
| 1554768_a_at | MAD2L1 | MAD2 mitotic arrest deficient-like 1 (yeast) | 2.91E-07 | 6.81257 |
| 202675_at | SDHB | succinate dehydrogenase complex, subunit B, iron sulfur (Ip) | 2.37E-06 | 6.81248 |
| 212251_at | MTDH | metadherin | 4.34E-06 | 6.81218 |
| 209630_s_at | FBXW2 | F-box and WD repeat domain containing 2 | 1.74E-05 | 6.81119 |
| 225898_at | WDR54 | WD repeat domain 54 | 0.0002122 | 6.80952 |
| 202759_s_at | AKAP2 /// PALM2-AKAP2 | A kinase (PRKA) anchor protein 2 /// PALM2-AKAP2 readthrough | 1.70E-07 | 6.80645 |
| 239042_at | TSR1 | TSR1, 20S rRNA accumulation, homolog (S. cerevisiae) | 2.07E-08 | 6.80618 |
| 218567_x_at | DPP3 | dipeptidyl-peptidase 3 | 4.96E-07 | 6.80594 |
| 200079_s_at | KARS | lysyl-tRNA synthetase | 4.04E-09 | 6.80266 |
| 218120_s_at | HMOX2 | heme oxygenase (decycling) 2 | 9.51E-07 | 6.80001 |
| 203466_at | MPV17 | MpV17 mitochondrial inner membrane protein | 2.60E-05 | 6.79188 |
| 218051_s_at | NT5DC2 | 5'-nucleotidase domain containing 2 | 2.91E-07 | 6.79065 |
| 204089_x_at | MAP3K4 | mitogen-activated protein kinase kinase kinase 4 | 9.01E-11 | 6.78694 |
| 218643_s_at | CRIPT | cysteine-rich PDZ-binding protein | 1.19E-06 | 6.78621 |
| 226744_at | METT10D | methyltransferase 10 domain containing | 1.24E-07 | 6.78604 |
| 221566_s_at | NOL3 | nucleolar protein 3 (apoptosis repressor with CARD domain) | 3.01E-07 | 6.78379 |
| 201772_at | AZIN1 | antizyme inhibitor 1 | 3.75E-06 | 6.78377 |
| 210621_s_at | RASA1 | RAS p21 protein activator (GTPase activating protein) 1 | 2.75E-07 | 6.78278 |
| 233587_s_at | SIPA1L2 | signal-induced proliferation-associated 1 like 2 | 8.20E-09 | 6.782 |
| 201166_s_at | PUM1 | pumilio homolog 1 (Drosophila) | 6.80E-07 | 6.78187 |
| 228009_x_at | ZNRD1 | zinc ribbon domain containing 1 | 4.57E-06 | 6.7817 |
| 219944_at | CLIP4 | CAP-GLY domain containing linker protein family, member 4 | 3.43E-05 | 6.77593 |
| 201394_s_at | RBM5 | RNA binding motif protein 5 | 1.31E-07 | 6.77563 |
| 1558028_x_at | LOC647979 | hypothetical LOC647979 | 1.54E-05 | 6.77075 |
| 209916_at | DHTKD1 | dehydrogenase E1 and transketolase domain containing 1 | 4.06E-08 | 6.76748 |
| 218356_at | FTSJ2 | FtsJ homolog 2 (E. coli) | 3.19E-05 | 6.76646 |
| 201573_s_at | ETF1 | eukaryotic translation termination factor 1 | 6.04E-08 | 6.76387 |
| 218827_s_at | CEP192 | centrosomal protein 192kDa | 7.49E-06 | 6.76367 |
| 206245_s_at | IVNS1ABP | influenza virus NS1A binding protein | 8.18E-06 | 6.76313 |
| 203379_at | RPS6KA1 | ribosomal protein S6 kinase, 90kDa, polypeptide 1 | 5.71E-06 | 6.76245 |
| 202860_at | DENND4B | DENN/MADD domain containing 4B | 2.95E-07 | 6.76222 |
| 208190_s_at | LSR | lipolysis stimulated lipoprotein receptor | 7.19E-07 | 6.76187 |
| 213612_x_at | NBPF10 /// NBPF15 /// NBPF16 /// NBPF8 /// NBPF9 | neuroblastoma breakpoint family, member 10 /// neuroblastoma breakpoint family, | 4.33E-11 | 6.75981 |
| 1555814_a_at | RHOA | ras homolog gene family, member A | 0.0001536 | 6.7578 |
| 224898_at | WDR26 | WD repeat domain 26 | 5.24E-06 | 6.75315 |
| 204254_s_at | VDR | vitamin D (1,25- dihydroxyvitamin D3) receptor | 2.09E-07 | 6.75168 |
| 215722_s_at | SNRPA1 | small nuclear ribonucleoprotein polypeptide A' | 1.12E-06 | 6.75069 |
| 221500_s_at | STX16 | syntaxin 16 | 2.49E-09 | 6.74948 |
| 201853_s_at | CDC25B | cell division cycle 25 homolog B (S. pombe) | 1.01E-05 | 6.74931 |
| 201099_at | USP9X | ubiquitin specific peptidase 9, X-linked | 5.09E-07 | 6.74693 |
| 226976_at | KPNA6 | karyopherin alpha 6 (importin alpha 7) | 1.89E-06 | 6.74673 |
| 223098_s_at | LONP2 | lon peptidase 2, peroxisomal | 6.76E-07 | 6.74645 |
| 207760_s_at | NCOR2 | nuclear receptor co-repressor 2 | 7.60E-07 | 6.74552 |
| 219123_at | ZNF232 | zinc finger protein 232 | 5.68E-09 | 6.74313 |
| 208117_s_at | LAS1L | LAS1-like (S. cerevisiae) | 1.84E-06 | 6.74248 |
| 226921_at | UBR1 | ubiquitin protein ligase E3 component n-recognin 1 | 4.56E-07 | 6.74198 |
| 218505_at | WDR59 | WD repeat domain 59 | 1.21E-05 | 6.74106 |
| 202119_s_at | CPNE3 | copine III | 8.80E-07 | 6.73971 |
| 212627_s_at | EXOSC7 | exosome component 7 | 1.01E-06 | 6.73797 |
| 202464_s_at | PFKFB3 | 6-phosphofructo-2-kinase/fructose-2,6-biphosphatase 3 | 5.46E-06 | 6.73624 |
| 219507_at | RSRC1 | arginine/serine-rich coiled-coil 1 | 7.15E-06 | 6.73122 |
| 231772_x_at | CENPH | centromere protein H | 1.30E-09 | 6.72764 |
| 222701_s_at | CHCHD7 | coiled-coil-helix-coiled-coil-helix domain containing 7 | 1.84E-07 | 6.72674 |
| 218625_at | NRN1 | neuritin 1 | 3.29E-06 | 6.7241 |
| 224068_x_at | RBM22 | RNA binding motif protein 22 | 5.78E-07 | 6.72409 |
| 201150_s_at | TIMP3 | TIMP metallopeptidase inhibitor 3 | 4.61E-06 | 6.72402 |
| 213037_x_at | STAU1 | staufen, RNA binding protein, homolog 1 (Drosophila) | 9.54E-09 | 6.72346 |
| 227607_at | STAMBPL1 | STAM binding protein-like 1 | 1.57E-06 | 6.71908 |
| 1555630_a_at | RAB34 | RAB34, member RAS oncogene family | 1.82E-06 | 6.7182 |
| 229069_at | SARNP | SAP domain containing ribonucleoprotein | 2.32E-06 | 6.71791 |
| 209609_s_at | MRPL9 | mitochondrial ribosomal protein L9 | 1.08E-07 | 6.71782 |
| 213024_at | TMF1 | TATA element modulatory factor 1 | 6.06E-05 | 6.71704 |
| 230535_s_at | ATP5E | ATP synthase, H+ transporting, mitochondrial F1 complex, epsilon subunit | 1.17E-05 | 6.71024 |
| 225195_at | DPH3 | DPH3, KTI11 homolog (S. cerevisiae) | 1.44E-06 | 6.70663 |
| 225562_at | RASA3 | RAS p21 protein activator 3 | 7.62E-09 | 6.70362 |
| 202820_at | AHR | aryl hydrocarbon receptor | 8.32E-06 | 6.70316 |
| 202408_s_at | PRPF31 | PRP31 pre-mRNA processing factor 31 homolog (S. cerevisiae) | 7.08E-09 | 6.6991 |
| 225203_at | PPP1R16A | protein phosphatase 1, regulatory (inhibitor) subunit 16A | 7.18E-06 | 6.69863 |
| 202905_x_at | NBN | nibrin | 1.05E-05 | 6.69794 |
| 214507_s_at | EXOSC2 | exosome component 2 | 1.43E-08 | 6.69738 |
| 211967_at | TMEM123 | transmembrane protein 123 | 8.20E-07 | 6.69728 |
| 202906_s_at | NBN | nibrin | 1.69E-07 | 6.69339 |
| 210236_at | PPFIA1 | protein tyrosine phosphatase, receptor type, f polypeptide (PTPRF), interacting | 2.72E-06 | 6.68795 |
| 223048_at | SDHAF2 | succinate dehydrogenase complex assembly factor 2 | 1.52E-05 | 6.68782 |
| 74694_s_at | RABEP2 | rabaptin, RAB GTPase binding effector protein 2 | 2.61E-07 | 6.6851 |
| 202461_at | EIF2B2 | eukaryotic translation initiation factor 2B, subunit 2 beta, 39kDa | 9.51E-10 | 6.68405 |
| 227527_at | MLL2 | myeloid/lymphoid or mixed-lineage leukemia 2 | 7.55E-08 | 6.68219 |
| 208710_s_at | AP3D1 | adaptor-related protein complex 3, delta 1 subunit | 7.73E-07 | 6.6818 |
| 231995_at | C9orf82 | chromosome 9 open reading frame 82 | 3.08E-06 | 6.67872 |
| 209744_x_at | ITCH | itchy E3 ubiquitin protein ligase homolog (mouse) | 3.26E-07 | 6.67819 |
| 201519_at | TOMM70A | translocase of outer mitochondrial membrane 70 homolog A (S. cerevisiae) | 8.44E-07 | 6.67389 |
| 206875_s_at | SLK | STE20-like kinase (yeast) | 5.45E-06 | 6.67319 |
| 222664_at | KCTD15 | potassium channel tetramerisation domain containing 15 | 4.08E-09 | 6.67247 |
| 209063_x_at | PAIP1 | poly(A) binding protein interacting protein 1 | 2.69E-05 | 6.67065 |
| 212540_at | CDC34 | cell division cycle 34 homolog (S. cerevisiae) | 8.58E-08 | 6.66701 |
| 225251_at | RAB24 | RAB24, member RAS oncogene family | 2.92E-06 | 6.66666 |
| 226234_at | GDF11 | growth differentiation factor 11 | 5.59E-08 | 6.66589 |
| 218035_s_at | RBM47 | RNA binding motif protein 47 | 7.85E-09 | 6.66571 |
| 202696_at | OXSR1 | oxidative-stress responsive 1 | 1.21E-08 | 6.66558 |
| 201112_s_at | CSE1L | CSE1 chromosome segregation 1-like (yeast) | 8.91E-07 | 6.66482 |
| 205184_at | GNG4 | guanine nucleotide binding protein (G protein), gamma 4 | 4.66E-06 | 6.66439 |
| 212228_s_at | COQ9 | coenzyme Q9 homolog (S. cerevisiae) | 1.74E-06 | 6.66417 |
| 221685_s_at | CCDC99 | coiled-coil domain containing 99 | 6.11E-08 | 6.66206 |
| 217736_s_at | EIF2AK1 | eukaryotic translation initiation factor 2-alpha kinase 1 | 3.42E-07 | 6.65826 |
| 235056_at | ETV6 | ets variant 6 | 3.21E-05 | 6.65754 |
| 200001_at | CAPNS1 | calpain, small subunit 1 | 3.56E-06 | 6.65729 |
| 205061_s_at | EXOSC9 | exosome component 9 | 9.57E-08 | 6.65603 |
| 227418_at | KIAA1826 | KIAA1826 | 5.76E-09 | 6.65309 |
| 224753_at | CDCA5 | cell division cycle associated 5 | 4.22E-08 | 6.65301 |
| 225614_at | SAAL1 | serum amyloid A-like 1 | 4.44E-05 | 6.6522 |
| 201521_s_at | NCBP2 | nuclear cap binding protein subunit 2, 20kDa | 1.32E-06 | 6.65151 |
| 201727_s_at | ELAVL1 | ELAV (embryonic lethal, abnormal vision, Drosophila)-like 1 (Hu antigen R) | 4.00E-07 | 6.64831 |
| 219007_at | NUP43 | nucleoporin 43kDa | 6.81E-07 | 6.64669 |
| 225170_at | WDR5 | WD repeat domain 5 | 6.08E-08 | 6.64629 |
| 221265_s_at | C15orf44 | chromosome 15 open reading frame 44 | 7.41E-06 | 6.64621 |
| 211270_x_at | PTBP1 | polypyrimidine tract binding protein 1 | 1.61E-10 | 6.64448 |
| 202068_s_at | LDLR | low density lipoprotein receptor | 8.69E-08 | 6.64341 |
| 227833_s_at | MBD6 | methyl-CpG binding domain protein 6 | 9.67E-06 | 6.64193 |
| 201516_at | SRM | spermidine synthase | 1.24E-06 | 6.6389 |
| 200597_at | EIF3A | eukaryotic translation initiation factor 3, subunit A | 9.59E-07 | 6.63775 |
| 222826_at | PLDN | pallidin homolog (mouse) | 1.44E-05 | 6.63718 |
| 223108_s_at | ZCCHC17 | zinc finger, CCHC domain containing 17 | 5.15E-09 | 6.62966 |
| 202528_at | GALE | UDP-galactose-4-epimerase | 4.29E-05 | 6.62726 |
| 225522_at | AAK1 | AP2 associated kinase 1 | 6.59E-07 | 6.62716 |
| 211928_at | DYNC1H1 | dynein, cytoplasmic 1, heavy chain 1 | 6.47E-09 | 6.62485 |
| 225375_at | TMEM199 | transmembrane protein 199 | 4.77E-06 | 6.62231 |
| 219449_s_at | TMEM70 | transmembrane protein 70 | 2.47E-09 | 6.6209 |
| 225472_at | BAT4 | HLA-B associated transcript 4 | 1.38E-05 | 6.61614 |
| 204839_at | POP5 | processing of precursor 5, ribonuclease P/MRP subunit (S. cerevisiae) | 1.01E-06 | 6.61216 |
| 204544_at | HPS5 | Hermansky-Pudlak syndrome 5 | 2.70E-05 | 6.61192 |
| 209286_at | CDC42EP3 | CDC42 effector protein (Rho GTPase binding) 3 | 1.03E-06 | 6.61171 |
| 209262_s_at | NR2F6 | nuclear receptor subfamily 2, group F, member 6 | 2.52E-07 | 6.61147 |
| 209120_at | NR2F2 | nuclear receptor subfamily 2, group F, member 2 | 2.04E-05 | 6.60875 |
| 229538_s_at | IQGAP3 | IQ motif containing GTPase activating protein 3 | 2.92E-05 | 6.60787 |
| 200691_s_at | HSPA9 | heat shock 70kDa protein 9 (mortalin) | 5.00E-07 | 6.60437 |
| 225818_s_at | TBRG1 | transforming growth factor beta regulator 1 | 3.21E-07 | 6.60279 |
| 209212_s_at | KLF5 | Kruppel-like factor 5 (intestinal) | 9.52E-08 | 6.60032 |
| 206989_s_at | SFRS2IP | splicing factor, arginine/serine-rich 2, interacting protein | 1.77E-05 | 6.59973 |
| 228384_s_at | PYROXD2 | pyridine nucleotide-disulphide oxidoreductase domain 2 | 3.99E-05 | 6.59838 |
| 215165_x_at | UMPS | uridine monophosphate synthetase | 3.93E-08 | 6.59188 |
| 212943_at | KIAA0528 | KIAA0528 | 2.75E-06 | 6.59093 |
| 225024_at | RPRD1B | regulation of nuclear pre-mRNA domain containing 1B | 2.29E-06 | 6.59033 |
| 201263_at | TARS | threonyl-tRNA synthetase | 7.86E-08 | 6.58884 |
| 227561_at | DDR2 | discoidin domain receptor tyrosine kinase 2 | 8.41E-07 | 6.58866 |
| 209461_x_at | WDR18 | WD repeat domain 18 | 3.04E-06 | 6.58427 |
| 209527_at | EXOSC2 | exosome component 2 | 1.05E-06 | 6.58166 |
| 207622_s_at | ABCF2 | ATP-binding cassette, sub-family F (GCN20), member 2 | 2.10E-08 | 6.57969 |
| 204351_at | S100P | S100 calcium binding protein P | 1.28E-05 | 6.57931 |
| 201042_at | TGM2 | transglutaminase 2 (C polypeptide, protein-glutamine-gamma-glutamyltransferase) | 3.00E-08 | 6.57592 |
| 217840_at | DDX41 | DEAD (Asp-Glu-Ala-Asp) box polypeptide 41 | 3.85E-05 | 6.5747 |
| 1554441_a_at | WAPAL | wings apart-like homolog (Drosophila) | 4.38E-07 | 6.57404 |
| 202151_s_at | UBAC1 | UBA domain containing 1 | 3.44E-09 | 6.5726 |
| 209479_at | CCDC28A | coiled-coil domain containing 28A | 3.83E-08 | 6.57069 |
| 201143_s_at | EIF2S1 | eukaryotic translation initiation factor 2, subunit 1 alpha, 35kDa | 3.47E-07 | 6.56878 |
| 221825_at | ANGEL2 | angel homolog 2 (Drosophila) | 3.60E-07 | 6.56815 |
| 218549_s_at | FAM82B | family with sequence similarity 82, member B | 2.78E-05 | 6.5679 |
| 202536_at | CHMP2B | chromatin modifying protein 2B | 9.08E-06 | 6.56292 |
| 212832_s_at | CKAP5 | cytoskeleton associated protein 5 | 8.31E-07 | 6.56285 |
| 223530_at | TDRKH | tudor and KH domain containing | 2.70E-06 | 6.562 |
| 224802_at | NDFIP2 | Nedd4 family interacting protein 2 | 0.0001161 | 6.56198 |
| 218894_s_at | MAGOHB | mago-nashi homolog B (Drosophila) | 4.97E-07 | 6.56194 |
| 204093_at | CCNH | cyclin H | 3.63E-06 | 6.55806 |
| 201043_s_at | ANP32A | acidic (leucine-rich) nuclear phosphoprotein 32 family, member A | 1.27E-05 | 6.55721 |
| 206116_s_at | TPM1 | tropomyosin 1 (alpha) | 6.21E-07 | 6.54796 |
| 224232_s_at | PRELID1 | PRELI domain containing 1 | 1.39E-08 | 6.54297 |
| 223003_at | C19orf43 | chromosome 19 open reading frame 43 | 1.21E-06 | 6.54187 |
| 217906_at | KLHDC2 | kelch domain containing 2 | 3.64E-07 | 6.54187 |
| 213287_s_at | KRT10 | keratin 10 | 6.52E-06 | 6.54102 |
| 225114_at | AGPS | alkylglycerone phosphate synthase | 1.94E-05 | 6.54056 |
| 212906_at | GRAMD1B | GRAM domain containing 1B | 5.08E-07 | 6.54043 |
| 200615_s_at | AP2B1 | adaptor-related protein complex 2, beta 1 subunit | 2.27E-09 | 6.53987 |
| 227351_at | C16orf52 | chromosome 16 open reading frame 52 | 7.67E-06 | 6.53813 |
| 201546_at | TRIP12 | thyroid hormone receptor interactor 12 | 2.05E-07 | 6.53325 |
| 201222_s_at | RAD23B | RAD23 homolog B (S. cerevisiae) | 2.81E-06 | 6.52747 |
| 201351_s_at | YME1L1 | YME1-like 1 (S. cerevisiae) | 4.12E-06 | 6.52619 |
| 218339_at | MRPL22 | mitochondrial ribosomal protein L22 | 1.45E-08 | 6.52153 |
| 228266_s_at | HDGFRP3 | hepatoma-derived growth factor, related protein 3 | 1.06E-05 | 6.518 |
| 205130_at | RAGE | renal tumor antigen | 2.87E-05 | 6.5134 |
| 225051_at | EPB41 | erythrocyte membrane protein band 4.1 (elliptocytosis 1, RH-linked) | 2.64E-06 | 6.51157 |
| 203409_at | DDB2 | damage-specific DNA binding protein 2, 48kDa | 3.94E-07 | 6.51128 |
| 235603_at | HNRNPU | heterogeneous nuclear ribonucleoprotein U (scaffold attachment factor A) | 4.89E-06 | 6.51042 |
| 223389_s_at | ZNF581 | zinc finger protein 581 | 1.30E-05 | 6.50785 |
| 226434_at | C7orf47 | chromosome 7 open reading frame 47 | 6.42E-06 | 6.50647 |
| 203403_s_at | RNF6 | ring finger protein (C3H2C3 type) 6 | 6.56E-06 | 6.50601 |
| 209252_at | HARS2 | histidyl-tRNA synthetase 2, mitochondrial (putative) | 9.88E-08 | 6.50276 |
| 231131_at | FAM133A | family with sequence similarity 133, member A | 3.24E-06 | 6.49805 |
| 218134_s_at | RBM22 | RNA binding motif protein 22 | 5.39E-06 | 6.49492 |
| 224830_at | NUDT21 | nudix (nucleoside diphosphate linked moiety X)-type motif 21 | 7.97E-06 | 6.4946 |
| 221771_s_at | MPHOSPH8 | M-phase phosphoprotein 8 | 7.32E-06 | 6.49316 |
| 224569_s_at | IRF2BP2 | interferon regulatory factor 2 binding protein 2 | 3.78E-05 | 6.48608 |
| 209799_at | PRKAA1 | protein kinase, AMP-activated, alpha 1 catalytic subunit | 8.56E-07 | 6.4827 |
| 235260_s_at | PACRGL | PARK2 co-regulated-like | 1.47E-06 | 6.48058 |
| 200605_s_at | PRKAR1A | protein kinase, cAMP-dependent, regulatory, type I, alpha (tissue specific extin | 5.72E-06 | 6.47434 |
| 203569_s_at | OFD1 | oral-facial-digital syndrome 1 | 1.45E-06 | 6.47383 |
| 208861_s_at | ATRX | alpha thalassemia/mental retardation syndrome X-linked (RAD54 homolog, S. cerevi | 7.70E-09 | 6.47304 |
| 1555831_s_at | LRRC41 | leucine rich repeat containing 41 | 3.27E-07 | 6.4698 |
| 222395_s_at | UBE2Z | ubiquitin-conjugating enzyme E2Z | 1.85E-06 | 6.46606 |
| 225640_at | LOC401504 | hypothetical gene supported by AK091718 | 2.16E-06 | 6.46508 |
| 201156_s_at | RAB5C | RAB5C, member RAS oncogene family | 3.08E-08 | 6.46219 |
| 202084_s_at | SEC14L1 | SEC14-like 1 (S. cerevisiae) | 4.25E-07 | 6.46216 |
| 201359_at | COPB1 | coatomer protein complex, subunit beta 1 | 1.24E-05 | 6.45816 |
| 225173_at | ARHGAP18 | Rho GTPase activating protein 18 | 3.12E-07 | 6.45711 |
| 218740_s_at | CDK5RAP3 | CDK5 regulatory subunit associated protein 3 | 3.60E-07 | 6.45452 |
| 209620_s_at | ABCB7 | ATP-binding cassette, sub-family B (MDR/TAP), member 7 | 1.13E-07 | 6.45408 |
| 211026_s_at | MGLL | monoglyceride lipase | 3.28E-06 | 6.4529 |
| 202743_at | PIK3R3 | phosphoinositide-3-kinase, regulatory subunit 3 (gamma) | 5.60E-07 | 6.45179 |
| 202731_at | PDCD4 | programmed cell death 4 (neoplastic transformation inhibitor) | 5.93E-06 | 6.45042 |
| 223308_s_at | WDR5 | WD repeat domain 5 | 1.86E-08 | 6.45042 |
| 212759_s_at | TCF7L2 | transcription factor 7-like 2 (T-cell specific, HMG-box) | 1.02E-05 | 6.44963 |
| 212782_x_at | POLR2J | polymerase (RNA) II (DNA directed) polypeptide J, 13.3kDa | 1.43E-06 | 6.44948 |
| 223808_s_at | PTPMT1 | protein tyrosine phosphatase, mitochondrial 1 | 3.46E-06 | 6.44462 |
| 1553672_at | ENAH | enabled homolog (Drosophila) | 2.27E-07 | 6.44289 |
| 208673_s_at | SFRS3 | splicing factor, arginine/serine-rich 3 | 7.75E-06 | 6.44134 |
| 210793_s_at | NUP98 | nucleoporin 98kDa | 2.28E-06 | 6.44127 |
| 210910_s_at | POMZP3 | POM (POM121 homolog, rat) and ZP3 fusion | 2.45E-05 | 6.44037 |
| 203624_at | SFRS17A | splicing factor, arginine/serine-rich 17A | 7.84E-06 | 6.43769 |
| 1553099_at | TIGD1 | tigger transposable element derived 1 | 2.16E-07 | 6.43678 |
| 202720_at | TES | testis derived transcript (3 LIM domains) | 1.33E-06 | 6.43673 |
| 201715_s_at | ACIN1 | apoptotic chromatin condensation inducer 1 | 1.71E-05 | 6.43633 |
| 200054_at | ZNF259 | zinc finger protein 259 | 7.66E-06 | 6.43521 |
| 203356_at | CAPN7 | calpain 7 | 3.68E-06 | 6.43473 |
| 219350_s_at | DIABLO | diablo homolog (Drosophila) | 2.43E-07 | 6.43369 |
| 210093_s_at | MAGOH | mago-nashi homolog, proliferation-associated (Drosophila) | 1.49E-05 | 6.43361 |
| 212104_s_at | RBM9 | RNA binding motif protein 9 | 2.95E-06 | 6.43351 |
| 210820_x_at | COQ7 | coenzyme Q7 homolog, ubiquinone (yeast) | 1.67E-06 | 6.43338 |
| 228822_s_at | USP16 | ubiquitin specific peptidase 16 | 2.18E-06 | 6.43292 |
| 231059_x_at | SCAND1 | SCAN domain containing 1 | 0.0001596 | 6.43267 |
| 212470_at | SPAG9 | sperm associated antigen 9 | 1.08E-06 | 6.43171 |
| 226520_at | LCOR | ligand dependent nuclear receptor corepressor | 2.23E-05 | 6.42988 |
| 211600_at | PTPRO | protein tyrosine phosphatase, receptor type, O | 5.66E-08 | 6.42287 |
| 217725_x_at | SERBP1 | SERPINE1 mRNA binding protein 1 | 1.24E-05 | 6.41891 |
| 201825_s_at | SCCPDH | saccharopine dehydrogenase (putative) | 1.91E-07 | 6.41693 |
| 218970_s_at | CUTC | cutC copper transporter homolog (E. coli) | 1.16E-05 | 6.41363 |
| 203351_s_at | ORC4L | origin recognition complex, subunit 4-like (yeast) | 7.90E-06 | 6.41229 |
| 218084_x_at | FXYD5 | FXYD domain containing ion transport regulator 5 | 8.21E-07 | 6.41204 |
| 221547_at | PRPF18 | PRP18 pre-mRNA processing factor 18 homolog (S. cerevisiae) | 1.79E-08 | 6.41191 |
| 201303_at | EIF4A3 | eukaryotic translation initiation factor 4A3 | 8.13E-09 | 6.40839 |
| 227205_at | TAF1 | TAF1 RNA polymerase II, TATA box binding protein (TBP)-associated factor, 250kDa | 8.93E-08 | 6.4079 |
| 202605_at | GUSB | glucuronidase, beta | 1.99E-08 | 6.40645 |
| 201571_s_at | DCTD | dCMP deaminase | 1.90E-06 | 6.40564 |
| 208398_s_at | TBPL1 | TBP-like 1 | 3.30E-08 | 6.40416 |
| 202116_at | DPF2 | D4, zinc and double PHD fingers family 2 | 1.38E-06 | 6.40391 |
| 226265_at | QSER1 | glutamine and serine rich 1 | 2.93E-08 | 6.40215 |
| 202799_at | CLPP | ClpP caseinolytic peptidase, ATP-dependent, proteolytic subunit homolog (E. coli | 3.31E-06 | 6.39849 |
| 228217_s_at | PSMG4 | proteasome (prosome, macropain) assembly chaperone 4 | 2.25E-08 | 6.39691 |
| 212894_at | SUPV3L1 | suppressor of var1, 3-like 1 (S. cerevisiae) | 1.49E-07 | 6.39579 |
| 218328_at | COQ4 | coenzyme Q4 homolog (S. cerevisiae) | 2.74E-06 | 6.3952 |
| 212918_at | RECQL | RecQ protein-like (DNA helicase Q1-like) | 5.79E-07 | 6.39208 |
| 204617_s_at | ACD | adrenocortical dysplasia homolog (mouse) | 3.88E-07 | 6.39081 |
| 208021_s_at | RFC1 | replication factor C (activator 1) 1, 145kDa | 1.06E-05 | 6.38919 |
| 221472_at | SERINC3 | serine incorporator 3 | 4.24E-07 | 6.38863 |
| 1569454_a_at | LOC283352 | hypothetical protein LOC283352 | 3.12E-05 | 6.38774 |
| 218716_x_at | MTO1 | mitochondrial translation optimization 1 homolog (S. cerevisiae) | 3.85E-06 | 6.38679 |
| 206095_s_at | SFRS13A | splicing factor, arginine/serine-rich 13A | 8.14E-07 | 6.38676 |
| 223819_x_at | COMMD5 | COMM domain containing 5 | 8.42E-07 | 6.38645 |
| 212993_at | NACC2 | NACC family member 2, BEN and BTB (POZ) domain containing | 1.05E-08 | 6.38547 |
| 200599_s_at | HSP90B1 | heat shock protein 90kDa beta (Grp94), member 1 | 8.14E-07 | 6.38494 |
| 212015_x_at | PTBP1 | polypyrimidine tract binding protein 1 | 1.39E-07 | 6.38489 |
| 218737_at | SBNO1 | strawberry notch homolog 1 (Drosophila) | 2.04E-06 | 6.38304 |
| 219459_at | POLR3B | polymerase (RNA) III (DNA directed) polypeptide B | 3.18E-07 | 6.37989 |
| 207630_s_at | CREM | cAMP responsive element modulator | 8.03E-10 | 6.37755 |
| 209393_s_at | EIF4E2 | eukaryotic translation initiation factor 4E family member 2 | 6.51E-06 | 6.37316 |
| 220477_s_at | C20orf30 | chromosome 20 open reading frame 30 | 2.28E-06 | 6.37146 |
| 224367_at | BEX2 | brain expressed X-linked 2 | 1.40E-07 | 6.36879 |
| 208447_s_at | PRPS1 | phosphoribosyl pyrophosphate synthetase 1 | 1.89E-09 | 6.36727 |
| 214092_x_at | SFRS14 | splicing factor, arginine/serine-rich 14 | 3.90E-05 | 6.36665 |
| 1553978_at | LOC729991 | hypothetical protein LOC729991 | 3.24E-06 | 6.36596 |
| 208994_s_at | PPIG | peptidylprolyl isomerase G (cyclophilin G) | 4.13E-08 | 6.3636 |
| 217943_s_at | MAP7D1 | MAP7 domain containing 1 | 2.27E-07 | 6.36347 |
| 224535_s_at | MRP63 | mitochondrial ribosomal protein 63 | 1.17E-05 | 6.36344 |
| 224925_at | PREX1 | phosphatidylinositol-3,4,5-trisphosphate-dependent Rac exchange factor 1 | 5.53E-05 | 6.36301 |
| 203644_s_at | MON1B | MON1 homolog B (yeast) | 1.07E-06 | 6.36035 |
| 203145_at | SPAG5 | sperm associated antigen 5 | 1.49E-06 | 6.35913 |
| 213189_at | MINA | MYC induced nuclear antigen | 1.73E-07 | 6.35791 |
| 200967_at | PPIB | peptidylprolyl isomerase B (cyclophilin B) | 8.65E-08 | 6.3579 |
| 209852_x_at | PSME3 | proteasome (prosome, macropain) activator subunit 3 (PA28 gamma; Ki) | 2.08E-07 | 6.35364 |
| 202030_at | BCKDK | branched chain ketoacid dehydrogenase kinase | 3.59E-07 | 6.35331 |
| 219384_s_at | ADAT1 | adenosine deaminase, tRNA-specific 1 | 4.45E-06 | 6.35199 |
| 228129_at | SERBP1 | SERPINE1 mRNA binding protein 1 | 1.70E-05 | 6.34864 |
| 209056_s_at | CDC5L | CDC5 cell division cycle 5-like (S. pombe) | 1.33E-05 | 6.34764 |
| 225642_at | KTI12 | KTI12 homolog, chromatin associated (S. cerevisiae) | 1.90E-07 | 6.34696 |
| 209044_x_at | SF3B4 | splicing factor 3b, subunit 4, 49kDa | 4.87E-06 | 6.34448 |
| 202683_s_at | RNMT | RNA (guanine-7-) methyltransferase | 3.55E-06 | 6.34389 |
| 202034_x_at | RB1CC1 | RB1-inducible coiled-coil 1 | 9.31E-06 | 6.34356 |
| 228378_at | C12orf29 | chromosome 12 open reading frame 29 | 1.26E-06 | 6.34159 |
| 222765_x_at | ESF1 | ESF1, nucleolar pre-rRNA processing protein, homolog (S. cerevisiae) | 2.85E-05 | 6.34067 |
| 203561_at | FCGR2A | Fc fragment of IgG, low affinity IIa, receptor (CD32) | 1.54E-07 | 6.3404 |
| 212630_at | EXOC3 | exocyst complex component 3 | 9.58E-06 | 6.33642 |
| 224855_at | PYCR2 | pyrroline-5-carboxylate reductase family, member 2 | 1.12E-05 | 6.33622 |
| 227220_at | NFXL1 | nuclear transcription factor, X-box binding-like 1 | 9.98E-08 | 6.33459 |
| 1567014_s_at | NFE2L2 | nuclear factor (erythroid-derived 2)-like 2 | 3.00E-06 | 6.33388 |
| 225187_at | KIAA1967 | KIAA1967 | 1.27E-05 | 6.33379 |
| 202622_s_at | ATXN2 | ataxin 2 | 1.30E-06 | 6.33096 |
| 208854_s_at | STK24 | serine/threonine kinase 24 (STE20 homolog, yeast) | 2.17E-08 | 6.33022 |
| 203611_at | TERF2 | telomeric repeat binding factor 2 | 8.56E-08 | 6.32989 |
| 223452_s_at | ATL3 | atlastin GTPase 3 | 6.92E-06 | 6.32811 |
| 218129_s_at | NFYB | nuclear transcription factor Y, beta | 4.28E-05 | 6.32703 |
| 211630_s_at | GSS | glutathione synthetase | 9.51E-09 | 6.32653 |
| 223491_at | COMMD2 | COMM domain containing 2 | 1.02E-06 | 6.32607 |
| 201145_at | HAX1 | HCLS1 associated protein X-1 | 4.64E-08 | 6.32516 |
| 200854_at | NCOR1 | nuclear receptor co-repressor 1 | 6.22E-08 | 6.32103 |
| 212066_s_at | USP34 | ubiquitin specific peptidase 34 | 1.87E-07 | 6.31695 |
| 214073_at | CTTN | cortactin | 5.15E-07 | 6.31342 |
| 225000_at | PRKAR2A | Protein kinase, cAMP-dependent, regulatory, type II, alpha | 2.32E-07 | 6.31184 |
| 224963_at | SLC26A2 | solute carrier family 26 (sulfate transporter), member 2 | 1.27E-07 | 6.30868 |
| 201071_x_at | SF3B1 | splicing factor 3b, subunit 1, 155kDa | 1.24E-08 | 6.30797 |
| 218152_at | HMG20A | high-mobility group 20A | 2.63E-06 | 6.30686 |
| 201073_s_at | SMARCC1 | SWI/SNF related, matrix associated, actin dependent regulator of chromatin, subf | 1.02E-05 | 6.30603 |
| 221827_at | RBCK1 | RanBP-type and C3HC4-type zinc finger containing 1 | 4.88E-07 | 6.30562 |
| 224767_at | RPL37 | Ribosomal protein L37 | 2.53E-06 | 6.30445 |
| 201055_s_at | HNRNPA0 | heterogeneous nuclear ribonucleoprotein A0 | 4.25E-07 | 6.30425 |
| 203447_at | PSMD5 | proteasome (prosome, macropain) 26S subunit, non-ATPase, 5 | 1.60E-06 | 6.30392 |
| 208801_at | SRP72 | signal recognition particle 72kDa | 3.81E-08 | 6.30119 |
| 203427_at | ASF1A | ASF1 anti-silencing function 1 homolog A (S. cerevisiae) | 1.46E-06 | 6.29872 |
| 238076_at | GATAD2B | GATA zinc finger domain containing 2B | 6.60E-06 | 6.29644 |
| 223077_at | TMOD3 | tropomodulin 3 (ubiquitous) | 3.73E-05 | 6.2963 |
| 207186_s_at | BPTF | bromodomain PHD finger transcription factor | 5.14E-09 | 6.2958 |
| 208822_s_at | DAP3 | death associated protein 3 | 4.73E-06 | 6.29481 |
| 218157_x_at | CDC42SE1 | CDC42 small effector 1 | 2.91E-08 | 6.29448 |
| 226015_at | ZNF12 | zinc finger protein 12 | 3.70E-08 | 6.29423 |
| 225289_at | STAT3 | signal transducer and activator of transcription 3 (acute-phase response factor) | 6.85E-07 | 6.29167 |
| 202929_s_at | DDT | D-dopachrome tautomerase | 1.72E-08 | 6.29138 |
| 226352_at | JMY | junction mediating and regulatory protein, p53 cofactor | 6.61E-07 | 6.28952 |
| 200768_s_at | MAT2A | methionine adenosyltransferase II, alpha | 4.66E-07 | 6.28944 |
| 228131_at | ERCC1 | excision repair cross-complementing rodent repair deficiency, complementation gr | 5.28E-08 | 6.28691 |
| 209135_at | ASPH | aspartate beta-hydroxylase | 8.67E-06 | 6.28666 |
| 202007_at | NID1 | nidogen 1 | 4.03E-05 | 6.28233 |
| 213664_at | SLC1A1 | solute carrier family 1 (neuronal/epithelial high affinity glutamate transporter | 1.52E-05 | 6.27934 |
| 202462_s_at | DDX46 | DEAD (Asp-Glu-Ala-Asp) box polypeptide 46 | 2.63E-08 | 6.27528 |
| 221652_s_at | C12orf11 | chromosome 12 open reading frame 11 | 1.28E-06 | 6.27441 |
| 203940_s_at | VASH1 | vasohibin 1 | 3.89E-07 | 6.27264 |
| 222014_x_at | MTO1 | mitochondrial translation optimization 1 homolog (S. cerevisiae) | 0.0001521 | 6.27214 |
| 202455_at | HDAC5 | histone deacetylase 5 | 1.12E-05 | 6.2706 |
| 201434_at | TTC1 | tetratricopeptide repeat domain 1 | 1.48E-09 | 6.26824 |
| 223704_s_at | DMRT2 | doublesex and mab-3 related transcription factor 2 | 4.10E-06 | 6.26664 |
| 205740_s_at | RBM42 | RNA binding motif protein 42 | 3.10E-06 | 6.2663 |
| 202556_s_at | MCRS1 | microspherule protein 1 | 4.60E-08 | 6.26458 |
| 229353_s_at | NUCKS1 | nuclear casein kinase and cyclin-dependent kinase substrate 1 | 2.36E-07 | 6.26347 |
| 218176_at | MAGEF1 | melanoma antigen family F, 1 | 1.66E-08 | 6.26327 |
| 214193_s_at | C1orf107 | chromosome 1 open reading frame 107 | 4.75E-05 | 6.25982 |
| 213029_at | NFIB | nuclear factor I/B | 3.80E-06 | 6.25837 |
| 219373_at | DPM3 | dolichyl-phosphate mannosyltransferase polypeptide 3 | 2.74E-07 | 6.25739 |
| 201192_s_at | PITPNA | phosphatidylinositol transfer protein, alpha | 1.61E-08 | 6.25724 |
| 225342_at | AK3L1 | adenylate kinase 3-like 1 | 5.65E-08 | 6.25212 |
| 205596_s_at | SMURF2 | SMAD specific E3 ubiquitin protein ligase 2 | 3.52E-06 | 6.2495 |
| 1555467_a_at | CELF1 | CUGBP, Elav-like family member 1 | 6.77E-07 | 6.24949 |
| 203163_at | KATNB1 | katanin p80 (WD repeat containing) subunit B 1 | 1.24E-05 | 6.24738 |
| 204459_at | CSTF2 | cleavage stimulation factor, 3' pre-RNA, subunit 2, 64kDa | 1.77E-06 | 6.24277 |
| 210041_s_at | PGM3 | phosphoglucomutase 3 | 1.29E-07 | 6.24119 |
| 209375_at | XPC | xeroderma pigmentosum, complementation group C | 3.96E-06 | 6.24064 |
| 217787_s_at | GALNT2 | UDP-N-acetyl-alpha-D-galactosamine:polypeptide N-acetylgalactosaminyltransferase | 3.16E-06 | 6.23981 |
| 225719_s_at | MRPL55 | mitochondrial ribosomal protein L55 | 6.78E-07 | 6.23958 |
| 201604_s_at | PPP1R12A | protein phosphatase 1, regulatory (inhibitor) subunit 12A | 7.26E-07 | 6.23899 |
| 212131_at | LSM14A | LSM14A, SCD6 homolog A (S. cerevisiae) | 1.29E-07 | 6.23771 |
| 212407_at | METTL13 | methyltransferase like 13 | 9.90E-07 | 6.23528 |
| 217811_at | SELT | selenoprotein T | 8.57E-08 | 6.23441 |
| 218079_s_at | GGNBP2 | gametogenetin binding protein 2 | 5.78E-06 | 6.23417 |
| 55081_at | MICALL1 | MICAL-like 1 | 8.51E-06 | 6.2337 |
| 219188_s_at | MACROD1 | MACRO domain containing 1 | 1.63E-06 | 6.23294 |
| 200751_s_at | HNRNPC | heterogeneous nuclear ribonucleoprotein C (C1/C2) | 5.21E-06 | 6.22989 |
| 204348_s_at | AK3L1 | adenylate kinase 3-like 1 | 7.40E-08 | 6.22717 |
| 209579_s_at | MBD4 | methyl-CpG binding domain protein 4 | 1.34E-05 | 6.22514 |
| 209184_s_at | IRS2 | insulin receptor substrate 2 | 1.49E-06 | 6.22302 |
| 200664_s_at | DNAJB1 | DnaJ (Hsp40) homolog, subfamily B, member 1 | 1.06E-05 | 6.22258 |
| 233093_s_at | BIRC6 | baculoviral IAP repeat-containing 6 | 7.36E-06 | 6.22142 |
| 202471_s_at | IDH3G | isocitrate dehydrogenase 3 (NAD+) gamma | 1.74E-07 | 6.22116 |
| 1553252_a_at | BRWD3 | bromodomain and WD repeat domain containing 3 | 5.66E-06 | 6.2173 |
| 225110_at | OGFOD1 | 2-oxoglutarate and iron-dependent oxygenase domain containing 1 | 3.98E-07 | 6.21541 |
| 212441_at | KIAA0232 | KIAA0232 | 2.28E-07 | 6.21423 |
| 227685_at | TMF1 | TATA element modulatory factor 1 | 1.35E-06 | 6.21235 |
| 221763_at | JMJD1C | jumonji domain containing 1C | 2.93E-05 | 6.21176 |
| 224711_at | YY1 | YY1 transcription factor | 1.79E-05 | 6.21156 |
| 205460_at | NPAS2 | neuronal PAS domain protein 2 | 7.72E-07 | 6.20939 |
| 203011_at | IMPA1 | inositol(myo)-1(or 4)-monophosphatase 1 | 1.21E-08 | 6.20789 |
| 230652_at | ARAF | v-raf murine sarcoma 3611 viral oncogene homolog | 2.79E-06 | 6.20628 |
| 222989_s_at | UBQLN1 | ubiquilin 1 | 4.99E-06 | 6.20575 |
| 217969_at | C11orf2 | chromosome 11 open reading frame 2 | 3.32E-06 | 6.20452 |
| 208975_s_at | KPNB1 | karyopherin (importin) beta 1 | 8.37E-06 | 6.20304 |
| 209222_s_at | OSBPL2 | oxysterol binding protein-like 2 | 4.63E-07 | 6.20167 |
| 224622_at | TBC1D14 | TBC1 domain family, member 14 | 3.50E-06 | 6.19796 |
| 206508_at | CD70 | CD70 molecule | 2.54E-07 | 6.19772 |
| 203120_at | TP53BP2 | tumor protein p53 binding protein, 2 | 4.92E-07 | 6.1953 |
| 212979_s_at | FAM115A | family with sequence similarity 115, member A | 9.38E-06 | 6.19445 |
| 225392_at | GFM2 | G elongation factor, mitochondrial 2 | 1.66E-05 | 6.18592 |
| 203046_s_at | TIMELESS | timeless homolog (Drosophila) | 2.58E-09 | 6.17915 |
| 211168_s_at | UPF1 | UPF1 regulator of nonsense transcripts homolog (yeast) | 6.33E-07 | 6.17904 |
| 200702_s_at | DDX24 | DEAD (Asp-Glu-Ala-Asp) box polypeptide 24 | 4.34E-06 | 6.17265 |
| 215329_s_at | CDK11A /// CDK11B | cyclin-dependent kinase 11A /// cyclin-dependent kinase 11B | 1.50E-06 | 6.17255 |
| 202472_at | MPI | mannose phosphate isomerase | 2.20E-06 | 6.17129 |
| 201127_s_at | ACLY | ATP citrate lyase | 8.31E-07 | 6.17089 |
| 202227_s_at | BRD8 | bromodomain containing 8 | 2.26E-06 | 6.16661 |
| 223056_s_at | XPO5 | exportin 5 | 2.46E-06 | 6.15843 |
| 213470_s_at | HNRNPH1 | heterogeneous nuclear ribonucleoprotein H1 (H) | 9.93E-06 | 6.15796 |
| 202673_at | DPM1 | dolichyl-phosphate mannosyltransferase polypeptide 1, catalytic subunit | 6.37E-06 | 6.15384 |
| 227941_at | LOC339803 | hypothetical protein LOC339803 | 1.13E-06 | 6.15378 |
| 202611_s_at | MED14 | mediator complex subunit 14 | 1.62E-05 | 6.14785 |
| 228559_at | CENPN | centromere protein N | 9.30E-06 | 6.14744 |
| 225077_at | CHD2 | chromodomain helicase DNA binding protein 2 | 7.38E-07 | 6.14722 |
| 201230_s_at | ARIH2 | ariadne homolog 2 (Drosophila) | 8.27E-08 | 6.14715 |
| 226159_at | C5orf51 | chromosome 5 open reading frame 51 | 3.18E-07 | 6.14343 |
| 227063_at | C17orf61 | chromosome 17 open reading frame 61 | 8.89E-09 | 6.14238 |
| 222883_at | C1orf163 | chromosome 1 open reading frame 163 | 2.18E-06 | 6.13864 |
| 219143_s_at | RPP25 | ribonuclease P/MRP 25kDa subunit | 3.54E-06 | 6.13796 |
| 1554411_at | CTNNB1 | catenin (cadherin-associated protein), beta 1, 88kDa | 9.57E-06 | 6.13794 |
| 225210_s_at | FAM103A1 | family with sequence similarity 103, member A1 | 9.64E-07 | 6.1369 |
| 214550_s_at | TNPO3 | transportin 3 | 1.63E-06 | 6.13662 |
| 202816_s_at | SS18 | synovial sarcoma translocation, chromosome 18 | 4.13E-08 | 6.13588 |
| 202232_s_at | EIF3M | eukaryotic translation initiation factor 3, subunit M | 0.0001595 | 6.13491 |
| 204616_at | UCHL3 | ubiquitin carboxyl-terminal esterase L3 (ubiquitin thiolesterase) | 1.26E-08 | 6.13262 |
| 211519_s_at | KIF2C | kinesin family member 2C | 2.73E-05 | 6.13175 |
| 213220_at | NCRNA00081 | non-protein coding RNA 81 | 7.81E-06 | 6.13067 |
| 209709_s_at | HMMR | hyaluronan-mediated motility receptor (RHAMM) | 5.24E-07 | 6.12897 |
| 215210_s_at | DLST | dihydrolipoamide S-succinyltransferase (E2 component of 2-oxo-glutarate complex) | 9.71E-05 | 6.12843 |
| 225783_at | UBE2F | ubiquitin-conjugating enzyme E2F (putative) | 2.06E-07 | 6.12804 |
| 236557_at | ZBTB38 | zinc finger and BTB domain containing 38 | 0.0004684 | 6.12554 |
| 200980_s_at | PDHA1 | pyruvate dehydrogenase (lipoamide) alpha 1 | 5.33E-09 | 6.12159 |
| 226195_at | C14orf179 | chromosome 14 open reading frame 179 | 5.29E-07 | 6.12145 |
| 201149_s_at | TIMP3 | TIMP metallopeptidase inhibitor 3 | 1.59E-07 | 6.12119 |
| 202854_at | HPRT1 | hypoxanthine phosphoribosyltransferase 1 | 6.82E-09 | 6.1197 |
| 203787_at | SSBP2 | single-stranded DNA binding protein 2 | 3.40E-05 | 6.11643 |
| 209666_s_at | CHUK | conserved helix-loop-helix ubiquitous kinase | 0.0001576 | 6.11122 |
| 205809_s_at | WASL | Wiskott-Aldrich syndrome-like | 2.28E-05 | 6.10991 |
| 200078_s_at | ATP6V0B | ATPase, H+ transporting, lysosomal 21kDa, V0 subunit b | 6.45E-08 | 6.10906 |
| 214686_at | ZNF266 | zinc finger protein 266 | 6.15E-06 | 6.10808 |
| 201696_at | SFRS4 | splicing factor, arginine/serine-rich 4 | 2.04E-07 | 6.10712 |
| 1555193_a_at | ZNF277 | zinc finger protein 277 | 9.04E-05 | 6.1066 |
| 226232_at | GDF11 | growth differentiation factor 11 | 3.90E-05 | 6.10532 |
| 214220_s_at | ALMS1 | Alstrom syndrome 1 | 7.90E-07 | 6.10525 |
| 202635_s_at | POLR2K | polymerase (RNA) II (DNA directed) polypeptide K, 7.0kDa | 1.42E-06 | 6.10423 |
| 203867_s_at | NLE1 | notchless homolog 1 (Drosophila) | 2.38E-06 | 6.10416 |
| 242260_at | MATR3 | Matrin 3 | 6.80E-05 | 6.10135 |
| 209391_at | DPM2 | dolichyl-phosphate mannosyltransferase polypeptide 2, regulatory subunit | 2.08E-05 | 6.1003 |
| 208872_s_at | REEP5 | receptor accessory protein 5 | 2.98E-09 | 6.09852 |
| 209486_at | UTP3 | UTP3, small subunit (SSU) processome component, homolog (S. cerevisiae) | 1.03E-05 | 6.09815 |
| 217992_s_at | EFHD2 | EF-hand domain family, member D2 | 1.66E-09 | 6.09785 |
| 200624_s_at | MATR3 | matrin 3 | 1.24E-06 | 6.09565 |
| 209069_s_at | H3F3B | H3 histone, family 3B (H3.3B) | 1.65E-05 | 6.09551 |
| 208174_x_at | ZRSR2 | zinc finger (CCCH type), RNA-binding motif and serine/arginine rich 2 | 7.91E-09 | 6.09437 |
| 201662_s_at | ACSL3 | acyl-CoA synthetase long-chain family member 3 | 1.23E-05 | 6.09357 |
| 221753_at | SSH1 | slingshot homolog 1 (Drosophila) | 1.34E-05 | 6.0919 |
| 218071_s_at | MKRN2 | makorin ring finger protein 2 | 2.87E-07 | 6.09076 |
| 227080_at | ZNF697 | zinc finger protein 697 | 5.23E-06 | 6.09019 |
| 213111_at | PIKFYVE | phosphoinositide kinase, FYVE finger containing | 3.75E-06 | 6.08965 |
| 216591_s_at | SDHC | succinate dehydrogenase complex, subunit C, integral membrane protein, 15kDa | 1.50E-06 | 6.08875 |
| 208270_s_at | RNPEP | arginyl aminopeptidase (aminopeptidase B) | 2.62E-07 | 6.08858 |
| 217196_s_at | CAMSAP1L1 | calmodulin regulated spectrin-associated protein 1-like 1 | 2.38E-06 | 6.08681 |
| 223382_s_at | ZNRF1 | zinc and ring finger 1 | 4.11E-06 | 6.08637 |
| 219219_at | TMEM160 | transmembrane protein 160 | 7.44E-07 | 6.08605 |
| 222459_at | AKIRIN1 | akirin 1 | 0.0002674 | 6.0815 |
| 210978_s_at | TAGLN2 | transgelin 2 | 4.32E-06 | 6.0792 |
| 209927_s_at | C1orf77 | chromosome 1 open reading frame 77 | 3.72E-07 | 6.07869 |
| 212542_s_at | PHIP | pleckstrin homology domain interacting protein | 3.04E-06 | 6.0784 |
| 203333_at | KIFAP3 | kinesin-associated protein 3 | 2.05E-07 | 6.07522 |
| 201210_at | DDX3X | DEAD (Asp-Glu-Ala-Asp) box polypeptide 3, X-linked | 2.01E-08 | 6.07515 |
| 208699_x_at | TKT | transketolase | 4.47E-05 | 6.07456 |
| 211949_s_at | NOLC1 | nucleolar and coiled-body phosphoprotein 1 | 4.08E-06 | 6.0685 |
| 226922_at | RANBP2 | RAN binding protein 2 | 2.49E-05 | 6.06465 |
| 228556_at | YTHDC1 | YTH domain containing 1 | 3.38E-05 | 6.0646 |
| 231319_x_at | KIF9 | kinesin family member 9 | 3.85E-08 | 6.06444 |
| 213090_s_at | TAF4 | TAF4 RNA polymerase II, TATA box binding protein (TBP)-associated factor, 135kDa | 3.34E-06 | 6.06432 |
| 32099_at | SAFB2 | scaffold attachment factor B2 | 4.11E-09 | 6.06135 |
| 225748_at | LTV1 | LTV1 homolog (S. cerevisiae) | 1.00E-05 | 6.06014 |
| 230069_at | SFXN1 | sideroflexin 1 | 2.83E-06 | 6.05983 |
| 202155_s_at | NUP214 | nucleoporin 214kDa | 5.48E-05 | 6.0539 |
| 225884_s_at | GZF1 | GDNF-inducible zinc finger protein 1 | 7.84E-06 | 6.05318 |
| 203630_s_at | COG5 | component of oligomeric golgi complex 5 | 6.80E-06 | 6.05295 |
| 230660_at | SERTAD4 | SERTA domain containing 4 | 3.20E-06 | 6.05208 |
| 211946_s_at | BAT2L2 | HLA-B associated transcript 2-like 2 | 6.72E-05 | 6.05151 |
| 225064_at | RABEP1 | rabaptin, RAB GTPase binding effector protein 1 | 4.47E-07 | 6.05058 |
| 204670_x_at | HLA-DRB1 /// HLA-DRB4 | major histocompatibility complex, class II, DR beta 1 /// major histocompatibili | 4.11E-07 | 6.04887 |
| 215230_x_at | EIF3C /// EIF3CL | eukaryotic translation initiation factor 3, subunit C /// eukaryotic translation | 1.65E-08 | 6.04828 |
| 210011_s_at | EWSR1 | Ewing sarcoma breakpoint region 1 | 1.58E-05 | 6.04542 |
| 228992_at | MED28 | Mediator complex subunit 28 | 1.31E-07 | 6.04198 |
| 225859_at | XIAP | X-linked inhibitor of apoptosis | 1.08E-06 | 6.03826 |
| 204975_at | EMP2 | epithelial membrane protein 2 | 5.62E-08 | 6.03808 |
| 212034_s_at | EXOC7 | exocyst complex component 7 | 3.95E-07 | 6.03435 |
| 235783_at | MRTO4 | mRNA turnover 4 homolog (S. cerevisiae) | 1.90E-07 | 6.03317 |
| 201003_x_at | RNPEP /// TMEM189 /// TMEM189-UBE2V1 /// UBE2V1 | arginyl aminopeptidase (aminopeptidase B) /// transmembrane protein 189 /// TMEM | 8.40E-08 | 6.03074 |
| 217780_at | C19orf56 | chromosome 19 open reading frame 56 | 3.50E-06 | 6.02809 |
| 231406_at | ORAI2 | ORAI calcium release-activated calcium modulator 2 | 3.26E-07 | 6.02805 |
| 204566_at | PPM1D | protein phosphatase, Mg2+/Mn2+ dependent, 1D | 6.86E-06 | 6.0257 |
| 212787_at | YLPM1 | YLP motif containing 1 | 7.65E-07 | 6.02257 |
| 225145_at | NCOA5 | nuclear receptor coactivator 5 | 1.46E-07 | 6.02256 |
| 225189_s_at | RAPH1 | Ras association (RalGDS/AF-6) and pleckstrin homology domains 1 | 2.11E-06 | 6.02158 |
| 212994_at | THOC2 | THO complex 2 | 9.98E-08 | 6.02011 |
| 215424_s_at | SNW1 | SNW domain containing 1 | 1.67E-08 | 6.01803 |
| 209675_s_at | HNRNPUL1 | heterogeneous nuclear ribonucleoprotein U-like 1 | 3.28E-05 | 6.01752 |
| 210875_s_at | ZEB1 | zinc finger E-box binding homeobox 1 | 0.0001664 | 6.01726 |
| 225780_at | RSC1A1 | regulatory solute carrier protein, family 1, member 1 | 7.82E-06 | 6.01677 |
| 221604_s_at | PEX16 | peroxisomal biogenesis factor 16 | 6.86E-06 | 6.01505 |
| 218118_s_at | LOC10431 /// TIMM23 | translocase of inner mitochondrial membrane 23 homolog (yeast)-like /// transloc | 9.85E-08 | 6.01344 |
| 202918_s_at | MOBKL3 | MOB1, Mps One Binder kinase activator-like 3 (yeast) | 1.27E-06 | 6.01316 |
| 203244_at | PEX5 | peroxisomal biogenesis factor 5 | 1.04E-08 | 6.01171 |
| 213937_s_at | FTSJ1 | FtsJ homolog 1 (E. coli) | 6.51E-06 | 6.01166 |
| 210555_s_at | NFATC3 | nuclear factor of activated T-cells, cytoplasmic, calcineurin-dependent 3 | 3.14E-06 | 6.01129 |
| 208716_s_at | TMCO1 | transmembrane and coiled-coil domains 1 | 9.28E-08 | 6.01088 |
| 218304_s_at | OSBPL11 | oxysterol binding protein-like 11 | 0.0001057 | 6.01085 |
| 203563_at | AFAP1 | actin filament associated protein 1 | 2.97E-06 | 6.00984 |
| 64432_at | C12orf47 | chromosome 12 open reading frame 47 | 1.91E-07 | 6.00923 |
| 242283_at | DNAH14 | dynein, axonemal, heavy chain 14 | 2.45E-06 | 6.00797 |
| 200975_at | PPT1 | palmitoyl-protein thioesterase 1 | 6.22E-05 | 6.0079 |
| 213086_s_at | CSNK1A1 | casein kinase 1, alpha 1 | 3.32E-07 | 6.00747 |
| 228961_at | MIER3 | mesoderm induction early response 1, family member 3 | 0.0002049 | 6.00644 |
| 217919_s_at | MRPL42 | mitochondrial ribosomal protein L42 | 2.41E-07 | 6.00216 |
| 216248_s_at | NR4A2 | nuclear receptor subfamily 4, group A, member 2 | 5.66E-06 | 5.99988 |
| 208313_s_at | SF1 | splicing factor 1 | 2.98E-09 | 5.99963 |
| 202304_at | FNDC3A | fibronectin type III domain containing 3A | 9.67E-07 | 5.9993 |
| 222158_s_at | PPPDE1 | PPPDE peptidase domain containing 1 | 1.79E-07 | 5.99826 |
| 202477_s_at | TUBGCP2 | tubulin, gamma complex associated protein 2 | 3.77E-06 | 5.9973 |
| 228904_at | HOXB3 | homeobox B3 | 1.28E-05 | 5.99504 |
| 203721_s_at | UTP18 | UTP18, small subunit (SSU) processome component, homolog (yeast) | 3.95E-07 | 5.99222 |
| 223246_s_at | STRBP | spermatid perinuclear RNA binding protein | 1.80E-06 | 5.99193 |
| 200629_at | WARS | tryptophanyl-tRNA synthetase | 3.97E-08 | 5.99149 |
| 201648_at | JAK1 | Janus kinase 1 | 1.97E-07 | 5.99058 |
| 202147_s_at | IFRD1 | interferon-related developmental regulator 1 | 5.97E-06 | 5.98743 |
| 218710_at | TTC27 | tetratricopeptide repeat domain 27 | 1.94E-05 | 5.98059 |
| 222538_s_at | APPL1 | adaptor protein, phosphotyrosine interaction, PH domain and leucine zipper conta | 5.51E-07 | 5.97879 |
| 202253_s_at | DNM2 | dynamin 2 | 2.21E-05 | 5.97851 |
| 211185_s_at | SF3B1 | splicing factor 3b, subunit 1, 155kDa | 2.62E-07 | 5.9781 |
| 222428_s_at | LARS | leucyl-tRNA synthetase | 2.93E-06 | 5.97477 |
| 231828_at | LOC253039 | hypothetical LOC253039 | 4.84E-05 | 5.97293 |
| 235059_at | RAB12 | RAB12, member RAS oncogene family | 1.41E-06 | 5.97221 |
| 218480_at | AGBL5 | ATP/GTP binding protein-like 5 | 5.46E-06 | 5.97183 |
| 225462_at | TMEM128 | transmembrane protein 128 | 1.02E-06 | 5.97075 |
| 225901_at | PTPMT1 | protein tyrosine phosphatase, mitochondrial 1 | 2.17E-07 | 5.97003 |
| 225765_at | TNPO1 | transportin 1 | 4.49E-08 | 5.96592 |
| 212032_s_at | PTOV1 | prostate tumor overexpressed 1 | 1.15E-06 | 5.96456 |
| 201769_at | CLINT1 | clathrin interactor 1 | 3.42E-07 | 5.96439 |
| 225507_at | SFRS18 | splicing factor, arginine/serine-rich 18 | 1.32E-06 | 5.96171 |
| 205176_s_at | ITGB3BP | integrin beta 3 binding protein (beta3-endonexin) | 6.81E-09 | 5.95856 |
| 209330_s_at | HNRNPD | heterogeneous nuclear ribonucleoprotein D (AU-rich element RNA binding protein 1 | 1.86E-07 | 5.95805 |
| 201930_at | MCM6 | minichromosome maintenance complex component 6 | 6.12E-06 | 5.95723 |
| 212513_s_at | USP33 | ubiquitin specific peptidase 33 | 3.09E-07 | 5.95651 |
| 225768_at | NR1D2 | nuclear receptor subfamily 1, group D, member 2 | 8.16E-05 | 5.95649 |
| 201771_at | SCAMP3 | secretory carrier membrane protein 3 | 2.88E-07 | 5.95566 |
| 223114_at | COQ5 | coenzyme Q5 homolog, methyltransferase (S. cerevisiae) | 2.01E-07 | 5.95344 |
| 202798_at | SEC24B | SEC24 family, member B (S. cerevisiae) | 3.69E-06 | 5.953 |
| 218957_s_at | PAAF1 | proteasomal ATPase-associated factor 1 | 7.25E-07 | 5.95221 |
| 213311_s_at | TCF25 | transcription factor 25 (basic helix-loop-helix) | 1.92E-06 | 5.95115 |
| 225995_x_at | WASH1 /// WASH2P | WAS protein family homolog 1 /// WAS protein family homolog 2 pseudogene | 0.0001369 | 5.94911 |
| 218280_x_at | HIST2H2AA3 /// HIST2H2AA4 | histone cluster 2, H2aa3 /// histone cluster 2, H2aa4 | 5.46E-06 | 5.94817 |
| 202852_s_at | AAGAB | alpha- and gamma-adaptin binding protein | 1.37E-07 | 5.94791 |
| 1568597_at | LOC646762 | hypothetical LOC646762 | 2.06E-07 | 5.94749 |
| 224783_at | FAM100B | family with sequence similarity 100, member B | 1.25E-06 | 5.9467 |
| 36830_at | MIPEP | mitochondrial intermediate peptidase | 1.16E-07 | 5.94535 |
| 223351_at | C17orf80 | chromosome 17 open reading frame 80 | 1.22E-06 | 5.94522 |
| 205089_at | ZNF7 | zinc finger protein 7 | 2.14E-07 | 5.94294 |
| 235409_at | MGA | MAX gene associated | 1.54E-06 | 5.94278 |
| 225416_at | RLIM | ring finger protein, LIM domain interacting | 9.03E-07 | 5.94213 |
| 204059_s_at | ME1 | malic enzyme 1, NADP(+)-dependent, cytosolic | 1.30E-08 | 5.94144 |
| 223288_at | USP38 | ubiquitin specific peptidase 38 | 7.09E-06 | 5.94128 |
| 211271_x_at | PTBP1 | polypyrimidine tract binding protein 1 | 7.71E-07 | 5.94124 |
| 1556285_s_at | PPA2 | pyrophosphatase (inorganic) 2 | 7.53E-07 | 5.93848 |
| 202954_at | UBE2C | ubiquitin-conjugating enzyme E2C | 3.43E-07 | 5.93651 |
| 219861_at | DNAJC17 | DnaJ (Hsp40) homolog, subfamily C, member 17 | 3.42E-06 | 5.93618 |
| 208802_at | SRP72 | signal recognition particle 72kDa | 1.19E-05 | 5.93386 |
| 212590_at | RRAS2 | related RAS viral (r-ras) oncogene homolog 2 | 1.45E-08 | 5.93373 |
| 225761_at | PAPD4 | PAP associated domain containing 4 | 8.21E-06 | 5.93349 |
| 225936_at | EID2 | EP300 interacting inhibitor of differentiation 2 | 1.57E-06 | 5.93283 |
| 226972_s_at | CCDC136 | coiled-coil domain containing 136 | 1.85E-05 | 5.92866 |
| 203241_at | UVRAG | UV radiation resistance associated gene | 1.58E-07 | 5.92544 |
| 217968_at | TSSC1 | tumor suppressing subtransferable candidate 1 | 5.60E-09 | 5.92426 |
| 208908_s_at | CAST | calpastatin | 1.14E-05 | 5.92338 |
| 222698_s_at | IMPACT | Impact homolog (mouse) | 2.14E-06 | 5.92232 |
| 201740_at | NDUFS3 | NADH dehydrogenase (ubiquinone) Fe-S protein 3, 30kDa (NADH-coenzyme Q reductase | 1.67E-07 | 5.92212 |
| 203983_at | TSNAX | translin-associated factor X | 2.74E-05 | 5.92113 |
| 209282_at | PRKD2 | protein kinase D2 | 2.30E-06 | 5.92062 |
| 212330_at | TFDP1 | transcription factor Dp-1 | 2.49E-09 | 5.91988 |
| 224320_s_at | MCM8 | minichromosome maintenance complex component 8 | 5.04E-06 | 5.91832 |
| 202907_s_at | NBN | nibrin | 2.04E-08 | 5.91577 |
| 226413_at | LOC400027 | hypothetical protein LOC400027 | 2.92E-06 | 5.91556 |
| 222138_s_at | WDR13 | WD repeat domain 13 | 2.67E-08 | 5.91405 |
| 203785_s_at | DDX28 | DEAD (Asp-Glu-Ala-Asp) box polypeptide 28 | 1.51E-05 | 5.91226 |
| 218496_at | RNASEH1 | ribonuclease H1 | 1.32E-09 | 5.91121 |
| 225012_at | HDLBP | high density lipoprotein binding protein | 2.71E-05 | 5.91075 |
| 227350_at | HELLS | helicase, lymphoid-specific | 6.75E-06 | 5.91039 |
| 224768_at | IWS1 | IWS1 homolog (S. cerevisiae) | 2.59E-06 | 5.91023 |
| 201179_s_at | GNAI3 | guanine nucleotide binding protein (G protein), alpha inhibiting activity polype | 7.15E-08 | 5.90774 |
| 219979_s_at | C11orf73 | chromosome 11 open reading frame 73 | 3.66E-05 | 5.90773 |
| 204905_s_at | EEF1E1 | eukaryotic translation elongation factor 1 epsilon 1 | 1.66E-07 | 5.90625 |
| 217749_at | COPG | coatomer protein complex, subunit gamma | 1.33E-07 | 5.90091 |
| 224718_at | YY1 | YY1 transcription factor | 1.00E-05 | 5.8987 |
| 225731_at | ANKRD50 | ankyrin repeat domain 50 | 1.38E-05 | 5.89666 |
| 223197_s_at | SMARCAD1 | SWI/SNF-related, matrix-associated actin-dependent regulator of chromatin, subfa | 1.02E-05 | 5.89664 |
| 226496_at | ZCCHC7 | zinc finger, CCHC domain containing 7 | 2.68E-06 | 5.89587 |
| 233656_s_at | VPS54 | vacuolar protein sorting 54 homolog (S. cerevisiae) | 4.04E-07 | 5.89408 |
| 235944_at | HMCN1 | hemicentin 1 | 1.02E-05 | 5.89394 |
| 224966_s_at | DUS3L | dihydrouridine synthase 3-like (S. cerevisiae) | 8.95E-05 | 5.89108 |
| 225384_at | DOCK7 | dedicator of cytokinesis 7 | 1.25E-07 | 5.89089 |
| 225806_at | JUB | jub, ajuba homolog (Xenopus laevis) | 7.24E-07 | 5.89013 |
| 205395_s_at | MRE11A | MRE11 meiotic recombination 11 homolog A (S. cerevisiae) | 0.0001643 | 5.88942 |
| 225593_at | LSM10 | LSM10, U7 small nuclear RNA associated | 2.41E-06 | 5.88706 |
| 221699_s_at | DDX50 | DEAD (Asp-Glu-Ala-Asp) box polypeptide 50 | 1.39E-05 | 5.88641 |
| 225386_s_at | HNRPLL | heterogeneous nuclear ribonucleoprotein L-like | 5.24E-08 | 5.88513 |
| 233878_s_at | XRN2 | 5'-3' exoribonuclease 2 | 7.12E-07 | 5.88382 |
| 214305_s_at | SF3B1 | splicing factor 3b, subunit 1, 155kDa | 7.31E-06 | 5.88358 |
| 228829_at | ATF7 | activating transcription factor 7 | 3.12E-07 | 5.88354 |
| 211783_s_at | MTA1 | metastasis associated 1 | 2.50E-07 | 5.8824 |
| 212447_at | KBTBD2 | kelch repeat and BTB (POZ) domain containing 2 | 8.68E-08 | 5.88147 |
| 218774_at | DCPS | decapping enzyme, scavenger | 2.46E-07 | 5.88076 |
| 214452_at | BCAT1 | branched chain amino-acid transaminase 1, cytosolic | 1.00E-05 | 5.87838 |
| 224754_at | SP1 | Sp1 transcription factor | 2.02E-07 | 5.87814 |
| 219081_at | ANKHD1 | ankyrin repeat and KH domain containing 1 | 1.80E-05 | 5.87737 |
| 207564_x_at | OGT | O-linked N-acetylglucosamine (GlcNAc) transferase (UDP-N-acetylglucosamine:polyp | 3.08E-06 | 5.87629 |
| 211947_s_at | BAT2L2 | HLA-B associated transcript 2-like 2 | 7.90E-05 | 5.87619 |
| 207239_s_at | CDK16 | cyclin-dependent kinase 16 | 7.85E-06 | 5.87554 |
| 201688_s_at | TPD52 | tumor protein D52 | 5.28E-07 | 5.87543 |
| 224944_at | TMPO | thymopoietin | 3.09E-06 | 5.87282 |
| 229232_at | LRRC57 | leucine rich repeat containing 57 | 5.13E-05 | 5.87158 |
| 201966_at | NDUFS2 | NADH dehydrogenase (ubiquinone) Fe-S protein 2, 49kDa (NADH-coenzyme Q reductase | 6.68E-10 | 5.87154 |
| 223225_s_at | SEH1L | SEH1-like (S. cerevisiae) | 0.0007183 | 5.86992 |
| 201585_s_at | SFPQ | splicing factor proline/glutamine-rich | 2.17E-05 | 5.86905 |
| 225208_s_at | FAM103A1 | family with sequence similarity 103, member A1 | 2.15E-07 | 5.86841 |
| 202188_at | NUP93 | nucleoporin 93kDa | 8.13E-05 | 5.86048 |
| 217760_at | TRIM44 | tripartite motif-containing 44 | 6.75E-08 | 5.85964 |
| 213526_s_at | LIN37 | lin-37 homolog (C. elegans) | 1.08E-05 | 5.85952 |
| 209482_at | POP7 | processing of precursor 7, ribonuclease P/MRP subunit (S. cerevisiae) | 6.06E-08 | 5.85927 |
| 228729_at | CCNB1 | cyclin B1 | 7.13E-05 | 5.85602 |
| 218809_at | PANK2 | pantothenate kinase 2 | 4.23E-07 | 5.85591 |
| 217954_s_at | PHF3 | PHD finger protein 3 | 5.44E-06 | 5.85562 |
| 226524_at | C3orf38 | chromosome 3 open reading frame 38 | 3.44E-06 | 5.85513 |
| 226236_at | LOC388789 | hypothetical LOC388789 | 6.97E-08 | 5.85352 |
| 216392_s_at | SEC23IP | SEC23 interacting protein | 6.13E-06 | 5.85242 |
| 202281_at | GAK | cyclin G associated kinase | 1.36E-06 | 5.85097 |
| 225479_at | LRRC58 | leucine rich repeat containing 58 | 6.65E-08 | 5.84809 |
| 202680_at | GTF2E2 | general transcription factor IIE, polypeptide 2, beta 34kDa | 5.45E-07 | 5.84715 |
| 221042_s_at | CLMN | calmin (calponin-like, transmembrane) | 5.28E-06 | 5.84597 |
| 225712_at | GEMIN5 | gem (nuclear organelle) associated protein 5 | 1.56E-07 | 5.84531 |
| 226257_x_at | MRPS22 | mitochondrial ribosomal protein S22 | 1.16E-07 | 5.84502 |
| 201870_at | TOMM34 | translocase of outer mitochondrial membrane 34 | 9.76E-06 | 5.8442 |
| 212114_at | ATXN7L3B | ataxin 7-like 3B | 9.88E-08 | 5.84302 |
| 208643_s_at | XRCC5 | X-ray repair complementing defective repair in Chinese hamster cells 5 (double-s | 1.34E-08 | 5.84196 |
| 233759_s_at | SMEK2 | SMEK homolog 2, suppressor of mek1 (Dictyostelium) | 1.91E-06 | 5.83999 |
| 218712_at | C1orf109 | chromosome 1 open reading frame 109 | 9.35E-06 | 5.83823 |
| 219555_s_at | CENPN | centromere protein N | 1.21E-06 | 5.83657 |
| 225858_s_at | XIAP | X-linked inhibitor of apoptosis | 1.08E-06 | 5.83547 |
| 205474_at | CRLF3 | cytokine receptor-like factor 3 | 2.33E-06 | 5.83438 |
| 209025_s_at | SYNCRIP | synaptotagmin binding, cytoplasmic RNA interacting protein | 9.94E-07 | 5.83319 |
| 236248_x_at | TADA2B | transcriptional adaptor 2B | 6.32E-05 | 5.8328 |
| 208629_s_at | HADHA | hydroxyacyl-CoA dehydrogenase/3-ketoacyl-CoA thiolase/enoyl-CoA hydratase (trifu | 1.93E-07 | 5.83239 |
| 212998_x_at | HLA-DQB1 /// LOC100294318 | major histocompatibility complex, class II, DQ beta 1 /// similar to major histo | 4.42E-06 | 5.83023 |
| 236656_s_at | LOC100288911 | hypothetical protein LOC100288911 | 1.19E-07 | 5.82998 |
| 218951_s_at | PLCXD1 | phosphatidylinositol-specific phospholipase C, X domain containing 1 | 2.01E-07 | 5.82987 |
| 205224_at | SURF2 | surfeit 2 | 1.14E-05 | 5.82899 |
| 231896_s_at | DENR | density-regulated protein | 5.67E-08 | 5.8284 |
| 219000_s_at | DSCC1 | defective in sister chromatid cohesion 1 homolog (S. cerevisiae) | 2.83E-07 | 5.82832 |
| 235542_at | TET3 | tet oncogene family member 3 | 0.0003988 | 5.82808 |
| 204128_s_at | RFC3 | replication factor C (activator 1) 3, 38kDa | 2.82E-06 | 5.82774 |
| 223386_at | FAM118B | family with sequence similarity 118, member B | 1.57E-06 | 5.82655 |
| 218104_at | TEX10 | testis expressed 10 | 9.34E-05 | 5.82563 |
| 202160_at | CREBBP | CREB binding protein | 2.86E-06 | 5.82467 |
| 212212_s_at | INTS1 | integrator complex subunit 1 | 6.61E-07 | 5.82429 |
| 213223_at | RPL28 | ribosomal protein L28 | 5.15E-06 | 5.82397 |
| 203277_at | DFFA | DNA fragmentation factor, 45kDa, alpha polypeptide | 1.36E-06 | 5.82269 |
| 203303_at | DYNLT3 | dynein, light chain, Tctex-type 3 | 3.76E-09 | 5.82147 |
| 212145_at | MRPS27 | mitochondrial ribosomal protein S27 | 2.33E-08 | 5.82125 |
| 231876_at | TRIM56 | tripartite motif-containing 56 | 2.48E-06 | 5.81897 |
| 40225_at | GAK | cyclin G associated kinase | 5.53E-08 | 5.8175 |
| 230588_s_at | LOC285074 /// LOC730268 | anaphase promoting complex subunit 1 pseudogene /// similar to anaphase promotin | 2.33E-07 | 5.81695 |
| 222442_s_at | ARL8B | ADP-ribosylation factor-like 8B | 2.32E-07 | 5.81607 |
| 212218_s_at | FASN | fatty acid synthase | 1.43E-05 | 5.81359 |
| 218254_s_at | SAR1B | SAR1 homolog B (S. cerevisiae) | 3.89E-05 | 5.81336 |
| 208644_at | PARP1 | poly (ADP-ribose) polymerase 1 | 7.33E-07 | 5.81287 |
| 205599_at | TRAF1 | TNF receptor-associated factor 1 | 2.31E-06 | 5.81156 |
| 201189_s_at | ITPR3 | inositol 1,4,5-triphosphate receptor, type 3 | 1.58E-05 | 5.80853 |
| 202653_s_at | 7-三月 | membrane-associated ring finger (C3HC4) 7 | 4.88E-06 | 5.80846 |
| 209214_s_at | EWSR1 | Ewing sarcoma breakpoint region 1 | 3.68E-06 | 5.80789 |
| 208887_at | EIF3G | eukaryotic translation initiation factor 3, subunit G | 8.94E-07 | 5.80659 |
| 203089_s_at | HTRA2 | HtrA serine peptidase 2 | 5.24E-06 | 5.80649 |
| 202763_at | CASP3 | caspase 3, apoptosis-related cysteine peptidase | 2.14E-07 | 5.80525 |
| 201953_at | CIB1 | calcium and integrin binding 1 (calmyrin) | 1.47E-07 | 5.80455 |
| 201152_s_at | MBNL1 | muscleblind-like (Drosophila) | 6.83E-09 | 5.80432 |
| 201411_s_at | PLEKHB2 | pleckstrin homology domain containing, family B (evectins) member 2 | 1.17E-05 | 5.80407 |
| 201756_at | RPA2 | replication protein A2, 32kDa | 3.99E-06 | 5.80255 |
| 200835_s_at | MAP4 | microtubule-associated protein 4 | 1.47E-05 | 5.80187 |
| 224962_at | C9orf69 | chromosome 9 open reading frame 69 | 8.48E-07 | 5.79961 |
| 221711_s_at | C19orf62 | chromosome 19 open reading frame 62 | 1.36E-09 | 5.79716 |
| 212408_at | TOR1AIP1 | torsin A interacting protein 1 | 3.18E-07 | 5.79511 |
| 209820_s_at | TBL3 | transducin (beta)-like 3 | 1.52E-06 | 5.79335 |
| 224593_at | ZNF664 | zinc finger protein 664 | 2.19E-06 | 5.79319 |
| 218580_x_at | AURKAIP1 | aurora kinase A interacting protein 1 | 2.59E-08 | 5.79249 |
| 200852_x_at | GNB2 | guanine nucleotide binding protein (G protein), beta polypeptide 2 | 8.05E-07 | 5.79239 |
| 203653_s_at | COIL | coilin | 1.14E-05 | 5.79168 |
| 218381_s_at | U2AF2 | U2 small nuclear RNA auxiliary factor 2 | 1.42E-07 | 5.79032 |
| 229666_s_at | CSTF3 | cleavage stimulation factor, 3' pre-RNA, subunit 3, 77kDa | 1.77E-06 | 5.78873 |
| 203566_s_at | AGL | amylo-alpha-1, 6-glucosidase, 4-alpha-glucanotransferase | 1.23E-06 | 5.78552 |
| 207338_s_at | ZNF200 | zinc finger protein 200 | 1.39E-06 | 5.78526 |
| 208756_at | EIF3I | eukaryotic translation initiation factor 3, subunit I | 1.99E-06 | 5.78119 |
| 226588_at | CWC22 | CWC22 spliceosome-associated protein homolog (S. cerevisiae) | 1.94E-05 | 5.78093 |
| 1558093_s_at | MATR3 | matrin 3 | 3.94E-05 | 5.77972 |
| 218495_at | UXT | ubiquitously-expressed transcript | 3.99E-06 | 5.77887 |
| 212715_s_at | MICAL3 | microtubule associated monoxygenase, calponin and LIM domain containing 3 | 2.62E-06 | 5.77854 |
| 222105_s_at | NKIRAS2 | NFKB inhibitor interacting Ras-like 2 | 5.09E-06 | 5.77829 |
| 208776_at | PSMD11 | proteasome (prosome, macropain) 26S subunit, non-ATPase, 11 | 3.26E-05 | 5.77771 |
| 209862_s_at | CEP57 | centrosomal protein 57kDa | 7.16E-05 | 5.77627 |
| 209667_at | CES2 | carboxylesterase 2 (intestine, liver) | 1.20E-07 | 5.77593 |
| 205434_s_at | AAK1 | AP2 associated kinase 1 | 5.35E-08 | 5.77428 |
| 228840_at | AMOTL1 | angiomotin like 1 | 8.21E-07 | 5.77364 |
| 202041_s_at | FIBP | fibroblast growth factor (acidic) intracellular binding protein | 3.96E-06 | 5.77213 |
| 226337_at | GORAB | golgin, RAB6-interacting | 1.22E-06 | 5.7708 |
| 206600_s_at | SLC16A5 | solute carrier family 16, member 5 (monocarboxylic acid transporter 6) | 7.12E-05 | 5.77073 |
| 200896_x_at | HDGF | hepatoma-derived growth factor | 3.12E-08 | 5.77057 |
| 225455_at | TADA1 | transcriptional adaptor 1 | 1.03E-05 | 5.77005 |
| 209337_at | PSIP1 | PC4 and SFRS1 interacting protein 1 | 2.81E-07 | 5.76859 |
| 225161_at | GFM1 | G elongation factor, mitochondrial 1 | 2.78E-08 | 5.7649 |
| 203492_x_at | CEP57 | centrosomal protein 57kDa | 1.58E-05 | 5.76394 |
| 218378_s_at | PRKRIP1 | PRKR interacting protein 1 (IL11 inducible) | 2.44E-07 | 5.76026 |
| 223433_at | C7orf36 | chromosome 7 open reading frame 36 | 1.43E-06 | 5.75783 |
| 203536_s_at | CIAO1 | cytosolic iron-sulfur protein assembly 1 homolog (S. cerevisiae) | 3.68E-06 | 5.75783 |
| 215794_x_at | GLUD2 | glutamate dehydrogenase 2 | 2.46E-07 | 5.75756 |
| 202569_s_at | MARK3 | MAP/microtubule affinity-regulating kinase 3 | 3.76E-05 | 5.75705 |
| 219124_at | C8orf41 | chromosome 8 open reading frame 41 | 9.84E-07 | 5.75504 |
| 225558_at | GIT2 | G protein-coupled receptor kinase interacting ArfGAP 2 | 1.26E-06 | 5.75294 |
| 213899_at | METAP2 | methionyl aminopeptidase 2 | 2.14E-05 | 5.74952 |
| 216693_x_at | HDGFRP3 | hepatoma-derived growth factor, related protein 3 | 5.59E-07 | 5.74798 |
| 211595_s_at | MRPS11 | mitochondrial ribosomal protein S11 | 1.71E-09 | 5.74756 |
| 223004_s_at | C3orf1 | chromosome 3 open reading frame 1 | 1.07E-07 | 5.7472 |
| 226011_at | CCDC12 | coiled-coil domain containing 12 | 0.0001075 | 5.74712 |
| 201384_s_at | NBR1 | neighbor of BRCA1 gene 1 | 1.69E-06 | 5.74563 |
| 209449_at | LSM2 | LSM2 homolog, U6 small nuclear RNA associated (S. cerevisiae) | 4.19E-07 | 5.74391 |
| 209406_at | BAG2 | BCL2-associated athanogene 2 | 1.77E-07 | 5.74318 |
| 208093_s_at | NDEL1 | nudE nuclear distribution gene E homolog (A. nidulans)-like 1 | 1.07E-06 | 5.74236 |
| 209549_s_at | DGUOK | deoxyguanosine kinase | 4.24E-10 | 5.74222 |
| 220949_s_at | C7orf49 | chromosome 7 open reading frame 49 | 1.39E-07 | 5.74162 |
| 212955_s_at | POLR2I | polymerase (RNA) II (DNA directed) polypeptide I, 14.5kDa | 3.08E-06 | 5.74134 |
| 203605_at | SRP54 | signal recognition particle 54kDa | 2.02E-06 | 5.73995 |
| 225805_at | HNRNPU | heterogeneous nuclear ribonucleoprotein U (scaffold attachment factor A) | 1.89E-06 | 5.73986 |
| 205687_at | UBFD1 | ubiquitin family domain containing 1 | 1.03E-05 | 5.73951 |
| 54632_at | THADA | thyroid adenoma associated | 6.47E-06 | 5.73564 |
| 205048_s_at | PSPH | phosphoserine phosphatase | 2.38E-05 | 5.73489 |
| 211603_s_at | ETV4 | ets variant 4 | 6.35E-07 | 5.73337 |
| 208950_s_at | ALDH7A1 | aldehyde dehydrogenase 7 family, member A1 | 1.24E-07 | 5.73276 |
| 209838_at | COPS2 | COP9 constitutive photomorphogenic homolog subunit 2 (Arabidopsis) | 1.30E-08 | 5.73271 |
| 224812_at | HIBADH | 3-hydroxyisobutyrate dehydrogenase | 2.09E-07 | 5.73138 |
| 222047_s_at | SRRT | serrate RNA effector molecule homolog (Arabidopsis) | 2.01E-08 | 5.73123 |
| 214040_s_at | GSN | gelsolin | 1.17E-06 | 5.7293 |
| 218585_s_at | DTL | denticleless homolog (Drosophila) | 3.09E-06 | 5.72793 |
| 213074_at | PHIP | pleckstrin homology domain interacting protein | 8.00E-07 | 5.72749 |
| 1553997_a_at | ASPHD1 | aspartate beta-hydroxylase domain containing 1 | 2.68E-06 | 5.72642 |
| 208840_s_at | G3BP2 | GTPase activating protein (SH3 domain) binding protein 2 | 8.45E-07 | 5.72638 |
| 216037_x_at | TCF7L2 | transcription factor 7-like 2 (T-cell specific, HMG-box) | 1.07E-05 | 5.72419 |
| 204634_at | NEK4 | NIMA (never in mitosis gene a)-related kinase 4 | 2.56E-05 | 5.7232 |
| 202291_s_at | MGP | matrix Gla protein | 1.61E-05 | 5.72238 |
| 221428_s_at | TBL1XR1 | transducin (beta)-like 1 X-linked receptor 1 | 3.41E-05 | 5.72119 |
| 226463_at | ATP6V1C1 | ATPase, H+ transporting, lysosomal 42kDa, V1 subunit C1 | 6.05E-07 | 5.72079 |
| 218366_x_at | LOC731602 /// METT11D1 | similar to methyltransferase 11 domain containing 1 isoform 2 /// methyltransfer | 2.01E-07 | 5.71877 |
| 217741_s_at | ZFAND5 | zinc finger, AN1-type domain 5 | 2.95E-05 | 5.71831 |
| 225338_at | ZYG11B | zyg-11 homolog B (C. elegans) | 1.44E-05 | 5.71824 |
| 204608_at | ASL | argininosuccinate lyase | 1.06E-06 | 5.7159 |
| 201917_s_at | SLC25A36 | solute carrier family 25, member 36 | 3.71E-07 | 5.71474 |
| 222449_at | PMEPA1 | prostate transmembrane protein, androgen induced 1 | 5.19E-07 | 5.71195 |
| 224562_at | WASF2 | WAS protein family, member 2 | 1.55E-07 | 5.711 |
| 201755_at | MCM5 | minichromosome maintenance complex component 5 | 1.45E-06 | 5.71085 |
| 212781_at | RBBP6 | retinoblastoma binding protein 6 | 0.000322 | 5.70969 |
| 222963_s_at | IL1RAPL1 | interleukin 1 receptor accessory protein-like 1 | 4.63E-06 | 5.70862 |
| 217915_s_at | RSL24D1 | ribosomal L24 domain containing 1 | 8.00E-07 | 5.7082 |
| 226409_at | TBC1D20 | TBC1 domain family, member 20 | 4.71E-06 | 5.70716 |
| 213793_s_at | HOMER1 | homer homolog 1 (Drosophila) | 3.76E-06 | 5.707 |
| 222593_s_at | SPATS2 | spermatogenesis associated, serine-rich 2 | 1.92E-07 | 5.70595 |
| 220072_at | CSPP1 | centrosome and spindle pole associated protein 1 | 7.58E-07 | 5.70407 |
| 222759_at | SUV420H1 | suppressor of variegation 4-20 homolog 1 (Drosophila) | 1.85E-06 | 5.70275 |
| 211615_s_at | LRPPRC | leucine-rich PPR-motif containing | 8.40E-08 | 5.70266 |
| 208993_s_at | PPIG | peptidylprolyl isomerase G (cyclophilin G) | 2.72E-05 | 5.69982 |
| 202022_at | ALDOC | aldolase C, fructose-bisphosphate | 6.23E-07 | 5.6996 |
| 209160_at | AKR1C3 | aldo-keto reductase family 1, member C3 (3-alpha hydroxysteroid dehydrogenase, t | 6.68E-07 | 5.69885 |
| 203272_s_at | TUSC2 | tumor suppressor candidate 2 | 3.35E-05 | 5.69757 |
| 200011_s_at | ARF3 | ADP-ribosylation factor 3 | 5.41E-09 | 5.69737 |
| 204240_s_at | SMC2 | structural maintenance of chromosomes 2 | 2.91E-07 | 5.694 |
| 209143_s_at | CLNS1A | chloride channel, nucleotide-sensitive, 1A | 4.27E-07 | 5.69011 |
| 204524_at | PDPK1 | 3-phosphoinositide dependent protein kinase-1 | 4.15E-08 | 5.68672 |
| 225452_at | MED1 | mediator complex subunit 1 | 1.43E-05 | 5.68608 |
| 209669_s_at | SERBP1 | SERPINE1 mRNA binding protein 1 | 1.99E-09 | 5.68603 |
| 212763_at | CAMSAP1L1 | calmodulin regulated spectrin-associated protein 1-like 1 | 6.74E-06 | 5.68485 |
| 207845_s_at | ANAPC10 | anaphase promoting complex subunit 10 | 1.85E-05 | 5.68481 |
| 214752_x_at | FLNA | filamin A, alpha | 1.38E-07 | 5.683 |
| 202483_s_at | RANBP1 | RAN binding protein 1 | 1.24E-07 | 5.68282 |
| 1560587_s_at | PRDX5 | peroxiredoxin 5 | 4.91E-08 | 5.6828 |
| 223033_s_at | SCYL1 | SCY1-like 1 (S. cerevisiae) | 1.68E-06 | 5.68278 |
| 221499_s_at | STX16 | syntaxin 16 | 4.35E-07 | 5.68268 |
| 241908_at | C1orf58 | Chromosome 1 open reading frame 58 | 4.38E-06 | 5.68142 |
| 221580_s_at | TAF1D | TATA box binding protein (TBP)-associated factor, RNA polymerase I, D, 41kDa | 3.51E-05 | 5.68114 |
| 209430_at | BTAF1 | BTAF1 RNA polymerase II, B-TFIID transcription factor-associated, 170kDa (Mot1 h | 2.35E-06 | 5.67997 |
| 214595_at | KCNG1 | potassium voltage-gated channel, subfamily G, member 1 | 6.67E-06 | 5.67935 |
| 218503_at | KIAA1797 | KIAA1797 | 6.37E-07 | 5.67774 |
| 221704_s_at | VPS37B | vacuolar protein sorting 37 homolog B (S. cerevisiae) | 5.13E-08 | 5.6768 |
| 224935_at | EIF2S3 | eukaryotic translation initiation factor 2, subunit 3 gamma, 52kDa | 1.02E-05 | 5.67617 |
| 210028_s_at | ORC3L | origin recognition complex, subunit 3-like (yeast) | 1.78E-05 | 5.67577 |
| 210947_s_at | MSH3 | mutS homolog 3 (E. coli) | 8.50E-09 | 5.67199 |
| 222875_at | DHX33 | DEAH (Asp-Glu-Ala-His) box polypeptide 33 | 1.73E-08 | 5.67047 |
| 214764_at | RRP15 | ribosomal RNA processing 15 homolog (S. cerevisiae) | 5.36E-06 | 5.66846 |
| 203364_s_at | KIAA0652 | KIAA0652 | 1.56E-07 | 5.66653 |
| 234915_s_at | DENR | density-regulated protein | 5.33E-05 | 5.66565 |
| 200744_s_at | GNB1 | guanine nucleotide binding protein (G protein), beta polypeptide 1 | 4.76E-07 | 5.66333 |
| 202353_s_at | PSMD12 | proteasome (prosome, macropain) 26S subunit, non-ATPase, 12 | 1.00E-07 | 5.66285 |
| 218578_at | CDC73 | cell division cycle 73, Paf1/RNA polymerase II complex component, homolog (S. ce | 7.21E-07 | 5.66073 |
| 218289_s_at | UBA5 | ubiquitin-like modifier activating enzyme 5 | 5.27E-07 | 5.66026 |
| 203064_s_at | FOXK2 | forkhead box K2 | 5.32E-05 | 5.65965 |
| 224909_s_at | PREX1 | phosphatidylinositol-3,4,5-trisphosphate-dependent Rac exchange factor 1 | 1.03E-06 | 5.65928 |
| 201376_s_at | HNRNPF | heterogeneous nuclear ribonucleoprotein F | 5.46E-08 | 5.6537 |
| 202990_at | PYGL | phosphorylase, glycogen, liver | 9.22E-07 | 5.65191 |
| 222424_s_at | NUCKS1 | nuclear casein kinase and cyclin-dependent kinase substrate 1 | 4.64E-08 | 5.65057 |
| 221545_x_at | MED16 | mediator complex subunit 16 | 2.21E-05 | 5.65031 |
| 201736_s_at | 6-三月 | membrane-associated ring finger (C3HC4) 6 | 1.32E-06 | 5.65004 |
| 207332_s_at | TFRC | transferrin receptor (p90, CD71) | 6.38E-08 | 5.64986 |
| 219682_s_at | TBX3 | T-box 3 | 2.28E-08 | 5.64977 |
| 226370_at | KLHL15 | kelch-like 15 (Drosophila) | 1.21E-05 | 5.6496 |
| 222465_at | RSL24D1 | ribosomal L24 domain containing 1 | 2.30E-05 | 5.64881 |
| 213465_s_at | PPP1R7 | protein phosphatase 1, regulatory (inhibitor) subunit 7 | 3.10E-08 | 5.64781 |
| 204594_s_at | SMCR7L | Smith-Magenis syndrome chromosome region, candidate 7-like | 1.91E-07 | 5.64724 |
| 201703_s_at | PPP1R10 | protein phosphatase 1, regulatory (inhibitor) subunit 10 | 3.60E-06 | 5.64593 |
| 218147_s_at | GLT8D1 | glycosyltransferase 8 domain containing 1 | 5.49E-06 | 5.64434 |
| 201567_s_at | GOLGA4 | golgin A4 | 1.40E-05 | 5.64433 |
| 226285_at | CAPRIN1 | cell cycle associated protein 1 | 7.54E-06 | 5.64217 |
| 212785_s_at | LARP7 | La ribonucleoprotein domain family, member 7 | 2.78E-07 | 5.64082 |
| 227157_at | CCDC111 | coiled-coil domain containing 111 | 8.89E-09 | 5.6397 |
| 242560_at | FANCD2 | Fanconi anemia, complementation group D2 | 1.09E-05 | 5.63805 |
| 225011_at | PRKAR2A | Protein kinase, cAMP-dependent, regulatory, type II, alpha | 6.33E-08 | 5.63803 |
| 202996_at | POLD4 | polymerase (DNA-directed), delta 4 | 2.64E-06 | 5.63685 |
| 202131_s_at | RIOK3 | RIO kinase 3 (yeast) | 0.0001177 | 5.63527 |
| 203931_s_at | MRPL12 | mitochondrial ribosomal protein L12 | 1.71E-05 | 5.63486 |
| 228700_at | CXorf38 | chromosome X open reading frame 38 | 3.92E-05 | 5.63472 |
| 219192_at | UBAP2 | ubiquitin associated protein 2 | 9.63E-08 | 5.63361 |
| 228429_x_at | KIF9 | kinesin family member 9 | 1.31E-07 | 5.6321 |
| 1569110_x_at | LOC728613 | programmed cell death 6 pseudogene | 5.11E-07 | 5.63184 |
| 238477_at | KIF1C | kinesin family member 1C | 2.39E-06 | 5.63175 |
| 219193_at | WDR70 | WD repeat domain 70 | 4.62E-08 | 5.62928 |
| 204080_at | TOE1 | target of EGR1, member 1 (nuclear) | 1.70E-07 | 5.62859 |
| 219235_s_at | PHACTR4 | phosphatase and actin regulator 4 | 7.98E-07 | 5.62842 |
| 203007_x_at | LYPLA1 | lysophospholipase I | 9.03E-08 | 5.62757 |
| 225422_at | CDC26 | cell division cycle 26 homolog (S. cerevisiae) | 1.40E-07 | 5.62672 |
| 209003_at | SLC25A11 | solute carrier family 25 (mitochondrial carrier; oxoglutarate carrier), member 1 | 5.65E-07 | 5.62471 |
| 213934_s_at | ZNF23 | zinc finger protein 23 (KOX 16) | 2.79E-05 | 5.62228 |
| 226784_at | TWISTNB | TWIST neighbor | 2.28E-08 | 5.62213 |
| 212318_at | TNPO3 | transportin 3 | 2.05E-08 | 5.62187 |
| 223721_s_at | DNAJC12 | DnaJ (Hsp40) homolog, subfamily C, member 12 | 3.26E-06 | 5.6212 |
| 215030_at | GRSF1 | G-rich RNA sequence binding factor 1 | 9.15E-05 | 5.62063 |
| 235117_at | CHAC2 | ChaC, cation transport regulator homolog 2 (E. coli) | 5.26E-05 | 5.62005 |
| 216713_at | KRIT1 | KRIT1, ankyrin repeat containing | 1.03E-05 | 5.61967 |
| 1553551_s_at | LOC100131754 | similar to NADH dehydrogenase subunit 2 | 4.68E-05 | 5.61819 |
| 218909_at | RPS6KC1 | ribosomal protein S6 kinase, 52kDa, polypeptide 1 | 4.76E-07 | 5.61801 |
| 209836_x_at | BOLA2 /// BOLA2B | bolA homolog 2 (E. coli) /// bolA homolog 2B (E. coli) | 3.36E-06 | 5.61682 |
| 204028_s_at | RABGAP1 | RAB GTPase activating protein 1 | 6.61E-08 | 5.61391 |
| 212406_s_at | PCMTD2 | protein-L-isoaspartate (D-aspartate) O-methyltransferase domain containing 2 | 1.60E-06 | 5.61345 |
| 218993_at | RNMTL1 | RNA methyltransferase like 1 | 3.17E-06 | 5.61276 |
| 237465_at | USP53 | ubiquitin specific peptidase 53 | 1.19E-07 | 5.61231 |
| 225415_at | DTX3L | deltex 3-like (Drosophila) | 5.48E-07 | 5.61191 |
| 1554447_at | NCRNA00183 | non-protein coding RNA 183 | 9.64E-06 | 5.61072 |
| 228543_at | PET117 | cytochrome c oxidase assembly factor-like | 5.19E-06 | 5.61011 |
| 1553689_s_at | METTL6 | methyltransferase like 6 | 1.16E-07 | 5.60848 |
| 228114_x_at | C16orf13 | chromosome 16 open reading frame 13 | 1.12E-05 | 5.60779 |
| 200712_s_at | MAPRE1 | microtubule-associated protein, RP/EB family, member 1 | 6.04E-06 | 5.60617 |
| 206256_at | CPN1 | carboxypeptidase N, polypeptide 1 | 2.60E-07 | 5.60105 |
| 204490_s_at | CD44 | CD44 molecule (Indian blood group) | 2.26E-08 | 5.60076 |
| 219819_s_at | MRPS28 | mitochondrial ribosomal protein S28 | 2.29E-08 | 5.59922 |
| 210999_s_at | GRB10 | growth factor receptor-bound protein 10 | 1.79E-06 | 5.59756 |
| 225063_at | UBL7 | ubiquitin-like 7 (bone marrow stromal cell-derived) | 5.02E-08 | 5.59672 |
| 223700_at | MND1 | meiotic nuclear divisions 1 homolog (S. cerevisiae) | 0.0006821 | 5.59668 |
| 218422_s_at | RBM26 | RNA binding motif protein 26 | 1.25E-05 | 5.59507 |
| 223014_at | UBE2R2 | ubiquitin-conjugating enzyme E2R 2 | 4.37E-08 | 5.59447 |
| 207941_s_at | RBM39 | RNA binding motif protein 39 | 5.35E-07 | 5.59416 |
| 222685_at | HAUS6 | HAUS augmin-like complex, subunit 6 | 1.90E-05 | 5.59386 |
| 202916_s_at | FAM20B | family with sequence similarity 20, member B | 3.95E-05 | 5.5937 |
| 226054_at | BRD4 | bromodomain containing 4 | 2.02E-06 | 5.59297 |
| 205167_s_at | CDC25C | cell division cycle 25 homolog C (S. pombe) | 3.40E-05 | 5.59206 |
| 208968_s_at | CIAPIN1 | cytokine induced apoptosis inhibitor 1 | 1.37E-05 | 5.59177 |
| 209460_at | ABAT | 4-aminobutyrate aminotransferase | 3.56E-06 | 5.59128 |
| 202076_at | BIRC2 | baculoviral IAP repeat-containing 2 | 1.52E-06 | 5.59122 |
| 225344_at | NCOA7 | nuclear receptor coactivator 7 | 3.03E-06 | 5.59015 |
| 217553_at | MGC87042 | STEAP family protein MGC87042 | 3.72E-06 | 5.58983 |
| 222821_s_at | GEMIN7 | gem (nuclear organelle) associated protein 7 | 4.37E-06 | 5.58941 |
| 201009_s_at | TXNIP | thioredoxin interacting protein | 0.0001158 | 5.58592 |
| 213461_at | NUDT21 | nudix (nucleoside diphosphate linked moiety X)-type motif 21 | 2.38E-05 | 5.58425 |
| 225563_at | PAN3 | PAN3 poly(A) specific ribonuclease subunit homolog (S. cerevisiae) | 4.69E-06 | 5.58382 |
| 214937_x_at | PCM1 | pericentriolar material 1 | 5.72E-07 | 5.58357 |
| 202864_s_at | SP100 | SP100 nuclear antigen | 8.36E-06 | 5.58223 |
| 200959_at | FUS | fused in sarcoma | 1.68E-05 | 5.58134 |
| 225771_at | AP1G1 | adaptor-related protein complex 1, gamma 1 subunit | 1.04E-06 | 5.57647 |
| 225131_at | ZRANB1 | zinc finger, RAN-binding domain containing 1 | 7.42E-07 | 5.57628 |
| 223047_at | CMTM6 | CKLF-like MARVEL transmembrane domain containing 6 | 1.51E-06 | 5.5761 |
| 217978_s_at | UBE2Q1 | ubiquitin-conjugating enzyme E2Q family member 1 | 2.37E-06 | 5.57577 |
| 200934_at | DEK | DEK oncogene | 1.15E-06 | 5.5754 |
| 206034_at | SERPINB8 | serpin peptidase inhibitor, clade B (ovalbumin), member 8 | 8.31E-07 | 5.57467 |
| 222490_at | POLR3E | polymerase (RNA) III (DNA directed) polypeptide E (80kD) | 8.90E-05 | 5.57356 |
| 226431_at | FAM117B | family with sequence similarity 117, member B | 8.64E-06 | 5.57326 |
| 213145_at | FBXL14 | F-box and leucine-rich repeat protein 14 | 5.14E-08 | 5.5724 |
| 222684_s_at | NOL10 | nucleolar protein 10 | 2.92E-07 | 5.57168 |
| 201890_at | RRM2 | ribonucleotide reductase M2 | 2.65E-07 | 5.57074 |
| 210097_s_at | NOL7 | nucleolar protein 7, 27kDa | 4.47E-06 | 5.57007 |
| 201545_s_at | PABPN1 | poly(A) binding protein, nuclear 1 | 3.35E-05 | 5.56937 |
| 202374_s_at | RAB3GAP2 | RAB3 GTPase activating protein subunit 2 (non-catalytic) | 3.64E-06 | 5.56924 |
| 213773_x_at | NSUN5 | NOP2/Sun domain family, member 5 | 1.18E-07 | 5.56878 |
| 225655_at | UHRF1 | ubiquitin-like with PHD and ring finger domains 1 | 9.84E-08 | 5.56874 |
| 218320_s_at | NDUFB11 | NADH dehydrogenase (ubiquinone) 1 beta subcomplex, 11, 17.3kDa | 5.42E-08 | 5.56822 |
| 209130_at | SNAP23 | synaptosomal-associated protein, 23kDa | 8.99E-06 | 5.56677 |
| 212248_at | MTDH | metadherin | 2.03E-07 | 5.56506 |
| 201161_s_at | CSDA | cold shock domain protein A | 0.0001492 | 5.56434 |
| 1554747_a_at | 2-九月 | septin 2 | 3.68E-06 | 5.56387 |
| 222231_s_at | LRRC59 | leucine rich repeat containing 59 | 3.60E-08 | 5.56387 |
| 212405_s_at | METTL13 | methyltransferase like 13 | 8.75E-06 | 5.56358 |
| 203537_at | PRPSAP2 | phosphoribosyl pyrophosphate synthetase-associated protein 2 | 5.27E-06 | 5.56085 |
| 203519_s_at | UPF2 | UPF2 regulator of nonsense transcripts homolog (yeast) | 3.97E-06 | 5.56009 |
| 203594_at | RTCD1 | RNA terminal phosphate cyclase domain 1 | 4.65E-07 | 5.56008 |
| 212981_s_at | FAM115A | family with sequence similarity 115, member A | 1.31E-05 | 5.55972 |
| 203732_at | TRIP4 | thyroid hormone receptor interactor 4 | 1.09E-08 | 5.5594 |
| 209759_s_at | DCI | dodecenoyl-CoA isomerase | 2.46E-08 | 5.55848 |
| 203276_at | LMNB1 | lamin B1 | 8.04E-05 | 5.55721 |
| 225651_at | UBE2E2 | ubiquitin-conjugating enzyme E2E 2 (UBC4/5 homolog, yeast) | 0.0001467 | 5.55638 |
| 212414_s_at | GLYR1 /// SEPT6 | glyoxylate reductase 1 homolog (Arabidopsis) /// septin 6 | 3.10E-05 | 5.55572 |
| 229265_at | SKI | v-ski sarcoma viral oncogene homolog (avian) | 2.48E-07 | 5.55541 |
| 219053_s_at | VPS37C | vacuolar protein sorting 37 homolog C (S. cerevisiae) | 7.48E-06 | 5.55364 |
| 213754_s_at | LOC645139 /// PAIP1 | similar to poly(A) binding protein interacting protein 1 /// poly(A) binding pro | 2.25E-05 | 5.55308 |
| 203274_at | F8A1 /// F8A2 /// F8A3 | coagulation factor VIII-associated (intronic transcript) 1 /// coagulation facto | 4.60E-08 | 5.54983 |
| 201000_at | AARS | alanyl-tRNA synthetase | 1.20E-07 | 5.54714 |
| 222584_at | MSTO1 | misato homolog 1 (Drosophila) | 5.27E-06 | 5.54549 |
| 200921_s_at | BTG1 | B-cell translocation gene 1, anti-proliferative | 1.47E-07 | 5.54548 |
| 222488_s_at | DCTN4 | dynactin 4 (p62) | 4.70E-06 | 5.542 |
| 203517_at | MTX2 | metaxin 2 | 8.51E-07 | 5.54071 |
| 213786_at | TAX1BP1 | Tax1 (human T-cell leukemia virus type I) binding protein 1 | 7.60E-05 | 5.53882 |
| 201209_at | HDAC1 | histone deacetylase 1 | 1.75E-05 | 5.53723 |
| 212154_at | SDC2 | syndecan 2 | 1.84E-05 | 5.53398 |
| 223329_x_at | SUGT1 | SGT1, suppressor of G2 allele of SKP1 (S. cerevisiae) | 1.97E-06 | 5.5338 |
| 219299_at | TRMT12 | tRNA methyltransferase 12 homolog (S. cerevisiae) | 6.06E-09 | 5.53345 |
| 203195_s_at | NUP98 | nucleoporin 98kDa | 9.64E-08 | 5.53278 |
| 218724_s_at | TGIF2 | TGFB-induced factor homeobox 2 | 2.39E-06 | 5.52909 |
| 224760_at | SP1 | Sp1 transcription factor | 3.86E-07 | 5.5217 |
| 221692_s_at | MRPL34 | mitochondrial ribosomal protein L34 | 8.92E-05 | 5.51157 |
| 225947_at | MYO19 | myosin XIX | 4.32E-07 | 5.51145 |
| 229158_at | WNK4 | WNK lysine deficient protein kinase 4 | 2.53E-06 | 5.51112 |
| 227133_at | FAM199X | family with sequence similarity 199, X-linked | 2.59E-05 | 5.51052 |
| 216241_s_at | TCEA1 | transcription elongation factor A (SII), 1 | 5.35E-11 | 5.50849 |
| 224910_at | CARHSP1 | calcium regulated heat stable protein 1, 24kDa | 5.18E-07 | 5.50832 |
| 207405_s_at | RAD17 | RAD17 homolog (S. pombe) | 8.81E-06 | 5.50718 |
| 204092_s_at | AURKA | aurora kinase A | 7.51E-07 | 5.50686 |
| 228370_at | SNRPN | small nuclear ribonucleoprotein polypeptide N | 1.48E-05 | 5.50649 |
| 200767_s_at | FAM120A | family with sequence similarity 120A | 9.21E-06 | 5.50595 |
| 224582_s_at | NUCKS1 | nuclear casein kinase and cyclin-dependent kinase substrate 1 | 9.62E-06 | 5.50532 |
| 1554078_s_at | DNAJA3 | DnaJ (Hsp40) homolog, subfamily A, member 3 | 1.50E-07 | 5.50204 |
| 200695_at | PPP2R1A | protein phosphatase 2, regulatory subunit A, alpha | 1.67E-05 | 5.501 |
| 213065_at | ZFC3H1 | zinc finger, C3H1-type containing | 3.36E-07 | 5.49618 |
| 201581_at | TMX4 | thioredoxin-related transmembrane protein 4 | 1.37E-05 | 5.49547 |
| 223337_at | CWC27 | CWC27 spliceosome-associated protein homolog (S. cerevisiae) | 8.61E-08 | 5.49439 |
| 209189_at | FOS | FBJ murine osteosarcoma viral oncogene homolog | 3.46E-07 | 5.49407 |
| 203736_s_at | PPFIBP1 | PTPRF interacting protein, binding protein 1 (liprin beta 1) | 5.14E-06 | 5.49348 |
| 225890_at | C20orf72 | chromosome 20 open reading frame 72 | 5.92E-06 | 5.49339 |
| 219816_s_at | RBM23 | RNA binding motif protein 23 | 3.29E-05 | 5.49308 |
| 202388_at | RGS2 | regulator of G-protein signaling 2, 24kDa | 3.23E-07 | 5.49177 |
| 225127_at | TMEM181 | transmembrane protein 181 | 2.83E-07 | 5.49132 |
| 209300_s_at | NECAP1 | NECAP endocytosis associated 1 | 2.19E-05 | 5.49098 |
| 204279_at | PSMB9 | proteasome (prosome, macropain) subunit, beta type, 9 (large multifunctional pep | 4.19E-07 | 5.49092 |
| 213280_at | RAP1GAP2 | RAP1 GTPase activating protein 2 | 6.01E-06 | 5.49052 |
| 226909_at | ZNF518B | zinc finger protein 518B | 3.76E-05 | 5.49051 |
| 225335_at | ZNF496 | zinc finger protein 496 | 7.74E-08 | 5.49046 |
| 208814_at | HSPA4 | Heat shock 70kDa protein 4 | 0.0001098 | 5.48999 |
| 221473_x_at | SERINC3 | serine incorporator 3 | 1.49E-05 | 5.48828 |
| 224687_at | ANKIB1 | ankyrin repeat and IBR domain containing 1 | 1.42E-05 | 5.48587 |
| 222154_s_at | SPATS2L | spermatogenesis associated, serine-rich 2-like | 2.52E-10 | 5.48413 |
| 218360_at | RAB22A | RAB22A, member RAS oncogene family | 3.07E-07 | 5.48282 |
| 239377_at | EIF1AD | eukaryotic translation initiation factor 1A domain containing | 8.05E-07 | 5.48133 |
| 219289_at | HEATR3 | HEAT repeat containing 3 | 5.59E-07 | 5.48119 |
| 209433_s_at | PPAT | phosphoribosyl pyrophosphate amidotransferase | 1.73E-06 | 5.48103 |
| 222715_s_at | SYNRG | synergin, gamma | 1.69E-06 | 5.48057 |
| 55872_at | ZNF512B | zinc finger protein 512B | 1.74E-05 | 5.4777 |
| 202757_at | COBRA1 | cofactor of BRCA1 | 1.19E-06 | 5.47512 |
| 202153_s_at | NUP62 | nucleoporin 62kDa | 2.88E-07 | 5.47254 |
| 200647_x_at | EIF3C /// EIF3CL | eukaryotic translation initiation factor 3, subunit C /// eukaryotic translation | 9.85E-08 | 5.4724 |
| 218046_s_at | MRPS16 | mitochondrial ribosomal protein S16 | 8.14E-08 | 5.47229 |
| 223093_at | ANKH | ankylosis, progressive homolog (mouse) | 1.39E-05 | 5.47176 |
| 204178_s_at | RBM14 | RNA binding motif protein 14 | 2.52E-07 | 5.46816 |
| 212877_at | KLC1 | kinesin light chain 1 | 1.50E-05 | 5.46753 |
| 217971_at | MAPKSP1 | MAPK scaffold protein 1 | 6.45E-07 | 5.46588 |
| 201649_at | UBE2L6 | ubiquitin-conjugating enzyme E2L 6 | 3.99E-07 | 5.46438 |
| 210235_s_at | PPFIA1 | protein tyrosine phosphatase, receptor type, f polypeptide (PTPRF), interacting | 4.27E-07 | 5.46409 |
| 212271_at | MAPK1 | mitogen-activated protein kinase 1 | 9.85E-06 | 5.46403 |
| 201018_at | EIF1AX | eukaryotic translation initiation factor 1A, X-linked | 9.71E-08 | 5.46386 |
| 225410_at | C2orf64 | chromosome 2 open reading frame 64 | 9.64E-10 | 5.46251 |
| 1569594_a_at | SDCCAG1 | serologically defined colon cancer antigen 1 | 3.48E-06 | 5.46133 |
| 202703_at | DUSP11 | dual specificity phosphatase 11 (RNA/RNP complex 1-interacting) | 7.19E-06 | 5.461 |
| 222737_s_at | BRD7 | bromodomain containing 7 | 7.52E-06 | 5.45975 |
| 201458_s_at | BUB3 | budding uninhibited by benzimidazoles 3 homolog (yeast) | 6.50E-07 | 5.45708 |
| 202348_s_at | TOR1A | torsin family 1, member A (torsin A) | 1.17E-06 | 5.45651 |
| 235791_x_at | CHD1 | chromodomain helicase DNA binding protein 1 | 3.22E-06 | 5.45626 |
| 223175_s_at | FEM1A | fem-1 homolog a (C. elegans) | 4.41E-07 | 5.45574 |
| 218479_s_at | XPO4 | exportin 4 | 0.0001024 | 5.45539 |
| 219156_at | SYNJ2BP | synaptojanin 2 binding protein | 2.40E-07 | 5.45167 |
| 218167_at | AMZ2 | archaelysin family metallopeptidase 2 | 5.92E-07 | 5.44941 |
| 202347_s_at | UBE2K | ubiquitin-conjugating enzyme E2K (UBC1 homolog, yeast) | 1.52E-05 | 5.44921 |
| 1552364_s_at | MSI2 | musashi homolog 2 (Drosophila) | 2.35E-05 | 5.44851 |
| 213205_s_at | RAD54L2 | RAD54-like 2 (S. cerevisiae) | 3.00E-07 | 5.44785 |
| 209067_s_at | HNRPDL | heterogeneous nuclear ribonucleoprotein D-like | 1.82E-05 | 5.44724 |
| 203207_s_at | MTFR1 | mitochondrial fission regulator 1 | 4.78E-07 | 5.44546 |
| 203266_s_at | MAP2K4 | mitogen-activated protein kinase kinase 4 | 0.0001746 | 5.44239 |
| 233642_s_at | HEATR5B | HEAT repeat containing 5B | 1.72E-06 | 5.44206 |
| 202093_s_at | PAF1 | Paf1, RNA polymerase II associated factor, homolog (S. cerevisiae) | 6.22E-06 | 5.4399 |
| 200690_at | HSPA9 | heat shock 70kDa protein 9 (mortalin) | 4.27E-06 | 5.43988 |
| 200797_s_at | MCL1 | myeloid cell leukemia sequence 1 (BCL2-related) | 1.34E-07 | 5.43843 |
| 212846_at | RRP1B | ribosomal RNA processing 1 homolog B (S. cerevisiae) | 2.22E-06 | 5.42946 |
| 208775_at | XPO1 | exportin 1 (CRM1 homolog, yeast) | 6.58E-09 | 5.42946 |
| 225336_at | SFRS2IP | splicing factor, arginine/serine-rich 2, interacting protein | 5.22E-09 | 5.42847 |
| 225296_at | ZNF317 | zinc finger protein 317 | 1.36E-06 | 5.42679 |
| 221911_at | ETV1 | ets variant 1 | 8.76E-06 | 5.4264 |
| 204113_at | CELF1 | CUGBP, Elav-like family member 1 | 6.44E-08 | 5.42602 |
| 210465_s_at | SNAPC3 | small nuclear RNA activating complex, polypeptide 3, 50kDa | 1.17E-05 | 5.42569 |
| 218624_s_at | MGC2752 | hypothetical LOC65996 | 1.08E-05 | 5.4219 |
| 38158_at | ESPL1 | extra spindle pole bodies homolog 1 (S. cerevisiae) | 5.38E-09 | 5.41965 |
| 208787_at | MRPL3 | mitochondrial ribosomal protein L3 | 2.11E-08 | 5.41533 |
| 218455_at | NFS1 | NFS1 nitrogen fixation 1 homolog (S. cerevisiae) | 0.0007941 | 5.41531 |
| 40465_at | DDX23 | DEAD (Asp-Glu-Ala-Asp) box polypeptide 23 | 2.47E-07 | 5.41357 |
| 212373_at | FEM1B | fem-1 homolog b (C. elegans) | 7.68E-05 | 5.41235 |
| 1567458_s_at | RAC1 | ras-related C3 botulinum toxin substrate 1 (rho family, small GTP binding protei | 3.93E-05 | 5.41216 |
| 208631_s_at | HADHA | hydroxyacyl-CoA dehydrogenase/3-ketoacyl-CoA thiolase/enoyl-CoA hydratase (trifu | 1.81E-07 | 5.41114 |
| 218212_s_at | MOCS2 | molybdenum cofactor synthesis 2 | 9.13E-06 | 5.4099 |
| 221598_s_at | MED27 | mediator complex subunit 27 | 1.86E-07 | 5.40805 |
| 229106_at | DYNLL2 | dynein, light chain, LC8-type 2 | 2.96E-06 | 5.40747 |
| 201086_x_at | SON | SON DNA binding protein | 2.90E-06 | 5.40735 |
| 223818_s_at | RSF1 | remodeling and spacing factor 1 | 1.77E-05 | 5.40712 |
| 208967_s_at | AK2 | adenylate kinase 2 | 4.50E-09 | 5.40701 |
| 201956_s_at | GNPAT | glyceronephosphate O-acyltransferase | 6.91E-09 | 5.40638 |
| 223538_at | SERF1A | small EDRK-rich factor 1A (telomeric) | 1.48E-05 | 5.40505 |
| 222406_s_at | PNRC2 | proline-rich nuclear receptor coactivator 2 | 5.75E-05 | 5.40468 |
| 221693_s_at | MRPS18A | mitochondrial ribosomal protein S18A | 1.27E-06 | 5.40425 |
| 221934_s_at | DALRD3 | DALR anticodon binding domain containing 3 | 4.21E-08 | 5.40281 |
| 233255_s_at | BIVM | basic, immunoglobulin-like variable motif containing | 1.96E-05 | 5.40217 |
| 229674_at | SERTAD4 | SERTA domain containing 4 | 1.30E-08 | 5.40213 |
| 202318_s_at | SENP6 | SUMO1/sentrin specific peptidase 6 | 4.24E-05 | 5.40047 |
| 201031_s_at | HNRNPH1 | heterogeneous nuclear ribonucleoprotein H1 (H) | 1.11E-06 | 5.39924 |
| 218112_at | MRPS34 | mitochondrial ribosomal protein S34 | 1.00E-06 | 5.39875 |
| 226298_at | RUNDC1 | RUN domain containing 1 | 6.43E-06 | 5.39855 |
| 218616_at | INTS12 | integrator complex subunit 12 | 5.49E-06 | 5.39653 |
| 200654_at | P4HB | prolyl 4-hydroxylase, beta polypeptide | 7.55E-07 | 5.39466 |
| 200698_at | KDELR2 | KDEL (Lys-Asp-Glu-Leu) endoplasmic reticulum protein retention receptor 2 | 3.46E-07 | 5.39377 |
| 229061_s_at | SLC25A13 | solute carrier family 25, member 13 (citrin) | 4.77E-06 | 5.39266 |
| 212246_at | MCFD2 | multiple coagulation factor deficiency 2 | 8.09E-06 | 5.3916 |
| 226558_at | LOC389834 | ankyrin repeat domain 57 pseudogene | 4.40E-07 | 5.38945 |
| 222601_at | UBA6 | ubiquitin-like modifier activating enzyme 6 | 7.20E-06 | 5.38933 |
| 204540_at | EEF1A2 | eukaryotic translation elongation factor 1 alpha 2 | 6.56E-05 | 5.38648 |
| 200728_at | ACTR2 | ARP2 actin-related protein 2 homolog (yeast) | 8.12E-07 | 5.38625 |
| 223489_x_at | EXOSC3 | exosome component 3 | 5.93E-06 | 5.38614 |
| 214151_s_at | CCPG1 | cell cycle progression 1 | 2.49E-07 | 5.38453 |
| 219433_at | BCOR | BCL6 co-repressor | 0.0002333 | 5.38368 |
| 219303_at | RNF219 | ring finger protein 219 | 2.87E-06 | 5.38317 |
| 219686_at | STK32B | serine/threonine kinase 32B | 4.67E-07 | 5.38097 |
| 221257_x_at | FBXO38 | F-box protein 38 | 3.89E-06 | 5.37901 |
| 224524_s_at | ASB3 /// GPR75 /// LOC100302652 | ankyrin repeat and SOCS box-containing 3 /// G protein-coupled receptor 75 /// G | 3.30E-07 | 5.37751 |
| 223542_at | ANKRD32 | ankyrin repeat domain 32 | 3.55E-06 | 5.37607 |
| 225032_at | FNDC3B | fibronectin type III domain containing 3B | 8.17E-06 | 5.37568 |
| 1565951_s_at | CHML | choroideremia-like (Rab escort protein 2) | 1.17E-07 | 5.37527 |
| 236344_at | PDE1C | phosphodiesterase 1C, calmodulin-dependent 70kDa | 1.18E-05 | 5.37477 |
| 200800_s_at | HSPA1A /// HSPA1B | heat shock 70kDa protein 1A /// heat shock 70kDa protein 1B | 9.65E-09 | 5.37414 |
| 235122_at | HIVEP3 | human immunodeficiency virus type I enhancer binding protein 3 | 6.73E-06 | 5.37412 |
| 207830_s_at | PPP1R8 | protein phosphatase 1, regulatory (inhibitor) subunit 8 | 8.09E-06 | 5.37403 |
| 218682_s_at | SLC4A1AP | solute carrier family 4 (anion exchanger), member 1, adaptor protein | 1.78E-05 | 5.37269 |
| 227212_s_at | PHF19 | PHD finger protein 19 | 7.16E-06 | 5.37126 |
| 222369_at | NAA40 | N(alpha)-acetyltransferase 40, NatD catalytic subunit, homolog (S. cerevisiae) | 9.53E-06 | 5.37095 |
| 230645_at | FRMD3 | FERM domain containing 3 | 5.67E-05 | 5.37047 |
| 219306_at | KIF15 | kinesin family member 15 | 1.10E-06 | 5.37044 |
| 214221_at | ALMS1 | Alstrom syndrome 1 | 2.39E-06 | 5.37009 |
| 212756_s_at | UBR2 | ubiquitin protein ligase E3 component n-recognin 2 | 8.70E-07 | 5.36732 |
| 219324_at | NOL12 /// TRIOBP | nucleolar protein 12 /// TRIO and F-actin binding protein | 9.83E-07 | 5.36617 |
| 223539_s_at | SERF1A /// SERF1B | small EDRK-rich factor 1A (telomeric) /// small EDRK-rich factor 1B (centromeric | 1.97E-06 | 5.36388 |
| 218067_s_at | ARGLU1 | arginine and glutamate rich 1 | 3.97E-06 | 5.36375 |
| 225451_at | GRIPAP1 | GRIP1 associated protein 1 | 1.58E-07 | 5.3636 |
| 200857_s_at | NCOR1 | nuclear receptor co-repressor 1 | 1.00E-05 | 5.36297 |
| 226100_at | MLL5 | myeloid/lymphoid or mixed-lineage leukemia 5 (trithorax homolog, Drosophila) | 1.69E-05 | 5.36249 |
| 229845_at | MAPKAP1 | mitogen-activated protein kinase associated protein 1 | 4.93E-05 | 5.36175 |
| 215253_s_at | RCAN1 | regulator of calcineurin 1 | 9.49E-05 | 5.36089 |
| 44669_at | SDHAF1 | succinate dehydrogenase complex assembly factor 1 | 2.79E-07 | 5.35976 |
| 218199_s_at | NOL6 | nucleolar protein family 6 (RNA-associated) | 7.22E-08 | 5.35964 |
| 209186_at | ATP2A2 | ATPase, Ca++ transporting, cardiac muscle, slow twitch 2 | 2.45E-07 | 5.35917 |
| 206138_s_at | PI4KB | phosphatidylinositol 4-kinase, catalytic, beta | 3.09E-07 | 5.35776 |
| 223707_at | RPL27A | ribosomal protein L27a | 0.0001128 | 5.35769 |
| 223145_s_at | AKIRIN2 | akirin 2 | 8.78E-09 | 5.35713 |
| 201698_s_at | SFRS9 | splicing factor, arginine/serine-rich 9 | 2.21E-09 | 5.35659 |
| 226052_at | BRD4 | bromodomain containing 4 | 1.29E-07 | 5.35538 |
| 224844_at | SLAIN2 | SLAIN motif family, member 2 | 6.80E-07 | 5.35403 |
| 208620_at | PCBP1 | poly(rC) binding protein 1 | 2.49E-07 | 5.35308 |
| 218575_at | ANAPC1 | anaphase promoting complex subunit 1 | 4.60E-05 | 5.35215 |
| 201845_s_at | RYBP | RING1 and YY1 binding protein | 4.58E-07 | 5.3515 |
| 227079_at | DHX8 | DEAH (Asp-Glu-Ala-His) box polypeptide 8 | 7.40E-07 | 5.34905 |
| 212673_at | METAP1 | methionyl aminopeptidase 1 | 7.54E-06 | 5.34843 |
| 217879_at | CDC27 | cell division cycle 27 homolog (S. cerevisiae) | 1.03E-05 | 5.34687 |
| 208973_at | ERI3 | ERI1 exoribonuclease family member 3 | 6.61E-08 | 5.34416 |
| 209662_at | CETN3 | centrin, EF-hand protein, 3 (CDC31 homolog, yeast) | 6.52E-05 | 5.34374 |
| 202626_s_at | LYN | v-yes-1 Yamaguchi sarcoma viral related oncogene homolog | 1.60E-07 | 5.34288 |
| 209350_s_at | GPS2 | G protein pathway suppressor 2 | 0.000157 | 5.34278 |
| 238479_at | DCUN1D5 | DCN1, defective in cullin neddylation 1, domain containing 5 (S. cerevisiae) | 8.47E-06 | 5.34097 |
| 227376_at | GLI3 | GLI family zinc finger 3 | 1.22E-06 | 5.33916 |
| 235174_s_at | LOC100128822 | hypothetical LOC100128822 | 2.16E-05 | 5.33868 |
| 1556060_a_at | ZNF451 | zinc finger protein 451 | 5.21E-06 | 5.33793 |
| 218617_at | TRIT1 | tRNA isopentenyltransferase 1 | 3.06E-07 | 5.33773 |
| 1554020_at | BICD1 | bicaudal D homolog 1 (Drosophila) | 3.74E-06 | 5.3376 |
| 224581_s_at | NUCKS1 | nuclear casein kinase and cyclin-dependent kinase substrate 1 | 1.24E-10 | 5.33695 |
| 206499_s_at | RCC1 /// SNHG3-RCC1 | regulator of chromosome condensation 1 /// SNHG3-RCC1 readthrough transcript | 2.35E-05 | 5.33476 |
| 211987_at | TOP2B | topoisomerase (DNA) II beta 180kDa | 2.45E-07 | 5.33334 |
| 222753_s_at | SPCS3 | signal peptidase complex subunit 3 homolog (S. cerevisiae) | 5.62E-08 | 5.33228 |
| 226193_x_at | CBWD1 | COBW domain containing 1 | 4.78E-08 | 5.3322 |
| 226012_at | ANKRD11 | ankyrin repeat domain 11 | 1.98E-05 | 5.33158 |
| 212009_s_at | STIP1 | stress-induced-phosphoprotein 1 | 3.27E-06 | 5.32857 |
| 201889_at | FAM3C | family with sequence similarity 3, member C | 6.65E-05 | 5.3277 |
| 226764_at | ZNF827 | zinc finger protein 827 | 9.73E-07 | 5.32466 |
| 222402_at | POMP | proteasome maturation protein | 0.0001198 | 5.3246 |
| 202718_at | IGFBP2 | insulin-like growth factor binding protein 2, 36kDa | 5.29E-06 | 5.32399 |
| 222512_at | NUB1 | negative regulator of ubiquitin-like proteins 1 | 5.54E-07 | 5.32324 |
| 227357_at | TAB3 | TGF-beta activated kinase 1/MAP3K7 binding protein 3 | 1.63E-06 | 5.32074 |
| 202882_x_at | NOL7 | nucleolar protein 7, 27kDa | 3.53E-08 | 5.3178 |
| 218838_s_at | TTC31 | tetratricopeptide repeat domain 31 | 8.16E-05 | 5.31577 |
| 218240_at | NKIRAS2 | NFKB inhibitor interacting Ras-like 2 | 3.96E-07 | 5.31499 |
| 226503_at | RIF1 | RAP1 interacting factor homolog (yeast) | 5.64E-05 | 5.31302 |
| 1555882_at | SPIN3 | spindlin family, member 3 | 5.17E-06 | 5.31285 |
| 31807_at | DDX49 | DEAD (Asp-Glu-Ala-Asp) box polypeptide 49 | 1.65E-06 | 5.31118 |
| 202272_s_at | FBXO28 | F-box protein 28 | 1.37E-07 | 5.31101 |
| 225808_at | C17orf95 | chromosome 17 open reading frame 95 | 2.54E-07 | 5.30975 |
| 213980_s_at | CTBP1 | C-terminal binding protein 1 | 5.76E-07 | 5.30884 |
| 229113_s_at | C1orf86 | chromosome 1 open reading frame 86 | 4.84E-05 | 5.30791 |
| 223128_at | FOXRED1 | FAD-dependent oxidoreductase domain containing 1 | 2.16E-07 | 5.3047 |
| 226049_at | ERC1 | ELKS/RAB6-interacting/CAST family member 1 | 2.11E-08 | 5.30469 |
| 200969_at | SERP1 | stress-associated endoplasmic reticulum protein 1 | 2.32E-07 | 5.30432 |
| 203342_at | TIMM17B | translocase of inner mitochondrial membrane 17 homolog B (yeast) | 2.79E-07 | 5.3043 |
| 209128_s_at | SART3 | squamous cell carcinoma antigen recognized by T cells 3 | 1.45E-05 | 5.30187 |
| 203380_x_at | SFRS5 | splicing factor, arginine/serine-rich 5 | 9.24E-05 | 5.30159 |
| 226642_s_at | NUDCD2 | NudC domain containing 2 | 3.51E-06 | 5.30095 |
| 223283_s_at | TSHZ1 | teashirt zinc finger homeobox 1 | 2.27E-07 | 5.30095 |
| 211358_s_at | CIZ1 | CDKN1A interacting zinc finger protein 1 | 6.66E-05 | 5.29866 |
| 202565_s_at | SVIL | supervillin | 1.93E-07 | 5.29756 |
| 219571_s_at | ZNF12 | zinc finger protein 12 | 0.0002048 | 5.29597 |
| 201878_at | ARIH1 | ariadne homolog, ubiquitin-conjugating enzyme E2 binding protein, 1 (Drosophila) | 0.0001158 | 5.29584 |
| 223165_s_at | IP6K2 | inositol hexakisphosphate kinase 2 | 3.56E-06 | 5.29232 |
| 213637_at | DDX52 | DEAD (Asp-Glu-Ala-Asp) box polypeptide 52 | 1.37E-07 | 5.29146 |
| 32069_at | N4BP1 | NEDD4 binding protein 1 | 5.57E-06 | 5.29113 |
| 218163_at | MCTS1 | malignant T cell amplified sequence 1 | 2.61E-07 | 5.29073 |
| 226116_at | DFFA | DNA fragmentation factor, 45kDa, alpha polypeptide | 3.18E-07 | 5.28971 |
| 205219_s_at | GALK2 | galactokinase 2 | 5.37E-06 | 5.28904 |
| 204812_at | ZW10 | ZW10, kinetochore associated, homolog (Drosophila) | 4.50E-08 | 5.28387 |
| 200662_s_at | TOMM20 | translocase of outer mitochondrial membrane 20 homolog (yeast) | 9.04E-08 | 5.28356 |
| 224468_s_at | C19orf48 | chromosome 19 open reading frame 48 | 4.60E-08 | 5.28193 |
| 222671_s_at | JMJD4 | jumonji domain containing 4 | 1.88E-06 | 5.28185 |
| 223996_s_at | MRPL30 | mitochondrial ribosomal protein L30 | 1.05E-05 | 5.28104 |
| 209078_s_at | TXN2 | thioredoxin 2 | 1.64E-08 | 5.2805 |
| 225634_at | ZC3HAV1 | zinc finger CCCH-type, antiviral 1 | 0.0001859 | 5.27887 |
| 225138_at | ZRANB1 | zinc finger, RAN-binding domain containing 1 | 9.68E-06 | 5.27774 |
| 214512_s_at | SUB1 | SUB1 homolog (S. cerevisiae) | 4.59E-06 | 5.27728 |
| 212683_at | SLC25A44 | solute carrier family 25, member 44 | 6.12E-05 | 5.27682 |
| 223055_s_at | XPO5 | exportin 5 | 2.01E-07 | 5.27664 |
| 223758_s_at | GTF2H2 | general transcription factor IIH, polypeptide 2, 44kDa | 0.0001821 | 5.27552 |
| 201572_x_at | DCTD | dCMP deaminase | 1.22E-07 | 5.27509 |
| 209457_at | DUSP5 | dual specificity phosphatase 5 | 2.31E-07 | 5.27508 |
| 203871_at | SENP3 | SUMO1/sentrin/SMT3 specific peptidase 3 | 2.03E-06 | 5.27445 |
| 212529_at | LSM12 | LSM12 homolog (S. cerevisiae) | 5.59E-06 | 5.27356 |
| 226154_at | DNM1L | dynamin 1-like | 2.38E-05 | 5.27351 |
| 201155_s_at | MFN2 | mitofusin 2 | 2.74E-06 | 5.27116 |
| 200874_s_at | NOP56 | NOP56 ribonucleoprotein homolog (yeast) | 1.07E-05 | 5.26802 |
| 223598_at | RAD23B | RAD23 homolog B (S. cerevisiae) | 1.73E-05 | 5.26773 |
| 213604_at | TCEB3 | transcription elongation factor B (SIII), polypeptide 3 (110kDa, elongin A) | 8.22E-05 | 5.26656 |
| 217865_at | RNF130 | ring finger protein 130 | 3.57E-08 | 5.26644 |
| 218579_s_at | DHX35 | DEAH (Asp-Glu-Ala-His) box polypeptide 35 | 7.10E-06 | 5.26528 |
| 225951_s_at | CHD2 | Chromodomain helicase DNA binding protein 2 | 5.94E-05 | 5.26331 |
| 233173_x_at | GTF3C5 | general transcription factor IIIC, polypeptide 5, 63kDa | 7.83E-07 | 5.26277 |
| 233907_s_at | SERTAD4 | SERTA domain containing 4 | 1.36E-05 | 5.26219 |
| 225934_at | LOC148413 | hypothetical LOC148413 | 9.14E-07 | 5.26207 |
| 213839_at | CLMN | calmin (calponin-like, transmembrane) | 1.83E-05 | 5.26203 |
| 221229_s_at | TRMT61B | tRNA methyltransferase 61 homolog B (S. cerevisiae) | 0.0003915 | 5.25902 |
| 1553993_s_at | MED25 | mediator complex subunit 25 | 1.16E-06 | 5.2586 |
| 212835_at | FAM175B | family with sequence similarity 175, member B | 1.54E-05 | 5.25618 |
| 226141_at | CCDC149 | coiled-coil domain containing 149 | 8.28E-06 | 5.25566 |
| 202421_at | IGSF3 | immunoglobulin superfamily, member 3 | 7.18E-07 | 5.25496 |
| 202591_s_at | SSBP1 | single-stranded DNA binding protein 1 | 5.62E-08 | 5.25483 |
| 223040_at | NAA20 | N(alpha)-acetyltransferase 20, NatB catalytic subunit | 3.11E-08 | 5.25454 |
| 214629_x_at | RTN4 | reticulon 4 | 2.64E-07 | 5.25418 |
| 218672_at | SCNM1 | sodium channel modifier 1 | 7.49E-06 | 5.25413 |
| 217529_at | ORAI2 | ORAI calcium release-activated calcium modulator 2 | 4.63E-05 | 5.25406 |
| 226331_at | BBX | bobby sox homolog (Drosophila) | 4.45E-07 | 5.25349 |
| 215031_x_at | RNF126 | ring finger protein 126 | 7.34E-07 | 5.25189 |
| 223216_x_at | ZNF395 | zinc finger protein 395 | 1.95E-06 | 5.25163 |
| 204699_s_at | C1orf107 | chromosome 1 open reading frame 107 | 9.94E-05 | 5.24651 |
| 218440_at | MCCC1 | methylcrotonoyl-CoA carboxylase 1 (alpha) | 1.80E-05 | 5.246 |
| 207515_s_at | POLR1C | polymerase (RNA) I polypeptide C, 30kDa | 4.75E-08 | 5.245 |
| 216526_x_at | HLA-C | major histocompatibility complex, class I, C | 5.28E-07 | 5.2446 |
| 225362_at | FAM122B | family with sequence similarity 122B | 8.53E-06 | 5.2424 |
| 209323_at | PRKRIR | protein-kinase, interferon-inducible double stranded RNA dependent inhibitor, re | 3.09E-05 | 5.24172 |
| 227812_at | TNFRSF19 | tumor necrosis factor receptor superfamily, member 19 | 1.06E-06 | 5.24139 |
| 221123_x_at | ZNF395 | zinc finger protein 395 | 1.69E-07 | 5.2401 |
| 235195_at | FBXW2 | F-box and WD repeat domain containing 2 | 0.0002651 | 5.23993 |
| 209529_at | PPAP2C | phosphatidic acid phosphatase type 2C | 4.10E-06 | 5.23971 |
| 224740_at | C5orf43 | chromosome 5 open reading frame 43 | 0.0001433 | 5.23896 |
| 212745_s_at | BBS4 | Bardet-Biedl syndrome 4 | 2.89E-07 | 5.23742 |
| 222748_s_at | TXNL4B | thioredoxin-like 4B | 5.81E-07 | 5.23702 |
| 235054_at | NUDT16 | nudix (nucleoside diphosphate linked moiety X)-type motif 16 | 2.66E-08 | 5.23444 |
| 221669_s_at | ACAD8 | acyl-CoA dehydrogenase family, member 8 | 5.69E-05 | 5.23187 |
| 223107_s_at | ZCCHC17 | zinc finger, CCHC domain containing 17 | 3.42E-08 | 5.23006 |
| 205335_s_at | SRP19 | signal recognition particle 19kDa | 3.22E-07 | 5.2295 |
| 227374_at | EARS2 | glutamyl-tRNA synthetase 2, mitochondrial (putative) | 3.63E-06 | 5.22944 |
| 213427_at | RPP40 | ribonuclease P/MRP 40kDa subunit | 6.10E-08 | 5.22689 |
| 226713_at | CCDC50 | coiled-coil domain containing 50 | 1.42E-06 | 5.22582 |
| 213528_at | C1orf156 | chromosome 1 open reading frame 156 | 5.50E-07 | 5.22274 |
| 204015_s_at | DUSP4 | dual specificity phosphatase 4 | 2.47E-07 | 5.22175 |
| 201191_at | PITPNA | phosphatidylinositol transfer protein, alpha | 5.77E-06 | 5.21926 |
| 203194_s_at | NUP98 | nucleoporin 98kDa | 1.24E-06 | 5.21919 |
| 201520_s_at | GRSF1 | G-rich RNA sequence binding factor 1 | 1.22E-07 | 5.21887 |
| 223193_x_at | FAM162A | family with sequence similarity 162, member A | 6.13E-09 | 5.21866 |
| 200083_at | USP22 | ubiquitin specific peptidase 22 | 6.49E-09 | 5.21843 |
| 227748_at | RBMXL1 | RNA binding motif protein, X-linked-like 1 | 0.0001551 | 5.2182 |
| 203612_at | BYSL | bystin-like | 9.65E-05 | 5.21711 |
| 205733_at | BLM | Bloom syndrome, RecQ helicase-like | 5.05E-07 | 5.21699 |
| 205932_s_at | MSX1 | msh homeobox 1 | 1.57E-05 | 5.21693 |
| 201584_s_at | DDX39 | DEAD (Asp-Glu-Ala-Asp) box polypeptide 39 | 1.06E-07 | 5.21571 |
| 221069_s_at | TACO1 | translational activator of mitochondrially encoded cytochrome c oxidase I | 9.57E-06 | 5.21541 |
| 201129_at | SFRS7 | splicing factor, arginine/serine-rich 7, 35kDa | 1.20E-08 | 5.21346 |
| 213682_at | NUP50 | nucleoporin 50kDa | 6.27E-08 | 5.21316 |
| 212266_s_at | SFRS5 | splicing factor, arginine/serine-rich 5 | 4.10E-05 | 5.21313 |
| 207621_s_at | PEMT | phosphatidylethanolamine N-methyltransferase | 1.90E-07 | 5.21294 |
| 212074_at | SUN1 | Sad1 and UNC84 domain containing 1 | 6.95E-07 | 5.21252 |
| 212794_s_at | KIAA1033 | KIAA1033 | 8.69E-06 | 5.21101 |
| 1554390_s_at | ACTR2 | ARP2 actin-related protein 2 homolog (yeast) | 3.39E-06 | 5.20687 |
| 202149_at | NEDD9 | neural precursor cell expressed, developmentally down-regulated 9 | 4.49E-07 | 5.20667 |
| 227196_at | RHPN2 | rhophilin, Rho GTPase binding protein 2 | 1.71E-07 | 5.2066 |
| 201424_s_at | CUL4A | cullin 4A | 3.25E-05 | 5.20504 |
| 224600_at | CGGBP1 | CGG triplet repeat binding protein 1 | 7.75E-06 | 5.20364 |
| 208248_x_at | APLP2 | amyloid beta (A4) precursor-like protein 2 | 1.62E-05 | 5.20278 |
| 203622_s_at | PNO1 | partner of NOB1 homolog (S. cerevisiae) | 1.09E-07 | 5.20212 |
| 202361_at | SEC24C | SEC24 family, member C (S. cerevisiae) | 1.40E-05 | 5.20179 |
| 208824_x_at | CDK16 | cyclin-dependent kinase 16 | 8.46E-06 | 5.19974 |
| 218512_at | WDR12 | WD repeat domain 12 | 2.74E-06 | 5.19712 |
| 218938_at | FBXL15 | F-box and leucine-rich repeat protein 15 | 5.28E-05 | 5.19667 |
| 221736_at | RALGAPB | Ral GTPase activating protein, beta subunit (non-catalytic) | 1.15E-05 | 5.1963 |
| 222006_at | LETM1 | leucine zipper-EF-hand containing transmembrane protein 1 | 8.71E-08 | 5.19484 |
| 221498_at | SNX27 | sorting nexin family member 27 | 4.64E-06 | 5.19483 |
| 203851_at | IGFBP6 | insulin-like growth factor binding protein 6 | 7.56E-06 | 5.19383 |
| 209317_at | POLR1C | polymerase (RNA) I polypeptide C, 30kDa | 0.0001156 | 5.19372 |
| 202123_s_at | ABL1 | c-abl oncogene 1, receptor tyrosine kinase | 1.49E-05 | 5.19303 |
| 211989_at | SMARCE1 | SWI/SNF related, matrix associated, actin dependent regulator of chromatin, subf | 8.32E-07 | 5.19266 |
| 201493_s_at | PUM2 | pumilio homolog 2 (Drosophila) | 1.31E-07 | 5.19192 |
| 1555948_s_at | FAM120A | family with sequence similarity 120A | 0.0001716 | 5.19112 |
| 223151_at | DCUN1D5 | DCN1, defective in cullin neddylation 1, domain containing 5 (S. cerevisiae) | 7.16E-07 | 5.18969 |
| 209526_s_at | HDGFRP3 | hepatoma-derived growth factor, related protein 3 | 9.53E-07 | 5.18944 |
| 235570_at | RBMS3 | RNA binding motif, single stranded interacting protein 3 | 7.53E-06 | 5.18936 |
| 223295_s_at | LUC7L | LUC7-like (S. cerevisiae) | 2.50E-06 | 5.18762 |
| 200596_s_at | EIF3A | eukaryotic translation initiation factor 3, subunit A | 5.35E-07 | 5.18538 |
| 209084_s_at | RAB28 | RAB28, member RAS oncogene family | 2.24E-07 | 5.18461 |
| 223268_at | C11orf54 | chromosome 11 open reading frame 54 | 1.49E-05 | 5.18445 |
| 202577_s_at | DDX19A | DEAD (Asp-Glu-Ala-As) box polypeptide 19A | 1.40E-05 | 5.18382 |
| 225371_at | GLE1 | GLE1 RNA export mediator homolog (yeast) | 3.46E-06 | 5.18371 |
| 217234_s_at | EZR | ezrin | 2.24E-06 | 5.18088 |
| 203880_at | COX17 | COX17 cytochrome c oxidase assembly homolog (S. cerevisiae) | 2.65E-08 | 5.17859 |
| 200626_s_at | MATR3 | matrin 3 | 1.43E-05 | 5.17768 |
| 224885_s_at | KRTCAP2 | keratinocyte associated protein 2 | 2.96E-07 | 5.17561 |
| 223978_s_at | CRLS1 | cardiolipin synthase 1 | 6.37E-05 | 5.17377 |
| 212099_at | RHOB | ras homolog gene family, member B | 4.31E-06 | 5.17179 |
| 219178_at | QTRTD1 | queuine tRNA-ribosyltransferase domain containing 1 | 1.26E-05 | 5.16992 |
| 213471_at | NPHP4 | nephronophthisis 4 | 7.10E-05 | 5.16588 |
| 1552937_s_at | ATRIP | ATR interacting protein | 4.86E-06 | 5.16533 |
| 225583_at | UXS1 | UDP-glucuronate decarboxylase 1 | 0.0002757 | 5.16493 |
| 201619_at | PRDX3 | peroxiredoxin 3 | 7.76E-07 | 5.16457 |
| 203387_s_at | TBC1D4 | TBC1 domain family, member 4 | 8.11E-06 | 5.16408 |
| 202723_s_at | FOXO1 | forkhead box O1 | 1.40E-05 | 5.16376 |
| 219469_at | DYNC2H1 | dynein, cytoplasmic 2, heavy chain 1 | 1.67E-06 | 5.16325 |
| 223411_at | MIF4GD | MIF4G domain containing | 1.96E-06 | 5.16275 |
| 211609_x_at | PSMD4 | proteasome (prosome, macropain) 26S subunit, non-ATPase, 4 | 5.90E-09 | 5.1618 |
| 202598_at | S100A13 | S100 calcium binding protein A13 | 1.31E-06 | 5.15976 |
| 225068_at | KLHL12 | kelch-like 12 (Drosophila) | 1.17E-09 | 5.15932 |
| 202322_s_at | GGPS1 | geranylgeranyl diphosphate synthase 1 | 7.41E-07 | 5.15914 |
| 208424_s_at | CIAPIN1 | cytokine induced apoptosis inhibitor 1 | 2.30E-06 | 5.1589 |
| 223247_at | MED10 | mediator complex subunit 10 | 2.60E-06 | 5.15879 |
| 221761_at | ADSS | adenylosuccinate synthase | 5.97E-08 | 5.15837 |
| 202576_s_at | DDX19A /// DDX19B | DEAD (Asp-Glu-Ala-As) box polypeptide 19A /// DEAD (Asp-Glu-Ala-As) box polypept | 7.76E-07 | 5.15835 |
| 225304_s_at | NDUFA11 | NADH dehydrogenase (ubiquinone) 1 alpha subcomplex, 11, 14.7kDa | 3.35E-06 | 5.15783 |
| 227784_s_at | COG1 | component of oligomeric golgi complex 1 | 6.04E-05 | 5.15721 |
| 222578_s_at | UBA5 | ubiquitin-like modifier activating enzyme 5 | 1.37E-08 | 5.15606 |
| 225150_s_at | RTKN | rhotekin | 8.04E-06 | 5.15576 |
| 217990_at | GMPR2 | guanosine monophosphate reductase 2 | 0.0004348 | 5.15539 |
| 225694_at | CDK12 | cyclin-dependent kinase 12 | 1.01E-05 | 5.15439 |
| 204604_at | CDK14 | cyclin-dependent kinase 14 | 3.85E-07 | 5.15253 |
| 217926_at | C19orf53 | chromosome 19 open reading frame 53 | 1.44E-08 | 5.15252 |
| 229650_s_at | C19orf42 | chromosome 19 open reading frame 42 | 1.05E-06 | 5.14928 |
| 221021_s_at | CTNNBL1 | catenin, beta like 1 | 5.65E-06 | 5.14906 |
| 221536_s_at | LSG1 | large subunit GTPase 1 homolog (S. cerevisiae) | 3.98E-06 | 5.14712 |
| 208952_s_at | LARP4B | La ribonucleoprotein domain family, member 4B | 3.93E-06 | 5.14328 |
| 234294_x_at | GATAD2A | GATA zinc finger domain containing 2A | 1.45E-06 | 5.1428 |
| 200988_s_at | PSME3 | proteasome (prosome, macropain) activator subunit 3 (PA28 gamma; Ki) | 6.01E-07 | 5.14195 |
| 213330_s_at | STIP1 | stress-induced-phosphoprotein 1 | 2.15E-07 | 5.14095 |
| 212245_at | MCFD2 | multiple coagulation factor deficiency 2 | 6.80E-06 | 5.14071 |
| 225889_at | AEBP2 | AE binding protein 2 | 5.69E-05 | 5.14067 |
| 223104_at | JAGN1 | jagunal homolog 1 (Drosophila) | 2.28E-07 | 5.14015 |
| 223479_s_at | CHCHD5 | coiled-coil-helix-coiled-coil-helix domain containing 5 | 0.0004465 | 5.13914 |
| 228920_at | ZNF260 | zinc finger protein 260 | 2.23E-07 | 5.13888 |
| 211563_s_at | C19orf2 | chromosome 19 open reading frame 2 | 1.67E-05 | 5.13873 |
| 223198_x_at | COMMD5 | COMM domain containing 5 | 7.11E-07 | 5.13584 |
| 37950_at | PREP | prolyl endopeptidase | 4.53E-06 | 5.13496 |
| 204639_at | ADA | adenosine deaminase | 1.32E-06 | 5.13471 |
| 227242_s_at | EBF3 | early B-cell factor 3 | 1.44E-05 | 5.13456 |
| 218968_s_at | ZFP64 | zinc finger protein 64 homolog (mouse) | 1.42E-07 | 5.13437 |
| 203577_at | GTF2H4 | general transcription factor IIH, polypeptide 4, 52kDa | 0.0001333 | 5.13403 |
| 200779_at | ATF4 | activating transcription factor 4 (tax-responsive enhancer element B67) | 8.04E-07 | 5.13337 |
| 218349_s_at | ZWILCH | Zwilch, kinetochore associated, homolog (Drosophila) | 1.71E-05 | 5.13148 |
| 220992_s_at | C1orf25 | chromosome 1 open reading frame 25 | 9.61E-07 | 5.13124 |
| 204147_s_at | TFDP1 | transcription factor Dp-1 | 6.36E-06 | 5.12834 |
| 226108_at | ZC3H18 | zinc finger CCCH-type containing 18 | 1.38E-07 | 5.12707 |
| 202092_s_at | ARL2BP | ADP-ribosylation factor-like 2 binding protein | 5.37E-07 | 5.12595 |
| 227093_at | USP36 | Ubiquitin specific peptidase 36 | 3.08E-07 | 5.12576 |
| 209984_at | KDM4C | lysine (K)-specific demethylase 4C | 5.30E-05 | 5.12433 |
| 203298_s_at | JARID2 | jumonji, AT rich interactive domain 2 | 2.04E-06 | 5.12291 |
| 218239_s_at | GTPBP4 | GTP binding protein 4 | 3.40E-05 | 5.12219 |
| 206302_s_at | NUDT4 /// NUDT4P1 | nudix (nucleoside diphosphate linked moiety X)-type motif 4 /// nudix (nucleosid | 1.38E-06 | 5.12196 |
| 202494_at | PPIE | peptidylprolyl isomerase E (cyclophilin E) | 1.40E-06 | 5.12073 |
| 218388_at | PGLS | 6-phosphogluconolactonase | 2.04E-07 | 5.12054 |
| 208906_at | BSCL2 | Berardinelli-Seip congenital lipodystrophy 2 (seipin) | 1.82E-07 | 5.12016 |
| 225231_at | CBL | Cas-Br-M (murine) ecotropic retroviral transforming sequence | 4.37E-06 | 5.11929 |
| 209388_at | PAPOLA | poly(A) polymerase alpha | 6.69E-05 | 5.1192 |
| 221434_s_at | C14orf156 | chromosome 14 open reading frame 156 | 9.04E-10 | 5.11881 |
| 202031_s_at | WIPI2 | WD repeat domain, phosphoinositide interacting 2 | 1.53E-07 | 5.11704 |
| 1554576_a_at | ETV4 | ets variant 4 | 1.16E-05 | 5.11645 |
| 211797_s_at | NFYC | nuclear transcription factor Y, gamma | 9.82E-06 | 5.11636 |
| 212527_at | PPPDE2 | PPPDE peptidase domain containing 2 | 4.58E-07 | 5.11636 |
| 204862_s_at | NME3 | non-metastatic cells 3, protein expressed in | 6.39E-07 | 5.11291 |
| 202243_s_at | PSMB4 | proteasome (prosome, macropain) subunit, beta type, 4 | 2.99E-06 | 5.11249 |
| 200844_s_at | PRDX6 | peroxiredoxin 6 | 1.48E-06 | 5.11248 |
| 212165_at | TMEM183A /// TMEM183B | transmembrane protein 183A /// transmembrane protein 183B | 7.40E-08 | 5.11184 |
| 226020_s_at | DAB1 /// OMA1 | disabled homolog 1 (Drosophila) /// OMA1 homolog, zinc metallopeptidase (S. cere | 4.86E-06 | 5.11183 |
| 223369_at | METTL11A | methyltransferase like 11A | 8.91E-07 | 5.11159 |
| 202610_s_at | MED14 | mediator complex subunit 14 | 3.84E-06 | 5.11148 |
| 209746_s_at | COQ7 | coenzyme Q7 homolog, ubiquinone (yeast) | 1.57E-06 | 5.11099 |
| 219214_s_at | NT5C | 5', 3'-nucleotidase, cytosolic | 6.38E-06 | 5.11073 |
| 213286_at | ZFR | zinc finger RNA binding protein | 0.0005819 | 5.11001 |
| 206085_s_at | CTH | cystathionase (cystathionine gamma-lyase) | 2.34E-06 | 5.10699 |
| 203235_at | THOP1 | thimet oligopeptidase 1 | 6.08E-06 | 5.10684 |
| 218441_s_at | RPAP1 | RNA polymerase II associated protein 1 | 9.96E-06 | 5.10632 |
| 201010_s_at | TXNIP | thioredoxin interacting protein | 4.55E-07 | 5.10612 |
| 201122_x_at | EIF5A | eukaryotic translation initiation factor 5A | 2.14E-05 | 5.10394 |
| 212738_at | ARHGAP19 | Rho GTPase activating protein 19 | 2.14E-06 | 5.10376 |
| 213876_x_at | ZRSR2 | zinc finger (CCCH type), RNA-binding motif and serine/arginine rich 2 | 2.25E-05 | 5.10309 |
| 203013_at | ECD | ecdysoneless homolog (Drosophila) | 2.90E-06 | 5.10283 |
| 214263_x_at | POLR2C | polymerase (RNA) II (DNA directed) polypeptide C, 33kDa | 1.54E-06 | 5.10262 |
| 219494_at | RAD54B | RAD54 homolog B (S. cerevisiae) | 2.15E-06 | 5.10255 |
| 213165_at | CEP350 | centrosomal protein 350kDa | 2.68E-06 | 5.10133 |
| 203200_s_at | MTRR | 5-methyltetrahydrofolate-homocysteine methyltransferase reductase | 8.81E-07 | 5.09974 |
| 218287_s_at | EIF2C1 | eukaryotic translation initiation factor 2C, 1 | 8.38E-08 | 5.09915 |
| 200764_s_at | CTNNA1 | catenin (cadherin-associated protein), alpha 1, 102kDa | 1.90E-06 | 5.09902 |
| 210567_s_at | SKP2 | S-phase kinase-associated protein 2 (p45) | 1.74E-05 | 5.09837 |
| 202692_s_at | UBTF | upstream binding transcription factor, RNA polymerase I | 6.63E-05 | 5.09615 |
| 213019_at | RANBP6 | RAN binding protein 6 | 1.87E-05 | 5.09339 |
| 202511_s_at | ATG5 | ATG5 autophagy related 5 homolog (S. cerevisiae) | 0.0001122 | 5.09288 |
| 205661_s_at | FLAD1 | FAD1 flavin adenine dinucleotide synthetase homolog (S. cerevisiae) | 1.44E-06 | 5.09256 |
| 229115_at | DYNC1H1 | dynein, cytoplasmic 1, heavy chain 1 | 0.0002234 | 5.08907 |
| 206067_s_at | WT1 | Wilms tumor 1 | 1.93E-05 | 5.08809 |
| 222130_s_at | FTSJ2 | FtsJ homolog 2 (E. coli) | 7.92E-07 | 5.08786 |
| 218355_at | KIF4A | kinesin family member 4A | 8.54E-06 | 5.08612 |
| 201509_at | IDH3B | isocitrate dehydrogenase 3 (NAD+) beta | 8.96E-07 | 5.08592 |
| 230251_at | C6orf176 | chromosome 6 open reading frame 176 | 1.90E-06 | 5.08523 |
| 220175_s_at | CBWD1 /// CBWD2 /// CBWD3 /// CBWD5 /// CBWD6 /// CBWD7 | COBW domain containing 1 /// COBW domain containing 2 /// COBW domain containing | 4.44E-06 | 5.08511 |
| 60474_at | FERMT1 | fermitin family homolog 1 (Drosophila) | 3.52E-06 | 5.08289 |
| 1554627_a_at | ASCC1 | activating signal cointegrator 1 complex subunit 1 | 3.12E-06 | 5.08237 |
| 213298_at | NFIC | nuclear factor I/C (CCAAT-binding transcription factor) | 1.64E-06 | 5.08075 |
| 230532_at | CXorf38 | chromosome X open reading frame 38 | 9.89E-07 | 5.07733 |
| 226327_at | ZNF507 | zinc finger protein 507 | 2.59E-08 | 5.07723 |
| 212556_at | SCRIB | scribbled homolog (Drosophila) | 6.92E-07 | 5.07658 |
| 209605_at | TST | thiosulfate sulfurtransferase (rhodanese) | 2.61E-07 | 5.07552 |
| 225118_at | SETD8 | SET domain containing (lysine methyltransferase) 8 | 7.80E-05 | 5.07352 |
| 218669_at | RAP2C | RAP2C, member of RAS oncogene family | 3.66E-08 | 5.07319 |
| 227636_at | THAP5 | THAP domain containing 5 | 1.14E-05 | 5.0725 |
| 218365_s_at | DARS2 | aspartyl-tRNA synthetase 2, mitochondrial | 2.89E-06 | 5.07207 |
| 230528_s_at | MGC2752 | hypothetical LOC65996 | 8.30E-07 | 5.07196 |
| 1568864_at | LOC100131691 | hypothetical protein LOC100131691 | 1.65E-05 | 5.07187 |
| 212399_s_at | VGLL4 | vestigial like 4 (Drosophila) | 2.96E-06 | 5.07118 |
| 201184_s_at | CHD4 | chromodomain helicase DNA binding protein 4 | 1.36E-07 | 5.07095 |
| 228347_at | SIX1 | SIX homeobox 1 | 4.94E-05 | 5.06924 |
| 223982_s_at | PNPLA8 | patatin-like phospholipase domain containing 8 | 4.70E-05 | 5.06804 |
| 214052_x_at | BAT2L2 | HLA-B associated transcript 2-like 2 | 4.07E-05 | 5.06752 |
| 201083_s_at | BCLAF1 | BCL2-associated transcription factor 1 | 0.0002296 | 5.06655 |
| 226943_at | C12orf73 | chromosome 12 open reading frame 73 | 1.12E-06 | 5.06232 |
| 221847_at | LOC100129361 | similar to mCG115122 | 1.28E-06 | 5.06178 |
| 205281_s_at | PIGA | phosphatidylinositol glycan anchor biosynthesis, class A | 7.28E-09 | 5.06084 |
| 219129_s_at | SAP30L | SAP30-like | 6.25E-07 | 5.06058 |
| 219133_at | OXSM | 3-oxoacyl-ACP synthase, mitochondrial | 9.02E-06 | 5.05927 |
| 225294_s_at | TRAPPC1 | trafficking protein particle complex 1 | 3.44E-05 | 5.05924 |
| 223067_at | CWC15 | CWC15 spliceosome-associated protein homolog (S. cerevisiae) | 2.33E-07 | 5.05811 |
| 203093_s_at | TIMM44 | translocase of inner mitochondrial membrane 44 homolog (yeast) | 2.59E-07 | 5.05553 |
| 227481_at | CNKSR3 | CNKSR family member 3 | 1.01E-06 | 5.05351 |
| 209512_at | HSDL2 | hydroxysteroid dehydrogenase like 2 | 9.93E-05 | 5.05275 |
| 1568596_a_at | TROAP | trophinin associated protein (tastin) | 4.95E-08 | 5.05272 |
| 214459_x_at | HLA-C | major histocompatibility complex, class I, C | 1.31E-06 | 5.05198 |
| 208830_s_at | SUPT6H | suppressor of Ty 6 homolog (S. cerevisiae) | 1.27E-05 | 5.05177 |
| 225037_at | SLC35C2 | solute carrier family 35, member C2 | 1.83E-06 | 5.05165 |
| 210186_s_at | FKBP1A | FK506 binding protein 1A, 12kDa | 5.92E-07 | 5.05093 |
| 220934_s_at | TMEM223 | transmembrane protein 223 | 1.15E-05 | 5.05063 |
| 225169_at | INTS4 | integrator complex subunit 4 | 1.43E-06 | 5.04845 |
| 211962_s_at | ZFP36L1 | zinc finger protein 36, C3H type-like 1 | 0.0002485 | 5.04811 |
| 224387_at | COMMD5 | COMM domain containing 5 | 6.65E-06 | 5.04759 |
| 219161_s_at | CKLF | chemokine-like factor | 6.07E-07 | 5.04588 |
| 207304_at | ZNF45 | zinc finger protein 45 | 2.48E-06 | 5.04364 |
| 229632_s_at | INTS10 | integrator complex subunit 10 | 5.20E-07 | 5.04164 |
| 218973_at | EFTUD1 | elongation factor Tu GTP binding domain containing 1 | 9.28E-06 | 5.04162 |
| 224699_s_at | ESYT2 | extended synaptotagmin-like protein 2 | 9.77E-07 | 5.04072 |
| 222697_s_at | ABHD10 | abhydrolase domain containing 10 | 4.12E-05 | 5.03913 |
| 228291_s_at | PLK1S1 | polo-like kinase 1 substrate 1 | 0.0002061 | 5.03866 |
| 223614_at | MMP16 | matrix metallopeptidase 16 (membrane-inserted) | 2.04E-07 | 5.03676 |
| 204507_s_at | PPP3R1 | protein phosphatase 3, regulatory subunit B, alpha | 6.18E-05 | 5.03623 |
| 235561_at | TXNL1 | thioredoxin-like 1 | 2.12E-06 | 5.03433 |
| 208074_s_at | AP2S1 | adaptor-related protein complex 2, sigma 1 subunit | 3.10E-06 | 5.03432 |
| 217839_at | TFG | TRK-fused gene | 1.37E-07 | 5.03401 |
| 226433_at | RNF157 | ring finger protein 157 | 1.65E-07 | 5.03242 |
| 209899_s_at | PUF60 | poly-U binding splicing factor 60KDa | 3.18E-07 | 5.03089 |
| 1553960_at | SNX21 | sorting nexin family member 21 | 9.31E-06 | 5.02787 |
| 222425_s_at | POLDIP2 | polymerase (DNA-directed), delta interacting protein 2 | 3.37E-06 | 5.02715 |
| 227008_at | HDDC3 | HD domain containing 3 | 7.66E-07 | 5.02681 |
| 205394_at | CHEK1 | CHK1 checkpoint homolog (S. pombe) | 4.38E-06 | 5.02645 |
| 201659_s_at | ARL1 | ADP-ribosylation factor-like 1 | 6.15E-09 | 5.0264 |
| 213140_s_at | SS18L1 | synovial sarcoma translocation gene on chromosome 18-like 1 | 2.44E-06 | 5.02598 |
| 201880_at | ARIH1 | ariadne homolog, ubiquitin-conjugating enzyme E2 binding protein, 1 (Drosophila) | 1.72E-07 | 5.02581 |
| 226082_s_at | SFRS15 | splicing factor, arginine/serine-rich 15 | 8.24E-06 | 5.02577 |
| 218568_at | AGK | acylglycerol kinase | 1.41E-06 | 5.02479 |
| 53968_at | INTS5 | integrator complex subunit 5 | 1.44E-07 | 5.02444 |
| 221215_s_at | RIPK4 | receptor-interacting serine-threonine kinase 4 | 2.74E-06 | 5.02407 |
| 219207_at | EDC3 | enhancer of mRNA decapping 3 homolog (S. cerevisiae) | 7.87E-07 | 5.02379 |
| 202892_at | CDC23 | cell division cycle 23 homolog (S. cerevisiae) | 2.32E-06 | 5.02311 |
| 214112_s_at | CXorf40A /// CXorf40B | chromosome X open reading frame 40A /// chromosome X open reading frame 40B | 8.91E-08 | 5.02308 |
| 218089_at | C20orf4 | chromosome 20 open reading frame 4 | 3.89E-05 | 5.02164 |
| 226262_at | DHX33 | DEAH (Asp-Glu-Ala-His) box polypeptide 33 | 6.24E-08 | 5.02137 |
| 223001_at | OSTC | oligosaccharyltransferase complex subunit | 4.99E-07 | 5.0212 |
| 229268_at | FAM105B | family with sequence similarity 105, member B | 3.74E-06 | 5.02105 |
| 218671_s_at | ATPIF1 | ATPase inhibitory factor 1 | 2.15E-07 | 5.0205 |
| 201040_at | GNAI2 | guanine nucleotide binding protein (G protein), alpha inhibiting activity polype | 2.15E-07 | 5.0177 |
| 1556242_a_at | LOC100310756 | hypothetical LOC100310756 | 6.50E-09 | 5.01482 |
| 202191_s_at | GAS7 | growth arrest-specific 7 | 5.58E-08 | 5.01455 |
| 213237_at | C16orf88 | chromosome 16 open reading frame 88 | 7.58E-07 | 5.0134 |
| 213599_at | OIP5 | Opa interacting protein 5 | 3.23E-06 | 5.01331 |
| 212137_at | LARP1 | La ribonucleoprotein domain family, member 1 | 2.66E-07 | 5.0127 |
| 223282_at | TSHZ1 | teashirt zinc finger homeobox 1 | 3.85E-06 | 5.01102 |
| 216640_s_at | PDIA6 | protein disulfide isomerase family A, member 6 | 2.19E-08 | 5.01068 |
| 216305_s_at | C2orf3 | chromosome 2 open reading frame 3 | 3.52E-05 | 5.01016 |
| 203386_at | TBC1D4 | TBC1 domain family, member 4 | 2.07E-06 | 5.00828 |
| 225876_at | NIPAL3 | NIPA-like domain containing 3 | 9.41E-05 | 5.00703 |
| 213161_at | TMOD1 /// TSTD2 | tropomodulin 1 /// thiosulfate sulfurtransferase (rhodanese)-like domain contain | 3.46E-07 | 5.00688 |
| 226758_at | LUC7L2 | LUC7-like 2 (S. cerevisiae) | 1.17E-07 | 5.00556 |
| 212825_at | PAXIP1 | PAX interacting (with transcription-activation domain) protein 1 | 1.46E-06 | 5.00407 |
| 202220_at | KIAA0907 | KIAA0907 | 0.000105 | 5.00391 |
| 1558097_at | C22orf30 | chromosome 22 open reading frame 30 | 4.73E-05 | 5.00307 |
| 201860_s_at | PLAT | plasminogen activator, tissue | 5.44E-05 | 5.00278 |
| 217370_x_at | FUS | fused in sarcoma | 1.08E-07 | 5.00209 |
| 223318_s_at | ALKBH7 | alkB, alkylation repair homolog 7 (E. coli) | 0.0002438 | 5.00192 |
| 225910_at | HELZ | helicase with zinc finger | 4.94E-06 | 5.00038 |
| 229253_at | THEM4 | thioesterase superfamily member 4 | 1.86E-05 | 5.00026 |
| 201294_s_at | WSB1 | WD repeat and SOCS box-containing 1 | 9.80E-05 | -5.00154 |
| 228502_at | RPS6KA6 | ribosomal protein S6 kinase, 90kDa, polypeptide 6 | 0.0005043 | -5.00802 |
| 1552865_a_at | IFLTD1 | intermediate filament tail domain containing 1 | 1.32E-05 | -5.01051 |
| 242711_x_at | FANCM | Fanconi anemia, complementation group M | 4.59E-07 | -5.01469 |
| 213702_x_at | ASAH1 | N-acylsphingosine amidohydrolase (acid ceramidase) 1 | 1.74E-07 | -5.04433 |
| 210964_s_at | GYG2 | glycogenin 2 | 1.08E-06 | -5.04702 |
| 235155_at | BDH2 | 3-hydroxybutyrate dehydrogenase, type 2 | 1.14E-05 | -5.04759 |
| 244395_at | FLJ41455 | hypothetical gene supported by AK123449; BX641014 | 0.0005186 | -5.05052 |
| 206169_x_at | ZC3H7B | zinc finger CCCH-type containing 7B | 0.0005796 | -5.05282 |
| 229084_at | CNTN4 | contactin 4 | 0.0001246 | -5.06657 |
| 220591_s_at | EFHC2 | EF-hand domain (C-terminal) containing 2 | 1.63E-06 | -5.0705 |
| 220608_s_at | ZNF770 | zinc finger protein 770 | 3.63E-05 | -5.07527 |
| 227253_at | CP | ceruloplasmin (ferroxidase) | 3.11E-06 | -5.07671 |
| 214120_at | RFPL1S | RFPL1 antisense RNA (non-protein coding) | 1.12E-05 | -5.07674 |
| 211568_at | BAI3 | brain-specific angiogenesis inhibitor 3 | 1.14E-05 | -5.07934 |
| 225897_at | MARCKS | myristoylated alanine-rich protein kinase C substrate | 7.40E-07 | -5.08251 |
| 244350_at | MYO10 | myosin X | 1.66E-06 | -5.09144 |
| 243998_at | KRT222 | keratin 222 | 1.27E-06 | -5.10407 |
| 215228_at | NHLH2 | nescient helix loop helix 2 | 1.11E-05 | -5.10999 |
| 1557548_at | C10orf108 | chromosome 10 open reading frame 108 | 4.84E-06 | -5.1584 |
| 1558467_a_at | UGGT2 | UDP-glucose glycoprotein glucosyltransferase 2 | 2.01E-12 | -5.15907 |
| 224005_at | LOC100289550 | Similar to hCG1724266 | 3.30E-06 | -5.16933 |
| 1557618_at | LOC285768 | hypothetical LOC285768 | 6.39E-05 | -5.17353 |
| 215446_s_at | LOX | lysyl oxidase | 7.89E-07 | -5.17624 |
| 244353_s_at | SLC2A12 | solute carrier family 2 (facilitated glucose transporter), member 12 | 1.86E-06 | -5.18019 |
| 236160_at | TRIP11 | thyroid hormone receptor interactor 11 | 1.60E-05 | -5.19579 |
| 229073_at | PRTG | protogenin homolog (Gallus gallus) | 8.89E-05 | -5.19925 |
| 227835_at | LOC389831 | Hypothetical gene supported by AL713796 | 0.0001574 | -5.20161 |
| 1559224_at | LCE1E | late cornified envelope 1E | 5.35E-05 | -5.20272 |
| 215573_at | CAT | Catalase | 3.85E-05 | -5.20616 |
| 205893_at | NLGN1 | neuroligin 1 | 7.18E-06 | -5.20892 |
| 220115_s_at | CDH10 | cadherin 10, type 2 (T2-cadherin) | 8.66E-07 | -5.20921 |
| 215513_at | HYMAI | hydatidiform mole associated and imprinted (non-protein coding) | 2.54E-06 | -5.21175 |
| 220405_at | SNTG1 | syntrophin, gamma 1 | 1.12E-05 | -5.22009 |
| 1556183_at | FLJ40330 | hypothetical LOC645784 | 3.55E-05 | -5.22551 |
| 242197_x_at | CD36 | CD36 molecule (thrombospondin receptor) | 5.07E-07 | -5.22591 |
| 1570007_at | LRRC8C | leucine rich repeat containing 8 family, member C | 1.55E-06 | -5.22779 |
| 1554141_s_at | WDR78 | WD repeat domain 78 | 8.30E-06 | -5.23181 |
| 1564093_at | NEK1 | NIMA (never in mitosis gene a)-related kinase 1 | 1.91E-05 | -5.23688 |
| 1554038_at | LARP1B | La ribonucleoprotein domain family, member 1B | 5.88E-07 | -5.24519 |
| 211617_at | ALDOAP2 | aldolase A, fructose-bisphosphate pseudogene 2 | 5.19E-06 | -5.24848 |
| 236283_x_at | LOC646214 | p21-activated kinase 2 pseudogene | 0.000473 | -5.26148 |
| 211585_at | NPAT | nuclear protein, ataxia-telangiectasia locus | 5.02E-06 | -5.26768 |
| 209614_at | ADH1B | alcohol dehydrogenase 1B (class I), beta polypeptide | 1.01E-05 | -5.27125 |
| 230518_at | MPZL2 | myelin protein zero-like 2 | 0.0001013 | -5.2722 |
| 211368_s_at | CASP1 | caspase 1, apoptosis-related cysteine peptidase (interleukin 1, beta, convertase | 3.72E-06 | -5.27565 |
| 206639_x_at | HTN1 | histatin 1 | 7.38E-05 | -5.27841 |
| 221601_s_at | FAIM3 | Fas apoptotic inhibitory molecule 3 | 6.16E-09 | -5.2927 |
| 1570048_at | DNAJC24 | DnaJ (Hsp40) homolog, subfamily C, member 24 | 2.01E-06 | -5.31001 |
| 232073_at | PPFIA2 | protein tyrosine phosphatase, receptor type, f polypeptide (PTPRF), interacting | 0.0001473 | -5.32039 |
| 220703_at | C10orf110 | chromosome 10 open reading frame 110 | 5.08E-06 | -5.3309 |
| 208305_at | PGR | progesterone receptor | 8.24E-07 | -5.35066 |
| 236029_at | FAT3 | FAT tumor suppressor homolog 3 (Drosophila) | 8.98E-06 | -5.35785 |
| 1561225_at | LOC338579 | hypothetical protein LOC338579 | 1.76E-06 | -5.36313 |
| 1557770_at | IPO11 | importin 11 | 1.28E-05 | -5.36435 |
| 206960_at | LPAR4 | lysophosphatidic acid receptor 4 | 1.30E-05 | -5.36501 |
| 1554855_at | PARK2 | Parkinson disease (autosomal recessive, juvenile) 2, parkin | 6.98E-06 | -5.39893 |
| 243254_at | HIVEP2 | human immunodeficiency virus type I enhancer binding protein 2 | 7.43E-08 | -5.40817 |
| 1562439_at | NCOA3 | Nuclear receptor coactivator 3 | 3.22E-05 | -5.41433 |
| 239487_at | FAM98A | Family with sequence similarity 98, member A | 2.82E-06 | -5.41948 |
| 227235_at | GUCY1A3 | guanylate cyclase 1, soluble, alpha 3 | 6.55E-06 | -5.42649 |
| 1554528_at | C3orf15 | chromosome 3 open reading frame 15 | 9.88E-06 | -5.42701 |
| 1566190_at | SUZ12 | Suppressor of zeste 12 homolog (Drosophila) | 3.29E-05 | -5.43074 |
| 229975_at | BMPR1B | bone morphogenetic protein receptor, type IB | 4.42E-07 | -5.43381 |
| 1561884_at | CEPT1 | choline/ethanolamine phosphotransferase 1 | 7.92E-06 | -5.43605 |
| 206797_at | NAT2 | N-acetyltransferase 2 (arylamine N-acetyltransferase) | 8.23E-05 | -5.45042 |
| 236181_at | LOC389831 | Hypothetical gene supported by AL713796 | 0.0006153 | -5.45066 |
| 229994_at | NFIA | Nuclear factor I/A | 5.41E-06 | -5.46804 |
| 201893_x_at | DCN | decorin | 1.85E-05 | -5.47051 |
| 230644_at | LRFN5 | leucine rich repeat and fibronectin type III domain containing 5 | 2.39E-07 | -5.4883 |
| 234655_at | LOC100290132 | hypothetical protein LOC100290132 | 5.72E-05 | -5.49821 |
| 228614_at | NCRNA00116 | non-protein coding RNA 116 | 3.59E-09 | -5.50667 |
| 1565483_at | EGFR | epidermal growth factor receptor (erythroblastic leukemia viral (v-erb-b) oncoge | 6.31E-05 | -5.51243 |
| 214162_at | LOC284244 | hypothetical protein LOC284244 | 4.82E-05 | -5.51602 |
| 209540_at | IGF1 | insulin-like growth factor 1 (somatomedin C) | 1.52E-06 | -5.5196 |
| 244766_at | LOC100271836 /// LOC100288704 /// LOC440354 /// LOC595101 /// LOC641298 /// SMG1 | SMG1 homolog, phosphatidylinositol 3-kinase-related kinase pseudogene /// simila | 1.94E-05 | -5.5219 |
| 223796_at | CNTNAP3 | contactin associated protein-like 3 | 0.000102 | -5.53425 |
| 227761_at | MYO5A | myosin VA (heavy chain 12, myoxin) | 4.91E-05 | -5.53774 |
| 241977_s_at | RAB3C | RAB3C, member RAS oncogene family | 5.93E-05 | -5.55519 |
| 207870_at | AKAP9 | A kinase (PRKA) anchor protein (yotiao) 9 | 4.21E-05 | -5.55654 |
| 227662_at | SYNPO2 | synaptopodin 2 | 1.45E-06 | -5.57801 |
| 208054_at | HERC4 | hect domain and RLD 4 | 5.47E-07 | -5.58407 |
| 214415_at | PLGLB1 /// PLGLB2 | plasminogen-like B1 /// plasminogen-like B2 | 0.00013 | -5.5882 |
| 242735_x_at | ELF2 | E74-like factor 2 (ets domain transcription factor) | 5.69E-06 | -5.59741 |
| 239151_at | LOC399753 | hypothetical LOC399753 | 2.61E-05 | -5.6037 |
| 1559950_at | FAM66C /// FAM66D | family with sequence similarity 66, member C /// family with sequence similarity | 1.28E-05 | -5.6178 |
| 223434_at | GBP3 | guanylate binding protein 3 | 9.33E-06 | -5.62875 |
| 1553422_s_at | A2BP1 | ataxin 2-binding protein 1 | 2.58E-05 | -5.6403 |
| 219454_at | EGFL6 | EGF-like-domain, multiple 6 | 1.01E-07 | -5.64802 |
| 230400_s_at | PKN2 | protein kinase N2 | 8.76E-06 | -5.64911 |
| 231527_at | FLJ36840 | Hypothetical LOC645524 | 2.74E-05 | -5.66597 |
| 1560734_at | LOC727924 | hypothetical LOC727924 | 1.15E-05 | -5.66634 |
| 224321_at | TMEFF2 | transmembrane protein with EGF-like and two follistatin-like domains 2 | 1.62E-08 | -5.68191 |
| 225720_at | SYNPO2 | synaptopodin 2 | 7.20E-07 | -5.70293 |
| 204527_at | MYO5A | myosin VA (heavy chain 12, myoxin) | 1.54E-06 | -5.71308 |
| 216229_x_at | HCG2P7 | HLA complex group 2 pseudogene 7 | 3.16E-05 | -5.71873 |
| 236081_at | SNCA | synuclein, alpha (non A4 component of amyloid precursor) | 1.11E-08 | -5.72332 |
| 1562664_at | LOC286009 | hypothetical protein LOC286009 | 1.97E-05 | -5.73038 |
| 243109_at | MCTP2 | multiple C2 domains, transmembrane 2 | 0.0001009 | -5.74465 |
| 1555370_a_at | CAMTA1 | calmodulin binding transcription activator 1 | 3.66E-06 | -5.74674 |
| 1556770_a_at | FBXL13 | F-box and leucine-rich repeat protein 13 | 1.18E-07 | -5.75186 |
| 232975_at | HCG18 | HLA complex group 18 | 0.0001105 | -5.79639 |
| 224424_x_at | FKSG73 | ARP3 actin-related protein 3 homolog B pseudogene | 6.33E-05 | -5.79795 |
| 242903_at | IFNGR1 | interferon gamma receptor 1 | 2.75E-06 | -5.80273 |
| 211682_x_at | UGT2B28 | UDP glucuronosyltransferase 2 family, polypeptide B28 | 1.78E-05 | -5.80342 |
| 222073_at | COL4A3 | collagen, type IV, alpha 3 (Goodpasture antigen) | 2.11E-06 | -5.8277 |
| 227384_s_at | LOC727820 | hypothetical protein LOC727820 | 5.84E-07 | -5.82872 |
| 1562527_at | LOC283027 | hypothetical protein LOC283027 | 3.77E-05 | -5.83011 |
| 212634_at | KIAA0776 | KIAA0776 | 3.92E-05 | -5.83444 |
| 226844_at | MOBKL2B | MOB1, Mps One Binder kinase activator-like 2B (yeast) | 0.0002827 | -5.84971 |
| 1561197_at | LOC100294357 /// LOC442028 | hypothetical protein LOC100294357 /// hypothetical LOC442028 | 9.26E-06 | -5.86093 |
| 209685_s_at | PRKCB | protein kinase C, beta | 1.03E-05 | -5.86241 |
| 1556288_at | C18orf62 | chromosome 18 open reading frame 62 | 8.27E-06 | -5.89553 |
| 235377_at | C6orf142 | chromosome 6 open reading frame 142 | 4.01E-07 | -5.90749 |
| 227178_at | CELF2 | CUGBP, Elav-like family member 2 | 2.50E-06 | -5.91192 |
| 224418_x_at | PMCHL1 | pro-melanin-concentrating hormone-like 1 | 3.49E-05 | -5.91598 |
| 229218_at | COL1A2 | collagen, type I, alpha 2 | 5.06E-06 | -5.91619 |
| 228945_s_at | SLC39A8 | Solute carrier family 39 (zinc transporter), member 8 | 4.53E-06 | -5.92345 |
| 220394_at | FGF20 | fibroblast growth factor 20 | 2.34E-06 | -5.94033 |
| 203974_at | HDHD1A | haloacid dehalogenase-like hydrolase domain containing 1A | 1.06E-05 | -5.94463 |
| 201976_s_at | MYO10 | myosin X | 1.37E-06 | -5.96876 |
| 1564160_at | DTHD1 | death domain containing 1 | 6.23E-06 | -5.97182 |
| 224289_s_at | FKSG83 | FKSG83 | 7.04E-05 | -5.97287 |
| 206370_at | PIK3CG | phosphoinositide-3-kinase, catalytic, gamma polypeptide | 6.25E-07 | -5.97305 |
| 235879_at | MBNL1 | Muscleblind-like (Drosophila) | 0.0003195 | -5.98515 |
| 215470_at | GTF2H2B | general transcription factor IIH, polypeptide 2B | 4.98E-06 | -5.98653 |
| 220232_at | SCD5 | stearoyl-CoA desaturase 5 | 2.16E-06 | -6.00238 |
| 1560684_x_at | BCL8 | B-cell CLL/lymphoma 8 | 3.19E-06 | -6.00814 |
| 1555141_a_at | NHEDC1 | Na+/H+ exchanger domain containing 1 | 6.39E-06 | -6.01235 |
| 206667_s_at | SCAMP1 | secretory carrier membrane protein 1 | 1.61E-05 | -6.0124 |
| 1562294_x_at | ANKRD30B | ankyrin repeat domain 30B | 1.44E-05 | -6.04339 |
| 1553666_at | CCDC34 | coiled-coil domain containing 34 | 3.65E-05 | -6.05383 |
| 231213_at | PDE1A | phosphodiesterase 1A, calmodulin-dependent | 9.82E-05 | -6.05758 |
| 1569960_at | BRD7P3 | bromodomain containing 7 pseudogene 3 | 8.36E-05 | -6.05811 |
| 209343_at | EFHD1 | EF-hand domain family, member D1 | 1.19E-05 | -6.06482 |
| 226534_at | KITLG | KIT ligand | 3.91E-05 | -6.08386 |
| 1557664_at | LOC340239 | Hypothetical LOC340239 | 8.15E-05 | -6.09643 |
| 207706_at | USH2A | Usher syndrome 2A (autosomal recessive, mild) | 4.14E-05 | -6.11619 |
| 233549_at | PDE1A | phosphodiesterase 1A, calmodulin-dependent | 5.21E-08 | -6.13422 |
| 226876_at | FAM101B | family with sequence similarity 101, member B | 2.20E-07 | -6.14736 |
| 1552411_at | DEFB106A /// DEFB106B | defensin, beta 106A /// defensin, beta 106B | 3.67E-05 | -6.18334 |
| 209569_x_at | D4S234E /// FOXP1 | DNA segment on chromosome 4 (unique) 234 expressed sequence /// forkhead box P1 | 9.65E-06 | -6.19931 |
| 1558982_at | LOC375010 | ankyrin repeat domain 20 family, member A pseudogene | 8.40E-06 | -6.21169 |
| 1557122_s_at | GABRB2 | gamma-aminobutyric acid (GABA) A receptor, beta 2 | 2.59E-06 | -6.22844 |
| 236862_at | GOPC | Golgi-associated PDZ and coiled-coil motif containing | 1.11E-06 | -6.23222 |
| 1557080_s_at | ITGBL1 | integrin, beta-like 1 (with EGF-like repeat domains) | 5.41E-09 | -6.23772 |
| 1554701_a_at | TBC1D16 | TBC1 domain family, member 16 | 9.33E-06 | -6.28075 |
| 1564164_at | DENND1B | DENN/MADD domain containing 1B | 2.53E-07 | -6.2815 |
| 1564190_x_at | ZNF519 | zinc finger protein 519 | 2.72E-05 | -6.30044 |
| 1569353_at | CP110 | CP110 protein | 1.78E-05 | -6.30063 |
| 224422_x_at | PMCHL2 | pro-melanin-concentrating hormone-like 2 | 1.10E-05 | -6.30434 |
| 234314_at | RALGAPA2 | Ral GTPase activating protein, alpha subunit 2 (catalytic) | 1.18E-06 | -6.30439 |
| 1555367_at | ZNF479 | zinc finger protein 479 | 2.24E-05 | -6.31362 |
| 207598_x_at | XRCC2 | X-ray repair complementing defective repair in Chinese hamster cells 2 | 1.54E-05 | -6.3353 |
| 206794_at | ERBB4 | v-erb-a erythroblastic leukemia viral oncogene homolog 4 (avian) | 8.89E-05 | -6.34704 |
| 240735_at | CDC42BPA | CDC42 binding protein kinase alpha (DMPK-like) | 4.37E-06 | -6.37514 |
| 209970_x_at | CASP1 | caspase 1, apoptosis-related cysteine peptidase (interleukin 1, beta, convertase | 3.75E-07 | -6.39019 |
| 239629_at | CFLAR | CASP8 and FADD-like apoptosis regulator | 6.52E-07 | -6.39601 |
| 215554_at | GPLD1 | glycosylphosphatidylinositol specific phospholipase D1 | 2.96E-05 | -6.40386 |
| 1555368_x_at | ZNF479 | zinc finger protein 479 | 4.90E-06 | -6.43966 |
| 235374_at | MDH1 | Malate dehydrogenase 1, NAD (soluble) | 4.30E-07 | -6.45393 |
| 203455_s_at | SAT1 | spermidine/spermine N1-acetyltransferase 1 | 5.99E-06 | -6.47678 |
| 1569940_at | SLC6A16 | Solute carrier family 6, member 16 | 4.29E-06 | -6.47695 |
| 212631_at | STX7 | syntaxin 7 | 2.81E-06 | -6.49429 |
| 241922_at | LMO4 | LIM domain only 4 | 1.24E-05 | -6.49486 |
| 1555462_at | PPP1R1C | protein phosphatase 1, regulatory (inhibitor) subunit 1C | 9.45E-06 | -6.51376 |
| 1570222_at | NDST4 | N-deacetylase/N-sulfotransferase (heparan glucosaminyl) 4 | 1.30E-07 | -6.55811 |
| 236367_at | SMG7 | Smg-7 homolog, nonsense mediated mRNA decay factor (C. elegans) | 2.14E-08 | -6.56467 |
| 224837_at | FOXP1 | forkhead box P1 | 3.94E-08 | -6.58733 |
| 220026_at | CLCA4 | chloride channel accessory 4 | 0.0001055 | -6.59753 |
| 242601_at | HEPACAM2 | HEPACAM family member 2 | 1.09E-06 | -6.60642 |
| 226402_at | CYP2U1 | cytochrome P450, family 2, subfamily U, polypeptide 1 | 4.83E-05 | -6.61436 |
| 240863_at | CYP19A1 | cytochrome P450, family 19, subfamily A, polypeptide 1 | 7.55E-05 | -6.62256 |
| 1555505_a_at | TYR | tyrosinase (oculocutaneous albinism IA) | 0.0002033 | -6.63923 |
| 1554741_s_at | FGF7 /// KGFLP1 /// KGFLP2 | fibroblast growth factor 7 (keratinocyte growth factor) /// keratinocyte growth | 6.13E-05 | -6.63982 |
| 1555095_at | C6orf123 | chromosome 6 open reading frame 123 | 8.46E-06 | -6.64819 |
| 224370_s_at | CAPS2 | calcyphosine 2 | 9.18E-06 | -6.66532 |
| 219497_s_at | BCL11A | B-cell CLL/lymphoma 11A (zinc finger protein) | 7.86E-07 | -6.67429 |
| 214984_at | LOC100271836 /// LOC440354 /// LOC595101 /// LOC641298 /// SMG1 | SMG1 homolog, phosphatidylinositol 3-kinase-related kinase pseudogene /// PI-3-k | 4.59E-06 | -6.70488 |
| 209555_s_at | CD36 | CD36 molecule (thrombospondin receptor) | 5.78E-07 | -6.72973 |
| 234974_at | GALM | galactose mutarotase (aldose 1-epimerase) | 2.39E-06 | -6.73252 |
| 210742_at | CDC14A | CDC14 cell division cycle 14 homolog A (S. cerevisiae) | 1.66E-06 | -6.73828 |
| 217487_x_at | FOLH1 | folate hydrolase (prostate-specific membrane antigen) 1 | 7.82E-07 | -6.75951 |
| 224425_x_at | FKSG73 | ARP3 actin-related protein 3 homolog B pseudogene | 4.55E-06 | -6.76119 |
| 219498_s_at | BCL11A | B-cell CLL/lymphoma 11A (zinc finger protein) | 9.10E-05 | -6.77473 |
| 235957_at | GRIP1 | glutamate receptor interacting protein 1 | 8.59E-06 | -6.80301 |
| 232704_s_at | LRRFIP2 | leucine rich repeat (in FLII) interacting protein 2 | 8.33E-06 | -6.86083 |
| 235638_at | RASSF6 | Ras association (RalGDS/AF-6) domain family member 6 | 4.79E-06 | -6.86327 |
| 216837_at | EPHA5 | EPH receptor A5 | 3.20E-09 | -6.87091 |
| 236761_at | LHFPL3 | lipoma HMGIC fusion partner-like 3 | 3.02E-06 | -6.88524 |
| 233241_at | PLK1S1 | polo-like kinase 1 substrate 1 | 1.48E-06 | -6.89257 |
| 233463_at | RASSF6 | Ras association (RalGDS/AF-6) domain family member 6 | 5.54E-07 | -6.89743 |
| 230061_at | TM4SF18 | Transmembrane 4 L six family member 18 | 7.39E-05 | -6.92795 |
| 223824_at | RNLS | renalase, FAD-dependent amine oxidase | 5.15E-07 | -6.95168 |
| 206584_at | LY96 | lymphocyte antigen 96 | 1.06E-07 | -6.96914 |
| 224402_s_at | FCRL4 | Fc receptor-like 4 | 3.89E-06 | -6.9885 |
| 205824_at | HSPB2 | heat shock 27kDa protein 2 | 6.19E-06 | -7.00484 |
| 205730_s_at | ABLIM3 | actin binding LIM protein family, member 3 | 2.25E-10 | -7.04 |
| 205681_at | BCL2A1 | BCL2-related protein A1 | 9.32E-06 | -7.04754 |
| 204409_s_at | EIF1AY | eukaryotic translation initiation factor 1A, Y-linked | 4.03E-05 | -7.07177 |
| 213939_s_at | RUFY3 | RUN and FYVE domain containing 3 | 1.63E-06 | -7.10061 |
| 235023_at | VPS13C | Vacuolar protein sorting 13 homolog C (S. cerevisiae) | 5.65E-05 | -7.10223 |
| 228610_at | TM9SF3 | Transmembrane 9 superfamily member 3 | 2.84E-05 | -7.10339 |
| 240204_at | SNRPN | small nuclear ribonucleoprotein polypeptide N | 1.01E-05 | -7.10359 |
| 210592_s_at | SAT1 | spermidine/spermine N1-acetyltransferase 1 | 5.10E-08 | -7.13669 |
| 207135_at | HTR2A | 5-hydroxytryptamine (serotonin) receptor 2A | 1.07E-06 | -7.14847 |
| 244134_at | OXCT1 | 3-oxoacid CoA transferase 1 | 1.40E-08 | -7.18142 |
| 231252_at | C2orf67 | chromosome 2 open reading frame 67 | 3.02E-05 | -7.22058 |
| 216834_at | RGS1 | regulator of G-protein signaling 1 | 7.49E-06 | -7.24592 |
| 206134_at | ADAMDEC1 | ADAM-like, decysin 1 | 1.25E-06 | -7.28636 |
| 224838_at | FOXP1 | forkhead box P1 | 2.65E-07 | -7.29609 |
| 205338_s_at | DCT | dopachrome tautomerase (dopachrome delta-isomerase, tyrosine-related protein 2) | 7.78E-06 | -7.29835 |
| 206740_x_at | SYCP1 | synaptonemal complex protein 1 | 6.66E-05 | -7.31117 |
| 209866_s_at | LPHN3 | latrophilin 3 | 2.68E-05 | -7.37703 |
| 37892_at | COL11A1 | collagen, type XI, alpha 1 | 1.07E-07 | -7.38116 |
| 210409_at | C6orf124 | chromosome 6 open reading frame 124 | 3.17E-06 | -7.38323 |
| 221698_s_at | CLEC7A | C-type lectin domain family 7, member A | 1.95E-05 | -7.40452 |
| 1558534_at | LOC641298 | SMG1 homolog, phosphatidylinositol 3-kinase-related kinase pseudogene | 7.45E-07 | -7.46498 |
| 229649_at | NRXN3 | neurexin 3 | 2.85E-06 | -7.49375 |
| 217137_x_at | LOC100289563 | hypothetical protein LOC100289563 | 1.35E-05 | -7.50195 |
| 1552736_a_at | NETO1 | neuropilin (NRP) and tolloid (TLL)-like 1 | 2.69E-05 | -7.52763 |
| 242814_at | SERPINB9 | serpin peptidase inhibitor, clade B (ovalbumin), member 9 | 2.05E-05 | -7.52848 |
| 236266_at | RORA | RAR-related orphan receptor A | 5.21E-06 | -7.54655 |
| 208109_s_at | C15orf5 | chromosome 15 open reading frame 5 | 8.74E-06 | -7.57638 |
| 235369_at | C14orf28 | chromosome 14 open reading frame 28 | 3.86E-07 | -7.58137 |
| 219757_s_at | C14orf101 | chromosome 14 open reading frame 101 | 2.40E-05 | -7.59579 |
| 230040_at | ADAMTS18 | ADAM metallopeptidase with thrombospondin type 1 motif, 18 | 6.16E-06 | -7.6759 |
| 220576_at | PGAP1 | post-GPI attachment to proteins 1 | 4.10E-05 | -7.67664 |
| 205626_s_at | CALB1 | calbindin 1, 28kDa | 1.96E-07 | -7.69018 |
| 1557290_at | DPY19L2 /// DPY19L2P1 /// DPY19L2P2 /// DPY19L2P4 | dpy-19-like 2 (C. elegans) /// dpy-19-like 2 pseudogene 1 (C. elegans) /// dpy-1 | 2.93E-06 | -7.72166 |
| 220374_at | KLHL28 | kelch-like 28 (Drosophila) | 4.61E-05 | -7.75733 |
| 232953_at | C20orf69 /// LOC100287060 /// LOC100287654 /// LOC100288169 /// LOC728323 /// PCMTD2 | chromosome 20 open reading frame 69 /// similar to hCG1984118 /// similar to Put | 2.18E-06 | -7.77437 |
| 224940_s_at | PAPPA | pregnancy-associated plasma protein A, pappalysin 1 | 7.60E-06 | -7.80886 |
| 233604_at | FLJ22763 | hypothetical gene supported by AK026416 | 1.84E-05 | -7.87389 |
| 1561079_at | ANKRD28 | ankyrin repeat domain 28 | 3.36E-05 | -7.89283 |
| 224421_x_at | PMCHL1 | pro-melanin-concentrating hormone-like 1 | 9.95E-07 | -7.91719 |
| 1555890_at | OR2A20P /// OR2A9P | olfactory receptor, family 2, subfamily A, member 20 pseudogene /// olfactory re | 2.37E-05 | -7.92128 |
| 214723_x_at | ANKRD36 | ankyrin repeat domain 36 | 1.99E-05 | -7.96808 |
| 212681_at | EPB41L3 | erythrocyte membrane protein band 4.1-like 3 | 0.0004912 | -7.97158 |
| 232964_at | SPDYE1 | speedy homolog E1 (Xenopus laevis) | 2.50E-05 | -7.988 |
| 1560207_at | C8orf81 | chromosome 8 open reading frame 81 | 4.27E-07 | -8.00828 |
| 1555786_s_at | C14orf34 | chromosome 14 open reading frame 34 | 2.15E-08 | -8.03368 |
| 215303_at | DCLK1 | doublecortin-like kinase 1 | 4.49E-06 | -8.0593 |
| 215425_at | BTG3 | BTG family, member 3 | 7.09E-07 | -8.07323 |
| 241705_at | ABCA5 | ATP-binding cassette, sub-family A (ABC1), member 5 | 3.18E-06 | -8.08089 |
| 232138_at | MBNL2 | Muscleblind-like 2 (Drosophila) | 1.17E-06 | -8.14435 |
| 242414_at | QPRT | quinolinate phosphoribosyltransferase | 2.27E-07 | -8.16784 |
| 1556277_a_at | PAPD4 | PAP associated domain containing 4 | 9.09E-06 | -8.20926 |
| 243709_at | SLC38A9 | solute carrier family 38, member 9 | 3.78E-05 | -8.211 |
| 238605_at | NOL4 | nucleolar protein 4 | 2.30E-05 | -8.25356 |
| 1556057_s_at | NEUROD1 | neurogenic differentiation 1 | 4.27E-06 | -8.25602 |
| 216877_at | DKFZp686O1327 | Hypothetical gene supported by BC043549; BX648102 | 1.99E-06 | -8.26011 |
| 205174_s_at | QPCT | glutaminyl-peptide cyclotransferase | 8.23E-06 | -8.27604 |
| 231152_at | INO80D | INO80 complex subunit D | 1.02E-05 | -8.29444 |
| 204712_at | WIF1 | WNT inhibitory factor 1 | 2.46E-05 | -8.34369 |
| 202391_at | BASP1 | brain abundant, membrane attached signal protein 1 | 2.26E-05 | -8.36018 |
| 212632_at | STX7 | syntaxin 7 | 1.30E-06 | -8.40335 |
| 1558640_a_at | GUSBP1 | Glucuronidase, beta pseudogene 1 | 9.06E-07 | -8.46404 |
| 1556126_s_at | GPATCH2 | G patch domain containing 2 | 1.69E-06 | -8.49594 |
| 204653_at | TFAP2A | transcription factor AP-2 alpha (activating enhancer binding protein 2 alpha) | 3.05E-08 | -8.51593 |
| 203917_at | CXADR | coxsackie virus and adenovirus receptor | 2.24E-06 | -8.57804 |
| 206826_at | PMP2 | peripheral myelin protein 2 | 5.34E-07 | -8.5985 |
| 233096_at | KIAA1109 | KIAA1109 | 9.72E-05 | -8.63023 |
| 215076_s_at | COL3A1 | collagen, type III, alpha 1 | 1.73E-05 | -8.68963 |
| 204214_s_at | RAB32 | RAB32, member RAS oncogene family | 6.84E-08 | -8.69885 |
| 235368_at | ADAMTS5 | ADAM metallopeptidase with thrombospondin type 1 motif, 5 | 3.27E-06 | -8.73198 |
| 220551_at | SLC17A6 | solute carrier family 17 (sodium-dependent inorganic phosphate cotransporter), m | 5.11E-07 | -8.8719 |
| 220351_at | CCRL1 | chemokine (C-C motif) receptor-like 1 | 3.26E-05 | -8.89501 |
| 1565898_at | METT5D1 | Methyltransferase 5 domain containing 1 | 6.11E-05 | -8.91467 |
| 206786_at | HTN3 | histatin 3 | 1.15E-06 | -8.94257 |
| 231183_s_at | JAG1 | Jagged 1 (Alagille syndrome) | 1.59E-05 | -8.94295 |
| 234949_at | FRG1B | FSHD region gene 1 family, member B | 2.09E-06 | -9.00762 |
| 209840_s_at | LRRN3 | leucine rich repeat neuronal 3 | 3.09E-07 | -9.01401 |
| 224095_at | LOC100128175 | similar to PRO2591 | 1.53E-07 | -9.01849 |
| 213139_at | SNAI2 | snail homolog 2 (Drosophila) | 4.33E-08 | -9.08322 |
| 204731_at | TGFBR3 | transforming growth factor, beta receptor III | 3.42E-05 | -9.17231 |
| 228260_at | ELAVL2 | ELAV (embryonic lethal, abnormal vision, Drosophila)-like 2 (Hu antigen B) | 3.34E-07 | -9.19155 |
| 238303_at | STT3B | STT3, subunit of the oligosaccharyltransferase complex, homolog B (S. cerevisiae | 1.36E-06 | -9.21711 |
| 210718_s_at | ARL17A /// LOC100294341 | ADP-ribosylation factor-like 17A /// similar to ADP-ribosylation factor-like 17 | 6.94E-06 | -9.21748 |
| 220180_at | CCDC68 | coiled-coil domain containing 68 | 8.88E-06 | -9.25572 |
| 241403_at | CLK4 | CDC-like kinase 4 | 1.24E-05 | -9.2952 |
| 1562440_at | MAP3K13 | Mitogen-activated protein kinase kinase kinase 13 | 6.02E-07 | -9.36186 |
| 204777_s_at | MAL | mal, T-cell differentiation protein | 1.39E-05 | -9.43834 |
| 233815_at | NAALAD2 | N-acetylated alpha-linked acidic dipeptidase 2 | 2.04E-06 | -9.55006 |
| 216917_s_at | SYCP1 | synaptonemal complex protein 1 | 3.72E-07 | -9.65791 |
| 204044_at | QPRT | quinolinate phosphoribosyltransferase | 5.58E-08 | -9.8247 |
| 1555439_at | GTF3C3 | general transcription factor IIIC, polypeptide 3, 102kDa | 4.96E-06 | -9.85732 |
| 234331_s_at | FAM84A | family with sequence similarity 84, member A | 1.60E-06 | -9.89601 |
| 206030_at | ASPA | aspartoacylase (Canavan disease) | 2.53E-07 | -9.92222 |
| 244427_at | KIF23 | Kinesin family member 23 | 5.17E-06 | -9.99022 |
| 1568287_at | HMGA2 | high mobility group AT-hook 2 | 3.40E-05 | -10.102 |
| 212805_at | PRUNE2 | prune homolog 2 (Drosophila) | 2.24E-07 | -10.1527 |
| 1557283_a_at | ZNF519 | zinc finger protein 519 | 4.51E-07 | -10.2526 |
| 242829_x_at | FBXL3 | F-box and leucine-rich repeat protein 3 | 6.13E-07 | -10.2933 |
| 1556876_s_at | TPTE2P2 | transmembrane phosphoinositide 3-phosphatase and tensin homolog 2 pseudogene 2 | 1.18E-05 | -10.2954 |
| 1559545_at | SNRPN | small nuclear ribonucleoprotein polypeptide N | 1.87E-07 | -10.3561 |
| 201163_s_at | IGFBP7 | insulin-like growth factor binding protein 7 | 0.0001644 | -10.3583 |
| 228063_s_at | NAP1L5 | nucleosome assembly protein 1-like 5 | 2.65E-07 | -10.5599 |
| 218729_at | LXN | latexin | 6.59E-07 | -10.635 |
| 1552582_at | ABCC13 | ATP-binding cassette, sub-family C (CFTR/MRP), member 13, pseudogene | 1.24E-06 | -10.6882 |
| 221666_s_at | PYCARD | PYD and CARD domain containing | 1.69E-08 | -10.7645 |
| 204542_at | ST6GALNAC2 | ST6 (alpha-N-acetyl-neuraminyl-2,3-beta-galactosyl-1,3)-N-acetylgalactosaminide | 2.43E-07 | -10.8036 |
| 209283_at | CRYAB | crystallin, alpha B | 2.97E-07 | -10.8149 |
| 205782_at | FGF7 | fibroblast growth factor 7 (keratinocyte growth factor) | 1.31E-06 | -10.8196 |
| 1570255_s_at | ANKRD20A1 /// ANKRD20A2 /// ANKRD20A3 /// ANKRD20A4 /// ANKRD20B /// LOC375010 | ankyrin repeat domain 20 family, member A1 /// ankyrin repeat domain 20 family, | 2.04E-05 | -10.8483 |
| 209570_s_at | D4S234E /// FOXP1 | DNA segment on chromosome 4 (unique) 234 expressed sequence /// forkhead box P1 | 1.97E-07 | -10.8851 |
| 209515_s_at | RAB27A | RAB27A, member RAS oncogene family | 8.43E-07 | -10.902 |
| 219935_at | ADAMTS5 | ADAM metallopeptidase with thrombospondin type 1 motif, 5 | 1.72E-05 | -10.9065 |
| 222895_s_at | BCL11B | B-cell CLL/lymphoma 11B (zinc finger protein) | 5.10E-06 | -10.9436 |
| 1560751_at | C18orf16 | chromosome 18 open reading frame 16 | 1.69E-06 | -10.9735 |
| 225846_at | ESRP1 | epithelial splicing regulatory protein 1 | 3.54E-07 | -11.1816 |
| 211494_s_at | SLC4A4 | solute carrier family 4, sodium bicarbonate cotransporter, member 4 | 2.28E-06 | -11.1816 |
| 226905_at | FAM101B | family with sequence similarity 101, member B | 1.08E-09 | -11.2135 |
| 232523_at | MEGF10 | multiple EGF-like-domains 10 | 6.61E-09 | -11.2472 |
| 205337_at | DCT | dopachrome tautomerase (dopachrome delta-isomerase, tyrosine-related protein 2) | 5.25E-06 | -11.2548 |
| 231647_s_at | FCRL5 | Fc receptor-like 5 | 0.0007897 | -11.2575 |
| 225407_at | MBP | myelin basic protein | 1.44E-07 | -11.2783 |
| 202156_s_at | CELF2 | CUGBP, Elav-like family member 2 | 1.66E-06 | -11.6798 |
| 224099_at | KCNH7 | potassium voltage-gated channel, subfamily H (eag-related), member 7 | 1.29E-06 | -11.6856 |
| 230229_at | DLG1 | Discs, large homolog 1 (Drosophila) | 6.32E-06 | -11.7476 |
| 1561271_at | CCDC144C /// LOC100134159 | coiled-coil domain containing 144C /// similar to Coiled-coil domain containing | 3.39E-06 | -11.9343 |
| 225540_at | MAP2 | microtubule-associated protein 2 | 4.59E-07 | -11.9483 |
| 210136_at | MBP | myelin basic protein | 7.05E-07 | -12.0331 |
| 235507_at | PCMTD1 | protein-L-isoaspartate (D-aspartate) O-methyltransferase domain containing 1 | 9.48E-07 | -12.4947 |
| 213737_x_at | LOC728498 | Golgin subfamily A member 8-like protein 1 | 4.42E-07 | -12.5425 |
| 237069_s_at | TRPM1 | transient receptor potential cation channel, subfamily M, member 1 | 7.17E-08 | -12.5481 |
| 209848_s_at | SILV | silver homolog (mouse) | 1.46E-06 | -12.6431 |
| 244508_at | 7-九月 | Septin 7 | 3.33E-06 | -12.7325 |
| 224012_at | ANKRD20A1 /// ANKRD20A2 /// ANKRD20A3 /// ANKRD20A4 | ankyrin repeat domain 20 family, member A1 /// ankyrin repeat domain 20 family, | 2.42E-06 | -12.786 |
| 216922_x_at | DAZ1 /// DAZ2 /// DAZ3 /// DAZ4 | deleted in azoospermia 1 /// deleted in azoospermia 2 /// deleted in azoospermia | 1.39E-06 | -13.2775 |
| 216351_x_at | DAZ1 /// DAZ2 /// DAZ3 /// DAZ4 | deleted in azoospermia 1 /// deleted in azoospermia 2 /// deleted in azoospermia | 2.22E-05 | -13.4636 |
| 218211_s_at | MLPH | melanophilin | 2.91E-08 | -13.6325 |
| 208281_x_at | DAZ1 /// DAZ2 /// DAZ3 /// DAZ4 | deleted in azoospermia 1 /// deleted in azoospermia 2 /// deleted in azoospermia | 3.48E-06 | -13.9462 |
| 1553633_s_at | NHEDC1 | Na+/H+ exchanger domain containing 1 | 1.33E-07 | -14.0605 |
| 232382_s_at | PCMTD1 | protein-L-isoaspartate (D-aspartate) O-methyltransferase domain containing 1 | 5.56E-06 | -14.1928 |
| 222891_s_at | BCL11A | B-cell CLL/lymphoma 11A (zinc finger protein) | 1.92E-07 | -14.918 |
| 229800_at | DCLK1 | Doublecortin-like kinase 1 | 6.20E-09 | -15.0157 |
| 238964_at | FIGN | fidgetin | 3.09E-08 | -15.0462 |
| 215695_s_at | GYG2 | glycogenin 2 | 1.20E-07 | -15.2167 |
| 244631_at | LOC389834 | ankyrin repeat domain 57 pseudogene | 1.34E-06 | -15.5645 |
| 243167_at | ABCB5 | ATP-binding cassette, sub-family B (MDR/TAP), member 5 | 6.48E-07 | -15.8997 |
| 209072_at | MBP | myelin basic protein | 2.33E-07 | -16.0225 |
| 1560431_at | PGM5P1 | phosphoglucomutase 5 pseudogene 1 | 1.21E-06 | -16.9434 |
| 224403_at | FCRL4 | Fc receptor-like 4 | 7.15E-07 | -17.0018 |
| 226632_at | CYGB | cytoglobin | 1.71E-07 | -17.2401 |
| 232122_s_at | VEPH1 | ventricular zone expressed PH domain homolog 1 (zebrafish) | 3.46E-07 | -17.4782 |
| 1564856_s_at | LOC727924 | hypothetical LOC727924 | 8.27E-08 | -18.4441 |
| 228321_s_at | NAA30 | N(alpha)-acetyltransferase 30, NatC catalytic subunit | 1.68E-07 | -18.8662 |
| 210800_at | TIMM8A | translocase of inner mitochondrial membrane 8 homolog A (yeast) | 5.57E-06 | -19.0401 |
| 204284_at | PPP1R3C | protein phosphatase 1, regulatory (inhibitor) subunit 3C | 8.28E-08 | -19.0554 |
| 211751_at | PDE4DIP | phosphodiesterase 4D interacting protein | 7.67E-07 | -19.1517 |
| 242712_x_at | RANBP2 /// RGPD1 /// RGPD2 /// RGPD3 /// RGPD4 /// RGPD5 /// RGPD6 /// RGPD8 | RAN binding protein 2 /// RANBP2-like and GRIP domain containing 1 /// RANBP2-li | 8.62E-07 | -20.2506 |
| 244829_at | C6orf218 | chromosome 6 open reading frame 218 | 4.44E-07 | -21.4162 |
| 209170_s_at | GPM6B | glycoprotein M6B | 2.84E-09 | -23.2041 |
| 211565_at | SH3GL3 | SH3-domain GRB2-like 3 | 2.94E-07 | -23.7257 |
| 236972_at | TRIM63 | tripartite motif-containing 63 | 5.17E-07 | -23.8255 |
| 243689_s_at | FRG1B | FSHD region gene 1 family, member B | 1.43E-06 | -24.3482 |
| 219478_at | WFDC1 | WAP four-disulfide core domain 1 | 2.51E-07 | -24.695 |
| 242344_at | GABRB2 | gamma-aminobutyric acid (GABA) A receptor, beta 2 | 1.71E-07 | -24.9122 |
| 211890_x_at | CAPN3 | calpain 3, (p94) | 8.44E-07 | -25.5374 |
| 230748_at | SLC16A6 | solute carrier family 16, member 6 (monocarboxylic acid transporter 7) | 3.91E-08 | -25.5383 |
| 218694_at | ARMCX1 | armadillo repeat containing, X-linked 1 | 4.62E-07 | -26.1203 |
| 204187_at | GMPR | guanosine monophosphate reductase | 1.79E-09 | -26.7979 |
| 1558579_at | FLJ37786 | hypothetical LOC642691 | 3.59E-06 | -27.3236 |
| 202158_s_at | CELF2 | CUGBP, Elav-like family member 2 | 1.63E-07 | -27.3259 |
| 209167_at | GPM6B | glycoprotein M6B | 1.43E-08 | -27.4975 |
| 221031_s_at | APOLD1 | apolipoprotein L domain containing 1 | 9.43E-10 | -30.0336 |
| 206630_at | TYR | tyrosinase (oculocutaneous albinism IA) | 1.57E-09 | -34.8832 |
| 225061_at | DNAJA4 | DnaJ (Hsp40) homolog, subfamily A, member 4 | 2.60E-09 | -36.9149 |
| 240717_at | ABCB5 | ATP-binding cassette, sub-family B (MDR/TAP), member 5 | 6.25E-09 | -39.3163 |
| 202157_s_at | CELF2 | CUGBP, Elav-like family member 2 | 2.95E-08 | -43.7686 |
| 214475_x_at | CAPN3 | calpain 3, (p94) | 6.37E-10 | -46.3977 |
| 237070_at | TRPM1 | transient receptor potential cation channel, subfamily M, member 1 | 4.22E-06 | -48.6691 |
| 213638_at | PHACTR1 | phosphatase and actin regulator 1 | 7.19E-09 | -48.8598 |
| 201909_at | RPS4Y1 | ribosomal protein S4, Y-linked 1 | 6.33E-10 | -77.5149 |
| 210944_s_at | CAPN3 | calpain 3, (p94) | 5.88E-10 | -78.1748 |
| 206426_at | MLANA | melan-A | 1.19E-07 | -151.066 |
| 206427_s_at | MLANA | melan-A | 1.04E-10 | -289.633 |
| 205694_at | TYRP1 | tyrosinase-related protein 1 | 1.01E-08 | -377.17 |
