# Supplementary material for: Identifying mRNA, MicroRNA and Protein Profiles of Melanoma Exosomes
Source: PLoS One. 2012 Oct 9;7(10):e46874. doi: 10.1371/journal.pone.0046874 (PMC3467276; doi:10.1371/journal.pone.0046874)
Supplement: Table S4 — Differentially expressed miRNAs in HEMa-LP exosomes versus HEMa-LP cells. (DOC) [file pone.0046874.s006.doc]

**Supplementary table S4. Differentially expressed miRNAs in HEMa-LP exosomes versus HEMa-LP cells**

| **Probeset ID** | **Transcript ID** | **p-value** | **Fold change** |
| --- | --- | --- | --- |
| hsa-miR-1281_st | hsa-mir-1281 | 5.97E-05 | 24.5167 |
| hsa-miR-937_st | hsa-mir-937 | 6.98E-05 | 14.6535 |
| hsa-miR-485-3p_st | hsa-mir-485 | 0.000503 | 13.5511 |
| hsa-miR-149_st | hsa-mir-149 | 0.000281 | 11.4336 |
| hsa-let-7b-star_st | hsa-let-7b | 0.000448 | 11.0758 |
| hsa-miR-411-star_st | hsa-mir-411 | 0.000483 | 9.73162 |
| hsa-miR-138-1-star_st | hsa-mir-138-2 // hsa-mir-138-1 | 0.000141 | 8.52909 |
| hsa-miR-885-5p_st | hsa-mir-885 | 0.000739 | 8.49963 |
| hsa-miR-935_st | hsa-mir-935 | 3.84E-05 | 8.29224 |
| hsa-miR-1178_st | hsa-mir-1178 | 0.000191 | 7.73233 |
| hsa-miR-605_st | hsa-mir-605 | 0.000653 | 5.01592 |
| hsa-miR-105_st | hsa-mir-105-1 // hsa-mir-105-2 | 4.30E-05 | 4.79352 |
| hsa-miR-574-3p_st | hsa-mir-574 | 0.000586 | 4.43565 |
| hsa-miR-345_st | hsa-mir-345 | 5.31E-07 | 4.43372 |
| hsa-miR-92a_st | hsa-mir-92a-1 // hsa-mir-92a-2 | 0.000318 | -2.12635 |
| hsa-miR-93_st | hsa-mir-93 | 0.000446 | -2.51837 |
| hsa-miR-106a_st | hsa-mir-106a | 4.92E-05 | -2.80903 |
| hsa-miR-25_st | hsa-mir-25 | 8.28E-05 | -3.24971 |
| hsa-miR-152_st | hsa-mir-152 | 9.25E-05 | -3.70427 |
| hsa-miR-501-3p_st | hsa-mir-501 | 0.000488 | -4.18186 |
| hsa-miR-221_st | hsa-mir-221 | 5.36E-07 | -4.36727 |
| hsa-miR-222_st | hsa-mir-222 | 1.55E-08 | -4.48084 |
| hsa-miR-425_st | hsa-mir-425 | 0.000103 | -4.70861 |
| hsa-miR-1307_st | hsa-mir-1307 | 0.000652 | -4.96735 |
| hsa-miR-361-5p_st | hsa-mir-361 | 0.000136 | -5.06123 |
| hsa-miR-1275_st | hsa-mir-1275 | 0.00079 | -5.13698 |
| hsa-miR-584_st | hsa-mir-584 | 5.57E-06 | -5.17213 |
| hsa-miR-16_st | hsa-mir-16-1 // hsa-mir-16-2 | 7.15E-09 | -5.19881 |
| hsa-miR-320c_st | hsa-mir-320c-1 // hsa-mir-320c-2 | 1.10E-05 | -5.2862 |
| hsa-miR-26a_st | hsa-mir-26a-2 // hsa-mir-26a-1 | 5.91E-08 | -5.29931 |
| hsa-miR-886-5p_st | hsa-mir-886 | 5.33E-06 | -5.3338 |
| hsa-miR-1231_st | hsa-mir-1231 | 5.23E-05 | -5.38751 |
| hsa-miR-675_st | hsa-mir-675 | 0.00065 | -6.02073 |
| hsa-miR-193b_st | hsa-mir-193b | 0.000743 | -6.26696 |
| hsa-miR-1301_st | hsa-mir-1301 | 9.04E-06 | -6.36714 |
| hsa-miR-151-5p_st | hsa-mir-151 | 6.69E-07 | -6.53045 |
| hsa-miR-100_st | hsa-mir-100 | 4.70E-07 | -6.97581 |
| hsa-miR-509-3p_st | hsa-mir-509-2 // hsa-mir-509-3 // hsa-mir-509-1 | 7.22E-05 | -7.70573 |
| hsa-miR-125b_st | hsa-mir-125b-1 // hsa-mir-125b-2 | 0.000285 | -8.20664 |
| hsa-let-7a_st | hsa-let-7a-2 // hsa-let-7a-3 // hsa-let-7a-1 | 2.54E-05 | -8.29224 |
| hsa-miR-23a-star_st | hsa-mir-23a | 0.000156 | -8.34206 |
| hsa-miR-423-5p_st | hsa-mir-423 | 0.000219 | -8.6186 |
| hsa-miR-508-5p_st | hsa-mir-508 | 0.000807 | -9.60974 |
| hsa-miR-15b_st | hsa-mir-15b | 1.03E-06 | -9.74131 |
| hsa-miR-324-3p_st | hsa-mir-324 | 2.49E-05 | -10.2972 |
| hsa-miR-181a_st | hsa-mir-181a-1 // hsa-mir-181a-2 | 1.71E-07 | -10.4074 |
| hsa-miR-193a-5p_st | hsa-mir-193a | 1.70E-05 | -10.4598 |
| hsa-miR-500-star_st | hsa-mir-500 | 0.000179 | -10.8967 |
| hsa-miR-362-5p_st | hsa-mir-362 | 0.000153 | -10.9864 |
| hsa-miR-28-3p_st | hsa-mir-28 | 6.82E-06 | -10.9904 |
| hsa-miR-532-5p_st | hsa-mir-532 | 4.76E-08 | -11.2502 |
| hsa-miR-652_st | hsa-mir-652 | 1.96E-05 | -11.6938 |
| hsa-miR-140-3p_st | hsa-mir-140 | 1.77E-07 | -12.6096 |
| hsa-miR-210_st | hsa-mir-210 | 0.000183 | -13.3195 |
| hsa-miR-502-3p_st | hsa-mir-502 | 1.90E-05 | -14.5111 |
| hsa-miR-31_st | hsa-mir-31 | 3.18E-07 | -15.0315 |
| hsa-miR-1826_st | hsa-mir-1826 | 3.19E-05 | -15.7061 |
| hsa-miR-27a_st | hsa-mir-27a | 1.88E-07 | -16.1907 |
| hsa-miR-20b_st | hsa-mir-20b | 6.32E-07 | -16.9816 |
| hsa-miR-744_st | hsa-mir-744 | 1.13E-06 | -17.4215 |
| hsa-let-7i_st | hsa-let-7i | 2.21E-05 | -17.56 |
| hsa-miR-423-3p_st | hsa-mir-423 | 2.29E-06 | -17.7594 |
| hsa-miR-638_st | hsa-mir-638 | 2.30E-05 | -18.2626 |
| hsa-miR-28-5p_st | hsa-mir-28 | 2.77E-05 | -18.5953 |
| hsa-miR-130b_st | hsa-mir-130b | 3.10E-06 | -19.2517 |
| hsa-miR-181b_st | hsa-mir-181b-1 // hsa-mir-181b-2 | 2.21E-06 | -19.5105 |
| hsa-miR-324-5p_st | hsa-mir-324 | 4.19E-06 | -19.6655 |
| hsa-miR-125a-5p_st | hsa-mir-125a | 4.45E-06 | -19.8658 |
| hsa-miR-1268_st | hsa-mir-1268 | 6.63E-05 | -20.0557 |
| hsa-miR-422a_st | hsa-mir-422a | 1.38E-05 | -21.72 |
| hsa-miR-151-3p_st | hsa-mir-151 | 0.000149 | -21.9186 |
| hsa-miR-20a_st | hsa-mir-20a | 5.17E-07 | -22.8969 |
| hsa-miR-29a_st | hsa-mir-29a | 1.86E-06 | -24.6981 |
| hsa-miR-1207-5p_st | hsa-mir-1207 | 4.38E-05 | -27.1836 |
| hsa-miR-19b_st | hsa-mir-19b-1 // hsa-mir-19b-2 | 5.45E-05 | -27.9059 |
| hsa-miR-378_st | hsa-mir-378 | 8.55E-07 | -29.3103 |
| hsa-miR-22_st | hsa-mir-22 | 8.21E-08 | -31.7421 |
| hsa-miR-30d_st | hsa-mir-30d | 5.55E-06 | -37.9814 |
| hsa-miR-155_st | hsa-mir-155 | 0.000206 | -40.6898 |
| hsa-miR-663_st | hsa-mir-663 | 5.72E-08 | -41.699 |
| hsa-let-7c_st | hsa-let-7c | 1.01E-07 | -46.9213 |
| hsa-miR-138_st | hsa-mir-138-2 // hsa-mir-138-1 | 4.31E-07 | -65.9011 |
| hsa-miR-18a_st | hsa-mir-18a | 7.53E-08 | -66.776 |
| hsa-miR-320d_st | hsa-mir-320d-1 // hsa-mir-320d-2 | 1.32E-05 | -70.6651 |
| hsa-miR-130a_st | hsa-mir-130a | 3.98E-07 | -86.2747 |
| hsa-miR-1228-star_st | hsa-mir-1228 | 6.17E-06 | -110.902 |
| hsa-miR-106b_st | hsa-mir-106b | 3.35E-08 | -128.764 |
| hsa-miR-34a_st | hsa-mir-34a | 5.84E-07 | -155.528 |
| hsa-miR-768-5p_st | hsa-mir-768 | 0.000278 | -250.527 |
