# Supplementary material for: Identifying mRNA, MicroRNA and Protein Profiles of Melanoma Exosomes
Source: PLoS One. 2012 Oct 9;7(10):e46874. doi: 10.1371/journal.pone.0046874 (PMC3467276; doi:10.1371/journal.pone.0046874)
Supplement: Table S5 — Differentially expressed miRNAs in A375 exosomes versus A375 cells. (DOC) [file pone.0046874.s007.doc]

**Supplementary table S5. Differentially expressed miRNAs in A375 exosomes versus A375 cells**

| **Probeset ID** | **Transcript ID** | ***p*-value** | **Fold change** |
| --- | --- | --- | --- |
| hsa-miR-575_st | hsa-mir-575 | 2.61E-05 | 46.3023 |
| hsa-miR-149-star_st | hsa-mir-149 | 7.64E-05 | 38.1905 |
| hsa-miR-1300_st | hsa-mir-1300 | 0.000231 | 32.7947 |
| hsa-miR-297_st | hsa-mir-297 | 3.98E-05 | 24.6012 |
| hsa-miR-1224-5p_st | hsa-mir-1224 | 0.000138 | 23.4908 |
| hsa-miR-1225-5p_st | hsa-mir-1225 | 0.000184 | 23.2575 |
| hsa-miR-1228-star_st | hsa-mir-1228 | 1.70E-05 | 22.8388 |
| hsa-miR-92b-star_st | hsa-mir-92b | 0.00015 | 18.8363 |
| hsa-miR-874_st | hsa-mir-874 | 8.62E-05 | 17.8127 |
| hsa-miR-1246_st | hsa-mir-1246 | 3.39E-06 | 17.7516 |
| hsa-miR-195-star_st | hsa-mir-195 | 3.27E-05 | 15.4636 |
| hsa-miR-1290_st | hsa-mir-1290 | 4.81E-05 | 15.3229 |
| hsa-miR-920_st | hsa-mir-920 | 7.50E-05 | 12.6116 |
| hsa-miR-1202_st | hsa-mir-1202 | 3.25E-05 | 12.5472 |
| hsa-miR-135a-star_st | hsa-mir-135a-2 // hsa-mir-135a-1 | 9.27E-05 | 11.7042 |
| hsa-miR-494_st | hsa-mir-494 | 9.13E-05 | 11.1061 |
| hsa-miR-663_st | hsa-mir-663 | 1.94E-07 | 10.8396 |
| hsa-miR-665_st | hsa-mir-665 | 4.57E-05 | 10.3746 |
| hsa-miR-638_st | hsa-mir-638 | 2.59E-05 | 10.1198 |
| hsa-miR-939_st | hsa-mir-939 | 6.23E-05 | 10.068 |
| hsa-miR-923_st | hsa-mir-923 | 6.78E-07 | 9.24476 |
| hsa-miR-1207-5p_st | hsa-mir-1207 | 0.000148 | 8.13818 |
| hsa-miR-346_st | hsa-mir-346 | 0.000246 | 5.91361 |
| hsa-miR-1268_st | hsa-mir-1268 | 0.000433 | 5.23168 |
| hsa-miR-1308_st | hsa-mir-1308 | 1.20E-05 | 3.7476 |
| hsa-miR-563_st | hsa-mir-563 | 0.000297 | 3.49676 |
| hsa-miR-555_st | hsa-mir-555 | 8.85E-05 | 3.32992 |
| hsa-miR-1231_st | hsa-mir-1231 | 0.000275 | 2.64968 |
| hsa-miR-339-5p_st | hsa-mir-339 | 0.000178 | -2.9755 |
| hsa-miR-193a-5p_st | hsa-mir-193a | 0.00024 | -3.03975 |
| hsa-miR-28-3p_st | hsa-mir-28 | 9.31E-05 | -3.152 |
| hsa-miR-125a-5p_st | hsa-mir-125a | 0.000111 | -3.55086 |
| hsa-miR-935_st | hsa-mir-935 | 2.90E-05 | -6.22801 |
