# Supplementary material for: Identifying mRNA, MicroRNA and Protein Profiles of Melanoma Exosomes
Source: PLoS One. 2012 Oct 9;7(10):e46874. doi: 10.1371/journal.pone.0046874 (PMC3467276; doi:10.1371/journal.pone.0046874)
Supplement: Table S6 — Differentially expressed miRNAs in A375 exosomes versus HEMa-LP exosomes. (DOC) [file pone.0046874.s008.doc]

**Supplementary table S6. Differentially expressed miRNAs in A375 exosomes versus HEMa-LP exosomes**

| **Probeset ID** | **Transcript ID** | ***p*-value** | **Fold change** |
| --- | --- | --- | --- |
| hsa-miR-1228-star_st | hsa-mir-1228 | 1.55E-06 | 505.036 |
| hsa-miR-1207-5p_st | hsa-mir-1207 | 2.59E-06 | 350.885 |
| hsa-miR-1246_st | hsa-mir-1246 | 3.17E-07 | 291.088 |
| hsa-miR-1268_st | hsa-mir-1268 | 3.48E-06 | 234.973 |
| hsa-miR-320d_st | hsa-mir-320d-1 // hsa-mir-320d-2 | 4.70E-06 | 190.473 |
| hsa-miR-106b_st | hsa-mir-106b | 2.51E-08 | 171.728 |
| hsa-miR-92b-star_st | hsa-mir-92b | 2.70E-05 | 166.596 |
| hsa-miR-18a_st | hsa-mir-18a | 3.74E-08 | 125.793 |
| hsa-miR-1225-5p_st | hsa-mir-1225 | 6.53E-05 | 118.907 |
| hsa-miR-574-5p_st | hsa-mir-574 | 0.000230898 | 83.9087 |
| hsa-miR-1275_st | hsa-mir-1275 | 6.73E-06 | 80.5778 |
| hsa-miR-31_st | hsa-mir-31 | 2.91E-08 | 79.5696 |
| hsa-miR-19b_st | hsa-mir-19b-1 // hsa-mir-19b-2 | 1.59E-05 | 72.2847 |
| hsa-miR-1300_st | hsa-mir-1300 | 0.000229558 | 72.2301 |
| hsa-miR-939_st | hsa-mir-939 | 8.71E-06 | 68.3327 |
| hsa-miR-663_st | hsa-mir-663 | 3.15E-08 | 66.9031 |
| hsa-miR-483-5p_st | hsa-mir-483 | 0.000181898 | 65.3621 |
| hsa-miR-149-star_st | hsa-mir-149 | 0.000108949 | 63.2017 |
| hsa-let-7c_st | hsa-let-7c | 7.27E-08 | 61.0263 |
| hsa-miR-140-3p_st | hsa-mir-140 | 1.80E-08 | 55.0637 |
| hsa-miR-25-star_st | hsa-mir-25 | 3.68E-05 | 54.4529 |
| hsa-miR-1224-5p_st | hsa-mir-1224 | 0.000118885 | 53.8878 |
| hsa-miR-138_st | hsa-mir-138-2 // hsa-mir-138-1 | 5.88E-07 | 51.1915 |
| hsa-miR-20a_st | hsa-mir-20a | 1.67E-07 | 50.9196 |
| hsa-miR-885-3p_st | hsa-mir-885 | 0.000330485 | 50.1928 |
| hsa-miR-100_st | hsa-mir-100 | 1.47E-08 | 49.0119 |
| hsa-miR-125b_st | hsa-mir-125b-1 // hsa-mir-125b-2 | 1.66E-05 | 43.9443 |
| hsa-miR-671-5p_st | hsa-mir-671 | 8.02E-05 | 42.4832 |
| hsa-miR-23a-star_st | hsa-mir-23a | 1.00E-05 | 41.3548 |
| hsa-miR-130a_st | hsa-mir-130a | 1.04E-06 | 39.3173 |
| hsa-miR-1826_st | hsa-mir-1826 | 8.06E-06 | 38.1896 |
| hsa-miR-516b_st | hsa-mir-516b-2 // hsa-mir-516b-1 | 7.30E-05 | 36.6498 |
| hsa-miR-1202_st | hsa-mir-1202 | 1.65E-05 | 35.0684 |
| hsa-miR-34a_st | hsa-mir-34a | 3.46E-06 | 34.0248 |
| hsa-miR-17-star_st | hsa-mir-17 | 0.000463554 | 33.1164 |
| hsa-miR-744_st | hsa-mir-744 | 4.12E-07 | 33.102 |
| hsa-miR-638_st | hsa-mir-638 | 9.39E-06 | 32.5748 |
| hsa-miR-182_st | hsa-mir-182 | 3.78E-07 | 30.4335 |
| hsa-miR-151-3p_st | hsa-mir-151 | 9.55E-05 | 29.602 |
| hsa-miR-125b-1-star_st | hsa-mir-125b-1 // hsa-mir-125b-2 | 0.000180706 | 28.2338 |
| hsa-miR-575_st | hsa-mir-575 | 0.00013777 | 28.1233 |
| hsa-miR-30d_st | hsa-mir-30d | 8.60E-06 | 27.9004 |
| hsa-miR-423-5p_st | hsa-mir-423 | 2.80E-05 | 26.9011 |
| hsa-miR-297_st | hsa-mir-297 | 9.36E-05 | 26.8554 |
| hsa-miR-1290_st | hsa-mir-1290 | 5.63E-05 | 25.465 |
| hsa-miR-874_st | hsa-mir-874 | 0.000131947 | 25.2651 |
| hsa-miR-936_st | hsa-mir-936 | 0.000474295 | 24.1406 |
| hsa-miR-658_st | hsa-mir-658 | 0.000765788 | 24.0177 |
| hsa-miR-584_st | hsa-mir-584 | 2.17E-07 | 23.4809 |
| hsa-miR-920_st | hsa-mir-920 | 7.50E-05 | 22.3056 |
| hsa-miR-675_st | hsa-mir-675 | 5.00E-05 | 21.5369 |
| hsa-miR-494_st | hsa-mir-494 | 7.95E-05 | 20.784 |
| hsa-miR-18b_st | hsa-mir-18b | 0.000232157 | 19.0607 |
| hsa-miR-196a_st | hsa-mir-196a-2 // hsa-mir-196a-1 | 0.000363491 | 18.635 |
| hsa-miR-99a_st | hsa-mir-99a | 8.93E-06 | 18.5486 |
| hsa-miR-422a_st | hsa-mir-422a | 1.91E-05 | 17.8381 |
| hsa-miR-21_st | hsa-mir-21 | 0.000775614 | 17.2606 |
| hsa-miR-378_st | hsa-mir-378 | 2.21E-06 | 16.2888 |
| hsa-miR-30a_st | hsa-mir-30a | 0.000313186 | 16.2258 |
| hsa-miR-125a-3p_st | hsa-mir-125a | 0.000113581 | 16.1671 |
| hsa-miR-1226-star_st | hsa-mir-1226 | 0.000847071 | 16.1019 |
| hsa-miR-135a-star_st | hsa-mir-135a-2 // hsa-mir-135a-1 | 0.000142887 | 15.7096 |
| hsa-miR-130b_st | hsa-mir-130b | 4.63E-06 | 15.2999 |
| hsa-miR-498_st | hsa-mir-498 | 8.43E-06 | 14.7746 |
| hsa-miR-324-3p_st | hsa-mir-324 | 1.29E-05 | 14.3844 |
| hsa-miR-30b-star_st | hsa-mir-30b | 0.00170916 | 14.3131 |
| hsa-miR-92a-1-star_st | hsa-mir-92a-1 // hsa-mir-92a-2 | 0.00121319 | 14.1813 |
| hsa-miR-22_st | hsa-mir-22 | 3.09E-07 | 14.1611 |
| hsa-miR-210_st | hsa-mir-210 | 0.000164975 | 14.0979 |
| hsa-miR-652_st | hsa-mir-652 | 1.38E-05 | 14.0021 |
| hsa-let-7g_st | hsa-let-7g | 0.00176958 | 13.9158 |
| hsa-miR-15a_st | hsa-mir-15a | 0.000787008 | 13.5709 |
| hsa-miR-152_st | hsa-mir-152 | 3.22E-06 | 13.3912 |
| hsa-miR-140-5p_st | hsa-mir-140 | 1.83E-05 | 13.3794 |
| hsa-miR-512-3p_st | hsa-mir-512-1 // hsa-mir-512-2 | 0.00111791 | 13.1842 |
| hsa-miR-629_st | hsa-mir-629 | 0.000343276 | 12.5262 |
| hsa-miR-877_st | hsa-mir-877 | 8.96E-05 | 12.2348 |
| hsa-miR-886-5p_st | hsa-mir-886 | 7.54E-07 | 11.9645 |
| hsa-miR-20b_st | hsa-mir-20b | 1.29E-06 | 11.6251 |
| hsa-miR-532-5p_st | hsa-mir-532 | 5.01E-08 | 10.9735 |
| hsa-miR-222_st | hsa-mir-222 | 1.59E-09 | 10.6388 |
| hsa-miR-27a-star_st | hsa-mir-27a | 0.0016846 | 10.301 |
| hsa-miR-1323_st | hsa-mir-1323 | 0.000238 | 9.87272 |
| hsa-miR-519b-5p_st | hsa-mir-519b | 0.00130342 | 9.82561 |
| hsa-miR-324-5p_st | hsa-mir-324 | 1.62E-05 | 9.6257 |
| hsa-miR-425_st | hsa-mir-425 | 1.72E-05 | 9.35747 |
| hsa-miR-181b_st | hsa-mir-181b-1 // hsa-mir-181b-2 | 1.03E-05 | 8.80305 |
| hsa-miR-199b-3p_st | hsa-mir-199b | 0.000800773 | 8.67688 |
| hsa-miR-423-3p_st | hsa-mir-423 | 9.66E-06 | 8.58766 |
| hsa-miR-193b-star_st | hsa-mir-193b | 0.000462364 | 8.55467 |
| hsa-let-7a_st | hsa-let-7a-2 // hsa-let-7a-3 // hsa-let-7a-1 | 2.45E-05 | 8.42611 |
| hsa-miR-424-star_st | hsa-mir-424 | 0.000157474 | 8.23534 |
| hsa-miR-502-3p_st | hsa-mir-502 | 7.66E-05 | 7.46927 |
| hsa-miR-30c-2-star_st | hsa-mir-30c-1 // hsa-mir-30c-2 | 0.000280572 | 7.44762 |
| hsa-miR-1231_st | hsa-mir-1231 | 2.43E-05 | 7.16585 |
| hsa-miR-221_st | hsa-mir-221 | 1.29E-07 | 7.1091 |
| hsa-miR-29a_st | hsa-mir-29a | 2.17E-05 | 7.03373 |
| hsa-miR-1307_st | hsa-mir-1307 | 0.000295605 | 6.6647 |
| hsa-miR-660_st | hsa-mir-660 | 0.000489615 | 6.59906 |
| hsa-miR-320c_st | hsa-mir-320c-1 // hsa-mir-320c-2 | 6.38E-06 | 6.42105 |
| hsa-miR-193a-5p_st | hsa-mir-193a | 5.59E-05 | 6.30261 |
| hsa-miR-665_st | hsa-mir-665 | 0.000413014 | 6.12525 |
| hsa-miR-25_st | hsa-mir-25 | 1.02E-05 | 6.08933 |
| hsa-miR-27a_st | hsa-mir-27a | 1.77E-06 | 5.90604 |
| hsa-miR-500-star_st | hsa-mir-500 | 0.000804433 | 5.68279 |
| hsa-miR-523-star_st | hsa-mir-523 | 0.000948735 | 5.54336 |
| hsa-miR-15b_st | hsa-mir-15b | 4.82E-06 | 5.29454 |
| hsa-miR-1180_st | hsa-mir-1180 | 0.000833146 | 4.74018 |
| hsa-miR-28-5p_st | hsa-mir-28 | 0.000639848 | 4.57202 |
| hsa-miR-940_st | hsa-mir-940 | 0.00178096 | 4.56052 |
| hsa-miR-106a_st | hsa-mir-106a | 8.16E-06 | 4.43263 |
| hsa-miR-28-3p_st | hsa-mir-28 | 7.20E-05 | 4.4045 |
| hsa-miR-151-5p_st | hsa-mir-151 | 2.32E-06 | 4.30919 |
| hsa-miR-191-star_st | hsa-mir-191 | 0.000678889 | 4.09067 |
| hsa-miR-181a_st | hsa-mir-181a-1 // hsa-mir-181a-2 | 2.30E-06 | 4.0121 |
| hsa-miR-1308_st | hsa-mir-1308 | 2.60E-05 | 3.98399 |
| hsa-miR-106b-star_st | hsa-mir-106b | 0.000235592 | 3.76911 |
| hsa-miR-16_st | hsa-mir-16-1 // hsa-mir-16-2 | 2.22E-08 | 3.72143 |
| hsa-miR-92a_st | hsa-mir-92a-1 // hsa-mir-92a-2 | 2.25E-05 | 3.68391 |
| hsa-miR-620_st | hsa-mir-620 | 0.000159954 | 3.58654 |
| hsa-miR-27b_st | hsa-mir-27b | 0.000937003 | 3.48137 |
| hsa-miR-601_st | hsa-mir-601 | 0.000416147 | 3.32931 |
| hsa-miR-17_st | hsa-mir-17 | 8.00E-05 | 3.29328 |
| hsa-miR-98_st | hsa-mir-98 | 0.0006643 | 3.02189 |
| hsa-miR-330-3p_st | hsa-mir-330 | 0.000508667 | 2.9645 |
| hsa-miR-1301_st | hsa-mir-1301 | 0.000145987 | 2.84978 |
| hsa-miR-125a-5p_st | hsa-mir-125a | 0.000861431 | 2.73428 |
| hsa-miR-923_st | hsa-mir-923 | 9.71E-05 | 2.70408 |
| hsa-miR-93_st | hsa-mir-93 | 0.000332297 | 2.67258 |
| hsa-miR-208a_st | hsa-mir-208a | 0.00100027 | 2.15041 |
| hsa-miR-200b_st | hsa-mir-200b | 0.000735376 | -2.35632 |
| hsa-miR-23b_st | hsa-mir-23b | 0.000145511 | -2.46459 |
| hsa-miR-146a_st | hsa-mir-146a | 8.76E-05 | -2.69393 |
| hsa-miR-433_st | hsa-mir-433 | 0.000797291 | -2.97062 |
| hsa-let-7d_st | hsa-let-7d | 2.71E-05 | -3.00489 |
| hsa-miR-186_st | hsa-mir-186 | 0.0018126 | -3.31305 |
| hsa-miR-429_st | hsa-mir-429 | 0.00153772 | -3.38207 |
| hsa-miR-136-star_st | hsa-mir-136 | 0.000960818 | -4.22928 |
| hsa-miR-302f_st | hsa-mir-302f | 0.001394 | -4.25339 |
| hsa-miR-376a-star_st | hsa-mir-376a-2 // hsa-mir-376a-1 | 0.00129308 | -4.76854 |
| hsa-miR-7-2-star_st | hsa-mir-7-2 // hsa-mir-7-3 // hsa-mir-7-1 | 0.000885861 | -5.7772 |
| hsa-miR-646_st | hsa-mir-646 | 0.000680921 | -5.97694 |
| hsa-miR-511_st | hsa-mir-511-1 // hsa-mir-511-2 | 0.000104729 | -6.19338 |
| hsa-miR-551a_st | hsa-mir-551a | 0.000355265 | -6.23238 |
| hsa-miR-1282_st | hsa-mir-1282 | 0.000419911 | -6.52095 |
| hsa-miR-340-star_st | hsa-mir-340 | 0.00105972 | -6.86451 |
| hsa-miR-550-star_st | hsa-mir-550-1 // hsa-mir-550-2 | 0.000947093 | -6.88596 |
| hsa-miR-1184_st | hsa-mir-1184 // hsa-mir-1184 // hsa-mir-1184 | 0.000702152 | -7.12768 |
| hsa-miR-598_st | hsa-mir-598 | 0.0015775 | -7.24971 |
| hsa-miR-548d-3p_st | hsa-mir-548d-2 // hsa-mir-548d-1 | 4.36E-05 | -7.27992 |
| hsa-miR-1207-3p_st | hsa-mir-1207 | 0.000769104 | -8.02748 |
| hsa-miR-589_st | hsa-mir-589 | 0.000239178 | -8.03378 |
| hsa-miR-518d-3p_st | hsa-mir-518d | 0.000647638 | -8.17986 |
| hsa-miR-892b_st | hsa-mir-892b | 0.00143163 | -8.44038 |
| hsa-miR-345_st | hsa-mir-345 | 8.66E-08 | -8.52211 |
| hsa-miR-1281_st | hsa-mir-1281 | 0.000407935 | -8.52596 |
| hsa-miR-431_st | hsa-mir-431 | 0.00127159 | -8.55391 |
| hsa-miR-148a-star_st | hsa-mir-148a | 0.00120094 | -8.60528 |
| hsa-miR-30c_st | hsa-mir-30c-1 // hsa-mir-30c-2 | 5.21E-05 | -8.7249 |
| hsa-miR-181a-2-star_st | hsa-mir-181a-1 // hsa-mir-181a-2 | 8.22E-06 | -8.84383 |
| hsa-miR-182-star_st | hsa-mir-182 | 0.000294902 | -8.91097 |
| hsa-miR-499-3p_st | hsa-mir-499 | 0.000430822 | -9.46698 |
| hsa-miR-219-5p_st | hsa-mir-219-1 // hsa-mir-219-2 | 0.000261388 | -9.9485 |
| hsa-miR-770-5p_st | hsa-mir-770 | 0.001502 | -10.0632 |
| hsa-miR-603_st | hsa-mir-603 | 0.00102662 | -10.7806 |
| hsa-miR-1296_st | hsa-mir-1296 | 0.000220099 | -11.0339 |
| hsa-miR-1279_st | hsa-mir-1279 | 0.000457544 | -11.2268 |
| hsa-miR-299-5p_st | hsa-mir-299 | 0.00028146 | -13.8065 |
| hsa-miR-337-3p_st | hsa-mir-337 | 0.000542528 | -14.5948 |
| hsa-miR-631_st | hsa-mir-631 | 0.0015868 | -15.7695 |
| hsa-miR-431-star_st | hsa-mir-431 | 0.00110193 | -16.4517 |
| hsa-miR-30b_st | hsa-mir-30b | 0.000803366 | -16.8321 |
| hsa-miR-613_st | hsa-mir-613 | 0.000731338 | -18.1017 |
| hsa-miR-614_st | hsa-mir-614 | 0.000632223 | -18.2974 |
| hsa-miR-657_st | hsa-mir-657 | 8.23E-05 | -18.7241 |
| hsa-miR-205_st | hsa-mir-205 | 0.00138379 | -18.9218 |
| hsa-miR-197_st | hsa-mir-197 | 1.41E-05 | -20.2304 |
| hsa-miR-574-3p_st | hsa-mir-574 | 1.99E-05 | -20.2365 |
| hsa-miR-378-star_st | hsa-mir-378 | 3.62E-05 | -22.2091 |
| hsa-miR-605_st | hsa-mir-605 | 2.83E-05 | -22.2354 |
| hsa-miR-218-2-star_st | hsa-mir-218-1 // hsa-mir-218-2 | 0.000505579 | -22.4836 |
| hsa-miR-1200_st | hsa-mir-1200 | 4.40E-05 | -24.0724 |
| hsa-miR-199a-5p_st | hsa-mir-199a-1 // hsa-mir-199a-2 | 0.000281009 | -24.2237 |
| hsa-miR-885-5p_st | hsa-mir-885 | 0.000103042 | -25.5669 |
| hsa-miR-618_st | hsa-mir-618 | 0.000286278 | -26.1404 |
| hsa-miR-609_st | hsa-mir-609 | 3.74E-05 | -27.927 |
| hsa-miR-576-5p_st | hsa-mir-576 | 0.00015573 | -28.0799 |
| hsa-miR-335-star_st | hsa-mir-335 | 0.00142341 | -28.9084 |
| hsa-miR-33a-star_st | hsa-mir-33a | 0.000254151 | -29.8004 |
| hsa-miR-504_st | hsa-mir-504 | 0.000463793 | -31.1698 |
| hsa-miR-218-1-star_st | hsa-mir-218-1 // hsa-mir-218-2 | 0.00076827 | -32.6357 |
| hsa-let-7f-2-star_st | hsa-let-7f-1 // hsa-let-7f-2 | 2.68E-05 | -34.575 |
| hsa-miR-302e_st | hsa-mir-302e | 9.49E-06 | -38.0075 |
| hsa-miR-668_st | hsa-mir-668 | 0.000659835 | -38.4515 |
| hsa-miR-935_st | hsa-mir-935 | 2.58E-06 | -38.746 |
| hsa-let-7g-star_st | hsa-let-7g | 0.0013729 | -39.2665 |
| hsa-miR-597_st | hsa-mir-597 | 0.000753708 | -40.9511 |
| hsa-miR-640_st | hsa-mir-640 | 0.00150222 | -42.7494 |
| hsa-miR-1284_st | hsa-mir-1284 | 0.000293852 | -44.0038 |
| hsa-miR-671-3p_st | hsa-mir-671 | 0.000619384 | -44.4936 |
| hsa-miR-138-1-star_st | hsa-mir-138-2 // hsa-mir-138-1 | 7.97E-06 | -47.3233 |
| hsa-miR-29c-star_st | hsa-mir-29c | 1.50E-05 | -51.8072 |
| hsa-miR-509-3p_st | hsa-mir-509-2 // hsa-mir-509-3 // hsa-mir-509-1 | 2.81E-06 | -51.9599 |
| hsa-miR-149_st | hsa-mir-149 | 1.98E-05 | -67.5136 |
| hsa-miR-220a_st | hsa-mir-220a | 0.000119622 | -69.8681 |
| hsa-let-7e-star_st | hsa-let-7e | 3.94E-05 | -74.8668 |
| hsa-miR-212_st | hsa-mir-212 | 0.000565766 | -75.2433 |
| hsa-miR-432-star_st | hsa-mir-432 | 0.000128676 | -85.8363 |
| hsa-miR-34c-3p_st | hsa-mir-34c | 6.57E-05 | -90.7391 |
| hsa-miR-214-star_st | hsa-mir-214 | 5.98E-06 | -95.3038 |
| hsa-miR-1229_st | hsa-mir-1229 | 0.000806139 | -108.761 |
| hsa-miR-758_st | hsa-mir-758 | 9.19E-05 | -120.233 |
| hsa-let-7b-star_st | hsa-let-7b | 1.57E-05 | -123.021 |
| hsa-let-7d-star_st | hsa-let-7d | 0.0010806 | -141.371 |
| hsa-miR-654-3p_st | hsa-mir-654 | 4.31E-05 | -144.764 |
| hsa-miR-130b-star_st | hsa-mir-130b | 9.98E-06 | -149.159 |
| hsa-miR-185-star_st | hsa-mir-185 | 0.00117822 | -159.808 |
| hsa-miR-483-3p_st | hsa-mir-483 | 0.000642504 | -162.386 |
| hsa-miR-1178_st | hsa-mir-1178 | 2.05E-06 | -174.264 |
| hsa-miR-26b-star_st | hsa-mir-26b | 0.000412254 | -190.45 |
| hsa-miR-411-star_st | hsa-mir-411 | 7.74E-06 | -208.439 |
| hsa-miR-211_st | hsa-mir-211 | 2.49E-05 | -213.089 |
| hsa-miR-485-3p_st | hsa-mir-485 | 1.53E-05 | -215.4 |
| hsa-miR-412_st | hsa-mir-412 | 0.000154358 | -245.148 |
| hsa-miR-744-star_st | hsa-mir-744 | 1.96E-05 | -288.359 |
| hsa-miR-34b_st | hsa-mir-34b | 7.95E-05 | -322.916 |
| hsa-miR-937_st | hsa-mir-937 | 1.44E-06 | -366.943 |
| hsa-miR-647_st | hsa-mir-647 | 0.00020118 | -505.16 |
